# Global burden of diarrheal diseases attributable to 13 pathogens in children under five: a comprehensive analysis from GBD 2021

**DOI:** 10.3389/fped.2026.1846669

**Published:** 2026-09-18

**Authors:** Xue Li, Liping Guo, Yongyan Chen, Hanjuan Zhang

**Affiliations:** 1 Department of Pharmacy, The Seventh People’s Hospital of Zhengzhou, Zhengzhou, China; 2 Institute of Biological Therapy, Henan Academy of Innovations in Medical Science, Zhengzhou, China

**Keywords:** burden of diarrheal disease, children, mortality, neonates, pathogens

## Abstract

**Introduction:**

Diarrheal diseases remain a major cause of death and disability among children under five, especially in low- and middle-income countries (LMICs).

**Methods:**

Using Global Burden of Disease Study 2021 (GBD 2021) data, we assessed mortality and disability-adjusted life years (DALYs) attributable to 13 diarrheal pathogens across 204 countries from 1990 to 2021. Analyses included age-stratified burden estimation, temporal trend analysis using estimated annual percentage change (EAPC), and frontier analysis with the Socio-demographic Index (SDI).

**Results:**

From 1990 to 2021, global diarrheal deaths declined from 2.43 million to 0.56 million, and DALYs from 218.14 million (95% UI: 131.58–335.90 million) to 50.63 million (95% UI: 28.77–82.70 million). *Rotavirus and Shigella* remained leading causes of mortality, while Clostridioides difficile was the only pathogen with a positive EAPC, indicating an increasing burden. Considerable regional and national disparities persisted, with the highest mortality in Chad (842.44 per 100,000) and the lowest in Andorra (0.05 per 100,000).

**Discussion:**

These findings underscore the need for pathogen-specific, regionally targeted interventions to further reduce child mortality and disability.

## 1 Introduction

Diarrheal disease remains a significant public health challenge, particularly among children under five years of age in LMICs (1). According to the World Health Organization (WHO), it is the third leading cause of death in this age group, accounting for an estimated 443,832 deaths annually (2). Reducing child mortality is a core priority of the global health agenda, as reflected in the United Nations Sustainable Development Goals (SDGs). Specifically, SDG 3.2 aims to eliminate preventable child mortality, reduce neonatal deaths to fewer than 12 per 1,000 live births, and reduce the mortality rate of children under the age of five to fewer than 25 per 1,000 live births in every country by 2030 (3). Achieving these targets necessitates comprehensive efforts to address preventable childhood illnesses such as diarrheal disease. Significant policy investments have focused on improving access to safe water, sanitation, hygiene, and expanding vaccination programs—particularly the rotavirus vaccine, which has demonstrated effectiveness in reducing diarrhea-related morbidity and mortality (4, 5). Nonetheless, these interventions alone are insufficient, as numerous infectious pathogens and socio-environmental determinants contributing to the disease burden remain inadequately addressed.

Diarrhea is a common infectious condition caused by a wide range of viral, bacterial, and parasitic pathogens, most of which are transmitted through feces–contaminated water sources. In addition to waterborne transmission, contaminated food products—particularly raw animal-derived products—also serve as major reservoirs for diarrheal pathogens (6). Among children under five years of age, the predominant viral agents include rotavirus, norovirus, and astrovirus (7, 8); common bacterial pathogens are *Escherichia coli*, *Salmonella spp.*, and *Shigella spp.* (9); while parasitic infections are primarily caused by *Cryptosporidium spp.* and *Giardia spp* (10, 11). The Global Enteric Multicenter Study (GEMS) emphasized the diverse etiological spectrum of diarrheal diseases and underscored the need for pathogen-specific prevention and control strategies to mitigate the impact on child health. Several independent studies have further explored diarrheal disease burden using data derived from the GBD framework, illustrating growing research interest in pathogen-specific trends and regional disparities. However, as these analyses were conducted outside the official GBD Collaborator network, their findings should be interpreted as contextual background rather than formal extensions of GBD results (12, 13).

Extensive research has been conducted to quantify the global burden of diarrheal disease. The official GBD 2019 assessment provided global mortality estimates associated with 33 bacterial pathogens (11), and subsequent country-specific studies, such as that by Behera et al., detailed national-level patterns and determinants of diarrheal mortality in India between 1990 and 2019 (14). Despite these efforts, there are still key gaps in understanding the comprehensive impact of less-studied pathogens, regional differences, and socio-environmental factors. These limitations highlight the urgent need for comprehensive, multidimensional research to address these challenges.

In this study, we present updated estimates of mortality and DALYs due to diarrheal disease among neonates and children under five years of age attributable to 13 specific pathogens from 1990 to 2021. Additionally, frontier analysis was employed to assess the relationship between disease burden and socio-demographic development, and to identify countries or regions with the greatest potential for reducing disease burden. The findings aim to support evidence-based decision-making in disease prevention, resource allocation, and public health policy formulation.

## 2 Methods

### 2.1 Data sources and overview of GBD 2021 estimation

This study utilized publicly available data from GBD 2021, coordinated by the Institute for Health Metrics and Evaluation (IHME). The present study represents an independent secondary analysis focusing on diarrheal diseases attributable to specific pathogens among children younger than five years across 204 countries and territories from 1990 to 2021 (15).

Briefly, in GBD 2021, diarrheal mortality was estimated using the Cause of Death Ensemble model (CODEm), while non-fatal burden was estimated using DisMod-MR 2.1. DALYs were calculated as the sum of years of life lost (YLLs) and years lived with disability (YLDs). Pathogen-specific burden was estimated using the GBD etiological attribution framework. Uncertainty was propagated through 1,000 draws to generate 95% uncertainty intervals (UIs). Further methodological details are provided in Supplementary Appendix S1 and the original GBD 2021 Diarrhoeal Diseases publication (15).

### 2.2 Estimation of pathogen-specific burden

Pathogen-attributable diarrheal burden was derived from the GBD 2021 etiologic attribution framework. Briefly, pathogen-specific population-attributable fractions (PAFs) were estimated using a counterfactual approach that incorporates pathogen prevalence and the pathogen-specific risk of diarrhea. These PAFs were applied to the overall diarrheal disease burden to estimate pathogen-attributable deaths and DALYs by age, sex, year, and location. The present study analyzed GBD 2021 estimates attributable to 13 diarrheal pathogens among children younger than five years.

### 2.3 Analytical framework

To ensure consistency across spatial scales, all analyses were conducted using a hierarchical analytical framework. First, global-level analyses were performed to quantify overall mortality, DALYs, and long-term temporal trends attributable to each pathogen among children under five years of age. Second, regional analyses were carried out across the 21 GBD regions to examine geographic heterogeneity in pathogen-specific burden and trends. Finally, national-level analyses were conducted for all 204 countries and territories to identify country-specific patterns, disparities, and potential improvement gaps.

### 2.4 Age stratification

This analysis focused on diarrheal diseases attributable to 13 specific pathogens in neonates (aged <28 days) and children under five years of age. To characterize vulnerability by developmental stage, children under five years were divided into six age groups: 0–6 days, 7–27 days, 1–5 months, 6–11 months, 12–23 months, and 2–4 years (16, 17). All outcome measures were stratified by age group to assess differential patterns in pathogen burden.

### 2.5 Temporal trend analysis

To assess the temporal trends in disease burden from 1990 to 2021, we calculated the EAPC for age-standardized rates (ASRs), including age-standardized DALY rate (ASDR) and age-standardized mortality rate (ASMR). EAPC was calculated by fitting a linear regression model to the natural logarithm of the ASR over time, as follows: ln(ASR)=α+β×Year+ε, where *α* is the intercept, *β* is the slope, and *ε* is the error term. The EAPC was then derived using the formula: $EAPC=100\times (e^{\beta }-1).$ A negative EAPC indicates a declining trend in burden over time (18).

### 2.6 Frontier analysis

To assess the relationship between pathogen-specific diarrheal burden and socio-demographic development, we performed a bootstrap-based non-parametric empirical frontier analysis using ASDR and SDI. For each pathogen, 100 bootstrap samples were generated by resampling country-year observations with replacement. Within each sample, a monotonic lower envelope was constructed using the cumulative minimum of DALY rate ordered by SDI. The bootstrap frontiers were averaged and subsequently smoothed using LOESS (degree = 1, span = 0.20). The frontier gap was calculated as the difference between the observed DALY rate and the corresponding smoothed frontier value, with larger values indicating greater potential for burden reduction. Analyses were implemented in R using base R and the stats package.

### 2.7 Statistical methods

EAPCs were reported with 95% confidence intervals (CIs), and trends were considered statistically significant when the 95% CI did not include zero. GBD-derived burden estimates are presented with their corresponding 95% uncertainty intervals (UIs). All statistical analyses were performed using R software (version 4.4.2), and figures were generated using the ggplot2 and plotly packages. The overall study design and analytical workflow are presented in Supplementary Figure S1.

## 3 Results

### 3.1 Global trends in diarrheal disease burden attributable to specific pathogens

In 2021, diarrheal diseases in children under five years of age caused 562.76 thousand deaths globally (95% UI: 317.95–922.19), down from 2,431.13 thousand (95% UI: 1,462.19–3,748.43) in 1990, with an ASMR reduction from 392.16 to 85.50 per 100,000 population (EAPC: −4.80, 95% CI: −5.17 to −4.44). Mortality decreased for both females and males, with ASMRs declining to 80.22 (95% UI: 44.75–131.23) and 90.45 (95% UI: 49.07–153.83) per 100,000, respectively (Table 1). Between 1990 and 2021, there has been a notable decline in the number of deaths attributable to various pathogens (Figure 1A). *Rotavirus* and *Shigella* caused the greatest mortality in 2021, 119.86 thousand (95% UI: 83.14–169.41) and 81.77 thousand (95% UI: 47.92–137.95) deaths respectively, though their ASMRs declined substantially from 1990 (EAPC: −5.13, 95% CI: −5.54 to −4.72 and −4.23, 95% CI: −4.62 to −3.83). From 1990 to 2021, the proportion of deaths attributed to rotavirus significantly decreased from 25.58% to 21.30%, while the proportion of deaths attributed to *Shigella* increased from 12.33% to 14.53% during the same period (Figures 1B,C). *Cholera, Campylobacter* and *Entamoeba* showed the most rapid ASMR declines (EAPCs: −5.70, −5.69 and −5.12), respectively, while *Clostridioides difficile* demonstrated minimal change (EAPC: 0.18, 95% CI: 0.07 to 0.28) (Table 1).

**Table 1:** Global deaths of neonates and children under 5 years of age diarrheal diseases attributable to pathogen in 1990 and 2021, and the temporal trend from 1990 to 2021.

Category	Deaths (95% UI)				EAPC (95% CI)
	1990		2021		1990−2021
	Death cases, No.×103	ASMR per 100,000	Death cases, No.×103	ASMR per 100,000	
all pathogens	2,431.13 (1,462.19–3,748.43)	392.16 (235.86–604.65)	562.76 (317.95–922.19)	85.50 (48.31–140.11)	−4.80 (−5.17– −4.44)
sex					
Female	1,130.50 (672.74–1,762.03)	376.33 (223.95–586.56)	255.26 (142.38–417.58)	80.22 (44.75–131.23)	−4.85 (−5.27– −4.44)
Male	1,300.63 (745.44–2,063.04)	407.04 (233.29–645.63)	307.50 (166.83–523.00)	90.45 (49.07–153.83)	−4.76 (−5.08– −4.45)
13 pathogens					
Adenovirus	352.00 (202.70–559.70)	56.78 (32.70–90.28)	81.08 (44.88–133.05)	12.32 (6.82–20.21)	−4.98 (−5.20– −4.76)
Aeromonas	51.49 (22.46–93.91)	8.31 (3.62–15.15)	10.74 (4.80–20.18)	1.63 (0.73–3.07)	−5.05 (−5.30– −4.80)
Campylobacter	142.46 (63.06–262.88)	22.98 (10.17–42.40)	25.31 (10.26–48.75)	3.84 (1.56–7.41)	−5.69 (−6.00– −5.39)
Cholera	110.11 (91.44–132.85)	17.76 (14.75–21.43)	25.78 (18.51–34.92)	3.92 (2.81–5.31)	−5.70 (−6.41– −4.99)
Clostridioides difficile	0.10 (0.06–0.16)	0.02 (0.01–0.03)	0.10 (0.05–0.17)	0.02 (0.01–0.03)	0.18 (0.07–0.28)
Cryptosporidium	237.55 (146.64–371.65)	38.32 (23.65–59.95)	68.48 (38.91–109.57)	10.40 (5.91–16.65)	−4.07 (−4.54– −3.60)
Entamoeba	76.16 (36.45–142.78)	12.28 (5.88–23.03)	16.09 (7.06–32.34)	2.44 (1.07–4.91)	−5.12 (−5.36– −4.88)
Enteropathogenic E coli	166.32 (95.85–261.21)	26.83 (15.46–42.14)	46.37 (25.98–76.26)	7.04 (3.95–11.59)	−4.25 (−4.66– −3.83)
Enterotoxigenic E coli	184.26 (107.39–291.93)	29.72 (17.32–47.09)	45.80 (25.21–77.14)	6.96 (3.83–11.72)	−4.70 (−5.03– −4.36)
Non-typhoidal Salmonella	50.59 (7.44–110.16)	8.16 (1.20–17.77)	11.83 (1.58–27.44)	1.80 (0.24–4.17)	−4.67 (−4.94– −4.39)
Norovirus	138.53 (47.38–248.34)	22.35 (7.64–40.06)	29.56 (9.65–55.02)	4.49 (1.47–8.36)	−5.00 (−5.24– −4.75)
Rotavirus	621.83 (460.07–788.80)	100.31 (74.21–127.24)	119.86 (83.14–169.41)	18.21 (12.63–25.74)	−5.13 (−5.54– −4.72)
Shigella	299.74 (181.25–484.07)	48.35 (29.24–78.08)	81.77 (47.92–137.95)	12.42 (7.28–20.96)	−4.23 (−4.62– −3.83)

No., number; UI, uncertainty interval; ASMR, age-standardized mortality rate; EAPC, estimated annual percentage change; CI, confidence interval.

**Figure 1:**
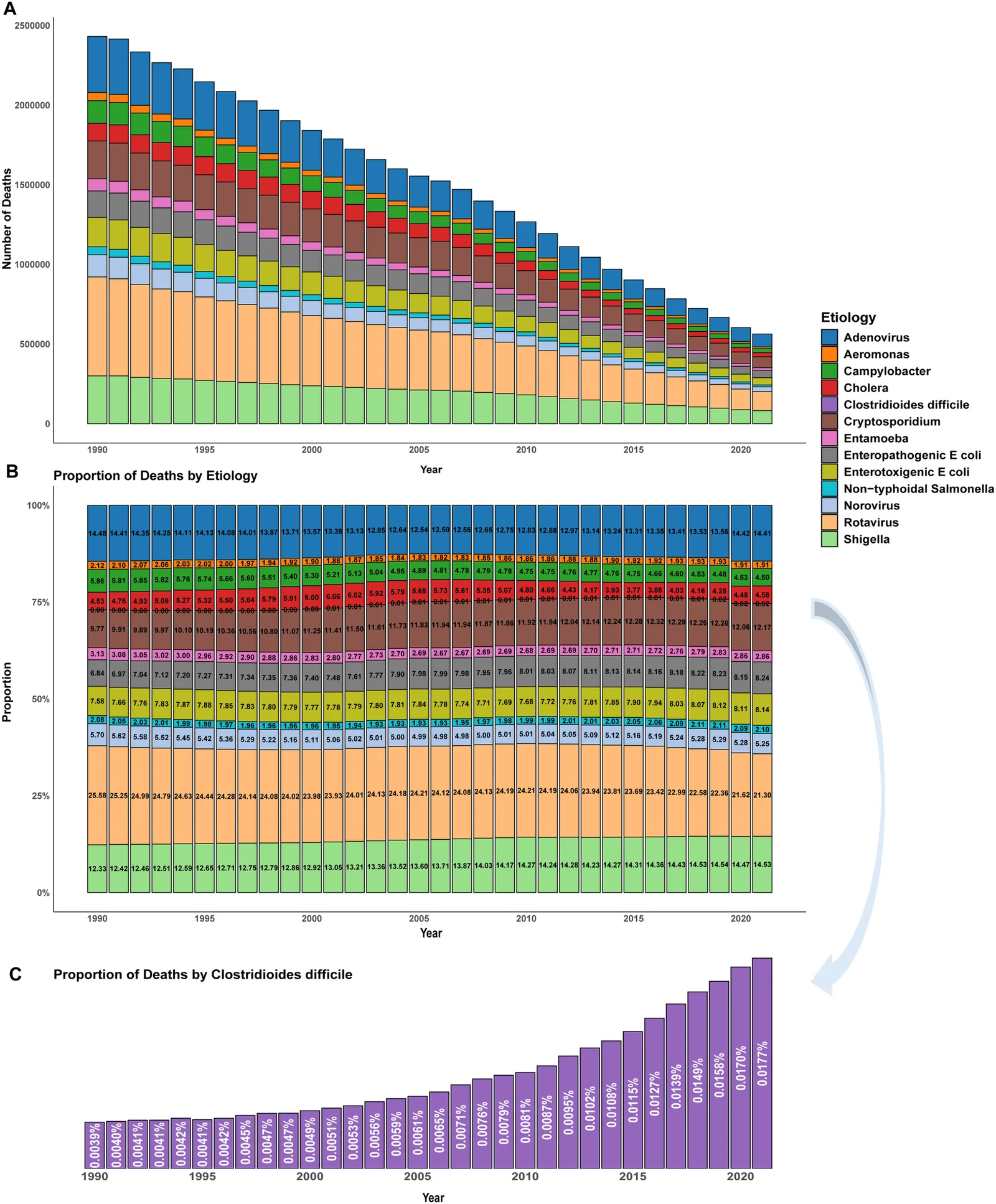
Global trends in diarrheal deaths by pathogen, 1990–2021. **(A)** Number of deaths attributable to each pathogen. **(B)** Relative contribution of each pathogen to total diarrheal deaths. **(C)** Proportion of diarrheal deaths attributable to Clostridioides difficile over time. Globally, total diarrheal deaths among children under five declined substantially from 1990 to 2021, while the relative contribution of most pathogens decreased. However, the proportion of deaths attributable to Clostridioides difficile showed a slight increase, contrasting with the overall downward trend. Although all 13 pathogens are included, Clostridioides difficile is not visually prominent in the bar plots due to relatively low death counts.

In 2021, the global burden of diarrheal diseases among children under five years attributable to 13 specific pathogens reached 50.63 million DALYs (95% UI 28.77–82.70 million), with ASDR of 7,692 per 100,000 population (95% UI 4,371.15–12,565.78). From 1990 to 2021, the overall burden showed a significant decline, with an EAPC of −4.79 (95% CI: −5.16 to −4.43). This downward trend was consistent across sexes, with females showing an EAPC of −4.84 (95% CI: −5.26 to −4.42) and males −4.75 (95% CI: −5.07 to −4.44). Among the 13 pathogens, *rotavirus* remained the leading contributor to DALYs in 2021, accounting for 10.79 million DALYs (95% UI: 7.52–15.20 million), followed by *Shigella* (7.34 million, 95% UI: 4.32–12.37 million) and *Cryptosporidium* (6.12 million, 95% UI: 3.50–9.78 million). While most pathogens showed notable reductions in DALYs over the study period, *Clostridioides difficile* exhibited a slight increase, with an EAPC of 0.17 (95% CI: 0.06 to 0.27) (Table 2).

**Table 2:** Global disability-adjusted life years of neonates and children under 5 years of age diarrheal diseases attributable to pathogen in 1990 and 2021, and the temporal trend from 1990 to 2021.

Category	DALYs (95% UI)				EAPC (95% CI)
	1990		2021		1990–2021
	DALY cases, No.×103	ASDR per 100,000	DALY cases, No.×103	ASDR per 100,000	
all pathogens	218,137.81 (131,580.90–335,904.20)	35,187.04 (21,224.85–54,183.52)	50,626.43 (28,769.67–82,704.41)	7,691.98 (4,371.15–12,565.78)	−4.79 (−5.16–−4.43)
sex					
Female	101,503.81 (60,713.35–157,920.16)	33,789.48 (20,210.79–52,569.84)	22,999.96 (12,902.85–37,497.48)	7,228.32 (4,055.05–11,784.53)	−4.84 (−5.26–−4.42)
Male	116,634.00 (67,129.01–184,635.74)	36,500.91 (21,008.19–57,782.22)	27,626.47 (15,082.31–46,850.13)	8,125.93 (4,436.24–13,780.29)	−4.75 (−5.07–−4.44)
13 pathogens					
Adenovirus	31,594.54 (18,205.50–50,373.02)	5,096.40 (2,936.66–8,125.49)	7,315.65 (4,046.72–11,970.48)	1,111.51 (614.84–1,818.75)	−4.96 (−5.18–−4.74)
Aeromonas	4,596.53 (2,006.35–8,406.55)	741.45 (323.64–1,356.03)	960.83 (432.38–1,806.73)	145.98 (65.69–274.51)	−5.04 (−5.29–−4.79)
Campylobacter	12,890.44 (5,698.83–23,647.18)	2,079.31 (919.26–3,814.44)	2,315.13 (978.99–4,411.46)	351.75 (148.74–670.26)	−5.66 (−5.96–−5.35)
Cholera	9,769.39 (8,111.56–11,796.11)	1,575.87 (1,308.45–1,902.79)	2,283.18 (1,638.81–3,093.65)	346.90 (248.99–470.04)	−5.71 (−6.42–−4.99)
Clostridioides difficile	8.42 (4.87–13.89)	1.36 (0.78–2.24)	8.78 (4.66–15.21)	1.33 (0.71–2.31)	0.17 (0.06–0.27)
Cryptosporidium	21,230.45 (13,120.04–33,181.11)	3,424.61 (2,116.35–5,352.33)	6,123.83 (3,495.92–9,775.96)	930.43 (531.16–1,485.32)	−4.06 (−4.53–−3.60)
Entamoeba	6,811.30 (3,300.20–12,738.81)	1,098.71 (532.34–2,054.85)	1,441.16 (636.07–2,883.09)	218.96 (96.64–438.05)	−5.12 (−5.36–−4.88)
Enteropathogenic E coli	14,928.96 (8,645.59–23,431.44)	2,408.14 (1,394.59–3,779.64)	4,169.50 (2,340.82–6,842.40)	633.50 (355.65–1,039.61)	−4.24 (−4.66–−3.82)
Enterotoxigenic E coli	16,567.59 (9,684.53–26,227.23)	2,672.46 (1,562.18–4,230.62)	4,129.55 (2,281.50–6,940.50)	627.43 (346.64–1,054.51)	−4.68 (−5.02–−4.34)
Non-typhoidal Salmonella	4,531.75 (701.87–9,812.96)	731.00 (113.22–1,582.89)	1,061.46 (155.45–2,447.92)	161.27 (23.62–371.93)	−4.66 (−4.94–−4.38)
Norovirus	12,488.30 (4,394.59–22,222.42)	2,014.44 (708.88–3,584.62)	2,684.02 (921.20–4,941.65)	407.80 (139.96–750.81)	−4.97 (−5.21–−4.72)
Rotavirus	55,840.43 (41,403.64–70,696.02)	9,007.42 (6,678.68–11,403.73)	10,788.94 (7,518.47–15,202.23)	1,639.23 (1,142.33–2,309.77)	−5.12 (−5.53–−4.70)
Shigella	26,879.73 (16,303.33–43,357.45)	4,335.87 (2,629.83–6,993.84)	7,344.39 (4,318.67–12,373.13)	1,115.88 (656.16–1,879.92)	−4.22 (−4.61–−3.82)

No., number; UI, uncertainty interval; DALYs, Disability-Adjusted Life Years; ASDR, age-standardized DALY rate; EAPC, estimated annual percentage change; CI, confidence interval.

### 3.2 Regional disparities in the burden of diarrheal diseases caused by pathogens

In 2021, the burden of diarrheal diseases among children under five years of age varied significantly across regions, with different pathogens dominating in each area. Despite the decline in its overall share of disease, *rotavirus* remained a significant contributor to diarrheal burden, with its highest DALY rate observed in Western Sub-Saharan Africa (6,859.81, 95% UI: 4,576.38–10,059.29), and substantial burden also present in Eastern Sub-Saharan Africa (3,157.20, 95% UI: 2,061.73–4,717.71). Among all regions, the burden of *Shigella*-attributable diarrheal disease was highest in Western Sub-Saharan Africa, with a DALY rate of 5,107.60 per 100,000 population (95% UI: 2,807.08–8,791.04), while *Entamoeba* had its greatest impact in Oceania, reporting a DALY rate of 1,052.31 (95% UI: 433.34–2,256.89). *Cholera* also contributed substantially in Oceania and the Caribbean, with DALY rate of 2,103.64 (95% UI: 1,406.34–2,843.18) and 1,534.37 (95% UI: 1,054.42–2,114.47), respectively. In contrast, the burden of *cholera* was negligible in Eastern Europe and the High-income Asia Pacific, with several regions reporting zero DALYs. *Non-typhoidal Salmonella* showed a substantial impact in Western Sub-Saharan Africa, with a DALY rate of 700.75 (95% UI: 88.95–1,679.92), and in Southern Sub-Saharan Africa, with a rate of 487.46 (95% UI: 92.58–1,080.78) (Figure 2, Supplementary Table S1).

**Figure 2:**
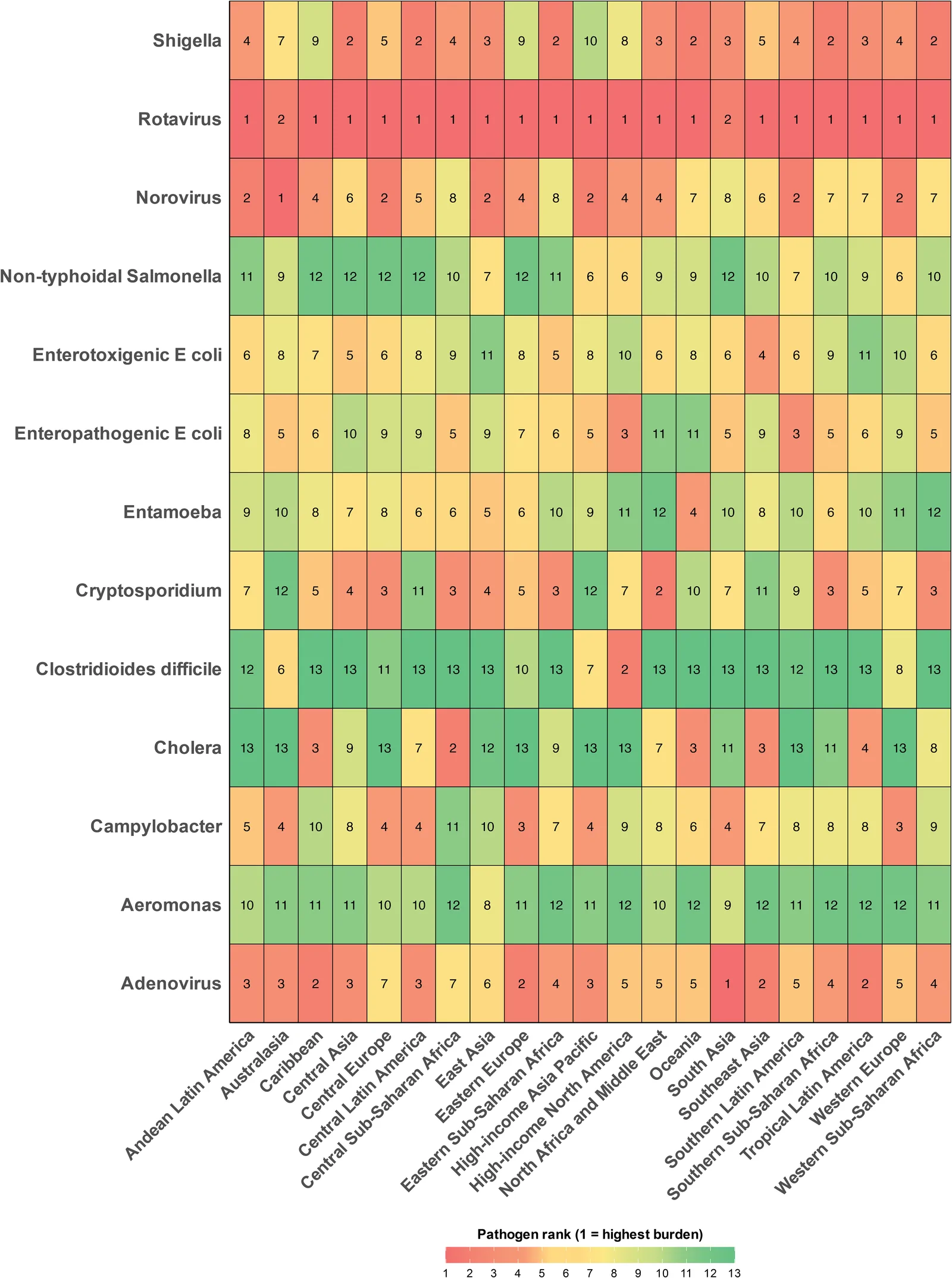
Regional ranking of pathogen-specific ASDR for diarrheal diseases among children younger than five years in 2021. Each cell displays the rank of the corresponding pathogen within each region (1 = highest ASDR and 13 = lowest ASDR), with the background color representing the same ranking scale. ASDR, age-standardized disability-adjusted life-year rate.

From 1990 to 2021, the ASDR attributable to diarrheal diseases in children under five declined globally, with an overall EAPC of −4.79 (95% CI: −5.16 to −4.43). However, the rate of decline varied considerably across the 21 GBD regions. East Asia experienced the most substantial reduction, with an EAPC of −13.50 (95% CI: −13.95 to −13.05), followed by Tropical Latin America (−11.13, 95% CI: −11.33 to −10.92) and Andean Latin America (−9.72, 95% CI: −10.01 to −9.43). In contrast, several high-income regions exhibited only modest decreases or statistically non-significant trends, such as Western Europe (−0.18, 95% CI: −0.89 to 0.54) and High-income Asia Pacific (−0.63, 95% CI: −0.93 to −0.32). Regions with historically high disease burden, such as Western Sub-Saharan Africa and Southern Sub-Saharan Africa, demonstrated moderate reductions in ASDR, with EAPCs of −4.59 and −3.81, respectively (Supplementary Table S2).

### 3.3 National variations in the burden of diarrheal diseases caused by pathogens

The ASMR associated with diarrheal diseases among children under five years varied significantly across countries in 2021. The highest ASMR was observed in Chad at 842.44 per 100,000 population (95% UI: 431.21–1,571.62), while the lowest was in Andorra at 0.05 per 100,000 population (95% UI: 0.02–0.08) (Figure 3A, Supplementary Table S3). Similarly, Chad exhibited the highest burden of DALYs, at 74,963.90 per 100,000 population (95% UI: 38,479.99–139,665.70), followed by South Sudan with 67,926.70 DALYs per 100,000 population (95% UI: 34,507.40–122,348.17). Other sub-Saharan African countries also demonstrated high DALY rates, including Nigeria (47,276.80 DALYs, 95% UI: 24,923.27–79,430.49) and Niger (41,874.43 DALYs, 95% UI: 21,295.99–74,929.87). In contrast, high-income countries, such as Monaco had significantly lower rates, with 111.06 DALYs (95% UI: 58.61–192.65) per 100,000 population (Figure 3B, Supplementary Table S4).

**Figure 3:**
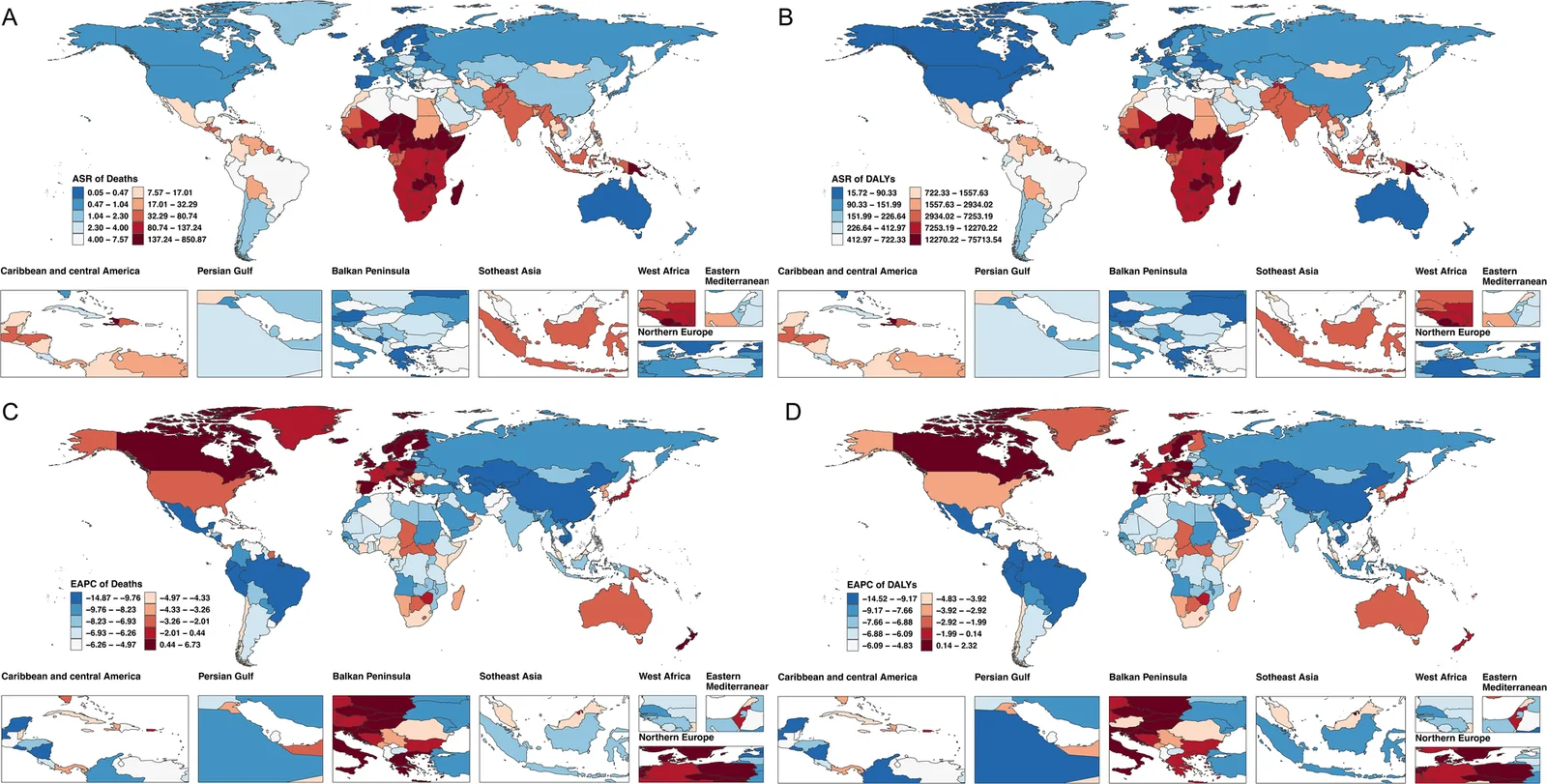
Global Maps of ASMR and ASDR in 2021 and EAPC of Deaths and DALYs from 1990 to 2021 for Pathogens Causing Diarrheal Disease. **(A)** ASMR **(B)** ASDR **(C)** EAPC of ASMR **(D)** EAPC of ASDR. ASMR, age-standardized mortality rate; ASDR, age-standardized DALY rate; EAPC, estimated annual percentage change; DALYs, disability-adjusted life years.

Between 1990 and 2021, the EAPC of ASMR due to diarrheal diseases attributed to pathogens in children under five years of age varied widely across countries. Armenia exhibited the largest decline (EAPC = −14.72, 95% CI: −15.64 to −13.79), followed closely by Uzbekistan (EAPC = −14.47, 95% CI: −15.14 to −13.79). In contrast, a few countries experienced increases in mortality, most notably Sweden, where the ASMR rose with an EAPC of 6.67 (95% CI: 5.52 to 7.82) (Figure 3C, Supplementary Table S5). Similarly, the EAPC of DALY for diarrheal diseases from 1990 to 2021 varies widely across countries. Some countries such as Uzbekistan, Armenia and Kazakhstan showed larger reductions in DALYs, with EAPCs of −14.37 (95% CI: −15.03 to −13.71), −14.33 (95% CI: −15.19 to −13.47), and −14.12 (95% CI: −14.93 to −13.30), respectively. Conversely, some high-income countries exhibited minimal or no significant changes. Countries like Japan and Republic of Korea showed a slight decline in DALYs, with EAPCs of −0.14 (95% CI: −0.24 to 0.52) and −3.20 (95% CI: −3.44 to −2.96), respectively (Figure 3D, Supplementary Table S6).

### 3.4 Age differences in the burden of diarrheal diseases caused by pathogens

In 2021, among all age groups under five years, children aged 1–5 months experienced the highest number of deaths attributable to diarrheal pathogens, *Rotavirus* was the leading cause, accounting for 35,793.70 deaths (95% UI: 25,261.06–48,787.67), followed by *Adenovirus* with 25,636.27 deaths (95% UI: 15,132.80–41,797.90), and *Shigella* with 21,587.21 deaths (95% UI: 11,365.50–37,790.22). In contrast, *Clostridioides difficile* contributed the fewest deaths across all age groups, with 50.99 deaths (95% UI: 25.71–89.91) reported in children aged 2–4 years, and only 0.09 deaths (95% UI: 0.04–0.18) among neonates aged 0–6 days (Figure 4A, Supplementary Table S7). *Rotavirus* also imposed the highest burden in terms of DALYs, with 3,234,300.78 DALYs (95% UI: 2,283,763.34–4,404,994.55) among children aged 1–5 months and 2,109,664.26 DALYs (95% UI: 1,333,663.63–3,134,654.54) in those aged 12–23 months. *Shigella* was the second leading contributor, responsible for 190,577.24 DALYs (95% UI: 89,265.34–347,700.01) in children aged 0–6 days, and 256,693.39 DALYs (95% UI: 125,959.34–473,790.66) in infants aged 7–27 days. *Non-typhoidal Salmonella* also demonstrated a substantial burden in older age groups, with 245,312.69 DALYs (95% UI: 672.58–741,853.29) in children aged 12–23 months and 212,686.60 DALYs (95% UI: 1,610.13–660,525.90) in those aged 2–4 years. In contrast, *Clostridioides difficile* exhibited the lowest DALY burden, with 8.24 DALYs (95% UI: 3.80–16.09) in neonates aged 0–6 days, and 78.85 DALYs (95% UI: 35.36–154.27) in infants aged 7–27 days. Despite the low overall DALY count, *Clostridioides difficile* demonstrated an increasing trend, especially in children aged 2–4 years, contributing to 4,432.52 DALYs (95% UI: 2,240.60–7,807.55) (Figure 4B, Supplementary Table S8).

**Figure 4:**
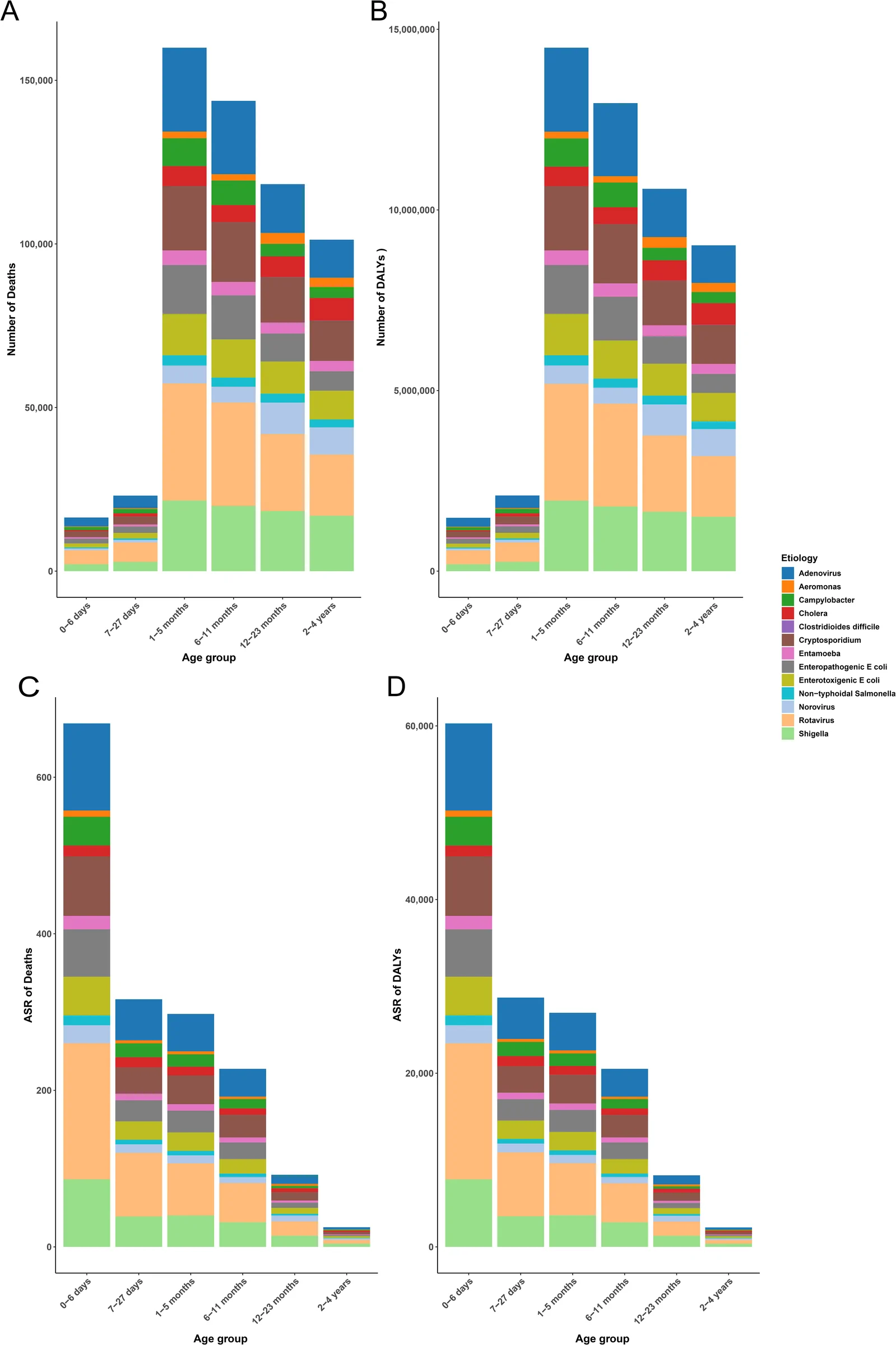
Global diarrhea-related mortality and DALYs in neonates and children under five years of Age attributable to specific pathogens in 2021. **(A)** Death Number **(B)** DALY Number **(C)** ASMR **(D)** ASDR. ASMR, age-standardized mortality rate; ASDR, age-standardized DALY rate; DALYs, disability-adjusted life years.

Regarding age-specific mortality rates per 100,000 population in 2021, *rotavirus* exhibited the highest rates, with 174.03 (95% UI: 114.60–266.68) in children aged 0–6 days and 81.38 (95% UI: 56.61–118.33) in those aged 7–27 days. *Shigella* was also a major contributor, with mortality rates of 86.28 (95% UI: 40.39–157.46) and 38.83 (95% UI: 19.01–71.81) per 100,000 in the same respective age groups. *Cryptosporidium* contributed 76.06 deaths (95% UI: 38.91–138.18) per 100,000 in neonates aged 0–6 days and 36.62 (95% UI: 17.99–57.53) in children aged 1–5 months. Other pathogens such as *enterotoxigenic E. coli* showed lower mortality rates, with 2.18 deaths (95% UI: 1.00–4.13) per 100,000 in children aged 2–4 years (Figure 4C, Supplementary Table S9). In terms of DALYs per 100,000 population, *rotavirus* remained the leading contributor across multiple age groups, with 15,682.02 DALYs (95% UI: 10,331.68–24,018.99) in children aged 0–6 days and 6,016.52 (95% UI: 4,248.31–8,194.27) in those aged 1–5 months. *Shigella* also caused substantial DALYs, particularly in the 0–6 days age group (7,774.80, 95% UI: 3,641.67–14,184.79) and in infants aged 1–5 months (3,631.29, 95% UI: 1,916.05–6,340.85). In contrast, *Clostridioides difficile* was responsible for minimal DALYs, with only 0.34 (95% UI: 0.15–0.66) in neonates aged 0–6 days. *Aeromonas* also showed a moderate contribution, with 62.95 DALYs (95% UI: 25.58–121.15) in children aged 2–4 years (Figure 4D, Supplementary Table S10).

### 3.5 Frontier analysis of the relationship between pathogen-specific DALY rates and country development status

Frontier analyses based on DALY rates and SDI were conducted for rotavirus, Shigella, and adenovirus from 1990 to 2021. In 2021, the largest frontier gaps for *rotavirus* were observed in Chad, South Sudan, the Central African Republic, Nigeria, and Madagascar, whereas several low-SDI countries, including Afghanistan, Somalia, and Mali, were located on or near the frontier (Figures 5A,B, Supplementary Table S11). For *Shigella*, the largest gaps were observed in Chad, the Central African Republic, South Sudan, Nigeria, and Lesotho, while Afghanistan and Somalia were among the low-SDI countries closest to the frontier (Figures 5C,D, Supplementary Table S12). For *adenovirus*, Chad, Nigeria, South Sudan, Haiti, and Benin showed the largest gaps, whereas Afghanistan and Somalia were located near the frontier (Figures 5E,F, Supplementary Table S13). Notably, substantial gaps were also observed in some high-SDI settings across the three pathogens, indicating that higher socio-demographic development did not uniformly correspond to lower pathogen-specific burden. Overall, many countries moved toward the frontier between 1990 and 2021 as SDI increased and DALY rates declined, although considerable gaps persisted in some settings.

**Figure 5:**
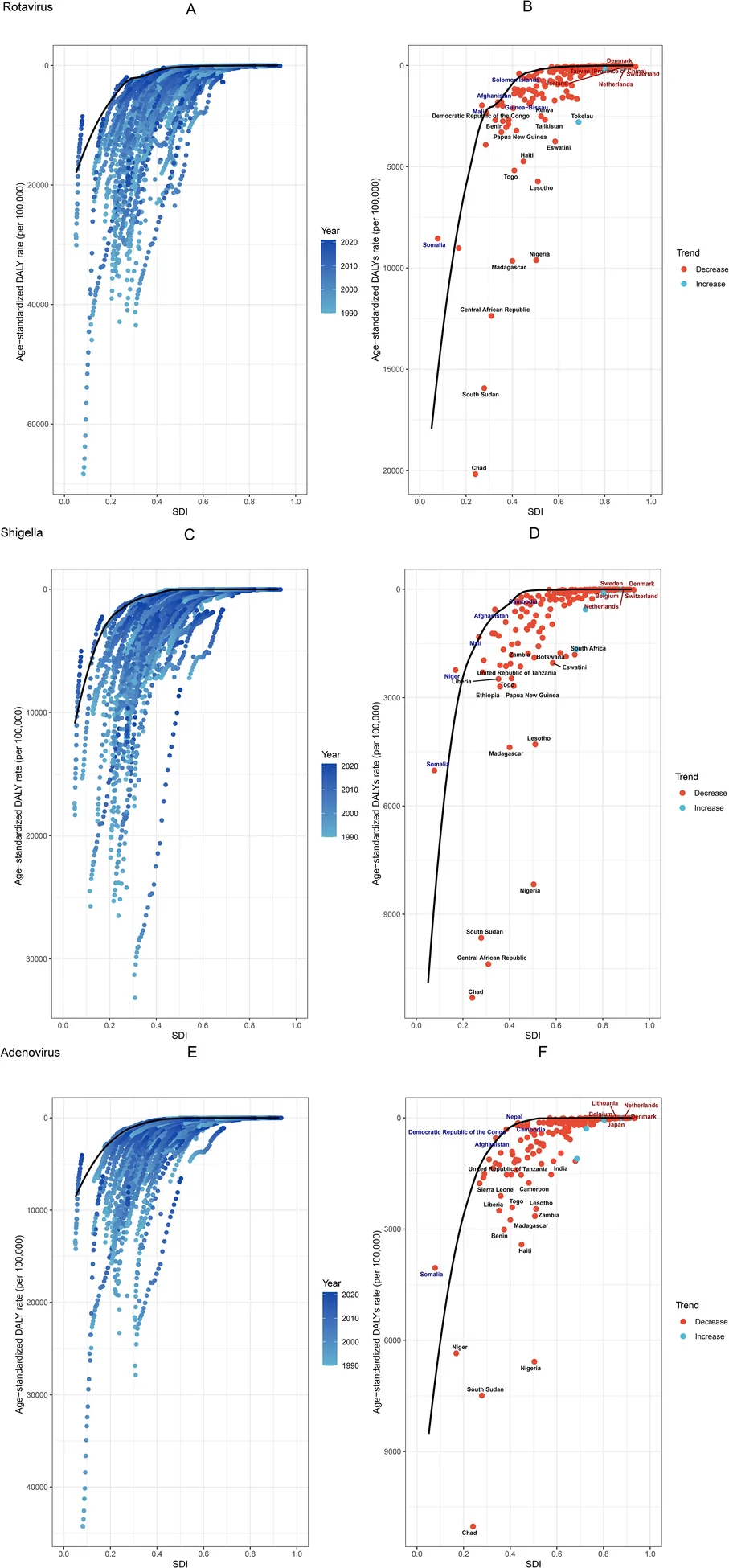
Frontier analysis based on SDI and DALYs for *rotavirus* associated diarrheal **(A,B)**, *Shigella* associated diarrheal **(C,D)**, and *adenovirus* associated diarrheal **(E,F)** in neonates and children under five years of Age in 204 countries and territories. In Figures A, C, and E the color change from light blue (1990) to dark blue (2021) represents the change in years. In Figures B, D, and F, each point represents a specific country or territory in 2021, the frontier line is shown in black, and the top 15 countries and territories with the largest differences from the frontier are marked in black. Blue represents low-SDI with the smallest differences from the frontier, red represents high-SDI with the largest differences from the frontier. Red dots indicate a decrease in DALYs, while blue dots indicate an increase in DALYs from 1990 to 2021. DALYs, disability-adjusted life years; SDI, socio-demographic index.

## 4 Discussion

Our analysis of GBD 2021 data reveals marked disparities in the burden of diarrheal diseases attributable to 13 major pathogens among neonates and children under five across global, regional, and national levels. Between 1990 and 2021, there was a marked global decline in both mortality and DALYs associated with diarrheal diseases, reflecting the significant impact of child health interventions.

Despite this progress, pathogens such as *rotavirus, Shigella,* and *Cryptosporidium* remain leading contributors to morbidity and mortality, particularly in low-SDI regions such as sub-Saharan Africa. Our findings also highlight significant age-related variation, with the highest number of deaths observed among children aged 1–5 months, indicating a critical period of vulnerability in early infancy. Furthermore, the frontier analysis identified notable room for improvement in both low- and high-SDI countries, suggesting that socioeconomic development alone does not fully account for the disparities in disease burden.

The observed global reduction in diarrheal disease burden aligns closely with findings from previous GBD iterations and epidemiological studies, reflecting improvements in sanitation, hygiene practices, vaccine coverage, and health infrastructure advancements (5, 19). In particular, the introduction and widespread use of rotavirus vaccines have played a pivotal role in reducing disease-associated morbidity and mortality (20, 21). Nonetheless, our study underscores that diarrheal diseases continue to pose major public health challenges in low-income settings, a finding consistent with recent reviews (22, 23).

Regional disparities observed in our analysis highlight uneven progress in diarrheal disease control. East Asia and Tropical Latin America exhibited the most significant declines, likely reflecting effective rotavirus vaccine rollout, improved sanitation, and enhanced child health systems (5). Conversely, regions such as Western Europe and High-income Asia Pacific showed minimal changes, potentially due to already low baseline burdens and residual cases linked to healthcare-associated infections (19). Meanwhile, moderate reductions in high-burden areas such as Western and Southern Sub-Saharan Africa highlight persistent challenges in health system capacity, undernutrition, and limited vaccine accessibility. These enduring inequalities continue to impede accelerated reductions in disease burden (15, 24, 25). Therefore, it is essential to have region-specific strategies that address vaccine access barriers, nutritional vulnerabilities, and water, sanitation, and hygiene (WASH) deficits differently in order to reduce current disparities and attain fair global diarrheal disease control (26).

Of particular concern is the rising burden of *Clostridioides difficile*, which contrasts with the declining trends of other major pathogens. This finding may reflect multiple interacting factors. First, inappropriate or excessive use of broad-spectrum antibiotics in both inpatient and outpatient settings likely increased susceptibility to *Clostridioides difficile* infection through microbiome disruption and reduced colonization resistance (27). Second, improvements in diagnostic capacity, increased clinician awareness, and the adoption of molecular detection techniques have enhanced case identification, particularly in high-income countries (28). Third, accumulating evidence indicates a growing proportion of community-acquired *Clostridioides difficile* infections, suggesting that transmission is no longer confined to hospital environments but may occur in households, daycare centers, and food chains (29). Furthermore, the developing gut microbiome in infants and young children, combined with frequent antibiotic exposure during early life, may heighten vulnerability to colonization and symptomatic infection (30).

The findings of this study have important implications for research, clinical practice, and policy. They reinforce the need for context-specific strategies that go beyond generalized interventions. Expanding immunization programs, improving sanitation and hygiene, and investing in targeted healthcare services are essential to achieving further reductions in disease burden. Given the substantial variation across regions, a one-size-fits-all approach is insufficient; instead, tailored national strategies are imperative (31, 32).

The sustained pathogen-specific burden highlights the limitations of current health interventions and necessitates targeted pathogen-specific strategies, including broader introduction and equitable distribution of existing vaccines (33, 34). From a policy perspective, substantial regional disparities suggest urgent resource allocation towards sanitation infrastructure, clean water access, and health education, especially in low-SDI countries (35, 36). The significant DALY burden in nations such as Chad, South Sudan, and Nigeria underscores a critical gap in healthcare access and intervention effectiveness, necessitating intensified international cooperation, increased funding, and strengthened health systems (37, 38).

Moreover, our frontier analysis, demonstrating that high-SDI countries like the Netherlands, Denmark, and Switzerland have significant room for improvement, challenges the prevailing assumption that diarrheal diseases are primarily issues in low-resource contexts (39, 40). Policymakers in these high-income nations should prioritize enhanced surveillance systems, targeted community-level interventions, and preventive strategies against emerging pathogens such as *Clostridioides difficile* (41, 42).

Although our study used publicly available data from GBD 2021, it provides several complementary contributions to the existing literature. First, building on the official GBD 2021 analysis and previous GBD-based studies of childhood diarrhea, we provide an integrated assessment of the burden attributable to 13 diarrheal pathogens among children under five years, with detailed characterization of pathogen-specific and age-specific patterns. Second, we adopted quantitative indicators of change (EAPC) to capture long-term trends, and further applied frontier analysis to link disease burden with the SDI, identifying frontier gaps and improvement potential across countries. Third, by conducting fine-grained age and sex stratifications, we revealed heterogeneity in both magnitude and trajectory of disease burden among different subpopulations.

The study period includes 2020–2021, coinciding with the onset of the COVID-19 pandemic, which may have indirectly affected diarrhea burden estimates. Although diarrhea can occur as a gastrointestinal manifestation of SARS-CoV-2 infection, the GBD 2021 modeling framework did not attribute COVID-19–related diarrhea as a separate cause (43). Instead, pandemic-related disruptions—such as altered healthcare-seeking behavior, reduced outpatient reporting, changes in hygiene practices, and restrictions on mobility—may have temporarily influenced data availability and transmission dynamics of enteric pathogens (44). Therefore, while our findings represent the overall diarrheal burden during this period, they should be interpreted with caution regarding the specific influence of COVID-19. Future GBD iterations incorporating pandemic-specific data will be essential to refine these estimates.

Although this study provides long-term global estimates, diarrheal pathogens often exhibit seasonal peaks influenced by temperature, rainfall, water quality, and sanitation patterns. *Rotavirus* infections, for example, typically surge during cooler and drier months in many regions, whereas bacterial pathogens such as *Shigella* tend to rise during warmer, rainy seasons (45, 46). Moreover, co-infection with multiple enteric pathogens is common among young children, particularly in low-resource settings, and may exacerbate clinical severity or complicate etiologic attribution in surveillance and modeling systems (47). While GBD 2021 does not explicitly model seasonal or co-infection patterns, recognizing these dynamics is essential for interpreting burden estimates and designing targeted prevention.

The findings of this study carry direct implications for global child health policy. The identification of *Rotavirus* and *Shigella* as leading contributors to diarrheal mortality supports the continued scale-up of rotavirus vaccine programs and underscores the importance of accelerating clinical development and introduction of *Shigella* vaccines in high-burden countries (4, 48). Meanwhile, the persistent regional inequalities linked to SDI highlight the need for sustained investment in WASH initiatives, including access to clean water, improved sanitation, and hygiene education—interventions proven to reduce diarrheal incidence at the community level (49). The observed increase in *Clostridioides difficile* burden further underscores the urgency of antimicrobial stewardship, particularly in pediatric populations, along with integrated hospital–community surveillance systems to monitor emerging enteric pathogens. Together, these measures can help countries close the gap toward achieving the SDG 3.2, which calls for ending preventable deaths among children under five by 2030.

This study presents several notable strengths. Foremost, it offers a comprehensive and systematic analysis based on high-quality global data from GBD 2021. The inclusion of age-specific, pathogen-specific, and regional disaggregation enables nuanced understanding of diarrheal disease burden, supporting targeted public health interventions. Additionally, the application of SDI-based frontier analysis provides a benchmarking framework for comparing pathogen-specific burden across countries at similar levels of socio-demographic development and identifying gaps between the observed burden and the empirical frontier (50).

## 5 Limitation

Several limitations should be considered when interpreting our findings. First, this study inherits the inherent limitations of the GBD 2021 modeling framework. Because GBD estimates are derived from heterogeneous data sources and rely partly on statistical modeling, particularly in data-sparse settings, they may either overestimate or underestimate the true disease burden, with potential bias varying across locations and pathogens. Second, limited diagnostic capacity and surveillance, pathogen misclassification, incomplete pathogen testing, and co-infections may affect the accuracy of pathogen-specific attribution, particularly in low-resource settings. Third, as an ecological secondary analysis of aggregated GBD estimates, this study cannot establish causal relationships between socio-demographic development and disease burden; therefore, the frontier analysis should be interpreted as a benchmarking approach for identifying potential improvement gaps rather than as evidence of causality.

## 6 Conclusion

This study complements previous GBD publications by providing an integrated assessment of pathogen-specific diarrheal disease burden among children younger than five years using GBD 2021 data. The combination of age-stratified analysis, EAPC-based trend quantification, and SDI frontier assessment provides additional insights into temporal, etiological, and socioeconomic heterogeneity in childhood diarrheal burden. These novel approaches identify persistent disparities, highlight frontier gaps between countries, and propose actionable priorities for accelerating progress toward child health goals under SDG 3.2.

## Data Availability

The original contributions presented in the study are included in the article/Supplementary Material, further inquiries can be directed to the corresponding authors.
