# Supplementary material for: Global burden of diarrheal diseases attributable to 13 pathogens in children under five: a comprehensive analysis from GBD 2021
Source: Front Pediatr. 2026 Sep 18;14:1846669. doi: 10.3389/fped.2026.1846669 (PMC13631008; doi:10.3389/fped.2026.1846669)
Supplement: Supplementary file 2 [file Supplementaryfile2.docx]

**Supplementary Code S1**

**Reproducible Bootstrap-Based Empirical Frontier Analysis**

**Description.** R code used to reproduce the bootstrap-based non-parametric empirical frontier analysis of pathogen-specific age-standardized DALY rates in relation to the Socio-demographic Index (SDI). The procedure uses 100 bootstrap resamples, an empirical monotonic lower envelope, and LOESS smoothing (degree = 1; span = 0.20). It does not fit a parametric stochastic frontier model; therefore, Cobb-Douglas and translog functional forms are not applicable.

**Software environment.** Verified in R 4.4.2; estimation uses functions from base R and the stats package.

**R code**

```
# Reproducible bootstrap-based empirical frontier analysis
#
# Purpose
# -------
# This script implements the non-parametric empirical burden-frontier method
# used for the SDI analysis. It does NOT fit a parametric stochastic frontier
# model; therefore, Cobb-Douglas and translog functional forms are not used.
#
# Required input columns
# ----------------------
# location_id : numeric or character location identifier
# year        : calendar year
# SDI         : Socio-demographic Index, normally in [0, 1]
# val         : age-standardized burden rate per 100,000 population
#
# Optional columns (retained in the output)
# ---------------------------------------------------------
# location_name, rei_name, measure_name, and other identifiers
#
# Verified environment
# --------------------
# R 4.5.0; estimation uses only functions from base R and stats.

estimate_empirical_frontier <- function(
  data,
  n_boot = 100L,
  span = 0.20,
  degree = 1L,
  seed = 20250826L
) {
  required <- c("location_id", "year", "SDI", "val")
  missing_columns <- setdiff(required, names(data))
  if (length(missing_columns) > 0L) {
    stop(
      "Missing required columns: ",
      paste(missing_columns, collapse = ", ")
    )
  }

  if (!is.numeric(data$SDI) || !is.numeric(data$val)) {
    stop("SDI and val must both be numeric.")
  }
  if (any(!is.finite(data$SDI)) || any(!is.finite(data$val))) {
    stop("SDI and val must not contain missing or non-finite values.")
  }
  if (nrow(data) < 10L) {
    stop("At least 10 complete country-year observations are required.")
  }
  if (n_boot < 2L) {
    stop("n_boot must be at least 2.")
  }
  if (span <= 0 || span > 1) {
    stop("span must be in the interval (0, 1].")
  }

  set.seed(seed)

  # Evaluate every bootstrap frontier at the SDI values observed in the
  # original data. Each bootstrap frontier is a monotone lower envelope:
  # after sorting observations by increasing SDI, the cumulative minimum of
  # the burden rate represents the lowest burden observed up to that SDI.
  target_sdi <- data$SDI
  boot_frontiers <- matrix(
    NA_real_,
    nrow = nrow(data),
    ncol = as.integer(n_boot)
  )

  for (b in seq_len(n_boot)) {
    sampled_rows <- sample.int(nrow(data), size = nrow(data), replace = TRUE)
    boot <- data[sampled_rows, c("SDI", "val"), drop = FALSE]
    boot <- boot[order(boot$SDI, -boot$val), , drop = FALSE]
    boot$lower_envelope <- cummin(boot$val)

    # Collapse duplicate SDI values and retain the lowest envelope at each SDI.
    envelope <- aggregate(
      lower_envelope ~ SDI,
      data = boot,
      FUN = min
    )
    envelope <- envelope[order(envelope$SDI), , drop = FALSE]

    # A right-continuous step interpolation evaluates the bootstrap lower
    # envelope at every original SDI value; rule = 2 carries endpoints outward.
    boot_frontiers[, b] <- stats::approx(
      x = envelope$SDI,
      y = envelope$lower_envelope,
      xout = target_sdi,
      method = "constant",
      f = 0,
      rule = 2,
      ties = "ordered"
    )$y
  }

  mean_bootstrap_frontier <- rowMeans(boot_frontiers)

  # Smooth the mean empirical frontier with a degree-one LOESS curve.
  loess_fit <- stats::loess(
    mean_bootstrap_frontier ~ target_sdi,
    degree = as.integer(degree),
    span = span,
    family = "gaussian",
    control = stats::loess.control(surface = "direct")
  )
  smoothed_frontier <- as.numeric(
    stats::predict(loess_fit, newdata = data.frame(target_sdi = target_sdi))
  )

  # If LOESS cannot predict an isolated boundary value, retain the unsmoothed
  # bootstrap estimate for that observation.
  failed_predictions <- !is.finite(smoothed_frontier)
  smoothed_frontier[failed_predictions] <-
    mean_bootstrap_frontier[failed_predictions]

  result <- data
  result$frontier_empirical <- mean_bootstrap_frontier
  result$frontier_smoothed <- pmax(smoothed_frontier, 0)
  result$efficiency_gap <- result$val - result$frontier_smoothed

  attr(result, "method") <- paste0(
    "Bootstrap empirical lower-envelope frontier; ",
    n_boot, " resamples; stats::loess degree = ", degree,
    ", span = ", span, "; seed = ", seed
  )
  result
}


run_frontier_by_group <- function(
  data,
  group_columns = intersect(c("rei_name", "measure_name"), names(data)),
  n_boot = 100L,
  span = 0.20,
  degree = 1L,
  seed = 20250826L
) {
  if (length(group_columns) == 0L) {
    return(
      estimate_empirical_frontier(
        data,
        n_boot = n_boot,
        span = span,
        degree = degree,
        seed = seed
      )
    )
  }

  missing_groups <- setdiff(group_columns, names(data))
  if (length(missing_groups) > 0L) {
    stop(
      "Missing grouping columns: ",
      paste(missing_groups, collapse = ", ")
    )
  }

  group_key <- interaction(data[group_columns], drop = TRUE, lex.order = TRUE)
  groups <- split(data, group_key, drop = TRUE)

  analysed <- Map(
    f = function(group_data, group_index) {
      estimate_empirical_frontier(
        group_data,
        n_boot = n_boot,
        span = span,
        degree = degree,
        seed = seed + group_index - 1L
      )
    },
    group_data = groups,
    group_index = seq_along(groups)
  )

  output <- do.call(rbind, analysed)
  rownames(output) <- NULL
  output
}


# Command-line use
# ----------------
# Rscript empirical_frontier_analysis.R input.csv output.csv
#
# The input CSV must already contain one row per country-year with columns
# location_id, year, SDI, and val. If rei_name and measure_name are present,
# the script estimates a separate frontier for every pathogen-outcome group.
if (sys.nframe() == 0L) {
  arguments <- commandArgs(trailingOnly = TRUE)

  if (length(arguments) == 0L) {
    message(
      "Script loaded successfully. Provide input.csv and output.csv to run ",
      "the analysis from the command line."
    )
  } else if (length(arguments) != 2L) {
    stop(
      "Usage: Rscript empirical_frontier_analysis.R input.csv output.csv"
    )
  } else {
    input_file <- arguments[[1L]]
    output_file <- arguments[[2L]]

    input_data <- utils::read.csv(
      input_file,
      check.names = FALSE,
      stringsAsFactors = FALSE
    )
    output_data <- run_frontier_by_group(input_data)
    utils::write.csv(output_data, output_file, row.names = FALSE)

    message("Saved frontier estimates to: ", normalizePath(output_file))
    message("R version: ", R.version.string)
  }
}
```
