# Supplementary material for: Global burden of diarrheal diseases attributable to 13 pathogens in children under five: a comprehensive analysis from GBD 2021
Source: Front Pediatr. 2026 Sep 18;14:1846669. doi: 10.3389/fped.2026.1846669 (PMC13631008; doi:10.3389/fped.2026.1846669)
Supplement: Supplementary file 3 [file Supplementaryfile1.docx]

**Supplementary Materials**

**Supplementary Appendix S1. Overview of the GBD 2021 methodology for estimating diarrheal disease burden**

The present study is an independent secondary analysis of publicly available estimates from the Global Burden of Disease Study 2021 (GBD 2021). The underlying estimates were generated within the GBD 2021 framework developed by the Institute for Health Metrics and Evaluation (IHME), and the original GBD estimation models were not reimplemented in the present study. The following sections summarize the principal methodological components relevant to the diarrheal disease and pathogen-specific estimates analyzed in this study, based on the official GBD 2021 Diarrhoeal Diseases publication and its supplementary appendix [1].

S1.1. Estimation of diarrheal mortality

GBD 2021 estimated mortality due to diarrheal diseases using the Cause of Death Ensemble model (CODEm). Input data included vital registration, sample-based vital registration, verbal autopsy, mortality surveillance, and minimally invasive tissue sampling. Data were processed and adjusted, where appropriate, for incomplete death registration, misclassification, and redistribution of insufficiently specific or implausible causes of death. CODEm evaluates multiple candidate models and constructs ensemble models by weighting component models according to their predictive validity. The final ensemble is selected based on out-of-sample predictive performance. For the final CODEm ensemble, 1,000 draws were generated, with the number of draws contributed by each component model proportional to its weight. The mean of these draws was used as the final CODEm mortality estimate, and the 2.5th and 97.5th percentiles were used to derive the corresponding 95% uncertainty interval (UI) [1].

S1.2. Estimation of non-fatal diarrheal burden

Incidence and prevalence data were obtained from multiple sources, including systematic reviews, population-based surveys, administrative claims, and inpatient records. GBD 2021 used DisMod-MR 2.1, a Bayesian meta-regression framework, to synthesize these heterogeneous epidemiological data and generate internally consistent estimates of diarrheal incidence and prevalence across locations, years, ages, and sexes. Differences in definitions and measurement methods across data sources were addressed within the GBD data-processing and modeling framework [1].

S1.3. Estimation of YLLs, YLDs, and DALYs

The overall health loss associated with diarrheal diseases was quantified using disability-adjusted life years (DALYs), calculated as:

DALYs = YLLs + YLDs

Years of life lost (YLLs) quantify premature mortality and were estimated using the number of deaths and the corresponding standard life expectancy at the age of death. Years lived with disability (YLDs) quantify non-fatal health loss and incorporate the prevalence and severity of diarrheal disease. Thus, DALYs integrate both fatal and non-fatal components of diarrheal disease burden [1].

S1.4. Pathogen-specific etiological attribution

After estimating the overall diarrheal disease burden, GBD 2021 attributed a proportion of this burden to specific pathogens using a counterfactual etiological attribution framework. This approach incorporated the prevalence of individual pathogens among diarrheal episodes and the pathogen-specific risk of diarrhea to estimate population-attributable fractions (PAFs). The resulting PAFs were applied to the corresponding overall diarrheal burden to derive pathogen-attributable estimates by location, year, age, and sex [1].

The present study focused on 13 diarrheal pathogens included in GBD 2021: adenovirus, Aeromonas spp., Campylobacter spp., Clostridioides difficile, Cryptosporidium spp., Entamoeba histolytica, enteropathogenic Escherichia coli, enterotoxigenic Escherichia coli, non-typhoidal Salmonella spp., norovirus, rotavirus, Shigella spp., and Vibrio cholerae.

S1.5. Estimation of uncertainty

GBD 2021 incorporated uncertainty arising from input data, data processing, and statistical modeling throughout the estimation framework using draw-based procedures. For the final CODEm ensemble, 1,000 draws were generated, and the 2.5th and 97.5th percentiles of the draws were used to derive the 95% uncertainty interval (UI) for mortality estimates. Uncertainty was also propagated through the estimation of non-fatal and pathogen-specific disease burden, allowing the reported UIs to reflect uncertainty from the relevant underlying data and modeling processes. Further details regarding uncertainty estimation and propagation at different stages of the GBD 2021 framework are provided in the official GBD 2021 methodological appendix [1].

S1.6. Data used in the present secondary analysi

For the present study, publicly available GBD 2021 estimates were extracted for neonates and children younger than five years across 204 countries and territories from 1990 to 2021. We analyzed deaths, mortality rates, DALYs, and DALY rates attributable to 13 diarrheal pathogens, stratified by location, year, age, and sex. The underlying CODEm, DisMod-MR 2.1, and etiological attribution models were not reconstructed or re-estimated in this study. Instead, the publicly available GBD 2021 estimates were used for subsequent descriptive analyses, temporal trend analyses using estimated annual percentage change (EAPC), and frontier analyses based on the Socio-demographic Index (SDI).

Complete details regarding data sources, data processing, model specifications, covariates, etiological attribution, and uncertainty estimation are available in the official GBD 2021 Diarrhoeal Diseases publication and its accompanying supplementary appendix [1].

[1] GBD 2021 Diarrhoeal Diseases Collaborators. Global, regional, and national age-sex-specific burden of diarrhoeal diseases, their risk factors, and aetiologies, 1990-2021, for 204 countries and territories: a systematic analysis for the Global Burden of Disease Study 2021. Lancet Infect Dis 2025;25:519–36. https://doi.org/10.1016/S1473-3099(24)00691-1.

**Figure S1. Workflow of the study assessing the burden of diarrheal diseases attributable to 13 pathogens in children under five**

| Table S1 ASDR Values and Rankings of 13 Diarrheal Pathogens in Neonates and Children Under Five in 21 Regions in 2021 | | | | | | |
| --- | --- | --- | --- | --- | --- | --- |
| location | age | pathogens | metric | year | rank | ASDR |
| Andean Latin America | <5 years | Rotavirus | Rate | 2021 | 1 | 222.65 (135.54, 332.11) |
| Andean Latin America | <5 years | Norovirus | Rate | 2021 | 2 | 137.62 (48.90, 241.06) |
| Andean Latin America | <5 years | Adenovirus | Rate | 2021 | 3 | 134.46 (66.68, 239.39) |
| Andean Latin America | <5 years | Shigella | Rate | 2021 | 4 | 108.11 (59.49, 189.67) |
| Andean Latin America | <5 years | Campylobacter | Rate | 2021 | 5 | 88.82 (36.19, 181.75) |
| Andean Latin America | <5 years | Enterotoxigenic E coli | Rate | 2021 | 6 | 65.18 (35.63, 117.45) |
| Andean Latin America | <5 years | Cryptosporidium | Rate | 2021 | 7 | 59.50 (32.48, 101.16) |
| Andean Latin America | <5 years | Enteropathogenic E coli | Rate | 2021 | 8 | 48.54 (25.55, 86.43) |
| Andean Latin America | <5 years | Entamoeba | Rate | 2021 | 9 | 28.42 (11.60, 61.98) |
| Andean Latin America | <5 years | Aeromonas | Rate | 2021 | 10 | 28.08 (12.25, 56.70) |
| Andean Latin America | <5 years | Non-typhoidal Salmonella | Rate | 2021 | 11 | 8.69 (1.23, 20.12) |
| Andean Latin America | <5 years | Clostridioides difficile | Rate | 2021 | 12 | 1.31 (0.57, 2.50) |
| Andean Latin America | <5 years | Cholera | Rate | 2021 | 13 | 0.00 (0.00, 0.00) |
| Australasia | <5 years | Norovirus | Rate | 2021 | 1 | 5.21 (2.59, 8.83) |
| Australasia | <5 years | Rotavirus | Rate | 2021 | 2 | 4.03 (2.95, 5.42) |
| Australasia | <5 years | Adenovirus | Rate | 2021 | 3 | 3.78 (2.09, 6.35) |
| Australasia | <5 years | Campylobacter | Rate | 2021 | 4 | 2.74 (1.50, 4.67) |
| Australasia | <5 years | Enteropathogenic E coli | Rate | 2021 | 5 | 2.50 (1.46, 4.00) |
| Australasia | <5 years | Clostridioides difficile | Rate | 2021 | 6 | 1.82 (1.04, 3.03) |
| Australasia | <5 years | Shigella | Rate | 2021 | 7 | 1.70 (0.99, 2.94) |
| Australasia | <5 years | Enterotoxigenic E coli | Rate | 2021 | 8 | 1.07 (0.65, 1.66) |
| Australasia | <5 years | Non-typhoidal Salmonella | Rate | 2021 | 9 | 1.01 (0.33, 2.01) |
| Australasia | <5 years | Entamoeba | Rate | 2021 | 10 | 0.62 (0.30, 1.15) |
| Australasia | <5 years | Aeromonas | Rate | 2021 | 11 | 0.44 (0.21, 0.75) |
| Australasia | <5 years | Cryptosporidium | Rate | 2021 | 12 | 0.41 (0.25, 0.66) |
| Australasia | <5 years | Cholera | Rate | 2021 | 13 | 0.00 (0.00, 0.00) |
| Caribbean | <5 years | Rotavirus | Rate | 2021 | 1 | 2294.25 (1481.13, 3284.18) |
| Caribbean | <5 years | Adenovirus | Rate | 2021 | 2 | 1566.52 (785.58, 2711.99) |
| Caribbean | <5 years | Cholera | Rate | 2021 | 3 | 1534.37 (1054.42, 2114.47) |
| Caribbean | <5 years | Norovirus | Rate | 2021 | 4 | 849.29 (287.86, 1642.34) |
| Caribbean | <5 years | Cryptosporidium | Rate | 2021 | 5 | 473.84 (251.91, 810.47) |
| Caribbean | <5 years | Enteropathogenic E coli | Rate | 2021 | 6 | 444.69 (227.72, 793.37) |
| Caribbean | <5 years | Enterotoxigenic E coli | Rate | 2021 | 7 | 431.51 (220.72, 735.94) |
| Caribbean | <5 years | Entamoeba | Rate | 2021 | 8 | 417.25 (171.62, 851.09) |
| Caribbean | <5 years | Shigella | Rate | 2021 | 9 | 354.27 (179.59, 665.31) |
| Caribbean | <5 years | Campylobacter | Rate | 2021 | 10 | 229.82 (92.48, 467.62) |
| Caribbean | <5 years | Aeromonas | Rate | 2021 | 11 | 179.34 (71.17, 355.04) |
| Caribbean | <5 years | Non-typhoidal Salmonella | Rate | 2021 | 12 | 178.10 (30.07, 384.48) |
| Caribbean | <5 years | Clostridioides difficile | Rate | 2021 | 13 | 1.63 (0.67, 3.15) |
| Central Asia | <5 years | Rotavirus | Rate | 2021 | 1 | 453.02 (298.41, 640.95) |
| Central Asia | <5 years | Shigella | Rate | 2021 | 2 | 219.27 (116.18, 372.20) |
| Central Asia | <5 years | Adenovirus | Rate | 2021 | 3 | 138.65 (73.65, 239.54) |
| Central Asia | <5 years | Cryptosporidium | Rate | 2021 | 4 | 127.42 (71.37, 211.31) |
| Central Asia | <5 years | Enterotoxigenic E coli | Rate | 2021 | 5 | 127.05 (66.78, 216.70) |
| Central Asia | <5 years | Norovirus | Rate | 2021 | 6 | 102.04 (38.96, 191.33) |
| Central Asia | <5 years | Entamoeba | Rate | 2021 | 7 | 61.65 (24.69, 122.46) |
| Central Asia | <5 years | Campylobacter | Rate | 2021 | 8 | 52.82 (20.44, 102.94) |
| Central Asia | <5 years | Cholera | Rate | 2021 | 9 | 44.97 (29.31, 63.13) |
| Central Asia | <5 years | Enteropathogenic E coli | Rate | 2021 | 10 | 42.36 (22.88, 73.45) |
| Central Asia | <5 years | Aeromonas | Rate | 2021 | 11 | 37.35 (14.60, 77.50) |
| Central Asia | <5 years | Non-typhoidal Salmonella | Rate | 2021 | 12 | 9.75 (1.89, 22.40) |
| Central Asia | <5 years | Clostridioides difficile | Rate | 2021 | 13 | 1.39 (0.62, 2.72) |
| Central Europe | <5 years | Rotavirus | Rate | 2021 | 1 | 134.25 (105.14, 170.86) |
| Central Europe | <5 years | Norovirus | Rate | 2021 | 2 | 21.71 (8.85, 38.95) |
| Central Europe | <5 years | Cryptosporidium | Rate | 2021 | 3 | 21.23 (12.55, 33.40) |
| Central Europe | <5 years | Campylobacter | Rate | 2021 | 4 | 19.86 (8.94, 36.91) |
| Central Europe | <5 years | Shigella | Rate | 2021 | 5 | 15.58 (8.56, 26.59) |
| Central Europe | <5 years | Enterotoxigenic E coli | Rate | 2021 | 6 | 14.59 (8.38, 23.77) |
| Central Europe | <5 years | Adenovirus | Rate | 2021 | 7 | 13.32 (7.21, 22.56) |
| Central Europe | <5 years | Entamoeba | Rate | 2021 | 8 | 12.30 (5.49, 24.76) |
| Central Europe | <5 years | Enteropathogenic E coli | Rate | 2021 | 9 | 7.86 (4.40, 13.70) |
| Central Europe | <5 years | Aeromonas | Rate | 2021 | 10 | 7.13 (2.94, 14.08) |
| Central Europe | <5 years | Clostridioides difficile | Rate | 2021 | 11 | 6.13 (3.51, 10.02) |
| Central Europe | <5 years | Non-typhoidal Salmonella | Rate | 2021 | 12 | 3.71 (0.80, 7.86) |
| Central Europe | <5 years | Cholera | Rate | 2021 | 13 | 0.00 (0.00, 0.00) |
| Central Latin America | <5 years | Rotavirus | Rate | 2021 | 1 | 432.31 (282.64, 624.78) |
| Central Latin America | <5 years | Shigella | Rate | 2021 | 2 | 233.60 (129.86, 401.34) |
| Central Latin America | <5 years | Adenovirus | Rate | 2021 | 3 | 168.82 (85.96, 286.84) |
| Central Latin America | <5 years | Campylobacter | Rate | 2021 | 4 | 144.11 (65.30, 273.56) |
| Central Latin America | <5 years | Norovirus | Rate | 2021 | 5 | 130.56 (42.59, 242.84) |
| Central Latin America | <5 years | Entamoeba | Rate | 2021 | 6 | 120.48 (55.16, 229.06) |
| Central Latin America | <5 years | Cholera | Rate | 2021 | 7 | 97.84 (68.56, 133.98) |
| Central Latin America | <5 years | Enterotoxigenic E coli | Rate | 2021 | 8 | 66.70 (37.50, 110.87) |
| Central Latin America | <5 years | Enteropathogenic E coli | Rate | 2021 | 9 | 52.08 (28.19, 88.42) |
| Central Latin America | <5 years | Aeromonas | Rate | 2021 | 10 | 38.12 (16.94, 73.21) |
| Central Latin America | <5 years | Cryptosporidium | Rate | 2021 | 11 | 35.40 (19.46, 60.73) |
| Central Latin America | <5 years | Non-typhoidal Salmonella | Rate | 2021 | 12 | 24.40 (3.55, 54.19) |
| Central Latin America | <5 years | Clostridioides difficile | Rate | 2021 | 13 | 2.99 (1.37, 5.67) |
| Central Sub-Saharan Africa | <5 years | Rotavirus | Rate | 2021 | 1 | 2889.82 (1608.85, 4921.46) |
| Central Sub-Saharan Africa | <5 years | Cholera | Rate | 2021 | 2 | 1489.29 (918.87, 2080.94) |
| Central Sub-Saharan Africa | <5 years | Cryptosporidium | Rate | 2021 | 3 | 1255.28 (642.21, 2238.18) |
| Central Sub-Saharan Africa | <5 years | Shigella | Rate | 2021 | 4 | 1181.52 (583.79, 2212.12) |
| Central Sub-Saharan Africa | <5 years | Enteropathogenic E coli | Rate | 2021 | 5 | 670.36 (326.92, 1266.62) |
| Central Sub-Saharan Africa | <5 years | Entamoeba | Rate | 2021 | 6 | 324.62 (128.24, 700.52) |
| Central Sub-Saharan Africa | <5 years | Adenovirus | Rate | 2021 | 7 | 323.00 (140.84, 627.62) |
| Central Sub-Saharan Africa | <5 years | Norovirus | Rate | 2021 | 8 | 316.04 (112.19, 641.59) |
| Central Sub-Saharan Africa | <5 years | Enterotoxigenic E coli | Rate | 2021 | 9 | 241.88 (117.27, 436.50) |
| Central Sub-Saharan Africa | <5 years | Non-typhoidal Salmonella | Rate | 2021 | 10 | 167.08 (27.00, 416.37) |
| Central Sub-Saharan Africa | <5 years | Campylobacter | Rate | 2021 | 11 | 165.10 (61.29, 361.74) |
| Central Sub-Saharan Africa | <5 years | Aeromonas | Rate | 2021 | 12 | 102.34 (40.75, 207.05) |
| Central Sub-Saharan Africa | <5 years | Clostridioides difficile | Rate | 2021 | 13 | 0.90 (0.35, 1.74) |
| East Asia | <5 years | Rotavirus | Rate | 2021 | 1 | 46.62 (33.01, 66.23) |
| East Asia | <5 years | Norovirus | Rate | 2021 | 2 | 11.15 (4.99, 19.46) |
| East Asia | <5 years | Shigella | Rate | 2021 | 3 | 11.11 (6.45, 18.90) |
| East Asia | <5 years | Cryptosporidium | Rate | 2021 | 4 | 9.78 (5.35, 17.37) |
| East Asia | <5 years | Entamoeba | Rate | 2021 | 5 | 7.97 (3.64, 14.97) |
| East Asia | <5 years | Adenovirus | Rate | 2021 | 6 | 6.15 (3.29, 10.32) |
| East Asia | <5 years | Non-typhoidal Salmonella | Rate | 2021 | 7 | 5.43 (1.39, 11.67) |
| East Asia | <5 years | Aeromonas | Rate | 2021 | 8 | 3.93 (1.76, 7.70) |
| East Asia | <5 years | Enteropathogenic E coli | Rate | 2021 | 9 | 3.48 (1.88, 6.27) |
| East Asia | <5 years | Campylobacter | Rate | 2021 | 10 | 2.55 (1.15, 4.96) |
| East Asia | <5 years | Enterotoxigenic E coli | Rate | 2021 | 11 | 2.00 (1.06, 3.62) |
| East Asia | <5 years | Cholera | Rate | 2021 | 12 | 0.60 (0.33, 1.04) |
| East Asia | <5 years | Clostridioides difficile | Rate | 2021 | 13 | 0.57 (0.20, 1.12) |
| Eastern Europe | <5 years | Rotavirus | Rate | 2021 | 1 | 23.75 (18.37, 30.43) |
| Eastern Europe | <5 years | Adenovirus | Rate | 2021 | 2 | 14.43 (7.96, 22.91) |
| Eastern Europe | <5 years | Campylobacter | Rate | 2021 | 3 | 13.04 (7.27, 22.30) |
| Eastern Europe | <5 years | Norovirus | Rate | 2021 | 4 | 9.70 (4.85, 15.88) |
| Eastern Europe | <5 years | Cryptosporidium | Rate | 2021 | 5 | 8.69 (5.19, 14.44) |
| Eastern Europe | <5 years | Entamoeba | Rate | 2021 | 6 | 4.59 (2.21, 8.42) |
| Eastern Europe | <5 years | Enteropathogenic E coli | Rate | 2021 | 7 | 4.57 (2.70, 7.28) |
| Eastern Europe | <5 years | Enterotoxigenic E coli | Rate | 2021 | 8 | 3.58 (2.23, 5.56) |
| Eastern Europe | <5 years | Shigella | Rate | 2021 | 9 | 3.47 (2.12, 5.78) |
| Eastern Europe | <5 years | Clostridioides difficile | Rate | 2021 | 10 | 3.38 (1.88, 5.43) |
| Eastern Europe | <5 years | Aeromonas | Rate | 2021 | 11 | 3.02 (1.52, 5.36) |
| Eastern Europe | <5 years | Non-typhoidal Salmonella | Rate | 2021 | 12 | 2.67 (0.87, 5.10) |
| Eastern Europe | <5 years | Cholera | Rate | 2021 | 13 | 0.00 (0.00, 0.00) |
| Eastern Sub-Saharan Africa | <5 years | Rotavirus | Rate | 2021 | 1 | 3157.20 (2061.73, 4717.71) |
| Eastern Sub-Saharan Africa | <5 years | Shigella | Rate | 2021 | 2 | 2449.87 (1355.38, 4288.42) |
| Eastern Sub-Saharan Africa | <5 years | Cryptosporidium | Rate | 2021 | 3 | 1900.47 (1069.56, 3118.16) |
| Eastern Sub-Saharan Africa | <5 years | Adenovirus | Rate | 2021 | 4 | 1692.78 (897.84, 2873.64) |
| Eastern Sub-Saharan Africa | <5 years | Enterotoxigenic E coli | Rate | 2021 | 5 | 1682.46 (909.89, 2916.62) |
| Eastern Sub-Saharan Africa | <5 years | Enteropathogenic E coli | Rate | 2021 | 6 | 1153.10 (637.71, 1947.50) |
| Eastern Sub-Saharan Africa | <5 years | Campylobacter | Rate | 2021 | 7 | 927.57 (373.62, 1833.30) |
| Eastern Sub-Saharan Africa | <5 years | Norovirus | Rate | 2021 | 8 | 816.95 (272.89, 1535.04) |
| Eastern Sub-Saharan Africa | <5 years | Cholera | Rate | 2021 | 9 | 802.64 (599.29, 1085.43) |
| Eastern Sub-Saharan Africa | <5 years | Entamoeba | Rate | 2021 | 10 | 726.07 (316.45, 1452.25) |
| Eastern Sub-Saharan Africa | <5 years | Non-typhoidal Salmonella | Rate | 2021 | 11 | 312.76 (43.86, 761.34) |
| Eastern Sub-Saharan Africa | <5 years | Aeromonas | Rate | 2021 | 12 | 154.64 (64.59, 301.45) |
| Eastern Sub-Saharan Africa | <5 years | Clostridioides difficile | Rate | 2021 | 13 | 0.91 (0.35, 1.75) |
| High-income Asia Pacific | <5 years | Rotavirus | Rate | 2021 | 1 | 53.91 (40.05, 74.42) |
| High-income Asia Pacific | <5 years | Norovirus | Rate | 2021 | 2 | 41.73 (16.47, 75.94) |
| High-income Asia Pacific | <5 years | Adenovirus | Rate | 2021 | 3 | 9.15 (4.29, 16.45) |
| High-income Asia Pacific | <5 years | Campylobacter | Rate | 2021 | 4 | 6.12 (3.32, 10.40) |
| High-income Asia Pacific | <5 years | Enteropathogenic E coli | Rate | 2021 | 5 | 5.67 (3.34, 9.16) |
| High-income Asia Pacific | <5 years | Non-typhoidal Salmonella | Rate | 2021 | 6 | 5.37 (2.44, 8.87) |
| High-income Asia Pacific | <5 years | Clostridioides difficile | Rate | 2021 | 7 | 5.04 (3.13, 7.81) |
| High-income Asia Pacific | <5 years | Enterotoxigenic E coli | Rate | 2021 | 8 | 4.14 (2.46, 6.90) |
| High-income Asia Pacific | <5 years | Entamoeba | Rate | 2021 | 9 | 3.45 (1.57, 6.67) |
| High-income Asia Pacific | <5 years | Shigella | Rate | 2021 | 10 | 2.99 (1.71, 5.40) |
| High-income Asia Pacific | <5 years | Aeromonas | Rate | 2021 | 11 | 1.46 (0.67, 2.77) |
| High-income Asia Pacific | <5 years | Cryptosporidium | Rate | 2021 | 12 | 1.12 (0.69, 1.79) |
| High-income Asia Pacific | <5 years | Cholera | Rate | 2021 | 13 | 0.00 (0.00, 0.00) |
| High-income North America | <5 years | Rotavirus | Rate | 2021 | 1 | 15.76 (11.78, 20.51) |
| High-income North America | <5 years | Clostridioides difficile | Rate | 2021 | 2 | 9.13 (6.01, 12.55) |
| High-income North America | <5 years | Enteropathogenic E coli | Rate | 2021 | 3 | 6.47 (3.55, 10.74) |
| High-income North America | <5 years | Norovirus | Rate | 2021 | 4 | 4.06 (1.96, 7.43) |
| High-income North America | <5 years | Adenovirus | Rate | 2021 | 5 | 3.94 (2.20, 6.41) |
| High-income North America | <5 years | Non-typhoidal Salmonella | Rate | 2021 | 6 | 2.38 (0.64, 4.84) |
| High-income North America | <5 years | Cryptosporidium | Rate | 2021 | 7 | 1.96 (1.18, 3.01) |
| High-income North America | <5 years | Shigella | Rate | 2021 | 8 | 1.35 (0.73, 2.39) |
| High-income North America | <5 years | Campylobacter | Rate | 2021 | 9 | 1.21 (0.57, 2.12) |
| High-income North America | <5 years | Enterotoxigenic E coli | Rate | 2021 | 10 | 1.14 (0.66, 1.89) |
| High-income North America | <5 years | Entamoeba | Rate | 2021 | 11 | 0.89 (0.42, 1.79) |
| High-income North America | <5 years | Aeromonas | Rate | 2021 | 12 | 0.66 (0.26, 1.31) |
| High-income North America | <5 years | Cholera | Rate | 2021 | 13 | 0.00 (0.00, 0.00) |
| North Africa and Middle East | <5 years | Rotavirus | Rate | 2021 | 1 | 530.26 (345.00, 871.41) |
| North Africa and Middle East | <5 years | Cryptosporidium | Rate | 2021 | 2 | 189.45 (110.52, 303.63) |
| North Africa and Middle East | <5 years | Shigella | Rate | 2021 | 3 | 140.82 (73.57, 264.55) |
| North Africa and Middle East | <5 years | Norovirus | Rate | 2021 | 4 | 136.10 (51.90, 277.99) |
| North Africa and Middle East | <5 years | Adenovirus | Rate | 2021 | 5 | 130.86 (65.30, 233.36) |
| North Africa and Middle East | <5 years | Enterotoxigenic E coli | Rate | 2021 | 6 | 123.66 (66.72, 213.64) |
| North Africa and Middle East | <5 years | Cholera | Rate | 2021 | 7 | 107.87 (72.45, 166.74) |
| North Africa and Middle East | <5 years | Campylobacter | Rate | 2021 | 8 | 104.71 (47.08, 197.76) |
| North Africa and Middle East | <5 years | Non-typhoidal Salmonella | Rate | 2021 | 9 | 69.24 (14.49, 154.26) |
| North Africa and Middle East | <5 years | Aeromonas | Rate | 2021 | 10 | 64.85 (27.01, 139.92) |
| North Africa and Middle East | <5 years | Enteropathogenic E coli | Rate | 2021 | 11 | 60.85 (31.64, 107.58) |
| North Africa and Middle East | <5 years | Entamoeba | Rate | 2021 | 12 | 52.37 (20.96, 107.32) |
| North Africa and Middle East | <5 years | Clostridioides difficile | Rate | 2021 | 13 | 0.63 (0.20, 1.26) |
| Oceania | <5 years | Rotavirus | Rate | 2021 | 1 | 2712.24 (1589.82, 4430.48) |
| Oceania | <5 years | Shigella | Rate | 2021 | 2 | 2258.75 (1154.31, 4344.76) |
| Oceania | <5 years | Cholera | Rate | 2021 | 3 | 2103.64 (1406.34, 2843.18) |
| Oceania | <5 years | Entamoeba | Rate | 2021 | 4 | 1052.31 (433.34, 2256.89) |
| Oceania | <5 years | Adenovirus | Rate | 2021 | 5 | 1027.12 (487.32, 1855.24) |
| Oceania | <5 years | Campylobacter | Rate | 2021 | 6 | 911.78 (327.78, 1840.81) |
| Oceania | <5 years | Norovirus | Rate | 2021 | 7 | 534.42 (154.86, 1039.27) |
| Oceania | <5 years | Enterotoxigenic E coli | Rate | 2021 | 8 | 423.12 (203.13, 782.76) |
| Oceania | <5 years | Non-typhoidal Salmonella | Rate | 2021 | 9 | 367.92 (39.97, 901.70) |
| Oceania | <5 years | Cryptosporidium | Rate | 2021 | 10 | 316.27 (153.61, 610.70) |
| Oceania | <5 years | Enteropathogenic E coli | Rate | 2021 | 11 | 251.61 (121.69, 467.05) |
| Oceania | <5 years | Aeromonas | Rate | 2021 | 12 | 214.71 (84.71, 437.44) |
| Oceania | <5 years | Clostridioides difficile | Rate | 2021 | 13 | 0.70 (0.21, 1.36) |
| South Asia | <5 years | Adenovirus | Rate | 2021 | 1 | 1199.12 (595.92, 2069.39) |
| South Asia | <5 years | Rotavirus | Rate | 2021 | 2 | 817.90 (464.12, 1272.21) |
| South Asia | <5 years | Shigella | Rate | 2021 | 3 | 585.44 (289.62, 1079.32) |
| South Asia | <5 years | Campylobacter | Rate | 2021 | 4 | 463.17 (191.91, 875.00) |
| South Asia | <5 years | Enteropathogenic E coli | Rate | 2021 | 5 | 419.24 (195.49, 754.68) |
| South Asia | <5 years | Enterotoxigenic E coli | Rate | 2021 | 6 | 352.88 (172.59, 618.47) |
| South Asia | <5 years | Cryptosporidium | Rate | 2021 | 7 | 347.40 (173.49, 619.15) |
| South Asia | <5 years | Norovirus | Rate | 2021 | 8 | 270.25 (96.48, 521.97) |
| South Asia | <5 years | Aeromonas | Rate | 2021 | 9 | 126.51 (52.24, 256.20) |
| South Asia | <5 years | Entamoeba | Rate | 2021 | 10 | 122.19 (50.10, 243.46) |
| South Asia | <5 years | Cholera | Rate | 2021 | 11 | 81.31 (49.49, 121.03) |
| South Asia | <5 years | Non-typhoidal Salmonella | Rate | 2021 | 12 | 70.58 (13.56, 163.37) |
| South Asia | <5 years | Clostridioides difficile | Rate | 2021 | 13 | 0.59 (0.20, 1.14) |
| Southeast Asia | <5 years | Rotavirus | Rate | 2021 | 1 | 966.18 (675.46, 1323.83) |
| Southeast Asia | <5 years | Adenovirus | Rate | 2021 | 2 | 439.92 (250.78, 723.06) |
| Southeast Asia | <5 years | Cholera | Rate | 2021 | 3 | 338.25 (240.21, 447.98) |
| Southeast Asia | <5 years | Enterotoxigenic E coli | Rate | 2021 | 4 | 335.25 (182.60, 562.63) |
| Southeast Asia | <5 years | Shigella | Rate | 2021 | 5 | 252.20 (138.19, 451.19) |
| Southeast Asia | <5 years | Norovirus | Rate | 2021 | 6 | 199.48 (87.47, 368.57) |
| Southeast Asia | <5 years | Campylobacter | Rate | 2021 | 7 | 132.77 (59.23, 261.74) |
| Southeast Asia | <5 years | Entamoeba | Rate | 2021 | 8 | 92.23 (43.30, 188.57) |
| Southeast Asia | <5 years | Enteropathogenic E coli | Rate | 2021 | 9 | 77.03 (43.43, 134.71) |
| Southeast Asia | <5 years | Non-typhoidal Salmonella | Rate | 2021 | 10 | 75.82 (15.60, 163.61) |
| Southeast Asia | <5 years | Cryptosporidium | Rate | 2021 | 11 | 73.46 (41.64, 121.95) |
| Southeast Asia | <5 years | Aeromonas | Rate | 2021 | 12 | 56.64 (25.44, 109.39) |
| Southeast Asia | <5 years | Clostridioides difficile | Rate | 2021 | 13 | 0.68 (0.25, 1.32) |
| Southern Latin America | <5 years | Rotavirus | Rate | 2021 | 1 | 51.77 (37.25, 70.70) |
| Southern Latin America | <5 years | Norovirus | Rate | 2021 | 2 | 21.32 (8.53, 37.30) |
| Southern Latin America | <5 years | Enteropathogenic E coli | Rate | 2021 | 3 | 20.90 (11.89, 33.31) |
| Southern Latin America | <5 years | Shigella | Rate | 2021 | 4 | 20.25 (11.58, 34.14) |
| Southern Latin America | <5 years | Adenovirus | Rate | 2021 | 5 | 18.98 (10.35, 30.96) |
| Southern Latin America | <5 years | Enterotoxigenic E coli | Rate | 2021 | 6 | 10.98 (6.57, 17.23) |
| Southern Latin America | <5 years | Non-typhoidal Salmonella | Rate | 2021 | 7 | 6.29 (1.35, 12.88) |
| Southern Latin America | <5 years | Campylobacter | Rate | 2021 | 8 | 4.83 (2.39, 8.80) |
| Southern Latin America | <5 years | Cryptosporidium | Rate | 2021 | 9 | 3.82 (2.23, 6.13) |
| Southern Latin America | <5 years | Entamoeba | Rate | 2021 | 10 | 3.10 (1.50, 6.07) |
| Southern Latin America | <5 years | Aeromonas | Rate | 2021 | 11 | 2.26 (1.03, 4.10) |
| Southern Latin America | <5 years | Clostridioides difficile | Rate | 2021 | 12 | 1.76 (0.96, 3.12) |
| Southern Latin America | <5 years | Cholera | Rate | 2021 | 13 | 0.00 (0.00, 0.00) |
| Southern Sub-Saharan Africa | <5 years | Rotavirus | Rate | 2021 | 1 | 1860.65 (1191.77, 2698.02) |
| Southern Sub-Saharan Africa | <5 years | Shigella | Rate | 2021 | 2 | 1679.03 (947.85, 2947.33) |
| Southern Sub-Saharan Africa | <5 years | Cryptosporidium | Rate | 2021 | 3 | 1626.24 (974.67, 2623.56) |
| Southern Sub-Saharan Africa | <5 years | Adenovirus | Rate | 2021 | 4 | 1145.51 (613.85, 1914.90) |
| Southern Sub-Saharan Africa | <5 years | Enteropathogenic E coli | Rate | 2021 | 5 | 903.89 (476.55, 1543.47) |
| Southern Sub-Saharan Africa | <5 years | Entamoeba | Rate | 2021 | 6 | 874.73 (385.38, 1704.60) |
| Southern Sub-Saharan Africa | <5 years | Norovirus | Rate | 2021 | 7 | 812.73 (305.21, 1534.35) |
| Southern Sub-Saharan Africa | <5 years | Campylobacter | Rate | 2021 | 8 | 732.03 (316.95, 1376.10) |
| Southern Sub-Saharan Africa | <5 years | Enterotoxigenic E coli | Rate | 2021 | 9 | 532.04 (292.07, 907.67) |
| Southern Sub-Saharan Africa | <5 years | Non-typhoidal Salmonella | Rate | 2021 | 10 | 487.46 (92.58, 1080.78) |
| Southern Sub-Saharan Africa | <5 years | Cholera | Rate | 2021 | 11 | 479.86 (310.20, 680.29) |
| Southern Sub-Saharan Africa | <5 years | Aeromonas | Rate | 2021 | 12 | 166.08 (66.52, 349.40) |
| Southern Sub-Saharan Africa | <5 years | Clostridioides difficile | Rate | 2021 | 13 | 1.22 (0.52, 2.29) |
| Tropical Latin America | <5 years | Rotavirus | Rate | 2021 | 1 | 80.57 (56.89, 113.13) |
| Tropical Latin America | <5 years | Adenovirus | Rate | 2021 | 2 | 69.12 (36.04, 116.63) |
| Tropical Latin America | <5 years | Shigella | Rate | 2021 | 3 | 65.13 (37.16, 115.95) |
| Tropical Latin America | <5 years | Cholera | Rate | 2021 | 4 | 64.27 (46.35, 84.00) |
| Tropical Latin America | <5 years | Cryptosporidium | Rate | 2021 | 5 | 33.93 (19.26, 57.39) |
| Tropical Latin America | <5 years | Enteropathogenic E coli | Rate | 2021 | 6 | 24.35 (13.36, 41.72) |
| Tropical Latin America | <5 years | Norovirus | Rate | 2021 | 7 | 23.02 (10.54, 41.54) |
| Tropical Latin America | <5 years | Campylobacter | Rate | 2021 | 8 | 21.49 (9.83, 39.50) |
| Tropical Latin America | <5 years | Non-typhoidal Salmonella | Rate | 2021 | 9 | 14.83 (3.23, 31.70) |
| Tropical Latin America | <5 years | Entamoeba | Rate | 2021 | 10 | 12.91 (5.80, 25.19) |
| Tropical Latin America | <5 years | Enterotoxigenic E coli | Rate | 2021 | 11 | 12.72 (7.25, 21.13) |
| Tropical Latin America | <5 years | Aeromonas | Rate | 2021 | 12 | 11.51 (5.15, 21.43) |
| Tropical Latin America | <5 years | Clostridioides difficile | Rate | 2021 | 13 | 1.42 (0.68, 2.64) |
| Western Europe | <5 years | Rotavirus | Rate | 2021 | 1 | 43.83 (31.31, 63.37) |
| Western Europe | <5 years | Norovirus | Rate | 2021 | 2 | 16.93 (7.72, 30.12) |
| Western Europe | <5 years | Campylobacter | Rate | 2021 | 3 | 11.57 (6.51, 18.65) |
| Western Europe | <5 years | Shigella | Rate | 2021 | 4 | 7.98 (4.64, 13.86) |
| Western Europe | <5 years | Adenovirus | Rate | 2021 | 5 | 7.55 (4.04, 12.58) |
| Western Europe | <5 years | Non-typhoidal Salmonella | Rate | 2021 | 6 | 6.70 (3.11, 11.08) |
| Western Europe | <5 years | Cryptosporidium | Rate | 2021 | 7 | 5.25 (3.09, 8.72) |
| Western Europe | <5 years | Clostridioides difficile | Rate | 2021 | 8 | 4.14 (2.66, 6.26) |
| Western Europe | <5 years | Enteropathogenic E coli | Rate | 2021 | 9 | 2.88 (1.69, 4.63) |
| Western Europe | <5 years | Enterotoxigenic E coli | Rate | 2021 | 10 | 2.46 (1.47, 4.07) |
| Western Europe | <5 years | Entamoeba | Rate | 2021 | 11 | 1.04 (0.48, 1.91) |
| Western Europe | <5 years | Aeromonas | Rate | 2021 | 12 | 0.88 (0.43, 1.73) |
| Western Europe | <5 years | Cholera | Rate | 2021 | 13 | 0.00 (0.00, 0.00) |
| Western Sub-Saharan Africa | <5 years | Rotavirus | Rate | 2021 | 1 | 6859.81 (4576.38, 10059.29) |
| Western Sub-Saharan Africa | <5 years | Shigella | Rate | 2021 | 2 | 5107.60 (2807.08, 8791.04) |
| Western Sub-Saharan Africa | <5 years | Cryptosporidium | Rate | 2021 | 3 | 4680.95 (2513.97, 7714.70) |
| Western Sub-Saharan Africa | <5 years | Adenovirus | Rate | 2021 | 4 | 4611.08 (2326.34, 7858.19) |
| Western Sub-Saharan Africa | <5 years | Enteropathogenic E coli | Rate | 2021 | 5 | 3031.27 (1563.99, 5143.79) |
| Western Sub-Saharan Africa | <5 years | Enterotoxigenic E coli | Rate | 2021 | 6 | 2597.75 (1389.49, 4464.52) |
| Western Sub-Saharan Africa | <5 years | Norovirus | Rate | 2021 | 7 | 1620.37 (484.94, 3052.13) |
| Western Sub-Saharan Africa | <5 years | Cholera | Rate | 2021 | 8 | 1122.87 (795.58, 1574.77) |
| Western Sub-Saharan Africa | <5 years | Campylobacter | Rate | 2021 | 9 | 848.89 (338.94, 1764.28) |
| Western Sub-Saharan Africa | <5 years | Non-typhoidal Salmonella | Rate | 2021 | 10 | 700.75 (88.95, 1679.92) |
| Western Sub-Saharan Africa | <5 years | Aeromonas | Rate | 2021 | 11 | 656.03 (271.26, 1290.79) |
| Western Sub-Saharan Africa | <5 years | Entamoeba | Rate | 2021 | 12 | 603.42 (254.77, 1202.93) |
| Western Sub-Saharan Africa | <5 years | Clostridioides difficile | Rate | 2021 | 13 | 0.94 (0.38, 1.84) |

| Table S2 EAPC of ASDR of Diarrheal Diseases in Neonates and Children Under Five in 21 Regions, 1990-2021 | | |
| --- | --- | --- |
| Location | Sex | EAPC of ASDR |
| Global | Both | -4.79 (-5.16, -4.43) |
| Andean Latin America | Both | -9.72 (-10.01, -9.43) |
| Australasia | Both | -1.75 (-2.60, -0.90) |
| Caribbean | Both | -2.78 (-3.32, -2.25) |
| Central Asia | Both | -7.86 (-8.17, -7.55) |
| Central Europe | Both | -2.47 (-3.55, -1.39) |
| Central Latin America | Both | -8.06 (-8.55, -7.56) |
| Central Sub-Saharan Africa | Both | -6.78 (-7.72, -5.84) |
| East Asia | Both | -13.50 (-13.95, -13.05) |
| Eastern Europe | Both | -7.71 (-8.26, -7.15) |
| Eastern Sub-Saharan Africa | Both | -5.67 (-5.86, -5.48) |
| High-income Asia Pacific | Both | -0.63 (-0.93, -0.32) |
| High-income North America | Both | -3.08 (-3.62, -2.53) |
| North Africa and Middle East | Both | -7.17 (-7.45, -6.88) |
| Oceania | Both | -2.01 (-2.19, -1.83) |
| South Asia | Both | -7.13 (-7.44, -6.83) |
| Southeast Asia | Both | -7.70 (-7.81, -7.59) |
| Southern Latin America | Both | -6.05 (-6.36, -5.73) |
| Southern Sub-Saharan Africa | Both | -3.81 (-4.33, -3.30) |
| Tropical Latin America | Both | -11.13 (-11.33, -10.92) |
| Western Europe | Both | -0.18 (-0.89, 0.54) |
| Western Sub-Saharan Africa | Both | -4.59 (-5.07, -4.11) |

Table S3 ASMR of Diarrheal Diseases in Neonates and Children Under Five Across Countries in 2021

| location | cause | age | metric | year | ASMR |
| --- | --- | --- | --- | --- | --- |
| China | Diarrheal diseases | <5 years | Rate | 2021 | 1.09 (0.59, 1.86) |
| Democratic People's Republic of Korea | Diarrheal diseases | <5 years | Rate | 2021 | 1.07 (0.40, 2.06) |
| Taiwan (Province of China) | Diarrheal diseases | <5 years | Rate | 2021 | 0.43 (0.27, 0.66) |
| Cambodia | Diarrheal diseases | <5 years | Rate | 2021 | 19.81 (11.42, 32.89) |
| Indonesia | Diarrheal diseases | <5 years | Rate | 2021 | 48.96 (27.93, 80.96) |
| Lao People's Democratic Republic | Diarrheal diseases | <5 years | Rate | 2021 | 59.27 (30.92, 103.08) |
| Malaysia | Diarrheal diseases | <5 years | Rate | 2021 | 4.55 (2.49, 7.62) |
| Maldives | Diarrheal diseases | <5 years | Rate | 2021 | 13.57 (7.56, 22.91) |
| Myanmar | Diarrheal diseases | <5 years | Rate | 2021 | 60.26 (34.15, 99.01) |
| Philippines | Diarrheal diseases | <5 years | Rate | 2021 | 25.33 (13.85, 42.82) |
| Sri Lanka | Diarrheal diseases | <5 years | Rate | 2021 | 3.84 (2.00, 6.69) |
| Thailand | Diarrheal diseases | <5 years | Rate | 2021 | 8.21 (4.40, 13.70) |
| Timor-Leste | Diarrheal diseases | <5 years | Rate | 2021 | 46.78 (22.79, 84.46) |
| Viet Nam | Diarrheal diseases | <5 years | Rate | 2021 | 1.40 (0.67, 2.47) |
| Fiji | Diarrheal diseases | <5 years | Rate | 2021 | 23.60 (10.15, 44.48) |
| Kiribati | Diarrheal diseases | <5 years | Rate | 2021 | 80.74 (37.89, 145.18) |
| Marshall Islands | Diarrheal diseases | <5 years | Rate | 2021 | 22.52 (11.04, 40.52) |
| Micronesia (Federated States of) | Diarrheal diseases | <5 years | Rate | 2021 | 15.19 (7.70, 26.40) |
| Papua New Guinea | Diarrheal diseases | <5 years | Rate | 2021 | 158.07 (78.32, 287.08) |
| Samoa | Diarrheal diseases | <5 years | Rate | 2021 | 5.81 (2.46, 10.72) |
| Solomon Islands | Diarrheal diseases | <5 years | Rate | 2021 | 32.29 (18.08, 52.83) |
| Tonga | Diarrheal diseases | <5 years | Rate | 2021 | 5.90 (2.86, 10.57) |
| Vanuatu | Diarrheal diseases | <5 years | Rate | 2021 | 36.82 (17.92, 71.06) |
| Armenia | Diarrheal diseases | <5 years | Rate | 2021 | 1.79 (0.96, 3.04) |
| Azerbaijan | Diarrheal diseases | <5 years | Rate | 2021 | 19.31 (9.53, 35.79) |
| Georgia | Diarrheal diseases | <5 years | Rate | 2021 | 0.71 (0.37, 1.23) |
| Kazakhstan | Diarrheal diseases | <5 years | Rate | 2021 | 1.44 (0.77, 2.43) |
| Kyrgyzstan | Diarrheal diseases | <5 years | Rate | 2021 | 5.42 (2.96, 9.13) |
| Mongolia | Diarrheal diseases | <5 years | Rate | 2021 | 8.13 (3.56, 16.56) |
| Tajikistan | Diarrheal diseases | <5 years | Rate | 2021 | 90.75 (47.61, 153.81) |
| Turkmenistan | Diarrheal diseases | <5 years | Rate | 2021 | 7.81 (4.26, 13.11) |
| Uzbekistan | Diarrheal diseases | <5 years | Rate | 2021 | 1.72 (0.92, 2.91) |
| Albania | Diarrheal diseases | <5 years | Rate | 2021 | 2.74 (1.22, 5.09) |
| Bosnia and Herzegovina | Diarrheal diseases | <5 years | Rate | 2021 | 1.89 (1.00, 3.44) |
| Bulgaria | Diarrheal diseases | <5 years | Rate | 2021 | 2.96 (1.64, 4.96) |
| Croatia | Diarrheal diseases | <5 years | Rate | 2021 | 3.15 (1.92, 4.89) |
| Czechia | Diarrheal diseases | <5 years | Rate | 2021 | 4.54 (2.66, 7.26) |
| Hungary | Diarrheal diseases | <5 years | Rate | 2021 | 4.67 (2.61, 7.69) |
| North Macedonia | Diarrheal diseases | <5 years | Rate | 2021 | 4.94 (2.55, 8.79) |
| Montenegro | Diarrheal diseases | <5 years | Rate | 2021 | 0.21 (0.10, 0.44) |
| Poland | Diarrheal diseases | <5 years | Rate | 2021 | 2.47 (1.53, 3.74) |
| Romania | Diarrheal diseases | <5 years | Rate | 2021 | 3.76 (2.19, 6.05) |
| Serbia | Diarrheal diseases | <5 years | Rate | 2021 | 1.23 (0.63, 2.25) |
| Slovakia | Diarrheal diseases | <5 years | Rate | 2021 | 1.62 (0.78, 3.04) |
| Slovenia | Diarrheal diseases | <5 years | Rate | 2021 | 0.66 (0.38, 1.09) |
| Belarus | Diarrheal diseases | <5 years | Rate | 2021 | 0.40 (0.21, 0.70) |
| Estonia | Diarrheal diseases | <5 years | Rate | 2021 | 0.20 (0.11, 0.34) |
| Latvia | Diarrheal diseases | <5 years | Rate | 2021 | 0.19 (0.10, 0.33) |
| Lithuania | Diarrheal diseases | <5 years | Rate | 2021 | 0.48 (0.26, 0.80) |
| Republic of Moldova | Diarrheal diseases | <5 years | Rate | 2021 | 2.16 (1.14, 3.80) |
| Russian Federation | Diarrheal diseases | <5 years | Rate | 2021 | 0.81 (0.44, 1.33) |
| Ukraine | Diarrheal diseases | <5 years | Rate | 2021 | 0.73 (0.43, 1.20) |
| Brunei Darussalam | Diarrheal diseases | <5 years | Rate | 2021 | 1.07 (0.59, 1.78) |
| Japan | Diarrheal diseases | <5 years | Rate | 2021 | 0.62 (0.35, 0.98) |
| Republic of Korea | Diarrheal diseases | <5 years | Rate | 2021 | 0.66 (0.38, 1.08) |
| Singapore | Diarrheal diseases | <5 years | Rate | 2021 | 0.47 (0.28, 0.72) |
| Australia | Diarrheal diseases | <5 years | Rate | 2021 | 0.12 (0.06, 0.22) |
| New Zealand | Diarrheal diseases | <5 years | Rate | 2021 | 0.58 (0.30, 0.98) |
| Andorra | Diarrheal diseases | <5 years | Rate | 2021 | 0.05 (0.02, 0.08) |
| Austria | Diarrheal diseases | <5 years | Rate | 2021 | 0.17 (0.09, 0.28) |
| Belgium | Diarrheal diseases | <5 years | Rate | 2021 | 1.00 (0.50, 1.74) |
| Cyprus | Diarrheal diseases | <5 years | Rate | 2021 | 0.83 (0.41, 1.49) |
| Denmark | Diarrheal diseases | <5 years | Rate | 2021 | 1.04 (0.58, 1.69) |
| Finland | Diarrheal diseases | <5 years | Rate | 2021 | 0.36 (0.20, 0.58) |
| France | Diarrheal diseases | <5 years | Rate | 2021 | 0.68 (0.39, 1.08) |
| Germany | Diarrheal diseases | <5 years | Rate | 2021 | 0.52 (0.29, 0.84) |
| Greece | Diarrheal diseases | <5 years | Rate | 2021 | 0.12 (0.07, 0.20) |
| Iceland | Diarrheal diseases | <5 years | Rate | 2021 | 0.42 (0.23, 0.69) |
| Ireland | Diarrheal diseases | <5 years | Rate | 2021 | 0.18 (0.09, 0.30) |
| Israel | Diarrheal diseases | <5 years | Rate | 2021 | 1.11 (0.64, 1.77) |
| Italy | Diarrheal diseases | <5 years | Rate | 2021 | 0.80 (0.43, 1.30) |
| Luxembourg | Diarrheal diseases | <5 years | Rate | 2021 | 0.56 (0.27, 0.95) |
| Malta | Diarrheal diseases | <5 years | Rate | 2021 | 0.28 (0.15, 0.45) |
| Netherlands | Diarrheal diseases | <5 years | Rate | 2021 | 0.55 (0.33, 0.84) |
| Norway | Diarrheal diseases | <5 years | Rate | 2021 | 0.20 (0.10, 0.35) |
| Portugal | Diarrheal diseases | <5 years | Rate | 2021 | 0.39 (0.21, 0.63) |
| Spain | Diarrheal diseases | <5 years | Rate | 2021 | 0.44 (0.26, 0.69) |
| Sweden | Diarrheal diseases | <5 years | Rate | 2021 | 0.43 (0.22, 0.73) |
| Switzerland | Diarrheal diseases | <5 years | Rate | 2021 | 0.53 (0.29, 0.88) |
| United Kingdom | Diarrheal diseases | <5 years | Rate | 2021 | 0.23 (0.12, 0.39) |
| Argentina | Diarrheal diseases | <5 years | Rate | 2021 | 1.61 (0.84, 2.69) |
| Chile | Diarrheal diseases | <5 years | Rate | 2021 | 1.47 (0.84, 2.34) |
| Uruguay | Diarrheal diseases | <5 years | Rate | 2021 | 3.16 (1.74, 5.33) |
| Canada | Diarrheal diseases | <5 years | Rate | 2021 | 0.62 (0.34, 1.06) |
| United States of America | Diarrheal diseases | <5 years | Rate | 2021 | 0.50 (0.30, 0.78) |
| Antigua and Barbuda | Diarrheal diseases | <5 years | Rate | 2021 | 4.67 (2.76, 7.29) |
| Bahamas | Diarrheal diseases | <5 years | Rate | 2021 | 2.33 (1.19, 4.04) |
| Barbados | Diarrheal diseases | <5 years | Rate | 2021 | 2.83 (1.54, 4.67) |
| Belize | Diarrheal diseases | <5 years | Rate | 2021 | 14.36 (8.66, 21.65) |
| Cuba | Diarrheal diseases | <5 years | Rate | 2021 | 2.62 (1.48, 4.17) |
| Dominica | Diarrheal diseases | <5 years | Rate | 2021 | 13.52 (6.66, 24.27) |
| Dominican Republic | Diarrheal diseases | <5 years | Rate | 2021 | 33.69 (16.29, 57.79) |
| Grenada | Diarrheal diseases | <5 years | Rate | 2021 | 4.00 (2.50, 5.99) |
| Guyana | Diarrheal diseases | <5 years | Rate | 2021 | 20.44 (10.93, 34.43) |
| Haiti | Diarrheal diseases | <5 years | Rate | 2021 | 211.43 (111.23, 353.02) |
| Jamaica | Diarrheal diseases | <5 years | Rate | 2021 | 6.87 (3.74, 11.35) |
| Saint Lucia | Diarrheal diseases | <5 years | Rate | 2021 | 7.83 (4.71, 12.38) |
| Saint Vincent and the Grenadines | Diarrheal diseases | <5 years | Rate | 2021 | 8.84 (5.02, 14.32) |
| Suriname | Diarrheal diseases | <5 years | Rate | 2021 | 42.96 (23.68, 70.64) |
| Trinidad and Tobago | Diarrheal diseases | <5 years | Rate | 2021 | 5.40 (2.96, 8.92) |
| Bolivia (Plurinational State of) | Diarrheal diseases | <5 years | Rate | 2021 | 25.61 (11.71, 46.60) |
| Ecuador | Diarrheal diseases | <5 years | Rate | 2021 | 4.31 (2.09, 7.63) |
| Peru | Diarrheal diseases | <5 years | Rate | 2021 | 7.29 (3.13, 13.86) |
| Colombia | Diarrheal diseases | <5 years | Rate | 2021 | 7.85 (3.95, 13.85) |
| Costa Rica | Diarrheal diseases | <5 years | Rate | 2021 | 2.73 (1.48, 4.52) |
| El Salvador | Diarrheal diseases | <5 years | Rate | 2021 | 9.57 (4.51, 17.30) |
| Guatemala | Diarrheal diseases | <5 years | Rate | 2021 | 57.76 (28.88, 101.50) |
| Honduras | Diarrheal diseases | <5 years | Rate | 2021 | 39.68 (18.95, 70.14) |
| Mexico | Diarrheal diseases | <5 years | Rate | 2021 | 10.37 (5.62, 17.63) |
| Nicaragua | Diarrheal diseases | <5 years | Rate | 2021 | 15.63 (7.52, 27.69) |
| Panama | Diarrheal diseases | <5 years | Rate | 2021 | 17.52 (8.92, 29.98) |
| Venezuela (Bolivarian Republic of) | Diarrheal diseases | <5 years | Rate | 2021 | 24.97 (11.02, 42.31) |
| Brazil | Diarrheal diseases | <5 years | Rate | 2021 | 4.41 (2.50, 7.26) |
| Paraguay | Diarrheal diseases | <5 years | Rate | 2021 | 8.31 (3.58, 16.55) |
| Algeria | Diarrheal diseases | <5 years | Rate | 2021 | 4.57 (1.79, 8.19) |
| Bahrain | Diarrheal diseases | <5 years | Rate | 2021 | 1.92 (0.96, 3.35) |
| Egypt | Diarrheal diseases | <5 years | Rate | 2021 | 21.42 (11.13, 37.70) |
| Iran (Islamic Republic of) | Diarrheal diseases | <5 years | Rate | 2021 | 2.30 (1.37, 3.54) |
| Iraq | Diarrheal diseases | <5 years | Rate | 2021 | 9.84 (4.87, 17.81) |
| Jordan | Diarrheal diseases | <5 years | Rate | 2021 | 2.76 (1.38, 4.93) |
| Kuwait | Diarrheal diseases | <5 years | Rate | 2021 | 0.88 (0.48, 1.47) |
| Lebanon | Diarrheal diseases | <5 years | Rate | 2021 | 7.57 (3.86, 13.37) |
| Libya | Diarrheal diseases | <5 years | Rate | 2021 | 5.92 (2.01, 11.33) |
| Morocco | Diarrheal diseases | <5 years | Rate | 2021 | 17.01 (7.88, 32.14) |
| Palestine | Diarrheal diseases | <5 years | Rate | 2021 | 2.69 (1.37, 4.75) |
| Oman | Diarrheal diseases | <5 years | Rate | 2021 | 4.58 (2.32, 8.24) |
| Qatar | Diarrheal diseases | <5 years | Rate | 2021 | 1.20 (0.57, 2.18) |
| Saudi Arabia | Diarrheal diseases | <5 years | Rate | 2021 | 2.79 (1.24, 5.18) |
| Syrian Arab Republic | Diarrheal diseases | <5 years | Rate | 2021 | 2.90 (1.25, 5.33) |
| Tunisia | Diarrheal diseases | <5 years | Rate | 2021 | 3.18 (1.07, 6.04) |
| Turkiye | Diarrheal diseases | <5 years | Rate | 2021 | 4.93 (2.54, 8.49) |
| United Arab Emirates | Diarrheal diseases | <5 years | Rate | 2021 | 2.11 (1.03, 3.80) |
| Yemen | Diarrheal diseases | <5 years | Rate | 2021 | 24.99 (7.35, 57.55) |
| Afghanistan | Diarrheal diseases | <5 years | Rate | 2021 | 67.97 (33.63, 125.34) |
| Bangladesh | Diarrheal diseases | <5 years | Rate | 2021 | 23.51 (11.09, 44.49) |
| Bhutan | Diarrheal diseases | <5 years | Rate | 2021 | 50.22 (19.97, 103.89) |
| India | Diarrheal diseases | <5 years | Rate | 2021 | 52.64 (24.29, 93.90) |
| Nepal | Diarrheal diseases | <5 years | Rate | 2021 | 20.21 (9.37, 37.16) |
| Pakistan | Diarrheal diseases | <5 years | Rate | 2021 | 73.05 (32.30, 139.23) |
| Angola | Diarrheal diseases | <5 years | Rate | 2021 | 84.33 (46.19, 138.92) |
| Central African Republic | Diarrheal diseases | <5 years | Rate | 2021 | 483.05 (253.57, 851.90) |
| Congo | Diarrheal diseases | <5 years | Rate | 2021 | 72.70 (29.42, 154.06) |
| Democratic Republic of the Congo | Diarrheal diseases | <5 years | Rate | 2021 | 88.16 (36.86, 176.31) |
| Equatorial Guinea | Diarrheal diseases | <5 years | Rate | 2021 | 28.79 (12.76, 54.94) |
| Gabon | Diarrheal diseases | <5 years | Rate | 2021 | 36.96 (10.95, 89.63) |
| Burundi | Diarrheal diseases | <5 years | Rate | 2021 | 148.71 (56.06, 333.52) |
| Comoros | Diarrheal diseases | <5 years | Rate | 2021 | 85.15 (33.98, 171.13) |
| Djibouti | Diarrheal diseases | <5 years | Rate | 2021 | 75.14 (29.98, 154.71) |
| Eritrea | Diarrheal diseases | <5 years | Rate | 2021 | 145.41 (66.70, 266.82) |
| Ethiopia | Diarrheal diseases | <5 years | Rate | 2021 | 140.30 (71.86, 248.72) |
| Kenya | Diarrheal diseases | <5 years | Rate | 2021 | 128.54 (67.64, 218.14) |
| Madagascar | Diarrheal diseases | <5 years | Rate | 2021 | 338.62 (153.62, 627.21) |
| Malawi | Diarrheal diseases | <5 years | Rate | 2021 | 139.65 (69.09, 254.16) |
| Mauritius | Diarrheal diseases | <5 years | Rate | 2021 | 8.28 (4.76, 13.01) |
| Mozambique | Diarrheal diseases | <5 years | Rate | 2021 | 127.80 (58.19, 245.28) |
| Rwanda | Diarrheal diseases | <5 years | Rate | 2021 | 103.67 (49.87, 192.18) |
| Seychelles | Diarrheal diseases | <5 years | Rate | 2021 | 5.88 (2.86, 10.49) |
| Somalia | Diarrheal diseases | <5 years | Rate | 2021 | 443.60 (223.48, 780.50) |
| United Republic of Tanzania | Diarrheal diseases | <5 years | Rate | 2021 | 116.90 (54.04, 221.22) |
| Uganda | Diarrheal diseases | <5 years | Rate | 2021 | 80.99 (32.48, 168.47) |
| Zambia | Diarrheal diseases | <5 years | Rate | 2021 | 168.95 (89.29, 294.37) |
| Botswana | Diarrheal diseases | <5 years | Rate | 2021 | 131.66 (61.14, 243.58) |
| Lesotho | Diarrheal diseases | <5 years | Rate | 2021 | 319.51 (147.77, 583.11) |
| Namibia | Diarrheal diseases | <5 years | Rate | 2021 | 123.50 (54.22, 230.16) |
| South Africa | Diarrheal diseases | <5 years | Rate | 2021 | 125.42 (65.34, 218.21) |
| Eswatini | Diarrheal diseases | <5 years | Rate | 2021 | 162.96 (76.11, 297.82) |
| Zimbabwe | Diarrheal diseases | <5 years | Rate | 2021 | 106.29 (47.94, 187.32) |
| Benin | Diarrheal diseases | <5 years | Rate | 2021 | 137.24 (56.30, 304.89) |
| Burkina Faso | Diarrheal diseases | <5 years | Rate | 2021 | 191.06 (92.46, 343.01) |
| Cameroon | Diarrheal diseases | <5 years | Rate | 2021 | 121.01 (47.00, 264.97) |
| Cabo Verde | Diarrheal diseases | <5 years | Rate | 2021 | 31.07 (15.28, 55.67) |
| Chad | Diarrheal diseases | <5 years | Rate | 2021 | 842.44 (431.21, 1571.62) |
| Cote d'Ivoire | Diarrheal diseases | <5 years | Rate | 2021 | 94.59 (43.51, 184.55) |
| Gambia | Diarrheal diseases | <5 years | Rate | 2021 | 89.45 (45.73, 154.43) |
| Ghana | Diarrheal diseases | <5 years | Rate | 2021 | 43.23 (18.64, 82.95) |
| Guinea | Diarrheal diseases | <5 years | Rate | 2021 | 91.64 (34.22, 197.31) |
| Guinea-Bissau | Diarrheal diseases | <5 years | Rate | 2021 | 83.51 (39.18, 155.48) |
| Liberia | Diarrheal diseases | <5 years | Rate | 2021 | 174.05 (70.78, 365.96) |
| Mali | Diarrheal diseases | <5 years | Rate | 2021 | 122.53 (61.63, 217.97) |
| Mauritania | Diarrheal diseases | <5 years | Rate | 2021 | 69.72 (27.06, 153.69) |
| Niger | Diarrheal diseases | <5 years | Rate | 2021 | 469.47 (237.69, 841.44) |
| Nigeria | Diarrheal diseases | <5 years | Rate | 2021 | 530.10 (278.35, 891.52) |
| Sao Tome and Principe | Diarrheal diseases | <5 years | Rate | 2021 | 22.58 (10.86, 41.70) |
| Senegal | Diarrheal diseases | <5 years | Rate | 2021 | 73.39 (34.98, 134.04) |
| Sierra Leone | Diarrheal diseases | <5 years | Rate | 2021 | 150.30 (72.16, 289.14) |
| Togo | Diarrheal diseases | <5 years | Rate | 2021 | 221.58 (99.72, 434.93) |
| American Samoa | Diarrheal diseases | <5 years | Rate | 2021 | 11.78 (5.79, 21.64) |
| Bermuda | Diarrheal diseases | <5 years | Rate | 2021 | 1.11 (0.66, 1.69) |
| Cook Islands | Diarrheal diseases | <5 years | Rate | 2021 | 2.72 (1.41, 4.64) |
| Greenland | Diarrheal diseases | <5 years | Rate | 2021 | 1.19 (0.64, 2.04) |
| Guam | Diarrheal diseases | <5 years | Rate | 2021 | 6.98 (3.51, 12.31) |
| Monaco | Diarrheal diseases | <5 years | Rate | 2021 | 0.25 (0.12, 0.44) |
| Nauru | Diarrheal diseases | <5 years | Rate | 2021 | 21.99 (11.02, 39.08) |
| Niue | Diarrheal diseases | <5 years | Rate | 2021 | 29.25 (12.32, 55.03) |
| Northern Mariana Islands | Diarrheal diseases | <5 years | Rate | 2021 | 4.34 (2.33, 7.34) |
| Palau | Diarrheal diseases | <5 years | Rate | 2021 | 15.03 (7.15, 27.48) |
| Puerto Rico | Diarrheal diseases | <5 years | Rate | 2021 | 2.58 (1.52, 4.10) |
| Saint Kitts and Nevis | Diarrheal diseases | <5 years | Rate | 2021 | 13.26 (7.26, 22.43) |
| San Marino | Diarrheal diseases | <5 years | Rate | 2021 | 0.12 (0.05, 0.24) |
| Tokelau | Diarrheal diseases | <5 years | Rate | 2021 | 109.97 (49.34, 208.34) |
| Tuvalu | Diarrheal diseases | <5 years | Rate | 2021 | 17.11 (8.97, 29.93) |
| United States Virgin Islands | Diarrheal diseases | <5 years | Rate | 2021 | 2.93 (1.49, 5.12) |
| South Sudan | Diarrheal diseases | <5 years | Rate | 2021 | 761.93 (386.29, 1373.21) |
| Sudan | Diarrheal diseases | <5 years | Rate | 2021 | 30.94 (10.04, 94.30) |

Table S4 ASDR of Diarrheal Diseases in Neonates and Children Under Five Across Countries in 2021

| location | cause | age | metric | year | ASDR |
| --- | --- | --- | --- | --- | --- |
| China | Diarrheal diseases | <5 years | Rate | 2021 | 109.15 (62.55, 181.87) |
| Democratic People's Republic of Korea | Diarrheal diseases | <5 years | Rate | 2021 | 170.07 (89.98, 290.40) |
| Taiwan (Province of China) | Diarrheal diseases | <5 years | Rate | 2021 | 202.92 (117.26, 341.89) |
| Cambodia | Diarrheal diseases | <5 years | Rate | 2021 | 1838.26 (1077.00, 3021.35) |
| Indonesia | Diarrheal diseases | <5 years | Rate | 2021 | 4583.09 (2665.75, 7511.18) |
| Lao People's Democratic Republic | Diarrheal diseases | <5 years | Rate | 2021 | 5422.08 (2858.68, 9375.97) |
| Malaysia | Diarrheal diseases | <5 years | Rate | 2021 | 511.97 (296.72, 827.21) |
| Maldives | Diarrheal diseases | <5 years | Rate | 2021 | 1361.88 (794.32, 2228.82) |
| Myanmar | Diarrheal diseases | <5 years | Rate | 2021 | 5622.51 (3250.31, 9126.03) |
| Philippines | Diarrheal diseases | <5 years | Rate | 2021 | 2370.22 (1326.44, 3948.14) |
| Sri Lanka | Diarrheal diseases | <5 years | Rate | 2021 | 428.27 (243.24, 704.90) |
| Thailand | Diarrheal diseases | <5 years | Rate | 2021 | 811.73 (461.33, 1316.18) |
| Timor-Leste | Diarrheal diseases | <5 years | Rate | 2021 | 4297.18 (2140.65, 7685.17) |
| Viet Nam | Diarrheal diseases | <5 years | Rate | 2021 | 204.51 (114.91, 332.61) |
| Fiji | Diarrheal diseases | <5 years | Rate | 2021 | 2205.20 (995.06, 4066.10) |
| Kiribati | Diarrheal diseases | <5 years | Rate | 2021 | 7253.19 (3433.90, 12988.51) |
| Marshall Islands | Diarrheal diseases | <5 years | Rate | 2021 | 2051.75 (1023.18, 3662.97) |
| Micronesia (Federated States of) | Diarrheal diseases | <5 years | Rate | 2021 | 1400.35 (729.48, 2405.50) |
| Papua New Guinea | Diarrheal diseases | <5 years | Rate | 2021 | 14260.63 (7137.31, 25784.12) |
| Samoa | Diarrheal diseases | <5 years | Rate | 2021 | 581.79 (273.38, 1034.01) |
| Solomon Islands | Diarrheal diseases | <5 years | Rate | 2021 | 2934.02 (1659.43, 4773.86) |
| Tonga | Diarrheal diseases | <5 years | Rate | 2021 | 571.61 (295.01, 992.90) |
| Vanuatu | Diarrheal diseases | <5 years | Rate | 2021 | 3364.31 (1674.57, 6421.19) |
| Armenia | Diarrheal diseases | <5 years | Rate | 2021 | 177.62 (100.51, 295.52) |
| Azerbaijan | Diarrheal diseases | <5 years | Rate | 2021 | 1750.58 (872.80, 3231.64) |
| Georgia | Diarrheal diseases | <5 years | Rate | 2021 | 81.63 (45.91, 135.44) |
| Kazakhstan | Diarrheal diseases | <5 years | Rate | 2021 | 143.43 (80.90, 237.20) |
| Kyrgyzstan | Diarrheal diseases | <5 years | Rate | 2021 | 499.53 (277.70, 836.08) |
| Mongolia | Diarrheal diseases | <5 years | Rate | 2021 | 736.09 (326.74, 1486.97) |
| Tajikistan | Diarrheal diseases | <5 years | Rate | 2021 | 8139.47 (4284.18, 13772.81) |
| Turkmenistan | Diarrheal diseases | <5 years | Rate | 2021 | 713.75 (394.20, 1190.46) |
| Uzbekistan | Diarrheal diseases | <5 years | Rate | 2021 | 160.28 (87.72, 267.52) |
| Albania | Diarrheal diseases | <5 years | Rate | 2021 | 254.40 (117.40, 465.03) |
| Bosnia and Herzegovina | Diarrheal diseases | <5 years | Rate | 2021 | 186.31 (102.73, 328.91) |
| Bulgaria | Diarrheal diseases | <5 years | Rate | 2021 | 274.36 (155.18, 456.20) |
| Croatia | Diarrheal diseases | <5 years | Rate | 2021 | 297.96 (185.60, 452.87) |
| Czechia | Diarrheal diseases | <5 years | Rate | 2021 | 416.66 (247.60, 662.12) |
| Hungary | Diarrheal diseases | <5 years | Rate | 2021 | 429.79 (245.19, 703.82) |
| North Macedonia | Diarrheal diseases | <5 years | Rate | 2021 | 457.45 (240.75, 805.03) |
| Montenegro | Diarrheal diseases | <5 years | Rate | 2021 | 31.71 (17.05, 55.08) |
| Poland | Diarrheal diseases | <5 years | Rate | 2021 | 226.64 (141.60, 340.40) |
| Romania | Diarrheal diseases | <5 years | Rate | 2021 | 347.86 (205.09, 554.28) |
| Serbia | Diarrheal diseases | <5 years | Rate | 2021 | 124.56 (68.61, 217.91) |
| Slovakia | Diarrheal diseases | <5 years | Rate | 2021 | 151.99 (75.55, 279.81) |
| Slovenia | Diarrheal diseases | <5 years | Rate | 2021 | 80.07 (49.07, 125.35) |
| Belarus | Diarrheal diseases | <5 years | Rate | 2021 | 83.90 (49.12, 133.46) |
| Estonia | Diarrheal diseases | <5 years | Rate | 2021 | 65.37 (35.94, 109.91) |
| Latvia | Diarrheal diseases | <5 years | Rate | 2021 | 46.16 (25.71, 76.51) |
| Lithuania | Diarrheal diseases | <5 years | Rate | 2021 | 92.05 (55.51, 145.37) |
| Republic of Moldova | Diarrheal diseases | <5 years | Rate | 2021 | 244.37 (141.19, 403.16) |
| Russian Federation | Diarrheal diseases | <5 years | Rate | 2021 | 94.49 (54.91, 151.47) |
| Ukraine | Diarrheal diseases | <5 years | Rate | 2021 | 89.90 (56.00, 140.37) |
| Brunei Darussalam | Diarrheal diseases | <5 years | Rate | 2021 | 102.51 (58.64, 167.40) |
| Japan | Diarrheal diseases | <5 years | Rate | 2021 | 169.42 (92.11, 282.52) |
| Republic of Korea | Diarrheal diseases | <5 years | Rate | 2021 | 71.29 (43.93, 111.36) |
| Singapore | Diarrheal diseases | <5 years | Rate | 2021 | 48.24 (30.25, 72.59) |
| Australia | Diarrheal diseases | <5 years | Rate | 2021 | 15.88 (8.74, 26.57) |
| New Zealand | Diarrheal diseases | <5 years | Rate | 2021 | 70.84 (40.10, 117.11) |
| Andorra | Diarrheal diseases | <5 years | Rate | 2021 | 101.58 (48.21, 185.81) |
| Austria | Diarrheal diseases | <5 years | Rate | 2021 | 63.29 (32.55, 110.24) |
| Belgium | Diarrheal diseases | <5 years | Rate | 2021 | 123.41 (69.74, 202.35) |
| Cyprus | Diarrheal diseases | <5 years | Rate | 2021 | 172.70 (98.61, 285.97) |
| Denmark | Diarrheal diseases | <5 years | Rate | 2021 | 184.45 (116.11, 286.88) |
| Finland | Diarrheal diseases | <5 years | Rate | 2021 | 103.54 (57.62, 174.44) |
| France | Diarrheal diseases | <5 years | Rate | 2021 | 175.13 (103.22, 285.28) |
| Germany | Diarrheal diseases | <5 years | Rate | 2021 | 90.33 (53.13, 145.46) |
| Greece | Diarrheal diseases | <5 years | Rate | 2021 | 61.01 (30.51, 109.22) |
| Iceland | Diarrheal diseases | <5 years | Rate | 2021 | 181.63 (102.67, 302.91) |
| Ireland | Diarrheal diseases | <5 years | Rate | 2021 | 80.37 (40.69, 142.37) |
| Israel | Diarrheal diseases | <5 years | Rate | 2021 | 154.02 (96.78, 240.99) |
| Italy | Diarrheal diseases | <5 years | Rate | 2021 | 107.83 (64.80, 169.38) |
| Luxembourg | Diarrheal diseases | <5 years | Rate | 2021 | 86.13 (48.29, 139.84) |
| Malta | Diarrheal diseases | <5 years | Rate | 2021 | 108.91 (59.59, 185.81) |
| Netherlands | Diarrheal diseases | <5 years | Rate | 2021 | 205.25 (115.60, 346.58) |
| Norway | Diarrheal diseases | <5 years | Rate | 2021 | 55.48 (28.98, 97.45) |
| Portugal | Diarrheal diseases | <5 years | Rate | 2021 | 86.64 (53.25, 136.01) |
| Spain | Diarrheal diseases | <5 years | Rate | 2021 | 111.49 (65.83, 180.12) |
| Sweden | Diarrheal diseases | <5 years | Rate | 2021 | 91.13 (51.63, 150.17) |
| Switzerland | Diarrheal diseases | <5 years | Rate | 2021 | 176.37 (103.35, 288.03) |
| United Kingdom | Diarrheal diseases | <5 years | Rate | 2021 | 48.74 (26.57, 81.70) |
| Argentina | Diarrheal diseases | <5 years | Rate | 2021 | 162.20 (89.82, 263.51) |
| Chile | Diarrheal diseases | <5 years | Rate | 2021 | 153.20 (92.25, 236.58) |
| Uruguay | Diarrheal diseases | <5 years | Rate | 2021 | 302.54 (172.30, 499.82) |
| Canada | Diarrheal diseases | <5 years | Rate | 2021 | 62.65 (36.05, 103.91) |
| United States of America | Diarrheal diseases | <5 years | Rate | 2021 | 47.54 (28.97, 72.83) |
| Antigua and Barbuda | Diarrheal diseases | <5 years | Rate | 2021 | 441.15 (265.70, 681.24) |
| Bahamas | Diarrheal diseases | <5 years | Rate | 2021 | 219.59 (115.67, 376.75) |
| Barbados | Diarrheal diseases | <5 years | Rate | 2021 | 284.43 (163.00, 459.52) |
| Belize | Diarrheal diseases | <5 years | Rate | 2021 | 1306.88 (795.80, 1966.55) |
| Cuba | Diarrheal diseases | <5 years | Rate | 2021 | 265.42 (158.68, 417.93) |
| Dominica | Diarrheal diseases | <5 years | Rate | 2021 | 1225.63 (612.33, 2186.81) |
| Dominican Republic | Diarrheal diseases | <5 years | Rate | 2021 | 3037.60 (1482.22, 5192.04) |
| Grenada | Diarrheal diseases | <5 years | Rate | 2021 | 382.77 (243.84, 569.41) |
| Guyana | Diarrheal diseases | <5 years | Rate | 2021 | 1833.53 (983.59, 3084.06) |
| Haiti | Diarrheal diseases | <5 years | Rate | 2021 | 18864.45 (9935.72, 31481.86) |
| Jamaica | Diarrheal diseases | <5 years | Rate | 2021 | 634.15 (350.58, 1040.08) |
| Saint Lucia | Diarrheal diseases | <5 years | Rate | 2021 | 729.32 (445.24, 1143.37) |
| Saint Vincent and the Grenadines | Diarrheal diseases | <5 years | Rate | 2021 | 810.75 (463.48, 1304.82) |
| Suriname | Diarrheal diseases | <5 years | Rate | 2021 | 3860.37 (2133.34, 6337.36) |
| Trinidad and Tobago | Diarrheal diseases | <5 years | Rate | 2021 | 500.75 (279.83, 821.18) |
| Bolivia (Plurinational State of) | Diarrheal diseases | <5 years | Rate | 2021 | 2305.39 (1070.17, 4179.22) |
| Ecuador | Diarrheal diseases | <5 years | Rate | 2021 | 412.97 (211.79, 714.84) |
| Peru | Diarrheal diseases | <5 years | Rate | 2021 | 695.18 (320.91, 1288.11) |
| Colombia | Diarrheal diseases | <5 years | Rate | 2021 | 742.33 (385.53, 1293.44) |
| Costa Rica | Diarrheal diseases | <5 years | Rate | 2021 | 288.98 (164.40, 466.50) |
| El Salvador | Diarrheal diseases | <5 years | Rate | 2021 | 877.67 (421.33, 1573.21) |
| Guatemala | Diarrheal diseases | <5 years | Rate | 2021 | 5187.44 (2609.03, 9090.32) |
| Honduras | Diarrheal diseases | <5 years | Rate | 2021 | 3580.52 (1728.99, 6305.18) |
| Mexico | Diarrheal diseases | <5 years | Rate | 2021 | 952.29 (521.81, 1605.96) |
| Nicaragua | Diarrheal diseases | <5 years | Rate | 2021 | 1418.58 (693.85, 2505.46) |
| Panama | Diarrheal diseases | <5 years | Rate | 2021 | 1642.03 (864.05, 2776.66) |
| Venezuela (Bolivarian Republic of) | Diarrheal diseases | <5 years | Rate | 2021 | 2273.07 (1025.22, 3838.72) |
| Brazil | Diarrheal diseases | <5 years | Rate | 2021 | 422.32 (244.54, 688.53) |
| Paraguay | Diarrheal diseases | <5 years | Rate | 2021 | 765.02 (338.70, 1502.04) |
| Algeria | Diarrheal diseases | <5 years | Rate | 2021 | 445.00 (192.27, 775.91) |
| Bahrain | Diarrheal diseases | <5 years | Rate | 2021 | 192.04 (102.62, 326.44) |
| Egypt | Diarrheal diseases | <5 years | Rate | 2021 | 2012.45 (1084.21, 3484.14) |
| Iran (Islamic Republic of) | Diarrheal diseases | <5 years | Rate | 2021 | 239.47 (148.10, 359.24) |
| Iraq | Diarrheal diseases | <5 years | Rate | 2021 | 922.84 (473.27, 1645.78) |
| Jordan | Diarrheal diseases | <5 years | Rate | 2021 | 275.10 (147.37, 479.12) |
| Kuwait | Diarrheal diseases | <5 years | Rate | 2021 | 114.64 (67.02, 183.03) |
| Lebanon | Diarrheal diseases | <5 years | Rate | 2021 | 730.52 (390.72, 1260.87) |
| Libya | Diarrheal diseases | <5 years | Rate | 2021 | 551.47 (202.18, 1035.47) |
| Morocco | Diarrheal diseases | <5 years | Rate | 2021 | 1557.63 (737.25, 2919.64) |
| Palestine | Diarrheal diseases | <5 years | Rate | 2021 | 255.13 (133.98, 441.50) |
| Oman | Diarrheal diseases | <5 years | Rate | 2021 | 440.03 (231.81, 771.53) |
| Qatar | Diarrheal diseases | <5 years | Rate | 2021 | 138.27 (73.26, 236.33) |
| Saudi Arabia | Diarrheal diseases | <5 years | Rate | 2021 | 278.19 (132.15, 498.94) |
| Syrian Arab Republic | Diarrheal diseases | <5 years | Rate | 2021 | 295.53 (143.55, 521.33) |
| Tunisia | Diarrheal diseases | <5 years | Rate | 2021 | 336.92 (142.32, 604.36) |
| T眉rkiye | Diarrheal diseases | <5 years | Rate | 2021 | 473.49 (254.81, 803.00) |
| United Arab Emirates | Diarrheal diseases | <5 years | Rate | 2021 | 214.04 (111.29, 375.65) |
| Yemen | Diarrheal diseases | <5 years | Rate | 2021 | 2304.76 (722.85, 5233.27) |
| Afghanistan | Diarrheal diseases | <5 years | Rate | 2021 | 6115.39 (3042.38, 11243.46) |
| Bangladesh | Diarrheal diseases | <5 years | Rate | 2021 | 2118.41 (1008.03, 3985.73) |
| Bhutan | Diarrheal diseases | <5 years | Rate | 2021 | 4501.08 (1806.00, 9279.98) |
| India | Diarrheal diseases | <5 years | Rate | 2021 | 4820.58 (2270.19, 8524.74) |
| Nepal | Diarrheal diseases | <5 years | Rate | 2021 | 1832.04 (862.45, 3351.67) |
| Pakistan | Diarrheal diseases | <5 years | Rate | 2021 | 6631.43 (2974.91, 12567.59) |
| Angola | Diarrheal diseases | <5 years | Rate | 2021 | 7588.09 (4176.60, 12453.39) |
| Central African Republic | Diarrheal diseases | <5 years | Rate | 2021 | 43078.18 (22651.07, 75803.38) |
| Congo | Diarrheal diseases | <5 years | Rate | 2021 | 6565.32 (2705.20, 13818.23) |
| Democratic Republic of the Congo | Diarrheal diseases | <5 years | Rate | 2021 | 7966.46 (3397.79, 15849.29) |
| Equatorial Guinea | Diarrheal diseases | <5 years | Rate | 2021 | 2627.32 (1190.68, 4961.89) |
| Gabon | Diarrheal diseases | <5 years | Rate | 2021 | 3371.63 (1049.55, 8069.04) |
| Burundi | Diarrheal diseases | <5 years | Rate | 2021 | 13353.46 (5117.49, 29770.77) |
| Comoros | Diarrheal diseases | <5 years | Rate | 2021 | 7705.48 (3126.47, 15351.24) |
| Djibouti | Diarrheal diseases | <5 years | Rate | 2021 | 6754.36 (2725.87, 13843.13) |
| Eritrea | Diarrheal diseases | <5 years | Rate | 2021 | 13013.97 (6014.48, 23799.89) |
| Ethiopia | Diarrheal diseases | <5 years | Rate | 2021 | 12601.34 (6494.57, 22261.80) |
| Kenya | Diarrheal diseases | <5 years | Rate | 2021 | 11641.29 (6172.54, 19710.71) |
| Madagascar | Diarrheal diseases | <5 years | Rate | 2021 | 30236.17 (13788.81, 55856.72) |
| Malawi | Diarrheal diseases | <5 years | Rate | 2021 | 12516.82 (6237.02, 22698.73) |
| Mauritius | Diarrheal diseases | <5 years | Rate | 2021 | 856.10 (516.44, 1318.08) |
| Mozambique | Diarrheal diseases | <5 years | Rate | 2021 | 11425.92 (5228.31, 21854.77) |
| Rwanda | Diarrheal diseases | <5 years | Rate | 2021 | 9282.30 (4491.24, 17166.55) |
| Seychelles | Diarrheal diseases | <5 years | Rate | 2021 | 564.73 (285.91, 989.59) |
| Somalia | Diarrheal diseases | <5 years | Rate | 2021 | 39589.57 (20004.22, 69568.95) |
| United Republic of Tanzania | Diarrheal diseases | <5 years | Rate | 2021 | 10455.32 (4859.32, 19742.68) |
| Uganda | Diarrheal diseases | <5 years | Rate | 2021 | 7279.73 (2956.12, 15083.33) |
| Zambia | Diarrheal diseases | <5 years | Rate | 2021 | 15160.93 (8056.46, 26337.58) |
| Botswana | Diarrheal diseases | <5 years | Rate | 2021 | 11780.30 (5508.52, 21748.40) |
| Lesotho | Diarrheal diseases | <5 years | Rate | 2021 | 28595.91 (13254.96, 52143.91) |
| Namibia | Diarrheal diseases | <5 years | Rate | 2021 | 11062.65 (4881.71, 20568.02) |
| South Africa | Diarrheal diseases | <5 years | Rate | 2021 | 11292.30 (5913.79, 19590.44) |
| Eswatini | Diarrheal diseases | <5 years | Rate | 2021 | 14626.25 (6868.76, 26681.18) |
| Zimbabwe | Diarrheal diseases | <5 years | Rate | 2021 | 9495.47 (4300.81, 16721.62) |
| Benin | Diarrheal diseases | <5 years | Rate | 2021 | 12270.22 (5077.39, 27124.91) |
| Burkina Faso | Diarrheal diseases | <5 years | Rate | 2021 | 17051.63 (8278.69, 30564.24) |
| Cameroon | Diarrheal diseases | <5 years | Rate | 2021 | 10856.87 (4267.69, 23676.11) |
| Cabo Verde | Diarrheal diseases | <5 years | Rate | 2021 | 2802.30 (1388.84, 5004.61) |
| Chad | Diarrheal diseases | <5 years | Rate | 2021 | 74963.90 (38479.99, 139665.70) |
| C么te d'Ivoire | Diarrheal diseases | <5 years | Rate | 2021 | 8484.22 (3932.09, 16490.43) |
| Gambia | Diarrheal diseases | <5 years | Rate | 2021 | 7964.87 (4081.84, 13729.31) |
| Ghana | Diarrheal diseases | <5 years | Rate | 2021 | 3890.58 (1692.16, 7440.54) |
| Guinea | Diarrheal diseases | <5 years | Rate | 2021 | 8218.31 (3107.61, 17625.45) |
| Guinea-Bissau | Diarrheal diseases | <5 years | Rate | 2021 | 7469.56 (3521.27, 13864.16) |
| Liberia | Diarrheal diseases | <5 years | Rate | 2021 | 15598.91 (6405.50, 32668.59) |
| Mali | Diarrheal diseases | <5 years | Rate | 2021 | 10962.50 (5543.47, 19455.64) |
| Mauritania | Diarrheal diseases | <5 years | Rate | 2021 | 6301.71 (2498.05, 13763.49) |
| Niger | Diarrheal diseases | <5 years | Rate | 2021 | 41874.43 (21295.99, 74929.87) |
| Nigeria | Diarrheal diseases | <5 years | Rate | 2021 | 47276.80 (24923.27, 79430.49) |
| Sao Tome and Principe | Diarrheal diseases | <5 years | Rate | 2021 | 2029.32 (982.16, 3738.96) |
| Senegal | Diarrheal diseases | <5 years | Rate | 2021 | 6627.73 (3190.02, 12022.92) |
| Sierra Leone | Diarrheal diseases | <5 years | Rate | 2021 | 13435.99 (6477.73, 25742.39) |
| Togo | Diarrheal diseases | <5 years | Rate | 2021 | 19808.03 (8971.23, 38774.73) |
| American Samoa | Diarrheal diseases | <5 years | Rate | 2021 | 1119.31 (571.33, 2016.75) |
| Bermuda | Diarrheal diseases | <5 years | Rate | 2021 | 143.15 (87.67, 217.24) |
| Cook Islands | Diarrheal diseases | <5 years | Rate | 2021 | 337.17 (186.41, 544.23) |
| Greenland | Diarrheal diseases | <5 years | Rate | 2021 | 110.82 (61.15, 187.31) |
| Guam | Diarrheal diseases | <5 years | Rate | 2021 | 722.33 (388.93, 1225.33) |
| Monaco | Diarrheal diseases | <5 years | Rate | 2021 | 111.06 (58.61, 192.65) |
| Nauru | Diarrheal diseases | <5 years | Rate | 2021 | 2029.29 (1051.41, 3567.93) |
| Niue | Diarrheal diseases | <5 years | Rate | 2021 | 2657.29 (1153.70, 4950.42) |
| Northern Mariana Islands | Diarrheal diseases | <5 years | Rate | 2021 | 480.55 (277.32, 783.09) |
| Palau | Diarrheal diseases | <5 years | Rate | 2021 | 1394.58 (676.56, 2513.95) |
| Puerto Rico | Diarrheal diseases | <5 years | Rate | 2021 | 261.33 (157.73, 408.92) |
| Saint Kitts and Nevis | Diarrheal diseases | <5 years | Rate | 2021 | 1211.28 (671.66, 2032.93) |
| San Marino | Diarrheal diseases | <5 years | Rate | 2021 | 91.84 (45.95, 162.39) |
| Tokelau | Diarrheal diseases | <5 years | Rate | 2021 | 9829.12 (4421.70, 18603.85) |
| Tuvalu | Diarrheal diseases | <5 years | Rate | 2021 | 1568.86 (837.00, 2720.93) |
| United States Virgin Islands | Diarrheal diseases | <5 years | Rate | 2021 | 278.81 (147.58, 475.58) |
| South Sudan | Diarrheal diseases | <5 years | Rate | 2021 | 67926.70 (34507.40, 122348.17) |
| Sudan | Diarrheal diseases | <5 years | Rate | 2021 | 2860.07 (985.21, 8502.70) |

Table S5 EAPC of ASMR of Diarrheal Diseases in Neonates and Children Under Five Across Countries, 1990-2021

| location | EAPC of ASMR |
| --- | --- |
| China | -13.96 (-14.43, -13.49) |
| Democratic People's Republic of Korea | -4.19 (-4.44, -3.94) |
| Taiwan (Province of China) | -6.73 (-7.60, -5.85) |
| Cambodia | -9.76 (-10.17, -9.34) |
| Indonesia | -7.92 (-8.08, -7.76) |
| Lao People's Democratic Republic | -8.86 (-9.19, -8.54) |
| Malaysia | -4.48 (-5.17, -3.79) |
| Maldives | -10.36 (-11.08, -9.64) |
| Myanmar | -8.80 (-9.02, -8.57) |
| Philippines | -6.15 (-6.34, -5.96) |
| Sri Lanka | -7.99 (-8.39, -7.59) |
| Thailand | -5.88 (-6.13, -5.63) |
| Timor-Leste | -9.26 (-9.57, -8.95) |
| Viet Nam | -10.38 (-10.70, -10.07) |
| Fiji | -2.36 (-2.76, -1.96) |
| Kiribati | -4.74 (-4.93, -4.55) |
| Marshall Islands | -3.66 (-4.40, -2.92) |
| Micronesia (Federated States of) | -4.97 (-6.04, -3.88) |
| Papua New Guinea | -2.35 (-2.54, -2.16) |
| Samoa | -3.11 (-3.41, -2.81) |
| Solomon Islands | -4.71 (-4.92, -4.51) |
| Tonga | -3.23 (-3.44, -3.02) |
| Vanuatu | -3.91 (-4.14, -3.69) |
| Armenia | -14.72 (-15.64, -13.79) |
| Azerbaijan | -6.69 (-6.96, -6.43) |
| Georgia | -13.56 (-14.27, -12.85) |
| Kazakhstan | -14.42 (-15.26, -13.56) |
| Kyrgyzstan | -9.86 (-10.65, -9.07) |
| Mongolia | -7.19 (-7.43, -6.95) |
| Tajikistan | -5.42 (-5.86, -4.98) |
| Turkmenistan | -12.53 (-13.21, -11.84) |
| Uzbekistan | -14.47 (-15.14, -13.79) |
| Albania | -6.08 (-6.75, -5.42) |
| Bosnia and Herzegovina | -1.55 (-1.90, -1.19) |
| Bulgaria | -0.34 (-1.40, 0.74) |
| Croatia | 2.24 (0.35, 4.16) |
| Czechia | 1.59 (-0.28, 3.49) |
| Hungary | 2.50 (0.64, 4.40) |
| North Macedonia | -6.45 (-7.46, -5.43) |
| Montenegro | -4.12 (-4.50, -3.74) |
| Poland | 2.25 (0.09, 4.46) |
| Romania | -4.86 (-5.76, -3.95) |
| Serbia | -4.04 (-4.68, -3.39) |
| Slovakia | 0.46 (-0.08, 1.00) |
| Slovenia | 0.85 (-0.80, 2.52) |
| Belarus | -9.20 (-10.14, -8.26) |
| Estonia | -10.37 (-11.01, -9.73) |
| Latvia | -10.09 (-10.65, -9.53) |
| Lithuania | -8.22 (-8.88, -7.57) |
| Republic of Moldova | -8.64 (-9.26, -8.02) |
| Russian Federation | -8.44 (-9.14, -7.74) |
| Ukraine | -8.23 (-9.15, -7.30) |
| Brunei Darussalam | 0.32 (0.09, 0.56) |
| Japan | -0.83 (-1.38, -0.29) |
| Republic of Korea | -3.62 (-3.87, -3.37) |
| Singapore | -3.30 (-4.02, -2.57) |
| Australia | -2.69 (-3.62, -1.75) |
| New Zealand | 1.37 (-0.42, 3.19) |
| Andorra | -3.27 (-3.56, -2.97) |
| Austria | -0.90 (-1.89, 0.09) |
| Belgium | 0.44 (-0.40, 1.29) |
| Cyprus | -5.06 (-5.23, -4.90) |
| Denmark | 1.29 (0.44, 2.15) |
| Finland | 0.45 (-0.58, 1.50) |
| France | -1.64 (-2.14, -1.14) |
| Germany | 0.44 (-0.50, 1.38) |
| Greece | 2.83 (2.22, 3.44) |
| Iceland | 1.53 (0.73, 2.33) |
| Ireland | 0.32 (-0.88, 1.53) |
| Israel | -0.49 (-1.39, 0.41) |
| Italy | 6.65 (4.87, 8.47) |
| Luxembourg | -0.54 (-1.24, 0.16) |
| Malta | 3.26 (2.39, 4.14) |
| Netherlands | 2.32 (1.59, 3.04) |
| Norway | 1.09 (-0.20, 2.39) |
| Portugal | -4.13 (-4.83, -3.43) |
| Spain | 0.79 (0.07, 1.52) |
| Sweden | 6.67 (5.52, 7.82) |
| Switzerland | 0.33 (-0.41, 1.08) |
| United Kingdom | 0.75 (-2.19, 3.78) |
| Argentina | -6.72 (-7.07, -6.38) |
| Chile | -4.89 (-5.52, -4.25) |
| Uruguay | -5.25 (-5.60, -4.91) |
| Canada | 4.64 (2.83, 6.47) |
| United States of America | -2.93 (-3.48, -2.37) |
| Antigua and Barbuda | -2.01 (-2.72, -1.30) |
| Bahamas | -5.29 (-6.02, -4.56) |
| Barbados | -3.26 (-4.04, -2.47) |
| Belize | -4.85 (-5.60, -4.09) |
| Cuba | -4.57 (-5.48, -3.66) |
| Dominica | 0.21 (-0.48, 0.90) |
| Dominican Republic | -5.53 (-6.14, -4.92) |
| Grenada | -2.84 (-3.94, -1.72) |
| Guyana | -6.26 (-6.57, -5.96) |
| Haiti | -3.72 (-4.25, -3.20) |
| Jamaica | -4.61 (-6.23, -2.97) |
| Saint Lucia | -2.82 (-3.98, -1.65) |
| Saint Vincent and the Grenadines | -4.78 (-5.64, -3.90) |
| Suriname | -2.96 (-3.46, -2.45) |
| Trinidad and Tobago | -4.49 (-4.90, -4.08) |
| Bolivia (Plurinational State of) | -8.15 (-8.40, -7.90) |
| Ecuador | -12.22 (-12.75, -11.68) |
| Peru | -10.23 (-10.71, -9.74) |
| Colombia | -9.28 (-9.98, -8.58) |
| Costa Rica | -6.85 (-7.45, -6.24) |
| El Salvador | -10.50 (-11.04, -9.96) |
| Guatemala | -5.55 (-5.80, -5.30) |
| Honduras | -7.49 (-7.73, -7.25) |
| Mexico | -9.89 (-10.40, -9.37) |
| Nicaragua | -9.91 (-10.38, -9.43) |
| Panama | -3.46 (-3.82, -3.10) |
| Venezuela (Bolivarian Republic of) | -5.97 (-6.97, -4.96) |
| Brazil | -11.33 (-11.55, -11.12) |
| Paraguay | -8.83 (-9.18, -8.49) |
| Algeria | -5.67 (-5.80, -5.53) |
| Bahrain | -5.85 (-6.19, -5.51) |
| Egypt | -7.77 (-8.24, -7.29) |
| Iran (Islamic Republic of) | -8.76 (-9.22, -8.30) |
| Iraq | -6.60 (-6.91, -6.29) |
| Jordan | -5.96 (-6.21, -5.71) |
| Kuwait | -3.33 (-4.34, -2.32) |
| Lebanon | -4.71 (-4.93, -4.48) |
| Libya | -6.96 (-7.65, -6.27) |
| Morocco | -8.94 (-9.31, -8.57) |
| Palestine | -7.79 (-8.17, -7.40) |
| Oman | -4.47 (-5.40, -3.52) |
| Qatar | -5.25 (-5.55, -4.95) |
| Saudi Arabia | -9.60 (-9.80, -9.40) |
| Syrian Arab Republic | -7.26 (-8.02, -6.49) |
| Tunisia | -6.40 (-6.75, -6.05) |
| Turkey | -9.03 (-9.25, -8.81) |
| United Arab Emirates | -3.11 (-3.89, -2.33) |
| Yemen | -9.42 (-9.89, -8.95) |
| Afghanistan | -6.22 (-6.90, -5.54) |
| Bangladesh | -8.30 (-8.69, -7.91) |
| Bhutan | -9.19 (-9.42, -8.95) |
| India | -7.48 (-7.84, -7.12) |
| Nepal | -9.94 (-10.20, -9.68) |
| Pakistan | -6.07 (-6.47, -5.67) |
| Angola | -9.05 (-9.71, -8.38) |
| Central African Republic | -2.61 (-2.80, -2.41) |
| Congo | -6.37 (-7.04, -5.69) |
| Democratic Republic of the Congo | -6.56 (-7.76, -5.36) |
| Equatorial Guinea | -13.31 (-13.92, -12.71) |
| Gabon | -6.91 (-7.23, -6.60) |
| Burundi | -4.46 (-4.99, -3.94) |
| Comoros | -5.38 (-5.81, -4.95) |
| Djibouti | -7.19 (-7.64, -6.74) |
| Eritrea | -6.67 (-6.90, -6.43) |
| Ethiopia | -6.85 (-7.07, -6.63) |
| Kenya | -4.63 (-5.09, -4.17) |
| Madagascar | -3.29 (-3.68, -2.90) |
| Malawi | -7.70 (-7.89, -7.50) |
| Mauritius | -3.36 (-3.83, -2.88) |
| Mozambique | -6.93 (-7.27, -6.60) |
| Rwanda | -7.34 (-8.15, -6.51) |
| Seychelles | -2.99 (-3.63, -2.35) |
| Somalia | -4.41 (-4.61, -4.21) |
| United Republic of Tanzania | -6.26 (-6.54, -5.99) |
| Uganda | -7.29 (-7.56, -7.03) |
| Zambia | -7.20 (-7.88, -6.51) |
| Botswana | -2.90 (-3.40, -2.39) |
| Lesotho | -2.10 (-2.41, -1.79) |
| Namibia | -3.57 (-4.04, -3.10) |
| South Africa | -4.40 (-4.87, -3.92) |
| Eswatini | -4.55 (-5.29, -3.80) |
| Zimbabwe | -0.98 (-2.01, 0.06) |
| Benin | -5.73 (-6.03, -5.43) |
| Burkina Faso | -5.08 (-5.33, -4.84) |
| Cameroon | -4.33 (-5.06, -3.59) |
| Cabo Verde | -8.53 (-9.20, -7.86) |
| Chad | -2.50 (-2.68, -2.32) |
| C么te d'Ivoire | -4.52 (-5.02, -4.01) |
| Gambia | -6.60 (-6.99, -6.22) |
| Ghana | -6.88 (-7.36, -6.39) |
| Guinea | -7.18 (-7.53, -6.83) |
| Guinea-Bissau | -7.41 (-8.32, -6.50) |
| Liberia | -7.07 (-7.49, -6.65) |
| Mali | -6.41 (-6.82, -6.00) |
| Mauritania | -6.47 (-7.12, -5.81) |
| Niger | -6.82 (-7.21, -6.43) |
| Nigeria | -4.40 (-4.93, -3.86) |
| Sao Tome and Principe | -10.45 (-11.10, -9.79) |
| Senegal | -8.62 (-9.25, -7.98) |
| Sierra Leone | -6.64 (-7.33, -5.93) |
| Togo | -4.86 (-5.31, -4.41) |
| American Samoa | -1.43 (-1.64, -1.21) |
| Bermuda | -3.52 (-4.72, -2.30) |
| Cook Islands | -3.97 (-4.77, -3.16) |
| Greenland | -1.99 (-2.26, -1.73) |
| Guam | 0.30 (-0.14, 0.74) |
| Monaco | -1.80 (-2.58, -1.01) |
| Nauru | -2.59 (-3.24, -1.93) |
| Niue | -2.07 (-2.79, -1.34) |
| Northern Mariana Islands | -0.91 (-1.24, -0.58) |
| Palau | -3.89 (-4.06, -3.71) |
| Puerto Rico | -1.73 (-2.96, -0.48) |
| Saint Kitts and Nevis | -4.01 (-4.88, -3.13) |
| San Marino | -2.85 (-3.08, -2.62) |
| Tokelau | -3.39 (-5.27, -1.48) |
| Tuvalu | -5.79 (-6.30, -5.29) |
| United States Virgin Islands | -1.84 (-2.36, -1.31) |
| South Sudan | -2.09 (-2.34, -1.85) |
| Sudan | -8.88 (-9.39, -8.36) |

Table S6 EAPC of ASDR of Diarrheal Diseases in Neonates and Children Under Five Across Countries,1990-2021

| location | EAPC of ASDR |
| --- | --- |
| China | -13.71 (-14.18, -13.23) |
| Democratic People's Republic of Korea | -2.92 (-3.31, -2.53) |
| Taiwan (Province of China) | -1.73 (-2.59, -0.86) |
| Cambodia | -9.65 (-10.06, -9.24) |
| Indonesia | -7.79 (-7.94, -7.63) |
| Lao People's Democratic Republic | -8.79 (-9.11, -8.47) |
| Malaysia | -3.92 (-4.60, -3.24) |
| Maldives | -10.02 (-10.74, -9.30) |
| Myanmar | -8.69 (-8.91, -8.47) |
| Philippines | -6.08 (-6.26, -5.90) |
| Sri Lanka | -7.50 (-7.88, -7.11) |
| Thailand | -5.75 (-5.99, -5.51) |
| Timor-Leste | -9.15 (-9.45, -8.86) |
| Viet Nam | -9.04 (-9.40, -8.67) |
| Fiji | -2.32 (-2.72, -1.92) |
| Kiribati | -4.74 (-4.92, -4.55) |
| Marshall Islands | -3.71 (-4.44, -2.98) |
| Micronesia (Federated States of) | -5.02 (-6.05, -3.98) |
| Papua New Guinea | -2.34 (-2.53, -2.16) |
| Samoa | -2.96 (-3.22, -2.70) |
| Solomon Islands | -4.70 (-4.90, -4.50) |
| Tonga | -3.14 (-3.34, -2.94) |
| Vanuatu | -3.90 (-4.11, -3.68) |
| Armenia | -14.33 (-15.19, -13.47) |
| Azerbaijan | -6.68 (-6.94, -6.43) |
| Georgia | -12.75 (-13.41, -12.09) |
| Kazakhstan | -14.12 (-14.93, -13.30) |
| Kyrgyzstan | -9.79 (-10.57, -9.01) |
| Mongolia | -7.19 (-7.44, -6.93) |
| Tajikistan | -5.43 (-5.87, -4.98) |
| Turkmenistan | -12.48 (-13.15, -11.80) |
| Uzbekistan | -14.37 (-15.03, -13.71) |
| Albania | -6.01 (-6.62, -5.40) |
| Bosnia and Herzegovina | -1.60 (-1.89, -1.30) |
| Bulgaria | -0.45 (-1.44, 0.55) |
| Croatia | 2.01 (0.32, 3.73) |
| Czechia | 1.53 (-0.20, 3.29) |
| Hungary | 2.08 (0.37, 3.82) |
| North Macedonia | -6.44 (-7.41, -5.47) |
| Montenegro | -3.18 (-3.66, -2.71) |
| Poland | 1.49 (-0.30, 3.32) |
| Romania | -4.81 (-5.66, -3.95) |
| Serbia | -3.57 (-4.02, -3.11) |
| Slovakia | 0.43 (-0.08, 0.93) |
| Slovenia | 0.82 (-0.10, 1.75) |
| Belarus | -6.92 (-7.58, -6.26) |
| Estonia | -6.16 (-6.41, -5.91) |
| Latvia | -7.13 (-7.49, -6.77) |
| Lithuania | -6.15 (-6.70, -5.60) |
| Republic of Moldova | -7.96 (-8.39, -7.54) |
| Russian Federation | -7.81 (-8.48, -7.15) |
| Ukraine | -7.66 (-8.38, -6.94) |
| Brunei Darussalam | 0.16 (-0.05, 0.37) |
| Japan | 0.14 (-0.24, 0.52) |
| Republic of Korea | -3.20 (-3.44, -2.96) |
| Singapore | -3.13 (-3.81, -2.45) |
| Australia | -2.76 (-3.56, -1.95) |
| New Zealand | 0.04 (-1.07, 1.16) |
| Andorra | 0.18 (-0.33, 0.68) |
| Austria | -4.41 (-5.50, -3.31) |
| Belgium | -0.48 (-1.19, 0.23) |
| Cyprus | -2.56 (-2.79, -2.33) |
| Denmark | 1.52 (0.80, 2.24) |
| Finland | -2.20 (-3.13, -1.26) |
| France | 0.11 (-0.19, 0.41) |
| Germany | -1.10 (-2.16, -0.03) |
| Greece | 0.01 (-0.72, 0.75) |
| Iceland | 0.79 (0.37, 1.22) |
| Ireland | 1.08 (-0.14, 2.31) |
| Israel | -1.19 (-2.03, -0.34) |
| Italy | 2.30 (1.78, 2.82) |
| Luxembourg | -1.56 (-2.24, -0.88) |
| Malta | 0.94 (0.39, 1.49) |
| Netherlands | 2.09 (1.49, 2.69) |
| Norway | -1.62 (-2.32, -0.91) |
| Portugal | -2.48 (-2.84, -2.11) |
| Spain | 0.58 (0.20, 0.95) |
| Sweden | 2.09 (1.65, 2.53) |
| Switzerland | 0.36 (-0.10, 0.82) |
| United Kingdom | -1.57 (-3.44, 0.34) |
| Argentina | -6.51 (-6.89, -6.12) |
| Chile | -4.77 (-5.15, -4.38) |
| Uruguay | -5.27 (-5.58, -4.97) |
| Canada | 1.21 (-0.15, 2.58) |
| United States of America | -3.57 (-4.11, -3.02) |
| Antigua and Barbuda | -2.03 (-2.68, -1.38) |
| Bahamas | -5.20 (-5.87, -4.52) |
| Barbados | -3.07 (-3.77, -2.37) |
| Belize | -4.83 (-5.57, -4.08) |
| Cuba | -4.26 (-5.10, -3.41) |
| Dominica | 0.15 (-0.51, 0.82) |
| Dominican Republic | -5.54 (-6.14, -4.94) |
| Grenada | -2.80 (-3.81, -1.78) |
| Guyana | -6.28 (-6.58, -5.97) |
| Haiti | -3.72 (-4.25, -3.20) |
| Jamaica | -4.65 (-6.21, -3.07) |
| Saint Lucia | -2.79 (-3.90, -1.68) |
| Saint Vincent and the Grenadines | -4.77 (-5.61, -3.91) |
| Suriname | -2.98 (-3.48, -2.48) |
| Trinidad and Tobago | -4.43 (-4.82, -4.03) |
| Bolivia (Plurinational State of) | -8.16 (-8.41, -7.92) |
| Ecuador | -12.15 (-12.71, -11.60) |
| Peru | -10.06 (-10.46, -9.65) |
| Colombia | -9.17 (-9.85, -8.49) |
| Costa Rica | -6.37 (-6.91, -5.82) |
| El Salvador | -10.44 (-10.96, -9.92) |
| Guatemala | -5.54 (-5.78, -5.29) |
| Honduras | -7.47 (-7.71, -7.23) |
| Mexico | -9.84 (-10.36, -9.32) |
| Nicaragua | -9.90 (-10.37, -9.43) |
| Panama | -3.47 (-3.80, -3.13) |
| Venezuela (Bolivarian Republic of) | -5.94 (-6.93, -4.95) |
| Brazil | -11.21 (-11.42, -11.01) |
| Paraguay | -8.96 (-9.32, -8.60) |
| Algeria | -5.69 (-5.83, -5.56) |
| Bahrain | -5.68 (-6.02, -5.34) |
| Egypt | -7.63 (-8.10, -7.16) |
| Iran (Islamic Republic of) | -8.69 (-9.12, -8.26) |
| Iraq | -6.54 (-6.84, -6.23) |
| Jordan | -5.78 (-6.03, -5.53) |
| Kuwait | -3.20 (-3.97, -2.42) |
| Lebanon | -4.56 (-4.74, -4.37) |
| Libya | -6.91 (-7.54, -6.28) |
| Morocco | -8.91 (-9.28, -8.54) |
| Palestine | -7.57 (-7.97, -7.16) |
| Oman | -4.43 (-5.34, -3.51) |
| Qatar | -4.90 (-5.18, -4.61) |
| Saudi Arabia | -9.36 (-9.56, -9.16) |
| Syrian Arab Republic | -7.08 (-7.75, -6.41) |
| Tunisia | -6.09 (-6.42, -5.76) |
| Turkey | -8.87 (-9.06, -8.68) |
| United Arab Emirates | -3.23 (-3.96, -2.49) |
| Yemen | -9.21 (-9.64, -8.77) |
| Afghanistan | -6.21 (-6.88, -5.53) |
| Bangladesh | -8.28 (-8.65, -7.90) |
| Bhutan | -9.16 (-9.39, -8.92) |
| India | -7.42 (-7.77, -7.06) |
| Nepal | -9.89 (-10.15, -9.63) |
| Pakistan | -6.04 (-6.44, -5.64) |
| Angola | -9.02 (-9.68, -8.35) |
| Central African Republic | -2.62 (-2.81, -2.42) |
| Congo | -6.35 (-7.03, -5.67) |
| Democratic Republic of the Congo | -6.54 (-7.73, -5.34) |
| Equatorial Guinea | -13.22 (-13.80, -12.63) |
| Gabon | -6.88 (-7.20, -6.56) |
| Burundi | -4.45 (-4.98, -3.92) |
| Comoros | -5.37 (-5.80, -4.94) |
| Djibouti | -7.19 (-7.64, -6.74) |
| Eritrea | -6.65 (-6.89, -6.41) |
| Ethiopia | -6.82 (-7.04, -6.61) |
| Kenya | -4.62 (-5.08, -4.15) |
| Madagascar | -3.29 (-3.69, -2.90) |
| Malawi | -7.68 (-7.88, -7.48) |
| Mauritius | -3.35 (-3.80, -2.89) |
| Mozambique | -6.93 (-7.27, -6.59) |
| Rwanda | -7.33 (-8.14, -6.50) |
| Seychelles | -2.92 (-3.55, -2.29) |
| Somalia | -4.41 (-4.60, -4.21) |
| United Republic of Tanzania | -6.28 (-6.56, -6.00) |
| Uganda | -7.28 (-7.54, -7.01) |
| Zambia | -7.19 (-7.88, -6.50) |
| Botswana | -2.91 (-3.42, -2.41) |
| Lesotho | -2.11 (-2.43, -1.80) |
| Namibia | -3.58 (-4.05, -3.10) |
| South Africa | -4.41 (-4.89, -3.93) |
| Eswatini | -4.55 (-5.29, -3.80) |
| Zimbabwe | -1.03 (-2.06, 0.00) |
| Benin | -5.73 (-6.03, -5.43) |
| Burkina Faso | -5.08 (-5.33, -4.83) |
| Cameroon | -4.33 (-5.07, -3.59) |
| Cabo Verde | -8.56 (-9.21, -7.90) |
| Chad | -2.50 (-2.68, -2.32) |
| C么te d'Ivoire | -4.53 (-5.03, -4.02) |
| Gambia | -6.60 (-6.99, -6.22) |
| Ghana | -6.88 (-7.37, -6.38) |
| Guinea | -7.17 (-7.53, -6.82) |
| Guinea-Bissau | -7.40 (-8.31, -6.49) |
| Liberia | -7.05 (-7.47, -6.63) |
| Mali | -6.40 (-6.81, -5.99) |
| Mauritania | -6.43 (-7.08, -5.77) |
| Niger | -6.81 (-7.19, -6.42) |
| Nigeria | -4.39 (-4.92, -3.85) |
| Sao Tome and Principe | -10.42 (-11.08, -9.77) |
| Senegal | -8.57 (-9.20, -7.93) |
| Sierra Leone | -6.63 (-7.33, -5.93) |
| Togo | -4.86 (-5.31, -4.41) |
| American Samoa | -1.38 (-1.57, -1.19) |
| Bermuda | -2.83 (-3.69, -1.96) |
| Cook Islands | -2.63 (-3.15, -2.11) |
| Greenland | -2.08 (-2.33, -1.84) |
| Guam | 0.35 (-0.00, 0.71) |
| Monaco | 0.05 (-0.35, 0.45) |
| Nauru | -2.55 (-3.19, -1.91) |
| Niue | -1.99 (-2.67, -1.31) |
| Northern Mariana Islands | -0.78 (-1.05, -0.51) |
| Palau | -3.87 (-4.04, -3.69) |
| Puerto Rico | -1.67 (-2.55, -0.78) |
| Saint Kitts and Nevis | -3.99 (-4.84, -3.14) |
| San Marino | -0.14 (-0.55, 0.27) |
| Tokelau | -3.33 (-5.17, -1.46) |
| Tuvalu | -5.75 (-6.25, -5.26) |
| United States Virgin Islands | -1.91 (-2.36, -1.45) |
| South Sudan | -2.10 (-2.34, -1.86) |
| Sudan | -8.79 (-9.30, -8.27) |

Table S7 Global Diarrhea-Related Death Number in Neonates and Children Under Five Years of Age Attributable to Specific Pathogens in 2021

| age | cause | pathogens | metric | year | death |
| --- | --- | --- | --- | --- | --- |
| 12-23 months | Diarrheal diseases | Non-typhoidal Salmonella | Number | 2021 | 2749.76 (0.00, 8350.13) |
| 1-5 months | Diarrheal diseases | Non-typhoidal Salmonella | Number | 2021 | 3137.63 (517.45, 7263.36) |
| 6-11 months | Diarrheal diseases | Non-typhoidal Salmonella | Number | 2021 | 2797.75 (448.05, 6764.25) |
| 0-6 days | Diarrheal diseases | Shigella | Number | 2021 | 2114.80 (990.09, 3859.71) |
| 7-27 days | Diarrheal diseases | Shigella | Number | 2021 | 2831.98 (1386.75, 5237.94) |
| 2-4 years | Diarrheal diseases | Enterotoxigenic E coli | Number | 2021 | 8806.25 (4049.02, 16661.34) |
| 0-6 days | Diarrheal diseases | Cholera | Number | 2021 | 338.84 (220.88, 479.90) |
| 7-27 days | Diarrheal diseases | Cholera | Number | 2021 | 944.82 (620.91, 1323.81) |
| 12-23 months | Diarrheal diseases | Entamoeba | Number | 2021 | 3363.56 (1398.67, 6939.51) |
| 1-5 months | Diarrheal diseases | Entamoeba | Number | 2021 | 4454.03 (1823.24, 9112.53) |
| 6-11 months | Diarrheal diseases | Entamoeba | Number | 2021 | 4108.63 (1677.72, 8361.43) |
| 0-6 days | Diarrheal diseases | Cryptosporidium | Number | 2021 | 1864.37 (953.65, 3387.18) |
| 7-27 days | Diarrheal diseases | Cryptosporidium | Number | 2021 | 2452.70 (1311.87, 4195.99) |
| 2-4 years | Diarrheal diseases | Aeromonas | Number | 2021 | 2881.42 (1134.95, 5567.38) |
| 12-23 months | Diarrheal diseases | Aeromonas | Number | 2021 | 3368.11 (1235.04, 6911.63) |
| 1-5 months | Diarrheal diseases | Aeromonas | Number | 2021 | 2069.61 (524.22, 4914.36) |
| 6-11 months | Diarrheal diseases | Aeromonas | Number | 2021 | 1941.93 (469.82, 5216.06) |
| 1-5 months | Diarrheal diseases | Norovirus | Number | 2021 | 5470.57 (1852.85, 11203.87) |
| 6-11 months | Diarrheal diseases | Norovirus | Number | 2021 | 4832.65 (1522.10, 10416.64) |
| 0-6 days | Diarrheal diseases | Adenovirus | Number | 2021 | 2727.85 (1420.40, 4776.14) |
| 7-27 days | Diarrheal diseases | Adenovirus | Number | 2021 | 3821.78 (2076.16, 6426.72) |
| 2-4 years | Diarrheal diseases | Shigella | Number | 2021 | 16934.36 (8074.60, 32087.33) |
| 2-4 years | Diarrheal diseases | Cholera | Number | 2021 | 6903.77 (4489.34, 9874.54) |
| 12-23 months | Diarrheal diseases | Cholera | Number | 2021 | 6334.36 (4379.39, 8920.79) |
| 1-5 months | Diarrheal diseases | Cholera | Number | 2021 | 6049.20 (4022.29, 8548.92) |
| 6-11 months | Diarrheal diseases | Cholera | Number | 2021 | 5207.04 (3560.59, 7302.71) |
| 0-6 days | Diarrheal diseases | Non-typhoidal Salmonella | Number | 2021 | 308.59 (54.82, 757.64) |
| 6-11 months | Diarrheal diseases | Shigella | Number | 2021 | 19921.12 (9875.07, 36372.15) |
| 0-6 days | Diarrheal diseases | Enteropathogenic E coli | Number | 2021 | 1485.33 (650.88, 2952.18) |
| 7-27 days | Diarrheal diseases | Enteropathogenic E coli | Number | 2021 | 1975.77 (887.21, 3852.40) |
| 12-23 months | Diarrheal diseases | Shigella | Number | 2021 | 18384.58 (9213.14, 31408.03) |
| 1-5 months | Diarrheal diseases | Shigella | Number | 2021 | 21587.21 (11365.50, 37790.22) |
| 12-23 months | Diarrheal diseases | Enterotoxigenic E coli | Number | 2021 | 9815.22 (4670.07, 19268.77) |
| 1-5 months | Diarrheal diseases | Enterotoxigenic E coli | Number | 2021 | 12610.19 (6707.73, 22144.34) |
| 6-11 months | Diarrheal diseases | Enterotoxigenic E coli | Number | 2021 | 11657.28 (5517.42, 21624.00) |
| 0-6 days | Diarrheal diseases | Campylobacter | Number | 2021 | 899.13 (319.98, 1986.47) |
| 7-27 days | Diarrheal diseases | Campylobacter | Number | 2021 | 1300.93 (488.18, 2617.96) |
| 2-4 years | Diarrheal diseases | Enteropathogenic E coli | Number | 2021 | 5935.39 (1980.05, 12028.95) |
| 2-4 years | Diarrheal diseases | Campylobacter | Number | 2021 | 3312.58 (527.09, 8271.01) |
| 12-23 months | Diarrheal diseases | Campylobacter | Number | 2021 | 3796.29 (611.56, 9234.63) |
| 2-4 years | Diarrheal diseases | Cryptosporidium | Number | 2021 | 12309.53 (5561.56, 22609.59) |
| 12-23 months | Diarrheal diseases | Cryptosporidium | Number | 2021 | 13886.06 (6350.38, 25841.79) |
| 1-5 months | Diarrheal diseases | Cryptosporidium | Number | 2021 | 19688.19 (11272.80, 30644.43) |
| 6-11 months | Diarrheal diseases | Cryptosporidium | Number | 2021 | 18277.27 (9764.28, 32624.27) |
| 0-6 days | Diarrheal diseases | Rotavirus | Number | 2021 | 4265.82 (2809.10, 6536.86) |
| 7-27 days | Diarrheal diseases | Rotavirus | Number | 2021 | 5935.78 (4129.16, 8631.32) |
| 0-6 days | Diarrheal diseases | Clostridioides difficile | Number | 2021 | 0.09 (0.04, 0.18) |
| 7-27 days | Diarrheal diseases | Clostridioides difficile | Number | 2021 | 0.87 (0.39, 1.71) |
| 1-5 months | Diarrheal diseases | Campylobacter | Number | 2021 | 8508.03 (3337.16, 16782.96) |
| 6-11 months | Diarrheal diseases | Campylobacter | Number | 2021 | 7489.08 (2777.16, 15429.67) |
| 0-6 days | Diarrheal diseases | Entamoeba | Number | 2021 | 420.65 (160.83, 921.98) |
| 7-27 days | Diarrheal diseases | Entamoeba | Number | 2021 | 602.42 (233.26, 1303.78) |
| 2-4 years | Diarrheal diseases | Clostridioides difficile | Number | 2021 | 50.99 (25.71, 89.91) |
| 2-4 years | Diarrheal diseases | Adenovirus | Number | 2021 | 11598.88 (5412.30, 22889.93) |
| 2-4 years | Diarrheal diseases | Rotavirus | Number | 2021 | 18709.88 (11050.34, 29684.45) |
| 12-23 months | Diarrheal diseases | Adenovirus | Number | 2021 | 14880.62 (6923.36, 26589.69) |
| 1-5 months | Diarrheal diseases | Adenovirus | Number | 2021 | 25636.27 (15132.80, 41797.90) |
| 6-11 months | Diarrheal diseases | Adenovirus | Number | 2021 | 22414.37 (11557.17, 37477.27) |
| 12-23 months | Diarrheal diseases | Clostridioides difficile | Number | 2021 | 25.25 (12.44, 45.10) |
| 1-5 months | Diarrheal diseases | Clostridioides difficile | Number | 2021 | 9.84 (4.27, 19.39) |
| 6-11 months | Diarrheal diseases | Clostridioides difficile | Number | 2021 | 12.68 (5.96, 22.82) |
| 0-6 days | Diarrheal diseases | Norovirus | Number | 2021 | 561.12 (185.36, 1192.65) |
| 7-27 days | Diarrheal diseases | Norovirus | Number | 2021 | 791.41 (265.17, 1658.69) |
| 7-27 days | Diarrheal diseases | Non-typhoidal Salmonella | Number | 2021 | 431.81 (69.54, 1078.94) |
| 2-4 years | Diarrheal diseases | Non-typhoidal Salmonella | Number | 2021 | 2407.96 (0.00, 7551.91) |
| 12-23 months | Diarrheal diseases | Enteropathogenic E coli | Number | 2021 | 8540.73 (3227.18, 16200.53) |
| 1-5 months | Diarrheal diseases | Enteropathogenic E coli | Number | 2021 | 14971.32 (7189.32, 26925.27) |
| 6-11 months | Diarrheal diseases | Enteropathogenic E coli | Number | 2021 | 13458.61 (6289.56, 24751.55) |
| 0-6 days | Diarrheal diseases | Enterotoxigenic E coli | Number | 2021 | 1209.36 (590.90, 2348.05) |
| 7-27 days | Diarrheal diseases | Enterotoxigenic E coli | Number | 2021 | 1702.28 (886.20, 3137.29) |
| 2-4 years | Diarrheal diseases | Entamoeba | Number | 2021 | 3141.09 (1322.92, 6494.10) |
| 12-23 months | Diarrheal diseases | Rotavirus | Number | 2021 | 23538.13 (14842.89, 35089.82) |
| 1-5 months | Diarrheal diseases | Rotavirus | Number | 2021 | 35793.70 (25261.06, 48787.67) |
| 6-11 months | Diarrheal diseases | Rotavirus | Number | 2021 | 31618.03 (20057.41, 46868.27) |
| 0-6 days | Diarrheal diseases | Aeromonas | Number | 2021 | 196.54 (46.96, 519.33) |
| 7-27 days | Diarrheal diseases | Aeromonas | Number | 2021 | 282.29 (70.89, 710.09) |
| 2-4 years | Diarrheal diseases | Norovirus | Number | 2021 | 8309.38 (1138.78, 16592.04) |
| 12-23 months | Diarrheal diseases | Norovirus | Number | 2021 | 9590.02 (1397.02, 18269.00) |

Table S8 Global Diarrhea-Related DALYs Number in Neonates and Children Under Five Years of Age Attributable to Specific Pathogens in 2021

| age | cause | pathogens | metric | year | DALYs |
| --- | --- | --- | --- | --- | --- |
| 7-27 days | Diarrheal diseases | Non-typhoidal Salmonella | Number | 2021 | 39192.81 (6583.37, 97543.84) |
| 12-23 months | Diarrheal diseases | Non-typhoidal Salmonella | Number | 2021 | 245312.69 (672.58, 741853.29) |
| 1-5 months | Diarrheal diseases | Non-typhoidal Salmonella | Number | 2021 | 284222.70 (48757.94, 655549.96) |
| 6-11 months | Diarrheal diseases | Non-typhoidal Salmonella | Number | 2021 | 252222.45 (41951.27, 605991.36) |
| 0-6 days | Diarrheal diseases | Shigella | Number | 2021 | 190577.24 (89265.34, 347700.01) |
| 7-27 days | Diarrheal diseases | Shigella | Number | 2021 | 256693.39 (125959.34, 473790.66) |
| 2-4 years | Diarrheal diseases | Enterotoxigenic E coli | Number | 2021 | 787526.36 (370738.20, 1486415.46) |
| 12-23 months | Diarrheal diseases | Entamoeba | Number | 2021 | 299999.12 (126070.33, 615532.26) |
| 1-5 months | Diarrheal diseases | Entamoeba | Number | 2021 | 402099.24 (165316.91, 821921.10) |
| 6-11 months | Diarrheal diseases | Entamoeba | Number | 2021 | 369146.85 (151985.74, 755080.14) |
| 0-6 days | Diarrheal diseases | Cryptosporidium | Number | 2021 | 167953.78 (85924.74, 305088.54) |
| 7-27 days | Diarrheal diseases | Cryptosporidium | Number | 2021 | 221981.90 (118862.32, 378941.17) |
| 2-4 years | Diarrheal diseases | Non-typhoidal Salmonella | Number | 2021 | 212686.60 (1610.13, 660525.90) |
| 12-23 months | Diarrheal diseases | Enteropathogenic E coli | Number | 2021 | 763020.86 (289265.55, 1442207.95) |
| 1-5 months | Diarrheal diseases | Enteropathogenic E coli | Number | 2021 | 1355545.45 (653926.21, 2440209.39) |
| 6-11 months | Diarrheal diseases | Enteropathogenic E coli | Number | 2021 | 1212438.37 (571198.75, 2222160.39) |
| 2-4 years | Diarrheal diseases | Aeromonas | Number | 2021 | 253732.63 (103102.31, 488325.07) |
| 12-23 months | Diarrheal diseases | Aeromonas | Number | 2021 | 300066.70 (110220.14, 614046.79) |
| 1-5 months | Diarrheal diseases | Aeromonas | Number | 2021 | 188011.92 (48714.42, 444921.30) |
| 6-11 months | Diarrheal diseases | Aeromonas | Number | 2021 | 175603.92 (43124.15, 468204.97) |
| 1-5 months | Diarrheal diseases | Norovirus | Number | 2021 | 505890.96 (180364.02, 1022727.58) |
| 6-11 months | Diarrheal diseases | Norovirus | Number | 2021 | 445105.88 (152101.55, 946511.70) |
| 0-6 days | Diarrheal diseases | Adenovirus | Number | 2021 | 245934.05 (128083.75, 430403.07) |
| 7-27 days | Diarrheal diseases | Adenovirus | Number | 2021 | 347013.67 (188295.79, 583395.69) |
| 0-6 days | Diarrheal diseases | Enterotoxigenic E coli | Number | 2021 | 109057.60 (53273.52, 211601.00) |
| 7-27 days | Diarrheal diseases | Enterotoxigenic E coli | Number | 2021 | 154730.80 (80557.98, 284708.99) |
| 2-4 years | Diarrheal diseases | Entamoeba | Number | 2021 | 277524.01 (119907.73, 568970.08) |
| 12-23 months | Diarrheal diseases | Rotavirus | Number | 2021 | 2109664.26 (1333663.63, 3134654.54) |
| 0-6 days | Diarrheal diseases | Cholera | Number | 2021 | 30489.73 (19875.31, 43182.17) |
| 7-27 days | Diarrheal diseases | Cholera | Number | 2021 | 85022.65 (55874.96, 119123.12) |
| 1-5 months | Diarrheal diseases | Rotavirus | Number | 2021 | 3234300.78 (2283763.34, 4404994.55) |
| 6-11 months | Diarrheal diseases | Rotavirus | Number | 2021 | 2843633.07 (1807007.67, 4209114.09) |
| 0-6 days | Diarrheal diseases | Aeromonas | Number | 2021 | 17727.91 (4263.70, 46793.93) |
| 7-27 days | Diarrheal diseases | Aeromonas | Number | 2021 | 25684.37 (6639.63, 64117.81) |
| 2-4 years | Diarrheal diseases | Norovirus | Number | 2021 | 748256.20 (124520.89, 1457909.44) |
| 12-23 months | Diarrheal diseases | Norovirus | Number | 2021 | 860812.92 (135385.20, 1640709.07) |
| 2-4 years | Diarrheal diseases | Shigella | Number | 2021 | 1507359.64 (716686.69, 2859010.03) |
| 12-23 months | Diarrheal diseases | Shigella | Number | 2021 | 1645321.76 (825046.35, 2808811.61) |
| 1-5 months | Diarrheal diseases | Shigella | Number | 2021 | 1952072.17 (1030011.13, 3408653.53) |
| 12-23 months | Diarrheal diseases | Enterotoxigenic E coli | Number | 2021 | 881769.32 (420674.06, 1719090.29) |
| 1-5 months | Diarrheal diseases | Enterotoxigenic E coli | Number | 2021 | 1144036.04 (610285.15, 2004075.09) |
| 6-11 months | Diarrheal diseases | Enterotoxigenic E coli | Number | 2021 | 1052430.39 (499492.34, 1954096.07) |
| 0-6 days | Diarrheal diseases | Campylobacter | Number | 2021 | 81224.93 (28991.95, 179107.59) |
| 7-27 days | Diarrheal diseases | Campylobacter | Number | 2021 | 119138.71 (45271.81, 237808.92) |
| 2-4 years | Diarrheal diseases | Cryptosporidium | Number | 2021 | 1079526.73 (491891.85, 1975409.84) |
| 12-23 months | Diarrheal diseases | Cryptosporidium | Number | 2021 | 1235995.65 (564928.84, 2300863.30) |
| 1-5 months | Diarrheal diseases | Cryptosporidium | Number | 2021 | 1777088.60 (1017638.73, 2764698.43) |
| 6-11 months | Diarrheal diseases | Cryptosporidium | Number | 2021 | 1641287.12 (878881.28, 2929411.00) |
| 0-6 days | Diarrheal diseases | Rotavirus | Number | 2021 | 384400.24 (253251.85, 588757.49) |
| 7-27 days | Diarrheal diseases | Rotavirus | Number | 2021 | 537766.26 (374072.07, 780975.18) |
| 0-6 days | Diarrheal diseases | Clostridioides difficile | Number | 2021 | 8.24 (3.80, 16.09) |
| 7-27 days | Diarrheal diseases | Clostridioides difficile | Number | 2021 | 78.85 (35.36, 154.27) |
| 2-4 years | Diarrheal diseases | Clostridioides difficile | Number | 2021 | 4432.52 (2240.60, 7807.55) |
| 2-4 years | Diarrheal diseases | Adenovirus | Number | 2021 | 1040123.08 (493050.48, 2028624.34) |
| 12-23 months | Diarrheal diseases | Adenovirus | Number | 2021 | 1334914.51 (620270.89, 2378611.72) |
| 1-5 months | Diarrheal diseases | Adenovirus | Number | 2021 | 2324815.49 (1375081.34, 3772422.83) |
| 6-11 months | Diarrheal diseases | Adenovirus | Number | 2021 | 2022851.61 (1044206.41, 3374023.08) |
| 2-4 years | Diarrheal diseases | Cholera | Number | 2021 | 598185.61 (389033.93, 855501.15) |
| 12-23 months | Diarrheal diseases | Cholera | Number | 2021 | 561219.76 (388053.19, 790327.03) |
| 1-5 months | Diarrheal diseases | Cholera | Number | 2021 | 543083.06 (361135.30, 767471.53) |
| 6-11 months | Diarrheal diseases | Cholera | Number | 2021 | 465182.15 (318108.38, 652367.67) |
| 0-6 days | Diarrheal diseases | Non-typhoidal Salmonella | Number | 2021 | 27818.87 (4990.43, 68248.63) |
| 6-11 months | Diarrheal diseases | Shigella | Number | 2021 | 1792369.23 (893116.08, 3266068.33) |
| 0-6 days | Diarrheal diseases | Enteropathogenic E coli | Number | 2021 | 133889.41 (58721.04, 266027.18) |
| 7-27 days | Diarrheal diseases | Enteropathogenic E coli | Number | 2021 | 179324.52 (81039.05, 349142.83) |
| 2-4 years | Diarrheal diseases | Enteropathogenic E coli | Number | 2021 | 525283.31 (178364.68, 1055746.65) |
| 2-4 years | Diarrheal diseases | Campylobacter | Number | 2021 | 306739.04 (64365.30, 736972.52) |
| 12-23 months | Diarrheal diseases | Campylobacter | Number | 2021 | 344407.58 (62937.65, 825523.02) |
| 1-5 months | Diarrheal diseases | Campylobacter | Number | 2021 | 779779.61 (318142.16, 1526313.38) |
| 6-11 months | Diarrheal diseases | Campylobacter | Number | 2021 | 683835.00 (257414.68, 1406368.83) |
| 0-6 days | Diarrheal diseases | Entamoeba | Number | 2021 | 37894.20 (14513.81, 83041.97) |
| 7-27 days | Diarrheal diseases | Entamoeba | Number | 2021 | 54499.02 (21337.17, 117305.48) |
| 2-4 years | Diarrheal diseases | Rotavirus | Number | 2021 | 1679172.33 (1009025.79, 2628557.42) |
| 12-23 months | Diarrheal diseases | Clostridioides difficile | Number | 2021 | 2243.43 (1107.58, 4001.98) |
| 1-5 months | Diarrheal diseases | Clostridioides difficile | Number | 2021 | 885.55 (384.20, 1744.50) |
| 6-11 months | Diarrheal diseases | Clostridioides difficile | Number | 2021 | 1135.61 (534.33, 2043.76) |
| 0-6 days | Diarrheal diseases | Norovirus | Number | 2021 | 50792.74 (16912.19, 107724.33) |
| 7-27 days | Diarrheal diseases | Norovirus | Number | 2021 | 73160.70 (25444.17, 152293.89) |

Table S9 Global Diarrhea-Related ASMR in Neonates and Children Under Five Years of Age Attributable to Specific Pathogens in 2021

| age | cause | pathogens | metric | year | ASMR |
| --- | --- | --- | --- | --- | --- |
| 12-23 months | Diarrheal diseases | Non-typhoidal Salmonella | Rate | 2021 | 2.14 (0.00, 6.50) |
| 1-5 months | Diarrheal diseases | Non-typhoidal Salmonella | Rate | 2021 | 5.84 (0.96, 13.51) |
| 6-11 months | Diarrheal diseases | Non-typhoidal Salmonella | Rate | 2021 | 4.43 (0.71, 10.70) |
| 0-6 days | Diarrheal diseases | Shigella | Rate | 2021 | 86.28 (40.39, 157.46) |
| 7-27 days | Diarrheal diseases | Shigella | Rate | 2021 | 38.83 (19.01, 71.81) |
| 2-4 years | Diarrheal diseases | Enterotoxigenic E coli | Rate | 2021 | 2.18 (1.00, 4.13) |
| 0-6 days | Diarrheal diseases | Cholera | Rate | 2021 | 13.82 (9.01, 19.58) |
| 7-27 days | Diarrheal diseases | Cholera | Rate | 2021 | 12.95 (8.51, 18.15) |
| 12-23 months | Diarrheal diseases | Entamoeba | Rate | 2021 | 2.62 (1.09, 5.40) |
| 1-5 months | Diarrheal diseases | Entamoeba | Rate | 2021 | 8.29 (3.39, 16.95) |
| 6-11 months | Diarrheal diseases | Entamoeba | Rate | 2021 | 6.50 (2.65, 13.23) |
| 0-6 days | Diarrheal diseases | Cryptosporidium | Rate | 2021 | 76.06 (38.91, 138.18) |
| 7-27 days | Diarrheal diseases | Cryptosporidium | Rate | 2021 | 33.63 (17.99, 57.53) |
| 2-4 years | Diarrheal diseases | Aeromonas | Rate | 2021 | 0.71 (0.28, 1.38) |
| 12-23 months | Diarrheal diseases | Aeromonas | Rate | 2021 | 2.62 (0.96, 5.38) |
| 1-5 months | Diarrheal diseases | Aeromonas | Rate | 2021 | 3.85 (0.98, 9.14) |
| 6-11 months | Diarrheal diseases | Aeromonas | Rate | 2021 | 3.07 (0.74, 8.25) |
| 1-5 months | Diarrheal diseases | Norovirus | Rate | 2021 | 10.18 (3.45, 20.84) |
| 6-11 months | Diarrheal diseases | Norovirus | Rate | 2021 | 7.65 (2.41, 16.48) |
| 0-6 days | Diarrheal diseases | Adenovirus | Rate | 2021 | 111.29 (57.95, 194.85) |
| 7-27 days | Diarrheal diseases | Adenovirus | Rate | 2021 | 52.40 (28.46, 88.11) |
| 2-4 years | Diarrheal diseases | Shigella | Rate | 2021 | 4.20 (2.00, 7.96) |
| 2-4 years | Diarrheal diseases | Cholera | Rate | 2021 | 1.71 (1.11, 2.45) |
| 12-23 months | Diarrheal diseases | Cholera | Rate | 2021 | 4.93 (3.41, 6.95) |
| 1-5 months | Diarrheal diseases | Cholera | Rate | 2021 | 11.25 (7.48, 15.90) |
| 6-11 months | Diarrheal diseases | Cholera | Rate | 2021 | 8.24 (5.63, 11.56) |
| 0-6 days | Diarrheal diseases | Non-typhoidal Salmonella | Rate | 2021 | 12.59 (2.24, 30.91) |
| 6-11 months | Diarrheal diseases | Shigella | Rate | 2021 | 31.52 (15.63, 57.56) |
| 0-6 days | Diarrheal diseases | Enteropathogenic E coli | Rate | 2021 | 60.60 (26.55, 120.44) |
| 7-27 days | Diarrheal diseases | Enteropathogenic E coli | Rate | 2021 | 27.09 (12.16, 52.82) |
| 12-23 months | Diarrheal diseases | Shigella | Rate | 2021 | 14.32 (7.17, 24.46) |
| 1-5 months | Diarrheal diseases | Shigella | Rate | 2021 | 40.16 (21.14, 70.30) |
| 12-23 months | Diarrheal diseases | Enterotoxigenic E coli | Rate | 2021 | 7.64 (3.64, 15.01) |
| 1-5 months | Diarrheal diseases | Enterotoxigenic E coli | Rate | 2021 | 23.46 (12.48, 41.19) |
| 6-11 months | Diarrheal diseases | Enterotoxigenic E coli | Rate | 2021 | 18.45 (8.73, 34.22) |
| 0-6 days | Diarrheal diseases | Campylobacter | Rate | 2021 | 36.68 (13.05, 81.04) |
| 7-27 days | Diarrheal diseases | Campylobacter | Rate | 2021 | 17.84 (6.69, 35.89) |
| 2-4 years | Diarrheal diseases | Enteropathogenic E coli | Rate | 2021 | 1.47 (0.49, 2.98) |
| 2-4 years | Diarrheal diseases | Campylobacter | Rate | 2021 | 0.82 (0.13, 2.05) |
| 12-23 months | Diarrheal diseases | Campylobacter | Rate | 2021 | 2.96 (0.48, 7.19) |
| 2-4 years | Diarrheal diseases | Cryptosporidium | Rate | 2021 | 3.05 (1.38, 5.61) |
| 12-23 months | Diarrheal diseases | Cryptosporidium | Rate | 2021 | 10.81 (4.95, 20.12) |
| 1-5 months | Diarrheal diseases | Cryptosporidium | Rate | 2021 | 36.62 (20.97, 57.01) |
| 6-11 months | Diarrheal diseases | Cryptosporidium | Rate | 2021 | 28.92 (15.45, 51.63) |
| 0-6 days | Diarrheal diseases | Rotavirus | Rate | 2021 | 174.03 (114.60, 266.68) |
| 7-27 days | Diarrheal diseases | Rotavirus | Rate | 2021 | 81.38 (56.61, 118.33) |
| 0-6 days | Diarrheal diseases | Clostridioides difficile | Rate | 2021 | 0.00 (0.00, 0.01) |
| 7-27 days | Diarrheal diseases | Clostridioides difficile | Rate | 2021 | 0.01 (0.01, 0.02) |
| 1-5 months | Diarrheal diseases | Campylobacter | Rate | 2021 | 15.83 (6.21, 31.22) |
| 6-11 months | Diarrheal diseases | Campylobacter | Rate | 2021 | 11.85 (4.39, 24.42) |
| 0-6 days | Diarrheal diseases | Entamoeba | Rate | 2021 | 17.16 (6.56, 37.61) |
| 7-27 days | Diarrheal diseases | Entamoeba | Rate | 2021 | 8.26 (3.20, 17.87) |
| 2-4 years | Diarrheal diseases | Clostridioides difficile | Rate | 2021 | 0.01 (0.01, 0.02) |
| 2-4 years | Diarrheal diseases | Adenovirus | Rate | 2021 | 2.88 (1.34, 5.68) |
| 2-4 years | Diarrheal diseases | Rotavirus | Rate | 2021 | 4.64 (2.74, 7.36) |
| 12-23 months | Diarrheal diseases | Adenovirus | Rate | 2021 | 11.59 (5.39, 20.71) |
| 1-5 months | Diarrheal diseases | Adenovirus | Rate | 2021 | 47.69 (28.15, 77.75) |
| 6-11 months | Diarrheal diseases | Adenovirus | Rate | 2021 | 35.47 (18.29, 59.31) |
| 12-23 months | Diarrheal diseases | Clostridioides difficile | Rate | 2021 | 0.02 (0.01, 0.04) |
| 1-5 months | Diarrheal diseases | Clostridioides difficile | Rate | 2021 | 0.02 (0.01, 0.04) |
| 6-11 months | Diarrheal diseases | Clostridioides difficile | Rate | 2021 | 0.02 (0.01, 0.04) |
| 0-6 days | Diarrheal diseases | Norovirus | Rate | 2021 | 22.89 (7.56, 48.66) |
| 7-27 days | Diarrheal diseases | Norovirus | Rate | 2021 | 10.85 (3.64, 22.74) |
| 7-27 days | Diarrheal diseases | Non-typhoidal Salmonella | Rate | 2021 | 5.92 (0.95, 14.79) |
| 2-4 years | Diarrheal diseases | Non-typhoidal Salmonella | Rate | 2021 | 0.60 (0.00, 1.87) |
| 12-23 months | Diarrheal diseases | Enteropathogenic E coli | Rate | 2021 | 6.65 (2.51, 12.62) |
| 1-5 months | Diarrheal diseases | Enteropathogenic E coli | Rate | 2021 | 27.85 (13.37, 50.09) |
| 6-11 months | Diarrheal diseases | Enteropathogenic E coli | Rate | 2021 | 21.30 (9.95, 39.17) |
| 0-6 days | Diarrheal diseases | Enterotoxigenic E coli | Rate | 2021 | 49.34 (24.11, 95.79) |
| 7-27 days | Diarrheal diseases | Enterotoxigenic E coli | Rate | 2021 | 23.34 (12.15, 43.01) |
| 2-4 years | Diarrheal diseases | Entamoeba | Rate | 2021 | 0.78 (0.33, 1.61) |
| 12-23 months | Diarrheal diseases | Rotavirus | Rate | 2021 | 18.33 (11.56, 27.33) |
| 1-5 months | Diarrheal diseases | Rotavirus | Rate | 2021 | 66.58 (46.99, 90.76) |
| 6-11 months | Diarrheal diseases | Rotavirus | Rate | 2021 | 50.03 (31.74, 74.17) |
| 0-6 days | Diarrheal diseases | Aeromonas | Rate | 2021 | 8.02 (1.92, 21.19) |
| 7-27 days | Diarrheal diseases | Aeromonas | Rate | 2021 | 3.87 (0.97, 9.74) |
| 2-4 years | Diarrheal diseases | Norovirus | Rate | 2021 | 2.06 (0.28, 4.12) |
| 12-23 months | Diarrheal diseases | Norovirus | Rate | 2021 | 7.47 (1.09, 14.23) |

Table S10 Global Diarrhea-Related ASDR in Neonates and Children Under Five Years of Age Attributable to Specific Pathogens in 2021

| age | cause | pathogens | metric | year | ASDR |
| --- | --- | --- | --- | --- | --- |
| 7-27 days | Diarrheal diseases | Non-typhoidal Salmonella | Rate | 2021 | 537.33 (90.26, 1337.32) |
| 12-23 months | Diarrheal diseases | Non-typhoidal Salmonella | Rate | 2021 | 191.04 (0.52, 577.72) |
| 1-5 months | Diarrheal diseases | Non-typhoidal Salmonella | Rate | 2021 | 528.72 (90.70, 1219.47) |
| 6-11 months | Diarrheal diseases | Non-typhoidal Salmonella | Rate | 2021 | 399.14 (66.39, 958.96) |
| 0-6 days | Diarrheal diseases | Shigella | Rate | 2021 | 7774.80 (3641.67, 14184.79) |
| 7-27 days | Diarrheal diseases | Shigella | Rate | 2021 | 3519.24 (1726.89, 6495.63) |
| 2-4 years | Diarrheal diseases | Enterotoxigenic E coli | Rate | 2021 | 195.38 (91.98, 368.78) |
| 12-23 months | Diarrheal diseases | Entamoeba | Rate | 2021 | 233.62 (98.18, 479.35) |
| 1-5 months | Diarrheal diseases | Entamoeba | Rate | 2021 | 747.99 (307.53, 1528.96) |
| 6-11 months | Diarrheal diseases | Entamoeba | Rate | 2021 | 584.16 (240.51, 1194.89) |
| 0-6 days | Diarrheal diseases | Cryptosporidium | Rate | 2021 | 6851.85 (3505.39, 12446.41) |
| 7-27 days | Diarrheal diseases | Cryptosporidium | Rate | 2021 | 3043.35 (1629.59, 5195.25) |
| 2-4 years | Diarrheal diseases | Non-typhoidal Salmonella | Rate | 2021 | 52.77 (0.40, 163.88) |
| 12-23 months | Diarrheal diseases | Enteropathogenic E coli | Rate | 2021 | 594.20 (225.27, 1123.12) |
| 1-5 months | Diarrheal diseases | Enteropathogenic E coli | Rate | 2021 | 2521.61 (1216.45, 4539.33) |
| 6-11 months | Diarrheal diseases | Enteropathogenic E coli | Rate | 2021 | 1918.65 (903.91, 3516.51) |
| 2-4 years | Diarrheal diseases | Aeromonas | Rate | 2021 | 62.95 (25.58, 121.15) |
| 12-23 months | Diarrheal diseases | Aeromonas | Rate | 2021 | 233.68 (85.83, 478.19) |
| 1-5 months | Diarrheal diseases | Aeromonas | Rate | 2021 | 349.74 (90.62, 827.65) |
| 6-11 months | Diarrheal diseases | Aeromonas | Rate | 2021 | 277.89 (68.24, 740.92) |
| 1-5 months | Diarrheal diseases | Norovirus | Rate | 2021 | 941.07 (335.52, 1902.50) |
| 6-11 months | Diarrheal diseases | Norovirus | Rate | 2021 | 704.37 (240.70, 1497.83) |
| 0-6 days | Diarrheal diseases | Adenovirus | Rate | 2021 | 10033.14 (5225.31, 17558.75) |
| 7-27 days | Diarrheal diseases | Adenovirus | Rate | 2021 | 4757.53 (2581.52, 7998.30) |
| 0-6 days | Diarrheal diseases | Enterotoxigenic E coli | Rate | 2021 | 4449.12 (2173.35, 8632.49) |
| 7-27 days | Diarrheal diseases | Enterotoxigenic E coli | Rate | 2021 | 2121.35 (1104.44, 3903.34) |
| 2-4 years | Diarrheal diseases | Entamoeba | Rate | 2021 | 68.85 (29.75, 141.16) |
| 12-23 months | Diarrheal diseases | Rotavirus | Rate | 2021 | 1642.90 (1038.59, 2441.12) |
| 0-6 days | Diarrheal diseases | Cholera | Rate | 2021 | 1243.86 (810.83, 1761.66) |
| 7-27 days | Diarrheal diseases | Cholera | Rate | 2021 | 1165.65 (766.04, 1633.17) |
| 1-5 months | Diarrheal diseases | Rotavirus | Rate | 2021 | 6016.52 (4248.31, 8194.27) |
| 6-11 months | Diarrheal diseases | Rotavirus | Rate | 2021 | 4499.97 (2859.54, 6660.81) |
| 0-6 days | Diarrheal diseases | Aeromonas | Rate | 2021 | 723.23 (173.94, 1909.01) |
| 7-27 days | Diarrheal diseases | Aeromonas | Rate | 2021 | 352.13 (91.03, 879.05) |
| 2-4 years | Diarrheal diseases | Norovirus | Rate | 2021 | 185.64 (30.89, 361.70) |
| 12-23 months | Diarrheal diseases | Norovirus | Rate | 2021 | 670.36 (105.43, 1277.70) |
| 2-4 years | Diarrheal diseases | Shigella | Rate | 2021 | 373.97 (177.81, 709.31) |
| 12-23 months | Diarrheal diseases | Shigella | Rate | 2021 | 1281.30 (642.51, 2187.36) |
| 1-5 months | Diarrheal diseases | Shigella | Rate | 2021 | 3631.29 (1916.05, 6340.85) |
| 12-23 months | Diarrheal diseases | Enterotoxigenic E coli | Rate | 2021 | 686.68 (327.60, 1338.74) |
| 1-5 months | Diarrheal diseases | Enterotoxigenic E coli | Rate | 2021 | 2128.16 (1135.27, 3728.02) |
| 6-11 months | Diarrheal diseases | Enterotoxigenic E coli | Rate | 2021 | 1665.44 (790.43, 3092.30) |
| 0-6 days | Diarrheal diseases | Campylobacter | Rate | 2021 | 3313.66 (1182.76, 7306.88) |
| 7-27 days | Diarrheal diseases | Campylobacter | Rate | 2021 | 1633.38 (620.67, 3260.34) |
| 2-4 years | Diarrheal diseases | Cryptosporidium | Rate | 2021 | 267.83 (122.04, 490.10) |
| 12-23 months | Diarrheal diseases | Cryptosporidium | Rate | 2021 | 962.53 (439.94, 1791.80) |
| 1-5 months | Diarrheal diseases | Cryptosporidium | Rate | 2021 | 3305.78 (1893.03, 5142.95) |
| 6-11 months | Diarrheal diseases | Cryptosporidium | Rate | 2021 | 2597.29 (1390.81, 4635.71) |
| 0-6 days | Diarrheal diseases | Rotavirus | Rate | 2021 | 15682.02 (10331.68, 24018.99) |
| 7-27 days | Diarrheal diseases | Rotavirus | Rate | 2021 | 7372.73 (5128.49, 10707.10) |
| 0-6 days | Diarrheal diseases | Clostridioides difficile | Rate | 2021 | 0.34 (0.15, 0.66) |
| 7-27 days | Diarrheal diseases | Clostridioides difficile | Rate | 2021 | 1.08 (0.48, 2.11) |
| 2-4 years | Diarrheal diseases | Clostridioides difficile | Rate | 2021 | 1.10 (0.56, 1.94) |
| 2-4 years | Diarrheal diseases | Adenovirus | Rate | 2021 | 258.05 (122.32, 503.30) |
| 12-23 months | Diarrheal diseases | Adenovirus | Rate | 2021 | 1039.57 (483.04, 1852.35) |
| 1-5 months | Diarrheal diseases | Adenovirus | Rate | 2021 | 4324.67 (2557.96, 7017.54) |
| 6-11 months | Diarrheal diseases | Adenovirus | Rate | 2021 | 3201.11 (1652.43, 5339.30) |
| 2-4 years | Diarrheal diseases | Cholera | Rate | 2021 | 148.41 (96.52, 212.25) |
| 12-23 months | Diarrheal diseases | Cholera | Rate | 2021 | 437.05 (302.20, 615.47) |
| 1-5 months | Diarrheal diseases | Cholera | Rate | 2021 | 1010.25 (671.79, 1427.67) |
| 6-11 months | Diarrheal diseases | Cholera | Rate | 2021 | 736.14 (503.40, 1032.35) |
| 0-6 days | Diarrheal diseases | Non-typhoidal Salmonella | Rate | 2021 | 1134.90 (203.59, 2784.28) |
| 6-11 months | Diarrheal diseases | Shigella | Rate | 2021 | 2836.37 (1413.33, 5168.46) |
| 0-6 days | Diarrheal diseases | Enteropathogenic E coli | Rate | 2021 | 5462.16 (2395.59, 10852.86) |
| 7-27 days | Diarrheal diseases | Enteropathogenic E coli | Rate | 2021 | 2458.52 (1111.04, 4786.72) |
| 2-4 years | Diarrheal diseases | Enteropathogenic E coli | Rate | 2021 | 130.32 (44.25, 261.93) |
| 2-4 years | Diarrheal diseases | Campylobacter | Rate | 2021 | 76.10 (15.97, 182.84) |
| 12-23 months | Diarrheal diseases | Campylobacter | Rate | 2021 | 268.21 (49.01, 642.88) |
| 1-5 months | Diarrheal diseases | Campylobacter | Rate | 2021 | 1450.56 (591.81, 2839.28) |
| 6-11 months | Diarrheal diseases | Campylobacter | Rate | 2021 | 1082.15 (407.35, 2225.54) |
| 0-6 days | Diarrheal diseases | Entamoeba | Rate | 2021 | 1545.93 (592.11, 3387.79) |
| 7-27 days | Diarrheal diseases | Entamoeba | Rate | 2021 | 747.18 (292.53, 1608.25) |
| 2-4 years | Diarrheal diseases | Rotavirus | Rate | 2021 | 416.60 (250.34, 652.14) |
| 12-23 months | Diarrheal diseases | Clostridioides difficile | Rate | 2021 | 1.75 (0.86, 3.12) |
| 1-5 months | Diarrheal diseases | Clostridioides difficile | Rate | 2021 | 1.65 (0.71, 3.25) |
| 6-11 months | Diarrheal diseases | Clostridioides difficile | Rate | 2021 | 1.80 (0.85, 3.23) |
| 0-6 days | Diarrheal diseases | Norovirus | Rate | 2021 | 2072.14 (689.95, 4394.73) |
| 7-27 days | Diarrheal diseases | Norovirus | Rate | 2021 | 1003.03 (348.84, 2087.94) |

| Table S11 Frontier Analysis Based on SDI and DALYs for Rotavirus Associated Diarrheal in Neonates and Children Under Five Years of Age in 204 Countries and Territories | | | | | |
| --- | --- | --- | --- | --- | --- |
| year | val | SDI | frontier | eff_diff | location |
| 1990 | 3030.59817057979 | 0.458668935267616 | 396.343239614335 | 2634.25493096545 | China |
| 1991 | 2769.43961035203 | 0.467301969740226 | 356.737488013771 | 2412.70212233826 | China |
| 1992 | 2425.89211562916 | 0.475784030669638 | 340.365437621658 | 2085.5266780075 | China |
| 1993 | 2157.11451412841 | 0.484353830709851 | 324.684851753428 | 1832.42966237498 | China |
| 1994 | 1951.90564620882 | 0.49257864459854 | 292.414987461339 | 1659.49065874748 | China |
| 1995 | 1767.92973272943 | 0.501500907580649 | 228.836735175513 | 1539.09299755392 | China |
| 1996 | 1592.09969708203 | 0.512033995790715 | 212.720465836304 | 1379.37923124573 | China |
| 1997 | 1426.36268378902 | 0.522326866102753 | 199.422383750337 | 1226.94030003868 | China |
| 1998 | 1277.71960644474 | 0.531379484348035 | 180.462167886767 | 1097.25743855797 | China |
| 1999 | 1148.51862914223 | 0.540333688117168 | 156.561652082907 | 991.956977059323 | China |
| 2000 | 1034.47049424113 | 0.547989192872889 | 149.078919742305 | 885.391574498825 | China |
| 2001 | 894.03083801799 | 0.553680373692626 | 144.640182241385 | 749.390655776605 | China |
| 2002 | 764.93850852651 | 0.560542936414416 | 139.341430611357 | 625.597077915153 | China |
| 2003 | 674.85285220148 | 0.569018827276063 | 108.830291208456 | 566.022560993024 | China |
| 2004 | 590.26099771045 | 0.578178090807068 | 84.1702498889409 | 506.090747821509 | China |
| 2005 | 498.40650109762 | 0.58805219357166 | 82.7852660210741 | 415.621235076546 | China |
| 2006 | 410.79272488991 | 0.598758170327953 | 76.7438040277038 | 334.048920862206 | China |
| 2007 | 328.17912468635 | 0.609520246163436 | 68.4461300754033 | 259.732994610947 | China |
| 2008 | 259.93654486903 | 0.619454832781724 | 62.9281431816819 | 197.008401687348 | China |
| 2009 | 206.43123014193 | 0.62930772749348 | 55.962046322738 | 150.469183819192 | China |
| 2010 | 163.13915230204 | 0.64152144342547 | 51.036108607061 | 112.103043694979 | China |
| 2011 | 134.75928036227 | 0.651447977618785 | 45.5402114310989 | 89.2190689311711 | China |
| 2012 | 120.39235242731 | 0.657144292359298 | 45.3052056142495 | 75.0871468130605 | China |
| 2013 | 110.07568090674 | 0.66310300729639 | 40.5475692162791 | 69.5281116904609 | China |
| 2014 | 100.33360775707 | 0.668640847002655 | 39.2765594218199 | 61.0570483352501 | China |
| 2015 | 94.54190096 | 0.671940110944872 | 38.3761209261624 | 56.1657800291476 | China |
| 2016 | 85.99675953 | 0.676218437253933 | 37.9317662731524 | 48.0649932538276 | China |
| 2017 | 68.64185743 | 0.6846196607825 | 37.3143254749828 | 31.3275319576772 | China |
| 2018 | 54.45919704 | 0.693717996192013 | 36.3371296845495 | 18.1220673601505 | China |
| 2019 | 44.55438745 | 0.703686823937232 | 34.9322150037225 | 9.6221724438775 | China |
| 2020 | 51.09390493 | 0.713364584593745 | 31.2081464169944 | 19.8857585168456 | China |
| 2021 | 45.45932151 | 0.721629759719777 | 28.9764979014862 | 16.4828236128438 | China |
| 1990 | 332.2118452 | 0.497780128289885 | 235.234325273451 | 96.9775198976494 | Democratic People's Republic of Korea |
| 1991 | 319.34986073548 | 0.49912051226047 | 236.218738078032 | 83.1311226574479 | Democratic People's Republic of Korea |
| 1992 | 307.26087751418 | 0.499629841478392 | 235.31327009529 | 71.9476074188903 | Democratic People's Republic of Korea |
| 1993 | 293.41936047421 | 0.49970064634474 | 228.956108800878 | 64.463251673332 | Democratic People's Republic of Korea |
| 1994 | 281.35693357427 | 0.499531339948334 | 235.51698058377 | 45.8399529905003 | Democratic People's Republic of Korea |
| 1995 | 269.42357985157 | 0.499080529317303 | 237.577166716217 | 31.8464131353528 | Democratic People's Republic of Korea |
| 1996 | 258.86264045286 | 0.498429013609349 | 237.3405134 | 21.5221270911603 | Democratic People's Republic of Korea |
| 1997 | 250.68842933384 | 0.497136868597342 | 242.227283440927 | 8.46114589291318 | Democratic People's Republic of Korea |
| 1998 | 243.15561270916 | 0.49615920966594 | 243.15561270916 | 0 | Democratic People's Republic of Korea |
| 1999 | 235.83329988831 | 0.496484753521051 | 235.83329988831 | 0 | Democratic People's Republic of Korea |
| 2000 | 228.46776007318 | 0.497461994823193 | 228.46776007318 | 0 | Democratic People's Republic of Korea |
| 2001 | 222.13058325102 | 0.499674042505593 | 222.13058325102 | 0 | Democratic People's Republic of Korea |
| 2002 | 218.1604053 | 0.502633568731371 | 218.1604053 | 0 | Democratic People's Republic of Korea |
| 2003 | 216.02226245621 | 0.506165243055573 | 216.02226245621 | 0 | Democratic People's Republic of Korea |
| 2004 | 212.85665126887 | 0.510229134162392 | 212.232707600879 | 0.623943667990915 | Democratic People's Republic of Korea |
| 2005 | 208.40295597143 | 0.515097762842417 | 203.136730502854 | 5.26622546857635 | Democratic People's Republic of Korea |
| 2006 | 208.12821516082 | 0.519741439913669 | 198.713642528472 | 9.41457263234807 | Democratic People's Republic of Korea |
| 2007 | 210.84185159536 | 0.524066965087203 | 188.325293279005 | 22.5165583163549 | Democratic People's Republic of Korea |
| 2008 | 217.11205679935 | 0.52881349761598 | 178.607582282977 | 38.5044745163731 | Democratic People's Republic of Korea |
| 2009 | 219.51707342707 | 0.533114411172631 | 171.807899014868 | 47.7091744122019 | Democratic People's Republic of Korea |
| 2010 | 218.79405477845 | 0.53719291837066 | 159.50993313161 | 59.2841216468397 | Democratic People's Republic of Korea |
| 2011 | 210.31705879329 | 0.541212269847829 | 155.27528011951 | 55.04177867 | Democratic People's Republic of Korea |
| 2012 | 196.08884218977 | 0.545331713402902 | 153.10493770407 | 42.9839044857004 | Democratic People's Republic of Korea |
| 2013 | 179.02318546128 | 0.549483297235091 | 146.545920116513 | 32.4772653447674 | Democratic People's Republic of Korea |
| 2014 | 163.64899680605 | 0.553603559944788 | 144.030875085578 | 19.6181217204719 | Democratic People's Republic of Korea |
| 2015 | 153.09839568805 | 0.557321271453133 | 141.68928173427 | 11.4091139537798 | Democratic People's Republic of Korea |
| 2016 | 146.41733326059 | 0.560743562553338 | 138.679334270106 | 7.73799899048404 | Democratic People's Republic of Korea |
| 2017 | 140.4070505 | 0.563388288880609 | 138.196638842503 | 2.21041161299732 | Democratic People's Republic of Korea |
| 2018 | 134.17466116508 | 0.565161484391211 | 110.603969988685 | 23.5706911763947 | Democratic People's Republic of Korea |
| 2019 | 129.03851020976 | 0.566765705893725 | 109.948654297249 | 19.0898559125107 | Democratic People's Republic of Korea |
| 2020 | 125.31513173128 | 0.56818855103523 | 105.915748478551 | 19.399383252729 | Democratic People's Republic of Korea |
| 2021 | 68.27467013 | 0.569854634067777 | 68.27467013 | 0 | Democratic People's Republic of Korea |
| 1990 | 268.66816853211 | 0.667633853818721 | 39.2253663266124 | 229.442802205498 | Taiwan (Province of China) |
| 1991 | 268.93319397064 | 0.676537236907717 | 37.9048986599262 | 231.028295310714 | Taiwan (Province of China) |
| 1992 | 224.37089545899 | 0.684803635601178 | 37.3516895438754 | 187.019205915115 | Taiwan (Province of China) |
| 1993 | 188.79287353338 | 0.692924130193594 | 36.4594709519084 | 152.333402581472 | Taiwan (Province of China) |
| 1994 | 182.57117125796 | 0.701078749506874 | 35.5981584674597 | 146.9730128 | Taiwan (Province of China) |
| 1995 | 171.26606978161 | 0.709626820870811 | 33.5265297564291 | 137.739540025181 | Taiwan (Province of China) |
| 1996 | 155.45902638099 | 0.719128415346056 | 28.8548199362585 | 126.604206444731 | Taiwan (Province of China) |
| 1997 | 135.06511423527 | 0.72914549717473 | 24.7616401865535 | 110.303474048716 | Taiwan (Province of China) |
| 1998 | 117.03215990394 | 0.738125787434293 | 21.0810622827036 | 95.9510976212364 | Taiwan (Province of China) |
| 1999 | 102.68083907483 | 0.745338399386342 | 19.3117427409361 | 83.3690963338939 | Taiwan (Province of China) |
| 2000 | 99.14456805 | 0.752926841487622 | 18.7627798481066 | 80.3817882065534 | Taiwan (Province of China) |
| 2001 | 94.54741772 | 0.760829809097496 | 17.1388560855658 | 77.4085616330042 | Taiwan (Province of China) |
| 2002 | 85.7135813 | 0.769008983647841 | 14.1704659441519 | 71.5431153542681 | Taiwan (Province of China) |
| 2003 | 75.7643043 | 0.776955803379445 | 12.1648931724072 | 63.5994111310928 | Taiwan (Province of China) |
| 2004 | 104.42857105246 | 0.784677977861477 | 11.2633775057496 | 93.1651935467104 | Taiwan (Province of China) |
| 2005 | 110.17442381549 | 0.792445129331779 | 11.9728007639232 | 98.2016230515668 | Taiwan (Province of China) |
| 2006 | 102.37324109464 | 0.800174674875457 | 10.5772567396894 | 91.7959843549506 | Taiwan (Province of China) |
| 2007 | 99.88181269 | 0.808137693890723 | 8.87282755866577 | 91.0089851295642 | Taiwan (Province of China) |
| 2008 | 104.62906539714 | 0.81540069133486 | 9.19171151319917 | 95.4373538839408 | Taiwan (Province of China) |
| 2009 | 104.44904388985 | 0.821432279265941 | 8.42286578700481 | 96.0261781028452 | Taiwan (Province of China) |
| 2010 | 106.77086048567 | 0.827226752015366 | 7.60031287181269 | 99.1705476138573 | Taiwan (Province of China) |
| 2011 | 110.76546510534 | 0.832064073807912 | 7.65081525797196 | 103.114649847368 | Taiwan (Province of China) |
| 2012 | 121.80122541393 | 0.836677664574998 | 7.03734115357085 | 114.763884260359 | Taiwan (Province of China) |
| 2013 | 118.724133 | 0.841280462756768 | 6.91519119852283 | 111.808941822877 | Taiwan (Province of China) |
| 2014 | 122.31807408408 | 0.845695560406305 | 5.87927210154975 | 116.43880198253 | Taiwan (Province of China) |
| 2015 | 124.77782095705 | 0.850055268699097 | 6.5665530612241 | 118.211267895826 | Taiwan (Province of China) |
| 2016 | 118.29106665949 | 0.854466037759635 | 6.12568085407196 | 112.165385805418 | Taiwan (Province of China) |
| 2017 | 115.28924979569 | 0.858888018761485 | 6.37186434232754 | 108.917385453362 | Taiwan (Province of China) |
| 2018 | 114.32309634763 | 0.863158360983117 | 6.21040530241838 | 108.112691045212 | Taiwan (Province of China) |
| 2019 | 112.34025143913 | 0.86741184993072 | 6.05011063988268 | 106.290140799247 | Taiwan (Province of China) |
| 2020 | 112.48798321917 | 0.871247947250766 | 6.10461472456746 | 106.383368494603 | Taiwan (Province of China) |
| 2021 | 111.00388704127 | 0.874747053187929 | 6.00863086228796 | 104.995256178982 | Taiwan (Province of China) |
| 1990 | 9695.82651064261 | 0.289075059080125 | 2083.69965652905 | 7612.12685411356 | Cambodia |
| 1991 | 9437.66285778502 | 0.29247920029669 | 2136.69819299686 | 7300.96466478816 | Cambodia |
| 1992 | 8954.8749681894 | 0.296900954885372 | 2109.54875208825 | 6845.32621610115 | Cambodia |
| 1993 | 8617.38695046934 | 0.301627201679918 | 2101.93051565122 | 6515.45643481812 | Cambodia |
| 1994 | 8425.48999001013 | 0.305722592004088 | 2169.7113702487 | 6255.77861976143 | Cambodia |
| 1995 | 7935.55738095975 | 0.310678881281017 | 2119.95467028283 | 5815.60271067692 | Cambodia |
| 1996 | 7417.05856310616 | 0.31563096971546 | 2088.61587034594 | 5328.44269276022 | Cambodia |
| 1997 | 7048.83665654793 | 0.320503749678941 | 2131.03831915766 | 4917.79833739027 | Cambodia |
| 1998 | 6754.80344032583 | 0.324974298583536 | 2058.65115782197 | 4696.15228250386 | Cambodia |
| 1999 | 6374.49862494156 | 0.330224246107348 | 1990.1260867946 | 4384.37253814696 | Cambodia |
| 2000 | 5753.94677795637 | 0.336075029953322 | 1985.18025338077 | 3768.7665245756 | Cambodia |
| 2001 | 5209.51557558677 | 0.342507910583009 | 1773.94081175574 | 3435.57476383103 | Cambodia |
| 2002 | 4624.00826687404 | 0.349465712270985 | 1762.50875158762 | 2861.49951528642 | Cambodia |
| 2003 | 4057.49407026622 | 0.356929660458733 | 1712.98920556567 | 2344.50486470055 | Cambodia |
| 2004 | 3593.59394103927 | 0.364854644690823 | 1307.31441342847 | 2286.2795276108 | Cambodia |
| 2005 | 3144.51332081447 | 0.373331814716366 | 1207.89770823335 | 1936.61561258112 | Cambodia |
| 2006 | 2719.03801221818 | 0.381698166189418 | 1127.28675571365 | 1591.75125650453 | Cambodia |
| 2007 | 2384.52379238095 | 0.389876229632594 | 1024.22116028511 | 1360.30263209584 | Cambodia |
| 2008 | 2082.25998590044 | 0.397679519850279 | 979.381323450866 | 1102.87866244957 | Cambodia |
| 2009 | 1810.37861316242 | 0.404220933365413 | 878.413230426867 | 931.965382735553 | Cambodia |
| 2010 | 1598.54982343303 | 0.410211121336958 | 872.206179845394 | 726.343643587636 | Cambodia |
| 2011 | 1410.4491625644 | 0.415964408667437 | 796.330100865027 | 614.119061699373 | Cambodia |
| 2012 | 1241.44897700878 | 0.4217697537185 | 771.291555824054 | 470.157421184726 | Cambodia |
| 2013 | 1068.1446228507 | 0.427715060183387 | 525.88194770919 | 542.26267514151 | Cambodia |
| 2014 | 948.80053476545 | 0.433597748994237 | 417.818855399586 | 530.981679365864 | Cambodia |
| 2015 | 859.98726718625 | 0.43948735809131 | 421.52754989169 | 438.45971729456 | Cambodia |
| 2016 | 785.47555584625 | 0.445358172105636 | 418.885426126682 | 366.590129719568 | Cambodia |
| 2017 | 717.14262737335 | 0.451221744385535 | 410.707638523794 | 306.434988849556 | Cambodia |
| 2018 | 662.33008450954 | 0.457341489845745 | 396.855846270736 | 265.474238238804 | Cambodia |
| 2019 | 609.96245844759 | 0.463643430446969 | 381.233231454284 | 228.729226993306 | Cambodia |
| 2020 | 569.98447540431 | 0.468876053670987 | 343.982117389039 | 226.002358015271 | Cambodia |
| 2021 | 534.70739474685 | 0.473621490708013 | 334.36051115122 | 200.34688359563 | Cambodia |
| 1990 | 20266.7134655865 | 0.457134954421362 | 397.432377041824 | 19869.2810885447 | Indonesia |
| 1991 | 18429.2856054321 | 0.466505583269134 | 359.361126963054 | 18069.924478469 | Indonesia |
| 1992 | 16656.1460406276 | 0.475753157454434 | 335.116464327761 | 16321.0295762998 | Indonesia |
| 1993 | 15145.3021727378 | 0.484775240859362 | 326.228036047108 | 14819.0741366907 | Indonesia |
| 1994 | 13725.8365286305 | 0.493607099523711 | 293.813754854139 | 13432.0227737764 | Indonesia |
| 1995 | 12374.0616263377 | 0.502008381392835 | 228.020922349748 | 12146.040703988 | Indonesia |
| 1996 | 11042.3946121236 | 0.510108146446556 | 214.200280353942 | 10828.1943317697 | Indonesia |
| 1997 | 9935.30891619747 | 0.51810218881129 | 203.961889372372 | 9731.3470268251 | Indonesia |
| 1998 | 9156.71606229666 | 0.524045603868488 | 189.02371025812 | 8967.69235203854 | Indonesia |
| 1999 | 8373.14338741049 | 0.529176329629629 | 180.531624245199 | 8192.61176316529 | Indonesia |
| 2000 | 7576.02295803258 | 0.533789587270659 | 171.937266539047 | 7404.08569149353 | Indonesia |
| 2001 | 6911.552302 | 0.537977981115366 | 158.63410392149 | 6752.91819765351 | Indonesia |
| 2002 | 6224.60979679475 | 0.542245676060877 | 155.342364735453 | 6069.2674320593 | Indonesia |
| 2003 | 5579.51846723169 | 0.546550264351478 | 149.90493156731 | 5429.61353566438 | Indonesia |
| 2004 | 5030.96186784499 | 0.550902314227472 | 146.349535699514 | 4884.61233214548 | Indonesia |
| 2005 | 4630.33682885717 | 0.555424913216581 | 144.027130463633 | 4486.30969839354 | Indonesia |
| 2006 | 4281.3834547364 | 0.560265924242442 | 138.888586479223 | 4142.49486825718 | Indonesia |
| 2007 | 3999.05886082694 | 0.565811896183758 | 108.220992601066 | 3890.83786822587 | Indonesia |
| 2008 | 3729.36579870529 | 0.572225266933015 | 87.3805146067456 | 3641.98528409854 | Indonesia |
| 2009 | 3423.53162226712 | 0.578866902230273 | 85.6737905905727 | 3337.85783167655 | Indonesia |
| 2010 | 3116.13986043635 | 0.585982419406785 | 80.7535852617474 | 3035.3862751746 | Indonesia |
| 2011 | 2823.07465249602 | 0.593652984587084 | 78.7483107522732 | 2744.32634174375 | Indonesia |
| 2012 | 2573.7670763307 | 0.601185701344525 | 75.9901203102643 | 2497.77695602044 | Indonesia |
| 2013 | 2379.92639233718 | 0.608509904334102 | 68.0454838657253 | 2311.88090847145 | Indonesia |
| 2014 | 2210.9486091675 | 0.615499887091834 | 68.8375285710792 | 2142.11108059642 | Indonesia |
| 2015 | 2070.38371377859 | 0.622222469472476 | 63.0819870556321 | 2007.30172672296 | Indonesia |
| 2016 | 1931.46464810409 | 0.62866662466714 | 56.679708474885 | 1874.7849396292 | Indonesia |
| 2017 | 1880.15275253145 | 0.634895744678987 | 54.0592507889293 | 1826.09350174252 | Indonesia |
| 2018 | 1855.25639311354 | 0.640948750163115 | 51.4476365888136 | 1803.80875652473 | Indonesia |
| 2019 | 1780.46848549826 | 0.64686545689526 | 50.5122869568968 | 1729.95619854136 | Indonesia |
| 2020 | 1655.34988993681 | 0.651926647922518 | 45.5563996173398 | 1609.79349031947 | Indonesia |
| 2021 | 1565.11505002322 | 0.656868336261681 | 45.6914571743049 | 1519.42359284892 | Indonesia |
| 1990 | 26389.6937125921 | 0.264283163752662 | 2482.87509124168 | 23906.8186213504 | Lao People's Democratic Republic |
| 1991 | 24827.2626044879 | 0.268787617854408 | 2164.28720978653 | 22662.9753947014 | Lao People's Democratic Republic |
| 1992 | 23301.0426539316 | 0.273435312470827 | 2154.03203358435 | 21147.0106203472 | Lao People's Democratic Republic |
| 1993 | 22940.7584037182 | 0.278140417914198 | 2148.675156 | 20792.0832479002 | Lao People's Democratic Republic |
| 1994 | 22528.2746575313 | 0.283270229855729 | 2205.12822 | 20323.1464378493 | Lao People's Democratic Republic |
| 1995 | 19540.9469709848 | 0.288795341159004 | 2162.03901141874 | 17378.9079595661 | Lao People's Democratic Republic |
| 1996 | 18501.2794545031 | 0.294838372041673 | 2139.57388245412 | 16361.705572049 | Lao People's Democratic Republic |
| 1997 | 17328.9267939169 | 0.301477967015192 | 2127.35442872846 | 15201.5723651884 | Lao People's Democratic Republic |
| 1998 | 16265.1925547554 | 0.308402544731754 | 2126.0043620053 | 14139.1881927501 | Lao People's Democratic Republic |
| 1999 | 15181.2013484702 | 0.315972615438411 | 2093.28965754041 | 13087.9116909298 | Lao People's Democratic Republic |
| 2000 | 14204.1145234625 | 0.323947359495962 | 2075.37153532825 | 12128.7429881342 | Lao People's Democratic Republic |
| 2001 | 13018.3974890408 | 0.332580408807204 | 1986.77489377339 | 11031.6225952674 | Lao People's Democratic Republic |
| 2002 | 12536.0833604516 | 0.341667271144462 | 1863.3427989211 | 10672.7405615305 | Lao People's Democratic Republic |
| 2003 | 11945.2527261148 | 0.350658178383381 | 1780.39791013243 | 10164.8548159824 | Lao People's Democratic Republic |
| 2004 | 11396.9264448881 | 0.359681759216616 | 1572.7059527303 | 9824.2204921578 | Lao People's Democratic Republic |
| 2005 | 10719.8322262757 | 0.368728677775792 | 1235.96128025847 | 9483.87094601723 | Lao People's Democratic Republic |
| 2006 | 9924.84692737493 | 0.378122280697802 | 1125.44284223678 | 8799.40408513815 | Lao People's Democratic Republic |
| 2007 | 9072.66845752739 | 0.387723649113709 | 1094.83534778392 | 7977.83310974347 | Lao People's Democratic Republic |
| 2008 | 8813.53476660791 | 0.397011298824877 | 990.753745632868 | 7822.78102097504 | Lao People's Democratic Republic |
| 2009 | 8032.78998871803 | 0.406139021683818 | 879.11212544431 | 7153.67786327372 | Lao People's Democratic Republic |
| 2010 | 7241.11669597517 | 0.414865566497825 | 828.945653196342 | 6412.17104277883 | Lao People's Democratic Republic |
| 2011 | 6403.42749434146 | 0.42335452259834 | 683.341224857104 | 5720.08626948436 | Lao People's Democratic Republic |
| 2012 | 5865.70911077319 | 0.431149360954477 | 440.971052693973 | 5424.73805807922 | Lao People's Democratic Republic |
| 2013 | 5272.93801579556 | 0.438691038635463 | 421.523666520358 | 4851.4143492752 | Lao People's Democratic Republic |
| 2014 | 4680.98897528704 | 0.4458694242056 | 421.371260773364 | 4259.61771451368 | Lao People's Democratic Republic |
| 2015 | 4229.05756265297 | 0.452749603276049 | 408.124881902546 | 3820.93268075042 | Lao People's Democratic Republic |
| 2016 | 3760.12022129153 | 0.459583995539449 | 396.481956230093 | 3363.63826506144 | Lao People's Democratic Republic |
| 2017 | 3269.8013258704 | 0.466168891657656 | 358.103420721931 | 2911.69790514847 | Lao People's Democratic Republic |
| 2018 | 2917.8126354717 | 0.472618102538068 | 342.134456201454 | 2575.67817927025 | Lao People's Democratic Republic |
| 2019 | 2565.28589767749 | 0.478811372841658 | 324.307127914614 | 2240.97876976288 | Lao People's Democratic Republic |
| 2020 | 1731.38981436563 | 0.484192959345392 | 328.668670271733 | 1402.7211440939 | Lao People's Democratic Republic |
| 2021 | 1628.92933551023 | 0.489136091456599 | 309.636962982748 | 1319.29237252748 | Lao People's Democratic Republic |
| 1990 | 595.49639950948 | 0.545799405460294 | 150.044028232357 | 445.452371277123 | Malaysia |
| 1991 | 505.70518648551 | 0.551683548009526 | 145.772269017884 | 359.932917467626 | Malaysia |
| 1992 | 489.04975178969 | 0.558186752245966 | 141.767082552759 | 347.282669236931 | Malaysia |
| 1993 | 449.84884161116 | 0.565845656303133 | 114.733555944818 | 335.115285666342 | Malaysia |
| 1994 | 385.77503227638 | 0.574380445989544 | 84.1548128378848 | 301.620219438495 | Malaysia |
| 1995 | 377.29172239918 | 0.583441102596569 | 81.8162837740535 | 295.475438625126 | Malaysia |
| 1996 | 299.72640248639 | 0.593152135005711 | 79.5226294788425 | 220.203773007548 | Malaysia |
| 1997 | 285.98217820269 | 0.603418105152686 | 76.8357792197846 | 209.146398982905 | Malaysia |
| 1998 | 261.88022326864 | 0.612645412003281 | 69.673165371348 | 192.207057897292 | Malaysia |
| 1999 | 242.22826549062 | 0.621724901078498 | 62.4018884238315 | 179.826377066788 | Malaysia |
| 2000 | 195.77448240401 | 0.630523543762275 | 56.436535552209 | 139.337946851801 | Malaysia |
| 2001 | 184.8225216 | 0.638257476929833 | 50.8797688959499 | 133.94275271925 | Malaysia |
| 2002 | 175.08833595949 | 0.645044248017871 | 49.253418400636 | 125.834917558854 | Malaysia |
| 2003 | 164.55419602027 | 0.65088567830407 | 47.67453382 | 116.87966219862 | Malaysia |
| 2004 | 165.65772249119 | 0.656243325827196 | 45.4098527453027 | 120.247869745887 | Malaysia |
| 2005 | 174.3693183 | 0.661242241061834 | 43.2884505019424 | 131.080867752558 | Malaysia |
| 2006 | 169.22586877905 | 0.665956287100151 | 39.3792673185703 | 129.84660146048 | Malaysia |
| 2007 | 171.59688187597 | 0.670843725887123 | 38.443819059557 | 133.153062816413 | Malaysia |
| 2008 | 176.54282148666 | 0.676399910213952 | 37.9585082657212 | 138.584313220939 | Malaysia |
| 2009 | 180.12851027757 | 0.682070653985994 | 37.5452277736855 | 142.583282503885 | Malaysia |
| 2010 | 164.7288342 | 0.688258621606068 | 37.5759583702253 | 127.152875837675 | Malaysia |
| 2011 | 169.39002267587 | 0.69408340345083 | 36.2933393544784 | 133.096683321392 | Malaysia |
| 2012 | 165.48736947883 | 0.699664734135674 | 35.9146057962326 | 129.572763682597 | Malaysia |
| 2013 | 156.04555377539 | 0.70493071683803 | 34.9522200348323 | 121.093333740558 | Malaysia |
| 2014 | 163.85655525739 | 0.709953756222256 | 32.6350971874396 | 131.22145806995 | Malaysia |
| 2015 | 173.02487029709 | 0.714659871236347 | 31.9833021403676 | 141.041568156722 | Malaysia |
| 2016 | 165.61860999662 | 0.719223424980679 | 29.7692062957914 | 135.849403700829 | Malaysia |
| 2017 | 170.48393338929 | 0.724235411990887 | 29.1453046617508 | 141.338628727539 | Malaysia |
| 2018 | 163.85883374902 | 0.72938807812528 | 24.7117716203069 | 139.147062128713 | Malaysia |
| 2019 | 164.09473588131 | 0.734587751127645 | 20.7321753729647 | 143.362560508345 | Malaysia |
| 2020 | 152.11697409129 | 0.738743992650626 | 20.4601510596868 | 131.656823031603 | Malaysia |
| 2021 | 147.90110235089 | 0.742523828414751 | 19.5840523487064 | 128.317050002184 | Malaysia |
| 1990 | 13185.2120672619 | 0.331601544442323 | 1991.92551263622 | 11193.2865546257 | Maldives |
| 1991 | 11586.9813346052 | 0.344398606997735 | 1767.12592707957 | 9819.85540752563 | Maldives |
| 1992 | 10014.1426600756 | 0.358252339504646 | 1656.0580245161 | 8358.08463555951 | Maldives |
| 1993 | 8638.64383980633 | 0.372806998520911 | 1224.70843941652 | 7413.93540038981 | Maldives |
| 1994 | 7251.54178065069 | 0.388081604129663 | 1036.93478716084 | 6214.60699348985 | Maldives |
| 1995 | 5779.92611699032 | 0.403945582505039 | 883.865169940686 | 4896.06094704963 | Maldives |
| 1996 | 5285.96172449967 | 0.420519127829701 | 772.842086951975 | 4513.1196375477 | Maldives |
| 1997 | 4262.88620329213 | 0.437436025558501 | 419.41206505363 | 3843.4741382385 | Maldives |
| 1998 | 3772.75394695954 | 0.453973361371121 | 411.727741178769 | 3361.02620578077 | Maldives |
| 1999 | 3091.69775668389 | 0.469338065717424 | 341.839683916984 | 2749.85807276691 | Maldives |
| 2000 | 2764.77027772358 | 0.483495670503175 | 324.504586097821 | 2440.26569162576 | Maldives |
| 2001 | 2376.13252961304 | 0.495923695035155 | 280.592320583978 | 2095.54020902906 | Maldives |
| 2002 | 1992.88396802532 | 0.507404859766873 | 218.76596058849 | 1774.11800743683 | Maldives |
| 2003 | 1808.67614509284 | 0.518926864396739 | 198.651543757618 | 1610.02460133522 | Maldives |
| 2004 | 1619.99728237288 | 0.529910190937908 | 180.906540256109 | 1439.09074211677 | Maldives |
| 2005 | 1405.70288674699 | 0.53825333247429 | 157.210344085881 | 1248.49254266111 | Maldives |
| 2006 | 1350.16689675377 | 0.547950411527519 | 149.153304029968 | 1201.0135927238 | Maldives |
| 2007 | 1228.26941242816 | 0.557529246632533 | 141.983731749696 | 1086.28568067846 | Maldives |
| 2008 | 1124.19222721242 | 0.566839634379964 | 109.675933458917 | 1014.5162937535 | Maldives |
| 2009 | 1041.24634679107 | 0.574442059853853 | 83.1431280352021 | 958.103218755868 | Maldives |
| 2010 | 971.36639505335 | 0.581980986393534 | 78.4940653647146 | 892.872329688635 | Maldives |
| 2011 | 903.00172340405 | 0.589684459954112 | 79.7110992783032 | 823.290624125747 | Maldives |
| 2012 | 842.01180363927 | 0.596948519185372 | 76.9708868786551 | 765.040916760615 | Maldives |
| 2013 | 786.04449542356 | 0.604238519255647 | 76.6518603065625 | 709.392635116997 | Maldives |
| 2014 | 733.43300720274 | 0.611578071403856 | 69.4688522158626 | 663.964154986877 | Maldives |
| 2015 | 687.47883788308 | 0.618454389892933 | 64.1389442274576 | 623.339893655622 | Maldives |
| 2016 | 648.06152056036 | 0.624875949954596 | 59.3250076741007 | 588.736512886259 | Maldives |
| 2017 | 610.69117515686 | 0.631083288156097 | 53.0871028263552 | 557.604072330505 | Maldives |
| 2018 | 569.05205724102 | 0.637205659403498 | 49.843448044814 | 519.208609196206 | Maldives |
| 2019 | 540.90781548075 | 0.643082793641918 | 51.4526395255424 | 489.455175955208 | Maldives |
| 2020 | 502.70496802285 | 0.647608589281031 | 48.9561872639593 | 453.748780758891 | Maldives |
| 2021 | 501.02477925307 | 0.650886627343107 | 47.6489788803784 | 453.375800372692 | Maldives |
| 1990 | 30345.2976848761 | 0.319219723709677 | 2092.41989961554 | 28252.8777852606 | Myanmar |
| 1991 | 29408.8685649179 | 0.322034718697423 | 2063.0499788118 | 27345.8185861061 | Myanmar |
| 1992 | 27989.7356026262 | 0.325628883748026 | 2095.81048191073 | 25893.9251207155 | Myanmar |
| 1993 | 26380.7253435383 | 0.329707434417887 | 2070.03796427349 | 24310.6873792648 | Myanmar |
| 1994 | 24502.82795 | 0.334290120297598 | 1993.30012994766 | 22509.5278211723 | Myanmar |
| 1995 | 22603.5163407309 | 0.339590975967104 | 1859.33058348713 | 20744.1857572438 | Myanmar |
| 1996 | 20854.282395986 | 0.345191641257382 | 1771.53678508944 | 19082.7456108966 | Myanmar |
| 1997 | 19123.9035357673 | 0.350892883218861 | 1770.58378554216 | 17353.3197502251 | Myanmar |
| 1998 | 17421.52674 | 0.356696902058465 | 1710.98299006729 | 15710.5437521427 | Myanmar |
| 1999 | 15684.1917750796 | 0.36303773228042 | 1394.060385 | 14290.1313899476 | Myanmar |
| 2000 | 14031.8172119445 | 0.370360017053284 | 1223.87690662606 | 12807.9403053184 | Myanmar |
| 2001 | 12055.4717072105 | 0.378171980483692 | 1139.33775311328 | 10916.1339540972 | Myanmar |
| 2002 | 10293.0021681164 | 0.386343628220189 | 1075.72735096649 | 9217.27481714991 | Myanmar |
| 2003 | 8803.80658097414 | 0.395218766380276 | 976.957604848947 | 7826.84897612519 | Myanmar |
| 2004 | 8103.3372259578 | 0.40469235815513 | 887.691885930247 | 7215.64534002755 | Myanmar |
| 2005 | 7405.70533499667 | 0.414662082498037 | 852.504470258242 | 6553.20086473843 | Myanmar |
| 2006 | 6682.93925411351 | 0.424705070565889 | 693.224987089524 | 5989.71426702399 | Myanmar |
| 2007 | 5996.59941413373 | 0.434536927066905 | 417.999634326231 | 5578.5997798075 | Myanmar |
| 2008 | 5527.81514598633 | 0.443595531234043 | 424.004570785099 | 5103.81057520123 | Myanmar |
| 2009 | 5046.31794589361 | 0.451954883959276 | 409.690778561332 | 4636.62716733228 | Myanmar |
| 2010 | 4679.91417072624 | 0.460180429354898 | 395.483650577397 | 4284.43052014884 | Myanmar |
| 2011 | 4362.98775035036 | 0.467850871609568 | 362.213206922317 | 4000.77454342804 | Myanmar |
| 2012 | 4068.45013951697 | 0.475336422547245 | 335.305921218747 | 3733.14421829822 | Myanmar |
| 2013 | 3843.28494606109 | 0.482821855058075 | 324.960082963654 | 3518.32486309744 | Myanmar |
| 2014 | 3588.34656864283 | 0.490255145456161 | 311.449170367079 | 3276.89739827575 | Myanmar |
| 2015 | 3337.67798636297 | 0.497374156127321 | 245.887695596041 | 3091.79029076693 | Myanmar |
| 2016 | 3157.59986019962 | 0.504115978117272 | 223.68220149786 | 2933.91765870176 | Myanmar |
| 2017 | 2916.30895860767 | 0.510823431317416 | 212.949044671766 | 2703.3599139359 | Myanmar |
| 2018 | 2674.63049822191 | 0.517557469010629 | 203.853052911653 | 2470.77744531026 | Myanmar |
| 2019 | 2470.81428141035 | 0.524108970350153 | 188.878596425212 | 2281.93568498514 | Myanmar |
| 2020 | 1869.77712143292 | 0.529434702877063 | 180.187484992254 | 1689.58963644067 | Myanmar |
| 2021 | 1613.46705699017 | 0.533900839611906 | 164.29610224823 | 1449.17095474194 | Myanmar |
| 1990 | 4962.94824262478 | 0.510011796397722 | 214.327282094193 | 4748.62096053059 | Philippines |
| 1991 | 4509.27780509727 | 0.513707183203398 | 208.380982463769 | 4300.8968226335 | Philippines |
| 1992 | 4166.02328639449 | 0.517109502298333 | 203.584077985691 | 3962.4392084088 | Philippines |
| 1993 | 3865.85531612792 | 0.520600378172241 | 199.634325353426 | 3666.22099077449 | Philippines |
| 1994 | 3454.59335304059 | 0.524613606961947 | 190.56530064259 | 3264.028052 | Philippines |
| 1995 | 3148.35042661264 | 0.528827907097189 | 180.212266068461 | 2968.13816054418 | Philippines |
| 1996 | 2840.87324626548 | 0.532938848718821 | 169.262087300532 | 2671.61115896495 | Philippines |
| 1997 | 2574.73588285309 | 0.536589867582292 | 157.994537591168 | 2416.74134526192 | Philippines |
| 1998 | 2348.83034409217 | 0.539496470377039 | 156.831511770875 | 2191.9988323213 | Philippines |
| 1999 | 2339.81122078443 | 0.542257968063782 | 155.116984646309 | 2184.69423613812 | Philippines |
| 2000 | 2282.43790446777 | 0.544997722532077 | 152.028650605133 | 2130.40925386264 | Philippines |
| 2001 | 2151.11864839922 | 0.547409431560733 | 149.772285533752 | 2001.34636286547 | Philippines |
| 2002 | 1988.48411225726 | 0.549521153378409 | 146.160052576657 | 1842.3240596806 | Philippines |
| 2003 | 1821.84970628149 | 0.551567967518738 | 146.243648421116 | 1675.60605786037 | Philippines |
| 2004 | 1624.2335185863 | 0.553704287358934 | 143.677878941256 | 1480.55563964504 | Philippines |
| 2005 | 1589.02535476294 | 0.555664102826939 | 143.742157020147 | 1445.28319774279 | Philippines |
| 2006 | 1512.09102955756 | 0.557976265254754 | 142.028903385639 | 1370.06212617192 | Philippines |
| 2007 | 1447.52160489453 | 0.560822023745201 | 138.901155925833 | 1308.6204489687 | Philippines |
| 2008 | 1379.91102345946 | 0.563974721376474 | 137.436612769437 | 1242.47441069002 | Philippines |
| 2009 | 1318.80348920419 | 0.567355819963632 | 109.762734027648 | 1209.04075517654 | Philippines |
| 2010 | 1244.58911193725 | 0.571765888250344 | 85.2954598055016 | 1159.29365213175 | Philippines |
| 2011 | 1169.54679691477 | 0.577240283466517 | 82.0745019076675 | 1087.4722950071 | Philippines |
| 2012 | 1033.4164866161 | 0.58376762501942 | 81.1399804395102 | 952.27650617659 | Philippines |
| 2013 | 896.77108812698 | 0.590837549266941 | 80.8495911449607 | 815.921496982019 | Philippines |
| 2014 | 810.52440025834 | 0.598384520042983 | 76.8842187560951 | 733.640181502245 | Philippines |
| 2015 | 746.79163871499 | 0.606215624393378 | 75.4168501671028 | 671.374788547887 | Philippines |
| 2016 | 745.12289771218 | 0.614224960974851 | 66.2620864747145 | 678.860811237465 | Philippines |
| 2017 | 739.47157199569 | 0.622315028960912 | 62.7712520998565 | 676.700319895834 | Philippines |
| 2018 | 736.03545911551 | 0.630464275930184 | 56.0018384598775 | 680.033620655632 | Philippines |
| 2019 | 725.6630645 | 0.638547384381803 | 50.5374145286878 | 675.125649934212 | Philippines |
| 2020 | 668.68896864554 | 0.644867233233664 | 49.9840558965709 | 618.704912748969 | Philippines |
| 2021 | 640.70314336649 | 0.651219328702707 | 47.6739230044456 | 593.029220362044 | Philippines |
| 1990 | 2078.89054268437 | 0.522622552851521 | 199.040708485214 | 1879.84983419916 | Sri Lanka |
| 1991 | 1708.6243712882 | 0.528239202759485 | 180.388934729775 | 1528.23543655843 | Sri Lanka |
| 1992 | 1555.9364385859 | 0.533345535471094 | 170.564887677922 | 1385.37155090798 | Sri Lanka |
| 1993 | 1481.1864727072 | 0.538891102297622 | 156.882193384282 | 1324.30427932292 | Sri Lanka |
| 1994 | 1352.26821149296 | 0.545124551180714 | 152.208809721992 | 1200.05940177097 | Sri Lanka |
| 1995 | 1109.91869457327 | 0.551744058346111 | 146.367298866744 | 963.551395706526 | Sri Lanka |
| 1996 | 1056.38349014018 | 0.558938887614985 | 142.170003560549 | 914.21348657963 | Sri Lanka |
| 1997 | 945.00789608373 | 0.566128422363729 | 112.34043836904 | 832.66745771469 | Sri Lanka |
| 1998 | 796.8423768 | 0.572326186668561 | 83.6049458059911 | 713.237430944709 | Sri Lanka |
| 1999 | 661.35903269624 | 0.57763004764168 | 80.5360927008357 | 580.822939995404 | Sri Lanka |
| 2000 | 602.11634018164 | 0.582942637570578 | 85.4097468929715 | 516.706593288669 | Sri Lanka |
| 2001 | 561.63176044602 | 0.587556599070554 | 83.283512061867 | 478.348248384153 | Sri Lanka |
| 2002 | 498.10803941935 | 0.59235912694583 | 79.0581652624083 | 419.049874156942 | Sri Lanka |
| 2003 | 462.87308867279 | 0.597410284061105 | 78.3924336392441 | 384.480655033546 | Sri Lanka |
| 2004 | 438.42336018562 | 0.60225006214936 | 74.844610294229 | 363.578749891391 | Sri Lanka |
| 2005 | 451.08757547959 | 0.606906655593188 | 74.5069041228359 | 376.580671356754 | Sri Lanka |
| 2006 | 410.03055929066 | 0.611807083317854 | 68.3615811520534 | 341.668978138607 | Sri Lanka |
| 2007 | 374.04504616973 | 0.616979265050006 | 63.4495257411607 | 310.595520428569 | Sri Lanka |
| 2008 | 366.40535254668 | 0.622414317259024 | 64.9417668615463 | 301.463585685134 | Sri Lanka |
| 2009 | 359.06488959265 | 0.628149630493025 | 58.1975810232941 | 300.867308569356 | Sri Lanka |
| 2010 | 337.84933287892 | 0.634263123413356 | 53.1652103055862 | 284.684122573334 | Sri Lanka |
| 2011 | 310.24417249595 | 0.640799571244289 | 51.3378517555189 | 258.906320740431 | Sri Lanka |
| 2012 | 281.53884874206 | 0.648049815659369 | 49.7662284347524 | 231.772620307308 | Sri Lanka |
| 2013 | 268.23417781365 | 0.655435837718287 | 45.8727114629653 | 222.361466350685 | Sri Lanka |
| 2014 | 243.70738576413 | 0.662782434226903 | 40.7650118919272 | 202.942373872203 | Sri Lanka |
| 2015 | 221.52797994731 | 0.669760291875565 | 38.5078581585911 | 183.020121788719 | Sri Lanka |
| 2016 | 206.95054550245 | 0.67627744148358 | 37.9848535322907 | 168.965691970159 | Sri Lanka |
| 2017 | 195.11355263516 | 0.682405274024772 | 37.5237064893257 | 157.589846145834 | Sri Lanka |
| 2018 | 185.61339536445 | 0.688125858195178 | 37.0888149750596 | 148.52458038939 | Sri Lanka |
| 2019 | 177.12949951613 | 0.69351606326589 | 36.8084286106925 | 140.321070905438 | Sri Lanka |
| 2020 | 170.19918598367 | 0.697648535082413 | 35.628427398756 | 134.570758584914 | Sri Lanka |
| 2021 | 152.48815838478 | 0.70153493519423 | 35.9267177330232 | 116.561440651757 | Sri Lanka |
| 1990 | 1957.9759826577 | 0.506644860754466 | 219.144512141949 | 1738.83147051575 | Thailand |
| 1991 | 2006.59766617324 | 0.515911465809998 | 203.109810149903 | 1803.48785602334 | Thailand |
| 1992 | 1963.68537098366 | 0.525161868897941 | 190.58438344222 | 1773.10098754144 | Thailand |
| 1993 | 1860.57984936718 | 0.534303709087494 | 162.772477785611 | 1697.80737158157 | Thailand |
| 1994 | 1715.69864121904 | 0.543325546554827 | 155.2807474 | 1560.41789385724 | Thailand |
| 1995 | 1549.91750247533 | 0.552452208814004 | 145.737169348877 | 1404.18033312645 | Thailand |
| 1996 | 1345.99058802676 | 0.561302486534141 | 138.645057532443 | 1207.34553049432 | Thailand |
| 1997 | 1154.21952088315 | 0.568449492093676 | 109.420719713141 | 1044.79880117001 | Thailand |
| 1998 | 1024.07313446821 | 0.573817902004847 | 86.1333566856573 | 937.939777782553 | Thailand |
| 1999 | 908.95953755896 | 0.578854167794919 | 84.829944439049 | 824.129593119911 | Thailand |
| 2000 | 852.14898460008 | 0.58350091705013 | 84.950507242846 | 767.198477357234 | Thailand |
| 2001 | 819.72191188141 | 0.587661560775708 | 82.2233110891439 | 737.498600792266 | Thailand |
| 2002 | 854.43916054131 | 0.59175909789129 | 78.7962073729775 | 775.642953168332 | Thailand |
| 2003 | 896.58132750407 | 0.596048920136592 | 77.0686325261054 | 819.512694977965 | Thailand |
| 2004 | 929.93364061729 | 0.600520665140167 | 76.8151893259219 | 853.118451291368 | Thailand |
| 2005 | 912.57123896664 | 0.605040775160747 | 76.2915998471471 | 836.279639119493 | Thailand |
| 2006 | 894.12047613481 | 0.610356678024317 | 68.7702421297344 | 825.350234005076 | Thailand |
| 2007 | 877.61148722147 | 0.616368591131489 | 67.8010767497692 | 809.810410471701 | Thailand |
| 2008 | 827.18953440986 | 0.621067542847306 | 64.1562589317286 | 763.033275478131 | Thailand |
| 2009 | 762.7636731 | 0.624329070216978 | 59.0914964148855 | 703.672176671814 | Thailand |
| 2010 | 735.28828959113 | 0.62858943983977 | 55.3587161519253 | 679.929573439205 | Thailand |
| 2011 | 668.77151596392 | 0.633226727672561 | 53.533307847986 | 615.238208115934 | Thailand |
| 2012 | 593.31810379107 | 0.638410972213962 | 50.0165620696808 | 543.301541721389 | Thailand |
| 2013 | 495.40732326963 | 0.643672915801455 | 50.9604862878096 | 444.44683698182 | Thailand |
| 2014 | 430.33316035143 | 0.648772057471972 | 47.1733429545719 | 383.159817396858 | Thailand |
| 2015 | 394.96587445034 | 0.654015622617677 | 45.7613628704394 | 349.204511579901 | Thailand |
| 2016 | 390.43373122555 | 0.659369302952365 | 43.2125336295425 | 347.221197596007 | Thailand |
| 2017 | 366.36719089201 | 0.664804133690469 | 40.7347592525527 | 325.632431639457 | Thailand |
| 2018 | 357.79758958604 | 0.670225586853729 | 38.4628731335566 | 319.334716452483 | Thailand |
| 2019 | 334.2105461 | 0.675273900833315 | 37.9671760787744 | 296.243370015126 | Thailand |
| 2020 | 309.02337631905 | 0.679119989171207 | 37.4233831476751 | 271.599993171375 | Thailand |
| 2021 | 289.23911921441 | 0.682547932974593 | 37.542245107755 | 251.696874106655 | Thailand |
| 1990 | 26752.8607867954 | 0.262468083434918 | 2709.69490163446 | 24043.1658851609 | Timor-Leste |
| 1991 | 25051.4507021236 | 0.270943348789144 | 2132.29526682252 | 22919.1554353011 | Timor-Leste |
| 1992 | 23271.4702010954 | 0.279916760012752 | 2135.50739113865 | 21135.9628099567 | Timor-Leste |
| 1993 | 21532.0633569255 | 0.289266628937942 | 2129.47964879301 | 19402.5837081325 | Timor-Leste |
| 1994 | 19715.1875981332 | 0.298763188841394 | 2145.90946374102 | 17569.2781343922 | Timor-Leste |
| 1995 | 17888.5151127814 | 0.308018683645478 | 2141.10660524351 | 15747.4085075379 | Timor-Leste |
| 1996 | 16110.5615228331 | 0.317588750054214 | 2143.9422540305 | 13966.6192688026 | Timor-Leste |
| 1997 | 14475.6354519064 | 0.326705373863583 | 2067.16784114747 | 12408.4676107589 | Timor-Leste |
| 1998 | 13010.3137123666 | 0.334894497001343 | 1991.54646787663 | 11018.76724 | Timor-Leste |
| 1999 | 12084.255342025 | 0.339252140436899 | 1870.28056141045 | 10213.9747806145 | Timor-Leste |
| 2000 | 10973.7705976422 | 0.343793649201481 | 1773.75016727989 | 9200.02043036231 | Timor-Leste |
| 2001 | 9679.89195040799 | 0.349490320159863 | 1771.02550979274 | 7908.86644061525 | Timor-Leste |
| 2002 | 8614.37888669026 | 0.354162019516716 | 1764.32748055836 | 6850.0514061319 | Timor-Leste |
| 2003 | 7890.93393108976 | 0.35844002540281 | 1644.6979224477 | 6246.23600864206 | Timor-Leste |
| 2004 | 6671.36506193757 | 0.362687998477417 | 1422.06478636314 | 5249.30027557443 | Timor-Leste |
| 2005 | 5988.02163262844 | 0.366913584279333 | 1251.17407781216 | 4736.84755481628 | Timor-Leste |
| 2006 | 5149.79670935109 | 0.370654092006941 | 1199.6406865813 | 3950.15602276979 | Timor-Leste |
| 2007 | 4577.93901198498 | 0.375390767982559 | 1175.09182579276 | 3402.84718619222 | Timor-Leste |
| 2008 | 3922.18861769689 | 0.381227527778766 | 1126.77991172854 | 2795.40870596835 | Timor-Leste |
| 2009 | 3595.40967158009 | 0.387944823498752 | 1086.01088252435 | 2509.39878905574 | Timor-Leste |
| 2010 | 3275.16775874606 | 0.394887019369648 | 972.801431431843 | 2302.36632731422 | Timor-Leste |
| 2011 | 3108.49702572522 | 0.401872356301348 | 933.791708783079 | 2174.70531694214 | Timor-Leste |
| 2012 | 2909.10236219773 | 0.408910300117845 | 873.18916669942 | 2035.91319549831 | Timor-Leste |
| 2013 | 2672.74426289712 | 0.41521787782997 | 825.84377608176 | 1846.90048681536 | Timor-Leste |
| 2014 | 2519.51084019637 | 0.421155537971188 | 776.255299785284 | 1743.25554041109 | Timor-Leste |
| 2015 | 2408.03158691406 | 0.426383620035982 | 546.55499213718 | 1861.47659477688 | Timor-Leste |
| 2016 | 2211.19917680739 | 0.431207272013621 | 443.722855108497 | 1767.47632169889 | Timor-Leste |
| 2017 | 1924.56769191147 | 0.434519733594658 | 421.555922105655 | 1503.01176980581 | Timor-Leste |
| 2018 | 1676.95132046996 | 0.437018662969572 | 424.353619842275 | 1252.59770062768 | Timor-Leste |
| 2019 | 1427.83670482469 | 0.44019584549571 | 417.035150658708 | 1010.80155416598 | Timor-Leste |
| 2020 | 1396.53248106977 | 0.442349336035765 | 422.213671257538 | 974.318809812232 | Timor-Leste |
| 2021 | 1317.70637992162 | 0.444667618881833 | 427.509969459569 | 890.196410462051 | Timor-Leste |
| 1990 | 1701.07347058043 | 0.407630047693296 | 876.383603747718 | 824.689866832712 | Viet Nam |
| 1991 | 1505.7117331921 | 0.413217249557429 | 856.927150783968 | 648.784582408132 | Viet Nam |
| 1992 | 1363.07684317406 | 0.419781657140794 | 775.339208832231 | 587.737634341829 | Viet Nam |
| 1993 | 1152.57756464444 | 0.427072184949202 | 548.432470787033 | 604.145093857407 | Viet Nam |
| 1994 | 1016.72048868479 | 0.435106272492167 | 425.603604480814 | 591.116884203976 | Viet Nam |
| 1995 | 889.7881383 | 0.443858785203356 | 420.878047147029 | 468.910091178071 | Viet Nam |
| 1996 | 774.65032785133 | 0.453102410398964 | 409.352177563133 | 365.298150288197 | Viet Nam |
| 1997 | 689.32907467609 | 0.462853518365539 | 376.803578129505 | 312.525496546585 | Viet Nam |
| 1998 | 624.6254371 | 0.47247889159957 | 342.64279857843 | 281.98263856987 | Viet Nam |
| 1999 | 568.60313126718 | 0.48144577410536 | 324.10719596532 | 244.495935301859 | Viet Nam |
| 2000 | 514.33333094719 | 0.489802045414549 | 310.541589813774 | 203.791741133416 | Viet Nam |
| 2001 | 462.68444301397 | 0.497630349065707 | 235.50018408007 | 227.1842589 | Viet Nam |
| 2002 | 422.68707250447 | 0.505453447065355 | 223.506601652532 | 199.180470851938 | Viet Nam |
| 2003 | 399.8207846 | 0.513367511118634 | 207.093153982174 | 192.727630628826 | Viet Nam |
| 2004 | 379.11439259851 | 0.521229389687461 | 199.182576062041 | 179.931816536469 | Viet Nam |
| 2005 | 347.21547455316 | 0.528956332075378 | 181.504577942777 | 165.710896610383 | Viet Nam |
| 2006 | 310.5822692 | 0.53630065301368 | 157.776242897452 | 152.806026277248 | Viet Nam |
| 2007 | 277.55254042863 | 0.543342652110377 | 155.485965401457 | 122.066575027173 | Viet Nam |
| 2008 | 251.95807611128 | 0.550127119086299 | 146.260317572758 | 105.697758538522 | Viet Nam |
| 2009 | 228.80295330646 | 0.55645594448371 | 144.714604922247 | 84.0883483842131 | Viet Nam |
| 2010 | 209.72257290263 | 0.562768571224979 | 139.015138967575 | 70.7074339350548 | Viet Nam |
| 2011 | 197.44913266827 | 0.568945458254757 | 108.496645222283 | 88.9524874459873 | Viet Nam |
| 2012 | 182.33913865943 | 0.575078671720175 | 82.9836761666251 | 99.3554624928049 | Viet Nam |
| 2013 | 167.40673987814 | 0.581029214733769 | 85.9379654887149 | 81.4687743894251 | Viet Nam |
| 2014 | 155.24045452789 | 0.586913814919078 | 78.3809199407661 | 76.8595345871239 | Viet Nam |
| 2015 | 148.77181739313 | 0.592849573281283 | 77.7210761669937 | 71.0507412261363 | Viet Nam |
| 2016 | 139.13428491372 | 0.598849485299412 | 76.9303945795512 | 62.2038903341688 | Viet Nam |
| 2017 | 127.78797767353 | 0.605019420569412 | 75.5102901262335 | 52.2776875472965 | Viet Nam |
| 2018 | 117.65718639318 | 0.611351406113218 | 69.823582906314 | 47.833603486866 | Viet Nam |
| 2019 | 110.47502961126 | 0.6177338152062 | 63.737017060247 | 46.738012551013 | Viet Nam |
| 2020 | 106.54644793949 | 0.623090320139283 | 62.1496041535941 | 44.3968437858959 | Viet Nam |
| 2021 | 52.28444392 | 0.627933720680671 | 51.7787237059433 | 0.505720213696662 | Viet Nam |
| 1990 | 1151.33332117303 | 0.53464890774309 | 162.233118916102 | 989.100202256928 | Fiji |
| 1991 | 1133.05211695557 | 0.538778259951718 | 157.275811180813 | 975.776305774757 | Fiji |
| 1992 | 1076.48823976858 | 0.543188551973659 | 155.424020335465 | 921.064219433115 | Fiji |
| 1993 | 1087.4983689727 | 0.547776473718793 | 148.865626729062 | 938.632742243638 | Fiji |
| 1994 | 1066.15177897158 | 0.552849019704515 | 146.499345212512 | 919.652433759068 | Fiji |
| 1995 | 1031.41691446882 | 0.558530972290103 | 142.098306351084 | 889.318608117736 | Fiji |
| 1996 | 1008.12359571796 | 0.565041878958725 | 111.397986033465 | 896.725609684495 | Fiji |
| 1997 | 979.27604279266 | 0.571187752703497 | 85.9989447409733 | 893.277098051687 | Fiji |
| 1998 | 1015.69512993261 | 0.577172353765379 | 83.3002336801737 | 932.394896252436 | Fiji |
| 1999 | 1000.30240350683 | 0.583573942431191 | 81.2149677590357 | 919.087435747794 | Fiji |
| 2000 | 980.53182488496 | 0.589196631128333 | 79.858035607619 | 900.673789277341 | Fiji |
| 2001 | 957.62810824388 | 0.59449741944042 | 77.3523504665023 | 880.275757777378 | Fiji |
| 2002 | 923.98135523834 | 0.59932203091066 | 75.0279728119228 | 848.953382426417 | Fiji |
| 2003 | 886.80551547457 | 0.603489622121197 | 77.4807530764188 | 809.324762398151 | Fiji |
| 2004 | 885.85028704503 | 0.607544889853603 | 74.998771635928 | 810.851515409102 | Fiji |
| 2005 | 871.3917645 | 0.610812943594047 | 69.9063293773179 | 801.485435088082 | Fiji |
| 2006 | 887.74980639406 | 0.613932767413255 | 68.7054296470499 | 819.04437674701 | Fiji |
| 2007 | 920.02142546305 | 0.616582915169589 | 62.253475697133 | 857.767949765917 | Fiji |
| 2008 | 915.75649024089 | 0.618958809102014 | 62.1309277899701 | 853.62556245092 | Fiji |
| 2009 | 914.08957750373 | 0.620883465082065 | 64.1206853948524 | 849.968892108878 | Fiji |
| 2010 | 916.69356547377 | 0.62330878605608 | 61.8946373395693 | 854.798928134201 | Fiji |
| 2011 | 844.71478256953 | 0.626438251319165 | 57.7764847496905 | 786.93829781984 | Fiji |
| 2012 | 699.96099055101 | 0.629881334893454 | 56.4857521095632 | 643.475238441447 | Fiji |
| 2013 | 483.76871604249 | 0.634278114312534 | 54.5943762842883 | 429.174339758202 | Fiji |
| 2014 | 360.89163724293 | 0.6393900664467 | 51.5283686560251 | 309.363268586905 | Fiji |
| 2015 | 309.00647470164 | 0.645026352546626 | 48.7608227852941 | 260.245651916346 | Fiji |
| 2016 | 300.5758436 | 0.650742680102873 | 46.7015751449551 | 253.874268468945 | Fiji |
| 2017 | 286.93469030207 | 0.656464010258719 | 45.3343677583861 | 241.600322543684 | Fiji |
| 2018 | 274.47492553791 | 0.661991453647996 | 42.7918482530701 | 231.68307728484 | Fiji |
| 2019 | 259.75552095196 | 0.667060979290814 | 39.1302835180059 | 220.625237433954 | Fiji |
| 2020 | 294.02708749592 | 0.671431490601499 | 38.4078888053294 | 255.619198690591 | Fiji |
| 2021 | 230.80115639516 | 0.675051630810876 | 38.0142017 | 192.78695469695 | Fiji |
| 1990 | 8710.46668842741 | 0.410389821247376 | 863.61929098203 | 7846.84739744538 | Kiribati |
| 1991 | 8255.88102841033 | 0.412884199130178 | 857.234150345186 | 7398.64687806514 | Kiribati |
| 1992 | 7767.05394728415 | 0.415577609430112 | 795.649190748499 | 6971.40475653565 | Kiribati |
| 1993 | 7292.84200378813 | 0.418481660624087 | 795.969152379693 | 6496.87285140844 | Kiribati |
| 1994 | 6788.22676626431 | 0.421716217125176 | 779.007215965932 | 6009.21955029838 | Kiribati |
| 1995 | 6350.97044467138 | 0.425024354020195 | 686.699799798513 | 5664.27064487287 | Kiribati |
| 1996 | 5998.18195648745 | 0.428300682254543 | 513.666216236196 | 5484.51574025125 | Kiribati |
| 1997 | 5596.38038006248 | 0.431641940565898 | 430.502617362205 | 5165.87776270027 | Kiribati |
| 1998 | 5232.51970967921 | 0.435741904126434 | 422.363229420839 | 4810.15648025837 | Kiribati |
| 1999 | 5148.41325908875 | 0.439692923227333 | 417.853972331119 | 4730.55928675763 | Kiribati |
| 2000 | 4839.34641535417 | 0.444436339062538 | 420.418442233008 | 4418.92797312116 | Kiribati |
| 2001 | 4534.54485798378 | 0.448782300735609 | 405.390038964228 | 4129.15481901955 | Kiribati |
| 2002 | 4219.4185604438 | 0.453462297779448 | 411.993206019723 | 3807.42535442408 | Kiribati |
| 2003 | 3926.14278038181 | 0.458208373737044 | 398.913980961409 | 3527.2287994204 | Kiribati |
| 2004 | 3657.18923649579 | 0.462336213231137 | 377.615612809512 | 3279.57362368628 | Kiribati |
| 2005 | 3480.1204433423 | 0.466722276344394 | 364.134110203096 | 3115.9863331392 | Kiribati |
| 2006 | 3353.48678607357 | 0.470557196660738 | 342.343658139946 | 3011.14312793362 | Kiribati |
| 2007 | 3401.65667407571 | 0.474227450118041 | 340.19576059821 | 3061.4609134775 | Kiribati |
| 2008 | 3346.07155806302 | 0.47720193306214 | 337.229426833232 | 3008.84213122979 | Kiribati |
| 2009 | 3292.72250859936 | 0.480035601443872 | 325.382125463083 | 2967.34038313628 | Kiribati |
| 2010 | 3251.74926197877 | 0.482447689410665 | 324.308808206411 | 2927.44045377236 | Kiribati |
| 2011 | 3126.99170867471 | 0.485028211089828 | 324.781631149117 | 2802.21007752559 | Kiribati |
| 2012 | 2812.89039991659 | 0.488175863692029 | 311.322692506803 | 2501.56770740979 | Kiribati |
| 2013 | 2537.76365527273 | 0.491880767182311 | 298.865706324403 | 2238.89794894833 | Kiribati |
| 2014 | 2294.25948122552 | 0.495317241227584 | 280.914181016602 | 2013.34530020892 | Kiribati |
| 2015 | 1967.1877751223 | 0.500059699036215 | 230.201008012371 | 1736.98676710993 | Kiribati |
| 2016 | 1831.29477790511 | 0.505207837257253 | 222.609548686337 | 1608.68522921877 | Kiribati |
| 2017 | 1728.87037220173 | 0.510019240979789 | 214.540511460837 | 1514.32986074089 | Kiribati |
| 2018 | 1603.85483358302 | 0.514810383548853 | 206.645460006486 | 1397.20937357653 | Kiribati |
| 2019 | 1503.40449480818 | 0.519441321098362 | 199.196512787386 | 1304.20798202079 | Kiribati |
| 2020 | 1491.53912222751 | 0.523454113096381 | 190.306393888984 | 1301.23272833853 | Kiribati |
| 2021 | 1395.32765961495 | 0.527186582842681 | 191.033505399625 | 1204.29415421532 | Kiribati |
| 1990 | 1297.07102336912 | 0.430839288199048 | 439.749275499629 | 857.321747869491 | Marshall Islands |
| 1991 | 1275.22606194182 | 0.435571411977162 | 420.036948147727 | 855.189113794093 | Marshall Islands |
| 1992 | 1208.53794169406 | 0.440491018138956 | 418.611325961682 | 789.926615732378 | Marshall Islands |
| 1993 | 1130.59155452341 | 0.445479084259875 | 421.137202951144 | 709.454351572266 | Marshall Islands |
| 1994 | 1064.37880135078 | 0.450848221273844 | 412.843604684208 | 651.535196666572 | Marshall Islands |
| 1995 | 1005.53124978143 | 0.45685967435209 | 399.966182994377 | 605.565066787053 | Marshall Islands |
| 1996 | 973.19133881197 | 0.461209430189201 | 380.534039057233 | 592.657299754737 | Marshall Islands |
| 1997 | 926.30191966443 | 0.464752728246042 | 379.845942 | 546.45597766923 | Marshall Islands |
| 1998 | 912.45428291736 | 0.468133032354917 | 359.198883688836 | 553.255399228524 | Marshall Islands |
| 1999 | 908.23169444973 | 0.471292726535272 | 344.06264014025 | 564.16905430948 | Marshall Islands |
| 2000 | 3115.81377089106 | 0.474562536385418 | 336.396582715925 | 2779.41718817513 | Marshall Islands |
| 2001 | 877.08510772806 | 0.478588316369524 | 322.657651535492 | 554.427456192568 | Marshall Islands |
| 2002 | 861.78497751213 | 0.482776259493159 | 324.469232375859 | 537.315745136271 | Marshall Islands |
| 2003 | 861.46797538575 | 0.486248620277286 | 318.91718832841 | 542.55078705734 | Marshall Islands |
| 2004 | 849.40154147667 | 0.489573077431328 | 310.668375592667 | 538.733165884003 | Marshall Islands |
| 2005 | 838.25874620482 | 0.49327139054966 | 294.477147658382 | 543.781598546438 | Marshall Islands |
| 2006 | 795.05978763132 | 0.497456174718598 | 244.134690935946 | 550.925096695374 | Marshall Islands |
| 2007 | 734.50885163884 | 0.502250042471655 | 231.222247426588 | 503.286604212252 | Marshall Islands |
| 2008 | 653.45174333991 | 0.506359587700651 | 219.314757590249 | 434.136985749661 | Marshall Islands |
| 2009 | 571.07834350099 | 0.510794535917681 | 213.480651127189 | 357.597692373801 | Marshall Islands |
| 2010 | 539.40781560877 | 0.51608513761736 | 203.537177324001 | 335.870638284769 | Marshall Islands |
| 2011 | 495.74844470161 | 0.521177086955837 | 198.793313469893 | 296.955131231717 | Marshall Islands |
| 2012 | 452.56767103873 | 0.526042019095978 | 190.298684834202 | 262.268986204528 | Marshall Islands |
| 2013 | 411.37405 | 0.531446300319586 | 179.753813697144 | 231.620236307056 | Marshall Islands |
| 2014 | 382.42793423953 | 0.536640130947104 | 158.507554782224 | 223.920379457306 | Marshall Islands |
| 2015 | 365.40008888653 | 0.541793166838195 | 154.980165789071 | 210.419923097459 | Marshall Islands |
| 2016 | 377.49024537083 | 0.546782936000428 | 149.1290554 | 228.36118995473 | Marshall Islands |
| 2017 | 353.88880153712 | 0.55206515210282 | 146.525426168578 | 207.363375368542 | Marshall Islands |
| 2018 | 343.75496511944 | 0.557599278468739 | 141.328608180318 | 202.426356939122 | Marshall Islands |
| 2019 | 324.27774765369 | 0.563428749789962 | 138.332610877844 | 185.945136775846 | Marshall Islands |
| 2020 | 325.13414747981 | 0.568837509186186 | 110.278739568772 | 214.855407911038 | Marshall Islands |
| 2021 | 284.67736105317 | 0.574091128177309 | 90.5275711283267 | 194.149789924843 | Marshall Islands |
| 1990 | 1501.56015247455 | 0.462511616715497 | 380.945676415222 | 1120.61447605933 | Micronesia (Federated States of) |
| 1991 | 1274.35175174951 | 0.468144831239529 | 360.605489045858 | 913.746262703652 | Micronesia (Federated States of) |
| 1992 | 1205.65657390075 | 0.473792936437038 | 333.990616193104 | 871.665957707645 | Micronesia (Federated States of) |
| 1993 | 1132.57424423692 | 0.479939595446532 | 325.004645560374 | 807.569598676546 | Micronesia (Federated States of) |
| 1994 | 1070.15923811421 | 0.485300005800881 | 325.706479533714 | 744.452758580496 | Micronesia (Federated States of) |
| 1995 | 993.27811015793 | 0.491015185412641 | 299.063763248456 | 694.214346909474 | Micronesia (Federated States of) |
| 1996 | 940.2562468 | 0.495570308271861 | 283.246770724619 | 657.009476095381 | Micronesia (Federated States of) |
| 1997 | 859.95991514704 | 0.499110105936474 | 238.380170238917 | 621.579744908123 | Micronesia (Federated States of) |
| 1998 | 806.9275943 | 0.5028615644834 | 222.258471531291 | 584.669122750909 | Micronesia (Federated States of) |
| 1999 | 754.99543343393 | 0.506663430751627 | 218.226018602988 | 536.769414830942 | Micronesia (Federated States of) |
| 2000 | 4298.53507526653 | 0.511004227582949 | 213.394182594785 | 4085.14089267175 | Micronesia (Federated States of) |
| 2001 | 650.31680379207 | 0.515316814346477 | 206.269440933358 | 444.047362858712 | Micronesia (Federated States of) |
| 2002 | 605.8759285 | 0.519545671945836 | 198.326469410552 | 407.549459062448 | Micronesia (Federated States of) |
| 2003 | 566.02252732153 | 0.523829598854531 | 190.579590706393 | 375.442936615137 | Micronesia (Federated States of) |
| 2004 | 548.48994691117 | 0.527610964634803 | 180.386174604814 | 368.103772306356 | Micronesia (Federated States of) |
| 2005 | 542.82822150769 | 0.531703321036516 | 171.096897641883 | 371.731323865807 | Micronesia (Federated States of) |
| 2006 | 505.17451362832 | 0.535718681873052 | 163.37319264011 | 341.80132098821 | Micronesia (Federated States of) |
| 2007 | 455.93085937026 | 0.53943146430145 | 156.587557065742 | 299.343302304518 | Micronesia (Federated States of) |
| 2008 | 382.61744627043 | 0.542766353836032 | 155.119277602118 | 227.498168668312 | Micronesia (Federated States of) |
| 2009 | 344.90005751715 | 0.546275496220168 | 149.455854104586 | 195.444203412564 | Micronesia (Federated States of) |
| 2010 | 318.97086140292 | 0.550122546321787 | 146.04822450565 | 172.92263689727 | Micronesia (Federated States of) |
| 2011 | 332.38693314608 | 0.554240410692063 | 144.140758253481 | 188.246174892599 | Micronesia (Federated States of) |
| 2012 | 329.65612514831 | 0.557965852891456 | 142.338164860171 | 187.31796028814 | Micronesia (Federated States of) |
| 2013 | 326.32146418257 | 0.561082820885874 | 139.267448427921 | 187.054015754649 | Micronesia (Federated States of) |
| 2014 | 323.68055901036 | 0.563840029745074 | 137.640963101288 | 186.039595909072 | Micronesia (Federated States of) |
| 2015 | 320.3435616 | 0.567151341569495 | 108.024765585596 | 212.318796042804 | Micronesia (Federated States of) |
| 2016 | 314.17178075415 | 0.570458101091096 | 92.605061762221 | 221.566718991929 | Micronesia (Federated States of) |
| 2017 | 296.19096736323 | 0.574039832793086 | 85.7479415706228 | 210.443025792607 | Micronesia (Federated States of) |
| 2018 | 275.04044189694 | 0.577555080818811 | 85.8321682580982 | 189.208273638842 | Micronesia (Federated States of) |
| 2019 | 252.29140378406 | 0.581195254501754 | 85.2620933627277 | 167.029310421332 | Micronesia (Federated States of) |
| 2020 | 221.47876203263 | 0.584527228769055 | 80.3705011765577 | 141.108260856072 | Micronesia (Federated States of) |
| 2021 | 194.93257116235 | 0.587534967103163 | 78.2999853613683 | 116.632585800982 | Micronesia (Federated States of) |
| 1990 | 7179.95984388729 | 0.310668628637835 | 2125.67113799376 | 5054.28870589353 | Papua New Guinea |
| 1991 | 6951.64738851541 | 0.314967305387282 | 2109.02161445846 | 4842.62577405695 | Papua New Guinea |
| 1992 | 6674.17591171828 | 0.319530259271955 | 2106.42220810221 | 4567.75370361607 | Papua New Guinea |
| 1993 | 6320.695883 | 0.32500598378048 | 2073.13131488671 | 4247.56456861129 | Papua New Guinea |
| 1994 | 5927.6302855766 | 0.330664467960637 | 1996.8291442122 | 3930.8011413644 | Papua New Guinea |
| 1995 | 5571.27519701071 | 0.335421285048649 | 1981.07373778608 | 3590.20145922463 | Papua New Guinea |
| 1996 | 5367.34252768672 | 0.340348698133771 | 1862.55584962723 | 3504.7866780595 | Papua New Guinea |
| 1997 | 5188.50709411834 | 0.344258324561358 | 1771.78990924741 | 3416.71718487093 | Papua New Guinea |
| 1998 | 5336.50253937191 | 0.347801407739934 | 1767.24697692114 | 3569.25556245077 | Papua New Guinea |
| 1999 | 5325.53224535927 | 0.351250620970096 | 1772.5968766118 | 3552.93536874747 | Papua New Guinea |
| 2000 | 5278.29226317302 | 0.354135790847746 | 1758.5748590314 | 3519.71740414162 | Papua New Guinea |
| 2001 | 5098.54305071425 | 0.356588986333728 | 1752.72153047692 | 3345.82152023733 | Papua New Guinea |
| 2002 | 4953.02145246381 | 0.358572086402703 | 1644.13177001311 | 3308.8896824507 | Papua New Guinea |
| 2003 | 4695.43150009121 | 0.360736816415114 | 1557.83808406068 | 3137.59341603053 | Papua New Guinea |
| 2004 | 4624.17338167234 | 0.36282754151245 | 1446.0515896174 | 3178.12179205494 | Papua New Guinea |
| 2005 | 4744.64229697983 | 0.365294964904992 | 1308.40839862009 | 3436.23389835974 | Papua New Guinea |
| 2006 | 4785.75835292426 | 0.367826759122837 | 1224.6214085896 | 3561.13694433466 | Papua New Guinea |
| 2007 | 4796.03750351914 | 0.370794146921454 | 1221.14767044985 | 3574.88983306929 | Papua New Guinea |
| 2008 | 4741.46795632193 | 0.37342620922524 | 1231.97614203106 | 3509.49181429087 | Papua New Guinea |
| 2009 | 4937.62407882399 | 0.376528284761462 | 1164.08669412679 | 3773.5373846972 | Papua New Guinea |
| 2010 | 4881.0306915805 | 0.380286777637627 | 1132.54661086704 | 3748.48408071346 | Papua New Guinea |
| 2011 | 4562.9968861366 | 0.383587539302927 | 1093.51428463266 | 3469.48260150394 | Papua New Guinea |
| 2012 | 4470.13565797229 | 0.386781027656579 | 1088.84185461059 | 3381.2938033617 | Papua New Guinea |
| 2013 | 4341.41315610465 | 0.389926437008588 | 1038.21680821416 | 3303.19634789049 | Papua New Guinea |
| 2014 | 4187.97266398007 | 0.394004571266826 | 976.067988830969 | 3211.9046751491 | Papua New Guinea |
| 2015 | 3979.57144481654 | 0.398285863731033 | 924.014867959932 | 3055.55657685661 | Papua New Guinea |
| 2016 | 3894.9079182571 | 0.402407266844816 | 874.745741184336 | 3020.16217707276 | Papua New Guinea |
| 2017 | 3774.10583439302 | 0.406153435448224 | 887.092981124303 | 2887.01285326872 | Papua New Guinea |
| 2018 | 3592.52169253544 | 0.409165082697567 | 866.791613941696 | 2725.73007859374 | Papua New Guinea |
| 2019 | 3448.20901446264 | 0.412311146526574 | 851.327413770137 | 2596.8816006925 | Papua New Guinea |
| 2020 | 3373.4640237044 | 0.415186878578667 | 822.920860544956 | 2550.54316315944 | Papua New Guinea |
| 2021 | 3216.91497975739 | 0.417797443084353 | 801.41545353602 | 2415.49952622137 | Papua New Guinea |
| 1990 | 302.06204371261 | 0.48749142771166 | 302.06204371261 | 0 | Samoa |
| 1991 | 286.73529357941 | 0.490987145586308 | 286.73529357941 | 0 | Samoa |
| 1992 | 271.24529432644 | 0.494027447933208 | 271.24529432644 | 0 | Samoa |
| 1993 | 257.35972718246 | 0.497079263536296 | 242.631515161403 | 14.7282120210567 | Samoa |
| 1994 | 244.15186499103 | 0.499484836329879 | 234.485135798501 | 9.66672919252943 | Samoa |
| 1995 | 231.87715866139 | 0.502369734936465 | 226.716394071992 | 5.1607645893985 | Samoa |
| 1996 | 221.25009669199 | 0.505778026475631 | 219.93090262177 | 1.31919407022045 | Samoa |
| 1997 | 211.48397519929 | 0.509096382265271 | 211.48397519929 | 0 | Samoa |
| 1998 | 199.05965272073 | 0.512367565788016 | 199.05965272073 | 0 | Samoa |
| 1999 | 204.04690162361 | 0.515409049865666 | 202.192668057155 | 1.85423356645538 | Samoa |
| 2000 | 193.46636567273 | 0.518902326533937 | 193.46636567273 | 0 | Samoa |
| 2001 | 184.06124662184 | 0.522971625527944 | 184.06124662184 | 0 | Samoa |
| 2002 | 172.23460350812 | 0.527242669970423 | 172.23460350812 | 0 | Samoa |
| 2003 | 163.24688116782 | 0.531603736377418 | 163.24688116782 | 0 | Samoa |
| 2004 | 157.57397992959 | 0.53600327592574 | 157.15812830105 | 0.415851628539997 | Samoa |
| 2005 | 154.29716398428 | 0.540557907991258 | 154.29716398428 | 0 | Samoa |
| 2006 | 148.82948836121 | 0.544812068358444 | 148.82948836121 | 0 | Samoa |
| 2007 | 144.11698789358 | 0.54911075247208 | 144.11698789358 | 0 | Samoa |
| 2008 | 140.53602416308 | 0.553164595646872 | 140.53602416308 | 0 | Samoa |
| 2009 | 140.19197001741 | 0.556474015274728 | 140.19197001741 | 0 | Samoa |
| 2010 | 142.82474223667 | 0.559622905103398 | 139.016236860867 | 3.80850537580255 | Samoa |
| 2011 | 142.58709371148 | 0.562891574259145 | 138.588476974264 | 3.99861673721588 | Samoa |
| 2012 | 140.7275387 | 0.565320388101849 | 104.734916687723 | 35.9926219729774 | Samoa |
| 2013 | 136.52587603938 | 0.56744853592103 | 109.036430272708 | 27.4894457666723 | Samoa |
| 2014 | 132.71813952286 | 0.569493660728475 | 106.973463092526 | 25.7446764303341 | Samoa |
| 2015 | 128.31977024227 | 0.571998982964235 | 84.534450150029 | 43.785320092241 | Samoa |
| 2016 | 126.04753336367 | 0.575402051396993 | 83.4835000525925 | 42.5640333110775 | Samoa |
| 2017 | 125.25114941465 | 0.578925435094317 | 83.0995789962612 | 42.1515704183888 | Samoa |
| 2018 | 126.85893656501 | 0.582320660518804 | 85.7584918622569 | 41.1004447027531 | Samoa |
| 2019 | 124.19529604043 | 0.586256695142635 | 82.6843054732483 | 41.5109905671817 | Samoa |
| 2020 | 125.45592756147 | 0.590075295612806 | 79.0577939535818 | 46.3981336078882 | Samoa |
| 2021 | 90.41258657 | 0.593392769469481 | 76.8195771720751 | 13.5930093982749 | Samoa |
| 1990 | 2711.16165980046 | 0.301217167448465 | 2123.11779888365 | 588.043860916808 | Solomon Islands |
| 1991 | 2600.4258229497 | 0.305830667512655 | 2101.47071284572 | 498.955110103976 | Solomon Islands |
| 1992 | 2445.29373320586 | 0.311431696691098 | 2098.68390850869 | 346.609824697168 | Solomon Islands |
| 1993 | 2297.83577285969 | 0.31715109185957 | 2109.90589174664 | 187.929881113055 | Solomon Islands |
| 1994 | 2149.06187800485 | 0.323300246722871 | 2043.07682555653 | 105.985052448325 | Solomon Islands |
| 1995 | 1985.72210800351 | 0.329968564261603 | 1976.39753279104 | 9.32457521246852 | Solomon Islands |
| 1996 | 1856.44246518952 | 0.336208053088528 | 1856.44246518952 | 0 | Solomon Islands |
| 1997 | 1737.16375575314 | 0.341835626898821 | 1737.16375575314 | 0 | Solomon Islands |
| 1998 | 1851.74236428104 | 0.347135060018002 | 1760.84103046804 | 90.9013338129971 | Solomon Islands |
| 1999 | 1827.98381132643 | 0.351867191410364 | 1762.49601939125 | 65.4877919351848 | Solomon Islands |
| 2000 | 1762.107217 | 0.354799143417026 | 1742.61117832495 | 19.4960386780515 | Solomon Islands |
| 2001 | 1682.4240046981 | 0.356626745168074 | 1682.4240046981 | 0 | Solomon Islands |
| 2002 | 1600.87274712252 | 0.357910115982585 | 1600.87274712252 | 0 | Solomon Islands |
| 2003 | 1448.76587880621 | 0.359424358912125 | 1448.76587880621 | 0 | Solomon Islands |
| 2004 | 1287.06959255581 | 0.361290558162268 | 1287.06959255581 | 0 | Solomon Islands |
| 2005 | 1238.18420311885 | 0.363580018964866 | 1238.18420311885 | 0 | Solomon Islands |
| 2006 | 1184.01605333901 | 0.366443697706899 | 1184.01605333901 | 0 | Solomon Islands |
| 2007 | 1208.71267242789 | 0.369827951415349 | 1189.33051567459 | 19.3821567532982 | Solomon Islands |
| 2008 | 1151.15824760468 | 0.3737648835675 | 1151.15824760468 | 0 | Solomon Islands |
| 2009 | 1094.44200530683 | 0.377821027515966 | 1094.44200530683 | 0 | Solomon Islands |
| 2010 | 1066.14023261013 | 0.382717589747291 | 1066.14023261013 | 0 | Solomon Islands |
| 2011 | 997.62841374402 | 0.388055336574909 | 997.62841374402 | 0 | Solomon Islands |
| 2012 | 912.9026833 | 0.393004639707306 | 912.9026833 | 0 | Solomon Islands |
| 2013 | 894.0774659 | 0.397906249572343 | 894.0774659 | 0 | Solomon Islands |
| 2014 | 846.55069328163 | 0.402244744701609 | 846.55069328163 | 0 | Solomon Islands |
| 2015 | 881.29879490053 | 0.406233896996201 | 861.376549972361 | 19.9222449281693 | Solomon Islands |
| 2016 | 841.47705007769 | 0.410406962023453 | 841.47705007769 | 0 | Solomon Islands |
| 2017 | 801.4794952 | 0.414676394150034 | 801.4794952 | 0 | Solomon Islands |
| 2018 | 755.75371464075 | 0.418889220879761 | 755.75371464075 | 0 | Solomon Islands |
| 2019 | 708.43858260135 | 0.422736386173248 | 678.612094211681 | 29.8264883896687 | Solomon Islands |
| 2020 | 439.97367243573 | 0.426112428669488 | 439.97367243573 | 0 | Solomon Islands |
| 2021 | 397.08099597904 | 0.429360316406646 | 397.08099597904 | 0 | Solomon Islands |
| 1990 | 365.58775739817 | 0.491806839500397 | 299.010626596127 | 66.577130802043 | Tonga |
| 1991 | 347.69770132828 | 0.498166205111113 | 237.709255152757 | 109.988446175523 | Tonga |
| 1992 | 329.62687069517 | 0.503659848303175 | 225.378365195625 | 104.248505499545 | Tonga |
| 1993 | 317.02529687596 | 0.508904449646887 | 219.169785764116 | 97.8555111118443 | Tonga |
| 1994 | 303.03894348152 | 0.514207834722848 | 207.437470276982 | 95.6014732045376 | Tonga |
| 1995 | 289.08139271326 | 0.520215020279115 | 198.74329781541 | 90.3380949 | Tonga |
| 1996 | 277.22211434247 | 0.525808135436488 | 192.118089801377 | 85.1040245410931 | Tonga |
| 1997 | 259.87636614394 | 0.531009091678332 | 179.213043206749 | 80.6633229371911 | Tonga |
| 1998 | 249.75902420715 | 0.536172313703023 | 157.545607793711 | 92.213416413439 | Tonga |
| 1999 | 261.91933523455 | 0.541317019874755 | 155.67548661805 | 106.2438486 | Tonga |
| 2000 | 251.73081452363 | 0.546303195085387 | 148.965320278134 | 102.765494245496 | Tonga |
| 2001 | 240.00434682809 | 0.551054251063729 | 145.696821582767 | 94.3075252453229 | Tonga |
| 2002 | 227.02159802394 | 0.556008345246101 | 144.038827812164 | 82.982770211776 | Tonga |
| 2003 | 215.14845631288 | 0.560736066200254 | 139.663305451591 | 75.4851508612894 | Tonga |
| 2004 | 200.31025827871 | 0.564806378895597 | 113.881113972681 | 86.4291443060285 | Tonga |
| 2005 | 198.74427296593 | 0.568449169182724 | 109.764735507459 | 88.979537458471 | Tonga |
| 2006 | 199.90879485631 | 0.57161986831651 | 83.035339981282 | 116.873454875028 | Tonga |
| 2007 | 196.64647769472 | 0.574351510103056 | 85.0010177155315 | 111.645459979189 | Tonga |
| 2008 | 190.32128324308 | 0.57730541521781 | 85.1278928686956 | 105.193390374384 | Tonga |
| 2009 | 189.68980085594 | 0.579170749598751 | 81.5896949421002 | 108.10010591384 | Tonga |
| 2010 | 189.43469328527 | 0.580657298614066 | 85.1221454067823 | 104.312547878488 | Tonga |
| 2011 | 186.03520090356 | 0.58265672287008 | 84.3056483559479 | 101.729552547612 | Tonga |
| 2012 | 181.66381435083 | 0.584770880221314 | 82.7421263280528 | 98.9216880227772 | Tonga |
| 2013 | 178.03136034255 | 0.587351141040311 | 80.91263769 | 97.11872266 | Tonga |
| 2014 | 173.51527255017 | 0.590826852746506 | 80.1885867497781 | 93.3266858003919 | Tonga |
| 2015 | 170.5044778 | 0.594965747674029 | 78.5680781969887 | 91.9363995956113 | Tonga |
| 2016 | 167.24732191078 | 0.60034366853075 | 75.7673270278257 | 91.4799948829543 | Tonga |
| 2017 | 159.36026740877 | 0.606243591849879 | 74.1835787909724 | 85.1766886177976 | Tonga |
| 2018 | 153.07472318419 | 0.611953941537156 | 69.5412340512425 | 83.5334891329475 | Tonga |
| 2019 | 146.24570413272 | 0.617324063407956 | 63.5539817637537 | 82.6917223689663 | Tonga |
| 2020 | 145.42924601763 | 0.622104455604093 | 62.3320494468525 | 83.0971965707775 | Tonga |
| 2021 | 67.35213981 | 0.626349935807987 | 58.9121686273735 | 8.43997118263654 | Tonga |
| 1990 | 2202.18379880763 | 0.353100252005321 | 1759.3594575494 | 442.824341258234 | Vanuatu |
| 1991 | 2106.20808492858 | 0.357005378847917 | 1711.89470398148 | 394.313380947097 | Vanuatu |
| 1992 | 1994.10663427033 | 0.360893295561675 | 1549.06501690627 | 445.041617364059 | Vanuatu |
| 1993 | 1914.42202751005 | 0.364444155251801 | 1296.26461951554 | 618.157407994508 | Vanuatu |
| 1994 | 1825.13619749362 | 0.368501002014456 | 1252.44880037832 | 572.687397115301 | Vanuatu |
| 1995 | 1746.35055462506 | 0.372479125756324 | 1225.40592807387 | 520.94462655119 | Vanuatu |
| 1996 | 1684.52360312491 | 0.376460884544884 | 1169.07975242399 | 515.443850700924 | Vanuatu |
| 1997 | 1608.71129700811 | 0.380667171234513 | 1123.28713010907 | 485.424166899038 | Vanuatu |
| 1998 | 1537.79291066084 | 0.384690196444633 | 1090.86491380341 | 446.927996857429 | Vanuatu |
| 1999 | 1689.19562253991 | 0.388300526237242 | 1043.30772495054 | 645.887897589368 | Vanuatu |
| 2000 | 1654.79320388296 | 0.392158348897928 | 1027.87447131876 | 626.918732564199 | Vanuatu |
| 2001 | 1598.07234025229 | 0.395325055353778 | 966.804481931655 | 631.267858320635 | Vanuatu |
| 2002 | 1518.86112751606 | 0.397723782197823 | 991.684766544078 | 527.176360971982 | Vanuatu |
| 2003 | 1420.79344555297 | 0.400396455507663 | 941.996800175328 | 478.796645377642 | Vanuatu |
| 2004 | 1258.81764325955 | 0.403226096857709 | 889.947973970182 | 368.869669289368 | Vanuatu |
| 2005 | 1205.11388067743 | 0.406488572045146 | 874.286972890391 | 330.826907787039 | Vanuatu |
| 2006 | 1131.76132691379 | 0.410501728664985 | 854.62617581488 | 277.13515109891 | Vanuatu |
| 2007 | 1182.85664415677 | 0.41491557794022 | 825.102535060946 | 357.754109095824 | Vanuatu |
| 2008 | 1138.51681020774 | 0.41985323747475 | 779.959175743097 | 358.557634464643 | Vanuatu |
| 2009 | 1077.9953230614 | 0.424854650160333 | 682.893544 | 395.1017791 | Vanuatu |
| 2010 | 1023.65205548295 | 0.429658048936263 | 430.164448107428 | 593.487607375522 | Vanuatu |
| 2011 | 958.1860925 | 0.434283044387506 | 425.889853394831 | 532.296239072869 | Vanuatu |
| 2012 | 852.68330992702 | 0.438661997905717 | 422.117127804617 | 430.566182122403 | Vanuatu |
| 2013 | 792.85817083631 | 0.442720176072726 | 420.411316900008 | 372.446853936302 | Vanuatu |
| 2014 | 781.87291876143 | 0.446687298111776 | 410.402048153798 | 371.470870607632 | Vanuatu |
| 2015 | 758.49719690759 | 0.450329861394857 | 414.473610010262 | 344.023586897328 | Vanuatu |
| 2016 | 821.34870298683 | 0.454072763242304 | 406.52098753578 | 414.82771545105 | Vanuatu |
| 2017 | 805.6859374 | 0.45801683397471 | 397.549497993899 | 408.136439412501 | Vanuatu |
| 2018 | 787.2693143 | 0.462080220969235 | 377.742735937945 | 409.526578344955 | Vanuatu |
| 2019 | 747.0916231 | 0.466276161662358 | 358.262525135547 | 388.829097953853 | Vanuatu |
| 2020 | 710.91481269029 | 0.470038390856612 | 343.442381180257 | 367.472431510033 | Vanuatu |
| 2021 | 624.06595922377 | 0.473100705820327 | 337.097756053513 | 286.968203170257 | Vanuatu |
| 1990 | 3961.75953598483 | 0.54441453483767 | 155.538758826634 | 3806.2207771582 | Armenia |
| 1991 | 3888.11986146191 | 0.547561862706728 | 149.449347663332 | 3738.67051379858 | Armenia |
| 1992 | 4055.58789546133 | 0.548974723873954 | 149.854301780015 | 3905.73359368132 | Armenia |
| 1993 | 3820.32677182875 | 0.551214673543035 | 146.186901086696 | 3674.13987074205 | Armenia |
| 1994 | 3685.09997309603 | 0.553783844340157 | 143.364360918704 | 3541.73561217733 | Armenia |
| 1995 | 3342.63756706401 | 0.556815513215437 | 142.206846122866 | 3200.43072094114 | Armenia |
| 1996 | 2890.84556042256 | 0.560537777557715 | 138.578730222096 | 2752.26683020046 | Armenia |
| 1997 | 2652.44611353814 | 0.564627461798536 | 113.784897163369 | 2538.66121637477 | Armenia |
| 1998 | 2255.28415173196 | 0.569556662578666 | 107.154712627594 | 2148.12943910437 | Armenia |
| 1999 | 1923.70846406542 | 0.574323064436665 | 85.6935536977694 | 1838.01491036765 | Armenia |
| 2000 | 1652.63484462401 | 0.579437569012042 | 82.5027097652659 | 1570.13213485874 | Armenia |
| 2001 | 1476.38913472051 | 0.585419295040696 | 83.0146866524412 | 1393.37444806807 | Armenia |
| 2002 | 1209.66814219968 | 0.592710614375924 | 78.7938088049425 | 1130.87433339474 | Armenia |
| 2003 | 1053.19205769013 | 0.600809170405726 | 76.169810050569 | 977.022247639561 | Armenia |
| 2004 | 973.87346611249 | 0.60903176604033 | 70.1372975043591 | 903.736168608131 | Armenia |
| 2005 | 882.71152611028 | 0.617759634102041 | 63.2581398788334 | 819.453386231447 | Armenia |
| 2006 | 794.50905562715 | 0.626688335239636 | 59.2972767657904 | 735.21177886136 | Armenia |
| 2007 | 576.98864628504 | 0.635870981516541 | 52.4576925571644 | 524.530953727876 | Armenia |
| 2008 | 475.4550011 | 0.64443159113194 | 50.0737821386577 | 425.381218942742 | Armenia |
| 2009 | 367.06061180657 | 0.649714844735141 | 47.4891479659042 | 319.571463840666 | Armenia |
| 2010 | 271.1229839 | 0.654811533750253 | 45.5943959981881 | 225.528587882412 | Armenia |
| 2011 | 224.26667787061 | 0.659818092317619 | 42.8111048857823 | 181.455572984828 | Armenia |
| 2012 | 126.55155053255 | 0.664833142570704 | 40.5777517122967 | 85.9737988202533 | Armenia |
| 2013 | 85.3323787 | 0.669534495666631 | 38.4201849623006 | 46.9121937392894 | Armenia |
| 2014 | 63.00638126 | 0.673932189530685 | 37.9082285635827 | 25.0981526922573 | Armenia |
| 2015 | 52.22110104 | 0.678054634729575 | 37.7139335820073 | 14.5071674610127 | Armenia |
| 2016 | 59.85686874 | 0.681746395416543 | 37.4913530910417 | 22.3655156481483 | Armenia |
| 2017 | 48.59579412 | 0.685980265385199 | 37.1215379067625 | 11.4742562172275 | Armenia |
| 2018 | 49.4417713 | 0.690276871944721 | 36.8942375264739 | 12.5475337706861 | Armenia |
| 2019 | 48.78338276 | 0.694765961567774 | 36.9783306968759 | 11.8050520621341 | Armenia |
| 2020 | 55.4640865 | 0.69826889937921 | 35.79106208 | 19.67302442 | Armenia |
| 2021 | 54.68433635 | 0.701833194147139 | 35.3949174762082 | 19.2894188703418 | Armenia |
| 1990 | 3868.20647501814 | 0.595986032704332 | 77.2274607652086 | 3790.97901425293 | Azerbaijan |
| 1991 | 3667.26134583129 | 0.595807711845649 | 77.5830243432124 | 3589.67832148808 | Azerbaijan |
| 1992 | 3494.41166115145 | 0.594651335166956 | 78.9139234743404 | 3415.49773767711 | Azerbaijan |
| 1993 | 3350.38583448182 | 0.59354267649819 | 77.3191489683771 | 3273.06668551344 | Azerbaijan |
| 1994 | 3200.61021503805 | 0.5920887850585 | 79.8189221725221 | 3120.79129286553 | Azerbaijan |
| 1995 | 3041.76121384986 | 0.589441456728463 | 82.4123769522563 | 2959.3488368976 | Azerbaijan |
| 1996 | 2894.91168313647 | 0.586315946466504 | 79.9133094948244 | 2814.99837364165 | Azerbaijan |
| 1997 | 2741.52140780482 | 0.583221812602983 | 82.4146404993901 | 2659.10676730543 | Azerbaijan |
| 1998 | 2606.20832691525 | 0.580392893004131 | 82.7557948384521 | 2523.4525320768 | Azerbaijan |
| 1999 | 2464.00520262975 | 0.579192506195412 | 83.4265802658836 | 2380.57862236387 | Azerbaijan |
| 2000 | 2275.78898854967 | 0.580020573148448 | 83.5283683846467 | 2192.26062016502 | Azerbaijan |
| 2001 | 2019.34708360616 | 0.582193708342128 | 82.2004781486618 | 1937.1466054575 | Azerbaijan |
| 2002 | 1826.79096705253 | 0.585716143276774 | 79.0454810738124 | 1747.74548597872 | Azerbaijan |
| 2003 | 1686.75914048759 | 0.590038705393348 | 82.4834979897934 | 1604.2756424978 | Azerbaijan |
| 2004 | 1566.29328314918 | 0.594646084750935 | 74.8602442235755 | 1491.4330389256 | Azerbaijan |
| 2005 | 1430.75860021438 | 0.602485193033144 | 76.6329484740183 | 1354.12565174036 | Azerbaijan |
| 2006 | 1293.12816495698 | 0.61377109654975 | 69.1531927985278 | 1223.97497215845 | Azerbaijan |
| 2007 | 1161.19620595052 | 0.626110925531736 | 59.5132521524136 | 1101.68295379811 | Azerbaijan |
| 2008 | 1063.26719669009 | 0.636901109120982 | 49.3583764995863 | 1013.9088201905 | Azerbaijan |
| 2009 | 980.70875453419 | 0.645163340636595 | 49.8848990793307 | 930.823855454859 | Azerbaijan |
| 2010 | 919.68180922434 | 0.652056507002412 | 45.7325786084305 | 873.94923061591 | Azerbaijan |
| 2011 | 864.45472801761 | 0.657971629199692 | 45.0748792141942 | 819.379848803416 | Azerbaijan |
| 2012 | 814.20033120908 | 0.662897001762113 | 40.9287289723148 | 773.271602236765 | Azerbaijan |
| 2013 | 768.21424075236 | 0.667658779253801 | 39.4727973477798 | 728.74144340458 | Azerbaijan |
| 2014 | 722.62381111008 | 0.672162741185153 | 38.0601358897496 | 684.56367522033 | Azerbaijan |
| 2015 | 688.65998730957 | 0.676244911134277 | 38.1072335716844 | 650.552753737886 | Azerbaijan |
| 2016 | 664.74290778523 | 0.679807642651878 | 37.831100361805 | 626.911807423425 | Azerbaijan |
| 2017 | 646.17224181025 | 0.683263203610851 | 37.6460422813788 | 608.526199528871 | Azerbaijan |
| 2018 | 628.38738858548 | 0.68656109318329 | 37.3205692793552 | 591.066819306125 | Azerbaijan |
| 2019 | 612.45240054889 | 0.689834714583805 | 37.333664732901 | 575.118735815989 | Azerbaijan |
| 2020 | 554.98810309604 | 0.692605192199032 | 36.1729936450166 | 518.815109451023 | Azerbaijan |
| 2021 | 545.05541620329 | 0.694851274415291 | 36.6702306249319 | 508.385185578358 | Azerbaijan |
| 1990 | 1240.37151759102 | 0.656136044291497 | 45.6649728744879 | 1194.70654471653 | Georgia |
| 1991 | 1045.41136610128 | 0.662113185865682 | 42.7636649821577 | 1002.64770111912 | Georgia |
| 1992 | 1041.75496167916 | 0.665021427814483 | 39.8576381293731 | 1001.89732354979 | Georgia |
| 1993 | 1214.60656074385 | 0.663466862184598 | 40.6478004403334 | 1173.95876030352 | Georgia |
| 1994 | 1402.01317781996 | 0.658134139130798 | 44.860791199028 | 1357.15238662093 | Georgia |
| 1995 | 1282.642452 | 0.651363604039082 | 47.2110470119568 | 1235.43140501904 | Georgia |
| 1996 | 1111.65154002029 | 0.644558090070583 | 49.0412600806756 | 1062.61027993961 | Georgia |
| 1997 | 1084.02411909423 | 0.63852563128688 | 51.185655879019 | 1032.83846321521 | Georgia |
| 1998 | 884.9101591 | 0.633347931127712 | 53.0934412128619 | 831.816717925738 | Georgia |
| 1999 | 703.57040492735 | 0.63027794625322 | 55.2751627712367 | 648.295242156113 | Georgia |
| 2000 | 502.91861688616 | 0.630265990578475 | 55.399798779772 | 447.518818106388 | Georgia |
| 2001 | 450.56896447177 | 0.633388300444106 | 52.6109953458834 | 397.957969125887 | Georgia |
| 2002 | 397.30814704394 | 0.638370108205142 | 51.3722117788759 | 345.935935265064 | Georgia |
| 2003 | 334.8286854 | 0.644065578985868 | 51.4320832456978 | 283.396602170402 | Georgia |
| 2004 | 294.27556131989 | 0.648920978029192 | 47.36361534 | 246.911946 | Georgia |
| 2005 | 213.37618324919 | 0.65361600499668 | 45.6363493228422 | 167.739833926348 | Georgia |
| 2006 | 159.9642929 | 0.658403371769116 | 45.0683688838327 | 114.895924027267 | Georgia |
| 2007 | 152.66720600607 | 0.663697966220568 | 40.9913720708537 | 111.675833935216 | Georgia |
| 2008 | 144.4826609 | 0.668442495947816 | 38.9966793404091 | 105.485981542791 | Georgia |
| 2009 | 130.90280239706 | 0.672449380923053 | 38.1425579551032 | 92.7602444419568 | Georgia |
| 2010 | 118.24347368562 | 0.677034892187658 | 37.9133201135928 | 80.3301535720272 | Georgia |
| 2011 | 98.02239829 | 0.681567044251293 | 37.4940753916218 | 60.5283228983582 | Georgia |
| 2012 | 82.21966089 | 0.685519223189507 | 37.0584335146166 | 45.1612273771234 | Georgia |
| 2013 | 67.70112912 | 0.688603268140652 | 37.2871275755598 | 30.4140015455602 | Georgia |
| 2014 | 48.43130408 | 0.692155206871812 | 36.6102153744581 | 11.8210887069319 | Georgia |
| 2015 | 39.85640091 | 0.697316132597582 | 35.9459265594603 | 3.9104743504697 | Georgia |
| 2016 | 37.81237342 | 0.703731490142933 | 34.3306702259946 | 3.48170319230538 | Georgia |
| 2017 | 26.19845671 | 0.710874718456081 | 26.19845671 | 0 | Georgia |
| 2018 | 22.97851551 | 0.717993653891294 | 22.97851551 | 0 | Georgia |
| 2019 | 18.48846547 | 0.724536461948472 | 18.48846547 | 0 | Georgia |
| 2020 | 18.4168795 | 0.729125888954526 | 18.4168795 | 0 | Georgia |
| 2021 | 15.31549275 | 0.732473604476306 | 15.31549275 | 0 | Georgia |
| 1990 | 2654.67662852823 | 0.589435803839118 | 83.2485315930333 | 2571.4280969352 | Kazakhstan |
| 1991 | 2577.67058619397 | 0.590967831817803 | 78.0748805102455 | 2499.59570568372 | Kazakhstan |
| 1992 | 2883.64241220237 | 0.596127664006257 | 77.952169311403 | 2805.69024289097 | Kazakhstan |
| 1993 | 2882.09713287965 | 0.603138328452166 | 75.8087788439417 | 2806.28835403571 | Kazakhstan |
| 1994 | 2920.52470237078 | 0.610591100120018 | 69.0243336538272 | 2851.50036871695 | Kazakhstan |
| 1995 | 2824.60309597643 | 0.618573582182698 | 63.442380208613 | 2761.16071576782 | Kazakhstan |
| 1996 | 2559.31733208611 | 0.626013137564744 | 59.7866401976361 | 2499.53069188847 | Kazakhstan |
| 1997 | 2615.65881316569 | 0.631166626279517 | 51.6457976999303 | 2564.01301546576 | Kazakhstan |
| 1998 | 2006.31404929155 | 0.635388623908262 | 51.870707976403 | 1954.44334131515 | Kazakhstan |
| 1999 | 1688.72042550157 | 0.64018048502742 | 51.7083090238858 | 1637.01211647768 | Kazakhstan |
| 2000 | 1393.38313846682 | 0.644689623243828 | 48.6390926504624 | 1344.74404581636 | Kazakhstan |
| 2001 | 1105.33774822481 | 0.648397258264821 | 47.7314210510681 | 1057.60632717374 | Kazakhstan |
| 2002 | 842.4906247 | 0.651529335252827 | 47.6426682274572 | 794.847956449543 | Kazakhstan |
| 2003 | 662.76070124836 | 0.654835925835483 | 45.6976325604234 | 617.063068687936 | Kazakhstan |
| 2004 | 594.17494799154 | 0.658733071329599 | 45.0442724658138 | 549.130675525726 | Kazakhstan |
| 2005 | 495.5801202 | 0.662943941893493 | 41.0591700314937 | 454.520950215206 | Kazakhstan |
| 2006 | 492.18762720402 | 0.667048858291012 | 39.0919789533199 | 453.0956483 | Kazakhstan |
| 2007 | 459.72802471381 | 0.671367207568052 | 38.53018813 | 421.19783658278 | Kazakhstan |
| 2008 | 464.95493028047 | 0.676352868998898 | 37.8364895068544 | 427.118440773616 | Kazakhstan |
| 2009 | 354.44737069209 | 0.681606838672477 | 37.6038098762554 | 316.843560815835 | Kazakhstan |
| 2010 | 273.7047824 | 0.686996643005111 | 37.3861743422968 | 236.318608014003 | Kazakhstan |
| 2011 | 212.81810553659 | 0.691758951738966 | 36.7990090778617 | 176.019096458728 | Kazakhstan |
| 2012 | 147.81530629254 | 0.695312766608011 | 36.4482691397522 | 111.367037152788 | Kazakhstan |
| 2013 | 112.49502977684 | 0.699053593413088 | 35.679494407093 | 76.815535369747 | Kazakhstan |
| 2014 | 86.04301421 | 0.703511920757132 | 34.119541064426 | 51.923473150324 | Kazakhstan |
| 2015 | 88.9121198 | 0.70822973614897 | 34.5877395316204 | 54.3243802657296 | Kazakhstan |
| 2016 | 75.71459787 | 0.712569040397695 | 31.1438532159516 | 44.5707446557984 | Kazakhstan |
| 2017 | 62.28310453 | 0.716026687894304 | 31.9927670909897 | 30.2903374374303 | Kazakhstan |
| 2018 | 58.7389445 | 0.718531998551374 | 28.8857101360007 | 29.8532343641493 | Kazakhstan |
| 2019 | 58.31405549 | 0.720797601617411 | 28.4742255908914 | 29.8398298959686 | Kazakhstan |
| 2020 | 52.98125429 | 0.722788768207953 | 28.950203556602 | 24.031050736908 | Kazakhstan |
| 2021 | 49.94265805 | 0.725144495100349 | 26.351644763366 | 23.591013289794 | Kazakhstan |
| 1990 | 2902.22112558111 | 0.519407652158871 | 200.691727386344 | 2701.52939819477 | Kyrgyzstan |
| 1991 | 2812.85784454434 | 0.523746714213702 | 189.418349052323 | 2623.43949549202 | Kyrgyzstan |
| 1992 | 2886.14841435175 | 0.52790155215442 | 180.199261973889 | 2705.94915237786 | Kyrgyzstan |
| 1993 | 2636.45122623479 | 0.532431008334573 | 171.269997977795 | 2465.18122825699 | Kyrgyzstan |
| 1994 | 2620.3488563009 | 0.536232980141406 | 157.571803293659 | 2462.77705300724 | Kyrgyzstan |
| 1995 | 2532.95346949098 | 0.539052820319823 | 156.131467327284 | 2376.8220021637 | Kyrgyzstan |
| 1996 | 2346.09868976808 | 0.541628927165702 | 155.252108755667 | 2190.84658101241 | Kyrgyzstan |
| 1997 | 2672.96557524622 | 0.543341150499015 | 155.296493497621 | 2517.6690817486 | Kyrgyzstan |
| 1998 | 2668.44605566453 | 0.543258545983634 | 154.994768045718 | 2513.45128761881 | Kyrgyzstan |
| 1999 | 2553.64099626864 | 0.54286728228234 | 155.160551280898 | 2398.48044498774 | Kyrgyzstan |
| 2000 | 2145.07233554647 | 0.543036486604175 | 155.300077842235 | 1989.77225770424 | Kyrgyzstan |
| 2001 | 1877.49986404756 | 0.543805550685403 | 155.321066259411 | 1722.17879778815 | Kyrgyzstan |
| 2002 | 1566.82048502735 | 0.544374670344746 | 155.557192317617 | 1411.26329270973 | Kyrgyzstan |
| 2003 | 1241.593908 | 0.545649261714426 | 149.319352865298 | 1092.2745547077 | Kyrgyzstan |
| 2004 | 1110.86995438242 | 0.547262342515335 | 149.037263529908 | 961.832690852512 | Kyrgyzstan |
| 2005 | 1081.47216500604 | 0.548260448903511 | 148.614099146672 | 932.858065859368 | Kyrgyzstan |
| 2006 | 1094.54496236972 | 0.549383593833376 | 145.79748160086 | 948.74748076886 | Kyrgyzstan |
| 2007 | 1043.56125591064 | 0.551146191596044 | 146.527390190285 | 897.033865720355 | Kyrgyzstan |
| 2008 | 936.28732350693 | 0.553206382142723 | 144.008009111088 | 792.279314395842 | Kyrgyzstan |
| 2009 | 762.18669318613 | 0.55512386607295 | 144.098118872981 | 618.088574313149 | Kyrgyzstan |
| 2010 | 631.23521622838 | 0.556416544522946 | 144.017644996209 | 487.217571232171 | Kyrgyzstan |
| 2011 | 599.06289007997 | 0.55892333472436 | 142.259145041863 | 456.803745038107 | Kyrgyzstan |
| 2012 | 511.18357492067 | 0.561541573671693 | 139.139773227304 | 372.043801693366 | Kyrgyzstan |
| 2013 | 414.22636840244 | 0.565776769347093 | 109.341579743564 | 304.884788658876 | Kyrgyzstan |
| 2014 | 397.44292536443 | 0.570569357153487 | 90.9442357992566 | 306.498689565173 | Kyrgyzstan |
| 2015 | 379.64817781376 | 0.575463230818727 | 87.100110670863 | 292.548067142897 | Kyrgyzstan |
| 2016 | 269.52759756829 | 0.580388550869674 | 82.2659746661882 | 187.261622902102 | Kyrgyzstan |
| 2017 | 169.51868157416 | 0.585766095993466 | 84.3805417086241 | 85.138139865536 | Kyrgyzstan |
| 2018 | 117.54482772165 | 0.591237134219733 | 78.4255881120244 | 39.1192396096256 | Kyrgyzstan |
| 2019 | 99.69120829 | 0.596501638087194 | 76.9711766360634 | 22.7200316529366 | Kyrgyzstan |
| 2020 | 114.90556030435 | 0.600499553804348 | 75.061458624741 | 39.844101679609 | Kyrgyzstan |
| 2021 | 110.35522435481 | 0.603979328412055 | 75.7234782441866 | 34.6317461106235 | Kyrgyzstan |
| 1990 | 1872.77570367924 | 0.466550117857047 | 359.276833630151 | 1513.49887004909 | Mongolia |
| 1991 | 1728.06602138271 | 0.472199013666192 | 344.648861185332 | 1383.41716019738 | Mongolia |
| 1992 | 1608.03031843254 | 0.477586553799195 | 336.219350070176 | 1271.81096836236 | Mongolia |
| 1993 | 1512.66202862812 | 0.482726727049412 | 326.81020926034 | 1185.85181936778 | Mongolia |
| 1994 | 1421.96713331763 | 0.488276683033118 | 312.618117955477 | 1109.34901536215 | Mongolia |
| 1995 | 1325.05420222111 | 0.495006781085721 | 286.263444409829 | 1038.79075781128 | Mongolia |
| 1996 | 1315.37986133221 | 0.501703717883367 | 227.945987712435 | 1087.43387361978 | Mongolia |
| 1997 | 1173.80915617612 | 0.50855826800798 | 218.468880573328 | 955.340275602792 | Mongolia |
| 1998 | 1097.6993822117 | 0.514793382702856 | 207.957356537572 | 889.742025674128 | Mongolia |
| 1999 | 1030.8513382104 | 0.520781189245005 | 199.6990509 | 831.1522873 | Mongolia |
| 2000 | 972.62198812584 | 0.526650344907534 | 189.54378392846 | 783.07820419738 | Mongolia |
| 2001 | 862.01806004434 | 0.532620200843235 | 168.891668562108 | 693.126391482233 | Mongolia |
| 2002 | 786.27926076853 | 0.538424834911619 | 156.687789066545 | 629.591471701985 | Mongolia |
| 2003 | 731.84360938208 | 0.543971650708528 | 155.101676125753 | 576.741933256327 | Mongolia |
| 2004 | 683.2695709 | 0.549551499752672 | 146.022280452827 | 537.247290444473 | Mongolia |
| 2005 | 641.11134190161 | 0.55468028439734 | 144.249517498059 | 496.861824403551 | Mongolia |
| 2006 | 614.15697432211 | 0.55956113814646 | 139.596356986811 | 474.560617335299 | Mongolia |
| 2007 | 593.3847359 | 0.564236795141046 | 137.841040494767 | 455.543695409733 | Mongolia |
| 2008 | 569.9491431 | 0.568500598715617 | 106.053156707141 | 463.895986358859 | Mongolia |
| 2009 | 518.01318407437 | 0.571341685964544 | 83.9344275351496 | 434.07875653922 | Mongolia |
| 2010 | 464.01370848784 | 0.574406669398145 | 88.9068883908486 | 375.106820096991 | Mongolia |
| 2011 | 422.43582826302 | 0.57860066312097 | 81.9196501884061 | 340.516178074614 | Mongolia |
| 2012 | 397.86785516006 | 0.583255885768315 | 81.5984097797722 | 316.269445380288 | Mongolia |
| 2013 | 357.4546294 | 0.588080810956893 | 82.1308101766306 | 275.323819246469 | Mongolia |
| 2014 | 318.13753085521 | 0.592867352927119 | 80.5023654272116 | 237.635165427998 | Mongolia |
| 2015 | 260.75285706973 | 0.59685964756958 | 77.9588759830657 | 182.793981086664 | Mongolia |
| 2016 | 234.8580762 | 0.600260766781331 | 75.6913235082006 | 159.166752713999 | Mongolia |
| 2017 | 227.11545784234 | 0.60372196450642 | 75.6645704939573 | 151.450887348383 | Mongolia |
| 2018 | 211.72573947047 | 0.607501833007913 | 75.6867553664075 | 136.038984104063 | Mongolia |
| 2019 | 201.73424038106 | 0.611250680890372 | 69.8632653771753 | 131.870975003885 | Mongolia |
| 2020 | 170.21451054617 | 0.61462963730677 | 69.9050226623157 | 100.309487883854 | Mongolia |
| 2021 | 176.7881117 | 0.617621564638127 | 62.0571056438836 | 114.731006019116 | Mongolia |
| 1990 | 16246.4203485963 | 0.466155413419203 | 360.558288242308 | 15885.862060354 | Tajikistan |
| 1991 | 16142.7699675671 | 0.472342173046228 | 345.899597814011 | 15796.8703697531 | Tajikistan |
| 1992 | 16297.9551001991 | 0.47574519497737 | 338.626859914956 | 15959.3282402841 | Tajikistan |
| 1993 | 16472.4347877268 | 0.478238812995371 | 336.786313713682 | 16135.6484740131 | Tajikistan |
| 1994 | 16370.8492122448 | 0.478726011215724 | 322.550463250263 | 16048.2987489945 | Tajikistan |
| 1995 | 16306.5652529024 | 0.477586868115015 | 336.449561355994 | 15970.1156915464 | Tajikistan |
| 1996 | 16171.7409069864 | 0.474346027462684 | 337.760515768077 | 15833.9803912183 | Tajikistan |
| 1997 | 15858.2178044252 | 0.470274795927363 | 343.521800364391 | 15514.6960040608 | Tajikistan |
| 1998 | 15518.5328507711 | 0.465341742120163 | 360.198751092569 | 15158.3340996785 | Tajikistan |
| 1999 | 15066.4963101612 | 0.46050561615396 | 397.040150932111 | 14669.4561592291 | Tajikistan |
| 2000 | 14520.4896460074 | 0.457061333395443 | 398.349887568477 | 14122.1397584389 | Tajikistan |
| 2001 | 13703.0967037283 | 0.45625651276009 | 395.316395806731 | 13307.7803079216 | Tajikistan |
| 2002 | 12731.0817584024 | 0.458807780304158 | 396.803616000364 | 12334.278142402 | Tajikistan |
| 2003 | 11834.5529348643 | 0.463329706383786 | 379.3277907 | 11455.2251441192 | Tajikistan |
| 2004 | 10979.6257216483 | 0.469154214043517 | 344.260605201189 | 10635.3651164471 | Tajikistan |
| 2005 | 10180.6676294056 | 0.474637785688857 | 336.948656465089 | 9843.71897294051 | Tajikistan |
| 2006 | 9707.15530891224 | 0.479651831177988 | 326.592710616728 | 9380.56259829551 | Tajikistan |
| 2007 | 9170.12324300939 | 0.484211156232784 | 325.658251919596 | 8844.46499108979 | Tajikistan |
| 2008 | 8609.67026825947 | 0.488441135968382 | 309.634715346076 | 8300.03555291339 | Tajikistan |
| 2009 | 8107.48968225364 | 0.49206404051479 | 293.136923318622 | 7814.35275893502 | Tajikistan |
| 2010 | 7526.0771018705 | 0.495893963626257 | 283.275239360296 | 7242.8018625102 | Tajikistan |
| 2011 | 6821.8982015473 | 0.499869804401369 | 229.217630951642 | 6592.68057059566 | Tajikistan |
| 2012 | 5919.85417576938 | 0.503953884549027 | 223.111314351371 | 5696.74286141801 | Tajikistan |
| 2013 | 5036.41049567311 | 0.507970675899606 | 218.643410453562 | 4817.76708521955 | Tajikistan |
| 2014 | 4306.66420301822 | 0.511989517777097 | 212.947624543374 | 4093.71657847485 | Tajikistan |
| 2015 | 3477.50007680388 | 0.515783244686634 | 204.089162031649 | 3273.41091477223 | Tajikistan |
| 2016 | 3286.27790286706 | 0.519897002395847 | 199.366805279951 | 3086.91109758711 | Tajikistan |
| 2017 | 3106.39036293298 | 0.524492606909067 | 190.106578428426 | 2916.28378450455 | Tajikistan |
| 2018 | 2987.36418435575 | 0.529153916295379 | 179.014926336154 | 2808.3492580196 | Tajikistan |
| 2019 | 2968.00990668263 | 0.533769951484441 | 169.382575279475 | 2798.62733140316 | Tajikistan |
| 2020 | 2974.32208665419 | 0.537690530515565 | 157.67139092341 | 2816.65069573078 | Tajikistan |
| 2021 | 2685.06515182374 | 0.541511187051998 | 155.607643152309 | 2529.45750867143 | Tajikistan |
| 1990 | 7843.04170593266 | 0.563126887105135 | 139.534797090416 | 7703.50690884224 | Turkmenistan |
| 1991 | 7800.36324632062 | 0.564939385894165 | 111.202453610455 | 7689.16079271016 | Turkmenistan |
| 1992 | 7953.97340869012 | 0.565587505223265 | 111.360471320182 | 7842.61293736994 | Turkmenistan |
| 1993 | 8104.89816082482 | 0.567174449333284 | 106.429406065202 | 7998.46875475962 | Turkmenistan |
| 1994 | 7915.00401500992 | 0.567288975507041 | 108.835209405015 | 7806.1688056049 | Turkmenistan |
| 1995 | 7805.28972600867 | 0.567198634711689 | 108.269687576342 | 7697.02003843233 | Turkmenistan |
| 1996 | 7225.06167431035 | 0.567061261285221 | 106.545500024571 | 7118.51617428578 | Turkmenistan |
| 1997 | 6444.45788298337 | 0.565623083668948 | 111.97391435417 | 6332.4839686292 | Turkmenistan |
| 1998 | 5600.19634025431 | 0.564405264391893 | 137.453051495699 | 5462.74328875861 | Turkmenistan |
| 1999 | 4896.03801517744 | 0.564716005636632 | 108.752479974116 | 4787.28553520332 | Turkmenistan |
| 2000 | 4190.99875751747 | 0.567421033351252 | 110.419389487584 | 4080.57936802989 | Turkmenistan |
| 2001 | 3517.21507837261 | 0.57104381892434 | 86.1648437162352 | 3431.05023465637 | Turkmenistan |
| 2002 | 2843.08936112108 | 0.575596165867541 | 87.2993504515382 | 2755.79001066954 | Turkmenistan |
| 2003 | 2237.5616821092 | 0.580820125927832 | 84.7553323071025 | 2152.8063498021 | Turkmenistan |
| 2004 | 1806.67979169965 | 0.586712349142411 | 83.9576493662047 | 1722.72214233345 | Turkmenistan |
| 2005 | 1723.65160482951 | 0.593388894864117 | 81.7376191504289 | 1641.91398567908 | Turkmenistan |
| 2006 | 1508.89137809022 | 0.599896069365258 | 75.0273185164475 | 1433.86405957377 | Turkmenistan |
| 2007 | 1343.74999155751 | 0.606042198134731 | 76.9701135852075 | 1266.7798779723 | Turkmenistan |
| 2008 | 1299.80322599522 | 0.611923308807263 | 70.3634138363019 | 1229.43981215892 | Turkmenistan |
| 2009 | 1179.93942769326 | 0.617652905277457 | 62.0394558514992 | 1117.89997184176 | Turkmenistan |
| 2010 | 987.90737257482 | 0.623234588644236 | 64.3009757315123 | 923.606396843308 | Turkmenistan |
| 2011 | 797.11372347547 | 0.629233721702303 | 55.5764877838749 | 741.537235691595 | Turkmenistan |
| 2012 | 513.89428994582 | 0.635263183091614 | 52.7518135364692 | 461.142476409351 | Turkmenistan |
| 2013 | 451.50757039909 | 0.641361893351002 | 50.3974898355403 | 401.11008056355 | Turkmenistan |
| 2014 | 324.32923374991 | 0.647542416932676 | 50.3836677360336 | 273.945566013876 | Turkmenistan |
| 2015 | 330.97853017063 | 0.65335278951701 | 45.6549501141459 | 285.323580056484 | Turkmenistan |
| 2016 | 319.2497167 | 0.658945613392641 | 45.1128755562938 | 274.136841103106 | Turkmenistan |
| 2017 | 312.28024490877 | 0.664284691942071 | 40.9869388273054 | 271.293306081465 | Turkmenistan |
| 2018 | 277.97525986203 | 0.669433598508271 | 38.510769361369 | 239.464490500661 | Turkmenistan |
| 2019 | 257.49784804424 | 0.674303535870544 | 38.1735599586732 | 219.324288085567 | Turkmenistan |
| 2020 | 237.82248904753 | 0.678298179976148 | 37.2458620290976 | 200.576627018432 | Turkmenistan |
| 2021 | 221.98340745449 | 0.682160775869611 | 37.3404028582046 | 184.643004596285 | Turkmenistan |
| 1990 | 3792.00529277761 | 0.500241735138187 | 230.777578899011 | 3561.2277138786 | Uzbekistan |
| 1991 | 3794.29267922503 | 0.501544609458551 | 230.507704966778 | 3563.78497425825 | Uzbekistan |
| 1992 | 3946.94149039204 | 0.502905194308151 | 225.039280695356 | 3721.90220969668 | Uzbekistan |
| 1993 | 3836.47118417668 | 0.506556615160302 | 219.536181370303 | 3616.93500280638 | Uzbekistan |
| 1994 | 3774.58061088449 | 0.5110185249116 | 212.917586535124 | 3561.66302434937 | Uzbekistan |
| 1995 | 3315.31724251916 | 0.51698626355212 | 204.454293270718 | 3110.86294924844 | Uzbekistan |
| 1996 | 2691.31592054693 | 0.524392909803147 | 190.989770941376 | 2500.32614960555 | Uzbekistan |
| 1997 | 2112.92475758281 | 0.532686328470226 | 171.695428881609 | 1941.2293287012 | Uzbekistan |
| 1998 | 1483.26441856178 | 0.541279069137397 | 155.306856812998 | 1327.95756174878 | Uzbekistan |
| 1999 | 1178.4331101775 | 0.549739390933737 | 146.323611524878 | 1032.10949865262 | Uzbekistan |
| 2000 | 889.08705105292 | 0.557522310762499 | 142.258306025147 | 746.828745027773 | Uzbekistan |
| 2001 | 818.94981391533 | 0.564803382897778 | 110.039187551262 | 708.910626364068 | Uzbekistan |
| 2002 | 760.54384601602 | 0.571690142599581 | 88.5143596985343 | 672.029486317486 | Uzbekistan |
| 2003 | 710.63258872157 | 0.57800653838209 | 84.8042348940004 | 625.82835382757 | Uzbekistan |
| 2004 | 653.05877631244 | 0.583942986424626 | 80.039023564286 | 573.019752748154 | Uzbekistan |
| 2005 | 504.48200506698 | 0.589530776570668 | 81.4111324071475 | 423.070872659832 | Uzbekistan |
| 2006 | 295.85247807134 | 0.594498056671551 | 78.9470875054995 | 216.90539056584 | Uzbekistan |
| 2007 | 248.89852710876 | 0.599628054727829 | 75.281613956871 | 173.616913151889 | Uzbekistan |
| 2008 | 237.86212132567 | 0.604889158157396 | 75.0819668684474 | 162.780154457223 | Uzbekistan |
| 2009 | 228.27808504058 | 0.610351745244056 | 70.1283099860594 | 158.149775054521 | Uzbekistan |
| 2010 | 218.51028554109 | 0.616097907401681 | 68.1798496669395 | 150.33043587415 | Uzbekistan |
| 2011 | 194.32522970444 | 0.621344618930939 | 63.4472746652506 | 130.877955039189 | Uzbekistan |
| 2012 | 142.18884244155 | 0.62620212543294 | 59.9977840444587 | 82.1910583970913 | Uzbekistan |
| 2013 | 88.67702771 | 0.630796403406203 | 55.4127672002619 | 33.2642605102981 | Uzbekistan |
| 2014 | 71.42497598 | 0.635257049718365 | 52.6673647991242 | 18.7576111809458 | Uzbekistan |
| 2015 | 69.97694536 | 0.639805638905225 | 51.6885560683102 | 18.2883892870998 | Uzbekistan |
| 2016 | 66.46670667 | 0.644230597300764 | 49.8288772744349 | 16.6378293966851 | Uzbekistan |
| 2017 | 41.6815624 | 0.648119346591586 | 41.6815624 | 0 | Uzbekistan |
| 2018 | 49.02336018 | 0.651918144182724 | 45.3980849856326 | 3.62527519542739 | Uzbekistan |
| 2019 | 46.12774768 | 0.655836959268026 | 45.1517557928722 | 0.975991890957808 | Uzbekistan |
| 2020 | 39.00210065 | 0.659124204371032 | 39.00210065 | 0 | Uzbekistan |
| 2021 | 40.21468902 | 0.662621694181816 | 39.9274970419445 | 0.287191983005528 | Uzbekistan |
| 1990 | 574.62196697203 | 0.557773300317074 | 141.857245038716 | 432.764721933314 | Albania |
| 1991 | 596.31426832988 | 0.555629411790174 | 143.949649388192 | 452.364618941688 | Albania |
| 1992 | 584.74436661896 | 0.553107897492912 | 146.322997423818 | 438.421369195142 | Albania |
| 1993 | 562.95217038194 | 0.552453327893755 | 146.121450726875 | 416.830719655065 | Albania |
| 1994 | 650.15211923292 | 0.553763669315605 | 143.679038901319 | 506.473080331601 | Albania |
| 1995 | 483.43766985733 | 0.557040889185472 | 142.1341167 | 341.30355319273 | Albania |
| 1996 | 439.02188775618 | 0.562248886206679 | 138.662184244445 | 300.359703511735 | Albania |
| 1997 | 411.05546002642 | 0.566802804232969 | 107.050461400206 | 304.004998626214 | Albania |
| 1998 | 376.60544656681 | 0.572534101516668 | 83.0979317107824 | 293.507514856028 | Albania |
| 1999 | 319.6039927 | 0.578295461805887 | 85.8522407421361 | 233.751751921064 | Albania |
| 2000 | 278.01010625915 | 0.58466418835901 | 82.4690305603821 | 195.541075698768 | Albania |
| 2001 | 248.4259462 | 0.592412946510661 | 79.891784791694 | 168.534161421206 | Albania |
| 2002 | 222.64904271771 | 0.599865977568749 | 74.7965302677765 | 147.852512449934 | Albania |
| 2003 | 199.87990101254 | 0.606971165987994 | 74.352258713266 | 125.527642299274 | Albania |
| 2004 | 179.46450535449 | 0.613864246664272 | 68.4804207234129 | 110.984084631077 | Albania |
| 2005 | 164.85902849297 | 0.620679614590226 | 62.6412348303292 | 102.217793662641 | Albania |
| 2006 | 156.60111721961 | 0.627485617497143 | 60.5733009717889 | 96.0278162478211 | Albania |
| 2007 | 150.19859241933 | 0.634167968985356 | 53.7986337282377 | 96.3999586910923 | Albania |
| 2008 | 146.13416705821 | 0.640766134349698 | 50.7868181159932 | 95.3473489422169 | Albania |
| 2009 | 142.82581962105 | 0.646816130123751 | 48.4573079937233 | 94.3685116273267 | Albania |
| 2010 | 141.2221213 | 0.652654202642964 | 45.9652132669522 | 95.2569080302478 | Albania |
| 2011 | 143.66628364939 | 0.658196772707548 | 44.7013971254431 | 98.9648865239469 | Albania |
| 2012 | 143.31879121399 | 0.66355677961127 | 40.4672290693162 | 102.851562144674 | Albania |
| 2013 | 141.09644403431 | 0.668739558985928 | 39.3587390042749 | 101.737705030035 | Albania |
| 2014 | 137.32936603221 | 0.67411298539372 | 38.0642361975781 | 99.2651298346319 | Albania |
| 2015 | 134.41938936703 | 0.679668600655757 | 37.4680323994635 | 96.9513569675665 | Albania |
| 2016 | 127.57627518859 | 0.68503664759447 | 37.1709543204439 | 90.4053208681461 | Albania |
| 2017 | 113.43552446959 | 0.690379199197076 | 36.5144043581678 | 76.9211201114222 | Albania |
| 2018 | 99.15280059 | 0.695566877664306 | 36.9109915966183 | 62.2418089917417 | Albania |
| 2019 | 84.48543124 | 0.700287863295906 | 35.7085452533829 | 48.776885983987 | Albania |
| 2020 | 76.87079463 | 0.703790245372837 | 34.1877072283974 | 42.6830874047026 | Albania |
| 2021 | 68.66758398 | 0.706849790878611 | 34.1856345776467 | 34.4819493988833 | Albania |
| 1990 | 164.47855229468 | 0.541132539726015 | 155.318448010081 | 9.16010428459853 | Bosnia and Herzegovina |
| 1991 | 157.33282093361 | 0.541346250550883 | 155.244794194175 | 2.08802673943509 | Bosnia and Herzegovina |
| 1992 | 156.39643393276 | 0.539303227421668 | 155.585642060275 | 0.810791872484998 | Bosnia and Herzegovina |
| 1993 | 154.77485018779 | 0.53603188160081 | 154.77485018779 | 0 | Bosnia and Herzegovina |
| 1994 | 156.74227667251 | 0.533853389744284 | 156.74227667251 | 0 | Bosnia and Herzegovina |
| 1995 | 157.83246043442 | 0.534025536110865 | 157.232069377136 | 0.600391057283758 | Bosnia and Herzegovina |
| 1996 | 145.99141891236 | 0.545549215040903 | 145.99141891236 | 0 | Bosnia and Herzegovina |
| 1997 | 137.73816091291 | 0.563565414584231 | 136.715550582859 | 1.02261033005095 | Bosnia and Herzegovina |
| 1998 | 130.77349772894 | 0.58182951538312 | 85.8733355235462 | 44.9001622053938 | Bosnia and Herzegovina |
| 1999 | 114.84311681685 | 0.597997468748356 | 79.0824698084145 | 35.7606470084355 | Bosnia and Herzegovina |
| 2000 | 108.04533046445 | 0.610950684608214 | 70.1647172907733 | 37.8806131736767 | Bosnia and Herzegovina |
| 2001 | 100.17274222701 | 0.621989463929258 | 64.5037398823721 | 35.6690023446379 | Bosnia and Herzegovina |
| 2002 | 112.59187 | 0.632075517723657 | 54.9767503829244 | 57.6151196584756 | Bosnia and Herzegovina |
| 2003 | 117.03700836202 | 0.640635102031521 | 49.8580520350959 | 67.1789563269241 | Bosnia and Herzegovina |
| 2004 | 117.52747687101 | 0.64779373168743 | 50.4856909220163 | 67.0417859489937 | Bosnia and Herzegovina |
| 2005 | 116.83585163214 | 0.653859136710253 | 45.6648731573884 | 71.1709784747516 | Bosnia and Herzegovina |
| 2006 | 120.13932330138 | 0.659813662252595 | 43.1929945282775 | 76.9463287731026 | Bosnia and Herzegovina |
| 2007 | 121.11371323925 | 0.665578735044097 | 39.7672755960178 | 81.3464376432322 | Bosnia and Herzegovina |
| 2008 | 119.86907399472 | 0.671100813673566 | 38.2829074168282 | 81.5861665778918 | Bosnia and Herzegovina |
| 2009 | 117.36420793313 | 0.676032900022704 | 37.822982086873 | 79.541225846257 | Bosnia and Herzegovina |
| 2010 | 116.05799114116 | 0.680824195636093 | 37.4300956157373 | 78.6278955254227 | Bosnia and Herzegovina |
| 2011 | 108.83758606339 | 0.685399264182467 | 37.319635270456 | 71.517950792934 | Bosnia and Herzegovina |
| 2012 | 107.97529928789 | 0.689506566189932 | 37.1993885126394 | 70.7759107752506 | Bosnia and Herzegovina |
| 2013 | 110.6428592 | 0.693508711211908 | 36.7097874637935 | 73.9330716931065 | Bosnia and Herzegovina |
| 2014 | 103.67386939232 | 0.697031893012906 | 35.844926857311 | 67.828942535009 | Bosnia and Herzegovina |
| 2015 | 108.53931123064 | 0.700446047366461 | 35.9286636592132 | 72.6106475714268 | Bosnia and Herzegovina |
| 2016 | 108.07827158473 | 0.704079343718599 | 34.6067215431723 | 73.4715500415577 | Bosnia and Herzegovina |
| 2017 | 105.78685503031 | 0.70805343112809 | 34.6773530241946 | 71.1095020061154 | Bosnia and Herzegovina |
| 2018 | 101.13161607663 | 0.712347302038319 | 31.8477499719059 | 69.2838661047241 | Bosnia and Herzegovina |
| 2019 | 98.5634931 | 0.716857124768862 | 30.762085508982 | 67.801407591888 | Bosnia and Herzegovina |
| 2020 | 88.75079769 | 0.720202056378697 | 28.8805879357646 | 59.8702097497254 | Bosnia and Herzegovina |
| 2021 | 82.6176254 | 0.72307789321024 | 29.2755949633714 | 53.3420304320786 | Bosnia and Herzegovina |
| 1990 | 158.6448057 | 0.633446498017686 | 52.3413910387987 | 106.303414692101 | Bulgaria |
| 1991 | 184.9174074 | 0.643069487769455 | 51.1052960959915 | 133.812111284708 | Bulgaria |
| 1992 | 188.71397132729 | 0.651186876165789 | 47.1836110963756 | 141.530360230914 | Bulgaria |
| 1993 | 167.97838662547 | 0.659161569264411 | 43.9223963184419 | 124.055990307028 | Bulgaria |
| 1994 | 198.66972442914 | 0.667430460276086 | 39.3490889215593 | 159.320635507581 | Bulgaria |
| 1995 | 159.71307029191 | 0.673999976701174 | 37.9069347300894 | 121.806135561821 | Bulgaria |
| 1996 | 152.80605618146 | 0.678862583541026 | 37.4708901971083 | 115.335165984352 | Bulgaria |
| 1997 | 173.5975085 | 0.680221466342146 | 37.5854310581644 | 136.012077456936 | Bulgaria |
| 1998 | 145.56370914152 | 0.678496876732148 | 37.6772652358875 | 107.886443905633 | Bulgaria |
| 1999 | 122.64615933015 | 0.677446543282232 | 37.55387701 | 85.09228232 | Bulgaria |
| 2000 | 133.46930513793 | 0.681004523812614 | 37.5278363361848 | 95.9414688017453 | Bulgaria |
| 2001 | 115.4198589 | 0.686487366008627 | 37.258687153728 | 78.161171700872 | Bulgaria |
| 2002 | 117.06452981224 | 0.69151167375307 | 36.6931987176096 | 80.3713310946303 | Bulgaria |
| 2003 | 104.11567102752 | 0.69556596473307 | 36.561069645856 | 67.554601381664 | Bulgaria |
| 2004 | 96.93936221 | 0.699881950859544 | 35.443570661608 | 61.495791546422 | Bulgaria |
| 2005 | 86.80230739 | 0.704471206966935 | 35.1986818357644 | 51.6036255525056 | Bulgaria |
| 2006 | 82.70913185 | 0.708868293577424 | 34.2658960807673 | 48.4432357668227 | Bulgaria |
| 2007 | 80.23743568 | 0.713192402424104 | 31.4881371121562 | 48.7492985722037 | Bulgaria |
| 2008 | 89.92622407 | 0.717672450499802 | 31.3436547946144 | 58.5825692707555 | Bulgaria |
| 2009 | 90.76346368 | 0.723676626795129 | 28.459696670638 | 62.303767013272 | Bulgaria |
| 2010 | 105.7699181 | 0.730701737095494 | 24.6032311834697 | 81.1666869075303 | Bulgaria |
| 2011 | 128.08554846835 | 0.735749281014389 | 20.1924460584321 | 107.893102409918 | Bulgaria |
| 2012 | 153.56651654255 | 0.739177517343213 | 20.7975254831172 | 132.768991059433 | Bulgaria |
| 2013 | 171.93381942911 | 0.742135948467762 | 20.6480711886254 | 151.285748240485 | Bulgaria |
| 2014 | 192.42586665792 | 0.745372642577071 | 19.470756859152 | 172.955109798768 | Bulgaria |
| 2015 | 177.25934040693 | 0.748979058373083 | 18.7957590682934 | 158.463581338637 | Bulgaria |
| 2016 | 162.41231349932 | 0.752259198563744 | 18.1542718915812 | 144.258041607739 | Bulgaria |
| 2017 | 159.86367545985 | 0.75566586464625 | 17.7818395845835 | 142.081835875266 | Bulgaria |
| 2018 | 142.13299453591 | 0.759148460251881 | 15.9059203063998 | 126.22707422951 | Bulgaria |
| 2019 | 124.49478080947 | 0.762359790844767 | 14.573902166551 | 109.920878642919 | Bulgaria |
| 2020 | 107.64103676616 | 0.765089710642011 | 14.532863728485 | 93.108173037675 | Bulgaria |
| 2021 | 105.89128928591 | 0.768150939342518 | 13.7783614313594 | 92.1129278545506 | Bulgaria |
| 1990 | 149.71295569759 | 0.668906358291928 | 39.0280339740893 | 110.684921723501 | Croatia |
| 1991 | 145.58184250431 | 0.674724599866063 | 37.7493263258027 | 107.832516178507 | Croatia |
| 1992 | 152.4618957 | 0.678093133084916 | 37.496577102454 | 114.965318592746 | Croatia |
| 1993 | 143.05274824744 | 0.678221047043161 | 37.5761456448266 | 105.476602602613 | Croatia |
| 1994 | 142.57926394594 | 0.677548132655056 | 37.4220251640225 | 105.157238781917 | Croatia |
| 1995 | 118.57933638409 | 0.677029465132868 | 38.187284292923 | 80.392052091167 | Croatia |
| 1996 | 110.7572796 | 0.679507318367616 | 37.5345290112612 | 73.2227506081388 | Croatia |
| 1997 | 108.93217458118 | 0.685365010443468 | 37.3429036438648 | 71.5892709373152 | Croatia |
| 1998 | 94.89073126 | 0.691365518598319 | 36.7288948262283 | 58.1618364303817 | Croatia |
| 1999 | 75.60863639 | 0.696636411104102 | 35.8675766442562 | 39.7410597468938 | Croatia |
| 2000 | 75.21142684 | 0.703039898563322 | 34.6216084445623 | 40.5898183911077 | Croatia |
| 2001 | 72.19749983 | 0.709536840872917 | 33.6504124380419 | 38.5470873947481 | Croatia |
| 2002 | 68.36154129 | 0.715383421635164 | 31.8475472861807 | 36.5139940008093 | Croatia |
| 2003 | 60.8814706 | 0.721130377216859 | 29.3676385486742 | 31.5138320472258 | Croatia |
| 2004 | 58.46176217 | 0.726327651623968 | 26.6646882068841 | 31.7970739594159 | Croatia |
| 2005 | 57.46832684 | 0.732154139815163 | 24.3307059007241 | 33.1376209422659 | Croatia |
| 2006 | 55.09351966 | 0.738066878515765 | 22.4426643176182 | 32.6508553435718 | Croatia |
| 2007 | 58.74152478 | 0.743148776515466 | 20.9632049179561 | 37.7783198625539 | Croatia |
| 2008 | 63.83822553 | 0.748469602229701 | 18.063097548118 | 45.775127981822 | Croatia |
| 2009 | 76.19434479 | 0.754065605198048 | 18.4297086925325 | 57.7646361010075 | Croatia |
| 2010 | 91.62659863 | 0.759472251706366 | 16.4492551861392 | 75.1773434418008 | Croatia |
| 2011 | 122.40389123164 | 0.763686381293279 | 15.3907535289078 | 107.013137702732 | Croatia |
| 2012 | 144.14703114796 | 0.767405335599999 | 13.3113870331049 | 130.835644114855 | Croatia |
| 2013 | 173.76677971866 | 0.771151880279813 | 12.9493715247844 | 160.817408193876 | Croatia |
| 2014 | 193.03900415421 | 0.774479240521915 | 13.1332953459806 | 179.905708808229 | Croatia |
| 2015 | 215.12501117001 | 0.777865888163329 | 12.5600557017822 | 202.564955468228 | Croatia |
| 2016 | 225.64216107384 | 0.781443605436965 | 10.7926975151474 | 214.849463558693 | Croatia |
| 2017 | 238.64796858924 | 0.785074536823775 | 11.7987875251613 | 226.849181064079 | Croatia |
| 2018 | 234.79062030455 | 0.788671410129235 | 11.6080431196811 | 223.182577184869 | Croatia |
| 2019 | 207.08472396063 | 0.792475415454183 | 11.1252799884216 | 195.959443972208 | Croatia |
| 2020 | 193.96597865495 | 0.795462271729482 | 10.5305441810396 | 183.43543447391 | Croatia |
| 2021 | 185.01258259101 | 0.798341026936276 | 10.4271745911616 | 174.585407999848 | Croatia |
| 1990 | 186.96650630644 | 0.681848021213857 | 37.5732603968902 | 149.39324590955 | Czechia |
| 1991 | 191.14950616734 | 0.688002859136679 | 37.1694998683355 | 153.980006299005 | Czechia |
| 1992 | 190.13832490167 | 0.697577809122723 | 35.6111005843209 | 154.527224317349 | Czechia |
| 1993 | 166.20427394998 | 0.710853415862456 | 33.1741981522568 | 133.030075797723 | Czechia |
| 1994 | 155.58994421491 | 0.725969686167497 | 27.2457399667834 | 128.344204248127 | Czechia |
| 1995 | 121.5249469 | 0.737981721993762 | 22.577055424426 | 98.947891459274 | Czechia |
| 1996 | 102.58267127593 | 0.746041379938535 | 18.4980285707709 | 84.0846427051591 | Czechia |
| 1997 | 106.2319716 | 0.751680343695868 | 19.0388541990179 | 87.1931173558821 | Czechia |
| 1998 | 96.29727158 | 0.757033755457614 | 16.5900846008693 | 79.7071869787107 | Czechia |
| 1999 | 78.78551964 | 0.762428880357609 | 15.0650796413772 | 63.7204400015328 | Czechia |
| 2000 | 78.5949296 | 0.767766159168669 | 13.8786616109093 | 64.7162679842007 | Czechia |
| 2001 | 74.00813117 | 0.772167239650309 | 14.4770504354665 | 59.5310807344635 | Czechia |
| 2002 | 76.17777618 | 0.776927934062507 | 12.1533457017357 | 64.0244304817843 | Czechia |
| 2003 | 69.57779978 | 0.782448081111072 | 11.7471831813907 | 57.8306165996293 | Czechia |
| 2004 | 65.42678702 | 0.787126404974676 | 11.3441051658037 | 54.0826818498263 | Czechia |
| 2005 | 59.53027544 | 0.79133394820415 | 11.2936073248743 | 48.2366681193257 | Czechia |
| 2006 | 58.27143444 | 0.794922783687463 | 11.1264983846843 | 47.1449360548358 | Czechia |
| 2007 | 60.23870552 | 0.798254117717088 | 10.3294512657772 | 49.9092542494828 | Czechia |
| 2008 | 62.65013282 | 0.8017648456328 | 10.2994332349849 | 52.3506995818251 | Czechia |
| 2009 | 74.92627212 | 0.804962224676367 | 9.90538802103347 | 65.0208840947265 | Czechia |
| 2010 | 94.86042135 | 0.808747327653098 | 9.21074300735039 | 85.6496783436896 | Czechia |
| 2011 | 118.92315727309 | 0.812054784320429 | 9.66462841898102 | 109.258528854109 | Czechia |
| 2012 | 141.61537065841 | 0.814230401372392 | 9.15763592615017 | 132.45773473226 | Czechia |
| 2013 | 157.38661245437 | 0.815615361108922 | 8.84976214774586 | 148.536850306624 | Czechia |
| 2014 | 178.34284746364 | 0.816227257169909 | 8.75554305377174 | 169.587304409868 | Czechia |
| 2015 | 204.68569554011 | 0.816564684423948 | 8.87570847394662 | 195.809987066163 | Czechia |
| 2016 | 225.98660164195 | 0.817153025533518 | 8.82901653253055 | 217.157585109419 | Czechia |
| 2017 | 228.02066933254 | 0.819067680353287 | 9.14460159706339 | 218.876067735477 | Czechia |
| 2018 | 234.16727237464 | 0.822034486079137 | 7.98489188271954 | 226.18238049192 | Czechia |
| 2019 | 249.48172072274 | 0.824784472798171 | 8.525952676 | 240.95576804716 | Czechia |
| 2020 | 205.71500056848 | 0.82662663116323 | 8.16602147228559 | 197.548979096194 | Czechia |
| 2021 | 194.13192637928 | 0.828450433122785 | 8.07870235917227 | 186.053224020108 | Czechia |
| 1990 | 121.69999404564 | 0.649419990664883 | 46.7648821283713 | 74.9351119172687 | Hungary |
| 1991 | 126.84034550047 | 0.653707154681922 | 45.5129104459505 | 81.3274350545194 | Hungary |
| 1992 | 120.7022386 | 0.660382905455002 | 42.5980822924259 | 78.1041562894741 | Hungary |
| 1993 | 109.50137371995 | 0.667543556201878 | 39.1288004679888 | 70.3725732519612 | Hungary |
| 1994 | 103.08820525012 | 0.675051406249796 | 37.6901920121252 | 65.3980132379948 | Hungary |
| 1995 | 84.89330702 | 0.683186411756315 | 37.5879420135663 | 47.3053650065537 | Hungary |
| 1996 | 78.61650841 | 0.690883333624564 | 36.6336179829649 | 41.9828904227751 | Hungary |
| 1997 | 76.15181517 | 0.697935513627811 | 35.7066590707667 | 40.4451560955633 | Hungary |
| 1998 | 73.78808693 | 0.704202719984894 | 34.8793476656617 | 38.9087392621883 | Hungary |
| 1999 | 61.40183699 | 0.709980626709343 | 33.4272211883368 | 27.9746158014932 | Hungary |
| 2000 | 67.12218363 | 0.71580528057596 | 31.1724490258846 | 35.9497346016054 | Hungary |
| 2001 | 59.066604 | 0.722099055327699 | 27.5790181726704 | 31.4875858256796 | Hungary |
| 2002 | 54.87907038 | 0.7283845957697 | 26.4022599696106 | 28.4768104142094 | Hungary |
| 2003 | 50.38604565 | 0.734450657925147 | 21.5032901425579 | 28.882755509182 | Hungary |
| 2004 | 46.60055188 | 0.740099974680479 | 20.726478712883 | 25.874073165397 | Hungary |
| 2005 | 45.38461114 | 0.745356806381464 | 20.2625470463475 | 25.1220640960325 | Hungary |
| 2006 | 43.45623374 | 0.750515380971436 | 18.1769856048648 | 25.2792481314352 | Hungary |
| 2007 | 43.60080915 | 0.754942942046377 | 18.010596081757 | 25.590213067563 | Hungary |
| 2008 | 47.08449476 | 0.75931746766686 | 18.1216954168315 | 28.9627993394585 | Hungary |
| 2009 | 53.44792764 | 0.763563686880312 | 16.4250329647244 | 37.0228946776356 | Hungary |
| 2010 | 70.38033031 | 0.767328649261652 | 13.3331041378098 | 57.0472261740802 | Hungary |
| 2011 | 96.78856545 | 0.769701942198238 | 13.782876447993 | 83.005689004837 | Hungary |
| 2012 | 127.73098443284 | 0.770784245285382 | 12.1600710032259 | 115.570913429614 | Hungary |
| 2013 | 154.40920904786 | 0.77156169789519 | 13.5625332230852 | 140.846675824775 | Hungary |
| 2014 | 169.18325452151 | 0.772575045716759 | 13.9446703588505 | 155.23858416266 | Hungary |
| 2015 | 179.27965995118 | 0.774047382518708 | 13.6949342917864 | 165.584725659394 | Hungary |
| 2016 | 169.22260432006 | 0.776150305326983 | 11.8749327251008 | 157.347671594959 | Hungary |
| 2017 | 161.59979080569 | 0.779116417184348 | 12.7263006067716 | 148.873490198918 | Hungary |
| 2018 | 161.77121534396 | 0.782083661193681 | 11.235482967692 | 150.535732376268 | Hungary |
| 2019 | 189.99099570119 | 0.784910308752534 | 12.5703722806143 | 177.420623420576 | Hungary |
| 2020 | 171.48595178916 | 0.787620389699141 | 11.4504415540216 | 160.035510235138 | Hungary |
| 2021 | 159.73189857285 | 0.790754768173338 | 11.5451114400191 | 148.186787132831 | Hungary |
| 1990 | 2675.02562869477 | 0.60902609384517 | 70.1689466308405 | 2604.85668206393 | North Macedonia |
| 1991 | 2496.49965028539 | 0.611337452940336 | 69.6161720228897 | 2426.8834782625 | North Macedonia |
| 1992 | 2398.47368881152 | 0.612708856229581 | 68.6328290801767 | 2329.84085973134 | North Macedonia |
| 1993 | 2021.4191821453 | 0.613195098529578 | 70.5235729634034 | 1950.8956091819 | North Macedonia |
| 1994 | 1882.69006404827 | 0.614362905009181 | 69.2147473617557 | 1813.47531668651 | North Macedonia |
| 1995 | 1646.45909464599 | 0.616994877821318 | 64.2301910125633 | 1582.22890363343 | North Macedonia |
| 1996 | 1340.20330687333 | 0.620944017369103 | 65.5719234537454 | 1274.63138341958 | North Macedonia |
| 1997 | 1205.91144679824 | 0.626223811356564 | 60.1210423726802 | 1145.79040442556 | North Macedonia |
| 1998 | 1146.1690872744 | 0.632059410181692 | 53.0410850867018 | 1093.1280021877 | North Macedonia |
| 1999 | 827.41438312468 | 0.63668725909008 | 51.3596703575188 | 776.054712767161 | North Macedonia |
| 2000 | 668.31814582293 | 0.641651242944231 | 52.8920998123807 | 615.426046010549 | North Macedonia |
| 2001 | 621.15539285531 | 0.648049675806335 | 49.9279109880612 | 571.227481867249 | North Macedonia |
| 2002 | 571.32209719793 | 0.655398790077733 | 44.9757903990309 | 526.346306798899 | North Macedonia |
| 2003 | 574.55242252759 | 0.663408891257403 | 41.2013903675528 | 533.351032160037 | North Macedonia |
| 2004 | 509.10963412414 | 0.671237520250547 | 38.5322843256592 | 470.577349798481 | North Macedonia |
| 2005 | 446.35506167533 | 0.678047272834883 | 37.5694371340359 | 408.785624541294 | North Macedonia |
| 2006 | 460.12579152595 | 0.684044336465219 | 37.3577346417101 | 422.76805688424 | North Macedonia |
| 2007 | 444.30379692837 | 0.689729480929241 | 37.5140607768132 | 406.789736151557 | North Macedonia |
| 2008 | 453.7587431 | 0.695376955204976 | 36.5288517690277 | 417.229891376772 | North Macedonia |
| 2009 | 480.2796027 | 0.700453052486829 | 35.5497001476262 | 444.729902541074 | North Macedonia |
| 2010 | 424.98461839333 | 0.705589323990971 | 35.0176088397926 | 389.967009553537 | North Macedonia |
| 2011 | 428.38259371693 | 0.710592520608989 | 33.4601254460905 | 394.92246827084 | North Macedonia |
| 2012 | 480.66800822737 | 0.715173147623766 | 32.2445929800343 | 448.423415247336 | North Macedonia |
| 2013 | 485.24324693978 | 0.719671149159541 | 28.9347359371158 | 456.308511002664 | North Macedonia |
| 2014 | 474.75621545266 | 0.724089588503488 | 29.1358863480095 | 445.62032910465 | North Macedonia |
| 2015 | 466.72146903026 | 0.728603773495479 | 25.667686381206 | 441.053782649054 | North Macedonia |
| 2016 | 519.58190791357 | 0.733173624358204 | 21.953437095097 | 497.628470818473 | North Macedonia |
| 2017 | 451.21870237273 | 0.737339955393252 | 21.7330430140054 | 429.485659358725 | North Macedonia |
| 2018 | 341.05949939794 | 0.741334369868002 | 20.8575223817059 | 320.201977016234 | North Macedonia |
| 2019 | 329.19127619673 | 0.74519356873628 | 20.7864874229616 | 308.404788773768 | North Macedonia |
| 2020 | 236.03500740571 | 0.747849899969173 | 19.698724922046 | 216.336282483664 | North Macedonia |
| 2021 | 189.11630563856 | 0.750629703270047 | 19.1442130703583 | 169.972092568202 | North Macedonia |
| 1990 | 43.1329798 | 0.674225719793669 | 38.1634126304685 | 4.96956717331147 | Montenegro |
| 1991 | 40.34443063 | 0.674305722529628 | 37.6044755307569 | 2.73995509677312 | Montenegro |
| 1992 | 39.11148662 | 0.672664771832277 | 38.0715857760234 | 1.03990084501658 | Montenegro |
| 1993 | 39.14921212 | 0.668665719204089 | 38.8041739547225 | 0.345038161087501 | Montenegro |
| 1994 | 39.42008014 | 0.665011931002947 | 39.1446585106594 | 0.275421630850559 | Montenegro |
| 1995 | 39.08539498 | 0.662765437115331 | 39.0691988610072 | 0.0161961193527773 | Montenegro |
| 1996 | 39.05838344 | 0.663414768746409 | 39.0374784064017 | 0.0209050365382879 | Montenegro |
| 1997 | 38.28736419 | 0.665542031695028 | 38.28736419 | 0 | Montenegro |
| 1998 | 37.42406942 | 0.669024770846263 | 37.42406942 | 0 | Montenegro |
| 1999 | 36.91361937 | 0.672096102691543 | 36.91361937 | 0 | Montenegro |
| 2000 | 36.66687205 | 0.677306850284635 | 36.66687205 | 0 | Montenegro |
| 2001 | 36.6230055 | 0.683433454098582 | 36.6230055 | 0 | Montenegro |
| 2002 | 34.98833365 | 0.68993948313756 | 34.98833365 | 0 | Montenegro |
| 2003 | 33.56931501 | 0.696517506230494 | 33.56931501 | 0 | Montenegro |
| 2004 | 31.59362838 | 0.702998276030296 | 31.59362838 | 0 | Montenegro |
| 2005 | 30.44361341 | 0.709065464700775 | 30.44361341 | 0 | Montenegro |
| 2006 | 29.62068947 | 0.715459074033141 | 28.8154582320559 | 0.80523123789412 | Montenegro |
| 2007 | 28.69198323 | 0.722503939575403 | 27.140902984381 | 1.55108024331896 | Montenegro |
| 2008 | 29.92501428 | 0.729981755712811 | 22.7976775348208 | 7.1273367468392 | Montenegro |
| 2009 | 30.39311773 | 0.736538925645409 | 21.0726341067135 | 9.32048362763652 | Montenegro |
| 2010 | 30.52898255 | 0.743347873367499 | 20.2106222224435 | 10.3183603312165 | Montenegro |
| 2011 | 28.29183605 | 0.750080328162275 | 19.6199442394397 | 8.67189180810031 | Montenegro |
| 2012 | 26.74055628 | 0.755578379953158 | 18.0342439520997 | 8.70631232518033 | Montenegro |
| 2013 | 25.26748548 | 0.760886907477753 | 17.1770039158709 | 8.09048156413912 | Montenegro |
| 2014 | 23.79875239 | 0.765776526527122 | 14.4579920950017 | 9.34076029193826 | Montenegro |
| 2015 | 21.4399751 | 0.770561277708901 | 12.8724696819183 | 8.56750541374167 | Montenegro |
| 2016 | 19.91640406 | 0.775200770239438 | 11.53428829 | 8.382115773 | Montenegro |
| 2017 | 17.01977548 | 0.779764631848314 | 11.3803332042371 | 5.63944227960286 | Montenegro |
| 2018 | 14.95042251 | 0.784408971391706 | 10.3027867978918 | 4.64763571343824 | Montenegro |
| 2019 | 14.22722148 | 0.789026903095641 | 10.7295354655038 | 3.49768601582615 | Montenegro |
| 2020 | 13.19099964 | 0.792554392852858 | 10.5242082380967 | 2.66679139767333 | Montenegro |
| 2021 | 12.80462147 | 0.795800584021403 | 10.3521496795995 | 2.45247178953048 | Montenegro |
| 1990 | 142.64597429568 | 0.62722788770829 | 59.5209423161872 | 83.1250319794928 | Poland |
| 1991 | 113.04025262187 | 0.63267147050056 | 54.6264731444008 | 58.4137794774691 | Poland |
| 1992 | 100.77042466159 | 0.64066179337278 | 50.6893229457566 | 50.0811017158334 | Poland |
| 1993 | 81.12767474 | 0.648950499724012 | 48.4514342031528 | 32.6762405323572 | Poland |
| 1994 | 82.35737517 | 0.658010514323026 | 45.4390369922822 | 36.9183381825078 | Poland |
| 1995 | 74.78648716 | 0.666678866524195 | 39.6438963960165 | 35.1425907623735 | Poland |
| 1996 | 74.7016763 | 0.674378829090982 | 38.0756189871275 | 36.6260573146025 | Poland |
| 1997 | 63.35721965 | 0.682587612300048 | 37.4703477629702 | 25.8868718885598 | Poland |
| 1998 | 57.19871675 | 0.690805670678968 | 36.7106873901055 | 20.4880293583945 | Poland |
| 1999 | 53.81204235 | 0.6985360555797 | 35.5731277640015 | 18.2389145826685 | Poland |
| 2000 | 49.05828406 | 0.706662051990276 | 33.8596962643137 | 15.1985877980863 | Poland |
| 2001 | 48.80074684 | 0.714831304495085 | 30.4735878636147 | 18.3271589739353 | Poland |
| 2002 | 44.39009581 | 0.722009458087754 | 27.6077392278323 | 16.7823565824977 | Poland |
| 2003 | 36.61394593 | 0.728155253652566 | 24.3796573275935 | 12.2342886073465 | Poland |
| 2004 | 32.30352232 | 0.733673191846001 | 23.8596217338016 | 8.44390058764842 | Poland |
| 2005 | 33.20777926 | 0.738428766593663 | 20.9631533899571 | 12.2446258663029 | Poland |
| 2006 | 30.55909352 | 0.742363708580365 | 21.6701539797773 | 8.88893954449267 | Poland |
| 2007 | 38.68927288 | 0.746008817400637 | 20.2675933617334 | 18.4216795163366 | Poland |
| 2008 | 40.85995652 | 0.750521426475047 | 20.7668703898364 | 20.0930861326236 | Poland |
| 2009 | 46.55260704 | 0.756427378476364 | 19.008722261534 | 27.543884779206 | Poland |
| 2010 | 53.56326426 | 0.763184973687688 | 13.9993082380706 | 39.5639560231094 | Poland |
| 2011 | 56.32225205 | 0.769407164213607 | 13.8415307417222 | 42.4807213067678 | Poland |
| 2012 | 65.03521528 | 0.774680767244991 | 11.914699583545 | 53.120515695565 | Poland |
| 2013 | 84.48223413 | 0.779434257316854 | 11.3591734114965 | 73.1230607216935 | Poland |
| 2014 | 98.17133208 | 0.783682368684721 | 11.1524682220005 | 87.0188638616395 | Poland |
| 2015 | 109.80755889094 | 0.78744534315882 | 11.0227195916438 | 98.7848392992962 | Poland |
| 2016 | 125.35225484286 | 0.790651014611623 | 11.0758882436411 | 114.276366599219 | Poland |
| 2017 | 154.7727123 | 0.79453034463794 | 11.0411603824565 | 143.731551911044 | Poland |
| 2018 | 155.52136950957 | 0.79947898187813 | 10.2233837879186 | 145.297985721651 | Poland |
| 2019 | 149.13159081319 | 0.804791331675008 | 10.1395831481102 | 138.99200766508 | Poland |
| 2020 | 136.37580015982 | 0.808795317506383 | 9.16938431910143 | 127.206415840719 | Poland |
| 2021 | 130.22759206099 | 0.812042808667712 | 9.14406081062475 | 121.083531250365 | Poland |
| 1990 | 743.05112770996 | 0.619298862250078 | 61.9055603763299 | 681.14556733363 | Romania |
| 1991 | 552.09515436277 | 0.626795627433335 | 60.2590113535354 | 491.836143009235 | Romania |
| 1992 | 548.65344812145 | 0.630475081501162 | 54.9520846133579 | 493.701363508092 | Romania |
| 1993 | 570.76681142833 | 0.633801422118109 | 54.4248854400843 | 516.341925988246 | Romania |
| 1994 | 643.90941550747 | 0.638371931788576 | 52.8650466345119 | 591.044368872958 | Romania |
| 1995 | 526.50519344732 | 0.643718879089372 | 50.9100980986765 | 475.595095348644 | Romania |
| 1996 | 563.55354023568 | 0.648104195492848 | 48.1724645197772 | 515.381075715903 | Romania |
| 1997 | 536.09760135227 | 0.651225576178548 | 48.0039230705448 | 488.093678281725 | Romania |
| 1998 | 525.241948 | 0.654927768840234 | 45.8106668377548 | 479.431281199545 | Romania |
| 1999 | 460.36745464112 | 0.65954484605753 | 43.4233337997132 | 416.944120841407 | Romania |
| 2000 | 471.09777520427 | 0.66529434615185 | 39.8130232560497 | 431.28475194822 | Romania |
| 2001 | 403.31982071392 | 0.672020166603488 | 38.4525435882131 | 364.867277125707 | Romania |
| 2002 | 355.11834312392 | 0.678297416747085 | 37.7727187947239 | 317.345624329196 | Romania |
| 2003 | 323.39691035638 | 0.68407492928742 | 37.4279718207567 | 285.968938535623 | Romania |
| 2004 | 302.74416485163 | 0.690417054639929 | 36.561147345099 | 266.183017506531 | Romania |
| 2005 | 259.11748239912 | 0.694998949594957 | 36.4426479893581 | 222.674834409762 | Romania |
| 2006 | 216.78325506232 | 0.699490765699603 | 36.0655240074034 | 180.717731054917 | Romania |
| 2007 | 155.30193910288 | 0.704523726685579 | 34.2692646282769 | 121.032674474603 | Romania |
| 2008 | 152.29636580197 | 0.710757127721139 | 33.13716673 | 119.15919907084 | Romania |
| 2009 | 146.90811265158 | 0.717445122377497 | 31.6221837036209 | 115.285928947959 | Romania |
| 2010 | 156.34910390625 | 0.723891348732225 | 28.0917768612049 | 128.257327045045 | Romania |
| 2011 | 169.76047392088 | 0.729018272021873 | 26.3461306354748 | 143.414343285405 | Romania |
| 2012 | 178.47328662052 | 0.73389248238628 | 21.4101897241243 | 157.063096896396 | Romania |
| 2013 | 192.01391651317 | 0.737880472910291 | 21.330790405747 | 170.683126107423 | Romania |
| 2014 | 191.48524540982 | 0.740395466703106 | 20.4164562256912 | 171.068789184129 | Romania |
| 2015 | 199.27553350116 | 0.743473529634312 | 19.79246301 | 179.48307048814 | Romania |
| 2016 | 199.0683593 | 0.747058709528536 | 20.3863077972862 | 178.682051503714 | Romania |
| 2017 | 211.77436713142 | 0.751117366992228 | 18.3072895112519 | 193.467077620168 | Romania |
| 2018 | 204.00498345552 | 0.755533333480467 | 18.4252209890967 | 185.579762466423 | Romania |
| 2019 | 187.07309813675 | 0.760283970607593 | 16.8703605490623 | 170.202737587688 | Romania |
| 2020 | 172.75995434596 | 0.764276444363666 | 15.9177719220153 | 156.842182423945 | Romania |
| 2021 | 171.34015440179 | 0.768453864365965 | 14.724013302548 | 156.616141099242 | Romania |
| 1990 | 195.86827183032 | 0.630511019536119 | 56.9813274263681 | 138.886944403952 | Serbia |
| 1991 | 184.90069056793 | 0.638016934037528 | 51.8546093108945 | 133.046081257035 | Serbia |
| 1992 | 158.32375646291 | 0.641971993828041 | 51.5544864783739 | 106.769269984536 | Serbia |
| 1993 | 156.23365163776 | 0.642096034424249 | 50.5116532888941 | 105.721998348866 | Serbia |
| 1994 | 164.93641773817 | 0.642223220931048 | 50.1884888570839 | 114.747928881086 | Serbia |
| 1995 | 156.00743117814 | 0.642739671389532 | 51.8669621924837 | 104.140468985656 | Serbia |
| 1996 | 141.93522533863 | 0.644846417030162 | 49.1113020514793 | 92.8239232871507 | Serbia |
| 1997 | 129.64802942959 | 0.64716209445227 | 50.1200971132041 | 79.5279323163859 | Serbia |
| 1998 | 119.79873260362 | 0.65024598899818 | 47.2961454497048 | 72.5025871539152 | Serbia |
| 1999 | 101.79303386944 | 0.655534174135398 | 46.0146063045202 | 55.7784275649198 | Serbia |
| 2000 | 97.63253323 | 0.660752800457895 | 43.8651265031731 | 53.7674067252369 | Serbia |
| 2001 | 99.34334062 | 0.665407334806451 | 39.9410681599313 | 59.4022724622287 | Serbia |
| 2002 | 104.21174536013 | 0.671511071572143 | 38.6657886119145 | 65.5459567482154 | Serbia |
| 2003 | 114.48758385131 | 0.678334928718905 | 37.5012885718416 | 76.9862952794684 | Serbia |
| 2004 | 100.43700087122 | 0.686819841842618 | 37.1210835053639 | 63.3159173658561 | Serbia |
| 2005 | 89.0812799 | 0.69609756790601 | 36.7456913513023 | 52.3355885498577 | Serbia |
| 2006 | 82.38740411 | 0.704945018093173 | 34.5728716372693 | 47.8145324736607 | Serbia |
| 2007 | 76.17491493 | 0.713242304525204 | 32.3679312096603 | 43.8069837159697 | Serbia |
| 2008 | 59.03719549 | 0.721505305994409 | 28.0884287386513 | 30.9487667481787 | Serbia |
| 2009 | 69.90468334 | 0.729965595799672 | 24.4401807730946 | 45.4645025627654 | Serbia |
| 2010 | 67.37822361 | 0.740091311255812 | 21.37156585 | 46.00665776 | Serbia |
| 2011 | 66.24136959 | 0.750072761758797 | 19.3905282133369 | 46.8508413756231 | Serbia |
| 2012 | 61.83873038 | 0.757105082 | 16.305703767107 | 45.533026609233 | Serbia |
| 2013 | 66.91292163 | 0.762170908106883 | 14.2203378326079 | 52.6925837960821 | Serbia |
| 2014 | 68.22449455 | 0.766043091132074 | 14.0084718029476 | 54.2160227508124 | Serbia |
| 2015 | 70.3099298 | 0.769842222617687 | 14.6642992357568 | 55.6456305653932 | Serbia |
| 2016 | 71.41966137 | 0.773654077278106 | 13.9943887062271 | 57.4252726598829 | Serbia |
| 2017 | 71.79893814 | 0.777460384270565 | 13.236322166843 | 58.562615974867 | Serbia |
| 2018 | 68.11548041 | 0.781494973790012 | 11.7319929848293 | 56.3834874234206 | Serbia |
| 2019 | 65.86708284 | 0.785768300182747 | 11.691952240443 | 54.175130603937 | Serbia |
| 2020 | 58.94217272 | 0.789183332686432 | 10.8243753125462 | 48.1177974056938 | Serbia |
| 2021 | 55.84429322 | 0.792416293685483 | 11.8403109005856 | 44.0039823183144 | Serbia |
| 1990 | 80.0971039 | 0.653853505137647 | 45.6444535612836 | 34.4526503418064 | Slovakia |
| 1991 | 82.2018601 | 0.658731402179317 | 44.9239230202313 | 37.2779370797887 | Slovakia |
| 1992 | 77.57290258 | 0.665830110587888 | 39.0984049774923 | 38.4744975975077 | Slovakia |
| 1993 | 70.42654577 | 0.67596793706605 | 38.0657558890215 | 32.3607898802985 | Slovakia |
| 1994 | 66.13842839 | 0.688136476595487 | 37.6513779878622 | 28.4870504043478 | Slovakia |
| 1995 | 62.14626233 | 0.697476536273994 | 35.4471909236565 | 26.6990714061235 | Slovakia |
| 1996 | 60.12776588 | 0.704405003157211 | 34.9900640084656 | 25.1377018715544 | Slovakia |
| 1997 | 58.44834103 | 0.711513209633452 | 32.6494904469295 | 25.7988505797705 | Slovakia |
| 1998 | 58.5887284 | 0.719058766219034 | 29.1859676837376 | 29.4027607148024 | Slovakia |
| 1999 | 56.73074007 | 0.726144172630556 | 26.460249548687 | 30.270490524433 | Slovakia |
| 2000 | 55.20546316 | 0.734046240313494 | 21.65257674 | 33.55288642 | Slovakia |
| 2001 | 49.39685346 | 0.741392553570632 | 20.2523564999565 | 29.1444969577935 | Slovakia |
| 2002 | 51.48570609 | 0.746791141582656 | 19.1959619901302 | 32.2897440959998 | Slovakia |
| 2003 | 55.96208285 | 0.75116726952438 | 19.7785242148712 | 36.1835586369288 | Slovakia |
| 2004 | 57.8958023 | 0.755836693705671 | 18.1325514267018 | 39.7632508738882 | Slovakia |
| 2005 | 58.93228749 | 0.761724185107018 | 14.2022340408035 | 44.7300534442565 | Slovakia |
| 2006 | 57.75508259 | 0.767673036224795 | 12.9054171825016 | 44.8496654099784 | Slovakia |
| 2007 | 58.26011628 | 0.772914152845047 | 12.8959898804855 | 45.3641263983445 | Slovakia |
| 2008 | 60.81784512 | 0.777197399879439 | 12.5187721954311 | 48.2990729243289 | Slovakia |
| 2009 | 64.89799194 | 0.781052260265285 | 11.8197871931663 | 53.0782047454437 | Slovakia |
| 2010 | 65.74053421 | 0.786254422327679 | 11.6688530157102 | 54.0716811905998 | Slovakia |
| 2011 | 64.58667701 | 0.791465382423228 | 10.9533064775267 | 53.6333705292533 | Slovakia |
| 2012 | 66.02299157 | 0.795456215611276 | 10.3471300400181 | 55.6758615280619 | Slovakia |
| 2013 | 66.46776468 | 0.797903722940591 | 9.9017319415754 | 56.5660327422746 | Slovakia |
| 2014 | 70.08317697 | 0.799618796308309 | 10.675016936747 | 59.408160028833 | Slovakia |
| 2015 | 75.71602808 | 0.800394179928449 | 9.91889742815583 | 65.7971306530542 | Slovakia |
| 2016 | 80.09035825 | 0.800878210489993 | 10.3097152755086 | 69.7806429720514 | Slovakia |
| 2017 | 82.24267269 | 0.802277316569847 | 10.3710025937092 | 71.8716700985908 | Slovakia |
| 2018 | 82.67084837 | 0.804357302109054 | 9.2678323607122 | 73.4030160071978 | Slovakia |
| 2019 | 78.53425814 | 0.80644845685688 | 9.96456 | 68.569698144566 | Slovakia |
| 2020 | 73.59550893 | 0.80830596588062 | 9.37602082721574 | 64.2194880980943 | Slovakia |
| 2021 | 69.90888811 | 0.810610529921693 | 9.10456621062167 | 60.8043218986483 | Slovakia |
| 1990 | 36.81381669 | 0.727463928368452 | 26.206945426218 | 10.606871261982 | Slovenia |
| 1991 | 35.45403399 | 0.732666985968602 | 20.1848667303831 | 15.2691672571669 | Slovenia |
| 1992 | 36.18513932 | 0.737391551733707 | 21.145089639655 | 15.040049680175 | Slovenia |
| 1993 | 32.64016821 | 0.741774171511168 | 19.02235056 | 13.61781765 | Slovenia |
| 1994 | 31.49049966 | 0.746234861332899 | 19.345702289264 | 12.144797367176 | Slovenia |
| 1995 | 26.26246096 | 0.750994974989173 | 19.6271777199052 | 6.63528323639476 | Slovenia |
| 1996 | 23.94123456 | 0.756348987126594 | 18.1398267473452 | 5.80140781382481 | Slovenia |
| 1997 | 23.93739738 | 0.762275291907702 | 16.8279774159715 | 7.10941995992852 | Slovenia |
| 1998 | 22.88366928 | 0.767728525502705 | 12.9367510325274 | 9.94691825126261 | Slovenia |
| 1999 | 19.91531646 | 0.773236192288015 | 12.0716566326048 | 7.84365982564524 | Slovenia |
| 2000 | 20.19574744 | 0.779578009745196 | 12.0391910389191 | 8.15655639619087 | Slovenia |
| 2001 | 19.21381919 | 0.786240775635761 | 11.8493439854613 | 7.3644752070487 | Slovenia |
| 2002 | 19.53578861 | 0.791805569443613 | 11.6672810207759 | 7.86850758698407 | Slovenia |
| 2003 | 19.01169853 | 0.796371530737719 | 11.3167786219109 | 7.69491990713913 | Slovenia |
| 2004 | 18.59769242 | 0.800962240122577 | 9.99593726337556 | 8.60175515550445 | Slovenia |
| 2005 | 18.60731559 | 0.805601248185604 | 9.509424114 | 9.09789148 | Slovenia |
| 2006 | 17.95315421 | 0.809708595222897 | 8.98866539225333 | 8.96448882249667 | Slovenia |
| 2007 | 18.08989229 | 0.813006319197598 | 10.45543574 | 7.634456557 | Slovenia |
| 2008 | 18.92385913 | 0.816347250277984 | 8.71476709635045 | 10.2090920336195 | Slovenia |
| 2009 | 21.07150083 | 0.818878560380979 | 8.001893644 | 13.06960719 | Slovenia |
| 2010 | 24.38226702 | 0.82117848598052 | 7.5989098049125 | 16.7833572119075 | Slovenia |
| 2011 | 28.96210793 | 0.823023560855118 | 7.500067048 | 21.46204088 | Slovenia |
| 2012 | 31.0371985 | 0.824336562677773 | 8.83425334465267 | 22.2029451579473 | Slovenia |
| 2013 | 34.27864017 | 0.825826275074906 | 7.68351336279133 | 26.5951268064787 | Slovenia |
| 2014 | 33.94865874 | 0.827799245827941 | 7.56749444950129 | 26.3811642858887 | Slovenia |
| 2015 | 35.50505393 | 0.829837070735453 | 6.74501562207346 | 28.7600383127565 | Slovenia |
| 2016 | 36.12656299 | 0.831367915132541 | 7.768722234 | 28.357840760334 | Slovenia |
| 2017 | 35.91987391 | 0.833038139449118 | 7.91969376392103 | 28.000180144049 | Slovenia |
| 2018 | 36.03154168 | 0.835472480610787 | 6.93803154702844 | 29.0935101326716 | Slovenia |
| 2019 | 38.92928849 | 0.838266782297842 | 6.43616915593937 | 32.4931193377906 | Slovenia |
| 2020 | 35.61463705 | 0.840373845574037 | 7.03399681341824 | 28.5806402356618 | Slovenia |
| 2021 | 34.20188499 | 0.842430731281465 | 6.82481753308943 | 27.3770674545606 | Slovenia |
| 1990 | 194.70279645291 | 0.622446576329452 | 61.8692254512876 | 132.833571001622 | Belarus |
| 1991 | 199.93302887639 | 0.627038266774523 | 59.3128522972808 | 140.620176579109 | Belarus |
| 1992 | 203.93742463319 | 0.632891737136784 | 54.6837718072227 | 149.253652825967 | Belarus |
| 1993 | 223.20242979335 | 0.639394169351269 | 52.0126622652192 | 171.189767528131 | Belarus |
| 1994 | 247.56406466329 | 0.64465278058669 | 50.9904786143395 | 196.57358604895 | Belarus |
| 1995 | 236.32428773597 | 0.648959503797064 | 47.7052831536134 | 188.619004582357 | Belarus |
| 1996 | 201.35081422798 | 0.652855220750827 | 45.8825579993515 | 155.468256228628 | Belarus |
| 1997 | 189.23173659719 | 0.655627296459504 | 45.7788701616424 | 143.452866435548 | Belarus |
| 1998 | 206.02979941052 | 0.657246466657203 | 45.4201863199735 | 160.609613090546 | Belarus |
| 1999 | 209.21307341555 | 0.660171009515746 | 43.4009721539177 | 165.812101261632 | Belarus |
| 2000 | 184.30178704899 | 0.664705822252137 | 40.642603093266 | 143.659183955724 | Belarus |
| 2001 | 165.8133806 | 0.671333268854148 | 38.6912806155734 | 127.122100012227 | Belarus |
| 2002 | 121.51929647355 | 0.678121782135605 | 37.5131519463769 | 84.0061445271731 | Belarus |
| 2003 | 105.61357288001 | 0.68486855613275 | 37.3633041545213 | 68.2502687254887 | Belarus |
| 2004 | 94.73924953 | 0.692502135029653 | 36.4822163028565 | 58.2570332282235 | Belarus |
| 2005 | 82.7186907 | 0.698985482904355 | 35.9976882343037 | 46.7210024631863 | Belarus |
| 2006 | 70.14203159 | 0.705119102674127 | 34.5976958785766 | 35.5443357140934 | Belarus |
| 2007 | 63.98570317 | 0.711496561875696 | 31.3672302400909 | 32.6184729341291 | Belarus |
| 2008 | 56.28804896 | 0.719120871487284 | 30.6664345719483 | 25.6216143840017 | Belarus |
| 2009 | 53.11658407 | 0.727292783613496 | 25.741400673168 | 27.375183392892 | Belarus |
| 2010 | 49.5345055 | 0.734954626211817 | 22.4148815728542 | 27.1196239230258 | Belarus |
| 2011 | 48.67942078 | 0.739504325946655 | 21.0793097989318 | 27.6001109796482 | Belarus |
| 2012 | 46.64049461 | 0.743468837307909 | 20.7093708276315 | 25.9311237782485 | Belarus |
| 2013 | 45.00312748 | 0.748529322885796 | 20.8088436971934 | 24.1942837841866 | Belarus |
| 2014 | 43.08031336 | 0.753511479851413 | 17.4526282146194 | 25.6276851481706 | Belarus |
| 2015 | 41.29822219 | 0.758205742923593 | 16.3244539638369 | 24.9737682272731 | Belarus |
| 2016 | 42.06104794 | 0.764328221802501 | 15.8719007065464 | 26.1891472371336 | Belarus |
| 2017 | 41.87246058 | 0.771131465670885 | 11.9835172315014 | 29.8889433523887 | Belarus |
| 2018 | 38.87316318 | 0.776568956673705 | 12.0517983377058 | 26.8213648412542 | Belarus |
| 2019 | 36.56271713 | 0.780508018535969 | 11.8793278431249 | 24.6833892911751 | Belarus |
| 2020 | 33.66649461 | 0.782381564892966 | 10.9232596154639 | 22.7432349984161 | Belarus |
| 2021 | 32.91703598 | 0.784484710906639 | 11.9354167380686 | 20.9816192456914 | Belarus |
| 1990 | 140.71435459357 | 0.674967632130919 | 37.9309433527616 | 102.783411240808 | Estonia |
| 1991 | 128.04974954076 | 0.685223089296634 | 37.0188723008677 | 91.0308772398923 | Estonia |
| 1992 | 130.7337038 | 0.695586673779285 | 36.7252356215686 | 94.0084681950314 | Estonia |
| 1993 | 134.71636011039 | 0.703693149995024 | 34.2138940836644 | 100.502466026726 | Estonia |
| 1994 | 136.01024695688 | 0.708456180899464 | 34.1151859385635 | 101.895061018316 | Estonia |
| 1995 | 130.27948248834 | 0.713172725189506 | 32.2416854342824 | 98.0377970540576 | Estonia |
| 1996 | 106.66423077836 | 0.718813024217038 | 29.1051396413694 | 77.5590911369905 | Estonia |
| 1997 | 99.87105358 | 0.724652439639785 | 25.9884078860363 | 73.8826456927237 | Estonia |
| 1998 | 100.69399923046 | 0.729027035230187 | 26.4221863533457 | 74.2718128771143 | Estonia |
| 1999 | 106.78377011823 | 0.733250616186121 | 22.012340916755 | 84.771429201475 | Estonia |
| 2000 | 93.44696974 | 0.739713154351944 | 20.0132451620064 | 73.4337245804836 | Estonia |
| 2001 | 82.39516102 | 0.746602207163017 | 21.1322538004944 | 61.2629072232856 | Estonia |
| 2002 | 69.37434875 | 0.752629898252246 | 17.973314065178 | 51.401034689232 | Estonia |
| 2003 | 65.75647019 | 0.75781567757793 | 16.7678933567232 | 48.9885768367468 | Estonia |
| 2004 | 58.86773352 | 0.763223727128029 | 15.4620518747544 | 43.4056816481456 | Estonia |
| 2005 | 54.74221885 | 0.769112181926314 | 14.480888298359 | 40.261330547251 | Estonia |
| 2006 | 49.88724805 | 0.774451841771662 | 13.0442657205545 | 36.8429823298855 | Estonia |
| 2007 | 48.24534783 | 0.781074442846485 | 11.946141009391 | 36.299206820089 | Estonia |
| 2008 | 46.605937 | 0.788598873704278 | 11.1767064479893 | 35.4292305486007 | Estonia |
| 2009 | 43.88164911 | 0.794728212699059 | 10.3123394640446 | 33.5693096438854 | Estonia |
| 2010 | 42.95839391 | 0.800829717976428 | 10.6243006145556 | 32.3340932958244 | Estonia |
| 2011 | 37.97948123 | 0.806633077522166 | 10.9333717605559 | 27.0461094675341 | Estonia |
| 2012 | 32.28496453 | 0.811109357704526 | 9.10938735574778 | 23.1755771719222 | Estonia |
| 2013 | 25.64775639 | 0.81488717677447 | 9.38576431866846 | 16.2619920725915 | Estonia |
| 2014 | 19.92519596 | 0.818770732051979 | 8.76560460477167 | 11.1595913566383 | Estonia |
| 2015 | 16.52034687 | 0.823221278492715 | 8.78917859944952 | 7.73116826609047 | Estonia |
| 2016 | 13.92791501 | 0.827364360092018 | 7.38254986641174 | 6.54536514240826 | Estonia |
| 2017 | 12.06998188 | 0.830975311283378 | 7.76047704140526 | 4.30950483477474 | Estonia |
| 2018 | 10.38858508 | 0.834566296193434 | 7.08004978106042 | 3.30853529488958 | Estonia |
| 2019 | 9.64926323 | 0.838573508963879 | 6.20486599905562 | 3.44439723080438 | Estonia |
| 2020 | 10.47372671 | 0.841817139147138 | 6.56657501145071 | 3.90715170198929 | Estonia |
| 2021 | 12.04512 | 0.844917787360624 | 5.74792167679091 | 6.29719832333909 | Estonia |
| 1990 | 147.11861885444 | 0.680193638285329 | 37.6044757433862 | 109.514143111054 | Latvia |
| 1991 | 160.08441616741 | 0.688117297290074 | 37.2537478997708 | 122.830668267639 | Latvia |
| 1992 | 168.38389219773 | 0.696157035504877 | 36.5478680627691 | 131.836024134961 | Latvia |
| 1993 | 178.79615675671 | 0.705249855159515 | 34.9485665183322 | 143.847590238378 | Latvia |
| 1994 | 182.37332295428 | 0.712531352821191 | 31.2047373382022 | 151.168585616078 | Latvia |
| 1995 | 172.80044029241 | 0.717639459211637 | 30.7959980395916 | 142.004442252818 | Latvia |
| 1996 | 137.95059234223 | 0.721139537892551 | 28.5621511217687 | 109.388441220461 | Latvia |
| 1997 | 121.37834876924 | 0.723746580428208 | 28.2833542168418 | 93.0949945523982 | Latvia |
| 1998 | 117.73454953483 | 0.725427335933284 | 25.8070271500498 | 91.9275223847802 | Latvia |
| 1999 | 115.1544815 | 0.727219684959525 | 25.365260258611 | 89.789221217889 | Latvia |
| 2000 | 100.90375587389 | 0.730951448728138 | 24.7072899497632 | 76.1964659241268 | Latvia |
| 2001 | 93.88573605 | 0.73629419637135 | 21.3457976723408 | 72.5399383823592 | Latvia |
| 2002 | 83.35393359 | 0.741870440285041 | 19.3923783845311 | 63.9615552014389 | Latvia |
| 2003 | 73.64382754 | 0.748168161846997 | 19.6295136027485 | 54.0143139337315 | Latvia |
| 2004 | 63.78392392 | 0.755263211009698 | 18.3268513324574 | 45.4570725900525 | Latvia |
| 2005 | 58.22821665 | 0.761929432669523 | 14.4822501110676 | 43.7459665343924 | Latvia |
| 2006 | 52.76114592 | 0.768459928850117 | 13.774441247946 | 38.986704668784 | Latvia |
| 2007 | 50.78324532 | 0.776481958669804 | 12.0088227074723 | 38.7744226172977 | Latvia |
| 2008 | 46.33482374 | 0.786324573303851 | 11.2393034488082 | 35.0955202905418 | Latvia |
| 2009 | 43.31279076 | 0.794504426648315 | 10.5649330620411 | 32.7478576959089 | Latvia |
| 2010 | 41.70719567 | 0.798171594063957 | 10.0153593963157 | 31.6918362761643 | Latvia |
| 2011 | 40.21953193 | 0.799080776427927 | 10.4199813725317 | 29.7995505597083 | Latvia |
| 2012 | 36.63184982 | 0.801026473960894 | 10.5987538552449 | 26.0330959611251 | Latvia |
| 2013 | 32.64571145 | 0.802660490277978 | 10.2395097679914 | 22.4062016794186 | Latvia |
| 2014 | 28.96334698 | 0.803721486035489 | 9.52422921745114 | 19.4391177606689 | Latvia |
| 2015 | 25.63804595 | 0.806985994927815 | 9.48614769430923 | 16.1518982557808 | Latvia |
| 2016 | 20.90438808 | 0.811463590660605 | 8.84143095551647 | 12.0629571282835 | Latvia |
| 2017 | 16.12905807 | 0.816923118079976 | 9.8346842793544 | 6.2943737876556 | Latvia |
| 2018 | 11.74814872 | 0.821419809272627 | 8.09670463977737 | 3.65144408152263 | Latvia |
| 2019 | 9.950284537 | 0.824781776742666 | 7.8859560080288 | 2.0643285286912 | Latvia |
| 2020 | 9.667386382 | 0.827900448409988 | 7.19373787043227 | 2.47364851192773 | Latvia |
| 2021 | 7.166056912 | 0.830663516299797 | 6.86192777391833 | 0.304129138031667 | Latvia |
| 1990 | 204.03860544216 | 0.668503937884641 | 39.3155398722611 | 164.723065569899 | Lithuania |
| 1991 | 250.69022095934 | 0.673234120792409 | 37.7872605670649 | 212.902960392275 | Lithuania |
| 1992 | 275.35735114441 | 0.680992879643758 | 37.4780524309577 | 237.879298713452 | Lithuania |
| 1993 | 257.2725413 | 0.687480621018131 | 37.28637683 | 219.9861644 | Lithuania |
| 1994 | 245.03412713296 | 0.69115165298429 | 36.6360030211403 | 208.39812411182 | Lithuania |
| 1995 | 215.52446823372 | 0.694734380082166 | 36.4920960968116 | 179.032372136908 | Lithuania |
| 1996 | 174.01332403719 | 0.698591300871233 | 35.9837779827514 | 138.029546054439 | Lithuania |
| 1997 | 156.3808887 | 0.702840949666947 | 36.1344440915641 | 120.246444610136 | Lithuania |
| 1998 | 147.75574326501 | 0.707468182196716 | 34.6621586588045 | 113.093584606206 | Lithuania |
| 1999 | 153.64993197867 | 0.712834170940662 | 32.5329895764315 | 121.116942402238 | Lithuania |
| 2000 | 134.95211488667 | 0.719896959838609 | 27.5038013608434 | 107.448313525827 | Lithuania |
| 2001 | 116.26167148737 | 0.727133584877313 | 27.6051133334472 | 88.6565581539228 | Lithuania |
| 2002 | 103.19436543082 | 0.733793397915696 | 22.073749619882 | 81.120615810938 | Lithuania |
| 2003 | 90.81444358 | 0.741229600875632 | 19.4763788036865 | 71.3380647774735 | Lithuania |
| 2004 | 85.42742803 | 0.748949762305515 | 19.0671754736633 | 66.3602525606467 | Lithuania |
| 2005 | 76.75438022 | 0.756352359983789 | 17.8544360675122 | 58.8999441553878 | Lithuania |
| 2006 | 70.88758925 | 0.763059124350272 | 15.1985478700418 | 55.6890413822382 | Lithuania |
| 2007 | 66.09157465 | 0.770229638520692 | 12.1840224172449 | 53.9075522359651 | Lithuania |
| 2008 | 63.34675191 | 0.778273711505288 | 11.8907164325612 | 51.4560354765788 | Lithuania |
| 2009 | 59.07276864 | 0.785689739125479 | 10.8730544977794 | 48.1997141416506 | Lithuania |
| 2010 | 56.54689343 | 0.793364681757828 | 10.7325011207866 | 45.8143923068034 | Lithuania |
| 2011 | 53.23984903 | 0.800924994333688 | 11.2316559387796 | 42.0081930884004 | Lithuania |
| 2012 | 49.56995183 | 0.8078270261595 | 9.24134016450635 | 40.3286116698836 | Lithuania |
| 2013 | 50.04893065 | 0.813219722329104 | 8.03276067718326 | 42.0161699699067 | Lithuania |
| 2014 | 51.45718635 | 0.817886376021861 | 9.2435128095738 | 42.2136735388362 | Lithuania |
| 2015 | 53.92267521 | 0.823112629019743 | 7.87834618811358 | 46.0443290186564 | Lithuania |
| 2016 | 53.83788947 | 0.829303695106582 | 7.19017356351075 | 46.6477159093292 | Lithuania |
| 2017 | 48.11125324 | 0.8358717398516 | 7.17488052273238 | 40.9363727165776 | Lithuania |
| 2018 | 44.66843815 | 0.841918910402675 | 6.55162411076351 | 38.1168140429365 | Lithuania |
| 2019 | 40.59121877 | 0.847906338850069 | 5.87610410046882 | 34.7151146699312 | Lithuania |
| 2020 | 35.30446382 | 0.85275238761541 | 6.24806036680409 | 29.0564034529659 | Lithuania |
| 2021 | 31.07314371 | 0.856484049109405 | 5.748922576 | 25.324221129028 | Lithuania |
| 1990 | 1270.61199730224 | 0.604251762377742 | 76.0690654087016 | 1194.54293189354 | Republic of Moldova |
| 1991 | 1058.51023568355 | 0.607270773865529 | 75.9679499228446 | 982.542285760705 | Republic of Moldova |
| 1992 | 1045.5790712724 | 0.609664558440288 | 68.2156499568557 | 977.363421315544 | Republic of Moldova |
| 1993 | 1104.57184076509 | 0.614152920432239 | 69.516230726787 | 1035.0556100383 | Republic of Moldova |
| 1994 | 1186.31993814466 | 0.617603766824611 | 60.9731534878881 | 1125.34678465677 | Republic of Moldova |
| 1995 | 1043.60405460004 | 0.620951321121127 | 63.11503479 | 980.48901981107 | Republic of Moldova |
| 1996 | 926.46285104271 | 0.623113983844076 | 61.6907044401241 | 864.772146602586 | Republic of Moldova |
| 1997 | 818.74717279374 | 0.624370104753213 | 59.424080140407 | 759.323092653333 | Republic of Moldova |
| 1998 | 740.42545963191 | 0.62411676120849 | 60.71916635 | 679.70629327759 | Republic of Moldova |
| 1999 | 696.00265692921 | 0.622834528688654 | 63.2141571780719 | 632.788499751138 | Republic of Moldova |
| 2000 | 638.99937002694 | 0.622004933502906 | 62.2400929675079 | 576.759277059432 | Republic of Moldova |
| 2001 | 599.39956283186 | 0.622936409943787 | 61.8362380019478 | 537.563324829912 | Republic of Moldova |
| 2002 | 532.42542889319 | 0.625874844526255 | 60.6494945840209 | 471.775934309169 | Republic of Moldova |
| 2003 | 487.12027403278 | 0.629985044767633 | 55.7873137678902 | 431.33296026489 | Republic of Moldova |
| 2004 | 428.69159692886 | 0.635394821992876 | 51.9873530762821 | 376.704243852578 | Republic of Moldova |
| 2005 | 363.55397865052 | 0.641530965095913 | 50.8306688046364 | 312.723309845884 | Republic of Moldova |
| 2006 | 297.51300604097 | 0.647996263561811 | 49.9246995200104 | 247.58830652096 | Republic of Moldova |
| 2007 | 270.82858941325 | 0.65421585059876 | 46.0569285811806 | 224.771660832069 | Republic of Moldova |
| 2008 | 259.54206965845 | 0.661046597063232 | 42.5315050348363 | 217.010564623614 | Republic of Moldova |
| 2009 | 253.4659079 | 0.666284393801024 | 39.1732396191918 | 214.292668284508 | Republic of Moldova |
| 2010 | 231.19163693842 | 0.671989615961323 | 38.3118840101019 | 192.879752928318 | Republic of Moldova |
| 2011 | 235.21779341671 | 0.678004136643775 | 37.5005471582773 | 197.717246258433 | Republic of Moldova |
| 2012 | 188.59267891531 | 0.682979795888934 | 37.2812203756519 | 151.311458539658 | Republic of Moldova |
| 2013 | 156.91559801142 | 0.688519174704066 | 37.3591329751593 | 119.556465036261 | Republic of Moldova |
| 2014 | 132.30673693887 | 0.694104557276994 | 36.6738802942526 | 95.6328566446174 | Republic of Moldova |
| 2015 | 129.85768549893 | 0.699355166217478 | 36.0123698754473 | 93.8453156234827 | Republic of Moldova |
| 2016 | 117.47098709132 | 0.70514454200476 | 34.5306851032336 | 82.9403019880864 | Republic of Moldova |
| 2017 | 118.49834640636 | 0.711206637139458 | 31.6534372968955 | 86.8449091094645 | Republic of Moldova |
| 2018 | 114.98352154532 | 0.717244426652362 | 31.1188581023517 | 83.8646634429683 | Republic of Moldova |
| 2019 | 104.64561932341 | 0.723011647628002 | 28.929271607364 | 75.716347716046 | Republic of Moldova |
| 2020 | 107.05889551942 | 0.727710540349217 | 24.5875437342286 | 82.4713517851914 | Republic of Moldova |
| 2021 | 99.88319476 | 0.732214875427507 | 23.7691351385113 | 76.1140596190887 | Republic of Moldova |
| 1990 | 308.81743499128 | 0.671600578021767 | 38.1920251831332 | 270.625409808147 | Russian Federation |
| 1991 | 297.77979611953 | 0.680099211484661 | 37.3201307107144 | 260.459665408816 | Russian Federation |
| 1992 | 289.39800156804 | 0.688068021982768 | 37.3346515509124 | 252.063350017128 | Russian Federation |
| 1993 | 324.94059460869 | 0.692036194318927 | 36.3538608947816 | 288.586733713908 | Russian Federation |
| 1994 | 327.57464176523 | 0.694144966076361 | 36.6422144938081 | 290.932427271422 | Russian Federation |
| 1995 | 326.67057526546 | 0.697854213747881 | 35.7682006024992 | 290.902374662961 | Russian Federation |
| 1996 | 290.77664299341 | 0.701468230734779 | 35.7918192392967 | 254.984823754113 | Russian Federation |
| 1997 | 276.66773237479 | 0.703752867289891 | 34.8419050442265 | 241.825827330563 | Russian Federation |
| 1998 | 280.74264250971 | 0.705198518088381 | 34.8953075510493 | 245.847334958661 | Russian Federation |
| 1999 | 311.48536165027 | 0.706863537909859 | 34.3926083057651 | 277.092753344505 | Russian Federation |
| 2000 | 286.03679693792 | 0.708643647769121 | 34.8177550295027 | 251.219041908417 | Russian Federation |
| 2001 | 265.36907334077 | 0.710570359001004 | 33.0694883408682 | 232.299584999902 | Russian Federation |
| 2002 | 227.54477527709 | 0.713460956547605 | 32.2525833885717 | 195.292191888518 | Russian Federation |
| 2003 | 205.0125534 | 0.718875116049301 | 29.222260691245 | 175.790292669155 | Russian Federation |
| 2004 | 172.50696060788 | 0.726305319497695 | 26.1438518938872 | 146.363108713993 | Russian Federation |
| 2005 | 151.75719532168 | 0.733592776524904 | 23.3212534049595 | 128.43594191672 | Russian Federation |
| 2006 | 130.27224993066 | 0.740064663588135 | 20.59436373 | 109.67788620263 | Russian Federation |
| 2007 | 119.09874729851 | 0.746191512265979 | 19.234803270019 | 99.863944028491 | Russian Federation |
| 2008 | 103.2037687 | 0.752623243282606 | 17.7183091255182 | 85.4854595668818 | Russian Federation |
| 2009 | 92.14299902 | 0.757960404689196 | 16.2173235610905 | 75.9256754561195 | Russian Federation |
| 2010 | 87.04981556 | 0.763320116187813 | 15.9482498881149 | 71.1015656683451 | Russian Federation |
| 2011 | 83.1553073 | 0.767633587927234 | 14.1385709201417 | 69.0167363832783 | Russian Federation |
| 2012 | 89.09158883 | 0.77192038427212 | 12.7302519543124 | 76.3613368717076 | Russian Federation |
| 2013 | 81.15324522 | 0.776526978961411 | 12.5780087295146 | 68.5752364948754 | Russian Federation |
| 2014 | 72.9804423 | 0.780617524284297 | 11.5763924806567 | 61.4040498234033 | Russian Federation |
| 2015 | 61.56493106 | 0.784913773964283 | 11.2550176361926 | 50.3099134191874 | Russian Federation |
| 2016 | 51.22631606 | 0.790330149439335 | 11.0491895392233 | 40.1771265209867 | Russian Federation |
| 2017 | 37.38132529 | 0.79566948446832 | 10.9142742299575 | 26.4670510588625 | Russian Federation |
| 2018 | 25.138246 | 0.800108144129611 | 10.4424898997321 | 14.6957560988179 | Russian Federation |
| 2019 | 19.85883875 | 0.803658201264112 | 9.36048611651087 | 10.4983526355891 | Russian Federation |
| 2020 | 19.50257105 | 0.806011474298182 | 9.7816747437595 | 9.7208963094805 | Russian Federation |
| 2021 | 18.96437625 | 0.808536004906566 | 9.361013522 | 9.603362726 | Russian Federation |
| 1990 | 231.14368390911 | 0.647461465528387 | 49.4779814396654 | 181.665702469445 | Ukraine |
| 1991 | 230.48832049567 | 0.651950021194653 | 45.30052924 | 185.18779125232 | Ukraine |
| 1992 | 249.02560477981 | 0.657600333165733 | 45.2691207081959 | 203.756484071614 | Ukraine |
| 1993 | 282.15568691429 | 0.66286740459174 | 40.3734104968056 | 241.782276417484 | Ukraine |
| 1994 | 375.16198315371 | 0.665506374866164 | 39.7123848347825 | 335.449598318928 | Ukraine |
| 1995 | 326.87986568806 | 0.666950166648749 | 39.0520299507439 | 287.827835737316 | Ukraine |
| 1996 | 291.10519928078 | 0.668104744367524 | 39.2564435540101 | 251.84875572677 | Ukraine |
| 1997 | 275.88507303595 | 0.669207845755283 | 38.4988204513473 | 237.386252584603 | Ukraine |
| 1998 | 231.11814641459 | 0.670322300548544 | 38.3458663122097 | 192.77228010238 | Ukraine |
| 1999 | 208.51258453528 | 0.670642905451183 | 38.6602425482823 | 169.852341986998 | Ukraine |
| 2000 | 169.44523247933 | 0.671095000943321 | 38.569710518735 | 130.875521960595 | Ukraine |
| 2001 | 143.63929434275 | 0.672467819796801 | 37.9694407865155 | 105.669853556235 | Ukraine |
| 2002 | 131.20134290608 | 0.674543220896083 | 37.7499695682217 | 93.4513733378583 | Ukraine |
| 2003 | 108.79641881919 | 0.678614636476308 | 37.6205015454988 | 71.1759172736912 | Ukraine |
| 2004 | 91.65151274 | 0.685380936146257 | 37.3484149065434 | 54.3030978315266 | Ukraine |
| 2005 | 83.4196406 | 0.692023300673985 | 36.7035977622856 | 46.7160428393144 | Ukraine |
| 2006 | 76.27674164 | 0.698093382536473 | 35.9082436427688 | 40.3684979952712 | Ukraine |
| 2007 | 68.67697476 | 0.704202307705725 | 34.955388883059 | 33.721585877931 | Ukraine |
| 2008 | 70.56071624 | 0.710980183909559 | 32.2401903801085 | 38.3205258629015 | Ukraine |
| 2009 | 59.62971249 | 0.716185465482395 | 32.2366945454505 | 27.3930179436995 | Ukraine |
| 2010 | 55.75932542 | 0.72101987022281 | 28.8021567260639 | 26.9571686940161 | Ukraine |
| 2011 | 53.59936591 | 0.724891957373533 | 26.3697664088428 | 27.2295995008772 | Ukraine |
| 2012 | 51.51095591 | 0.728817199844111 | 26.0657152842998 | 25.4452406218802 | Ukraine |
| 2013 | 50.23123305 | 0.734033572821072 | 21.0124494283106 | 29.2187836200494 | Ukraine |
| 2014 | 38.61102561 | 0.739883556959807 | 20.7159797300073 | 17.8950458773627 | Ukraine |
| 2015 | 42.3101914 | 0.743790863159826 | 19.0637584763975 | 23.2464329219125 | Ukraine |
| 2016 | 43.09514856 | 0.746857988843031 | 19.6944806862802 | 23.4006678763798 | Ukraine |
| 2017 | 41.34704761 | 0.750593324392773 | 19.2539516573497 | 22.0930959522103 | Ukraine |
| 2018 | 37.7742698 | 0.754451727274318 | 16.8179622996135 | 20.9563074974265 | Ukraine |
| 2019 | 36.14033807 | 0.757980081219807 | 15.4093594188263 | 20.7309786484937 | Ukraine |
| 2020 | 36.99278375 | 0.759929872218543 | 15.5753969035019 | 21.4173868480781 | Ukraine |
| 2021 | 37.41201597 | 0.760773912846782 | 17.4896403616246 | 19.9223756047754 | Ukraine |
| 1990 | 66.72186133 | 0.66608191698766 | 39.065683140595 | 27.656178189305 | Brunei Darussalam |
| 1991 | 66.73473618 | 0.671233831388631 | 38.3959792885165 | 28.3387568956935 | Brunei Darussalam |
| 1992 | 65.82923152 | 0.676702526223706 | 38.1609619452222 | 27.6682695725978 | Brunei Darussalam |
| 1993 | 64.81406283 | 0.682175439911044 | 37.4451966046305 | 27.3688662254895 | Brunei Darussalam |
| 1994 | 63.56094955 | 0.68739409348098 | 37.1341626046058 | 26.4267869473142 | Brunei Darussalam |
| 1995 | 61.82688572 | 0.692471156008533 | 36.5247397661805 | 25.3021459587995 | Brunei Darussalam |
| 1996 | 60.25442892 | 0.697602233240377 | 35.6764879725739 | 24.5779409435461 | Brunei Darussalam |
| 1997 | 58.98019439 | 0.702944386203433 | 35.6176176354972 | 23.3625767540528 | Brunei Darussalam |
| 1998 | 57.86417855 | 0.708630119469791 | 34.3542554787033 | 23.5099230731467 | Brunei Darussalam |
| 1999 | 57.19298279 | 0.714851237118356 | 31.9841237362165 | 25.2088590538935 | Brunei Darussalam |
| 2000 | 58.06372008 | 0.72155249267874 | 29.5031857519956 | 28.5605343291444 | Brunei Darussalam |
| 2001 | 58.26015553 | 0.728535069338112 | 26.7788717789177 | 31.4812837496923 | Brunei Darussalam |
| 2002 | 60.17648509 | 0.735343916434566 | 20.98428871 | 39.19219638 | Brunei Darussalam |
| 2003 | 62.02877195 | 0.741578091315293 | 19.9475993373621 | 42.0811726123179 | Brunei Darussalam |
| 2004 | 62.64852755 | 0.747311714682537 | 20.4800264454278 | 42.1685011084722 | Brunei Darussalam |
| 2005 | 63.55211009 | 0.752481792632817 | 18.6485144510164 | 44.9035956347136 | Brunei Darussalam |
| 2006 | 64.01735475 | 0.75731865511743 | 16.757841086514 | 47.259513668136 | Brunei Darussalam |
| 2007 | 65.07277346 | 0.761643128654341 | 16.5884597702667 | 48.4843136940833 | Brunei Darussalam |
| 2008 | 66.28698532 | 0.765714260433754 | 13.8933941765595 | 52.3935911407605 | Brunei Darussalam |
| 2009 | 67.2839451 | 0.769601681624648 | 13.9076867125353 | 53.3762583881447 | Brunei Darussalam |
| 2010 | 68.28955878 | 0.773219221249055 | 13.1134445548398 | 55.1761142293202 | Brunei Darussalam |
| 2011 | 70.3113129 | 0.776631893191597 | 13.6940888074864 | 56.6172240916136 | Brunei Darussalam |
| 2012 | 71.04839586 | 0.780216551684601 | 11.6522444767198 | 59.3961513801802 | Brunei Darussalam |
| 2013 | 70.68027607 | 0.784017040989581 | 11.4154366618717 | 59.2648394089383 | Brunei Darussalam |
| 2014 | 69.83134027 | 0.787733289361499 | 11.6996248889698 | 58.1317153765102 | Brunei Darussalam |
| 2015 | 68.89704862 | 0.791367566956237 | 10.6731439239658 | 58.2239046947842 | Brunei Darussalam |
| 2016 | 67.42337593 | 0.794980772397229 | 10.3976249323195 | 57.0257509970605 | Brunei Darussalam |
| 2017 | 66.56417307 | 0.798309936375838 | 10.6608850995467 | 55.9032879687133 | Brunei Darussalam |
| 2018 | 65.22368664 | 0.801436553226482 | 9.79621393284424 | 55.4274727066958 | Brunei Darussalam |
| 2019 | 63.91256508 | 0.804499388246421 | 10.0027924121528 | 53.9097726694872 | Brunei Darussalam |
| 2020 | 61.55429207 | 0.80742409075008 | 9.50277504694182 | 52.0515170183082 | Brunei Darussalam |
| 2021 | 58.7146099 | 0.810234366947283 | 10.0266066081087 | 48.6880032899513 | Brunei Darussalam |
| 1990 | 63.18600574 | 0.790253515528297 | 11.3832077993684 | 51.8027979412116 | Japan |
| 1991 | 64.11483655 | 0.794616189497547 | 11.5644719176195 | 52.5503646324005 | Japan |
| 1992 | 64.63777984 | 0.799156224748283 | 10.9963233911446 | 53.6414564497054 | Japan |
| 1993 | 64.86868618 | 0.802972994199584 | 10.4242514389419 | 54.4444347424881 | Japan |
| 1994 | 67.15595202 | 0.806689483098495 | 9.921940725 | 57.23401129 | Japan |
| 1995 | 66.39705562 | 0.810510771484257 | 8.776002976 | 57.621052646654 | Japan |
| 1996 | 65.45616391 | 0.813870745535327 | 9.11688224222083 | 56.3392816647192 | Japan |
| 1997 | 67.99522125 | 0.816675782376649 | 8.582158394 | 59.413062859795 | Japan |
| 1998 | 69.59688527 | 0.819016201504856 | 8.10348954042346 | 61.4933957271665 | Japan |
| 1999 | 67.17282548 | 0.82106663705133 | 8.04385036178342 | 59.1289751181066 | Japan |
| 2000 | 67.19554912 | 0.822938998623378 | 8.10705977879115 | 59.0884893391689 | Japan |
| 2001 | 67.66978452 | 0.824723241254759 | 7.64260128473405 | 60.027183236096 | Japan |
| 2002 | 70.24806988 | 0.82693677356819 | 7.94830270456875 | 62.2997671793913 | Japan |
| 2003 | 73.91095277 | 0.829392223273124 | 7.12635005149407 | 66.7846027153159 | Japan |
| 2004 | 76.48560282 | 0.832037381275171 | 7.41732473087489 | 69.0682780916351 | Japan |
| 2005 | 82.21045781 | 0.834288685362289 | 6.93633048726029 | 75.2741273270197 | Japan |
| 2006 | 86.68952856 | 0.836222264006662 | 7.78048645551377 | 78.9090421022062 | Japan |
| 2007 | 86.65068601 | 0.83829415979033 | 6.39242128281038 | 80.2582647253696 | Japan |
| 2008 | 84.05561505 | 0.840301217168455 | 6.72306549953677 | 77.3325495549732 | Japan |
| 2009 | 79.78136294 | 0.842133664144895 | 5.7965229834642 | 73.9848399596558 | Japan |
| 2010 | 80.60303387 | 0.844361810771781 | 6.51385517494717 | 74.0891786935928 | Japan |
| 2011 | 80.4670275 | 0.846750266822805 | 6.39506427922028 | 74.0719632254997 | Japan |
| 2012 | 81.48802984 | 0.849061489406189 | 5.80804760167309 | 75.6799822368469 | Japan |
| 2013 | 76.86773753 | 0.851356993626047 | 6.19890248639723 | 70.6688350401428 | Japan |
| 2014 | 72.55138036 | 0.85369274935716 | 6.42635236309426 | 66.1250279944957 | Japan |
| 2015 | 70.3585926 | 0.856097498738686 | 6.18305044399774 | 64.1755421557223 | Japan |
| 2016 | 68.64512037 | 0.858698173210648 | 5.97726226228708 | 62.6678581031429 | Japan |
| 2017 | 65.43114819 | 0.861393308858287 | 5.61309209109958 | 59.8180561034204 | Japan |
| 2018 | 63.28588389 | 0.864237136659234 | 5.84311395907619 | 57.4427699331738 | Japan |
| 2019 | 59.05339837 | 0.867148183486851 | 6.34000020732778 | 52.7133981621522 | Japan |
| 2020 | 55.99971206 | 0.869106879336459 | 6.03142289903754 | 49.9682891586925 | Japan |
| 2021 | 57.71180749 | 0.871241812572128 | 6.1870292089825 | 51.5247782852675 | Japan |
| 1990 | 160.78334640424 | 0.692329306595283 | 36.4032484830496 | 124.38009792119 | Republic of Korea |
| 1991 | 149.95714661502 | 0.702631128494537 | 35.7913419618226 | 114.165804653197 | Republic of Korea |
| 1992 | 141.34745435197 | 0.712440841630159 | 31.8690172820111 | 109.478437069959 | Republic of Korea |
| 1993 | 131.53363243078 | 0.722286739631754 | 28.8278480184044 | 102.705784412376 | Republic of Korea |
| 1994 | 122.79468969031 | 0.732197906155207 | 24.5559630688059 | 98.2387266215041 | Republic of Korea |
| 1995 | 114.48886486906 | 0.741940029590316 | 19.7447141550975 | 94.7441507139625 | Republic of Korea |
| 1996 | 108.34614576864 | 0.751553240476733 | 19.9187060939369 | 88.4274396747031 | Republic of Korea |
| 1997 | 103.42155157726 | 0.760591683388116 | 15.86433455 | 87.55721703 | Republic of Korea |
| 1998 | 99.41130309 | 0.76828188532262 | 13.5280010923375 | 85.8833019942426 | Republic of Korea |
| 1999 | 95.32969216 | 0.776773436337718 | 12.359447955478 | 82.970244202912 | Republic of Korea |
| 2000 | 96.12169127 | 0.785428821409438 | 11.2588844221466 | 84.8628068459034 | Republic of Korea |
| 2001 | 95.74720683 | 0.793496280690955 | 10.9882558884539 | 84.7589509430161 | Republic of Korea |
| 2002 | 93.50444992 | 0.801024295470106 | 10.4704305734952 | 83.0340193459348 | Republic of Korea |
| 2003 | 92.21235048 | 0.807687785876648 | 9.9278275707738 | 82.2845229123262 | Republic of Korea |
| 2004 | 90.14169066 | 0.813720926701095 | 9.80775068282945 | 80.3339399792605 | Republic of Korea |
| 2005 | 87.33873403 | 0.819093399889013 | 8.69716824695837 | 78.6415657798316 | Republic of Korea |
| 2006 | 87.62395267 | 0.824179820431794 | 8.34448175720828 | 79.2794709091817 | Republic of Korea |
| 2007 | 88.29825837 | 0.82912795326169 | 7.04762403517113 | 81.2506343364789 | Republic of Korea |
| 2008 | 86.76088819 | 0.833894626872214 | 7.8237150306125 | 78.9371731563275 | Republic of Korea |
| 2009 | 84.02752737 | 0.838108330520764 | 5.95251460765946 | 78.0750127650405 | Republic of Korea |
| 2010 | 82.12250978 | 0.84251142929369 | 6.47836437527279 | 75.6441454045072 | Republic of Korea |
| 2011 | 77.49253369 | 0.846913228744834 | 6.54973104546674 | 70.9428026485533 | Republic of Korea |
| 2012 | 71.50402113 | 0.851140262773848 | 5.92136283780708 | 65.5826582941329 | Republic of Korea |
| 2013 | 64.75829791 | 0.855441000170575 | 5.81862279555089 | 58.9396751166891 | Republic of Korea |
| 2014 | 59.34882716 | 0.859665567500037 | 5.9215926672002 | 53.4272344880898 | Republic of Korea |
| 2015 | 56.14664093 | 0.863907408481354 | 6.41551554155726 | 49.7311253931527 | Republic of Korea |
| 2016 | 55.18465463 | 0.868317824083966 | 5.73202028753026 | 49.4526343411897 | Republic of Korea |
| 2017 | 54.55776531 | 0.87261776393019 | 6.489769036 | 48.067996274055 | Republic of Korea |
| 2018 | 55.1102974 | 0.876823316849569 | 5.93580866463382 | 49.1744887367262 | Republic of Korea |
| 2019 | 56.83842429 | 0.88073150966869 | 5.69980339569907 | 51.1386208915609 | Republic of Korea |
| 2020 | 53.51971554 | 0.883717569055359 | 5.72621671800137 | 47.7934988223986 | Republic of Korea |
| 2021 | 47.58814739 | 0.886675266756997 | 5.9597139412069 | 41.6284334464631 | Republic of Korea |
| 1990 | 124.37025593315 | 0.686404444349548 | 37.2075653036883 | 87.1626906294617 | Singapore |
| 1991 | 96.5267244 | 0.695055557646282 | 36.4835393175784 | 60.0431850777316 | Singapore |
| 1992 | 79.07538251 | 0.703427834229016 | 34.6000674798866 | 44.4753150276634 | Singapore |
| 1993 | 68.23910057 | 0.712315203759158 | 31.7562521619438 | 36.4828484060262 | Singapore |
| 1994 | 65.97816903 | 0.721256796938456 | 28.8479911944757 | 37.1301778318443 | Singapore |
| 1995 | 56.71804334 | 0.729982231248165 | 23.7370334120087 | 32.9810099261513 | Singapore |
| 1996 | 50.89781782 | 0.739028234741754 | 19.6743921289596 | 31.2234256917704 | Singapore |
| 1997 | 49.83335695 | 0.747571897605582 | 19.1154934495042 | 30.7178634981758 | Singapore |
| 1998 | 61.34970697 | 0.754873203577067 | 16.814037471462 | 44.535669495748 | Singapore |
| 1999 | 44.67266581 | 0.760831136110222 | 16.6472410296785 | 28.0254247842615 | Singapore |
| 2000 | 44.84016302 | 0.767717370394546 | 14.3847393941451 | 30.4554236261349 | Singapore |
| 2001 | 37.14303455 | 0.774294656621655 | 13.5840679431632 | 23.5589666095068 | Singapore |
| 2002 | 35.77036228 | 0.77973576984914 | 12.8024854772447 | 22.9678768069053 | Singapore |
| 2003 | 33.36329752 | 0.784165555316375 | 10.7091475450279 | 22.6541499719921 | Singapore |
| 2004 | 32.00614057 | 0.789852773877056 | 10.2651912586311 | 21.7409493131889 | Singapore |
| 2005 | 34.35916403 | 0.796455750753104 | 10.5929112197267 | 23.7662528117133 | Singapore |
| 2006 | 39.46159348 | 0.803342694397764 | 9.92868258914445 | 29.5329108896056 | Singapore |
| 2007 | 40.65777414 | 0.810625304101189 | 9.89018365660404 | 30.767590480986 | Singapore |
| 2008 | 42.69801929 | 0.818124162030734 | 8.57730412914439 | 34.1207151641456 | Singapore |
| 2009 | 45.79925345 | 0.824251515940882 | 8.54900404704455 | 37.2502493994355 | Singapore |
| 2010 | 46.86124902 | 0.830222159553543 | 8.00621932441741 | 38.8550296999826 | Singapore |
| 2011 | 45.75941824 | 0.833829607920547 | 7.00831733996633 | 38.7511009036537 | Singapore |
| 2012 | 44.87592585 | 0.837391806757054 | 6.88722933843488 | 37.9886965084951 | Singapore |
| 2013 | 40.90198223 | 0.840420177683154 | 6.94114533185118 | 33.9608368987188 | Singapore |
| 2014 | 34.31672638 | 0.842894537933952 | 6.79407211538485 | 27.5226542689452 | Singapore |
| 2015 | 33.19206668 | 0.84540185703118 | 6.53442861772269 | 26.6576380593973 | Singapore |
| 2016 | 33.93502612 | 0.847596998735509 | 6.34144543671286 | 27.5935806801771 | Singapore |
| 2017 | 30.24362881 | 0.849051517606326 | 5.90106883934261 | 24.3425599676374 | Singapore |
| 2018 | 30.70975887 | 0.850762728725121 | 5.888404391 | 24.821354475953 | Singapore |
| 2019 | 29.01612633 | 0.852497337757222 | 6.04525452425758 | 22.9708718084424 | Singapore |
| 2020 | 25.38870633 | 0.854296009592945 | 5.88565851985875 | 19.5030478139412 | Singapore |
| 2021 | 26.6530098 | 0.856097766112729 | 6.79514502379846 | 19.8578647802315 | Singapore |
| 1990 | 29.76804174 | 0.725982519157417 | 24.2918779087148 | 5.47616383623516 | Australia |
| 1991 | 25.39731273 | 0.729759858781312 | 22.56022972 | 2.837083009 | Australia |
| 1992 | 21.96668553 | 0.73390140434283 | 19.6298936342845 | 2.33679189180553 | Australia |
| 1993 | 17.35193724 | 0.738374743085692 | 17.0464705703085 | 0.305466673891498 | Australia |
| 1994 | 15.60014555 | 0.742846571880912 | 15.493400748015 | 0.106744797855001 | Australia |
| 1995 | 14.26956479 | 0.747426824081883 | 14.26956479 | 0 | Australia |
| 1996 | 13.22559616 | 0.751991736090285 | 13.22559616 | 0 | Australia |
| 1997 | 10.6306612 | 0.756633901347637 | 10.6306612 | 0 | Australia |
| 1998 | 8.624051363 | 0.761133265356106 | 8.624051363 | 0 | Australia |
| 1999 | 9.625775104 | 0.765697505930953 | 9.425430356 | 0.200344748 | Australia |
| 2000 | 9.028360251 | 0.770223843075912 | 8.92196317496684 | 0.106397075663157 | Australia |
| 2001 | 9.64250794 | 0.774662787384155 | 9.45353942004154 | 0.188968519738461 | Australia |
| 2002 | 10.67793188 | 0.779360940006122 | 10.1265965226805 | 0.551335356279525 | Australia |
| 2003 | 12.16548168 | 0.783600180802991 | 10.8842923679015 | 1.28118930851846 | Australia |
| 2004 | 13.91456542 | 0.787016543130049 | 10.2792429387162 | 3.63532248264375 | Australia |
| 2005 | 14.30646851 | 0.789615521287164 | 10.8824279437037 | 3.42404056637625 | Australia |
| 2006 | 14.56516247 | 0.791155687742942 | 10.196660812533 | 4.368501662 | Australia |
| 2007 | 12.65485017 | 0.792532682754436 | 10.8754224924724 | 1.77942768126762 | Australia |
| 2008 | 9.353549813 | 0.795292900277512 | 9.17965227127857 | 0.173897542031428 | Australia |
| 2009 | 8.065133462 | 0.799174373217167 | 8.065133462 | 0 | Australia |
| 2010 | 6.200207316 | 0.803561543809643 | 6.200207316 | 0 | Australia |
| 2011 | 6.30713168 | 0.807520219913819 | 6.29068177786385 | 0.0164499022261539 | Australia |
| 2012 | 5.952442721 | 0.811644461488936 | 5.952442721 | 0 | Australia |
| 2013 | 5.142202214 | 0.816453661448137 | 5.142202214 | 0 | Australia |
| 2014 | 5.034740616 | 0.820840850321621 | 5.034740616 | 0 | Australia |
| 2015 | 4.544841067 | 0.824860715173108 | 4.544841067 | 0 | Australia |
| 2016 | 3.866454239 | 0.828559943182664 | 3.866454239 | 0 | Australia |
| 2017 | 3.50532198 | 0.831798265550707 | 3.50532198 | 0 | Australia |
| 2018 | 3.113191897 | 0.835354624190932 | 3.113191897 | 0 | Australia |
| 2019 | 3.026802097 | 0.839317426059217 | 3.026802097 | 0 | Australia |
| 2020 | 3.790799997 | 0.842051314482691 | 3.52361198408444 | 0.267188013235556 | Australia |
| 2021 | 2.183371498 | 0.844252813727575 | 2.183371498 | 0 | Australia |
| 1990 | 60.60392327 | 0.752321654884833 | 17.2405397089022 | 43.3633835624278 | New Zealand |
| 1991 | 50.05412825 | 0.755616090554557 | 17.7024095226525 | 32.3517187298575 | New Zealand |
| 1992 | 39.95801948 | 0.759461372351944 | 17.5051960157321 | 22.4528234679579 | New Zealand |
| 1993 | 37.42381447 | 0.763634604405382 | 15.2729108272261 | 22.1509036432239 | New Zealand |
| 1994 | 37.79129258 | 0.767118657876439 | 13.1193709451697 | 24.6719216341503 | New Zealand |
| 1995 | 36.21061867 | 0.769998036424639 | 14.3130165537069 | 21.8976021115731 | New Zealand |
| 1996 | 36.79937861 | 0.773575283251135 | 12.2353592746853 | 24.5640193355947 | New Zealand |
| 1997 | 40.84472103 | 0.778205776484177 | 12.2989149530612 | 28.5458060776388 | New Zealand |
| 1998 | 33.17739689 | 0.78173075719617 | 13.1009350170981 | 20.0764618714419 | New Zealand |
| 1999 | 36.00331401 | 0.784428637621383 | 11.4104676691074 | 24.5928463379726 | New Zealand |
| 2000 | 40.26913674 | 0.787659557491679 | 11.6541612715919 | 28.6149754656781 | New Zealand |
| 2001 | 35.46420021 | 0.791426079291696 | 10.8756152872327 | 24.5885849203273 | New Zealand |
| 2002 | 33.7397104 | 0.794519590986489 | 11.2626676430056 | 22.4770427608944 | New Zealand |
| 2003 | 30.74768498 | 0.796286883438684 | 11.7986370087506 | 18.9490479678394 | New Zealand |
| 2004 | 32.07817498 | 0.798177476508371 | 9.7125498571384 | 22.3656251214716 | New Zealand |
| 2005 | 33.49268209 | 0.799107294338124 | 10.6690552539662 | 22.8236268328038 | New Zealand |
| 2006 | 39.93402848 | 0.798456865675295 | 9.94075612713029 | 29.9932723554097 | New Zealand |
| 2007 | 56.35153353 | 0.798447615942533 | 11.3158444893888 | 45.0356890455513 | New Zealand |
| 2008 | 66.51710293 | 0.799669513648105 | 9.93735089405432 | 56.5797520401457 | New Zealand |
| 2009 | 76.68323489 | 0.801727534577573 | 10.814454948094 | 65.868779939126 | New Zealand |
| 2010 | 72.1520757 | 0.805133184751295 | 9.55242110636182 | 62.5996545922682 | New Zealand |
| 2011 | 68.64974864 | 0.809760351959867 | 9.42481776594647 | 59.2249308780635 | New Zealand |
| 2012 | 60.41927174 | 0.814957563823326 | 9.31502849997204 | 51.104243241748 | New Zealand |
| 2013 | 44.73581268 | 0.820145989650422 | 7.57463829816415 | 37.1611743838359 | New Zealand |
| 2014 | 40.67119349 | 0.823908943687562 | 7.77824827709578 | 32.8929452171042 | New Zealand |
| 2015 | 27.89115022 | 0.82746249334817 | 8.32952985246545 | 19.5616203640045 | New Zealand |
| 2016 | 21.18037152 | 0.831843966678668 | 7.50909459477083 | 13.6712769206692 | New Zealand |
| 2017 | 20.18686872 | 0.836301794613403 | 6.61171499096965 | 13.5751537321003 | New Zealand |
| 2018 | 16.09327907 | 0.840597863184845 | 7.01487877603261 | 9.07840028960739 | New Zealand |
| 2019 | 16.17064719 | 0.844533208807291 | 6.70261181975652 | 9.46803536579348 | New Zealand |
| 2020 | 14.58817925 | 0.847398736836909 | 5.219834789 | 9.368344461 | New Zealand |
| 2021 | 12.88797601 | 0.849442498970719 | 5.56678259902471 | 7.32119341166529 | New Zealand |
| 1990 | 50.93088011 | 0.761463880346533 | 15.480786073419 | 35.450094038731 | Andorra |
| 1991 | 52.37543499 | 0.764709071138693 | 15.5416263395422 | 36.8338086525178 | Andorra |
| 1992 | 54.37862971 | 0.767188282487657 | 13.127310245406 | 41.251319460654 | Andorra |
| 1993 | 55.23628197 | 0.768953827567811 | 14.4109161181456 | 40.8253658513044 | Andorra |
| 1994 | 56.52027985 | 0.77002376454004 | 14.8449181085398 | 41.6753617431402 | Andorra |
| 1995 | 58.10419984 | 0.77098868338163 | 12.6371527314711 | 45.4670471046889 | Andorra |
| 1996 | 59.51530992 | 0.772403470644367 | 13.493763404619 | 46.021546519171 | Andorra |
| 1997 | 61.06526183 | 0.774580970737089 | 14.4728270768727 | 46.5924347495373 | Andorra |
| 1998 | 62.82498166 | 0.776972577800595 | 12.5509238522184 | 50.2740578077716 | Andorra |
| 1999 | 64.33290095 | 0.779629634395052 | 11.8172754515715 | 52.5156254980285 | Andorra |
| 2000 | 65.72541717 | 0.781976505693047 | 12.2422826989557 | 53.4831344744743 | Andorra |
| 2001 | 66.85441388 | 0.785417034980389 | 11.2255879365232 | 55.6288259395868 | Andorra |
| 2002 | 67.75074627 | 0.79015875913382 | 11.9496797464557 | 55.8010665218143 | Andorra |
| 2003 | 69.07565443 | 0.797278900724701 | 10.7030874383041 | 58.3725669959959 | Andorra |
| 2004 | 70.27675254 | 0.80473197995372 | 9.84540111558291 | 60.4313514218871 | Andorra |
| 2005 | 71.27962201 | 0.812213979716634 | 9.04054651944723 | 62.2390754872528 | Andorra |
| 2006 | 72.00562271 | 0.819787259850839 | 9.01680437977583 | 62.9888183305542 | Andorra |
| 2007 | 72.78720119 | 0.826617254254178 | 7.97248811715206 | 64.8147130722779 | Andorra |
| 2008 | 73.1415861 | 0.83182171450476 | 6.85489968485462 | 66.2866864137654 | Andorra |
| 2009 | 73.86405598 | 0.835929886554712 | 6.90088283610966 | 66.9631731461503 | Andorra |
| 2010 | 73.98291282 | 0.839489966459192 | 7.21596425188744 | 66.7669485648126 | Andorra |
| 2011 | 74.01172553 | 0.842794802354863 | 6.67677836138864 | 67.3349471660114 | Andorra |
| 2012 | 73.83807632 | 0.845810830539451 | 6.3292440642686 | 67.5088322596914 | Andorra |
| 2013 | 73.4272345 | 0.848329909768827 | 5.54216602267765 | 67.8850684733024 | Andorra |
| 2014 | 72.74313358 | 0.851010491664786 | 6.180213127 | 66.56292045 | Andorra |
| 2015 | 71.72947766 | 0.853591217830289 | 6.23916127989184 | 65.4903163783082 | Andorra |
| 2016 | 68.51999309 | 0.856257052608717 | 5.58624943239275 | 62.9337436566473 | Andorra |
| 2017 | 61.97044061 | 0.85947703010266 | 5.63585307621854 | 56.3345875298815 | Andorra |
| 2018 | 55.18242424 | 0.862588950920789 | 6.20840704426919 | 48.9740171947808 | Andorra |
| 2019 | 51.35480078 | 0.865621023596139 | 5.56073855966756 | 45.7940622226824 | Andorra |
| 2020 | 50.16608779 | 0.867449169102346 | 5.86615568609782 | 44.2999321068822 | Andorra |
| 2021 | 48.65172728 | 0.869444112735696 | 6.65990682618394 | 41.9918204549561 | Andorra |
| 1990 | 103.42487375595 | 0.749853692572025 | 19.0361613790408 | 84.3887123769092 | Austria |
| 1991 | 105.48537938663 | 0.751652734176599 | 19.7015796905786 | 85.7837996960514 | Austria |
| 1992 | 107.62110807977 | 0.754375285612199 | 19.0235493682718 | 88.5975587114982 | Austria |
| 1993 | 109.60878279068 | 0.758804553724473 | 15.8757434869851 | 93.7330393036949 | Austria |
| 1994 | 112.61309881816 | 0.764704364300466 | 14.7375468984239 | 97.875551919736 | Austria |
| 1995 | 113.16265382775 | 0.769827471008921 | 14.2368161550617 | 98.9258376726883 | Austria |
| 1996 | 114.08313257884 | 0.774446565189292 | 13.4800569889574 | 100.603075589883 | Austria |
| 1997 | 114.70028871195 | 0.77923285637593 | 12.025490771055 | 102.674797940895 | Austria |
| 1998 | 116.45272189247 | 0.783521015501574 | 12.1202047932191 | 104.332517099251 | Austria |
| 1999 | 118.1152649 | 0.787451584183882 | 11.5858457986669 | 106.529419069933 | Austria |
| 2000 | 122.28442523936 | 0.791847679870807 | 11.05723729 | 111.227188 | Austria |
| 2001 | 125.58260790272 | 0.795770117733764 | 10.5232210409531 | 115.059386861767 | Austria |
| 2002 | 129.68678503715 | 0.799154119574311 | 10.9420538827685 | 118.744731154381 | Austria |
| 2003 | 136.13453481713 | 0.802043253134005 | 10.4996127904119 | 125.634922026718 | Austria |
| 2004 | 140.23296972325 | 0.804889143740108 | 9.65322798196065 | 130.579741741289 | Austria |
| 2005 | 143.39081652807 | 0.808419194340084 | 9.37841507682655 | 134.012401451243 | Austria |
| 2006 | 135.72883176388 | 0.812064609467116 | 8.86074908055561 | 126.868082683324 | Austria |
| 2007 | 115.18743553869 | 0.815480521576858 | 9.16052814396365 | 106.026907394726 | Austria |
| 2008 | 89.15906517 | 0.818793658319811 | 7.946315098 | 81.21275007 | Austria |
| 2009 | 65.86376324 | 0.821541167154033 | 8.03192091777674 | 57.8318423204333 | Austria |
| 2010 | 50.90378399 | 0.824500109333185 | 7.86021590112222 | 43.0435680879878 | Austria |
| 2011 | 39.79808751 | 0.82800199897916 | 7.61617158610848 | 32.1819159194615 | Austria |
| 2012 | 31.01046808 | 0.83111330933878 | 7.78466507402355 | 23.2258030046365 | Austria |
| 2013 | 24.19676933 | 0.833679780262374 | 7.89900057099522 | 16.2977687592448 | Austria |
| 2014 | 19.64614496 | 0.835838577321076 | 7.76602267386042 | 11.8801222908296 | Austria |
| 2015 | 17.56408019 | 0.837579895310124 | 7.64117350144667 | 9.92290668783333 | Austria |
| 2016 | 16.51520792 | 0.839883789894186 | 6.617043508 | 9.898164409 | Austria |
| 2017 | 15.8855283 | 0.843173330746292 | 6.55613509906444 | 9.32939319836555 | Austria |
| 2018 | 14.88779717 | 0.846614470307794 | 6.78904668515526 | 8.09875048730474 | Austria |
| 2019 | 14.60997162 | 0.849862514846872 | 5.56425298514273 | 9.04571863054727 | Austria |
| 2020 | 18.23135557 | 0.852020385174284 | 5.05958177564125 | 13.1717737894587 | Austria |
| 2021 | 13.41833531 | 0.853837004256539 | 6.36393316035833 | 7.05440214847167 | Austria |
| 1990 | 56.36571595 | 0.737390656400179 | 22.2383331491102 | 34.1273827982998 | Belgium |
| 1991 | 60.00653005 | 0.74217307419685 | 22.1831390247574 | 37.8233910292026 | Belgium |
| 1992 | 63.00658628 | 0.748012299186744 | 18.5953911518512 | 44.4111951284788 | Belgium |
| 1993 | 60.9779444 | 0.753685087006788 | 18.5537835134005 | 42.4241608835195 | Belgium |
| 1994 | 58.57034072 | 0.758903637592037 | 16.1936380637645 | 42.3767026559355 | Belgium |
| 1995 | 54.55619062 | 0.763170400784407 | 15.0243402596836 | 39.5318503622164 | Belgium |
| 1996 | 51.50257652 | 0.766826075213302 | 13.3232042791787 | 38.1793722368112 | Belgium |
| 1997 | 51.83021128 | 0.770813953023256 | 12.90160111 | 38.92861017 | Belgium |
| 1998 | 54.20120374 | 0.774228267511882 | 12.4993551647723 | 41.7018485719577 | Belgium |
| 1999 | 56.14711179 | 0.777041040124219 | 11.8848879302653 | 44.2622238618647 | Belgium |
| 2000 | 61.37209448 | 0.780396331183289 | 11.3416908284529 | 50.0304036524771 | Belgium |
| 2001 | 63.99845668 | 0.784713043367342 | 11.7974798707402 | 52.2009768122398 | Belgium |
| 2002 | 66.59953837 | 0.788836054052762 | 11.371576493985 | 55.227961875335 | Belgium |
| 2003 | 70.85986891 | 0.792014383577294 | 10.681394049762 | 60.178474857938 | Belgium |
| 2004 | 73.33110854 | 0.795224935706338 | 11.2366072427673 | 62.0945012980127 | Belgium |
| 2005 | 81.65594247 | 0.798497235786438 | 10.5411278400102 | 71.1148146337698 | Belgium |
| 2006 | 86.12410215 | 0.801572762913827 | 10.3282570161134 | 75.7958451378566 | Belgium |
| 2007 | 80.53535988 | 0.804612664215848 | 9.5025838484549 | 71.0327760346651 | Belgium |
| 2008 | 63.80208122 | 0.807757944740662 | 10.0610186067632 | 53.7410626103268 | Belgium |
| 2009 | 49.34344055 | 0.811055825478368 | 9.10943444651679 | 40.2340061019032 | Belgium |
| 2010 | 40.63442786 | 0.815030206751301 | 9.0036982997252 | 31.6307295624948 | Belgium |
| 2011 | 33.18946527 | 0.819470362048624 | 8.63980451419548 | 24.5496607512445 | Belgium |
| 2012 | 31.0019565 | 0.824226016040103 | 8.99927709630148 | 22.0026794021685 | Belgium |
| 2013 | 28.35021949 | 0.828619831627193 | 7.2106720027864 | 21.1395474910636 | Belgium |
| 2014 | 25.50037731 | 0.832425051502658 | 7.05413051501111 | 18.4462467989689 | Belgium |
| 2015 | 23.71395171 | 0.835878400447772 | 6.39411640083087 | 17.3198353126291 | Belgium |
| 2016 | 21.90268992 | 0.839462940488534 | 6.65116753389609 | 15.2515223878039 | Belgium |
| 2017 | 22.45049565 | 0.843212044031047 | 6.66770923412391 | 15.7827864202161 | Belgium |
| 2018 | 21.93262579 | 0.846617625234845 | 6.579009264 | 15.353616527914 | Belgium |
| 2019 | 20.67549981 | 0.849498394807879 | 6.4394091538871 | 14.2360906590429 | Belgium |
| 2020 | 25.00914743 | 0.851346648590241 | 6.76950491912444 | 18.2396425077456 | Belgium |
| 2021 | 27.20639897 | 0.853654015777112 | 6.83732905696097 | 20.369069912189 | Belgium |
| 1990 | 214.51342639369 | 0.648230871689669 | 48.2202142545989 | 166.293212139091 | Cyprus |
| 1991 | 208.04703456395 | 0.655726169264618 | 45.823958666697 | 162.223075897253 | Cyprus |
| 1992 | 198.83661329568 | 0.666011849072818 | 39.457873536547 | 159.378739759133 | Cyprus |
| 1993 | 187.85839252755 | 0.677547476543954 | 37.7563692645723 | 150.102023262978 | Cyprus |
| 1994 | 178.07799927991 | 0.688829360602013 | 37.1672589573945 | 140.910740322515 | Cyprus |
| 1995 | 169.56474423165 | 0.699923898437876 | 35.9019397998179 | 133.662804431832 | Cyprus |
| 1996 | 163.21561954613 | 0.709839946474019 | 33.7575444712835 | 129.458075074847 | Cyprus |
| 1997 | 158.0983786 | 0.718864243512848 | 28.9714067965824 | 129.126971813018 | Cyprus |
| 1998 | 152.79389940063 | 0.727964381748969 | 25.5084916818587 | 127.285407718771 | Cyprus |
| 1999 | 148.11281320595 | 0.736846134920381 | 22.1577008929651 | 125.955112312985 | Cyprus |
| 2000 | 144.24402623316 | 0.745199300565367 | 19.5613797406525 | 124.682646492508 | Cyprus |
| 2001 | 141.11032329535 | 0.753594717257543 | 17.7381928822848 | 123.372130413065 | Cyprus |
| 2002 | 139.76009932202 | 0.76150246522841 | 15.3521820545556 | 124.407917267464 | Cyprus |
| 2003 | 138.59503463596 | 0.767896215824759 | 14.4985150165172 | 124.096519619443 | Cyprus |
| 2004 | 138.51954465512 | 0.77376477492064 | 13.1500285818604 | 125.36951607326 | Cyprus |
| 2005 | 138.59938485994 | 0.779031361179798 | 12.9559567980216 | 125.643428061918 | Cyprus |
| 2006 | 137.01573451117 | 0.784560964983202 | 11.1097915120867 | 125.905942999083 | Cyprus |
| 2007 | 135.21669714002 | 0.792068382747098 | 10.8065865325378 | 124.410110607482 | Cyprus |
| 2008 | 133.49572018176 | 0.799454089093792 | 10.7035260970024 | 122.792194084758 | Cyprus |
| 2009 | 131.68965362462 | 0.805112085484562 | 10.5281493282244 | 121.161504296396 | Cyprus |
| 2010 | 128.06572000001 | 0.809840187817991 | 9.442162613 | 118.623557387028 | Cyprus |
| 2011 | 124.19393625353 | 0.813726132185002 | 9.09686880076714 | 115.097067452763 | Cyprus |
| 2012 | 120.52088147055 | 0.816978525424299 | 8.43070352886948 | 112.090177941681 | Cyprus |
| 2013 | 115.68418211247 | 0.819380601631525 | 8.38664502537065 | 107.297537087099 | Cyprus |
| 2014 | 113.28269774374 | 0.821184302169431 | 8.76209987714227 | 104.520597866598 | Cyprus |
| 2015 | 110.81489093391 | 0.822732850898765 | 8.18037961563524 | 102.634511318275 | Cyprus |
| 2016 | 105.71880116778 | 0.824283624143557 | 7.60625057637667 | 98.1125505914033 | Cyprus |
| 2017 | 97.45035194 | 0.826139877968847 | 7.74975182321677 | 89.7006001135832 | Cyprus |
| 2018 | 90.18201077 | 0.828343243063834 | 7.82062520111845 | 82.3613855663615 | Cyprus |
| 2019 | 85.33927085 | 0.830980727806796 | 7.41690472041393 | 77.9223661273361 | Cyprus |
| 2020 | 82.14114041 | 0.833059522677264 | 7.47097341288526 | 74.6701669957647 | Cyprus |
| 2021 | 80.99967198 | 0.835630545002355 | 6.59944726057787 | 74.4002247185421 | Cyprus |
| 1990 | 71.29480969 | 0.801154655014013 | 10.6086318861862 | 60.6861778020538 | Denmark |
| 1991 | 68.73562441 | 0.804540349639536 | 9.83024697872959 | 58.9053774267304 | Denmark |
| 1992 | 64.47010794 | 0.808164433760271 | 9.36101209539038 | 55.1090958413696 | Denmark |
| 1993 | 55.84242487 | 0.811585712633093 | 9.30799872921263 | 46.5344261439074 | Denmark |
| 1994 | 54.54550302 | 0.815138614432107 | 9.42575232238342 | 45.1197506950466 | Denmark |
| 1995 | 52.46697787 | 0.819104611612713 | 7.89624914186103 | 44.570728730519 | Denmark |
| 1996 | 52.61602463 | 0.82337524309543 | 8.99080929103591 | 43.6252153356941 | Denmark |
| 1997 | 52.1325951 | 0.827679979394938 | 7.82375577510683 | 44.3088393265132 | Denmark |
| 1998 | 52.37881865 | 0.831683237018017 | 7.42644475678975 | 44.9523738882203 | Denmark |
| 1999 | 53.24185518 | 0.835381542910635 | 6.32843407203114 | 46.9134211115789 | Denmark |
| 2000 | 59.46672314 | 0.8391842975615 | 7.45124192778591 | 52.0154812108841 | Denmark |
| 2001 | 63.90473917 | 0.843177750467757 | 6.32443060098905 | 57.580308565821 | Denmark |
| 2002 | 67.81065649 | 0.847086356838798 | 5.47498912909776 | 62.3356673655222 | Denmark |
| 2003 | 74.50329184 | 0.850441325949586 | 6.54079112752151 | 67.9625007100585 | Denmark |
| 2004 | 80.95335502 | 0.853304103185433 | 6.28606625836779 | 74.6672887582122 | Denmark |
| 2005 | 89.52779199 | 0.855690732240651 | 6.56647063715364 | 82.9613213574364 | Denmark |
| 2006 | 97.23182653 | 0.858033209615474 | 6.3585979416781 | 90.8732285844019 | Denmark |
| 2007 | 115.33138426369 | 0.859781733794886 | 6.53504938691914 | 108.796334876771 | Denmark |
| 2008 | 124.40456843621 | 0.861782061067554 | 6.75915991215061 | 117.645408524059 | Denmark |
| 2009 | 123.21033186225 | 0.86394804976016 | 5.61508560549719 | 117.595246256753 | Denmark |
| 2010 | 122.8915883 | 0.866767079164022 | 6.1283441079936 | 116.763244185606 | Denmark |
| 2011 | 112.48267086533 | 0.869778682281296 | 6.21335282009541 | 106.269318045235 | Denmark |
| 2012 | 105.40744825409 | 0.872517510381731 | 6.53773267342018 | 98.8697155806698 | Denmark |
| 2013 | 101.31070933572 | 0.874932973936641 | 6.40013598011377 | 94.9105733556062 | Denmark |
| 2014 | 97.71831264 | 0.876814931654032 | 6.32243617372583 | 91.3958764685642 | Denmark |
| 2015 | 93.24485286 | 0.878543944867921 | 6.24441108258679 | 87.0004417734232 | Denmark |
| 2016 | 90.20099743 | 0.8805897834924 | 6.42054180190942 | 83.7804556262706 | Denmark |
| 2017 | 90.08205119 | 0.883688486994588 | 6.03293555980114 | 84.0491156308589 | Denmark |
| 2018 | 84.18029214 | 0.887529846224258 | 6.90628965687484 | 77.2740024850052 | Denmark |
| 2019 | 75.05551315 | 0.891624571480342 | 5.64823100203904 | 69.407282152411 | Denmark |
| 2020 | 72.28873638 | 0.894368296843327 | 5.53188583524709 | 66.7568505449329 | Denmark |
| 2021 | 71.16640949 | 0.896424204101212 | 5.81429054756415 | 65.3521189439958 | Denmark |
| 1990 | 107.50691121237 | 0.756221508893542 | 17.7897160084271 | 89.7171952039429 | Finland |
| 1991 | 110.93901235697 | 0.758410686787082 | 16.3286821241996 | 94.6103302327704 | Finland |
| 1992 | 113.44400381026 | 0.760804099620088 | 15.706767887997 | 97.737235922263 | Finland |
| 1993 | 113.09368157498 | 0.763526603650282 | 14.4919680221544 | 98.6017135528256 | Finland |
| 1994 | 115.32868126249 | 0.767101900863416 | 14.643594201311 | 100.685087061179 | Finland |
| 1995 | 117.38481038646 | 0.771163742219649 | 12.4724458022258 | 104.912364584234 | Finland |
| 1996 | 119.98585789524 | 0.774809058593956 | 13.6595580741869 | 106.326299821053 | Finland |
| 1997 | 123.10411347663 | 0.778814214501226 | 11.388846704963 | 111.715266771667 | Finland |
| 1998 | 126.51599855448 | 0.782305059190857 | 12.510419323211 | 114.005579231269 | Finland |
| 1999 | 129.77335601429 | 0.78577398760406 | 11.4536403802925 | 118.319715633998 | Finland |
| 2000 | 134.85988068005 | 0.78998522981789 | 11.23189882 | 123.6279819 | Finland |
| 2001 | 138.79827428762 | 0.79445383485974 | 10.892345065077 | 127.905929222543 | Finland |
| 2002 | 145.82688180024 | 0.798770274163901 | 11.0814400798882 | 134.745441720352 | Finland |
| 2003 | 154.7224488 | 0.802325430617225 | 10.192659297113 | 144.529789462287 | Finland |
| 2004 | 161.72523622269 | 0.805694227411326 | 9.82633317945758 | 151.898903043232 | Finland |
| 2005 | 165.90462517617 | 0.808936787277645 | 9.07616308277724 | 156.828462093393 | Finland |
| 2006 | 161.86347568297 | 0.812081481875967 | 8.46566810064088 | 153.397807582329 | Finland |
| 2007 | 151.19855431142 | 0.815597613277792 | 8.88276683718986 | 142.31578747423 | Finland |
| 2008 | 134.24016871523 | 0.818875543929948 | 8.91117655351392 | 125.328992161716 | Finland |
| 2009 | 115.38837766051 | 0.821457587971858 | 8.41359476390259 | 106.974782896607 | Finland |
| 2010 | 100.98814845874 | 0.824844982269378 | 8.02836373446036 | 92.9597847242796 | Finland |
| 2011 | 87.40936126 | 0.82827057673312 | 8.56699267363462 | 78.8423685886354 | Finland |
| 2012 | 74.58522778 | 0.831434483518693 | 7.29930187828353 | 67.2859258981765 | Finland |
| 2013 | 61.19020222 | 0.834425400661261 | 7.05873244023679 | 54.1314697756332 | Finland |
| 2014 | 53.36310392 | 0.837431871657021 | 6.75151462881841 | 46.6115892939416 | Finland |
| 2015 | 46.82060367 | 0.840878476913666 | 6.41451636518341 | 40.4060873044366 | Finland |
| 2016 | 44.0629538 | 0.844622338388364 | 6.58469234143472 | 37.4782614587653 | Finland |
| 2017 | 42.86978713 | 0.848586127623286 | 6.45515527403733 | 36.4146318571227 | Finland |
| 2018 | 41.14341451 | 0.852202943239694 | 6.09387834272286 | 35.0495361721571 | Finland |
| 2019 | 40.38329982 | 0.855365178519151 | 6.61825188450083 | 33.7650479365192 | Finland |
| 2020 | 42.85120884 | 0.857655552665083 | 5.76247751663718 | 37.0887313269928 | Finland |
| 2021 | 47.11114236 | 0.859831367662218 | 5.90398912721455 | 41.2071532345155 | Finland |
| 1990 | 91.08294343 | 0.730747465817975 | 23.7457243880623 | 67.3372190424277 | France |
| 1991 | 97.09143805 | 0.736452382335769 | 21.9584259708234 | 75.1330120764566 | France |
| 1992 | 101.59204526777 | 0.742871973277562 | 20.4431184005263 | 81.1489268672437 | France |
| 1993 | 103.39499990857 | 0.748582326771669 | 18.7503208573104 | 84.6446790512596 | France |
| 1994 | 98.18629257 | 0.753176131807871 | 17.235379275426 | 80.950913297224 | France |
| 1995 | 93.35996553 | 0.75712578885604 | 16.3641042650207 | 76.9958612632093 | France |
| 1996 | 94.29011997 | 0.760899578763994 | 16.1535870347263 | 78.1365329357137 | France |
| 1997 | 93.95230395 | 0.764650672066045 | 14.4558763814195 | 79.4964275704105 | France |
| 1998 | 97.10090544 | 0.767852560919953 | 14.0491574126655 | 83.0517480240345 | France |
| 1999 | 99.18814205 | 0.770304362540569 | 13.9699666775084 | 85.2181753708516 | France |
| 2000 | 102.71197195861 | 0.772792033272814 | 13.5983497209148 | 89.1136222376952 | France |
| 2001 | 104.90342878051 | 0.776225157250438 | 12.5454224940168 | 92.3580062864932 | France |
| 2002 | 100.53076072178 | 0.780088871976379 | 12.260405413664 | 88.270355308116 | France |
| 2003 | 105.20554157198 | 0.783178757362352 | 11.6510847349056 | 93.5544568370744 | France |
| 2004 | 106.40728972172 | 0.786084654751087 | 11.7464508983084 | 94.6608388234116 | France |
| 2005 | 107.53710126198 | 0.789042086328118 | 10.63781037 | 96.89929089 | France |
| 2006 | 113.15018129942 | 0.792321919625469 | 11.4739133451074 | 101.676267954313 | France |
| 2007 | 115.55120614029 | 0.795557651447132 | 10.037199436281 | 105.514006704009 | France |
| 2008 | 117.34424206771 | 0.798177429586454 | 11.2920783832476 | 106.052163684462 | France |
| 2009 | 115.03940211044 | 0.800508669748406 | 10.1692817591227 | 104.870120351317 | France |
| 2010 | 110.70057307338 | 0.803240246888394 | 10.3886390770779 | 100.311933996302 | France |
| 2011 | 102.0831327 | 0.806378738306578 | 9.88304259922741 | 92.2000900903726 | France |
| 2012 | 104.13847638766 | 0.809615388940868 | 10.0608994790239 | 94.0775769086361 | France |
| 2013 | 107.25078680441 | 0.812984015183301 | 8.88756140174662 | 98.3632254026634 | France |
| 2014 | 108.13924415651 | 0.816551447578728 | 8.23959469648635 | 99.8996494600237 | France |
| 2015 | 110.52104187515 | 0.820238385480897 | 8.63835606136679 | 101.882685813783 | France |
| 2016 | 103.94316269891 | 0.82373092859985 | 8.21410806852705 | 95.729054630383 | France |
| 2017 | 101.70066843495 | 0.827098440641564 | 8.28116010321839 | 93.4195083317316 | France |
| 2018 | 99.74157193 | 0.830448590187259 | 7.77124517082563 | 91.9703267640544 | France |
| 2019 | 96.22721874 | 0.833772804699999 | 7.475498526 | 88.751720209556 | France |
| 2020 | 90.77585754 | 0.836049443246129 | 6.46785626797864 | 84.3080012699214 | France |
| 2021 | 88.59411351 | 0.838364875196954 | 6.54880716428083 | 82.0453063493392 | France |
| 1990 | 84.71193452 | 0.817077665790386 | 9.1094490448694 | 75.6024854759206 | Germany |
| 1991 | 91.90379858 | 0.823339898340462 | 8.30341861470687 | 83.6003799666631 | Germany |
| 1992 | 97.24577583 | 0.828323503378164 | 8.15863532667587 | 89.0871405043441 | Germany |
| 1993 | 101.06891071883 | 0.832286809367201 | 7.38504027624836 | 93.6838704425816 | Germany |
| 1994 | 104.22241023531 | 0.835561963052536 | 6.68966213512877 | 97.5327481001812 | Germany |
| 1995 | 106.40377690995 | 0.837643151522198 | 6.50119065218414 | 99.9025862577659 | Germany |
| 1996 | 104.53586186895 | 0.839188129123403 | 7.41939130486511 | 97.1164705640849 | Germany |
| 1997 | 100.48204381564 | 0.841385902988361 | 6.09452256637891 | 94.3875212492611 | Germany |
| 1998 | 94.68459169 | 0.843854978100034 | 6.37580076453687 | 88.3087909249731 | Germany |
| 1999 | 90.02491501 | 0.846232398245621 | 6.24589641421918 | 83.7790185974208 | Germany |
| 2000 | 91.10073867 | 0.848925756438545 | 6.12941640959444 | 84.9713222588556 | Germany |
| 2001 | 94.01182882 | 0.852070412843074 | 6.15260914898564 | 87.8592196744744 | Germany |
| 2002 | 97.67179463 | 0.855066792837203 | 5.77346966405981 | 91.8983249693802 | Germany |
| 2003 | 103.88761020148 | 0.857675790269356 | 5.4473485819286 | 98.4402616195514 | Germany |
| 2004 | 108.50347153694 | 0.860240332885484 | 5.8661325267504 | 102.63733901019 | Germany |
| 2005 | 114.15851710958 | 0.862894178369357 | 5.95703897979212 | 108.201478129788 | Germany |
| 2006 | 122.17454024091 | 0.865873706890654 | 6.19673980056983 | 115.97780044034 | Germany |
| 2007 | 131.0086723 | 0.869140440024023 | 6.53092930918691 | 124.477742945613 | Germany |
| 2008 | 130.42167713509 | 0.872552849301022 | 6.03967543398246 | 124.382001701108 | Germany |
| 2009 | 129.7470367 | 0.875087911282131 | 5.90730577286536 | 123.839730976735 | Germany |
| 2010 | 125.79830312795 | 0.877946710427414 | 6.13366933132782 | 119.664633796622 | Germany |
| 2011 | 116.47035058099 | 0.881027507503078 | 6.02892381248034 | 110.44142676851 | Germany |
| 2012 | 106.08118660138 | 0.88365640827103 | 6.44170377976449 | 99.6394828216155 | Germany |
| 2013 | 98.68391398 | 0.885926953064586 | 5.50765668768073 | 93.1762572948193 | Germany |
| 2014 | 88.64946815 | 0.887651614379013 | 5.73707446949533 | 82.9123936773347 | Germany |
| 2015 | 82.4241888 | 0.888867853966898 | 5.87102167985879 | 76.5531671200612 | Germany |
| 2016 | 71.29343126 | 0.89089636945676 | 6.05248722653526 | 65.2409440304947 | Germany |
| 2017 | 54.14473527 | 0.894050472424158 | 6.57502575102921 | 47.5697095218508 | Germany |
| 2018 | 40.566401 | 0.896975545420426 | 6.04370967313767 | 34.5226913245823 | Germany |
| 2019 | 30.88114584 | 0.899703158084962 | 6.44090028425654 | 24.4402455599435 | Germany |
| 2020 | 30.84806176 | 0.901438614019463 | 6.045045464 | 24.80301629 | Germany |
| 2021 | 29.11871892 | 0.902957091057572 | 6.03943617303864 | 23.0792827440814 | Germany |
| 1990 | 41.2324697 | 0.67418646507665 | 38.0909556067252 | 3.14151408827476 | Greece |
| 1991 | 40.74135119 | 0.680316480101809 | 37.649619378375 | 3.091731815 | Greece |
| 1992 | 40.46177224 | 0.687055389369745 | 37.350462740053 | 3.11130950389698 | Greece |
| 1993 | 40.27111945 | 0.69332892644804 | 36.5374170114782 | 3.73370243576179 | Greece |
| 1994 | 40.46551177 | 0.699153022352574 | 35.3418833043253 | 5.12362846900471 | Greece |
| 1995 | 40.97435175 | 0.704862873096307 | 33.5236150177093 | 7.45073673421066 | Greece |
| 1996 | 41.79480548 | 0.710139335918529 | 33.5659413490219 | 8.22886412786814 | Greece |
| 1997 | 42.71943037 | 0.715687267664404 | 30.4819782886268 | 12.2374520774632 | Greece |
| 1998 | 43.85453328 | 0.721593982464726 | 30.0075614025564 | 13.8469718763836 | Greece |
| 1999 | 44.91632288 | 0.727529454753368 | 25.2713190271377 | 19.6450038516923 | Greece |
| 2000 | 45.96431279 | 0.732710539506218 | 21.3655149035082 | 24.5987978850818 | Greece |
| 2001 | 47.22547192 | 0.737154008101854 | 22.2551360905143 | 24.9703358327157 | Greece |
| 2002 | 49.1566853 | 0.741945221017726 | 19.964247220693 | 29.192438082637 | Greece |
| 2003 | 50.88837793 | 0.747185955887149 | 19.9083636832149 | 30.9800142480851 | Greece |
| 2004 | 52.89404832 | 0.752418606565094 | 17.8936150107524 | 35.0004333098076 | Greece |
| 2005 | 54.3467202 | 0.75638439564551 | 16.6919429306091 | 37.6547772727709 | Greece |
| 2006 | 55.91269636 | 0.760209695750845 | 16.5299000025428 | 39.3827963611672 | Greece |
| 2007 | 57.35283751 | 0.763927173080314 | 14.5709651846436 | 42.7818723208364 | Greece |
| 2008 | 57.82465985 | 0.767527657538734 | 13.5399649587579 | 44.2846948912121 | Greece |
| 2009 | 58.82843959 | 0.771247355848723 | 12.8605019976151 | 45.9679375920549 | Greece |
| 2010 | 59.36026571 | 0.775155814653294 | 12.0094119562284 | 47.3508537524716 | Greece |
| 2011 | 57.87080657 | 0.778525866909133 | 13.289092785679 | 44.581713780731 | Greece |
| 2012 | 55.92403145 | 0.780789482881484 | 11.2774308126235 | 44.6466006392165 | Greece |
| 2013 | 54.07028506 | 0.781949051742451 | 11.8994902561825 | 42.1707948009475 | Greece |
| 2014 | 51.77442679 | 0.782442012668796 | 12.5753869526261 | 39.1990398351839 | Greece |
| 2015 | 49.38553548 | 0.782489523445532 | 11.2446031817617 | 38.1409322962883 | Greece |
| 2016 | 45.70019814 | 0.782798442836071 | 12.5293423523367 | 33.1708557838133 | Greece |
| 2017 | 39.51342117 | 0.784108593896727 | 12.0542981970016 | 27.4591229752784 | Greece |
| 2018 | 33.94924273 | 0.785999752836636 | 11.5640039590414 | 22.3852387738186 | Greece |
| 2019 | 30.18977313 | 0.788138138237775 | 10.9760091430878 | 19.2137639893822 | Greece |
| 2020 | 26.8526276 | 0.789632219998033 | 12.044090289195 | 14.808537310145 | Greece |
| 2021 | 21.62881713 | 0.791854407912829 | 11.503065201758 | 10.125751928942 | Greece |
| 1990 | 95.18114448 | 0.764212516960703 | 14.1862512431117 | 80.9948932358483 | Iceland |
| 1991 | 98.21686723 | 0.769027393143331 | 14.8257706932433 | 83.3910965354867 | Iceland |
| 1992 | 101.70140025645 | 0.772706414740194 | 14.194842493514 | 87.506557762936 | Iceland |
| 1993 | 103.59140201629 | 0.776389422872018 | 12.0337569874026 | 91.5576450288874 | Iceland |
| 1994 | 105.727555 | 0.77996796073086 | 12.0779811614087 | 93.6495738755913 | Iceland |
| 1995 | 108.03253810915 | 0.782740057259954 | 11.49307583 | 96.53946228 | Iceland |
| 1996 | 108.48791454977 | 0.785356419811944 | 11.6253450374462 | 96.8625695123238 | Iceland |
| 1997 | 100.77635981736 | 0.78852833520489 | 11.9854366420339 | 88.7909231753261 | Iceland |
| 1998 | 92.95510416 | 0.792626368445487 | 11.0962377273998 | 81.8588664318602 | Iceland |
| 1999 | 87.05205204 | 0.797368807995672 | 10.620089706899 | 76.431962328621 | Iceland |
| 2000 | 86.44960898 | 0.803030330314896 | 10.2810798691219 | 76.1685291066781 | Iceland |
| 2001 | 89.31117298 | 0.808965785947657 | 9.65854936967787 | 79.6526236134321 | Iceland |
| 2002 | 91.97483554 | 0.813400392129504 | 9.69379058028254 | 82.2810449579875 | Iceland |
| 2003 | 95.58575153 | 0.816597066979147 | 7.8354587736566 | 87.7502927520034 | Iceland |
| 2004 | 99.59372193 | 0.819528086895788 | 8.1551501502385 | 91.4385717822715 | Iceland |
| 2005 | 103.52691892096 | 0.822766536023531 | 7.85269916722434 | 95.6742197537357 | Iceland |
| 2006 | 108.85350281013 | 0.82624474349372 | 8.4556497116681 | 100.397853098462 | Iceland |
| 2007 | 113.70735763095 | 0.830289889861998 | 7.82808001168523 | 105.879277619265 | Iceland |
| 2008 | 115.70020522586 | 0.834622678087519 | 7.18419577255527 | 108.516009453305 | Iceland |
| 2009 | 115.58697589161 | 0.838121013089424 | 7.0732396370415 | 108.513736254568 | Iceland |
| 2010 | 114.56098714147 | 0.841384642074063 | 6.8696831589352 | 107.691303982535 | Iceland |
| 2011 | 114.88908593608 | 0.844619929908845 | 6.86587211795067 | 108.023213818129 | Iceland |
| 2012 | 122.57057267852 | 0.84729944372556 | 6.49123107214916 | 116.079341606371 | Iceland |
| 2013 | 133.18142239401 | 0.850173947991231 | 6.38012460070788 | 126.801297793302 | Iceland |
| 2014 | 141.52481977753 | 0.853558696790214 | 5.64222413298539 | 135.882595644545 | Iceland |
| 2015 | 144.01000165699 | 0.857724976230652 | 6.16830764726596 | 137.841694009724 | Iceland |
| 2016 | 137.08421354836 | 0.861844466505036 | 6.18287084604583 | 130.901342702314 | Iceland |
| 2017 | 126.55340076378 | 0.865702890708071 | 5.8937237318497 | 120.65967703193 | Iceland |
| 2018 | 114.21050758731 | 0.869219821760483 | 6.36205501867017 | 107.84845256864 | Iceland |
| 2019 | 104.71913075828 | 0.872425580161805 | 5.57819365689709 | 99.1409371013829 | Iceland |
| 2020 | 102.00031130515 | 0.874323153425611 | 5.65376779441577 | 96.3465435107342 | Iceland |
| 2021 | 100.61584968055 | 0.876361679802766 | 6.15477640065596 | 94.461073279894 | Iceland |
| 1990 | 49.33605754 | 0.719891818965305 | 29.0872711673468 | 20.2487863695732 | Ireland |
| 1991 | 49.32841554 | 0.725195018704741 | 26.0412460101775 | 23.2871695295725 | Ireland |
| 1992 | 49.66471948 | 0.731918691315293 | 23.9864437889633 | 25.6782756903967 | Ireland |
| 1993 | 50.18583442 | 0.738384073791795 | 21.5238823968721 | 28.6619520246579 | Ireland |
| 1994 | 50.15214605 | 0.744011727859478 | 20.7823212943077 | 29.3698247532223 | Ireland |
| 1995 | 51.35522976 | 0.748921575045051 | 18.1982515892421 | 33.1569781741479 | Ireland |
| 1996 | 52.37034611 | 0.754010123608321 | 17.4001803554603 | 34.9701657569297 | Ireland |
| 1997 | 54.05796961 | 0.759970383842882 | 16.7632856475345 | 37.2946839594255 | Ireland |
| 1998 | 55.90114927 | 0.766488720882709 | 14.2457506589143 | 41.6553986105657 | Ireland |
| 1999 | 58.71150103 | 0.773538834439684 | 11.7730321838392 | 46.9384688427508 | Ireland |
| 2000 | 61.58923096 | 0.780190655170267 | 12.0566266639223 | 49.5326042932077 | Ireland |
| 2001 | 64.61863018 | 0.786353740708812 | 11.328545315793 | 53.290084863637 | Ireland |
| 2002 | 67.35299189 | 0.793010605962827 | 10.8143826152036 | 56.5386092710664 | Ireland |
| 2003 | 71.49226848 | 0.800005865377216 | 9.99747070916148 | 61.4947977721485 | Ireland |
| 2004 | 73.81191315 | 0.806853140411776 | 10.1174542623151 | 63.6944588889449 | Ireland |
| 2005 | 76.6501466 | 0.811713818388319 | 9.55118753365771 | 67.0989590663623 | Ireland |
| 2006 | 82.25362063 | 0.814505479095719 | 9.50856876755632 | 72.7450518652737 | Ireland |
| 2007 | 88.53960014 | 0.816931026031536 | 7.67114616332785 | 80.8684539800121 | Ireland |
| 2008 | 94.47473905 | 0.819932128191974 | 8.80259358681588 | 85.6721454673741 | Ireland |
| 2009 | 97.47392382 | 0.823929666301939 | 7.73484600937035 | 89.7390778098896 | Ireland |
| 2010 | 99.90938028 | 0.828103848001623 | 7.51086502873055 | 92.3985152505895 | Ireland |
| 2011 | 99.76307069 | 0.83180479150489 | 7.66122930943696 | 92.101841383853 | Ireland |
| 2012 | 102.9693436 | 0.835325358632175 | 7.60523061574927 | 95.3641130220507 | Ireland |
| 2013 | 107.27649188303 | 0.838644432713483 | 6.82418561451735 | 100.452306268513 | Ireland |
| 2014 | 109.85135199469 | 0.842082114682265 | 5.97563858003339 | 103.875713414657 | Ireland |
| 2015 | 111.01152960061 | 0.847437886654412 | 6.23630532221317 | 104.775224278397 | Ireland |
| 2016 | 91.59964601 | 0.85264250194342 | 7.20082626299306 | 84.398819747467 | Ireland |
| 2017 | 55.57973468 | 0.857816163802237 | 6.14315609529451 | 49.4365785883555 | Ireland |
| 2018 | 27.40056808 | 0.863925284412195 | 6.42432123913967 | 20.9762468427803 | Ireland |
| 2019 | 16.36658577 | 0.869412503348037 | 6.02552772093458 | 10.3410580450754 | Ireland |
| 2020 | 24.44825431 | 0.871958822388357 | 5.99705225406756 | 18.4512020523224 | Ireland |
| 2021 | 22.92146022 | 0.873753849787408 | 5.98315459113882 | 16.9383056326512 | Ireland |
| 1990 | 167.88002945396 | 0.709178347365152 | 33.5077157607945 | 134.372313693166 | Israel |
| 1991 | 169.97340603081 | 0.713371633399082 | 31.0760923417567 | 138.897313689053 | Israel |
| 1992 | 183.43879817861 | 0.717897923179142 | 31.3101729804976 | 152.128625198112 | Israel |
| 1993 | 174.22218517109 | 0.72225017554813 | 29.7486247081023 | 144.473560462988 | Israel |
| 1994 | 164.58531391866 | 0.726654735721658 | 26.6976444256034 | 137.887669493057 | Israel |
| 1995 | 156.97650595818 | 0.731127859056384 | 23.7553256221961 | 133.221180335984 | Israel |
| 1996 | 156.39515060018 | 0.734714025875415 | 22.1682029464819 | 134.226947653698 | Israel |
| 1997 | 159.96989439833 | 0.738015276501763 | 21.9996376348566 | 137.970256763473 | Israel |
| 1998 | 167.0723152 | 0.741927189995428 | 21.1616989272477 | 145.910616316352 | Israel |
| 1999 | 175.17305659967 | 0.745443338031474 | 20.8501063530056 | 154.322950246664 | Israel |
| 2000 | 186.72384201327 | 0.749184084123824 | 18.3480544914878 | 168.375787521782 | Israel |
| 2001 | 189.82659034357 | 0.752834296773304 | 18.0459791270629 | 171.780611216507 | Israel |
| 2002 | 201.35966332427 | 0.755988075842232 | 17.5280027136185 | 183.831660610652 | Israel |
| 2003 | 212.23217857027 | 0.758875408886722 | 16.2230784336232 | 196.009100136647 | Israel |
| 2004 | 216.66711111779 | 0.762213291396151 | 14.9007111522071 | 201.766399965583 | Israel |
| 2005 | 226.25453646128 | 0.765543461435524 | 14.5414556511573 | 211.713080810123 | Israel |
| 2006 | 242.48822237282 | 0.768733897655938 | 14.3668296112241 | 228.121392761596 | Israel |
| 2007 | 239.88538236069 | 0.770756143247979 | 13.0254833140102 | 226.85989904668 | Israel |
| 2008 | 222.49353758122 | 0.772011614541036 | 13.3994201193797 | 209.09411746184 | Israel |
| 2009 | 197.35771899978 | 0.773303913379524 | 13.2904381320105 | 184.06728086777 | Israel |
| 2010 | 177.41179462445 | 0.775259530065334 | 12.2927616603298 | 165.11903296412 | Israel |
| 2011 | 153.14811648624 | 0.77764966916418 | 12.2896513552804 | 140.85846513096 | Israel |
| 2012 | 137.16894863609 | 0.780403414638078 | 11.3849337477765 | 125.784014888313 | Israel |
| 2013 | 121.6740984 | 0.783267166608914 | 13.0237702644294 | 108.650328087071 | Israel |
| 2014 | 108.3684112 | 0.78615108379553 | 11.7583602747846 | 96.6100509002154 | Israel |
| 2015 | 100.26338673884 | 0.788874456945394 | 11.4307611571426 | 88.8326255816974 | Israel |
| 2016 | 93.78367009 | 0.791772445367912 | 12.0388697160327 | 81.7448003736274 | Israel |
| 2017 | 90.39589686 | 0.795173437476151 | 10.4987681257575 | 79.8971287384725 | Israel |
| 2018 | 82.9135029 | 0.799124993659694 | 10.5926307211833 | 72.3208721799867 | Israel |
| 2019 | 78.07366132 | 0.803232990528098 | 9.88141506993716 | 68.1922462509228 | Israel |
| 2020 | 83.84895533 | 0.806351589333701 | 9.35834340245577 | 74.4906119312842 | Israel |
| 2021 | 74.04494035 | 0.809011651557631 | 9.39814402878143 | 64.6467963167486 | Israel |
| 1990 | 35.46922335 | 0.706255223740403 | 34.3234181525821 | 1.14580519289795 | Italy |
| 1991 | 34.42653316 | 0.711457348523815 | 32.1776340031597 | 2.24889915231029 | Italy |
| 1992 | 34.73183325 | 0.716649746121184 | 31.3022191092094 | 3.42961413819057 | Italy |
| 1993 | 33.09082466 | 0.721720992266354 | 30.0438922071705 | 3.0469324565895 | Italy |
| 1994 | 31.03502969 | 0.726870913262732 | 24.0835903553678 | 6.95143933177217 | Italy |
| 1995 | 28.94712869 | 0.731644081628044 | 23.6960517170315 | 5.25107697643848 | Italy |
| 1996 | 29.82755981 | 0.736050870453315 | 21.1254564922565 | 8.7021033202935 | Italy |
| 1997 | 31.30146617 | 0.739972336484864 | 20.30501142 | 10.99645475 | Italy |
| 1998 | 32.77455431 | 0.743101595998998 | 19.7909175764452 | 12.9836367307148 | Italy |
| 1999 | 35.07341618 | 0.746074084421097 | 20.4549792136183 | 14.6184369697117 | Italy |
| 2000 | 37.65916193 | 0.749675986238207 | 18.2664824983041 | 19.3926794341859 | Italy |
| 2001 | 41.2961486 | 0.753554839457876 | 16.7936244648457 | 24.5025241365543 | Italy |
| 2002 | 44.49646296 | 0.757191479404768 | 15.9994798065376 | 28.4969831508524 | Italy |
| 2003 | 50.72262004 | 0.76009374379023 | 18.1693362744572 | 32.5532837700428 | Italy |
| 2004 | 55.63034757 | 0.762787749063442 | 14.1491290219491 | 41.4812185443709 | Italy |
| 2005 | 58.22578622 | 0.765689914012792 | 14.4787369157474 | 43.7470493050326 | Italy |
| 2006 | 58.11173672 | 0.768402407690909 | 14.1415426137823 | 43.9701941029077 | Italy |
| 2007 | 58.76272369 | 0.770698380337301 | 12.6195189580515 | 46.1432047360085 | Italy |
| 2008 | 60.76619109 | 0.773068469998338 | 12.7523344501876 | 48.0138566391924 | Italy |
| 2009 | 62.8578461 | 0.775499262002478 | 12.0461867415674 | 50.8116593575226 | Italy |
| 2010 | 65.838849 | 0.778109205917719 | 12.825785133623 | 53.013063862587 | Italy |
| 2011 | 68.96794994 | 0.780703467869893 | 12.0499377574561 | 56.9180121871639 | Italy |
| 2012 | 72.58605877 | 0.783090981066118 | 11.3018687040719 | 61.2841900707181 | Italy |
| 2013 | 74.18458357 | 0.785356877137869 | 12.3338067410444 | 61.8507768336056 | Italy |
| 2014 | 77.07192626 | 0.787533956365705 | 11.10705089 | 65.96487538 | Italy |
| 2015 | 80.36090075 | 0.789902118846972 | 10.9620286891691 | 69.3988720650909 | Italy |
| 2016 | 82.0282783 | 0.792477244295977 | 10.9963623197641 | 71.0319159761959 | Italy |
| 2017 | 74.2295562 | 0.795311186445406 | 11.0406138144571 | 63.1889423889629 | Italy |
| 2018 | 64.76916598 | 0.798362690372083 | 10.4933841551851 | 54.2757818246349 | Italy |
| 2019 | 50.49500839 | 0.801530020572533 | 9.94383451897347 | 40.5511738700165 | Italy |
| 2020 | 46.24005219 | 0.803635679966778 | 10.4389562817956 | 35.8010959089844 | Italy |
| 2021 | 42.13602436 | 0.805773533613512 | 9.7583091572213 | 32.3777151988487 | Italy |
| 1990 | 71.10654471 | 0.781051609326011 | 11.7811051874608 | 59.3254395197992 | Luxembourg |
| 1991 | 74.71130015 | 0.785961442083159 | 10.5554440466578 | 64.1558561063122 | Luxembourg |
| 1992 | 74.74933587 | 0.789053363703187 | 12.0089719183466 | 62.7403639517534 | Luxembourg |
| 1993 | 71.58106296 | 0.792843836314663 | 10.4039207597536 | 61.1771422002764 | Luxembourg |
| 1994 | 70.11982369 | 0.797824008094164 | 10.5846265711351 | 59.5351971164949 | Luxembourg |
| 1995 | 66.40285449 | 0.802682469757881 | 10.3150584283898 | 56.0877960628802 | Luxembourg |
| 1996 | 68.98175978 | 0.80651613780194 | 10.1799367620058 | 58.8018230214542 | Luxembourg |
| 1997 | 68.09198309 | 0.810235288443831 | 9.84180487323213 | 58.2501782199179 | Luxembourg |
| 1998 | 73.32959597 | 0.813889026223085 | 9.77213962908392 | 63.5574563406761 | Luxembourg |
| 1999 | 73.31084589 | 0.817570995147661 | 9.05696446993204 | 64.253881423028 | Luxembourg |
| 2000 | 74.26207282 | 0.822006568181537 | 8.05760196649968 | 66.2044708505603 | Luxembourg |
| 2001 | 84.76969998 | 0.825312816180712 | 8.27491089076274 | 76.4947890925673 | Luxembourg |
| 2002 | 87.9361054 | 0.828223491084071 | 7.70513411839204 | 80.230971284018 | Luxembourg |
| 2003 | 91.3510985 | 0.830983945726346 | 7.85200516783333 | 83.4990933301167 | Luxembourg |
| 2004 | 91.11339739 | 0.833672567288171 | 7.22137431593066 | 83.8920230700993 | Luxembourg |
| 2005 | 91.14516126 | 0.836675263504864 | 7.14828071126433 | 83.9968805473257 | Luxembourg |
| 2006 | 90.08083614 | 0.840574453017841 | 6.70405661701904 | 83.376779519191 | Luxembourg |
| 2007 | 86.12801292 | 0.84470700603675 | 5.99690716197188 | 80.1311057581281 | Luxembourg |
| 2008 | 68.14439808 | 0.847978883152449 | 6.79026276966727 | 61.3541353057927 | Luxembourg |
| 2009 | 55.90988724 | 0.850688725320833 | 5.96335375120308 | 49.9465334842369 | Luxembourg |
| 2010 | 51.42848104 | 0.85325827666028 | 6.8341475363614 | 44.5943335083386 | Luxembourg |
| 2011 | 41.29962665 | 0.856216833587911 | 6.15894884648364 | 35.1406778045764 | Luxembourg |
| 2012 | 31.14545734 | 0.859789031199815 | 5.99296686757056 | 25.1524904689894 | Luxembourg |
| 2013 | 30.82837429 | 0.863368792293471 | 6.07086856611567 | 24.7575057263743 | Luxembourg |
| 2014 | 26.75065815 | 0.86669355608594 | 5.12824335083278 | 21.6224147975772 | Luxembourg |
| 2015 | 23.94598949 | 0.87011293699584 | 6.79534086169478 | 17.1506486249452 | Luxembourg |
| 2016 | 24.40191271 | 0.873420879802852 | 6.29372719616419 | 18.1081855115458 | Luxembourg |
| 2017 | 21.55273576 | 0.876347684357551 | 6.03756853332692 | 15.5151672217131 | Luxembourg |
| 2018 | 22.43915099 | 0.878637895036722 | 5.96509656687375 | 16.4740544248363 | Luxembourg |
| 2019 | 18.50111943 | 0.880593895218451 | 6.60418367350654 | 11.8969357515935 | Luxembourg |
| 2020 | 25.96106457 | 0.882495316094676 | 5.74190889228067 | 20.2191556791693 | Luxembourg |
| 2021 | 15.43927237 | 0.884428955125693 | 6.8023377259729 | 8.6369346406771 | Luxembourg |
| 1990 | 47.11611821 | 0.656504584147482 | 45.0226659465162 | 2.09345226006375 | Malta |
| 1991 | 49.37983996 | 0.66111269147347 | 43.428846546099 | 5.95099341801102 | Malta |
| 1992 | 50.56224791 | 0.666324647620912 | 39.6096725787065 | 10.9525753266635 | Malta |
| 1993 | 51.51959006 | 0.672120257474545 | 38.0897448446102 | 13.4298452200098 | Malta |
| 1994 | 52.60341447 | 0.67784731358543 | 37.5679161061266 | 15.0354983646834 | Malta |
| 1995 | 54.12751168 | 0.682617703672922 | 37.6219867076427 | 16.5055249723473 | Malta |
| 1996 | 54.65210473 | 0.68662326099066 | 37.0468249998003 | 17.6052797347597 | Malta |
| 1997 | 55.35484516 | 0.691756406092708 | 36.5423671339023 | 18.8124780230177 | Malta |
| 1998 | 56.67539712 | 0.698095287002014 | 35.8359751651031 | 20.8394219585869 | Malta |
| 1999 | 58.12958743 | 0.704734545351825 | 34.2404983689042 | 23.8890890643758 | Malta |
| 2000 | 59.91406262 | 0.711805254226466 | 31.1081707873377 | 28.8058918306723 | Malta |
| 2001 | 61.55132616 | 0.717803037638719 | 31.4394174009961 | 30.1119087594439 | Malta |
| 2002 | 63.51850506 | 0.722790274731056 | 28.8884241335302 | 34.6300809223698 | Malta |
| 2003 | 65.70274189 | 0.727511133046943 | 26.3006739800711 | 39.4020679124889 | Malta |
| 2004 | 68.01530042 | 0.731933852781332 | 23.7802238752535 | 44.2350765455865 | Malta |
| 2005 | 70.50982595 | 0.736188746914511 | 22.0692567621362 | 48.4405691886037 | Malta |
| 2006 | 73.63111669 | 0.740140181028362 | 20.5428590033883 | 53.0882576866617 | Malta |
| 2007 | 76.88475984 | 0.744237811910071 | 19.7393961103418 | 57.1453637337582 | Malta |
| 2008 | 78.64718032 | 0.748427095431231 | 18.5075772848418 | 60.1396030395982 | Malta |
| 2009 | 78.92229291 | 0.752171058957125 | 18.7668829030226 | 60.1554100064174 | Malta |
| 2010 | 78.77358875 | 0.756250500159098 | 18.3413478136104 | 60.4322409332496 | Malta |
| 2011 | 76.49989798 | 0.760310546511914 | 15.7790913185425 | 60.7208066658675 | Malta |
| 2012 | 75.50074023 | 0.764537880070192 | 13.5263583457151 | 61.9743818838049 | Malta |
| 2013 | 74.83339091 | 0.768616139641066 | 14.4583029364059 | 60.3750879765441 | Malta |
| 2014 | 73.63657338 | 0.772728741377431 | 14.3951870807643 | 59.2413862990757 | Malta |
| 2015 | 73.1318752 | 0.777276905450885 | 13.9801651386235 | 59.1517100584065 | Malta |
| 2016 | 70.27318955 | 0.781673409574621 | 12.0327060731422 | 58.2404834754378 | Malta |
| 2017 | 65.36918967 | 0.786410183585369 | 11.6044426896733 | 53.7647469821867 | Malta |
| 2018 | 59.58860202 | 0.79096561824237 | 11.480715062398 | 48.107886955552 | Malta |
| 2019 | 56.1183249 | 0.795197845728697 | 10.7507896326317 | 45.3675352661683 | Malta |
| 2020 | 52.77681018 | 0.798375166241004 | 10.3469326215977 | 42.4298775604123 | Malta |
| 2021 | 51.82410385 | 0.801585033998971 | 9.94871730578232 | 41.8753865412377 | Malta |
| 1990 | 68.26609016 | 0.794612123302941 | 10.5737155458818 | 57.6923746101982 | Netherlands |
| 1991 | 70.01294883 | 0.799285437176668 | 10.2354194436444 | 59.7775293843056 | Netherlands |
| 1992 | 72.5926379 | 0.803965978232379 | 9.73690208636138 | 62.8557358143886 | Netherlands |
| 1993 | 74.57713822 | 0.808278967039783 | 9.68906214952037 | 64.8880760683996 | Netherlands |
| 1994 | 75.74409248 | 0.812530215874006 | 9.462975661 | 66.28111682 | Netherlands |
| 1995 | 77.71787939 | 0.816662769214157 | 9.27025038759102 | 68.447629005419 | Netherlands |
| 1996 | 80.31739556 | 0.819984219587417 | 9.58257192807875 | 70.7348236349312 | Netherlands |
| 1997 | 82.47511499 | 0.82288177029344 | 7.694706355 | 74.78040863 | Netherlands |
| 1998 | 85.46923936 | 0.825882233724006 | 7.532352727 | 77.936886628884 | Netherlands |
| 1999 | 89.36706155 | 0.828693827126542 | 7.94450562174632 | 81.4225559292837 | Netherlands |
| 2000 | 95.02378749 | 0.831913983967645 | 6.98384846813353 | 88.0399390173865 | Netherlands |
| 2001 | 101.21136296018 | 0.835518148042208 | 5.56596502205222 | 95.6453979381278 | Netherlands |
| 2002 | 107.77378925633 | 0.838676076741848 | 6.81854013286114 | 100.955249123469 | Netherlands |
| 2003 | 114.38271255842 | 0.841639477052547 | 7.04468341496214 | 107.338029143458 | Netherlands |
| 2004 | 120.2981293 | 0.844807347619539 | 6.03974045794926 | 114.258388792351 | Netherlands |
| 2005 | 129.29773229083 | 0.847934189839563 | 6.21223664504696 | 123.085495645783 | Netherlands |
| 2006 | 134.96144914409 | 0.850798807603514 | 6.92579320625796 | 128.035655937832 | Netherlands |
| 2007 | 138.56805936198 | 0.853595391042959 | 6.38513240193632 | 132.182926960044 | Netherlands |
| 2008 | 141.37202483219 | 0.8563684666871 | 6.38605885340431 | 134.985965978786 | Netherlands |
| 2009 | 143.68598241644 | 0.858600459092211 | 5.81732964477393 | 137.868652771666 | Netherlands |
| 2010 | 143.4615074 | 0.861255297781524 | 5.56740576416041 | 137.89410164224 | Netherlands |
| 2011 | 138.48253 | 0.864246678180707 | 6.34329829144726 | 132.139231699753 | Netherlands |
| 2012 | 138.33626126168 | 0.866950626243189 | 5.91254398712857 | 132.423717274551 | Netherlands |
| 2013 | 138.15517131838 | 0.8694029789072 | 6.18569348465437 | 131.969477833726 | Netherlands |
| 2014 | 137.02642082775 | 0.871848914319002 | 5.945020487 | 131.08140034054 | Netherlands |
| 2015 | 135.78966914932 | 0.874402841072396 | 5.8371849267052 | 129.952484222615 | Netherlands |
| 2016 | 132.42438812574 | 0.876784051787216 | 6.58726071099688 | 125.837127414743 | Netherlands |
| 2017 | 125.19584324321 | 0.87930015206581 | 5.25566916298353 | 119.940174080226 | Netherlands |
| 2018 | 116.11616247937 | 0.881900218359353 | 6.10834551568836 | 110.007816963682 | Netherlands |
| 2019 | 111.80220612372 | 0.884643839807538 | 5.84215363677716 | 105.960052486943 | Netherlands |
| 2020 | 108.1185793 | 0.886558566295774 | 6.0363875456214 | 102.082191731679 | Netherlands |
| 2021 | 105.51608949915 | 0.888464256397732 | 5.7403704367253 | 99.7757190624247 | Netherlands |
| 1990 | 50.9416562 | 0.795887277333644 | 10.9442215024416 | 39.9974346998084 | Norway |
| 1991 | 48.91439919 | 0.800468885755463 | 10.3850899378775 | 38.5293092512625 | Norway |
| 1992 | 49.88832739 | 0.805437473231399 | 10.2664885624765 | 39.6218388322735 | Norway |
| 1993 | 50.41607722 | 0.810565689028095 | 9.36662639238049 | 41.0494508310795 | Norway |
| 1994 | 51.8117342 | 0.815414668396239 | 10.3200437929231 | 41.4916904090769 | Norway |
| 1995 | 53.2458832 | 0.820223522303065 | 8.94759954748675 | 44.2982836568032 | Norway |
| 1996 | 54.93319649 | 0.825701668363619 | 8.46485412882163 | 46.4683423645484 | Norway |
| 1997 | 53.98649013 | 0.831468770271884 | 7.95032861196795 | 46.0361615185321 | Norway |
| 1998 | 54.70155822 | 0.836562809269057 | 6.91214448237644 | 47.7894137410136 | Norway |
| 1999 | 56.31630592 | 0.841130236756497 | 6.88998892862778 | 49.4263169901022 | Norway |
| 2000 | 57.11802272 | 0.846475451318379 | 6.152395212 | 50.9656275 | Norway |
| 2001 | 56.71736843 | 0.852111935736606 | 6.00842711544611 | 50.7089413138739 | Norway |
| 2002 | 57.39103925 | 0.856757888176133 | 6.70920812417204 | 50.681831125738 | Norway |
| 2003 | 56.39696356 | 0.860302576313355 | 6.5850987966138 | 49.8118647635962 | Norway |
| 2004 | 53.85578323 | 0.863468908330188 | 6.02348379328784 | 47.8322994376422 | Norway |
| 2005 | 57.52296667 | 0.866217487824484 | 6.18473179027568 | 51.3382348843243 | Norway |
| 2006 | 58.92903273 | 0.868588434600934 | 5.978504293 | 52.950528435628 | Norway |
| 2007 | 59.54827578 | 0.870929669588489 | 5.8351185235793 | 53.7131572525707 | Norway |
| 2008 | 62.61599647 | 0.873745121433024 | 6.41116074143852 | 56.2048357242515 | Norway |
| 2009 | 65.90085138 | 0.876582817210667 | 5.98454536627343 | 59.9163060107566 | Norway |
| 2010 | 63.43628616 | 0.880378381331352 | 5.90965354857677 | 57.5266326078532 | Norway |
| 2011 | 56.20617287 | 0.884351666714408 | 6.39406596455545 | 49.8121069075045 | Norway |
| 2012 | 46.36356837 | 0.888160475097858 | 6.2927681519354 | 40.0708002167846 | Norway |
| 2013 | 34.90735901 | 0.891989617651275 | 6.80483376062941 | 28.1025252520506 | Norway |
| 2014 | 25.47133826 | 0.895499545979859 | 5.683724018 | 19.787614237679 | Norway |
| 2015 | 20.0444557 | 0.89891264398603 | 6.3111265449185 | 13.7333291511115 | Norway |
| 2016 | 16.42448237 | 0.902584186845854 | 5.88401563299381 | 10.5404667374362 | Norway |
| 2017 | 14.91471503 | 0.906044048809343 | 5.27775039117706 | 9.63696463532294 | Norway |
| 2018 | 14.27976244 | 0.909261782035669 | 6.185221177 | 8.09454126 | Norway |
| 2019 | 14.08927311 | 0.912278760654598 | 5.76377386736368 | 8.32549924058631 | Norway |
| 2020 | 19.57706258 | 0.914529920283541 | 6.21289836623056 | 13.3641642129694 | Norway |
| 2021 | 12.1429592 | 0.916132810119013 | 4.78558061276667 | 7.35737858487333 | Norway |
| 1990 | 114.50548755589 | 0.599777756742926 | 75.4318195397005 | 39.0736680161895 | Portugal |
| 1991 | 101.15580904018 | 0.607269448643355 | 75.2922456168913 | 25.8635634232887 | Portugal |
| 1992 | 95.59783589 | 0.614808079780387 | 69.1431246103774 | 26.4547112749326 | Portugal |
| 1993 | 99.55111688 | 0.622101176817746 | 62.8455702981496 | 36.7055465825304 | Portugal |
| 1994 | 79.06795177 | 0.629306386686867 | 54.8915469970762 | 24.1764047687438 | Portugal |
| 1995 | 71.55113125 | 0.635498761462007 | 52.4861054922932 | 19.0650257527468 | Portugal |
| 1996 | 79.08263749 | 0.640864525833465 | 52.0659520869514 | 27.0166854019186 | Portugal |
| 1997 | 80.11508439 | 0.64619290440225 | 50.9408493569674 | 29.1742350373626 | Portugal |
| 1998 | 78.42394746 | 0.651335993931501 | 47.825524240048 | 30.598423221492 | Portugal |
| 1999 | 69.97495934 | 0.656120666715453 | 45.6895644645374 | 24.2853948798526 | Portugal |
| 2000 | 65.18957125 | 0.66161085244449 | 42.7382684808135 | 22.4513027666665 | Portugal |
| 2001 | 64.50145736 | 0.667247150668184 | 39.1163765081004 | 25.3850808515996 | Portugal |
| 2002 | 58.79634251 | 0.67258393952723 | 37.9643949151511 | 20.8319475953189 | Portugal |
| 2003 | 55.32448525 | 0.677663411506582 | 37.5736542386516 | 17.7508310159784 | Portugal |
| 2004 | 53.98756935 | 0.682352926478464 | 37.3181467584573 | 16.6694225933427 | Portugal |
| 2005 | 54.74079532 | 0.686887104684271 | 37.3572766283521 | 17.3835186866779 | Portugal |
| 2006 | 55.45777102 | 0.691158795071141 | 36.9060391659177 | 18.5517318505723 | Portugal |
| 2007 | 58.91237943 | 0.694974852517783 | 36.6917858384667 | 22.2205935878933 | Portugal |
| 2008 | 59.67204 | 0.69860459852573 | 35.4283450087969 | 24.2436949902331 | Portugal |
| 2009 | 60.64415756 | 0.702223398701003 | 35.5112045860304 | 25.1329529734496 | Portugal |
| 2010 | 54.7725409 | 0.706563554661067 | 34.9637480658372 | 19.8087928295528 | Portugal |
| 2011 | 52.32600387 | 0.71126289291359 | 32.5300369663588 | 19.7959669041512 | Portugal |
| 2012 | 52.7159226 | 0.715804084405661 | 31.1313501337513 | 21.5845724689087 | Portugal |
| 2013 | 50.1767659 | 0.719836991474169 | 27.6397411060737 | 22.5370247910463 | Portugal |
| 2014 | 49.00738425 | 0.723021281895034 | 28.4895634799618 | 20.5178207742182 | Portugal |
| 2015 | 50.27613243 | 0.725756220353222 | 27.457798743337 | 22.818333687833 | Portugal |
| 2016 | 51.48170237 | 0.728300849268768 | 26.1781425725808 | 25.3035598023792 | Portugal |
| 2017 | 51.5608257 | 0.73122553459241 | 23.1689055189517 | 28.3919201845183 | Portugal |
| 2018 | 50.30351198 | 0.73440294716366 | 22.4877024555707 | 27.8158095252693 | Portugal |
| 2019 | 46.73801913 | 0.738090480746574 | 18.9005524075371 | 27.8374667230829 | Portugal |
| 2020 | 41.53950226 | 0.741037384212082 | 20.4501264695167 | 21.0893757949433 | Portugal |
| 2021 | 39.88350213 | 0.744151850665712 | 20.5371808102805 | 19.3463213153395 | Portugal |
| 1990 | 47.30845586 | 0.63667316626401 | 47.1542959327931 | 0.154159925096941 | Spain |
| 1991 | 46.14878038 | 0.644130744620668 | 46.14878038 | 0 | Spain |
| 1992 | 46.70919151 | 0.65159759683081 | 45.5775731844072 | 1.13161832653275 | Spain |
| 1993 | 45.40144647 | 0.658637568907277 | 44.1614851127533 | 1.23996135497666 | Spain |
| 1994 | 43.16927012 | 0.665321549207614 | 39.8097888481075 | 3.3594812731325 | Spain |
| 1995 | 42.32371959 | 0.671576034104742 | 38.4579605935276 | 3.86575899273238 | Spain |
| 1996 | 41.48875064 | 0.677088943314314 | 38.0907306771051 | 3.39801996368488 | Spain |
| 1997 | 39.98835954 | 0.68242949710256 | 37.4382374549459 | 2.55012208606409 | Spain |
| 1998 | 39.14889858 | 0.687490716991391 | 37.3116445285812 | 1.83725404695879 | Spain |
| 1999 | 37.93749472 | 0.692245196919437 | 36.58039795 | 1.357096771 | Spain |
| 2000 | 39.045398 | 0.697056031108428 | 35.9223893372754 | 3.12300866207458 | Spain |
| 2001 | 39.64467431 | 0.701726069724186 | 35.5745260978809 | 4.07014820872909 | Spain |
| 2002 | 41.86191509 | 0.706309654864074 | 34.4475375931112 | 7.41437749951881 | Spain |
| 2003 | 44.74884823 | 0.710678584323191 | 32.9155118046639 | 11.8333364246261 | Spain |
| 2004 | 47.47478181 | 0.714886229907558 | 31.3253459131242 | 16.1494358985058 | Spain |
| 2005 | 49.51742687 | 0.718717288882423 | 28.9526570102646 | 20.5647698594754 | Spain |
| 2006 | 52.96856336 | 0.721970128302216 | 29.142488734899 | 23.8260746236811 | Spain |
| 2007 | 56.82483565 | 0.725211442824273 | 26.7140315909244 | 30.1108040637956 | Spain |
| 2008 | 57.0272647 | 0.729411041261585 | 24.0908768415966 | 32.9363878598234 | Spain |
| 2009 | 57.16052871 | 0.733956054531859 | 20.8734170572048 | 36.2871116483152 | Spain |
| 2010 | 55.59548525 | 0.738352055066564 | 22.6990682592018 | 32.8964169942682 | Spain |
| 2011 | 52.99166932 | 0.742096450004589 | 21.499782981473 | 31.491886340017 | Spain |
| 2012 | 53.72427332 | 0.745181867042751 | 19.7833766905481 | 33.9408966287219 | Spain |
| 2013 | 54.23420756 | 0.747651982075541 | 18.8849939833795 | 35.3492135735405 | Spain |
| 2014 | 56.83812478 | 0.74980092875112 | 18.7159419429086 | 38.1221828393614 | Spain |
| 2015 | 58.02735018 | 0.752269822995419 | 18.6063998177684 | 39.4209503652316 | Spain |
| 2016 | 57.0433312 | 0.75475793948614 | 17.6346240721312 | 39.4087071247688 | Spain |
| 2017 | 54.26962753 | 0.757663276271395 | 15.4297176245512 | 38.8399099056487 | Spain |
| 2018 | 50.04967965 | 0.760659574783565 | 17.7166842794553 | 32.3329953703247 | Spain |
| 2019 | 47.78486105 | 0.763942918439527 | 15.6959501880774 | 32.0889108619026 | Spain |
| 2020 | 44.81068881 | 0.766506025058573 | 13.087661918093 | 31.723026890507 | Spain |
| 2021 | 44.17682294 | 0.769283697979998 | 14.7032785108494 | 29.4735444292806 | Spain |
| 1990 | 17.43144691 | 0.78553579188163 | 11.77758204 | 5.65386487 | Sweden |
| 1991 | 17.05597276 | 0.790096440686927 | 10.7205522467685 | 6.33542051388154 | Sweden |
| 1992 | 16.9940491 | 0.795431457884408 | 11.0950211601396 | 5.89902794053038 | Sweden |
| 1993 | 16.98327764 | 0.801009521825474 | 10.8831815079424 | 6.10009613128762 | Sweden |
| 1994 | 16.41924859 | 0.806700264491967 | 9.95617378003864 | 6.46307481378136 | Sweden |
| 1995 | 16.37031031 | 0.81224465438799 | 8.66392213331035 | 7.70638818139965 | Sweden |
| 1996 | 16.45062826 | 0.817227025218912 | 8.34207409021087 | 8.10855417259913 | Sweden |
| 1997 | 16.52775124 | 0.82169405939303 | 8.09659075249406 | 8.43116049063594 | Sweden |
| 1998 | 17.06061841 | 0.825731994976955 | 7.42172093708818 | 9.63889747185182 | Sweden |
| 1999 | 17.47252707 | 0.82948709884742 | 8.56156657488826 | 8.91096049897174 | Sweden |
| 2000 | 18.2289358 | 0.833028111989963 | 7.65364254069333 | 10.5752932605167 | Sweden |
| 2001 | 20.34696438 | 0.835956398134875 | 7.35354529716577 | 12.9934190851042 | Sweden |
| 2002 | 21.68749734 | 0.838741526580818 | 7.1976828315145 | 14.4898145083355 | Sweden |
| 2003 | 23.16744001 | 0.841663138093711 | 6.28120871063417 | 16.8862312988958 | Sweden |
| 2004 | 22.33622094 | 0.844506881898062 | 6.6936062456787 | 15.6426146946513 | Sweden |
| 2005 | 23.19742681 | 0.846876343860947 | 5.06639818279286 | 18.1310286303271 | Sweden |
| 2006 | 24.09020501 | 0.849115871424855 | 6.04613111764613 | 18.0440738957239 | Sweden |
| 2007 | 25.08731932 | 0.851420847176242 | 6.02721831176167 | 19.0601010069383 | Sweden |
| 2008 | 25.57719681 | 0.853705850789255 | 6.38980037817071 | 19.1873964339493 | Sweden |
| 2009 | 29.50096094 | 0.85570274765727 | 6.00295519684375 | 23.4980057449063 | Sweden |
| 2010 | 30.85460961 | 0.858727940541151 | 5.9137083471605 | 24.9409012638395 | Sweden |
| 2011 | 29.88525168 | 0.861892379268929 | 6.09258735636536 | 23.7926643211246 | Sweden |
| 2012 | 30.83403048 | 0.864706671533252 | 6.43381610289522 | 24.4002143812548 | Sweden |
| 2013 | 31.00339687 | 0.867383767613666 | 5.793488585 | 25.209908290045 | Sweden |
| 2014 | 29.18733221 | 0.869923325091982 | 6.05199209185643 | 23.1353401176236 | Sweden |
| 2015 | 29.7227141 | 0.87238851487302 | 6.66441228955897 | 23.058301811681 | Sweden |
| 2016 | 29.72933613 | 0.874661654943114 | 7.70843258009917 | 22.0209035503708 | Sweden |
| 2017 | 27.70460003 | 0.877120611340732 | 5.73383650038419 | 21.9707635334558 | Sweden |
| 2018 | 25.25497402 | 0.879888911893351 | 6.12258738891333 | 19.1323866316567 | Sweden |
| 2019 | 23.31494407 | 0.882984115679233 | 5.90197919241556 | 17.4129648756744 | Sweden |
| 2020 | 21.82257307 | 0.88506201036603 | 6.11960368045174 | 15.7029693858883 | Sweden |
| 2021 | 19.00700194 | 0.886880298870342 | 6.84254856315941 | 12.1644533727906 | Sweden |
| 1990 | 64.05314645 | 0.862766843874175 | 6.21926242477043 | 57.8338840214296 | Switzerland |
| 1991 | 65.79721712 | 0.864796867138941 | 5.69814964152057 | 60.0990674810794 | Switzerland |
| 1992 | 66.1667952 | 0.86773607877979 | 6.04125732188182 | 60.1255378733982 | Switzerland |
| 1993 | 65.16242641 | 0.870863557610096 | 6.29584360386111 | 58.8665828073789 | Switzerland |
| 1994 | 63.35197231 | 0.873140871296934 | 5.68402961233189 | 57.6679426962181 | Switzerland |
| 1995 | 61.92490088 | 0.874835330674703 | 6.02647070021204 | 55.898430180888 | Switzerland |
| 1996 | 62.00098822 | 0.876640556809649 | 6.17135732759119 | 55.8296308885988 | Switzerland |
| 1997 | 63.57913724 | 0.87848554526934 | 5.50357040461051 | 58.0755668392495 | Switzerland |
| 1998 | 65.74382325 | 0.880035152091359 | 6.3670416872548 | 59.3767815671552 | Switzerland |
| 1999 | 67.92559771 | 0.882045586320071 | 6.60433811 | 61.3212596 | Switzerland |
| 2000 | 71.38837431 | 0.885243530890309 | 6.37128643329491 | 65.0170878746951 | Switzerland |
| 2001 | 73.35814408 | 0.888445951179249 | 6.15745675438289 | 67.2006873229871 | Switzerland |
| 2002 | 74.54095955 | 0.890902811034425 | 6.01478393499525 | 68.5261756110447 | Switzerland |
| 2003 | 77.31059514 | 0.892780807435311 | 5.98764639258575 | 71.3229487491542 | Switzerland |
| 2004 | 80.40068179 | 0.894909812216857 | 6.09643976111579 | 74.3042420311842 | Switzerland |
| 2005 | 83.62384965 | 0.897272629528173 | 6.55075546273273 | 77.0730941895173 | Switzerland |
| 2006 | 88.17824719 | 0.899962304608863 | 6.35462131439367 | 81.8236258754263 | Switzerland |
| 2007 | 88.64828716 | 0.902913412619355 | 6.39794953631854 | 82.2503376190315 | Switzerland |
| 2008 | 88.61710569 | 0.90577845443162 | 6.15727485077557 | 82.4598308405644 | Switzerland |
| 2009 | 88.40714334 | 0.908049997864177 | 6.22103202013083 | 82.1861113164192 | Switzerland |
| 2010 | 85.43302484 | 0.910530522112102 | 6.46864846210985 | 78.9643763765501 | Switzerland |
| 2011 | 81.9476908 | 0.91327657336798 | 5.90137703850164 | 76.0463137570984 | Switzerland |
| 2012 | 80.61473586 | 0.91571489343801 | 6.20316870963768 | 74.4115671509223 | Switzerland |
| 2013 | 80.59885656 | 0.917955005608177 | 6.09494502527113 | 74.5039115325189 | Switzerland |
| 2014 | 79.61624535 | 0.919994182026162 | 5.51649450980583 | 74.0997508390242 | Switzerland |
| 2015 | 79.12674914 | 0.921832990505373 | 6.01114358422275 | 73.1156055559473 | Switzerland |
| 2016 | 74.93636623 | 0.923930598455048 | 6.05985778247688 | 68.8765084466331 | Switzerland |
| 2017 | 72.21947779 | 0.926202291104072 | 6.45659158171019 | 65.7628862126998 | Switzerland |
| 2018 | 66.71507469 | 0.928550590010717 | 6.11908798132182 | 60.5959867069382 | Switzerland |
| 2019 | 62.56482276 | 0.930682101951976 | 6.29094141388128 | 56.2738813465587 | Switzerland |
| 2020 | 60.7784082 | 0.932027644512362 | 6.29577797315025 | 54.4826302313697 | Switzerland |
| 2021 | 59.33126777 | 0.933059110986638 | 5.77481201093185 | 53.5564557611981 | Switzerland |
| 1990 | 39.42817182 | 0.744334126300131 | 21.0503456948229 | 18.3778261289171 | United Kingdom |
| 1991 | 39.63803123 | 0.749148231365388 | 18.8885946263349 | 20.7494365990451 | United Kingdom |
| 1992 | 40.69179832 | 0.754556689127024 | 17.1267873979977 | 23.5650109200423 | United Kingdom |
| 1993 | 43.30226994 | 0.760154350107152 | 15.334203314519 | 27.968066624581 | United Kingdom |
| 1994 | 44.96873016 | 0.765164600469893 | 15.6106297194994 | 29.3581004407806 | United Kingdom |
| 1995 | 47.09908771 | 0.768656863963456 | 12.5796661192084 | 34.5194215888916 | United Kingdom |
| 1996 | 50.47916327 | 0.771576484075751 | 12.1026240776177 | 38.3765391967823 | United Kingdom |
| 1997 | 53.22612147 | 0.775050121968743 | 12.6978307369471 | 40.5282907283129 | United Kingdom |
| 1998 | 56.50056319 | 0.779057165114363 | 13.7138380727242 | 42.7867251187758 | United Kingdom |
| 1999 | 60.10601115 | 0.784036475142408 | 12.1000123244471 | 48.0059988241829 | United Kingdom |
| 2000 | 63.66107369 | 0.789218959863246 | 11.4485624427068 | 52.2125112439332 | United Kingdom |
| 2001 | 67.26657278 | 0.793513165354113 | 11.0966177062429 | 56.1699550714471 | United Kingdom |
| 2002 | 70.90624966 | 0.796655559574601 | 10.4938968095884 | 60.4123528533316 | United Kingdom |
| 2003 | 78.5849946 | 0.799186111437993 | 10.1455521922481 | 68.4394424123019 | United Kingdom |
| 2004 | 87.13646229 | 0.801950870000537 | 10.3660097697915 | 76.7704525183286 | United Kingdom |
| 2005 | 105.54908368037 | 0.804489193801038 | 10.3484709699819 | 95.2006127103881 | United Kingdom |
| 2006 | 131.9707421 | 0.806817045960102 | 9.62804336963163 | 122.342698687668 | United Kingdom |
| 2007 | 136.3214639 | 0.80935277163371 | 9.411849318 | 126.9096146 | United Kingdom |
| 2008 | 115.16960801279 | 0.812039287236426 | 9.14340432128098 | 106.026203691509 | United Kingdom |
| 2009 | 94.21139773 | 0.814793642346203 | 8.79574838188435 | 85.4156493523556 | United Kingdom |
| 2010 | 79.16866844 | 0.818033993642746 | 8.63228357504209 | 70.5363848615079 | United Kingdom |
| 2011 | 66.46634717 | 0.821718995421102 | 7.67200394476409 | 58.7943432225259 | United Kingdom |
| 2012 | 49.21838318 | 0.826453754133025 | 7.57631766536647 | 41.6420655162235 | United Kingdom |
| 2013 | 33.22832695 | 0.831508668228002 | 7.89434278657061 | 25.3339841607394 | United Kingdom |
| 2014 | 21.48354198 | 0.835516642361539 | 7.3619925058421 | 14.1215494788779 | United Kingdom |
| 2015 | 16.21669224 | 0.838951918781599 | 7.81425829082727 | 8.40243395108273 | United Kingdom |
| 2016 | 13.90614554 | 0.842460232628565 | 5.10673848034321 | 8.79940705800679 | United Kingdom |
| 2017 | 11.50749454 | 0.846263617759747 | 7.85508822 | 3.652406316 | United Kingdom |
| 2018 | 9.803040029 | 0.849969787501756 | 7.10072645183235 | 2.70231357671765 | United Kingdom |
| 2019 | 9.091441128 | 0.854130572600886 | 7.163640193 | 1.927800935 | United Kingdom |
| 2020 | 7.583325437 | 0.856920290432659 | 5.73384449343471 | 1.84948094370529 | United Kingdom |
| 2021 | 7.642941398 | 0.859000181772086 | 5.23763857083556 | 2.40530282705444 | United Kingdom |
| 1990 | 608.45493435976 | 0.587397284272094 | 78.9842346423705 | 529.47069971739 | Argentina |
| 1991 | 548.94298189272 | 0.591707540464012 | 80.6548407184145 | 468.288141174306 | Argentina |
| 1992 | 467.27705129643 | 0.597996324377555 | 77.7221245177812 | 389.554926778649 | Argentina |
| 1993 | 402.60842547067 | 0.603739312309972 | 76.083795668436 | 326.524629802234 | Argentina |
| 1994 | 381.48743543111 | 0.609787330316145 | 70.5694625244619 | 310.917972906648 | Argentina |
| 1995 | 398.27513461497 | 0.614473705638113 | 67.754281957694 | 330.520852657276 | Argentina |
| 1996 | 392.11788269345 | 0.618942623582172 | 65.1887108214145 | 326.929171872036 | Argentina |
| 1997 | 424.31595115645 | 0.623934408870184 | 58.8360533160227 | 365.479897840427 | Argentina |
| 1998 | 407.37081998908 | 0.6287234356344 | 55.8015760718152 | 351.569243917265 | Argentina |
| 1999 | 354.00920925916 | 0.633175129405293 | 54.5240433043706 | 299.485165954789 | Argentina |
| 2000 | 312.23029996944 | 0.638194471801002 | 50.5509668699314 | 261.679333099509 | Argentina |
| 2001 | 297.09015398815 | 0.642175704746832 | 50.8878660720736 | 246.202287916076 | Argentina |
| 2002 | 300.58970695526 | 0.644472506550469 | 50.0225078883093 | 250.567199066951 | Argentina |
| 2003 | 251.51699629218 | 0.645906577030896 | 51.0522775963444 | 200.464718695836 | Argentina |
| 2004 | 198.72333724057 | 0.648296828210031 | 49.2500676115006 | 149.473269629069 | Argentina |
| 2005 | 180.76795683946 | 0.653368829991767 | 45.7512668339635 | 135.016690005497 | Argentina |
| 2006 | 176.61376298464 | 0.657445899915566 | 46.0913681949082 | 130.522394789732 | Argentina |
| 2007 | 167.93013609907 | 0.660187575972401 | 43.3513200218802 | 124.57881607719 | Argentina |
| 2008 | 171.06131909703 | 0.6630792353937 | 40.5990240206878 | 130.462295076342 | Argentina |
| 2009 | 170.11191722053 | 0.665911944148118 | 38.9775443378695 | 131.134372882661 | Argentina |
| 2010 | 168.74417718533 | 0.669925880151809 | 38.5393685102507 | 130.204808675079 | Argentina |
| 2011 | 150.25236591637 | 0.675242697368294 | 38.1458535229346 | 112.106512393435 | Argentina |
| 2012 | 120.49759629336 | 0.679218239775544 | 37.5818091356519 | 82.9157871577081 | Argentina |
| 2013 | 106.91952248848 | 0.681633214735039 | 37.472051320347 | 69.447471168133 | Argentina |
| 2014 | 94.56221621 | 0.684974509115572 | 37.183871883008 | 57.378344323242 | Argentina |
| 2015 | 80.05746523 | 0.691710125973835 | 36.6358573703665 | 43.4216078580535 | Argentina |
| 2016 | 70.85118757 | 0.698618031454613 | 35.7167496756198 | 35.1344378970102 | Argentina |
| 2017 | 61.75665023 | 0.705135528765331 | 35.3503182990983 | 26.4063319260417 | Argentina |
| 2018 | 51.76873278 | 0.712888693454577 | 32.5089815046217 | 19.2597512730683 | Argentina |
| 2019 | 49.27189614 | 0.719002842751183 | 29.5971779627428 | 19.6747181751272 | Argentina |
| 2020 | 47.48590425 | 0.721294302628992 | 29.3378007838642 | 18.1481034701758 | Argentina |
| 2021 | 46.48558558 | 0.723122972582249 | 27.840859419475 | 18.644726164935 | Argentina |
| 1990 | 351.38425157252 | 0.586495099638694 | 84.2445406190295 | 267.13971095349 | Chile |
| 1991 | 298.09262536849 | 0.593006580479257 | 79.9728368683553 | 218.119788500135 | Chile |
| 1992 | 250.036396 | 0.599106083381125 | 75.788420722803 | 174.247975261597 | Chile |
| 1993 | 204.04158498384 | 0.604828509357091 | 74.4211552490124 | 129.620429734828 | Chile |
| 1994 | 188.09198439465 | 0.61067393285511 | 70.1547078328979 | 117.937276561752 | Chile |
| 1995 | 192.61558654166 | 0.617234676665645 | 63.688609254632 | 128.926977287028 | Chile |
| 1996 | 193.59234195406 | 0.623694459543547 | 61.5670422751992 | 132.025299678861 | Chile |
| 1997 | 186.56335645909 | 0.630540084786116 | 56.4268434019102 | 130.13651305718 | Chile |
| 1998 | 177.52306515923 | 0.638082758454141 | 51.8761455190442 | 125.646919640186 | Chile |
| 1999 | 163.46934336654 | 0.645928084390867 | 49.5058595359431 | 113.963483830597 | Chile |
| 2000 | 148.69609395161 | 0.652989027528663 | 45.8336528313192 | 102.862441120291 | Chile |
| 2001 | 139.45419890293 | 0.660265510120952 | 42.7018310196923 | 96.7523678832377 | Chile |
| 2002 | 135.38370881798 | 0.667888408106028 | 39.2862467816078 | 96.0974620363722 | Chile |
| 2003 | 128.02105268299 | 0.673445927084645 | 37.8663821456735 | 90.1546705373165 | Chile |
| 2004 | 115.92332922223 | 0.678258212006936 | 37.7241195822539 | 78.1992096399761 | Chile |
| 2005 | 102.46642632566 | 0.682589191171846 | 37.5535748102883 | 64.9128515153717 | Chile |
| 2006 | 94.58147077 | 0.685892748248682 | 37.271222532745 | 57.310248238395 | Chile |
| 2007 | 87.95470251 | 0.688930323304188 | 37.3518947861062 | 50.6028077200638 | Chile |
| 2008 | 89.00626373 | 0.692359787453618 | 36.5974283012285 | 52.4088354287915 | Chile |
| 2009 | 88.38804194 | 0.696602140685353 | 35.600558427494 | 52.787483513646 | Chile |
| 2010 | 81.34757714 | 0.702304703544999 | 35.3788225034433 | 45.9687546354467 | Chile |
| 2011 | 78.17709003 | 0.708597935629908 | 34.4309577877631 | 43.7461322394869 | Chile |
| 2012 | 75.57594206 | 0.714743477340726 | 31.9168925323198 | 43.6590495236702 | Chile |
| 2013 | 75.51208208 | 0.720448781307689 | 28.5968281254894 | 46.9152539584006 | Chile |
| 2014 | 84.73527559 | 0.727275926904869 | 26.5024027590461 | 58.2328728319239 | Chile |
| 2015 | 79.52655497 | 0.736098082475337 | 21.4797592058819 | 58.0467957637781 | Chile |
| 2016 | 72.80051169 | 0.744972224319701 | 19.4657105572757 | 53.3348011347943 | Chile |
| 2017 | 74.4591523 | 0.752058077213427 | 18.5224545724333 | 55.9366977290267 | Chile |
| 2018 | 73.11086589 | 0.758625713610628 | 16.616322295987 | 56.494543597043 | Chile |
| 2019 | 70.59840982 | 0.765120322816907 | 14.6245487580425 | 55.9738610572175 | Chile |
| 2020 | 59.36874458 | 0.769213676196926 | 14.5531138080149 | 44.8156307671451 | Chile |
| 2021 | 56.1937126 | 0.771514715903929 | 13.0565818839149 | 43.1371307131051 | Chile |
| 1990 | 615.48348003731 | 0.58192185512198 | 85.3882286784272 | 530.095251358883 | Uruguay |
| 1991 | 554.56414080374 | 0.586156118239456 | 79.0935140229636 | 475.470626780776 | Uruguay |
| 1992 | 446.31969798386 | 0.589822355398741 | 80.274076113798 | 366.045621870062 | Uruguay |
| 1993 | 431.10346355428 | 0.592295025274764 | 79.0048280218497 | 352.09863553243 | Uruguay |
| 1994 | 433.29674400059 | 0.595188424402801 | 77.8819734032583 | 355.414770597332 | Uruguay |
| 1995 | 466.64712829286 | 0.596825409130546 | 78.5961430616606 | 388.050985231199 | Uruguay |
| 1996 | 468.94676457934 | 0.5997751037831 | 75.207125726965 | 393.739638852375 | Uruguay |
| 1997 | 491.75300763559 | 0.605665051157748 | 76.8508100696103 | 414.90219756598 | Uruguay |
| 1998 | 440.05391305522 | 0.612700815160604 | 68.967734238008 | 371.086178817212 | Uruguay |
| 1999 | 378.91175737265 | 0.619009271243139 | 63.1363876127003 | 315.77536975995 | Uruguay |
| 2000 | 328.88595842204 | 0.623208290785701 | 65.5297110921275 | 263.356247329913 | Uruguay |
| 2001 | 302.54532103597 | 0.62579606753003 | 59.0767398292449 | 243.468581206725 | Uruguay |
| 2002 | 310.91188422967 | 0.628285799994443 | 55.7435547024287 | 255.168329527241 | Uruguay |
| 2003 | 318.62337981325 | 0.631437773906681 | 53.6743063371737 | 264.949073476076 | Uruguay |
| 2004 | 282.68061747222 | 0.634216906381842 | 53.2466399782926 | 229.433977493927 | Uruguay |
| 2005 | 249.73230243485 | 0.63697576957158 | 51.4526131936983 | 198.279689241152 | Uruguay |
| 2006 | 207.17624221244 | 0.640590696178162 | 51.262418912572 | 155.913823299868 | Uruguay |
| 2007 | 212.96095361582 | 0.64633171377937 | 50.668342623423 | 162.292610992397 | Uruguay |
| 2008 | 206.21380906225 | 0.653525522497867 | 45.5281368740216 | 160.685672188228 | Uruguay |
| 2009 | 202.14584945782 | 0.660321134636261 | 43.6156493826528 | 158.530200075167 | Uruguay |
| 2010 | 214.00112640119 | 0.666099135301691 | 39.2652527049782 | 174.735873696212 | Uruguay |
| 2011 | 202.26587058715 | 0.667079733158413 | 39.2829568737596 | 162.98291371339 | Uruguay |
| 2012 | 187.17817655278 | 0.665802191838499 | 38.9741455019785 | 148.204031050802 | Uruguay |
| 2013 | 183.16136465343 | 0.668228360836938 | 39.3349045529494 | 143.826460100481 | Uruguay |
| 2014 | 159.30885977285 | 0.673334260363154 | 38.189281747787 | 121.119578025063 | Uruguay |
| 2015 | 144.07491602404 | 0.680595506073599 | 37.5403852048747 | 106.534530819165 | Uruguay |
| 2016 | 135.49523063546 | 0.689816733014055 | 37.25835526 | 98.23687537 | Uruguay |
| 2017 | 125.15827914214 | 0.699189455881847 | 36.0548653856198 | 89.1034137565202 | Uruguay |
| 2018 | 122.418147 | 0.706904930126484 | 33.9098835294308 | 88.5082634730693 | Uruguay |
| 2019 | 120.55150512482 | 0.712536588078123 | 32.1618810702541 | 88.3896240545659 | Uruguay |
| 2020 | 116.6084654 | 0.716169766093523 | 31.4771194090776 | 85.1313460360224 | Uruguay |
| 2021 | 108.63310603166 | 0.719283444839432 | 28.7523062958349 | 79.8807997358251 | Uruguay |
| 1990 | 35.09975352 | 0.781977864427607 | 11.5925864844722 | 23.5071670350478 | Canada |
| 1991 | 35.07184397 | 0.784159360080438 | 10.8827738833606 | 24.1890700841194 | Canada |
| 1992 | 34.56508547 | 0.787030536551619 | 12.0910308099097 | 22.4740546580803 | Canada |
| 1993 | 37.27083418 | 0.789716593022703 | 10.9847716449362 | 26.2860625384638 | Canada |
| 1994 | 37.10440794 | 0.79259851803789 | 10.76346587 | 26.34094207 | Canada |
| 1995 | 37.4064549 | 0.796521165940602 | 11.2930200583207 | 26.1134348424393 | Canada |
| 1996 | 37.21723777 | 0.801064970257054 | 10.1232173710807 | 27.0940203959893 | Canada |
| 1997 | 37.96940065 | 0.804833356387694 | 9.74730515289149 | 28.2220955013985 | Canada |
| 1998 | 39.52340483 | 0.808135440512777 | 9.27950350152552 | 30.2439013299645 | Canada |
| 1999 | 43.70071902 | 0.812265940092679 | 9.27223413241312 | 34.4284848917769 | Canada |
| 2000 | 49.71758935 | 0.816685059408778 | 8.6007404223495 | 41.1168489318705 | Canada |
| 2001 | 54.71569095 | 0.820696250274983 | 8.15509094925163 | 46.5605999958284 | Canada |
| 2002 | 62.37052274 | 0.824402271645546 | 7.91440289680918 | 54.4561198476208 | Canada |
| 2003 | 68.63225054 | 0.827801096249711 | 7.57110611622386 | 61.0611444209861 | Canada |
| 2004 | 76.76731209 | 0.830928109937158 | 7.52226493435635 | 69.2450471550637 | Canada |
| 2005 | 85.27211962 | 0.833288293645254 | 7.13987857929872 | 78.1322410375613 | Canada |
| 2006 | 88.87238542 | 0.834915660275685 | 7.2596911824037 | 81.6126942353763 | Canada |
| 2007 | 91.78197757 | 0.836625096194775 | 6.76809839462544 | 85.0138791759846 | Canada |
| 2008 | 91.78518813 | 0.838913684557505 | 6.4932013101682 | 85.2919868165818 | Canada |
| 2009 | 88.70167585 | 0.841583670650067 | 6.05310940902294 | 82.6485664437871 | Canada |
| 2010 | 87.21638156 | 0.844853488930283 | 6.06602629613373 | 81.1503552641963 | Canada |
| 2011 | 82.72979186 | 0.848017833149098 | 6.271329817 | 76.45846204 | Canada |
| 2012 | 79.87097627 | 0.850978048108266 | 5.28502349642275 | 74.5859527779972 | Canada |
| 2013 | 77.77602976 | 0.853844945122214 | 6.22423386997736 | 71.5517958907626 | Canada |
| 2014 | 71.05126534 | 0.856759933938005 | 5.70900989110185 | 65.3422554517681 | Canada |
| 2015 | 67.12352354 | 0.859532903766548 | 5.85590141075708 | 61.2676221250129 | Canada |
| 2016 | 56.17047751 | 0.862174225749794 | 6.19125060009105 | 49.979226911819 | Canada |
| 2017 | 41.16313464 | 0.865053829759503 | 6.00058903949703 | 35.162545603353 | Canada |
| 2018 | 30.13750402 | 0.867905541356997 | 6.06448566156034 | 24.0730183578297 | Canada |
| 2019 | 23.56987818 | 0.870554479228955 | 6.08695126566568 | 17.4829269160443 | Canada |
| 2020 | 25.99044879 | 0.871992631299453 | 6.10611159479222 | 19.8843371919478 | Canada |
| 2021 | 21.92448531 | 0.873170679735405 | 6.52224645377409 | 15.4022388551859 | Canada |
| 1990 | 55.55004815 | 0.763647690398232 | 14.9683900116764 | 40.5816581417136 | United States of America |
| 1991 | 54.93786706 | 0.766044294817203 | 14.621096909742 | 40.316770153808 | United States of America |
| 1992 | 50.39305268 | 0.769595491571717 | 13.0001579648457 | 37.3928947142243 | United States of America |
| 1993 | 47.33598585 | 0.773103516745741 | 13.7834984212328 | 33.5524874245572 | United States of America |
| 1994 | 50.45114484 | 0.776368587517635 | 13.1080105843585 | 37.3431342520615 | United States of America |
| 1995 | 49.1924691 | 0.779410253958491 | 10.4405808242314 | 38.7518882730486 | United States of America |
| 1996 | 51.94338213 | 0.781934769815664 | 11.3362704706504 | 40.6071116574496 | United States of America |
| 1997 | 55.42112932 | 0.783979277617936 | 10.8337133172385 | 44.5874160053315 | United States of America |
| 1998 | 57.97119155 | 0.786076331639174 | 10.7432867981343 | 47.2279047502757 | United States of America |
| 1999 | 53.90961127 | 0.788544535320099 | 11.7151935071604 | 42.1944177582796 | United States of America |
| 2000 | 55.50011419 | 0.792316650292886 | 10.9360103918173 | 44.5641037934627 | United States of America |
| 2001 | 56.40304474 | 0.796676792932995 | 11.2146437318349 | 45.1884010105651 | United States of America |
| 2002 | 58.43991296 | 0.800403318331674 | 10.0522650578595 | 48.3876479022005 | United States of America |
| 2003 | 61.45932318 | 0.803228024762672 | 10.4937718212873 | 50.9655513546727 | United States of America |
| 2004 | 60.02536114 | 0.805304692151156 | 10.1269133622947 | 49.8984477766853 | United States of America |
| 2005 | 49.81799127 | 0.80603307970225 | 9.37674332437098 | 40.441247943169 | United States of America |
| 2006 | 42.24315941 | 0.80678358318377 | 10.1682587084354 | 32.0749007060146 | United States of America |
| 2007 | 37.24458109 | 0.809688049965476 | 9.62686245469108 | 27.6177186400589 | United States of America |
| 2008 | 31.14652356 | 0.814577102408992 | 9.85161807040046 | 21.2949054850395 | United States of America |
| 2009 | 25.91123894 | 0.820294574449102 | 7.80054926787818 | 18.1106896715118 | United States of America |
| 2010 | 22.72669395 | 0.825884658277638 | 8.19123525368281 | 14.5354586973272 | United States of America |
| 2011 | 22.40055354 | 0.830266387766787 | 7.801930941 | 14.598622599514 | United States of America |
| 2012 | 21.59002863 | 0.833958192158326 | 6.86214851474923 | 14.7278801140708 | United States of America |
| 2013 | 21.20267198 | 0.837412988286127 | 7.04414878227816 | 14.1585231979418 | United States of America |
| 2014 | 19.68696934 | 0.84074652832772 | 5.86003947424278 | 13.8269298652972 | United States of America |
| 2015 | 19.69270063 | 0.844370336219248 | 5.58166848988682 | 14.1110321434132 | United States of America |
| 2016 | 18.48894987 | 0.848015521315013 | 6.8485401741875 | 11.6404096921225 | United States of America |
| 2017 | 16.08622553 | 0.851630299087012 | 6.5585639879868 | 9.5276615453132 | United States of America |
| 2018 | 14.39791696 | 0.855049193484192 | 4.79082694372067 | 9.60709001519933 | United States of America |
| 2019 | 13.5824562 | 0.858578064552935 | 6.061402261 | 7.521053941 | United States of America |
| 2020 | 15.74358728 | 0.860792773409847 | 6.21301569228546 | 9.53057158555454 | United States of America |
| 2021 | 15.11978072 | 0.862448354015145 | 5.77496444949375 | 9.34481627145625 | United States of America |
| 1990 | 356.96373746091 | 0.612104590576197 | 70.2666807165639 | 286.697056744346 | Antigua and Barbuda |
| 1991 | 320.956611 | 0.618817128671612 | 63.2513755315101 | 257.70523544549 | Antigua and Barbuda |
| 1992 | 316.84040898117 | 0.624695818117472 | 57.9271839758676 | 258.913225005302 | Antigua and Barbuda |
| 1993 | 338.22784528578 | 0.630075853661038 | 56.3747221687519 | 281.853123117028 | Antigua and Barbuda |
| 1994 | 328.44495872757 | 0.634768955380056 | 54.1862370345399 | 274.25872169303 | Antigua and Barbuda |
| 1995 | 329.04547515392 | 0.638008628575253 | 51.33155649 | 277.71391866236 | Antigua and Barbuda |
| 1996 | 322.74842929589 | 0.641647192231223 | 50.0148917167385 | 272.733537579151 | Antigua and Barbuda |
| 1997 | 300.23666103586 | 0.645895590676441 | 51.1788151789998 | 249.05784585686 | Antigua and Barbuda |
| 1998 | 273.02953116647 | 0.650614287301836 | 50.2493789878399 | 222.78015217863 | Antigua and Barbuda |
| 1999 | 240.41395926258 | 0.655534994042462 | 45.6078021940353 | 194.806157068545 | Antigua and Barbuda |
| 2000 | 208.20398416242 | 0.660819064213085 | 42.9205132648804 | 165.28347089754 | Antigua and Barbuda |
| 2001 | 174.22446362712 | 0.665620631875941 | 39.3496740191588 | 134.874789607961 | Antigua and Barbuda |
| 2002 | 159.54657424574 | 0.670360801218314 | 38.4968443796326 | 121.049729866107 | Antigua and Barbuda |
| 2003 | 158.05936653696 | 0.67531148966572 | 38.331091500427 | 119.728275036533 | Antigua and Barbuda |
| 2004 | 158.64724825981 | 0.680412429142464 | 37.8605008062663 | 120.786747453544 | Antigua and Barbuda |
| 2005 | 168.72166083819 | 0.685486437018442 | 37.5786082680293 | 131.143052570161 | Antigua and Barbuda |
| 2006 | 168.74021526377 | 0.690826021180964 | 36.8283413959893 | 131.911873867781 | Antigua and Barbuda |
| 2007 | 167.92063114444 | 0.69636003908324 | 36.5479268975542 | 131.372704246886 | Antigua and Barbuda |
| 2008 | 159.61370927983 | 0.702175362144575 | 35.9049223946178 | 123.708786885212 | Antigua and Barbuda |
| 2009 | 150.00193965284 | 0.707128563802673 | 34.4404935355967 | 115.561446117243 | Antigua and Barbuda |
| 2010 | 144.6044898 | 0.711432245950457 | 32.0871443391189 | 112.517345479481 | Antigua and Barbuda |
| 2011 | 138.12641183544 | 0.715489989160124 | 31.6754719921128 | 106.450939843327 | Antigua and Barbuda |
| 2012 | 133.31030696207 | 0.719509323328322 | 28.980805912816 | 104.329501049254 | Antigua and Barbuda |
| 2013 | 135.31192334741 | 0.722864977889834 | 27.8611044045554 | 107.450818942855 | Antigua and Barbuda |
| 2014 | 142.59226886637 | 0.725585978309816 | 26.4874229348641 | 116.104845931506 | Antigua and Barbuda |
| 2015 | 137.12946084751 | 0.728372762315346 | 27.2389454531353 | 109.890515394375 | Antigua and Barbuda |
| 2016 | 133.34174106434 | 0.731598943677227 | 24.1294450457255 | 109.212296018614 | Antigua and Barbuda |
| 2017 | 131.70216163352 | 0.734963713327539 | 22.0951100323221 | 109.607051601198 | Antigua and Barbuda |
| 2018 | 128.30899855454 | 0.738879105607608 | 18.7847960261306 | 109.524202528409 | Antigua and Barbuda |
| 2019 | 125.31636118738 | 0.743052960248658 | 20.4210397781281 | 104.895321409252 | Antigua and Barbuda |
| 2020 | 117.10906872638 | 0.746345330021488 | 20.0449741818217 | 97.0640945445583 | Antigua and Barbuda |
| 2021 | 110.10212918915 | 0.749886887369379 | 19.2226513106171 | 90.8794778785329 | Antigua and Barbuda |
| 1990 | 459.73479680061 | 0.693509268349695 | 36.6037394042087 | 423.131057396401 | Bahamas |
| 1991 | 487.96659959208 | 0.689126783528471 | 37.1653850807383 | 450.801214511342 | Bahamas |
| 1992 | 486.61467569383 | 0.68734383814569 | 37.1768960147632 | 449.437779679067 | Bahamas |
| 1993 | 448.87420364054 | 0.693816501452916 | 36.7245347196634 | 412.149668920877 | Bahamas |
| 1994 | 369.28871724243 | 0.706819891740348 | 34.4573770965244 | 334.831340145906 | Bahamas |
| 1995 | 315.7783327 | 0.71938160672878 | 30.3708474419693 | 285.407485245831 | Bahamas |
| 1996 | 272.88544648222 | 0.728478844477514 | 26.3981614012794 | 246.487285080941 | Bahamas |
| 1997 | 250.70787394196 | 0.734781214819761 | 21.4033091823508 | 229.304564759609 | Bahamas |
| 1998 | 229.55728791221 | 0.739281293356304 | 20.2145299108208 | 209.342758001389 | Bahamas |
| 1999 | 209.40691958847 | 0.741622847933781 | 19.8705853441951 | 189.536334244275 | Bahamas |
| 2000 | 185.47007781556 | 0.742517946132475 | 20.5139769263673 | 164.956100889193 | Bahamas |
| 2001 | 153.84408281239 | 0.743592881699638 | 19.2778386314227 | 134.566244180967 | Bahamas |
| 2002 | 142.40016470576 | 0.74625032205653 | 19.7514759752727 | 122.648688730487 | Bahamas |
| 2003 | 135.16387534848 | 0.74991671397758 | 18.8870762948373 | 116.276799053643 | Bahamas |
| 2004 | 131.82267350695 | 0.753759643287481 | 18.1284046608335 | 113.694268846117 | Bahamas |
| 2005 | 134.64959078341 | 0.756782661013009 | 16.0539815713309 | 118.595609212079 | Bahamas |
| 2006 | 131.87830086797 | 0.759185938528465 | 16.0378725952345 | 115.840428272735 | Bahamas |
| 2007 | 129.74669392023 | 0.761990996911577 | 15.390132658154 | 114.356561262076 | Bahamas |
| 2008 | 122.110971 | 0.765590503207612 | 15.0510152842374 | 107.059955728663 | Bahamas |
| 2009 | 114.7504532 | 0.769561673980251 | 13.1013894871533 | 101.649063703547 | Bahamas |
| 2010 | 103.76735831884 | 0.774119809925167 | 12.4886168767677 | 91.2787414420723 | Bahamas |
| 2011 | 100.68270113833 | 0.778281890749903 | 11.4070924854521 | 89.2756086528779 | Bahamas |
| 2012 | 93.48337466 | 0.781838639259066 | 11.6329959166584 | 81.8503787418116 | Bahamas |
| 2013 | 93.52471823 | 0.784623910745264 | 11.139553312676 | 82.385164917194 | Bahamas |
| 2014 | 98.80869356 | 0.787253492401512 | 11.4908328944279 | 87.3178606664721 | Bahamas |
| 2015 | 86.72407579 | 0.789956145087734 | 11.1102055374926 | 75.6138702523074 | Bahamas |
| 2016 | 76.88065653 | 0.792738695555901 | 10.91027051 | 65.97038602 | Bahamas |
| 2017 | 62.43716658 | 0.795347180655707 | 10.7240317416916 | 51.7131348340384 | Bahamas |
| 2018 | 49.77504186 | 0.798179034346548 | 10.6796568925815 | 39.0953849639785 | Bahamas |
| 2019 | 43.90185883 | 0.801033070483021 | 10.6750632954021 | 33.2267955296679 | Bahamas |
| 2020 | 48.98773936 | 0.802948018163753 | 10.2677471027633 | 38.7199922561067 | Bahamas |
| 2021 | 36.75461445 | 0.805020667882124 | 9.95046894769308 | 26.8041455027569 | Bahamas |
| 1990 | 299.14721622532 | 0.653582516816854 | 45.7977228473456 | 253.349493377974 | Barbados |
| 1991 | 281.71658894409 | 0.656654073238155 | 45.2203550484342 | 236.496233895656 | Barbados |
| 1992 | 250.13915134326 | 0.662446635199289 | 43.1547392198932 | 206.984412123367 | Barbados |
| 1993 | 230.3316692 | 0.668815011829235 | 38.9885602932723 | 191.343108909328 | Barbados |
| 1994 | 232.17311285281 | 0.673409365188399 | 38.0949751791279 | 194.078137673682 | Barbados |
| 1995 | 235.44712822143 | 0.676012246725867 | 37.8432216031289 | 197.603906618301 | Barbados |
| 1996 | 234.76528101755 | 0.677193053529371 | 38.019219314547 | 196.746061703003 | Barbados |
| 1997 | 211.3433202 | 0.678644826009168 | 37.6009695088833 | 173.742350730717 | Barbados |
| 1998 | 164.26606068457 | 0.679827167153174 | 37.5148972226272 | 126.751163461943 | Barbados |
| 1999 | 147.83087066269 | 0.680398459240343 | 37.5422253923737 | 110.288645270316 | Barbados |
| 2000 | 140.25859935657 | 0.681304198777811 | 37.6487084255058 | 102.609890931064 | Barbados |
| 2001 | 106.31653945394 | 0.683329389407697 | 37.3267574381297 | 68.9897820158103 | Barbados |
| 2002 | 108.65773171702 | 0.68780757593531 | 37.3328726869452 | 71.3248590300748 | Barbados |
| 2003 | 124.8017348 | 0.693235961982327 | 36.5488018216663 | 88.2529329638337 | Barbados |
| 2004 | 100.36015131825 | 0.698029702584033 | 35.9984093855047 | 64.3617419327453 | Barbados |
| 2005 | 108.28374636842 | 0.701510049493398 | 35.6571127629125 | 72.6266336055075 | Barbados |
| 2006 | 108.13794416253 | 0.703856185878429 | 34.8302971493661 | 73.3076470131639 | Barbados |
| 2007 | 101.82564418723 | 0.705871649427123 | 34.5490769453396 | 67.2765672418904 | Barbados |
| 2008 | 97.01946983 | 0.70851449513133 | 34.4242448391429 | 62.5952249883671 | Barbados |
| 2009 | 90.01306626 | 0.712143361929882 | 32.0320996942809 | 57.9809665652691 | Barbados |
| 2010 | 86.99428001 | 0.716208546340619 | 30.5942718733111 | 56.4000081331489 | Barbados |
| 2011 | 96.27458452 | 0.720060687326812 | 29.4264672603142 | 66.8481172638958 | Barbados |
| 2012 | 76.85670865 | 0.724162457760429 | 28.1082317939107 | 48.7484768554593 | Barbados |
| 2013 | 78.76076071 | 0.727826170485545 | 25.2320802563624 | 53.5286804538776 | Barbados |
| 2014 | 78.6446103 | 0.730383286769139 | 25.0825792745909 | 53.5620310233691 | Barbados |
| 2015 | 77.9035851 | 0.73260299258046 | 23.3102022123895 | 54.5933828843805 | Barbados |
| 2016 | 76.11205842 | 0.73491253498334 | 21.3946393146343 | 54.7174191035257 | Barbados |
| 2017 | 75.17977574 | 0.737157255293201 | 21.3902209466633 | 53.7895547892067 | Barbados |
| 2018 | 72.22213172 | 0.739531436514553 | 20.3707367569944 | 51.8513949611956 | Barbados |
| 2019 | 70.9029697 | 0.742239308607991 | 19.6022450288398 | 51.3007246673502 | Barbados |
| 2020 | 68.9896946 | 0.74436664552373 | 19.2340075202999 | 49.7556870800101 | Barbados |
| 2021 | 68.46093951 | 0.746748764251474 | 18.879778351691 | 49.581161157249 | Barbados |
| 1990 | 2805.55160568942 | 0.423726992172569 | 686.302011176769 | 2119.24959451265 | Belize |
| 1991 | 2347.32320723185 | 0.433834392305066 | 418.381282980024 | 1928.94192425183 | Belize |
| 1992 | 2204.22509344594 | 0.444198507291524 | 422.85181658416 | 1781.37327686178 | Belize |
| 1993 | 2062.35988763395 | 0.455258576782351 | 402.876218227457 | 1659.48366940649 | Belize |
| 1994 | 1773.55114739534 | 0.465729308156419 | 357.152327570972 | 1416.39881982437 | Belize |
| 1995 | 1661.78916907972 | 0.475278511253692 | 335.307778934808 | 1326.48139014491 | Belize |
| 1996 | 1576.29791566092 | 0.482702164726655 | 322.989113943673 | 1253.30880171725 | Belize |
| 1997 | 1506.93737597236 | 0.488020129434782 | 312.236964700642 | 1194.70041127172 | Belize |
| 1998 | 1401.65659688881 | 0.491819335269081 | 300.425552804106 | 1101.2310440847 | Belize |
| 1999 | 1192.29076855355 | 0.495070195578772 | 281.937964596618 | 910.352803956931 | Belize |
| 2000 | 992.66315004359 | 0.498643730395849 | 236.324382154317 | 756.338767889273 | Belize |
| 2001 | 799.05815451474 | 0.502399433843715 | 228.122439989464 | 570.935714525276 | Belize |
| 2002 | 684.0879381 | 0.506871655304811 | 220.150692488563 | 463.937245641137 | Belize |
| 2003 | 693.45251368856 | 0.512679459131973 | 206.433472608429 | 487.019041080131 | Belize |
| 2004 | 661.64203530822 | 0.519697333321487 | 199.333016853499 | 462.309018454721 | Belize |
| 2005 | 693.7481867 | 0.527231061781742 | 188.834418441549 | 504.913768250451 | Belize |
| 2006 | 633.08969232795 | 0.534889621081104 | 162.896687250431 | 470.193005077519 | Belize |
| 2007 | 580.15285516588 | 0.541900694400229 | 155.22616886271 | 424.92668630317 | Belize |
| 2008 | 546.59022271254 | 0.548093223354439 | 150.896795334149 | 395.693427378391 | Belize |
| 2009 | 516.59967771268 | 0.553465869255233 | 143.903416223951 | 372.696261488729 | Belize |
| 2010 | 441.70556649446 | 0.558546893327125 | 141.603352286747 | 300.102214207713 | Belize |
| 2011 | 421.56543717024 | 0.563440362640477 | 138.998356129586 | 282.567081040654 | Belize |
| 2012 | 377.02968496907 | 0.568456235800016 | 111.537946273826 | 265.491738695244 | Belize |
| 2013 | 367.1127705 | 0.573236457430755 | 80.6452769793145 | 286.467493507686 | Belize |
| 2014 | 383.88537652624 | 0.578091325788653 | 83.2345737121108 | 300.650802814129 | Belize |
| 2015 | 378.0173401 | 0.583279094493031 | 82.664979289411 | 295.352360774789 | Belize |
| 2016 | 367.7271584 | 0.588275912195168 | 81.6868104579471 | 286.040347927753 | Belize |
| 2017 | 350.50259610398 | 0.593337290122785 | 76.7320783398211 | 273.770517764159 | Belize |
| 2018 | 340.34493574847 | 0.598512014950432 | 77.7169425448085 | 262.627993203662 | Belize |
| 2019 | 322.29598443081 | 0.603351601174544 | 74.9800578392681 | 247.315926591542 | Belize |
| 2020 | 308.63345064463 | 0.607060046133776 | 74.9775334297739 | 233.655917214856 | Belize |
| 2021 | 293.77329882552 | 0.610229002222948 | 68.937396127164 | 224.835902698356 | Belize |
| 1990 | 491.91048809896 | 0.558019070765374 | 141.860133293623 | 350.050354805337 | Cuba |
| 1991 | 430.99516 | 0.563413638292236 | 138.510068770377 | 292.485091225023 | Cuba |
| 1992 | 363.33275978544 | 0.566169005573441 | 114.842730880473 | 248.490028904967 | Cuba |
| 1993 | 367.40048545844 | 0.565441046515952 | 114.69214610796 | 252.70833935048 | Cuba |
| 1994 | 380.46776843381 | 0.563186312783606 | 139.212703953303 | 241.255064480507 | Cuba |
| 1995 | 364.82679958294 | 0.560765902424453 | 138.923957154584 | 225.902842428356 | Cuba |
| 1996 | 293.06472619227 | 0.559346328872574 | 142.421219687799 | 150.643506504471 | Cuba |
| 1997 | 264.51592853424 | 0.558924568054562 | 141.770987558163 | 122.744940976077 | Cuba |
| 1998 | 239.24755 | 0.559363220275698 | 141.708867683247 | 97.5386822809535 | Cuba |
| 1999 | 202.79729816338 | 0.561820952733584 | 139.78225714612 | 63.0150410172603 | Cuba |
| 2000 | 174.05827176035 | 0.566676901631981 | 113.246386416778 | 60.8118853435719 | Cuba |
| 2001 | 136.7522578 | 0.572753860839461 | 79.4663815256687 | 57.2858762974313 | Cuba |
| 2002 | 123.21933084913 | 0.579968290233664 | 81.3896842819694 | 41.8296465671607 | Cuba |
| 2003 | 127.12278017622 | 0.587647761532625 | 79.6387334154175 | 47.4840467608025 | Cuba |
| 2004 | 120.67003472772 | 0.594348715355523 | 79.2255993859781 | 41.4444353417419 | Cuba |
| 2005 | 136.06694659233 | 0.599863798514665 | 74.526937830532 | 61.540008761798 | Cuba |
| 2006 | 124.66216365206 | 0.604970567275803 | 75.765733609996 | 48.896430042064 | Cuba |
| 2007 | 119.69223899855 | 0.607618098577978 | 73.4030627558989 | 46.2891762426511 | Cuba |
| 2008 | 124.37067669222 | 0.60940092330195 | 68.6360989903698 | 55.7345777018502 | Cuba |
| 2009 | 120.41365023953 | 0.611775641002492 | 66.8417661249053 | 53.5718841146247 | Cuba |
| 2010 | 106.9446859 | 0.615261262451076 | 69.6103611838793 | 37.3343246665207 | Cuba |
| 2011 | 105.88494273931 | 0.620386689831088 | 64.3899836721261 | 41.4949590671839 | Cuba |
| 2012 | 105.40881709139 | 0.626156024890616 | 58.3104178310186 | 47.0983992603714 | Cuba |
| 2013 | 98.77929177 | 0.631839801039436 | 54.3430003242186 | 44.4362914411514 | Cuba |
| 2014 | 104.169386 | 0.637304129079553 | 51.6142629786695 | 52.5551230534305 | Cuba |
| 2015 | 93.700682 | 0.642818963057111 | 51.6698493044849 | 42.0308326909851 | Cuba |
| 2016 | 87.27929197 | 0.647907866261711 | 49.6506396621993 | 37.6286523085307 | Cuba |
| 2017 | 90.72413507 | 0.652309286413179 | 45.1024732299076 | 45.6216618375024 | Cuba |
| 2018 | 91.48017542 | 0.65685865401747 | 45.1065382747321 | 46.3736371448979 | Cuba |
| 2019 | 101.58418344702 | 0.661674716522849 | 43.7584161339726 | 57.8257673130474 | Cuba |
| 2020 | 85.64803125 | 0.665210747223401 | 40.2232932908993 | 45.4247379629907 | Cuba |
| 2021 | 74.54762075 | 0.668729864363244 | 39.30906018 | 35.23856057 | Cuba |
| 1990 | 518.87081939768 | 0.563602590366912 | 137.585986907141 | 381.284832490539 | Dominica |
| 1991 | 510.68634859286 | 0.565237843041271 | 111.290055725248 | 399.396292867612 | Dominica |
| 1992 | 495.49661508301 | 0.569570683969256 | 104.293751686068 | 391.202863396942 | Dominica |
| 1993 | 474.26480726709 | 0.578811810513113 | 85.306663188047 | 388.958144079043 | Dominica |
| 1994 | 455.97076056887 | 0.590119147153383 | 81.4971173974706 | 374.473643171399 | Dominica |
| 1995 | 428.39815368228 | 0.601986931512718 | 76.2058075905639 | 352.192346091716 | Dominica |
| 1996 | 404.59842630161 | 0.614382049775589 | 69.6045130395914 | 334.993913262019 | Dominica |
| 1997 | 383.40598476352 | 0.625057352694054 | 59.3222189098046 | 324.083765853715 | Dominica |
| 1998 | 364.55717441744 | 0.63301799374648 | 52.5198027481563 | 312.037371669284 | Dominica |
| 1999 | 358.3971748 | 0.63945391525062 | 50.2195902590856 | 308.177584539714 | Dominica |
| 2000 | 335.52876323341 | 0.645830936825434 | 49.4698218529925 | 286.058941380418 | Dominica |
| 2001 | 328.92585410719 | 0.652368567094588 | 46.1777775826467 | 282.748076524543 | Dominica |
| 2002 | 312.00121060475 | 0.658423554407679 | 44.6011150842792 | 267.400095520471 | Dominica |
| 2003 | 308.86705271594 | 0.663678867784012 | 40.7687215103434 | 268.098331205597 | Dominica |
| 2004 | 298.1101578 | 0.668952131656395 | 39.092907496849 | 259.017250314651 | Dominica |
| 2005 | 294.61729233175 | 0.67454907679023 | 37.992355559775 | 256.624936771975 | Dominica |
| 2006 | 286.06467891118 | 0.680342481749478 | 37.3613953191524 | 248.703283592028 | Dominica |
| 2007 | 293.51364658236 | 0.685415917499458 | 37.3808824069539 | 256.132764175406 | Dominica |
| 2008 | 288.19965596166 | 0.69019751901742 | 36.5315965435526 | 251.668059418107 | Dominica |
| 2009 | 311.62566484229 | 0.694461869014732 | 36.806173289449 | 274.819491552841 | Dominica |
| 2010 | 326.7137717 | 0.698489193418408 | 35.7093406348891 | 291.004431094911 | Dominica |
| 2011 | 352.00423160896 | 0.702463260148908 | 35.9822819508666 | 316.021949658093 | Dominica |
| 2012 | 363.2172501 | 0.706870607879807 | 34.4926589717306 | 328.724591083969 | Dominica |
| 2013 | 379.74164336423 | 0.71221047502743 | 31.6500793017445 | 348.091564062486 | Dominica |
| 2014 | 380.37109456671 | 0.718455556016605 | 28.6894471855325 | 351.681647381177 | Dominica |
| 2015 | 375.06390883664 | 0.724785536415403 | 25.8694724066612 | 349.194436429979 | Dominica |
| 2016 | 365.24968793047 | 0.73176080052363 | 24.3200474046171 | 340.929640525853 | Dominica |
| 2017 | 364.53738727959 | 0.736062472571312 | 22.2713351558431 | 342.266052123747 | Dominica |
| 2018 | 368.16083585513 | 0.738935953651369 | 20.3099620178013 | 347.850873837329 | Dominica |
| 2019 | 374.22015322008 | 0.74213206575999 | 20.3708995847864 | 353.849253635294 | Dominica |
| 2020 | 378.80836060969 | 0.744419943891979 | 20.5014809331572 | 358.306879676533 | Dominica |
| 2021 | 381.12663484271 | 0.746967184749286 | 20.8428018897575 | 360.283832952952 | Dominica |
| 1990 | 10135.6293036634 | 0.442654076123481 | 423.892125215049 | 9711.73717844835 | Dominican Republic |
| 1991 | 9464.55401227721 | 0.446144577831215 | 413.680439033363 | 9050.87357324385 | Dominican Republic |
| 1992 | 8709.43999577346 | 0.449953080528465 | 408.123311330208 | 8301.31668444325 | Dominican Republic |
| 1993 | 8033.07657337996 | 0.454229297152068 | 407.4018304 | 7625.67474297396 | Dominican Republic |
| 1994 | 7255.82753404831 | 0.458620613654854 | 398.285340821082 | 6857.54219322723 | Dominican Republic |
| 1995 | 6566.19726862354 | 0.463313869288046 | 377.812054298598 | 6188.38521432494 | Dominican Republic |
| 1996 | 5836.20555624493 | 0.468516938419476 | 361.633447284404 | 5474.57210896053 | Dominican Republic |
| 1997 | 5174.36176167803 | 0.474504388639617 | 335.480826212841 | 4838.88093546519 | Dominican Republic |
| 1998 | 4523.1058973538 | 0.481170939346086 | 326.442489734258 | 4196.66340761954 | Dominican Republic |
| 1999 | 3984.26858847212 | 0.488178541467036 | 310.447952992639 | 3673.82063547948 | Dominican Republic |
| 2000 | 3623.79566261368 | 0.495468586151319 | 285.142233237513 | 3338.65342937617 | Dominican Republic |
| 2001 | 3409.26485312447 | 0.50277541519763 | 223.414658128021 | 3185.85019499645 | Dominican Republic |
| 2002 | 3134.69379329474 | 0.510298018698881 | 212.852699000724 | 2921.84109429402 | Dominican Republic |
| 2003 | 2944.97026903061 | 0.517395574464313 | 203.733982455855 | 2741.23628657475 | Dominican Republic |
| 2004 | 2877.64814618783 | 0.524543274827677 | 190.173341567234 | 2687.4748046206 | Dominican Republic |
| 2005 | 2807.61248751179 | 0.532228649477556 | 171.751471557826 | 2635.86101595396 | Dominican Republic |
| 2006 | 2590.85984957483 | 0.540222689966408 | 156.278645444659 | 2434.58120413017 | Dominican Republic |
| 2007 | 2382.04874370724 | 0.548187021157434 | 149.78602912771 | 2232.26271457953 | Dominican Republic |
| 2008 | 2168.80208880848 | 0.555371313172153 | 143.805532312473 | 2024.99655649601 | Dominican Republic |
| 2009 | 2024.843073 | 0.561783754117237 | 139.361049933268 | 1885.48202332573 | Dominican Republic |
| 2010 | 1981.38170464471 | 0.568130581856948 | 109.781156566824 | 1871.60054807789 | Dominican Republic |
| 2011 | 2256.65355536113 | 0.573834812622029 | 88.514672392642 | 2168.13888296849 | Dominican Republic |
| 2012 | 2191.22117978468 | 0.578966380988324 | 83.6062513253857 | 2107.61492845929 | Dominican Republic |
| 2013 | 1623.51960727108 | 0.583625825103645 | 84.3824278950468 | 1539.13717937603 | Dominican Republic |
| 2014 | 1383.9146885119 | 0.588236742676906 | 77.5704817537235 | 1306.34420675818 | Dominican Republic |
| 2015 | 1190.90844536468 | 0.592924164325808 | 76.4983632616517 | 1114.41008210303 | Dominican Republic |
| 2016 | 1120.54423808511 | 0.597619609207884 | 78.2727428477019 | 1042.27149523741 | Dominican Republic |
| 2017 | 1017.30853478449 | 0.602140291257967 | 74.1990851764208 | 943.109449608069 | Dominican Republic |
| 2018 | 955.38157936975 | 0.606960838531609 | 73.8719190178548 | 881.509660351895 | Dominican Republic |
| 2019 | 908.31106425792 | 0.611913021025697 | 70.2803379191984 | 838.030726338722 | Dominican Republic |
| 2020 | 962.56758870674 | 0.615635625027175 | 70.7122455393097 | 891.85534316743 | Dominican Republic |
| 2021 | 929.04772257597 | 0.619388201281128 | 62.8026144344857 | 866.245108141484 | Dominican Republic |
| 1990 | 484.93733853177 | 0.436734419397497 | 420.180773437492 | 64.756565094278 | Grenada |
| 1991 | 405.33153529059 | 0.445887952925777 | 401.206265634815 | 4.12526965577501 | Grenada |
| 1992 | 398.7384205 | 0.455910058376885 | 394.606068405251 | 4.13235210534936 | Grenada |
| 1993 | 402.05314134093 | 0.465994889867929 | 361.298931084818 | 40.7542102561125 | Grenada |
| 1994 | 367.38341993001 | 0.476410340600106 | 336.747248563617 | 30.6361713663926 | Grenada |
| 1995 | 344.86340411328 | 0.486558998595925 | 318.131904720161 | 26.7314993931186 | Grenada |
| 1996 | 328.42918076282 | 0.496589807805192 | 256.785765816584 | 71.6434149462365 | Grenada |
| 1997 | 304.45031530753 | 0.50621760158124 | 218.866756232365 | 85.5835590751649 | Grenada |
| 1998 | 259.37354700081 | 0.516590019085488 | 202.538057547584 | 56.8354894532264 | Grenada |
| 1999 | 185.78697484982 | 0.527333411440571 | 181.302019582952 | 4.48495526686801 | Grenada |
| 2000 | 157.743829 | 0.538388116408646 | 155.981577246736 | 1.76225170436399 | Grenada |
| 2001 | 148.45611000981 | 0.549058969647808 | 147.606216527931 | 0.84989348187932 | Grenada |
| 2002 | 135.33007852279 | 0.559473127591996 | 135.33007852279 | 0 | Grenada |
| 2003 | 134.20132124004 | 0.569718037363691 | 108.91209884083 | 25.2892223992103 | Grenada |
| 2004 | 132.35101239936 | 0.578487456332464 | 81.2338186552319 | 51.1171937441281 | Grenada |
| 2005 | 139.57328602604 | 0.587158876770892 | 82.2888786799724 | 57.2844073460676 | Grenada |
| 2006 | 140.1506636 | 0.59405451978646 | 79.1157977608784 | 61.0348658200216 | Grenada |
| 2007 | 134.42267408186 | 0.600766335502731 | 75.1572494159481 | 59.2654246659119 | Grenada |
| 2008 | 130.66506220976 | 0.606879281941233 | 74.4997574690972 | 56.1653047406628 | Grenada |
| 2009 | 123.77389051618 | 0.61180525701265 | 67.0604880480644 | 56.7134024681156 | Grenada |
| 2010 | 115.74690244031 | 0.616486997376644 | 63.0415638512078 | 52.7053385891022 | Grenada |
| 2011 | 116.77397131065 | 0.620978521316098 | 63.6037825475981 | 53.1701887630519 | Grenada |
| 2012 | 113.22632845155 | 0.624977512249081 | 59.5588188910125 | 53.6675095605375 | Grenada |
| 2013 | 113.25462880511 | 0.629048335732456 | 57.0138989034547 | 56.2407299016553 | Grenada |
| 2014 | 122.87970768755 | 0.633734695952301 | 53.5472575021835 | 69.3324501853665 | Grenada |
| 2015 | 114.11519652616 | 0.638936083726672 | 50.2148697314381 | 63.9003267947219 | Grenada |
| 2016 | 105.85021099722 | 0.644335233932856 | 50.4656824374632 | 55.3845285597568 | Grenada |
| 2017 | 98.88489221 | 0.649831521980215 | 48.3087113624185 | 50.5761808447915 | Grenada |
| 2018 | 96.5340881 | 0.655372772077438 | 45.608488473054 | 50.925599626006 | Grenada |
| 2019 | 91.64626169 | 0.660808511595714 | 42.7411590767934 | 48.9051026088966 | Grenada |
| 2020 | 88.21157311 | 0.665086346623039 | 39.9048538789802 | 48.3067192353598 | Grenada |
| 2021 | 83.07800009 | 0.668993028326993 | 38.9815449311618 | 44.0964551618782 | Grenada |
| 1990 | 6555.02688273035 | 0.460430128793009 | 396.640714525857 | 6158.38616820449 | Guyana |
| 1991 | 5876.55192450363 | 0.462677068801868 | 380.468113404961 | 5496.08381109867 | Guyana |
| 1992 | 5560.18288199427 | 0.466753989262645 | 359.457146061583 | 5200.72573593269 | Guyana |
| 1993 | 5446.57845885708 | 0.472174228924914 | 342.769955764619 | 5103.80850309246 | Guyana |
| 1994 | 5047.62107708043 | 0.478992924260878 | 324.217769231401 | 4723.40330784903 | Guyana |
| 1995 | 4674.04526065493 | 0.486573457084383 | 316.980825639819 | 4357.06443501511 | Guyana |
| 1996 | 4252.15630066914 | 0.495216036467396 | 283.610569446003 | 3968.54573122314 | Guyana |
| 1997 | 4083.93781748009 | 0.504362778751228 | 224.068434002373 | 3859.86938347772 | Guyana |
| 1998 | 3877.65334598069 | 0.513068898204645 | 207.249770002684 | 3670.40357597801 | Guyana |
| 1999 | 4163.83727402199 | 0.521595978096732 | 198.189697243321 | 3965.64757677867 | Guyana |
| 2000 | 4958.36097590215 | 0.528693991503479 | 181.033020110892 | 4777.32795579126 | Guyana |
| 2001 | 3634.94158095547 | 0.534869805890103 | 162.334881298503 | 3472.60669965697 | Guyana |
| 2002 | 2990.96436343012 | 0.54051427331456 | 156.317462603233 | 2834.64690082689 | Guyana |
| 2003 | 2707.91689608595 | 0.545516473248843 | 152.304324620265 | 2555.61257146569 | Guyana |
| 2004 | 2457.76374225842 | 0.550326370085983 | 146.657026487437 | 2311.10671577098 | Guyana |
| 2005 | 2686.60745668628 | 0.554844797400739 | 143.845615756539 | 2542.76184092974 | Guyana |
| 2006 | 2457.61116152377 | 0.55946561076404 | 142.329968972965 | 2315.2811925508 | Guyana |
| 2007 | 1973.80902351417 | 0.564541135103012 | 112.874583541403 | 1860.93443997277 | Guyana |
| 2008 | 1664.87768317743 | 0.569595205505363 | 113.840170604422 | 1551.03751257301 | Guyana |
| 2009 | 1556.45161815296 | 0.574769931497353 | 83.2751111135715 | 1473.17650703939 | Guyana |
| 2010 | 1267.00116460594 | 0.580083911433493 | 84.9296698399407 | 1182.071495 | Guyana |
| 2011 | 1042.1738109126 | 0.585688269016646 | 76.9728162943956 | 965.200994618204 | Guyana |
| 2012 | 911.05144907765 | 0.591336582127807 | 80.2282063855894 | 830.823242692061 | Guyana |
| 2013 | 823.51372197567 | 0.596826294655764 | 75.8531678274879 | 747.660554148182 | Guyana |
| 2014 | 777.43864587562 | 0.602136510795571 | 75.8316989505149 | 701.606946925105 | Guyana |
| 2015 | 699.51399887697 | 0.60760548677096 | 77.158987483602 | 622.355011393368 | Guyana |
| 2016 | 649.99805545942 | 0.613914359668043 | 70.2753991823891 | 579.722656277031 | Guyana |
| 2017 | 612.04919654494 | 0.620602607413008 | 63.2600247544084 | 548.789171790532 | Guyana |
| 2018 | 603.37613755961 | 0.627167040808132 | 58.2923818623787 | 545.083755697231 | Guyana |
| 2019 | 526.4584191 | 0.633640346503077 | 53.0666950723866 | 473.391724058813 | Guyana |
| 2020 | 512.26348903102 | 0.642284644781673 | 50.4160247875533 | 461.847464243467 | Guyana |
| 2021 | 421.76011957304 | 0.650812335285137 | 47.5100016254524 | 374.250117947588 | Guyana |
| 1990 | 21119.3503351876 | 0.310334630409749 | 2113.02461803328 | 19006.3257171543 | Haiti |
| 1991 | 19989.8422765578 | 0.315455441278148 | 2100.96837392895 | 17888.8739026288 | Haiti |
| 1992 | 19349.0403868835 | 0.320389978747448 | 2105.27866813439 | 17243.7617187491 | Haiti |
| 1993 | 18596.0652286502 | 0.324895850993773 | 2061.60458692565 | 16534.4606417245 | Haiti |
| 1994 | 17885.9625657891 | 0.328399455968527 | 2049.11512628427 | 15836.8474395048 | Haiti |
| 1995 | 17173.9932855702 | 0.332999151562276 | 1986.38632401288 | 15187.6069615573 | Haiti |
| 1996 | 16195.2961551704 | 0.33822939619677 | 1855.11899567363 | 14340.1771594968 | Haiti |
| 1997 | 15335.5507815429 | 0.343880049177945 | 1779.77263047186 | 13555.778151071 | Haiti |
| 1998 | 14159.9641643883 | 0.349698073003298 | 1771.31629021004 | 12388.6478741783 | Haiti |
| 1999 | 13391.4797654452 | 0.355980742770846 | 1753.28111596986 | 11638.1986494753 | Haiti |
| 2000 | 12441.7570925351 | 0.36257220895481 | 1397.8789728233 | 11043.8781197118 | Haiti |
| 2001 | 11876.3236110332 | 0.368643507933658 | 1231.41132383863 | 10644.9122871946 | Haiti |
| 2002 | 11353.1787432786 | 0.374473324901094 | 1174.82377618856 | 10178.35497 | Haiti |
| 2003 | 10944.3353888463 | 0.380067254956705 | 1124.48286670192 | 9819.85252214438 | Haiti |
| 2004 | 10586.4446242943 | 0.385061511883196 | 1102.34462034853 | 9484.10000394577 | Haiti |
| 2005 | 10207.6259028466 | 0.389845419506401 | 1035.16103954778 | 9172.46486329882 | Haiti |
| 2006 | 9690.23968970333 | 0.394322001247404 | 983.942768164904 | 8706.29692153843 | Haiti |
| 2007 | 9278.76175689343 | 0.39897344059179 | 925.994417670254 | 8352.76733922318 | Haiti |
| 2008 | 8837.98882650618 | 0.40328587122688 | 893.920352036168 | 7944.06847447001 | Haiti |
| 2009 | 8401.33988929801 | 0.407944423962943 | 866.617814825113 | 7534.7220744729 | Haiti |
| 2010 | 10187.2190302144 | 0.411840105174603 | 852.905731335276 | 9334.31329887913 | Haiti |
| 2011 | 15813.5285567099 | 0.416177190225703 | 792.77438651059 | 15020.7541701993 | Haiti |
| 2012 | 7125.6845224484 | 0.420021527912646 | 780.016131033574 | 6345.66839141483 | Haiti |
| 2013 | 7081.57799000785 | 0.423985549390571 | 683.454927443477 | 6398.12306256437 | Haiti |
| 2014 | 5795.27501816718 | 0.427940902171937 | 537.536789516336 | 5257.73822865084 | Haiti |
| 2015 | 5257.89382454215 | 0.431756760061604 | 430.206480228958 | 4827.68734431319 | Haiti |
| 2016 | 5389.46808623397 | 0.435288722267081 | 421.055735734078 | 4968.41235049989 | Haiti |
| 2017 | 4729.67577635818 | 0.438541003090222 | 427.164303649202 | 4302.51147270898 | Haiti |
| 2018 | 4523.80983360616 | 0.44167892537118 | 421.318802907112 | 4102.49103069905 | Haiti |
| 2019 | 4388.25861161197 | 0.444328853815805 | 422.603815522902 | 3965.65479608907 | Haiti |
| 2020 | 4748.10597404821 | 0.446390613582292 | 412.735994841484 | 4335.36997920673 | Haiti |
| 2021 | 4737.272221 | 0.448278284992742 | 410.56775779949 | 4326.70446314151 | Haiti |
| 1990 | 1728.44504581795 | 0.534781233684701 | 162.395789440309 | 1566.04925637764 | Jamaica |
| 1991 | 1485.55465797531 | 0.539594479788248 | 156.208862864049 | 1329.34579511126 | Jamaica |
| 1992 | 1347.24041181534 | 0.545088752562521 | 152.363955620484 | 1194.87645619486 | Jamaica |
| 1993 | 1197.58258304712 | 0.551218491600381 | 145.842355729928 | 1051.74022731719 | Jamaica |
| 1994 | 1053.42456809942 | 0.557643773203113 | 142.24636971046 | 911.17819838896 | Jamaica |
| 1995 | 835.15278380385 | 0.564315037773069 | 137.373176539899 | 697.77960726395 | Jamaica |
| 1996 | 518.26648635896 | 0.570940131286319 | 86.9353211639367 | 431.331165195023 | Jamaica |
| 1997 | 398.52481188744 | 0.577212091097575 | 83.2212076123783 | 315.303604275062 | Jamaica |
| 1998 | 354.29250465697 | 0.58304803468123 | 81.1597514300461 | 273.132753226924 | Jamaica |
| 1999 | 320.7482319 | 0.588846467179198 | 85.9657087835862 | 234.782523115114 | Jamaica |
| 2000 | 290.94164435612 | 0.594592916825086 | 76.5365278637532 | 214.405116492367 | Jamaica |
| 2001 | 230.44229206891 | 0.600192022232271 | 76.0657570045539 | 154.376535064356 | Jamaica |
| 2002 | 211.81922100145 | 0.605591748141932 | 75.5439297705993 | 136.275291230851 | Jamaica |
| 2003 | 221.79224267257 | 0.610877423593022 | 68.0948072855812 | 153.697435386989 | Jamaica |
| 2004 | 213.56396305529 | 0.615836278207827 | 68.721056959229 | 144.842906096061 | Jamaica |
| 2005 | 218.11271839637 | 0.620400056664326 | 62.7092431941478 | 155.403475202222 | Jamaica |
| 2006 | 255.16222346994 | 0.625018859668551 | 58.1821879761987 | 196.980035493741 | Jamaica |
| 2007 | 245.25206644757 | 0.629549469674028 | 54.9121088597241 | 190.339957587846 | Jamaica |
| 2008 | 233.22292662419 | 0.633967851311631 | 53.8454851076897 | 179.3774415 | Jamaica |
| 2009 | 227.62284684995 | 0.638110835001433 | 50.2327774491692 | 177.390069400781 | Jamaica |
| 2010 | 216.1437166 | 0.642129387979891 | 50.1317730118875 | 166.011943541913 | Jamaica |
| 2011 | 215.43662329183 | 0.646395830061733 | 50.0193090622209 | 165.417314229609 | Jamaica |
| 2012 | 214.72534300685 | 0.650583760450656 | 46.9379717142473 | 167.787371292603 | Jamaica |
| 2013 | 197.9364519 | 0.654573624159258 | 45.9822199324869 | 151.954231976913 | Jamaica |
| 2014 | 235.59366607864 | 0.658505341253061 | 45.3173579357997 | 190.27630814284 | Jamaica |
| 2015 | 222.58486763877 | 0.662465809295028 | 43.3720763973297 | 179.21279124144 | Jamaica |
| 2016 | 214.04604333731 | 0.666469278740004 | 39.034178709769 | 175.011864627541 | Jamaica |
| 2017 | 205.92473559527 | 0.670291045200821 | 38.7213019137836 | 167.203433681486 | Jamaica |
| 2018 | 197.88524593167 | 0.674075312952044 | 37.8776663239996 | 160.00757960767 | Jamaica |
| 2019 | 191.17434680803 | 0.677778539230129 | 37.4961045307996 | 153.67824227723 | Jamaica |
| 2020 | 183.50404086568 | 0.680567176976565 | 37.2707344998013 | 146.233306365879 | Jamaica |
| 2021 | 170.67316629778 | 0.683263063531091 | 37.3948645695915 | 133.278301728189 | Jamaica |
| 1990 | 926.81673620844 | 0.496296570103573 | 268.228381536348 | 658.588354672092 | Saint Lucia |
| 1991 | 804.02281828657 | 0.505975554986447 | 221.253978292827 | 582.768839993743 | Saint Lucia |
| 1992 | 744.4240727 | 0.515368260657711 | 205.859147228172 | 538.564925442228 | Saint Lucia |
| 1993 | 765.34291592675 | 0.524234949005948 | 190.841015611403 | 574.501900315347 | Saint Lucia |
| 1994 | 681.18339850588 | 0.532731082607556 | 170.528881627757 | 510.654516878123 | Saint Lucia |
| 1995 | 633.23962599226 | 0.541479793625538 | 155.134380812667 | 478.105245179593 | Saint Lucia |
| 1996 | 586.08214410282 | 0.550242411723114 | 145.573705176623 | 440.508438926197 | Saint Lucia |
| 1997 | 534.46880488516 | 0.558293460901167 | 141.863320243716 | 392.605484641444 | Saint Lucia |
| 1998 | 447.68871255123 | 0.566138702625704 | 110.082757774818 | 337.605954776412 | Saint Lucia |
| 1999 | 371.735116 | 0.573428555367193 | 83.9182904831733 | 287.816825529527 | Saint Lucia |
| 2000 | 307.91595228504 | 0.579788896229167 | 84.4212445027377 | 223.494707782302 | Saint Lucia |
| 2001 | 255.4582841 | 0.585087557754993 | 78.3762858288953 | 177.081998252905 | Saint Lucia |
| 2002 | 229.35955268416 | 0.590167578497987 | 78.6552355610566 | 150.704317123103 | Saint Lucia |
| 2003 | 225.98566609258 | 0.595675145724796 | 78.1037760244588 | 147.881890068121 | Saint Lucia |
| 2004 | 224.06179267169 | 0.601625481597697 | 74.4785984448032 | 149.583194226887 | Saint Lucia |
| 2005 | 243.56589436751 | 0.60723986604169 | 75.3328002717368 | 168.233094095773 | Saint Lucia |
| 2006 | 234.59714480215 | 0.612755299779844 | 69.4170954605864 | 165.180049341564 | Saint Lucia |
| 2007 | 230.72404881672 | 0.617994714022193 | 64.1799869830807 | 166.544061833639 | Saint Lucia |
| 2008 | 226.49389289501 | 0.62327300064738 | 62.5220697642096 | 163.9718231 | Saint Lucia |
| 2009 | 209.864471 | 0.628169848746241 | 55.1809158921913 | 154.683555154209 | Saint Lucia |
| 2010 | 191.99666020207 | 0.632782904571138 | 53.1874178548185 | 138.809242347251 | Saint Lucia |
| 2011 | 196.02234260521 | 0.637439441748952 | 51.3198149253541 | 144.702527679856 | Saint Lucia |
| 2012 | 188.53013478738 | 0.641567127255833 | 53.7331876547282 | 134.796947132652 | Saint Lucia |
| 2013 | 188.85074003205 | 0.645153079091964 | 48.9387098764137 | 139.912030155636 | Saint Lucia |
| 2014 | 204.96505038735 | 0.648743506935936 | 47.4093930684313 | 157.555657318919 | Saint Lucia |
| 2015 | 196.25097877937 | 0.652157480490287 | 45.8920744094158 | 150.358904369954 | Saint Lucia |
| 2016 | 183.97189454612 | 0.655653340205716 | 45.8937493552688 | 138.078145190851 | Saint Lucia |
| 2017 | 175.49226784029 | 0.659341345742569 | 43.4963756342922 | 131.995892205998 | Saint Lucia |
| 2018 | 167.69463760676 | 0.663086681831917 | 40.8680426204398 | 126.82659498632 | Saint Lucia |
| 2019 | 161.27564487364 | 0.666840358993444 | 39.215448940354 | 122.060195933286 | Saint Lucia |
| 2020 | 149.57204113368 | 0.669720489592338 | 38.7323611374756 | 110.839679996204 | Saint Lucia |
| 2021 | 145.85337543771 | 0.672509735365292 | 37.916330884157 | 107.937044553553 | Saint Lucia |
| 1990 | 1735.78250068457 | 0.475930186469041 | 333.936185177956 | 1401.84631550661 | Saint Vincent and the Grenadines |
| 1991 | 1609.86650322111 | 0.481075658352439 | 322.525694999513 | 1287.3408082216 | Saint Vincent and the Grenadines |
| 1992 | 1457.25151425644 | 0.485977871294048 | 314.716350938418 | 1142.53516331802 | Saint Vincent and the Grenadines |
| 1993 | 1364.23466476864 | 0.490998790902684 | 299.393755752262 | 1064.84090901638 | Saint Vincent and the Grenadines |
| 1994 | 1248.89131248717 | 0.496379132267915 | 262.493654095713 | 986.397658391457 | Saint Vincent and the Grenadines |
| 1995 | 1126.87580458853 | 0.502986089658165 | 224.426503199885 | 902.449301388645 | Saint Vincent and the Grenadines |
| 1996 | 1026.12922670374 | 0.509527157829315 | 214.996634832549 | 811.13259187119 | Saint Vincent and the Grenadines |
| 1997 | 925.04549441096 | 0.51548261862783 | 204.538492199107 | 720.507002211853 | Saint Vincent and the Grenadines |
| 1998 | 805.01767195424 | 0.52120967882065 | 198.183925135253 | 606.833746818987 | Saint Vincent and the Grenadines |
| 1999 | 684.87028262633 | 0.527099551882504 | 190.396887702581 | 494.473394923749 | Saint Vincent and the Grenadines |
| 2000 | 572.78801650149 | 0.533173289927656 | 171.776613949034 | 401.011402552456 | Saint Vincent and the Grenadines |
| 2001 | 489.47944585737 | 0.539455711639871 | 155.895636898497 | 333.583808958873 | Saint Vincent and the Grenadines |
| 2002 | 413.62070486107 | 0.546063615669324 | 150.055143771578 | 263.565561089492 | Saint Vincent and the Grenadines |
| 2003 | 429.91563211933 | 0.552693724058086 | 145.926434770463 | 283.989197348867 | Saint Vincent and the Grenadines |
| 2004 | 397.86912435255 | 0.558663542945828 | 142.35671841317 | 255.51240593938 | Saint Vincent and the Grenadines |
| 2005 | 411.45845402847 | 0.563588116289597 | 137.465901042801 | 273.992552985669 | Saint Vincent and the Grenadines |
| 2006 | 398.79111383719 | 0.568221747811185 | 111.800494303111 | 286.990619534079 | Saint Vincent and the Grenadines |
| 2007 | 391.51146348188 | 0.572819052303515 | 85.8506149984885 | 305.660848483392 | Saint Vincent and the Grenadines |
| 2008 | 363.28741458619 | 0.577611144975623 | 82.6349812702315 | 280.652433315958 | Saint Vincent and the Grenadines |
| 2009 | 335.03058899254 | 0.582549107234974 | 85.4056128628096 | 249.62497612973 | Saint Vincent and the Grenadines |
| 2010 | 305.65416162844 | 0.587190383760787 | 82.8670155052418 | 222.787146123198 | Saint Vincent and the Grenadines |
| 2011 | 301.21472637079 | 0.590259095650209 | 77.9416369817102 | 223.27308938908 | Saint Vincent and the Grenadines |
| 2012 | 288.95586771069 | 0.592153907530225 | 76.2402794048608 | 212.715588305829 | Saint Vincent and the Grenadines |
| 2013 | 305.35195998993 | 0.594614074720551 | 78.7491840213372 | 226.602775968593 | Saint Vincent and the Grenadines |
| 2014 | 342.22964119584 | 0.598378671838632 | 77.7564289168308 | 264.473212279009 | Saint Vincent and the Grenadines |
| 2015 | 310.87172091489 | 0.603754134616218 | 76.6060507121782 | 234.265670202712 | Saint Vincent and the Grenadines |
| 2016 | 263.72100421331 | 0.610059210417339 | 67.47274432 | 196.24825989708 | Saint Vincent and the Grenadines |
| 2017 | 242.97158893684 | 0.616438472725892 | 70.3160174644562 | 172.655571472384 | Saint Vincent and the Grenadines |
| 2018 | 227.99091605776 | 0.622634944331176 | 63.5193702892146 | 164.471545768545 | Saint Vincent and the Grenadines |
| 2019 | 213.59321796951 | 0.628325985755241 | 56.6863117410757 | 156.906906228434 | Saint Vincent and the Grenadines |
| 2020 | 195.9107552 | 0.632860904072373 | 54.1732353168504 | 141.73751987355 | Saint Vincent and the Grenadines |
| 2021 | 194.76476513161 | 0.637195962512539 | 51.3490943991885 | 143.415670732422 | Saint Vincent and the Grenadines |
| 1990 | 4229.61552913956 | 0.50205430482691 | 230.226072290958 | 3999.3894568486 | Suriname |
| 1991 | 4082.34105716716 | 0.506655794671834 | 218.901197808528 | 3863.43985935863 | Suriname |
| 1992 | 3934.94898524618 | 0.510959772316403 | 213.043913030471 | 3721.90507221571 | Suriname |
| 1993 | 3711.21159465925 | 0.514419436992343 | 206.335639881245 | 3504.875955 | Suriname |
| 1994 | 3547.49293145373 | 0.517650245235505 | 203.078022753943 | 3344.41490869979 | Suriname |
| 1995 | 3365.17271814024 | 0.521002622374316 | 198.780257597909 | 3166.39246054233 | Suriname |
| 1996 | 3182.36692187472 | 0.525059318240488 | 190.137566146876 | 2992.22935572784 | Suriname |
| 1997 | 3013.71583199822 | 0.529750811513369 | 179.624051907407 | 2834.09178009081 | Suriname |
| 1998 | 2807.77374012638 | 0.534599696353116 | 162.841422805253 | 2644.93231732113 | Suriname |
| 1999 | 2607.2259922116 | 0.538932791302378 | 157.183327480927 | 2450.04266473067 | Suriname |
| 2000 | 2313.96693490262 | 0.543660023350067 | 155.141478321583 | 2158.82545658104 | Suriname |
| 2001 | 2101.11420317838 | 0.548683523928075 | 149.234340956203 | 1951.87986222218 | Suriname |
| 2002 | 2019.20469420553 | 0.553879432238792 | 143.891511564843 | 1875.31318264069 | Suriname |
| 2003 | 1896.17287850455 | 0.559418373479841 | 141.962021830371 | 1754.21085667418 | Suriname |
| 2004 | 1776.91032802561 | 0.565109708384423 | 112.627584111244 | 1664.28274391437 | Suriname |
| 2005 | 1677.23850485722 | 0.570462186195307 | 91.7775437102552 | 1585.46096114696 | Suriname |
| 2006 | 1584.34362366309 | 0.575647048246499 | 80.2256190162633 | 1504.11800464683 | Suriname |
| 2007 | 1536.69505115948 | 0.580654574084671 | 84.0468259956842 | 1452.6482251638 | Suriname |
| 2008 | 1457.43554056023 | 0.585644825421143 | 81.8395475959937 | 1375.59599296424 | Suriname |
| 2009 | 1385.09020406352 | 0.590512804838156 | 81.6562691795364 | 1303.43393488398 | Suriname |
| 2010 | 1303.93422281656 | 0.595469745192738 | 77.6829538705097 | 1226.25126894605 | Suriname |
| 2011 | 1223.34417970991 | 0.600356336126983 | 76.3457920428429 | 1146.99838766707 | Suriname |
| 2012 | 1171.41146909628 | 0.604741870150833 | 76.0218064628584 | 1095.38966263342 | Suriname |
| 2013 | 1141.63362355951 | 0.608910612397383 | 70.0553100077385 | 1071.57831355177 | Suriname |
| 2014 | 1103.17554910078 | 0.61270442287871 | 69.2098050045058 | 1033.96574409627 | Suriname |
| 2015 | 1062.48257698401 | 0.616097315384298 | 68.4314754250209 | 994.051101558989 | Suriname |
| 2016 | 1037.39777431715 | 0.618868087951188 | 62.3109877192905 | 975.08678659786 | Suriname |
| 2017 | 1038.13151361745 | 0.621684179841572 | 63.2283292441443 | 974.903184373306 | Suriname |
| 2018 | 1013.96867712498 | 0.624708011000566 | 59.3135474547604 | 954.65512967022 | Suriname |
| 2019 | 996.09978714042 | 0.62789678100938 | 59.0378442460286 | 937.061942894391 | Suriname |
| 2020 | 940.04970441015 | 0.630683705083472 | 55.4908215159335 | 884.558882894216 | Suriname |
| 2021 | 916.21023769134 | 0.63366573910901 | 53.3954544905049 | 862.814783200835 | Suriname |
| 1990 | 702.74901449172 | 0.623970150133519 | 59.0655671366572 | 643.683447355063 | Trinidad and Tobago |
| 1991 | 650.42658120001 | 0.62875891817345 | 54.6987662404991 | 595.727814959511 | Trinidad and Tobago |
| 1992 | 632.02478068872 | 0.634039551508711 | 53.7835149929037 | 578.241265695816 | Trinidad and Tobago |
| 1993 | 697.5725952 | 0.639191913980039 | 51.3243414238434 | 646.248253824157 | Trinidad and Tobago |
| 1994 | 663.41757122905 | 0.644242910113573 | 50.3401937444639 | 613.077377484586 | Trinidad and Tobago |
| 1995 | 626.96491337408 | 0.649220055474201 | 48.5050930206006 | 578.459820353479 | Trinidad and Tobago |
| 1996 | 587.35294734416 | 0.654169455231313 | 45.7579078585255 | 541.595039485635 | Trinidad and Tobago |
| 1997 | 571.09348714087 | 0.659293633341366 | 43.6707401650769 | 527.422746975793 | Trinidad and Tobago |
| 1998 | 541.20150199103 | 0.664507714329849 | 40.3563522850649 | 500.845149705965 | Trinidad and Tobago |
| 1999 | 526.46772907496 | 0.670244573860158 | 38.5821828265448 | 487.885546248415 | Trinidad and Tobago |
| 2000 | 469.46541621312 | 0.676683835395074 | 37.9416531541846 | 431.523763058935 | Trinidad and Tobago |
| 2001 | 370.04081330849 | 0.682763494477584 | 37.6643509457277 | 332.376462362762 | Trinidad and Tobago |
| 2002 | 335.96023935249 | 0.688872270964441 | 37.3841316946978 | 298.576107657792 | Trinidad and Tobago |
| 2003 | 332.88869562849 | 0.695828341273334 | 36.328613741542 | 296.560081886948 | Trinidad and Tobago |
| 2004 | 277.09627644572 | 0.702610808512884 | 35.8165251442712 | 241.279751301449 | Trinidad and Tobago |
| 2005 | 295.23011600816 | 0.708520984658684 | 33.9686480002934 | 261.261468007867 | Trinidad and Tobago |
| 2006 | 275.55995460776 | 0.714600209518824 | 31.761391250297 | 243.798563357463 | Trinidad and Tobago |
| 2007 | 243.30455565013 | 0.72019641678818 | 28.9571194450958 | 214.347436205034 | Trinidad and Tobago |
| 2008 | 249.23654308809 | 0.725720701823497 | 26.9745167797079 | 222.262026308382 | Trinidad and Tobago |
| 2009 | 227.33973788923 | 0.729538891843969 | 25.3701465956061 | 201.969591293624 | Trinidad and Tobago |
| 2010 | 204.04181342651 | 0.733237076528245 | 23.0203433265575 | 181.021470099952 | Trinidad and Tobago |
| 2011 | 208.76752099276 | 0.736802404534694 | 21.9329681901979 | 186.834552802562 | Trinidad and Tobago |
| 2012 | 199.66334608617 | 0.739999073209787 | 21.3828380543034 | 178.280508031867 | Trinidad and Tobago |
| 2013 | 190.68281814889 | 0.743593955236757 | 20.1917741880604 | 170.49104396083 | Trinidad and Tobago |
| 2014 | 181.89184390725 | 0.747338962511986 | 20.4869643247566 | 161.404879582493 | Trinidad and Tobago |
| 2015 | 171.1394092 | 0.751510682495009 | 19.2412162912094 | 151.898192861391 | Trinidad and Tobago |
| 2016 | 171.55606816717 | 0.754953347556518 | 16.8525266806091 | 154.703541486561 | Trinidad and Tobago |
| 2017 | 159.70637353525 | 0.758226987870251 | 15.5678012540663 | 144.138572281184 | Trinidad and Tobago |
| 2018 | 149.85780339028 | 0.761241990776049 | 15.150479142415 | 134.707324247865 | Trinidad and Tobago |
| 2019 | 147.96782143287 | 0.764169599010201 | 15.2747027853051 | 132.693118647565 | Trinidad and Tobago |
| 2020 | 140.04627193996 | 0.766422080818681 | 13.1241906105788 | 126.922081329381 | Trinidad and Tobago |
| 2021 | 138.41583130751 | 0.768763253980677 | 14.4373387695272 | 123.978492537983 | Trinidad and Tobago |
| 1990 | 8314.19811303363 | 0.423917961315613 | 685.669749162404 | 7628.52836387123 | Bolivia (Plurinational State of) |
| 1991 | 8715.79674550252 | 0.429397795077356 | 426.064918251598 | 8289.73182725092 | Bolivia (Plurinational State of) |
| 1992 | 8368.25469949781 | 0.434835541945787 | 416.472574635308 | 7951.7821248625 | Bolivia (Plurinational State of) |
| 1993 | 7494.74500074675 | 0.440601645503372 | 418.601613484674 | 7076.14338726208 | Bolivia (Plurinational State of) |
| 1994 | 6446.08708022321 | 0.446822500209606 | 411.983037525643 | 6034.10404269757 | Bolivia (Plurinational State of) |
| 1995 | 6082.61327913128 | 0.45347924306274 | 409.559129624943 | 5673.05414950634 | Bolivia (Plurinational State of) |
| 1996 | 5752.52969021866 | 0.460220668843174 | 396.706804991059 | 5355.8228852276 | Bolivia (Plurinational State of) |
| 1997 | 5166.36519922168 | 0.466982896503528 | 361.730786496877 | 4804.6344127248 | Bolivia (Plurinational State of) |
| 1998 | 4790.79911946983 | 0.473796200765922 | 336.210299631423 | 4454.58881983841 | Bolivia (Plurinational State of) |
| 1999 | 4453.39463936768 | 0.480194310456872 | 326.249409698442 | 4127.14522966924 | Bolivia (Plurinational State of) |
| 2000 | 4138.29206514452 | 0.486327903067974 | 320.5118625 | 3817.78020263622 | Bolivia (Plurinational State of) |
| 2001 | 3857.45136803894 | 0.492030763865071 | 295.142367861904 | 3562.30900017704 | Bolivia (Plurinational State of) |
| 2002 | 3558.20404844741 | 0.497348836505264 | 243.39672564541 | 3314.807323 | Bolivia (Plurinational State of) |
| 2003 | 3259.22587863793 | 0.502365802781048 | 227.898932639157 | 3031.32694599877 | Bolivia (Plurinational State of) |
| 2004 | 2973.33965680987 | 0.507273035649156 | 219.645975457305 | 2753.69368135256 | Bolivia (Plurinational State of) |
| 2005 | 2730.7795485317 | 0.512166491375209 | 213.180744824398 | 2517.5988037073 | Bolivia (Plurinational State of) |
| 2006 | 2407.93547252125 | 0.517146193689981 | 203.915007762465 | 2204.02046475879 | Bolivia (Plurinational State of) |
| 2007 | 2028.48695975595 | 0.521946130357677 | 198.805688043524 | 1829.68127171243 | Bolivia (Plurinational State of) |
| 2008 | 1571.77796242646 | 0.526943750292372 | 190.777643736442 | 1381.00031869002 | Bolivia (Plurinational State of) |
| 2009 | 1225.05790431022 | 0.531792440464007 | 170.090886616716 | 1054.9670176935 | Bolivia (Plurinational State of) |
| 2010 | 1061.32412477603 | 0.5370090684329 | 157.81518918835 | 903.50893558768 | Bolivia (Plurinational State of) |
| 2011 | 967.72343130828 | 0.542680744993221 | 155.082200533481 | 812.641230774799 | Bolivia (Plurinational State of) |
| 2012 | 888.40958619507 | 0.548615481889587 | 148.89463356082 | 739.51495263425 | Bolivia (Plurinational State of) |
| 2013 | 826.54388226411 | 0.554788215843767 | 143.828908657475 | 682.714973606635 | Bolivia (Plurinational State of) |
| 2014 | 767.34415172671 | 0.560934581048908 | 139.057474142161 | 628.28667758455 | Bolivia (Plurinational State of) |
| 2015 | 714.52594058192 | 0.567000897613403 | 114.349904482604 | 600.176036099316 | Bolivia (Plurinational State of) |
| 2016 | 666.29556671674 | 0.573080316306652 | 85.2403284844003 | 581.05523823234 | Bolivia (Plurinational State of) |
| 2017 | 628.55443993773 | 0.579241284614228 | 78.9314326556252 | 549.623007282105 | Bolivia (Plurinational State of) |
| 2018 | 593.58150599097 | 0.585184106450667 | 80.3167111174833 | 513.264794873487 | Bolivia (Plurinational State of) |
| 2019 | 571.84435427234 | 0.590692043089904 | 77.8403135724031 | 494.004040699937 | Bolivia (Plurinational State of) |
| 2020 | 650.28697903566 | 0.594854443460131 | 77.7000658541209 | 572.586913181539 | Bolivia (Plurinational State of) |
| 2021 | 619.37264678842 | 0.5990107988623 | 75.0644001773516 | 544.308246611068 | Bolivia (Plurinational State of) |
| 1990 | 5155.3436636337 | 0.518430614137285 | 203.727180287481 | 4951.61648334622 | Ecuador |
| 1991 | 6086.74367304788 | 0.517679421435991 | 203.275218706015 | 5883.46845434187 | Ecuador |
| 1992 | 5054.22561775561 | 0.518379070003192 | 203.760083962656 | 4850.46553379295 | Ecuador |
| 1993 | 3733.85151260254 | 0.520707290609383 | 199.101504341809 | 3534.75000826073 | Ecuador |
| 1994 | 2838.04884152031 | 0.524908290908027 | 191.46948607827 | 2646.57935544204 | Ecuador |
| 1995 | 2771.19734840387 | 0.529380751671009 | 181.362819288116 | 2589.83452911575 | Ecuador |
| 1996 | 2739.46636607018 | 0.532691399133141 | 171.563264468569 | 2567.90310160161 | Ecuador |
| 1997 | 2517.42619757599 | 0.535349652252826 | 161.265339116163 | 2356.16085845983 | Ecuador |
| 1998 | 2385.08396896053 | 0.537196329914338 | 158.271353511611 | 2226.81261544892 | Ecuador |
| 1999 | 2112.88101984232 | 0.53903230782875 | 156.54343568859 | 1956.33758415373 | Ecuador |
| 2000 | 2026.79608614836 | 0.5430447789024 | 155.277879664217 | 1871.51820648414 | Ecuador |
| 2001 | 1858.65669957786 | 0.548552149690973 | 149.563221566699 | 1709.09347801116 | Ecuador |
| 2002 | 1686.7289139019 | 0.554897325204538 | 143.675014762969 | 1543.05389913893 | Ecuador |
| 2003 | 1581.78953015358 | 0.560955484518967 | 139.013191109321 | 1442.77633904426 | Ecuador |
| 2004 | 1382.72268302224 | 0.566290292625305 | 107.337064294466 | 1275.38561872777 | Ecuador |
| 2005 | 1219.62845459939 | 0.569927344748438 | 95.0496706901285 | 1124.57878390926 | Ecuador |
| 2006 | 1042.22715287219 | 0.572516273171735 | 82.3918952171374 | 959.835257655052 | Ecuador |
| 2007 | 815.42965931435 | 0.574704702310921 | 82.620221139886 | 732.809438174464 | Ecuador |
| 2008 | 561.48480454203 | 0.578125040269336 | 83.5117305405015 | 477.973074001529 | Ecuador |
| 2009 | 418.32635078393 | 0.58248425757379 | 83.2823310811706 | 335.044019702759 | Ecuador |
| 2010 | 303.57756981827 | 0.588763055747133 | 83.657899279854 | 219.919670538416 | Ecuador |
| 2011 | 259.94440991122 | 0.596729076106647 | 78.8362322771186 | 181.108177634101 | Ecuador |
| 2012 | 211.38953083202 | 0.605454319333254 | 76.6293227841581 | 134.760208047862 | Ecuador |
| 2013 | 177.08190030378 | 0.614080274696922 | 68.4978273879911 | 108.584072915789 | Ecuador |
| 2014 | 165.26274268806 | 0.622027432994838 | 62.2598645018797 | 103.00287818618 | Ecuador |
| 2015 | 173.64261843232 | 0.628546709567119 | 55.3744522262929 | 118.268166206027 | Ecuador |
| 2016 | 144.09852501219 | 0.634281142866422 | 53.2039817181422 | 90.8945432940478 | Ecuador |
| 2017 | 113.15075365467 | 0.640011218750157 | 50.4075743311926 | 62.7431793234774 | Ecuador |
| 2018 | 98.44630577 | 0.645888520186892 | 49.2916071531252 | 49.1546986133548 | Ecuador |
| 2019 | 98.79457271 | 0.651787987044895 | 46.7446415327265 | 52.0499311762435 | Ecuador |
| 2020 | 102.69717980838 | 0.65671445724893 | 45.366042416111 | 57.331137392269 | Ecuador |
| 2021 | 91.94484116 | 0.66101705262103 | 42.6391815521747 | 49.3056596119454 | Ecuador |
| 1990 | 3819.59663130648 | 0.510419852074136 | 212.952611777237 | 3606.64401952924 | Peru |
| 1991 | 4877.80883204615 | 0.512161524663903 | 212.988373411545 | 4664.82045863461 | Peru |
| 1992 | 3802.44980001195 | 0.513821551605533 | 208.980155092126 | 3593.46964491982 | Peru |
| 1993 | 3401.85171939082 | 0.515981506410929 | 203.407503817931 | 3198.44421557289 | Peru |
| 1994 | 2784.71757998451 | 0.519306080333588 | 199.510102306722 | 2585.20747767779 | Peru |
| 1995 | 2490.6417973166 | 0.523640526677899 | 191.060866567626 | 2299.58093074897 | Peru |
| 1996 | 2162.11500611989 | 0.528312042097199 | 179.868032483669 | 1982.24697363622 | Peru |
| 1997 | 1945.12174338398 | 0.533576491199309 | 170.367274455972 | 1774.75446892801 | Peru |
| 1998 | 1785.97266970918 | 0.538721717199262 | 156.729827538309 | 1629.24284217087 | Peru |
| 1999 | 1602.23048223694 | 0.543853376891845 | 155.407564160312 | 1446.82291807663 | Peru |
| 2000 | 1433.66993536809 | 0.548952201243799 | 148.941026416118 | 1284.72890895197 | Peru |
| 2001 | 1236.57783991516 | 0.553775453477556 | 144.081392996644 | 1092.49644691852 | Peru |
| 2002 | 1082.69449555112 | 0.558853262904608 | 141.749233691307 | 940.945261859813 | Peru |
| 2003 | 959.45296 | 0.563871071930545 | 137.49075161036 | 821.96220836874 | Peru |
| 2004 | 845.23867119853 | 0.56906302441994 | 110.458702416354 | 734.779968782176 | Peru |
| 2005 | 750.58724091216 | 0.574517001323253 | 86.9100974950069 | 663.677143417153 | Peru |
| 2006 | 645.92356526204 | 0.580503364933972 | 82.2223031786084 | 563.701262083432 | Peru |
| 2007 | 532.00709969807 | 0.586958489258033 | 84.2788131256758 | 447.728286572394 | Peru |
| 2008 | 453.45390741262 | 0.593750810824365 | 78.678453632948 | 374.775453779672 | Peru |
| 2009 | 362.47608390694 | 0.599954992710361 | 74.2950146182131 | 288.181069288727 | Peru |
| 2010 | 295.82061469689 | 0.606520931663104 | 74.7143772218827 | 221.106237475007 | Peru |
| 2011 | 245.10602911434 | 0.613051220911462 | 68.4006668741398 | 176.7053622 | Peru |
| 2012 | 203.48302607604 | 0.619355264313384 | 62.2880847151015 | 141.194941360938 | Peru |
| 2013 | 173.31208047592 | 0.62551902425403 | 60.5795107968907 | 112.732569679029 | Peru |
| 2014 | 151.20809779986 | 0.631114354280049 | 53.1378954863669 | 98.0702023134931 | Peru |
| 2015 | 135.52914022976 | 0.636278270548633 | 53.6348425841638 | 81.8942976455962 | Peru |
| 2016 | 127.096238 | 0.641285279069023 | 51.2123322360966 | 75.8839057302034 | Peru |
| 2017 | 120.13476616201 | 0.64618085494837 | 49.5378910522562 | 70.5968751097537 | Peru |
| 2018 | 116.87339150015 | 0.650977317324287 | 46.739503700009 | 70.133887800141 | Peru |
| 2019 | 104.15279164065 | 0.655433734747709 | 45.5565351557017 | 58.5962564849483 | Peru |
| 2020 | 156.66146392155 | 0.658672243500517 | 45.1349069955309 | 111.526556926019 | Peru |
| 2021 | 144.89946259199 | 0.662054036755862 | 43.3781160376322 | 101.521346554358 | Peru |
| 1990 | 3854.14971398343 | 0.480720053627064 | 324.588214206276 | 3529.56149977715 | Colombia |
| 1991 | 3626.50965226501 | 0.482339893974629 | 325.288820984017 | 3301.22083128099 | Colombia |
| 1992 | 3579.36586172613 | 0.484572358989267 | 325.115729316295 | 3254.25013240984 | Colombia |
| 1993 | 3175.62715008074 | 0.488229373054794 | 312.198924367082 | 2863.42822571366 | Colombia |
| 1994 | 2870.25991266256 | 0.493278181852073 | 295.661533126641 | 2574.59837953592 | Colombia |
| 1995 | 2581.16120106225 | 0.49910275736379 | 236.785312040815 | 2344.37588902143 | Colombia |
| 1996 | 2324.81149148008 | 0.504986969396651 | 224.569012248085 | 2100.24247923199 | Colombia |
| 1997 | 2103.18934840072 | 0.511079502295084 | 212.973297532772 | 1890.21605086795 | Colombia |
| 1998 | 1929.10833025043 | 0.517022504946273 | 203.311424935891 | 1725.79690531454 | Colombia |
| 1999 | 1587.20411531498 | 0.522435940409787 | 199.086296811121 | 1388.11781850386 | Colombia |
| 2000 | 1400.35459359328 | 0.527839094091296 | 181.299375869847 | 1219.05521772343 | Colombia |
| 2001 | 1207.76561955637 | 0.533076500590306 | 172.581011193943 | 1035.18460836243 | Colombia |
| 2002 | 1318.6952419367 | 0.538189342486614 | 156.72380566401 | 1161.97143627269 | Colombia |
| 2003 | 1291.58322886773 | 0.54326513104442 | 155.139986120904 | 1136.44324274683 | Colombia |
| 2004 | 1107.16483173925 | 0.548601831852621 | 149.237581008788 | 957.927250730462 | Colombia |
| 2005 | 941.85480244389 | 0.554089190974456 | 143.856733874071 | 797.998068569819 | Colombia |
| 2006 | 791.86412042181 | 0.56018273477503 | 139.236640008513 | 652.627480413297 | Colombia |
| 2007 | 641.97171010409 | 0.566822644330018 | 111.898105683444 | 530.073604420646 | Colombia |
| 2008 | 463.43146133436 | 0.573494651915472 | 88.5956210582441 | 374.835840276116 | Colombia |
| 2009 | 352.14999187615 | 0.579876468776209 | 82.089865154543 | 270.060126721607 | Colombia |
| 2010 | 249.80458128865 | 0.586408108388396 | 79.5638389391664 | 170.240742349484 | Colombia |
| 2011 | 199.51349250851 | 0.593340477060868 | 79.9300807095389 | 119.583411798971 | Colombia |
| 2012 | 184.00503112237 | 0.600254237718889 | 77.5187051493657 | 106.486325973004 | Colombia |
| 2013 | 181.13135060816 | 0.607250636585575 | 74.7994634455422 | 106.331887162618 | Colombia |
| 2014 | 178.93510289645 | 0.614151922522411 | 68.586985454768 | 110.348117441682 | Colombia |
| 2015 | 180.52421741669 | 0.620889228107476 | 65.0655821435409 | 115.458635273149 | Colombia |
| 2016 | 164.28671615788 | 0.627554497248436 | 57.1247882272898 | 107.16192793059 | Colombia |
| 2017 | 155.8467897 | 0.634130359426935 | 55.8363456361905 | 100.01044408531 | Colombia |
| 2018 | 168.98688483374 | 0.640255071652482 | 50.1106126680703 | 118.87627216567 | Colombia |
| 2019 | 215.59233793694 | 0.646038852086803 | 47.8664736342202 | 167.72586430272 | Colombia |
| 2020 | 232.29362564701 | 0.650855354021142 | 46.1109192541124 | 186.182706392898 | Colombia |
| 2021 | 208.5911334 | 0.655442912568827 | 46.3534695622388 | 162.237663856061 | Colombia |
| 1990 | 783.11754333422 | 0.534125181350537 | 161.527699077826 | 621.589844256394 | Costa Rica |
| 1991 | 647.48241983427 | 0.539342163753933 | 156.907459502198 | 490.574960332072 | Costa Rica |
| 1992 | 552.2376499 | 0.5446242375755 | 155.018620072043 | 397.219029803657 | Costa Rica |
| 1993 | 519.24217659479 | 0.549946625586597 | 145.978689642773 | 373.263486952016 | Costa Rica |
| 1994 | 510.87230864614 | 0.555171250068523 | 144.155459592147 | 366.716849053993 | Costa Rica |
| 1995 | 482.7207641 | 0.560547086077833 | 139.464806214805 | 343.255957876495 | Costa Rica |
| 1996 | 456.10978080043 | 0.565180861960769 | 112.572155560935 | 343.537625239495 | Costa Rica |
| 1997 | 456.48313946591 | 0.570128417464587 | 88.7655010745046 | 367.717638391405 | Costa Rica |
| 1998 | 415.74060323778 | 0.576067050570833 | 84.6798551263801 | 331.0607481 | Costa Rica |
| 1999 | 355.24262688145 | 0.582229491567193 | 80.5999875167453 | 274.642639364705 | Costa Rica |
| 2000 | 297.9252006 | 0.588243191082194 | 80.9643665390417 | 216.960834101858 | Costa Rica |
| 2001 | 278.44914990273 | 0.594889114531861 | 78.8042026972438 | 199.644947205486 | Costa Rica |
| 2002 | 251.30785772765 | 0.600894432967727 | 74.8048732478679 | 176.502984479782 | Costa Rica |
| 2003 | 234.50631244764 | 0.605765686833733 | 75.5218446656973 | 158.984467781943 | Costa Rica |
| 2004 | 201.81682767796 | 0.610315699466228 | 69.9996472944047 | 131.817180383555 | Costa Rica |
| 2005 | 182.90829208607 | 0.614412618734781 | 65.9878369974638 | 116.920455088606 | Costa Rica |
| 2006 | 158.68111834208 | 0.617850162499434 | 63.2814727493669 | 95.3996455927131 | Costa Rica |
| 2007 | 165.27636728206 | 0.621200571426243 | 62.0575403317245 | 103.218826950336 | Costa Rica |
| 2008 | 147.3020962 | 0.625703910462395 | 58.2748416411541 | 89.0272545682459 | Costa Rica |
| 2009 | 140.51325218311 | 0.631291379026346 | 54.5133201095926 | 85.9999320735174 | Costa Rica |
| 2010 | 136.59555203309 | 0.63659542601002 | 53.28654951 | 83.30900253 | Costa Rica |
| 2011 | 121.75240035702 | 0.641201295145018 | 51.2260546008489 | 70.5263457561711 | Costa Rica |
| 2012 | 119.40222618625 | 0.646574092912526 | 49.0665903002797 | 70.3356358859703 | Costa Rica |
| 2013 | 104.61815148621 | 0.651945812639188 | 45.5116543900615 | 59.1064970961485 | Costa Rica |
| 2014 | 114.09515264571 | 0.656890456365803 | 45.2874794151596 | 68.8076732305504 | Costa Rica |
| 2015 | 104.83869509373 | 0.662030135918026 | 43.9935914463732 | 60.8451036473568 | Costa Rica |
| 2016 | 104.18853195251 | 0.667430091500307 | 39.7997876389675 | 64.3887443135425 | Costa Rica |
| 2017 | 87.55148689 | 0.673621391630886 | 38.091756420181 | 49.459730465389 | Costa Rica |
| 2018 | 75.00608799 | 0.681296147310122 | 37.6518970604549 | 37.3541909284751 | Costa Rica |
| 2019 | 82.54323826 | 0.690049088214507 | 36.5025150640675 | 46.0407231982825 | Costa Rica |
| 2020 | 76.59167039 | 0.696194605172093 | 36.6164871087005 | 39.9751832765495 | Costa Rica |
| 2021 | 65.14662895 | 0.700340476999325 | 35.9910196944651 | 29.1556092532649 | Costa Rica |
| 1990 | 6132.27602289073 | 0.373057974861269 | 1208.90782393317 | 4923.36819895756 | El Salvador |
| 1991 | 6046.29125041904 | 0.374855325024228 | 1167.43970080561 | 4878.85154961343 | El Salvador |
| 1992 | 5248.17684291616 | 0.377076642695912 | 1177.6430098613 | 4070.53383305486 | El Salvador |
| 1993 | 4620.69273870221 | 0.380307326030095 | 1131.80662710818 | 3488.88611159403 | El Salvador |
| 1994 | 4246.19668969943 | 0.38514909404887 | 1086.9020587219 | 3159.29463097753 | El Salvador |
| 1995 | 3700.76634560829 | 0.391780266086918 | 1046.05225916876 | 2654.71408643953 | El Salvador |
| 1996 | 3273.06983891082 | 0.400178467721391 | 936.655007002066 | 2336.41483190875 | El Salvador |
| 1997 | 2842.00775796624 | 0.410455825709854 | 856.33360515141 | 1985.67415281483 | El Salvador |
| 1998 | 2438.37654918507 | 0.421421189598308 | 777.886615755572 | 1660.4899334295 | El Salvador |
| 1999 | 2059.00409772113 | 0.432733147562664 | 428.583541004405 | 1630.42055671672 | El Salvador |
| 2000 | 1767.91514461548 | 0.443962665887944 | 425.824099088689 | 1342.09104552679 | El Salvador |
| 2001 | 1522.28849456943 | 0.454177658570425 | 414.27829606536 | 1108.01019850407 | El Salvador |
| 2002 | 1332.54112568387 | 0.462968859826757 | 379.642449406832 | 952.898676277038 | El Salvador |
| 2003 | 1166.54313702693 | 0.470397412659385 | 342.952306340206 | 823.590830686724 | El Salvador |
| 2004 | 1049.86651755896 | 0.476536664263465 | 334.55023232714 | 715.31628523182 | El Salvador |
| 2005 | 945.30518367862 | 0.48204171929172 | 324.871682039045 | 620.433501639575 | El Salvador |
| 2006 | 745.84137166843 | 0.486826000431208 | 319.934059754706 | 425.907311913724 | El Salvador |
| 2007 | 583.03157592712 | 0.490809751570084 | 307.116734334427 | 275.914841592693 | El Salvador |
| 2008 | 444.54329681136 | 0.495077738859345 | 281.18632452156 | 163.3569723 | El Salvador |
| 2009 | 362.03193999672 | 0.499489714249212 | 235.669797523115 | 126.362142473605 | El Salvador |
| 2010 | 299.721797 | 0.504171335202455 | 223.760436205474 | 75.9613607843262 | El Salvador |
| 2011 | 276.42252623062 | 0.508969239001561 | 220.52577246493 | 55.8967537656904 | El Salvador |
| 2012 | 254.97846571144 | 0.513396874335326 | 207.09826234597 | 47.8802033654695 | El Salvador |
| 2013 | 236.99270103614 | 0.518121907716899 | 203.887453882576 | 33.1052471535639 | El Salvador |
| 2014 | 217.58226500964 | 0.523468900855703 | 189.50638615485 | 28.0758788547901 | El Salvador |
| 2015 | 196.95169644556 | 0.529386950266238 | 180.953537323119 | 15.9981591224413 | El Salvador |
| 2016 | 184.27409739736 | 0.535650269148693 | 160.182447461772 | 24.0916499355877 | El Salvador |
| 2017 | 174.84226319129 | 0.541998957448254 | 155.141946605936 | 19.700316585354 | El Salvador |
| 2018 | 167.72906541248 | 0.548143173580896 | 149.77136745395 | 17.9576979585303 | El Salvador |
| 2019 | 162.99620437423 | 0.55403164778242 | 143.457909902342 | 19.5382944718878 | El Salvador |
| 2020 | 189.2055745 | 0.558919894116032 | 142.3571998 | 46.8483747546998 | El Salvador |
| 2021 | 179.13246121346 | 0.563775187564695 | 137.6220295 | 41.5104317606599 | El Salvador |
| 1990 | 8623.62703542074 | 0.311792455277134 | 2107.3533731455 | 6516.27366227524 | Guatemala |
| 1991 | 8143.0971333061 | 0.314329483676405 | 2119.00100231671 | 6024.09613098939 | Guatemala |
| 1992 | 6872.67534042015 | 0.319165481239087 | 2100.6524090283 | 4772.02293139185 | Guatemala |
| 1993 | 6236.29304953808 | 0.326840818669512 | 2059.81446758563 | 4176.47858195245 | Guatemala |
| 1994 | 5985.29505697657 | 0.33346910005957 | 1982.64219549132 | 4002.65286148525 | Guatemala |
| 1995 | 5596.12556905563 | 0.339642100810868 | 1858.14955824248 | 3737.97601081315 | Guatemala |
| 1996 | 5299.97242442708 | 0.346961212155122 | 1770.9505670418 | 3529.02185738528 | Guatemala |
| 1997 | 4963.57700913827 | 0.355995963296002 | 1750.19009282636 | 3213.38691631191 | Guatemala |
| 1998 | 4868.71066899134 | 0.366461192871903 | 1259.12569690334 | 3609.584972 | Guatemala |
| 1999 | 4408.36081840801 | 0.374455316256445 | 1193.05332501241 | 3215.3074933956 | Guatemala |
| 2000 | 4473.89203366641 | 0.379877296535484 | 1133.58750307108 | 3340.30453059533 | Guatemala |
| 2001 | 4379.75224568624 | 0.386927685682523 | 1092.8119461795 | 3286.94029950674 | Guatemala |
| 2002 | 4113.22268117149 | 0.396899639101221 | 979.921077208879 | 3133.30160396261 | Guatemala |
| 2003 | 3714.75536161753 | 0.408234164151934 | 870.549966546226 | 2844.2053950713 | Guatemala |
| 2004 | 3339.59145055757 | 0.419777064914249 | 776.841536903773 | 2562.7499136538 | Guatemala |
| 2005 | 3102.5887165871 | 0.430327529380029 | 440.580619208425 | 2662.00809737868 | Guatemala |
| 2006 | 2701.3968115683 | 0.438856375714513 | 420.62308401268 | 2280.77372755562 | Guatemala |
| 2007 | 2425.42301163454 | 0.447245964554675 | 411.04915280615 | 2014.37385882839 | Guatemala |
| 2008 | 2105.74009072865 | 0.456541865045794 | 398.279520581397 | 1707.46057014725 | Guatemala |
| 2009 | 1746.11639876078 | 0.464003076644959 | 376.537632027576 | 1369.5787667332 | Guatemala |
| 2010 | 1608.11684919178 | 0.470477363646759 | 342.507807906293 | 1265.60904128549 | Guatemala |
| 2011 | 1544.40903267127 | 0.476909684291273 | 333.46619140305 | 1210.94284126822 | Guatemala |
| 2012 | 1497.03511148497 | 0.481862853245964 | 324.375620993246 | 1172.65949049172 | Guatemala |
| 2013 | 1411.42947808692 | 0.487286692231106 | 317.458089567738 | 1093.97138851918 | Guatemala |
| 2014 | 1393.88566724329 | 0.494027188122075 | 295.539804981141 | 1098.34586226215 | Guatemala |
| 2015 | 1328.34126093798 | 0.500655606612947 | 230.449656739684 | 1097.8916041983 | Guatemala |
| 2016 | 1332.30384551589 | 0.507149791967016 | 218.034686052414 | 1114.26915946348 | Guatemala |
| 2017 | 1184.77803168668 | 0.51373568188905 | 206.613253733987 | 978.164777952693 | Guatemala |
| 2018 | 1095.23983786722 | 0.52099546880295 | 197.591986184341 | 897.647851682879 | Guatemala |
| 2019 | 1127.50570556081 | 0.528630469662807 | 179.99144732268 | 947.51425823813 | Guatemala |
| 2020 | 1115.48699450495 | 0.534571414248156 | 160.246646660563 | 955.240347844387 | Guatemala |
| 2021 | 970.98633614322 | 0.539972423611408 | 156.189107765946 | 814.797228377274 | Guatemala |
| 1990 | 9786.98961194256 | 0.332042889051331 | 1990.26416827973 | 7796.72544366283 | Honduras |
| 1991 | 9297.16807991487 | 0.337034649170168 | 1921.41734010524 | 7375.75073980963 | Honduras |
| 1992 | 8701.18161621257 | 0.342305656800435 | 1765.28294186804 | 6935.89867434453 | Honduras |
| 1993 | 8418.18220245378 | 0.347838445661094 | 1773.0517245129 | 6645.13047794088 | Honduras |
| 1994 | 7816.60134757618 | 0.353164851646287 | 1765.83050894788 | 6050.7708386283 | Honduras |
| 1995 | 7112.75748867155 | 0.358787268251117 | 1646.5301992613 | 5466.22728941026 | Honduras |
| 1996 | 6233.66657708528 | 0.364328424323186 | 1283.51437726579 | 4950.15219981949 | Honduras |
| 1997 | 5604.21457158967 | 0.369983893774573 | 1210.32264514967 | 4393.891926 | Honduras |
| 1998 | 5113.99657010463 | 0.375950111095537 | 1168.11563891272 | 3945.88093119191 | Honduras |
| 1999 | 4608.76547486254 | 0.38175231641304 | 1135.63799427662 | 3473.12748058592 | Honduras |
| 2000 | 4171.02408157185 | 0.387988052509884 | 1100.73240710567 | 3070.29167446618 | Honduras |
| 2001 | 3825.34731076889 | 0.394174011784655 | 976.609144401968 | 2848.73816636692 | Honduras |
| 2002 | 3523.56173561083 | 0.400371798466223 | 929.267713152851 | 2594.29402245798 | Honduras |
| 2003 | 3260.46395354288 | 0.406668380994452 | 870.327338766811 | 2390.13661477607 | Honduras |
| 2004 | 3032.33181326607 | 0.413061815539536 | 856.57515033528 | 2175.75666293079 | Honduras |
| 2005 | 2812.73120959683 | 0.419612331829645 | 778.040897287124 | 2034.69031230971 | Honduras |
| 2006 | 2539.56875219296 | 0.426352996817521 | 529.29424790229 | 2010.27450429067 | Honduras |
| 2007 | 2156.8399703652 | 0.433240314987847 | 419.496758395799 | 1737.3432119694 | Honduras |
| 2008 | 1817.14420741149 | 0.439998163793669 | 429.826298067151 | 1387.31790934434 | Honduras |
| 2009 | 1400.24451299161 | 0.446151054184542 | 410.241611997518 | 990.002900994092 | Honduras |
| 2010 | 1253.91679232199 | 0.452382226389866 | 410.275925819367 | 843.640866502623 | Honduras |
| 2011 | 1177.59374014119 | 0.458700328960259 | 396.187923356234 | 781.405816784956 | Honduras |
| 2012 | 1100.22092058264 | 0.46492515492495 | 382.053625044146 | 718.167295538494 | Honduras |
| 2013 | 1047.61118118476 | 0.470766833403007 | 341.38053617338 | 706.23064501138 | Honduras |
| 2014 | 995.81865836956 | 0.476587799009017 | 336.381412555069 | 659.437245814491 | Honduras |
| 2015 | 941.41968830169 | 0.482294176352814 | 325.540323774494 | 615.879364527196 | Honduras |
| 2016 | 894.31157618376 | 0.487705931888242 | 310.01376568497 | 584.29781049879 | Honduras |
| 2017 | 855.34880779273 | 0.493130694434939 | 295.020348425286 | 560.328459367444 | Honduras |
| 2018 | 799.75664588076 | 0.498657226690384 | 236.798509289864 | 562.958136590896 | Honduras |
| 2019 | 750.7078729 | 0.504156505325121 | 223.051834013839 | 527.656038846061 | Honduras |
| 2020 | 795.41224550997 | 0.508669245938408 | 219.035052119051 | 576.377193390919 | Honduras |
| 2021 | 713.99490182591 | 0.513037248307344 | 207.03441391456 | 506.96048791135 | Honduras |
| 1990 | 10337.5562560416 | 0.504996083384899 | 224.043934767239 | 10113.5123212744 | Mexico |
| 1991 | 9111.47079144684 | 0.509510670028127 | 214.192245989888 | 8897.27854545695 | Mexico |
| 1992 | 7500.43748455888 | 0.514638056848767 | 207.237365267813 | 7293.20011929107 | Mexico |
| 1993 | 6394.9744244118 | 0.52049316730006 | 199.179381389806 | 6195.795043 | Mexico |
| 1994 | 5261.59203285636 | 0.526749240631648 | 189.79687237271 | 5071.79516048365 | Mexico |
| 1995 | 4558.25343817688 | 0.531554141210122 | 179.9677261 | 4378.28571205888 | Mexico |
| 1996 | 3953.35683489594 | 0.536530826504039 | 157.991508686769 | 3795.36532620917 | Mexico |
| 1997 | 3538.03403094036 | 0.541988171199547 | 154.959963097843 | 3383.07406784252 | Mexico |
| 1998 | 3113.30459524126 | 0.547842121275417 | 149.225697905434 | 2964.07889733583 | Mexico |
| 1999 | 2746.52330383199 | 0.553813369752955 | 144.005250284816 | 2602.51805354717 | Mexico |
| 2000 | 2498.51399203287 | 0.559532546937005 | 139.146970603421 | 2359.36702142945 | Mexico |
| 2001 | 2272.46079094811 | 0.564774176443707 | 114.79434479916 | 2157.66644614895 | Mexico |
| 2002 | 2014.8825542345 | 0.570113531020311 | 86.9393521562198 | 1927.94320207828 | Mexico |
| 2003 | 1758.2955858574 | 0.575698214652787 | 86.6380016621191 | 1671.65758419528 | Mexico |
| 2004 | 1477.86076327524 | 0.580984982494851 | 80.5954727249376 | 1397.2652905503 | Mexico |
| 2005 | 1301.09180684959 | 0.585224080225789 | 84.2716757668048 | 1216.82013108279 | Mexico |
| 2006 | 1065.74637609056 | 0.588543226375938 | 81.5749906817647 | 984.171385408795 | Mexico |
| 2007 | 893.03956602307 | 0.591447407661459 | 80.3609376773108 | 812.678628345759 | Mexico |
| 2008 | 690.98407734536 | 0.594454558028247 | 75.3862388888809 | 615.597838456479 | Mexico |
| 2009 | 525.73159042138 | 0.596684357960512 | 78.5564753663792 | 447.175115055001 | Mexico |
| 2010 | 436.97633928314 | 0.599527028083825 | 76.5087034358758 | 360.467635847264 | Mexico |
| 2011 | 423.12055730051 | 0.60364609507818 | 76.1526980623812 | 346.967859238129 | Mexico |
| 2012 | 453.14271272027 | 0.608914289338676 | 69.7082781693056 | 383.434434550964 | Mexico |
| 2013 | 494.3687621 | 0.615089992820613 | 69.4157429910201 | 424.95301913398 | Mexico |
| 2014 | 470.6765645 | 0.621857404659516 | 61.8906340881137 | 408.785930427586 | Mexico |
| 2015 | 448.40481918498 | 0.628896723914502 | 55.760827172989 | 392.643992011991 | Mexico |
| 2016 | 422.57689067961 | 0.635925348800174 | 52.4402402721609 | 370.136650407449 | Mexico |
| 2017 | 356.46722914651 | 0.642667198400828 | 51.4345970780401 | 305.03263206847 | Mexico |
| 2018 | 328.39491983902 | 0.649053317386265 | 47.3724033684771 | 281.022516470543 | Mexico |
| 2019 | 341.92321907539 | 0.655095338007006 | 45.356437209011 | 296.566781866379 | Mexico |
| 2020 | 334.64136817872 | 0.660119061584711 | 43.0132814870263 | 291.628086691694 | Mexico |
| 2021 | 322.98334639711 | 0.664575304259183 | 40.7417862676432 | 282.241560129467 | Mexico |
| 1990 | 9192.93622772302 | 0.346035234504054 | 1765.63503012867 | 7427.30119759435 | Nicaragua |
| 1991 | 8277.93962051651 | 0.35174062237875 | 1763.04679074153 | 6514.89282977498 | Nicaragua |
| 1992 | 7561.89159002529 | 0.357712704444006 | 1716.79117442075 | 5845.10041560454 | Nicaragua |
| 1993 | 7469.62368076408 | 0.36356206850317 | 1411.01202090543 | 6058.61165985865 | Nicaragua |
| 1994 | 6455.03697089934 | 0.369727967895652 | 1252.06039267685 | 5202.97657822249 | Nicaragua |
| 1995 | 5915.21360069346 | 0.37662534522155 | 1173.30888846746 | 4741.904712 | Nicaragua |
| 1996 | 5093.62195751984 | 0.384087266113663 | 1087.75462967826 | 4005.86732784158 | Nicaragua |
| 1997 | 4271.32864784928 | 0.391775893494114 | 1044.66891276433 | 3226.65973508495 | Nicaragua |
| 1998 | 3766.73744416327 | 0.399807534850283 | 934.241927260493 | 2832.49551690278 | Nicaragua |
| 1999 | 3232.5914019065 | 0.408085964812584 | 876.998658364977 | 2355.59274354152 | Nicaragua |
| 2000 | 2829.94937404825 | 0.416108362910212 | 795.917385569813 | 2034.03198847844 | Nicaragua |
| 2001 | 2515.68056685183 | 0.423790295277938 | 685.630015140021 | 1830.05055171181 | Nicaragua |
| 2002 | 2224.33102919293 | 0.43081375452391 | 427.377395370586 | 1796.95363382234 | Nicaragua |
| 2003 | 1959.89084890951 | 0.437248152494534 | 419.393437077537 | 1540.49741183197 | Nicaragua |
| 2004 | 1741.65868851686 | 0.443274510891091 | 423.224002700942 | 1318.43468581592 | Nicaragua |
| 2005 | 1559.33039658869 | 0.448844739044126 | 409.261308885903 | 1150.06908770279 | Nicaragua |
| 2006 | 1191.43957193042 | 0.453950499137161 | 406.194772125023 | 785.244799805397 | Nicaragua |
| 2007 | 953.28097943782 | 0.459042797856105 | 397.61330146982 | 555.667678 | Nicaragua |
| 2008 | 737.32345872284 | 0.463837699218163 | 381.270057211618 | 356.053401511222 | Nicaragua |
| 2009 | 583.86567707824 | 0.467961838989771 | 362.547967332477 | 221.317709745763 | Nicaragua |
| 2010 | 499.66150872053 | 0.472171999845355 | 344.412094250853 | 155.249414469677 | Nicaragua |
| 2011 | 454.37116748066 | 0.4765934659805 | 334.1831465 | 120.18802101586 | Nicaragua |
| 2012 | 414.85058688741 | 0.481296900452524 | 322.989999651032 | 91.8605872363776 | Nicaragua |
| 2013 | 384.62743841678 | 0.486105470178736 | 317.397891962359 | 67.2295464544214 | Nicaragua |
| 2014 | 355.36308583579 | 0.491207346240634 | 298.996629405802 | 56.3664564299876 | Nicaragua |
| 2015 | 332.30543079315 | 0.496391662501118 | 266.087134851907 | 66.2182959412435 | Nicaragua |
| 2016 | 305.28983929273 | 0.501853344913168 | 230.510095631639 | 74.7797436610907 | Nicaragua |
| 2017 | 291.26723430543 | 0.507482021944309 | 219.861132751168 | 71.4061015542623 | Nicaragua |
| 2018 | 281.03754296048 | 0.512338227599941 | 213.492090856527 | 67.5454521039533 | Nicaragua |
| 2019 | 275.54833072356 | 0.516597045511253 | 203.004748140514 | 72.5435825830457 | Nicaragua |
| 2020 | 307.48455201903 | 0.520292670400273 | 199.468163031795 | 108.016388987235 | Nicaragua |
| 2021 | 262.48701406773 | 0.523958472235925 | 189.565142958037 | 72.9218711096933 | Nicaragua |
| 1990 | 2045.40216439099 | 0.546048123421677 | 149.059524423483 | 1896.34263996751 | Panama |
| 1991 | 1932.25634339247 | 0.550484218189226 | 146.382253410369 | 1785.8740899821 | Panama |
| 1992 | 1711.14482017595 | 0.55497275492945 | 143.304532609107 | 1567.84028756684 | Panama |
| 1993 | 1333.95833882138 | 0.55916866562951 | 142.304864016466 | 1191.65347480491 | Panama |
| 1994 | 1203.96371604807 | 0.561714534049174 | 139.416702683718 | 1064.54701336435 | Panama |
| 1995 | 1181.69944261033 | 0.56335302312626 | 139.235856962064 | 1042.46358564827 | Panama |
| 1996 | 1164.15209265775 | 0.565310070207258 | 111.729483151687 | 1052.42260950606 | Panama |
| 1997 | 1146.12931773255 | 0.569395938819109 | 110.660287840863 | 1035.46902989169 | Panama |
| 1998 | 1210.68059112415 | 0.574697317744379 | 86.2782121658545 | 1124.4023789583 | Panama |
| 1999 | 1064.56321341217 | 0.580432842358969 | 83.5763754421758 | 980.986837969994 | Panama |
| 2000 | 1039.87033465843 | 0.587378392850747 | 80.6276961243608 | 959.242638534069 | Panama |
| 2001 | 1034.29866715554 | 0.594535700601609 | 76.8902471283246 | 957.408420027215 | Panama |
| 2002 | 971.72255418728 | 0.600138677301078 | 75.9245185682774 | 895.798035619003 | Panama |
| 2003 | 1022.92338612736 | 0.604043364755692 | 75.3964513946406 | 947.526934732719 | Panama |
| 2004 | 1046.22020552164 | 0.606877030015399 | 74.3949916797228 | 971.825213841917 | Panama |
| 2005 | 1074.12961234627 | 0.609494124501125 | 70.2839999171546 | 1003.84561242912 | Panama |
| 2006 | 886.34784737779 | 0.612249712793622 | 71.395547957103 | 814.952299420687 | Panama |
| 2007 | 840.78204275068 | 0.615285417478616 | 69.6283236427998 | 771.15371910788 | Panama |
| 2008 | 632.97081035866 | 0.61895890692398 | 61.9071266587704 | 571.06368369989 | Panama |
| 2009 | 613.23471832252 | 0.622402805678368 | 60.6403728420618 | 552.594345480458 | Panama |
| 2010 | 503.62968328449 | 0.625116323303972 | 60.0281242692734 | 443.601559015217 | Panama |
| 2011 | 451.73910146918 | 0.628633942402595 | 56.093238240481 | 395.645863228699 | Panama |
| 2012 | 439.32959648056 | 0.634263435389173 | 53.0949130430529 | 386.234683437507 | Panama |
| 2013 | 438.74211138449 | 0.641324352588811 | 50.2893946257485 | 388.452716758742 | Panama |
| 2014 | 415.21084037735 | 0.649108173564656 | 46.8352721397077 | 368.375568237642 | Panama |
| 2015 | 362.69003935926 | 0.657962046843146 | 45.3135912863427 | 317.376448072917 | Panama |
| 2016 | 405.8144868 | 0.667276758021587 | 39.5505445325859 | 366.263942280914 | Panama |
| 2017 | 403.00764358183 | 0.676783503431544 | 38.1661534136738 | 364.841490168156 | Panama |
| 2018 | 353.56013239585 | 0.686719924606419 | 37.2066058589885 | 316.353526536862 | Panama |
| 2019 | 326.12895648486 | 0.696670680246603 | 35.9458781291733 | 290.183078355687 | Panama |
| 2020 | 392.72034357719 | 0.703788489528527 | 34.6267335734159 | 358.093610003774 | Panama |
| 2021 | 374.39982750853 | 0.708864827518009 | 34.3750479166541 | 340.024779591876 | Panama |
| 1990 | 5267.93388801896 | 0.516891218383329 | 203.672550005036 | 5064.26133801392 | Venezuela (Bolivarian Republic of) |
| 1991 | 4002.97344704915 | 0.522272295981046 | 198.597981586419 | 3804.37546546273 | Venezuela (Bolivarian Republic of) |
| 1992 | 3834.59305631513 | 0.529517990878198 | 178.653934174763 | 3655.93912214037 | Venezuela (Bolivarian Republic of) |
| 1993 | 3865.95437802153 | 0.536279978196324 | 158.846303289952 | 3707.10807473158 | Venezuela (Bolivarian Republic of) |
| 1994 | 4565.11525988462 | 0.541866057904128 | 155.265606323128 | 4409.84965356149 | Venezuela (Bolivarian Republic of) |
| 1995 | 4350.79491119077 | 0.546348233569071 | 149.31246101921 | 4201.48245017156 | Venezuela (Bolivarian Republic of) |
| 1996 | 3935.39229595079 | 0.548649226233627 | 149.275856425091 | 3786.1164395257 | Venezuela (Bolivarian Republic of) |
| 1997 | 3336.79981416987 | 0.552364437231236 | 145.8818675 | 3190.91794671657 | Venezuela (Bolivarian Republic of) |
| 1998 | 3057.24542328892 | 0.557243117253564 | 141.747885902813 | 2915.49753738611 | Venezuela (Bolivarian Republic of) |
| 1999 | 2639.71737471306 | 0.560277999100226 | 139.06031769882 | 2500.65705701424 | Venezuela (Bolivarian Republic of) |
| 2000 | 2326.35516561609 | 0.56350626012771 | 138.928451992435 | 2187.42671362365 | Venezuela (Bolivarian Republic of) |
| 2001 | 2135.55564409535 | 0.565458381141816 | 111.422917889922 | 2024.13272620543 | Venezuela (Bolivarian Republic of) |
| 2002 | 2125.84312345329 | 0.562604461024786 | 138.737990210881 | 1987.10513324241 | Venezuela (Bolivarian Republic of) |
| 2003 | 2539.1797309908 | 0.555212224179424 | 144.078496481202 | 2395.1012345096 | Venezuela (Bolivarian Republic of) |
| 2004 | 1913.65754932245 | 0.55097225564581 | 146.235747289675 | 1767.42180203277 | Venezuela (Bolivarian Republic of) |
| 2005 | 1529.21677873721 | 0.553941679272419 | 144.137772645022 | 1385.07900609219 | Venezuela (Bolivarian Republic of) |
| 2006 | 1185.06954246076 | 0.563334189710813 | 138.629958164513 | 1046.43958429625 | Venezuela (Bolivarian Republic of) |
| 2007 | 937.58714639473 | 0.575367174436532 | 85.5543610407774 | 852.032785353953 | Venezuela (Bolivarian Republic of) |
| 2008 | 766.67396294513 | 0.586184886513804 | 81.7516645280403 | 684.92229841709 | Venezuela (Bolivarian Republic of) |
| 2009 | 704.97009655265 | 0.593288421972038 | 79.4993744154589 | 625.470722137191 | Venezuela (Bolivarian Republic of) |
| 2010 | 702.06358855852 | 0.599127922953431 | 75.7970837517876 | 626.266504806732 | Venezuela (Bolivarian Republic of) |
| 2011 | 621.21506008668 | 0.604823483689087 | 75.7339213939441 | 545.481138692736 | Venezuela (Bolivarian Republic of) |
| 2012 | 545.91305986427 | 0.610187465323048 | 69.6952801352029 | 476.217779729067 | Venezuela (Bolivarian Republic of) |
| 2013 | 492.48870934042 | 0.614441024133659 | 68.5934530444778 | 423.895256295942 | Venezuela (Bolivarian Republic of) |
| 2014 | 512.98157898902 | 0.614686806192499 | 70.2851742972167 | 442.696404691803 | Venezuela (Bolivarian Republic of) |
| 2015 | 539.32096627243 | 0.610675356525105 | 69.3199130581806 | 470.001053214249 | Venezuela (Bolivarian Republic of) |
| 2016 | 701.84057824835 | 0.606993212893547 | 75.1888061901738 | 626.651772058176 | Venezuela (Bolivarian Republic of) |
| 2017 | 917.66588353053 | 0.605160130674623 | 75.763939469404 | 841.901944061126 | Venezuela (Bolivarian Republic of) |
| 2018 | 1019.58835083162 | 0.604068247287088 | 74.3698959334145 | 945.218454898206 | Venezuela (Bolivarian Republic of) |
| 2019 | 1064.86375272332 | 0.602313886713059 | 74.3427095871059 | 990.521043136214 | Venezuela (Bolivarian Republic of) |
| 2020 | 998.67439099376 | 0.60003478118243 | 76.2123356931459 | 922.462055300614 | Venezuela (Bolivarian Republic of) |
| 2021 | 935.23518911616 | 0.596513058857335 | 77.0425930920475 | 858.192596024113 | Venezuela (Bolivarian Republic of) |
| 1990 | 5329.37882601348 | 0.500070509167008 | 228.891417582451 | 5100.48740843103 | Brazil |
| 1991 | 4902.33658793081 | 0.50478479847852 | 223.745338394491 | 4678.59124953632 | Brazil |
| 1992 | 4482.17474254426 | 0.508539943299266 | 220.001257505537 | 4262.17348503872 | Brazil |
| 1993 | 4181.30333170758 | 0.512081793142278 | 212.975975898592 | 3968.32735580899 | Brazil |
| 1994 | 3755.75898734566 | 0.515760842475151 | 203.855075994002 | 3551.90391135166 | Brazil |
| 1995 | 3315.18146053965 | 0.519685962646796 | 198.87629309057 | 3116.30516744908 | Brazil |
| 1996 | 2865.02728185606 | 0.523863329360558 | 190.43081829589 | 2674.59646356017 | Brazil |
| 1997 | 2467.90176170096 | 0.528177888704233 | 180.479767966705 | 2287.42199373425 | Brazil |
| 1998 | 2264.70836076308 | 0.532481169179124 | 170.842628097394 | 2093.86573266569 | Brazil |
| 1999 | 1955.22383289551 | 0.536972034740505 | 158.274673351205 | 1796.9491595443 | Brazil |
| 2000 | 1649.3552608966 | 0.542051497254562 | 155.300981209607 | 1494.05427968699 | Brazil |
| 2001 | 1482.44767496193 | 0.547304429543334 | 149.318079194177 | 1333.12959576775 | Brazil |
| 2002 | 1416.81504342542 | 0.552776034620744 | 146.271667099786 | 1270.54337632563 | Brazil |
| 2003 | 1357.39922666595 | 0.55814571028409 | 142.375581682836 | 1215.02364498311 | Brazil |
| 2004 | 1272.07814614965 | 0.563772906923854 | 137.71177976738 | 1134.36636638227 | Brazil |
| 2005 | 1196.55657209208 | 0.569458117692368 | 110.792880775217 | 1085.76369131686 | Brazil |
| 2006 | 1045.97414731849 | 0.575338774786184 | 89.1238083290704 | 956.85033898942 | Brazil |
| 2007 | 817.73401538262 | 0.581588755483488 | 80.9886906858317 | 736.745324696788 | Brazil |
| 2008 | 624.56853223612 | 0.588077817972719 | 81.3462288619416 | 543.222303374178 | Brazil |
| 2009 | 470.53625191121 | 0.594073296681944 | 78.6785502430672 | 391.857701668143 | Brazil |
| 2010 | 370.20775079638 | 0.600445846154568 | 74.1839724839275 | 296.023778312452 | Brazil |
| 2011 | 297.54578636451 | 0.606777290886098 | 74.3989982161838 | 223.146788148326 | Brazil |
| 2012 | 269.1064658 | 0.612619336859312 | 67.9585514479452 | 201.147914392655 | Brazil |
| 2013 | 245.56796144867 | 0.61821823662824 | 64.6585342983967 | 180.909427150273 | Brazil |
| 2014 | 204.46708291073 | 0.623433813964528 | 61.8501755104382 | 142.616907400292 | Brazil |
| 2015 | 176.79838420824 | 0.628149803496659 | 56.0422919554681 | 120.756092252772 | Brazil |
| 2016 | 162.31802036663 | 0.632235114321365 | 54.4855401614611 | 107.832480205169 | Brazil |
| 2017 | 141.73071952732 | 0.636422187733083 | 53.8407117723158 | 87.8900077550042 | Brazil |
| 2018 | 119.98195143148 | 0.640802974287966 | 50.8140349718025 | 69.1679164596775 | Brazil |
| 2019 | 101.49667820428 | 0.645298005000543 | 49.8728471312377 | 51.6238310730423 | Brazil |
| 2020 | 90.07316387 | 0.649201568351328 | 46.2679545746634 | 43.8052092956166 | Brazil |
| 2021 | 80.15480565 | 0.65304388724724 | 45.6286635664155 | 34.5261420806145 | Brazil |
| 1990 | 2207.35172290542 | 0.469527785365773 | 342.732290531959 | 1864.61943237346 | Paraguay |
| 1991 | 2111.42045766079 | 0.474352286489022 | 337.726891055484 | 1773.69356660531 | Paraguay |
| 1992 | 2179.99419913946 | 0.479314168681291 | 324.454347317732 | 1855.53985182173 | Paraguay |
| 1993 | 2014.3838388983 | 0.484614200390594 | 323.378924140163 | 1691.00491475814 | Paraguay |
| 1994 | 1855.55719593043 | 0.490226934517601 | 310.055118103066 | 1545.50207782736 | Paraguay |
| 1995 | 1748.85409085645 | 0.496336876135338 | 264.346221091766 | 1484.50786976468 | Paraguay |
| 1996 | 1578.25991266791 | 0.502322804124491 | 229.270003384345 | 1348.98990928356 | Paraguay |
| 1997 | 1380.5058638761 | 0.508286947996083 | 219.454039584046 | 1161.05182429205 | Paraguay |
| 1998 | 1220.48745685642 | 0.513819895911737 | 207.471881443639 | 1013.01557541278 | Paraguay |
| 1999 | 1097.11914170442 | 0.518720583214312 | 204.369102471713 | 892.750039232707 | Paraguay |
| 2000 | 991.88304551595 | 0.52300779310211 | 190.404621906465 | 801.478423609484 | Paraguay |
| 2001 | 938.34676029701 | 0.526916763005844 | 192.195770402483 | 746.150989894527 | Paraguay |
| 2002 | 900.48995478656 | 0.531177357815443 | 179.430390501942 | 721.059564284618 | Paraguay |
| 2003 | 832.46278942439 | 0.53536012554352 | 163.321680603955 | 669.141108820435 | Paraguay |
| 2004 | 760.25768106786 | 0.539556655420765 | 156.315507070942 | 603.942173996918 | Paraguay |
| 2005 | 698.26159629185 | 0.543622697051923 | 155.279367444245 | 542.982228847605 | Paraguay |
| 2006 | 604.51245454223 | 0.547943304899878 | 148.904011775452 | 455.608442766778 | Paraguay |
| 2007 | 469.28058290302 | 0.552758676429378 | 146.465206066678 | 322.815376836342 | Paraguay |
| 2008 | 354.20093457361 | 0.55801690264969 | 143.133463380286 | 211.067471193324 | Paraguay |
| 2009 | 273.69159289151 | 0.562900058514746 | 138.715978661566 | 134.975614229944 | Paraguay |
| 2010 | 202.56907113303 | 0.568846562482396 | 108.861366447509 | 93.7077046855214 | Paraguay |
| 2011 | 180.57504864829 | 0.574954463115124 | 85.4730450949831 | 95.1020035533068 | Paraguay |
| 2012 | 164.13746763974 | 0.580650185867766 | 82.3578274638216 | 81.7796401759184 | Paraguay |
| 2013 | 150.35048975934 | 0.587247042264483 | 83.6882213466091 | 66.6622684127309 | Paraguay |
| 2014 | 136.08908846056 | 0.593953153052431 | 77.9472362838387 | 58.1418521767213 | Paraguay |
| 2015 | 117.40978253358 | 0.600484125243426 | 74.2972476780465 | 43.1125348555335 | Paraguay |
| 2016 | 110.42670773617 | 0.607018144917351 | 74.8336244870142 | 35.5930832491558 | Paraguay |
| 2017 | 109.64510929711 | 0.613581590966399 | 68.5898965564784 | 41.0552127406316 | Paraguay |
| 2018 | 105.64556561014 | 0.620032361543249 | 62.6673000641777 | 42.9782655459623 | Paraguay |
| 2019 | 99.89853963 | 0.626070139299015 | 58.0504542654533 | 41.8480853684267 | Paraguay |
| 2020 | 106.91225578326 | 0.631057688560962 | 54.4756154601894 | 52.4366403230706 | Paraguay |
| 2021 | 91.04223452 | 0.635718098852038 | 52.7347106557685 | 38.3075238619715 | Paraguay |
| 1990 | 946.68805345102 | 0.460486905849358 | 398.151335549539 | 548.536717901481 | Algeria |
| 1991 | 853.75395374342 | 0.468319196672477 | 359.915835535607 | 493.838118207813 | Algeria |
| 1992 | 781.04660010181 | 0.475950937138063 | 338.083955139827 | 442.962644961983 | Algeria |
| 1993 | 721.71175647864 | 0.483255591216591 | 326.331318737565 | 395.380437741075 | Algeria |
| 1994 | 670.39834409602 | 0.490450660309052 | 311.060515976753 | 359.337828119267 | Algeria |
| 1995 | 617.04031870421 | 0.497957543180561 | 237.496919606068 | 379.543399098142 | Algeria |
| 1996 | 570.44140470102 | 0.505990967161627 | 220.547217315098 | 349.894187385922 | Algeria |
| 1997 | 544.59738018829 | 0.514056565839869 | 207.172807514782 | 337.424572673508 | Algeria |
| 1998 | 514.21122252777 | 0.522382418796352 | 198.269642340628 | 315.941580187142 | Algeria |
| 1999 | 488.86830941388 | 0.530785582833015 | 180.930751634331 | 307.937557779549 | Algeria |
| 2000 | 465.38013013903 | 0.539481507570335 | 156.100164637791 | 309.279965501239 | Algeria |
| 2001 | 445.05861029919 | 0.54752927722376 | 149.917225736175 | 295.141384563015 | Algeria |
| 2002 | 424.71852864998 | 0.555172221658197 | 144.112996301032 | 280.605532348948 | Algeria |
| 2003 | 407.3502781 | 0.562536956171446 | 139.737944739936 | 267.612333313464 | Algeria |
| 2004 | 395.59621067687 | 0.569352851934701 | 108.952083952482 | 286.644126724388 | Algeria |
| 2005 | 377.64169282371 | 0.576001964539977 | 84.5594751863714 | 293.082217637339 | Algeria |
| 2006 | 357.47721904066 | 0.582126361864573 | 79.6977175179346 | 277.779501522725 | Algeria |
| 2007 | 335.12113585841 | 0.587790538325364 | 79.5553188292194 | 255.565817029191 | Algeria |
| 2008 | 314.52331872382 | 0.593190973247903 | 81.5387148081395 | 232.98460391568 | Algeria |
| 2009 | 292.17558472361 | 0.597870383895018 | 77.907848989252 | 214.267735734358 | Algeria |
| 2010 | 273.80787865934 | 0.602823534656761 | 74.4669974904785 | 199.340881168861 | Algeria |
| 2011 | 256.50880355606 | 0.607825529405856 | 75.1160599151561 | 181.392743640904 | Algeria |
| 2012 | 240.65427914583 | 0.612703533153522 | 68.5752934495351 | 172.078985696295 | Algeria |
| 2013 | 230.18689187443 | 0.617422204461461 | 63.0410255520555 | 167.145866322374 | Algeria |
| 2014 | 219.93886872702 | 0.622087291667118 | 62.2838287432744 | 157.655039983746 | Algeria |
| 2015 | 208.31713760992 | 0.626745568394753 | 57.9822956720305 | 150.33484193789 | Algeria |
| 2016 | 194.24774078811 | 0.631710735505334 | 54.054933012624 | 140.192807775486 | Algeria |
| 2017 | 176.93527653849 | 0.636973175875889 | 51.1791235219739 | 125.756153016516 | Algeria |
| 2018 | 164.81741091702 | 0.642500039273145 | 49.625398566072 | 115.192012350948 | Algeria |
| 2019 | 152.77289347421 | 0.648210784879009 | 47.7486138225021 | 105.024279651708 | Algeria |
| 2020 | 139.88077792493 | 0.65365146606094 | 45.896078795578 | 93.984699129352 | Algeria |
| 2021 | 132.56297207048 | 0.659500924324217 | 43.5874619 | 88.97551017 | Algeria |
| 1990 | 445.06348281346 | 0.584578851787694 | 80.1884540889829 | 364.875028724477 | Bahrain |
| 1991 | 417.16148163325 | 0.590596896738332 | 81.062130600467 | 336.099351032783 | Bahrain |
| 1992 | 388.2561848 | 0.596040229262693 | 78.9456401248727 | 309.310544680927 | Bahrain |
| 1993 | 356.81538149154 | 0.602165621752441 | 76.5586919432891 | 280.256689548251 | Bahrain |
| 1994 | 324.17638463052 | 0.60813372491926 | 68.6272518647705 | 255.549132765749 | Bahrain |
| 1995 | 288.36548431171 | 0.613850721750031 | 70.3425001029875 | 218.022984208722 | Bahrain |
| 1996 | 251.50379045369 | 0.619485376148992 | 63.8039719580186 | 187.699818495671 | Bahrain |
| 1997 | 227.89267217694 | 0.624596061581476 | 60.6670472988577 | 167.225624878082 | Bahrain |
| 1998 | 215.25270792171 | 0.630738301217212 | 56.1599948840406 | 159.092713037669 | Bahrain |
| 1999 | 204.59209941763 | 0.638083186958308 | 50.3113894592975 | 154.280709958333 | Bahrain |
| 2000 | 199.03097903703 | 0.646750697322184 | 50.1114033803205 | 148.91957565671 | Bahrain |
| 2001 | 197.59475631873 | 0.657196063695041 | 44.8961445211604 | 152.69861179757 | Bahrain |
| 2002 | 196.92570500276 | 0.664901201084495 | 40.1744259743131 | 156.751279028447 | Bahrain |
| 2003 | 198.36445294147 | 0.669564110612245 | 38.4757785399227 | 159.888674401547 | Bahrain |
| 2004 | 198.89601865706 | 0.674147775423852 | 37.8495007039177 | 161.046517953142 | Bahrain |
| 2005 | 196.37099080776 | 0.679790598294553 | 37.6584541428872 | 158.712536664873 | Bahrain |
| 2006 | 188.08852462174 | 0.686144289377392 | 37.144249137542 | 150.944275484198 | Bahrain |
| 2007 | 174.75610189878 | 0.693045146522931 | 36.5134554043858 | 138.242646494394 | Bahrain |
| 2008 | 139.74863057196 | 0.699806387259847 | 35.843623755368 | 103.905006816592 | Bahrain |
| 2009 | 123.98451738364 | 0.704885840093356 | 34.4194085304408 | 89.5651088531992 | Bahrain |
| 2010 | 117.34514890395 | 0.707902620468186 | 34.3638429196623 | 82.9813059842877 | Bahrain |
| 2011 | 93.04433829 | 0.708958738960692 | 34.3700675252144 | 58.6742707636956 | Bahrain |
| 2012 | 76.1270206 | 0.710362417505028 | 33.8289125658723 | 42.2981080378977 | Bahrain |
| 2013 | 60.31234245 | 0.713343155899609 | 33.1666019620958 | 27.1457404863642 | Bahrain |
| 2014 | 48.41972824 | 0.716768059555516 | 31.2497644574666 | 17.1699637811134 | Bahrain |
| 2015 | 41.64887965 | 0.719926604884411 | 28.7589508278592 | 12.8899288222208 | Bahrain |
| 2016 | 37.76132783 | 0.723611296528675 | 28.4464878585822 | 9.31483997014778 | Bahrain |
| 2017 | 34.77229911 | 0.729323102530322 | 25.068429300261 | 9.703869812 | Bahrain |
| 2018 | 32.02881024 | 0.736192583172625 | 22.7247213427103 | 9.30408889282965 | Bahrain |
| 2019 | 29.9355641 | 0.742847804247627 | 19.147876121281 | 10.787687983139 | Bahrain |
| 2020 | 34.27846442 | 0.748103083095047 | 18.5687154449152 | 15.7097489755948 | Bahrain |
| 2021 | 33.95647581 | 0.753043204117567 | 17.3513934965783 | 16.6050823129517 | Bahrain |
| 1990 | 7648.35692048755 | 0.417182741758852 | 794.462192375826 | 6853.89472811172 | Egypt |
| 1991 | 7182.51143955138 | 0.426909452028558 | 534.714272791954 | 6647.79716675943 | Egypt |
| 1992 | 6647.00184644725 | 0.437744555478775 | 420.617147037017 | 6226.38469941023 | Egypt |
| 1993 | 5974.63849665233 | 0.448490053084385 | 406.076646963668 | 5568.56184968866 | Egypt |
| 1994 | 5324.03643372252 | 0.458727004203869 | 397.048499195437 | 4926.98793452708 | Egypt |
| 1995 | 4782.36192244547 | 0.468035368434364 | 359.493571271854 | 4422.86835117362 | Egypt |
| 1996 | 4039.8205842353 | 0.476116627128094 | 336.610332255854 | 3703.21025197945 | Egypt |
| 1997 | 3521.5119858312 | 0.482970886044098 | 326.175501726869 | 3195.33648410433 | Egypt |
| 1998 | 3097.47756873366 | 0.489184070606589 | 312.941789893015 | 2784.53577884064 | Egypt |
| 1999 | 2786.93156658498 | 0.495502619174384 | 284.983112352256 | 2501.94845423272 | Egypt |
| 2000 | 2522.69531107021 | 0.502169886288414 | 229.435170372005 | 2293.2601406982 | Egypt |
| 2001 | 2260.38256130499 | 0.508716824226239 | 218.663241278711 | 2041.71932002628 | Egypt |
| 2002 | 1998.99193120175 | 0.515038129463872 | 208.379912542373 | 1790.61201865938 | Egypt |
| 2003 | 1793.4422718049 | 0.520623395469455 | 198.981119284632 | 1594.46115252027 | Egypt |
| 2004 | 1617.56704581261 | 0.525181677996097 | 190.342393687574 | 1427.22465212504 | Egypt |
| 2005 | 1462.82407386094 | 0.528665483094012 | 179.929953532685 | 1282.89412032825 | Egypt |
| 2006 | 1360.37308926126 | 0.530395807271736 | 181.50880318195 | 1178.86428607931 | Egypt |
| 2007 | 1300.35780347305 | 0.529457593890747 | 180.204295121209 | 1120.15350835184 | Egypt |
| 2008 | 1236.30342764548 | 0.5247438325858 | 190.194624745218 | 1046.10880290026 | Egypt |
| 2009 | 1200.67768913787 | 0.51625583066946 | 203.81736266236 | 996.86032647551 | Egypt |
| 2010 | 1137.56792025451 | 0.507715477095215 | 219.05412071223 | 918.51379954228 | Egypt |
| 2011 | 1097.926637 | 0.504594044306814 | 223.110040202092 | 874.816596931908 | Egypt |
| 2012 | 1112.81939257699 | 0.509157068362998 | 214.913975155564 | 897.905417421425 | Egypt |
| 2013 | 1175.53004836854 | 0.51933446386018 | 198.798773882532 | 976.731274486008 | Egypt |
| 2014 | 1135.54478687799 | 0.531749809455032 | 169.796122236621 | 965.748664641369 | Egypt |
| 2015 | 1094.61850588132 | 0.544370148675277 | 155.065433465781 | 939.553072415539 | Egypt |
| 2016 | 1025.29848163715 | 0.556012469227609 | 143.344736851225 | 881.953744785925 | Egypt |
| 2017 | 961.84737717077 | 0.566914631824628 | 110.372750566001 | 851.474626604769 | Egypt |
| 2018 | 906.54418008269 | 0.577442786164725 | 81.593226351711 | 824.950953730979 | Egypt |
| 2019 | 863.09573415733 | 0.587736876524439 | 81.3276694096294 | 781.768064747701 | Egypt |
| 2020 | 748.06112072896 | 0.597363341129599 | 76.2361687104038 | 671.824952018556 | Egypt |
| 2021 | 712.20255684585 | 0.6067870937016 | 73.3900602056701 | 638.81249664018 | Egypt |
| 1990 | 2128.96827072034 | 0.453799943957447 | 407.453965642976 | 1721.51430507736 | Iran (Islamic Republic of) |
| 1991 | 1877.68990161513 | 0.468982857673188 | 344.410952894143 | 1533.27894872099 | Iran (Islamic Republic of) |
| 1992 | 1677.09077356563 | 0.480626696650433 | 323.661687108322 | 1353.42908645731 | Iran (Islamic Republic of) |
| 1993 | 1541.8954793536 | 0.492277025057908 | 292.77581471258 | 1249.11966464102 | Iran (Islamic Republic of) |
| 1994 | 1442.79544401681 | 0.505072857378303 | 224.218575662749 | 1218.57686835406 | Iran (Islamic Republic of) |
| 1995 | 1384.05532658295 | 0.517531493818527 | 203.793113057106 | 1180.26221352584 | Iran (Islamic Republic of) |
| 1996 | 1258.98918101491 | 0.528600719906526 | 180.054267835886 | 1078.93491317902 | Iran (Islamic Republic of) |
| 1997 | 1225.89029047533 | 0.537727779314693 | 156.826582455661 | 1069.06370801967 | Iran (Islamic Republic of) |
| 1998 | 1182.82729192732 | 0.546366017294819 | 149.329429126775 | 1033.49786280054 | Iran (Islamic Republic of) |
| 1999 | 1136.27235395667 | 0.55562433168066 | 143.944162284518 | 992.328191672152 | Iran (Islamic Republic of) |
| 2000 | 1081.91436080658 | 0.565474561419707 | 110.071741985031 | 971.842618821549 | Iran (Islamic Republic of) |
| 2001 | 904.40699392584 | 0.574618208021481 | 84.0379386524632 | 820.369055273377 | Iran (Islamic Republic of) |
| 2002 | 793.33915749652 | 0.584097268490781 | 78.8603001390806 | 714.47885735744 | Iran (Islamic Republic of) |
| 2003 | 690.10078344283 | 0.593963012213832 | 77.2041569945584 | 612.896626448272 | Iran (Islamic Republic of) |
| 2004 | 610.46047328712 | 0.603717449978768 | 76.920536870243 | 533.539936416877 | Iran (Islamic Republic of) |
| 2005 | 559.43721169757 | 0.613291055585393 | 71.5433388260656 | 487.893872871504 | Iran (Islamic Republic of) |
| 2006 | 518.90419061642 | 0.621268095172314 | 61.044207104184 | 457.859983512236 | Iran (Islamic Republic of) |
| 2007 | 481.96712126266 | 0.627749441937933 | 58.1405021548775 | 423.826619107782 | Iran (Islamic Republic of) |
| 2008 | 443.91525993893 | 0.632862230135051 | 53.4508085705964 | 390.464451368334 | Iran (Islamic Republic of) |
| 2009 | 401.49796016017 | 0.637398379541013 | 50.9345561246895 | 350.56340403548 | Iran (Islamic Republic of) |
| 2010 | 361.88322549924 | 0.642559170275463 | 52.0159971490478 | 309.867228350192 | Iran (Islamic Republic of) |
| 2011 | 333.31718983578 | 0.647899089013155 | 48.6743959689118 | 284.642793866868 | Iran (Islamic Republic of) |
| 2012 | 299.34141243342 | 0.651500586996546 | 46.0182268199042 | 253.323185613516 | Iran (Islamic Republic of) |
| 2013 | 278.09074416359 | 0.654475289198172 | 45.5292429663072 | 232.561501197283 | Iran (Islamic Republic of) |
| 2014 | 247.46311733003 | 0.657961510912541 | 45.2425358317405 | 202.22058149829 | Iran (Islamic Republic of) |
| 2015 | 217.1896556 | 0.662057986508516 | 42.4945944978794 | 174.695061105521 | Iran (Islamic Republic of) |
| 2016 | 183.75190014517 | 0.667652445135305 | 39.5745806981761 | 144.177319446994 | Iran (Islamic Republic of) |
| 2017 | 167.4894947 | 0.674343462089889 | 37.8323169575712 | 129.657177786829 | Iran (Islamic Republic of) |
| 2018 | 157.0758269 | 0.680909620594576 | 37.694942403799 | 119.380884509501 | Iran (Islamic Republic of) |
| 2019 | 148.72851029943 | 0.686741661948379 | 37.2894914573956 | 111.439018842034 | Iran (Islamic Republic of) |
| 2020 | 103.31134785307 | 0.691918763371909 | 36.6468530048664 | 66.6644948482036 | Iran (Islamic Republic of) |
| 2021 | 72.31778397 | 0.697207397661899 | 35.7052111718156 | 36.6125728026444 | Iran (Islamic Republic of) |
| 1990 | 1931.40096661596 | 0.412044172813982 | 857.336497359022 | 1074.06446925694 | Iraq |
| 1991 | 1991.62990546479 | 0.415814115814778 | 798.366801774357 | 1193.26310369043 | Iraq |
| 1992 | 1967.91392205084 | 0.42035359441045 | 775.186952007472 | 1192.72697004337 | Iraq |
| 1993 | 1887.09423455329 | 0.42483493253075 | 684.764151850493 | 1202.3300827028 | Iraq |
| 1994 | 1799.31749724582 | 0.428751730675342 | 509.596751852036 | 1289.72074539378 | Iraq |
| 1995 | 1746.54018378962 | 0.432075545408255 | 439.181182024838 | 1307.35900176478 | Iraq |
| 1996 | 1699.2266762873 | 0.436818478749736 | 426.226538107364 | 1273.00013817994 | Iraq |
| 1997 | 1615.53104427169 | 0.44310402693832 | 425.463424958914 | 1190.06761931278 | Iraq |
| 1998 | 1511.68583549674 | 0.452384092352145 | 412.030757362747 | 1099.65507813399 | Iraq |
| 1999 | 1417.78469414788 | 0.462736441391784 | 380.551758125964 | 1037.23293602192 | Iraq |
| 2000 | 1301.62753812193 | 0.472376399238703 | 343.556485159753 | 958.071052962177 | Iraq |
| 2001 | 1200.14038398732 | 0.481458319619666 | 326.966467207716 | 873.173916779604 | Iraq |
| 2002 | 1129.10325292208 | 0.488664736761323 | 309.15765854293 | 819.94559437915 | Iraq |
| 2003 | 1102.88862905565 | 0.492174444727086 | 295.424508103404 | 807.464120952246 | Iraq |
| 2004 | 1026.90413488386 | 0.498933881288036 | 234.088734916506 | 792.815399967354 | Iraq |
| 2005 | 961.4683924 | 0.504719430828624 | 223.501181791826 | 737.967210629974 | Iraq |
| 2006 | 896.32031450283 | 0.510918049053858 | 212.953703551272 | 683.366610951558 | Iraq |
| 2007 | 859.21308419774 | 0.517698972378274 | 203.77749510396 | 655.43558909378 | Iraq |
| 2008 | 792.40909739533 | 0.525242907568095 | 190.114633058402 | 602.294464336928 | Iraq |
| 2009 | 714.48523395457 | 0.533200913619797 | 170.014976125867 | 544.470257828703 | Iraq |
| 2010 | 655.77559014929 | 0.542343278401218 | 155.011873281593 | 500.763716867697 | Iraq |
| 2011 | 585.74104920455 | 0.553048555118566 | 146.23696747648 | 439.50408172807 | Iraq |
| 2012 | 493.23613824167 | 0.564745467448645 | 112.811594093323 | 380.424544148347 | Iraq |
| 2013 | 424.68348360881 | 0.576968997890727 | 85.3553386501892 | 339.328144958621 | Iraq |
| 2014 | 396.70331534432 | 0.588317651574763 | 80.6574169746634 | 316.045898369657 | Iraq |
| 2015 | 355.50539895815 | 0.599494007105483 | 74.4352417791541 | 281.070157178996 | Iraq |
| 2016 | 344.8070858 | 0.611508645751767 | 68.8788259402585 | 275.928259854842 | Iraq |
| 2017 | 347.13803955382 | 0.622674531557115 | 64.7670531692126 | 282.370986384607 | Iraq |
| 2018 | 343.93390035009 | 0.633232108100178 | 53.1435485366911 | 290.790351813399 | Iraq |
| 2019 | 379.14106076418 | 0.64363531790137 | 51.8482913196426 | 327.292769444537 | Iraq |
| 2020 | 240.10159428137 | 0.653300379638783 | 45.5436859861957 | 194.557908295174 | Iraq |
| 2021 | 221.10853646918 | 0.662626230762701 | 41.5215857782273 | 179.586950690953 | Iraq |
| 1990 | 675.19069803419 | 0.53914746775111 | 156.819580340989 | 518.371117693201 | Jordan |
| 1991 | 654.09689424925 | 0.54281941081865 | 155.307799472534 | 498.789094776716 | Jordan |
| 1992 | 627.85435335711 | 0.547458846457137 | 149.538682156882 | 478.315671200228 | Jordan |
| 1993 | 601.17921963889 | 0.552182350404081 | 146.501659771562 | 454.677559867328 | Jordan |
| 1994 | 577.16536378385 | 0.557254334819626 | 142.070847886629 | 435.094515897221 | Jordan |
| 1995 | 553.61590364186 | 0.562571536302321 | 139.09632400761 | 414.51957963425 | Jordan |
| 1996 | 532.19055561151 | 0.567529655438914 | 112.164751917277 | 420.025803694233 | Jordan |
| 1997 | 508.7613325 | 0.572581508488338 | 85.7868876702898 | 422.97444484261 | Jordan |
| 1998 | 496.34058929213 | 0.577643570372855 | 79.4668713211958 | 416.873717970934 | Jordan |
| 1999 | 478.57674142647 | 0.582759198560708 | 78.377152595019 | 400.199588831451 | Jordan |
| 2000 | 460.07234428937 | 0.587909393807207 | 79.4896966422518 | 380.582647647118 | Jordan |
| 2001 | 438.53845857481 | 0.593182802809495 | 81.9395383631153 | 356.598920211695 | Jordan |
| 2002 | 412.32582002087 | 0.59900038468688 | 76.3654307991564 | 335.960389221714 | Jordan |
| 2003 | 381.30823746915 | 0.60494595024538 | 75.8177492526935 | 305.490488216457 | Jordan |
| 2004 | 357.08602692912 | 0.611934403689721 | 70.4577229345144 | 286.628303994606 | Jordan |
| 2005 | 336.16829878372 | 0.619909227769316 | 63.5340411005085 | 272.634257683212 | Jordan |
| 2006 | 310.77748270874 | 0.627803812947762 | 58.5011383638443 | 252.276344344896 | Jordan |
| 2007 | 283.87111820383 | 0.635670276218909 | 52.3450274118719 | 231.526090791958 | Jordan |
| 2008 | 264.07761712867 | 0.643892693421901 | 50.9906970111786 | 213.086920117491 | Jordan |
| 2009 | 247.36346271511 | 0.652196319480578 | 45.4766023246535 | 201.886860390456 | Jordan |
| 2010 | 230.12564972588 | 0.659949494519026 | 42.9199344522427 | 187.205715273637 | Jordan |
| 2011 | 208.97339720976 | 0.667396679645637 | 39.1774533457338 | 169.795943864026 | Jordan |
| 2012 | 181.54465990213 | 0.674568924665591 | 37.8874129668224 | 143.657246935308 | Jordan |
| 2013 | 152.31121986482 | 0.681119962167508 | 37.6814599071555 | 114.629759957665 | Jordan |
| 2014 | 127.89078114391 | 0.687134039943201 | 37.3504182113091 | 90.5403629326009 | Jordan |
| 2015 | 102.66279869357 | 0.692952031873164 | 36.8858058856614 | 65.7769928079086 | Jordan |
| 2016 | 99.27292109 | 0.698473302300541 | 35.9498298592263 | 63.3230912338437 | Jordan |
| 2017 | 94.37372845 | 0.703923826241846 | 35.6080103312617 | 58.7657181146783 | Jordan |
| 2018 | 87.24886575 | 0.709412879293601 | 33.7434778866056 | 53.5053878646844 | Jordan |
| 2019 | 84.88502865 | 0.714998945753142 | 30.8100414155548 | 54.0749872359752 | Jordan |
| 2020 | 83.98164201 | 0.720068239915947 | 29.0472743646358 | 54.9343676468742 | Jordan |
| 2021 | 76.74411925 | 0.725307226832443 | 26.3834596122749 | 50.3606596414651 | Jordan |
| 1990 | 206.74936462414 | 0.66451790400099 | 40.614373668398 | 166.134990955742 | Kuwait |
| 1991 | 125.310525 | 0.66880962078386 | 38.9546609549661 | 86.3558640690339 | Kuwait |
| 1992 | 132.7443428 | 0.670159110400931 | 38.6242905045356 | 94.1200522886644 | Kuwait |
| 1993 | 112.48017237052 | 0.670036252235978 | 38.2194996459236 | 74.2606727245964 | Kuwait |
| 1994 | 138.03837286839 | 0.671393212861768 | 38.6525874604884 | 99.3857854079016 | Kuwait |
| 1995 | 82.93291495 | 0.677215523332779 | 38.0386331011964 | 44.8942818524736 | Kuwait |
| 1996 | 59.40294154 | 0.686093303524213 | 37.2512603541471 | 22.1516811816729 | Kuwait |
| 1997 | 59.91203743 | 0.695789388591065 | 36.7698596232936 | 23.1421778032664 | Kuwait |
| 1998 | 73.31376556 | 0.704383735664098 | 34.6758063395727 | 38.6379592227673 | Kuwait |
| 1999 | 91.86987943 | 0.711841328373508 | 32.2041123961602 | 59.6657670290698 | Kuwait |
| 2000 | 103.19869287454 | 0.718357562401374 | 29.0078739414611 | 74.1908189330789 | Kuwait |
| 2001 | 82.15585697 | 0.722840631295124 | 27.4810066116254 | 54.6748503551646 | Kuwait |
| 2002 | 91.19330849 | 0.72681697562953 | 26.2586551895396 | 64.9346532996504 | Kuwait |
| 2003 | 96.09782631 | 0.731778684982861 | 23.8699205464514 | 72.2279057615686 | Kuwait |
| 2004 | 90.32828321 | 0.738153706974425 | 21.5313243912116 | 68.7969588221984 | Kuwait |
| 2005 | 90.63726227 | 0.746384913346372 | 20.1054798602992 | 70.5317824078508 | Kuwait |
| 2006 | 83.62849764 | 0.754928492574677 | 17.9167697861049 | 65.7117278585351 | Kuwait |
| 2007 | 88.19111769 | 0.762411483964357 | 14.362280812923 | 73.828836874407 | Kuwait |
| 2008 | 91.16799365 | 0.770002614917808 | 13.0971370306285 | 78.0708566165915 | Kuwait |
| 2009 | 81.76528884 | 0.777546551267578 | 12.2912037560729 | 69.4740850789571 | Kuwait |
| 2010 | 68.43003335 | 0.784873929223183 | 12.07046665 | 56.3595667 | Kuwait |
| 2011 | 68.77955756 | 0.791812065474587 | 10.9034026650804 | 57.8761548900096 | Kuwait |
| 2012 | 69.9074159 | 0.798356987724222 | 11.1763774208926 | 58.7310384828874 | Kuwait |
| 2013 | 66.57270922 | 0.80458383431311 | 9.53349791022214 | 57.0392113099779 | Kuwait |
| 2014 | 65.53774733 | 0.810743232598608 | 9.55618479236093 | 55.9815625329591 | Kuwait |
| 2015 | 61.4210288 | 0.816998412782804 | 8.18814516353732 | 53.2328836320827 | Kuwait |
| 2016 | 57.46045997 | 0.823026527054531 | 8.53964299962177 | 48.9208169727182 | Kuwait |
| 2017 | 45.55371421 | 0.828886986643824 | 7.43033205486136 | 38.1233821590186 | Kuwait |
| 2018 | 38.74814277 | 0.834371461321704 | 6.9192022741402 | 31.8289404958598 | Kuwait |
| 2019 | 34.92522897 | 0.839688306553232 | 6.16677141880462 | 28.7584575554054 | Kuwait |
| 2020 | 29.81506447 | 0.843459812023503 | 5.96325426163458 | 23.8518102085854 | Kuwait |
| 2021 | 27.89827577 | 0.846651054742869 | 6.07080857223824 | 21.8274672008018 | Kuwait |
| 1990 | 839.23408097832 | 0.536718968751988 | 158.519992990884 | 680.714087987436 | Lebanon |
| 1991 | 800.2440641 | 0.539197491838137 | 156.26337657391 | 643.98068749099 | Lebanon |
| 1992 | 759.09106774339 | 0.541793515767809 | 155.211634149143 | 603.879433594247 | Lebanon |
| 1993 | 867.21582451919 | 0.544581306172966 | 155.124644719153 | 712.091179800037 | Lebanon |
| 1994 | 679.78543462247 | 0.547684033143162 | 149.301661377084 | 530.483773245386 | Lebanon |
| 1995 | 645.04846117109 | 0.5514717204066 | 146.088638049192 | 498.959823121898 | Lebanon |
| 1996 | 608.1121757 | 0.556436158565937 | 144.365593530553 | 463.746582166447 | Lebanon |
| 1997 | 568.39159508559 | 0.562120817980966 | 139.427946771849 | 428.963648313741 | Lebanon |
| 1998 | 537.7680751 | 0.569059070131885 | 109.161882241207 | 428.606192854693 | Lebanon |
| 1999 | 516.5023086 | 0.576078527772757 | 85.4456091791058 | 431.056699422694 | Lebanon |
| 2000 | 490.88835741013 | 0.582698277151947 | 80.9182587162642 | 409.970098693866 | Lebanon |
| 2001 | 477.53894191279 | 0.589185502386114 | 80.147440105572 | 397.391501807218 | Lebanon |
| 2002 | 453.75899720391 | 0.595978787643548 | 77.4264271760721 | 376.332570027838 | Lebanon |
| 2003 | 432.46863807764 | 0.602967240409336 | 74.6339254976705 | 357.834712579969 | Lebanon |
| 2004 | 411.17726626548 | 0.610845760385912 | 67.342941926921 | 343.834324338559 | Lebanon |
| 2005 | 395.45334499968 | 0.619391355094372 | 62.9871649801772 | 332.466180019503 | Lebanon |
| 2006 | 369.2005224 | 0.627791217131142 | 60.02861298 | 309.17190946875 | Lebanon |
| 2007 | 351.00966951248 | 0.636938234123424 | 51.614437431643 | 299.395232080837 | Lebanon |
| 2008 | 332.27932583085 | 0.646667663001479 | 49.2523795400606 | 283.026946290789 | Lebanon |
| 2009 | 325.07499164131 | 0.657433186212217 | 45.578880543607 | 279.496111097703 | Lebanon |
| 2010 | 312.92897426733 | 0.668804524925046 | 39.2874244732536 | 273.641549794076 | Lebanon |
| 2011 | 306.66716397519 | 0.680920616624062 | 37.6026740469515 | 269.064489928239 | Lebanon |
| 2012 | 299.21462378033 | 0.693910983172664 | 36.6143132032241 | 262.600310577106 | Lebanon |
| 2013 | 279.38980887706 | 0.704811001480686 | 35.1266195005011 | 244.263189376559 | Lebanon |
| 2014 | 257.86545456086 | 0.71242387252594 | 32.1145676232387 | 225.750886937621 | Lebanon |
| 2015 | 251.9393612 | 0.7186972923939 | 28.8121354283374 | 223.127225797563 | Lebanon |
| 2016 | 252.95826813218 | 0.724583346730978 | 25.4443119896972 | 227.513956142483 | Lebanon |
| 2017 | 251.14039312444 | 0.729963293744747 | 23.9264060019035 | 227.213987122537 | Lebanon |
| 2018 | 244.48176580709 | 0.734746243401704 | 21.4616744905365 | 223.020091316554 | Lebanon |
| 2019 | 239.05127354336 | 0.738644009352405 | 20.4836756988091 | 218.567597844551 | Lebanon |
| 2020 | 220.70682656836 | 0.742009720142934 | 20.2738396899572 | 200.432986878403 | Lebanon |
| 2021 | 218.1691355 | 0.74474635096338 | 18.9284466727895 | 199.240688872511 | Lebanon |
| 1990 | 1955.41739368735 | 0.527998168231366 | 180.785361826449 | 1774.6320318609 | Libya |
| 1991 | 1637.60395799962 | 0.541432398663791 | 155.26016607011 | 1482.34379192951 | Libya |
| 1992 | 1344.86101213601 | 0.55446822725249 | 144.290873441593 | 1200.57013869442 | Libya |
| 1993 | 1151.49619945413 | 0.566798415093121 | 112.162838938495 | 1039.33336051563 | Libya |
| 1994 | 1009.78402682324 | 0.578651415542216 | 82.7150032146741 | 927.069023608566 | Libya |
| 1995 | 876.11909003832 | 0.589578634415899 | 78.9532342586316 | 797.165855779688 | Libya |
| 1996 | 705.6534385 | 0.60035004409905 | 76.3701341343159 | 629.283304408284 | Libya |
| 1997 | 638.8901818 | 0.610620315262943 | 69.2827515542532 | 569.607430221447 | Libya |
| 1998 | 572.65010846265 | 0.619973983675942 | 64.731453152279 | 507.918655310371 | Libya |
| 1999 | 518.69871382076 | 0.628600604307387 | 56.7599326545774 | 461.938781166183 | Libya |
| 2000 | 465.54541725105 | 0.63704793460596 | 51.7289490857963 | 413.816468165254 | Libya |
| 2001 | 431.85649476371 | 0.644685131438978 | 49.0537345509877 | 382.802760212722 | Libya |
| 2002 | 405.77518314459 | 0.651669147460857 | 47.1029870229692 | 358.672196121621 | Libya |
| 2003 | 381.86685840801 | 0.659345124852632 | 43.0207322948995 | 338.84612611311 | Libya |
| 2004 | 351.02165553141 | 0.666913926262631 | 39.0218840307581 | 311.999771500652 | Libya |
| 2005 | 328.64240920151 | 0.675322267622666 | 38.1327672197499 | 290.50964198176 | Libya |
| 2006 | 313.91702485054 | 0.683701999618589 | 37.2212572090704 | 276.69576764147 | Libya |
| 2007 | 301.83056573082 | 0.691855134182363 | 36.4400255523176 | 265.390540178502 | Libya |
| 2008 | 283.8615354 | 0.698873253326827 | 35.9895016544024 | 247.872033781198 | Libya |
| 2009 | 261.76435904003 | 0.704998470605237 | 34.639644480457 | 227.124714559573 | Libya |
| 2010 | 255.50974549194 | 0.711362496328616 | 31.9383752258772 | 223.571370266063 | Libya |
| 2011 | 223.70351035315 | 0.711466657224803 | 31.4466945618171 | 192.256815791333 | Libya |
| 2012 | 188.05575845537 | 0.716700693100353 | 31.8245607232809 | 156.231197732089 | Libya |
| 2013 | 137.4168754 | 0.717665249612534 | 30.5574948535364 | 106.859380512064 | Libya |
| 2014 | 116.93615749231 | 0.716042207086096 | 31.869803667479 | 85.066353824831 | Libya |
| 2015 | 109.80168315225 | 0.713651367022809 | 31.8114156729736 | 77.9902674792764 | Libya |
| 2016 | 120.04887387785 | 0.710760140384142 | 33.5653520478318 | 86.4835218300182 | Libya |
| 2017 | 121.44217016133 | 0.71066626361534 | 34.0454444029886 | 87.3967257583414 | Libya |
| 2018 | 109.37563028497 | 0.712720437649946 | 32.1254593839517 | 77.2501709010183 | Libya |
| 2019 | 105.282956 | 0.716157238515615 | 31.0272555736115 | 74.2557004114885 | Libya |
| 2020 | 114.41013572325 | 0.720270392863655 | 29.4854913512871 | 84.9246443719629 | Libya |
| 2021 | 121.98560914483 | 0.725771398888229 | 26.9029163615287 | 95.0826927833013 | Libya |
| 1990 | 8477.79377630921 | 0.358072869533996 | 1650.51391547698 | 6827.27986083223 | Morocco |
| 1991 | 7666.72072189082 | 0.3649846287647 | 1320.31568280497 | 6346.40503908585 | Morocco |
| 1992 | 7068.53294245979 | 0.371187194023026 | 1214.5577521986 | 5853.97519026119 | Morocco |
| 1993 | 6514.70518249731 | 0.37677897731043 | 1172.84587004384 | 5341.85931245347 | Morocco |
| 1994 | 6019.68426310482 | 0.382849450670084 | 1094.72657254119 | 4924.95769056363 | Morocco |
| 1995 | 5580.38271607957 | 0.388164109911114 | 1033.98281320468 | 4546.39990287489 | Morocco |
| 1996 | 5160.41466304579 | 0.394150096117346 | 977.508424929051 | 4182.90623811674 | Morocco |
| 1997 | 4811.86222195552 | 0.399504009010139 | 939.874023643593 | 3871.98819831193 | Morocco |
| 1998 | 4480.43615461204 | 0.405038791986878 | 886.555228025017 | 3593.88092658702 | Morocco |
| 1999 | 4179.92283522005 | 0.410303190621115 | 871.265465328026 | 3308.65736989202 | Morocco |
| 2000 | 3910.05290994996 | 0.415344026415974 | 789.941760790721 | 3120.11114915924 | Morocco |
| 2001 | 3665.18768228141 | 0.420595133422507 | 774.008781395871 | 2891.17890088554 | Morocco |
| 2002 | 3440.47618764467 | 0.425775750183888 | 694.865052547196 | 2745.61113509747 | Morocco |
| 2003 | 3156.2417942536 | 0.431217810181359 | 429.290119517445 | 2726.95167473615 | Morocco |
| 2004 | 2934.0721201129 | 0.436822351876544 | 419.158337455509 | 2514.91378265739 | Morocco |
| 2005 | 2722.42239427181 | 0.442531339370103 | 420.124468679029 | 2302.29792559278 | Morocco |
| 2006 | 2486.91305272805 | 0.448676160448964 | 412.389741244719 | 2074.52331148333 | Morocco |
| 2007 | 2222.48301921492 | 0.455036324601373 | 401.042208503649 | 1821.44081071127 | Morocco |
| 2008 | 1944.66218439628 | 0.461803552255995 | 380.781072840997 | 1563.88111155528 | Morocco |
| 2009 | 1718.93801953003 | 0.468929893059803 | 343.170797441901 | 1375.76722208813 | Morocco |
| 2010 | 1429.4976225067 | 0.476269799414457 | 338.038606076624 | 1091.45901643008 | Morocco |
| 2011 | 1149.45282120053 | 0.483922151996701 | 324.126432157347 | 825.326389043183 | Morocco |
| 2012 | 908.89599576101 | 0.491603391761499 | 299.582682277052 | 609.313313483958 | Morocco |
| 2013 | 717.47697171288 | 0.499528606645883 | 236.154885454579 | 481.3220863 | Morocco |
| 2014 | 618.98020042068 | 0.507443265160336 | 219.629852584716 | 399.350347835964 | Morocco |
| 2015 | 528.02693806556 | 0.515582892643316 | 203.801645430083 | 324.225292635477 | Morocco |
| 2016 | 469.65871059031 | 0.523518592215067 | 190.724000978204 | 278.934709612106 | Morocco |
| 2017 | 420.06654877237 | 0.531610861944961 | 169.890875507429 | 250.175673264941 | Morocco |
| 2018 | 380.36306807341 | 0.539729670716936 | 156.46070271962 | 223.90236535379 | Morocco |
| 2019 | 341.42317751451 | 0.547837444469332 | 149.599909149588 | 191.823268364922 | Morocco |
| 2020 | 344.02729795003 | 0.555246068582659 | 143.906668516778 | 200.120629433252 | Morocco |
| 2021 | 315.52271565721 | 0.562698300927195 | 139.304368063818 | 176.218347593392 | Morocco |
| 1990 | 1229.03212748537 | 0.401792210145272 | 930.301696637859 | 298.730430847511 | Palestine |
| 1991 | 1099.49557171097 | 0.405727150469488 | 883.563335389808 | 215.932236321162 | Palestine |
| 1992 | 969.44522332161 | 0.411146868747336 | 857.777417044165 | 111.667806277445 | Palestine |
| 1993 | 845.27071505471 | 0.416779648694491 | 798.210488665217 | 47.0602263894929 | Palestine |
| 1994 | 728.55336466945 | 0.423136672782837 | 681.333131289302 | 47.2202333801481 | Palestine |
| 1995 | 633.33234801128 | 0.42930702715431 | 501.080375181107 | 132.251972830173 | Palestine |
| 1996 | 554.46293064427 | 0.434731371082746 | 421.073769203409 | 133.389161440861 | Palestine |
| 1997 | 488.56548333273 | 0.440901776082897 | 422.363229420839 | 66.2022539118911 | Palestine |
| 1998 | 433.19283804388 | 0.447782415404353 | 404.703907855981 | 28.4889301878986 | Palestine |
| 1999 | 390.92288068395 | 0.454909692070064 | 390.92288068395 | 0 | Palestine |
| 2000 | 363.0792423 | 0.460681496412606 | 363.0792423 | 0 | Palestine |
| 2001 | 344.80961649537 | 0.465299102197142 | 344.80961649537 | 0 | Palestine |
| 2002 | 335.10890630579 | 0.468600256734998 | 335.10890630579 | 0 | Palestine |
| 2003 | 324.87792258974 | 0.472881732260502 | 324.87792258974 | 0 | Palestine |
| 2004 | 314.20479988302 | 0.478527322159265 | 314.20479988302 | 0 | Palestine |
| 2005 | 308.61399919056 | 0.485320585803801 | 308.61399919056 | 0 | Palestine |
| 2006 | 294.48700735017 | 0.491890406552455 | 290.660211944352 | 3.82679540581825 | Palestine |
| 2007 | 283.66883908195 | 0.498978425791333 | 239.396266225409 | 44.2725728565412 | Palestine |
| 2008 | 261.12522240544 | 0.506849478804306 | 219.537405636513 | 41.5878167689268 | Palestine |
| 2009 | 235.07354085898 | 0.515815835660256 | 203.887693920285 | 31.185846938695 | Palestine |
| 2010 | 211.55190461465 | 0.525711905369362 | 190.119766424776 | 21.4321381898738 | Palestine |
| 2011 | 196.02550579498 | 0.536777629566224 | 157.826450314272 | 38.1990554807077 | Palestine |
| 2012 | 180.07284396901 | 0.548295425023352 | 150.415516705524 | 29.6573272634857 | Palestine |
| 2013 | 166.13339932821 | 0.55916348091297 | 142.54530804649 | 23.5880912817202 | Palestine |
| 2014 | 154.21484674061 | 0.569239881995993 | 104.339604490313 | 49.8752422502968 | Palestine |
| 2015 | 146.34404594334 | 0.579204260933414 | 83.8680741155844 | 62.4759718277556 | Palestine |
| 2016 | 111.52789096649 | 0.589492769984704 | 81.2674794182383 | 30.2604115482517 | Palestine |
| 2017 | 85.92525323 | 0.599157186635533 | 74.7227984048027 | 11.2024548222873 | Palestine |
| 2018 | 62.2412108 | 0.608124860877158 | 62.2412108 | 0 | Palestine |
| 2019 | 50.76728328 | 0.616466838514416 | 50.76728328 | 0 | Palestine |
| 2020 | 54.99120528 | 0.623810015023286 | 53.5004092778965 | 1.49079599964352 | Palestine |
| 2021 | 46.80486677 | 0.631011665054344 | 46.80486677 | 0 | Palestine |
| 1990 | 889.31964094606 | 0.429270948783208 | 506.92124907205 | 382.39839187401 | Oman |
| 1991 | 785.67227113916 | 0.442268122012418 | 422.717829819104 | 362.954441320056 | Oman |
| 1992 | 672.28406910318 | 0.457737972074553 | 397.442777692764 | 274.841291410416 | Oman |
| 1993 | 589.74385268747 | 0.474999032775331 | 337.954881826214 | 251.788970861256 | Oman |
| 1994 | 527.88992014807 | 0.493513107048941 | 292.704868060627 | 235.185052087443 | Oman |
| 1995 | 462.95717655336 | 0.513194359370639 | 206.750014299608 | 256.207162253752 | Oman |
| 1996 | 407.43902339613 | 0.533246393920158 | 172.708093737048 | 234.730929659082 | Oman |
| 1997 | 361.74451775391 | 0.554315537130654 | 143.911823184361 | 217.832694569549 | Oman |
| 1998 | 326.61234263238 | 0.574474902223599 | 88.5196775263832 | 238.092665105997 | Oman |
| 1999 | 297.08246244531 | 0.592398470428405 | 78.9163799479377 | 218.166082497372 | Oman |
| 2000 | 269.49297111341 | 0.608421540646462 | 69.8458139645968 | 199.647157148813 | Oman |
| 2001 | 246.11452132285 | 0.622160627189522 | 62.7669122765938 | 183.347609046256 | Oman |
| 2002 | 227.16418279948 | 0.63516212584598 | 53.3740283177669 | 173.790154481713 | Oman |
| 2003 | 210.91218789005 | 0.647249092690596 | 50.0694230661937 | 160.842764823856 | Oman |
| 2004 | 197.04184635825 | 0.65762611814533 | 44.8420621534357 | 152.199784204814 | Oman |
| 2005 | 184.16246298799 | 0.666401230917978 | 39.4730815483478 | 144.689381439642 | Oman |
| 2006 | 182.09222056997 | 0.675353885063336 | 38.1981864663854 | 143.894034103585 | Oman |
| 2007 | 184.99419301403 | 0.685055582772953 | 37.306977843254 | 147.687215170776 | Oman |
| 2008 | 192.72870180194 | 0.694952262906783 | 36.76107102 | 155.96763077959 | Oman |
| 2009 | 200.62436374596 | 0.703098570806704 | 34.7267549108545 | 165.897608835106 | Oman |
| 2010 | 200.02234078051 | 0.710688694921138 | 32.8957663160616 | 167.126574464448 | Oman |
| 2011 | 201.94363224069 | 0.718264528020208 | 28.5337020433936 | 173.409930197296 | Oman |
| 2012 | 202.0848043 | 0.726450074292121 | 25.7894364850882 | 176.295367808112 | Oman |
| 2013 | 199.9640501 | 0.733432490235765 | 22.2644352604363 | 177.699614809264 | Oman |
| 2014 | 196.72699997685 | 0.738743771923443 | 19.807375205834 | 176.919624771016 | Oman |
| 2015 | 198.28572021122 | 0.743597182373955 | 20.6598076067786 | 177.625912604441 | Oman |
| 2016 | 202.36485402997 | 0.74879719338257 | 19.5306655882983 | 182.834188441672 | Oman |
| 2017 | 200.77431188845 | 0.753786057672964 | 18.6533472679692 | 182.120964620481 | Oman |
| 2018 | 193.46830218351 | 0.759179513636833 | 15.66338639 | 177.80491579587 | Oman |
| 2019 | 185.61744985919 | 0.764527863267196 | 14.7895487971436 | 170.827901062046 | Oman |
| 2020 | 170.84099640073 | 0.768854215658189 | 12.3916965071332 | 158.449299893597 | Oman |
| 2021 | 159.09648620739 | 0.773391601871737 | 14.1610694890514 | 144.935416718339 | Oman |
| 1990 | 204.49181931643 | 0.651208375960545 | 48.4824113587362 | 156.009407957694 | Qatar |
| 1991 | 193.37116050664 | 0.655338766647052 | 45.7582284022352 | 147.612932104405 | Qatar |
| 1992 | 182.33163729884 | 0.660673763366912 | 43.2825235134453 | 139.049113785395 | Qatar |
| 1993 | 172.54581501956 | 0.666194398536698 | 39.1937294241225 | 133.352085595438 | Qatar |
| 1994 | 164.57178474562 | 0.672025383885115 | 38.2193526989416 | 126.352432046678 | Qatar |
| 1995 | 157.03446749381 | 0.678320339337967 | 37.5861641933996 | 119.44830330041 | Qatar |
| 1996 | 151.34207305548 | 0.68483591897584 | 37.362282052543 | 113.979791002937 | Qatar |
| 1997 | 145.82965552887 | 0.693109653614432 | 36.5668556469184 | 109.262799881952 | Qatar |
| 1998 | 141.28620973263 | 0.701409063669436 | 35.9201034687066 | 105.366106263923 | Qatar |
| 1999 | 137.38853623624 | 0.708880981468347 | 34.5933138884891 | 102.795222347751 | Qatar |
| 2000 | 133.04500548052 | 0.714831609105106 | 32.6853020894711 | 100.359703391049 | Qatar |
| 2001 | 131.30301275411 | 0.720831064006284 | 28.3967515257928 | 102.906261228317 | Qatar |
| 2002 | 127.39497879318 | 0.727112174151443 | 25.690531879891 | 101.704446913289 | Qatar |
| 2003 | 123.34301662111 | 0.733536599318407 | 22.0140555421838 | 101.328961078926 | Qatar |
| 2004 | 120.02008958696 | 0.740223208352271 | 21.097339569854 | 98.922750017106 | Qatar |
| 2005 | 118.07788120312 | 0.747189358624771 | 19.4976788303405 | 98.5802023727795 | Qatar |
| 2006 | 108.44807077619 | 0.754123028435068 | 17.5551956042468 | 90.8928751719432 | Qatar |
| 2007 | 90.84936648 | 0.760960134224895 | 16.4064374024607 | 74.4429290797393 | Qatar |
| 2008 | 70.66549134 | 0.767659233393271 | 14.9827747401383 | 55.6827165988717 | Qatar |
| 2009 | 46.95860332 | 0.77427684914439 | 12.1040108024045 | 34.8545925170955 | Qatar |
| 2010 | 37.2297662 | 0.780807073920169 | 12.6246458264727 | 24.6051203776073 | Qatar |
| 2011 | 32.91183841 | 0.7872124503355 | 11.7181484260116 | 21.1936899792584 | Qatar |
| 2012 | 29.61117492 | 0.793494900808854 | 11.8234024307909 | 17.7877724902191 | Qatar |
| 2013 | 26.44991138 | 0.799611554876573 | 10.7556932013144 | 15.6942181813356 | Qatar |
| 2014 | 24.15063071 | 0.805725710213966 | 9.79273409208824 | 14.3578966165518 | Qatar |
| 2015 | 22.25743819 | 0.811872851894902 | 8.42137437673684 | 13.8360638153732 | Qatar |
| 2016 | 20.78433896 | 0.817979840558073 | 9.14555158846935 | 11.6387873680206 | Qatar |
| 2017 | 19.68544066 | 0.824016868434921 | 8.08223730944263 | 11.6032033510974 | Qatar |
| 2018 | 18.71818495 | 0.829962126581261 | 8.21328119658647 | 10.5049037490435 | Qatar |
| 2019 | 17.97150346 | 0.835805284348454 | 6.85982710775258 | 11.1116763565974 | Qatar |
| 2020 | 23.79658103 | 0.841467395353032 | 6.32829665539313 | 17.4682843763669 | Qatar |
| 2021 | 14.65893427 | 0.846860584038945 | 5.7353204076555 | 8.9236138608645 | Qatar |
| 1990 | 1346.32132965437 | 0.538954515496368 | 156.349810959723 | 1189.97151869465 | Saudi Arabia |
| 1991 | 1211.66238418565 | 0.549907716626613 | 145.977461599454 | 1065.6849225862 | Saudi Arabia |
| 1992 | 1089.355465 | 0.561144266304623 | 139.142583049046 | 950.212881573954 | Saudi Arabia |
| 1993 | 977.53054420214 | 0.572186913451422 | 87.1870007186023 | 890.343543483538 | Saudi Arabia |
| 1994 | 880.81073295975 | 0.582878871179675 | 85.6342773514147 | 795.176455608335 | Saudi Arabia |
| 1995 | 792.43038341192 | 0.593251423065509 | 79.3376946413448 | 713.092688770575 | Saudi Arabia |
| 1996 | 704.71175086429 | 0.603648418303976 | 74.7840537416843 | 629.927697122606 | Saudi Arabia |
| 1997 | 634.32127143895 | 0.613749116453829 | 69.6010758855492 | 564.720195553401 | Saudi Arabia |
| 1998 | 575.02034228729 | 0.623518214245881 | 63.6023433678005 | 511.41799891949 | Saudi Arabia |
| 1999 | 526.15881612038 | 0.632796447356413 | 54.3336493950262 | 471.825166725354 | Saudi Arabia |
| 2000 | 481.45856534562 | 0.642311774343419 | 51.6349366718601 | 429.82362867376 | Saudi Arabia |
| 2001 | 442.85738185745 | 0.651502091755966 | 48.0060925032097 | 394.85128935424 | Saudi Arabia |
| 2002 | 405.95600231497 | 0.660356855701209 | 43.1199112396314 | 362.836091075339 | Saudi Arabia |
| 2003 | 370.26837825339 | 0.669839576958755 | 38.7269071062729 | 331.541471147117 | Saudi Arabia |
| 2004 | 335.31061459657 | 0.679917856369452 | 37.7288818377781 | 297.581732758792 | Saudi Arabia |
| 2005 | 304.45907315459 | 0.690520851691753 | 36.5399656482821 | 267.919107506308 | Saudi Arabia |
| 2006 | 276.03366779335 | 0.70115261257815 | 35.8577862262175 | 240.175881567132 | Saudi Arabia |
| 2007 | 251.83394496158 | 0.711442141049593 | 31.6203563097435 | 220.213588651836 | Saudi Arabia |
| 2008 | 229.31789177602 | 0.721960204973632 | 29.2148025135611 | 200.103089262459 | Saudi Arabia |
| 2009 | 210.20429118175 | 0.731158488162749 | 24.8810991765714 | 185.323192005179 | Saudi Arabia |
| 2010 | 193.26449948542 | 0.740300669205333 | 20.559170833474 | 172.705328651946 | Saudi Arabia |
| 2011 | 166.83143203386 | 0.749959451001828 | 19.4674568981861 | 147.363975135674 | Saudi Arabia |
| 2012 | 128.89879064412 | 0.759054935383679 | 15.8967762860593 | 113.002014358061 | Saudi Arabia |
| 2013 | 81.70291065 | 0.767387864281482 | 14.1911026329666 | 67.5118080189634 | Saudi Arabia |
| 2014 | 56.75334898 | 0.775178132630626 | 12.7157893575728 | 44.0375596213272 | Saudi Arabia |
| 2015 | 44.56715468 | 0.782300801582157 | 12.5500271987982 | 32.0171274801318 | Saudi Arabia |
| 2016 | 40.67082194 | 0.788867882259991 | 11.3859662057174 | 29.2848557376326 | Saudi Arabia |
| 2017 | 37.31568474 | 0.794886287697922 | 10.4219107775457 | 26.8937739590443 | Saudi Arabia |
| 2018 | 34.77537675 | 0.800656984976864 | 11.6783134562391 | 23.0970632952409 | Saudi Arabia |
| 2019 | 33.186341 | 0.806087840988648 | 10.277985029195 | 22.908355974835 | Saudi Arabia |
| 2020 | 40.14481689 | 0.810541860714478 | 8.93416583104973 | 31.2106510627103 | Saudi Arabia |
| 2021 | 42.70284684 | 0.815143493214829 | 8.838404082 | 33.864442762498 | Saudi Arabia |
| 1990 | 1121.87974617728 | 0.430492642865419 | 419.77978181559 | 702.09996436169 | Syrian Arab Republic |
| 1991 | 1025.65536353427 | 0.437907493360423 | 416.509777125411 | 609.145586408859 | Syrian Arab Republic |
| 1992 | 925.54804078494 | 0.445904235849208 | 410.129998263132 | 515.418042521808 | Syrian Arab Republic |
| 1993 | 825.06397459227 | 0.45414785985736 | 406.588052175122 | 418.475922417148 | Syrian Arab Republic |
| 1994 | 741.10629235161 | 0.462437297890694 | 378.817522235319 | 362.288770116291 | Syrian Arab Republic |
| 1995 | 659.43148651008 | 0.470692042726613 | 340.366744211298 | 319.064742298782 | Syrian Arab Republic |
| 1996 | 563.9519692 | 0.478743085009033 | 326.628348223893 | 237.323620940207 | Syrian Arab Republic |
| 1997 | 481.4419993 | 0.48623235022627 | 316.842275370026 | 164.599723950974 | Syrian Arab Republic |
| 1998 | 405.79490113364 | 0.493707991654398 | 292.10619703178 | 113.68870410186 | Syrian Arab Republic |
| 1999 | 367.25710229729 | 0.500474869218632 | 228.025752066592 | 139.231350230698 | Syrian Arab Republic |
| 2000 | 312.58314959272 | 0.507143685736932 | 218.725983778092 | 93.8571658146277 | Syrian Arab Republic |
| 2001 | 276.23665634366 | 0.513850111268667 | 206.948505630564 | 69.2881507130961 | Syrian Arab Republic |
| 2002 | 250.98419756609 | 0.521309744436802 | 199.480966443292 | 51.5032311227983 | Syrian Arab Republic |
| 2003 | 218.49466219903 | 0.528935756199872 | 182.370690754019 | 36.1239714450113 | Syrian Arab Republic |
| 2004 | 204.20841667754 | 0.538084469307822 | 157.619845504761 | 46.5885711727788 | Syrian Arab Republic |
| 2005 | 204.86718972363 | 0.549750448700287 | 145.79723239294 | 59.0699573306895 | Syrian Arab Republic |
| 2006 | 205.225174 | 0.560484761476108 | 139.856418056373 | 65.3687559680269 | Syrian Arab Republic |
| 2007 | 189.33288368912 | 0.570028120377629 | 90.978174713132 | 98.354708975988 | Syrian Arab Republic |
| 2008 | 189.81576592843 | 0.578990492745018 | 81.4778043980615 | 108.337961530369 | Syrian Arab Republic |
| 2009 | 178.78192043525 | 0.587789117723187 | 81.2106958096974 | 97.5712246255526 | Syrian Arab Republic |
| 2010 | 169.48251151516 | 0.59596776440437 | 75.8469487488893 | 93.6355627662707 | Syrian Arab Republic |
| 2011 | 164.21314503179 | 0.60152445568341 | 75.783176546767 | 88.429968485023 | Syrian Arab Republic |
| 2012 | 152.69021673497 | 0.60287260117891 | 74.8294854942547 | 77.8607312407153 | Syrian Arab Republic |
| 2013 | 146.56434986561 | 0.6024101339716 | 76.392835762852 | 70.171514102758 | Syrian Arab Republic |
| 2014 | 149.44874295741 | 0.602235759940116 | 75.0814747505911 | 74.3672682068189 | Syrian Arab Republic |
| 2015 | 148.25618384019 | 0.602282913873841 | 75.7553077863843 | 72.5008760538057 | Syrian Arab Republic |
| 2016 | 141.14488543473 | 0.602911181092802 | 74.2707620769033 | 66.8741233578267 | Syrian Arab Republic |
| 2017 | 130.59812606624 | 0.604896366029073 | 76.0458306924372 | 54.5522953738028 | Syrian Arab Republic |
| 2018 | 120.01012684625 | 0.608507163893069 | 68.1434739321796 | 51.8666529140704 | Syrian Arab Republic |
| 2019 | 109.3240918 | 0.613336835963498 | 69.6000952304869 | 39.7239965969131 | Syrian Arab Republic |
| 2020 | 96.04739695 | 0.61772759307296 | 63.6716707066321 | 32.3757262437179 | Syrian Arab Republic |
| 2021 | 88.96047776 | 0.62300407463119 | 64.5223079877477 | 24.4381697676123 | Syrian Arab Republic |
| 1990 | 713.74025965114 | 0.471138521303638 | 342.793886016993 | 370.946373634147 | Tunisia |
| 1991 | 640.03479462366 | 0.479543056615384 | 324.513529333048 | 315.521265290612 | Tunisia |
| 1992 | 574.55824329956 | 0.488285281381984 | 308.937034346779 | 265.621208952781 | Tunisia |
| 1993 | 515.55705933681 | 0.496852038879262 | 249.587911738436 | 265.969147598374 | Tunisia |
| 1994 | 459.94898370049 | 0.505564882262378 | 223.241937945262 | 236.707045755228 | Tunisia |
| 1995 | 410.99168351448 | 0.514196940056936 | 206.951229439938 | 204.040454074542 | Tunisia |
| 1996 | 365.96591823007 | 0.523294273698708 | 189.528607081374 | 176.437311148696 | Tunisia |
| 1997 | 330.6705691 | 0.532064044257322 | 170.665986114009 | 160.004582939291 | Tunisia |
| 1998 | 301.34171814493 | 0.540498160807659 | 155.989259714952 | 145.352458429978 | Tunisia |
| 1999 | 283.35558693143 | 0.548724563004994 | 149.216326965264 | 134.139259966166 | Tunisia |
| 2000 | 268.20972290351 | 0.556672649979971 | 141.193822014274 | 127.015900889236 | Tunisia |
| 2001 | 254.38104098845 | 0.564421010157184 | 137.136072774651 | 117.244968213799 | Tunisia |
| 2002 | 244.38374119179 | 0.571719533904953 | 83.3958660548616 | 160.987875136928 | Tunisia |
| 2003 | 233.55395679832 | 0.578882668513321 | 79.4510629022746 | 154.102893896045 | Tunisia |
| 2004 | 219.38608254735 | 0.586032594266679 | 83.4391619467224 | 135.946920600628 | Tunisia |
| 2005 | 209.24092598328 | 0.592937759304517 | 78.6104237745319 | 130.630502208748 | Tunisia |
| 2006 | 200.44184166492 | 0.59975851290592 | 74.2714347937867 | 126.170406871133 | Tunisia |
| 2007 | 191.91527662948 | 0.606696784272835 | 74.5078059385538 | 117.407470690926 | Tunisia |
| 2008 | 183.31684313721 | 0.613529322048559 | 71.3273860170484 | 111.989457120162 | Tunisia |
| 2009 | 175.97098441806 | 0.620106833480493 | 62.951528897481 | 113.019455520579 | Tunisia |
| 2010 | 169.34787555636 | 0.626432773734803 | 59.1264395768677 | 110.221435979492 | Tunisia |
| 2011 | 158.7064444 | 0.632019840139461 | 52.2589677124917 | 106.447476696808 | Tunisia |
| 2012 | 147.55789600368 | 0.637578344563551 | 50.6755105606561 | 96.8823854430239 | Tunisia |
| 2013 | 137.75482027788 | 0.643008511949972 | 51.7651825915257 | 85.9896376863543 | Tunisia |
| 2014 | 128.74954602704 | 0.648297395047989 | 45.8907234572392 | 82.8588225698009 | Tunisia |
| 2015 | 121.28107475729 | 0.653413537772423 | 45.346722841482 | 75.934351915808 | Tunisia |
| 2016 | 115.80069283614 | 0.658353516621193 | 45.4717206355373 | 70.3289722006027 | Tunisia |
| 2017 | 106.89143639709 | 0.663222845186053 | 40.5761330763346 | 66.3153033207554 | Tunisia |
| 2018 | 99.34788903 | 0.668218997464981 | 39.543864178342 | 59.804024852888 | Tunisia |
| 2019 | 94.09068554 | 0.673170551594905 | 37.9248974386118 | 56.1657881024982 | Tunisia |
| 2020 | 87.64850725 | 0.677615147910612 | 37.3919687346291 | 50.2565385157209 | Tunisia |
| 2021 | 83.72620364 | 0.682432215876962 | 37.4366220273035 | 46.2895816081565 | Tunisia |
| 1990 | 2065.23230148217 | 0.461606983777208 | 379.65170264396 | 1685.58059883821 | Turkey |
| 1991 | 1872.24734463612 | 0.469244066030772 | 346.638170456437 | 1525.60917417968 | Turkey |
| 1992 | 1705.69853471908 | 0.476814569059173 | 335.546403464982 | 1370.1521312541 | Turkey |
| 1993 | 1559.4918874903 | 0.484656970229106 | 323.404323159506 | 1236.08756433079 | Turkey |
| 1994 | 1451.54203774302 | 0.491619950496974 | 300.279952760956 | 1151.26208498206 | Turkey |
| 1995 | 1342.92115783508 | 0.498824840241401 | 238.166670180364 | 1104.75448765472 | Turkey |
| 1996 | 1233.29252384526 | 0.506238546050969 | 220.153730315077 | 1013.13879353018 | Turkey |
| 1997 | 1144.5096237395 | 0.513933355794628 | 207.089836524615 | 937.419787214885 | Turkey |
| 1998 | 1053.10241778424 | 0.521509897761014 | 197.775006126675 | 855.327411657566 | Turkey |
| 1999 | 949.85188303474 | 0.528512415528019 | 180.606594843762 | 769.245288190978 | Turkey |
| 2000 | 859.2852072 | 0.535866331151872 | 163.159698029556 | 696.125509217644 | Turkey |
| 2001 | 786.70637224233 | 0.542359130151394 | 155.293358528889 | 631.413013713441 | Turkey |
| 2002 | 705.69846471062 | 0.549023644141264 | 149.652165432121 | 556.046299278499 | Turkey |
| 2003 | 605.93737711221 | 0.556102526706668 | 143.653035643466 | 462.284341468744 | Turkey |
| 2004 | 530.11055676884 | 0.564023999784751 | 137.338981997299 | 392.771574771541 | Turkey |
| 2005 | 463.66068149582 | 0.572495886408665 | 81.6135587789199 | 382.0471227 | Turkey |
| 2006 | 414.47103776599 | 0.581383178782649 | 82.3145835576695 | 332.15645420832 | Turkey |
| 2007 | 373.44366367736 | 0.590442387296341 | 79.0441900387769 | 294.399473638583 | Turkey |
| 2008 | 340.43742888479 | 0.599212061486959 | 74.8533396553157 | 265.584089229474 | Turkey |
| 2009 | 305.36832576071 | 0.607104617902237 | 75.7232941507023 | 229.645031610008 | Turkey |
| 2010 | 277.6140385 | 0.615597227656952 | 69.4975307380282 | 208.116507795672 | Turkey |
| 2011 | 241.39370976025 | 0.624787210625257 | 59.7788778887133 | 181.614831871537 | Turkey |
| 2012 | 220.75601683404 | 0.634097091952487 | 54.1191986251797 | 166.63681820886 | Turkey |
| 2013 | 205.02221972072 | 0.643723822649817 | 50.8232354220895 | 154.19898429863 | Turkey |
| 2014 | 191.32347255003 | 0.653388834851172 | 45.4711044165641 | 145.852368133466 | Turkey |
| 2015 | 175.87241181914 | 0.662971738649364 | 40.549866291653 | 135.322545527487 | Turkey |
| 2016 | 164.13710296619 | 0.671948147539094 | 38.5379296093921 | 125.599173356798 | Turkey |
| 2017 | 154.70591270245 | 0.681096272126073 | 37.348303306519 | 117.357609395931 | Turkey |
| 2018 | 148.04197191802 | 0.689895866860746 | 37.1332353152133 | 110.908736602807 | Turkey |
| 2019 | 141.80042250191 | 0.69826377357682 | 35.4359202626807 | 106.364502239229 | Turkey |
| 2020 | 129.363415 | 0.70579974360086 | 34.3430056177908 | 95.0204094008092 | Turkey |
| 2021 | 125.336235 | 0.712692673098478 | 31.6584631320313 | 93.6777718821687 | Turkey |
| 1990 | 343.82096844603 | 0.644412271353864 | 50.637277704408 | 293.183690741622 | United Arab Emirates |
| 1991 | 318.18735717166 | 0.660739899044445 | 43.1171202405513 | 275.070236931109 | United Arab Emirates |
| 1992 | 302.59420400075 | 0.675926211137966 | 37.9279079380708 | 264.666296062679 | United Arab Emirates |
| 1993 | 263.01422952309 | 0.689463800557258 | 37.1683433832571 | 225.845886139833 | United Arab Emirates |
| 1994 | 237.84208102701 | 0.701656792310882 | 35.9097229122449 | 201.932358114765 | United Arab Emirates |
| 1995 | 210.31611354665 | 0.713138287743939 | 32.3192092072283 | 177.996904339422 | United Arab Emirates |
| 1996 | 186.68433932679 | 0.724181976276083 | 26.7995695293963 | 159.884769797394 | United Arab Emirates |
| 1997 | 181.3492481 | 0.734723175496013 | 22.5215647908573 | 158.827683264643 | United Arab Emirates |
| 1998 | 186.04561083066 | 0.744476623052101 | 20.5699301629188 | 165.475680667741 | United Arab Emirates |
| 1999 | 199.54617702245 | 0.753253916450152 | 17.6387605971348 | 181.907416425315 | United Arab Emirates |
| 2000 | 202.4793262 | 0.760969094123031 | 16.0907670214275 | 186.388559205672 | United Arab Emirates |
| 2001 | 206.46041599664 | 0.767858792936623 | 14.104540728734 | 192.355875267906 | United Arab Emirates |
| 2002 | 211.13934237208 | 0.774346424129537 | 14.8801061830377 | 196.259236189042 | United Arab Emirates |
| 2003 | 209.89807542613 | 0.781143991614649 | 11.4354073528035 | 198.462668073327 | United Arab Emirates |
| 2004 | 196.91556632227 | 0.788525951082133 | 11.0652521330976 | 185.850314189172 | United Arab Emirates |
| 2005 | 189.90499698668 | 0.796428085714925 | 10.3792409817472 | 179.525756004933 | United Arab Emirates |
| 2006 | 180.08695630089 | 0.805537022535992 | 10.0057293772017 | 170.081226923688 | United Arab Emirates |
| 2007 | 192.20573310846 | 0.814870878080389 | 8.76956998438693 | 183.436163124073 | United Arab Emirates |
| 2008 | 205.75714618136 | 0.822793435350208 | 8.23964558139561 | 197.517500599964 | United Arab Emirates |
| 2009 | 223.15864000411 | 0.82830416539166 | 7.78558108406017 | 215.37305892005 | United Arab Emirates |
| 2010 | 230.8222965 | 0.831284254488885 | 6.85535452555407 | 223.966942019746 | United Arab Emirates |
| 2011 | 211.03816262459 | 0.832912803937778 | 7.57815035590328 | 203.460012268687 | United Arab Emirates |
| 2012 | 170.44187710875 | 0.833586109601576 | 7.19382194017244 | 163.248055168578 | United Arab Emirates |
| 2013 | 125.09767853527 | 0.833803818475535 | 7.3701592124288 | 117.727519322841 | United Arab Emirates |
| 2014 | 76.15343663 | 0.833858726882329 | 6.8769010485425 | 69.2765355840475 | United Arab Emirates |
| 2015 | 58.99240449 | 0.834175498027292 | 7.04806610468137 | 51.9443383843086 | United Arab Emirates |
| 2016 | 54.13934376 | 0.835220709910024 | 7.22056681285173 | 46.9187769441683 | United Arab Emirates |
| 2017 | 50.30717133 | 0.837213552633191 | 6.55076831112082 | 43.7564030201892 | United Arab Emirates |
| 2018 | 43.65393622 | 0.839900140178067 | 6.49213069124879 | 37.1618055283112 | United Arab Emirates |
| 2019 | 38.09944844 | 0.842985326255825 | 6.50010705019656 | 31.5993413889134 | United Arab Emirates |
| 2020 | 47.44489556 | 0.846151975991622 | 6.60906618876429 | 40.8358293670757 | United Arab Emirates |
| 2021 | 29.0794531 | 0.849317734035032 | 5.82153574968682 | 23.2579173511032 | United Arab Emirates |
| 1990 | 15926.511879182 | 0.215664585570251 | 5646.49747244467 | 10280.0144067373 | Yemen |
| 1991 | 14966.5153954193 | 0.222885408676441 | 5078.10476442958 | 9888.41063098972 | Yemen |
| 1992 | 14186.4054949978 | 0.230415045958638 | 3753.81691035977 | 10432.588584638 | Yemen |
| 1993 | 13354.1250118579 | 0.238025246263208 | 3598.49094710256 | 9755.63406475534 | Yemen |
| 1994 | 12498.6673184706 | 0.245879426395306 | 3470.21995235251 | 9028.44736611809 | Yemen |
| 1995 | 11529.467552222 | 0.253962571622302 | 3007.71386005683 | 8521.75369216518 | Yemen |
| 1996 | 10622.1254605618 | 0.262278612845771 | 2748.02780096082 | 7874.09765960098 | Yemen |
| 1997 | 9786.82307458467 | 0.270782535711374 | 2173.60271719185 | 7613.22035739282 | Yemen |
| 1998 | 8923.38955265571 | 0.279500507186773 | 2103.31546571258 | 6820.07408694313 | Yemen |
| 1999 | 8107.78007637113 | 0.288261301367107 | 2155.36359973609 | 5952.41647663504 | Yemen |
| 2000 | 7348.74951808385 | 0.297145157465494 | 2119.80199407672 | 5228.94752400713 | Yemen |
| 2001 | 6603.38362163139 | 0.306112423990989 | 2088.76840910244 | 4514.61521252895 | Yemen |
| 2002 | 6167.30460170487 | 0.31506502273303 | 2102.2328152373 | 4065.07178646757 | Yemen |
| 2003 | 5574.00292016721 | 0.324081688959523 | 2064.95123506634 | 3509.05168510087 | Yemen |
| 2004 | 4934.63396146605 | 0.333371285035603 | 1996.23006041397 | 2938.40390105208 | Yemen |
| 2005 | 4300.93790577289 | 0.343462561815387 | 1774.44020466267 | 2526.49770111022 | Yemen |
| 2006 | 3746.52912084301 | 0.353472947292631 | 1754.9990212448 | 1991.53009959821 | Yemen |
| 2007 | 3545.39270097187 | 0.363301217705729 | 1419.91747557768 | 2125.47522539419 | Yemen |
| 2008 | 3189.95172517522 | 0.373140288261273 | 1219.93208077517 | 1970.01964440005 | Yemen |
| 2009 | 2829.75298680548 | 0.382762252118378 | 1092.82686677283 | 1736.92612003265 | Yemen |
| 2010 | 2482.08346454596 | 0.392936641182874 | 1035.91879971969 | 1446.16466482627 | Yemen |
| 2011 | 2245.0897145293 | 0.40147180053512 | 929.252686771782 | 1315.83702775752 | Yemen |
| 2012 | 1743.65012337812 | 0.409561348808444 | 873.183072028601 | 870.467051349519 | Yemen |
| 2013 | 1500.36894140325 | 0.417642404804605 | 793.980303325782 | 706.388638077468 | Yemen |
| 2014 | 1252.88075530709 | 0.425189667293499 | 692.643081316567 | 560.237673990523 | Yemen |
| 2015 | 1119.91917320947 | 0.430197789315438 | 435.654511281077 | 684.264661928393 | Yemen |
| 2016 | 1198.39279217182 | 0.434232982818394 | 424.316339089144 | 774.076453082676 | Yemen |
| 2017 | 1625.59873266855 | 0.437549155246091 | 422.716827091354 | 1202.8819055772 | Yemen |
| 2018 | 1250.52010299869 | 0.440727991364019 | 416.414085851624 | 834.106017147066 | Yemen |
| 2019 | 1115.49949869247 | 0.444001399873635 | 425.09542809889 | 690.40407059358 | Yemen |
| 2020 | 700.79670435541 | 0.44689306923482 | 408.906916169699 | 291.889788185711 | Yemen |
| 2021 | 630.21064390967 | 0.450376375244811 | 409.406209376219 | 220.804434533451 | Yemen |
| 1990 | 13336.4085060713 | 0.173832165473389 | 8713.48754476696 | 4622.92096130434 | Afghanistan |
| 1991 | 13827.2015170868 | 0.176472549509622 | 8733.17642476628 | 5094.02509232051 | Afghanistan |
| 1992 | 14222.2336757103 | 0.179633724437178 | 8700.85190849044 | 5521.38176721986 | Afghanistan |
| 1993 | 14919.7118479783 | 0.18018371261059 | 8651.58627732686 | 6268.12557065144 | Afghanistan |
| 1994 | 13479.5987567428 | 0.178518660381455 | 8719.58971643066 | 4760.00904031214 | Afghanistan |
| 1995 | 13954.8548591206 | 0.17827912756413 | 8678.91433865523 | 5275.94052046537 | Afghanistan |
| 1996 | 13510.4102655369 | 0.178085403812013 | 8671.36671546338 | 4839.04355007352 | Afghanistan |
| 1997 | 13728.4874203502 | 0.177884124107706 | 8696.53580991913 | 5031.95161043107 | Afghanistan |
| 1998 | 13565.32264 | 0.177513403151835 | 8756.63810488316 | 4808.68453713685 | Afghanistan |
| 1999 | 13074.0055740128 | 0.177075239430658 | 8788.26751834896 | 4285.73805566384 | Afghanistan |
| 2000 | 12623.1945481269 | 0.177025771968387 | 8678.33229434068 | 3944.86225378622 | Afghanistan |
| 2001 | 12435.4771844038 | 0.177773144391168 | 8734.82531695723 | 3700.65186744657 | Afghanistan |
| 2002 | 11724.8889756718 | 0.183808429860528 | 8722.16706525565 | 3002.72191041615 | Afghanistan |
| 2003 | 11784.9196286598 | 0.19051899147664 | 8096.1373875852 | 3688.7822410746 | Afghanistan |
| 2004 | 11247.8570450156 | 0.196903541380771 | 7376.04870872472 | 3871.80833629088 | Afghanistan |
| 2005 | 10378.3778619469 | 0.203928133334003 | 6632.9807331157 | 3745.3971288312 | Afghanistan |
| 2006 | 9221.31598622692 | 0.211074194179476 | 6090.46642736319 | 3130.84955886373 | Afghanistan |
| 2007 | 8251.11929937702 | 0.219627610708056 | 4999.34414193599 | 3251.77515744103 | Afghanistan |
| 2008 | 7225.98383769404 | 0.22808917063877 | 4455.89772516833 | 2770.08611252571 | Afghanistan |
| 2009 | 6315.7202983862 | 0.237900824236794 | 3576.8814833313 | 2738.8388150549 | Afghanistan |
| 2010 | 5742.60016002401 | 0.247759948877388 | 3329.70839580461 | 2412.8917642194 | Afghanistan |
| 2011 | 5224.39947398866 | 0.257041675822107 | 2730.96164614378 | 2493.43782784488 | Afghanistan |
| 2012 | 4763.39189703813 | 0.266484406571128 | 2483.37180337246 | 2280.02009366567 | Afghanistan |
| 2013 | 4678.55690594687 | 0.275636558072642 | 2142.28171296542 | 2536.27519298145 | Afghanistan |
| 2014 | 4387.08325306485 | 0.284030351035349 | 2120.39847466545 | 2266.6847783994 | Afghanistan |
| 2015 | 4126.28501964528 | 0.291849504924728 | 2146.30694298257 | 1979.97807666271 | Afghanistan |
| 2016 | 3876.66717618699 | 0.299630695812728 | 2155.71692828697 | 1720.95024790002 | Afghanistan |
| 2017 | 3484.73093093441 | 0.307424618143983 | 2142.31686006087 | 1342.41407087354 | Afghanistan |
| 2018 | 2785.85864436565 | 0.314866093354221 | 2128.17883238544 | 657.67981198021 | Afghanistan |
| 2019 | 2456.02188292822 | 0.322454046974177 | 2113.12464608167 | 342.897236846547 | Afghanistan |
| 2020 | 2075.79518978397 | 0.32983006796412 | 2010.22456421628 | 65.5706255676894 | Afghanistan |
| 2021 | 1806.65990452904 | 0.337199997837472 | 1806.65990452904 | 0 | Afghanistan |
| 1990 | 7112.40531548862 | 0.228548934038183 | 4501.22306680833 | 2611.18224868029 | Bangladesh |
| 1991 | 7357.21252725759 | 0.237207412585347 | 3459.41867099119 | 3897.7938562664 | Bangladesh |
| 1992 | 6748.03479818051 | 0.245247955858354 | 3338.07555135241 | 3409.9592468281 | Bangladesh |
| 1993 | 6329.51167942253 | 0.252090719955727 | 2989.16006228119 | 3340.35161714134 | Bangladesh |
| 1994 | 5864.00114033885 | 0.258997294481156 | 2717.21408226629 | 3146.78705807256 | Bangladesh |
| 1995 | 5347.39174141892 | 0.265293918356151 | 2459.73222574202 | 2887.6595156769 | Bangladesh |
| 1996 | 4744.31088730252 | 0.271177780650834 | 2182.27907876571 | 2562.03180853681 | Bangladesh |
| 1997 | 4180.72047404274 | 0.277773280387452 | 2156.74567266724 | 2023.9748013755 | Bangladesh |
| 1998 | 3620.49293280867 | 0.284515940436142 | 2176.03434322098 | 1444.45858958769 | Bangladesh |
| 1999 | 3156.02734330232 | 0.290951431147001 | 2132.52965683119 | 1023.49768647113 | Bangladesh |
| 2000 | 2804.15308354355 | 0.297251975652563 | 2120.60994173132 | 683.543141812231 | Bangladesh |
| 2001 | 2589.65675208308 | 0.303188546281531 | 2134.43013248554 | 455.226619597536 | Bangladesh |
| 2002 | 2479.92085800292 | 0.308640093479048 | 2114.04678969914 | 365.874068303775 | Bangladesh |
| 2003 | 2389.47451951205 | 0.3143600400365 | 2128.57060332509 | 260.903916186964 | Bangladesh |
| 2004 | 2322.0358676939 | 0.320922851356659 | 2094.2124963471 | 227.823371346797 | Bangladesh |
| 2005 | 2177.09175126926 | 0.328090510389871 | 2071.03727354891 | 106.054477720351 | Bangladesh |
| 2006 | 2047.9362935306 | 0.3356905979604 | 1983.27843679676 | 64.6578567338427 | Bangladesh |
| 2007 | 2027.85667832931 | 0.343932545818298 | 1768.36524100697 | 259.491437322345 | Bangladesh |
| 2008 | 1898.38316009196 | 0.352520032963442 | 1761.85993991139 | 136.523220180575 | Bangladesh |
| 2009 | 1763.80040543667 | 0.361481512825909 | 1430.68669937691 | 333.113706059759 | Bangladesh |
| 2010 | 1621.58444751817 | 0.370598956236852 | 1214.14084319112 | 407.443604327053 | Bangladesh |
| 2011 | 1514.4774199641 | 0.380124885726479 | 1125.92302939328 | 388.55439057082 | Bangladesh |
| 2012 | 1368.93811952877 | 0.39062355195618 | 1035.70041184841 | 333.237707680361 | Bangladesh |
| 2013 | 1284.4661118517 | 0.401706477929242 | 951.457047492986 | 333.009064358714 | Bangladesh |
| 2014 | 1208.65115286966 | 0.413263168891725 | 864.074319815218 | 344.576833054442 | Bangladesh |
| 2015 | 1202.68605589875 | 0.42548212139419 | 685.489155623867 | 517.196900274883 | Bangladesh |
| 2016 | 1163.47024266396 | 0.437709906182211 | 418.019789119028 | 745.450453544932 | Bangladesh |
| 2017 | 1090.83960678375 | 0.449752413112756 | 410.321378043662 | 680.518228740088 | Bangladesh |
| 2018 | 981.64794389554 | 0.461717748857041 | 379.722064106158 | 601.925879789382 | Bangladesh |
| 2019 | 898.54395155209 | 0.473264997623618 | 335.694934746141 | 562.849016805949 | Bangladesh |
| 2020 | 598.87732725301 | 0.483079169271683 | 327.017352145002 | 271.859975108008 | Bangladesh |
| 2021 | 536.03146589853 | 0.492420884955353 | 295.252883383663 | 240.778582514867 | Bangladesh |
| 1990 | 18463.3923330111 | 0.215039850206523 | 5564.80756117889 | 12898.5847718322 | Bhutan |
| 1991 | 17550.988217906 | 0.221312439958873 | 5092.8140891437 | 12458.1741287623 | Bhutan |
| 1992 | 16626.6490968547 | 0.228148271920468 | 4462.12552220072 | 12164.523574654 | Bhutan |
| 1993 | 16427.5207289285 | 0.236004687298356 | 3858.14290844154 | 12569.377820487 | Bhutan |
| 1994 | 15187.2282350649 | 0.244331246046266 | 3357.44346915113 | 11829.7847659138 | Bhutan |
| 1995 | 14222.8705467162 | 0.253291956861352 | 2974.77512235119 | 11248.095424365 | Bhutan |
| 1996 | 12862.821222598 | 0.262362745638857 | 2760.28629113529 | 10102.5349314627 | Bhutan |
| 1997 | 12260.0399278132 | 0.271487422119903 | 2167.80332777216 | 10092.236600041 | Bhutan |
| 1998 | 10407.6731905473 | 0.280440384324268 | 2179.57445675711 | 8228.09873379019 | Bhutan |
| 1999 | 7966.41217852943 | 0.28951048779873 | 2139.57637504722 | 5826.83580348221 | Bhutan |
| 2000 | 8550.44142143692 | 0.298804874805939 | 2144.05920093913 | 6406.38222049779 | Bhutan |
| 2001 | 7580.20570449045 | 0.308200652419302 | 2113.73553381388 | 5466.47017067657 | Bhutan |
| 2002 | 6872.77415023097 | 0.3179435358725 | 2109.34538162651 | 4763.42876860446 | Bhutan |
| 2003 | 6443.32721529117 | 0.327670422082998 | 2071.22679731063 | 4372.10041798054 | Bhutan |
| 2004 | 5906.75985911374 | 0.337286494753829 | 1857.61730615865 | 4049.14255295509 | Bhutan |
| 2005 | 5334.7910391338 | 0.346998577564248 | 1798.59089026982 | 3536.20014886398 | Bhutan |
| 2006 | 4747.81498334304 | 0.356824209168712 | 1704.66034839801 | 3043.15463494503 | Bhutan |
| 2007 | 4227.46388994276 | 0.367560497563767 | 1229.16869925158 | 2998.29519069118 | Bhutan |
| 2008 | 3724.60400290595 | 0.378233542838379 | 1142.3174026607 | 2582.28660024525 | Bhutan |
| 2009 | 3206.74454390365 | 0.388977161920706 | 1036.12423187547 | 2170.62031202818 | Bhutan |
| 2010 | 2834.87102446758 | 0.399751936485664 | 937.511316999378 | 1897.3597074682 | Bhutan |
| 2011 | 2581.92829441194 | 0.410149248617919 | 873.225305856473 | 1708.70298855547 | Bhutan |
| 2012 | 2357.85374994897 | 0.419443216044258 | 771.876323402536 | 1585.97742654643 | Bhutan |
| 2013 | 2158.89475509753 | 0.427790674624113 | 522.825674423746 | 1636.06908067378 | Bhutan |
| 2014 | 1998.82901934254 | 0.434969269672115 | 423.816539552527 | 1575.01247979001 | Bhutan |
| 2015 | 1860.60340833934 | 0.44181074828187 | 426.036061856706 | 1434.56734648263 | Bhutan |
| 2016 | 1721.88596729019 | 0.448401854533875 | 410.436614100965 | 1311.44935318923 | Bhutan |
| 2017 | 1585.55477340088 | 0.454280496545609 | 410.493962462783 | 1175.0608109381 | Bhutan |
| 2018 | 1467.09230798733 | 0.459624710991057 | 395.352281585953 | 1071.74002640138 | Bhutan |
| 2019 | 1363.33019712468 | 0.464633353490442 | 381.563696469049 | 981.766500655631 | Bhutan |
| 2020 | 1276.46632245657 | 0.468714478980695 | 345.936471304118 | 930.529851152452 | Bhutan |
| 2021 | 1187.55496786854 | 0.473062378179926 | 335.006922136674 | 852.548045731866 | Bhutan |
| 1990 | 10805.4425772926 | 0.332593603012361 | 1985.03559755329 | 8820.40697973931 | India |
| 1991 | 10491.9261080774 | 0.338591779142716 | 1854.67339371491 | 8637.25271436249 | India |
| 1992 | 10277.4346983665 | 0.344591727089114 | 1766.83575881319 | 8510.59893955331 | India |
| 1993 | 9826.02777658483 | 0.350685396969938 | 1767.48144409476 | 8058.54633249007 | India |
| 1994 | 9358.41884900454 | 0.356996030489868 | 1706.93919029602 | 7651.47965870852 | India |
| 1995 | 8992.03095416624 | 0.363418500556262 | 1413.31604752926 | 7578.71490663698 | India |
| 1996 | 8559.95373291031 | 0.370018643898342 | 1240.21673541072 | 7319.73699749959 | India |
| 1997 | 8137.68866400819 | 0.37635876286452 | 1176.97292102132 | 6960.71574298687 | India |
| 1998 | 7627.85136364675 | 0.382817848453708 | 1089.48490818763 | 6538.36645545912 | India |
| 1999 | 6996.4258156404 | 0.389453889040872 | 1025.49957250518 | 5970.92624313522 | India |
| 2000 | 6484.96283146859 | 0.395752331114503 | 982.515741418513 | 5502.44709005008 | India |
| 2001 | 6032.89040748362 | 0.401837472284192 | 928.069961412242 | 5104.82044607138 | India |
| 2002 | 5726.98809543792 | 0.407411413581757 | 873.713614116133 | 4853.27448132179 | India |
| 2003 | 5393.89240826899 | 0.413115782576536 | 852.976675685186 | 4540.9157325838 | India |
| 2004 | 5053.00383287944 | 0.419184960649408 | 775.178423761551 | 4277.82540911789 | India |
| 2005 | 4775.74426141474 | 0.425913061870066 | 691.414657 | 4084.32960443484 | India |
| 2006 | 4476.38680612766 | 0.433321558442149 | 422.336065983347 | 4054.05074014431 | India |
| 2007 | 4166.96711934758 | 0.441351460389445 | 413.636613936773 | 3753.33050541081 | India |
| 2008 | 3857.90151046744 | 0.44923585077668 | 408.77311499629 | 3449.12839547115 | India |
| 2009 | 3553.11975086167 | 0.457672331967412 | 396.748551869503 | 3156.37119899217 | India |
| 2010 | 3267.65120697814 | 0.467018995596075 | 361.936099598334 | 2905.71510737981 | India |
| 2011 | 2997.93600345971 | 0.476832909015635 | 335.186307837545 | 2662.74969562216 | India |
| 2012 | 2743.26476401226 | 0.487210875591002 | 320.846345770534 | 2422.41841824173 | India |
| 2013 | 2537.32239751702 | 0.498317981286522 | 235.613463920426 | 2301.70893359659 | India |
| 2014 | 2252.55341927635 | 0.509722099778239 | 215.04126989398 | 2037.51214938237 | India |
| 2015 | 2006.6533784804 | 0.521242718548368 | 199.066476046958 | 1807.58690243344 | India |
| 2016 | 1670.00086250098 | 0.532220023184224 | 169.79043829568 | 1500.2104242053 | India |
| 2017 | 1309.9757218229 | 0.54238850718096 | 155.203328382597 | 1154.7723934403 | India |
| 2018 | 1048.62242211834 | 0.551930326455295 | 145.909643106479 | 902.71277901186 | India |
| 2019 | 841.99395135923 | 0.560809926886154 | 139.57687855504 | 702.41707280419 | India |
| 2020 | 925.4653633 | 0.568138149330912 | 110.690686595002 | 814.774676668798 | India |
| 2021 | 809.09845793642 | 0.575401649396992 | 89.1191544828799 | 719.97930345354 | India |
| 1990 | 12826.160463441 | 0.199560654182432 | 7328.15430082788 | 5498.00616261312 | Nepal |
| 1991 | 12885.5136 | 0.205687442933642 | 6614.59120917944 | 6270.92239572056 | Nepal |
| 1992 | 11653.1770876345 | 0.212113089793247 | 5980.23273565122 | 5672.94435198328 | Nepal |
| 1993 | 11084.6018400951 | 0.218843431287675 | 5144.2538553456 | 5940.3479847495 | Nepal |
| 1994 | 10474.3458655501 | 0.226073059669684 | 4636.43184470326 | 5837.91402084684 | Nepal |
| 1995 | 9843.50293270793 | 0.233253154418138 | 3859.26874477563 | 5984.2341879323 | Nepal |
| 1996 | 9179.96745445956 | 0.240700615739874 | 3548.06607677377 | 5631.90137768579 | Nepal |
| 1997 | 8420.20616657305 | 0.248382796560591 | 3336.76880320548 | 5083.43736336758 | Nepal |
| 1998 | 7695.11966499729 | 0.256227178679174 | 2977.91775847172 | 4717.20190652557 | Nepal |
| 1999 | 6922.42150120068 | 0.264359180313156 | 2470.20981896916 | 4452.21168223152 | Nepal |
| 2000 | 6228.74575366081 | 0.272719114894473 | 2157.99330363235 | 4070.75245002846 | Nepal |
| 2001 | 5560.46245251829 | 0.281287467400935 | 2141.29568312299 | 3419.1667693953 | Nepal |
| 2002 | 4830.50519120362 | 0.289513607929153 | 2118.26109341236 | 2712.24409779126 | Nepal |
| 2003 | 4214.54603985199 | 0.297772595071107 | 2133.36295068995 | 2081.18308916204 | Nepal |
| 2004 | 3711.01844136341 | 0.306196530322722 | 2095.94862023035 | 1615.06982113307 | Nepal |
| 2005 | 3598.3549942072 | 0.314661796325301 | 2111.33831844236 | 1487.01667576484 | Nepal |
| 2006 | 3374.73640197752 | 0.323056345509418 | 2067.26106975117 | 1307.47533222635 | Nepal |
| 2007 | 3108.9411714907 | 0.331445610061963 | 1985.11451945471 | 1123.82665203599 | Nepal |
| 2008 | 2866.6926503349 | 0.340150705786803 | 1864.80383004883 | 1001.88882028607 | Nepal |
| 2009 | 2893.52789792758 | 0.348701893463869 | 1763.59792253612 | 1129.92997539146 | Nepal |
| 2010 | 2413.61616547696 | 0.356868100367112 | 1708.96352175861 | 704.652643718346 | Nepal |
| 2011 | 2044.30708049661 | 0.364642604726269 | 1306.00890600435 | 738.298174492258 | Nepal |
| 2012 | 1791.72814425809 | 0.372437587888826 | 1229.68068369099 | 562.047460567103 | Nepal |
| 2013 | 1677.44056293808 | 0.380022326031393 | 1134.34473721295 | 543.095825725127 | Nepal |
| 2014 | 1472.70372944521 | 0.387689088395059 | 1089.48729623238 | 383.216433212829 | Nepal |
| 2015 | 1342.36424671418 | 0.395021154474843 | 974.033233192135 | 368.331013522045 | Nepal |
| 2016 | 1159.84575782537 | 0.401628776477432 | 930.31375637408 | 229.53200145129 | Nepal |
| 2017 | 971.58084688937 | 0.408463874114472 | 874.390755991551 | 97.1900908978188 | Nepal |
| 2018 | 771.13333843981 | 0.41523668446613 | 771.13333843981 | 0 | Nepal |
| 2019 | 658.9266119 | 0.42200725174098 | 658.9266119 | 0 | Nepal |
| 2020 | 546.2197381 | 0.427417089099115 | 486.10683251811 | 60.1129055618896 | Nepal |
| 2021 | 480.49212840693 | 0.43317463481343 | 431.193803329612 | 49.2983250773181 | Nepal |
| 1990 | 10834.4324429219 | 0.310467621430628 | 2098.53525849818 | 8735.89718442372 | Pakistan |
| 1991 | 10278.0166560977 | 0.316265666853964 | 2129.08075891672 | 8148.93589718098 | Pakistan |
| 1992 | 9827.61497921334 | 0.322248050692924 | 2106.9198121311 | 7720.69516708224 | Pakistan |
| 1993 | 9414.50104102382 | 0.328219252463403 | 2033.77588409536 | 7380.72515692845 | Pakistan |
| 1994 | 8927.04501251518 | 0.334433128288919 | 1982.6219978895 | 6944.42301462568 | Pakistan |
| 1995 | 8456.12334696961 | 0.340926296299194 | 1863.21358933329 | 6592.90975763632 | Pakistan |
| 1996 | 7880.97635976533 | 0.347656202917864 | 1774.72735716233 | 6106.249003 | Pakistan |
| 1997 | 7439.5803007686 | 0.354218627290782 | 1760.5607960904 | 5679.0195046782 | Pakistan |
| 1998 | 7014.16347499241 | 0.360875335601641 | 1586.4332341549 | 5427.73024083751 | Pakistan |
| 1999 | 6732.13145047788 | 0.367641396836045 | 1238.06889225116 | 5494.06255822672 | Pakistan |
| 2000 | 6458.17324766174 | 0.374319132467609 | 1167.99989974284 | 5290.1733479189 | Pakistan |
| 2001 | 6228.53139835074 | 0.38059392725045 | 1126.06166619432 | 5102.46973215642 | Pakistan |
| 2002 | 5986.70751050111 | 0.386629992356461 | 1088.64187863515 | 4898.06563186596 | Pakistan |
| 2003 | 5763.68297657983 | 0.392719262841918 | 1033.7327546818 | 4729.95022189803 | Pakistan |
| 2004 | 5578.29324829136 | 0.399084167395961 | 924.371218772654 | 4653.92202951871 | Pakistan |
| 2005 | 5389.19372531547 | 0.40566743657674 | 892.896040491911 | 4496.29768482356 | Pakistan |
| 2006 | 5139.2437727596 | 0.412234830478788 | 853.456744831317 | 4285.78702792828 | Pakistan |
| 2007 | 4899.50847276115 | 0.418519755739044 | 791.268144328131 | 4108.24032843302 | Pakistan |
| 2008 | 4877.07188695089 | 0.424348199605747 | 680.212305459147 | 4196.85958149174 | Pakistan |
| 2009 | 4679.82215436663 | 0.430019832570808 | 429.502702982202 | 4250.31945138443 | Pakistan |
| 2010 | 4364.34102425786 | 0.4356406573129 | 424.367312771085 | 3939.97371148677 | Pakistan |
| 2011 | 4060.45827078837 | 0.441455585075092 | 425.330196509705 | 3635.12807427867 | Pakistan |
| 2012 | 3740.07492120615 | 0.447096599297877 | 414.59566322535 | 3325.4792579808 | Pakistan |
| 2013 | 3582.26234187349 | 0.452823095279952 | 410.627871191301 | 3171.63447068219 | Pakistan |
| 2014 | 3371.60019101531 | 0.458781424149631 | 396.096824919492 | 2975.50336609582 | Pakistan |
| 2015 | 3194.14456920663 | 0.464792667642097 | 378.263965873444 | 2815.88060333319 | Pakistan |
| 2016 | 2684.25615741544 | 0.471022167867849 | 343.446243972061 | 2340.80991344338 | Pakistan |
| 2017 | 2114.41782558348 | 0.4775441366317 | 334.053247541689 | 1780.36457804179 | Pakistan |
| 2018 | 1581.9564599283 | 0.484326128033838 | 323.041303506654 | 1258.91515642165 | Pakistan |
| 2019 | 1304.06095927373 | 0.491022981031026 | 296.904118729725 | 1007.15684054401 | Pakistan |
| 2020 | 1175.19858787152 | 0.497364405611904 | 244.340091318999 | 930.858496552521 | Pakistan |
| 2021 | 1021.58539164004 | 0.504028688613448 | 224.024093794067 | 797.561297845973 | Pakistan |
| 1990 | 35923.8771647579 | 0.270736935221319 | 2143.54851447767 | 33780.3286502802 | Angola |
| 1991 | 34332.165204127 | 0.275160624620397 | 2194.49910184772 | 32137.6661022793 | Angola |
| 1992 | 32353.7392093872 | 0.279231499654396 | 2167.51458980531 | 30186.2246195819 | Angola |
| 1993 | 31094.683187792 | 0.281867702816568 | 2141.57731471261 | 28953.1058730794 | Angola |
| 1994 | 30244.3485494335 | 0.284487594233541 | 2156.24116168941 | 28088.1073877441 | Angola |
| 1995 | 29216.4100445604 | 0.287447813395598 | 2126.72012254855 | 27089.6899220118 | Angola |
| 1996 | 27562.2438577213 | 0.29142127918547 | 2136.95186001523 | 25425.2919977061 | Angola |
| 1997 | 26475.1175820434 | 0.295433993200957 | 2147.18600984341 | 24327.93157 | Angola |
| 1998 | 25335.6929555232 | 0.299553353195731 | 2092.49469490236 | 23243.1982606208 | Angola |
| 1999 | 24108.6602 | 0.303720041599183 | 2132.5760715339 | 21976.0841293161 | Angola |
| 2000 | 22976.1119438601 | 0.307603311847111 | 2128.78965180379 | 20847.3222920563 | Angola |
| 2001 | 21637.3266950552 | 0.311697534329848 | 2137.21619519752 | 19500.1104998577 | Angola |
| 2002 | 20130.2934102006 | 0.316706570519731 | 2133.5290924283 | 17996.7643177723 | Angola |
| 2003 | 18534.2016513855 | 0.321777361754333 | 2099.25528316413 | 16434.9463682214 | Angola |
| 2004 | 17628.5478507219 | 0.32739526565521 | 2077.23500646406 | 15551.3128442578 | Angola |
| 2005 | 15929.3887088596 | 0.333785188103559 | 1986.74924797535 | 13942.6394608843 | Angola |
| 2006 | 16264.3215946758 | 0.34101262184868 | 1852.85490990258 | 14411.4666847732 | Angola |
| 2007 | 13668.4659827765 | 0.34884684076221 | 1775.39632595719 | 11893.0696568193 | Angola |
| 2008 | 11916.8488129274 | 0.357082005452131 | 1704.3051695938 | 10212.5436433336 | Angola |
| 2009 | 10469.3384235436 | 0.364407743836554 | 1308.94274010853 | 9160.39568343507 | Angola |
| 2010 | 9309.80807915324 | 0.372033537037443 | 1218.18558296555 | 8091.62249618769 | Angola |
| 2011 | 8126.11909810158 | 0.380006613064486 | 1123.05363684124 | 7003.06546126034 | Angola |
| 2012 | 6824.80761359148 | 0.387891857376226 | 1095.61638807734 | 5729.19122551414 | Angola |
| 2013 | 5637.40167918243 | 0.395796328674416 | 978.586745955203 | 4658.81493322723 | Angola |
| 2014 | 4443.91493601989 | 0.403720085675527 | 887.98993106264 | 3555.92500495725 | Angola |
| 2015 | 3831.5566561856 | 0.411372026050491 | 854.229177337658 | 2977.32747884794 | Angola |
| 2016 | 3315.7167414844 | 0.418822062446251 | 802.514109410407 | 2513.20263207399 | Angola |
| 2017 | 2884.27799841904 | 0.426177082467176 | 563.295982138653 | 2320.98201628039 | Angola |
| 2018 | 2487.95281092085 | 0.433355005074473 | 428.476922962071 | 2059.47588795878 | Angola |
| 2019 | 2242.07665221718 | 0.440454598010708 | 415.542829318113 | 1826.53382289907 | Angola |
| 2020 | 1961.17023817675 | 0.447283991858922 | 410.844854079263 | 1550.32538409749 | Angola |
| 2021 | 1699.19617741189 | 0.453721949492174 | 409.331888517982 | 1289.86428889391 | Angola |
| 1990 | 34354.9999366703 | 0.216825190875377 | 5664.11924111986 | 28690.8806955504 | Central African Republic |
| 1991 | 33715.3368600935 | 0.220191656485842 | 5034.86204683954 | 28680.474813254 | Central African Republic |
| 1992 | 33173.7012128458 | 0.223007541295157 | 5020.14794914891 | 28153.5532636969 | Central African Republic |
| 1993 | 32440.1064211417 | 0.225451125286014 | 4445.376697 | 27994.7297242287 | Central African Republic |
| 1994 | 31422.8281632067 | 0.228487724402372 | 4529.44431647572 | 26893.383846731 | Central African Republic |
| 1995 | 30686.0735880275 | 0.231696792476692 | 3893.35807901156 | 26792.7155090159 | Central African Republic |
| 1996 | 30191.4548823161 | 0.233989591180505 | 3812.29264348222 | 26379.1622388339 | Central African Republic |
| 1997 | 30091.4096377268 | 0.236671999328608 | 3570.73088247165 | 26520.6787552552 | Central African Republic |
| 1998 | 28805.1096497186 | 0.239743528872866 | 3688.17539444588 | 25116.9342552727 | Central African Republic |
| 1999 | 28071.5791047367 | 0.242988530190618 | 3336.5387393758 | 24735.0403653609 | Central African Republic |
| 2000 | 27410.8434972277 | 0.246021131876903 | 3318.94745436027 | 24091.8960428674 | Central African Republic |
| 2001 | 27102.388253924 | 0.249246740494197 | 3302.69065494288 | 23799.6975989811 | Central African Republic |
| 2002 | 26435.6458426828 | 0.252524369440269 | 3021.60542451564 | 23414.0404181672 | Central African Republic |
| 2003 | 25737.977430484 | 0.25510151863237 | 2995.11785269554 | 22742.8595777885 | Central African Republic |
| 2004 | 25396.0739908745 | 0.257778778286371 | 2709.73344567247 | 22686.340545202 | Central African Republic |
| 2005 | 24597.5540272429 | 0.26045191761313 | 2710.8260587681 | 21886.7279684748 | Central African Republic |
| 2006 | 24872.0438952965 | 0.263712446226446 | 2491.10712064625 | 22380.9367746503 | Central African Republic |
| 2007 | 24921.7258882835 | 0.2673321946143 | 2490.49534944243 | 22431.2305388411 | Central African Republic |
| 2008 | 24662.7555648766 | 0.27115811844024 | 2126.4123567764 | 22536.3432081002 | Central African Republic |
| 2009 | 24241.959959747 | 0.275338515436591 | 2154.23584546749 | 22087.7241142795 | Central African Republic |
| 2010 | 23453.0207950034 | 0.279981381762208 | 2183.42722229029 | 21269.5935727131 | Central African Republic |
| 2011 | 22295.4057795313 | 0.285097245088929 | 2169.6572540755 | 20125.7485254558 | Central African Republic |
| 2012 | 20785.7745768944 | 0.290460023001198 | 2146.64809253647 | 18639.1264843579 | Central African Republic |
| 2013 | 20018.1009679455 | 0.290104929568684 | 2172.45593502591 | 17845.6450329196 | Central African Republic |
| 2014 | 19585.6413453932 | 0.289999498191724 | 2139.89693307609 | 17445.7444123171 | Central African Republic |
| 2015 | 19197.1997323529 | 0.290827871178726 | 2135.50559543835 | 17061.6941369145 | Central African Republic |
| 2016 | 18586.4690656692 | 0.29243039953372 | 2086.62628207816 | 16499.842783591 | Central African Republic |
| 2017 | 17986.4417948306 | 0.294752243072343 | 2156.85549449274 | 15829.5863003379 | Central African Republic |
| 2018 | 17199.1832033147 | 0.298011223092936 | 2119.9327501896 | 15079.2504531251 | Central African Republic |
| 2019 | 16251.5915464728 | 0.301953064146936 | 2147.34044127439 | 14104.2511051984 | Central African Republic |
| 2020 | 13134.3730137075 | 0.305426298180724 | 2103.98853189875 | 11030.3844818087 | Central African Republic |
| 2021 | 12365.3630340661 | 0.309167689992842 | 2122.64551374831 | 10242.7175203178 | Central African Republic |
| 1990 | 18075.9677471635 | 0.420654655109748 | 775.03185743379 | 17300.9358897297 | Congo |
| 1991 | 17568.3290561391 | 0.426734373972141 | 568.244216386776 | 17000.0848397523 | Congo |
| 1992 | 17110.9836379794 | 0.431987661930376 | 421.902214846566 | 16689.0814231328 | Congo |
| 1993 | 16735.9477349051 | 0.436606593595936 | 421.347932911435 | 16314.5998019937 | Congo |
| 1994 | 16334.5698794382 | 0.440168346550654 | 424.514163478551 | 15910.0557159597 | Congo |
| 1995 | 15968.36537 | 0.443744125258797 | 414.675859944051 | 15553.689512646 | Congo |
| 1996 | 15715.5512777699 | 0.447566642471598 | 412.052879925152 | 15303.4983978447 | Congo |
| 1997 | 15609.3915998585 | 0.451026961311742 | 410.649825075482 | 15198.741774783 | Congo |
| 1998 | 15072.8506767809 | 0.454445045209992 | 410.826207968977 | 14662.0244688119 | Congo |
| 1999 | 14378.0155227206 | 0.457176539853177 | 396.60301440584 | 13981.4125083148 | Congo |
| 2000 | 13814.4744765794 | 0.460764813822723 | 376.684358003258 | 13437.7901185761 | Congo |
| 2001 | 13034.6224383417 | 0.463751682463704 | 380.91795218325 | 12653.7044861584 | Congo |
| 2002 | 12514.4555651152 | 0.467037267744411 | 359.873071245687 | 12154.5824938695 | Congo |
| 2003 | 11810.8777433318 | 0.470134373890519 | 342.095490050819 | 11468.782253281 | Congo |
| 2004 | 11103.5672081606 | 0.473612435565969 | 336.316693746912 | 10767.2505144137 | Congo |
| 2005 | 10940.3206647647 | 0.478137626987132 | 333.833293684271 | 10606.4873710804 | Congo |
| 2006 | 10361.0358187613 | 0.483355821647145 | 324.389119943485 | 10036.6466988178 | Congo |
| 2007 | 10048.7551223632 | 0.48764073952289 | 310.504659443041 | 9738.25046292016 | Congo |
| 2008 | 9362.69659133417 | 0.492822854998204 | 297.643713551517 | 9065.05287778265 | Congo |
| 2009 | 8595.24792306426 | 0.498299969081535 | 237.045818502332 | 8358.20210456193 | Congo |
| 2010 | 7648.60172985627 | 0.505542849123574 | 222.66076304991 | 7425.94096680636 | Congo |
| 2011 | 6872.02990747278 | 0.513815777196028 | 206.801052911471 | 6665.22885456131 | Congo |
| 2012 | 5761.37768135077 | 0.522928282789849 | 199.549283196055 | 5561.82839815472 | Congo |
| 2013 | 4638.5141871496 | 0.531551678858768 | 181.339490507779 | 4457.17469664182 | Congo |
| 2014 | 3555.97485785655 | 0.540594486203119 | 155.119528782487 | 3400.85532907406 | Congo |
| 2015 | 3118.42225753661 | 0.548414029703538 | 149.113506740541 | 2969.30875079607 | Congo |
| 2016 | 2805.08813532455 | 0.555108607713661 | 143.864806910734 | 2661.22332841382 | Congo |
| 2017 | 2498.82979357448 | 0.561834100154124 | 139.221150513524 | 2359.60864306096 | Congo |
| 2018 | 2235.23112623791 | 0.567793969630843 | 106.748825072538 | 2128.48230116537 | Congo |
| 2019 | 3181.92344136286 | 0.573373665303225 | 84.7985704999982 | 3097.12487086286 | Congo |
| 2020 | 2069.01127684328 | 0.578451546357964 | 83.9501201410622 | 1985.06115670222 | Congo |
| 2021 | 1734.48486595353 | 0.583075236262154 | 83.9513742390899 | 1650.53349171444 | Congo |
| 1990 | 28001.9154683608 | 0.289843210276958 | 2148.77518562757 | 25853.1402827332 | Democratic Republic of the Congo |
| 1991 | 27706.6046881802 | 0.290431567230931 | 2171.19076272719 | 25535.413925453 | Democratic Republic of the Congo |
| 1992 | 26991.7680909901 | 0.290959242467126 | 2151.818597 | 24839.9494938811 | Democratic Republic of the Congo |
| 1993 | 26954.6247353483 | 0.289628789613637 | 2169.9082069556 | 24784.7165283927 | Democratic Republic of the Congo |
| 1994 | 29948.1795513956 | 0.286859145655148 | 2175.24658411215 | 27772.9329672835 | Democratic Republic of the Congo |
| 1995 | 27169.1367602172 | 0.283967532380546 | 2207.73588759199 | 24961.4008726252 | Democratic Republic of the Congo |
| 1996 | 27648.4908403172 | 0.280413159880167 | 2187.63389252539 | 25460.8569477918 | Democratic Republic of the Congo |
| 1997 | 28126.955368649 | 0.276336502842209 | 2151.82273397786 | 25975.1326346711 | Democratic Republic of the Congo |
| 1998 | 29157.5473745866 | 0.271813823470493 | 2056.28003911121 | 27101.2673354754 | Democratic Republic of the Congo |
| 1999 | 29243.5058457396 | 0.267367662319482 | 2436.67954534425 | 26806.8263003954 | Democratic Republic of the Congo |
| 2000 | 29940.4262767789 | 0.262456746954109 | 2769.26910296625 | 27171.1571738127 | Democratic Republic of the Congo |
| 2001 | 29754.4890494991 | 0.257857504999669 | 2730.6484434744 | 27023.8406060247 | Democratic Republic of the Congo |
| 2002 | 29270.9070174912 | 0.254136360333323 | 2993.28987286446 | 26277.6171446267 | Democratic Republic of the Congo |
| 2003 | 28799.362420285 | 0.252333884691643 | 2995.17497091742 | 25804.1874493676 | Democratic Republic of the Congo |
| 2004 | 27065.2140229329 | 0.252470918843583 | 2969.8576819496 | 24095.3563409833 | Democratic Republic of the Congo |
| 2005 | 25744.8837737369 | 0.254161023299852 | 2998.44577765553 | 22746.4379960814 | Democratic Republic of the Congo |
| 2006 | 24343.522757634 | 0.257355122656431 | 2693.08024126557 | 21650.4425163684 | Democratic Republic of the Congo |
| 2007 | 22834.0001475601 | 0.262272267392268 | 2755.05379357301 | 20078.9463539871 | Democratic Republic of the Congo |
| 2008 | 20864.3374036118 | 0.268103872304045 | 2496.5602134131 | 18367.7771901987 | Democratic Republic of the Congo |
| 2009 | 19167.3876313904 | 0.274361143997572 | 2135.37821535602 | 17032.0094160344 | Democratic Republic of the Congo |
| 2010 | 17399.3357275711 | 0.282223754466439 | 2181.09682803102 | 15218.2388995401 | Democratic Republic of the Congo |
| 2011 | 15525.8205661376 | 0.290872953141928 | 2117.1532320231 | 13408.6673341145 | Democratic Republic of the Congo |
| 2012 | 13548.5339392864 | 0.300454783216411 | 2150.65555461582 | 11397.8783846706 | Democratic Republic of the Congo |
| 2013 | 11612.2332419789 | 0.310831256324369 | 2138.12294430848 | 9474.11029767042 | Democratic Republic of the Congo |
| 2014 | 9898.81271180721 | 0.321622927081767 | 2153.99355875606 | 7744.81915305115 | Democratic Republic of the Congo |
| 2015 | 8224.13166254291 | 0.33242130666296 | 1987.51512804499 | 6236.61653449792 | Democratic Republic of the Congo |
| 2016 | 6890.62785351796 | 0.341988787240158 | 1777.63931769845 | 5112.98853581951 | Democratic Republic of the Congo |
| 2017 | 5568.14874280163 | 0.351102035190521 | 1769.45139670928 | 3798.69734609235 | Democratic Republic of the Congo |
| 2018 | 4819.03797384769 | 0.36028767655131 | 1560.23582127988 | 3258.80215256782 | Democratic Republic of the Congo |
| 2019 | 3958.34010648505 | 0.36934068409702 | 1227.34009919767 | 2731.00000728738 | Democratic Republic of the Congo |
| 2020 | 3329.83641338858 | 0.376712271159843 | 1173.97471256317 | 2155.86170082541 | Democratic Republic of the Congo |
| 2021 | 2903.56059417756 | 0.38317984920136 | 1088.12515199976 | 1815.4354421778 | Democratic Republic of the Congo |
| 1990 | 40002.6989222967 | 0.268783629292318 | 2127.77809411717 | 37874.9208281795 | Equatorial Guinea |
| 1991 | 38906.6662473009 | 0.275296991793454 | 2203.67531858201 | 36702.9909287189 | Equatorial Guinea |
| 1992 | 37453.4693333579 | 0.283487763833002 | 2140.52442895809 | 35312.9449043998 | Equatorial Guinea |
| 1993 | 35936.6065915058 | 0.292236031708562 | 2150.23118075793 | 33786.3754107479 | Equatorial Guinea |
| 1994 | 34440.7575022519 | 0.30068634012364 | 2141.78931512153 | 32298.9681871304 | Equatorial Guinea |
| 1995 | 33073.4296165961 | 0.310443551427585 | 2122.55383575665 | 30950.8757808394 | Equatorial Guinea |
| 1996 | 30681.8699762329 | 0.324601220926654 | 2059.22371572687 | 28622.646260506 | Equatorial Guinea |
| 1997 | 25938.1216563196 | 0.350550963012752 | 1774.29857843918 | 24163.8230778804 | Equatorial Guinea |
| 1998 | 22315.6622629578 | 0.371237163466133 | 1233.31724059643 | 21082.3450223614 | Equatorial Guinea |
| 1999 | 19051.5868555252 | 0.391128022748702 | 1037.4485601904 | 18014.1382953348 | Equatorial Guinea |
| 2000 | 16186.2274285297 | 0.411383449802448 | 848.80179612431 | 15337.4256324054 | Equatorial Guinea |
| 2001 | 12872.2943868119 | 0.433590056530441 | 418.540670105994 | 12453.7537167059 | Equatorial Guinea |
| 2002 | 10542.7554784403 | 0.451938999588547 | 409.159082639553 | 10133.5963958007 | Equatorial Guinea |
| 2003 | 8690.49725703626 | 0.467846832610047 | 357.00109160895 | 8333.49616542731 | Equatorial Guinea |
| 2004 | 7318.23105011398 | 0.483879611699357 | 326.142645900193 | 6992.08840421379 | Equatorial Guinea |
| 2005 | 7189.78003117092 | 0.498683715051723 | 238.31993795786 | 6951.46009321306 | Equatorial Guinea |
| 2006 | 5358.87818068014 | 0.511579828259094 | 212.698955196772 | 5146.17922548337 | Equatorial Guinea |
| 2007 | 4519.61012580092 | 0.524076461332969 | 189.507658210164 | 4330.10246759076 | Equatorial Guinea |
| 2008 | 3866.20731077363 | 0.537018989870245 | 157.067927660285 | 3709.13938311334 | Equatorial Guinea |
| 2009 | 3454.83672201675 | 0.548514682896356 | 149.492866541159 | 3305.34385547559 | Equatorial Guinea |
| 2010 | 3016.01384298183 | 0.55910937700551 | 141.664583212201 | 2874.34925976963 | Equatorial Guinea |
| 2011 | 2595.45547679825 | 0.570277424014124 | 89.9468092065992 | 2505.50866759165 | Equatorial Guinea |
| 2012 | 2097.48253871008 | 0.581833899261353 | 83.6165944238619 | 2013.86594428622 | Equatorial Guinea |
| 2013 | 1692.68874571124 | 0.592800613077549 | 76.1055951850297 | 1616.58315052621 | Equatorial Guinea |
| 2014 | 1471.90524237248 | 0.60347316716397 | 74.7623933677257 | 1397.14284900475 | Equatorial Guinea |
| 2015 | 1282.13209032676 | 0.61313462386841 | 68.7382602421779 | 1213.39383008458 | Equatorial Guinea |
| 2016 | 1211.82717450182 | 0.621858156591175 | 62.9164035850608 | 1148.91077091676 | Equatorial Guinea |
| 2017 | 1194.53984189657 | 0.630234538276623 | 55.423512260583 | 1139.11632963599 | Equatorial Guinea |
| 2018 | 1165.35726742555 | 0.638022822887303 | 50.7021607900172 | 1114.65510663553 | Equatorial Guinea |
| 2019 | 1131.25290880152 | 0.645303092753852 | 49.6488277484967 | 1081.60408105302 | Equatorial Guinea |
| 2020 | 1052.46534608799 | 0.652124902941809 | 45.5134672523154 | 1006.95187883567 | Equatorial Guinea |
| 2021 | 998.11839344727 | 0.657857455522296 | 45.0300296482643 | 953.088363799006 | Equatorial Guinea |
| 1990 | 12870.1122150901 | 0.455421186671872 | 398.446748352388 | 12471.6654667377 | Gabon |
| 1991 | 12083.2337493263 | 0.46156309767229 | 381.826375236813 | 11701.4073740895 | Gabon |
| 1992 | 11424.2486391284 | 0.467024048058252 | 357.102288985441 | 11067.146350143 | Gabon |
| 1993 | 10804.5915906171 | 0.472394463 | 343.716702463924 | 10460.8748881532 | Gabon |
| 1994 | 10160.8773042273 | 0.47806194683023 | 335.838692885754 | 9825.03861134155 | Gabon |
| 1995 | 9506.13833652967 | 0.483826218373876 | 326.098210134724 | 9180.04012639495 | Gabon |
| 1996 | 8885.84240368227 | 0.48946145974564 | 309.91415402262 | 8575.92824965965 | Gabon |
| 1997 | 8309.28444210942 | 0.495074161867882 | 281.300393571181 | 8027.98404853824 | Gabon |
| 1998 | 7848.36788729914 | 0.500372549878702 | 229.458556177942 | 7618.9093311212 | Gabon |
| 1999 | 7471.67571974215 | 0.504806350324196 | 222.733538248149 | 7248.942181 | Gabon |
| 2000 | 7188.2957556467 | 0.509133087403875 | 214.134644220437 | 6974.16111142626 | Gabon |
| 2001 | 6724.18966424705 | 0.513045709061385 | 206.090230308567 | 6518.09943393848 | Gabon |
| 2002 | 6319.22366321164 | 0.516917620976304 | 204.325258806093 | 6114.89840440555 | Gabon |
| 2003 | 5940.97214428333 | 0.52105141238927 | 199.166043554907 | 5741.80610072842 | Gabon |
| 2004 | 5675.80653262192 | 0.525479071446482 | 188.168295103487 | 5487.63823751843 | Gabon |
| 2005 | 5631.87100583887 | 0.530488574568447 | 178.676444995926 | 5453.19456084294 | Gabon |
| 2006 | 5273.21365771639 | 0.534797715554935 | 161.324099537583 | 5111.88955817881 | Gabon |
| 2007 | 4913.7379160774 | 0.539441424484129 | 155.885949301611 | 4757.85196677579 | Gabon |
| 2008 | 4473.11401094001 | 0.544110892340982 | 155.089834970445 | 4318.02417596957 | Gabon |
| 2009 | 4056.63055660515 | 0.548591318536338 | 148.70562668427 | 3907.92492992088 | Gabon |
| 2010 | 3597.25599972051 | 0.554022705553671 | 143.032317160667 | 3454.22368255984 | Gabon |
| 2011 | 3194.19847535361 | 0.560232082065429 | 138.419990428724 | 3055.77848492489 | Gabon |
| 2012 | 2807.9660201338 | 0.566842963853275 | 113.647694128892 | 2694.31832600491 | Gabon |
| 2013 | 2629.87624502452 | 0.573808130133551 | 86.092077706362 | 2543.78416731816 | Gabon |
| 2014 | 2466.62293769322 | 0.580915656182647 | 84.7334560861113 | 2381.88948160711 | Gabon |
| 2015 | 2290.60180069506 | 0.58836499960774 | 78.6853947483143 | 2211.91640594675 | Gabon |
| 2016 | 2093.64673488529 | 0.596062304964046 | 77.1376810166349 | 2016.50905386865 | Gabon |
| 2017 | 1967.18792432698 | 0.603816251524064 | 75.6617383475188 | 1891.52618597946 | Gabon |
| 2018 | 1822.57196506583 | 0.611724379630504 | 69.8261787581718 | 1752.74578630766 | Gabon |
| 2019 | 1647.31010416304 | 0.619904726884927 | 63.9844586483716 | 1583.32564551467 | Gabon |
| 2020 | 1436.66103873269 | 0.627609647280361 | 59.3633851666764 | 1377.29765356601 | Gabon |
| 2021 | 1287.12599380351 | 0.634691392755259 | 53.6821163684643 | 1233.44387743505 | Gabon |
| 1990 | 14120.1173354398 | 0.205867359800096 | 6647.52485770364 | 7472.59247773616 | Burundi |
| 1991 | 13225.884204626 | 0.208495720433731 | 6118.11874007449 | 7107.76546455151 | Burundi |
| 1992 | 13204.3318406863 | 0.210864619064193 | 6012.46261497702 | 7191.86922570928 | Burundi |
| 1993 | 12710.0005370896 | 0.213249386007653 | 5629.42924863727 | 7080.57128845233 | Burundi |
| 1994 | 12351.3458927769 | 0.215222973163092 | 5591.16937609443 | 6760.17651668247 | Burundi |
| 1995 | 11237.3109868002 | 0.215631675834044 | 5650.31033743238 | 5587.00064936782 | Burundi |
| 1996 | 10419.6551513751 | 0.215526325356923 | 5669.8292263631 | 4749.825925 | Burundi |
| 1997 | 10439.738118072 | 0.215761819839635 | 5556.67432072824 | 4883.06379734376 | Burundi |
| 1998 | 10003.0395063152 | 0.216402603342113 | 5582.25995580682 | 4420.77955050838 | Burundi |
| 1999 | 9432.26196455486 | 0.216855766644471 | 5649.00581229061 | 3783.25615226425 | Burundi |
| 2000 | 9082.8631505447 | 0.21686985832205 | 5639.92712064259 | 3442.93602990211 | Burundi |
| 2001 | 8940.66113666049 | 0.216988725649785 | 5584.14006019895 | 3356.52107646154 | Burundi |
| 2002 | 9185.8899812708 | 0.217782476516408 | 5616.9202437826 | 3568.9697374882 | Burundi |
| 2003 | 9180.64922807189 | 0.21896153733539 | 5102.82874035518 | 4077.82048771671 | Burundi |
| 2004 | 9431.090166 | 0.220918708918727 | 5080.05221364106 | 4351.03795215994 | Burundi |
| 2005 | 9436.36447106687 | 0.223084371742497 | 5068.66526430552 | 4367.69920676135 | Burundi |
| 2006 | 9222.26308250165 | 0.225821889923957 | 4539.77244499217 | 4682.49063750948 | Burundi |
| 2007 | 9205.86526053031 | 0.228996818715961 | 4464.00326069507 | 4741.86199983525 | Burundi |
| 2008 | 8934.0627485855 | 0.232853551450426 | 3907.00034943703 | 5027.06239914847 | Burundi |
| 2009 | 8524.2932931388 | 0.236943039904587 | 3539.69580483865 | 4984.59748830015 | Burundi |
| 2010 | 8008.22008502833 | 0.241447785964046 | 3593.93409678924 | 4414.28598823909 | Burundi |
| 2011 | 7539.05216936068 | 0.246277277175607 | 3379.73307440406 | 4159.31909495662 | Burundi |
| 2012 | 6671.58599570787 | 0.251572302738587 | 3014.67468742557 | 3656.9113082823 | Burundi |
| 2013 | 5152.87478454746 | 0.257191355215958 | 2731.51918160638 | 2421.35560294108 | Burundi |
| 2014 | 4473.44810793278 | 0.262794721997486 | 2457.8698315248 | 2015.57827640798 | Burundi |
| 2015 | 3935.48429396995 | 0.267327938241439 | 2508.79862455282 | 1426.68566941713 | Burundi |
| 2016 | 3534.07599174844 | 0.271587851896567 | 2177.80897019375 | 1356.26702155469 | Burundi |
| 2017 | 3217.92040378837 | 0.275840504541502 | 2109.26710408458 | 1108.65329970379 | Burundi |
| 2018 | 2891.88930824142 | 0.279733289674752 | 2166.28721788123 | 725.602090360191 | Burundi |
| 2019 | 2672.26951655241 | 0.283442514974853 | 2124.80213019591 | 547.467386356501 | Burundi |
| 2020 | 2535.73548607276 | 0.286496000475545 | 2133.42508178406 | 402.310404288698 | Burundi |
| 2021 | 2344.62939412907 | 0.289374364823265 | 2092.59795177415 | 252.031442354923 | Burundi |
| 1990 | 10035.505173791 | 0.270048115983623 | 2127.16557613974 | 7908.33959765126 | Comoros |
| 1991 | 9518.42384505959 | 0.279145649193181 | 2171.796079 | 7346.62776626159 | Comoros |
| 1992 | 8901.12904613252 | 0.288506271423925 | 2179.60410676186 | 6721.52493937066 | Comoros |
| 1993 | 8293.93607209055 | 0.297607416571855 | 2152.69000895031 | 6141.24606314024 | Comoros |
| 1994 | 7758.27987685893 | 0.305706587428224 | 2098.6202424092 | 5659.65963444973 | Comoros |
| 1995 | 7135.41764848661 | 0.314220340550933 | 2105.23988686645 | 5030.17776162016 | Comoros |
| 1996 | 6647.64856724882 | 0.322249069327522 | 2088.1935639801 | 4559.45500326872 | Comoros |
| 1997 | 6382.78550159036 | 0.330148124863673 | 1986.3586689269 | 4396.42683266346 | Comoros |
| 1998 | 7634.33615589583 | 0.33751791405714 | 1845.31917313088 | 5789.01698276495 | Comoros |
| 1999 | 6401.9981611056 | 0.34477469444905 | 1773.85843051009 | 4628.13973059551 | Comoros |
| 2000 | 9000.98217253416 | 0.351875505604714 | 1762.59148113407 | 7238.39069140009 | Comoros |
| 2001 | 5621.88259319767 | 0.359211999278107 | 1651.09226832917 | 3970.7903248685 | Comoros |
| 2002 | 6953.8916661542 | 0.366486126823935 | 1270.58674792866 | 5683.30491822554 | Comoros |
| 2003 | 5074.80014394059 | 0.373410566246042 | 1214.31486983212 | 3860.48527410847 | Comoros |
| 2004 | 4836.91284068246 | 0.380035976109197 | 1134.07957977815 | 3702.83326090431 | Comoros |
| 2005 | 4685.44439925095 | 0.386891844663173 | 1088.28403512342 | 3597.16036412753 | Comoros |
| 2006 | 4578.77361653855 | 0.393521434414153 | 973.828792749011 | 3604.94482378954 | Comoros |
| 2007 | 5715.98786545986 | 0.399051094324319 | 934.441855705959 | 4781.5460097539 | Comoros |
| 2008 | 4534.54607685877 | 0.404649755421042 | 883.483426804365 | 3651.0626500544 | Comoros |
| 2009 | 4140.57479668194 | 0.410253575216193 | 872.704333462578 | 3267.87046321936 | Comoros |
| 2010 | 3656.68437253867 | 0.415543376261308 | 793.841448770804 | 2862.84292376787 | Comoros |
| 2011 | 3467.22796359635 | 0.421092971866532 | 772.179267548891 | 2695.04869604746 | Comoros |
| 2012 | 3101.66643031749 | 0.42672995742339 | 554.64692908737 | 2547.01950123012 | Comoros |
| 2013 | 2783.93405986105 | 0.432789931825945 | 431.225812845398 | 2352.70824701565 | Comoros |
| 2014 | 2836.48667603066 | 0.438844505062188 | 421.827631889316 | 2414.65904414134 | Comoros |
| 2015 | 2625.09209891695 | 0.444712393572096 | 423.933782929308 | 2201.15831598764 | Comoros |
| 2016 | 2401.10028759292 | 0.450506978607507 | 409.921862588812 | 1991.17842500411 | Comoros |
| 2017 | 2278.86003079279 | 0.456280841429385 | 397.016628831484 | 1881.84340196131 | Comoros |
| 2018 | 2098.27777841368 | 0.461951343654461 | 379.926018797783 | 1718.3517596159 | Comoros |
| 2019 | 1952.99359393384 | 0.46738801351741 | 360.764845614273 | 1592.22874831957 | Comoros |
| 2020 | 1917.39667583566 | 0.472003132433584 | 340.898380193361 | 1576.4982956423 | Comoros |
| 2021 | 1824.92131629349 | 0.475978688247514 | 334.2245788 | 1490.69673744709 | Comoros |
| 1990 | 16162.385050479 | 0.337781788993477 | 1862.37902764371 | 14300.0060228353 | Djibouti |
| 1991 | 14471.8872098367 | 0.338770010818962 | 1874.82806736506 | 12597.0591424716 | Djibouti |
| 1992 | 14376.1468738284 | 0.340986061300938 | 1852.77323183066 | 12523.3736419977 | Djibouti |
| 1993 | 19118.8785440335 | 0.344904393563471 | 1774.373896 | 17344.5046479815 | Djibouti |
| 1994 | 15047.8770302466 | 0.34798140392569 | 1780.38693102194 | 13267.4900992247 | Djibouti |
| 1995 | 14059.569480487 | 0.351245633337663 | 1758.1515470512 | 12301.4179334358 | Djibouti |
| 1996 | 13266.7126503292 | 0.354385634187117 | 1761.82700904677 | 11504.8856412824 | Djibouti |
| 1997 | 13513.8939621434 | 0.35809640096391 | 1647.23538849748 | 11866.6585736459 | Djibouti |
| 1998 | 11553.7851822765 | 0.361596763593584 | 1444.49615996029 | 10109.2890223162 | Djibouti |
| 1999 | 10487.5860668005 | 0.365305211425089 | 1293.87738240054 | 9193.70868439996 | Djibouti |
| 2000 | 9928.18662556067 | 0.369015094624057 | 1242.59134159025 | 8685.59528397042 | Djibouti |
| 2001 | 9174.67758419075 | 0.372853155297047 | 1214.70224518949 | 7959.97533900126 | Djibouti |
| 2002 | 8428.80484441067 | 0.37665000401959 | 1178.33748919567 | 7250.467355 | Djibouti |
| 2003 | 7992.38352779006 | 0.380686615222992 | 1134.23924333569 | 6858.14428445437 | Djibouti |
| 2004 | 7703.7736339058 | 0.384987705849076 | 1087.96741280609 | 6615.80622109971 | Djibouti |
| 2005 | 7030.16462302036 | 0.389568705689455 | 1033.3966718082 | 5996.76795121216 | Djibouti |
| 2006 | 6847.17546369595 | 0.395037470761535 | 982.740648762489 | 5864.43481493346 | Djibouti |
| 2007 | 6493.9150344609 | 0.40061854555971 | 934.926456584732 | 5558.98857787617 | Djibouti |
| 2008 | 5959.73445769171 | 0.406658303036044 | 866.426558897682 | 5093.30789879403 | Djibouti |
| 2009 | 5636.64307669846 | 0.411969334510517 | 855.056231384024 | 4781.58684531444 | Djibouti |
| 2010 | 5830.43782377066 | 0.416999349607211 | 794.834564326286 | 5035.60325944437 | Djibouti |
| 2011 | 4767.50421006195 | 0.422316140090975 | 694.087344741525 | 4073.41686532043 | Djibouti |
| 2012 | 4192.97580863082 | 0.427711440066409 | 508.220455120109 | 3684.75535351071 | Djibouti |
| 2013 | 3721.52222209461 | 0.43336043651961 | 417.078985709702 | 3304.44323638491 | Djibouti |
| 2014 | 2953.17234685336 | 0.439455721647865 | 416.225220201963 | 2536.9471266514 | Djibouti |
| 2015 | 2633.12083879725 | 0.445855284807829 | 412.418564661264 | 2220.70227413599 | Djibouti |
| 2016 | 2437.98288239086 | 0.453442853845541 | 410.671569427308 | 2027.31131296355 | Djibouti |
| 2017 | 2156.61149795267 | 0.460440151860865 | 399.389879388569 | 1757.2216185641 | Djibouti |
| 2018 | 1867.57883777236 | 0.46770567196373 | 363.356374325554 | 1504.22246344681 | Djibouti |
| 2019 | 1664.62957901828 | 0.475263242846912 | 338.334399536627 | 1326.29517948165 | Djibouti |
| 2020 | 1504.66930782883 | 0.481879945321652 | 325.451682933585 | 1179.21762489524 | Djibouti |
| 2021 | 1258.81894202433 | 0.4879583707169 | 312.639189213073 | 946.179752811257 | Djibouti |
| 1990 | 24218.8534775555 | 0.216028238900354 | 5585.56220023468 | 18633.2912773208 | Eritrea |
| 1991 | 23547.3828196625 | 0.222574737210924 | 5007.57982473244 | 18539.8029949301 | Eritrea |
| 1992 | 23803.2826682401 | 0.230296065775865 | 3938.13038211883 | 19865.1522861213 | Eritrea |
| 1993 | 22421.6595454352 | 0.239385041260314 | 3696.13147538928 | 18725.5280700459 | Eritrea |
| 1994 | 19928.0498524934 | 0.250752541052101 | 3025.43478201321 | 16902.6150704802 | Eritrea |
| 1995 | 17979.5921008573 | 0.261498484754305 | 2679.15590973292 | 15300.4361911244 | Eritrea |
| 1996 | 16165.2806721772 | 0.272527054525239 | 2149.96740970628 | 14015.3132624709 | Eritrea |
| 1997 | 14866.8021873932 | 0.283551720614883 | 2170.34674022828 | 12696.4554471649 | Eritrea |
| 1998 | 13905.0978479511 | 0.2937648278699 | 2152.60788752141 | 11752.4899604297 | Eritrea |
| 1999 | 13192.7960400898 | 0.303142339840286 | 2112.44729495354 | 11080.3487451363 | Eritrea |
| 2000 | 12571.51794 | 0.310248161765321 | 2125.6658559435 | 10445.8520875465 | Eritrea |
| 2001 | 12364.3738641553 | 0.317247987543295 | 2069.57001692727 | 10294.803847228 | Eritrea |
| 2002 | 11900.0638797316 | 0.323566584840077 | 2045.96037585274 | 9854.10350387886 | Eritrea |
| 2003 | 11031.3008293568 | 0.328593580738491 | 2052.71252473886 | 8978.58830461794 | Eritrea |
| 2004 | 10654.0284293816 | 0.332879380249727 | 1987.57199222661 | 8666.45643715499 | Eritrea |
| 2005 | 10052.6754206666 | 0.336682510811316 | 1922.83572375242 | 8129.83969691418 | Eritrea |
| 2006 | 9508.62827502171 | 0.339835823599651 | 1856.00474357943 | 7652.62353144228 | Eritrea |
| 2007 | 9058.18251314123 | 0.342843174407113 | 1776.28281131867 | 7281.89970182256 | Eritrea |
| 2008 | 8630.0713708576 | 0.343427800730465 | 1777.1143515115 | 6852.9570193461 | Eritrea |
| 2009 | 7870.62605752126 | 0.345147742630626 | 1766.12183510907 | 6104.50422241219 | Eritrea |
| 2010 | 7380.25562201294 | 0.348146176900867 | 1758.27848432731 | 5621.97713768563 | Eritrea |
| 2011 | 6542.71759999813 | 0.35416314653227 | 1761.91736825701 | 4780.80023174112 | Eritrea |
| 2012 | 5543.84208745525 | 0.360112751410199 | 1548.67737889554 | 3995.16470855971 | Eritrea |
| 2013 | 4759.93266642355 | 0.364343653311207 | 1299.93039092518 | 3460.00227549837 | Eritrea |
| 2014 | 3531.56327645138 | 0.372527587512835 | 1221.04892004149 | 2310.51435640989 | Eritrea |
| 2015 | 3149.43919585053 | 0.376935162282393 | 1169.0929838352 | 1980.34621201533 | Eritrea |
| 2016 | 2991.73148759464 | 0.382171665821118 | 1136.27586302403 | 1855.45562457061 | Eritrea |
| 2017 | 2790.94447499659 | 0.385936029208135 | 1085.66947812315 | 1705.27499687344 | Eritrea |
| 2018 | 2592.71548361093 | 0.39115488426675 | 1031.24968694204 | 1561.46579666889 | Eritrea |
| 2019 | 2450.16912624081 | 0.396330558249013 | 979.654561816359 | 1470.51456442445 | Eritrea |
| 2020 | 2291.27021024275 | 0.400459411382762 | 922.440874411777 | 1368.82933583097 | Eritrea |
| 2021 | 2111.65194665391 | 0.403863942828898 | 876.885592160061 | 1234.76635449385 | Eritrea |
| 1990 | 16064.8349314502 | 0.148033884957629 | 8732.93070545208 | 7331.90422599812 | Ethiopia |
| 1991 | 15605.7078217763 | 0.149599710414418 | 8819.80229749492 | 6785.90552428138 | Ethiopia |
| 1992 | 15272.7354679289 | 0.15017438640674 | 8845.53870588579 | 6427.19676204312 | Ethiopia |
| 1993 | 14907.4863865821 | 0.151582364387045 | 8754.23321310175 | 6153.25317348035 | Ethiopia |
| 1994 | 14523.2517318487 | 0.152982636940145 | 8846.3803023752 | 5676.8714294735 | Ethiopia |
| 1995 | 13857.3440907193 | 0.155085604929282 | 8785.5100848795 | 5071.8340058398 | Ethiopia |
| 1996 | 13076.9894750242 | 0.158127229482336 | 8750.55991537589 | 4326.42955964831 | Ethiopia |
| 1997 | 12323.4858656124 | 0.161874742949531 | 8839.79964219069 | 3483.68622342171 | Ethiopia |
| 1998 | 11595.1963704431 | 0.16507771008878 | 8783.42557569689 | 2811.77079474621 | Ethiopia |
| 1999 | 10827.0550635945 | 0.168903545355995 | 8695.50383879404 | 2131.55122480046 | Ethiopia |
| 2000 | 10128.5355623225 | 0.173418529946986 | 8756.49451620192 | 1372.04104612058 | Ethiopia |
| 2001 | 9408.32788000633 | 0.178972968614763 | 8733.37568663035 | 674.952193375979 | Ethiopia |
| 2002 | 8706.99140638453 | 0.184138012848519 | 8576.17984497469 | 130.811561409844 | Ethiopia |
| 2003 | 7916.65988484757 | 0.188694814030626 | 7916.65988484757 | 0 | Ethiopia |
| 2004 | 7183.95970269965 | 0.195030701395242 | 7183.95970269965 | 0 | Ethiopia |
| 2005 | 6790.6440727215 | 0.202868242862353 | 6543.33821615863 | 247.305856562868 | Ethiopia |
| 2006 | 6411.19507305234 | 0.211524228741913 | 6025.04517619763 | 386.149896854706 | Ethiopia |
| 2007 | 6034.07447960489 | 0.221819930759394 | 5055.34556628672 | 978.728913318166 | Ethiopia |
| 2008 | 5625.42785864003 | 0.232872185647818 | 3778.31203756292 | 1847.11582107711 | Ethiopia |
| 2009 | 5254.53100890607 | 0.244265818456334 | 3311.55649617492 | 1942.97451273115 | Ethiopia |
| 2010 | 4700.02994726934 | 0.256081459859031 | 3019.46802921954 | 1680.56191804979 | Ethiopia |
| 2011 | 4410.51872778455 | 0.267880862080398 | 2475.93150208755 | 1934.587226 | Ethiopia |
| 2012 | 3980.089462 | 0.279006957893266 | 2144.27390239165 | 1835.81556005835 | Ethiopia |
| 2013 | 3533.90501497402 | 0.289885266889051 | 2147.08817754072 | 1386.8168374333 | Ethiopia |
| 2014 | 3249.79266740717 | 0.300484547961246 | 2157.5536870379 | 1092.23898036927 | Ethiopia |
| 2015 | 2977.70710353992 | 0.310736418709888 | 2148.39863299036 | 829.308470549563 | Ethiopia |
| 2016 | 2773.65340697348 | 0.320431867242816 | 2080.95517814448 | 692.6982288 | Ethiopia |
| 2017 | 2702.32491623838 | 0.329469243719685 | 2067.47146822262 | 634.853448015762 | Ethiopia |
| 2018 | 2591.88668305457 | 0.338050074820716 | 1858.54430027359 | 733.342382780978 | Ethiopia |
| 2019 | 2431.28300061673 | 0.346422663706934 | 1778.83881863797 | 652.444181978756 | Ethiopia |
| 2020 | 2202.74987771476 | 0.353216349837522 | 1753.97190492299 | 448.777972791766 | Ethiopia |
| 2021 | 1995.06832009289 | 0.358823295416803 | 1649.38832994405 | 345.679990148836 | Ethiopia |
| 1990 | 13065.5476739797 | 0.333850293102656 | 1988.35270962862 | 11077.1949643511 | Kenya |
| 1991 | 12587.1618811684 | 0.340476220279675 | 1847.23526082292 | 10739.9266203455 | Kenya |
| 1992 | 12282.3431551231 | 0.346298636491363 | 1775.12598716007 | 10507.217167963 | Kenya |
| 1993 | 11925.9683416031 | 0.351611769443661 | 1766.03390686509 | 10159.934434738 | Kenya |
| 1994 | 11616.3960544822 | 0.356779193882903 | 1703.6546154093 | 9912.7414390729 | Kenya |
| 1995 | 11277.1251750192 | 0.361535806569139 | 1415.4870390509 | 9861.6381359683 | Kenya |
| 1996 | 10860.3026898593 | 0.366203076018008 | 1309.58747746008 | 9550.71521239922 | Kenya |
| 1997 | 10805.7386480836 | 0.370436464683744 | 1222.92523474204 | 9582.81341334156 | Kenya |
| 1998 | 10612.4714031338 | 0.374581400693507 | 1174.48939560123 | 9437.98200753258 | Kenya |
| 1999 | 10176.4919222421 | 0.378630009408301 | 1132.59354302572 | 9043.89837921638 | Kenya |
| 2000 | 9424.69099571662 | 0.382395616264527 | 1120.64015473927 | 8304.05084097736 | Kenya |
| 2001 | 9042.70976206821 | 0.386188414766973 | 1082.26811708769 | 7960.44164498052 | Kenya |
| 2002 | 9002.14681884223 | 0.389886256007927 | 1042.4236276758 | 7959.72319116643 | Kenya |
| 2003 | 9190.74444730417 | 0.393680479758456 | 986.73105272509 | 8204.01339457908 | Kenya |
| 2004 | 9107.20434173475 | 0.397743651275382 | 967.85285591371 | 8139.35148582104 | Kenya |
| 2005 | 9024.23505479902 | 0.402444011242538 | 877.325150557696 | 8146.90990424132 | Kenya |
| 2006 | 8785.19186563577 | 0.407745764308496 | 870.799891570613 | 7914.39197406516 | Kenya |
| 2007 | 8682.35926969262 | 0.413736227073594 | 852.778992941589 | 7829.58027675103 | Kenya |
| 2008 | 8244.79315535444 | 0.419738146655789 | 775.917429606179 | 7468.87572574826 | Kenya |
| 2009 | 8028.51986710869 | 0.426123888497405 | 564.987917972102 | 7463.53194913659 | Kenya |
| 2010 | 7777.87242360557 | 0.433305839416248 | 424.345222426713 | 7353.52720117886 | Kenya |
| 2011 | 7210.42168348879 | 0.440893830428021 | 427.127227796435 | 6783.29445569236 | Kenya |
| 2012 | 6365.58719606975 | 0.448666395692026 | 413.110826042698 | 5952.47637002705 | Kenya |
| 2013 | 5516.70197253597 | 0.456666941870395 | 396.82303928183 | 5119.87893325414 | Kenya |
| 2014 | 4487.59582791726 | 0.464724302152848 | 381.066892247475 | 4106.52893566978 | Kenya |
| 2015 | 3943.87638807902 | 0.473084051301145 | 338.6960116 | 3605.18037646442 | Kenya |
| 2016 | 3548.31422262676 | 0.481709072334755 | 324.7902007 | 3223.52402195736 | Kenya |
| 2017 | 3369.21340917957 | 0.490296619363422 | 310.705545643722 | 3058.50786353585 | Kenya |
| 2018 | 3067.31082621132 | 0.499101274473837 | 236.593723558903 | 2830.71710265242 | Kenya |
| 2019 | 2887.20715986925 | 0.508004210679317 | 219.603031027934 | 2667.60412884132 | Kenya |
| 2020 | 2701.52020616466 | 0.516136571818187 | 204.023384854145 | 2497.49682131052 | Kenya |
| 2021 | 2507.2554906743 | 0.523768077100117 | 190.014721254488 | 2317.24076941981 | Kenya |
| 1990 | 32337.1494705969 | 0.279889464787504 | 2153.17512393445 | 30183.9743466625 | Madagascar |
| 1991 | 31246.3564378867 | 0.280429588849502 | 2129.73517308542 | 29116.6212648013 | Madagascar |
| 1992 | 30314.6109991226 | 0.280501282838033 | 2138.11964712633 | 28176.4913519963 | Madagascar |
| 1993 | 29481.3972857277 | 0.280204299153331 | 2150.34393456976 | 27331.0533511579 | Madagascar |
| 1994 | 28693.8930721752 | 0.279615149051304 | 2154.14625417659 | 26539.7468179986 | Madagascar |
| 1995 | 27880.1655767042 | 0.279164003276889 | 2090.79041810619 | 25789.375158598 | Madagascar |
| 1996 | 27636.020236087 | 0.279066492612967 | 2175.0198381985 | 25461.0003978885 | Madagascar |
| 1997 | 28127.8913272686 | 0.279426189949827 | 2151.92829719559 | 25975.963030073 | Madagascar |
| 1998 | 28407.5813560508 | 0.280317670990881 | 2133.41607691484 | 26274.165279136 | Madagascar |
| 1999 | 29846.3808381271 | 0.281571072098772 | 2187.30770921923 | 27659.0731289079 | Madagascar |
| 2000 | 28239.4519042746 | 0.283190720794041 | 2165.42969748098 | 26074.0222067936 | Madagascar |
| 2001 | 27422.994717206 | 0.285668180077333 | 2159.43568604297 | 25263.559031163 | Madagascar |
| 2002 | 25979.8119270148 | 0.287153148772741 | 2146.89995625291 | 23832.9119707619 | Madagascar |
| 2003 | 25092.4217591856 | 0.289675457328935 | 2164.43907798898 | 22927.9826811966 | Madagascar |
| 2004 | 24335.1511924185 | 0.292629884521451 | 2104.56155732636 | 22230.5896350921 | Madagascar |
| 2005 | 25494.2604524269 | 0.295919326698766 | 2105.47608684299 | 23388.7843655839 | Madagascar |
| 2006 | 25206.8707531562 | 0.299723392356893 | 2157.00374677444 | 23049.8670063818 | Madagascar |
| 2007 | 25495.6124866879 | 0.304026244970223 | 2153.81796692364 | 23341.7945197643 | Madagascar |
| 2008 | 26077.8152392743 | 0.308990264960967 | 2121.58040971633 | 23956.234829558 | Madagascar |
| 2009 | 26143.4407420433 | 0.313450891076854 | 2121.92573891709 | 24021.5150031262 | Madagascar |
| 2010 | 24928.3478917198 | 0.318353184360276 | 2090.46163134183 | 22837.886260378 | Madagascar |
| 2011 | 24455.7190211141 | 0.323801042883395 | 2051.61096608169 | 22404.1080550324 | Madagascar |
| 2012 | 22916.7419666666 | 0.329947540970422 | 2022.87590005769 | 20893.8660666089 | Madagascar |
| 2013 | 20630.3235356292 | 0.336519231511991 | 1915.54442472916 | 18714.77911 | Madagascar |
| 2014 | 18231.9740693806 | 0.343352610546557 | 1765.65112981796 | 16466.3229395626 | Madagascar |
| 2015 | 16807.6921207084 | 0.350443595243704 | 1757.88183402431 | 15049.8102866841 | Madagascar |
| 2016 | 15780.3016924689 | 0.35802634126176 | 1648.74618061558 | 14131.5555118533 | Madagascar |
| 2017 | 14731.8464290089 | 0.36623558721941 | 1292.80536976226 | 13439.0410592466 | Madagascar |
| 2018 | 13613.1978930848 | 0.3749696444566 | 1169.86996030687 | 12443.3279327779 | Madagascar |
| 2019 | 12226.0129649624 | 0.384209195656002 | 1092.64495887223 | 11133.3680060902 | Madagascar |
| 2020 | 10745.2758680721 | 0.392778708989164 | 1029.22789449456 | 9716.04797357754 | Madagascar |
| 2021 | 9650.8920649426 | 0.40024694263784 | 928.330589819192 | 8722.56147512341 | Madagascar |
| 1990 | 38987.5728957273 | 0.204010244634822 | 6632.69269872448 | 32354.8801970028 | Malawi |
| 1991 | 37718.9066462783 | 0.206043785604482 | 6626.61433163486 | 31092.2923146434 | Malawi |
| 1992 | 35614.8192509691 | 0.206732358761786 | 6601.92595515336 | 29012.8932958157 | Malawi |
| 1993 | 34341.0310388157 | 0.20809120460636 | 6086.42659429682 | 28254.6044445189 | Malawi |
| 1994 | 31515.024717656 | 0.209144565934722 | 6118.99166317652 | 25396.0330544795 | Malawi |
| 1995 | 30165.3783531588 | 0.211756231927772 | 6023.6358475135 | 24141.7425056453 | Malawi |
| 1996 | 27688.4039647815 | 0.215264474919532 | 5566.09882988968 | 22122.3051348918 | Malawi |
| 1997 | 24908.9358179067 | 0.219416251726227 | 5054.1008106114 | 19854.8350072953 | Malawi |
| 1998 | 22718.0803746644 | 0.223122329682106 | 5013.58974345521 | 17704.4906312092 | Malawi |
| 1999 | 23232.0432447002 | 0.22706836834043 | 4458.37298863204 | 18773.6702560682 | Malawi |
| 2000 | 21054.3556673365 | 0.230900810051145 | 3910.42751409045 | 17143.9281532461 | Malawi |
| 2001 | 20719.747543968 | 0.234020506815413 | 3841.27621848615 | 16878.4713254818 | Malawi |
| 2002 | 18354.5240120563 | 0.237602155431587 | 3538.95621350542 | 14815.5677985509 | Malawi |
| 2003 | 16047.7298886396 | 0.241559227766521 | 3651.41970988687 | 12396.3101787527 | Malawi |
| 2004 | 14264.419981131 | 0.246434539760473 | 3364.37576844613 | 10900.0442126849 | Malawi |
| 2005 | 12922.1558329604 | 0.251672317552778 | 3016.46458630146 | 9905.69124665894 | Malawi |
| 2006 | 12240.2210958446 | 0.257595649191555 | 2790.78732204677 | 9449.43377379783 | Malawi |
| 2007 | 11197.832389181 | 0.265226927963354 | 2512.93106186039 | 8684.90132732061 | Malawi |
| 2008 | 10084.3729307305 | 0.273689856434299 | 2165.28549359122 | 7919.08743713928 | Malawi |
| 2009 | 9719.99499861848 | 0.282296508700983 | 2147.50851701264 | 7572.48648160584 | Malawi |
| 2010 | 9234.31984314286 | 0.291179619969706 | 2134.26595710154 | 7100.05388604132 | Malawi |
| 2011 | 8741.56961209722 | 0.300448509120473 | 2141.39318491326 | 6600.17642718396 | Malawi |
| 2012 | 7227.525083 | 0.30919762412021 | 2096.66235514608 | 5130.86272771492 | Malawi |
| 2013 | 6085.79991696023 | 0.317936213061346 | 2100.46748746921 | 3985.33242949102 | Malawi |
| 2014 | 5289.2992014967 | 0.327042425505685 | 2063.10871477896 | 3226.19048671774 | Malawi |
| 2015 | 4721.59882347715 | 0.33614013833142 | 1983.02249474883 | 2738.57632872832 | Malawi |
| 2016 | 4305.10440310593 | 0.344731541741046 | 1777.31789339131 | 2527.78650971462 | Malawi |
| 2017 | 3906.19914001087 | 0.353121067414073 | 1765.01882101292 | 2141.18031899795 | Malawi |
| 2018 | 3607.54218802286 | 0.361470410425433 | 1400.20988731813 | 2207.33230070473 | Malawi |
| 2019 | 3348.27630271668 | 0.37011460502829 | 1212.11359219635 | 2136.16271052033 | Malawi |
| 2020 | 3003.84515130812 | 0.377879020413869 | 1125.97592839709 | 1877.86922291103 | Malawi |
| 2021 | 2691.86768142192 | 0.384553633730301 | 1092.31814195656 | 1599.54953946537 | Malawi |
| 1990 | 1294.80559931737 | 0.544586533153919 | 155.268187650175 | 1139.5374116672 | Mauritius |
| 1991 | 910.70047332368 | 0.548209120513745 | 148.430581452001 | 762.269891871679 | Mauritius |
| 1992 | 858.76056256962 | 0.55345463047762 | 144.433278387411 | 714.327284182209 | Mauritius |
| 1993 | 745.5839709 | 0.560688639662726 | 139.350261618583 | 606.233709275117 | Mauritius |
| 1994 | 754.1572997 | 0.569092171296203 | 109.468217740215 | 644.689081995485 | Mauritius |
| 1995 | 710.72871296171 | 0.577319390278784 | 86.1811274357816 | 624.547585525928 | Mauritius |
| 1996 | 700.57611447469 | 0.584797060785555 | 85.3718325432809 | 615.204281931409 | Mauritius |
| 1997 | 570.10261384251 | 0.591497734589023 | 79.1423217350905 | 490.96029210742 | Mauritius |
| 1998 | 561.19553457269 | 0.597363039395481 | 80.325272660411 | 480.870261912279 | Mauritius |
| 1999 | 595.04950809762 | 0.602034233757503 | 75.7791029085445 | 519.270405189075 | Mauritius |
| 2000 | 567.02866941423 | 0.6070879010983 | 73.7225036871982 | 493.306165727032 | Mauritius |
| 2001 | 549.49431959106 | 0.612240817263126 | 69.3258837328655 | 480.168435858195 | Mauritius |
| 2002 | 536.03988305044 | 0.617190006807373 | 61.3068596089824 | 474.733023441458 | Mauritius |
| 2003 | 486.52901528119 | 0.622764532968127 | 63.9755617530025 | 422.553453528188 | Mauritius |
| 2004 | 513.17157307957 | 0.629030192646747 | 56.0103966546771 | 457.161176424893 | Mauritius |
| 2005 | 489.74820979901 | 0.634766579912803 | 53.8880224249773 | 435.860187374033 | Mauritius |
| 2006 | 545.8052587 | 0.639545182683383 | 51.6117274 | 494.19353126428 | Mauritius |
| 2007 | 586.32434573845 | 0.64435865560727 | 50.6846023615395 | 535.63974337691 | Mauritius |
| 2008 | 589.77655928799 | 0.649630852006361 | 47.4003905379741 | 542.376168750016 | Mauritius |
| 2009 | 565.60306357467 | 0.655000295441282 | 45.3010533021643 | 520.302010272506 | Mauritius |
| 2010 | 499.30497312061 | 0.660469800171028 | 43.8236670687758 | 455.481306051834 | Mauritius |
| 2011 | 471.79443556117 | 0.66649762677449 | 39.1643318536801 | 432.63010370749 | Mauritius |
| 2012 | 416.6758158 | 0.673127650407144 | 37.7900551958591 | 378.885760568641 | Mauritius |
| 2013 | 336.56543178263 | 0.680114023433391 | 37.4912210602707 | 299.074210722359 | Mauritius |
| 2014 | 259.56798023408 | 0.686616503167309 | 37.4924867002544 | 222.075493533826 | Mauritius |
| 2015 | 226.89502465483 | 0.692239729639954 | 36.3987920228355 | 190.496232631994 | Mauritius |
| 2016 | 211.1380684 | 0.697020562490311 | 35.5336187452796 | 175.60444961382 | Mauritius |
| 2017 | 200.21137666108 | 0.70161813647985 | 35.3900880259104 | 164.82128863517 | Mauritius |
| 2018 | 203.92482330446 | 0.706266650972885 | 34.8772054550564 | 169.047617849404 | Mauritius |
| 2019 | 185.99871785506 | 0.710935044634248 | 31.5996718850436 | 154.399045970016 | Mauritius |
| 2020 | 206.74874029915 | 0.714629803881546 | 31.8198813374752 | 174.928858961675 | Mauritius |
| 2021 | 148.74849698283 | 0.718260445552653 | 28.6134580418677 | 120.135038940962 | Mauritius |
| 1990 | 28988.6895308193 | 0.173064715394479 | 8678.26572818224 | 20310.4238026371 | Mozambique |
| 1991 | 26035.0251953366 | 0.175550435322203 | 8769.07104216249 | 17265.9541531741 | Mozambique |
| 1992 | 24933.5388575267 | 0.17728518828037 | 8692.83077460478 | 16240.7080829219 | Mozambique |
| 1993 | 23657.6012093295 | 0.179851807348523 | 8673.6676231242 | 14983.9335862053 | Mozambique |
| 1994 | 21698.8394879315 | 0.182236860582392 | 8698.96722576189 | 12999.8722621696 | Mozambique |
| 1995 | 20577.3678505517 | 0.183427981878199 | 8663.30391410614 | 11914.0639364456 | Mozambique |
| 1996 | 19806.7286442908 | 0.185705869370502 | 8600.14880722958 | 11206.5798370612 | Mozambique |
| 1997 | 19495.7428028866 | 0.188650435754761 | 8401.44261325491 | 11094.3001896317 | Mozambique |
| 1998 | 19032.4623030011 | 0.191998629373624 | 7963.3179667582 | 11069.1443362429 | Mozambique |
| 1999 | 18884.1936061351 | 0.195435287798378 | 7419.27666957733 | 11464.9169365578 | Mozambique |
| 2000 | 16921.5976141094 | 0.198534396867784 | 7303.18250142815 | 9618.41511268125 | Mozambique |
| 2001 | 15648.6586351245 | 0.20224192395185 | 6724.73669800893 | 8923.92193711557 | Mozambique |
| 2002 | 14973.7846523909 | 0.206390059635981 | 6620.65527086661 | 8353.12938152429 | Mozambique |
| 2003 | 14505.6759923328 | 0.210534489558039 | 6059.45911119585 | 8446.21688113696 | Mozambique |
| 2004 | 13952.5424046185 | 0.214972457474336 | 5515.38914981948 | 8437.15325479902 | Mozambique |
| 2005 | 13119.536704655 | 0.219692372119565 | 5125.84396686039 | 7993.69273779461 | Mozambique |
| 2006 | 13222.7200858046 | 0.22475808709223 | 4528.78646160002 | 8693.93362420458 | Mozambique |
| 2007 | 12464.3211355212 | 0.230158226784202 | 3973.83863627734 | 8490.48249924386 | Mozambique |
| 2008 | 11896.2978370767 | 0.235790170219554 | 4012.95950020512 | 7883.33833687157 | Mozambique |
| 2009 | 11239.8144948443 | 0.241360656275235 | 3554.91290901977 | 7684.90158582453 | Mozambique |
| 2010 | 10127.2838999928 | 0.246751499854601 | 3340.36459033067 | 6786.91930966213 | Mozambique |
| 2011 | 8916.70023753779 | 0.252232471060626 | 2997.8833054773 | 5918.8169320605 | Mozambique |
| 2012 | 8196.34764632186 | 0.258315562328104 | 2671.22842503832 | 5525.11922128354 | Mozambique |
| 2013 | 7318.13879314248 | 0.265280462139272 | 2446.56977480577 | 4871.56901833671 | Mozambique |
| 2014 | 6604.63922319502 | 0.272892813606321 | 2137.99697167983 | 4466.64225151519 | Mozambique |
| 2015 | 5346.71680122529 | 0.2810326852532 | 2153.15554834437 | 3193.56125288092 | Mozambique |
| 2016 | 4627.09221801955 | 0.289152424860155 | 2145.54947529556 | 2481.54274272399 | Mozambique |
| 2017 | 3915.24008860316 | 0.297164772494457 | 2165.50150027825 | 1749.73858832491 | Mozambique |
| 2018 | 3421.65971274584 | 0.305226771938642 | 2140.19080401692 | 1281.46890872892 | Mozambique |
| 2019 | 3317.34467596607 | 0.313174508096083 | 2127.6710761665 | 1189.67359979957 | Mozambique |
| 2020 | 3071.19593968282 | 0.320291181995807 | 2106.31942730477 | 964.876512378055 | Mozambique |
| 2021 | 2699.94099741786 | 0.326462613636377 | 2042.88585418064 | 657.055143237223 | Mozambique |
| 1990 | 16275.8277556682 | 0.275097189589071 | 2100.04395420774 | 14175.7838014605 | Rwanda |
| 1991 | 16543.1151768475 | 0.276194698465909 | 2178.81500140753 | 14364.30018 | Rwanda |
| 1992 | 15805.3852060608 | 0.277178171463842 | 2167.70044000708 | 13637.6847660537 | Rwanda |
| 1993 | 14065.340639428 | 0.276980133306619 | 2113.80516096006 | 11951.5354784679 | Rwanda |
| 1994 | 40959.6976333866 | 0.272331257765128 | 2116.74300893131 | 38842.9546244553 | Rwanda |
| 1995 | 18681.4303652577 | 0.271102226876129 | 2182.18802836498 | 16499.2423368927 | Rwanda |
| 1996 | 17850.4900394827 | 0.271097922261967 | 2131.03650828388 | 15719.4535311988 | Rwanda |
| 1997 | 15514.943031719 | 0.272455860850458 | 2171.66635089723 | 13343.2766808218 | Rwanda |
| 1998 | 14625.1844028356 | 0.274582550589084 | 2170.01311768145 | 12455.1712851542 | Rwanda |
| 1999 | 14212.1486978722 | 0.277014387103527 | 2159.23011534355 | 12052.9185825286 | Rwanda |
| 2000 | 13726.7203339065 | 0.280983454342867 | 2174.56602890635 | 11552.1543050002 | Rwanda |
| 2001 | 13050.7097399953 | 0.286082054881284 | 2165.59636540257 | 10885.1133745927 | Rwanda |
| 2002 | 12372.1866876633 | 0.292955430049408 | 2110.68706403905 | 10261.4996236243 | Rwanda |
| 2003 | 11630.1893833327 | 0.299676918389748 | 2140.73825457796 | 9489.45112875474 | Rwanda |
| 2004 | 11697.9371145088 | 0.306943916198519 | 2121.8382438963 | 9576.0988706125 | Rwanda |
| 2005 | 10896.8664472298 | 0.314219577820047 | 2107.88573944076 | 8788.98070778905 | Rwanda |
| 2006 | 10061.3786705681 | 0.321998519469469 | 2105.87687177333 | 7955.50179879477 | Rwanda |
| 2007 | 9044.58525936645 | 0.329616590545695 | 2046.23852423435 | 6998.3467351321 | Rwanda |
| 2008 | 8114.01504859005 | 0.337039226343398 | 1913.01538719493 | 6200.99966139512 | Rwanda |
| 2009 | 7061.54733943169 | 0.344494632549039 | 1776.0524411035 | 5285.49489832818 | Rwanda |
| 2010 | 6452.91778215988 | 0.351659415720748 | 1759.87776055013 | 4693.04002160975 | Rwanda |
| 2011 | 5720.10713366697 | 0.358940339871084 | 1655.56019380146 | 4064.54693986551 | Rwanda |
| 2012 | 4313.64629969266 | 0.366411820814214 | 1321.37224294054 | 2992.27405675213 | Rwanda |
| 2013 | 3383.12668504302 | 0.373769682430802 | 1188.69536382557 | 2194.43132121745 | Rwanda |
| 2014 | 2982.1507549326 | 0.381533179436292 | 1130.15280705132 | 1851.99794788128 | Rwanda |
| 2015 | 2666.14217914769 | 0.389895414951459 | 1033.86239387822 | 1632.27978526947 | Rwanda |
| 2016 | 2418.77511811332 | 0.39828795853881 | 935.200028901761 | 1483.57508921156 | Rwanda |
| 2017 | 2073.66136141323 | 0.406411474976626 | 875.49431364006 | 1198.16704777317 | Rwanda |
| 2018 | 1860.11281664938 | 0.414657911701104 | 853.522108844466 | 1006.59070780491 | Rwanda |
| 2019 | 1741.61595721829 | 0.42308672715334 | 694.670699115156 | 1046.94525810313 | Rwanda |
| 2020 | 1671.50516272739 | 0.429896595971404 | 422.550083275357 | 1248.95507945203 | Rwanda |
| 2021 | 1472.73931602497 | 0.435588705789772 | 426.081068903533 | 1046.65824712144 | Rwanda |
| 1990 | 665.32830743107 | 0.575526495579186 | 86.8832342441214 | 578.445073186949 | Seychelles |
| 1991 | 633.65671005721 | 0.582698331394814 | 82.0222587057367 | 551.634451351473 | Seychelles |
| 1992 | 604.22934469531 | 0.590052918893135 | 80.1229595539963 | 524.106385141314 | Seychelles |
| 1993 | 583.28770483242 | 0.598044193906834 | 75.8901429048621 | 507.397561927558 | Seychelles |
| 1994 | 547.19649216084 | 0.606041267633362 | 76.2828538127332 | 470.913638348107 | Seychelles |
| 1995 | 523.95696719872 | 0.613853280633323 | 70.2706824442366 | 453.686284754483 | Seychelles |
| 1996 | 496.26422749035 | 0.622135170108466 | 61.4833068852172 | 434.780920605133 | Seychelles |
| 1997 | 469.4017936 | 0.630697731713198 | 55.9771062051629 | 413.424687428637 | Seychelles |
| 1998 | 455.88423938064 | 0.63843888230513 | 50.7920346837907 | 405.092204696849 | Seychelles |
| 1999 | 454.24776905509 | 0.645339665687648 | 49.4667923378664 | 404.780976717224 | Seychelles |
| 2000 | 452.93454764502 | 0.651551988453137 | 46.0079906274525 | 406.926557017567 | Seychelles |
| 2001 | 448.20562246934 | 0.656490668328199 | 45.1973474334618 | 403.008275035878 | Seychelles |
| 2002 | 445.26119648546 | 0.660907789985091 | 43.241751095378 | 402.019445390082 | Seychelles |
| 2003 | 441.91375114456 | 0.664162026621557 | 40.5063616030381 | 401.407389541522 | Seychelles |
| 2004 | 442.9872223 | 0.666461353259832 | 39.3102613785917 | 403.676960917408 | Seychelles |
| 2005 | 440.38378066871 | 0.669264742237803 | 38.6075201307965 | 401.776260537913 | Seychelles |
| 2006 | 434.19969327749 | 0.672149914351772 | 37.8528691096218 | 396.346824167868 | Seychelles |
| 2007 | 814.97656008847 | 0.675077437552466 | 38.0487230576899 | 776.92783703078 | Seychelles |
| 2008 | 420.67782045138 | 0.676590946064689 | 37.904086488623 | 382.773733962757 | Seychelles |
| 2009 | 414.03960922529 | 0.67784702810111 | 37.3101650708971 | 376.729444154393 | Seychelles |
| 2010 | 410.64852765817 | 0.680163639820996 | 37.7663736289866 | 372.882154029183 | Seychelles |
| 2011 | 404.19417400884 | 0.683681491395532 | 37.2099859448722 | 366.984188063968 | Seychelles |
| 2012 | 398.2037123 | 0.687629394362418 | 37.3772929916253 | 360.826419295075 | Seychelles |
| 2013 | 389.38593533362 | 0.692525733840497 | 36.7689387590464 | 352.616996574574 | Seychelles |
| 2014 | 378.41463365547 | 0.697114588650508 | 35.6909572782327 | 342.723676377237 | Seychelles |
| 2015 | 367.06685754671 | 0.701722819856481 | 35.878211457017 | 331.188646089693 | Seychelles |
| 2016 | 323.96173789686 | 0.706183622956323 | 34.7146146148044 | 289.247123282056 | Seychelles |
| 2017 | 210.55295187436 | 0.711027281077533 | 31.9461962747562 | 178.606755599604 | Seychelles |
| 2018 | 142.61559763051 | 0.716407819415594 | 31.236538745535 | 111.379058884975 | Seychelles |
| 2019 | 110.83797347282 | 0.722073684746157 | 28.7381975038864 | 82.0997759689336 | Seychelles |
| 2020 | 140.05850466853 | 0.726663769455011 | 26.415430101963 | 113.643074566567 | Seychelles |
| 2021 | 103.39880076976 | 0.730150774629382 | 24.9179993088759 | 78.4808014608841 | Seychelles |
| 1990 | 28393.2848389321 | 0.0488485644444749 | 28393.2848389321 | 0 | Somalia |
| 1991 | 28316.2905355177 | 0.0494223815231112 | 28316.2905355177 | 0 | Somalia |
| 1992 | 28928.0654588881 | 0.050115531337966 | 28389.3332967421 | 538.7321621 | Somalia |
| 1993 | 28706.6985642012 | 0.0507979992557536 | 28373.5621751937 | 333.136389007454 | Somalia |
| 1994 | 30071.8113483119 | 0.0514716956452511 | 28383.9157834538 | 1687.89556485813 | Somalia |
| 1995 | 26453.7663076717 | 0.052148606291336 | 26453.7663076717 | 0 | Somalia |
| 1996 | 25339.7057278589 | 0.052840548398447 | 25339.7057278589 | 0 | Somalia |
| 1997 | 24809.1996964364 | 0.0535709673179049 | 24809.1996964364 | 0 | Somalia |
| 1998 | 24193.5727671971 | 0.054321526804773 | 24193.5727671971 | 0 | Somalia |
| 1999 | 23131.2473239589 | 0.0550718380312836 | 23131.2473239589 | 0 | Somalia |
| 2000 | 23200.5343718997 | 0.0558420632221002 | 23152.7733971056 | 47.7609747941424 | Somalia |
| 2001 | 21201.5916152394 | 0.0566431646698027 | 21201.5916152394 | 0 | Somalia |
| 2002 | 19841.9758529771 | 0.0574576117178968 | 19841.9758529771 | 0 | Somalia |
| 2003 | 18565.1311204715 | 0.0582721495971463 | 18565.1311204715 | 0 | Somalia |
| 2004 | 17328.7406736194 | 0.059076945378295 | 17328.7406736194 | 0 | Somalia |
| 2005 | 16119.5180704615 | 0.0598985393277897 | 16119.5180704615 | 0 | Somalia |
| 2006 | 15291.0469508363 | 0.0607414841530955 | 15291.0469508363 | 0 | Somalia |
| 2007 | 16611.5152068813 | 0.0615981512915218 | 15551.2993369321 | 1060.21586994923 | Somalia |
| 2008 | 15316.453125591 | 0.062475386934885 | 15297.2328020809 | 19.2203235100769 | Somalia |
| 2009 | 14491.1131607793 | 0.0633822252221486 | 14491.1131607793 | 0 | Somalia |
| 2010 | 13708.9718729171 | 0.0643221275495221 | 13708.9718729171 | 0 | Somalia |
| 2011 | 13993.6069867116 | 0.065306811339105 | 13778.2074411374 | 215.399545574217 | Somalia |
| 2012 | 13216.9866727765 | 0.0663548789687034 | 13216.9866727765 | 0 | Somalia |
| 2013 | 12925.4388253738 | 0.0674594862548888 | 12925.4388253738 | 0 | Somalia |
| 2014 | 12366.6336263709 | 0.0686092985197366 | 12366.6336263709 | 0 | Somalia |
| 2015 | 11694.8986441768 | 0.0698115214335177 | 11694.8986441768 | 0 | Somalia |
| 2016 | 11592.3199715114 | 0.0710640470412588 | 11592.3199715114 | 0 | Somalia |
| 2017 | 10589.8798250028 | 0.0723347759051063 | 10589.8798250028 | 0 | Somalia |
| 2018 | 9985.22565517485 | 0.0736422483519594 | 9985.22565517485 | 0 | Somalia |
| 2019 | 9829.90908663045 | 0.0749798109966195 | 9829.90908663045 | 0 | Somalia |
| 2020 | 9262.42462858172 | 0.0763633695596333 | 9262.42462858172 | 0 | Somalia |
| 2021 | 8546.23502971219 | 0.0776881089723748 | 8546.23502971219 | 0 | Somalia |
| 1990 | 12653.1140617185 | 0.259306073677984 | 2757.41327698896 | 9895.70078472954 | United Republic of Tanzania |
| 1991 | 12324.2852058225 | 0.262416238461239 | 2695.3552886643 | 9628.9299171582 | United Republic of Tanzania |
| 1992 | 13039.1643740173 | 0.264989447606401 | 2489.50772449365 | 10549.6566495236 | United Republic of Tanzania |
| 1993 | 11687.5568101361 | 0.26725491530928 | 2441.06937764659 | 9246.48743248951 | United Republic of Tanzania |
| 1994 | 11492.4690834201 | 0.269417204337003 | 2154.06514102697 | 9338.40394239313 | United Republic of Tanzania |
| 1995 | 11062.4103023025 | 0.27207820236791 | 2140.10515894145 | 8922.30514336105 | United Republic of Tanzania |
| 1996 | 10525.7364188383 | 0.2755079091009 | 2130.84680263437 | 8394.88961620393 | United Republic of Tanzania |
| 1997 | 10999.4353116626 | 0.279259183750542 | 2175.46250655297 | 8823.97280510963 | United Republic of Tanzania |
| 1998 | 10155.2790229987 | 0.283152610359192 | 2184.40118536842 | 7970.87783763028 | United Republic of Tanzania |
| 1999 | 8888.39907395169 | 0.287381679042618 | 2115.70102239455 | 6772.69805155714 | United Republic of Tanzania |
| 2000 | 7980.46494832476 | 0.291827264752142 | 2183.28997522533 | 5797.17497309943 | United Republic of Tanzania |
| 2001 | 7420.97083292083 | 0.296715645317522 | 2119.11969770575 | 5301.85113521508 | United Republic of Tanzania |
| 2002 | 6987.00586918825 | 0.301983995351723 | 2139.64921551452 | 4847.35665367373 | United Republic of Tanzania |
| 2003 | 6444.20485173856 | 0.307615488417032 | 2120.69535804529 | 4323.50949369327 | United Republic of Tanzania |
| 2004 | 6021.29230690605 | 0.313772983484529 | 2114.37466411178 | 3906.91764279427 | United Republic of Tanzania |
| 2005 | 5493.2480547784 | 0.320437339078038 | 2103.69274939965 | 3389.55530537875 | United Republic of Tanzania |
| 2006 | 5298.10907936671 | 0.327234001890678 | 2071.90583485465 | 3226.20324451206 | United Republic of Tanzania |
| 2007 | 5124.38438747806 | 0.334313236676657 | 1989.86014806509 | 3134.52423941297 | United Republic of Tanzania |
| 2008 | 4944.91734914277 | 0.341352630337404 | 1863.63821896908 | 3081.27913017369 | United Republic of Tanzania |
| 2009 | 4823.49408453926 | 0.348378034950913 | 1756.4839298728 | 3067.01015466646 | United Republic of Tanzania |
| 2010 | 4566.13044610282 | 0.355567563218872 | 1750.38210787989 | 2815.74833822293 | United Republic of Tanzania |
| 2011 | 4165.5188880758 | 0.363036319968136 | 1400.23011766861 | 2765.28877040719 | United Republic of Tanzania |
| 2012 | 3632.92166731729 | 0.370412488284105 | 1224.01082557664 | 2408.91084174065 | United Republic of Tanzania |
| 2013 | 2621.48493126524 | 0.377963171371427 | 1126.04258192086 | 1495.44234934438 | United Republic of Tanzania |
| 2014 | 2094.62901173695 | 0.385974579014964 | 1090.80771806605 | 1003.8212936709 | United Republic of Tanzania |
| 2015 | 1955.10300318558 | 0.394277756847982 | 972.09432728468 | 983.0086759 | United Republic of Tanzania |
| 2016 | 1859.52368712235 | 0.402879307812708 | 894.599109962641 | 964.924577159709 | United Republic of Tanzania |
| 2017 | 1735.77344731015 | 0.411824974100396 | 850.664992509316 | 885.108454800834 | United Republic of Tanzania |
| 2018 | 1548.72157701756 | 0.420985364340548 | 775.39072932134 | 773.33084769622 | United Republic of Tanzania |
| 2019 | 1417.56368231781 | 0.430225378925537 | 416.3882934 | 1001.17538893881 | United Republic of Tanzania |
| 2020 | 1333.33060709131 | 0.438747237740344 | 423.23185318481 | 910.0987539 | United Republic of Tanzania |
| 2021 | 1206.846565 | 0.446568273334685 | 413.417001255399 | 793.429563327601 | United Republic of Tanzania |
| 1990 | 13616.4872075491 | 0.18700109571602 | 8433.26484491109 | 5183.22236263801 | Uganda |
| 1991 | 13428.1556982785 | 0.188924156324117 | 8137.52484896914 | 5290.63084930936 | Uganda |
| 1992 | 13061.2290465274 | 0.191003433034892 | 8133.30548867942 | 4927.92355784798 | Uganda |
| 1993 | 12437.8663086277 | 0.193557455978809 | 7989.70334238983 | 4448.16296623787 | Uganda |
| 1994 | 11741.8585905886 | 0.197293192708182 | 7337.91353546925 | 4403.94505511935 | Uganda |
| 1995 | 11013.173579924 | 0.201835423450881 | 6764.06480895009 | 4249.10877097391 | Uganda |
| 1996 | 10418.6749371601 | 0.207080730880773 | 6626.30177320491 | 3792.37316395519 | Uganda |
| 1997 | 9923.15767573948 | 0.213091696195112 | 5570.63197776331 | 4352.52569797617 | Uganda |
| 1998 | 9188.04334201804 | 0.219712441898334 | 5020.63853909193 | 4167.40480292611 | Uganda |
| 1999 | 8516.62373148948 | 0.226916944982192 | 4561.37908482335 | 3955.24464666613 | Uganda |
| 2000 | 7818.83781615952 | 0.234323225413513 | 3886.66623826781 | 3932.17157789171 | Uganda |
| 2001 | 7367.35491357933 | 0.242186388084657 | 3555.81909443252 | 3811.53581914681 | Uganda |
| 2002 | 6853.16815060572 | 0.250552388304413 | 3026.68879023045 | 3826.47936037527 | Uganda |
| 2003 | 6103.18879556215 | 0.259164882748285 | 2708.01518687474 | 3395.1736086874 | Uganda |
| 2004 | 5841.81282087648 | 0.268004611155769 | 2484.31349573802 | 3357.49932513846 | Uganda |
| 2005 | 5289.19606499535 | 0.277369192598006 | 2153.50884088817 | 3135.68722410718 | Uganda |
| 2006 | 4728.88928869913 | 0.287030582587568 | 2152.7257517453 | 2576.16353695383 | Uganda |
| 2007 | 4283.69566475525 | 0.296740420486269 | 2119.56077579748 | 2164.13488895777 | Uganda |
| 2008 | 3897.06967804634 | 0.306748843313354 | 2144.00076387395 | 1753.06891417239 | Uganda |
| 2009 | 3593.16633282332 | 0.316731152894585 | 2144.41939886735 | 1448.74693395597 | Uganda |
| 2010 | 3489.34840801296 | 0.326543661078545 | 2066.23529694035 | 1423.11311107261 | Uganda |
| 2011 | 3232.97063971162 | 0.336217715783435 | 1925.8007016345 | 1307.16993807712 | Uganda |
| 2012 | 3068.157532 | 0.345364515038614 | 1781.44036777496 | 1286.71716382704 | Uganda |
| 2013 | 3055.95618074039 | 0.354381615123395 | 1764.99495359877 | 1290.96122714162 | Uganda |
| 2014 | 2975.51387190422 | 0.363430903180067 | 1427.36866689943 | 1548.14520500479 | Uganda |
| 2015 | 2776.27709445304 | 0.372537985916872 | 1229.61055080953 | 1546.66654364351 | Uganda |
| 2016 | 2645.0635889363 | 0.381312226396987 | 1126.5569962826 | 1518.5065926537 | Uganda |
| 2017 | 2139.37961500368 | 0.390082258680085 | 1033.00656821909 | 1106.37304678459 | Uganda |
| 2018 | 1591.07246270803 | 0.399117776823962 | 925.48477604413 | 665.5876867 | Uganda |
| 2019 | 1352.05021933129 | 0.408431557149204 | 868.755407766831 | 483.294811564459 | Uganda |
| 2020 | 1383.21502728534 | 0.416846079190348 | 805.164360843492 | 578.050666441848 | Uganda |
| 2021 | 1193.33306639346 | 0.423261181495743 | 689.447960516468 | 503.885105876992 | Uganda |
| 1990 | 19782.795861173 | 0.304008549420206 | 2135.11627040793 | 17647.6795907651 | Zambia |
| 1991 | 20721.4288118262 | 0.305842359512691 | 2109.18578257946 | 18612.2430292467 | Zambia |
| 1992 | 19990.3614119296 | 0.307316277250908 | 2147.90705476675 | 17842.4543571629 | Zambia |
| 1993 | 18726.1062195225 | 0.308780208280919 | 2132.33381856562 | 16593.7724009569 | Zambia |
| 1994 | 17667.9668967359 | 0.309223449091581 | 2139.08055991214 | 15528.8863368238 | Zambia |
| 1995 | 17139.0528790308 | 0.309995813177242 | 2147.09454919015 | 14991.9583298407 | Zambia |
| 1996 | 16616.7527088621 | 0.311326690947126 | 2088.05941678364 | 14528.6932920785 | Zambia |
| 1997 | 16113.479134951 | 0.313087329617191 | 2101.61687487131 | 14011.8622600797 | Zambia |
| 1998 | 15750.4830057208 | 0.314926434901042 | 2130.64362955719 | 13619.8393761636 | Zambia |
| 1999 | 16032.9154112139 | 0.317396437417001 | 2093.84548084073 | 13939.0699303732 | Zambia |
| 2000 | 15230.0856095048 | 0.320625321501856 | 2087.62692253141 | 13142.4586869734 | Zambia |
| 2001 | 18842.6119019056 | 0.324401368208402 | 2076.07010486465 | 16766.541797041 | Zambia |
| 2002 | 13446.0633670043 | 0.328680121871397 | 2041.34979054135 | 11404.7135764629 | Zambia |
| 2003 | 12976.7625004221 | 0.33372139842644 | 1984.97748038113 | 10991.785020041 | Zambia |
| 2004 | 13204.8378798371 | 0.339488916807796 | 1855.98303914856 | 11348.8548406885 | Zambia |
| 2005 | 12432.6253813649 | 0.346048863943135 | 1771.09565854299 | 10661.5297228219 | Zambia |
| 2006 | 11701.3834413393 | 0.353540709176488 | 1757.68315387482 | 9943.70028746448 | Zambia |
| 2007 | 10682.4232548319 | 0.362450967280465 | 1391.01231788667 | 9291.41093694523 | Zambia |
| 2008 | 9955.0988723303 | 0.371688495720519 | 1237.26831530145 | 8717.83055702885 | Zambia |
| 2009 | 8875.37903152954 | 0.381667118197368 | 1126.38560646037 | 7748.99342506917 | Zambia |
| 2010 | 7852.92452671415 | 0.392470988784596 | 1043.64536371538 | 6809.27916299877 | Zambia |
| 2011 | 6779.73947946937 | 0.403475629468659 | 876.103097026683 | 5903.63638244269 | Zambia |
| 2012 | 4954.54595446572 | 0.414574501227966 | 851.542484164144 | 4103.00347030158 | Zambia |
| 2013 | 3985.58098799712 | 0.425492301821784 | 688.5785559 | 3297.00243206772 | Zambia |
| 2014 | 3160.50623800351 | 0.436244005506199 | 417.303443422703 | 2743.20279458081 | Zambia |
| 2015 | 2697.94579747284 | 0.446744718544927 | 416.312510507659 | 2281.63328696518 | Zambia |
| 2016 | 2407.03986101889 | 0.457122596045687 | 396.675452915463 | 2010.36440810343 | Zambia |
| 2017 | 2304.95912837054 | 0.467532631330298 | 362.908441851023 | 1942.05068651952 | Zambia |
| 2018 | 2020.87131842623 | 0.477887711662038 | 334.742533876506 | 1686.12878454972 | Zambia |
| 2019 | 1835.52244216853 | 0.487911522977487 | 308.576622022801 | 1526.94582014573 | Zambia |
| 2020 | 1705.58741231581 | 0.49748556148019 | 237.131684470647 | 1468.45572784516 | Zambia |
| 2021 | 1435.67046560519 | 0.505948954434909 | 221.611500039982 | 1214.05896556521 | Zambia |
| 1990 | 6656.68317120123 | 0.418077748220986 | 795.567148136346 | 5861.11602306488 | Botswana |
| 1991 | 6350.28676595373 | 0.42975228585408 | 433.289729102341 | 5916.99703685139 | Botswana |
| 1992 | 6076.24698579485 | 0.440490843191281 | 420.192200759419 | 5656.05478503543 | Botswana |
| 1993 | 5817.05507859432 | 0.450523613884069 | 406.141926885974 | 5410.91315170835 | Botswana |
| 1994 | 5665.61476494876 | 0.459954966910237 | 397.502154547171 | 5268.11261040159 | Botswana |
| 1995 | 5535.31015536732 | 0.469266513316808 | 343.714994590241 | 5191.59516077708 | Botswana |
| 1996 | 5415.50150568283 | 0.478588352164802 | 324.427447698726 | 5091.0740579841 | Botswana |
| 1997 | 5327.19993793135 | 0.487767857070753 | 310.639054122882 | 5016.56088380847 | Botswana |
| 1998 | 5510.43456874545 | 0.496193621706379 | 267.400687423879 | 5243.03388132157 | Botswana |
| 1999 | 5493.66816130043 | 0.504978826176952 | 223.000029680995 | 5270.66813161943 | Botswana |
| 2000 | 5420.58427983965 | 0.513498223414294 | 206.337438771628 | 5214.24684106802 | Botswana |
| 2001 | 5345.37204507373 | 0.521370181612928 | 198.495364407371 | 5146.87668066636 | Botswana |
| 2002 | 5233.92247200948 | 0.529252963791219 | 180.043584869478 | 5053.878887 | Botswana |
| 2003 | 5030.15909836356 | 0.537000179431652 | 158.156295421441 | 4872.00280294212 | Botswana |
| 2004 | 4749.5505179999 | 0.544349222513256 | 155.01981018681 | 4594.53070781309 | Botswana |
| 2005 | 4583.23961925283 | 0.551706969608463 | 146.561709602179 | 4436.67790965065 | Botswana |
| 2006 | 8780.12163928557 | 0.559221620417772 | 141.827529268808 | 8638.29411001676 | Botswana |
| 2007 | 4641.79642586893 | 0.566702496208929 | 109.38182636081 | 4532.41459950812 | Botswana |
| 2008 | 4599.83231841744 | 0.57388380509654 | 84.9195501664494 | 4514.91276825099 | Botswana |
| 2009 | 4565.09191701154 | 0.579696411585388 | 85.9711476314821 | 4479.12076938006 | Botswana |
| 2010 | 4554.66088172765 | 0.585824150481272 | 78.385849845214 | 4476.27503188244 | Botswana |
| 2011 | 4196.28275042447 | 0.591985456875054 | 78.6829327664108 | 4117.59981765806 | Botswana |
| 2012 | 3372.90962442508 | 0.597739552771288 | 76.3904004551156 | 3296.51922396996 | Botswana |
| 2013 | 2744.13208269518 | 0.604064730659858 | 75.8164214887326 | 2668.31566120645 | Botswana |
| 2014 | 2260.66728022655 | 0.610086048809866 | 70.9182869112791 | 2189.74899331527 | Botswana |
| 2015 | 2010.12950848614 | 0.615411123648458 | 68.5811862925329 | 1941.54832219361 | Botswana |
| 2016 | 1927.66770159729 | 0.620797574794607 | 64.7381963500933 | 1862.9295052472 | Botswana |
| 2017 | 1877.52963870005 | 0.625704953034907 | 60.1589555861147 | 1817.37068311394 | Botswana |
| 2018 | 1813.81168397242 | 0.630606840950645 | 56.1233340516736 | 1757.68834992075 | Botswana |
| 2019 | 1757.53560147986 | 0.635324093279727 | 52.5466319377164 | 1704.98896954214 | Botswana |
| 2020 | 1685.1532576736 | 0.639275298342726 | 51.4111795808999 | 1633.7420780927 | Botswana |
| 2021 | 1522.55599962202 | 0.642721628921122 | 53.0496939394279 | 1469.50630568259 | Botswana |
| 1990 | 15786.496548594 | 0.339155125342909 | 1858.35773147452 | 13928.1388171195 | Lesotho |
| 1991 | 15599.8742192593 | 0.34549657132085 | 1782.13855400489 | 13817.7356652544 | Lesotho |
| 1992 | 15140.8242939827 | 0.352169629526247 | 1761.85585973264 | 13378.9684342501 | Lesotho |
| 1993 | 14578.3296430847 | 0.358780457204597 | 1648.85986801459 | 12929.4697750701 | Lesotho |
| 1994 | 13948.6628944191 | 0.365310801307453 | 1289.73801439935 | 12658.9248800198 | Lesotho |
| 1995 | 13195.2366409898 | 0.3712653154353 | 1226.69837282606 | 11968.5382681637 | Lesotho |
| 1996 | 12677.037491177 | 0.377221201711086 | 1173.84531219463 | 11503.1921789824 | Lesotho |
| 1997 | 12487.8421041248 | 0.383096647768047 | 1093.58916661517 | 11394.2529375096 | Lesotho |
| 1998 | 12380.1087198838 | 0.388406478488836 | 1035.03047128043 | 11345.0782486034 | Lesotho |
| 1999 | 12672.863432817 | 0.393495000758155 | 975.888907427076 | 11696.9745253899 | Lesotho |
| 2000 | 12929.1455318674 | 0.398635802456027 | 933.536542883006 | 11995.6089889844 | Lesotho |
| 2001 | 13241.2335180055 | 0.403990126208872 | 886.464052878197 | 12354.7694651273 | Lesotho |
| 2002 | 13391.8565418903 | 0.409021656522558 | 872.869611244806 | 12518.9869306455 | Lesotho |
| 2003 | 13341.4600403419 | 0.413982570645541 | 855.675819263862 | 12485.784221078 | Lesotho |
| 2004 | 13449.6444598875 | 0.418670918560125 | 792.534487572831 | 12657.1099723147 | Lesotho |
| 2005 | 13289.5560568148 | 0.423211227878272 | 689.71027914926 | 12599.8457776655 | Lesotho |
| 2006 | 13045.5546543184 | 0.427806731919536 | 502.487502948355 | 12543.06715 | Lesotho |
| 2007 | 13183.9784970047 | 0.432745038329554 | 428.768413799425 | 12755.2100832053 | Lesotho |
| 2008 | 13271.3632910153 | 0.438340567457201 | 425.088768309496 | 12846.2745227058 | Lesotho |
| 2009 | 12192.129262502 | 0.443580126941483 | 419.046655464691 | 11773.0826070373 | Lesotho |
| 2010 | 11423.9191944653 | 0.44896647618497 | 413.960226462668 | 11009.9589680026 | Lesotho |
| 2011 | 11440.5550021372 | 0.454530098376785 | 409.268559413583 | 11031.2864427236 | Lesotho |
| 2012 | 10915.3328496353 | 0.460419689024454 | 397.779607570827 | 10517.5532420645 | Lesotho |
| 2013 | 9629.08316486546 | 0.466692255685783 | 359.017113446382 | 9270.06605141908 | Lesotho |
| 2014 | 9093.38474195658 | 0.473188157042242 | 334.506579689259 | 8758.87816226732 | Lesotho |
| 2015 | 9128.91587750978 | 0.479754456396731 | 325.82672874373 | 8803.08914876605 | Lesotho |
| 2016 | 8736.3292947283 | 0.486144826981745 | 320.798967356779 | 8415.53032737152 | Lesotho |
| 2017 | 7799.94401955518 | 0.491978154790894 | 293.920584522298 | 7506.02343503288 | Lesotho |
| 2018 | 7041.82042543323 | 0.497381790902339 | 242.700748247606 | 6799.11967718562 | Lesotho |
| 2019 | 6511.60021677773 | 0.502457316539537 | 228.505881146337 | 6283.09433563139 | Lesotho |
| 2020 | 6274.69627195691 | 0.50674665490739 | 218.789687420722 | 6055.90658453619 | Lesotho |
| 2021 | 5727.92410636388 | 0.510393065681381 | 213.302985210169 | 5514.62112115371 | Lesotho |
| 1990 | 10119.151003043 | 0.45004023335046 | 407.828278004697 | 9711.3227250383 | Namibia |
| 1991 | 9628.22821980557 | 0.454534440388721 | 410.43996413241 | 9217.78825567316 | Namibia |
| 1992 | 9116.12439355453 | 0.460021313919932 | 396.748471607514 | 8719.37592194702 | Namibia |
| 1993 | 8609.85575979546 | 0.465734134805029 | 359.68780092007 | 8250.16795887539 | Namibia |
| 1994 | 8077.04819990624 | 0.47187321693073 | 342.093893246597 | 7734.95430665964 | Namibia |
| 1995 | 7590.15759005589 | 0.477794328919676 | 335.631751667121 | 7254.52583838877 | Namibia |
| 1996 | 7231.81733015968 | 0.483116378270553 | 320.984393026942 | 6910.83293713274 | Namibia |
| 1997 | 7030.53867188911 | 0.488012938242902 | 310.375120827122 | 6720.16355106199 | Namibia |
| 1998 | 7220.51954716751 | 0.492812548000801 | 296.744232743233 | 6923.77531442428 | Namibia |
| 1999 | 7230.82534197159 | 0.49761386200554 | 237.027320549263 | 6993.79802142233 | Namibia |
| 2000 | 7237.12521806088 | 0.502406957891221 | 228.148892992514 | 7008.97632506837 | Namibia |
| 2001 | 7109.82842074778 | 0.506719032722361 | 219.496408950355 | 6890.33201179743 | Namibia |
| 2002 | 7184.82988096691 | 0.510818274709975 | 213.18178148294 | 6971.64809948397 | Namibia |
| 2003 | 7139.84983234576 | 0.514574645155077 | 206.809185342322 | 6933.04064700344 | Namibia |
| 2004 | 6996.56006062317 | 0.51910360486724 | 199.996190158936 | 6796.56387046423 | Namibia |
| 2005 | 7018.43960792112 | 0.523881735538067 | 189.758583711566 | 6828.68102420955 | Namibia |
| 2006 | 6931.06421854653 | 0.529350117774113 | 180.792249065266 | 6750.27196948126 | Namibia |
| 2007 | 7045.43431059854 | 0.535062125820852 | 161.956135551712 | 6883.47817504683 | Namibia |
| 2008 | 6979.14113814404 | 0.54089699471582 | 155.475786795229 | 6823.66535134881 | Namibia |
| 2009 | 6722.67810932057 | 0.546516477315561 | 149.118493016513 | 6573.55961630406 | Namibia |
| 2010 | 6379.47958791728 | 0.552741565170742 | 146.179658008334 | 6233.29992990895 | Namibia |
| 2011 | 5762.31861830915 | 0.559618899481539 | 139.46079321954 | 5622.85782508961 | Namibia |
| 2012 | 4667.78838612437 | 0.566978697200188 | 108.363444908809 | 4559.42494121556 | Namibia |
| 2013 | 3816.81949418679 | 0.574590344212504 | 86.3773601163575 | 3730.44213407043 | Namibia |
| 2014 | 2796.8298768712 | 0.582321424930035 | 84.4068385866436 | 2712.42303828456 | Namibia |
| 2015 | 2271.86160358194 | 0.589821040312457 | 80.4176464516417 | 2191.4439571303 | Namibia |
| 2016 | 2053.20361961941 | 0.596283059335379 | 78.7231754100602 | 1974.48044420935 | Namibia |
| 2017 | 1885.40652383617 | 0.601754644588282 | 75.8624322384509 | 1809.54409159772 | Namibia |
| 2018 | 1787.47772921729 | 0.606618403183865 | 74.1462057104453 | 1713.33152350684 | Namibia |
| 2019 | 1723.07608085002 | 0.610921091420642 | 70.2862182082443 | 1652.78986264178 | Namibia |
| 2020 | 1722.3828606682 | 0.614435889089058 | 68.8192985767973 | 1653.5635620914 | Namibia |
| 2021 | 1586.73117714756 | 0.617564872149664 | 61.8852533377243 | 1524.84592380984 | Namibia |
| 1990 | 10430.1390491093 | 0.541571435204427 | 155.036876039565 | 10275.1021730697 | South Africa |
| 1991 | 9960.96595631359 | 0.546921135754198 | 149.540431953225 | 9811.42552436037 | South Africa |
| 1992 | 9560.53553235029 | 0.552066898744688 | 146.158984046686 | 9414.3765483036 | South Africa |
| 1993 | 9180.35253672418 | 0.557198439031159 | 141.51523842471 | 9038.83729829947 | South Africa |
| 1994 | 8878.6556621086 | 0.562448636018678 | 138.797965223903 | 8739.8576968847 | South Africa |
| 1995 | 8565.52046464061 | 0.567905303801023 | 111.669323104458 | 8453.85114153615 | South Africa |
| 1996 | 8247.0136474761 | 0.573453987768516 | 84.7713278396717 | 8162.24231963643 | South Africa |
| 1997 | 8074.49876631945 | 0.57909927788854 | 86.0874216594275 | 7988.41134466002 | South Africa |
| 1998 | 7972.32817649672 | 0.58443731296776 | 84.1988003013462 | 7888.12937619537 | South Africa |
| 1999 | 7830.64988546928 | 0.589407348753061 | 80.5773647881154 | 7750.07252068116 | South Africa |
| 2000 | 7888.57019257932 | 0.594093682936262 | 77.8225688392154 | 7810.7476237401 | South Africa |
| 2001 | 7925.27677436835 | 0.597884168238822 | 78.8875173341241 | 7846.38925703423 | South Africa |
| 2002 | 8001.42469163991 | 0.600921675691185 | 75.4430004588802 | 7925.98169118103 | South Africa |
| 2003 | 7919.76170789253 | 0.603515602003029 | 75.9608661442084 | 7843.80084174832 | South Africa |
| 2004 | 7849.45749469765 | 0.606262513759854 | 75.6730772064783 | 7773.78441749117 | South Africa |
| 2005 | 7713.21122547425 | 0.609989979082156 | 70.925013836946 | 7642.2862116373 | South Africa |
| 2006 | 7090.98052355362 | 0.615014689925222 | 69.5187447384662 | 7021.46177881515 | South Africa |
| 2007 | 6076.61756503019 | 0.620608149811677 | 63.468989540075 | 6013.14857549012 | South Africa |
| 2008 | 5020.87809633757 | 0.625948031125006 | 60.083691239156 | 4960.79440509841 | South Africa |
| 2009 | 3748.61251921434 | 0.630645626147581 | 55.9531505608522 | 3692.65936865349 | South Africa |
| 2010 | 3259.23513109255 | 0.635268538092523 | 53.1063765134063 | 3206.12875457914 | South Africa |
| 2011 | 3067.7469753341 | 0.639991722232976 | 50.7159405784772 | 3017.03103475562 | South Africa |
| 2012 | 2862.00667597095 | 0.644715660880732 | 49.1243686271591 | 2812.88230734379 | South Africa |
| 2013 | 2628.7825053826 | 0.64943055353059 | 47.2400158112805 | 2581.54248957132 | South Africa |
| 2014 | 2504.52112959465 | 0.653964494596397 | 45.2795834424142 | 2459.24154615224 | South Africa |
| 2015 | 2375.76570997833 | 0.658417761080757 | 44.9208773685146 | 2330.84483260982 | South Africa |
| 2016 | 2233.05107425575 | 0.662642486535973 | 41.5167778619929 | 2191.53429639376 | South Africa |
| 2017 | 2133.74276426919 | 0.666622608224758 | 39.3868314810637 | 2094.35593278813 | South Africa |
| 2018 | 2075.56053629131 | 0.670335365040043 | 38.527094633426 | 2037.03344165788 | South Africa |
| 2019 | 2027.53437774242 | 0.674041036954557 | 37.8982141430741 | 1989.63616359935 | South Africa |
| 2020 | 1753.4557517859 | 0.677166096009468 | 37.9269143475226 | 1715.52883743838 | South Africa |
| 2021 | 1659.29433365393 | 0.679626598156588 | 37.6161552047196 | 1621.67817844921 | South Africa |
| 1990 | 23040.3193357216 | 0.399420954712507 | 935.832452010091 | 22104.4868837115 | Eswatini |
| 1991 | 22402.504643787 | 0.408269968877275 | 867.092446898187 | 21535.4121968888 | Eswatini |
| 1992 | 22802.345859706 | 0.416683379093578 | 789.618523517702 | 22012.7273361883 | Eswatini |
| 1993 | 21543.8068175294 | 0.424802994934769 | 688.330001569605 | 20855.4768159598 | Eswatini |
| 1994 | 21208.5668752623 | 0.432503472188562 | 437.973915455678 | 20770.5929598066 | Eswatini |
| 1995 | 20841.7764549043 | 0.440127046710174 | 418.70208682318 | 20423.0743680811 | Eswatini |
| 1996 | 20501.9522452459 | 0.447399238142412 | 412.942802241738 | 20089.0094430042 | Eswatini |
| 1997 | 20217.9890482336 | 0.454089854339089 | 407.435276523474 | 19810.5537717101 | Eswatini |
| 1998 | 20055.3407818165 | 0.46013698389181 | 397.157582026114 | 19658.1831997904 | Eswatini |
| 1999 | 20326.044207894 | 0.465990845350804 | 363.095998596906 | 19962.9482092971 | Eswatini |
| 2000 | 21299.4813159931 | 0.471852843205366 | 342.16326477211 | 20957.318051221 | Eswatini |
| 2001 | 23479.4202301376 | 0.477233374481226 | 334.794166504847 | 23144.6260636328 | Eswatini |
| 2002 | 20832.674723964 | 0.482474423324763 | 328.35929883652 | 20504.3154251275 | Eswatini |
| 2003 | 21157.8072589945 | 0.487608829048368 | 311.546172725227 | 20846.2610862693 | Eswatini |
| 2004 | 21081.5466352552 | 0.492669158763782 | 296.951264245664 | 20784.5953710095 | Eswatini |
| 2005 | 19867.2120749669 | 0.498043848851584 | 236.199395492496 | 19631.0126794744 | Eswatini |
| 2006 | 18986.267878977 | 0.503658293748865 | 224.825560073867 | 18761.4423189031 | Eswatini |
| 2007 | 18473.4108752083 | 0.509260218872045 | 214.149384034607 | 18259.2614911737 | Eswatini |
| 2008 | 17691.617374725 | 0.514730895784455 | 206.868852657275 | 17484.7485220677 | Eswatini |
| 2009 | 16663.5233816299 | 0.520427398115377 | 199.050578542556 | 16464.4728030873 | Eswatini |
| 2010 | 15029.4259042531 | 0.526245801589325 | 191.274779089621 | 14838.1511251635 | Eswatini |
| 2011 | 13101.796052837 | 0.531913910153764 | 172.441944875165 | 12929.3541079618 | Eswatini |
| 2012 | 11000.5507396804 | 0.537798022788039 | 157.853300721597 | 10842.6974389588 | Eswatini |
| 2013 | 8821.26576593104 | 0.543836416273961 | 155.342509207109 | 8665.92325672393 | Eswatini |
| 2014 | 7040.91490124766 | 0.549613848251705 | 146.302808607093 | 6894.61209264057 | Eswatini |
| 2015 | 5534.99681700166 | 0.555373738956414 | 143.88016920482 | 5391.11664779684 | Eswatini |
| 2016 | 5248.21817030434 | 0.561061185783082 | 139.162641430265 | 5109.05552887408 | Eswatini |
| 2017 | 4954.0675032145 | 0.566698492780748 | 115.368646151468 | 4838.69885706303 | Eswatini |
| 2018 | 4549.46882745178 | 0.572154807441339 | 85.400579863954 | 4464.06824758783 | Eswatini |
| 2019 | 4349.68312559088 | 0.577206305034792 | 80.8810174729674 | 4268.80210811791 | Eswatini |
| 2020 | 4222.71909975086 | 0.581653071099534 | 81.9407712372389 | 4140.77832851362 | Eswatini |
| 2021 | 3749.55768490582 | 0.58545971329224 | 79.2566914918219 | 3670.300993 | Eswatini |
| 1990 | 3605.07538166044 | 0.398559340611047 | 935.453647077367 | 2669.62173458307 | Zimbabwe |
| 1991 | 3632.23655359114 | 0.406052876694226 | 879.41266363103 | 2752.82388996011 | Zimbabwe |
| 1992 | 4114.93640147899 | 0.412083825921141 | 851.655368080425 | 3263.28103339857 | Zimbabwe |
| 1993 | 4183.74577107888 | 0.417974200219438 | 798.161365493032 | 3385.58440558585 | Zimbabwe |
| 1994 | 3865.43733575866 | 0.424051564461987 | 689.500650969378 | 3175.93668478928 | Zimbabwe |
| 1995 | 3745.51047312902 | 0.428929040100791 | 511.112432038241 | 3234.39804109078 | Zimbabwe |
| 1996 | 3553.51391516632 | 0.43418719166794 | 424.18330381627 | 3129.33061135005 | Zimbabwe |
| 1997 | 3559.23760529038 | 0.438141987585594 | 424.712678938304 | 3134.52492635208 | Zimbabwe |
| 1998 | 3643.66246934445 | 0.441093134678486 | 429.464382527435 | 3214.19808681702 | Zimbabwe |
| 1999 | 3968.21839818111 | 0.443246603614544 | 420.421896791762 | 3547.79650138935 | Zimbabwe |
| 2000 | 4229.68109522591 | 0.444841401738061 | 415.79415604563 | 3813.88693918028 | Zimbabwe |
| 2001 | 4290.53222727085 | 0.446354680553508 | 414.077023638863 | 3876.45520363199 | Zimbabwe |
| 2002 | 4711.67495134147 | 0.446970895206592 | 413.502155703401 | 4298.17279563807 | Zimbabwe |
| 2003 | 4693.18518106949 | 0.446002181962281 | 415.517805066993 | 4277.6673760025 | Zimbabwe |
| 2004 | 4722.89271702346 | 0.443696404187693 | 421.383602598758 | 4301.5091144247 | Zimbabwe |
| 2005 | 5034.94894203319 | 0.439651656979407 | 421.269849907411 | 4613.67909212578 | Zimbabwe |
| 2006 | 5273.02079449757 | 0.434405758403472 | 423.517436477862 | 4849.50335801971 | Zimbabwe |
| 2007 | 5027.57883122202 | 0.429165139621798 | 511.54602481042 | 4516.0328064116 | Zimbabwe |
| 2008 | 8207.02737434672 | 0.422448974219341 | 709.221656053992 | 7497.80571829273 | Zimbabwe |
| 2009 | 9167.20414019142 | 0.418330613224402 | 790.707382762675 | 8376.49675742874 | Zimbabwe |
| 2010 | 5473.57101489796 | 0.417305356749818 | 798.806383084443 | 4674.76463181352 | Zimbabwe |
| 2011 | 5464.84205572058 | 0.419729694705381 | 776.282272833101 | 4688.55978288748 | Zimbabwe |
| 2012 | 4732.07240266408 | 0.425748519814194 | 692.308824935917 | 4039.76357772816 | Zimbabwe |
| 2013 | 3825.11687901733 | 0.432881888218656 | 425.553455619693 | 3399.56342339764 | Zimbabwe |
| 2014 | 2844.79748299033 | 0.439997226175148 | 425.051970917821 | 2419.74551207251 | Zimbabwe |
| 2015 | 2491.32296895442 | 0.446552872040041 | 411.01655746626 | 2080.30641148816 | Zimbabwe |
| 2016 | 2363.07210035787 | 0.452473036911982 | 409.196916935026 | 1953.87518342284 | Zimbabwe |
| 2017 | 2058.87413765192 | 0.458424984120402 | 398.59641279921 | 1660.27772485271 | Zimbabwe |
| 2018 | 2040.50216266345 | 0.464379262217529 | 378.685368778593 | 1661.81679388486 | Zimbabwe |
| 2019 | 1927.44171405548 | 0.46838443787862 | 356.820577416415 | 1570.62113663906 | Zimbabwe |
| 2020 | 1978.18176095734 | 0.471573170735407 | 340.01469273082 | 1638.16706822652 | Zimbabwe |
| 2021 | 1906.75029510414 | 0.473819486202707 | 336.918702954125 | 1569.83159215002 | Zimbabwe |
| 1990 | 23167.8725708062 | 0.218907153827645 | 5085.39052235849 | 18082.4820484477 | Benin |
| 1991 | 23717.5987171631 | 0.222539019363851 | 5123.14214104391 | 18594.4565761192 | Benin |
| 1992 | 21978.8604327121 | 0.226323465232837 | 4392.18018698543 | 17586.6802457267 | Benin |
| 1993 | 21174.1945052453 | 0.230312593690675 | 3823.8459075439 | 17350.3485977014 | Benin |
| 1994 | 20480.4594374954 | 0.234032035653662 | 3860.50905063665 | 16619.9503868587 | Benin |
| 1995 | 19573.605552814 | 0.238009696338161 | 3656.04763436432 | 15917.5579184497 | Benin |
| 1996 | 19551.550450324 | 0.241895322542988 | 3584.09549331986 | 15967.4549570041 | Benin |
| 1997 | 17846.8203936943 | 0.245731534038315 | 3281.23045669685 | 14565.5899369974 | Benin |
| 1998 | 16870.7679580991 | 0.249376450881329 | 3376.81495969331 | 13493.9529984058 | Benin |
| 1999 | 16165.9335351373 | 0.253215340715821 | 3024.75634174262 | 13141.1771933947 | Benin |
| 2000 | 15453.9311358349 | 0.257380681388323 | 2771.41184350475 | 12682.5192923301 | Benin |
| 2001 | 14573.3894678224 | 0.261388010509781 | 2720.21498827439 | 11853.174479548 | Benin |
| 2002 | 14146.2185573597 | 0.265417457761242 | 2482.45565226408 | 11663.7629050956 | Benin |
| 2003 | 13184.1478530961 | 0.269544281064264 | 2173.50236760554 | 11010.6454854906 | Benin |
| 2004 | 12826.1058505591 | 0.273941144522725 | 2181.04975368039 | 10645.0560968787 | Benin |
| 2005 | 12005.0522231399 | 0.278406750274251 | 2130.69543622421 | 9874.35678691569 | Benin |
| 2006 | 11512.956795857 | 0.282933935672546 | 2142.85905016924 | 9370.09774568776 | Benin |
| 2007 | 10728.5648835639 | 0.287673054621831 | 2124.77576548255 | 8603.78911808135 | Benin |
| 2008 | 10121.9715282485 | 0.292627446283673 | 2137.72848990129 | 7984.24303834721 | Benin |
| 2009 | 9685.69770901912 | 0.297702227030519 | 2137.08206682328 | 7548.61564219584 | Benin |
| 2010 | 9334.12330579288 | 0.302809008308589 | 2126.21530829081 | 7207.90799750207 | Benin |
| 2011 | 8772.84638137586 | 0.30792553783669 | 2127.95027466441 | 6644.89610671145 | Benin |
| 2012 | 8562.11414259245 | 0.313373588474229 | 2117.99116440623 | 6444.12297818622 | Benin |
| 2013 | 8070.17899917299 | 0.319318688191374 | 2097.7826888967 | 5972.39631027629 | Benin |
| 2014 | 7355.5972816132 | 0.325727271772744 | 2053.46057569338 | 5302.13670591982 | Benin |
| 2015 | 7110.71321235809 | 0.332216350180226 | 1989.15402960259 | 5121.5591827555 | Benin |
| 2016 | 6392.50258683528 | 0.338763921416219 | 1868.08979694686 | 4524.41278988842 | Benin |
| 2017 | 5458.18834953773 | 0.345606758793044 | 1770.02176659336 | 3688.16658294437 | Benin |
| 2018 | 4403.7310042839 | 0.352652695728689 | 1763.25607824896 | 2640.47492603494 | Benin |
| 2019 | 3418.22887678973 | 0.360031163133997 | 1566.67585825215 | 1851.55301853758 | Benin |
| 2020 | 3345.86516294917 | 0.36696448721315 | 1242.37080256053 | 2103.49436038864 | Benin |
| 2021 | 3053.42744609403 | 0.373486574239249 | 1224.06055747076 | 1829.36688862327 | Benin |
| 1990 | 22770.328856102 | 0.129695615380271 | 8828.12997400828 | 13942.1988820937 | Burkina Faso |
| 1991 | 21940.8190440794 | 0.133299127658948 | 8750.7907620747 | 13190.0282820047 | Burkina Faso |
| 1992 | 21179.0481893885 | 0.136594798422558 | 8764.1232225233 | 12414.9249668652 | Burkina Faso |
| 1993 | 20558.6008868846 | 0.139825464930799 | 8842.40689302732 | 11716.1939938573 | Burkina Faso |
| 1994 | 20015.124385412 | 0.142848972509425 | 8874.51008387276 | 11140.6143015392 | Burkina Faso |
| 1995 | 19599.8414667242 | 0.146115672699206 | 8779.2521047646 | 10820.5893619596 | Burkina Faso |
| 1996 | 18928.2981992823 | 0.150041686384544 | 8811.94827700025 | 10116.3499222821 | Burkina Faso |
| 1997 | 18221.9020915691 | 0.154228401481324 | 8753.33378969289 | 9468.56830187621 | Burkina Faso |
| 1998 | 17576.7091307588 | 0.158707048449452 | 8811.01114507544 | 8765.69798568336 | Burkina Faso |
| 1999 | 17236.7903162345 | 0.163468066858732 | 8801.10571121479 | 8435.68460501971 | Burkina Faso |
| 2000 | 17617.2667285714 | 0.168084340000467 | 8702.84432062044 | 8914.42240795096 | Burkina Faso |
| 2001 | 16676.7210628429 | 0.172996395327051 | 8756.89212406058 | 7919.82893878232 | Burkina Faso |
| 2002 | 16182.5269415899 | 0.17798198994173 | 8790.02758007865 | 7392.49936151125 | Burkina Faso |
| 2003 | 14918.8295841906 | 0.183253539277265 | 8640.21062746428 | 6278.61895672632 | Burkina Faso |
| 2004 | 13966.4849562205 | 0.188564995004729 | 8377.82426992182 | 5588.66068629868 | Burkina Faso |
| 2005 | 13280.0141251795 | 0.194086128180827 | 7928.23791958151 | 5351.77620559799 | Burkina Faso |
| 2006 | 12667.7259649923 | 0.199571952068224 | 7283.46629300837 | 5384.25967198393 | Burkina Faso |
| 2007 | 11527.7235009488 | 0.204891092363641 | 6586.52941771925 | 4941.19408322955 | Burkina Faso |
| 2008 | 10959.6818771508 | 0.210223294804678 | 6205.82414271387 | 4753.85773443693 | Burkina Faso |
| 2009 | 10260.7515554424 | 0.215423373997381 | 5597.51725728484 | 4663.23429815756 | Burkina Faso |
| 2010 | 9835.51969076218 | 0.221001030335074 | 5122.90900728527 | 4712.61068347691 | Burkina Faso |
| 2011 | 9261.75446122016 | 0.226696631667163 | 4458.7624266183 | 4802.99203460186 | Burkina Faso |
| 2012 | 8546.03827789408 | 0.23258913471079 | 3854.01887268621 | 4692.01940520787 | Burkina Faso |
| 2013 | 6839.98550418624 | 0.238497491410915 | 3548.80199917555 | 3291.18350501069 | Burkina Faso |
| 2014 | 6037.6928901623 | 0.244378901166049 | 3322.69744386218 | 2714.99544630012 | Burkina Faso |
| 2015 | 5789.52039106201 | 0.250208260823773 | 2979.40292697354 | 2810.11746408847 | Burkina Faso |
| 2016 | 5656.36630285774 | 0.256081773936025 | 2966.80472457997 | 2689.56157827777 | Burkina Faso |
| 2017 | 5331.68214854234 | 0.26203300853891 | 2719.27428166737 | 2612.40786687497 | Burkina Faso |
| 2018 | 4878.97149874637 | 0.268102072864973 | 2473.03828275546 | 2405.93321599091 | Burkina Faso |
| 2019 | 4558.66298415711 | 0.274287092352859 | 2174.89988692657 | 2383.76309723054 | Burkina Faso |
| 2020 | 4220.10661938667 | 0.279871317215145 | 2173.07389686275 | 2047.03272252392 | Burkina Faso |
| 2021 | 3915.01478343736 | 0.285118401552141 | 2162.235446 | 1752.77933741436 | Burkina Faso |
| 1990 | 11526.7195771883 | 0.303055334404352 | 2120.93873750262 | 9405.78083968568 | Cameroon |
| 1991 | 11430.4186049504 | 0.30962345806122 | 2128.02927067089 | 9302.38933427951 | Cameroon |
| 1992 | 10789.5866538579 | 0.315601268856928 | 2130.6917262465 | 8658.8949276114 | Cameroon |
| 1993 | 10493.5735722875 | 0.320831654317412 | 2096.40173671419 | 8397.17183557331 | Cameroon |
| 1994 | 10320.4874743999 | 0.325410122747214 | 2049.58487481956 | 8270.90259958034 | Cameroon |
| 1995 | 10041.9258095211 | 0.329539317887926 | 2030.93428728784 | 8010.99152223327 | Cameroon |
| 1996 | 10348.1506236589 | 0.333425981814059 | 1990.2066513595 | 8357.9439722994 | Cameroon |
| 1997 | 10025.3794752034 | 0.337346000940944 | 1848.65754849566 | 8176.72192670774 | Cameroon |
| 1998 | 9849.44531253838 | 0.341197089780886 | 1870.67367091647 | 7978.77164162191 | Cameroon |
| 1999 | 9706.06233452785 | 0.344955364212408 | 1764.87731140036 | 7941.1850231275 | Cameroon |
| 2000 | 9835.26692070963 | 0.348814788893637 | 1770.61522177744 | 8064.65169893218 | Cameroon |
| 2001 | 9628.51875077515 | 0.352631872778337 | 1758.91695457326 | 7869.60179620189 | Cameroon |
| 2002 | 10304.8926824359 | 0.356511816668113 | 1750.31037799394 | 8554.58230444196 | Cameroon |
| 2003 | 10165.7485856907 | 0.360668581210006 | 1568.20265639027 | 8597.54592930043 | Cameroon |
| 2004 | 9876.20620840622 | 0.365100029903911 | 1307.51722628195 | 8568.68898212427 | Cameroon |
| 2005 | 9663.2961237256 | 0.369545756449213 | 1233.66101448156 | 8429.63510924404 | Cameroon |
| 2006 | 9525.91464603315 | 0.374355877714557 | 1189.29904816474 | 8336.61559786841 | Cameroon |
| 2007 | 9084.93597252415 | 0.379490349180985 | 1128.57488350078 | 7956.36108902337 | Cameroon |
| 2008 | 8828.15740308817 | 0.38483830406879 | 1085.69248389009 | 7742.46491919808 | Cameroon |
| 2009 | 8730.67322715229 | 0.390288642984762 | 1041.28934757884 | 7689.38387957345 | Cameroon |
| 2010 | 8848.875006 | 0.396067145154693 | 977.202019783677 | 7871.67298635632 | Cameroon |
| 2011 | 8110.20643524089 | 0.402092798748116 | 920.586329459684 | 7189.6201057812 | Cameroon |
| 2012 | 6573.39949895236 | 0.408580122601571 | 867.868582740684 | 5705.53091621168 | Cameroon |
| 2013 | 5593.49390843982 | 0.415607964760584 | 799.069963895368 | 4794.42394454445 | Cameroon |
| 2014 | 4380.88682698327 | 0.423033560011266 | 693.763880516133 | 3687.12294646714 | Cameroon |
| 2015 | 3629.73459192952 | 0.430830276458225 | 430.610951543169 | 3199.12364038635 | Cameroon |
| 2016 | 3365.00901060061 | 0.438878945356817 | 420.335316605508 | 2944.6736939951 | Cameroon |
| 2017 | 2973.84091143213 | 0.447185100165971 | 412.285309496988 | 2561.55560193514 | Cameroon |
| 2018 | 2703.99927327317 | 0.455687846771621 | 402.984462323134 | 2301.01481095004 | Cameroon |
| 2019 | 2373.75336821807 | 0.464246472572462 | 379.463278819743 | 1994.29008939833 | Cameroon |
| 2020 | 2143.65607140431 | 0.472285846440991 | 341.403566555511 | 1802.2525048488 | Cameroon |
| 2021 | 1820.07823921763 | 0.479691223419746 | 327.31805726571 | 1492.76018195192 | Cameroon |
| 1990 | 10100.9832498877 | 0.27672336711542 | 2132.19109259817 | 7968.79215728953 | Cabo Verde |
| 1991 | 9346.46041527478 | 0.282931721669337 | 2160.75320928457 | 7185.70720599021 | Cabo Verde |
| 1992 | 8848.80399239905 | 0.288460371177891 | 2145.69853118019 | 6703.10546121886 | Cabo Verde |
| 1993 | 8424.00119628933 | 0.294517285990211 | 2123.49642024317 | 6300.50477604616 | Cabo Verde |
| 1994 | 9721.43525205723 | 0.302216794344931 | 2124.61262116549 | 7596.82263089174 | Cabo Verde |
| 1995 | 17592.099275431 | 0.310675633896478 | 2097.35829461694 | 15494.7409808141 | Cabo Verde |
| 1996 | 6618.13750148103 | 0.319736229276549 | 2094.64386533141 | 4523.49363614962 | Cabo Verde |
| 1997 | 5670.16658708376 | 0.329049585287328 | 2047.60397133686 | 3622.5626157469 | Cabo Verde |
| 1998 | 4958.19099504321 | 0.338786069354652 | 1854.29085858701 | 3103.9001364562 | Cabo Verde |
| 1999 | 4456.24363489912 | 0.349296918199719 | 1764.95188177113 | 2691.29175312799 | Cabo Verde |
| 2000 | 3914.22759347962 | 0.35989901703054 | 1565.26104527979 | 2348.96654819983 | Cabo Verde |
| 2001 | 3587.76634166341 | 0.370070275603364 | 1224.5580930088 | 2363.20824865461 | Cabo Verde |
| 2002 | 3321.02893629578 | 0.379846674271366 | 1133.7767600474 | 2187.25217624838 | Cabo Verde |
| 2003 | 3004.41640593532 | 0.389419785822276 | 1038.95049016432 | 1965.465916 | Cabo Verde |
| 2004 | 2733.98040067271 | 0.398838732156207 | 923.524553859181 | 1810.45584681353 | Cabo Verde |
| 2005 | 2469.60694336662 | 0.408009221053434 | 875.043337180423 | 1594.5636061862 | Cabo Verde |
| 2006 | 2408.82129584261 | 0.417433785875035 | 795.883748148496 | 1612.93754769411 | Cabo Verde |
| 2007 | 2188.7212051848 | 0.427379786660776 | 554.360549394455 | 1634.36065579034 | Cabo Verde |
| 2008 | 1978.00426731043 | 0.437372129942837 | 424.379600962936 | 1553.62466634749 | Cabo Verde |
| 2009 | 1776.55396104048 | 0.446691505793351 | 412.917075939088 | 1363.63688510139 | Cabo Verde |
| 2010 | 1735.48802911394 | 0.455647568382572 | 400.486339010939 | 1335.00169 | Cabo Verde |
| 2011 | 1561.53350348317 | 0.464473968892355 | 379.250658082401 | 1182.28284540077 | Cabo Verde |
| 2012 | 1480.44235041868 | 0.472857771380441 | 343.336462232964 | 1137.10588818572 | Cabo Verde |
| 2013 | 1364.31583744639 | 0.480796480653885 | 324.324121959088 | 1039.9917154873 | Cabo Verde |
| 2014 | 1341.9673217677 | 0.488259896216045 | 311.189861218769 | 1030.77746054893 | Cabo Verde |
| 2015 | 1238.85064051306 | 0.495229489103676 | 283.358424379504 | 955.492216133556 | Cabo Verde |
| 2016 | 1152.67208825064 | 0.501855423977514 | 230.45607360025 | 922.21601465039 | Cabo Verde |
| 2017 | 1049.17195840748 | 0.508435792446345 | 218.776779499275 | 830.395178908205 | Cabo Verde |
| 2018 | 981.87753077898 | 0.515285886261897 | 206.035156938328 | 775.842373840652 | Cabo Verde |
| 2019 | 897.22237878101 | 0.522488008219635 | 197.914279084834 | 699.308099696176 | Cabo Verde |
| 2020 | 814.63642381106 | 0.528461594777472 | 180.18986418949 | 634.44655962157 | Cabo Verde |
| 2021 | 765.6781271 | 0.53353453903661 | 172.289140851938 | 593.388986252562 | Cabo Verde |
| 1990 | 43359.9347602936 | 0.114638829376832 | 8798.37433145888 | 34561.5604288347 | Chad |
| 1991 | 45908.7683816813 | 0.117954633635022 | 8745.07033049058 | 37163.6980511907 | Chad |
| 1992 | 41709.9233871055 | 0.121132831941026 | 8724.5981310332 | 32985.3252560723 | Chad |
| 1993 | 41135.8882611959 | 0.123600072412422 | 8749.67620054778 | 32386.2120606481 | Chad |
| 1994 | 40491.4474231267 | 0.126080571521756 | 8798.75123389913 | 31692.6961892276 | Chad |
| 1995 | 39749.7473787002 | 0.128289491688625 | 8762.91669470076 | 30986.8306839994 | Chad |
| 1996 | 39374.9242397312 | 0.130169483922475 | 8766.12701445085 | 30608.7972252803 | Chad |
| 1997 | 39444.7803094121 | 0.132136021297442 | 8805.42785948553 | 30639.3524499266 | Chad |
| 1998 | 38393.6060866209 | 0.134090304250411 | 8826.52452035539 | 29567.0815662655 | Chad |
| 1999 | 37799.6066795578 | 0.135825260914887 | 8781.71395358852 | 29017.8927259693 | Chad |
| 2000 | 37658.1846248419 | 0.137412374447162 | 8702.76715347986 | 28955.417471362 | Chad |
| 2001 | 37452.8161257096 | 0.13966369176859 | 8825.30477850466 | 28627.5113472049 | Chad |
| 2002 | 37197.9708796875 | 0.142041700148116 | 8743.85813968016 | 28454.1127400073 | Chad |
| 2003 | 36400.0322558068 | 0.145348052064453 | 8811.92598878133 | 27588.1062670255 | Chad |
| 2004 | 35464.1573330414 | 0.150910867941762 | 8853.33063697263 | 26610.8266960688 | Chad |
| 2005 | 33836.7090365222 | 0.157033036048947 | 8767.5914700864 | 25069.1175664358 | Chad |
| 2006 | 33658.3569092438 | 0.162576402156357 | 8790.59910393457 | 24867.7578053092 | Chad |
| 2007 | 33524.8690450129 | 0.168161061828519 | 8755.23346218594 | 24769.635582827 | Chad |
| 2008 | 32540.9746958613 | 0.173396783580077 | 8702.10084850863 | 23838.8738473527 | Chad |
| 2009 | 31807.6439759821 | 0.178335360569271 | 8689.80024885553 | 23117.8437271266 | Chad |
| 2010 | 30832.5731415106 | 0.184110621219396 | 8708.77096964944 | 22123.8021718612 | Chad |
| 2011 | 29793.6834449241 | 0.189591609430813 | 8055.94612392767 | 21737.7373209964 | Chad |
| 2012 | 27733.2299898022 | 0.195262748994652 | 7449.73535440931 | 20283.4946353929 | Chad |
| 2013 | 27286.9850171624 | 0.200952883793321 | 7294.72735533659 | 19992.2576618258 | Chad |
| 2014 | 25981.7917930953 | 0.206658835963237 | 6633.08111623309 | 19348.7106768622 | Chad |
| 2015 | 25443.7597008483 | 0.212428696994434 | 6024.03784880062 | 19419.7218520477 | Chad |
| 2016 | 24663.034563319 | 0.217532179615503 | 5614.79343790147 | 19048.2411254175 | Chad |
| 2017 | 24100.0046532595 | 0.222206950439072 | 5037.46197929428 | 19062.5426739652 | Chad |
| 2018 | 23012.7619574934 | 0.226913880545936 | 4421.00599991929 | 18591.7559575741 | Chad |
| 2019 | 22234.8194579142 | 0.231704754518571 | 3840.5771275413 | 18394.2423303729 | Chad |
| 2020 | 21027.8989425033 | 0.236103082769538 | 3819.55532253698 | 17208.3436199663 | Chad |
| 2021 | 20173.6053227845 | 0.240436018662427 | 3588.23836994888 | 16585.3669528356 | Chad |
| 1990 | 9238.44789562794 | 0.279320352514124 | 2108.09543777166 | 7130.35245785628 | C么te d'Ivoire |
| 1991 | 9068.06445177496 | 0.286492681425857 | 2177.69178042129 | 6890.37267135367 | C么te d'Ivoire |
| 1992 | 8897.88031716048 | 0.293451293054487 | 2154.50983497135 | 6743.37048218913 | C么te d'Ivoire |
| 1993 | 8788.81309543086 | 0.299743215098528 | 2103.34833122983 | 6685.46476420103 | C么te d'Ivoire |
| 1994 | 8676.54621337306 | 0.305210652688978 | 2158.88353236563 | 6517.66268100742 | C么te d'Ivoire |
| 1995 | 8610.3049908622 | 0.310105507687377 | 2099.38382525599 | 6510.92116560621 | C么te d'Ivoire |
| 1996 | 8212.75542351831 | 0.314965385351403 | 2112.14099775879 | 6100.61442575952 | C么te d'Ivoire |
| 1997 | 7879.14506236043 | 0.319843979495224 | 2093.8551289032 | 5785.28993345723 | C么te d'Ivoire |
| 1998 | 7587.59779038697 | 0.324712872541106 | 2045.95304182883 | 5541.64474855814 | C么te d'Ivoire |
| 1999 | 7220.52977219206 | 0.329201009286605 | 2046.44923547398 | 5174.08053671808 | C么te d'Ivoire |
| 2000 | 6872.37072650838 | 0.333015393275065 | 1988.24850083337 | 4884.12222567501 | C么te d'Ivoire |
| 2001 | 6647.24898286647 | 0.336545511228203 | 1911.41224231007 | 4735.8367405564 | C么te d'Ivoire |
| 2002 | 6178.26985417271 | 0.339977638379859 | 1851.0597609866 | 4327.21009318611 | C么te d'Ivoire |
| 2003 | 5869.30742036369 | 0.343348931560052 | 1776.40696209976 | 4092.90045826393 | C么te d'Ivoire |
| 2004 | 5804.66411970463 | 0.346375411313471 | 1763.91001611099 | 4040.75410359364 | C么te d'Ivoire |
| 2005 | 5806.44618688189 | 0.34872791321654 | 1774.40637533754 | 4032.03981154435 | C么te d'Ivoire |
| 2006 | 5979.81581604459 | 0.35071594385815 | 1765.13862376904 | 4214.67719227555 | C么te d'Ivoire |
| 2007 | 5787.56097665439 | 0.35272747838259 | 1761.22939121691 | 4026.33158543748 | C么te d'Ivoire |
| 2008 | 5481.24321791296 | 0.355087642720634 | 1755.00135702608 | 3726.24186088688 | C么te d'Ivoire |
| 2009 | 5222.09136316572 | 0.357938754729774 | 1645.83393990947 | 3576.25742325625 | C么te d'Ivoire |
| 2010 | 4916.44563805942 | 0.361205186672734 | 1566.444181 | 3350.00145661542 | C么te d'Ivoire |
| 2011 | 4498.00494859226 | 0.364083650087326 | 1282.53713723078 | 3215.46781136148 | C么te d'Ivoire |
| 2012 | 4512.22915060526 | 0.367831685487642 | 1242.41315879044 | 3269.81599181482 | C么te d'Ivoire |
| 2013 | 4496.77249332221 | 0.372353790323331 | 1242.67126225833 | 3254.10123106388 | C么te d'Ivoire |
| 2014 | 4264.0381528669 | 0.377575443834257 | 1185.95192371398 | 3078.08622915292 | C么te d'Ivoire |
| 2015 | 4109.79578155192 | 0.383491379717949 | 1090.63192297985 | 3019.16385857208 | C么te d'Ivoire |
| 2016 | 3618.51616587132 | 0.389825191524618 | 1038.17611521216 | 2580.34005065916 | C么te d'Ivoire |
| 2017 | 2741.08324185074 | 0.396693974091431 | 968.691560052824 | 1772.39168179792 | C么te d'Ivoire |
| 2018 | 2144.79016170002 | 0.404091932623989 | 878.97888994081 | 1265.81127175921 | C么te d'Ivoire |
| 2019 | 1778.56712714488 | 0.411725580444537 | 851.626702769004 | 926.940424375876 | C么te d'Ivoire |
| 2020 | 1554.63738503058 | 0.419040103341885 | 776.000946906844 | 778.636438123736 | C么te d'Ivoire |
| 2021 | 1339.80611796888 | 0.425941883067361 | 682.571132703807 | 657.234985265073 | C么te d'Ivoire |
| 1990 | 11118.0861123015 | 0.238714846451473 | 3536.26353017635 | 7581.82258212515 | Gambia |
| 1991 | 10831.8270048038 | 0.2450101639184 | 3329.68338893109 | 7502.14361587271 | Gambia |
| 1992 | 10607.1069680078 | 0.251087863304524 | 3014.9609982931 | 7592.1459697147 | Gambia |
| 1993 | 10027.8233405322 | 0.25699614074155 | 2741.49600648288 | 7286.32733404932 | Gambia |
| 1994 | 9458.44468413724 | 0.262424792137193 | 2718.06777363915 | 6740.37691049809 | Gambia |
| 1995 | 9507.50075065431 | 0.267696506742935 | 2463.67330187503 | 7043.82744877928 | Gambia |
| 1996 | 8719.63108581826 | 0.272950190161523 | 2144.84936469936 | 6574.7817211189 | Gambia |
| 1997 | 8155.09297928837 | 0.278274122042772 | 2172.45359110574 | 5982.63938818263 | Gambia |
| 1998 | 7920.58513055396 | 0.283946056199827 | 2116.60921479482 | 5803.97591575914 | Gambia |
| 1999 | 7651.03774378619 | 0.290070621097832 | 2138.7233995326 | 5512.31434425359 | Gambia |
| 2000 | 6775.20976796914 | 0.296354282057957 | 2096.4979884633 | 4678.71177950584 | Gambia |
| 2001 | 6160.37508209586 | 0.302543256845694 | 2117.21487884982 | 4043.16020324604 | Gambia |
| 2002 | 5660.30342538857 | 0.307712926799456 | 2114.46057065603 | 3545.84285473254 | Gambia |
| 2003 | 5292.4296904353 | 0.312930916018188 | 2108.7063171193 | 3183.723373 | Gambia |
| 2004 | 5745.42913201206 | 0.31861502922509 | 2143.06523129486 | 3602.3639007172 | Gambia |
| 2005 | 5495.74749700281 | 0.323568350048743 | 2053.89695208211 | 3441.8505449207 | Gambia |
| 2006 | 5058.41928469986 | 0.328151431165807 | 2041.88293954318 | 3016.53634515669 | Gambia |
| 2007 | 4840.27521701496 | 0.332640147075474 | 1983.60556875289 | 2856.66964826207 | Gambia |
| 2008 | 4313.47887258616 | 0.337315245461033 | 1862.80371466816 | 2450.675158 | Gambia |
| 2009 | 3872.88089985314 | 0.342389614346251 | 1769.3649700023 | 2103.51592985084 | Gambia |
| 2010 | 3508.65212650806 | 0.347997829857342 | 1769.24167578292 | 1739.41045072514 | Gambia |
| 2011 | 2821.53642242522 | 0.35286923573207 | 1761.91538659966 | 1059.62103582556 | Gambia |
| 2012 | 2468.24123841374 | 0.358373649782742 | 1641.52250757664 | 826.718730837103 | Gambia |
| 2013 | 1825.1253156826 | 0.36425587830746 | 1325.0276090557 | 500.0977066 | Gambia |
| 2014 | 1641.61815472056 | 0.369901300206273 | 1233.34212379717 | 408.276030923394 | Gambia |
| 2015 | 1400.78838167257 | 0.375840484637477 | 1177.64872263758 | 223.139659034994 | Gambia |
| 2016 | 1386.78682024837 | 0.381569217140042 | 1130.42970969654 | 256.357110551826 | Gambia |
| 2017 | 1453.5809653743 | 0.387273644458255 | 1090.67913988301 | 362.901825491294 | Gambia |
| 2018 | 1366.3637555584 | 0.393155679437306 | 985.470571667466 | 380.893183890934 | Gambia |
| 2019 | 1332.72186345082 | 0.39918978005783 | 926.961810083975 | 405.760053366845 | Gambia |
| 2020 | 1329.69041801597 | 0.40452419422916 | 880.189000889106 | 449.501417126864 | Gambia |
| 2021 | 1164.08858858721 | 0.40971415951073 | 874.525881140044 | 289.562707447166 | Gambia |
| 1990 | 9535.20717686317 | 0.373112004793066 | 1221.51790899299 | 8313.68926787018 | Ghana |
| 1991 | 8963.67738443886 | 0.37923925587052 | 1137.29931131595 | 7826.37807312291 | Ghana |
| 1992 | 8550.26584723045 | 0.384856767297978 | 1092.57444010025 | 7457.6914071302 | Ghana |
| 1993 | 8233.77660531231 | 0.389968494182018 | 1033.44949636589 | 7200.32710894642 | Ghana |
| 1994 | 7945.50653558781 | 0.394953662708371 | 982.004629161952 | 6963.50190642586 | Ghana |
| 1995 | 7667.7869316793 | 0.39993020380395 | 934.719361601779 | 6733.06757007752 | Ghana |
| 1996 | 7326.84862699721 | 0.405003046925554 | 892.421178841063 | 6434.42744815615 | Ghana |
| 1997 | 6883.34734715582 | 0.410038073297741 | 871.178525376592 | 6012.16882177923 | Ghana |
| 1998 | 6591.08858701402 | 0.414888532103537 | 830.638677335397 | 5760.44990967862 | Ghana |
| 1999 | 6232.50276970467 | 0.419755496390431 | 771.620826728195 | 5460.88194297648 | Ghana |
| 2000 | 6040.26014671891 | 0.424725211650337 | 694.62815565689 | 5345.63199106202 | Ghana |
| 2001 | 5852.05918886065 | 0.42941184646569 | 432.199852211274 | 5419.85933664938 | Ghana |
| 2002 | 5806.66767551889 | 0.434104128009022 | 428.982725616174 | 5377.68494990272 | Ghana |
| 2003 | 6116.46994762667 | 0.439118212630728 | 421.22117289624 | 5695.24877473043 | Ghana |
| 2004 | 6070.43688828687 | 0.444236977254313 | 422.750781844959 | 5647.68610644191 | Ghana |
| 2005 | 5971.36605967004 | 0.449360019930072 | 411.379650066706 | 5559.98640960333 | Ghana |
| 2006 | 5746.34818263795 | 0.454692737065749 | 411.534807662142 | 5334.81337497581 | Ghana |
| 2007 | 5422.79211452132 | 0.460606245250364 | 396.423205555008 | 5026.36890896631 | Ghana |
| 2008 | 4608.81805822609 | 0.467259066649708 | 362.4530047 | 4246.36505352929 | Ghana |
| 2009 | 4197.80557154252 | 0.473848047331171 | 338.554155424294 | 3859.25141611823 | Ghana |
| 2010 | 3785.35850474983 | 0.480777891769648 | 323.152150004551 | 3462.20635474528 | Ghana |
| 2011 | 3519.82606338537 | 0.489020973808153 | 310.523230519862 | 3209.30283286551 | Ghana |
| 2012 | 2596.25041171006 | 0.497513931634325 | 239.742572056268 | 2356.50783965379 | Ghana |
| 2013 | 2134.1014081123 | 0.506004646555731 | 221.803281126342 | 1912.29812698596 | Ghana |
| 2014 | 1902.84438756686 | 0.514119061082341 | 207.441029968788 | 1695.40335759807 | Ghana |
| 2015 | 1669.63118716476 | 0.521539202423625 | 198.004882089811 | 1471.62630507495 | Ghana |
| 2016 | 1435.49341640374 | 0.528629585500254 | 178.707226767976 | 1256.78618963576 | Ghana |
| 2017 | 1287.38303005885 | 0.536169940006201 | 157.196977637895 | 1130.18605242096 | Ghana |
| 2018 | 1090.8044309369 | 0.543840539336404 | 155.089572735484 | 935.714858201416 | Ghana |
| 2019 | 954.49378159326 | 0.551632026781383 | 146.096436130682 | 808.397345462578 | Ghana |
| 2020 | 892.83670568824 | 0.558462173287852 | 141.935801111363 | 750.900904576877 | Ghana |
| 2021 | 753.49293189471 | 0.564930389658009 | 113.058267572731 | 640.434664321979 | Ghana |
| 1990 | 24866.9719142538 | 0.17829542065613 | 8761.2943024731 | 16105.6776117807 | Guinea |
| 1991 | 23500.158318308 | 0.181459390831103 | 8668.6110751517 | 14831.5472431563 | Guinea |
| 1992 | 22137.7866610877 | 0.184485016316931 | 8657.2753090852 | 13480.5113520025 | Guinea |
| 1993 | 21203.3487376418 | 0.187686241926773 | 8394.44030968957 | 12808.9084279522 | Guinea |
| 1994 | 21284.0035516342 | 0.191128836868019 | 8122.51624439046 | 13161.4873072437 | Guinea |
| 1995 | 20934.6372957072 | 0.194573244617025 | 8005.04820813781 | 12929.5890875694 | Guinea |
| 1996 | 18652.9299998747 | 0.198633755485464 | 7280.96034111954 | 11371.9696587552 | Guinea |
| 1997 | 17548.6233986502 | 0.203280448621307 | 6634.07474682046 | 10914.5486518297 | Guinea |
| 1998 | 16446.5114579712 | 0.208003382623507 | 6107.08476911927 | 10339.4266888519 | Guinea |
| 1999 | 14933.8058130543 | 0.212412705488692 | 6089.28435265928 | 8844.52146039502 | Guinea |
| 2000 | 13676.2976024469 | 0.216649791599883 | 5682.13401770018 | 7994.16358474672 | Guinea |
| 2001 | 12561.2997379627 | 0.221090940982099 | 5121.69430747874 | 7439.60543048396 | Guinea |
| 2002 | 11744.868870898 | 0.225898779303173 | 4466.66141722949 | 7278.20745366851 | Guinea |
| 2003 | 11237.9998580597 | 0.230522593906606 | 3833.27358789298 | 7404.72627016672 | Guinea |
| 2004 | 11381.525932692 | 0.235134970165926 | 3850.79139462334 | 7530.73453806867 | Guinea |
| 2005 | 10749.422625234 | 0.239691845090143 | 3603.96809108144 | 7145.45453415256 | Guinea |
| 2006 | 10296.8155006156 | 0.24401012651719 | 3375.89913383862 | 6920.91636677698 | Guinea |
| 2007 | 9582.46550316626 | 0.248471690169451 | 3368.76131897856 | 6213.7041841877 | Guinea |
| 2008 | 8696.92985505936 | 0.253167688592502 | 3017.16231036461 | 5679.76754469475 | Guinea |
| 2009 | 7668.9304346153 | 0.257552785014134 | 2767.88257836993 | 4901.04785624537 | Guinea |
| 2010 | 7271.60562996637 | 0.262405948891134 | 2734.84243735918 | 4536.76319260719 | Guinea |
| 2011 | 6605.19332648673 | 0.267624599664508 | 2514.28292380931 | 4090.91040267742 | Guinea |
| 2012 | 6045.23748324966 | 0.273428318991395 | 2158.74567611028 | 3886.49180713938 | Guinea |
| 2013 | 5575.30711076013 | 0.279600444049448 | 2134.61480207844 | 3440.69230868169 | Guinea |
| 2014 | 5077.05982041251 | 0.2859612643771 | 2153.91060778256 | 2923.14921262995 | Guinea |
| 2015 | 4701.06877407631 | 0.292363614463187 | 2124.53240465674 | 2576.53636941957 | Guinea |
| 2016 | 4361.97306539615 | 0.299112412084657 | 2136.34612517586 | 2225.62694022029 | Guinea |
| 2017 | 3886.11561146183 | 0.306881461581969 | 2121.4827653359 | 1764.63284612593 | Guinea |
| 2018 | 3416.33086214667 | 0.314839587576224 | 2127.03052361979 | 1289.30033852688 | Guinea |
| 2019 | 3049.67724923638 | 0.322839848435435 | 2092.28679381542 | 957.390455420955 | Guinea |
| 2020 | 2727.91750583416 | 0.329877997039031 | 2025.24708309712 | 702.670422737045 | Guinea |
| 2021 | 1963.74407509153 | 0.33640129335069 | 1906.43526071205 | 57.3088143794828 | Guinea |
| 1990 | 22041.8686328576 | 0.207614838569111 | 6034.75074626271 | 16007.1178865949 | Guinea-Bissau |
| 1991 | 21124.9021393625 | 0.212505984149041 | 5543.34260242802 | 15581.5595369345 | Guinea-Bissau |
| 1992 | 20314.0600820947 | 0.217026088469754 | 5597.39961043875 | 14716.660471656 | Guinea-Bissau |
| 1993 | 19514.1076864596 | 0.221678978443034 | 5182.05591368708 | 14332.0517727725 | Guinea-Bissau |
| 1994 | 22005.285256371 | 0.226434932988846 | 4570.27851197873 | 17435.0067443923 | Guinea-Bissau |
| 1995 | 17802.0273705868 | 0.231223526184368 | 3967.64981378521 | 13834.3775568016 | Guinea-Bissau |
| 1996 | 32753.9965371032 | 0.236106373654915 | 3930.87003684017 | 28823.126500263 | Guinea-Bissau |
| 1997 | 28672.4766076607 | 0.241360583940643 | 3538.83823359898 | 25133.6383740617 | Guinea-Bissau |
| 1998 | 15228.96816 | 0.244371797123174 | 3323.71278179222 | 11905.2553808378 | Guinea-Bissau |
| 1999 | 14388.1705434558 | 0.24821731725385 | 3344.12256867583 | 11044.04797 | Guinea-Bissau |
| 2000 | 14631.2287353666 | 0.252026519139868 | 3000.48815129662 | 11630.74058 | Guinea-Bissau |
| 2001 | 14561.6163408065 | 0.255786767601624 | 3008.39778856613 | 11553.2185522404 | Guinea-Bissau |
| 2002 | 14236.3850947028 | 0.259321815568398 | 2750.94539206461 | 11485.4397026382 | Guinea-Bissau |
| 2003 | 13561.1801984398 | 0.262747723500015 | 2465.89552152844 | 11095.2846769114 | Guinea-Bissau |
| 2004 | 12927.8775685408 | 0.26627173587343 | 2494.52211938471 | 10433.3554491561 | Guinea-Bissau |
| 2005 | 17216.6188375647 | 0.270056131535634 | 2203.90124449976 | 15012.7175930649 | Guinea-Bissau |
| 2006 | 11070.8508011177 | 0.273803986556405 | 2100.69915539336 | 8970.15164572434 | Guinea-Bissau |
| 2007 | 12117.1078178627 | 0.277805016731205 | 2170.1807018829 | 9946.9271159798 | Guinea-Bissau |
| 2008 | 13871.5096309991 | 0.282151501527761 | 2127.64824288942 | 11743.8613881097 | Guinea-Bissau |
| 2009 | 9861.49431492963 | 0.286423345485565 | 2144.61687001302 | 7716.87744491661 | Guinea-Bissau |
| 2010 | 8889.93858098271 | 0.291141639242703 | 2128.8308766963 | 6761.10770428642 | Guinea-Bissau |
| 2011 | 7547.17418736876 | 0.29668296654448 | 2146.29438395349 | 5400.87980341527 | Guinea-Bissau |
| 2012 | 5449.84716084793 | 0.301562341875562 | 2092.00863171854 | 3357.83852912939 | Guinea-Bissau |
| 2013 | 4868.918734 | 0.30660200942877 | 2124.20205656467 | 2744.71667749733 | Guinea-Bissau |
| 2014 | 3939.36661825165 | 0.311602905000269 | 2114.83974351629 | 1824.52687473536 | Guinea-Bissau |
| 2015 | 3344.17828961088 | 0.31709001451773 | 2133.46827920916 | 1210.71001040172 | Guinea-Bissau |
| 2016 | 3249.04142086178 | 0.32297967010132 | 2090.26641559294 | 1158.77500526884 | Guinea-Bissau |
| 2017 | 2939.86039398948 | 0.329326492160232 | 2037.85361723255 | 902.006776756935 | Guinea-Bissau |
| 2018 | 2529.70094767653 | 0.335667832836675 | 1991.90388481974 | 537.797062856786 | Guinea-Bissau |
| 2019 | 2230.21268681955 | 0.342156114875433 | 1767.06470264796 | 463.147984171591 | Guinea-Bissau |
| 2020 | 2086.14502630046 | 0.347798997943486 | 1770.27874499953 | 315.866281300932 | Guinea-Bissau |
| 2021 | 1805.13579510551 | 0.353109621364536 | 1760.57634708562 | 44.559448019887 | Guinea-Bissau |
| 1990 | 35313.5501643926 | 0.235296846955071 | 3908.84276223804 | 31404.7074021546 | Liberia |
| 1991 | 42892.9939793654 | 0.238212217752062 | 3566.0301541657 | 39326.9638251997 | Liberia |
| 1992 | 38372.7018844158 | 0.23828331265798 | 3607.15547374003 | 34765.5464106758 | Liberia |
| 1993 | 32581.3927511141 | 0.235984406116034 | 3785.21376471942 | 28796.1789863947 | Liberia |
| 1994 | 32951.9865076793 | 0.232656773774217 | 3817.53855852937 | 29134.4479491499 | Liberia |
| 1995 | 35181.922694406 | 0.228424478219389 | 4400.13664820034 | 30781.7860462057 | Liberia |
| 1996 | 34450.1477656238 | 0.222682591493435 | 5112.71943038406 | 29337.4283352397 | Liberia |
| 1997 | 31650.9172302283 | 0.219672764660528 | 5089.47376500114 | 26561.4434652272 | Liberia |
| 1998 | 30217.2287604732 | 0.219053854841174 | 4933.90393909356 | 25283.3248213796 | Liberia |
| 1999 | 28488.7490993438 | 0.222212867280137 | 5033.11221261371 | 23455.6368867301 | Liberia |
| 2000 | 26678.4584979152 | 0.231173361787615 | 3922.62440790181 | 22755.8340900134 | Liberia |
| 2001 | 24060.7772583728 | 0.239594386141111 | 3666.67248706125 | 20394.1047713116 | Liberia |
| 2002 | 21025.070185995 | 0.248311971373795 | 3367.48592554313 | 17657.5842604519 | Liberia |
| 2003 | 19374.6710973748 | 0.251595457910319 | 2995.79702248359 | 16378.8740748912 | Liberia |
| 2004 | 17530.4095834673 | 0.255179610402999 | 3035.25457828313 | 14495.1550051842 | Liberia |
| 2005 | 16984.8759767611 | 0.258967990380421 | 2740.43008357911 | 14244.445893182 | Liberia |
| 2006 | 14955.9157739592 | 0.262897258459796 | 2450.31329305541 | 12505.6024809038 | Liberia |
| 2007 | 12959.8714121846 | 0.267239568498419 | 2451.04827555136 | 10508.8231366332 | Liberia |
| 2008 | 12039.8821958221 | 0.271853595673486 | 2156.94415451909 | 9882.93804130301 | Liberia |
| 2009 | 12419.4302312152 | 0.276804110528483 | 2165.96195186807 | 10253.4682793471 | Liberia |
| 2010 | 12388.530012027 | 0.28223575229287 | 2146.50168094303 | 10242.028331084 | Liberia |
| 2011 | 12012.1493198371 | 0.288015144991527 | 2149.87828285978 | 9862.27103697732 | Liberia |
| 2012 | 11751.4156620871 | 0.294854315315019 | 2126.98019531811 | 9624.43546676899 | Liberia |
| 2013 | 10981.5624415625 | 0.302985656345751 | 2103.26810669232 | 8878.29433487018 | Liberia |
| 2014 | 9631.48726150063 | 0.310955439531843 | 2156.95669481161 | 7474.53056668902 | Liberia |
| 2015 | 8627.72418408745 | 0.318485165511089 | 2107.37353338735 | 6520.3506507001 | Liberia |
| 2016 | 7144.27279333834 | 0.325085173839682 | 2060.89495832171 | 5083.37783501663 | Liberia |
| 2017 | 5672.36586903486 | 0.331527785784429 | 1993.32912550983 | 3679.03674352503 | Liberia |
| 2018 | 4634.92487117727 | 0.337784577514724 | 1859.53388489295 | 2775.39098628432 | Liberia |
| 2019 | 4142.43233369753 | 0.343403262226321 | 1771.86413921706 | 2370.56819448047 | Liberia |
| 2020 | 3936.47299948969 | 0.348133889761756 | 1766.78383639318 | 2169.68916309651 | Liberia |
| 2021 | 3299.2948656178 | 0.352442451643965 | 1765.82449466049 | 1533.47037095731 | Liberia |
| 1990 | 18082.2064036543 | 0.126526427537221 | 8816.30103114574 | 9265.90537250856 | Mali |
| 1991 | 17438.8596901501 | 0.129128150017575 | 8882.37746450416 | 8556.48222564594 | Mali |
| 1992 | 16899.2627186692 | 0.131537448786118 | 8916.2846021252 | 7982.978117 | Mali |
| 1993 | 16283.727346767 | 0.134177054462465 | 8782.12750805444 | 7501.59983871256 | Mali |
| 1994 | 15642.9142927537 | 0.136937829291313 | 8771.40727811132 | 6871.50701464238 | Mali |
| 1995 | 15232.868521128 | 0.139866225457785 | 8781.53008555521 | 6451.33843557279 | Mali |
| 1996 | 15075.4926598065 | 0.143078915935706 | 8727.25987181249 | 6348.23278799401 | Mali |
| 1997 | 13098.0822549806 | 0.146414326236527 | 8773.42789327932 | 4324.65436170128 | Mali |
| 1998 | 12143.6284016158 | 0.149986729501612 | 8753.78888088648 | 3389.83952072932 | Mali |
| 1999 | 11557.0907900101 | 0.153737335688568 | 8919.51823684341 | 2637.57255316668 | Mali |
| 2000 | 11215.0705922066 | 0.157190027521405 | 8770.03753566711 | 2445.03305653949 | Mali |
| 2001 | 10581.4702562825 | 0.161344447555084 | 8755.36891972732 | 1826.10133655518 | Mali |
| 2002 | 11250.2449109187 | 0.165420695773092 | 8694.94198235726 | 2555.30292856144 | Mali |
| 2003 | 10836.7759175924 | 0.169733502039948 | 8743.2065689943 | 2093.5693485981 | Mali |
| 2004 | 10253.2009603719 | 0.173779552322233 | 8711.70841210647 | 1541.49254826543 | Mali |
| 2005 | 9722.81970043266 | 0.178029844366254 | 8707.77508174826 | 1015.0446186844 | Mali |
| 2006 | 9152.94726101546 | 0.182452468201007 | 8680.10759738818 | 472.839663627277 | Mali |
| 2007 | 8245.82328472621 | 0.186912994942121 | 8245.82328472621 | 0 | Mali |
| 2008 | 7866.22406943149 | 0.191536745613522 | 7866.22406943149 | 0 | Mali |
| 2009 | 7236.04144191562 | 0.196537650861103 | 7198.99360680323 | 37.0478351123911 | Mali |
| 2010 | 6454.308108 | 0.201803535998378 | 6454.308108 | 0 | Mali |
| 2011 | 5720.58083444681 | 0.207097221274123 | 5720.58083444681 | 0 | Mali |
| 2012 | 5307.017522 | 0.212465060096693 | 5307.017522 | 0 | Mali |
| 2013 | 4716.2554732931 | 0.217993867180652 | 4716.2554732931 | 0 | Mali |
| 2014 | 3974.67766741909 | 0.223729979734488 | 3974.67766741909 | 0 | Mali |
| 2015 | 3478.54111847336 | 0.229826262811848 | 3478.54111847336 | 0 | Mali |
| 2016 | 3416.37637507848 | 0.236186231331436 | 3416.37637507848 | 0 | Mali |
| 2017 | 3159.07571918772 | 0.242726134546644 | 3159.07571918772 | 0 | Mali |
| 2018 | 2849.11681303118 | 0.249419730729374 | 2849.11681303118 | 0 | Mali |
| 2019 | 2570.05415677333 | 0.256250745993336 | 2570.05415677333 | 0 | Mali |
| 2020 | 2314.71372187977 | 0.262629218759395 | 2314.71372187977 | 0 | Mali |
| 2021 | 1968.07201920848 | 0.268579940925684 | 1968.07201920848 | 0 | Mali |
| 1990 | 12768.8265608367 | 0.33578094163222 | 1981.16565406702 | 10787.6609067697 | Mauritania |
| 1991 | 12229.6104591151 | 0.341135174811723 | 1863.33074953486 | 10366.2797095802 | Mauritania |
| 1992 | 11744.9461815599 | 0.346531288170585 | 1765.37414576276 | 9979.57203579714 | Mauritania |
| 1993 | 11289.3513352115 | 0.35217657188477 | 1761.15964921044 | 9528.19168600106 | Mauritania |
| 1994 | 10394.989420887 | 0.357585911567431 | 1705.5461672011 | 8689.4432536859 | Mauritania |
| 1995 | 9831.87173887124 | 0.363037695183683 | 1396.3668999862 | 8435.50483888504 | Mauritania |
| 1996 | 10446.4575987482 | 0.368508328412644 | 1248.68754614699 | 9197.77005260121 | Mauritania |
| 1997 | 9882.0957912597 | 0.373021338681094 | 1216.18760483218 | 8665.90818642752 | Mauritania |
| 1998 | 9865.01548026074 | 0.377080249599675 | 1166.29301781322 | 8698.72246244752 | Mauritania |
| 1999 | 9410.59816693469 | 0.381112297471017 | 1122.31536398733 | 8288.28280294736 | Mauritania |
| 2000 | 9330.02841746178 | 0.384205379717954 | 1087.7663967054 | 8242.26202075638 | Mauritania |
| 2001 | 8995.6092477717 | 0.386487415894351 | 1095.49203744188 | 7900.11721032982 | Mauritania |
| 2002 | 8743.34532620507 | 0.388564477604617 | 1029.95734597807 | 7713.38798 | Mauritania |
| 2003 | 8143.25571944124 | 0.39110487913777 | 1037.29852807849 | 7105.95719136275 | Mauritania |
| 2004 | 7782.96281250492 | 0.394012933734168 | 959.38566246073 | 6823.57715004419 | Mauritania |
| 2005 | 8186.53309886833 | 0.39749910452189 | 971.47963457344 | 7215.05346429489 | Mauritania |
| 2006 | 7574.28141202732 | 0.403159466975078 | 872.492184870317 | 6701.789227 | Mauritania |
| 2007 | 6763.62022891994 | 0.408377122155099 | 872.852071186204 | 5890.76815773374 | Mauritania |
| 2008 | 5986.92862030634 | 0.413233621211221 | 850.282885429868 | 5136.64573487647 | Mauritania |
| 2009 | 5801.12661798934 | 0.4179106088283 | 787.915156717893 | 5013.21146127145 | Mauritania |
| 2010 | 5073.47780484165 | 0.423202436351832 | 690.436087507818 | 4383.04171733383 | Mauritania |
| 2011 | 4511.02712912402 | 0.428867289887467 | 513.51276871644 | 3997.51436040758 | Mauritania |
| 2012 | 4179.70440720176 | 0.434724375629838 | 421.879561390088 | 3757.82484581167 | Mauritania |
| 2013 | 3549.58738740042 | 0.441068910286714 | 422.310017093443 | 3127.27737030698 | Mauritania |
| 2014 | 2697.20258448561 | 0.447653881356557 | 413.434509135648 | 2283.76807534996 | Mauritania |
| 2015 | 2287.25186057123 | 0.454523487871252 | 405.460390082381 | 1881.79147048885 | Mauritania |
| 2016 | 1939.46424931379 | 0.461269544556856 | 375.616219609148 | 1563.84802970464 | Mauritania |
| 2017 | 1747.53420401581 | 0.468452365279582 | 358.951558901675 | 1388.58264511414 | Mauritania |
| 2018 | 1531.67754682424 | 0.475839507248469 | 336.406503209506 | 1195.27104361473 | Mauritania |
| 2019 | 1501.5206817023 | 0.483767737935156 | 324.623084193217 | 1176.89759750908 | Mauritania |
| 2020 | 1395.41135030058 | 0.491365602592056 | 299.96530041385 | 1095.44604988673 | Mauritania |
| 2021 | 1321.46588807037 | 0.498945100442996 | 237.398377822314 | 1084.06751024806 | Mauritania |
| 1990 | 68315.7653666349 | 0.0808684798233195 | 8762.95615472924 | 59552.8092119057 | Niger |
| 1991 | 68374.4658403201 | 0.0828482077018824 | 8757.27833755149 | 59617.1875027686 | Niger |
| 1992 | 67227.7497984215 | 0.0847715776568405 | 8790.41603109371 | 58437.3337673278 | Niger |
| 1993 | 65764.5886158142 | 0.0866964903938328 | 8813.23368754976 | 56951.3549282644 | Niger |
| 1994 | 63787.1810417985 | 0.0886087332313997 | 8782.4371181551 | 55004.7439236434 | Niger |
| 1995 | 61958.9475759265 | 0.090473708405212 | 8809.95905636485 | 53148.9885195617 | Niger |
| 1996 | 59247.4047606129 | 0.09234635941853 | 8843.31413049944 | 50404.0906301135 | Niger |
| 1997 | 56520.6403043519 | 0.0942191148661978 | 8837.58765620874 | 47683.0526481432 | Niger |
| 1998 | 53897.0878483592 | 0.0965451528067399 | 8750.15321819078 | 45146.9346301684 | Niger |
| 1999 | 51594.0120726044 | 0.0988136856452598 | 8777.76850847004 | 42816.2435641344 | Niger |
| 2000 | 50074.6623219442 | 0.100886948404838 | 8790.2941174574 | 41284.3682044868 | Niger |
| 2001 | 48023.8212634758 | 0.103271776631217 | 8860.8666444395 | 39162.9546190363 | Niger |
| 2002 | 44555.2863516311 | 0.105741108731078 | 8857.18103148508 | 35698.105320146 | Niger |
| 2003 | 42237.6345048683 | 0.108322891429361 | 8774.44335042012 | 33463.1911544482 | Niger |
| 2004 | 37507.8657524813 | 0.110764495003726 | 8873.94396200617 | 28633.9217904751 | Niger |
| 2005 | 38215.9290065909 | 0.11345202277039 | 8817.10442270319 | 29398.8245838877 | Niger |
| 2006 | 34317.1986182506 | 0.116247074448783 | 8807.7014436334 | 25509.4971746172 | Niger |
| 2007 | 31405.7909446857 | 0.11897162605295 | 8801.79687950666 | 22603.994065179 | Niger |
| 2008 | 26101.9475359051 | 0.121957786240242 | 8769.1205242084 | 17332.8270116967 | Niger |
| 2009 | 24250.5553416426 | 0.124829381330847 | 8777.44032978604 | 15473.1150118566 | Niger |
| 2010 | 22029.1817631288 | 0.127952995735601 | 8786.86763340878 | 13242.31413 | Niger |
| 2011 | 20819.3402246741 | 0.130958067074432 | 8803.44585448685 | 12015.8943701873 | Niger |
| 2012 | 18970.6362961769 | 0.134469718123206 | 8712.45371423429 | 10258.1825819426 | Niger |
| 2013 | 17885.2491100909 | 0.137978170438014 | 8777.7552059847 | 9107.4939041062 | Niger |
| 2014 | 15156.4311193763 | 0.141731738162587 | 8771.93470162042 | 6384.49641775588 | Niger |
| 2015 | 13402.883137118 | 0.14544050326966 | 8851.32375306686 | 4551.55938405114 | Niger |
| 2016 | 12793.9245874761 | 0.149200109150915 | 8752.68829960155 | 4041.23628787455 | Niger |
| 2017 | 12073.124868393 | 0.152959169593186 | 8772.30630895319 | 3300.81855943981 | Niger |
| 2018 | 10908.6286063471 | 0.15687519554195 | 8845.87591468094 | 2062.75269166616 | Niger |
| 2019 | 10071.3930292562 | 0.160889483579043 | 8796.3108492122 | 1275.08218 | Niger |
| 2020 | 9755.59193990865 | 0.164539264581005 | 8827.08544855119 | 928.506491357462 | Niger |
| 2021 | 9019.29562104101 | 0.168072773803995 | 8649.71703406537 | 369.57858697564 | Niger |
| 1990 | 41288.1846797509 | 0.305868046855462 | 2136.4050027729 | 39151.779676978 | Nigeria |
| 1991 | 43481.6530688513 | 0.308489225313095 | 2169.02028815775 | 41312.6327806936 | Nigeria |
| 1992 | 39872.7227411653 | 0.311234052054088 | 2120.89626501885 | 37751.8264761464 | Nigeria |
| 1993 | 38928.261439157 | 0.313983059877659 | 2118.70667657369 | 36809.5547625833 | Nigeria |
| 1994 | 38345.4124263693 | 0.316747446775232 | 2122.93896687428 | 36222.473459495 | Nigeria |
| 1995 | 37738.347343266 | 0.319637438693347 | 2087.49871414581 | 35650.8486291202 | Nigeria |
| 1996 | 37510.2260519424 | 0.323137684748282 | 2097.97645544827 | 35412.2495964941 | Nigeria |
| 1997 | 36497.2163142482 | 0.326690552886558 | 2070.08964546802 | 34427.1266687802 | Nigeria |
| 1998 | 35859.4382010387 | 0.330263851422919 | 1984.15286555583 | 33875.2853354829 | Nigeria |
| 1999 | 35483.2758209191 | 0.333936499661916 | 1987.10469874118 | 33496.1711221779 | Nigeria |
| 2000 | 34901.358930698 | 0.338201742797398 | 1845.94022852971 | 33055.4187021683 | Nigeria |
| 2001 | 34279.9169793809 | 0.343029750745818 | 1769.74094430924 | 32510.1760350717 | Nigeria |
| 2002 | 33422.6623359482 | 0.349092528396007 | 1768.50299772835 | 31654.1593382198 | Nigeria |
| 2003 | 32186.7141503543 | 0.355911392103181 | 1757.5596312435 | 30429.1545191108 | Nigeria |
| 2004 | 31235.3517533997 | 0.36378081411686 | 1292.7989210571 | 29942.5528323426 | Nigeria |
| 2005 | 29986.705373355 | 0.372583553203769 | 1225.86531076162 | 28760.8400625934 | Nigeria |
| 2006 | 29817.8921175502 | 0.381026040235948 | 1135.7898509233 | 28682.1022666269 | Nigeria |
| 2007 | 28986.3428944052 | 0.38977964917576 | 1031.54889192879 | 27954.7940024764 | Nigeria |
| 2008 | 27400.0655480738 | 0.398037706162251 | 934.608205805845 | 26465.457342268 | Nigeria |
| 2009 | 26210.3423250636 | 0.40635330747071 | 868.955081711333 | 25341.3872433523 | Nigeria |
| 2010 | 25392.8556790341 | 0.414997747961223 | 829.507239871463 | 24563.3484391626 | Nigeria |
| 2011 | 23452.9260825821 | 0.42335191474477 | 685.090484447302 | 22767.8355981348 | Nigeria |
| 2012 | 22221.4780230726 | 0.431780375863827 | 433.86428369061 | 21787.613739382 | Nigeria |
| 2013 | 21212.8274645312 | 0.440124663848588 | 419.048195180908 | 20793.7792693503 | Nigeria |
| 2014 | 20192.7983131596 | 0.44876682284048 | 407.745489136278 | 19785.0528240233 | Nigeria |
| 2015 | 18838.3163054406 | 0.457240991469025 | 396.07352146178 | 18442.2427839788 | Nigeria |
| 2016 | 17797.436532834 | 0.465183780994652 | 383.672476161332 | 17413.7640566727 | Nigeria |
| 2017 | 16343.5501266363 | 0.472972868984332 | 338.843110508085 | 16004.7070161282 | Nigeria |
| 2018 | 15051.1105159328 | 0.480844413426724 | 326.277011851877 | 14724.8335040809 | Nigeria |
| 2019 | 13732.5092105578 | 0.488787361344695 | 311.578669321647 | 13420.9305412362 | Nigeria |
| 2020 | 10391.6587307393 | 0.496204741160216 | 264.006390839514 | 10127.6523398998 | Nigeria |
| 2021 | 9615.62091893939 | 0.503390832763244 | 224.342706357728 | 9391.27821258166 | Nigeria |
| 1990 | 15687.1163203571 | 0.3095428523248 | 2153.76422536641 | 13533.3520949907 | Sao Tome and Principe |
| 1991 | 13960.2921238195 | 0.310601028486155 | 2135.59686864803 | 11824.6952551715 | Sao Tome and Principe |
| 1992 | 13513.0631539064 | 0.3115154473454 | 2102.40856054939 | 11410.654593357 | Sao Tome and Principe |
| 1993 | 13117.8047100508 | 0.31274355801833 | 2119.04683168634 | 10998.7578783645 | Sao Tome and Principe |
| 1994 | 12760.3084687024 | 0.314261087139278 | 2133.25808234401 | 10627.0503863584 | Sao Tome and Principe |
| 1995 | 11676.2641509601 | 0.315843919852344 | 2122.21136172572 | 9554.05278923438 | Sao Tome and Principe |
| 1996 | 10834.1397143903 | 0.317729288379101 | 2081.35851705771 | 8752.78119733259 | Sao Tome and Principe |
| 1997 | 9747.21446035701 | 0.319795669969502 | 2103.90379210105 | 7643.31066825596 | Sao Tome and Principe |
| 1998 | 8790.31035998162 | 0.322519218688325 | 2129.01061728451 | 6661.29974269711 | Sao Tome and Principe |
| 1999 | 8252.92564011408 | 0.325651915603451 | 2052.45986022082 | 6200.46577989326 | Sao Tome and Principe |
| 2000 | 5764.44285678396 | 0.329460109182343 | 2039.39385018789 | 3725.04900659607 | Sao Tome and Principe |
| 2001 | 5275.59940814271 | 0.334484786134682 | 1981.08317501785 | 3294.51623312486 | Sao Tome and Principe |
| 2002 | 4552.07382257962 | 0.340302836742805 | 1865.38275386828 | 2686.69106871134 | Sao Tome and Principe |
| 2003 | 4002.07183872275 | 0.346851981499259 | 1771.3237299194 | 2230.74810880335 | Sao Tome and Principe |
| 2004 | 3527.16057133581 | 0.353722383532282 | 1755.60803269769 | 1771.55253863812 | Sao Tome and Principe |
| 2005 | 6526.58496491115 | 0.361105881091861 | 1557.43793382592 | 4969.14703108523 | Sao Tome and Principe |
| 2006 | 4964.04953116983 | 0.369413007194338 | 1244.42476400508 | 3719.62476716475 | Sao Tome and Principe |
| 2007 | 3648.68104295044 | 0.377813517075578 | 1181.38032479703 | 2467.30071815341 | Sao Tome and Principe |
| 2008 | 3776.36871223069 | 0.386688943651063 | 1090.90827703548 | 2685.46043519521 | Sao Tome and Principe |
| 2009 | 2913.76790979806 | 0.39582123767217 | 967.243694199614 | 1946.52421559845 | Sao Tome and Principe |
| 2010 | 2805.99469560774 | 0.405530666154327 | 883.295628758164 | 1922.69906684958 | Sao Tome and Principe |
| 2011 | 2056.17138626264 | 0.41578372764052 | 796.420561702538 | 1259.7508245601 | Sao Tome and Principe |
| 2012 | 1557.09301010183 | 0.425721314870657 | 682.50843700557 | 874.58457309626 | Sao Tome and Principe |
| 2013 | 1428.9991966742 | 0.435592459999619 | 423.734585354177 | 1005.26461132002 | Sao Tome and Principe |
| 2014 | 1500.36509459918 | 0.44514623672643 | 424.385289775712 | 1075.97980482347 | Sao Tome and Principe |
| 2015 | 1324.03211934968 | 0.454051473195403 | 407.735565876437 | 916.296553473243 | Sao Tome and Principe |
| 2016 | 941.9277708 | 0.463230081728275 | 381.234359858302 | 560.693410964098 | Sao Tome and Principe |
| 2017 | 718.62366438923 | 0.47217092996608 | 343.254509717442 | 375.369154671788 | Sao Tome and Principe |
| 2018 | 556.16739263616 | 0.481098204433028 | 322.777986417553 | 233.389406218607 | Sao Tome and Principe |
| 2019 | 464.70538 | 0.489838229899105 | 312.516094634639 | 152.189285389061 | Sao Tome and Principe |
| 2020 | 452.27397723793 | 0.497861408762174 | 238.548569977583 | 213.725407260347 | Sao Tome and Principe |
| 2021 | 365.47065840425 | 0.505413746708186 | 224.322756890215 | 141.147901514035 | Sao Tome and Principe |
| 1990 | 25815.7720647721 | 0.238047613296703 | 3604.91042914835 | 22210.8616356238 | Senegal |
| 1991 | 24543.0117649331 | 0.244482178069769 | 3377.09742995104 | 21165.9143349821 | Senegal |
| 1992 | 23310.0160337483 | 0.250416531795468 | 3004.14431136503 | 20305.8717223833 | Senegal |
| 1993 | 22348.5518260478 | 0.255767963935604 | 3020.24026789071 | 19328.3115581571 | Senegal |
| 1994 | 21449.9073592958 | 0.260379997494734 | 2731.1844826574 | 18718.7228766384 | Senegal |
| 1995 | 20318.3726054116 | 0.264438773208067 | 2485.42242432537 | 17832.9501810862 | Senegal |
| 1996 | 19420.4182525528 | 0.268318964653468 | 2473.57142011846 | 16946.8468324343 | Senegal |
| 1997 | 17852.7486268466 | 0.272072875469797 | 2138.39701192728 | 15714.3516149193 | Senegal |
| 1998 | 16970.8859749865 | 0.275995656114005 | 2158.49469141373 | 14812.3912835728 | Senegal |
| 1999 | 16189.4160349532 | 0.280111918035525 | 2163.92234342266 | 14025.4936915305 | Senegal |
| 2000 | 14496.1570761862 | 0.283784902973132 | 2150.79396546265 | 12345.3631107235 | Senegal |
| 2001 | 16288.0327138612 | 0.287451899345797 | 2135.16442471612 | 14152.8682891451 | Senegal |
| 2002 | 14794.31616 | 0.291046287851898 | 2133.73504195874 | 12660.5811197013 | Senegal |
| 2003 | 12923.9550868468 | 0.294887172086492 | 2086.14745983679 | 10837.80763 | Senegal |
| 2004 | 13248.1984373142 | 0.298984520918874 | 2100.49014291404 | 11147.7082944002 | Senegal |
| 2005 | 12683.7999291241 | 0.30325720904165 | 2166.19029049108 | 10517.609638633 | Senegal |
| 2006 | 10745.7544036176 | 0.307633910231713 | 2128.27665452413 | 8617.47774909347 | Senegal |
| 2007 | 9753.8567413683 | 0.312570297567491 | 2106.86228379835 | 7646.99445756995 | Senegal |
| 2008 | 8756.29455301918 | 0.317894088643487 | 2090.7490890647 | 6665.54546395448 | Senegal |
| 2009 | 7766.88010125594 | 0.323657725834671 | 2045.69149608729 | 5721.18860516865 | Senegal |
| 2010 | 6531.60735385086 | 0.329804072240346 | 2054.23681672931 | 4477.37053712155 | Senegal |
| 2011 | 5656.01701545717 | 0.336059897035461 | 1982.81104147954 | 3673.20597397763 | Senegal |
| 2012 | 4372.48159999082 | 0.34297711398143 | 1771.89371759637 | 2600.58788239445 | Senegal |
| 2013 | 3556.30666363433 | 0.350134004435728 | 1755.76084864596 | 1800.54581498837 | Senegal |
| 2014 | 2970.98821747875 | 0.357181573156172 | 1717.79285408196 | 1253.19536339679 | Senegal |
| 2015 | 2452.33807940283 | 0.364144496085842 | 1285.95493641391 | 1166.38314298892 | Senegal |
| 2016 | 2330.41552487044 | 0.371114525115408 | 1225.79448513642 | 1104.62103973402 | Senegal |
| 2017 | 2058.83118500054 | 0.378351476689464 | 1127.33438540046 | 931.496799600078 | Senegal |
| 2018 | 1814.21164598932 | 0.386079665694996 | 1095.44631757375 | 718.765328415568 | Senegal |
| 2019 | 1632.54196977395 | 0.394012316088147 | 979.494056962821 | 653.047912811129 | Senegal |
| 2020 | 1644.10964757033 | 0.401197515620742 | 933.415574272671 | 710.694073297659 | Senegal |
| 2021 | 1407.88568185762 | 0.408054192634391 | 870.371266067185 | 537.514415790435 | Senegal |
| 1990 | 25558.8171243246 | 0.211569335210502 | 6020.81022304625 | 19538.0069012783 | Sierra Leone |
| 1991 | 24447.2775757394 | 0.213013566916176 | 5607.64480115094 | 18839.6327745885 | Sierra Leone |
| 1992 | 25146.3393210624 | 0.214223040293117 | 5529.52852014233 | 19616.8108009201 | Sierra Leone |
| 1993 | 25482.9606180896 | 0.216089200983916 | 5524.8398282759 | 19958.1207898137 | Sierra Leone |
| 1994 | 27110.0439929178 | 0.218277410522878 | 5105.62188817711 | 22004.4221047407 | Sierra Leone |
| 1995 | 25395.6533744099 | 0.219822603796338 | 5086.70223208531 | 20308.9511423246 | Sierra Leone |
| 1996 | 22852.3818270848 | 0.219969269802127 | 5061.32108070163 | 17791.0607463832 | Sierra Leone |
| 1997 | 21804.7422119594 | 0.219089790932757 | 5081.85706517037 | 16722.885146789 | Sierra Leone |
| 1998 | 21864.4979203867 | 0.218584312916824 | 5076.98903587792 | 16787.5088845088 | Sierra Leone |
| 1999 | 22196.328049569 | 0.217943041857131 | 5647.66616356857 | 16548.6618860004 | Sierra Leone |
| 2000 | 22605.4009370958 | 0.218492836742255 | 5103.95405666735 | 17501.4468804284 | Sierra Leone |
| 2001 | 21129.1674596054 | 0.22039123267412 | 5039.73993005183 | 16089.4275295536 | Sierra Leone |
| 2002 | 19371.6574739945 | 0.22458582518118 | 4482.85666367643 | 14888.8008103181 | Sierra Leone |
| 2003 | 17969.6963028099 | 0.229235900070887 | 4387.4866446966 | 13582.2096581133 | Sierra Leone |
| 2004 | 18738.1223612882 | 0.234131673808901 | 3796.5911265673 | 14941.5312347209 | Sierra Leone |
| 2005 | 17442.1898153513 | 0.23954993975681 | 3576.89070235791 | 13865.2991129934 | Sierra Leone |
| 2006 | 16467.3819904121 | 0.245647886413805 | 3377.7446912647 | 13089.6372991474 | Sierra Leone |
| 2007 | 14945.6143757417 | 0.252523076983211 | 2978.10717034724 | 11967.5072053945 | Sierra Leone |
| 2008 | 13543.1918753489 | 0.259781562101124 | 2723.91577694595 | 10819.276098403 | Sierra Leone |
| 2009 | 11360.8971847026 | 0.267058380231042 | 2501.96022605914 | 8858.93695864346 | Sierra Leone |
| 2010 | 10760.7998807723 | 0.274270274980285 | 2169.60666252031 | 8591.19321825199 | Sierra Leone |
| 2011 | 9652.09569973321 | 0.28167357489027 | 2183.49002110444 | 7468.60567862877 | Sierra Leone |
| 2012 | 9008.40421246297 | 0.290338511565667 | 2128.56330170715 | 6879.84091075582 | Sierra Leone |
| 2013 | 7168.96997411021 | 0.300736693229616 | 2150.72013407131 | 5018.2498400389 | Sierra Leone |
| 2014 | 5426.21612511405 | 0.310348867321001 | 2151.68765660008 | 3274.52846851397 | Sierra Leone |
| 2015 | 4712.04205020457 | 0.316998649992998 | 2129.91531406906 | 2582.12673613551 | Sierra Leone |
| 2016 | 4319.68299579125 | 0.324091564628372 | 2080.69932771446 | 2238.98366807679 | Sierra Leone |
| 2017 | 4013.00474389767 | 0.331213431016392 | 1979.19079274401 | 2033.81395115366 | Sierra Leone |
| 2018 | 3619.25051226632 | 0.338341563502228 | 1863.21483366106 | 1756.03567860526 | Sierra Leone |
| 2019 | 3324.14174849878 | 0.345770018534214 | 1767.45435317787 | 1556.68739532091 | Sierra Leone |
| 2020 | 3137.77868343713 | 0.352412021842938 | 1763.4332199487 | 1374.34546348843 | Sierra Leone |
| 2021 | 2749.98842349274 | 0.3586658807038 | 1654.28139426143 | 1095.70702923131 | Sierra Leone |
| 1990 | 31705.6497416807 | 0.269692273286049 | 2161.23607882264 | 29544.4136628581 | Togo |
| 1991 | 31438.1713456276 | 0.274530567688902 | 2106.81613304974 | 29331.3552125779 | Togo |
| 1992 | 30288.3763706867 | 0.278789310483021 | 2163.50008844311 | 28124.8762822436 | Togo |
| 1993 | 29430.9576234448 | 0.281224630552395 | 2127.3741539264 | 27303.5834695184 | Togo |
| 1994 | 28623.7387854005 | 0.284653387233478 | 2171.92768984389 | 26451.8110955566 | Togo |
| 1995 | 27521.1710153642 | 0.288536691543625 | 2151.94647826371 | 25369.2245371005 | Togo |
| 1996 | 26423.57027 | 0.292606677265023 | 2128.76804548555 | 24294.8022211944 | Togo |
| 1997 | 25180.5868868139 | 0.296947835200193 | 2155.2717118855 | 23025.3151749284 | Togo |
| 1998 | 25882.7329083441 | 0.300022332903466 | 2113.99311751829 | 23768.7397908258 | Togo |
| 1999 | 23807.5247131198 | 0.303020232352969 | 2153.27718290403 | 21654.2475302158 | Togo |
| 2000 | 23214.0161003319 | 0.305472963037407 | 2153.69177182963 | 21060.3243285023 | Togo |
| 2001 | 22751.032221515 | 0.307828257876202 | 2130.58968340268 | 20620.4425381123 | Togo |
| 2002 | 23867.0379912371 | 0.310469909626286 | 2143.33472347348 | 21723.7032677636 | Togo |
| 2003 | 22940.8083329272 | 0.313792063910754 | 2171.81315220825 | 20768.995180719 | Togo |
| 2004 | 22058.1705873978 | 0.316687944336242 | 2084.78083693342 | 19973.3897504644 | Togo |
| 2005 | 21224.2272367432 | 0.319025579582374 | 2093.14420072201 | 19131.0830360212 | Togo |
| 2006 | 20735.7636912808 | 0.321659389933158 | 2092.43154018338 | 18643.3321510974 | Togo |
| 2007 | 19298.5721002256 | 0.324382497112276 | 2055.59884288434 | 17242.9732573413 | Togo |
| 2008 | 18638.8356968293 | 0.327743136228821 | 2072.26733094994 | 16566.5683658794 | Togo |
| 2009 | 18447.2725882626 | 0.331558542074185 | 1990.06361448476 | 16457.2089737778 | Togo |
| 2010 | 17405.9367413136 | 0.335899583453674 | 1983.18661214038 | 15422.7501291732 | Togo |
| 2011 | 16314.1344321664 | 0.340777259848227 | 1856.42781390537 | 14457.706618261 | Togo |
| 2012 | 14334.1031841946 | 0.346320495474737 | 1767.00253481148 | 12567.1006493831 | Togo |
| 2013 | 12308.2581647938 | 0.352278604950937 | 1763.05901948242 | 10545.1991453114 | Togo |
| 2014 | 10020.0688062152 | 0.358661442459501 | 1648.418224 | 8371.6505820412 | Togo |
| 2015 | 8981.43529004248 | 0.365600168512118 | 1298.81216531062 | 7682.62312473186 | Togo |
| 2016 | 8453.3170656991 | 0.372910027688811 | 1230.96748764868 | 7222.34957805042 | Togo |
| 2017 | 7869.11531497034 | 0.380336231070946 | 1125.52215272908 | 6743.59316224126 | Togo |
| 2018 | 6997.97030165415 | 0.387921228739501 | 1091.15604300156 | 5906.81425865259 | Togo |
| 2019 | 6518.37663167405 | 0.395667268909215 | 981.708574255276 | 5536.66805741877 | Togo |
| 2020 | 5809.77873486403 | 0.402356922397572 | 880.620811755261 | 4929.15792310877 | Togo |
| 2021 | 5185.71899284086 | 0.408533695477986 | 874.296493594042 | 4311.42249924682 | Togo |
| 1990 | 385.73864672439 | 0.613633923933335 | 70.4709242645712 | 315.267722459819 | American Samoa |
| 1991 | 376.54914188029 | 0.615847611881171 | 68.1597564519741 | 308.389385428316 | American Samoa |
| 1992 | 366.67969212921 | 0.618583654206334 | 62.2498094032785 | 304.429882725932 | American Samoa |
| 1993 | 357.57196645487 | 0.622078597664072 | 62.7529207086841 | 294.819045746186 | American Samoa |
| 1994 | 346.76854650035 | 0.626053240674018 | 59.3509161647371 | 287.417630335613 | American Samoa |
| 1995 | 336.64140881133 | 0.629814053909773 | 55.5035805220523 | 281.137828289278 | American Samoa |
| 1996 | 328.97357522208 | 0.632706982787519 | 54.0885277906867 | 274.885047431393 | American Samoa |
| 1997 | 316.46683429729 | 0.634926685456248 | 52.9609491364627 | 263.505885160827 | American Samoa |
| 1998 | 311.8149957 | 0.636597975713911 | 52.4205599805154 | 259.394435743385 | American Samoa |
| 1999 | 304.01842183517 | 0.638322193134778 | 51.954548937423 | 252.063872897747 | American Samoa |
| 2000 | 296.42957273058 | 0.640609550029113 | 51.5258154909125 | 244.903757239667 | American Samoa |
| 2001 | 290.32385667288 | 0.643214421156583 | 50.8254821580544 | 239.498374514826 | American Samoa |
| 2002 | 288.81966591831 | 0.646002062148026 | 49.6996310827892 | 239.120034835521 | American Samoa |
| 2003 | 284.63605919187 | 0.648951964151548 | 49.3891214366066 | 235.246937755263 | American Samoa |
| 2004 | 279.93302641544 | 0.651921140709413 | 46.010174212007 | 233.922852203433 | American Samoa |
| 2005 | 273.41512532619 | 0.655061005497018 | 45.346664141052 | 228.068461185138 | American Samoa |
| 2006 | 268.99280527858 | 0.657725722991147 | 45.4471055786566 | 223.545699699923 | American Samoa |
| 2007 | 262.50524089305 | 0.660781175742006 | 43.2820292656564 | 219.223211627394 | American Samoa |
| 2008 | 256.72703339436 | 0.664025761610397 | 41.0833991482457 | 215.643634246114 | American Samoa |
| 2009 | 258.22565467308 | 0.667300532067021 | 39.5311542355385 | 218.694500437542 | American Samoa |
| 2010 | 257.88544808025 | 0.671248915725392 | 38.459672981696 | 219.425775098554 | American Samoa |
| 2011 | 257.2077752 | 0.675979288249651 | 37.8203901677541 | 219.387385012846 | American Samoa |
| 2012 | 256.63985804301 | 0.680861538744503 | 37.5870575202761 | 219.052800522734 | American Samoa |
| 2013 | 257.54613998582 | 0.685833061049061 | 37.2678941327225 | 220.278245853097 | American Samoa |
| 2014 | 259.91255561398 | 0.691343136814841 | 36.6631057307635 | 223.249449883216 | American Samoa |
| 2015 | 265.65186361907 | 0.697162912183353 | 35.8287937706005 | 229.82306984847 | American Samoa |
| 2016 | 272.72767520716 | 0.702726397771802 | 35.8028204704482 | 236.924854736712 | American Samoa |
| 2017 | 274.17892712638 | 0.707647909217363 | 33.8767884388214 | 240.302138687559 | American Samoa |
| 2018 | 273.56544374585 | 0.712800591003034 | 32.0589528902367 | 241.506490855613 | American Samoa |
| 2019 | 271.33151576802 | 0.717308133053242 | 31.5072160591549 | 239.824299708865 | American Samoa |
| 2020 | 294.65468339392 | 0.720838500394591 | 28.0142346403419 | 266.640448753578 | American Samoa |
| 2021 | 265.57309717474 | 0.723727533059592 | 27.1009782570645 | 238.472118917675 | American Samoa |
| 1990 | 156.07915687153 | 0.696451195907995 | 36.6956064351423 | 119.383550436388 | Bermuda |
| 1991 | 139.08684881182 | 0.700276851342371 | 35.9127071900213 | 103.174141621799 | Bermuda |
| 1992 | 133.56248579658 | 0.703984094837886 | 34.2647104831665 | 99.2977753134136 | Bermuda |
| 1993 | 134.43543491231 | 0.707678222374744 | 34.3071182244767 | 100.128316687833 | Bermuda |
| 1994 | 121.07132360491 | 0.711068997576994 | 32.1333589336821 | 88.9379646712279 | Bermuda |
| 1995 | 108.87776548684 | 0.714468828293519 | 31.2673060014267 | 77.6104594854133 | Bermuda |
| 1996 | 96.65768566 | 0.71783167141956 | 31.7418631068208 | 64.9158225529992 | Bermuda |
| 1997 | 85.94471066 | 0.721642812571346 | 29.6918611618986 | 56.2528495026114 | Bermuda |
| 1998 | 74.04659227 | 0.725853528350146 | 25.3834663220236 | 48.6631259513064 | Bermuda |
| 1999 | 62.22285749 | 0.730539822974827 | 24.3880663293504 | 37.8347911584196 | Bermuda |
| 2000 | 52.40404114 | 0.73599650207483 | 21.1209613371428 | 31.2830798066572 | Bermuda |
| 2001 | 48.67686741 | 0.741918907664508 | 21.6903061941044 | 26.9865612191356 | Bermuda |
| 2002 | 47.5888076 | 0.747544503099499 | 17.9159113518292 | 29.6728962528908 | Bermuda |
| 2003 | 47.86516271 | 0.753305163354703 | 18.1194782894854 | 29.7456844247546 | Bermuda |
| 2004 | 48.51617967 | 0.759008884830255 | 16.7646162268088 | 31.7515634446512 | Bermuda |
| 2005 | 50.64897471 | 0.764690902756676 | 15.1195156533386 | 35.5294590605414 | Bermuda |
| 2006 | 51.00564852 | 0.770596769707219 | 12.2881431996849 | 38.7175053164651 | Bermuda |
| 2007 | 49.31696615 | 0.776114264028476 | 14.2148439010423 | 35.1021222480077 | Bermuda |
| 2008 | 47.46846049 | 0.780358325453696 | 11.7024933162058 | 35.7659671707742 | Bermuda |
| 2009 | 44.86441676 | 0.7847591074268 | 11.602784982196 | 33.261631777434 | Bermuda |
| 2010 | 43.01908211 | 0.789181466953459 | 11.1456193640444 | 31.8734627495856 | Bermuda |
| 2011 | 42.11303806 | 0.793380523639009 | 11.8866542389547 | 30.2263838259353 | Bermuda |
| 2012 | 40.6917259 | 0.797484869823732 | 10.6919541022322 | 29.9997718006678 | Bermuda |
| 2013 | 39.86350926 | 0.801388200821251 | 10.3087100704377 | 29.5547991875623 | Bermuda |
| 2014 | 39.07978981 | 0.804923406558971 | 9.73486256630757 | 29.3449272440424 | Bermuda |
| 2015 | 37.58371347 | 0.807998794744826 | 9.52107543398571 | 28.0626380386143 | Bermuda |
| 2016 | 36.63698316 | 0.810550842307499 | 9.542745669 | 27.09423749 | Bermuda |
| 2017 | 35.06014664 | 0.812807228761017 | 8.62875436687091 | 26.4313922709891 | Bermuda |
| 2018 | 33.51182778 | 0.814892801994945 | 9.20842608177257 | 24.3034016991774 | Bermuda |
| 2019 | 32.73489215 | 0.817000338369953 | 8.20216929027857 | 24.5327228618614 | Bermuda |
| 2020 | 31.96593199 | 0.819203419907163 | 8.76690894502304 | 23.199023040647 | Bermuda |
| 2021 | 33.5877141 | 0.821365422389484 | 8.25750875971781 | 25.3302053452722 | Bermuda |
| 1990 | 89.39034793 | 0.564514853922858 | 89.39034793 | 0 | Cook Islands |
| 1991 | 85.7639199 | 0.570622775576656 | 79.0136831482835 | 6.75023675577648 | Cook Islands |
| 1992 | 82.60053056 | 0.57712346797279 | 73.9453232157767 | 8.65520734491334 | Cook Islands |
| 1993 | 80.25614036 | 0.584059175401459 | 73.4095859401629 | 6.84655441657715 | Cook Islands |
| 1994 | 78.86021816 | 0.591540867591394 | 74.5687797701149 | 4.29143839253513 | Cook Islands |
| 1995 | 78.06311248 | 0.598789967711714 | 75.0840213312778 | 2.97909115155217 | Cook Islands |
| 1996 | 76.67673938 | 0.606414151024421 | 72.5650884699019 | 4.11165091124808 | Cook Islands |
| 1997 | 73.92346671 | 0.613991564375745 | 66.9295475536829 | 6.99391915875708 | Cook Islands |
| 1998 | 71.9932857 | 0.621202227562338 | 60.4612656787658 | 11.5320200228442 | Cook Islands |
| 1999 | 72.78693379 | 0.628029029838686 | 54.7534587723634 | 18.0334750213466 | Cook Islands |
| 2000 | 70.68680267 | 0.635365607290649 | 52.8353928370505 | 17.8514098279495 | Cook Islands |
| 2001 | 68.14980508 | 0.644421442097654 | 49.0981421734884 | 19.0516629073816 | Cook Islands |
| 2002 | 65.87322873 | 0.652790488145509 | 45.4011692807781 | 20.4720594451019 | Cook Islands |
| 2003 | 64.1854014 | 0.661465037633189 | 42.8080801646842 | 21.3773212388258 | Cook Islands |
| 2004 | 62.14743256 | 0.668769675110248 | 38.8752825419363 | 23.2721500226137 | Cook Islands |
| 2005 | 61.61527448 | 0.67400410252196 | 38.0753351325495 | 23.5399393462505 | Cook Islands |
| 2006 | 60.75948284 | 0.680644001453893 | 37.6443313225302 | 23.1151515153398 | Cook Islands |
| 2007 | 60.2638904 | 0.687505190649195 | 37.3589889988636 | 22.9049014049164 | Cook Islands |
| 2008 | 57.51041475 | 0.695592068858646 | 36.5936522696688 | 20.9167624818912 | Cook Islands |
| 2009 | 54.73949513 | 0.70377697980234 | 34.7932126643925 | 19.9462824627575 | Cook Islands |
| 2010 | 53.10639385 | 0.711861611963853 | 31.6552996943454 | 21.4510941524346 | Cook Islands |
| 2011 | 50.69279269 | 0.719444665855079 | 28.5626209391307 | 22.1301717520293 | Cook Islands |
| 2012 | 47.77327202 | 0.726963385157533 | 26.1618082521963 | 21.6114637721937 | Cook Islands |
| 2013 | 44.55076689 | 0.73331701541165 | 21.6713161924955 | 22.8794506939345 | Cook Islands |
| 2014 | 43.44391165 | 0.73959570406711 | 21.7985227763682 | 21.6453888721718 | Cook Islands |
| 2015 | 43.36833887 | 0.746550296501143 | 20.8296787487456 | 22.5386601236844 | Cook Islands |
| 2016 | 43.44049096 | 0.752353086814977 | 18.6770907247752 | 24.7634002336248 | Cook Islands |
| 2017 | 42.28546054 | 0.758233571650419 | 16.2156346444351 | 26.0698258969249 | Cook Islands |
| 2018 | 41.33248416 | 0.764041181894148 | 16.0236388906821 | 25.3088452661179 | Cook Islands |
| 2019 | 40.43714807 | 0.769795912507863 | 14.1490499872374 | 26.2880980863826 | Cook Islands |
| 2020 | 68.18253003 | 0.774700577781853 | 12.9378099243211 | 55.2447201008389 | Cook Islands |
| 2021 | 70.80424208 | 0.779109954659683 | 12.2666737194933 | 58.5375683618767 | Cook Islands |
| 1990 | 134.86855737356 | 0.732258251665998 | 24.8463629648097 | 110.02219440875 | Greenland |
| 1991 | 129.1574587 | 0.730626532070962 | 23.9397508755424 | 105.217707862558 | Greenland |
| 1992 | 119.91988076286 | 0.729781103358168 | 23.939215649071 | 95.9806651137891 | Greenland |
| 1993 | 113.72052282929 | 0.729894841568107 | 24.6215337300886 | 89.0989890992014 | Greenland |
| 1994 | 106.84081665939 | 0.730875896131236 | 22.9110743608408 | 83.9297422985492 | Greenland |
| 1995 | 101.9733506 | 0.73083163848634 | 23.7598524866309 | 78.2134980707691 | Greenland |
| 1996 | 97.26489881 | 0.730216225093773 | 23.911312887766 | 73.353585920544 | Greenland |
| 1997 | 92.65833689 | 0.730551086919016 | 23.6595198052096 | 68.9988170832304 | Greenland |
| 1998 | 88.74369358 | 0.733242587548811 | 22.6435272698353 | 66.1001663071647 | Greenland |
| 1999 | 85.58670986 | 0.736557130056076 | 20.6479892817448 | 64.9387205830852 | Greenland |
| 2000 | 83.40052179 | 0.740857009793347 | 20.4752466783014 | 62.9252751101886 | Greenland |
| 2001 | 82.87838441 | 0.746289605484857 | 19.5946245654557 | 63.2837598423743 | Greenland |
| 2002 | 83.58169444 | 0.752878370778087 | 16.74459688 | 66.83709756 | Greenland |
| 2003 | 83.80394478 | 0.759444151937822 | 16.3961784807006 | 67.4077662961294 | Greenland |
| 2004 | 82.6684592 | 0.765401567469496 | 14.5164600318717 | 68.1519991696483 | Greenland |
| 2005 | 82.20715558 | 0.772099579848736 | 13.542045701048 | 68.665109874072 | Greenland |
| 2006 | 81.45066147 | 0.779141406404636 | 12.9975442709194 | 68.4531171994406 | Greenland |
| 2007 | 81.37026602 | 0.785321921636087 | 12.0249921170373 | 69.3452739010827 | Greenland |
| 2008 | 81.25462042 | 0.79144125736451 | 11.2630588308326 | 69.9915615856674 | Greenland |
| 2009 | 79.40988555 | 0.797025750029497 | 11.0330732317135 | 68.3768123188165 | Greenland |
| 2010 | 78.10393892 | 0.803003131492839 | 10.4454894235556 | 67.6584495013544 | Greenland |
| 2011 | 76.73446127 | 0.808105185095196 | 9.47896048354611 | 67.2555007825639 | Greenland |
| 2012 | 73.8654294 | 0.811684561566459 | 8.88211399557269 | 64.9833154028173 | Greenland |
| 2013 | 72.90350615 | 0.814368307545592 | 9.32143098831519 | 63.5820751584948 | Greenland |
| 2014 | 70.19658444 | 0.816527059982666 | 8.58577033114489 | 61.6108141061251 | Greenland |
| 2015 | 70.81664044 | 0.8177191894267 | 9.06782433327051 | 61.7488161102695 | Greenland |
| 2016 | 67.84998624 | 0.818961901690003 | 8.68005333519966 | 59.1699329019003 | Greenland |
| 2017 | 63.95107175 | 0.819781100917251 | 8.19720007892023 | 55.7538716752298 | Greenland |
| 2018 | 59.73628775 | 0.820815513545804 | 8.66631806724492 | 51.0699696822051 | Greenland |
| 2019 | 56.38821777 | 0.822339996424292 | 9.03171550879688 | 47.3565022615531 | Greenland |
| 2020 | 59.16162631 | 0.824359028380898 | 7.93383828499556 | 51.2277880214444 | Greenland |
| 2021 | 50.84025152 | 0.82621033634361 | 7.77403348365968 | 43.0662180345603 | Greenland |
| 1990 | 158.46207449301 | 0.67622030520232 | 37.8517064388079 | 120.610368054202 | Guam |
| 1991 | 160.51664825177 | 0.669974291504161 | 38.4275817398948 | 122.089066511875 | Guam |
| 1992 | 162.68718741879 | 0.666356340381872 | 39.1071861330507 | 123.580001285739 | Guam |
| 1993 | 162.04399772749 | 0.667318181427308 | 39.2273216730114 | 122.816676054479 | Guam |
| 1994 | 157.243204 | 0.672111711813636 | 38.1744177460528 | 119.068786235547 | Guam |
| 1995 | 151.85140565718 | 0.678120215047177 | 37.485367038213 | 114.366038618967 | Guam |
| 1996 | 146.85499563552 | 0.68417373470043 | 37.590104704514 | 109.264890931006 | Guam |
| 1997 | 140.77523031375 | 0.691684870710781 | 36.5728489327091 | 104.202381381041 | Guam |
| 1998 | 136.69897765422 | 0.701819289007173 | 36.2948141920705 | 100.404163462149 | Guam |
| 1999 | 132.99496417604 | 0.713641593327183 | 31.8755546356362 | 101.119409540404 | Guam |
| 2000 | 129.81090815109 | 0.724802357160022 | 26.3099654273567 | 103.500942723733 | Guam |
| 2001 | 128.80066020533 | 0.734267375715648 | 21.531152052027 | 107.269508153303 | Guam |
| 2002 | 131.3916742 | 0.741284245225143 | 19.9857080286278 | 111.405966210872 | Guam |
| 2003 | 137.34952508517 | 0.745911530185679 | 19.0222150346629 | 118.327310050507 | Guam |
| 2004 | 143.94597550058 | 0.749016519617639 | 18.2327512595918 | 125.713224240988 | Guam |
| 2005 | 150.53334893081 | 0.750865874718511 | 18.1934233694546 | 132.339925561355 | Guam |
| 2006 | 156.99410653945 | 0.75117624504969 | 18.9328190101839 | 138.061287529266 | Guam |
| 2007 | 162.27521560277 | 0.752625470789067 | 18.5992390880046 | 143.675976514765 | Guam |
| 2008 | 165.45605575281 | 0.755152408307309 | 18.1563270597411 | 147.299728693069 | Guam |
| 2009 | 170.25535005077 | 0.75825229605245 | 16.3407337937438 | 153.914616257026 | Guam |
| 2010 | 175.067942 | 0.761484317334794 | 15.5117186831725 | 159.556223319728 | Guam |
| 2011 | 179.29137253708 | 0.763987394174646 | 15.1062677740033 | 164.185104763077 | Guam |
| 2012 | 182.88068073677 | 0.766845984308226 | 13.1021813208526 | 169.778499415917 | Guam |
| 2013 | 185.74756232574 | 0.770224956660541 | 12.9479968808222 | 172.799565444918 | Guam |
| 2014 | 189.22994763925 | 0.773441684088001 | 14.8884648864713 | 174.341482752779 | Guam |
| 2015 | 192.95744375954 | 0.776723614176227 | 11.9025360128818 | 181.054907746658 | Guam |
| 2016 | 193.32910829402 | 0.780511644317696 | 11.7503070591821 | 181.578801234838 | Guam |
| 2017 | 188.76487797425 | 0.78555492004746 | 11.1344211503752 | 177.630456823875 | Guam |
| 2018 | 186.91222762577 | 0.791294772555245 | 11.3091991014287 | 175.603028524341 | Guam |
| 2019 | 183.42104026077 | 0.796775451912585 | 10.5088657879036 | 172.912174472866 | Guam |
| 2020 | 169.63641566648 | 0.800969472273547 | 10.1073854681121 | 159.529030198368 | Guam |
| 2021 | 165.5983451 | 0.803982202863145 | 9.38438799713895 | 156.213957073561 | Guam |
| 1990 | 52.77360001 | 0.845495153448926 | 5.68751187899356 | 47.0860881260064 | Monaco |
| 1991 | 53.72078934 | 0.848303689705517 | 7.03264305372622 | 46.6881462881738 | Monaco |
| 1992 | 54.22819411 | 0.850863939877657 | 5.84381782333591 | 48.3843762874141 | Monaco |
| 1993 | 55.6830477 | 0.853264991769943 | 5.74414719584105 | 49.938900501069 | Monaco |
| 1994 | 56.78730637 | 0.855626926997873 | 6.71299335067187 | 50.0743130219181 | Monaco |
| 1995 | 57.84514387 | 0.857986147163975 | 6.687409507 | 51.157734361831 | Monaco |
| 1996 | 59.00572799 | 0.860245431724109 | 6.21526365785571 | 52.7904643303643 | Monaco |
| 1997 | 60.21622232 | 0.862446719992273 | 6.13267545998189 | 54.0835468607281 | Monaco |
| 1998 | 61.35489957 | 0.8647165130785 | 5.86455489141125 | 55.4903446752087 | Monaco |
| 1999 | 62.27811436 | 0.866842394455442 | 5.70932459617143 | 56.5687897634886 | Monaco |
| 2000 | 63.13374408 | 0.868973583095515 | 5.7140222698193 | 57.4197218116707 | Monaco |
| 2001 | 63.74030551 | 0.871094754955376 | 5.78510768558492 | 57.9551978275251 | Monaco |
| 2002 | 64.52803796 | 0.873182768875724 | 5.88340010606041 | 58.6446378571696 | Monaco |
| 2003 | 65.21416868 | 0.875197652216285 | 6.61214228738022 | 58.6020263966798 | Monaco |
| 2004 | 65.8637006 | 0.87723154881107 | 5.93686859586684 | 59.9268320018032 | Monaco |
| 2005 | 66.45087978 | 0.879288088865188 | 6.17346791783961 | 60.2774118605504 | Monaco |
| 2006 | 67.16967683 | 0.881274148230096 | 6.39788732533093 | 60.7717895049191 | Monaco |
| 2007 | 68.22839702 | 0.883262291250478 | 6.30665473643833 | 61.9217422830417 | Monaco |
| 2008 | 68.93555572 | 0.885331393874596 | 5.51989997597375 | 63.4156557405962 | Monaco |
| 2009 | 69.61164068 | 0.887328278515505 | 6.03886934291242 | 63.5727713415976 | Monaco |
| 2010 | 69.93865248 | 0.889262841317756 | 6.02994215892121 | 63.9087103236388 | Monaco |
| 2011 | 69.82073007 | 0.891237592262334 | 6.49593394500515 | 63.3247961251548 | Monaco |
| 2012 | 69.38467895 | 0.893073821857862 | 5.89414317597268 | 63.4905357739873 | Monaco |
| 2013 | 68.57367364 | 0.89486447196548 | 5.7761172470559 | 62.7975563905041 | Monaco |
| 2014 | 67.47013784 | 0.896547873900622 | 6.54050287549982 | 60.9296349686702 | Monaco |
| 2015 | 66.22670062 | 0.89835633653211 | 6.57867270037158 | 59.6480279214684 | Monaco |
| 2016 | 62.78224455 | 0.900051302707716 | 6.22508876912468 | 56.5571557853453 | Monaco |
| 2017 | 57.17321894 | 0.901752711895654 | 6.12846187863227 | 51.0447570611777 | Monaco |
| 2018 | 51.43453708 | 0.903388686597922 | 5.85143197694021 | 45.5831050991298 | Monaco |
| 2019 | 48.38737258 | 0.905030990844668 | 6.88146766561159 | 41.5059049111684 | Monaco |
| 2020 | 53.63726534 | 0.90668543677348 | 5.84980821448161 | 47.7874571272884 | Monaco |
| 2021 | 52.42412473 | 0.908262830989574 | 6.31454478388974 | 46.1095799451603 | Monaco |
| 1990 | 974.84900089714 | 0.539145980867897 | 156.787505188407 | 818.061495708733 | Nauru |
| 1991 | 967.25944397165 | 0.538433557023675 | 157.720283068157 | 809.539160903493 | Nauru |
| 1992 | 960.14039708055 | 0.537038892847501 | 156.583785836111 | 803.556611244439 | Nauru |
| 1993 | 977.72147077575 | 0.535059741994823 | 162.09813615158 | 815.62333462417 | Nauru |
| 1994 | 997.05535223379 | 0.533001096624389 | 171.823470570446 | 825.231881663344 | Nauru |
| 1995 | 1018.28093915152 | 0.530890925037636 | 180.356746944116 | 837.924192207404 | Nauru |
| 1996 | 1044.09860848583 | 0.528544473806068 | 180.833453854239 | 863.265154631591 | Nauru |
| 1997 | 1046.37448398628 | 0.526241589925116 | 189.918759029847 | 856.455724956433 | Nauru |
| 1998 | 1096.23365396216 | 0.523885302498396 | 189.648010679818 | 906.585643282342 | Nauru |
| 1999 | 1116.06228862588 | 0.521894324468662 | 199.042754178798 | 917.019534447082 | Nauru |
| 2000 | 1135.18087920142 | 0.52025488766476 | 198.953674111201 | 936.227205090219 | Nauru |
| 2001 | 1137.5595305141 | 0.519235424968716 | 200.409389119124 | 937.150141394976 | Nauru |
| 2002 | 1119.8144795657 | 0.518438705224942 | 203.955457395057 | 915.859022170643 | Nauru |
| 2003 | 1081.648506 | 0.518459986012372 | 204.028633239874 | 877.619873205126 | Nauru |
| 2004 | 1067.14412004762 | 0.518809540150096 | 203.51135972559 | 863.63276032203 | Nauru |
| 2005 | 1040.80001169144 | 0.51989286311802 | 200.163686931563 | 840.636324759877 | Nauru |
| 2006 | 1002.68579344658 | 0.522673178762107 | 198.429439299988 | 804.256354146592 | Nauru |
| 2007 | 979.94797451336 | 0.523201350232829 | 191.712331562387 | 788.235642950973 | Nauru |
| 2008 | 921.52733217707 | 0.526084096271176 | 190.261194359164 | 731.266137817906 | Nauru |
| 2009 | 915.73771438713 | 0.530221091913464 | 180.892778434143 | 734.844935952987 | Nauru |
| 2010 | 887.87858076365 | 0.535689994782251 | 161.018468166893 | 726.860112596757 | Nauru |
| 2011 | 844.59245956085 | 0.542342742706768 | 155.148364509132 | 689.444095051718 | Nauru |
| 2012 | 798.42736782654 | 0.549890620999273 | 146.287622767794 | 652.139745058746 | Nauru |
| 2013 | 734.97086851571 | 0.560112906115882 | 139.124297136983 | 595.846571378727 | Nauru |
| 2014 | 660.37636229765 | 0.571424363669984 | 81.7145753744478 | 578.661786923202 | Nauru |
| 2015 | 618.48030955898 | 0.581283234356014 | 81.2886912339994 | 537.191618324981 | Nauru |
| 2016 | 588.51257766024 | 0.590102988337037 | 80.0492657333998 | 508.46331192684 | Nauru |
| 2017 | 549.5243496 | 0.597619075639454 | 76.7487444707274 | 472.775605159273 | Nauru |
| 2018 | 515.66843017689 | 0.605311878971834 | 75.4447627934706 | 440.223667383419 | Nauru |
| 2019 | 494.58538088384 | 0.612697252950554 | 68.8514510578875 | 425.733929825952 | Nauru |
| 2020 | 468.8232314 | 0.619330076183652 | 59.7276973945366 | 409.095533970863 | Nauru |
| 2021 | 446.30449931998 | 0.625177833565553 | 60.3628452176385 | 385.941654102341 | Nauru |
| 1990 | 577.31685976164 | 0.587532983713503 | 82.4095456080523 | 494.907314153588 | Niue |
| 1991 | 579.13033683187 | 0.593882056791176 | 76.4677723854033 | 502.662564446467 | Niue |
| 1992 | 578.41779660826 | 0.599881243015555 | 74.6669195575643 | 503.750877050696 | Niue |
| 1993 | 576.58554360706 | 0.605514778865402 | 75.3213518427572 | 501.264191764303 | Niue |
| 1994 | 578.6235641 | 0.610650175126661 | 69.1254532731163 | 509.498110826984 | Niue |
| 1995 | 579.17301419097 | 0.615374609096138 | 68.1271977879641 | 511.045816403006 | Niue |
| 1996 | 583.76264504777 | 0.619734554803463 | 63.1083910130897 | 520.65425403468 | Niue |
| 1997 | 570.32019393191 | 0.623913847776035 | 62.1270692886005 | 508.19312464331 | Niue |
| 1998 | 590.08989880702 | 0.627578934289331 | 59.8463280923901 | 530.24357071463 | Niue |
| 1999 | 584.57658439076 | 0.63085717205161 | 54.9798784638629 | 529.596705926897 | Niue |
| 2000 | 572.32562022583 | 0.634068950171114 | 53.6428451193283 | 518.682775106502 | Niue |
| 2001 | 556.71688888046 | 0.637366627073445 | 50.3557633590415 | 506.361125521419 | Niue |
| 2002 | 528.12046577616 | 0.641122021248484 | 49.9901859047498 | 478.13027987141 | Niue |
| 2003 | 498.60463986576 | 0.645779539849075 | 49.9144186191706 | 448.690221246589 | Niue |
| 2004 | 474.8851541 | 0.650614832382256 | 47.9901576286401 | 426.89499647986 | Niue |
| 2005 | 443.90811355936 | 0.657281264925745 | 45.3753697851771 | 398.532743774183 | Niue |
| 2006 | 417.6085302 | 0.664162983137176 | 40.1905220905319 | 377.418008065568 | Niue |
| 2007 | 398.14494999159 | 0.670199299468215 | 38.5851418757922 | 359.559808115798 | Niue |
| 2008 | 381.8025985 | 0.67639685901135 | 37.7909709205827 | 344.011627609317 | Niue |
| 2009 | 375.86187157706 | 0.682579172713086 | 37.5386140918136 | 338.323257485246 | Niue |
| 2010 | 378.4981385 | 0.686280630120473 | 37.2535541414483 | 341.244584336652 | Niue |
| 2011 | 382.13882829827 | 0.690315577811745 | 36.6406232084751 | 345.498205089795 | Niue |
| 2012 | 381.55136152258 | 0.694150948675056 | 36.4997578679526 | 345.051603654627 | Niue |
| 2013 | 381.72543256336 | 0.697221077939175 | 35.6339761962736 | 346.091456367086 | Niue |
| 2014 | 379.76832517211 | 0.700545923914193 | 35.7881327 | 343.9801925 | Niue |
| 2015 | 379.48654933366 | 0.704232979022297 | 34.8923088377265 | 344.594240495933 | Niue |
| 2016 | 297.63156289437 | 0.707476099423504 | 34.4444663421091 | 263.187096552261 | Niue |
| 2017 | 213.12470750158 | 0.711220995949878 | 32.6401748026879 | 180.484532698892 | Niue |
| 2018 | 131.8219765 | 0.7151082296411 | 31.0653904688942 | 100.756586040106 | Niue |
| 2019 | 94.41433703 | 0.719078885206029 | 29.4503540964079 | 64.9639829303921 | Niue |
| 2020 | 293.51944085537 | 0.722790249813625 | 28.1061741394042 | 265.413266715966 | Niue |
| 2021 | 250.52950454973 | 0.726222049709539 | 25.8516549569426 | 224.677849592787 | Niue |
| 1990 | 146.34754055528 | 0.708593838127328 | 34.6889867832793 | 111.658553772001 | Northern Mariana Islands |
| 1991 | 143.78712335371 | 0.712223642490096 | 31.4436347786533 | 112.343488575057 | Northern Mariana Islands |
| 1992 | 136.68294294981 | 0.715787607029664 | 32.0273762877818 | 104.655566662028 | Northern Mariana Islands |
| 1993 | 127.19673302431 | 0.719179395545622 | 28.4270469108107 | 98.7696861134993 | Northern Mariana Islands |
| 1994 | 118.31861551462 | 0.72251049388152 | 27.8823200711265 | 90.4362954434935 | Northern Mariana Islands |
| 1995 | 113.31150216773 | 0.726219053033259 | 26.6874637588874 | 86.6240384088426 | Northern Mariana Islands |
| 1996 | 114.74680658664 | 0.730074085649478 | 24.7862403064076 | 89.9605662802324 | Northern Mariana Islands |
| 1997 | 121.08222187035 | 0.733795365689826 | 23.2356083656165 | 97.8466135047335 | Northern Mariana Islands |
| 1998 | 129.93715314414 | 0.737423126925004 | 22.7810068966998 | 107.15614624744 | Northern Mariana Islands |
| 1999 | 135.35533478087 | 0.740934815031098 | 19.579844895422 | 115.775489885448 | Northern Mariana Islands |
| 2000 | 134.87505102787 | 0.744492865066434 | 20.2349460010981 | 114.640105026772 | Northern Mariana Islands |
| 2001 | 129.46076110379 | 0.747170605600551 | 20.483817345859 | 108.976943757931 | Northern Mariana Islands |
| 2002 | 120.28576157761 | 0.748755188793797 | 18.9347463966559 | 101.351015180954 | Northern Mariana Islands |
| 2003 | 109.50995302358 | 0.750171533040464 | 18.6730011742221 | 90.8369518493579 | Northern Mariana Islands |
| 2004 | 102.72828199181 | 0.751528840504646 | 18.8490550461969 | 83.8792269456131 | Northern Mariana Islands |
| 2005 | 102.38315851452 | 0.751826692283402 | 18.5680991305325 | 83.8150593839875 | Northern Mariana Islands |
| 2006 | 108.43381704975 | 0.751797809045081 | 18.6206423406474 | 89.8131747091026 | Northern Mariana Islands |
| 2007 | 114.57626118773 | 0.751608416153259 | 18.2476386822384 | 96.3286225054916 | Northern Mariana Islands |
| 2008 | 117.57855140507 | 0.750807191804257 | 18.561284189151 | 99.017267215919 | Northern Mariana Islands |
| 2009 | 118.08252525041 | 0.748597173133935 | 19.6864329458327 | 98.3960923045773 | Northern Mariana Islands |
| 2010 | 114.89388588536 | 0.746617261581083 | 18.8835381169626 | 96.0103477683975 | Northern Mariana Islands |
| 2011 | 111.75517099645 | 0.744411908430807 | 21.0226949221447 | 90.7324760743053 | Northern Mariana Islands |
| 2012 | 110.50482564952 | 0.742937350752288 | 19.9136066358493 | 90.5912190136707 | Northern Mariana Islands |
| 2013 | 111.4546346 | 0.742284360325222 | 21.0663921039945 | 90.3882424771055 | Northern Mariana Islands |
| 2014 | 114.21477729468 | 0.742605449321406 | 20.1471503454182 | 94.0676269492618 | Northern Mariana Islands |
| 2015 | 118.87651143323 | 0.743797398071258 | 20.97300078 | 97.90351065 | Northern Mariana Islands |
| 2016 | 119.91834912605 | 0.748455465810463 | 18.5881832785139 | 101.330165847536 | Northern Mariana Islands |
| 2017 | 115.83649406299 | 0.756495478910201 | 17.7710353819308 | 98.0654586810592 | Northern Mariana Islands |
| 2018 | 113.61476126327 | 0.761255400013642 | 14.8045700111852 | 98.8101912520848 | Northern Mariana Islands |
| 2019 | 110.87402270595 | 0.765770647172973 | 14.6171092348492 | 96.2569134711008 | Northern Mariana Islands |
| 2020 | 107.03050342866 | 0.76942765953698 | 15.0416269047142 | 91.9888765239458 | Northern Mariana Islands |
| 2021 | 101.92964398319 | 0.771535213383281 | 13.4274542018245 | 88.5021897813655 | Northern Mariana Islands |
| 1990 | 1195.87220396458 | 0.662909509792389 | 40.6653292990178 | 1155.20687466556 | Palau |
| 1991 | 1127.35871582742 | 0.668230731109012 | 39.123273631296 | 1088.23544219612 | Palau |
| 1992 | 1064.03101005294 | 0.673302462400621 | 37.8627009323648 | 1026.16830912058 | Palau |
| 1993 | 1020.38889240798 | 0.676648715394758 | 37.9320703934769 | 982.456822014503 | Palau |
| 1994 | 978.26691036988 | 0.680195774851961 | 37.6219635853718 | 940.644946784508 | Palau |
| 1995 | 930.33199780424 | 0.684832139585057 | 37.4876007714787 | 892.844397032761 | Palau |
| 1996 | 884.52905176182 | 0.69009567251107 | 36.5814877822253 | 847.947563979595 | Palau |
| 1997 | 827.09735579651 | 0.695122122982618 | 36.5898792121242 | 790.507476584386 | Palau |
| 1998 | 793.7912226 | 0.69965290951971 | 35.6709090603954 | 758.120313518505 | Palau |
| 1999 | 773.73520440119 | 0.703115321964044 | 34.1387353895515 | 739.596469011638 | Palau |
| 2000 | 750.66532575743 | 0.70519694495869 | 35.0153441718178 | 715.649981585612 | Palau |
| 2001 | 730.11632277311 | 0.707402386499998 | 34.5208560615529 | 695.595466711557 | Palau |
| 2002 | 715.34720316795 | 0.709587643886923 | 33.5557102879668 | 681.791492879983 | Palau |
| 2003 | 704.59958373054 | 0.711000220048301 | 31.9809934597581 | 672.618590270782 | Palau |
| 2004 | 701.41018508062 | 0.712543811157683 | 31.8860024444652 | 669.524182636155 | Palau |
| 2005 | 690.97407686892 | 0.71433983112227 | 31.9661956223031 | 659.007881246617 | Palau |
| 2006 | 655.34125098522 | 0.716353431401992 | 31.4262257386314 | 623.915025246589 | Palau |
| 2007 | 577.76485337009 | 0.718809541917246 | 29.7821314462525 | 547.982721923838 | Palau |
| 2008 | 429.84411674358 | 0.720806622774243 | 29.5371792970531 | 400.306937446527 | Palau |
| 2009 | 358.2152776 | 0.722288266788472 | 29.6373315906734 | 328.577946013327 | Palau |
| 2010 | 303.75779381541 | 0.724065956349117 | 28.7329626941961 | 275.024831121214 | Palau |
| 2011 | 267.08425985741 | 0.726733445911764 | 25.9582951926599 | 241.12596466475 | Palau |
| 2012 | 237.69171716459 | 0.729612899417556 | 24.7066994143928 | 212.985017750197 | Palau |
| 2013 | 215.09317008814 | 0.731953474727171 | 24.1325024066095 | 190.96066768153 | Palau |
| 2014 | 195.69849773082 | 0.734768296510748 | 20.5485689914486 | 175.149928739371 | Palau |
| 2015 | 182.79794830588 | 0.73816642523263 | 21.5283287784229 | 161.269619527457 | Palau |
| 2016 | 172.80060341719 | 0.741361910823289 | 20.7902481752725 | 152.010355241917 | Palau |
| 2017 | 162.58512532099 | 0.744224767037247 | 18.3098467322031 | 144.275278588787 | Palau |
| 2018 | 152.24056440232 | 0.747761249656357 | 18.730946818235 | 133.509617584085 | Palau |
| 2019 | 147.31971428465 | 0.750715306594841 | 20.2121013877224 | 127.107612896928 | Palau |
| 2020 | 164.30531611924 | 0.75278092905906 | 17.0686941492653 | 147.236621969975 | Palau |
| 2021 | 138.99102005857 | 0.75404693141209 | 18.9058406188436 | 120.085179439726 | Palau |
| 1990 | 151.39499875661 | 0.658758145866254 | 45.6368305577222 | 105.758168198888 | Puerto Rico |
| 1991 | 140.63258309435 | 0.66326172041123 | 40.3843029773316 | 100.248280117018 | Puerto Rico |
| 1992 | 149.07325315301 | 0.667272191591073 | 38.9088134501744 | 110.164439702836 | Puerto Rico |
| 1993 | 167.08580761541 | 0.671024105168892 | 38.3895481659191 | 128.696259449491 | Puerto Rico |
| 1994 | 160.15611390546 | 0.675450810240548 | 38.2134431250931 | 121.942670780367 | Puerto Rico |
| 1995 | 161.63969341513 | 0.680673949739819 | 37.4931908449303 | 124.1465026 | Puerto Rico |
| 1996 | 140.03525236111 | 0.685865893043239 | 37.3083533040817 | 102.726899057028 | Puerto Rico |
| 1997 | 135.19987278856 | 0.691274381965422 | 36.623535569796 | 98.576337218764 | Puerto Rico |
| 1998 | 117.72427072135 | 0.697040445469226 | 35.5284613427316 | 82.1958093786184 | Puerto Rico |
| 1999 | 104.02519718531 | 0.702850670729254 | 35.94361179 | 68.0815854 | Puerto Rico |
| 2000 | 91.50714219 | 0.710035652009262 | 33.8336088804549 | 57.6735333077051 | Puerto Rico |
| 2001 | 74.29566771 | 0.718955861108189 | 28.5674840136024 | 45.7281836949476 | Puerto Rico |
| 2002 | 71.79072377 | 0.725956855617272 | 28.167215195916 | 43.623508571554 | Puerto Rico |
| 2003 | 68.32387156 | 0.730475137768497 | 25.0645237659957 | 43.2593477924743 | Puerto Rico |
| 2004 | 66.09032563 | 0.734219545590062 | 22.090859435316 | 43.999466198604 | Puerto Rico |
| 2005 | 67.38300237 | 0.737742266137191 | 20.4007406212366 | 46.9822617469634 | Puerto Rico |
| 2006 | 64.40911266 | 0.741629431374531 | 20.8731373507778 | 43.5359753088622 | Puerto Rico |
| 2007 | 61.97270349 | 0.74588380735408 | 19.1269226400988 | 42.8457808463512 | Puerto Rico |
| 2008 | 62.36635414 | 0.750922167274437 | 19.1859950917778 | 43.1803590530322 | Puerto Rico |
| 2009 | 62.92588813 | 0.756361694934789 | 17.6016593109967 | 45.3242288190133 | Puerto Rico |
| 2010 | 58.61457908 | 0.761763253126039 | 14.3989520682072 | 44.2156270120028 | Puerto Rico |
| 2011 | 61.04689488 | 0.766784881779412 | 13.4877515066051 | 47.5591433739749 | Puerto Rico |
| 2012 | 64.14884862 | 0.772311804167716 | 12.2742232283698 | 51.8746253939802 | Puerto Rico |
| 2013 | 55.63546657 | 0.778461161794537 | 13.4387620438276 | 42.1967045250824 | Puerto Rico |
| 2014 | 58.85889934 | 0.785120344673962 | 11.6759890433471 | 47.1829103001029 | Puerto Rico |
| 2015 | 52.88506378 | 0.791800166848976 | 12.3705261945596 | 40.5145375810804 | Puerto Rico |
| 2016 | 42.76264582 | 0.799084082615032 | 10.8105371463657 | 31.9521086747743 | Puerto Rico |
| 2017 | 36.85215495 | 0.806648960516056 | 10.4354625857179 | 26.4166923620021 | Puerto Rico |
| 2018 | 50.43915321 | 0.812906066317222 | 9.05758511176028 | 41.3815681020197 | Puerto Rico |
| 2019 | 52.12613207 | 0.818843190899062 | 8.58230789893725 | 43.5438241737427 | Puerto Rico |
| 2020 | 54.14759799 | 0.822918436314863 | 9.03305384297333 | 45.1145441493067 | Puerto Rico |
| 2021 | 47.63795376 | 0.825525846914029 | 8.19863241999784 | 39.4393213414522 | Puerto Rico |
| 1990 | 1947.08256398547 | 0.580685876593206 | 83.0586846555354 | 1864.02387932993 | Saint Kitts and Nevis |
| 1991 | 1640.36168235019 | 0.590254950402484 | 80.1418040388496 | 1560.21987831134 | Saint Kitts and Nevis |
| 1992 | 1560.93343298509 | 0.599187189594131 | 76.1552895419434 | 1484.77814344315 | Saint Kitts and Nevis |
| 1993 | 1597.66887373011 | 0.607235891628758 | 75.7259425663016 | 1521.94293116381 | Saint Kitts and Nevis |
| 1994 | 1525.13168940378 | 0.614514657962512 | 69.0203269877004 | 1456.11136241608 | Saint Kitts and Nevis |
| 1995 | 1406.47702405684 | 0.620716497645849 | 62.4908745620825 | 1343.98614949476 | Saint Kitts and Nevis |
| 1996 | 1303.58316225422 | 0.626207410919295 | 60.4093847385235 | 1243.1737775157 | Saint Kitts and Nevis |
| 1997 | 1135.62511805306 | 0.631274740057709 | 51.6871451759653 | 1083.93797287709 | Saint Kitts and Nevis |
| 1998 | 1051.73875611314 | 0.635539987956464 | 53.3974522928806 | 998.341303820259 | Saint Kitts and Nevis |
| 1999 | 859.52282043982 | 0.63971373866775 | 50.0629431483562 | 809.459877291464 | Saint Kitts and Nevis |
| 2000 | 739.46843191687 | 0.644353376846687 | 50.7805248012677 | 688.687907115602 | Saint Kitts and Nevis |
| 2001 | 606.70355613845 | 0.64922665301934 | 47.3601598871218 | 559.343396251328 | Saint Kitts and Nevis |
| 2002 | 546.60109296927 | 0.654593408970176 | 45.5133986240842 | 501.087694345186 | Saint Kitts and Nevis |
| 2003 | 519.99665118735 | 0.659925311695556 | 44.3963130890503 | 475.6003381 | Saint Kitts and Nevis |
| 2004 | 503.4564291 | 0.667568129909661 | 39.159130725774 | 464.297298407826 | Saint Kitts and Nevis |
| 2005 | 524.06501890449 | 0.679145457347323 | 37.5045802429616 | 486.560438661528 | Saint Kitts and Nevis |
| 2006 | 531.83873819498 | 0.690078666648128 | 36.3280775676258 | 495.510660627354 | Saint Kitts and Nevis |
| 2007 | 518.7870405 | 0.697727627549003 | 35.7848595808839 | 483.002180892416 | Saint Kitts and Nevis |
| 2008 | 496.64972156235 | 0.704141493783467 | 34.3782180832475 | 462.271503479103 | Saint Kitts and Nevis |
| 2009 | 490.33321922588 | 0.709499498252707 | 33.4487314581315 | 456.884487767749 | Saint Kitts and Nevis |
| 2010 | 472.81503315705 | 0.714460415458321 | 32.0384166268467 | 440.776616530203 | Saint Kitts and Nevis |
| 2011 | 506.28083792769 | 0.719324380918298 | 29.6725781789976 | 476.608259748692 | Saint Kitts and Nevis |
| 2012 | 482.41504590984 | 0.723436152222763 | 28.7273202980453 | 453.687725611795 | Saint Kitts and Nevis |
| 2013 | 493.97803862367 | 0.726706592636212 | 26.3166362876956 | 467.661402335974 | Saint Kitts and Nevis |
| 2014 | 528.87399385048 | 0.729530218928803 | 25.145830367237 | 503.728163483243 | Saint Kitts and Nevis |
| 2015 | 496.16978481486 | 0.732220671984012 | 23.432528037682 | 472.737256777178 | Saint Kitts and Nevis |
| 2016 | 478.44854754811 | 0.735566138078794 | 21.3348280162792 | 457.113719531831 | Saint Kitts and Nevis |
| 2017 | 450.16469153499 | 0.739470681017895 | 20.6815372184484 | 429.483154316542 | Saint Kitts and Nevis |
| 2018 | 425.50685395834 | 0.743736461382785 | 19.8739659076605 | 405.63288805068 | Saint Kitts and Nevis |
| 2019 | 404.04240022457 | 0.748152066317114 | 18.669370302515 | 385.373029922055 | Saint Kitts and Nevis |
| 2020 | 372.3839339 | 0.75156840922289 | 17.8283779540369 | 354.555555979363 | Saint Kitts and Nevis |
| 2021 | 366.67441774646 | 0.754987054945241 | 17.7354275922587 | 348.938990154201 | Saint Kitts and Nevis |
| 1990 | 52.82521929 | 0.813244887891478 | 9.98270232608659 | 42.8425169609834 | San Marino |
| 1991 | 53.45408988 | 0.818282512759725 | 9.31032184496525 | 44.1437680392647 | San Marino |
| 1992 | 53.99062035 | 0.822782757963244 | 8.3894666406197 | 45.6011537044803 | San Marino |
| 1993 | 54.70309955 | 0.827382324106265 | 8.894503218 | 45.808596328105 | San Marino |
| 1994 | 55.30680138 | 0.8319709830207 | 6.67179265415673 | 48.6350087219033 | San Marino |
| 1995 | 56.05481355 | 0.836762725678003 | 6.33526566242378 | 49.7195478830262 | San Marino |
| 1996 | 56.71378676 | 0.841389494577046 | 6.35455934943472 | 50.3592274113153 | San Marino |
| 1997 | 57.26866261 | 0.845864864873086 | 5.66111030408195 | 51.607552306078 | San Marino |
| 1998 | 57.80579708 | 0.850474771150175 | 5.600224286 | 52.205572791905 | San Marino |
| 1999 | 58.32680828 | 0.855338613208904 | 6.28422162122579 | 52.0425866564142 | San Marino |
| 2000 | 59.04532507 | 0.859695363255401 | 6.06432437735372 | 52.9810006891863 | San Marino |
| 2001 | 59.56839102 | 0.863747756857469 | 6.55082706006154 | 53.0175639572785 | San Marino |
| 2002 | 60.37869331 | 0.865680591501536 | 6.52075689946227 | 53.8579364102877 | San Marino |
| 2003 | 60.95631277 | 0.867522008779968 | 5.87472444926929 | 55.0815883229207 | San Marino |
| 2004 | 61.72058164 | 0.869287134173222 | 6.15628812159771 | 55.5642935214723 | San Marino |
| 2005 | 62.40852753 | 0.87097959576402 | 6.01750282213944 | 56.3910247113306 | San Marino |
| 2006 | 63.00056745 | 0.872652149736731 | 5.89382819815462 | 57.1067392503054 | San Marino |
| 2007 | 63.87601281 | 0.874307253083233 | 5.60511939452911 | 58.2708934176509 | San Marino |
| 2008 | 64.84081971 | 0.875991881471404 | 6.166738895 | 58.674080813458 | San Marino |
| 2009 | 65.45722715 | 0.877705228262122 | 6.18334713519678 | 59.2738800182932 | San Marino |
| 2010 | 65.74891388 | 0.879440813617125 | 5.89926357871862 | 59.8496503039814 | San Marino |
| 2011 | 65.65821532 | 0.881197517807579 | 6.50261165102196 | 59.155603673278 | San Marino |
| 2012 | 65.35730654 | 0.88292453140369 | 6.69714814338455 | 58.6601583985855 | San Marino |
| 2013 | 64.90239381 | 0.884592626900131 | 6.04612075715808 | 58.8562730538619 | San Marino |
| 2014 | 64.18896537 | 0.885353693736433 | 6.06699801186982 | 58.1219673608502 | San Marino |
| 2015 | 63.43550664 | 0.884693365070092 | 6.03172192747788 | 57.4037847106921 | San Marino |
| 2016 | 60.64006978 | 0.884477664772925 | 6.28249978105918 | 54.3575699985608 | San Marino |
| 2017 | 55.25726444 | 0.884557606743879 | 5.778481006 | 49.478783437943 | San Marino |
| 2018 | 49.82331849 | 0.885133515535323 | 5.7380596148364 | 44.0852588776136 | San Marino |
| 2019 | 46.8834044 | 0.886200660194545 | 6.49120906064932 | 40.3921953402407 | San Marino |
| 2020 | 45.61578501 | 0.886867937194416 | 6.5564056033418 | 39.0593794022182 | San Marino |
| 2021 | 43.66888965 | 0.888005473958106 | 5.95715215896732 | 37.7117374884027 | San Marino |
| 1990 | 1570.87427145654 | 0.521942386048128 | 198.468090147236 | 1372.4061813093 | Tokelau |
| 1991 | 1415.93344793232 | 0.526628473491586 | 189.980043936034 | 1225.95340399629 | Tokelau |
| 1992 | 1292.85701276666 | 0.531307850030329 | 181.919005591335 | 1110.93800717533 | Tokelau |
| 1993 | 1205.71036339887 | 0.535935005438943 | 161.356535836296 | 1044.35382756257 | Tokelau |
| 1994 | 1141.2656247225 | 0.540523664725072 | 156.32417880666 | 984.94144591584 | Tokelau |
| 1995 | 1094.80372971008 | 0.545295634483764 | 151.439513379234 | 943.364216330846 | Tokelau |
| 1996 | 1058.28764177272 | 0.55028265171702 | 146.609609099811 | 911.678032672909 | Tokelau |
| 1997 | 982.7964484 | 0.555631325529965 | 143.271439846386 | 839.525008519614 | Tokelau |
| 1998 | 932.02578005693 | 0.561047610799914 | 138.716888199681 | 793.308891857249 | Tokelau |
| 1999 | 942.37821756621 | 0.566415405899331 | 109.521166529502 | 832.857051036708 | Tokelau |
| 2000 | 887.76974853107 | 0.571814369871981 | 83.2697236482136 | 804.500024882856 | Tokelau |
| 2001 | 817.98437746058 | 0.576915597204096 | 84.4410428634176 | 733.543334597162 | Tokelau |
| 2002 | 752.46773691579 | 0.581858317064991 | 82.0122035835262 | 670.455533332264 | Tokelau |
| 2003 | 679.43821276325 | 0.586854346188713 | 82.4371652892629 | 597.001047473987 | Tokelau |
| 2004 | 632.5024076 | 0.592342442430319 | 80.7363384501169 | 551.766069189083 | Tokelau |
| 2005 | 570.891568 | 0.59817611685198 | 77.7445720448018 | 493.146995967298 | Tokelau |
| 2006 | 521.38186470388 | 0.604429389636372 | 75.6475566864957 | 445.734308017384 | Tokelau |
| 2007 | 480.29107680781 | 0.61116323669051 | 68.7682772075786 | 411.522799600231 | Tokelau |
| 2008 | 443.24354101256 | 0.617940246542751 | 62.7851764143256 | 380.458364598234 | Tokelau |
| 2009 | 408.87246609477 | 0.624070078096847 | 59.2170968444112 | 349.655369250359 | Tokelau |
| 2010 | 404.86644173604 | 0.630175540158128 | 56.7751582527364 | 348.091283483304 | Tokelau |
| 2011 | 396.95641628288 | 0.636016518005944 | 52.4008453795247 | 344.555570903355 | Tokelau |
| 2012 | 386.49362462651 | 0.641611991197784 | 50.9673132334173 | 335.526311393093 | Tokelau |
| 2013 | 369.83797655255 | 0.647966148124354 | 49.8463385754879 | 319.991637977062 | Tokelau |
| 2014 | 356.27129962895 | 0.653231999411508 | 45.5767947581903 | 310.69450487076 | Tokelau |
| 2015 | 346.7551569 | 0.658798994926187 | 45.8017661306074 | 300.953390745993 | Tokelau |
| 2016 | 340.6296754 | 0.663974319058952 | 40.0391310989701 | 300.59054434293 | Tokelau |
| 2017 | 327.79096938256 | 0.669054133759442 | 38.6284438566262 | 289.162525525934 | Tokelau |
| 2018 | 319.26885719462 | 0.674179514031409 | 37.8041930083201 | 281.4646642 | Tokelau |
| 2019 | 311.71162342668 | 0.679247839201533 | 37.4154675950349 | 274.296155831645 | Tokelau |
| 2020 | 2760.60104102869 | 0.682937378311047 | 37.435174221549 | 2723.16586680714 | Tokelau |
| 2021 | 2797.99091532588 | 0.68642562083005 | 37.2055942010221 | 2760.78532112486 | Tokelau |
| 1990 | 2504.58550219701 | 0.406247565957081 | 867.873580841955 | 1636.71192135506 | Tuvalu |
| 1991 | 2203.74080268517 | 0.414819936897447 | 826.842188983706 | 1376.89861370146 | Tuvalu |
| 1992 | 1952.35066519858 | 0.422901979744667 | 691.448037435119 | 1260.90262776346 | Tuvalu |
| 1993 | 1750.82165650652 | 0.430518260028408 | 429.004911950773 | 1321.81674455575 | Tuvalu |
| 1994 | 1556.39168614228 | 0.438563050767836 | 431.305037615371 | 1125.08664852691 | Tuvalu |
| 1995 | 1431.49689513567 | 0.444962241977075 | 420.741295355689 | 1010.75559977998 | Tuvalu |
| 1996 | 1341.82779790062 | 0.449804979978272 | 407.525397345415 | 934.302400555205 | Tuvalu |
| 1997 | 1196.43058088072 | 0.455368427922084 | 405.944032758432 | 790.486548122288 | Tuvalu |
| 1998 | 1071.94288180407 | 0.462454678093366 | 378.768882618302 | 693.173999185768 | Tuvalu |
| 1999 | 1026.74121214707 | 0.468845801641229 | 344.34196399891 | 682.39924814816 | Tuvalu |
| 2000 | 917.84244572246 | 0.474956823462581 | 335.011026481076 | 582.831419241384 | Tuvalu |
| 2001 | 821.63271754034 | 0.480850886398825 | 325.240854616628 | 496.391862923712 | Tuvalu |
| 2002 | 740.17729486823 | 0.487252252447393 | 317.807969718503 | 422.369325149727 | Tuvalu |
| 2003 | 674.01111293544 | 0.492754711063713 | 297.969680591609 | 376.041432343831 | Tuvalu |
| 2004 | 593.27851034485 | 0.497692152366724 | 236.046894222685 | 357.231616122165 | Tuvalu |
| 2005 | 562.53802767452 | 0.501851309525404 | 229.69409746375 | 332.84393021077 | Tuvalu |
| 2006 | 585.20467708636 | 0.505975410569688 | 221.231236750406 | 363.973440335954 | Tuvalu |
| 2007 | 556.4850339 | 0.51050430052828 | 213.20535318532 | 343.27968071828 | Tuvalu |
| 2008 | 519.56749057398 | 0.515611600817149 | 204.475579 | 315.09191161528 | Tuvalu |
| 2009 | 510.06220132815 | 0.519870565592834 | 198.723889501577 | 311.338311826573 | Tuvalu |
| 2010 | 505.09902868361 | 0.52345550974778 | 188.958643675971 | 316.140385007639 | Tuvalu |
| 2011 | 489.22190445507 | 0.527744244866808 | 180.306060040432 | 308.915844414638 | Tuvalu |
| 2012 | 488.58809544783 | 0.531203234840239 | 179.589549532345 | 308.998545915485 | Tuvalu |
| 2013 | 482.83192895705 | 0.53516623462033 | 161.61271694969 | 321.21921200736 | Tuvalu |
| 2014 | 471.975516 | 0.539122244261915 | 156.609465327175 | 315.366050674125 | Tuvalu |
| 2015 | 467.21707280532 | 0.544047880204377 | 155.355825929508 | 311.861246875812 | Tuvalu |
| 2016 | 460.69195072402 | 0.549173928060589 | 146.531625762287 | 314.160324961732 | Tuvalu |
| 2017 | 430.82880403442 | 0.554466480141161 | 143.934098586052 | 286.894705448368 | Tuvalu |
| 2018 | 405.78092404121 | 0.559859395089525 | 138.974496406816 | 266.806427634394 | Tuvalu |
| 2019 | 378.61032184458 | 0.566045488622333 | 113.715022737388 | 264.895299107192 | Tuvalu |
| 2020 | 355.72755420806 | 0.571666102458017 | 88.2914056120417 | 267.436148596018 | Tuvalu |
| 2021 | 335.95111223508 | 0.576620529344684 | 84.7403072416584 | 251.210804993422 | Tuvalu |
| 1990 | 231.48221438697 | 0.655160855636097 | 45.9592228169161 | 185.522991570054 | United States Virgin Islands |
| 1991 | 216.75275756595 | 0.664018659064364 | 40.2174915137476 | 176.535266052202 | United States Virgin Islands |
| 1992 | 206.01926815516 | 0.67108187072399 | 38.7042213046058 | 167.315046850554 | United States Virgin Islands |
| 1993 | 197.24536131134 | 0.676872744796339 | 37.6872525681765 | 159.558108743163 | United States Virgin Islands |
| 1994 | 188.76000639337 | 0.681982223083549 | 37.4263966112944 | 151.333609782076 | United States Virgin Islands |
| 1995 | 179.43713911261 | 0.686730210603479 | 37.4771709666763 | 141.959968145934 | United States Virgin Islands |
| 1996 | 170.26720018545 | 0.691247698919766 | 36.4270263745139 | 133.840173810936 | United States Virgin Islands |
| 1997 | 161.84717389356 | 0.69559820385004 | 36.5110260563676 | 125.336147837192 | United States Virgin Islands |
| 1998 | 153.4236403 | 0.699768685957964 | 36.0783642962148 | 117.345275968285 | United States Virgin Islands |
| 1999 | 145.89609717378 | 0.703949341926433 | 34.9777171993902 | 110.91837997439 | United States Virgin Islands |
| 2000 | 138.65977348781 | 0.708436564859144 | 34.545623549166 | 104.114149938644 | United States Virgin Islands |
| 2001 | 132.27379572074 | 0.713751114883336 | 31.8750043012348 | 100.398791419505 | United States Virgin Islands |
| 2002 | 123.87132817594 | 0.724398272335199 | 29.4045323380449 | 94.4667958378951 | United States Virgin Islands |
| 2003 | 118.56888104492 | 0.734609849926843 | 21.1167619191813 | 97.4521191257387 | United States Virgin Islands |
| 2004 | 114.57965580884 | 0.744613598166223 | 20.3793157776447 | 94.2003400311953 | United States Virgin Islands |
| 2005 | 111.20806785985 | 0.754267557852673 | 17.941883910629 | 93.266183949221 | United States Virgin Islands |
| 2006 | 107.22188055648 | 0.763447495711391 | 13.4487392009563 | 93.7731413555237 | United States Virgin Islands |
| 2007 | 102.97781265643 | 0.772180593452306 | 12.8729158786567 | 90.1048967777733 | United States Virgin Islands |
| 2008 | 98.13951096 | 0.780342622959945 | 12.6204224566244 | 85.5190884996556 | United States Virgin Islands |
| 2009 | 94.18747363 | 0.787055145666672 | 10.4259395791198 | 83.7615340526002 | United States Virgin Islands |
| 2010 | 90.22232808 | 0.793219696295781 | 10.6464599208998 | 79.5758681606302 | United States Virgin Islands |
| 2011 | 86.46486783 | 0.798071698185583 | 10.4800373970984 | 75.9848304294916 | United States Virgin Islands |
| 2012 | 81.27096885 | 0.80109285050392 | 10.0598267832997 | 71.2111420683103 | United States Virgin Islands |
| 2013 | 75.2041096 | 0.803405789381199 | 10.7225788016598 | 64.4815308002102 | United States Virgin Islands |
| 2014 | 69.71483927 | 0.805641378374046 | 10.1494044318103 | 59.5654348336897 | United States Virgin Islands |
| 2015 | 64.62472993 | 0.807889245154254 | 9.66070308961744 | 54.9640268452226 | United States Virgin Islands |
| 2016 | 60.71880853 | 0.810199044380962 | 9.53937041039825 | 51.1794381175717 | United States Virgin Islands |
| 2017 | 57.25010812 | 0.812405171667542 | 8.38746348695706 | 48.8626446353729 | United States Virgin Islands |
| 2018 | 52.82977569 | 0.814825439654601 | 8.879171634 | 43.950604059421 | United States Virgin Islands |
| 2019 | 51.08940511 | 0.817429008110061 | 8.75780998945154 | 42.3315951225285 | United States Virgin Islands |
| 2020 | 50.5769877 | 0.819601712348942 | 9.75861845542368 | 40.8183692396163 | United States Virgin Islands |
| 2021 | 46.57484563 | 0.821830853383701 | 7.68884251567841 | 38.8860031116516 | United States Virgin Islands |
| 1990 | 33351.5372690725 | 0.20665649952096 | 6622.03991578007 | 26729.4973532924 | South Sudan |
| 1991 | 32392.2163077844 | 0.208932715815866 | 6176.64808287001 | 26215.5682249144 | South Sudan |
| 1992 | 31768.1570417513 | 0.21119940064631 | 6153.84251458351 | 25614.3145271678 | South Sudan |
| 1993 | 30998.5371747319 | 0.213416470699326 | 5614.31207073189 | 25384.2251 | South Sudan |
| 1994 | 30019.7702014405 | 0.21561208907535 | 5570.4160767579 | 24449.3541246826 | South Sudan |
| 1995 | 29425.6542351677 | 0.217920462775347 | 5579.38804728958 | 23846.2661878781 | South Sudan |
| 1996 | 28285.6576182475 | 0.220399437179258 | 5063.86780806112 | 23221.7898101864 | South Sudan |
| 1997 | 27254.0961791336 | 0.223037171256267 | 5096.02023835042 | 22158.0759407832 | South Sudan |
| 1998 | 26256.3569002288 | 0.225788868984577 | 4444.18010236955 | 21812.1767978593 | South Sudan |
| 1999 | 25090.6820694211 | 0.228648585296784 | 4428.94035653781 | 20661.7417128833 | South Sudan |
| 2000 | 24268.509771043 | 0.231730343895352 | 3798.27131638891 | 20470.2384546541 | South Sudan |
| 2001 | 23376.170482798 | 0.235014127905546 | 3938.68220590844 | 19437.4882768896 | South Sudan |
| 2002 | 22346.6020566047 | 0.238341185794991 | 3653.41415203059 | 18693.1879045741 | South Sudan |
| 2003 | 21461.1164018651 | 0.241797191221707 | 3507.25869072165 | 17953.8577111434 | South Sudan |
| 2004 | 21119.1878171477 | 0.245462498724322 | 3325.75611463167 | 17793.431702516 | South Sudan |
| 2005 | 21715.3310959241 | 0.249280903501851 | 3429.95339078663 | 18285.3777051375 | South Sudan |
| 2006 | 24171.716265446 | 0.253300507898377 | 2981.1934742122 | 21190.5227912338 | South Sudan |
| 2007 | 22872.8525257528 | 0.257491763500287 | 2720.37507023043 | 20152.4774555224 | South Sudan |
| 2008 | 21963.7728736489 | 0.261740458461839 | 2709.57193013321 | 19254.2009435157 | South Sudan |
| 2009 | 20926.4726576336 | 0.266179234510504 | 2475.61025109658 | 18450.862406537 | South Sudan |
| 2010 | 19554.9916390913 | 0.270572622006731 | 2166.78858077132 | 17388.20306 | South Sudan |
| 2011 | 18403.0572873379 | 0.274719142558607 | 2147.41903358412 | 16255.6382537538 | South Sudan |
| 2012 | 18361.939301056 | 0.275066142602006 | 2144.99335096091 | 16216.9459500951 | South Sudan |
| 2013 | 18500.4876440895 | 0.275813942458139 | 2189.57729206369 | 16310.9103520258 | South Sudan |
| 2014 | 18650.1634187702 | 0.276311461476096 | 2182.87564967282 | 16467.2877690974 | South Sudan |
| 2015 | 18578.1651887725 | 0.275833634320407 | 2159.80751113122 | 16418.3576776413 | South Sudan |
| 2016 | 18911.6439827444 | 0.274455491179089 | 2163.22984315412 | 16748.4141395903 | South Sudan |
| 2017 | 19399.9511877469 | 0.274387859268159 | 2174.59522400932 | 17225.3559637376 | South Sudan |
| 2018 | 19999.5548459149 | 0.27498784845828 | 2163.61563379157 | 17835.9392121233 | South Sudan |
| 2019 | 18640.1186324454 | 0.275706200830302 | 2099.80087875174 | 16540.3177536937 | South Sudan |
| 2020 | 17169.9710971347 | 0.276787923789994 | 2148.01957678448 | 15021.9515203502 | South Sudan |
| 2021 | 15935.2846967222 | 0.278371125484101 | 2182.69043648011 | 13752.5942602421 | South Sudan |
| 1990 | 20312.1080041346 | 0.292178643209656 | 2140.51472983691 | 18171.5932742977 | Sudan |
| 1991 | 19324.2119002103 | 0.296473445829652 | 2126.06316166683 | 17198.1487385435 | Sudan |
| 1992 | 18427.4766495401 | 0.30086367654421 | 2111.25566022121 | 16316.2209893189 | Sudan |
| 1993 | 17576.3745219944 | 0.305409325988089 | 2153.68596253156 | 15422.6885594628 | Sudan |
| 1994 | 16798.945029636 | 0.310096430272723 | 2128.76679762797 | 14670.178232008 | Sudan |
| 1995 | 15940.2895471581 | 0.315208259227984 | 2094.89306965505 | 13845.396477503 | Sudan |
| 1996 | 15719.2622653866 | 0.320680229352556 | 2117.00196889226 | 13602.2602964943 | Sudan |
| 1997 | 14199.3398097452 | 0.326761462290204 | 2057.22435392859 | 12142.1154558166 | Sudan |
| 1998 | 14534.7284660671 | 0.333087760646334 | 1990.94284609441 | 12543.7856199727 | Sudan |
| 1999 | 12558.3750259972 | 0.339754883653516 | 1863.55575146328 | 10694.8192745339 | Sudan |
| 2000 | 11461.2778891541 | 0.346875808615915 | 1768.77656990868 | 9692.50131924542 | Sudan |
| 2001 | 10581.3658093224 | 0.354215514631628 | 1759.45465722763 | 8821.91115209477 | Sudan |
| 2002 | 9808.42426409984 | 0.36172823705999 | 1409.84127929466 | 8398.58298480518 | Sudan |
| 2003 | 9052.18085344024 | 0.369693652760892 | 1244.66048765281 | 7807.52036578743 | Sudan |
| 2004 | 8452.2371778969 | 0.377920656442902 | 1126.28251200249 | 7325.95466589441 | Sudan |
| 2005 | 8043.10397998469 | 0.386685712077585 | 1088.20592414325 | 6954.89805584144 | Sudan |
| 2006 | 7922.71604720259 | 0.39615137434576 | 976.76770745815 | 6945.94833974444 | Sudan |
| 2007 | 6772.80147850334 | 0.406079992219235 | 887.574958292634 | 5885.2265202107 | Sudan |
| 2008 | 6169.58922932563 | 0.416087599939819 | 787.9709919 | 5381.61823743573 | Sudan |
| 2009 | 5563.31567654368 | 0.425975917452396 | 691.895573245837 | 4871.42010329784 | Sudan |
| 2010 | 4917.92460682474 | 0.436171097454113 | 423.851373966412 | 4494.07323285833 | Sudan |
| 2011 | 3911.16182827675 | 0.447239441530175 | 411.24125173471 | 3499.92057654204 | Sudan |
| 2012 | 3255.45697221763 | 0.456790264757693 | 397.28726201809 | 2858.16971019954 | Sudan |
| 2013 | 2699.47382972069 | 0.466435995212048 | 359.974841274638 | 2339.49898844605 | Sudan |
| 2014 | 2279.50682814845 | 0.476362134920594 | 337.849728614243 | 1941.65709953421 | Sudan |
| 2015 | 1930.49564702277 | 0.486549437288546 | 319.265801842457 | 1611.22984518031 | Sudan |
| 2016 | 1699.24634365883 | 0.496848218716792 | 250.62547900231 | 1448.62086465652 | Sudan |
| 2017 | 1814.06083636415 | 0.50688353389451 | 219.226026258814 | 1594.83481010534 | Sudan |
| 2018 | 1324.81773715039 | 0.516214086476663 | 203.460628876163 | 1121.35710827423 | Sudan |
| 2019 | 1198.35724613712 | 0.525009122461798 | 190.702675125464 | 1007.65457101166 | Sudan |
| 2020 | 1119.61941700239 | 0.533455421993626 | 172.022816300275 | 947.596600702115 | Sudan |
| 2021 | 1043.77440584689 | 0.54194973514933 | 155.012652962068 | 888.761752884822 | Sudan |

Table S12 Frontier Analysis Based on SDI and DALYs for Shigella Associated Diarrheal in Neonates and Children Under Five Years of Age in 204 Countries and Territories

| **Year** | **DALYs rate** | **SDI** | **Frontier** | **Effective difference** | **Location** |
| --- | --- | --- | --- | --- | --- |
| 1990 | 606.64261895953 | 0.458668935267616 | 65.6974224275544 | 540.945196531976 | China |
| 1991 | 564.56091663785 | 0.467301969740226 | 57.9835116047874 | 506.577405033063 | China |
| 1992 | 504.69851336985 | 0.475784030669638 | 57.5883989754462 | 447.110114394404 | China |
| 1993 | 455.01135459104 | 0.484353830709851 | 53.9537713113633 | 401.057583279677 | China |
| 1994 | 415.01891814195 | 0.49257864459854 | 50.3218991980225 | 364.697018943928 | China |
| 1995 | 376.63152803771 | 0.501500907580649 | 23.3882281295887 | 353.243299908121 | China |
| 1996 | 338.89798234041 | 0.512033995790715 | 22.4207972762095 | 316.4771850642 | China |
| 1997 | 302.71569308751 | 0.522326866102753 | 22.107822026074 | 280.607871061436 | China |
| 1998 | 270.45697898431 | 0.531379484348035 | 22.1741598112749 | 248.282819173035 | China |
| 1999 | 242.28106995417 | 0.540333688117168 | 21.1863350622744 | 221.094734891896 | China |
| 2000 | 217.63357234986 | 0.547989192872889 | 20.5885451767414 | 197.045027173119 | China |
| 2001 | 188.43354855102 | 0.553680373692626 | 18.6572439908892 | 169.776304560131 | China |
| 2002 | 161.83381356238 | 0.560542936414416 | 17.7756704558864 | 144.058143106494 | China |
| 2003 | 143.68709683198 | 0.569018827276063 | 14.0318893627383 | 129.655207469242 | China |
| 2004 | 126.55458602131 | 0.578178090807068 | 13.4225309540955 | 113.132055067215 | China |
| 2005 | 107.87201184095 | 0.58805219357166 | 13.4114409796137 | 94.4605708613363 | China |
| 2006 | 89.50551599539 | 0.598758170327953 | 13.3838513235016 | 76.1216646718884 | China |
| 2007 | 71.7851230435 | 0.609520246163436 | 13.1451913475941 | 58.6399316959058 | China |
| 2008 | 57.03367932034 | 0.619454832781724 | 13.0601265829288 | 43.9735527374112 | China |
| 2009 | 45.468572349 | 0.62930772749348 | 12.0848413978202 | 33.3837309511798 | China |
| 2010 | 36.35499487343 | 0.64152144342547 | 8.25920722232254 | 28.0957876511075 | China |
| 2011 | 30.12810990589 | 0.651447977618785 | 5.76044650364213 | 24.3676634022479 | China |
| 2012 | 26.37852948682 | 0.657144292359298 | 4.84842213658877 | 21.5301073502312 | China |
| 2013 | 23.683278196 | 0.66310300729639 | 4.73634689467385 | 18.9469313013262 | China |
| 2014 | 21.3480560076 | 0.668640847002655 | 3.70390488107541 | 17.6441511265246 | China |
| 2015 | 19.7442318512 | 0.671940110944872 | 3.33653551173298 | 16.407696339467 | China |
| 2016 | 18.08666179658 | 0.676218437253933 | 2.94713206441031 | 15.1395297321697 | China |
| 2017 | 15.1599364341 | 0.6846196607825 | 2.7813411524975 | 12.3785952816025 | China |
| 2018 | 12.71086127947 | 0.693717996192013 | 2.50373150716566 | 10.2071297723043 | China |
| 2019 | 10.76782120197 | 0.703686823937232 | 2.29158057970423 | 8.47624062226577 | China |
| 2020 | 11.87488945594 | 0.713364584593745 | 2.22635943000023 | 9.64853002593977 | China |
| 2021 | 11.1260453134 | 0.721629759719777 | 2.17923885987 | 8.94680645353 | China |
| 1990 | 32.76872330773 | 0.497780128289885 | 24.7226897679072 | 8.04603353982278 | Democratic People's Republic of Korea |
| 1991 | 31.61925294677 | 0.49912051226047 | 24.0456854738801 | 7.57356747288985 | Democratic People's Republic of Korea |
| 1992 | 30.50709084413 | 0.499629841478392 | 24.0466171668591 | 6.46047367727089 | Democratic People's Republic of Korea |
| 1993 | 29.22015683997 | 0.49970064634474 | 23.3477606779187 | 5.8723961620513 | Democratic People's Republic of Korea |
| 1994 | 28.08840945759 | 0.499531339948334 | 24.2317583737251 | 3.85665108386492 | Democratic People's Republic of Korea |
| 1995 | 27.01955467633 | 0.499080529317303 | 24.1876545373698 | 2.83190013896017 | Democratic People's Republic of Korea |
| 1996 | 26.08256879819 | 0.498429013609349 | 24.1530572663703 | 1.92951153181968 | Democratic People's Republic of Korea |
| 1997 | 25.36607821047 | 0.497136868597342 | 24.5535559007802 | 0.812522309689843 | Democratic People's Republic of Korea |
| 1998 | 24.71565390984 | 0.49615920966594 | 24.71565390984 | 0 | Democratic People's Republic of Korea |
| 1999 | 23.99906558605 | 0.496484753521051 | 23.99906558605 | 0 | Democratic People's Republic of Korea |
| 2000 | 23.32386356395 | 0.497461994823193 | 23.32386356395 | 0 | Democratic People's Republic of Korea |
| 2001 | 22.77158412909 | 0.499674042505593 | 22.77158412909 | 0 | Democratic People's Republic of Korea |
| 2002 | 22.52621015357 | 0.502633568731371 | 22.52621015357 | 0 | Democratic People's Republic of Korea |
| 2003 | 22.4906921947 | 0.506165243055573 | 22.4906921947 | 0 | Democratic People's Republic of Korea |
| 2004 | 22.29850514813 | 0.510229134162392 | 22.29850514813 | 0 | Democratic People's Republic of Korea |
| 2005 | 21.98330629942 | 0.515097762842417 | 21.98330629942 | 0 | Democratic People's Republic of Korea |
| 2006 | 22.08541600423 | 0.519741439913669 | 22.0264512451144 | 0.0589647591156357 | Democratic People's Republic of Korea |
| 2007 | 22.49216896689 | 0.524066965087203 | 22.1008453462733 | 0.391323620616713 | Democratic People's Republic of Korea |
| 2008 | 23.21464788586 | 0.52881349761598 | 22.0831241712918 | 1.13152371456821 | Democratic People's Republic of Korea |
| 2009 | 23.50308442969 | 0.533114411172631 | 22.1305962762264 | 1.37248815346364 | Democratic People's Republic of Korea |
| 2010 | 23.45733266585 | 0.53719291837066 | 21.389167444874 | 2.06816522097605 | Democratic People's Republic of Korea |
| 2011 | 22.53733733708 | 0.541212269847829 | 21.1859042354266 | 1.35143310165338 | Democratic People's Republic of Korea |
| 2012 | 20.91988572202 | 0.545331713402902 | 20.91988572202 | 0 | Democratic People's Republic of Korea |
| 2013 | 18.98440246849 | 0.549483297235091 | 18.98440246849 | 0 | Democratic People's Republic of Korea |
| 2014 | 17.256925224 | 0.553603559944788 | 17.256925224 | 0 | Democratic People's Republic of Korea |
| 2015 | 16.12858312074 | 0.557321271453133 | 16.12858312074 | 0 | Democratic People's Republic of Korea |
| 2016 | 15.46719711078 | 0.560743562553338 | 15.46719711078 | 0 | Democratic People's Republic of Korea |
| 2017 | 14.8871933917 | 0.563388288880609 | 14.8871933917 | 0 | Democratic People's Republic of Korea |
| 2018 | 14.27946916548 | 0.565161484391211 | 14.27946916548 | 0 | Democratic People's Republic of Korea |
| 2019 | 13.77729674505 | 0.566765705893725 | 13.77729674505 | 0 | Democratic People's Republic of Korea |
| 2020 | 13.41815177639 | 0.56818855103523 | 13.41815177639 | 0 | Democratic People's Republic of Korea |
| 2021 | 11.89156186223 | 0.569854634067777 | 11.89156186223 | 0 | Democratic People's Republic of Korea |
| 1990 | 17.4840453992 | 0.667633853818721 | 3.59073978762519 | 13.8933056115748 | Taiwan (Province of China) |
| 1991 | 17.60627085222 | 0.676537236907717 | 3.1770734127582 | 14.4291974394618 | Taiwan (Province of China) |
| 1992 | 14.86631388884 | 0.684803635601178 | 2.71243819864796 | 12.153875690192 | Taiwan (Province of China) |
| 1993 | 12.62598290815 | 0.692924130193594 | 2.42625064422778 | 10.1997322639222 | Taiwan (Province of China) |
| 1994 | 12.23022596116 | 0.701078749506874 | 2.40851018970163 | 9.82171577145837 | Taiwan (Province of China) |
| 1995 | 11.52553717986 | 0.709626820870811 | 2.28507522049581 | 9.24046195936419 | Taiwan (Province of China) |
| 1996 | 10.55121583345 | 0.719128415346056 | 2.19968456976558 | 8.35153126368442 | Taiwan (Province of China) |
| 1997 | 9.30968461084 | 0.72914549717473 | 2.17237859560725 | 7.13730601523275 | Taiwan (Province of China) |
| 1998 | 8.23736908904 | 0.738125787434293 | 2.16288743660824 | 6.07448165243176 | Taiwan (Province of China) |
| 1999 | 7.37847254789 | 0.745338399386342 | 2.07140506771694 | 5.30706748017306 | Taiwan (Province of China) |
| 2000 | 7.22054064465 | 0.752926841487622 | 2.03031040330973 | 5.19023024134027 | Taiwan (Province of China) |
| 2001 | 7.06067341911 | 0.760829809097496 | 1.83014818193448 | 5.23052523717552 | Taiwan (Province of China) |
| 2002 | 6.62911010713 | 0.769008983647841 | 1.68115847925413 | 4.94795162787587 | Taiwan (Province of China) |
| 2003 | 6.12507590953 | 0.776955803379445 | 1.51255712100319 | 4.61251878852681 | Taiwan (Province of China) |
| 2004 | 8.22309246783 | 0.784677977861477 | 1.47759031836143 | 6.74550214946857 | Taiwan (Province of China) |
| 2005 | 8.69590820959 | 0.792445129331779 | 1.44590201784125 | 7.25000619174875 | Taiwan (Province of China) |
| 2006 | 8.22963660427 | 0.800174674875457 | 1.48097124892556 | 6.74866535534444 | Taiwan (Province of China) |
| 2007 | 8.23549034065 | 0.808137693890723 | 1.47047513063673 | 6.76501521001326 | Taiwan (Province of China) |
| 2008 | 8.7008981938 | 0.81540069133486 | 1.41159877134065 | 7.28929942245935 | Taiwan (Province of China) |
| 2009 | 8.8375045752 | 0.821432279265941 | 1.41444923496405 | 7.42305534023595 | Taiwan (Province of China) |
| 2010 | 8.97902999331 | 0.827226752015366 | 1.4228774096251 | 7.5561525836849 | Taiwan (Province of China) |
| 2011 | 9.08009391691 | 0.832064073807912 | 1.43044297108711 | 7.64965094582289 | Taiwan (Province of China) |
| 2012 | 9.47802749515 | 0.836677664574998 | 1.42907620874836 | 8.04895128640164 | Taiwan (Province of China) |
| 2013 | 9.01819630001 | 0.841280462756768 | 1.40033557898053 | 7.61786072102947 | Taiwan (Province of China) |
| 2014 | 9.00500191949 | 0.845695560406305 | 1.36265150278677 | 7.64235041670323 | Taiwan (Province of China) |
| 2015 | 9.08197133816 | 0.850055268699097 | 1.35659259628767 | 7.72537874187233 | Taiwan (Province of China) |
| 2016 | 8.73487585864 | 0.854466037759635 | 1.29286028696351 | 7.44201557167649 | Taiwan (Province of China) |
| 2017 | 8.58920522811 | 0.858888018761485 | 1.31052440209 | 7.27868082602 | Taiwan (Province of China) |
| 2018 | 8.55906301921 | 0.863158360983117 | 1.26736520306524 | 7.29169781614476 | Taiwan (Province of China) |
| 2019 | 8.47850604646 | 0.86741184993072 | 1.27537043062326 | 7.20313561583674 | Taiwan (Province of China) |
| 2020 | 8.54686421778 | 0.871247947250766 | 1.26674724272094 | 7.28011697505906 | Taiwan (Province of China) |
| 2021 | 8.45803322089 | 0.874747053187929 | 1.27060350568333 | 7.18742971520667 | Taiwan (Province of China) |
| 1990 | 3108.14460072885 | 0.289075059080125 | 1128.48258672144 | 1979.66201400741 | Cambodia |
| 1991 | 3025.53819380193 | 0.29247920029669 | 1074.22695460051 | 1951.31123920142 | Cambodia |
| 1992 | 2873.0886551118 | 0.296900954885372 | 1086.4794579354 | 1786.6091971764 | Cambodia |
| 1993 | 2762.85300109003 | 0.301627201679918 | 1025.95518943659 | 1736.89781165344 | Cambodia |
| 1994 | 2701.40853685445 | 0.305722592004088 | 1029.48349626527 | 1671.92504058918 | Cambodia |
| 1995 | 2544.88995955871 | 0.310678881281017 | 960.991221712223 | 1583.89873784649 | Cambodia |
| 1996 | 2367.82665723095 | 0.31563096971546 | 831.862027737043 | 1535.96462949391 | Cambodia |
| 1997 | 2223.11486983909 | 0.320503749678941 | 836.665889990004 | 1386.44897984909 | Cambodia |
| 1998 | 2098.9559503477 | 0.324974298583536 | 751.990047740004 | 1346.9659026077 | Cambodia |
| 1999 | 1957.31764478828 | 0.330224246107348 | 678.355558237797 | 1278.96208655048 | Cambodia |
| 2000 | 1758.38506336134 | 0.336075029953322 | 677.571733267398 | 1080.81333009394 | Cambodia |
| 2001 | 1591.31904793768 | 0.342507910583009 | 596.139090596138 | 995.179957341541 | Cambodia |
| 2002 | 1414.81006884205 | 0.349465712270985 | 591.699268483128 | 823.110800358922 | Cambodia |
| 2003 | 1243.47538171486 | 0.356929660458733 | 585.729718812981 | 657.745662901879 | Cambodia |
| 2004 | 1102.94241892358 | 0.364854644690823 | 574.586959428752 | 528.355459494828 | Cambodia |
| 2005 | 965.55536689137 | 0.373331814716366 | 541.819202456305 | 423.736164435065 | Cambodia |
| 2006 | 835.71314671755 | 0.381698166189418 | 512.642548774998 | 323.070597942552 | Cambodia |
| 2007 | 732.91416555519 | 0.389876229632594 | 511.400396678638 | 221.513768876552 | Cambodia |
| 2008 | 639.80998686005 | 0.397679519850279 | 465.340565864947 | 174.469420995103 | Cambodia |
| 2009 | 556.15306874289 | 0.404220933365413 | 395.611848971462 | 160.541219771428 | Cambodia |
| 2010 | 490.78342977104 | 0.410211121336958 | 313.041243732168 | 177.742186038872 | Cambodia |
| 2011 | 436.03497255387 | 0.415964408667437 | 273.646889668914 | 162.388082884956 | Cambodia |
| 2012 | 389.76444975889 | 0.4217697537185 | 241.699063313743 | 148.065386445147 | Cambodia |
| 2013 | 341.57359510009 | 0.427715060183387 | 213.520739371887 | 128.052855728203 | Cambodia |
| 2014 | 308.40599961915 | 0.433597748994237 | 204.493148729793 | 103.912850889357 | Cambodia |
| 2015 | 281.45531972771 | 0.43948735809131 | 129.145415583158 | 152.309904144552 | Cambodia |
| 2016 | 256.94393604105 | 0.445358172105636 | 106.724459745348 | 150.219476295702 | Cambodia |
| 2017 | 234.47179793621 | 0.451221744385535 | 81.1688329325247 | 153.302965003685 | Cambodia |
| 2018 | 216.52009631146 | 0.457341489845745 | 67.7178716356777 | 148.802224675782 | Cambodia |
| 2019 | 199.19427588039 | 0.463643430446969 | 63.349542328687 | 135.844733551703 | Cambodia |
| 2020 | 185.3613541405 | 0.468876053670987 | 57.0007253827019 | 128.360628757798 | Cambodia |
| 2021 | 173.95155623435 | 0.473621490708013 | 57.3746032110925 | 116.576953023257 | Cambodia |
| 1990 | 4767.33880213154 | 0.457134954421362 | 64.0384020307409 | 4703.3004001008 | Indonesia |
| 1991 | 4304.95947958974 | 0.466505583269134 | 60.5208396298834 | 4244.43863995986 | Indonesia |
| 1992 | 3865.58602256309 | 0.475753157454434 | 57.2558138835134 | 3808.33020867958 | Indonesia |
| 1993 | 3497.1695811392 | 0.484775240859362 | 53.2824506481473 | 3443.88713049105 | Indonesia |
| 1994 | 3160.76526363904 | 0.493607099523711 | 49.8643484917195 | 3110.90091514732 | Indonesia |
| 1995 | 2841.92908501136 | 0.502008381392835 | 23.3282769154221 | 2818.60080809594 | Indonesia |
| 1996 | 2525.60020560209 | 0.510108146446556 | 22.75955780582 | 2502.84064779627 | Indonesia |
| 1997 | 2253.86509270411 | 0.51810218881129 | 22.2684975521062 | 2231.596595152 | Indonesia |
| 1998 | 2059.96668582111 | 0.524045603868488 | 22.0712339744175 | 2037.89545184669 | Indonesia |
| 1999 | 1877.69446278873 | 0.529176329629629 | 22.1620017160998 | 1855.53246107263 | Indonesia |
| 2000 | 1705.87422524843 | 0.533789587270659 | 22.158009327487 | 1683.71621592094 | Indonesia |
| 2001 | 1604.4161241377 | 0.537977981115366 | 21.3608657153566 | 1583.05525842234 | Indonesia |
| 2002 | 1530.79193298613 | 0.542245676060877 | 21.2272459989934 | 1509.56468698714 | Indonesia |
| 2003 | 1465.61902359116 | 0.546550264351478 | 20.5444302472494 | 1445.07459334391 | Indonesia |
| 2004 | 1401.11302084025 | 0.550902314227472 | 19.9148706584291 | 1381.19815018182 | Indonesia |
| 2005 | 1331.20740557307 | 0.555424913216581 | 18.743828326812 | 1312.46357724626 | Indonesia |
| 2006 | 1245.71706199221 | 0.560265924242442 | 17.4589673274242 | 1228.25809466479 | Indonesia |
| 2007 | 1175.0698004504 | 0.565811896183758 | 15.3930723012131 | 1159.67672814919 | Indonesia |
| 2008 | 1104.94017854067 | 0.572225266933015 | 13.4361371050709 | 1091.5040414356 | Indonesia |
| 2009 | 1019.1980189529 | 0.578866902230273 | 13.6658361534685 | 1005.53218279943 | Indonesia |
| 2010 | 929.32236195899 | 0.585982419406785 | 13.4092576239821 | 915.913104335008 | Indonesia |
| 2011 | 835.52444816077 | 0.593652984587084 | 13.4638814021518 | 822.060566758618 | Indonesia |
| 2012 | 746.51992209756 | 0.601185701344525 | 13.2644468689266 | 733.255475228633 | Indonesia |
| 2013 | 672.9452326736 | 0.608509904334102 | 13.3061518248551 | 659.639080848745 | Indonesia |
| 2014 | 611.34354225498 | 0.615499887091834 | 12.8845776884612 | 598.458964566519 | Indonesia |
| 2015 | 567.78142804066 | 0.622222469472476 | 12.9771958815188 | 554.804232159141 | Indonesia |
| 2016 | 520.70380005473 | 0.62866662466714 | 12.5610122198439 | 508.142787834886 | Indonesia |
| 2017 | 489.87370033052 | 0.634895744678987 | 11.037363930366 | 478.836336400154 | Indonesia |
| 2018 | 467.14223333851 | 0.640948750163115 | 7.56465741614453 | 459.577575922365 | Indonesia |
| 2019 | 441.19231004976 | 0.64686545689526 | 5.407087170283 | 435.785222879477 | Indonesia |
| 2020 | 409.75975759103 | 0.651926647922518 | 5.19902900488071 | 404.560728586149 | Indonesia |
| 2021 | 393.17232788039 | 0.656868336261681 | 4.90371777070655 | 388.268610109683 | Indonesia |
| 1990 | 7184.84130016955 | 0.264283163752662 | 1389.77291151147 | 5795.06838865808 | Lao People's Democratic Republic |
| 1991 | 6751.94903104701 | 0.268787617854408 | 1232.4014423503 | 5519.54758869671 | Lao People's Democratic Republic |
| 1992 | 6333.99156056672 | 0.273435312470827 | 1246.5495625005 | 5087.44199806622 | Lao People's Democratic Republic |
| 1993 | 6239.49709833038 | 0.278140417914198 | 1193.52905439592 | 5045.96804393446 | Lao People's Democratic Republic |
| 1994 | 6134.93335690826 | 0.283270229855729 | 1203.67170310159 | 4931.26165380667 | Lao People's Democratic Republic |
| 1995 | 5313.50551435943 | 0.288795341159004 | 1134.44189818282 | 4179.06361617661 | Lao People's Democratic Republic |
| 1996 | 5031.18536050258 | 0.294838372041673 | 1080.59067420968 | 3950.5946862929 | Lao People's Democratic Republic |
| 1997 | 4708.36016765728 | 0.301477967015192 | 1026.70711376817 | 3681.65305388911 | Lao People's Democratic Republic |
| 1998 | 4415.06857674059 | 0.308402544731754 | 961.546428767128 | 3453.52214797346 | Lao People's Democratic Republic |
| 1999 | 4116.39739441835 | 0.315972615438411 | 840.648931796023 | 3275.74846262233 | Lao People's Democratic Republic |
| 2000 | 3844.87042182362 | 0.323947359495962 | 758.910520029738 | 3085.95990179388 | Lao People's Democratic Republic |
| 2001 | 3456.511153396 | 0.332580408807204 | 686.705434776668 | 2769.80571861933 | Lao People's Democratic Republic |
| 2002 | 3194.90862102876 | 0.341667271144462 | 595.207985889663 | 2599.7006351391 | Lao People's Democratic Republic |
| 2003 | 2887.36882034739 | 0.350658178383381 | 594.901596402855 | 2292.46722394454 | Lao People's Democratic Republic |
| 2004 | 2620.53568809738 | 0.359681759216616 | 592.008859735186 | 2028.52682836219 | Lao People's Democratic Republic |
| 2005 | 2371.92120064493 | 0.368728677775792 | 577.167434080411 | 1794.75376656452 | Lao People's Democratic Republic |
| 2006 | 2133.8806489801 | 0.378122280697802 | 546.289422968699 | 1587.5912260114 | Lao People's Democratic Republic |
| 2007 | 1894.66568976015 | 0.387723649113709 | 506.236836249026 | 1388.42885351112 | Lao People's Democratic Republic |
| 2008 | 1799.57422604194 | 0.397011298824877 | 468.671777656657 | 1330.90244838528 | Lao People's Democratic Republic |
| 2009 | 1610.41427198449 | 0.406139021683818 | 360.860741684492 | 1249.5535303 | Lao People's Democratic Republic |
| 2010 | 1439.62861813233 | 0.414865566497825 | 272.591299007201 | 1167.03731912513 | Lao People's Democratic Republic |
| 2011 | 1266.77825727408 | 0.42335452259834 | 232.973466731617 | 1033.80479054246 | Lao People's Democratic Republic |
| 2012 | 1155.11110378721 | 0.431149360954477 | 206.051041902514 | 949.060061884696 | Lao People's Democratic Republic |
| 2013 | 1031.86227248821 | 0.438691038635463 | 117.584585814042 | 914.277686674168 | Lao People's Democratic Republic |
| 2014 | 911.93456756914 | 0.4458694242056 | 112.764158230227 | 799.170409338913 | Lao People's Democratic Republic |
| 2015 | 821.78420866456 | 0.452749603276049 | 73.1867523616813 | 748.597456302879 | Lao People's Democratic Republic |
| 2016 | 733.70383725708 | 0.459583995539449 | 64.4575891826904 | 669.24624807439 | Lao People's Democratic Republic |
| 2017 | 647.55966425673 | 0.466168891657656 | 56.9157628121582 | 590.643901444572 | Lao People's Democratic Republic |
| 2018 | 592.39292261434 | 0.472618102538068 | 58.2871246241118 | 534.105797990228 | Lao People's Democratic Republic |
| 2019 | 541.47811576218 | 0.478811372841658 | 52.7260370289815 | 488.752078733198 | Lao People's Democratic Republic |
| 2020 | 458.22231588114 | 0.484192959345392 | 54.3475651289466 | 403.874750752193 | Lao People's Democratic Republic |
| 2021 | 428.83595973768 | 0.489136091456599 | 50.0222244927713 | 378.813735244909 | Lao People's Democratic Republic |
| 1990 | 130.95909021745 | 0.545799405460294 | 20.6427186689906 | 110.316371548459 | Malaysia |
| 1991 | 111.90483144167 | 0.551683548009526 | 19.8228455217539 | 92.0819859199161 | Malaysia |
| 1992 | 108.89795261563 | 0.558186752245966 | 17.6996981227975 | 91.1982544928325 | Malaysia |
| 1993 | 100.83074402586 | 0.565845656303133 | 15.3323437481101 | 85.4984002777499 | Malaysia |
| 1994 | 86.96987606346 | 0.574380445989544 | 13.6214789341603 | 73.3483971292997 | Malaysia |
| 1995 | 85.46636534502 | 0.583441102596569 | 13.3349975828621 | 72.1313677621579 | Malaysia |
| 1996 | 68.57235043968 | 0.593152135005711 | 13.4416833626187 | 55.1306670770613 | Malaysia |
| 1997 | 65.88866361384 | 0.603418105152686 | 13.6956285858988 | 52.1930350279412 | Malaysia |
| 1998 | 60.90892535696 | 0.612645412003281 | 13.193172017196 | 47.715753339764 | Malaysia |
| 1999 | 56.66784565389 | 0.621724901078498 | 12.9350921027363 | 43.7327535511536 | Malaysia |
| 2000 | 46.13994934114 | 0.630523543762275 | 12.0112104782569 | 34.1287388628831 | Malaysia |
| 2001 | 43.60000716804 | 0.638257476929833 | 8.33712879849368 | 35.2628783695463 | Malaysia |
| 2002 | 41.37988560202 | 0.645044248017871 | 5.65586987980388 | 35.7240157222161 | Malaysia |
| 2003 | 38.93677310608 | 0.65088567830407 | 5.16673637646843 | 33.7700367296116 | Malaysia |
| 2004 | 39.16140881544 | 0.656243325827196 | 4.78123470807646 | 34.3801741073635 | Malaysia |
| 2005 | 41.235876934 | 0.661242241061834 | 4.49551698441313 | 36.7403599495869 | Malaysia |
| 2006 | 39.8851632976 | 0.665956287100151 | 4.31124616527377 | 35.5739171323262 | Malaysia |
| 2007 | 39.9316454914 | 0.670843725887123 | 3.67972121464889 | 36.2519242767511 | Malaysia |
| 2008 | 40.36465459409 | 0.676399910213952 | 3.1811092057631 | 37.1835453883269 | Malaysia |
| 2009 | 40.59097095829 | 0.682070653985994 | 2.85860791867305 | 37.732363039617 | Malaysia |
| 2010 | 36.98840145948 | 0.688258621606068 | 2.54328544376863 | 34.4451160157114 | Malaysia |
| 2011 | 38.04512248419 | 0.69408340345083 | 2.56709772983554 | 35.4780247543545 | Malaysia |
| 2012 | 37.10178437802 | 0.699664734135674 | 2.35502069197827 | 34.7467636860417 | Malaysia |
| 2013 | 35.03218643249 | 0.70493071683803 | 2.29394751152273 | 32.7382389209673 | Malaysia |
| 2014 | 36.70139879473 | 0.709953756222256 | 2.25292149119569 | 34.4484773035343 | Malaysia |
| 2015 | 38.70271376696 | 0.714659871236347 | 2.23000936712977 | 36.4727043998302 | Malaysia |
| 2016 | 37.09199132822 | 0.719223424980679 | 2.19529682404573 | 34.8966945041743 | Malaysia |
| 2017 | 38.17893765084 | 0.724235411990887 | 2.16713368401033 | 36.0118039668297 | Malaysia |
| 2018 | 36.78863084479 | 0.72938807812528 | 2.16545700555716 | 34.6231738392328 | Malaysia |
| 2019 | 36.90067533608 | 0.734587751127645 | 2.17750060743258 | 34.7231747286474 | Malaysia |
| 2020 | 34.29916219063 | 0.738743992650626 | 2.15465455338719 | 32.1445076372428 | Malaysia |
| 2021 | 33.42531860614 | 0.742523828414751 | 2.09575825486397 | 31.329560351276 | Malaysia |
| 1990 | 2572.7160554067 | 0.331601544442323 | 677.342703339909 | 1895.37335206679 | Maldives |
| 1991 | 2260.59492367349 | 0.344398606997735 | 593.446456593855 | 1667.14846707963 | Maldives |
| 1992 | 1953.63095799728 | 0.358252339504646 | 591.809039473305 | 1361.82191852398 | Maldives |
| 1993 | 1687.45479095304 | 0.372806998520911 | 544.24969424789 | 1143.20509670515 | Maldives |
| 1994 | 1416.75581208756 | 0.388081604129663 | 515.557956626491 | 901.197855461069 | Maldives |
| 1995 | 1128.71519223 | 0.403945582505039 | 395.63528479356 | 733.07990743644 | Maldives |
| 1996 | 1033.56223557218 | 0.420519127829701 | 244.079204864892 | 789.483030707288 | Maldives |
| 1997 | 832.32495084866 | 0.437436025558501 | 123.263641262701 | 709.061309585959 | Maldives |
| 1998 | 736.27522974616 | 0.453973361371121 | 73.9985855214605 | 662.2766442247 | Maldives |
| 1999 | 602.11953744708 | 0.469338065717424 | 57.1666738637584 | 544.952863583322 | Maldives |
| 2000 | 538.88734490604 | 0.483495670503175 | 54.284241720192 | 484.603103185848 | Maldives |
| 2001 | 463.29051081972 | 0.495923695035155 | 50.2557622552818 | 413.034748564438 | Maldives |
| 2002 | 388.36586321649 | 0.507404859766873 | 22.7757833051736 | 365.590079911316 | Maldives |
| 2003 | 352.38864719699 | 0.518926864396739 | 22.1901973336548 | 330.198449863335 | Maldives |
| 2004 | 315.47149448693 | 0.529910190937908 | 22.1483087083592 | 293.323185778571 | Maldives |
| 2005 | 273.64391627114 | 0.53825333247429 | 21.4112326032637 | 252.232683667876 | Maldives |
| 2006 | 263.36900605244 | 0.547950411527519 | 20.6767873936871 | 242.692218658753 | Maldives |
| 2007 | 239.86544936939 | 0.557529246632533 | 17.8992976749683 | 221.966151694422 | Maldives |
| 2008 | 219.86610903614 | 0.566839634379964 | 14.5286295932049 | 205.337479442935 | Maldives |
| 2009 | 203.72711947482 | 0.574442059853853 | 13.5773773846909 | 190.149742090129 | Maldives |
| 2010 | 189.96009217452 | 0.581980986393534 | 13.0986264404563 | 176.861465734064 | Maldives |
| 2011 | 176.54423015597 | 0.589684459954112 | 13.48266601185 | 163.06156414412 | Maldives |
| 2012 | 164.44156091401 | 0.596948519185372 | 13.2296652595871 | 151.211895654423 | Maldives |
| 2013 | 153.36558377653 | 0.604238519255647 | 13.3766530989034 | 139.988930677627 | Maldives |
| 2014 | 142.99798008762 | 0.611578071403856 | 13.2186073900315 | 129.779372697588 | Maldives |
| 2015 | 134.09359344908 | 0.618454389892933 | 13.0379912235896 | 121.05560222549 | Maldives |
| 2016 | 126.36973578008 | 0.624875949954596 | 12.9431907931388 | 113.426544986941 | Maldives |
| 2017 | 119.06308774648 | 0.631083288156097 | 12.1461117156327 | 106.916976030847 | Maldives |
| 2018 | 110.86685099548 | 0.637205659403498 | 7.18034565583418 | 103.686505339646 | Maldives |
| 2019 | 105.54173134479 | 0.643082793641918 | 7.76886661338469 | 97.7728647314053 | Maldives |
| 2020 | 98.26961923302 | 0.647608589281031 | 5.95422115321617 | 92.3153980798038 | Maldives |
| 2021 | 97.98005572163 | 0.650886627343107 | 5.4430881740242 | 92.5369675476058 | Maldives |
| 1990 | 6243.24268604174 | 0.319219723709677 | 848.731136842915 | 5394.51154919882 | Myanmar |
| 1991 | 6050.55010226047 | 0.322034718697423 | 818.12169596859 | 5232.42840629188 | Myanmar |
| 1992 | 5754.78597090879 | 0.325628883748026 | 766.842493451076 | 4987.94347745771 | Myanmar |
| 1993 | 5420.50971035268 | 0.329707434417887 | 747.768564694942 | 4672.74114565774 | Myanmar |
| 1994 | 5033.64389450255 | 0.334290120297598 | 676.302496478069 | 4357.34139802448 | Myanmar |
| 1995 | 4637.26797257379 | 0.339590975967104 | 593.99459227598 | 4043.27338029781 | Myanmar |
| 1996 | 4276.52733332189 | 0.345191641257382 | 595.220377532811 | 3681.30695578908 | Myanmar |
| 1997 | 3922.03673808173 | 0.350892883218861 | 595.619234144222 | 3326.41750393751 | Myanmar |
| 1998 | 3579.69392481082 | 0.356696902058465 | 596.33805699391 | 2983.35586781691 | Myanmar |
| 1999 | 3230.98425490597 | 0.36303773228042 | 568.350768714522 | 2662.63348619145 | Myanmar |
| 2000 | 2899.03457361655 | 0.370360017053284 | 582.208032968554 | 2316.826540648 | Myanmar |
| 2001 | 2511.29490212738 | 0.378171980483692 | 545.975492674903 | 1965.31940945248 | Myanmar |
| 2002 | 2180.38719714571 | 0.386343628220189 | 517.918787784343 | 1662.46840936137 | Myanmar |
| 2003 | 1902.7890441377 | 0.395218766380276 | 463.70247237298 | 1439.08657176472 | Myanmar |
| 2004 | 1784.52549717927 | 0.40469235815513 | 392.638953088723 | 1391.88654409055 | Myanmar |
| 2005 | 1643.36390279897 | 0.414662082498037 | 271.210533292314 | 1372.15336950666 | Myanmar |
| 2006 | 1480.51235293275 | 0.424705070565889 | 235.59236276894 | 1244.91999016381 | Myanmar |
| 2007 | 1323.62014137011 | 0.434536927066905 | 203.180150130883 | 1120.43999123923 | Myanmar |
| 2008 | 1214.58446714687 | 0.443595531234043 | 112.963416384982 | 1101.62105076189 | Myanmar |
| 2009 | 1102.90614993041 | 0.451954883959276 | 83.4194138573176 | 1019.48673607309 | Myanmar |
| 2010 | 1018.2785154421 | 0.460180429354898 | 66.5769670923343 | 951.701548349766 | Myanmar |
| 2011 | 938.34989779308 | 0.467850871609568 | 55.3931101137783 | 882.956787679302 | Myanmar |
| 2012 | 855.28492304421 | 0.475336422547245 | 58.4144366494032 | 796.870486394807 | Myanmar |
| 2013 | 788.85068041597 | 0.482821855058075 | 53.8076409343729 | 735.043039481597 | Myanmar |
| 2014 | 722.09981346771 | 0.490255145456161 | 49.388554868406 | 672.711258599304 | Myanmar |
| 2015 | 666.9256633369 | 0.497374156127321 | 26.6534895345169 | 640.272173802383 | Myanmar |
| 2016 | 631.11253867259 | 0.504115978117272 | 22.8617147472903 | 608.2508239253 | Myanmar |
| 2017 | 583.91847327329 | 0.510823431317416 | 22.4651365104062 | 561.453336762884 | Myanmar |
| 2018 | 536.01524246445 | 0.517557469010629 | 22.2528341075522 | 513.762408356898 | Myanmar |
| 2019 | 495.18704896716 | 0.524108970350153 | 22.2138574000803 | 472.97319156708 | Myanmar |
| 2020 | 424.18417810265 | 0.529434702877063 | 22.1551648904216 | 402.029013212228 | Myanmar |
| 2021 | 390.05979826783 | 0.533900839611906 | 21.6900156946865 | 368.369782573144 | Myanmar |
| 1990 | 1642.0037599059 | 0.510011796397722 | 22.8625383103285 | 1619.14122159557 | Philippines |
| 1991 | 1491.58474819229 | 0.513707183203398 | 22.5260591826651 | 1469.05868900962 | Philippines |
| 1992 | 1379.09149316439 | 0.517109502298333 | 22.3243927354586 | 1356.76710042893 | Philippines |
| 1993 | 1278.53898398762 | 0.520600378172241 | 22.190291479178 | 1256.34869250844 | Philippines |
| 1994 | 1142.90636181369 | 0.524613606961947 | 22.2036590780058 | 1120.70270273568 | Philippines |
| 1995 | 1041.53932645622 | 0.528827907097189 | 22.1556734841622 | 1019.38365297206 | Philippines |
| 1996 | 943.44551410797 | 0.532938848718821 | 22.1548192745255 | 921.290694833444 | Philippines |
| 1997 | 862.19846901764 | 0.536589867582292 | 21.3767290487914 | 840.821739968849 | Philippines |
| 1998 | 792.97538439933 | 0.539496470377039 | 21.2117493398723 | 771.763635059458 | Philippines |
| 1999 | 795.65468614745 | 0.542257968063782 | 21.1733517809815 | 774.481334366468 | Philippines |
| 2000 | 777.94472965588 | 0.544997722532077 | 21.1645216276474 | 756.780208028233 | Philippines |
| 2001 | 733.1846378427 | 0.547409431560733 | 20.6231708611644 | 712.561466981536 | Philippines |
| 2002 | 677.91794567446 | 0.549521153378409 | 19.8593959112947 | 658.058549763165 | Philippines |
| 2003 | 621.57423938347 | 0.551567967518738 | 19.6294275095425 | 601.944811873927 | Philippines |
| 2004 | 553.7948491556 | 0.553704287358934 | 18.8224270899604 | 534.97242206564 | Philippines |
| 2005 | 543.11656723582 | 0.555664102826939 | 18.6171387677215 | 524.499428468098 | Philippines |
| 2006 | 517.96450929224 | 0.557976265254754 | 17.7525406301089 | 500.211968662131 | Philippines |
| 2007 | 494.81118882853 | 0.560822023745201 | 16.5523076679201 | 478.25888116061 | Philippines |
| 2008 | 471.40693816976 | 0.563974721376474 | 16.0603235562623 | 455.346614613498 | Philippines |
| 2009 | 449.8949858893 | 0.567355819963632 | 14.6984072882582 | 435.196578601042 | Philippines |
| 2010 | 424.23880994096 | 0.571765888250344 | 13.2977358869005 | 410.94107405406 | Philippines |
| 2011 | 403.27083012591 | 0.577240283466517 | 13.3980751825569 | 389.872754943353 | Philippines |
| 2012 | 367.81139157702 | 0.58376762501942 | 13.4395236150134 | 354.371867962007 | Philippines |
| 2013 | 331.51853493652 | 0.590837549266941 | 13.1568595640482 | 318.361675372472 | Philippines |
| 2014 | 310.06123051564 | 0.598384520042983 | 13.5682692440718 | 296.492961271568 | Philippines |
| 2015 | 290.31017104703 | 0.606215624393378 | 13.3063416310698 | 277.00382941596 | Philippines |
| 2016 | 283.09447076952 | 0.614224960974851 | 13.0179645201481 | 270.076506249372 | Philippines |
| 2017 | 265.82733260807 | 0.622315028960912 | 12.9139793708386 | 252.913353237231 | Philippines |
| 2018 | 251.53252271554 | 0.630464275930184 | 11.9863691562 | 239.54615355934 | Philippines |
| 2019 | 242.35026310697 | 0.638547384381803 | 8.09904233136848 | 234.251220775602 | Philippines |
| 2020 | 223.56197403589 | 0.644867233233664 | 5.81767653693422 | 217.744297498956 | Philippines |
| 2021 | 214.43625092352 | 0.651219328702707 | 5.67448501002701 | 208.761765913493 | Philippines |
| 1990 | 412.32337773188 | 0.522622552851521 | 22.1683485758185 | 390.155029156062 | Sri Lanka |
| 1991 | 338.96379341945 | 0.528239202759485 | 22.1652622897513 | 316.798531129699 | Sri Lanka |
| 1992 | 308.23499680225 | 0.533345535471094 | 22.1669408375204 | 286.06805596473 | Sri Lanka |
| 1993 | 292.90573581107 | 0.538891102297622 | 21.3030469656999 | 271.60268884537 | Sri Lanka |
| 1994 | 267.13691254625 | 0.545124551180714 | 21.1874682063842 | 245.949444339866 | Sri Lanka |
| 1995 | 218.82694471394 | 0.551744058346111 | 19.821114416472 | 199.005830297468 | Sri Lanka |
| 1996 | 208.06436237826 | 0.558938887614985 | 17.7156207720637 | 190.348741606196 | Sri Lanka |
| 1997 | 185.9823957488 | 0.566128422363729 | 15.3518888252404 | 170.63050692356 | Sri Lanka |
| 1998 | 156.9554085616 | 0.572326186668561 | 13.3462144910538 | 143.609194070546 | Sri Lanka |
| 1999 | 130.16638518685 | 0.57763004764168 | 13.3598985955024 | 116.806486591348 | Sri Lanka |
| 2000 | 118.28040425983 | 0.582942637570578 | 13.3928959052207 | 104.887508354609 | Sri Lanka |
| 2001 | 110.43488687827 | 0.587556599070554 | 13.4219208907662 | 97.0129659875038 | Sri Lanka |
| 2002 | 98.12223890041 | 0.59235912694583 | 12.8546491837956 | 85.2675897166144 | Sri Lanka |
| 2003 | 91.09977564572 | 0.597410284061105 | 13.4920564695052 | 77.6077191762148 | Sri Lanka |
| 2004 | 86.37599041653 | 0.60225006214936 | 13.5545826231896 | 72.8214077933404 | Sri Lanka |
| 2005 | 88.97879105834 | 0.606906655593188 | 13.4104658860715 | 75.5683251722685 | Sri Lanka |
| 2006 | 80.74815382223 | 0.611807083317854 | 13.4268445383686 | 67.3213092838614 | Sri Lanka |
| 2007 | 73.69136862002 | 0.616979265050006 | 13.2162628428788 | 60.4751057771412 | Sri Lanka |
| 2008 | 72.1675460848 | 0.622414317259024 | 13.0053748521889 | 59.1621712326111 | Sri Lanka |
| 2009 | 70.67626522553 | 0.628149630493025 | 12.5041112988979 | 58.1721539266321 | Sri Lanka |
| 2010 | 66.4889808183 | 0.634263123413356 | 10.9956275487474 | 55.4933532695526 | Sri Lanka |
| 2011 | 61.05132151922 | 0.640799571244289 | 7.50791064774838 | 53.5434108714716 | Sri Lanka |
| 2012 | 55.38951388949 | 0.648049815659369 | 6.21179690431887 | 49.1777169851711 | Sri Lanka |
| 2013 | 52.73870061009 | 0.655435837718287 | 4.95255448008612 | 47.7861461300039 | Sri Lanka |
| 2014 | 47.90611123296 | 0.662782434226903 | 4.46220583956063 | 43.4439053933994 | Sri Lanka |
| 2015 | 43.54673858166 | 0.669760291875565 | 3.66383936266551 | 39.8828992189945 | Sri Lanka |
| 2016 | 40.67617290138 | 0.67627744148358 | 3.174029495665 | 37.502143405715 | Sri Lanka |
| 2017 | 38.36326714375 | 0.682405274024772 | 2.7844671363605 | 35.5788000073895 | Sri Lanka |
| 2018 | 36.50012106454 | 0.688125858195178 | 2.52859222977819 | 33.9715288347618 | Sri Lanka |
| 2019 | 34.82413612536 | 0.69351606326589 | 2.36129223284114 | 32.4628438925189 | Sri Lanka |
| 2020 | 33.43514222118 | 0.697648535082413 | 2.43796171732613 | 30.9971805038539 | Sri Lanka |
| 2021 | 30.01901829598 | 0.70153493519423 | 2.39806250160474 | 27.6209557943753 | Sri Lanka |
| 1990 | 344.47788531453 | 0.506644860754466 | 22.7341221580081 | 321.743763156522 | Thailand |
| 1991 | 302.79240138188 | 0.515911465809998 | 22.3525528367401 | 280.43984854514 | Thailand |
| 1992 | 265.02975218268 | 0.525161868897941 | 22.1292357466281 | 242.900516436052 | Thailand |
| 1993 | 232.38756799672 | 0.534303709087494 | 21.5407418795534 | 210.846826117167 | Thailand |
| 1994 | 204.6123146135 | 0.543325546554827 | 21.1689204578457 | 183.443394155654 | Thailand |
| 1995 | 181.92167377564 | 0.552452208814004 | 19.8817963806537 | 162.039877394986 | Thailand |
| 1996 | 162.66824140663 | 0.561302486534141 | 16.3056605076891 | 146.362580898941 | Thailand |
| 1997 | 150.15473200919 | 0.568449492093676 | 13.9830303021842 | 136.171701707006 | Thailand |
| 1998 | 146.26026363506 | 0.573817902004847 | 13.406625276144 | 132.853638358916 | Thailand |
| 1999 | 141.35556427281 | 0.578854167794919 | 13.5492784207459 | 127.806285852064 | Thailand |
| 2000 | 137.73135593799 | 0.58350091705013 | 13.2154306562853 | 124.515925281705 | Thailand |
| 2001 | 125.14906726255 | 0.587661560775708 | 13.3154506305784 | 111.833616631972 | Thailand |
| 2002 | 114.6808166788 | 0.59175909789129 | 13.3870967164563 | 101.293719962344 | Thailand |
| 2003 | 103.90231763858 | 0.596048920136592 | 13.4133815730211 | 90.488936065559 | Thailand |
| 2004 | 95.4516078468 | 0.600520665140167 | 13.4010370083121 | 82.0505708384879 | Thailand |
| 2005 | 88.17399850971 | 0.605040775160747 | 13.2613129155187 | 74.9126855941913 | Thailand |
| 2006 | 85.05198248055 | 0.610356678024317 | 13.304923499744 | 71.747058980806 | Thailand |
| 2007 | 82.39516706983 | 0.616368591131489 | 13.0631607284464 | 69.3320063413836 | Thailand |
| 2008 | 76.92533600794 | 0.621067542847306 | 12.9238834667801 | 64.0014525411599 | Thailand |
| 2009 | 70.37902833502 | 0.624329070216978 | 12.99152720474 | 57.38750113028 | Thailand |
| 2010 | 67.68699174541 | 0.62858943983977 | 12.8160062593375 | 54.8709854860725 | Thailand |
| 2011 | 63.33129555083 | 0.633226727672561 | 11.1533968663919 | 52.1778986844381 | Thailand |
| 2012 | 60.65755724687 | 0.638410972213962 | 7.99857787844371 | 52.6589793684263 | Thailand |
| 2013 | 56.05266985388 | 0.643672915801455 | 7.59007609470437 | 48.4625937591756 | Thailand |
| 2014 | 53.836947964 | 0.648772057471972 | 5.67717038637872 | 48.1597775776213 | Thailand |
| 2015 | 52.42330087162 | 0.654015622617677 | 4.93404020967911 | 47.4892606619409 | Thailand |
| 2016 | 53.60637591084 | 0.659369302952365 | 4.68009661691045 | 48.9262792939295 | Thailand |
| 2017 | 52.56767600472 | 0.664804133690469 | 4.35718703947433 | 48.2104889652457 | Thailand |
| 2018 | 53.48988125494 | 0.670225586853729 | 3.72946249988313 | 49.7604187550569 | Thailand |
| 2019 | 51.3216063521 | 0.675273900833315 | 3.1825313877573 | 48.1390749643427 | Thailand |
| 2020 | 49.03111770214 | 0.679119989171207 | 2.74128542590338 | 46.2898322762366 | Thailand |
| 2021 | 47.34188959348 | 0.682547932974593 | 2.78706374774827 | 44.5548258457317 | Thailand |
| 1990 | 5103.71058242354 | 0.262468083434918 | 1387.87732443228 | 3715.83325799126 | Timor-Leste |
| 1991 | 4777.28246742299 | 0.270943348789144 | 1247.96806324724 | 3529.31440417575 | Timor-Leste |
| 1992 | 4441.50056937197 | 0.279916760012752 | 1193.45679926598 | 3248.04377010599 | Timor-Leste |
| 1993 | 4112.49796819004 | 0.289266628937942 | 1126.89412685688 | 2985.60384133316 | Timor-Leste |
| 1994 | 3765.64102968992 | 0.298763188841394 | 1071.07911865419 | 2694.56191103573 | Timor-Leste |
| 1995 | 3419.49648688777 | 0.308018683645478 | 973.879645409878 | 2445.61684147789 | Timor-Leste |
| 1996 | 3082.46653391908 | 0.317588750054214 | 826.27925969501 | 2256.18727422407 | Timor-Leste |
| 1997 | 2770.26334738078 | 0.326705373863583 | 761.555008425928 | 2008.70833895485 | Timor-Leste |
| 1998 | 2494.20157114465 | 0.334894497001343 | 673.965926535966 | 1820.23564460868 | Timor-Leste |
| 1999 | 2315.34928278305 | 0.339252140436899 | 599.524405572019 | 1715.82487721103 | Timor-Leste |
| 2000 | 2105.36847690787 | 0.343793649201481 | 590.264717320064 | 1515.10375958781 | Timor-Leste |
| 2001 | 1856.85870898601 | 0.349490320159863 | 600.456164949784 | 1256.40254403623 | Timor-Leste |
| 2002 | 1652.66301639477 | 0.354162019516716 | 588.986011641192 | 1063.67700475358 | Timor-Leste |
| 2003 | 1515.1414667922 | 0.35844002540281 | 588.682639260373 | 926.458827531827 | Timor-Leste |
| 2004 | 1281.68117355689 | 0.362687998477417 | 573.992653797075 | 707.688519759815 | Timor-Leste |
| 2005 | 1150.71384973888 | 0.366913584279333 | 573.466671627537 | 577.247178111343 | Timor-Leste |
| 2006 | 989.1529715135 | 0.370654092006941 | 549.446606102331 | 439.706365411169 | Timor-Leste |
| 2007 | 879.45533523426 | 0.375390767982559 | 547.527878416265 | 331.927456817995 | Timor-Leste |
| 2008 | 753.53509179339 | 0.381227527778766 | 513.996434882099 | 239.538656911291 | Timor-Leste |
| 2009 | 690.79106823666 | 0.387944823498752 | 507.984308878015 | 182.806759358645 | Timor-Leste |
| 2010 | 629.28713430872 | 0.394887019369648 | 466.572510312107 | 162.714623996613 | Timor-Leste |
| 2011 | 598.1719940628 | 0.401872356301348 | 393.635784722839 | 204.536209339961 | Timor-Leste |
| 2012 | 559.9980140792 | 0.408910300117845 | 313.784632488817 | 246.213381590383 | Timor-Leste |
| 2013 | 514.95421736603 | 0.41521787782997 | 270.983664513564 | 243.970552852466 | Timor-Leste |
| 2014 | 486.29935046384 | 0.421155537971188 | 242.835161501665 | 243.464188962175 | Timor-Leste |
| 2015 | 464.18956606345 | 0.426383620035982 | 235.610098725324 | 228.579467338126 | Timor-Leste |
| 2016 | 443.51019572573 | 0.431207272013621 | 208.417280853015 | 235.092914872715 | Timor-Leste |
| 2017 | 423.40324673871 | 0.434519733594658 | 200.569264037455 | 222.833982701255 | Timor-Leste |
| 2018 | 408.58796846506 | 0.437018662969572 | 118.680748910435 | 289.907219554625 | Timor-Leste |
| 2019 | 363.36305562743 | 0.44019584549571 | 124.694882465913 | 238.668173161517 | Timor-Leste |
| 2020 | 350.96393529076 | 0.442349336035765 | 109.938041750546 | 241.025893540214 | Timor-Leste |
| 2021 | 340.60052217594 | 0.444667618881833 | 105.213858662208 | 235.386663513732 | Timor-Leste |
| 1990 | 269.2203104505 | 0.407630047693296 | 269.2203104505 | 0 | Viet Nam |
| 1991 | 246.69009959841 | 0.413217249557429 | 246.69009959841 | 0 | Viet Nam |
| 1992 | 230.3206092597 | 0.419781657140794 | 230.3206092597 | 0 | Viet Nam |
| 1993 | 200.28164945016 | 0.427072184949202 | 200.28164945016 | 0 | Viet Nam |
| 1994 | 179.97884048308 | 0.435106272492167 | 175.978381593334 | 4.00045888974617 | Viet Nam |
| 1995 | 158.65533326077 | 0.443858785203356 | 111.685590751905 | 46.9697425088655 | Viet Nam |
| 1996 | 138.53117412476 | 0.453102410398964 | 66.0221384204621 | 72.5090357042979 | Viet Nam |
| 1997 | 123.66435963484 | 0.462853518365539 | 58.1287770288757 | 65.5355826059643 | Viet Nam |
| 1998 | 112.48253376999 | 0.47247889159957 | 56.049554759465 | 56.432979010525 | Viet Nam |
| 1999 | 102.70648104929 | 0.48144577410536 | 55.0681879874113 | 47.6382930618787 | Viet Nam |
| 2000 | 92.91361930935 | 0.489802045414549 | 50.0696718291527 | 42.8439474801973 | Viet Nam |
| 2001 | 82.38496711524 | 0.497630349065707 | 23.8696793302797 | 58.5152877849603 | Viet Nam |
| 2002 | 73.03181681545 | 0.505453447065355 | 22.9502635789308 | 50.0815532365192 | Viet Nam |
| 2003 | 66.64353756474 | 0.513367511118634 | 22.5091738559783 | 44.1343637087617 | Viet Nam |
| 2004 | 61.13673067928 | 0.521229389687461 | 22.1554770246512 | 38.9812536546288 | Viet Nam |
| 2005 | 54.8904021265 | 0.528956332075378 | 22.1635008131949 | 32.7269013133051 | Viet Nam |
| 2006 | 48.28359583465 | 0.53630065301368 | 21.3051118984453 | 26.9784839362047 | Viet Nam |
| 2007 | 42.27958664876 | 0.543342652110377 | 21.1683209131376 | 21.1112657356224 | Viet Nam |
| 2008 | 37.49611699033 | 0.550127119086299 | 19.8239268346076 | 17.6721901557224 | Viet Nam |
| 2009 | 33.35510930941 | 0.55645594448371 | 18.8300434804901 | 14.5250658289198 | Viet Nam |
| 2010 | 30.14168150941 | 0.562768571224979 | 16.6731860543933 | 13.4684954550167 | Viet Nam |
| 2011 | 28.1852705497 | 0.568945458254757 | 14.1927244924438 | 13.9925460572562 | Viet Nam |
| 2012 | 25.82291589248 | 0.575078671720175 | 13.2919793680186 | 12.5309365244614 | Viet Nam |
| 2013 | 23.57948109964 | 0.581029214733769 | 13.3321419771151 | 10.2473391225249 | Viet Nam |
| 2014 | 21.80442520951 | 0.586913814919078 | 13.5754510250038 | 8.22897418450619 | Viet Nam |
| 2015 | 20.88857681597 | 0.592849573281283 | 13.3355716918597 | 7.5530051241103 | Viet Nam |
| 2016 | 20.09856204625 | 0.598849485299412 | 13.4639353216441 | 6.63462672460587 | Viet Nam |
| 2017 | 19.64621848195 | 0.605019420569412 | 13.7207775782343 | 5.92544090371574 | Viet Nam |
| 2018 | 19.27287388675 | 0.611351406113218 | 13.2706789054107 | 6.00219498133932 | Viet Nam |
| 2019 | 18.62345776146 | 0.6177338152062 | 13.0889384521003 | 5.53451930935967 | Viet Nam |
| 2020 | 17.96272977193 | 0.623090320139283 | 12.76392850036 | 5.19880127157 | Viet Nam |
| 2021 | 15.62423654514 | 0.627933720680671 | 12.5464769560117 | 3.07775958912831 | Viet Nam |
| 1990 | 514.71113139313 | 0.53464890774309 | 21.5010349264316 | 493.210096466698 | Fiji |
| 1991 | 507.22890275121 | 0.538778259951718 | 21.2698008565576 | 485.959101894652 | Fiji |
| 1992 | 483.29974453999 | 0.543188551973659 | 21.2151938332454 | 462.084550706745 | Fiji |
| 1993 | 488.39043043216 | 0.547776473718793 | 20.4518467141931 | 467.938583717967 | Fiji |
| 1994 | 478.71825146286 | 0.552849019704515 | 19.8446065649742 | 458.873644897886 | Fiji |
| 1995 | 463.44175500894 | 0.558530972290103 | 17.8589664270625 | 445.582788581877 | Fiji |
| 1996 | 452.44658801346 | 0.565041878958725 | 16.1882353538085 | 436.258352659651 | Fiji |
| 1997 | 438.97699528609 | 0.571187752703497 | 13.3774610445937 | 425.599534241496 | Fiji |
| 1998 | 457.68587035256 | 0.577172353765379 | 13.3082782338736 | 444.377592118686 | Fiji |
| 1999 | 450.59633317203 | 0.583573942431191 | 13.4659142232697 | 437.13041894876 | Fiji |
| 2000 | 442.09266084579 | 0.589196631128333 | 13.3641375230392 | 428.728523322751 | Fiji |
| 2001 | 436.74347947889 | 0.59449741944042 | 13.3379499667551 | 423.405529512135 | Fiji |
| 2002 | 434.24043222756 | 0.59932203091066 | 13.2622462392412 | 420.978185988319 | Fiji |
| 2003 | 430.82054453726 | 0.603489622121197 | 13.1965801187778 | 417.623964418482 | Fiji |
| 2004 | 443.30083861198 | 0.607544889853603 | 13.3134902244688 | 429.987348387511 | Fiji |
| 2005 | 441.63019151239 | 0.610812943594047 | 13.2521063144775 | 428.378085197912 | Fiji |
| 2006 | 449.14532293498 | 0.613932767413255 | 13.3023032477438 | 435.843019687236 | Fiji |
| 2007 | 466.2661683416 | 0.616582915169589 | 12.9947904132839 | 453.271377928316 | Fiji |
| 2008 | 463.71856817363 | 0.618958809102014 | 12.9630963810037 | 450.755471792626 | Fiji |
| 2009 | 462.93732425441 | 0.620883465082065 | 12.895540474372 | 450.041783780038 | Fiji |
| 2010 | 464.35828098538 | 0.62330878605608 | 13.0684348758 | 451.28984610958 | Fiji |
| 2011 | 453.41550395502 | 0.626438251319165 | 13.0597132282427 | 440.355790726777 | Fiji |
| 2012 | 439.00830741529 | 0.629881334893454 | 12.0089636155628 | 426.999343799727 | Fiji |
| 2013 | 379.36406380292 | 0.634278114312534 | 11.0596222846172 | 368.304441518303 | Fiji |
| 2014 | 359.67591695021 | 0.6393900664467 | 7.36813391381175 | 352.307783036398 | Fiji |
| 2015 | 350.51468431039 | 0.645026352546626 | 5.99709209524758 | 344.517592215142 | Fiji |
| 2016 | 345.70471124909 | 0.650742680102873 | 5.4743919911416 | 340.230319257948 | Fiji |
| 2017 | 333.42885961009 | 0.656464010258719 | 5.04882875437539 | 328.380030855715 | Fiji |
| 2018 | 321.47796221757 | 0.661991453647996 | 4.72217612267337 | 316.755786094897 | Fiji |
| 2019 | 305.19307739553 | 0.667060979290814 | 3.76577564484236 | 301.427301750688 | Fiji |
| 2020 | 293.17710653384 | 0.671431490601499 | 3.24063204219525 | 289.936474491645 | Fiji |
| 2021 | 279.60141613471 | 0.675051630810876 | 3.107263432075 | 276.494152702635 | Fiji |
| 1990 | 5396.80759905192 | 0.410389821247376 | 317.505745459552 | 5079.30185359237 | Kiribati |
| 1991 | 5126.54653577641 | 0.412884199130178 | 294.214235939138 | 4832.33229983727 | Kiribati |
| 1992 | 4834.39949606331 | 0.415577609430112 | 265.17115213599 | 4569.22834392732 | Kiribati |
| 1993 | 4550.84344188855 | 0.418481660624087 | 263.288306418015 | 4287.55513547053 | Kiribati |
| 1994 | 4237.54005604247 | 0.421716217125176 | 243.166658701772 | 3994.3733973407 | Kiribati |
| 1995 | 3966.40784670153 | 0.425024354020195 | 231.36519512995 | 3735.04265157158 | Kiribati |
| 1996 | 3744.61709260855 | 0.428300682254543 | 214.614269907596 | 3530.00282270095 | Kiribati |
| 1997 | 3490.09903851862 | 0.431641940565898 | 203.497090089116 | 3286.6019484295 | Kiribati |
| 1998 | 3268.97189156122 | 0.435741904126434 | 183.287544795096 | 3085.68434676612 | Kiribati |
| 1999 | 3232.82325037242 | 0.439692923227333 | 114.77262726548 | 3118.05062310694 | Kiribati |
| 2000 | 3043.49649233269 | 0.444436339062538 | 109.520157476416 | 2933.97633485627 | Kiribati |
| 2001 | 2853.04354353606 | 0.448782300735609 | 82.143168882764 | 2770.9003746533 | Kiribati |
| 2002 | 2654.16017793487 | 0.453462297779448 | 67.0303070354101 | 2587.12987089946 | Kiribati |
| 2003 | 2469.74202117952 | 0.458208373737044 | 64.699494367498 | 2405.04252681202 | Kiribati |
| 2004 | 2293.39004342698 | 0.462336213231137 | 63.6979967642886 | 2229.69204666269 | Kiribati |
| 2005 | 2178.54641229527 | 0.466722276344394 | 58.5448866072567 | 2120.00152568801 | Kiribati |
| 2006 | 2098.24572563563 | 0.470557196660738 | 58.3437891962765 | 2039.90193643935 | Kiribati |
| 2007 | 2140.56726283023 | 0.474227450118041 | 57.1742748076351 | 2083.3929880226 | Kiribati |
| 2008 | 2105.78898663338 | 0.47720193306214 | 53.7253162723595 | 2052.06367036102 | Kiribati |
| 2009 | 2069.95597749823 | 0.480035601443872 | 52.9176866359358 | 2017.03829086229 | Kiribati |
| 2010 | 2042.65920131665 | 0.482447689410665 | 53.1245496961254 | 1989.53465162052 | Kiribati |
| 2011 | 2002.51514224348 | 0.485028211089828 | 52.4326660655975 | 1950.08247617788 | Kiribati |
| 2012 | 1882.68796208873 | 0.488175863692029 | 50.6907563670033 | 1831.99720572173 | Kiribati |
| 2013 | 1807.98534036804 | 0.491880767182311 | 50.4669568194962 | 1757.51838354854 | Kiribati |
| 2014 | 1738.46373153806 | 0.495317241227584 | 50.707137183217 | 1687.75659435484 | Kiribati |
| 2015 | 1548.365826741 | 0.500059699036215 | 23.388975173613 | 1524.97685156739 | Kiribati |
| 2016 | 1466.00436055723 | 0.505207837257253 | 23.0777586815497 | 1442.92660187568 | Kiribati |
| 2017 | 1407.33839728312 | 0.510019240979789 | 22.7038615513019 | 1384.63453573182 | Kiribati |
| 2018 | 1319.68306230463 | 0.514810383548853 | 22.5212736322564 | 1297.16178867237 | Kiribati |
| 2019 | 1242.43658323795 | 0.519441321098362 | 22.1950169703241 | 1220.24156626763 | Kiribati |
| 2020 | 1225.22174799316 | 0.523454113096381 | 22.1429703162821 | 1203.07877767688 | Kiribati |
| 2021 | 1176.63704848587 | 0.527186582842681 | 22.1543519622167 | 1154.48269652365 | Kiribati |
| 1990 | 789.50120907788 | 0.430839288199048 | 200.781326301822 | 588.719882776058 | Marshall Islands |
| 1991 | 778.47938251374 | 0.435571411977162 | 181.542899827769 | 596.936482685971 | Marshall Islands |
| 1992 | 738.7502893208 | 0.440491018138956 | 120.384292804732 | 618.365996516068 | Marshall Islands |
| 1993 | 692.46778627432 | 0.445479084259875 | 102.698177124508 | 589.769609149812 | Marshall Islands |
| 1994 | 652.28498232983 | 0.450848221273844 | 70.1427414893592 | 582.142240840471 | Marshall Islands |
| 1995 | 616.7471234798 | 0.45685967435209 | 63.5884217777745 | 553.158701702026 | Marshall Islands |
| 1996 | 596.57301822325 | 0.461209430189201 | 61.276381831393 | 535.296636391857 | Marshall Islands |
| 1997 | 567.26904419087 | 0.464752728246042 | 65.591486681845 | 501.677557509025 | Marshall Islands |
| 1998 | 559.11498472629 | 0.468133032354917 | 59.7277488190584 | 499.387235907232 | Marshall Islands |
| 1999 | 556.67758960215 | 0.471292726535272 | 55.29590712278 | 501.38168247937 | Marshall Islands |
| 2000 | 1764.36895239397 | 0.474562536385418 | 56.4305611151821 | 1707.93839127879 | Marshall Islands |
| 2001 | 539.7552748055 | 0.478588316369524 | 53.2635775650533 | 486.491697240447 | Marshall Islands |
| 2002 | 532.72951909155 | 0.482776259493159 | 52.9066900011949 | 479.822829090355 | Marshall Islands |
| 2003 | 534.44631957635 | 0.486248620277286 | 52.6667310744282 | 481.779588501922 | Marshall Islands |
| 2004 | 529.79155160343 | 0.489573077431328 | 49.8148083817127 | 479.976743221717 | Marshall Islands |
| 2005 | 524.09727082241 | 0.49327139054966 | 49.0987103668399 | 474.99856045557 | Marshall Islands |
| 2006 | 503.90083908556 | 0.497456174718598 | 29.2289467816384 | 474.671892303922 | Marshall Islands |
| 2007 | 484.30983873268 | 0.502250042471655 | 23.3773837339159 | 460.932454998764 | Marshall Islands |
| 2008 | 454.22163226837 | 0.506359587700651 | 22.7190002518836 | 431.502632016486 | Marshall Islands |
| 2009 | 420.67072754093 | 0.510794535917681 | 22.5376290900353 | 398.133098450895 | Marshall Islands |
| 2010 | 419.19844216805 | 0.51608513761736 | 22.2517272985457 | 396.946714869504 | Marshall Islands |
| 2011 | 406.54974882781 | 0.521177086955837 | 22.1785114648799 | 384.37123736293 | Marshall Islands |
| 2012 | 394.69893205355 | 0.526042019095978 | 22.1099727320424 | 372.588959321508 | Marshall Islands |
| 2013 | 380.53371080693 | 0.531446300319586 | 22.1343427745893 | 358.399368032341 | Marshall Islands |
| 2014 | 371.25071355334 | 0.536640130947104 | 21.3797599098686 | 349.870953643471 | Marshall Islands |
| 2015 | 361.07790187224 | 0.541793166838195 | 21.2058272364982 | 339.872074635742 | Marshall Islands |
| 2016 | 369.46881892661 | 0.546782936000428 | 20.4584455639512 | 349.010373362659 | Marshall Islands |
| 2017 | 339.20882648158 | 0.55206515210282 | 19.8658238180275 | 319.343002663552 | Marshall Islands |
| 2018 | 323.28778656501 | 0.557599278468739 | 17.5984032307496 | 305.68938333426 | Marshall Islands |
| 2019 | 302.11740391162 | 0.563428749789962 | 16.1880443430736 | 285.929359568546 | Marshall Islands |
| 2020 | 310.46318773883 | 0.568837509186186 | 14.0225084103565 | 296.440679328474 | Marshall Islands |
| 2021 | 293.85209788493 | 0.574091128177309 | 13.5558896889921 | 280.296208195938 | Marshall Islands |
| 1990 | 910.02111078903 | 0.462511616715497 | 64.2319283743862 | 845.789182414644 | Micronesia (Federated States of) |
| 1991 | 784.6007904305 | 0.468144831239529 | 57.6020888467741 | 726.998701583726 | Micronesia (Federated States of) |
| 1992 | 744.0443387694 | 0.473792936437038 | 56.1874539526926 | 687.856884816708 | Micronesia (Federated States of) |
| 1993 | 700.64242937127 | 0.479939595446532 | 53.9690835436542 | 646.673345827616 | Micronesia (Federated States of) |
| 1994 | 663.33916929319 | 0.485300005800881 | 51.8649816008128 | 611.474187692377 | Micronesia (Federated States of) |
| 1995 | 616.24192251088 | 0.491015185412641 | 49.3550339655093 | 566.886888545371 | Micronesia (Federated States of) |
| 1996 | 584.84264166925 | 0.495570308271861 | 50.2395142913721 | 534.603127377878 | Micronesia (Federated States of) |
| 1997 | 535.7738078159 | 0.499110105936474 | 24.122122829356 | 511.651684986544 | Micronesia (Federated States of) |
| 1998 | 503.57386446354 | 0.5028615644834 | 22.9150869774546 | 480.658777486085 | Micronesia (Federated States of) |
| 1999 | 472.6172280679 | 0.506663430751627 | 22.7074909566031 | 449.909737111297 | Micronesia (Federated States of) |
| 2000 | 2416.88712683437 | 0.511004227582949 | 22.4375324112419 | 2394.44959442313 | Micronesia (Federated States of) |
| 2001 | 408.86853985467 | 0.515316814346477 | 22.430228406574 | 386.438311448096 | Micronesia (Federated States of) |
| 2002 | 382.06166523634 | 0.519545671945836 | 22.2804292384166 | 359.781235997923 | Micronesia (Federated States of) |
| 2003 | 358.44767462106 | 0.523829598854531 | 22.1061296613844 | 336.341544959676 | Micronesia (Federated States of) |
| 2004 | 348.20900880539 | 0.527610964634803 | 22.1129533516451 | 326.096055453745 | Micronesia (Federated States of) |
| 2005 | 346.35865495768 | 0.531703321036516 | 22.1487591844074 | 324.209895773273 | Micronesia (Federated States of) |
| 2006 | 328.63653769309 | 0.535718681873052 | 21.5903254052275 | 307.046212287863 | Micronesia (Federated States of) |
| 2007 | 311.2738872256 | 0.53943146430145 | 21.1678283004702 | 290.10605892513 | Micronesia (Federated States of) |
| 2008 | 277.94650874253 | 0.542766353836032 | 21.1421618654575 | 256.804346877072 | Micronesia (Federated States of) |
| 2009 | 264.3567605595 | 0.546275496220168 | 20.6110094486791 | 243.745751110821 | Micronesia (Federated States of) |
| 2010 | 251.09106696354 | 0.550122546321787 | 19.7767111934787 | 231.314355770061 | Micronesia (Federated States of) |
| 2011 | 263.57732833202 | 0.554240410692063 | 18.8194838337668 | 244.757844498253 | Micronesia (Federated States of) |
| 2012 | 262.19926007444 | 0.557965852891456 | 17.5201988754494 | 244.679061198991 | Micronesia (Federated States of) |
| 2013 | 260.11946535977 | 0.561082820885874 | 16.6289282171459 | 243.490537142624 | Micronesia (Federated States of) |
| 2014 | 258.64041172601 | 0.563840029745074 | 16.0995097716943 | 242.540901954316 | Micronesia (Federated States of) |
| 2015 | 256.76651031026 | 0.567151341569495 | 14.6625703930764 | 242.103939917184 | Micronesia (Federated States of) |
| 2016 | 253.59467873777 | 0.570458101091096 | 13.3246491292102 | 240.27002960856 | Micronesia (Federated States of) |
| 2017 | 243.69018916488 | 0.574039832793086 | 13.566919160031 | 230.123270004849 | Micronesia (Federated States of) |
| 2018 | 231.183136477 | 0.577555080818811 | 13.215164674953 | 217.967971802047 | Micronesia (Federated States of) |
| 2019 | 217.34403770696 | 0.581195254501754 | 13.354302583512 | 203.989735123448 | Micronesia (Federated States of) |
| 2020 | 195.99829541078 | 0.584527228769055 | 13.6039440785792 | 182.394351332201 | Micronesia (Federated States of) |
| 2021 | 185.94401022521 | 0.587534967103163 | 13.3519820756256 | 172.592028149584 | Micronesia (Federated States of) |
| 1990 | 6136.96848878275 | 0.310668628637835 | 977.544859796375 | 5159.42362898637 | Papua New Guinea |
| 1991 | 5957.93695288538 | 0.314967305387282 | 837.151425494011 | 5120.78552739137 | Papua New Guinea |
| 1992 | 5728.45093381186 | 0.319530259271955 | 834.943667434019 | 4893.50726637784 | Papua New Guinea |
| 1993 | 5430.58612189794 | 0.32500598378048 | 758.430680588159 | 4672.15544130978 | Papua New Guinea |
| 1994 | 5090.16284127739 | 0.330664467960637 | 680.094918538664 | 4410.06792273873 | Papua New Guinea |
| 1995 | 4782.20120849764 | 0.335421285048649 | 666.714584271646 | 4115.48662422599 | Papua New Guinea |
| 1996 | 4588.28434802103 | 0.340348698133771 | 593.535322826552 | 3994.74902519448 | Papua New Guinea |
| 1997 | 4391.05835287977 | 0.344258324561358 | 586.424431164447 | 3804.63392171532 | Papua New Guinea |
| 1998 | 4483.55433889246 | 0.347801407739934 | 595.188841692055 | 3888.3654972004 | Papua New Guinea |
| 1999 | 4438.6088970071 | 0.351250620970096 | 586.537202035442 | 3852.07169497166 | Papua New Guinea |
| 2000 | 4380.6825283349 | 0.354135790847746 | 584.191838928969 | 3796.49068940593 | Papua New Guinea |
| 2001 | 4236.56669288782 | 0.356588986333728 | 592.772708203817 | 3643.793984684 | Papua New Guinea |
| 2002 | 4128.15310638167 | 0.358572086402703 | 592.044583219987 | 3536.10852316168 | Papua New Guinea |
| 2003 | 3915.59095504133 | 0.360736816415114 | 588.960424433686 | 3326.63053060764 | Papua New Guinea |
| 2004 | 3864.28475493399 | 0.36282754151245 | 577.912983121388 | 3286.3717718126 | Papua New Guinea |
| 2005 | 3980.10602687689 | 0.365294964904992 | 575.488559154733 | 3404.61746772216 | Papua New Guinea |
| 2006 | 4022.89762406377 | 0.367826759122837 | 571.99011516271 | 3450.90750890106 | Papua New Guinea |
| 2007 | 4029.53024359532 | 0.370794146921454 | 545.270549204002 | 3484.25969439132 | Papua New Guinea |
| 2008 | 3977.55268333461 | 0.37342620922524 | 547.451271608788 | 3430.10141172582 | Papua New Guinea |
| 2009 | 4107.61334607534 | 0.376528284761462 | 550.830666959231 | 3556.78267911611 | Papua New Guinea |
| 2010 | 4066.60900362289 | 0.380286777637627 | 518.73057377622 | 3547.87842984667 | Papua New Guinea |
| 2011 | 3814.07904665515 | 0.383587539302927 | 510.002654806896 | 3304.07639184825 | Papua New Guinea |
| 2012 | 3739.32273871547 | 0.386781027656579 | 512.510403313722 | 3226.81233540175 | Papua New Guinea |
| 2013 | 3625.99189454578 | 0.389926437008588 | 512.596232495773 | 3113.39566205001 | Papua New Guinea |
| 2014 | 3504.52402758045 | 0.394004571266826 | 463.388412455576 | 3041.13561512487 | Papua New Guinea |
| 2015 | 3326.84877699129 | 0.398285863731033 | 454.85149941466 | 2871.99727757663 | Papua New Guinea |
| 2016 | 3252.98111472403 | 0.402407266844816 | 393.90378090208 | 2859.07733382195 | Papua New Guinea |
| 2017 | 3148.75237502552 | 0.406153435448224 | 360.069562378527 | 2788.68281264699 | Papua New Guinea |
| 2018 | 2987.36368677563 | 0.409165082697567 | 313.295914280355 | 2674.06777249527 | Papua New Guinea |
| 2019 | 2864.73112440789 | 0.412311146526574 | 298.615763365213 | 2566.11536104268 | Papua New Guinea |
| 2020 | 2811.09388861785 | 0.415186878578667 | 271.433026574696 | 2539.66086204315 | Papua New Guinea |
| 2021 | 2677.06314080873 | 0.417797443084353 | 257.003566451107 | 2420.05957435762 | Papua New Guinea |
| 1990 | 193.2396281846 | 0.48749142771166 | 50.5203664467597 | 142.71926173784 | Samoa |
| 1991 | 183.16480889464 | 0.490987145586308 | 48.8710107677278 | 134.293798126912 | Samoa |
| 1992 | 173.95428513315 | 0.494027447933208 | 51.2739568389138 | 122.680328294236 | Samoa |
| 1993 | 165.24436244716 | 0.497079263536296 | 32.2230202332659 | 133.021342213894 | Samoa |
| 1994 | 157.03214991157 | 0.499484836329879 | 24.2438230969171 | 132.788326814653 | Samoa |
| 1995 | 149.23521429701 | 0.502369734936465 | 23.4281867553566 | 125.807027541653 | Samoa |
| 1996 | 142.45662981715 | 0.505778026475631 | 22.9581256052284 | 119.498504211922 | Samoa |
| 1997 | 136.41711102186 | 0.509096382265271 | 22.6876885230576 | 113.729422498802 | Samoa |
| 1998 | 128.61224472086 | 0.512367565788016 | 22.426771574755 | 106.185473146105 | Samoa |
| 1999 | 132.09974856041 | 0.515409049865666 | 22.1986872252833 | 109.901061335127 | Samoa |
| 2000 | 125.87488604982 | 0.518902326533937 | 22.2217926169387 | 103.653093432881 | Samoa |
| 2001 | 119.9419874327 | 0.522971625527944 | 22.1216238916544 | 97.8203635410456 | Samoa |
| 2002 | 112.54050785305 | 0.527242669970423 | 22.1695037260883 | 90.3710041269617 | Samoa |
| 2003 | 106.83309520301 | 0.531603736377418 | 22.1472353666158 | 84.6858598363943 | Samoa |
| 2004 | 103.22518104863 | 0.53600327592574 | 21.4791161800071 | 81.7460648686229 | Samoa |
| 2005 | 101.38436071215 | 0.540557907991258 | 21.2380872758719 | 80.1462734362781 | Samoa |
| 2006 | 97.80964388121 | 0.544812068358444 | 21.187712274534 | 76.621931606676 | Samoa |
| 2007 | 94.79698347988 | 0.54911075247208 | 20.3040120958738 | 74.4929713840062 | Samoa |
| 2008 | 92.55729993303 | 0.553164595646872 | 19.8397814994539 | 72.7175184335761 | Samoa |
| 2009 | 92.08456180455 | 0.556474015274728 | 18.7776865022791 | 73.3068753022709 | Samoa |
| 2010 | 94.11442155129 | 0.559622905103398 | 17.5489808884352 | 76.5654406628548 | Samoa |
| 2011 | 93.84767896382 | 0.562891574259145 | 16.5823749604722 | 77.2653040033479 | Samoa |
| 2012 | 92.88516890869 | 0.565320388101849 | 15.309591274748 | 77.575577633942 | Samoa |
| 2013 | 89.85709512425 | 0.56744853592103 | 14.6938655546093 | 75.1632295696407 | Samoa |
| 2014 | 87.42457299082 | 0.569493660728475 | 14.1566715566722 | 73.2679014341478 | Samoa |
| 2015 | 84.56974151854 | 0.571998982964235 | 13.2292605010836 | 71.3404810174564 | Samoa |
| 2016 | 82.93868577321 | 0.575402051396993 | 13.4503272852481 | 69.4883584879619 | Samoa |
| 2017 | 82.41677883165 | 0.578925435094317 | 13.4048630144257 | 69.0119158172243 | Samoa |
| 2018 | 83.71464966834 | 0.582320660518804 | 13.8587364646448 | 69.8559132036952 | Samoa |
| 2019 | 82.00439615628 | 0.586256695142635 | 13.1972941241458 | 68.8071020321342 | Samoa |
| 2020 | 82.95093177406 | 0.590075295612806 | 13.282123961937 | 69.668807812123 | Samoa |
| 2021 | 76.92397721032 | 0.593392769469481 | 13.4083442578397 | 63.5156329524803 | Samoa |
| 1990 | 1554.84549311979 | 0.301217167448465 | 1013.858081912 | 540.987411207789 | Solomon Islands |
| 1991 | 1491.13766750743 | 0.305830667512655 | 1028.13683352296 | 463.00083398447 | Solomon Islands |
| 1992 | 1404.75312140974 | 0.311431696691098 | 957.247890572373 | 447.505230837367 | Solomon Islands |
| 1993 | 1320.31177788748 | 0.31715109185957 | 845.649440484812 | 474.662337402668 | Solomon Islands |
| 1994 | 1238.39906187597 | 0.323300246722871 | 761.251143270082 | 477.147918605888 | Solomon Islands |
| 1995 | 1145.90994590773 | 0.329968564261603 | 679.279288181985 | 466.630657725745 | Solomon Islands |
| 1996 | 1073.27482704566 | 0.336208053088528 | 668.303815345365 | 404.971011700295 | Solomon Islands |
| 1997 | 1006.46782532067 | 0.341835626898821 | 591.713458187016 | 414.754367133654 | Solomon Islands |
| 1998 | 1089.46448153588 | 0.347135060018002 | 596.026095533031 | 493.438386002849 | Solomon Islands |
| 1999 | 1081.92955203391 | 0.351867191410364 | 602.250725817619 | 479.678826216291 | Solomon Islands |
| 2000 | 1043.27960354215 | 0.354799143417026 | 591.038448187851 | 452.241155354299 | Solomon Islands |
| 2001 | 995.03764882788 | 0.356626745168074 | 597.826346730972 | 397.211302096908 | Solomon Islands |
| 2002 | 944.14678219583 | 0.357910115982585 | 588.461913964335 | 355.684868231495 | Solomon Islands |
| 2003 | 848.62803901595 | 0.359424358912125 | 597.841393904257 | 250.786645111693 | Solomon Islands |
| 2004 | 747.47322839803 | 0.361290558162268 | 586.818713481032 | 160.654514916998 | Solomon Islands |
| 2005 | 718.89093673153 | 0.363580018964866 | 581.98643311869 | 136.90450361284 | Solomon Islands |
| 2006 | 688.28219469907 | 0.366443697706899 | 571.956727550126 | 116.325467148944 | Solomon Islands |
| 2007 | 706.5820964415 | 0.369827951415349 | 583.985414560085 | 122.596681881415 | Solomon Islands |
| 2008 | 675.87748360389 | 0.3737648835675 | 540.419104919141 | 135.458378684749 | Solomon Islands |
| 2009 | 641.93883128789 | 0.377821027515966 | 542.272538741427 | 99.6662925464626 | Solomon Islands |
| 2010 | 628.54360132081 | 0.382717589747291 | 501.674316141813 | 126.869285178997 | Solomon Islands |
| 2011 | 589.4688433887 | 0.388055336574909 | 508.22951442616 | 81.2393289625396 | Solomon Islands |
| 2012 | 537.57819367149 | 0.393004639707306 | 454.865293780445 | 82.7128998910452 | Solomon Islands |
| 2013 | 528.78843934909 | 0.397906249572343 | 455.147546529896 | 73.6408928191944 | Solomon Islands |
| 2014 | 500.66967751856 | 0.402244744701609 | 399.097315391493 | 101.572362127067 | Solomon Islands |
| 2015 | 526.03379335888 | 0.406233896996201 | 364.979569668071 | 161.054223690809 | Solomon Islands |
| 2016 | 502.59497962414 | 0.410406962023453 | 301.518922555821 | 201.076057068319 | Solomon Islands |
| 2017 | 478.67038176116 | 0.414676394150034 | 268.012014483947 | 210.658367277213 | Solomon Islands |
| 2018 | 451.70124532968 | 0.418889220879761 | 261.555073750938 | 190.146171578742 | Solomon Islands |
| 2019 | 422.83871858506 | 0.422736386173248 | 244.231592854751 | 178.60712573031 | Solomon Islands |
| 2020 | 346.88297369529 | 0.426112428669488 | 232.697080691701 | 114.185893003589 | Solomon Islands |
| 2021 | 333.86813105479 | 0.429360316406646 | 204.382671454783 | 129.485459600007 | Solomon Islands |
| 1990 | 235.93447963774 | 0.491806839500397 | 49.2487022027822 | 186.685777434958 | Tonga |
| 1991 | 224.60313237525 | 0.498166205111113 | 24.253383724749 | 200.349748650501 | Tonga |
| 1992 | 213.25833982788 | 0.503659848303175 | 22.9676653656851 | 190.290674462195 | Tonga |
| 1993 | 205.38009481521 | 0.508904449646887 | 22.6970612872979 | 182.683033527912 | Tonga |
| 1994 | 196.52109902554 | 0.514207834722848 | 22.5132100922233 | 174.007888933317 | Tonga |
| 1995 | 187.7200686707 | 0.520215020279115 | 22.1435965291413 | 165.576472141559 | Tonga |
| 1996 | 180.39234579858 | 0.525808135436488 | 22.1235736288015 | 158.268772169779 | Tonga |
| 1997 | 169.48075753183 | 0.531009091678332 | 22.1113246185306 | 147.369432913299 | Tonga |
| 1998 | 163.2098547968 | 0.536172313703023 | 21.3872178214044 | 141.822636975396 | Tonga |
| 1999 | 172.40978920046 | 0.541317019874755 | 21.1942249343705 | 151.21556426609 | Tonga |
| 2000 | 165.93589591243 | 0.546303195085387 | 20.6813551197192 | 145.254540792711 | Tonga |
| 2001 | 158.21832879924 | 0.551054251063729 | 19.5880475485153 | 138.630281250725 | Tonga |
| 2002 | 149.74536281486 | 0.556008345246101 | 18.8254020125942 | 130.919960802266 | Tonga |
| 2003 | 141.98866495627 | 0.560736066200254 | 17.4190668145999 | 124.56959814167 | Tonga |
| 2004 | 132.0947394795 | 0.564806378895597 | 16.1712695037763 | 115.923469975724 | Tonga |
| 2005 | 131.30965765368 | 0.568449169182724 | 14.1644007188589 | 117.145256934821 | Tonga |
| 2006 | 132.24484752883 | 0.57161986831651 | 13.4349288488418 | 118.809918679988 | Tonga |
| 2007 | 130.12072663488 | 0.574351510103056 | 13.5297491643936 | 116.590977470486 | Tonga |
| 2008 | 126.10675210619 | 0.57730541521781 | 13.3355223456678 | 112.771229760522 | Tonga |
| 2009 | 125.74280018467 | 0.579170749598751 | 13.5869874760487 | 112.155812708621 | Tonga |
| 2010 | 125.60255997592 | 0.580657298614066 | 13.4682876609138 | 112.134272315006 | Tonga |
| 2011 | 123.37903815542 | 0.58265672287008 | 13.4007215398969 | 109.978316615523 | Tonga |
| 2012 | 120.59795875138 | 0.584770880221314 | 13.353563718269 | 107.244395033111 | Tonga |
| 2013 | 118.16723391723 | 0.587351141040311 | 13.5581403848116 | 104.609093532418 | Tonga |
| 2014 | 115.22014986705 | 0.590826852746506 | 13.3313416695018 | 101.888808197548 | Tonga |
| 2015 | 113.26486571748 | 0.594965747674029 | 13.3170214912067 | 99.9478442262733 | Tonga |
| 2016 | 111.14831132743 | 0.60034366853075 | 13.286961562861 | 97.861349764569 | Tonga |
| 2017 | 106.07422739258 | 0.606243591849879 | 13.5238609637834 | 92.5503664287966 | Tonga |
| 2018 | 102.01495138265 | 0.611953941537156 | 13.27427652828 | 88.74067485437 | Tonga |
| 2019 | 97.63250829619 | 0.617324063407956 | 13.0673093073123 | 84.5651989888777 | Tonga |
| 2020 | 97.50433033418 | 0.622104455604093 | 13.078853613258 | 84.425476720922 | Tonga |
| 2021 | 83.6447413147 | 0.626349935807987 | 12.9081903237526 | 70.7365509909474 | Tonga |
| 1990 | 1316.88550659598 | 0.353100252005321 | 600.18536909977 | 716.70013749621 | Vanuatu |
| 1991 | 1262.71766939195 | 0.357005378847917 | 583.011780336427 | 679.705889055523 | Vanuatu |
| 1992 | 1196.11981000927 | 0.360893295561675 | 592.152641683404 | 603.967168325866 | Vanuatu |
| 1993 | 1151.42057220174 | 0.364444155251801 | 575.673094903617 | 575.747477298123 | Vanuatu |
| 1994 | 1098.87593972644 | 0.368501002014456 | 576.556851308227 | 522.319088418213 | Vanuatu |
| 1995 | 1051.25663147785 | 0.372479125756324 | 548.538734447598 | 502.717897030252 | Vanuatu |
| 1996 | 1015.14564748276 | 0.376460884544884 | 550.344999842691 | 464.800647640069 | Vanuatu |
| 1997 | 968.94086418161 | 0.380667171234513 | 503.944577216881 | 464.996286964729 | Vanuatu |
| 1998 | 927.90288503381 | 0.384690196444633 | 513.879825289749 | 414.023059744061 | Vanuatu |
| 1999 | 1033.55780029154 | 0.388300526237242 | 504.178340684502 | 529.379459607038 | Vanuatu |
| 2000 | 1016.27196311212 | 0.392158348897928 | 460.103264110593 | 556.168699001527 | Vanuatu |
| 2001 | 982.34338858725 | 0.395325055353778 | 457.942479001866 | 524.400909585384 | Vanuatu |
| 2002 | 933.2095779961 | 0.397723782197823 | 457.325706778587 | 475.883871217513 | Vanuatu |
| 2003 | 871.85265307974 | 0.400396455507663 | 466.787008761811 | 405.065644317929 | Vanuatu |
| 2004 | 767.28722521796 | 0.403226096857709 | 391.47599853616 | 375.8112266818 | Vanuatu |
| 2005 | 734.95505120027 | 0.406488572045146 | 362.787650256352 | 372.167400943918 | Vanuatu |
| 2006 | 689.98753621523 | 0.410501728664985 | 314.012145133916 | 375.975391081314 | Vanuatu |
| 2007 | 728.75560907402 | 0.41491557794022 | 270.336708438746 | 458.418900635274 | Vanuatu |
| 2008 | 701.92657584357 | 0.41985323747475 | 247.019333789658 | 454.907242053912 | Vanuatu |
| 2009 | 666.38823951886 | 0.424854650160333 | 233.377205504768 | 433.011034014092 | Vanuatu |
| 2010 | 632.84298456557 | 0.429658048936263 | 207.191309453611 | 425.651675111959 | Vanuatu |
| 2011 | 591.63555709327 | 0.434283044387506 | 204.780948472526 | 386.854608620744 | Vanuatu |
| 2012 | 524.10090609869 | 0.438661997905717 | 118.001118122789 | 406.099787975901 | Vanuatu |
| 2013 | 487.86408053333 | 0.442720176072726 | 108.929838802712 | 378.934241730618 | Vanuatu |
| 2014 | 483.02581168911 | 0.446687298111776 | 80.3388512214599 | 402.68696046765 | Vanuatu |
| 2015 | 468.45292663018 | 0.450329861394857 | 70.4591303526826 | 397.993796277497 | Vanuatu |
| 2016 | 512.12301673324 | 0.454072763242304 | 72.8424841153869 | 439.280532617853 | Vanuatu |
| 2017 | 502.75252232471 | 0.45801683397471 | 67.7034723855653 | 435.049049939145 | Vanuatu |
| 2018 | 491.36298763089 | 0.462080220969235 | 65.6035154518953 | 425.759472178995 | Vanuatu |
| 2019 | 465.49659981444 | 0.466276161662358 | 56.9918992368422 | 408.504700577598 | Vanuatu |
| 2020 | 443.52441145883 | 0.470038390856612 | 57.5091399273142 | 386.015271531516 | Vanuatu |
| 2021 | 384.34258914226 | 0.473100705820327 | 58.3328088605352 | 326.009780281725 | Vanuatu |
| 1990 | 890.23326473946 | 0.54441453483767 | 21.1587402926342 | 869.074524446826 | Armenia |
| 1991 | 876.60028688687 | 0.547561862706728 | 20.402798442277 | 856.197488444593 | Armenia |
| 1992 | 915.34893541469 | 0.548974723873954 | 20.675391251766 | 894.673544162924 | Armenia |
| 1993 | 861.07321181439 | 0.551214673543035 | 19.8545924479499 | 841.21861936644 | Armenia |
| 1994 | 834.01011344812 | 0.553783844340157 | 18.620008747871 | 815.390104700249 | Armenia |
| 1995 | 755.76886452059 | 0.556815513215437 | 18.8033141956867 | 736.965550324903 | Armenia |
| 1996 | 656.69641695179 | 0.560537777557715 | 17.5568321789249 | 639.139584772865 | Armenia |
| 1997 | 603.31741911704 | 0.564627461798536 | 15.7910527176307 | 587.526366399409 | Armenia |
| 1998 | 514.52091567495 | 0.569556662578666 | 14.0300395208226 | 500.490876154127 | Armenia |
| 1999 | 437.88917610021 | 0.574323064436665 | 13.4610746415685 | 424.428101458642 | Armenia |
| 2000 | 381.54605464756 | 0.579437569012042 | 13.6389285357574 | 367.907126111803 | Armenia |
| 2001 | 338.0194403724 | 0.585419295040696 | 13.3842493653172 | 324.635191007083 | Armenia |
| 2002 | 278.45287490161 | 0.592710614375924 | 13.4336864189347 | 265.019188482675 | Armenia |
| 2003 | 240.42777072544 | 0.600809170405726 | 13.387099534924 | 227.040671190516 | Armenia |
| 2004 | 222.09491032306 | 0.60903176604033 | 13.1455174535128 | 208.949392869547 | Armenia |
| 2005 | 200.51099338135 | 0.617759634102041 | 13.0043718974043 | 187.506621483946 | Armenia |
| 2006 | 180.55055206617 | 0.626688335239636 | 13.0308552899159 | 167.519696776254 | Armenia |
| 2007 | 131.72318377882 | 0.635870981516541 | 10.4925429529417 | 121.230640825878 | Armenia |
| 2008 | 111.22949934749 | 0.64443159113194 | 5.86856875100612 | 105.360930596484 | Armenia |
| 2009 | 86.63206307529 | 0.649714844735141 | 5.2952262599279 | 81.3368368153621 | Armenia |
| 2010 | 65.29702568587 | 0.654811533750253 | 5.15920085380553 | 60.1378248320645 | Armenia |
| 2011 | 56.29964631712 | 0.659818092317619 | 4.47083182666603 | 51.828814490454 | Armenia |
| 2012 | 37.5845059943 | 0.664833142570704 | 4.42043018278185 | 33.1640758115182 | Armenia |
| 2013 | 31.4214085676 | 0.669534495666631 | 3.55080448270446 | 27.8706040848955 | Armenia |
| 2014 | 28.79686025405 | 0.673932189530685 | 3.19819408513358 | 25.5986661689164 | Armenia |
| 2015 | 26.35460420217 | 0.678054634729575 | 2.94539129407708 | 23.4092129080929 | Armenia |
| 2016 | 30.13957958063 | 0.681746395416543 | 2.76151997954607 | 27.3780596010839 | Armenia |
| 2017 | 24.47121281808 | 0.685980265385199 | 2.77235110346307 | 21.6988617146169 | Armenia |
| 2018 | 24.69674075103 | 0.690276871944721 | 2.59143413664464 | 22.1053066143854 | Armenia |
| 2019 | 24.54877823347 | 0.694765961567774 | 2.48867503271691 | 22.0601032007531 | Armenia |
| 2020 | 22.91063316932 | 0.69826889937921 | 2.34421021653723 | 20.5664229527828 | Armenia |
| 2021 | 21.38529570891 | 0.701833194147139 | 2.34657712664111 | 19.0387185822689 | Armenia |
| 1990 | 1591.78506561932 | 0.595986032704332 | 13.5230430297834 | 1578.26202258954 | Azerbaijan |
| 1991 | 1509.04222995546 | 0.595807711845649 | 13.3356573327879 | 1495.70657262267 | Azerbaijan |
| 1992 | 1440.19898705271 | 0.594651335166956 | 13.3046575093513 | 1426.89432954336 | Azerbaijan |
| 1993 | 1382.4104511177 | 0.59354267649819 | 13.3482094534522 | 1369.06224166425 | Azerbaijan |
| 1994 | 1321.41042541062 | 0.5920887850585 | 13.2199766136023 | 1308.19044879702 | Azerbaijan |
| 1995 | 1256.22051976551 | 0.589441456728463 | 13.6014275075197 | 1242.61909225799 | Azerbaijan |
| 1996 | 1196.34645591133 | 0.586315946466504 | 13.5289225659887 | 1182.81753334534 | Azerbaijan |
| 1997 | 1132.48460327137 | 0.583221812602983 | 13.680513778405 | 1118.80408949296 | Azerbaijan |
| 1998 | 1076.33666716865 | 0.580392893004131 | 13.2171760252329 | 1063.11949114342 | Azerbaijan |
| 1999 | 1016.35258024367 | 0.579192506195412 | 13.4554283480091 | 1002.89715189566 | Azerbaijan |
| 2000 | 937.87937944438 | 0.580020573148448 | 13.3284187261057 | 924.550960718274 | Azerbaijan |
| 2001 | 832.52820285116 | 0.582193708342128 | 13.2910350146921 | 819.237167836468 | Azerbaijan |
| 2002 | 752.81975810891 | 0.585716143276774 | 13.3217453179101 | 739.498012791 | Azerbaijan |
| 2003 | 695.82540124878 | 0.590038705393348 | 13.2837358694046 | 682.541665379375 | Azerbaijan |
| 2004 | 646.84461929822 | 0.594646084750935 | 13.3556123918444 | 633.489006906376 | Azerbaijan |
| 2005 | 591.22670735395 | 0.602485193033144 | 13.2538454753183 | 577.972861878632 | Azerbaijan |
| 2006 | 535.73454605801 | 0.61377109654975 | 13.1572516743425 | 522.577294383668 | Azerbaijan |
| 2007 | 483.37083624931 | 0.626110925531736 | 12.9227437696019 | 470.448092479708 | Azerbaijan |
| 2008 | 444.2960197051 | 0.636901109120982 | 8.15347093277571 | 436.142548772324 | Azerbaijan |
| 2009 | 410.96067252987 | 0.645163340636595 | 6.11836001935787 | 404.842312510512 | Azerbaijan |
| 2010 | 385.95560082828 | 0.652056507002412 | 4.999735612195 | 380.955865216085 | Azerbaijan |
| 2011 | 362.83949245424 | 0.657971629199692 | 4.85645621901324 | 357.983036235227 | Azerbaijan |
| 2012 | 341.86230295587 | 0.662897001762113 | 4.35192531550219 | 337.510377640368 | Azerbaijan |
| 2013 | 322.69727478628 | 0.667658779253801 | 3.62076671572042 | 319.07650807056 | Azerbaijan |
| 2014 | 303.31331604807 | 0.672162741185153 | 3.30869971319556 | 300.004616334874 | Azerbaijan |
| 2015 | 288.9825534952 | 0.676244911134277 | 3.13694466815517 | 285.845608827045 | Azerbaijan |
| 2016 | 278.76276129659 | 0.679807642651878 | 3.03959999985057 | 275.723161296739 | Azerbaijan |
| 2017 | 270.51406620864 | 0.683263203610851 | 2.81763228175885 | 267.696433926881 | Azerbaijan |
| 2018 | 263.00679409355 | 0.68656109318329 | 2.94347710806141 | 260.063316985489 | Azerbaijan |
| 2019 | 256.52193299697 | 0.689834714583805 | 2.42392583027209 | 254.098007166698 | Azerbaijan |
| 2020 | 232.45909179203 | 0.692605192199032 | 2.44128484179012 | 230.01780695024 | Azerbaijan |
| 2021 | 227.60429300419 | 0.694851274415291 | 2.41601725308082 | 225.188275751109 | Azerbaijan |
| 1990 | 414.54126676837 | 0.656136044291497 | 5.087152435964 | 409.454114332406 | Georgia |
| 1991 | 350.39227659685 | 0.662113185865682 | 4.56118532409915 | 345.831091272751 | Georgia |
| 1992 | 349.28777557364 | 0.665021427814483 | 4.42214699718244 | 344.865628576458 | Georgia |
| 1993 | 407.54872853801 | 0.663466862184598 | 4.35722098625524 | 403.191507551755 | Georgia |
| 1994 | 470.86365362973 | 0.658134139130798 | 4.88647413448965 | 465.97717949524 | Georgia |
| 1995 | 430.61026917901 | 0.651363604039082 | 5.7474842261446 | 424.862784952865 | Georgia |
| 1996 | 372.84617305427 | 0.644558090070583 | 5.44599896681529 | 367.400174087455 | Georgia |
| 1997 | 363.43112985537 | 0.63852563128688 | 8.54844316577026 | 354.8826866896 | Georgia |
| 1998 | 298.92438024311 | 0.633347931127712 | 11.165117453432 | 287.759262789678 | Georgia |
| 1999 | 239.95334262968 | 0.63027794625322 | 11.9793585102827 | 227.973984119397 | Georgia |
| 2000 | 171.18483026021 | 0.630265990578475 | 11.9435283112096 | 159.241301949 | Georgia |
| 2001 | 152.70080964551 | 0.633388300444106 | 11.5181112724624 | 141.182698373048 | Georgia |
| 2002 | 135.82928687655 | 0.638370108205142 | 8.2623691845245 | 127.566917692025 | Georgia |
| 2003 | 117.29678815603 | 0.644065578985868 | 7.80073943225457 | 109.496048723775 | Georgia |
| 2004 | 104.4821880081 | 0.648920978029192 | 5.99233606444211 | 98.4898519436579 | Georgia |
| 2005 | 75.2252690043 | 0.65361600499668 | 5.17148978755472 | 70.0537792167453 | Georgia |
| 2006 | 57.10568898189 | 0.658403371769116 | 4.779814919872 | 52.325874062018 | Georgia |
| 2007 | 55.00628717868 | 0.663697966220568 | 4.45682470726462 | 50.5494624714154 | Georgia |
| 2008 | 51.87587972519 | 0.668442495947816 | 3.75014592241212 | 48.1257338027779 | Georgia |
| 2009 | 46.9439869467 | 0.672449380923053 | 3.22667026410967 | 43.7173166825903 | Georgia |
| 2010 | 42.66422044292 | 0.677034892187658 | 2.86100574867621 | 39.8032146942438 | Georgia |
| 2011 | 36.8866036928 | 0.681567044251293 | 2.74293043814833 | 34.1436732546517 | Georgia |
| 2012 | 33.77138291438 | 0.685519223189507 | 2.89568995624309 | 30.8756929581369 | Georgia |
| 2013 | 31.3596469208 | 0.688603268140652 | 2.50252815255426 | 28.8571187682457 | Georgia |
| 2014 | 25.74493379004 | 0.692155206871812 | 2.61244034591943 | 23.1324934441206 | Georgia |
| 2015 | 23.25187055398 | 0.697316132597582 | 2.48972646933661 | 20.7621440846434 | Georgia |
| 2016 | 23.12342556498 | 0.703731490142933 | 2.28200521891316 | 20.8414203460668 | Georgia |
| 2017 | 17.27749495943 | 0.710874718456081 | 2.27114426880976 | 15.0063506906202 | Georgia |
| 2018 | 15.93257719042 | 0.717993653891294 | 2.17891228592923 | 13.7536649044908 | Georgia |
| 2019 | 13.05309098387 | 0.724536461948472 | 2.1524159146345 | 10.9006750692355 | Georgia |
| 2020 | 12.11259359996 | 0.729125888954526 | 2.18185432692407 | 9.93073927303593 | Georgia |
| 2021 | 11.59822468364 | 0.732473604476306 | 2.19164592952071 | 9.40657875411929 | Georgia |
| 1990 | 859.06875143671 | 0.589435803839118 | 13.4335218447546 | 845.635229591955 | Kazakhstan |
| 1991 | 833.20325440471 | 0.590967831817803 | 13.6256070244723 | 819.577647380238 | Kazakhstan |
| 1992 | 932.76479721181 | 0.596127664006257 | 13.2784569026104 | 919.4863403092 | Kazakhstan |
| 1993 | 935.19131294362 | 0.603138328452166 | 13.4474774959717 | 921.743835447648 | Kazakhstan |
| 1994 | 952.01531185678 | 0.610591100120018 | 13.2122943896853 | 938.803017467095 | Kazakhstan |
| 1995 | 926.24382959203 | 0.618573582182698 | 13.090411653599 | 913.153417938431 | Kazakhstan |
| 1996 | 839.59565513485 | 0.626013137564744 | 13.0169732402507 | 826.578681894599 | Kazakhstan |
| 1997 | 849.87384660933 | 0.631166626279517 | 11.8714079299446 | 838.002438679385 | Kazakhstan |
| 1998 | 644.90963918572 | 0.635388623908262 | 10.97397488098 | 633.93566430474 | Kazakhstan |
| 1999 | 539.45677425485 | 0.64018048502742 | 7.93473871254176 | 531.522035542308 | Kazakhstan |
| 2000 | 443.06065973328 | 0.644689623243828 | 6.05551421480124 | 437.005145518479 | Kazakhstan |
| 2001 | 351.15760604149 | 0.648397258264821 | 5.48072202026974 | 345.67688402122 | Kazakhstan |
| 2002 | 267.49658064829 | 0.651529335252827 | 5.41032630810471 | 262.086254340185 | Kazakhstan |
| 2003 | 210.31293047097 | 0.654835925835483 | 4.84650023727013 | 205.4664302337 | Kazakhstan |
| 2004 | 188.83423065504 | 0.658733071329599 | 4.611003597765 | 184.223227057275 | Kazakhstan |
| 2005 | 158.80066216313 | 0.662943941893493 | 4.65003446953097 | 154.150627693599 | Kazakhstan |
| 2006 | 157.53293782602 | 0.667048858291012 | 3.81128303554313 | 153.721654790477 | Kazakhstan |
| 2007 | 146.95109211391 | 0.671367207568052 | 3.32519567573381 | 143.625896438176 | Kazakhstan |
| 2008 | 148.33862003464 | 0.676352868998898 | 2.97005044727964 | 145.36856958736 | Kazakhstan |
| 2009 | 113.53138547254 | 0.681606838672477 | 2.76063434482898 | 110.770751127711 | Kazakhstan |
| 2010 | 88.30348996209 | 0.686996643005111 | 2.7550261821962 | 85.5484637798938 | Kazakhstan |
| 2011 | 69.36196379338 | 0.691758951738966 | 2.64904337491543 | 66.7129204184646 | Kazakhstan |
| 2012 | 48.79323492126 | 0.695312766608011 | 2.48683536793045 | 46.3063995533295 | Kazakhstan |
| 2013 | 37.77975933653 | 0.699053593413088 | 2.39566091647 | 35.38409842006 | Kazakhstan |
| 2014 | 29.13138171772 | 0.703511920757132 | 2.29974615808897 | 26.831635559631 | Kazakhstan |
| 2015 | 30.50111974567 | 0.70822973614897 | 2.24360242102729 | 28.2575173246427 | Kazakhstan |
| 2016 | 26.16534991977 | 0.712569040397695 | 2.23218261615896 | 23.933167303611 | Kazakhstan |
| 2017 | 21.79813704627 | 0.716026687894304 | 2.22016073090369 | 19.5779763153663 | Kazakhstan |
| 2018 | 20.55920663235 | 0.718531998551374 | 2.20126327092762 | 18.3579433614224 | Kazakhstan |
| 2019 | 20.36089968033 | 0.720797601617411 | 2.18407336909741 | 18.1768263112326 | Kazakhstan |
| 2020 | 18.47609173513 | 0.722788768207953 | 2.18118973041038 | 16.2949020047196 | Kazakhstan |
| 2021 | 17.39125343681 | 0.725144495100349 | 2.16825104746141 | 15.2230023893486 | Kazakhstan |
| 1990 | 1154.94857990073 | 0.519407652158871 | 22.2730847010944 | 1132.67549519964 | Kyrgyzstan |
| 1991 | 1123.99833723245 | 0.523746714213702 | 22.168097405021 | 1101.83023982743 | Kyrgyzstan |
| 1992 | 1153.32151228819 | 0.52790155215442 | 22.1011290226011 | 1131.22038326559 | Kyrgyzstan |
| 1993 | 1053.07326283672 | 0.532431008334573 | 22.1504038469191 | 1030.9228589898 | Kyrgyzstan |
| 1994 | 1047.12860679313 | 0.536232980141406 | 21.3730290999271 | 1025.7555776932 | Kyrgyzstan |
| 1995 | 1011.89089535455 | 0.539052820319823 | 21.3091343054712 | 990.581761049079 | Kyrgyzstan |
| 1996 | 937.69127511271 | 0.541628927165702 | 21.132107745127 | 916.559167367583 | Kyrgyzstan |
| 1997 | 1065.13688619297 | 0.543341150499015 | 21.219351464195 | 1043.91753472878 | Kyrgyzstan |
| 1998 | 1057.61422126669 | 0.543258545983634 | 21.1866484708174 | 1036.42757279587 | Kyrgyzstan |
| 1999 | 1010.96017824059 | 0.54286728228234 | 21.1640542837769 | 989.796123956813 | Kyrgyzstan |
| 2000 | 849.92169071265 | 0.543036486604175 | 21.1361379990808 | 828.785552713569 | Kyrgyzstan |
| 2001 | 744.21068213497 | 0.543805550685403 | 21.1637407141834 | 723.046941420787 | Kyrgyzstan |
| 2002 | 626.54955496242 | 0.544374670344746 | 21.2035003571688 | 605.346054605251 | Kyrgyzstan |
| 2003 | 502.96102358828 | 0.545649261714426 | 20.4355295891608 | 482.525493999119 | Kyrgyzstan |
| 2004 | 453.08450227791 | 0.547262342515335 | 20.4069535844364 | 432.677548693474 | Kyrgyzstan |
| 2005 | 440.64787294514 | 0.548260448903511 | 20.496262084476 | 420.151610860664 | Kyrgyzstan |
| 2006 | 446.48877033623 | 0.549383593833376 | 20.4328977030129 | 426.055872633217 | Kyrgyzstan |
| 2007 | 425.5232206906 | 0.551146191596044 | 19.9040262697813 | 405.619194420819 | Kyrgyzstan |
| 2008 | 381.39030088483 | 0.553206382142723 | 19.7424774812081 | 361.647823403622 | Kyrgyzstan |
| 2009 | 311.06570431418 | 0.55512386607295 | 18.5136959358682 | 292.552008378312 | Kyrgyzstan |
| 2010 | 258.99894748623 | 0.556416544522946 | 18.650857323788 | 240.348090162442 | Kyrgyzstan |
| 2011 | 246.78473477094 | 0.55892333472436 | 17.7208777078522 | 229.063857063088 | Kyrgyzstan |
| 2012 | 209.6983557869 | 0.561541573671693 | 16.5528923576208 | 193.145463429279 | Kyrgyzstan |
| 2013 | 170.45434920815 | 0.565776769347093 | 15.1935729017191 | 155.260776306431 | Kyrgyzstan |
| 2014 | 163.36543332055 | 0.570569357153487 | 13.3956590686003 | 149.96977425195 | Kyrgyzstan |
| 2015 | 156.15782609856 | 0.575463230818727 | 13.5895404370437 | 142.568285661516 | Kyrgyzstan |
| 2016 | 120.20222734061 | 0.580388550869674 | 13.4540673257231 | 106.748160014887 | Kyrgyzstan |
| 2017 | 93.20312413334 | 0.585766095993466 | 13.3945287228898 | 79.8085954104502 | Kyrgyzstan |
| 2018 | 83.36365667467 | 0.591237134219733 | 13.3934648588407 | 69.9701918158293 | Kyrgyzstan |
| 2019 | 81.49779865468 | 0.596501638087194 | 13.3179291627786 | 68.1798694919014 | Kyrgyzstan |
| 2020 | 74.84012397834 | 0.600499553804348 | 13.3453199354087 | 61.4948040429313 | Kyrgyzstan |
| 2021 | 73.71343043101 | 0.603979328412055 | 13.4285992481781 | 60.2848311828319 | Kyrgyzstan |
| 1990 | 612.02867308757 | 0.466550117857047 | 55.4954973435908 | 556.533175743979 | Mongolia |
| 1991 | 564.41609904084 | 0.472199013666192 | 58.1784272950571 | 506.237671745783 | Mongolia |
| 1992 | 524.81926759131 | 0.477586553799195 | 54.8574865883088 | 469.961781003001 | Mongolia |
| 1993 | 493.30873842995 | 0.482726727049412 | 56.0228470718363 | 437.285891358114 | Mongolia |
| 1994 | 463.21490410987 | 0.488276683033118 | 49.8766527471085 | 413.338251362762 | Mongolia |
| 1995 | 431.15404708243 | 0.495006781085721 | 51.1453584750472 | 380.008688607383 | Mongolia |
| 1996 | 427.86863787822 | 0.501703717883367 | 23.5166826567594 | 404.351955221461 | Mongolia |
| 1997 | 381.99100212876 | 0.50855826800798 | 22.6920697105497 | 359.29893241821 | Mongolia |
| 1998 | 358.18257328328 | 0.514793382702856 | 22.4943166969026 | 335.688256586377 | Mongolia |
| 1999 | 337.55165042773 | 0.520781189245005 | 22.1386207119938 | 315.413029715736 | Mongolia |
| 2000 | 319.18685670999 | 0.526650344907534 | 22.1703210377644 | 297.016535672226 | Mongolia |
| 2001 | 283.06553051998 | 0.532620200843235 | 22.1046989058056 | 260.960831614174 | Mongolia |
| 2002 | 258.90027107121 | 0.538424834911619 | 21.3078572761004 | 237.59241379511 | Mongolia |
| 2003 | 241.74008519423 | 0.543971650708528 | 21.1970862186557 | 220.542998975574 | Mongolia |
| 2004 | 226.1346895891 | 0.549551499752672 | 19.657159289642 | 206.477530299458 | Mongolia |
| 2005 | 212.2846594642 | 0.55468028439734 | 18.6568963618878 | 193.627763102312 | Mongolia |
| 2006 | 203.88612160995 | 0.55956113814646 | 17.658941273611 | 186.227180336339 | Mongolia |
| 2007 | 197.23009642771 | 0.564236795141046 | 16.0124464451321 | 181.217649982578 | Mongolia |
| 2008 | 189.78374121453 | 0.568500598715617 | 14.0782346149229 | 175.705506599607 | Mongolia |
| 2009 | 172.64678955406 | 0.571341685964544 | 13.2216473910641 | 159.425142162996 | Mongolia |
| 2010 | 154.9222280224 | 0.574406669398145 | 13.1950747288447 | 141.727153293555 | Mongolia |
| 2011 | 140.78462248005 | 0.57860066312097 | 13.4905483157865 | 127.294074164263 | Mongolia |
| 2012 | 131.97656220391 | 0.583255885768315 | 13.5573431444313 | 118.419219059479 | Mongolia |
| 2013 | 117.88035724227 | 0.588080810956893 | 13.531520577659 | 104.348836664611 | Mongolia |
| 2014 | 104.50230265829 | 0.592867352927119 | 13.5306175887235 | 90.9716850695665 | Mongolia |
| 2015 | 85.40192491357 | 0.59685964756958 | 13.2142038956818 | 72.1877210178882 | Mongolia |
| 2016 | 76.8483874142 | 0.600260766781331 | 13.40346745281 | 63.44491996139 | Mongolia |
| 2017 | 74.3314404382 | 0.60372196450642 | 13.3781736680196 | 60.9532667701804 | Mongolia |
| 2018 | 69.25640563802 | 0.607501833007913 | 13.0923286422449 | 56.1640769957751 | Mongolia |
| 2019 | 65.8253697723 | 0.611250680890372 | 13.2785323767319 | 52.546837395568 | Mongolia |
| 2020 | 55.51801055541 | 0.61462963730677 | 13.1240459662551 | 42.3939645891549 | Mongolia |
| 2021 | 57.68260510947 | 0.617621564638127 | 12.9778681814411 | 44.7047369280289 | Mongolia |
| 1990 | 5016.62462850739 | 0.466155413419203 | 58.9056195944856 | 4957.7190089129 | Tajikistan |
| 1991 | 4974.03234270686 | 0.472342173046228 | 58.2584372516318 | 4915.77390545523 | Tajikistan |
| 1992 | 5016.76210320227 | 0.47574519497737 | 58.2322604807341 | 4958.52984272154 | Tajikistan |
| 1993 | 5071.75987991529 | 0.478238812995371 | 53.6569257005176 | 5018.10295421477 | Tajikistan |
| 1994 | 5045.14447466498 | 0.478726011215724 | 53.4909161540027 | 4991.65355851098 | Tajikistan |
| 1995 | 5024.36007793799 | 0.477586868115015 | 53.8024190794787 | 4970.55765885851 | Tajikistan |
| 1996 | 4983.90341527564 | 0.474346027462684 | 55.4459740186299 | 4928.45744125701 | Tajikistan |
| 1997 | 4887.64701654435 | 0.470274795927363 | 56.6956003131653 | 4830.95141623118 | Tajikistan |
| 1998 | 4781.53297981321 | 0.465341742120163 | 65.4549272990189 | 4716.07805251419 | Tajikistan |
| 1999 | 4640.62797558597 | 0.46050561615396 | 63.56150407446 | 4577.06647151151 | Tajikistan |
| 2000 | 4465.65489651511 | 0.457061333395443 | 63.6879540733756 | 4401.96694244173 | Tajikistan |
| 2001 | 4209.88427266983 | 0.45625651276009 | 64.5819347712038 | 4145.30233789863 | Tajikistan |
| 2002 | 3910.20414338594 | 0.458807780304158 | 65.8897508708007 | 3844.31439251514 | Tajikistan |
| 2003 | 3634.07313989495 | 0.463329706383786 | 62.258782162614 | 3571.81435773234 | Tajikistan |
| 2004 | 3368.80039129964 | 0.469154214043517 | 56.4238152730482 | 3312.37657602659 | Tajikistan |
| 2005 | 3119.34537689533 | 0.474637785688857 | 58.2767004342217 | 3061.06867646111 | Tajikistan |
| 2006 | 2979.35753637799 | 0.479651831177988 | 53.7947229218269 | 2925.56281345616 | Tajikistan |
| 2007 | 2826.87573664688 | 0.484211156232784 | 53.5054761515473 | 2773.37026049533 | Tajikistan |
| 2008 | 2672.98452432712 | 0.488441135968382 | 49.3904927988276 | 2623.59403152829 | Tajikistan |
| 2009 | 2540.33542222177 | 0.49206404051479 | 50.738567870616 | 2489.59685435115 | Tajikistan |
| 2010 | 2384.88284359971 | 0.495893963626257 | 49.8680314764399 | 2335.01481212327 | Tajikistan |
| 2011 | 2259.67810335644 | 0.499869804401369 | 23.4388910566738 | 2236.23921229977 | Tajikistan |
| 2012 | 2153.76194236037 | 0.503953884549027 | 23.0762320782018 | 2130.68571028217 | Tajikistan |
| 2013 | 2065.93287313743 | 0.507970675899606 | 22.7014126112097 | 2043.23146052622 | Tajikistan |
| 2014 | 1979.45148437711 | 0.511989517777097 | 22.5248769178196 | 1956.92660745929 | Tajikistan |
| 2015 | 1697.89860190185 | 0.515783244686634 | 22.2664183739115 | 1675.63218352794 | Tajikistan |
| 2016 | 1615.84442585864 | 0.519897002395847 | 22.171690626404 | 1593.67273523224 | Tajikistan |
| 2017 | 1535.35367566986 | 0.524492606909067 | 22.1584206943551 | 1513.1952549755 | Tajikistan |
| 2018 | 1477.45605439719 | 0.529153916295379 | 22.1436556503993 | 1455.31239874679 | Tajikistan |
| 2019 | 1468.8725941846 | 0.533769951484441 | 22.1267372786247 | 1446.74585690598 | Tajikistan |
| 2020 | 1367.30540146305 | 0.537690530515565 | 21.2720419828425 | 1346.03335948021 | Tajikistan |
| 2021 | 1345.26003466457 | 0.541511187051998 | 21.1691880503416 | 1324.09084661423 | Tajikistan |
| 1990 | 2701.21857849492 | 0.563126887105135 | 16.622903905596 | 2684.59567458932 | Turkmenistan |
| 1991 | 2686.69029319858 | 0.564939385894165 | 16.358119581005 | 2670.33217361758 | Turkmenistan |
| 1992 | 2742.88133254995 | 0.565587505223265 | 15.3372411575334 | 2727.54409139242 | Turkmenistan |
| 1993 | 2801.74548221236 | 0.567174449333284 | 14.4286394493204 | 2787.31684276304 | Turkmenistan |
| 1994 | 2739.32500228101 | 0.567288975507041 | 14.5171247273953 | 2724.80787755361 | Turkmenistan |
| 1995 | 2708.30112731474 | 0.567198634711689 | 14.6952429483759 | 2693.60588436636 | Turkmenistan |
| 1996 | 2507.44062283655 | 0.567061261285221 | 14.6689064545955 | 2492.77171638195 | Turkmenistan |
| 1997 | 2230.93573747817 | 0.565623083668948 | 15.2742700499801 | 2215.66146742819 | Turkmenistan |
| 1998 | 1929.40699760268 | 0.564405264391893 | 16.165989971045 | 1913.24100763163 | Turkmenistan |
| 1999 | 1688.01197628122 | 0.564716005636632 | 16.0133195257492 | 1671.99865675547 | Turkmenistan |
| 2000 | 1439.99154643697 | 0.567421033351252 | 14.6110366272659 | 1425.3805098097 | Turkmenistan |
| 2001 | 1210.02770849853 | 0.57104381892434 | 13.3305611972622 | 1196.69714730127 | Turkmenistan |
| 2002 | 978.64075293965 | 0.575596165867541 | 13.2899492406165 | 965.350803699033 | Turkmenistan |
| 2003 | 770.65417519787 | 0.580820125927832 | 13.5459795234255 | 757.108195674445 | Turkmenistan |
| 2004 | 624.58621464038 | 0.586712349142411 | 13.604043859185 | 610.982170781195 | Turkmenistan |
| 2005 | 596.20479662117 | 0.593388894864117 | 13.319998516197 | 582.884798104973 | Turkmenistan |
| 2006 | 520.92774087106 | 0.599896069365258 | 13.2430391542887 | 507.684701716771 | Turkmenistan |
| 2007 | 462.201656449 | 0.606042198134731 | 13.1946756454552 | 449.006980803545 | Turkmenistan |
| 2008 | 450.08582108462 | 0.611923308807263 | 13.1733220518424 | 436.912499032778 | Turkmenistan |
| 2009 | 409.96284042949 | 0.617652905277457 | 13.0382170266677 | 396.924623402822 | Turkmenistan |
| 2010 | 343.21893954789 | 0.623234588644236 | 12.865958697125 | 330.352980850765 | Turkmenistan |
| 2011 | 275.07739243418 | 0.629233721702303 | 12.6590543389648 | 262.418338095215 | Turkmenistan |
| 2012 | 176.81970490769 | 0.635263183091614 | 10.8787953130639 | 165.940909594626 | Turkmenistan |
| 2013 | 156.17653521259 | 0.641361893351002 | 8.24089044390318 | 147.935644768687 | Turkmenistan |
| 2014 | 112.55645513847 | 0.647542416932676 | 5.68860180030693 | 106.867853338163 | Turkmenistan |
| 2015 | 114.62992838334 | 0.65335278951701 | 4.86273598494196 | 109.767192398398 | Turkmenistan |
| 2016 | 110.77415899031 | 0.658945613392641 | 4.74046043418932 | 106.033698556121 | Turkmenistan |
| 2017 | 109.17316848392 | 0.664284691942071 | 4.42316651061908 | 104.750001973301 | Turkmenistan |
| 2018 | 98.23056683593 | 0.669433598508271 | 3.56760197875528 | 94.6629648571747 | Turkmenistan |
| 2019 | 91.89402408894 | 0.674303535870544 | 2.99207666850731 | 88.9019474204327 | Turkmenistan |
| 2020 | 85.76830120516 | 0.678298179976148 | 2.8331823357616 | 82.9351188693984 | Turkmenistan |
| 2021 | 82.2549325749 | 0.682160775869611 | 2.92447996689607 | 79.3304526080039 | Turkmenistan |
| 1990 | 867.10582198336 | 0.500241735138187 | 23.3525369700388 | 843.753285013321 | Uzbekistan |
| 1991 | 867.7052749823 | 0.501544609458551 | 23.3408984329115 | 844.364376549389 | Uzbekistan |
| 1992 | 903.84108793124 | 0.502905194308151 | 23.1818945364642 | 880.659193394776 | Uzbekistan |
| 1993 | 884.55215420583 | 0.506556615160302 | 22.7564440301394 | 861.795710175691 | Uzbekistan |
| 1994 | 874.56401271068 | 0.5110185249116 | 22.4648555075386 | 852.099157203141 | Uzbekistan |
| 1995 | 768.77904747023 | 0.51698626355212 | 22.3088595868645 | 746.470187883365 | Uzbekistan |
| 1996 | 623.51403349614 | 0.524392909803147 | 22.1701914951376 | 601.343842001002 | Uzbekistan |
| 1997 | 490.65014369335 | 0.532686328470226 | 22.1834383785483 | 468.466705314802 | Uzbekistan |
| 1998 | 345.21094884546 | 0.541279069137397 | 21.257006003808 | 323.953942841652 | Uzbekistan |
| 1999 | 274.58139564903 | 0.549739390933737 | 19.7476951750648 | 254.833700473965 | Uzbekistan |
| 2000 | 206.88577648904 | 0.557522310762499 | 17.7605106517565 | 189.125265837284 | Uzbekistan |
| 2001 | 190.55052548406 | 0.564803382897778 | 16.2250901495386 | 174.325435334521 | Uzbekistan |
| 2002 | 177.0510517125 | 0.571690142599581 | 13.3242973158779 | 163.726754396622 | Uzbekistan |
| 2003 | 165.45857621063 | 0.57800653838209 | 13.4209010038338 | 152.037675206796 | Uzbekistan |
| 2004 | 152.05419373645 | 0.583942986424626 | 13.2993231121841 | 138.754870624266 | Uzbekistan |
| 2005 | 117.48085165203 | 0.589530776570668 | 13.5632032220505 | 103.91764842998 | Uzbekistan |
| 2006 | 69.00672034169 | 0.594498056671551 | 13.3139092888556 | 55.6928110528344 | Uzbekistan |
| 2007 | 58.00536990967 | 0.599628054727829 | 13.3606604538623 | 44.6447094558077 | Uzbekistan |
| 2008 | 55.3620842613 | 0.604889158157396 | 13.2442825868167 | 42.1178016744833 | Uzbekistan |
| 2009 | 53.07521332648 | 0.610351745244056 | 13.2667204713764 | 39.8084928551036 | Uzbekistan |
| 2010 | 50.80747819835 | 0.616097907401681 | 12.9229236943133 | 37.8845545040367 | Uzbekistan |
| 2011 | 47.48299671203 | 0.621344618930939 | 12.9245347429017 | 34.5584619691283 | Uzbekistan |
| 2012 | 39.55356667307 | 0.62620212543294 | 12.9764657871363 | 26.5771008859337 | Uzbekistan |
| 2013 | 29.97960430808 | 0.630796403406203 | 12.0593915349896 | 17.9202127730904 | Uzbekistan |
| 2014 | 29.43445418875 | 0.635257049718365 | 11.3458624716486 | 18.0885917171014 | Uzbekistan |
| 2015 | 31.44744949968 | 0.639805638905225 | 8.34075318314776 | 23.1066963165322 | Uzbekistan |
| 2016 | 28.99185079554 | 0.644230597300764 | 6.32588496092581 | 22.6659658346142 | Uzbekistan |
| 2017 | 17.18996715249 | 0.648119346591586 | 5.70015122192727 | 11.4898159305627 | Uzbekistan |
| 2018 | 19.67815963891 | 0.651918144182724 | 5.40174015710403 | 14.276419481806 | Uzbekistan |
| 2019 | 17.87128240528 | 0.655836959268026 | 5.32271261294785 | 12.5485697923322 | Uzbekistan |
| 2020 | 16.54141826911 | 0.659124204371032 | 4.45972436033547 | 12.0816939087745 | Uzbekistan |
| 2021 | 15.94941847581 | 0.662621694181816 | 4.78445702246792 | 11.1649614533421 | Uzbekistan |
| 1990 | 109.48532223769 | 0.557773300317074 | 17.6024464782813 | 91.8828757594087 | Albania |
| 1991 | 113.62885170713 | 0.555629411790174 | 18.6056863447356 | 95.0231653623944 | Albania |
| 1992 | 111.26025805878 | 0.553107897492912 | 19.9236542521847 | 91.3366038065953 | Albania |
| 1993 | 107.02742226537 | 0.552453327893755 | 19.765425042133 | 87.261997223237 | Albania |
| 1994 | 123.82274064558 | 0.553763669315605 | 18.8093097661419 | 105.013430879438 | Albania |
| 1995 | 91.7464151651 | 0.557040889185472 | 18.8810303505336 | 72.8653848145664 | Albania |
| 1996 | 83.14720539704 | 0.562248886206679 | 16.657666523288 | 66.489538873752 | Albania |
| 1997 | 77.68788996325 | 0.566802804232969 | 14.5781623712897 | 63.1097275919603 | Albania |
| 1998 | 71.12760378281 | 0.572534101516668 | 13.3405860149784 | 57.7870177678316 | Albania |
| 1999 | 60.42916425287 | 0.578295461805887 | 13.2315505836732 | 47.1976136691969 | Albania |
| 2000 | 52.64317155711 | 0.58466418835901 | 13.3691439029724 | 39.2740276541376 | Albania |
| 2001 | 47.19299960045 | 0.592412946510661 | 13.4107968218888 | 33.7822027785612 | Albania |
| 2002 | 42.48307480206 | 0.599865977568749 | 13.4419125381478 | 29.0411622639122 | Albania |
| 2003 | 38.34340426453 | 0.606971165987994 | 13.3308976271835 | 25.0125066373465 | Albania |
| 2004 | 34.58078097083 | 0.613864246664272 | 13.4178745366548 | 21.1629064341752 | Albania |
| 2005 | 31.86932220534 | 0.620679614590226 | 12.9706076390938 | 18.8987145662462 | Albania |
| 2006 | 30.30145725774 | 0.627485617497143 | 12.5241256612418 | 17.7773315964982 | Albania |
| 2007 | 29.04660893891 | 0.634167968985356 | 11.0980190030656 | 17.9485899358444 | Albania |
| 2008 | 28.22704203533 | 0.640766134349698 | 7.65143222333439 | 20.5756098119956 | Albania |
| 2009 | 27.55135769622 | 0.646816130123751 | 5.88909124988072 | 21.6622664463393 | Albania |
| 2010 | 27.21156202894 | 0.652654202642964 | 4.82338818393929 | 22.3881738450007 | Albania |
| 2011 | 27.61808406716 | 0.658196772707548 | 4.75520675535425 | 22.8628773118058 | Albania |
| 2012 | 27.48602274918 | 0.66355677961127 | 4.42202289300081 | 23.0639998561792 | Albania |
| 2013 | 26.99944421244 | 0.668739558985928 | 3.74264703363776 | 23.2567971788022 | Albania |
| 2014 | 26.23273359901 | 0.67411298539372 | 3.42023628411684 | 22.8124973148932 | Albania |
| 2015 | 25.66055377997 | 0.679668600655757 | 2.97038350244464 | 22.6901702775254 | Albania |
| 2016 | 25.20843349516 | 0.68503664759447 | 2.72630873444169 | 22.4821247607183 | Albania |
| 2017 | 24.37287059153 | 0.690379199197076 | 2.51131741343433 | 21.8615531780957 | Albania |
| 2018 | 23.57944504267 | 0.695566877664306 | 2.44991491815429 | 21.1295301245157 | Albania |
| 2019 | 21.7132511138 | 0.700287863295906 | 2.31272945478143 | 19.4005216590186 | Albania |
| 2020 | 20.51665192074 | 0.703790245372837 | 2.31694363636493 | 18.1997082843751 | Albania |
| 2021 | 19.11855060034 | 0.706849790878611 | 2.28144223207217 | 16.8371083682678 | Albania |
| 1990 | 22.08670888411 | 0.541132539726015 | 21.1793832069069 | 0.907325677203051 | Bosnia and Herzegovina |
| 1991 | 21.18002527553 | 0.541346250550883 | 21.0775218254355 | 0.10250345009451 | Bosnia and Herzegovina |
| 1992 | 21.16530558061 | 0.539303227421668 | 21.0850803983397 | 0.0802251822703255 | Bosnia and Herzegovina |
| 1993 | 20.98405609474 | 0.53603188160081 | 20.98405609474 | 0 | Bosnia and Herzegovina |
| 1994 | 21.31384215686 | 0.533853389744284 | 21.31384215686 | 0 | Bosnia and Herzegovina |
| 1995 | 21.44419330779 | 0.534025536110865 | 21.373675472041 | 0.0705178357490155 | Bosnia and Herzegovina |
| 1996 | 19.89764938335 | 0.545549215040903 | 19.89764938335 | 0 | Bosnia and Herzegovina |
| 1997 | 18.86701976401 | 0.563565414584231 | 16.027327547171 | 2.83969221683903 | Bosnia and Herzegovina |
| 1998 | 18.00068385481 | 0.58182951538312 | 13.5125995490263 | 4.48808430578368 | Bosnia and Herzegovina |
| 1999 | 15.9273961815 | 0.597997468748356 | 13.4119589800459 | 2.51543720145406 | Bosnia and Herzegovina |
| 2000 | 15.00314678806 | 0.610950684608214 | 13.3623677808429 | 1.64077900721714 | Bosnia and Herzegovina |
| 2001 | 14.00360352459 | 0.621989463929258 | 12.9826621639431 | 1.02094136064687 | Bosnia and Herzegovina |
| 2002 | 15.71030069905 | 0.632075517723657 | 12.0154881518111 | 3.69481254723893 | Bosnia and Herzegovina |
| 2003 | 16.34829655788 | 0.640635102031521 | 8.6982952703077 | 7.6500012875723 | Bosnia and Herzegovina |
| 2004 | 16.42758589452 | 0.64779373168743 | 6.3401477680963 | 10.0874381264237 | Bosnia and Herzegovina |
| 2005 | 16.34131689847 | 0.653859136710253 | 4.96032361177923 | 11.3809932866908 | Bosnia and Herzegovina |
| 2006 | 16.77334785385 | 0.659813662252595 | 4.64292271427369 | 12.1304251395763 | Bosnia and Herzegovina |
| 2007 | 16.9149748048 | 0.665578735044097 | 4.2771772236823 | 12.6377975811177 | Bosnia and Herzegovina |
| 2008 | 16.68022636175 | 0.671100813673566 | 3.68574752156963 | 12.9944788401804 | Bosnia and Herzegovina |
| 2009 | 16.31543908748 | 0.676032900022704 | 3.16899595639 | 13.14644313109 | Bosnia and Herzegovina |
| 2010 | 16.09884410364 | 0.680824195636093 | 3.09440323390576 | 13.0044408697342 | Bosnia and Herzegovina |
| 2011 | 15.14050602101 | 0.685399264182467 | 2.65474092904828 | 12.4857650919617 | Bosnia and Herzegovina |
| 2012 | 15.00078922767 | 0.689506566189932 | 2.49610816209923 | 12.5046810655708 | Bosnia and Herzegovina |
| 2013 | 15.29779316916 | 0.693508711211908 | 2.38017892350945 | 12.9176142456505 | Bosnia and Herzegovina |
| 2014 | 14.30090146356 | 0.697031893012906 | 2.46484188835096 | 11.836059575209 | Bosnia and Herzegovina |
| 2015 | 14.91701452756 | 0.700446047366461 | 2.32504735486977 | 12.5919671726902 | Bosnia and Herzegovina |
| 2016 | 14.82998844161 | 0.704079343718599 | 2.270027507128 | 12.559960934482 | Bosnia and Herzegovina |
| 2017 | 14.428883431 | 0.70805343112809 | 2.25487852601103 | 12.174004904989 | Bosnia and Herzegovina |
| 2018 | 13.7464815179 | 0.712347302038319 | 2.2416917180163 | 11.5047897998837 | Bosnia and Herzegovina |
| 2019 | 13.3988606755 | 0.716857124768862 | 2.22794021928964 | 11.1709204562104 | Bosnia and Herzegovina |
| 2020 | 12.09115852961 | 0.720202056378697 | 2.21267598636 | 9.87848254325 | Bosnia and Herzegovina |
| 2021 | 11.21713858243 | 0.72307789321024 | 2.19652717944833 | 9.02061140298167 | Bosnia and Herzegovina |
| 1990 | 19.84064442961 | 0.633446498017686 | 11.002677153631 | 8.83796727597898 | Bulgaria |
| 1991 | 23.07773656908 | 0.643069487769455 | 7.82346866233156 | 15.2542679067484 | Bulgaria |
| 1992 | 23.58597548096 | 0.651186876165789 | 5.5650780221537 | 18.0208974588063 | Bulgaria |
| 1993 | 21.08540361939 | 0.659161569264411 | 4.55784476302258 | 16.5275588563674 | Bulgaria |
| 1994 | 24.78350814721 | 0.667430460276086 | 3.76810044810127 | 21.0154076991087 | Bulgaria |
| 1995 | 19.91391601102 | 0.673999976701174 | 3.40725692783047 | 16.5066590831895 | Bulgaria |
| 1996 | 19.06473740308 | 0.678862583541026 | 2.7892499215219 | 16.2754874815581 | Bulgaria |
| 1997 | 21.56964375341 | 0.680221466342146 | 3.02269695661952 | 18.5469467967905 | Bulgaria |
| 1998 | 18.17814175402 | 0.678496876732148 | 2.82855391902938 | 15.3495878349906 | Bulgaria |
| 1999 | 15.35659974354 | 0.677446543282232 | 2.89128435022183 | 12.4653153933182 | Bulgaria |
| 2000 | 16.63341255705 | 0.681004523812614 | 2.8051519415098 | 13.8282606155402 | Bulgaria |
| 2001 | 14.44628840166 | 0.686487366008627 | 2.61192562063875 | 11.8343627810212 | Bulgaria |
| 2002 | 14.6865781137 | 0.69151167375307 | 2.49731382331566 | 12.1892642903843 | Bulgaria |
| 2003 | 13.05136127404 | 0.69556596473307 | 2.42746100556212 | 10.6239002684779 | Bulgaria |
| 2004 | 12.1392449034 | 0.699881950859544 | 2.35074719987667 | 9.78849770352333 | Bulgaria |
| 2005 | 10.92415710519 | 0.704471206966935 | 2.28034316668732 | 8.64381393850268 | Bulgaria |
| 2006 | 10.35894450876 | 0.708868293577424 | 2.24164297614091 | 8.11730153261909 | Bulgaria |
| 2007 | 10.04500480365 | 0.713192402424104 | 2.25382273690327 | 7.79118206674673 | Bulgaria |
| 2008 | 11.21047312537 | 0.717672450499802 | 2.17971750412556 | 9.03075562124444 | Bulgaria |
| 2009 | 11.42107444482 | 0.723676626795129 | 2.14339269635324 | 9.27768174846677 | Bulgaria |
| 2010 | 13.32773963867 | 0.730701737095494 | 2.18714639835938 | 11.1405932403106 | Bulgaria |
| 2011 | 16.05676600872 | 0.735749281014389 | 2.15194690011333 | 13.9048191086067 | Bulgaria |
| 2012 | 19.18802795253 | 0.739177517343213 | 2.167256045388 | 17.020771907142 | Bulgaria |
| 2013 | 21.3985418176 | 0.742135948467762 | 2.10711552076414 | 19.2914262968359 | Bulgaria |
| 2014 | 23.94286656698 | 0.745372642577071 | 2.08759940511952 | 21.8552671618605 | Bulgaria |
| 2015 | 22.05724179018 | 0.748979058373083 | 2.05184466956783 | 20.0053971206122 | Bulgaria |
| 2016 | 20.75813507102 | 0.752259198563744 | 2.021931356 | 18.73620371502 | Bulgaria |
| 2017 | 21.85281066472 | 0.75566586464625 | 1.98714986076909 | 19.8656608039509 | Bulgaria |
| 2018 | 21.05061708385 | 0.759148460251881 | 1.90950184786403 | 19.141115235986 | Bulgaria |
| 2019 | 19.74494490965 | 0.762359790844767 | 1.73963688865246 | 18.0053080209975 | Bulgaria |
| 2020 | 17.77779087986 | 0.765089710642011 | 1.77087658809131 | 16.0069142917687 | Bulgaria |
| 2021 | 18.02913094616 | 0.768150939342518 | 1.62985147225409 | 16.3992794739059 | Bulgaria |
| 1990 | 9.67216052715 | 0.668906358291928 | 3.68402719291686 | 5.98813333423314 | Croatia |
| 1991 | 9.42062880914 | 0.674724599866063 | 3.24850897517182 | 6.17211983396818 | Croatia |
| 1992 | 9.87370684847 | 0.678093133084916 | 3.24663624562622 | 6.62707060284378 | Croatia |
| 1993 | 9.25626239951 | 0.678221047043161 | 2.79719010417054 | 6.45907229533946 | Croatia |
| 1994 | 9.22370537713 | 0.677548132655056 | 3.15606687665273 | 6.06763850047727 | Croatia |
| 1995 | 7.6478529212 | 0.677029465132868 | 2.89341829713673 | 4.75443462406327 | Croatia |
| 1996 | 7.14268681857 | 0.679507318367616 | 2.77267301639063 | 4.37001380217937 | Croatia |
| 1997 | 7.04753627798 | 0.685365010443468 | 2.92662776555514 | 4.12090851242487 | Croatia |
| 1998 | 6.15162056672 | 0.691365518598319 | 2.55493209188297 | 3.59668847483703 | Croatia |
| 1999 | 4.90709543201 | 0.696636411104102 | 2.37806774988739 | 2.52902768212261 | Croatia |
| 2000 | 4.90204848515 | 0.703039898563322 | 2.2431743862498 | 2.6588740989002 | Croatia |
| 2001 | 4.70093386738 | 0.709536840872917 | 2.21871848310483 | 2.48221538427517 | Croatia |
| 2002 | 4.44956451513 | 0.715383421635164 | 2.18088171810794 | 2.26868279702206 | Croatia |
| 2003 | 3.94727587087 | 0.721130377216859 | 2.24168963125816 | 1.70558623961184 | Croatia |
| 2004 | 3.78092626965 | 0.726327651623968 | 2.21372807674897 | 1.56719819290103 | Croatia |
| 2005 | 3.71061524082 | 0.732154139815163 | 2.17832932418654 | 1.53228591663346 | Croatia |
| 2006 | 3.56076123923 | 0.738066878515765 | 2.14546688107912 | 1.41529435815088 | Croatia |
| 2007 | 3.80863500024 | 0.743148776515466 | 2.12105430826714 | 1.68758069197286 | Croatia |
| 2008 | 4.16173345314 | 0.748469602229701 | 2.06307915090037 | 2.09865430223963 | Croatia |
| 2009 | 4.97300435244 | 0.754065605198048 | 2.02354002697778 | 2.94946432546222 | Croatia |
| 2010 | 5.98762417005 | 0.759472251706366 | 1.92087493425703 | 4.06674923579297 | Croatia |
| 2011 | 8.00402114071 | 0.763686381293279 | 1.6870879069431 | 6.3169332337669 | Croatia |
| 2012 | 9.44403391267 | 0.767405335599999 | 1.59501276123509 | 7.84902115143491 | Croatia |
| 2013 | 11.42311067603 | 0.771151880279813 | 1.62613247165818 | 9.79697820437182 | Croatia |
| 2014 | 12.70696179696 | 0.774479240521915 | 1.58693331418899 | 11.120028482771 | Croatia |
| 2015 | 14.10277626237 | 0.777865888163329 | 1.60884976322911 | 12.4939264991409 | Croatia |
| 2016 | 14.76462444733 | 0.781443605436965 | 1.46023747902596 | 13.304386968304 | Croatia |
| 2017 | 15.60630080006 | 0.785074536823775 | 1.45877857293638 | 14.1475222271236 | Croatia |
| 2018 | 15.36357146402 | 0.788671410129235 | 1.47452017875722 | 13.8890512852628 | Croatia |
| 2019 | 13.53020408967 | 0.792475415454183 | 1.49514264175763 | 12.0350614479124 | Croatia |
| 2020 | 12.57661320984 | 0.795462271729482 | 1.44879881183518 | 11.1278143980048 | Croatia |
| 2021 | 12.00291986625 | 0.798341026936276 | 1.44207256150037 | 10.5608473047496 | Croatia |
| 1990 | 23.28152762071 | 0.681848021213857 | 2.67719636022211 | 20.6043312604879 | Czechia |
| 1991 | 23.80515247276 | 0.688002859136679 | 2.46029345076635 | 21.3448590219936 | Czechia |
| 1992 | 23.73446130615 | 0.697577809122723 | 2.42031349098288 | 21.3141478151671 | Czechia |
| 1993 | 20.85424975596 | 0.710853415862456 | 2.26869975806915 | 18.5855499978908 | Czechia |
| 1994 | 19.58470590858 | 0.725969686167497 | 2.19433357968396 | 17.390372328896 | Czechia |
| 1995 | 15.36258916208 | 0.737981721993762 | 2.17799839225852 | 13.1845907698215 | Czechia |
| 1996 | 12.98665459142 | 0.746041379938535 | 2.04741104759962 | 10.9392435438204 | Czechia |
| 1997 | 13.41222573193 | 0.751680343695868 | 2.04707502337725 | 11.3651507085528 | Czechia |
| 1998 | 12.1816416711 | 0.757033755457614 | 1.89546199572864 | 10.2861796753714 | Czechia |
| 1999 | 10.0133377836 | 0.762428880357609 | 1.75034101971763 | 8.26299676388237 | Czechia |
| 2000 | 9.99787630764 | 0.767766159168669 | 1.70967309065676 | 8.28820321698324 | Czechia |
| 2001 | 9.41089485547 | 0.772167239650309 | 1.58805045280614 | 7.82284440266386 | Czechia |
| 2002 | 9.67490343063 | 0.776927934062507 | 1.50599390177196 | 8.16890952885804 | Czechia |
| 2003 | 8.84837325172 | 0.782448081111072 | 1.53158424341024 | 7.31678900830976 | Czechia |
| 2004 | 8.32445055181 | 0.787126404974676 | 1.49419700891514 | 6.83025354289486 | Czechia |
| 2005 | 7.59441055741 | 0.79133394820415 | 1.50264140600925 | 6.09176915140075 | Czechia |
| 2006 | 7.42929313813 | 0.794922783687463 | 1.45763305322541 | 5.9716600849046 | Czechia |
| 2007 | 7.67450107263 | 0.798254117717088 | 1.45145615480769 | 6.22304491782231 | Czechia |
| 2008 | 7.97313271965 | 0.8017648456328 | 1.46568391696756 | 6.50744880268244 | Czechia |
| 2009 | 9.49091405632 | 0.804962224676367 | 1.42474620235634 | 8.06616785396366 | Czechia |
| 2010 | 11.9725121204 | 0.808747327653098 | 1.44152533662551 | 10.5309867837745 | Czechia |
| 2011 | 14.97435738646 | 0.812054784320429 | 1.44763379547288 | 13.5267235909871 | Czechia |
| 2012 | 17.7954507876 | 0.814230401372392 | 1.41698894242212 | 16.3784618451779 | Czechia |
| 2013 | 19.72458641894 | 0.815615361108922 | 1.47240004456778 | 18.2521863743722 | Czechia |
| 2014 | 22.33855115272 | 0.816227257169909 | 1.44579883330066 | 20.8927523194193 | Czechia |
| 2015 | 25.6364538899 | 0.816564684423948 | 1.46245390701709 | 24.1739999828829 | Czechia |
| 2016 | 28.26115746239 | 0.817153025533518 | 1.47644314054578 | 26.7847143218442 | Czechia |
| 2017 | 28.55724182629 | 0.819067680353287 | 1.46004621315954 | 27.0971956131305 | Czechia |
| 2018 | 29.40417541772 | 0.822034486079137 | 1.42843776695814 | 27.9757376507619 | Czechia |
| 2019 | 31.344371488 | 0.824784472798171 | 1.43404266568537 | 29.9103288223146 | Czechia |
| 2020 | 25.86644074812 | 0.82662663116323 | 1.42294322374846 | 24.4434975243715 | Czechia |
| 2021 | 24.43045887484 | 0.828450433122785 | 1.4581093854041 | 22.9723494894359 | Czechia |
| 1990 | 23.26932340334 | 0.649419990664883 | 5.58670669503269 | 17.6826167083073 | Hungary |
| 1991 | 24.25626120263 | 0.653707154681922 | 5.09667195108986 | 19.1595892515401 | Hungary |
| 1992 | 23.13932443975 | 0.660382905455002 | 4.72816514340383 | 18.4111592963462 | Hungary |
| 1993 | 21.05509398083 | 0.667543556201878 | 3.65180474198678 | 17.4032892388432 | Hungary |
| 1994 | 19.87387552889 | 0.675051406249796 | 3.25549676597929 | 16.6183787629107 | Hungary |
| 1995 | 16.50097594602 | 0.683186411756315 | 2.77536295682867 | 13.7256129891913 | Hungary |
| 1996 | 15.34079255503 | 0.690883333624564 | 2.68016567223623 | 12.6606268827938 | Hungary |
| 1997 | 14.90533018784 | 0.697935513627811 | 2.3831573449331 | 12.5221728429069 | Hungary |
| 1998 | 14.49128037214 | 0.704202719984894 | 2.2887026984314 | 12.2025776737086 | Hungary |
| 1999 | 12.15725298574 | 0.709980626709343 | 2.21687040031239 | 9.94038258542761 | Hungary |
| 2000 | 13.21215539504 | 0.71580528057596 | 2.2079033340014 | 11.0042520610386 | Hungary |
| 2001 | 11.63133709138 | 0.722099055327699 | 2.20323406163702 | 9.42810302974298 | Hungary |
| 2002 | 10.74005526345 | 0.7283845957697 | 2.16907013862 | 8.57098512483 | Hungary |
| 2003 | 9.767401675 | 0.734450657925147 | 2.14826004861548 | 7.61914162638452 | Hungary |
| 2004 | 8.97293995391 | 0.740099974680479 | 2.11095941882047 | 6.86198053508953 | Hungary |
| 2005 | 8.70823656223 | 0.745356806381464 | 2.07299960686933 | 6.63523695536067 | Hungary |
| 2006 | 8.34090283871 | 0.750515380971436 | 2.05815614544943 | 6.28274669326057 | Hungary |
| 2007 | 8.35621582563 | 0.754942942046377 | 1.96421970071343 | 6.39199612491657 | Hungary |
| 2008 | 9.00373122379 | 0.75931746766686 | 1.87593966275622 | 7.12779156103378 | Hungary |
| 2009 | 10.1532823655 | 0.763563686880312 | 1.73209580872225 | 8.42118655677775 | Hungary |
| 2010 | 13.25428587985 | 0.767328649261652 | 1.6323550391837 | 11.6219308406663 | Hungary |
| 2011 | 18.07905417567 | 0.769701942198238 | 1.65592151477492 | 16.4231326608951 | Hungary |
| 2012 | 23.75335772356 | 0.770784245285382 | 1.57173388607554 | 22.1816238374845 | Hungary |
| 2013 | 28.58318544039 | 0.77156169789519 | 1.58419768673348 | 26.9989877536565 | Hungary |
| 2014 | 31.31003240402 | 0.772575045716759 | 1.54321876455266 | 29.7668136394673 | Hungary |
| 2015 | 33.22878056574 | 0.774047382518708 | 1.60197316605139 | 31.6268073996886 | Hungary |
| 2016 | 31.4397124837 | 0.776150305326983 | 1.52394410318969 | 29.9157683805103 | Hungary |
| 2017 | 30.09426140645 | 0.779116417184348 | 1.51008291051507 | 28.5841784959349 | Hungary |
| 2018 | 30.09411294902 | 0.782083661193681 | 1.43970549415062 | 28.6544074548694 | Hungary |
| 2019 | 35.29163583251 | 0.784910308752534 | 1.50935529255382 | 33.7822805399562 | Hungary |
| 2020 | 31.86704281189 | 0.787620389699141 | 1.4663385355424 | 30.4007042763476 | Hungary |
| 2021 | 29.67645512186 | 0.790754768173338 | 1.44563437128714 | 28.2308207505729 | Hungary |
| 1990 | 326.38036579219 | 0.60902609384517 | 13.1285567534487 | 313.251809038741 | North Macedonia |
| 1991 | 304.89807863865 | 0.611337452940336 | 13.3353079813065 | 291.562770657344 | North Macedonia |
| 1992 | 293.21976939463 | 0.612708856229581 | 13.2290783254491 | 279.990691069181 | North Macedonia |
| 1993 | 247.16946090703 | 0.613195098529578 | 13.2015370152324 | 233.967923891798 | North Macedonia |
| 1994 | 230.63962466197 | 0.614362905009181 | 13.4094022041331 | 217.230222457837 | North Macedonia |
| 1995 | 202.13355185093 | 0.616994877821318 | 13.1861930660939 | 188.947358784836 | North Macedonia |
| 1996 | 165.14897665907 | 0.620944017369103 | 13.0920492355252 | 152.056927423545 | North Macedonia |
| 1997 | 148.92055781979 | 0.626223811356564 | 12.947778556949 | 135.972779262841 | North Macedonia |
| 1998 | 141.87103667829 | 0.632059410181692 | 12.0582694289693 | 129.812767249321 | North Macedonia |
| 1999 | 102.93862897836 | 0.63668725909008 | 8.93470623287952 | 94.0039227454805 | North Macedonia |
| 2000 | 83.28240038983 | 0.641651242944231 | 7.16369146192173 | 76.1187089279083 | North Macedonia |
| 2001 | 77.35335655773 | 0.648049675806335 | 5.93966642620838 | 71.4136901315216 | North Macedonia |
| 2002 | 71.17014836087 | 0.655398790077733 | 5.06467500941028 | 66.1054733514597 | North Macedonia |
| 2003 | 71.64872400392 | 0.663408891257403 | 4.55264345744677 | 67.0960805464732 | North Macedonia |
| 2004 | 63.56233192455 | 0.671237520250547 | 3.25974496594113 | 60.3025869586089 | North Macedonia |
| 2005 | 55.69664333624 | 0.678047272834883 | 2.94789995266763 | 52.7487433835724 | North Macedonia |
| 2006 | 57.49084756599 | 0.684044336465219 | 2.91665103688429 | 54.5741965291057 | North Macedonia |
| 2007 | 55.48142116174 | 0.689729480929241 | 2.57986264605093 | 52.9015585156891 | North Macedonia |
| 2008 | 56.67655984976 | 0.695376955204976 | 2.44248948173323 | 54.2340703680268 | North Macedonia |
| 2009 | 59.92939034624 | 0.700453052486829 | 2.4308543439881 | 57.4985360022519 | North Macedonia |
| 2010 | 53.09381176635 | 0.705589323990971 | 2.30141888402054 | 50.7923928823295 | North Macedonia |
| 2011 | 53.4421625627 | 0.710592520608989 | 2.2696648819819 | 51.1724976807181 | North Macedonia |
| 2012 | 59.91898408217 | 0.715173147623766 | 2.19261978992306 | 57.7263642922469 | North Macedonia |
| 2013 | 60.42104566291 | 0.719671149159541 | 2.21242232409987 | 58.2086233388101 | North Macedonia |
| 2014 | 59.06062815774 | 0.724089588503488 | 2.16916774456726 | 56.8914604131727 | North Macedonia |
| 2015 | 58.0194702683 | 0.728603773495479 | 2.15916725385 | 55.86030301445 | North Macedonia |
| 2016 | 64.52722560069 | 0.733173624358204 | 2.17780984566081 | 62.3494157550292 | North Macedonia |
| 2017 | 55.98160392542 | 0.737339955393252 | 2.14863379831338 | 53.8329701271066 | North Macedonia |
| 2018 | 42.24616360575 | 0.741334369868002 | 2.11462362728828 | 40.1315399784617 | North Macedonia |
| 2019 | 40.79006742482 | 0.74519356873628 | 2.07851283373407 | 38.7115545910859 | North Macedonia |
| 2020 | 34.8837620868 | 0.747849899969173 | 2.03737245384909 | 32.8463896329509 | North Macedonia |
| 2021 | 29.28782355472 | 0.750629703270047 | 2.03347335723881 | 27.2543501974812 | North Macedonia |
| 1990 | 5.81219671962 | 0.674225719793669 | 3.1832482283617 | 2.6289484912583 | Montenegro |
| 1991 | 5.42885406266 | 0.674305722529628 | 2.83574368084359 | 2.59311038181641 | Montenegro |
| 1992 | 5.25159222433 | 0.672664771832277 | 3.27077415991104 | 1.98081806441896 | Montenegro |
| 1993 | 5.23410419248 | 0.668665719204089 | 3.69666258107526 | 1.53744161140474 | Montenegro |
| 1994 | 5.26101128357 | 0.665011931002947 | 4.38851504385282 | 0.87249623971718 | Montenegro |
| 1995 | 5.2036644078 | 0.662765437115331 | 4.37948641908389 | 0.824177988716111 | Montenegro |
| 1996 | 5.21344119279 | 0.663414768746409 | 4.38741925839216 | 0.826021934397843 | Montenegro |
| 1997 | 5.13751265009 | 0.665542031695028 | 4.38811982803125 | 0.749392822058749 | Montenegro |
| 1998 | 5.05452484817 | 0.669024770846263 | 3.56306199171413 | 1.49146285645587 | Montenegro |
| 1999 | 5.01434264894 | 0.672096102691543 | 3.36688533152714 | 1.64745731741286 | Montenegro |
| 2000 | 4.99047487185 | 0.677306850284635 | 2.78461194571023 | 2.20586292613977 | Montenegro |
| 2001 | 4.98659996841 | 0.683433454098582 | 2.81138043945667 | 2.17521952895333 | Montenegro |
| 2002 | 4.7744374838 | 0.68993948313756 | 2.4368793483155 | 2.3375581354845 | Montenegro |
| 2003 | 4.59099682816 | 0.696517506230494 | 2.43620835941651 | 2.15478846874349 | Montenegro |
| 2004 | 4.33664471214 | 0.702998276030296 | 2.30781224138472 | 2.02883247075528 | Montenegro |
| 2005 | 4.19073638565 | 0.709065464700775 | 2.264863919398 | 1.925872466252 | Montenegro |
| 2006 | 4.09622458293 | 0.715459074033141 | 2.14906916258097 | 1.94715542034903 | Montenegro |
| 2007 | 4.00285052012 | 0.722503939575403 | 2.17618456610267 | 1.82666595401733 | Montenegro |
| 2008 | 4.18853641329 | 0.729981755712811 | 2.18744425447952 | 2.00109215881048 | Montenegro |
| 2009 | 4.26522072439 | 0.736538925645409 | 2.150637646194 | 2.114583078196 | Montenegro |
| 2010 | 4.30279264145 | 0.743347873367499 | 2.09437836292227 | 2.20841427852773 | Montenegro |
| 2011 | 4.02598787475 | 0.750080328162275 | 2.06073974420767 | 1.96524813054233 | Montenegro |
| 2012 | 3.80991878572 | 0.755578379953158 | 2.03756384219756 | 1.77235494352244 | Montenegro |
| 2013 | 3.60667764744 | 0.760886907477753 | 1.83789007559448 | 1.76878757184552 | Montenegro |
| 2014 | 3.39875415539 | 0.765776526527122 | 1.51697405191519 | 1.88178010347481 | Montenegro |
| 2015 | 3.08268979559 | 0.770561277708901 | 1.5761817494797 | 1.5065080461103 | Montenegro |
| 2016 | 2.84910663323 | 0.775200770239438 | 1.54156209711231 | 1.30754453611769 | Montenegro |
| 2017 | 2.42704500944 | 0.779764631848314 | 1.53972163599333 | 0.887323373446667 | Montenegro |
| 2018 | 2.11407063147 | 0.784408971391706 | 1.48303886193586 | 0.631031769534138 | Montenegro |
| 2019 | 1.99715381737 | 0.789026903095641 | 1.50840849703769 | 0.488745320332308 | Montenegro |
| 2020 | 1.84828960864 | 0.792554392852858 | 1.50794029531389 | 0.340349313326111 | Montenegro |
| 2021 | 1.79722963687 | 0.795800584021403 | 1.442712394266 | 0.354517242604 | Montenegro |
| 1990 | 11.82238968877 | 0.62722788770829 | 11.82238968877 | 0 | Poland |
| 1991 | 9.37010434134 | 0.63267147050056 | 9.37010434134 | 0 | Poland |
| 1992 | 8.34033840545 | 0.64066179337278 | 7.11165560688786 | 1.22868279856214 | Poland |
| 1993 | 6.70330831864 | 0.648950499724012 | 5.23346041434118 | 1.46984790429882 | Poland |
| 1994 | 6.78180249782 | 0.658010514323026 | 4.8050264791693 | 1.9767760186507 | Poland |
| 1995 | 6.14221376943 | 0.666678866524195 | 3.87140852419105 | 2.27080524523895 | Poland |
| 1996 | 6.10450042836 | 0.674378829090982 | 2.96786531498385 | 3.13663511337615 | Poland |
| 1997 | 5.15429476119 | 0.682587612300048 | 2.81907586703093 | 2.33521889415907 | Poland |
| 1998 | 4.63547821584 | 0.690805670678968 | 2.56016161000237 | 2.07531660583763 | Poland |
| 1999 | 4.34122668975 | 0.6985360555797 | 2.37390540293587 | 1.96732128681413 | Poland |
| 2000 | 3.95666763425 | 0.706662051990276 | 2.30125741047056 | 1.65541022377944 | Poland |
| 2001 | 3.95878355304 | 0.714831304495085 | 2.225298413285 | 1.733485139755 | Poland |
| 2002 | 3.66104302478 | 0.722009458087754 | 2.23511417219628 | 1.42592885258372 | Poland |
| 2003 | 3.09286716269 | 0.728155253652566 | 2.18032318566222 | 0.912543977027778 | Poland |
| 2004 | 2.77577655391 | 0.733673191846001 | 2.18758232630571 | 0.588194227604286 | Poland |
| 2005 | 2.87695383919 | 0.738428766593663 | 2.16020292075909 | 0.716750918430909 | Poland |
| 2006 | 2.64500817543 | 0.742363708580365 | 2.09401522515829 | 0.550992950271715 | Poland |
| 2007 | 3.33374591659 | 0.746008817400637 | 2.10367556056886 | 1.23007035602114 | Poland |
| 2008 | 3.52073033394 | 0.750521426475047 | 2.05985870336053 | 1.46087163057947 | Poland |
| 2009 | 4.02427045812 | 0.756427378476364 | 1.98799207466783 | 2.03627838345217 | Poland |
| 2010 | 4.62836936991 | 0.763184973687688 | 1.74282524853867 | 2.88554412137133 | Poland |
| 2011 | 4.87170348423 | 0.769407164213607 | 1.72118235436958 | 3.15052112986042 | Poland |
| 2012 | 5.6115290558 | 0.774680767244991 | 1.44287034949511 | 4.16865870630489 | Poland |
| 2013 | 7.28010753258 | 0.779434257316854 | 1.48890773599604 | 5.79119979658396 | Poland |
| 2014 | 8.45190425039 | 0.783682368684721 | 1.53176562236757 | 6.92013862802243 | Poland |
| 2015 | 9.43856989098 | 0.78744534315882 | 1.46026741642974 | 7.97830247455026 | Poland |
| 2016 | 10.6869529778 | 0.790651014611623 | 1.43863870576359 | 9.24831427203641 | Poland |
| 2017 | 13.01498947431 | 0.79453034463794 | 1.47068446013102 | 11.544305014179 | Poland |
| 2018 | 12.90368536021 | 0.79947898187813 | 1.42649852805367 | 11.4771868321563 | Poland |
| 2019 | 12.30996381532 | 0.804791331675008 | 1.4473713247232 | 10.8625924905968 | Poland |
| 2020 | 11.25671266469 | 0.808795317506383 | 1.45492189285151 | 9.80179077183849 | Poland |
| 2021 | 10.77674398513 | 0.812042808667712 | 1.41458275195038 | 9.36216123317962 | Poland |
| 1990 | 79.78390201942 | 0.619298862250078 | 12.9902255421003 | 66.7936764773197 | Romania |
| 1991 | 59.41603255667 | 0.626795627433335 | 13.0211469607069 | 46.3948855959631 | Romania |
| 1992 | 58.9671784149 | 0.630475081501162 | 11.9142750034267 | 47.0529034114733 | Romania |
| 1993 | 61.33414725946 | 0.633801422118109 | 11.1341704530562 | 50.1999768064038 | Romania |
| 1994 | 69.05148222639 | 0.638371931788576 | 7.87585954947797 | 61.175622676912 | Romania |
| 1995 | 56.64365954652 | 0.643718879089372 | 7.43548644269407 | 49.2081731038259 | Romania |
| 1996 | 60.3407273099 | 0.648104195492848 | 5.69038354926316 | 54.6503437606368 | Romania |
| 1997 | 57.09331874726 | 0.651225576178548 | 5.54907892909247 | 51.5442398181675 | Romania |
| 1998 | 55.68525901771 | 0.654927768840234 | 5.1560373055118 | 50.5292217121982 | Romania |
| 1999 | 49.19517297016 | 0.65954484605753 | 4.38215718945016 | 44.8130157807098 | Romania |
| 2000 | 50.47503426792 | 0.66529434615185 | 4.40115140375082 | 46.0738828641692 | Romania |
| 2001 | 43.14383896253 | 0.672020166603488 | 3.1794367418655 | 39.9644022206645 | Romania |
| 2002 | 37.99893559734 | 0.678297416747085 | 2.88287285809159 | 35.1160627392484 | Romania |
| 2003 | 34.672401732 | 0.68407492928742 | 2.68200900870238 | 31.9903927232976 | Romania |
| 2004 | 32.47803893347 | 0.690417054639929 | 2.47959438278851 | 29.9984445506815 | Romania |
| 2005 | 27.99779416974 | 0.694998949594957 | 2.4670723587102 | 25.5307218110298 | Romania |
| 2006 | 23.55303229597 | 0.699490765699603 | 2.31548100041478 | 21.2375512955552 | Romania |
| 2007 | 17.01905812123 | 0.704523726685579 | 2.30684729353841 | 14.7122108276916 | Romania |
| 2008 | 16.78528459901 | 0.710757127721139 | 2.24474294410979 | 14.5405416549002 | Romania |
| 2009 | 16.38546022771 | 0.717445122377497 | 2.1883119891607 | 14.1971482385493 | Romania |
| 2010 | 17.53962824631 | 0.723891348732225 | 2.18381486057755 | 15.3558133857325 | Romania |
| 2011 | 19.05313509567 | 0.729018272021873 | 2.17631103015083 | 16.8768240655192 | Romania |
| 2012 | 20.13216751687 | 0.73389248238628 | 2.18042974031557 | 17.9517377765544 | Romania |
| 2013 | 21.6586801806 | 0.737880472910291 | 2.16012762073 | 19.49855255987 | Romania |
| 2014 | 21.75555657079 | 0.740395466703106 | 2.10233122670092 | 19.6532253440891 | Romania |
| 2015 | 22.65514679567 | 0.743473529634312 | 2.06125278658056 | 20.5938940090894 | Romania |
| 2016 | 22.41332076367 | 0.747058709528536 | 2.05075532207818 | 20.3625654415918 | Romania |
| 2017 | 23.5591346971 | 0.751117366992228 | 2.04866893742614 | 21.5104657596739 | Romania |
| 2018 | 22.51054148829 | 0.755533333480467 | 1.97795863233085 | 20.5325828559592 | Romania |
| 2019 | 20.56199520299 | 0.760283970607593 | 1.8264081693478 | 18.7355870336422 | Romania |
| 2020 | 18.96900217579 | 0.764276444363666 | 1.71129101788049 | 17.2577111579095 | Romania |
| 2021 | 18.79042201116 | 0.768453864365965 | 1.57588393181385 | 17.2145380793462 | Romania |
| 1990 | 24.5922746163 | 0.630511019536119 | 12.0722965059478 | 12.5199781103522 | Serbia |
| 1991 | 23.25986486878 | 0.638016934037528 | 8.77906027234375 | 14.4808045964363 | Serbia |
| 1992 | 19.96920013923 | 0.641971993828041 | 7.6741150240436 | 12.2950851151864 | Serbia |
| 1993 | 19.65849241287 | 0.642096034424249 | 8.50926468177267 | 11.1492277310973 | Serbia |
| 1994 | 20.75456712357 | 0.642223220931048 | 7.69448984769019 | 13.0600772758798 | Serbia |
| 1995 | 19.67049656166 | 0.642739671389532 | 7.66817797353885 | 12.0023185881211 | Serbia |
| 1996 | 17.93757453497 | 0.644846417030162 | 5.9075343347663 | 12.0300402002037 | Serbia |
| 1997 | 16.44702612846 | 0.64716209445227 | 5.75532696743559 | 10.6916991610244 | Serbia |
| 1998 | 15.27809294065 | 0.65024598899818 | 5.52678093788924 | 9.75131200276076 | Serbia |
| 1999 | 13.05185953097 | 0.655534174135398 | 4.81014149482721 | 8.24171803614279 | Serbia |
| 2000 | 12.53932483913 | 0.660752800457895 | 4.62167197342097 | 7.91765286570903 | Serbia |
| 2001 | 12.78026637043 | 0.665407334806451 | 4.43240093213898 | 8.34786543829102 | Serbia |
| 2002 | 13.41670583235 | 0.671511071572143 | 3.43954421914712 | 9.97716161320288 | Serbia |
| 2003 | 14.7206385165 | 0.678334928718905 | 3.10904015685085 | 11.6115983596491 | Serbia |
| 2004 | 13.03965152071 | 0.686819841842618 | 2.74673465325241 | 10.2929168674576 | Serbia |
| 2005 | 11.66622896064 | 0.69609756790601 | 2.379753227653 | 9.286475732987 | Serbia |
| 2006 | 10.83659514353 | 0.704945018093173 | 2.27429537756521 | 8.56229976596479 | Serbia |
| 2007 | 10.08545300367 | 0.713242304525204 | 2.22112758319964 | 7.86432542047036 | Serbia |
| 2008 | 7.95685871771 | 0.721505305994409 | 2.1961943594 | 5.76066435831 | Serbia |
| 2009 | 9.31018068006 | 0.729965595799672 | 2.1588496916365 | 7.1513309884235 | Serbia |
| 2010 | 9.01169883259 | 0.740091311255812 | 2.10268244897881 | 6.90901638361119 | Serbia |
| 2011 | 8.87539934749 | 0.750072761758797 | 2.05600786789095 | 6.81939147959905 | Serbia |
| 2012 | 8.27725754985 | 0.75710508155 | 1.88778984340392 | 6.38946770644608 | Serbia |
| 2013 | 8.87764705594 | 0.762170908106883 | 1.71343061682129 | 7.16421643911871 | Serbia |
| 2014 | 8.98869118549 | 0.766043091132074 | 1.72657690916737 | 7.26211427632263 | Serbia |
| 2015 | 9.19156380149 | 0.769842222617687 | 1.626794114058 | 7.564769687432 | Serbia |
| 2016 | 9.26242247316 | 0.773654077278106 | 1.56220640866667 | 7.70021606449333 | Serbia |
| 2017 | 9.27692431183 | 0.777460384270565 | 1.49267651963971 | 7.78424779219029 | Serbia |
| 2018 | 8.76178468188 | 0.781494973790012 | 1.464994283764 | 7.296790398116 | Serbia |
| 2019 | 8.44206473468 | 0.785768300182747 | 1.46720971854429 | 6.97485501613571 | Serbia |
| 2020 | 7.54778709741 | 0.789183332686432 | 1.42331519896079 | 6.12447189844921 | Serbia |
| 2021 | 7.16189758177 | 0.792416293685483 | 1.49472117098314 | 5.66717641078686 | Serbia |
| 1990 | 9.97601540148 | 0.653853505137647 | 5.56942458997104 | 4.40659081150896 | Slovakia |
| 1991 | 10.24259176912 | 0.658731402179317 | 4.43287717594408 | 5.80971459317592 | Slovakia |
| 1992 | 9.67888124482 | 0.665830110587888 | 4.32866675262395 | 5.35021449219605 | Slovakia |
| 1993 | 8.8121023261 | 0.67596793706605 | 3.0203564112414 | 5.7917459148586 | Slovakia |
| 1994 | 8.29401785789 | 0.688136476595487 | 2.47630860233444 | 5.81770925555555 | Slovakia |
| 1995 | 7.79976845277 | 0.697476536273994 | 2.51182323865765 | 5.28794521411235 | Slovakia |
| 1996 | 7.54983024184 | 0.704405003157211 | 2.28311856752921 | 5.26671167431079 | Slovakia |
| 1997 | 7.34947973216 | 0.711513209633452 | 2.22886712194263 | 5.12061261021737 | Slovakia |
| 1998 | 7.36090112581 | 0.719058766219034 | 2.19956306179415 | 5.16133806401585 | Slovakia |
| 1999 | 7.13043022312 | 0.726144172630556 | 2.16915344701949 | 4.96127677610051 | Slovakia |
| 2000 | 6.95021384895 | 0.734046240313494 | 2.17063734611 | 4.77957650284 | Slovakia |
| 2001 | 6.230486371 | 0.741392553570632 | 2.08663561777971 | 4.14385075322029 | Slovakia |
| 2002 | 6.47664959476 | 0.746791141582656 | 2.08607340138114 | 4.39057619337886 | Slovakia |
| 2003 | 7.01854780799 | 0.75116726952438 | 2.03041321042894 | 4.98813459756106 | Slovakia |
| 2004 | 7.25134920248 | 0.755836693705671 | 2.0084797462625 | 5.2428694562175 | Slovakia |
| 2005 | 7.38147824964 | 0.761724185107018 | 1.71718667356167 | 5.66429157607833 | Slovakia |
| 2006 | 7.23338922297 | 0.767673036224795 | 1.54044806402692 | 5.69294115894308 | Slovakia |
| 2007 | 7.29001973126 | 0.772914152845047 | 1.55827394503911 | 5.73174578622089 | Slovakia |
| 2008 | 7.5947836929 | 0.777197399879439 | 1.49720432136029 | 6.09757937153971 | Slovakia |
| 2009 | 8.09059706173 | 0.781052260265285 | 1.51082093745561 | 6.57977612427439 | Slovakia |
| 2010 | 8.1925531557 | 0.786254422327679 | 1.47130857542833 | 6.72124458027167 | Slovakia |
| 2011 | 8.05417835258 | 0.791465382423228 | 1.43737706397122 | 6.61680128860878 | Slovakia |
| 2012 | 8.24118574517 | 0.795456215611276 | 1.49860464086469 | 6.74258110430531 | Slovakia |
| 2013 | 8.29868526528 | 0.797903722940591 | 1.44320809311023 | 6.85547717216977 | Slovakia |
| 2014 | 8.7360331097 | 0.799618796308309 | 1.45263488365702 | 7.28339822604298 | Slovakia |
| 2015 | 9.4093260085 | 0.800394179928449 | 1.43197818401842 | 7.97734782448158 | Slovakia |
| 2016 | 9.93071877288 | 0.800878210489993 | 1.53550470027 | 8.39521407261 | Slovakia |
| 2017 | 10.16114430258 | 0.802277316569847 | 1.4437761552575 | 8.7173681473225 | Slovakia |
| 2018 | 10.20666150926 | 0.804357302109054 | 1.48783380906256 | 8.71882770019744 | Slovakia |
| 2019 | 9.70943227359 | 0.80644845685688 | 1.48160388686778 | 8.22782838672222 | Slovakia |
| 2020 | 9.10271506538 | 0.80830596588062 | 1.40226876414343 | 7.70044630123657 | Slovakia |
| 2021 | 8.64765383174 | 0.810610529921693 | 1.39913041037929 | 7.24852342136071 | Slovakia |
| 1990 | 4.86375854766 | 0.727463928368452 | 2.20380243304959 | 2.65995611461041 | Slovenia |
| 1991 | 4.70229548088 | 0.732666985968602 | 2.17557646039585 | 2.52671902048415 | Slovenia |
| 1992 | 4.80119100911 | 0.737391551733707 | 2.12848611733914 | 2.67270489177086 | Slovenia |
| 1993 | 4.35772122539 | 0.741774171511168 | 2.07762131933805 | 2.28009990605195 | Slovenia |
| 1994 | 4.219195597 | 0.746234861332899 | 2.08592219914087 | 2.13327339785913 | Slovenia |
| 1995 | 3.57083030996 | 0.750994974989173 | 2.0384928589835 | 1.5323374509765 | Slovenia |
| 1996 | 3.2910935738 | 0.756348987126594 | 2.02326327535359 | 1.26783029844641 | Slovenia |
| 1997 | 3.30206400426 | 0.762275291907702 | 1.72930657699886 | 1.57275742726114 | Slovenia |
| 1998 | 3.18873443823 | 0.767728525502705 | 1.57049182822395 | 1.61824261000605 | Slovenia |
| 1999 | 2.81994861381 | 0.773236192288015 | 1.59602270384184 | 1.22392590996816 | Slovenia |
| 2000 | 2.86589673847 | 0.779578009745196 | 1.45736326675 | 1.40853347172 | Slovenia |
| 2001 | 2.75177503466 | 0.786240775635761 | 1.41332476284833 | 1.33845027181167 | Slovenia |
| 2002 | 2.80379117889 | 0.791805569443613 | 1.44895640197917 | 1.35483477691083 | Slovenia |
| 2003 | 2.74733296835 | 0.796371530737719 | 1.48404894288477 | 1.26328402546523 | Slovenia |
| 2004 | 2.70117571433 | 0.800962240122577 | 1.46646646094378 | 1.23470925338622 | Slovenia |
| 2005 | 2.70443128091 | 0.805601248185604 | 1.43722430669312 | 1.26720697421688 | Slovenia |
| 2006 | 2.62081057348 | 0.809708595222897 | 1.44729647208535 | 1.17351410139465 | Slovenia |
| 2007 | 2.63731055608 | 0.813006319197598 | 1.49933427733049 | 1.13797627874951 | Slovenia |
| 2008 | 2.74299524294 | 0.816347250277984 | 1.47451837224438 | 1.26847687069563 | Slovenia |
| 2009 | 3.01659614997 | 0.818878560380979 | 1.42017212085026 | 1.59642402911974 | Slovenia |
| 2010 | 3.42802530923 | 0.82117848598052 | 1.395401022232 | 2.032624286998 | Slovenia |
| 2011 | 4.00883543904 | 0.823023560855118 | 1.41777495923871 | 2.59106047980129 | Slovenia |
| 2012 | 4.26756302589 | 0.824336562677773 | 1.42428025999871 | 2.84328276589129 | Slovenia |
| 2013 | 4.67081750543 | 0.825826275074906 | 1.45566420687489 | 3.21515329855511 | Slovenia |
| 2014 | 4.62703070511 | 0.827799245827941 | 1.47444611930481 | 3.15258458580519 | Slovenia |
| 2015 | 4.82364067075 | 0.829837070735453 | 1.40708818575795 | 3.41655248499205 | Slovenia |
| 2016 | 4.8899203864 | 0.831367915132541 | 1.43344336901465 | 3.45647701738535 | Slovenia |
| 2017 | 4.84228047833 | 0.833038139449118 | 1.44791760665474 | 3.39436287167526 | Slovenia |
| 2018 | 4.84223314887 | 0.835472480610787 | 1.41283284695281 | 3.42940030191719 | Slovenia |
| 2019 | 5.18470843845 | 0.838266782297842 | 1.40595966043838 | 3.77874877801162 | Slovenia |
| 2020 | 4.75172406248 | 0.840373845574037 | 1.39943098628889 | 3.35229307619111 | Slovenia |
| 2021 | 4.57391262939 | 0.842430731281465 | 1.38098764610571 | 3.19292498328429 | Slovenia |
| 1990 | 14.03136071906 | 0.622446576329452 | 12.9783788736075 | 1.05298184545245 | Belarus |
| 1991 | 14.27208747595 | 0.627038266774523 | 12.95741275607 | 1.31467471988 | Belarus |
| 1992 | 14.5381442641 | 0.632891737136784 | 10.7706508423074 | 3.76749342179255 | Belarus |
| 1993 | 15.84204405789 | 0.639394169351269 | 8.97947262366653 | 6.86257143422347 | Belarus |
| 1994 | 17.50323405885 | 0.64465278058669 | 5.99184881943392 | 11.5113852394161 | Belarus |
| 1995 | 16.78587776397 | 0.648959503797064 | 6.34635316823963 | 10.4395245957304 | Belarus |
| 1996 | 14.3554701253 | 0.652855220750827 | 5.22225674275706 | 9.13321338254294 | Belarus |
| 1997 | 13.46948708886 | 0.655627296459504 | 4.94202827853596 | 8.52745881032403 | Belarus |
| 1998 | 14.54492158389 | 0.657246466657203 | 4.92079047018554 | 9.62413111370446 | Belarus |
| 1999 | 14.77337759189 | 0.660171009515746 | 4.5950365837078 | 10.1783410081822 | Belarus |
| 2000 | 13.15448610033 | 0.664705822252137 | 4.52079910929382 | 8.63368699103618 | Belarus |
| 2001 | 11.82054315994 | 0.671333268854148 | 3.23944498341278 | 8.58109817652722 | Belarus |
| 2002 | 8.85149297874 | 0.678121782135605 | 2.94747881941213 | 5.90401415932787 | Belarus |
| 2003 | 7.73273227524 | 0.68486855613275 | 2.95135220442415 | 4.78138007081585 | Belarus |
| 2004 | 6.99755587416 | 0.692502135029653 | 2.6033713939263 | 4.3941844802337 | Belarus |
| 2005 | 6.17959060688 | 0.698985482904355 | 2.28751712907421 | 3.89207347780579 | Belarus |
| 2006 | 5.31055462312 | 0.705119102674127 | 2.34196865119255 | 2.96858597192745 | Belarus |
| 2007 | 4.87036005424 | 0.711496561875696 | 2.21422122597404 | 2.65613882826596 | Belarus |
| 2008 | 4.35049987866 | 0.719120871487284 | 2.19795100004185 | 2.15254887861815 | Belarus |
| 2009 | 4.13623315438 | 0.727292783613496 | 2.17891431629054 | 1.95731883808946 | Belarus |
| 2010 | 3.87790685896 | 0.734954626211817 | 2.16265170363386 | 1.71525515532614 | Belarus |
| 2011 | 3.79821643045 | 0.739504325946655 | 2.1637578566424 | 1.6344585738076 | Belarus |
| 2012 | 3.64883322516 | 0.743468837307909 | 2.0966445583 | 1.55218866686 | Belarus |
| 2013 | 3.53056996516 | 0.748529322885796 | 2.05662846313758 | 1.47394150202242 | Belarus |
| 2014 | 3.3925325521 | 0.753511479851413 | 2.0262795791249 | 1.3662529729751 | Belarus |
| 2015 | 3.26232507786 | 0.758205742923593 | 1.94114294814391 | 1.32118212971609 | Belarus |
| 2016 | 3.3071083226 | 0.764328221802501 | 1.72088617491882 | 1.58622214768118 | Belarus |
| 2017 | 3.28053035207 | 0.771131465670885 | 1.54631095488394 | 1.73421939718606 | Belarus |
| 2018 | 3.05585198994 | 0.776568956673705 | 1.47248496342222 | 1.58336702651778 | Belarus |
| 2019 | 2.88199631705 | 0.780508018535969 | 1.487086644442 | 1.394909672608 | Belarus |
| 2020 | 2.65465021257 | 0.782381564892966 | 1.49412522563154 | 1.16052498693846 | Belarus |
| 2021 | 2.60147929298 | 0.784484710906639 | 1.47341950054743 | 1.12805979243257 | Belarus |
| 1990 | 12.3237076798 | 0.674967632130919 | 2.98324641595034 | 9.34046126384965 | Estonia |
| 1991 | 11.25879578406 | 0.685223089296634 | 2.84088074019206 | 8.41791504386794 | Estonia |
| 1992 | 11.49007445979 | 0.695586673779285 | 2.39633580927111 | 9.09373865051889 | Estonia |
| 1993 | 11.82818316436 | 0.703693149995024 | 2.32160121709333 | 9.50658194726667 | Estonia |
| 1994 | 11.93987498126 | 0.708456180899464 | 2.28737758325491 | 9.65249739800509 | Estonia |
| 1995 | 11.44109365616 | 0.713172725189506 | 2.26014431741971 | 9.1809493387403 | Estonia |
| 1996 | 9.42053350317 | 0.718813024217038 | 2.19623589362795 | 7.22429760954205 | Estonia |
| 1997 | 8.7479686386 | 0.724652439639785 | 2.16392737165079 | 6.58404126694921 | Estonia |
| 1998 | 8.65923435473 | 0.729027035230187 | 2.21880074362619 | 6.44043361110381 | Estonia |
| 1999 | 9.09087701363 | 0.733250616186121 | 2.16310311237951 | 6.92777390125049 | Estonia |
| 2000 | 8.00881502707 | 0.739713154351944 | 2.13979789584162 | 5.86901713122838 | Estonia |
| 2001 | 7.14012256498 | 0.746602207163017 | 2.09461821273579 | 5.04550435224421 | Estonia |
| 2002 | 6.10718184909 | 0.752629898252246 | 2.02222066050842 | 4.08496118858158 | Estonia |
| 2003 | 5.80891985918 | 0.75781567757793 | 1.97857837444067 | 3.83034148473933 | Estonia |
| 2004 | 5.28735860913 | 0.763223727128029 | 1.72939830653024 | 3.55796030259976 | Estonia |
| 2005 | 4.98922728995 | 0.769112181926314 | 1.64308477126611 | 3.34614251868389 | Estonia |
| 2006 | 4.61709260999 | 0.774451841771662 | 1.64263721007444 | 2.97445539991556 | Estonia |
| 2007 | 4.5033818444 | 0.781074442846485 | 1.48857719967743 | 3.01480464472257 | Estonia |
| 2008 | 4.38923824381 | 0.788598873704278 | 1.41280864395474 | 2.97642959985526 | Estonia |
| 2009 | 4.18843038753 | 0.794728212699059 | 1.45037102624273 | 2.73805936128727 | Estonia |
| 2010 | 4.10863624907 | 0.800829717976428 | 1.44419822246587 | 2.66443802660413 | Estonia |
| 2011 | 3.85055390606 | 0.806633077522166 | 1.46338231678853 | 2.38717158927147 | Estonia |
| 2012 | 3.72597563644 | 0.811109357704526 | 1.43620111887548 | 2.28977451756452 | Estonia |
| 2013 | 3.57589111557 | 0.81488717677447 | 1.43429882001167 | 2.14159229555833 | Estonia |
| 2014 | 3.43870029454 | 0.818770732051979 | 1.42809474351725 | 2.01060555102275 | Estonia |
| 2015 | 3.36202265056 | 0.823221278492715 | 1.41399109508595 | 1.94803155547405 | Estonia |
| 2016 | 3.20327699862 | 0.827364360092018 | 1.40758541222667 | 1.79569158639333 | Estonia |
| 2017 | 3.11140911888 | 0.830975311283378 | 1.40535136879516 | 1.70605775008484 | Estonia |
| 2018 | 2.94481605797 | 0.834566296193434 | 1.43467019375107 | 1.51014586421893 | Estonia |
| 2019 | 2.83342977068 | 0.838573508963879 | 1.42711839012077 | 1.40631138055923 | Estonia |
| 2020 | 2.75206981961 | 0.841817139147138 | 1.39126205943147 | 1.36080776017853 | Estonia |
| 2021 | 2.70211589456 | 0.844917787360624 | 1.34901107070576 | 1.35310482385424 | Estonia |
| 1990 | 10.60467921318 | 0.680193638285329 | 2.88671139880703 | 7.71796781437297 | Latvia |
| 1991 | 11.43225942215 | 0.688117297290074 | 2.51281943498487 | 8.91943998716513 | Latvia |
| 1992 | 12.00378595517 | 0.696157035504877 | 2.46539078578942 | 9.53839516938058 | Latvia |
| 1993 | 12.72823604477 | 0.705249855159515 | 2.2537801801398 | 10.4744558646302 | Latvia |
| 1994 | 12.96945652302 | 0.712531352821191 | 2.23613513124537 | 10.7333213917746 | Latvia |
| 1995 | 12.33919635216 | 0.717639459211637 | 2.22570408308611 | 10.1134922690739 | Latvia |
| 1996 | 9.99845088632 | 0.721139537892551 | 2.2335096773666 | 7.7649412089534 | Latvia |
| 1997 | 8.86093533941 | 0.723746580428208 | 2.18064202037737 | 6.68029331903263 | Latvia |
| 1998 | 8.59704547345 | 0.725427335933284 | 2.18179138548455 | 6.41525408796546 | Latvia |
| 1999 | 8.43562114429 | 0.727219684959525 | 2.1821628924162 | 6.2534582518738 | Latvia |
| 2000 | 7.46183467523 | 0.730951448728138 | 2.18805085319561 | 5.27378382203439 | Latvia |
| 2001 | 6.96751347029 | 0.73629419637135 | 2.14547386727762 | 4.82203960301238 | Latvia |
| 2002 | 6.25057859803 | 0.741870440285041 | 2.14889728006459 | 4.1016813179654 | Latvia |
| 2003 | 5.57144354371 | 0.748168161846997 | 2.05001410487949 | 3.52142943883051 | Latvia |
| 2004 | 4.88720374747 | 0.755263211009698 | 2.02650511633032 | 2.86069863113968 | Latvia |
| 2005 | 4.50889742324 | 0.761929432669523 | 1.75831431902479 | 2.75058310421521 | Latvia |
| 2006 | 4.12493821877 | 0.768459928850117 | 1.54746175443 | 2.57747646434 | Latvia |
| 2007 | 3.98561500115 | 0.776481958669804 | 1.55672721050577 | 2.42888779064423 | Latvia |
| 2008 | 3.66337299872 | 0.786324573303851 | 1.52020320971444 | 2.14316978900556 | Latvia |
| 2009 | 3.450148543 | 0.794504426648315 | 1.45911851693947 | 1.99103002606053 | Latvia |
| 2010 | 3.33060420069 | 0.798171594063957 | 1.47024743643969 | 1.86035676425031 | Latvia |
| 2011 | 3.28267855104 | 0.799080776427927 | 1.48254465053441 | 1.80013390050559 | Latvia |
| 2012 | 3.17784413383 | 0.801026473960894 | 1.44534733051778 | 1.73249680331222 | Latvia |
| 2013 | 3.09459351324 | 0.802660490277978 | 1.43690214387667 | 1.65769136936333 | Latvia |
| 2014 | 3.0421602222 | 0.803721486035489 | 1.44838595209974 | 1.59377427010026 | Latvia |
| 2015 | 2.99478840399 | 0.806985994927815 | 1.45721415937139 | 1.53757424461861 | Latvia |
| 2016 | 2.81806700343 | 0.811463590660605 | 1.48157767839774 | 1.33648932503226 | Latvia |
| 2017 | 2.63150440658 | 0.816923118079976 | 1.41986074851897 | 1.21164365806103 | Latvia |
| 2018 | 2.34312650518 | 0.821419809272627 | 1.44843061434143 | 0.894695890838571 | Latvia |
| 2019 | 2.1731768288 | 0.824781776742666 | 1.39932494465519 | 0.773851884144815 | Latvia |
| 2020 | 2.033204779 | 0.827900448409988 | 1.4458153860596 | 0.5873893929404 | Latvia |
| 2021 | 1.9595688963 | 0.830663516299797 | 1.41406932474 | 0.54549957156 | Latvia |
| 1990 | 14.8084618441 | 0.668503937884641 | 3.66419788499173 | 11.1442639591083 | Lithuania |
| 1991 | 17.9446235484 | 0.673234120792409 | 3.29086575959296 | 14.653757788807 | Lithuania |
| 1992 | 19.66815917152 | 0.680992879643758 | 2.73448283671627 | 16.9336763348037 | Lithuania |
| 1993 | 18.51558500052 | 0.687480621018131 | 2.44805935776824 | 16.0675256427518 | Lithuania |
| 1994 | 17.69410345286 | 0.69115165298429 | 2.65244743697906 | 15.0416560158809 | Lithuania |
| 1995 | 15.64391704534 | 0.694734380082166 | 2.44860863566186 | 13.1953084096781 | Lithuania |
| 1996 | 12.69695630462 | 0.698591300871233 | 2.311050099132 | 10.385906205488 | Lithuania |
| 1997 | 11.42320882635 | 0.702840949666947 | 2.3868311562537 | 9.0363776700963 | Lithuania |
| 1998 | 10.81167794002 | 0.707468182196716 | 2.25359661504714 | 8.55808132497286 | Lithuania |
| 1999 | 11.25485780083 | 0.712834170940662 | 2.23507233930921 | 9.01978546152079 | Lithuania |
| 2000 | 9.95682473515 | 0.719896959838609 | 2.18333808753871 | 7.77348664761129 | Lithuania |
| 2001 | 8.63267984625 | 0.727133584877313 | 2.15407725634967 | 6.47860258990033 | Lithuania |
| 2002 | 7.69760905706 | 0.733793397915696 | 2.16063335625875 | 5.53697570080125 | Lithuania |
| 2003 | 6.79366266179 | 0.741229600875632 | 2.08753091872757 | 4.70613174306243 | Lithuania |
| 2004 | 6.38769168868 | 0.748949762305515 | 2.06590990658103 | 4.32178178209897 | Lithuania |
| 2005 | 5.79148395067 | 0.756352359983789 | 2.00817133915475 | 3.78331261151525 | Lithuania |
| 2006 | 5.38401792219 | 0.763059124350272 | 1.73314818182725 | 3.65086974036275 | Lithuania |
| 2007 | 5.03735092266 | 0.770229638520692 | 1.48872657265138 | 3.54862435000862 | Lithuania |
| 2008 | 4.83128827204 | 0.778273711505288 | 1.470083031868 | 3.361205240172 | Lithuania |
| 2009 | 4.54010103636 | 0.785689739125479 | 1.43090541680309 | 3.10919561955691 | Lithuania |
| 2010 | 4.36570007064 | 0.793364681757828 | 1.46603869794161 | 2.89966137269839 | Lithuania |
| 2011 | 4.13231666675 | 0.800924994333688 | 1.45454998293457 | 2.67776668381544 | Lithuania |
| 2012 | 3.87223672242 | 0.8078270261595 | 1.46110462576333 | 2.41113209665667 | Lithuania |
| 2013 | 3.89981477853 | 0.813219722329104 | 1.4424611019981 | 2.4573536765319 | Lithuania |
| 2014 | 4.00785344527 | 0.817886376021861 | 1.45337718278105 | 2.55447626248895 | Lithuania |
| 2015 | 4.18228864384 | 0.823112629019743 | 1.44255830420543 | 2.73973033963457 | Lithuania |
| 2016 | 4.21844961356 | 0.829303695106582 | 1.43468128104 | 2.78376833252 | Lithuania |
| 2017 | 3.9153184739 | 0.8358717398516 | 1.45854961451931 | 2.45676885938069 | Lithuania |
| 2018 | 3.77823733988 | 0.841918910402675 | 1.38419725486641 | 2.39404008501359 | Lithuania |
| 2019 | 3.57326500081 | 0.847906338850069 | 1.37783203205625 | 2.19543296875375 | Lithuania |
| 2020 | 3.23714468047 | 0.85275238761541 | 1.33294304131081 | 1.90420163915919 | Lithuania |
| 2021 | 3.1139713911 | 0.856484049109405 | 1.31365863961103 | 1.80031275148897 | Lithuania |
| 1990 | 93.47905335405 | 0.604251762377742 | 13.3242260702669 | 80.1548272837831 | Republic of Moldova |
| 1991 | 78.06608431157 | 0.607270773865529 | 13.22226563706 | 64.84381867451 | Republic of Moldova |
| 1992 | 77.5951784602 | 0.609664558440288 | 13.4756861103758 | 64.1194923498242 | Republic of Moldova |
| 1993 | 81.73204830513 | 0.614152920432239 | 13.2878474767325 | 68.4442008283975 | Republic of Moldova |
| 1994 | 88.27244610382 | 0.617603766824611 | 12.8907117403073 | 75.3817343635127 | Republic of Moldova |
| 1995 | 77.40713407191 | 0.620951321121127 | 13.1433923410214 | 64.2637417308886 | Republic of Moldova |
| 1996 | 68.81095011659 | 0.623113983844076 | 13.0732507373555 | 55.7376993792346 | Republic of Moldova |
| 1997 | 60.61125761041 | 0.624370104753213 | 13.2306315194489 | 47.3806260909611 | Republic of Moldova |
| 1998 | 54.42785677649 | 0.62411676120849 | 13.0919193329402 | 41.3359374435499 | Republic of Moldova |
| 1999 | 51.96338816182 | 0.622834528688654 | 12.9405025329757 | 39.0228856288443 | Republic of Moldova |
| 2000 | 47.89864860888 | 0.622004933502906 | 13.1611111417548 | 34.7375374671252 | Republic of Moldova |
| 2001 | 45.04601381614 | 0.622936409943787 | 12.823697799428 | 32.222316016712 | Republic of Moldova |
| 2002 | 39.40103308528 | 0.625874844526255 | 13.0405773414511 | 26.3604557438289 | Republic of Moldova |
| 2003 | 36.29700922482 | 0.629985044767633 | 12.1380264270405 | 24.1589827977795 | Republic of Moldova |
| 2004 | 32.14279844782 | 0.635394821992876 | 10.9545666638725 | 21.1882317839475 | Republic of Moldova |
| 2005 | 27.73102497994 | 0.641530965095913 | 7.20751988059705 | 20.5235050993429 | Republic of Moldova |
| 2006 | 22.67078080802 | 0.647996263561811 | 5.90116785557778 | 16.7696129524422 | Republic of Moldova |
| 2007 | 20.40998098695 | 0.65421585059876 | 4.88056117809623 | 15.5294198088538 | Republic of Moldova |
| 2008 | 19.45419699266 | 0.661046597063232 | 4.79867115966723 | 14.6555258329928 | Republic of Moldova |
| 2009 | 19.00043504848 | 0.666284393801024 | 3.72669483994925 | 15.2737402085308 | Republic of Moldova |
| 2010 | 17.20286840682 | 0.671989615961323 | 3.26903761598305 | 13.9338307908369 | Republic of Moldova |
| 2011 | 18.09947944929 | 0.678004136643775 | 2.92636696476235 | 15.1731124845276 | Republic of Moldova |
| 2012 | 15.70170197409 | 0.682979795888934 | 2.77084045166348 | 12.9308615224265 | Republic of Moldova |
| 2013 | 14.59578136113 | 0.688519174704066 | 2.57175455432257 | 12.0240268068074 | Republic of Moldova |
| 2014 | 13.7195348991 | 0.694104557276994 | 2.48581627497804 | 11.233718624122 | Republic of Moldova |
| 2015 | 14.406270061 | 0.699355166217478 | 2.32612559946109 | 12.0801444615389 | Republic of Moldova |
| 2016 | 13.79934892226 | 0.70514454200476 | 2.267805798032 | 11.531543124228 | Republic of Moldova |
| 2017 | 14.36438080358 | 0.711206637139458 | 2.23794931978983 | 12.1264314837902 | Republic of Moldova |
| 2018 | 14.05506563004 | 0.717244426652362 | 2.19639278404466 | 11.8586728459953 | Republic of Moldova |
| 2019 | 12.99926842586 | 0.723011647628002 | 2.17302665291709 | 10.8262417729429 | Republic of Moldova |
| 2020 | 12.13104519102 | 0.727710540349217 | 2.18204819091911 | 9.94899700010089 | Republic of Moldova |
| 2021 | 11.42408785202 | 0.732214875427507 | 2.19023865816737 | 9.23384919385263 | Republic of Moldova |
| 1990 | 31.82613698888 | 0.671600578021767 | 3.46938399135797 | 28.356752997522 | Russian Federation |
| 1991 | 30.6722983842 | 0.680099211484661 | 2.94755893556952 | 27.7247394486305 | Russian Federation |
| 1992 | 29.84781007217 | 0.688068021982768 | 2.60631125764362 | 27.2414988145264 | Russian Federation |
| 1993 | 33.36293291238 | 0.692036194318927 | 2.51080661878509 | 30.8521262935949 | Russian Federation |
| 1994 | 33.6316502206 | 0.694144966076361 | 2.48168936605494 | 31.1499608545451 | Russian Federation |
| 1995 | 33.49397120844 | 0.697854213747881 | 2.35248630042847 | 31.1414849080115 | Russian Federation |
| 1996 | 29.77328625294 | 0.701468230734779 | 2.33248280716547 | 27.4408034457745 | Russian Federation |
| 1997 | 28.15792677724 | 0.703752867289891 | 2.3083109917325 | 25.8496157855075 | Russian Federation |
| 1998 | 28.20776143732 | 0.705198518088381 | 2.32283646727722 | 25.8849249700428 | Russian Federation |
| 1999 | 30.90572198962 | 0.706863537909859 | 2.25846869828742 | 28.6472532913326 | Russian Federation |
| 2000 | 28.38492512368 | 0.708643647769121 | 2.22390470949333 | 26.1610204141867 | Russian Federation |
| 2001 | 26.36000580434 | 0.710570359001004 | 2.25102164120246 | 24.1089841631375 | Russian Federation |
| 2002 | 22.69417682245 | 0.713460956547605 | 2.29180800694833 | 20.4023688155017 | Russian Federation |
| 2003 | 20.45047743543 | 0.718875116049301 | 2.21961919328147 | 18.2308582421485 | Russian Federation |
| 2004 | 17.31295251766 | 0.726305319497695 | 2.177281056586 | 15.135671461074 | Russian Federation |
| 2005 | 15.3625551394 | 0.733592776524904 | 2.18671102346091 | 13.1758441159391 | Russian Federation |
| 2006 | 13.28954259013 | 0.740064663588135 | 2.12362165735102 | 11.165920932779 | Russian Federation |
| 2007 | 12.17810801748 | 0.746191512265979 | 2.08756787646222 | 10.0905401410178 | Russian Federation |
| 2008 | 10.57155291547 | 0.752623243282606 | 2.03895874181245 | 8.53259417365755 | Russian Federation |
| 2009 | 9.46462246744 | 0.757960404689196 | 1.899148852248 | 7.565473615192 | Russian Federation |
| 2010 | 8.94716044227 | 0.763320116187813 | 1.73373144172614 | 7.21342900054386 | Russian Federation |
| 2011 | 8.52579688576 | 0.767633587927234 | 1.59004809778958 | 6.93574878797042 | Russian Federation |
| 2012 | 9.12210555025 | 0.77192038427212 | 1.59327533360636 | 7.52883021664364 | Russian Federation |
| 2013 | 8.43029695739 | 0.776526978961411 | 1.47462766773875 | 6.95566928965125 | Russian Federation |
| 2014 | 7.68134826872 | 0.780617524284297 | 1.5279132597713 | 6.1534350089487 | Russian Federation |
| 2015 | 6.61606198058 | 0.784913773964283 | 1.44045352180529 | 5.17560845877471 | Russian Federation |
| 2016 | 6.03378079467 | 0.790330149439335 | 1.44531478248067 | 4.58846601218933 | Russian Federation |
| 2017 | 5.37652123107 | 0.79566948446832 | 1.47422794281854 | 3.90229328825146 | Russian Federation |
| 2018 | 4.57090256466 | 0.800108144129611 | 1.49459346749462 | 3.07630909716538 | Russian Federation |
| 2019 | 4.08899794027 | 0.803658201264112 | 1.4302909186125 | 2.6587070216575 | Russian Federation |
| 2020 | 3.88860730845 | 0.806011474298182 | 1.42146530214143 | 2.46714200630857 | Russian Federation |
| 2021 | 3.75832394221 | 0.808536004906566 | 1.46397222953687 | 2.29435171267313 | Russian Federation |
| 1990 | 10.76654691738 | 0.647461465528387 | 5.75957346723857 | 5.00697345014143 | Ukraine |
| 1991 | 10.73249982132 | 0.651950021194653 | 4.56976216106714 | 6.16273766025286 | Ukraine |
| 1992 | 11.53510312126 | 0.657600333165733 | 4.75505562077156 | 6.78004750048845 | Ukraine |
| 1993 | 13.01649613536 | 0.66286740459174 | 4.49576834268385 | 8.52072779267615 | Ukraine |
| 1994 | 17.27525734616 | 0.665506374866164 | 4.37452346465696 | 12.900733881503 | Ukraine |
| 1995 | 15.0221917611 | 0.666950166648749 | 3.48364195108964 | 11.5385498100104 | Ukraine |
| 1996 | 13.30353030652 | 0.668104744367524 | 3.82953487201774 | 9.47399543450227 | Ukraine |
| 1997 | 12.65975856334 | 0.669207845755283 | 3.70634653051396 | 8.95341203282604 | Ukraine |
| 1998 | 10.66252383298 | 0.670322300548544 | 3.75030561354875 | 6.91221821943125 | Ukraine |
| 1999 | 9.65186507986 | 0.670642905451183 | 3.7415676609387 | 5.9102974189213 | Ukraine |
| 2000 | 7.89811312752 | 0.671095000943321 | 4.04212148189903 | 3.85599164562097 | Ukraine |
| 2001 | 6.74372459468 | 0.672467819796801 | 3.42336685338184 | 3.32035774129816 | Ukraine |
| 2002 | 6.2334672196 | 0.674543220896083 | 3.04301039542059 | 3.19045682417941 | Ukraine |
| 2003 | 5.29017700088 | 0.678614636476308 | 3.14495110770525 | 2.14522589317475 | Ukraine |
| 2004 | 4.55414001887 | 0.685380936146257 | 2.81673049842977 | 1.73740952044023 | Ukraine |
| 2005 | 4.1725948165 | 0.692023300673985 | 2.54156637910607 | 1.63102843739393 | Ukraine |
| 2006 | 3.81652340439 | 0.698093382536473 | 2.39275390620613 | 1.42376949818387 | Ukraine |
| 2007 | 3.44648627632 | 0.704202307705725 | 2.30356407689526 | 1.14292219942474 | Ukraine |
| 2008 | 3.54500873786 | 0.710980183909559 | 2.19324847797389 | 1.35176025988611 | Ukraine |
| 2009 | 3.0131534982 | 0.716185465482395 | 2.19661837154129 | 0.81653512665871 | Ukraine |
| 2010 | 2.8200198186 | 0.72101987022281 | 2.18449609584047 | 0.635523722759535 | Ukraine |
| 2011 | 2.70998357805 | 0.724891957373533 | 2.16376105463273 | 0.546222523417273 | Ukraine |
| 2012 | 2.5783416899 | 0.728817199844111 | 2.18526431833757 | 0.393077371562432 | Ukraine |
| 2013 | 2.47828507328 | 0.734033572821072 | 2.154729666688 | 0.323555406592 | Ukraine |
| 2014 | 1.90500574224 | 0.739883556959807 | 1.90500574224 | 0 | Ukraine |
| 2015 | 2.06506774432 | 0.743790863159826 | 2.02813035922462 | 0.0369373850953845 | Ukraine |
| 2016 | 2.0924129208 | 0.746857988843031 | 2.05424763887556 | 0.0381652819244445 | Ukraine |
| 2017 | 2.00692973597 | 0.750593324392773 | 1.97507848792938 | 0.031851248040625 | Ukraine |
| 2018 | 1.83278225496 | 0.754451727274318 | 1.83278225496 | 0 | Ukraine |
| 2019 | 1.75501689025 | 0.757980081219807 | 1.712553915584 | 0.0424629746659999 | Ukraine |
| 2020 | 1.79300735319 | 0.759929872218543 | 1.71352394972174 | 0.0794834034682608 | Ukraine |
| 2021 | 1.72612451379 | 0.760773912846782 | 1.67217672059294 | 0.0539477931970589 | Ukraine |
| 1990 | 2.41951890447 | 0.66608191698766 | 2.41951890447 | 0 | Brunei Darussalam |
| 1991 | 2.42136172127 | 0.671233831388631 | 2.42060291435235 | 0.000758806917647181 | Brunei Darussalam |
| 1992 | 2.39177040717 | 0.676702526223706 | 2.39177040717 | 0 | Brunei Darussalam |
| 1993 | 2.35539285793 | 0.682175439911044 | 2.35539285793 | 0 | Brunei Darussalam |
| 1994 | 2.3130875734 | 0.68739409348098 | 2.3130875734 | 0 | Brunei Darussalam |
| 1995 | 2.25079536941 | 0.692471156008533 | 2.25079536941 | 0 | Brunei Darussalam |
| 1996 | 2.19660493574 | 0.697602233240377 | 2.19660493574 | 0 | Brunei Darussalam |
| 1997 | 2.15099499752 | 0.702944386203433 | 2.15099499752 | 0 | Brunei Darussalam |
| 1998 | 2.11078933428 | 0.708630119469791 | 2.11078933428 | 0 | Brunei Darussalam |
| 1999 | 2.08728864312 | 0.714851237118356 | 2.08728864312 | 0 | Brunei Darussalam |
| 2000 | 2.11649465956 | 0.72155249267874 | 2.10671405157818 | 0.0097806079818179 | Brunei Darussalam |
| 2001 | 2.1176557175 | 0.728535069338112 | 2.10533715461083 | 0.0123185628891664 | Brunei Darussalam |
| 2002 | 2.18524816372 | 0.735343916434566 | 2.13279687543613 | 0.052451288283871 | Brunei Darussalam |
| 2003 | 2.24833006794 | 0.741578091315293 | 2.08822925703125 | 0.16010081090875 | Brunei Darussalam |
| 2004 | 2.26827436712 | 0.747311714682537 | 2.04928958310071 | 0.218984784019286 | Brunei Darussalam |
| 2005 | 2.30093284272 | 0.752481792632817 | 2.03435830693065 | 0.266574535789355 | Brunei Darussalam |
| 2006 | 2.3126445143 | 0.75731865511743 | 1.95203884016742 | 0.360605674132581 | Brunei Darussalam |
| 2007 | 2.34642536512 | 0.761643128654341 | 1.73134892054231 | 0.615076444577692 | Brunei Darussalam |
| 2008 | 2.38391364897 | 0.765714260433754 | 1.66839569990946 | 0.715517949060541 | Brunei Darussalam |
| 2009 | 2.41149159141 | 0.769601681624648 | 1.67902218407229 | 0.732469407337714 | Brunei Darussalam |
| 2010 | 2.44160129463 | 0.773219221249055 | 1.62949636107176 | 0.812104933558235 | Brunei Darussalam |
| 2011 | 2.50876098685 | 0.776631893191597 | 1.52546775291972 | 0.983293233930278 | Brunei Darussalam |
| 2012 | 2.53023693925 | 0.780216551684601 | 1.46818826944826 | 1.06204866980174 | Brunei Darussalam |
| 2013 | 2.51197112619 | 0.784017040989581 | 1.43515056388371 | 1.07682056230629 | Brunei Darussalam |
| 2014 | 2.47905205999 | 0.787733289361499 | 1.46036328931364 | 1.01868877067636 | Brunei Darussalam |
| 2015 | 2.44723795406 | 0.791367566956237 | 1.41986487251281 | 1.02737308154719 | Brunei Darussalam |
| 2016 | 2.39428952723 | 0.794980772397229 | 1.39234923637575 | 1.00194029085425 | Brunei Darussalam |
| 2017 | 2.36170249627 | 0.798309936375838 | 1.42511265877321 | 0.936589837496786 | Brunei Darussalam |
| 2018 | 2.31361310996 | 0.801436553226482 | 1.44934572265806 | 0.864267387301944 | Brunei Darussalam |
| 2019 | 2.26611514027 | 0.804499388246421 | 1.45379217457815 | 0.812322965691852 | Brunei Darussalam |
| 2020 | 2.18322199912 | 0.80742409075008 | 1.494284209769 | 0.688937789351 | Brunei Darussalam |
| 2021 | 2.08171701671 | 0.810234366947283 | 1.40188091451889 | 0.679836102191111 | Brunei Darussalam |
| 1990 | 4.0458318546 | 0.790253515528297 | 1.50308162753318 | 2.54275022706682 | Japan |
| 1991 | 4.09722025673 | 0.794616189497547 | 1.46030365096625 | 2.63691660576375 | Japan |
| 1992 | 4.12926779998 | 0.799156224748283 | 1.39540309376158 | 2.73386470621842 | Japan |
| 1993 | 4.14274527309 | 0.802972994199584 | 1.42568910874917 | 2.71705616434083 | Japan |
| 1994 | 4.27581784807 | 0.806689483098495 | 1.42030356407138 | 2.85551428399862 | Japan |
| 1995 | 4.23860534589 | 0.810510771484257 | 1.46809536169854 | 2.77050998419146 | Japan |
| 1996 | 4.18422439559 | 0.813870745535327 | 1.44682211548613 | 2.73740228010387 | Japan |
| 1997 | 4.31467612468 | 0.816675782376649 | 1.45647890780255 | 2.85819721687745 | Japan |
| 1998 | 4.40505787321 | 0.819016201504856 | 1.426336694395 | 2.978721178815 | Japan |
| 1999 | 4.26655077307 | 0.82106663705133 | 1.43447745208791 | 2.83207332098209 | Japan |
| 2000 | 4.27066119557 | 0.822938998623378 | 1.38903378154864 | 2.88162741402136 | Japan |
| 2001 | 4.32528893489 | 0.824723241254759 | 1.43929328804056 | 2.88599564684944 | Japan |
| 2002 | 4.51930978552 | 0.82693677356819 | 1.43677970544381 | 3.08253008007619 | Japan |
| 2003 | 4.79664035404 | 0.829392223273124 | 1.4028656328061 | 3.3937747212339 | Japan |
| 2004 | 5.00496183899 | 0.832037381275171 | 1.42385607503851 | 3.58110576395149 | Japan |
| 2005 | 5.35691229307 | 0.834288685362289 | 1.43986273099925 | 3.91704956207075 | Japan |
| 2006 | 5.64224002348 | 0.836222264006662 | 1.400387796598 | 4.241852226882 | Japan |
| 2007 | 5.69378151451 | 0.83829415979033 | 1.398057959323 | 4.295723555187 | Japan |
| 2008 | 5.59627010726 | 0.840301217168455 | 1.41654774733098 | 4.17972235992902 | Japan |
| 2009 | 5.39815534457 | 0.842133664144895 | 1.40183475317524 | 3.99632059139476 | Japan |
| 2010 | 5.47584044958 | 0.844361810771781 | 1.35188693855229 | 4.12395351102771 | Japan |
| 2011 | 5.48128582049 | 0.846750266822805 | 1.32633007620159 | 4.15495574428841 | Japan |
| 2012 | 5.55385824693 | 0.849061489406189 | 1.3501769284814 | 4.2036813184486 | Japan |
| 2013 | 5.26941771111 | 0.851356993626047 | 1.32866236981174 | 3.94075534129826 | Japan |
| 2014 | 5.00487088337 | 0.85369274935716 | 1.3125085316104 | 3.6923623517596 | Japan |
| 2015 | 4.86579071963 | 0.856097498738686 | 1.31032344207 | 3.55546727756 | Japan |
| 2016 | 4.74354471454 | 0.858698173210648 | 1.29866236336796 | 3.44488235117204 | Japan |
| 2017 | 4.51710608429 | 0.861393308858287 | 1.30811178061889 | 3.20899430367111 | Japan |
| 2018 | 4.36025095098 | 0.864237136659234 | 1.29009658531606 | 3.07015436566394 | Japan |
| 2019 | 4.10312672556 | 0.867148183486851 | 1.29934961702595 | 2.80377710853405 | Japan |
| 2020 | 3.90513168537 | 0.869106879336459 | 1.26690519441419 | 2.63822649095581 | Japan |
| 2021 | 3.81969228521 | 0.871241812572128 | 1.26614024336154 | 2.55355204184846 | Japan |
| 1990 | 3.15232431478 | 0.692329306595283 | 2.46117584775735 | 0.691148467022647 | Republic of Korea |
| 1991 | 2.91207918976 | 0.702631128494537 | 2.36946895249641 | 0.54261023726359 | Republic of Korea |
| 1992 | 2.71943462072 | 0.712440841630159 | 2.22821175036255 | 0.491222870357447 | Republic of Korea |
| 1993 | 2.50625189107 | 0.722286739631754 | 2.20327146751279 | 0.302980423557209 | Republic of Korea |
| 1994 | 2.32077935484 | 0.732197906155207 | 2.15288794153407 | 0.167891413305926 | Republic of Korea |
| 1995 | 2.14574745044 | 0.741940029590316 | 2.0677233561744 | 0.0780240942655999 | Republic of Korea |
| 1996 | 1.99758019306 | 0.751553240476733 | 1.97113034996857 | 0.0264498430914286 | Republic of Korea |
| 1997 | 1.85043938206 | 0.760591683388116 | 1.78190117922211 | 0.0685382028378947 | Republic of Korea |
| 1998 | 1.7276424782 | 0.76828188532262 | 1.517521978551 | 0.210120499649 | Republic of Korea |
| 1999 | 1.62043838077 | 0.776773436337718 | 1.48047028776222 | 0.139968093007778 | Republic of Korea |
| 2000 | 1.62028534327 | 0.785428821409438 | 1.46994311475111 | 0.150342228518889 | Republic of Korea |
| 2001 | 1.6228616862 | 0.793496280690955 | 1.41911270569833 | 0.203748980501667 | Republic of Korea |
| 2002 | 1.59391419745 | 0.801024295470106 | 1.45065072752 | 0.14326346993 | Republic of Korea |
| 2003 | 1.57324701104 | 0.807687785876648 | 1.39276576894714 | 0.180481242092857 | Republic of Korea |
| 2004 | 1.53792362749 | 0.813720926701095 | 1.425440090556 | 0.112483536934 | Republic of Korea |
| 2005 | 1.49003717565 | 0.819093399889013 | 1.39621546420125 | 0.0938217114487501 | Republic of Korea |
| 2006 | 1.47855070071 | 0.824179820431794 | 1.36366737014 | 0.11488333057 | Republic of Korea |
| 2007 | 1.45933803332 | 0.82912795326169 | 1.42807986199071 | 0.0312581713292857 | Republic of Korea |
| 2008 | 1.40383554148 | 0.833894626872214 | 1.35226194665632 | 0.0515735948236842 | Republic of Korea |
| 2009 | 1.33737545902 | 0.838108330520764 | 1.31615398802 | 0.021221471 | Republic of Korea |
| 2010 | 1.29639983677 | 0.84251142929369 | 1.2917552922404 | 0.00464454452959995 | Republic of Korea |
| 2011 | 1.24241331685 | 0.846913228744834 | 1.24241331685 | 0 | Republic of Korea |
| 2012 | 1.19547198872 | 0.851140262773848 | 1.19547198872 | 0 | Republic of Korea |
| 2013 | 1.14017276303 | 0.855441000170575 | 1.14017276303 | 0 | Republic of Korea |
| 2014 | 1.09214091983 | 0.859665567500037 | 1.09214091983 | 0 | Republic of Korea |
| 2015 | 1.05280407713 | 0.863907408481354 | 1.05280407713 | 0 | Republic of Korea |
| 2016 | 1.03313066219 | 0.868317824083966 | 1.03313066219 | 0 | Republic of Korea |
| 2017 | 1.01963412248 | 0.87261776393019 | 1.01963412248 | 0 | Republic of Korea |
| 2021 | 0.88540352714 | 0.886675266756997 | 0.88540352714 | 0 | Republic of Korea |
| 1990 | 5.38956934541 | 0.686404444349548 | 2.67350347317267 | 2.71606587223733 | Singapore |
| 1991 | 4.19961954176 | 0.695055557646282 | 2.34791104495552 | 1.85170849680448 | Singapore |
| 1992 | 3.44916254706 | 0.703427834229016 | 2.29462481020265 | 1.15453773685735 | Singapore |
| 1993 | 2.98211046021 | 0.712315203759158 | 2.25053883570667 | 0.731571624503333 | Singapore |
| 1994 | 2.88582245959 | 0.721256796938456 | 2.26664969956889 | 0.619172760021111 | Singapore |
| 1995 | 2.48921328062 | 0.729982231248165 | 2.18011911216931 | 0.30909416845069 | Singapore |
| 1996 | 2.28190120168 | 0.739028234741754 | 2.14335653957636 | 0.138544662103636 | Singapore |
| 1997 | 2.34418663382 | 0.747571897605582 | 2.0721759382128 | 0.2720106956072 | Singapore |
| 1998 | 3.04074397034 | 0.754873203577067 | 2.02555763847974 | 1.01518633186026 | Singapore |
| 1999 | 2.34649716597 | 0.760831136110222 | 1.80284171842412 | 0.543655447545882 | Singapore |
| 2000 | 2.40931090851 | 0.767717370394546 | 1.672433315921 | 0.736877592589 | Singapore |
| 2001 | 2.00359723082 | 0.774294656621655 | 1.60287418609792 | 0.400723044722083 | Singapore |
| 2002 | 1.93127470526 | 0.77973576984914 | 1.44422484948565 | 0.487049855774348 | Singapore |
| 2003 | 1.80523741315 | 0.784165555316375 | 1.51958893240286 | 0.285648480747143 | Singapore |
| 2004 | 1.73132592716 | 0.789852773877056 | 1.4042966984864 | 0.3270292286736 | Singapore |
| 2005 | 1.85176924367 | 0.796455750753104 | 1.3901352761481 | 0.461633967521905 | Singapore |
| 2006 | 2.11829281577 | 0.803342694397764 | 1.46705463361289 | 0.651238182157105 | Singapore |
| 2007 | 2.17425309147 | 0.810625304101189 | 1.44777745176743 | 0.726475639702571 | Singapore |
| 2008 | 2.27833476047 | 0.818124162030734 | 1.42588156684585 | 0.852453193624146 | Singapore |
| 2009 | 2.44042649307 | 0.824251515940882 | 1.37380342449103 | 1.06662306857897 | Singapore |
| 2010 | 2.49261923191 | 0.830222159553543 | 1.40752088370455 | 1.08509834820545 | Singapore |
| 2011 | 2.39429850505 | 0.833829607920547 | 1.43058553929289 | 0.963712965757105 | Singapore |
| 2012 | 2.27253968882 | 0.837391806757054 | 1.43971687774429 | 0.832822811075714 | Singapore |
| 2013 | 1.99560588309 | 0.840420177683154 | 1.37827969060571 | 0.617326192484286 | Singapore |
| 2014 | 1.62513722899 | 0.842894537933952 | 1.3490301444219 | 0.276107084568095 | Singapore |
| 2015 | 1.55106151236 | 0.84540185703118 | 1.414203497945 | 0.136858014415 | Singapore |
| 2016 | 1.58699576913 | 0.847596998735509 | 1.32298022459375 | 0.26401554453625 | Singapore |
| 2017 | 1.41972183536 | 0.849051517606326 | 1.34551186239 | 0.0742099729700001 | Singapore |
| 2018 | 1.44064163891 | 0.850762728725121 | 1.31390144559789 | 0.126740193312105 | Singapore |
| 2019 | 1.36410992757 | 0.852497337757222 | 1.32973751456318 | 0.0343724130068181 | Singapore |
| 2021 | 1.24477105546 | 0.856097766112729 | 1.22733800672167 | 0.0174330487383334 | Singapore |
| 1990 | 4.0277426102 | 0.725982519157417 | 2.20590138834732 | 1.82184122185268 | Australia |
| 1991 | 3.50237718296 | 0.729759858781312 | 2.182714215569 | 1.319662967391 | Australia |
| 1992 | 3.07774232497 | 0.73390140434283 | 2.1585568579697 | 0.919185467000303 | Australia |
| 1993 | 2.46560561858 | 0.738374743085692 | 2.17220429470111 | 0.293401323878889 | Australia |
| 1994 | 2.23302523288 | 0.742846571880912 | 2.1091257242671 | 0.123899508612904 | Australia |
| 1995 | 2.04866043997 | 0.747426824081883 | 2.01085657214632 | 0.0378038678236843 | Australia |
| 1996 | 1.90408765898 | 0.751991736090285 | 1.90408765898 | 0 | Australia |
| 1997 | 1.54270201692 | 0.756633901347637 | 1.54270201692 | 0 | Australia |
| 1998 | 1.26737143346 | 0.761133265356106 | 1.26737143346 | 0 | Australia |
| 1999 | 1.40332968442 | 0.765697505930953 | 1.36786231460435 | 0.0354673698156522 | Australia |
| 2000 | 1.32566340686 | 0.770223843075912 | 1.31109041351 | 0.0145729933500001 | Australia |
| 2001 | 1.40108958543 | 0.774662787384155 | 1.36896859666947 | 0.0321209887605263 | Australia |
| 2002 | 1.51539657626 | 0.779360940006122 | 1.429396332432 | 0.0860002438279999 | Australia |
| 2003 | 1.67930646803 | 0.783600180802991 | 1.45400068466187 | 0.225305783368125 | Australia |
| 2004 | 1.87996890612 | 0.787016543130049 | 1.46617525660455 | 0.413793649515455 | Australia |
| 2005 | 1.92054121113 | 0.789615521287164 | 1.44737617262038 | 0.473165038509615 | Australia |
| 2006 | 2.05971319401 | 0.791155687742942 | 1.441531908898 | 0.618181285112 | Australia |
| 2007 | 2.06644556499 | 0.792532682754436 | 1.50158821072913 | 0.56485735426087 | Australia |
| 2008 | 1.89300959685 | 0.795292900277512 | 1.49357812835292 | 0.399431468497083 | Australia |
| 2009 | 2.06351259391 | 0.799174373217167 | 1.40798834911667 | 0.655524244793333 | Australia |
| 2010 | 1.86670461905 | 0.803561543809643 | 1.519965391006 | 0.346739228044 | Australia |
| 2011 | 2.02113904855 | 0.807520219913819 | 1.411840120552 | 0.609298927998 | Australia |
| 2012 | 2.02319178483 | 0.811644461488936 | 1.48247380563571 | 0.540717979194286 | Australia |
| 2013 | 1.85701925622 | 0.816453661448137 | 1.44626414960519 | 0.410755106614815 | Australia |
| 2014 | 1.91121495708 | 0.820840850321621 | 1.44758021081692 | 0.463634746263077 | Australia |
| 2015 | 1.8196884681 | 0.824860715173108 | 1.45158523101217 | 0.368103237087826 | Australia |
| 2016 | 1.64518863486 | 0.828559943182664 | 1.36968801813833 | 0.275500616721667 | Australia |
| 2017 | 1.58109542358 | 0.831798265550707 | 1.39296750952846 | 0.188127914051539 | Australia |
| 2018 | 1.47341525959 | 0.835354624190932 | 1.35817738101706 | 0.115237878572941 | Australia |
| 2019 | 1.46063997682 | 0.839317426059217 | 1.36146482659267 | 0.0991751502273335 | Australia |
| 2020 | 1.33807398512 | 0.842051314482691 | 1.31943384134952 | 0.0186401437704762 | Australia |
| 2021 | 1.10456965795 | 0.844252813727575 | 1.10456965795 | 0 | Australia |
| 1990 | 7.01625288115 | 0.752321654884833 | 2.01203040560605 | 5.00422247554395 | New Zealand |
| 1991 | 5.83489085515 | 0.755616090554557 | 2.03098392083758 | 3.80390693431242 | New Zealand |
| 1992 | 4.69318460839 | 0.759461372351944 | 1.85334617125167 | 2.83983843713833 | New Zealand |
| 1993 | 4.40116961619 | 0.763634604405382 | 1.75150313651857 | 2.64966647967143 | New Zealand |
| 1994 | 4.4375935438 | 0.767118657876439 | 1.71431841026957 | 2.72327513353043 | New Zealand |
| 1995 | 4.25696657273 | 0.769998036424639 | 1.68497415704829 | 2.57199241568171 | New Zealand |
| 1996 | 4.32962053906 | 0.773575283251135 | 1.591861771681 | 2.737758767379 | New Zealand |
| 1997 | 4.79877103467 | 0.778205776484177 | 1.54207742857395 | 3.25669360609605 | New Zealand |
| 1998 | 3.96463025069 | 0.78173075719617 | 1.50745283059667 | 2.45717742009333 | New Zealand |
| 1999 | 4.29948292238 | 0.784428637621383 | 1.56383540947362 | 2.73564751290638 | New Zealand |
| 2000 | 4.78927530678 | 0.787659557491679 | 1.42954752399116 | 3.35972778278884 | New Zealand |
| 2001 | 4.29444854709 | 0.791426079291696 | 1.4253803527641 | 2.8690681943259 | New Zealand |
| 2002 | 4.17845990141 | 0.794519590986489 | 1.46553864302564 | 2.71292125838436 | New Zealand |
| 2003 | 3.93242590462 | 0.796286883438684 | 1.48322706725419 | 2.44919883736581 | New Zealand |
| 2004 | 4.17403953556 | 0.798177476508371 | 1.45896016398095 | 2.71507937157905 | New Zealand |
| 2005 | 4.39126713535 | 0.799107294338124 | 1.4382526076069 | 2.9530145277431 | New Zealand |
| 2006 | 5.16666027859 | 0.798456865675295 | 1.46860212041971 | 3.69805815817029 | New Zealand |
| 2007 | 7.14565899208 | 0.798447615942533 | 1.43792136422937 | 5.70773762785063 | New Zealand |
| 2008 | 8.376469302 | 0.799669513648105 | 1.46058367993452 | 6.91588562206548 | New Zealand |
| 2009 | 9.59895877783 | 0.801727534577573 | 1.44907113711333 | 8.14988764071667 | New Zealand |
| 2010 | 9.06876988873 | 0.805133184751295 | 1.44496326012714 | 7.62380662860286 | New Zealand |
| 2011 | 9.0022661459 | 0.809760351959867 | 1.41253724806128 | 7.58972889783872 | New Zealand |
| 2012 | 8.8122970541 | 0.814957563823326 | 1.4393068828664 | 7.3729901712336 | New Zealand |
| 2013 | 7.66295872176 | 0.820145989650422 | 1.43059168034738 | 6.23236704141262 | New Zealand |
| 2014 | 8.19796738974 | 0.823908943687562 | 1.41430155400333 | 6.78366583573667 | New Zealand |
| 2015 | 6.57998601452 | 0.82746249334817 | 1.43159937870707 | 5.14838663581293 | New Zealand |
| 2016 | 5.62126328523 | 0.831843966678668 | 1.42351943278295 | 4.19774385244705 | New Zealand |
| 2017 | 5.87034730909 | 0.836301794613403 | 1.4294255196794 | 4.4409217894106 | New Zealand |
| 2018 | 5.13479960848 | 0.840597863184845 | 1.36404791053837 | 3.77075169794163 | New Zealand |
| 2019 | 5.28191597947 | 0.844533208807291 | 1.35686311339767 | 3.92505286607233 | New Zealand |
| 2020 | 4.44006192706 | 0.847398736836909 | 1.36660098391226 | 3.07346094314774 | New Zealand |
| 2021 | 4.58614019542 | 0.849442498970719 | 1.32408013998022 | 3.26206005543978 | New Zealand |
| 1990 | 6.66878901434 | 0.761463880346533 | 1.65453824163679 | 5.01425077270321 | Andorra |
| 1991 | 6.8412999979 | 0.764709071138693 | 1.77361993696652 | 5.06768006093348 | Andorra |
| 1992 | 7.10559976553 | 0.767188282487657 | 1.6158780310063 | 5.4897217345237 | Andorra |
| 1993 | 7.20369089025 | 0.768953827567811 | 1.56317242860605 | 5.64051846164395 | Andorra |
| 1994 | 7.34059611244 | 0.77002376454004 | 1.58883359858407 | 5.75176251385593 | Andorra |
| 1995 | 7.52348387813 | 0.77098868338163 | 1.55367340272745 | 5.96981047540255 | Andorra |
| 1996 | 7.71173563506 | 0.772403470644367 | 1.60648720608653 | 6.10524842897347 | Andorra |
| 1997 | 7.93441152451 | 0.774580970737089 | 1.4759210752 | 6.45849044931 | Andorra |
| 1998 | 8.19380742981 | 0.776972577800595 | 1.46257098236102 | 6.73123644744898 | Andorra |
| 1999 | 8.39006348085 | 0.779629634395052 | 1.50848158398794 | 6.88158189686206 | Andorra |
| 2000 | 8.53342701442 | 0.781976505693047 | 1.54035222560205 | 6.99307478881795 | Andorra |
| 2001 | 8.60502010419 | 0.785417034980389 | 1.46205670641321 | 7.14296339777679 | Andorra |
| 2002 | 8.71156025275 | 0.79015875913382 | 1.48027422258829 | 7.23128603016171 | Andorra |
| 2003 | 8.89689467755 | 0.797278900724701 | 1.46006404797083 | 7.43683062957917 | Andorra |
| 2004 | 9.0737033022 | 0.80473197995372 | 1.4709317224213 | 7.6027715797787 | Andorra |
| 2005 | 9.18758929033 | 0.812213979716634 | 1.441862221252 | 7.745727069078 | Andorra |
| 2006 | 9.26570398404 | 0.819787259850839 | 1.40677866723846 | 7.85892531680154 | Andorra |
| 2007 | 9.35317946961 | 0.826617254254178 | 1.43413392931842 | 7.91904554029158 | Andorra |
| 2008 | 9.41763181249 | 0.83182171450476 | 1.45732498399114 | 7.96030682849886 | Andorra |
| 2009 | 9.52317229792 | 0.835929886554712 | 1.39090553924783 | 8.13226675867217 | Andorra |
| 2010 | 9.51265652836 | 0.839489966459192 | 1.39630392389568 | 8.11635260446432 | Andorra |
| 2011 | 9.49694557174 | 0.842794802354863 | 1.3654012080013 | 8.1315443637387 | Andorra |
| 2012 | 9.44522864215 | 0.845810830539451 | 1.34305659885159 | 8.10217204329841 | Andorra |
| 2013 | 9.36601871822 | 0.848329909768827 | 1.35001825884892 | 8.01600045937108 | Andorra |
| 2014 | 9.256456963 | 0.851010491664786 | 1.33091472804021 | 7.92554223495979 | Andorra |
| 2015 | 9.14125769072 | 0.853591217830289 | 1.33096496909686 | 7.81029272162314 | Andorra |
| 2016 | 8.72176029618 | 0.856257052608717 | 1.29972582378069 | 7.42203447239931 | Andorra |
| 2017 | 7.87049486571 | 0.85947703010266 | 1.32547236808209 | 6.54502249762791 | Andorra |
| 2018 | 7.01516615663 | 0.862588950920789 | 1.25590713606472 | 5.75925902056528 | Andorra |
| 2019 | 6.55312048287 | 0.865621023596139 | 1.27624094751 | 5.27687953536 | Andorra |
| 2020 | 6.42778954219 | 0.867449169102346 | 1.29110751372222 | 5.13668202846778 | Andorra |
| 2021 | 6.24235066189 | 0.869444112735696 | 1.28419457697179 | 4.9581560849182 | Andorra |
| 1990 | 13.32564964353 | 0.749853692572025 | 2.05004508541853 | 11.2756045581115 | Austria |
| 1991 | 13.5716917718 | 0.751652734176599 | 2.04395474158904 | 11.527737030211 | Austria |
| 1992 | 13.82659957152 | 0.754375285612199 | 2.04212187607196 | 11.784477695448 | Austria |
| 1993 | 14.05818703847 | 0.758804553724473 | 1.89176576728769 | 12.1664212711823 | Austria |
| 1994 | 14.4195355858 | 0.764704364300466 | 1.7218511620718 | 12.6976844237282 | Austria |
| 1995 | 14.43489667907 | 0.769827471008921 | 1.59573029422741 | 12.8391663848426 | Austria |
| 1996 | 14.5188873874 | 0.774446565189292 | 1.52872199725375 | 12.9901653901462 | Austria |
| 1997 | 14.59030711154 | 0.77923285637593 | 1.45725871593429 | 13.1330483956057 | Austria |
| 1998 | 14.80845854 | 0.783521015501574 | 1.43479429515193 | 13.3736642448481 | Austria |
| 1999 | 15.0077473697 | 0.787451584183882 | 1.45420107271829 | 13.5535462969817 | Austria |
| 2000 | 15.57426889676 | 0.791847679870807 | 1.44973683613092 | 14.1245320606291 | Austria |
| 2001 | 16.01414897258 | 0.795770117733764 | 1.4706982392375 | 14.5434507333425 | Austria |
| 2002 | 16.56505279095 | 0.799154119574311 | 1.452313082891 | 15.112739708059 | Austria |
| 2003 | 17.43931167992 | 0.802043253134005 | 1.44610758609098 | 15.993204093829 | Austria |
| 2004 | 17.98455507801 | 0.804889143740108 | 1.45789555017104 | 16.526659527839 | Austria |
| 2005 | 18.41996373144 | 0.808419194340084 | 1.44590144251268 | 16.9740622889273 | Austria |
| 2006 | 18.21897547703 | 0.812064609467116 | 1.46293813516844 | 16.7560373418616 | Austria |
| 2007 | 17.38266090752 | 0.815480521576858 | 1.4389666698518 | 15.9436942376682 | Austria |
| 2008 | 15.96005899489 | 0.818793658319811 | 1.45381384790828 | 14.5062451469817 | Austria |
| 2009 | 14.35273575351 | 0.821541167154033 | 1.43345788976621 | 12.9192778637438 | Austria |
| 2010 | 13.09836634446 | 0.824500109333185 | 1.45133239079672 | 11.6470339536633 | Austria |
| 2011 | 11.75223796617 | 0.82800199897916 | 1.42706499791316 | 10.3251729682568 | Austria |
| 2012 | 10.64438909577 | 0.83111330933878 | 1.44327723006436 | 9.20111186570564 | Austria |
| 2013 | 9.59651831586 | 0.833679780262374 | 1.40585750492132 | 8.19066081093868 | Austria |
| 2014 | 8.75402257713 | 0.835838577321076 | 1.40542181928162 | 7.34860075784838 | Austria |
| 2015 | 8.14092041188 | 0.837579895310124 | 1.43217442159615 | 6.70874599028385 | Austria |
| 2016 | 7.57815588985 | 0.839883789894186 | 1.37369386745154 | 6.20446202239846 | Austria |
| 2017 | 7.11725350658 | 0.843173330746292 | 1.33690212795744 | 5.78035137862256 | Austria |
| 2018 | 6.50860208947 | 0.846614470307794 | 1.35986698950957 | 5.14873509996043 | Austria |
| 2019 | 6.16796828131 | 0.849862514846872 | 1.35513462425447 | 4.81283365705553 | Austria |
| 2020 | 5.91500675016 | 0.852020385174284 | 1.31539918231906 | 4.59960756784094 | Austria |
| 2021 | 5.68856976317 | 0.853837004256539 | 1.31156602653 | 4.37700373664 | Austria |
| 1990 | 11.74920085412 | 0.737390656400179 | 2.15239894833625 | 9.59680190578375 | Belgium |
| 1991 | 12.50230891678 | 0.74217307419685 | 2.08681089253778 | 10.4154980242422 | Belgium |
| 1992 | 13.12999386452 | 0.748012299186744 | 2.06819164373965 | 11.0618022207804 | Belgium |
| 1993 | 12.70900341741 | 0.753685087006788 | 2.0192225565193 | 10.6897808608907 | Belgium |
| 1994 | 12.21622779728 | 0.758903637592037 | 1.89322541762851 | 10.3230023796515 | Belgium |
| 1995 | 11.38541687912 | 0.763170400784407 | 1.65104003277911 | 9.73437684634089 | Belgium |
| 1996 | 10.81406339179 | 0.766826075213302 | 1.55720731760413 | 9.25685607418587 | Belgium |
| 1997 | 10.99830509277 | 0.770813953023256 | 1.56223387439023 | 9.43607121837977 | Belgium |
| 1998 | 11.62639165635 | 0.774228267511882 | 1.61732448937705 | 10.009067166973 | Belgium |
| 1999 | 12.15038579303 | 0.777041040124219 | 1.48376395594167 | 10.6666218370883 | Belgium |
| 2000 | 13.30565565006 | 0.780396331183289 | 1.42884802359213 | 11.8768076264679 | Belgium |
| 2001 | 13.86713888268 | 0.784713043367342 | 1.42803478009114 | 12.4391041025889 | Belgium |
| 2002 | 14.43965136835 | 0.788836054052762 | 1.42398899309563 | 13.0156623752544 | Belgium |
| 2003 | 15.35291064759 | 0.792014383577294 | 1.47508565003754 | 13.8778249975525 | Belgium |
| 2004 | 15.88734208816 | 0.795224935706338 | 1.45082949044224 | 14.4365125977178 | Belgium |
| 2005 | 17.64736974134 | 0.798497235786438 | 1.45773019312639 | 16.1896395482136 | Belgium |
| 2006 | 19.54052734625 | 0.801572762913827 | 1.47420043836509 | 18.0663269078849 | Belgium |
| 2007 | 20.87504957295 | 0.804612664215848 | 1.46713932829565 | 19.4079102446544 | Belgium |
| 2008 | 20.13247757313 | 0.807757944740662 | 1.46898211625525 | 18.6634954568748 | Belgium |
| 2009 | 19.38121815772 | 0.811055825478368 | 1.46049691346787 | 17.9207212442521 | Belgium |
| 2010 | 18.64222635485 | 0.815030206751301 | 1.46491778893678 | 17.1773085659132 | Belgium |
| 2011 | 16.53378495124 | 0.819470362048624 | 1.39277098715778 | 15.1410139640822 | Belgium |
| 2012 | 16.33061809846 | 0.824226016040103 | 1.40973641853288 | 14.9208816799271 | Belgium |
| 2013 | 15.62707651971 | 0.828619831627193 | 1.45519042857471 | 14.1718860911353 | Belgium |
| 2014 | 14.65837684738 | 0.832425051502658 | 1.38686831371561 | 13.2715085336644 | Belgium |
| 2015 | 14.11241289116 | 0.835878400447772 | 1.42154425110436 | 12.6908686400556 | Belgium |
| 2016 | 13.58435210875 | 0.839462940488534 | 1.407966695075 | 12.176385413675 | Belgium |
| 2017 | 14.26513133463 | 0.843212044031047 | 1.3761525580886 | 12.8889787765414 | Belgium |
| 2018 | 14.19157141864 | 0.846617625234845 | 1.36425402792409 | 12.8273173907159 | Belgium |
| 2019 | 13.47947595006 | 0.849498394807879 | 1.34599618960449 | 12.1334797604555 | Belgium |
| 2020 | 11.7713847165 | 0.851346648590241 | 1.30983318909137 | 10.4615515274086 | Belgium |
| 2021 | 11.65240782376 | 0.853654015777112 | 1.31305338719235 | 10.3393544365676 | Belgium |
| 1990 | 29.64926588893 | 0.648230871689669 | 5.69413933259209 | 23.9551265563379 | Cyprus |
| 1991 | 28.74705158458 | 0.655726169264618 | 4.97019687277329 | 23.7768547118067 | Cyprus |
| 1992 | 27.42581722988 | 0.666011849072818 | 4.31403733327258 | 23.1117798966074 | Cyprus |
| 1993 | 25.84385866994 | 0.677547476543954 | 2.905119191292 | 22.938739478648 | Cyprus |
| 1994 | 24.43697289398 | 0.688829360602013 | 2.43876132314881 | 21.9982115708312 | Cyprus |
| 1995 | 23.20833041474 | 0.699923898437876 | 2.44074267553246 | 20.7675877392075 | Cyprus |
| 1996 | 22.28387785312 | 0.709839946474019 | 2.2390305970564 | 20.0448472560636 | Cyprus |
| 1997 | 21.54013134812 | 0.718864243512848 | 2.22385205824536 | 19.3162792898746 | Cyprus |
| 1998 | 20.77231889591 | 0.727964381748969 | 2.17517107839727 | 18.5971478175127 | Cyprus |
| 1999 | 20.09060104854 | 0.736846134920381 | 2.16675506135906 | 17.9238459871809 | Cyprus |
| 2000 | 19.53521298775 | 0.745199300565367 | 2.08085117175741 | 17.4543618159926 | Cyprus |
| 2001 | 19.08071277809 | 0.753594717257543 | 2.01082598979154 | 17.0698867882985 | Cyprus |
| 2002 | 18.88017768587 | 0.76150246522841 | 1.75272777377431 | 17.1274499120957 | Cyprus |
| 2003 | 18.7269681447 | 0.767896215824759 | 1.74153019975161 | 16.9854379449484 | Cyprus |
| 2004 | 18.72594975219 | 0.77376477492064 | 1.54503767196 | 17.18091208023 | Cyprus |
| 2005 | 18.75574892273 | 0.779031361179798 | 1.49536156795135 | 17.2603873547787 | Cyprus |
| 2006 | 18.54646432506 | 0.784560964983202 | 1.48775802454596 | 17.058706300514 | Cyprus |
| 2007 | 18.30636665719 | 0.792068382747098 | 1.476157635216 | 16.830209021974 | Cyprus |
| 2008 | 18.06828728934 | 0.799454089093792 | 1.46888474437905 | 16.599402544961 | Cyprus |
| 2009 | 17.81193212762 | 0.805112085484562 | 1.44574031499125 | 16.3661918126287 | Cyprus |
| 2010 | 17.29912057613 | 0.809840187817991 | 1.43566505963 | 15.8634555165 | Cyprus |
| 2011 | 16.73519624524 | 0.813726132185002 | 1.43369682091516 | 15.3014994243248 | Cyprus |
| 2012 | 16.2006043169 | 0.816978525424299 | 1.46464874178379 | 14.7359555751162 | Cyprus |
| 2013 | 15.52116176031 | 0.819380601631525 | 1.435918628068 | 14.085243132242 | Cyprus |
| 2014 | 15.19534450371 | 0.821184302169431 | 1.42263108592971 | 13.7727134177803 | Cyprus |
| 2015 | 14.85147978535 | 0.822732850898765 | 1.45618881109712 | 13.3952909742529 | Cyprus |
| 2016 | 14.17133343889 | 0.824283624143557 | 1.41125407953301 | 12.760079359357 | Cyprus |
| 2017 | 13.07037722225 | 0.826139877968847 | 1.42737358925458 | 11.6430036329954 | Cyprus |
| 2018 | 12.10966149631 | 0.828343243063834 | 1.41539798932327 | 10.6942635069867 | Cyprus |
| 2019 | 11.46225292617 | 0.830980727806796 | 1.44180563416889 | 10.0204472920011 | Cyprus |
| 2020 | 11.02363420044 | 0.833059522677264 | 1.45091159472057 | 9.57272260571943 | Cyprus |
| 2021 | 10.8560993536 | 0.835630545002355 | 1.41345064892826 | 9.44264870467174 | Cyprus |
| 1990 | 12.6746924379 | 0.801154655014013 | 1.45332817491085 | 11.2213642629892 | Denmark |
| 1991 | 12.76558738687 | 0.804540349639536 | 1.46242825466235 | 11.3031591322076 | Denmark |
| 1992 | 12.50554039578 | 0.808164433760271 | 1.47853395241415 | 11.0270064433658 | Denmark |
| 1993 | 11.29949521008 | 0.811585712633093 | 1.42144237477462 | 9.87805283530538 | Denmark |
| 1994 | 11.35629495542 | 0.815138614432107 | 1.44493165834298 | 9.91136329707702 | Denmark |
| 1995 | 11.06450366521 | 0.819104611612713 | 1.42558272272744 | 9.63892094248256 | Denmark |
| 1996 | 11.10998332789 | 0.82337524309543 | 1.41857838659306 | 9.69140494129694 | Denmark |
| 1997 | 11.02256868364 | 0.827679979394938 | 1.44372650439628 | 9.57884217924372 | Denmark |
| 1998 | 11.08450756 | 0.831683237018017 | 1.42150438492766 | 9.66300317507234 | Denmark |
| 1999 | 11.2717377723 | 0.835381542910635 | 1.36780243827935 | 9.90393533402065 | Denmark |
| 2000 | 12.53518069304 | 0.8391842975615 | 1.40289303140197 | 11.132287661638 | Denmark |
| 2001 | 13.34743614425 | 0.843177750467757 | 1.36038635073016 | 11.9870497935198 | Denmark |
| 2002 | 13.87158470668 | 0.847086356838798 | 1.32780839582709 | 12.5437763108529 | Denmark |
| 2003 | 14.76871570763 | 0.850441325949586 | 1.33235422263204 | 13.436361484998 | Denmark |
| 2004 | 15.52000137233 | 0.853304103185433 | 1.32729423090207 | 14.1927071414279 | Denmark |
| 2005 | 16.60818081776 | 0.855690732240651 | 1.32867259973492 | 15.2795082180251 | Denmark |
| 2006 | 17.23592777843 | 0.858033209615474 | 1.32402980307436 | 15.9118979753556 | Denmark |
| 2007 | 19.25622450654 | 0.859781733794886 | 1.30747403586789 | 17.9487504706721 | Denmark |
| 2008 | 19.58246013552 | 0.861782061067554 | 1.29646678179702 | 18.285993353723 | Denmark |
| 2009 | 18.50289805721 | 0.86394804976016 | 1.27460084396673 | 17.2282972132433 | Denmark |
| 2010 | 18.07918451693 | 0.866767079164022 | 1.27564101675102 | 16.803543500179 | Denmark |
| 2011 | 16.68233714263 | 0.869778682281296 | 1.25484684287574 | 15.4274902997543 | Denmark |
| 2012 | 16.17694071453 | 0.872517510381731 | 1.28898593063723 | 14.8879547838928 | Denmark |
| 2013 | 16.29253887853 | 0.874932973936641 | 1.26904398027052 | 15.0234948982595 | Denmark |
| 2014 | 16.43136794828 | 0.876814931654032 | 1.26244575082308 | 15.1689221974569 | Denmark |
| 2015 | 16.06328670524 | 0.878543944867921 | 1.26463958768176 | 14.7986471175582 | Denmark |
| 2016 | 15.66767009176 | 0.8805897834924 | 1.2698276628087 | 14.3978424289513 | Denmark |
| 2017 | 15.7291118156 | 0.883688486994588 | 1.28022317263367 | 14.4488886429663 | Denmark |
| 2018 | 14.74256313031 | 0.887529846224258 | 1.2710894109344 | 13.4714737193756 | Denmark |
| 2019 | 13.15590061642 | 0.891624571480342 | 1.26858021615604 | 11.887320400264 | Denmark |
| 2020 | 12.6901246289 | 0.894368296843327 | 1.26284198407429 | 11.4272826448257 | Denmark |
| 2021 | 12.4951744845 | 0.896424204101212 | 1.26775918380408 | 11.2274153006959 | Denmark |
| 1990 | 7.19428640853 | 0.756221508893542 | 2.01063337776524 | 5.18365303076476 | Finland |
| 1991 | 7.41928401195 | 0.758410686787082 | 1.89694285987978 | 5.52234115207022 | Finland |
| 1992 | 7.55365113343 | 0.760804099620088 | 1.77648497965658 | 5.77716615377342 | Finland |
| 1993 | 7.46150000898 | 0.763526603650282 | 1.77371955968917 | 5.68778044929083 | Finland |
| 1994 | 7.56178238616 | 0.767101900863416 | 1.62410761553736 | 5.93767477062264 | Finland |
| 1995 | 7.66642518075 | 0.771163742219649 | 1.56167137921048 | 6.10475380153952 | Finland |
| 1996 | 7.80094284316 | 0.774809058593956 | 1.50658739296333 | 6.29435545019667 | Finland |
| 1997 | 7.99423405632 | 0.778814214501226 | 1.53246007981286 | 6.46177397650714 | Finland |
| 1998 | 8.22199968701 | 0.782305059190857 | 1.51212357281028 | 6.70987611419972 | Finland |
| 1999 | 8.45040735125 | 0.78577398760406 | 1.47101405262425 | 6.97939329862575 | Finland |
| 2000 | 8.82990367729 | 0.78998522981789 | 1.45973448370488 | 7.37016919358512 | Finland |
| 2001 | 9.07641474157 | 0.79445383485974 | 1.43870695305442 | 7.63770778851558 | Finland |
| 2002 | 9.509531344 | 0.798770274163901 | 1.48170507798842 | 8.02782626601158 | Finland |
| 2003 | 10.12384605557 | 0.802325430617225 | 1.45645302410412 | 8.66739303146588 | Finland |
| 2004 | 10.62783640109 | 0.805694227411326 | 1.49157000536935 | 9.13626639572065 | Finland |
| 2005 | 10.99197546085 | 0.808936787277645 | 1.44394606467667 | 9.54802939617333 | Finland |
| 2006 | 11.15024432335 | 0.812081481875967 | 1.41604568028778 | 9.73419864306222 | Finland |
| 2007 | 11.36187613713 | 0.815597613277792 | 1.46825331931391 | 9.89362281781609 | Finland |
| 2008 | 11.32662145166 | 0.818875543929948 | 1.46769748552629 | 9.85892396613371 | Finland |
| 2009 | 11.03412972297 | 0.821457587971858 | 1.40985515592103 | 9.62427456704897 | Finland |
| 2010 | 10.74300517588 | 0.824844982269378 | 1.43085618736022 | 9.31214898851978 | Finland |
| 2011 | 10.17522595931 | 0.82827057673312 | 1.43545805933526 | 8.73976789997474 | Finland |
| 2012 | 9.60458849611 | 0.831434483518693 | 1.42001508083735 | 8.18457341527265 | Finland |
| 2013 | 8.67355943942 | 0.834425400661261 | 1.42492603082902 | 7.24863340859098 | Finland |
| 2014 | 8.14891796631 | 0.837431871657021 | 1.41528905585437 | 6.73362891045563 | Finland |
| 2015 | 7.35244346537 | 0.840878476913666 | 1.39753572322551 | 5.95490774214449 | Finland |
| 2016 | 6.86293839539 | 0.844622338388364 | 1.34925646447083 | 5.51368193091917 | Finland |
| 2017 | 6.54547910394 | 0.848586127623286 | 1.35625649299179 | 5.1892226109482 | Finland |
| 2018 | 6.14682252629 | 0.852202943239694 | 1.35262595382132 | 4.79419657246868 | Finland |
| 2019 | 5.87193411621 | 0.855365178519151 | 1.3424114077122 | 4.52952270849781 | Finland |
| 2020 | 5.62275068048 | 0.857655552665083 | 1.31728454033636 | 4.30546614014364 | Finland |
| 2021 | 5.57664559354 | 0.859831367662218 | 1.31292841331861 | 4.26371718022139 | Finland |
| 1990 | 12.84819936666 | 0.730747465817975 | 2.1577458719351 | 10.6904534947249 | France |
| 1991 | 13.69539884383 | 0.736452382335769 | 2.15278711985316 | 11.5426117239768 | France |
| 1992 | 14.32500438154 | 0.742871973277562 | 2.09235504478957 | 12.2326493367504 | France |
| 1993 | 14.57117570876 | 0.748582326771669 | 2.04011093240079 | 12.5310647763592 | France |
| 1994 | 13.78989669211 | 0.753176131807871 | 2.02340759493611 | 11.7664890971739 | France |
| 1995 | 13.07693559516 | 0.75712578885604 | 1.9701546176196 | 11.1067809775404 | France |
| 1996 | 13.20210592101 | 0.760899578763994 | 1.81792484600525 | 11.3841810750048 | France |
| 1997 | 13.14456209795 | 0.764650672066045 | 1.69193111678563 | 11.4526309811644 | France |
| 1998 | 13.57658193202 | 0.767852560919953 | 1.63477367401583 | 11.9418082580042 | France |
| 1999 | 13.86600709372 | 0.770304362540569 | 1.55456878794022 | 12.3114383057798 | France |
| 2000 | 14.36780219352 | 0.772792033272814 | 1.53036567470075 | 12.8374365188192 | France |
| 2001 | 14.67645824708 | 0.776225157250438 | 1.55216197990538 | 13.1242962671746 | France |
| 2002 | 14.05196685845 | 0.780088871976379 | 1.40020813420071 | 12.6517587242493 | France |
| 2003 | 14.72089590843 | 0.783178757362352 | 1.51329679414474 | 13.2075991142853 | France |
| 2004 | 14.89669876554 | 0.786084654751087 | 1.4618962577612 | 13.4348025077788 | France |
| 2005 | 15.06286442159 | 0.789042086328118 | 1.48106203314074 | 13.5818023884493 | France |
| 2006 | 15.96150209596 | 0.792321919625469 | 1.43233336551 | 14.52916873045 | France |
| 2007 | 16.54390061407 | 0.795557651447132 | 1.44062701939357 | 15.1032735946764 | France |
| 2008 | 17.07991742522 | 0.798177429586454 | 1.44734379333123 | 15.6325736318888 | France |
| 2009 | 16.96054408713 | 0.800508669748406 | 1.50353123354545 | 15.4570128535845 | France |
| 2010 | 16.39405382669 | 0.803240246888394 | 1.4750509137318 | 14.9190029129582 | France |
| 2011 | 14.9567044136 | 0.806378738306578 | 1.44614691785426 | 13.5105574957457 | France |
| 2012 | 14.9899684755 | 0.809615388940868 | 1.4238866821573 | 13.5660817933427 | France |
| 2013 | 15.14454809265 | 0.812984015183301 | 1.42768777409148 | 13.7168603185585 | France |
| 2014 | 15.0046218316 | 0.816551447578728 | 1.48085551553491 | 13.5237663160651 | France |
| 2015 | 15.24258324946 | 0.820238385480897 | 1.4277819047504 | 13.8148013447096 | France |
| 2016 | 14.27267499161 | 0.82373092859985 | 1.4301470573002 | 12.8425279343098 | France |
| 2017 | 13.9508125232 | 0.827098440641564 | 1.433328570507 | 12.517483952693 | France |
| 2018 | 13.70088804585 | 0.830448590187259 | 1.44429950873262 | 12.2565885371174 | France |
| 2019 | 13.22087953567 | 0.833772804699999 | 1.41003446557635 | 11.8108450700937 | France |
| 2020 | 12.48277057592 | 0.836049443246129 | 1.40860783081292 | 11.0741627451071 | France |
| 2021 | 12.17263536523 | 0.838364875196954 | 1.39170554162146 | 10.7809298236085 | France |
| 1990 | 9.34496908195 | 0.817077665790386 | 1.43547272614585 | 7.90949635580415 | Germany |
| 1991 | 10.06146763934 | 0.823339898340462 | 1.41718854780125 | 8.64427909153875 | Germany |
| 1992 | 10.55711854412 | 0.828323503378164 | 1.3955698824883 | 9.1615486616317 | Germany |
| 1993 | 10.9199176896 | 0.832286809367201 | 1.41530602889818 | 9.50461166070182 | Germany |
| 1994 | 11.23610170173 | 0.835561963052536 | 1.40559401836265 | 9.83050768336735 | Germany |
| 1995 | 11.49925304643 | 0.837643151522198 | 1.4017485672069 | 10.0975044792231 | Germany |
| 1996 | 11.60857984227 | 0.839188129123403 | 1.40394295346833 | 10.2046368888017 | Germany |
| 1997 | 11.86685662858 | 0.841385902988361 | 1.39718988837941 | 10.4696667402006 | Germany |
| 1998 | 12.04405471359 | 0.843854978100034 | 1.33577481629446 | 10.7082798972955 | Germany |
| 1999 | 12.21778034164 | 0.846232398245621 | 1.3965572996625 | 10.8212230419775 | Germany |
| 2000 | 12.69694849944 | 0.848925756438545 | 1.37226806959094 | 11.3246804298491 | Germany |
| 2001 | 13.09549658761 | 0.852070412843074 | 1.33865287869703 | 11.756843708913 | Germany |
| 2002 | 13.59327630203 | 0.855066792837203 | 1.3431282056549 | 12.2501480963751 | Germany |
| 2003 | 14.40913433486 | 0.857675790269356 | 1.29520804272289 | 13.1139262921371 | Germany |
| 2004 | 15.02581211143 | 0.860240332885484 | 1.2912555288681 | 13.7345565825619 | Germany |
| 2005 | 15.78302958576 | 0.862894178369357 | 1.27428777086386 | 14.5087418148961 | Germany |
| 2006 | 16.90391574299 | 0.865873706890654 | 1.2929450895569 | 15.6109706534331 | Germany |
| 2007 | 18.27253583031 | 0.869140440024023 | 1.2527448040087 | 17.0197910263013 | Germany |
| 2008 | 18.49410619552 | 0.872552849301022 | 1.25181196822776 | 17.2422942272922 | Germany |
| 2009 | 18.68988631888 | 0.875087911282131 | 1.29059180515056 | 17.3992945137294 | Germany |
| 2010 | 18.38412584564 | 0.877946710427414 | 1.27744881773269 | 17.1066770279073 | Germany |
| 2011 | 17.20853175911 | 0.881027507503078 | 1.23767107199904 | 15.970860687111 | Germany |
| 2012 | 15.83819649355 | 0.88365640827103 | 1.25420530773017 | 14.5839911858198 | Germany |
| 2013 | 14.92543141742 | 0.885926953064586 | 1.25295936114276 | 13.6724720562772 | Germany |
| 2014 | 13.66388293895 | 0.887651614379013 | 1.26113853198926 | 12.4027444069607 | Germany |
| 2015 | 13.03389950633 | 0.888867853966898 | 1.25571818297442 | 11.7781813233556 | Germany |
| 2016 | 12.26189289139 | 0.89089636945676 | 1.27967829192132 | 10.9822145994687 | Germany |
| 2017 | 10.88381796701 | 0.894050472424158 | 1.264335604895 | 9.619482362115 | Germany |
| 2018 | 9.63039225332 | 0.896975545420426 | 1.26774011297062 | 8.36265214034938 | Germany |
| 2019 | 7.99848820311 | 0.899703158084962 | 1.25585144641561 | 6.74263675669439 | Germany |
| 2020 | 7.0946083017 | 0.901438614019463 | 1.25007309425981 | 5.84453520744019 | Germany |
| 2021 | 6.7323680175 | 0.902957091057572 | 1.26177989246683 | 5.47058812503317 | Germany |
| 1990 | 2.6778200076 | 0.67418646507665 | 2.52851973957737 | 0.149300268022631 | Greece |
| 1991 | 2.69681865367 | 0.680316480101809 | 2.49185559768857 | 0.204963055981429 | Greece |
| 1992 | 2.73729795718 | 0.687055389369745 | 2.49524660147868 | 0.242051355701316 | Greece |
| 1993 | 2.77213513982 | 0.69332892644804 | 2.38631434011937 | 0.385820799700625 | Greece |
| 1994 | 2.82026895265 | 0.699153022352574 | 2.31180810048238 | 0.50846085216762 | Greece |
| 1995 | 2.86792702876 | 0.704862873096307 | 2.29529860823611 | 0.572628420523889 | Greece |
| 1996 | 2.93218740707 | 0.710139335918529 | 2.22257080821235 | 0.709616598857647 | Greece |
| 1997 | 3.01424845773 | 0.715687267664404 | 2.18461959801256 | 0.829628859717436 | Greece |
| 1998 | 3.12291198469 | 0.721593982464726 | 2.19601769576027 | 0.92689428892973 | Greece |
| 1999 | 3.22493786536 | 0.727529454753368 | 2.18270386014829 | 1.04223400521171 | Greece |
| 2000 | 3.3283167082 | 0.732710539506218 | 2.15374710747514 | 1.17456960072486 | Greece |
| 2001 | 3.43302667825 | 0.737154008101854 | 2.18492082438655 | 1.24810585386345 | Greece |
| 2002 | 3.56870255013 | 0.741945221017726 | 2.09559239268297 | 1.47311015744703 | Greece |
| 2003 | 3.6807553142 | 0.747185955887149 | 2.06327207073364 | 1.61748324346636 | Greece |
| 2004 | 3.81579616083 | 0.752418606565094 | 2.03331082313833 | 1.78248533769167 | Greece |
| 2005 | 3.91874763745 | 0.75638439564551 | 1.99010668827071 | 1.92864094917929 | Greece |
| 2006 | 4.04152898725 | 0.760209695750845 | 1.91768388229976 | 2.12384510495024 | Greece |
| 2007 | 4.15275861 | 0.763927173080314 | 1.78598125128 | 2.36677735872 | Greece |
| 2008 | 4.1910502627 | 0.767527657538734 | 1.60148458522575 | 2.58956567747425 | Greece |
| 2009 | 4.26355995874 | 0.771247355848723 | 1.6382490874627 | 2.6253108712773 | Greece |
| 2010 | 4.29547765959 | 0.775155814653294 | 1.55643056803696 | 2.73904709155304 | Greece |
| 2011 | 4.20844075416 | 0.778525866909133 | 1.56847717242788 | 2.63996358173212 | Greece |
| 2012 | 4.14509719183 | 0.780789482881484 | 1.49967387994615 | 2.64542331188385 | Greece |
| 2013 | 4.12399648614 | 0.781949051742451 | 1.5318111034216 | 2.5921853827184 | Greece |
| 2014 | 4.07397550138 | 0.782442012668796 | 1.48037946537185 | 2.59359603600815 | Greece |
| 2015 | 3.98813969609 | 0.782489523445532 | 1.50124754253594 | 2.48689215355406 | Greece |
| 2016 | 3.77743260645 | 0.782798442836071 | 1.47156187562 | 2.30587073083 | Greece |
| 2017 | 3.35798879884 | 0.784108593896727 | 1.43637454944694 | 1.92161424939306 | Greece |
| 2018 | 2.97431174267 | 0.785999752836636 | 1.50723544791714 | 1.46707629475286 | Greece |
| 2019 | 2.74501389081 | 0.788138138237775 | 1.49621483995154 | 1.24879905085846 | Greece |
| 2020 | 2.57168331958 | 0.789632219998033 | 1.4475573051571 | 1.1241260144229 | Greece |
| 2021 | 2.43838344828 | 0.791854407912829 | 1.43707052260189 | 1.00131292567811 | Greece |
| 1990 | 7.27996583308 | 0.764212516960703 | 1.7617455537639 | 5.5182202793161 | Iceland |
| 1991 | 7.48365133545 | 0.769027393143331 | 1.58183884865396 | 5.90181248679604 | Iceland |
| 1992 | 7.72458424631 | 0.772706414740194 | 1.57654239474464 | 6.14804185156536 | Iceland |
| 1993 | 7.81744296516 | 0.776389422872018 | 1.53721801994875 | 6.28022494521125 | Iceland |
| 1994 | 7.95069744904 | 0.77996796073086 | 1.50270834384918 | 6.44798910519082 | Iceland |
| 1995 | 8.10229670351 | 0.782740057259954 | 1.48677365286167 | 6.61552305064833 | Iceland |
| 1996 | 8.39919749028 | 0.785356419811944 | 1.52223318814053 | 6.87696430213947 | Iceland |
| 1997 | 8.40893974016 | 0.78852833520489 | 1.45427019018611 | 6.95466954997389 | Iceland |
| 1998 | 8.59241563484 | 0.792626368445487 | 1.49510145793553 | 7.09731417690447 | Iceland |
| 1999 | 8.84934763899 | 0.797368807995672 | 1.42568661107444 | 7.42366102791556 | Iceland |
| 2000 | 9.18339580039 | 0.803030330314896 | 1.46087840024143 | 7.72251740014857 | Iceland |
| 2001 | 9.48757330834 | 0.808965785947657 | 1.45274893335745 | 8.03482437498255 | Iceland |
| 2002 | 9.78240923338 | 0.813400392129504 | 1.45618658166804 | 8.32622265171196 | Iceland |
| 2003 | 10.17950105934 | 0.816597066979147 | 1.46467290712317 | 8.71482815221683 | Iceland |
| 2004 | 10.62207352025 | 0.819528086895788 | 1.43252019791154 | 9.18955332233846 | Iceland |
| 2005 | 11.07330132504 | 0.822766536023531 | 1.40742348739854 | 9.66587783764146 | Iceland |
| 2006 | 11.67352123156 | 0.82624474349372 | 1.46232516442273 | 10.2111960671373 | Iceland |
| 2007 | 12.2053744791 | 0.830289889861998 | 1.42760906088017 | 10.7777654182198 | Iceland |
| 2008 | 12.42656449948 | 0.834622678087519 | 1.42960826833512 | 10.9969562311449 | Iceland |
| 2009 | 12.40953065597 | 0.838121013089424 | 1.3994670276694 | 11.0100636283006 | Iceland |
| 2010 | 12.28978989266 | 0.841384642074063 | 1.38956580367491 | 10.9002240889851 | Iceland |
| 2011 | 11.80063888444 | 0.844619929908845 | 1.37765430118919 | 10.4229845832508 | Iceland |
| 2012 | 11.43738785964 | 0.84729944372556 | 1.36359326994323 | 10.0737945896968 | Iceland |
| 2013 | 11.19826330096 | 0.850173947991231 | 1.34342456120095 | 9.85483873975905 | Iceland |
| 2014 | 10.99918987952 | 0.853558696790214 | 1.33244364666182 | 9.66674623285818 | Iceland |
| 2015 | 10.84785873566 | 0.857724976230652 | 1.3050034937236 | 9.5428552419364 | Iceland |
| 2016 | 10.35439735879 | 0.861844466505036 | 1.28895345812021 | 9.06544390066979 | Iceland |
| 2017 | 9.61009406978 | 0.865702890708071 | 1.25638840982979 | 8.35370565995021 | Iceland |
| 2018 | 8.71449650539 | 0.869219821760483 | 1.282262078847 | 7.432234426543 | Iceland |
| 2019 | 7.98307313587 | 0.872425580161805 | 1.27382696917333 | 6.70924616669667 | Iceland |
| 2020 | 7.77270143575 | 0.874323153425611 | 1.28575975555814 | 6.48694168019186 | Iceland |
| 2021 | 7.6699628181 | 0.876361679802766 | 1.27328122520571 | 6.39668159289429 | Iceland |
| 1990 | 6.34672531478 | 0.719891818965305 | 2.2090523461181 | 4.1376729686619 | Ireland |
| 1991 | 6.35295546606 | 0.725195018704741 | 2.16283523610235 | 4.19012022995765 | Ireland |
| 1992 | 6.39981612029 | 0.731918691315293 | 2.18316410123136 | 4.21665201905864 | Ireland |
| 1993 | 6.46205722438 | 0.738384073791795 | 2.14721970903175 | 4.31483751534825 | Ireland |
| 1994 | 6.44392260566 | 0.744011727859478 | 2.07660653457576 | 4.36731607108424 | Ireland |
| 1995 | 6.61717052529 | 0.748921575045051 | 2.06088189702122 | 4.55628862826878 | Ireland |
| 1996 | 6.77481872456 | 0.754010123608321 | 2.04035764150911 | 4.73446108305089 | Ireland |
| 1997 | 7.02450377613 | 0.759970383842882 | 1.83729696117609 | 5.18720681495391 | Ireland |
| 1998 | 7.27954756284 | 0.766488720882709 | 1.65998171678949 | 5.61956584605051 | Ireland |
| 1999 | 7.65269115104 | 0.773538834439684 | 1.60201993110036 | 6.05067121993964 | Ireland |
| 2000 | 8.04507057538 | 0.780190655170267 | 1.45672636813216 | 6.58834420724784 | Ireland |
| 2001 | 8.4460281258 | 0.786353740708812 | 1.47144715163667 | 6.97458097416333 | Ireland |
| 2002 | 8.81800472887 | 0.793010605962827 | 1.48961209706048 | 7.32839263180952 | Ireland |
| 2003 | 9.37032072601 | 0.800005865377216 | 1.4462783811405 | 7.9240423448695 | Ireland |
| 2004 | 9.67596915316 | 0.806853140411776 | 1.43435656330667 | 8.24161258985333 | Ireland |
| 2005 | 10.05382132476 | 0.811713818388319 | 1.42479490293273 | 8.62902642182727 | Ireland |
| 2006 | 10.81215173006 | 0.814505479095719 | 1.4175546269 | 9.39459710316 | Ireland |
| 2007 | 11.67056648476 | 0.816931026031536 | 1.45970444123256 | 10.2108620435274 | Ireland |
| 2008 | 12.46726846124 | 0.819932128191974 | 1.43026837679475 | 11.0370000844453 | Ireland |
| 2009 | 12.82601887369 | 0.823929666301939 | 1.42864420710358 | 11.3973746665864 | Ireland |
| 2010 | 13.08203505594 | 0.828103848001623 | 1.41647521778968 | 11.6655598381503 | Ireland |
| 2011 | 12.9659332499 | 0.83180479150489 | 1.41329430749667 | 11.5526389424033 | Ireland |
| 2012 | 13.30805081078 | 0.835325358632175 | 1.42413877740328 | 11.8839120333767 | Ireland |
| 2013 | 13.81873444211 | 0.838644432713483 | 1.4015944037712 | 12.4171400383388 | Ireland |
| 2014 | 14.09676106623 | 0.842082114682265 | 1.39880380379327 | 12.6979572624367 | Ireland |
| 2015 | 14.23623121142 | 0.847437886654412 | 1.33725810235982 | 12.8989731090602 | Ireland |
| 2016 | 13.27468196776 | 0.85264250194342 | 1.32023858268421 | 11.9544433850758 | Ireland |
| 2017 | 11.28859340393 | 0.857816163802237 | 1.29108490581087 | 9.99750849811913 | Ireland |
| 2018 | 9.21627444713 | 0.863925284412195 | 1.27533601215488 | 7.94093843497512 | Ireland |
| 2019 | 7.75628763498 | 0.869412503348037 | 1.25502092270158 | 6.50126671227842 | Ireland |
| 2020 | 6.82891994965 | 0.871958822388357 | 1.26165375843881 | 5.56726619121119 | Ireland |
| 2021 | 6.71827017157 | 0.873753849787408 | 1.2948664635304 | 5.4234037080396 | Ireland |
| 1990 | 19.34932084629 | 0.709178347365152 | 2.20972370255564 | 17.1395971437344 | Israel |
| 1991 | 19.80610700856 | 0.713371633399082 | 2.20282813511725 | 17.6032788734428 | Israel |
| 1992 | 21.75676605028 | 0.717897923179142 | 2.22775112123968 | 19.5290149290403 | Israel |
| 1993 | 20.66715919204 | 0.72225017554813 | 2.18321885153062 | 18.4839403405094 | Israel |
| 1994 | 19.45724500132 | 0.726654735721658 | 2.18345549348967 | 17.2737895078303 | Israel |
| 1995 | 18.43674103835 | 0.731127859056384 | 2.16012570898574 | 16.2766153293643 | Israel |
| 1996 | 18.29788264243 | 0.734714025875415 | 2.16790254970667 | 16.1299800927233 | Israel |
| 1997 | 18.7210902094 | 0.738015276501763 | 2.16227373223768 | 16.5588164771623 | Israel |
| 1998 | 19.61108866245 | 0.741927189995428 | 2.07471018795928 | 17.5363784744907 | Israel |
| 1999 | 20.64391633218 | 0.745443338031474 | 2.06956287678032 | 18.5743534553997 | Israel |
| 2000 | 22.12434925664 | 0.749184084123824 | 2.03320444526623 | 20.0911448113738 | Israel |
| 2001 | 22.54118221478 | 0.752834296773304 | 2.01694387001619 | 20.5242383447638 | Israel |
| 2002 | 24.20767563502 | 0.755988075842232 | 1.97022652338578 | 22.2374491116342 | Israel |
| 2003 | 25.91440452256 | 0.758875408886722 | 1.88661212221208 | 24.0277924003479 | Israel |
| 2004 | 26.8478914867 | 0.762213291396151 | 1.66503631150277 | 25.1828551751972 | Israel |
| 2005 | 28.55950422217 | 0.765543461435524 | 1.76530861057373 | 26.7941956115963 | Israel |
| 2006 | 31.79364774761 | 0.768733897655938 | 1.65959929070033 | 30.1340484569097 | Israel |
| 2007 | 33.29567960547 | 0.770756143247979 | 1.61626520804736 | 31.6794143974226 | Israel |
| 2008 | 33.09413764979 | 0.772011614541036 | 1.5468298913 | 31.54730775849 | Israel |
| 2009 | 31.56728688149 | 0.773303913379524 | 1.59976558201328 | 29.9675212994767 | Israel |
| 2010 | 30.30805745743 | 0.775259530065334 | 1.57737783013525 | 28.7306796272947 | Israel |
| 2011 | 27.75081571351 | 0.77764966916418 | 1.54149657813934 | 26.2093191353707 | Israel |
| 2012 | 26.49003460844 | 0.780403414638078 | 1.4527592327958 | 25.0372753756442 | Israel |
| 2013 | 25.04305775309 | 0.783267166608914 | 1.47531648631095 | 23.5677412667791 | Israel |
| 2014 | 23.62945064549 | 0.78615108379553 | 1.466356239735 | 22.163094405755 | Israel |
| 2015 | 22.87422798597 | 0.788874456945394 | 1.46259545836585 | 21.4116325276042 | Israel |
| 2016 | 22.28434173309 | 0.791772445367912 | 1.44500270751032 | 20.8393390255797 | Israel |
| 2017 | 22.30561655058 | 0.795173437476151 | 1.48162219351157 | 20.8239943570684 | Israel |
| 2018 | 21.03262866901 | 0.799124993659694 | 1.40347446872679 | 19.6291542002832 | Israel |
| 2019 | 19.95730073211 | 0.803232990528098 | 1.49535150653953 | 18.4619492255705 | Israel |
| 2020 | 16.24744280926 | 0.806351589333701 | 1.4501646016381 | 14.7972782076219 | Israel |
| 2021 | 13.75495144668 | 0.809011651557631 | 1.4359099618375 | 12.3190414848425 | Israel |
| 1990 | 5.43715849543 | 0.706255223740403 | 2.29087680455 | 3.14628169088 | Italy |
| 1991 | 5.19760220157 | 0.711457348523815 | 2.26387862137125 | 2.93372358019875 | Italy |
| 1992 | 5.15809748232 | 0.716649746121184 | 2.26085479227618 | 2.89724269004382 | Italy |
| 1993 | 4.87017658531 | 0.721720992266354 | 2.24531327065811 | 2.62486331465189 | Italy |
| 1994 | 4.54329209222 | 0.726870913262732 | 2.18758144931206 | 2.35571064290794 | Italy |
| 1995 | 4.24324949774 | 0.731644081628044 | 2.15746350910289 | 2.08578598863711 | Italy |
| 1996 | 4.37527732613 | 0.736050870453315 | 2.16048671575783 | 2.21479061037217 | Italy |
| 1997 | 4.59868570517 | 0.739972336484864 | 2.05377867704053 | 2.54490702812947 | Italy |
| 1998 | 4.79989383724 | 0.743101595998998 | 2.10604549267312 | 2.69384834456688 | Italy |
| 1999 | 5.07981534288 | 0.746074084421097 | 2.04890494049167 | 3.03091040238833 | Italy |
| 2000 | 5.3537583352 | 0.749675986238207 | 2.04539280250687 | 3.30836553269313 | Italy |
| 2001 | 5.59624119374 | 0.753554839457876 | 2.0086210957925 | 3.5876200979475 | Italy |
| 2002 | 5.55959838111 | 0.757191479404768 | 1.97798011430717 | 3.58161826680283 | Italy |
| 2003 | 5.85196339459 | 0.76009374379023 | 1.89923978116784 | 3.95272361342216 | Italy |
| 2004 | 6.04614765527 | 0.762787749063442 | 1.63459763792702 | 4.41155001734298 | Italy |
| 2005 | 6.17459912062 | 0.765689914012792 | 1.75067482277972 | 4.42392429784028 | Italy |
| 2006 | 6.19456328761 | 0.768402407690909 | 1.63491727537302 | 4.55964601223698 | Italy |
| 2007 | 6.39002108008 | 0.770698380337301 | 1.54316293765098 | 4.84685814242902 | Italy |
| 2008 | 6.78953905644 | 0.773068469998338 | 1.60013740829647 | 5.18940164814353 | Italy |
| 2009 | 7.17490472033 | 0.775499262002478 | 1.5127389957249 | 5.6621657246051 | Italy |
| 2010 | 7.59098147716 | 0.778109205917719 | 1.51672010799361 | 6.07426136916639 | Italy |
| 2011 | 7.85625027024 | 0.780703467869893 | 1.54319511750943 | 6.31305515273057 | Italy |
| 2012 | 8.05823564851 | 0.783090981066118 | 1.50425818554956 | 6.55397746296044 | Italy |
| 2013 | 7.96073712596 | 0.785356877137869 | 1.50470610958094 | 6.45603101637906 | Italy |
| 2014 | 8.07733712423 | 0.787533956365705 | 1.47744225226944 | 6.59989487196056 | Italy |
| 2015 | 8.38324543798 | 0.789902118846972 | 1.46650563233679 | 6.91673980564321 | Italy |
| 2016 | 9.00588129559 | 0.792477244295977 | 1.43111310973595 | 7.57476818585405 | Italy |
| 2017 | 9.07216307869 | 0.795311186445406 | 1.454209964302 | 7.617953114388 | Italy |
| 2018 | 8.95402920476 | 0.798362690372083 | 1.4451517817282 | 7.5088774230318 | Italy |
| 2019 | 7.56028925962 | 0.801530020572533 | 1.47289999658263 | 6.08738926303737 | Italy |
| 2020 | 7.06796871239 | 0.803635679966778 | 1.43801286634125 | 5.62995584604875 | Italy |
| 2021 | 6.78871789374 | 0.805773533613512 | 1.47395761304133 | 5.31476028069867 | Italy |
| 1990 | 9.61505059978 | 0.781051609326011 | 1.49180820289553 | 8.12324239688447 | Luxembourg |
| 1991 | 10.10541185662 | 0.785961442083159 | 1.49760517183 | 8.60780668479 | Luxembourg |
| 1992 | 10.09255086922 | 0.789053363703187 | 1.46018867547326 | 8.63236219374674 | Luxembourg |
| 1993 | 9.62616308642 | 0.792843836314663 | 1.45079046674703 | 8.17537261967297 | Luxembourg |
| 1994 | 9.40331701693 | 0.797824008094164 | 1.48012158306692 | 7.92319543386308 | Luxembourg |
| 1995 | 8.86493299298 | 0.802682469757881 | 1.47258515195441 | 7.39234784102559 | Luxembourg |
| 1996 | 9.20771005657 | 0.80651613780194 | 1.4240533280608 | 7.7836567285092 | Luxembourg |
| 1997 | 9.05871528789 | 0.810235288443831 | 1.44562699530471 | 7.61308829258529 | Luxembourg |
| 1998 | 9.77880897974 | 0.813889026223085 | 1.42221051089026 | 8.35659846884974 | Luxembourg |
| 1999 | 9.76780867353 | 0.817570995147661 | 1.47928792686105 | 8.28852074666895 | Luxembourg |
| 2000 | 9.88355407232 | 0.822006568181537 | 1.45152403691844 | 8.43203003540156 | Luxembourg |
| 2001 | 11.33786306915 | 0.825312816180712 | 1.41530835565 | 9.9225547135 | Luxembourg |
| 2002 | 11.7567503518 | 0.828223491084071 | 1.43408997500533 | 10.3226603767947 | Luxembourg |
| 2003 | 12.22139063834 | 0.830983945726346 | 1.41891202409796 | 10.802478614242 | Luxembourg |
| 2004 | 12.18695414685 | 0.833672567288171 | 1.41452008516255 | 10.7724340616875 | Luxembourg |
| 2005 | 12.20545967115 | 0.836675263504864 | 1.40336451290863 | 10.8020951582414 | Luxembourg |
| 2006 | 12.61884217865 | 0.840574453017841 | 1.38138436882163 | 11.2374578098284 | Luxembourg |
| 2007 | 13.51140787161 | 0.84470700603675 | 1.38685898187447 | 12.1245488897355 | Luxembourg |
| 2008 | 12.53394780122 | 0.847978883152449 | 1.38993704192887 | 11.1440107592911 | Luxembourg |
| 2009 | 12.33107238727 | 0.850688725320833 | 1.35408581158612 | 10.9769865756839 | Luxembourg |
| 2010 | 13.10980717079 | 0.85325827666028 | 1.3059686042184 | 11.8038385665716 | Luxembourg |
| 2011 | 11.79861427123 | 0.856216833587911 | 1.32181533005804 | 10.476798941172 | Luxembourg |
| 2012 | 10.05823527375 | 0.859789031199815 | 1.29412767168548 | 8.76410760206452 | Luxembourg |
| 2013 | 10.89996686338 | 0.863368792293471 | 1.29752264717054 | 9.60244421620946 | Luxembourg |
| 2014 | 10.38540259881 | 0.86669355608594 | 1.28302550115024 | 9.10237709765976 | Luxembourg |
| 2015 | 9.96470265877 | 0.87011293699584 | 1.25070665061902 | 8.71399600815098 | Luxembourg |
| 2016 | 10.63368184964 | 0.873420879802852 | 1.24641303628968 | 9.38726881335032 | Luxembourg |
| 2017 | 9.9866480411 | 0.876347684357551 | 1.27675085112303 | 8.70989718997697 | Luxembourg |
| 2018 | 10.62849675292 | 0.878637895036722 | 1.29039211340205 | 9.33810463951795 | Luxembourg |
| 2019 | 8.99404422008 | 0.880593895218451 | 1.2659964908917 | 7.7280477291883 | Luxembourg |
| 2020 | 8.60675618575 | 0.882495316094676 | 1.278537424821 | 7.328218760929 | Luxembourg |
| 2021 | 8.27942856096 | 0.884428955125693 | 1.25584501694118 | 7.02358354401882 | Luxembourg |
| 1990 | 6.06923800468 | 0.656504584147482 | 4.66560886629175 | 1.40362913838825 | Malta |
| 1991 | 6.36712637991 | 0.66111269147347 | 4.66884608234364 | 1.69828029756636 | Malta |
| 1992 | 6.50323018188 | 0.666324647620912 | 3.43200360756389 | 3.07122657431611 | Malta |
| 1993 | 6.61232861512 | 0.672120257474545 | 3.08614434066486 | 3.52618427445514 | Malta |
| 1994 | 6.74451276743 | 0.67784731358543 | 2.834813069855 | 3.909699697575 | Malta |
| 1995 | 6.94178260545 | 0.682617703672922 | 2.84422463683571 | 4.09755796861429 | Malta |
| 1996 | 7.00900556896 | 0.68662326099066 | 2.93603562774152 | 4.07296994121848 | Malta |
| 1997 | 7.10078297117 | 0.691756406092708 | 2.65786490510237 | 4.44291806606763 | Malta |
| 1998 | 7.26930220548 | 0.698095287002014 | 2.368200678857 | 4.901101526623 | Malta |
| 1999 | 7.46070419798 | 0.704734545351825 | 2.29307935429359 | 5.16762484368641 | Malta |
| 2000 | 7.6903350759 | 0.711805254226466 | 2.24267692804976 | 5.44765814785024 | Malta |
| 2001 | 7.90555626252 | 0.717803037638719 | 2.24277948036816 | 5.66277678215184 | Malta |
| 2002 | 8.17089215663 | 0.722790274731056 | 2.14376945702146 | 6.02712269960854 | Malta |
| 2003 | 8.48079837127 | 0.727511133046943 | 2.17034973534524 | 6.31044863592476 | Malta |
| 2004 | 8.80980400659 | 0.731933852781332 | 2.18416239515867 | 6.62564161143133 | Malta |
| 2005 | 9.17728532285 | 0.736188746914511 | 2.16960012772311 | 7.00768519512689 | Malta |
| 2006 | 9.61151324066 | 0.740140181028362 | 2.07446101542119 | 7.53705222523881 | Malta |
| 2007 | 10.06320510856 | 0.744237811910071 | 2.079151668542 | 7.984053440018 | Malta |
| 2008 | 10.31561669486 | 0.748427095431231 | 2.02121888751298 | 8.29439780734702 | Malta |
| 2009 | 10.34879399073 | 0.752171058957125 | 2.00244435363527 | 8.34634963709473 | Malta |
| 2010 | 10.32121875707 | 0.756250500159098 | 1.98736721068656 | 8.33385154638344 | Malta |
| 2011 | 9.99684139635 | 0.760310546511914 | 1.85478328221838 | 8.14205811413162 | Malta |
| 2012 | 9.8563150796 | 0.764537880070192 | 1.70616496323759 | 8.15015011636241 | Malta |
| 2013 | 9.76685460056 | 0.768616139641066 | 1.6228634369071 | 8.1439911636529 | Malta |
| 2014 | 9.59879727067 | 0.772728741377431 | 1.62731168599065 | 7.97148558467935 | Malta |
| 2015 | 9.53800004902 | 0.777276905450885 | 1.47597194791543 | 8.06202810110457 | Malta |
| 2016 | 9.18118701065 | 0.781673409574621 | 1.4975809509907 | 7.6836060596593 | Malta |
| 2017 | 8.561808003 | 0.786410183585369 | 1.46303242156051 | 7.09877558143949 | Malta |
| 2018 | 7.80867614544 | 0.79096561824237 | 1.45779989520837 | 6.35087625023163 | Malta |
| 2019 | 7.36147416448 | 0.795197845728697 | 1.49378407726914 | 5.86769008721086 | Malta |
| 2020 | 6.89475972885 | 0.798375166241004 | 1.46095805012128 | 5.43380167872872 | Malta |
| 2021 | 6.76746777551 | 0.801585033998971 | 1.46382005924415 | 5.30364771626585 | Malta |
| 1990 | 8.94744668554 | 0.794612123302941 | 1.49165586939089 | 7.45579081614911 | Netherlands |
| 1991 | 9.16464739542 | 0.799285437176668 | 1.44992686007421 | 7.71472053534579 | Netherlands |
| 1992 | 9.49986918968 | 0.803965978232379 | 1.47436304380184 | 8.02550614587816 | Netherlands |
| 1993 | 9.74151431807 | 0.808278967039783 | 1.41855656651171 | 8.32295775155829 | Netherlands |
| 1994 | 9.86733073144 | 0.812530215874006 | 1.46055594156778 | 8.40677478987222 | Netherlands |
| 1995 | 10.10116279889 | 0.816662769214157 | 1.47497119107857 | 8.62619160781143 | Netherlands |
| 1996 | 10.43257738177 | 0.819984219587417 | 1.4354083858902 | 8.9971689958798 | Netherlands |
| 1997 | 10.71258650392 | 0.82288177029344 | 1.42445497997121 | 9.28813152394879 | Netherlands |
| 1998 | 11.11561106399 | 0.825882233724006 | 1.4490379651402 | 9.6665730988498 | Netherlands |
| 1999 | 11.64945772727 | 0.828693827126542 | 1.45278015321294 | 10.1966775740571 | Netherlands |
| 2000 | 12.40655404339 | 0.831913983967645 | 1.41277747971725 | 10.9937765636727 | Netherlands |
| 2001 | 13.21531179079 | 0.835518148042208 | 1.4104269159515 | 11.8048848748385 | Netherlands |
| 2002 | 14.06033914566 | 0.838676076741848 | 1.40204030600732 | 12.6582988396527 | Netherlands |
| 2003 | 14.88904531466 | 0.841639477052547 | 1.40008568929824 | 13.4889596253618 | Netherlands |
| 2004 | 15.64149134711 | 0.844807347619539 | 1.32568752216945 | 14.3158038249405 | Netherlands |
| 2005 | 16.84539285036 | 0.847934189839563 | 1.35970353342587 | 15.4856893169341 | Netherlands |
| 2006 | 17.59903591116 | 0.850798807603514 | 1.34207287635984 | 16.2569630348002 | Netherlands |
| 2007 | 18.10274254008 | 0.853595391042959 | 1.31906354930537 | 16.7836789907746 | Netherlands |
| 2008 | 18.4968119707 | 0.8563684666871 | 1.31459565838404 | 17.182216312316 | Netherlands |
| 2009 | 18.83262015566 | 0.858600459092211 | 1.32920986258158 | 17.5034102930784 | Netherlands |
| 2010 | 18.79372048012 | 0.861255297781524 | 1.29914960701161 | 17.4945708731084 | Netherlands |
| 2011 | 18.06637211187 | 0.864246678180707 | 1.260465786014 | 16.805906325856 | Netherlands |
| 2012 | 18.00838805953 | 0.866950626243189 | 1.29179262857319 | 16.7165954309568 | Netherlands |
| 2013 | 17.95487046801 | 0.8694029789072 | 1.24905188767 | 16.70581858034 | Netherlands |
| 2014 | 17.78731196809 | 0.871848914319002 | 1.26348853476425 | 16.5238234333257 | Netherlands |
| 2015 | 17.61776943841 | 0.874402841072396 | 1.25847972998871 | 16.3592897084213 | Netherlands |
| 2016 | 17.1966967183 | 0.876784051787216 | 1.26289922272385 | 15.9337974955762 | Netherlands |
| 2017 | 16.2905252304 | 0.87930015206581 | 1.27205345106241 | 15.0184717793376 | Netherlands |
| 2018 | 15.11293673332 | 0.881900218359353 | 1.2759176981181 | 13.8370190352019 | Netherlands |
| 2019 | 14.5835115875 | 0.884643839807538 | 1.25267760547419 | 13.3308339820258 | Netherlands |
| 2020 | 14.10173449595 | 0.886558566295774 | 1.27588092791189 | 12.8258535680381 | Netherlands |
| 2021 | 13.77986268875 | 0.888464256397732 | 1.29074452672886 | 12.4891181620211 | Netherlands |
| 1990 | 4.6708614397 | 0.795887277333644 | 1.453202248821 | 3.217659190879 | Norway |
| 1991 | 4.4476904861 | 0.800468885755463 | 1.44684576388579 | 3.00084472221421 | Norway |
| 1992 | 4.54140388747 | 0.805437473231399 | 1.40965102918644 | 3.13175285828355 | Norway |
| 1993 | 4.58970351819 | 0.810565689028095 | 1.4612548337213 | 3.1284486844687 | Norway |
| 1994 | 4.73301118493 | 0.815414668396239 | 1.45532011414475 | 3.27769107078525 | Norway |
| 1995 | 4.88369792895 | 0.820223522303065 | 1.43657782053444 | 3.44712010841556 | Norway |
| 1996 | 5.06099975268 | 0.825701668363619 | 1.41484056544556 | 3.64615918723444 | Norway |
| 1997 | 4.95688033095 | 0.831468770271884 | 1.4489936148275 | 3.5078867161225 | Norway |
| 1998 | 5.0260505512 | 0.836562809269057 | 1.37833323542167 | 3.64771731577833 | Norway |
| 1999 | 5.1934505468 | 0.841130236756497 | 1.37411748258423 | 3.81933306421577 | Norway |
| 2000 | 5.27981753961 | 0.846475451318379 | 1.32704750840771 | 3.95277003120229 | Norway |
| 2001 | 5.25201968788 | 0.852111935736606 | 1.34948410590023 | 3.90253558197977 | Norway |
| 2002 | 5.3623910292 | 0.856757888176133 | 1.31850412455111 | 4.04388690464889 | Norway |
| 2003 | 5.30278775242 | 0.860302576313355 | 1.28011474445488 | 4.02267300796512 | Norway |
| 2004 | 5.07534579729 | 0.863468908330188 | 1.26850066669519 | 3.80684513059481 | Norway |
| 2005 | 5.49755454797 | 0.866217487824484 | 1.28976859349238 | 4.20778595447762 | Norway |
| 2006 | 5.66013244545 | 0.868588434600934 | 1.28893671180133 | 4.37119573364867 | Norway |
| 2007 | 5.72956050208 | 0.870929669588489 | 1.26677437313488 | 4.46278612894512 | Norway |
| 2008 | 6.06586364795 | 0.873745121433024 | 1.26082933070854 | 4.80503431724146 | Norway |
| 2009 | 6.42225383522 | 0.876582817210667 | 1.25391555803556 | 5.16833827718444 | Norway |
| 2010 | 6.15214490584 | 0.880378381331352 | 1.25263288515239 | 4.89951202068761 | Norway |
| 2011 | 5.7356137972 | 0.884351666714408 | 1.24372920006405 | 4.49188459713595 | Norway |
| 2012 | 5.51507885303 | 0.888160475097858 | 1.26754881470047 | 4.24753003832954 | Norway |
| 2013 | 5.22310373171 | 0.891989617651275 | 1.27156858592648 | 3.95153514578352 | Norway |
| 2014 | 4.98438047 | 0.895499545979859 | 1.26416234239 | 3.72021812761 | Norway |
| 2015 | 4.83239591482 | 0.89891264398603 | 1.27139745823706 | 3.56099845658294 | Norway |
| 2016 | 4.51338029905 | 0.902584186845854 | 1.26536613761028 | 3.24801416143972 | Norway |
| 2017 | 4.64335099615 | 0.906044048809343 | 1.26714146201143 | 3.37620953413857 | Norway |
| 2018 | 4.88788681271 | 0.909261782035669 | 1.28245202896674 | 3.60543478374326 | Norway |
| 2019 | 5.00716486648 | 0.912278760654598 | 1.27764411130024 | 3.72952075517976 | Norway |
| 2020 | 4.59683780334 | 0.914529920283541 | 1.27921395437884 | 3.31762384896116 | Norway |
| 2021 | 4.55439730848 | 0.916132810119013 | 1.23711927641708 | 3.31727803206292 | Norway |
| 1990 | 15.96014680938 | 0.599777756742926 | 13.4296075764075 | 2.53053923297245 | Portugal |
| 1991 | 14.04500348142 | 0.607269448643355 | 13.0280400057758 | 1.01696347564421 | Portugal |
| 1992 | 13.26529706583 | 0.614808079780387 | 12.7637746899125 | 0.50152237591746 | Portugal |
| 1993 | 13.80311710638 | 0.622101176817746 | 12.7546301364977 | 1.04848696988232 | Portugal |
| 1994 | 10.89386506671 | 0.629306386686867 | 10.89386506671 | 0 | Portugal |
| 1995 | 9.818607768 | 0.635498761462007 | 9.68060671364308 | 0.138001054356923 | Portugal |
| 1996 | 10.90243543078 | 0.640864525833465 | 7.27645259445407 | 3.62598283632593 | Portugal |
| 1997 | 11.05734814874 | 0.64619290440225 | 6.10262228318054 | 4.95472586555946 | Portugal |
| 1998 | 10.80591585369 | 0.651335993931501 | 5.08623145622261 | 5.71968439746739 | Portugal |
| 1999 | 9.61341388126 | 0.656120666715453 | 4.93140741947342 | 4.68200646178658 | Portugal |
| 2000 | 8.91714008138 | 0.66161085244449 | 4.64580686681444 | 4.27133321456555 | Portugal |
| 2001 | 8.80260810172 | 0.667247150668184 | 3.68175359765935 | 5.12085450406065 | Portugal |
| 2002 | 7.97175656707 | 0.67258393952723 | 3.737625606347 | 4.234130960723 | Portugal |
| 2003 | 7.47362342236 | 0.677663411506582 | 2.69929382198647 | 4.77432960037353 | Portugal |
| 2004 | 7.2817876346 | 0.682352926478464 | 2.75385052400417 | 4.52793711059583 | Portugal |
| 2005 | 7.38900034063 | 0.686887104684271 | 2.8594150032144 | 4.5295853374156 | Portugal |
| 2006 | 7.49983757097 | 0.691158795071141 | 2.5127693432284 | 4.9870682277416 | Portugal |
| 2007 | 8.00757170269 | 0.694974852517783 | 2.40591416700767 | 5.60165753568233 | Portugal |
| 2008 | 8.12034189194 | 0.69860459852573 | 2.435636050215 | 5.684705841725 | Portugal |
| 2009 | 8.27590292786 | 0.702223398701003 | 2.46609610754257 | 5.80980682031743 | Portugal |
| 2010 | 7.46369223515 | 0.706563554661067 | 2.24790276526079 | 5.21578946988921 | Portugal |
| 2011 | 7.11965289846 | 0.71126289291359 | 2.24351775621159 | 4.87613514224841 | Portugal |
| 2012 | 7.16612055661 | 0.715804084405661 | 2.20880470860477 | 4.95731584800523 | Portugal |
| 2013 | 6.79947088799 | 0.719836991474169 | 2.16997232981538 | 4.62949855817462 | Portugal |
| 2014 | 6.63396357382 | 0.723021281895034 | 2.19186466901163 | 4.44209890480837 | Portugal |
| 2015 | 6.83919382601 | 0.725756220353222 | 2.14322007675085 | 4.69597374925915 | Portugal |
| 2016 | 7.03127087032 | 0.728300849268768 | 2.19425331958882 | 4.83701755073118 | Portugal |
| 2017 | 7.059352001 | 0.73122553459241 | 2.1845726227634 | 4.8747793782366 | Portugal |
| 2018 | 6.8807527316 | 0.73440294716366 | 2.15489291996911 | 4.72585981163089 | Portugal |
| 2019 | 6.38727583247 | 0.738090480746574 | 2.15832839983333 | 4.22894743263667 | Portugal |
| 2020 | 5.66346742708 | 0.741037384212082 | 2.11933431038814 | 3.54413311669186 | Portugal |
| 2021 | 5.42128655177 | 0.744151850665712 | 2.07892212044944 | 3.34236443132056 | Portugal |
| 1990 | 4.37074782548 | 0.63667316626401 | 4.37074782548 | 0 | Spain |
| 1991 | 4.28815962633 | 0.644130744620668 | 4.28815962633 | 0 | Spain |
| 1992 | 4.37856801887 | 0.65159759683081 | 4.33991108054163 | 0.0386569383283675 | Spain |
| 1993 | 4.29357402725 | 0.658637568907277 | 4.29150381513353 | 0.00207021211647085 | Spain |
| 1994 | 4.1312178033 | 0.665321549207614 | 4.1312178033 | 0 | Spain |
| 1995 | 4.10395831698 | 0.671576034104742 | 3.0097176847285 | 1.0942406322515 | Spain |
| 1996 | 4.09807214537 | 0.677088943314314 | 2.9123109693421 | 1.18576117602789 | Spain |
| 1997 | 4.05521118241 | 0.68242949710256 | 2.82648036669733 | 1.22873081571267 | Spain |
| 1998 | 4.07953257854 | 0.687490716991391 | 2.48207046535345 | 1.59746211318655 | Spain |
| 1999 | 4.05677892092 | 0.692245196919437 | 2.55163307954243 | 1.50514584137757 | Spain |
| 2000 | 4.23563172816 | 0.697056031108428 | 2.43750571488256 | 1.79812601327744 | Spain |
| 2001 | 4.28419578796 | 0.701726069724186 | 2.35748916446533 | 1.92670662349467 | Spain |
| 2002 | 4.40982340433 | 0.706309654864074 | 2.3008494659663 | 2.1089739383637 | Spain |
| 2003 | 4.56971981216 | 0.710678584323191 | 2.27512465466429 | 2.29459515749571 | Spain |
| 2004 | 4.73138609354 | 0.714886229907558 | 2.21565678765595 | 2.51572930588405 | Spain |
| 2005 | 4.8932943663 | 0.718717288882423 | 2.17557165124244 | 2.71772271505756 | Spain |
| 2006 | 5.25132040729 | 0.721970128302216 | 2.18953803611833 | 3.06178237117167 | Spain |
| 2007 | 5.63739921453 | 0.725211442824273 | 2.172856814402 | 3.464542400128 | Spain |
| 2008 | 5.63625775332 | 0.729411041261585 | 2.17039653700872 | 3.46586121631128 | Spain |
| 2009 | 5.61742559444 | 0.733956054531859 | 2.17726256711 | 3.44016302733 | Spain |
| 2010 | 5.42011691467 | 0.738352055066564 | 2.14736427745585 | 3.27275263721415 | Spain |
| 2011 | 5.03524912781 | 0.742096450004589 | 2.09828771954214 | 2.93696140826786 | Spain |
| 2012 | 4.88970881935 | 0.745181867042751 | 2.06783704236364 | 2.82187177698636 | Spain |
| 2013 | 4.68348612515 | 0.747651982075541 | 2.04618580285176 | 2.63730032229824 | Spain |
| 2014 | 4.7374628847 | 0.74980092875112 | 2.036552046348 | 2.700910838352 | Spain |
| 2015 | 4.744877381 | 0.752269822995419 | 2.01944028377907 | 2.72543709722093 | Spain |
| 2016 | 4.74360302886 | 0.75475793948614 | 1.9784431128875 | 2.7651599159725 | Spain |
| 2017 | 4.71607546488 | 0.757663276271395 | 1.90927534870872 | 2.80680011617128 | Spain |
| 2018 | 4.53469842364 | 0.760659574783565 | 1.8423032058966 | 2.6923952177434 | Spain |
| 2019 | 4.41139088642 | 0.763942918439527 | 1.70086789685167 | 2.71052298956833 | Spain |
| 2020 | 4.13898762204 | 0.766506025058573 | 1.72095026071212 | 2.41803736132788 | Spain |
| 2021 | 4.0790058141 | 0.769283697979998 | 1.63155422170289 | 2.44745159239711 | Spain |
| 1990 | 9.53193114166 | 0.78553579188163 | 1.47831572918837 | 8.05361541247163 | Sweden |
| 1991 | 9.32326564417 | 0.790096440686927 | 1.457514654385 | 7.865750989785 | Sweden |
| 1992 | 9.27662409792 | 0.795431457884408 | 1.49209665482476 | 7.78452744309524 | Sweden |
| 1993 | 9.24123731652 | 0.801009521825474 | 1.41644184829488 | 7.82479546822512 | Sweden |
| 1994 | 8.95723738152 | 0.806700264491967 | 1.48952713645973 | 7.46771024506027 | Sweden |
| 1995 | 8.92632710797 | 0.81224465438799 | 1.5277102968063 | 7.3986168111637 | Sweden |
| 1996 | 8.98161768966 | 0.817227025218912 | 1.43933341266 | 7.542284277 | Sweden |
| 1997 | 9.04987129215 | 0.82169405939303 | 1.4374581185417 | 7.6124131736083 | Sweden |
| 1998 | 9.33501549282 | 0.825731994976955 | 1.40623049723489 | 7.92878499558511 | Sweden |
| 1999 | 9.57207245138 | 0.82948709884742 | 1.38792191671031 | 8.18415053466969 | Sweden |
| 2000 | 9.97704367989 | 0.833028111989963 | 1.42141110667595 | 8.55563257321406 | Sweden |
| 2001 | 11.04938232611 | 0.835956398134875 | 1.41280698274385 | 9.63657534336615 | Sweden |
| 2002 | 11.74011589304 | 0.838741526580818 | 1.41164659887525 | 10.3284692941648 | Sweden |
| 2003 | 12.49365792485 | 0.841663138093711 | 1.40823973031603 | 11.085418194534 | Sweden |
| 2004 | 12.11670657615 | 0.844506881898062 | 1.38272495523088 | 10.7339816209191 | Sweden |
| 2005 | 12.55086440498 | 0.846876343860947 | 1.38723748522066 | 11.1636269197593 | Sweden |
| 2006 | 12.99085431259 | 0.849115871424855 | 1.33956002789978 | 11.6512942846902 | Sweden |
| 2007 | 13.46554004435 | 0.851420847176242 | 1.3225562928966 | 12.1429837514534 | Sweden |
| 2008 | 13.71010472239 | 0.853705850789255 | 1.33078962300282 | 12.3793150993872 | Sweden |
| 2009 | 15.60883380404 | 0.85570274765727 | 1.29679231466052 | 14.3120414893795 | Sweden |
| 2010 | 16.2673388314 | 0.858727940541151 | 1.31046673471719 | 14.9568720966828 | Sweden |
| 2011 | 15.77833945837 | 0.861892379268929 | 1.305664801009 | 14.472674657361 | Sweden |
| 2012 | 16.19760450667 | 0.864706671533252 | 1.27960847487895 | 14.9179960317911 | Sweden |
| 2013 | 16.24313684637 | 0.867383767613666 | 1.28560259503941 | 14.9575342513306 | Sweden |
| 2014 | 15.33590914975 | 0.869923325091982 | 1.25877595964719 | 14.0771331901028 | Sweden |
| 2015 | 15.56432068854 | 0.87238851487302 | 1.27527629731772 | 14.2890443912223 | Sweden |
| 2016 | 15.87011021472 | 0.874661654943114 | 1.26416301954472 | 14.6059471951753 | Sweden |
| 2017 | 15.5660246383 | 0.877120611340732 | 1.27991963564817 | 14.2861050026518 | Sweden |
| 2018 | 15.13756309943 | 0.879888911893351 | 1.2737653829266 | 13.8637977165034 | Sweden |
| 2019 | 14.75041543991 | 0.882984115679233 | 1.25584947328593 | 13.4945659666241 | Sweden |
| 2020 | 14.32214217379 | 0.88506201036603 | 1.23437111116411 | 13.0877710626259 | Sweden |
| 2021 | 13.53082597639 | 0.886880298870342 | 1.27464657069065 | 12.2561794056993 | Sweden |
| 1990 | 17.00982398443 | 0.862766843874175 | 1.27398499911981 | 15.7358389853102 | Switzerland |
| 1991 | 17.53370867674 | 0.864796867138941 | 1.29566094830355 | 16.2380477284365 | Switzerland |
| 1992 | 17.67057979412 | 0.86773607877979 | 1.28449299644642 | 16.3860867976736 | Switzerland |
| 1993 | 17.41950631115 | 0.870863557610096 | 1.24137255322917 | 16.1781337579208 | Switzerland |
| 1994 | 16.95274013039 | 0.873140871296934 | 1.25191489560306 | 15.7008252347869 | Switzerland |
| 1995 | 16.59461099505 | 0.874835330674703 | 1.25606774666558 | 15.3385432483844 | Switzerland |
| 1996 | 16.6153726644 | 0.876640556809649 | 1.26888594522667 | 15.3464867191733 | Switzerland |
| 1997 | 16.98864884573 | 0.87848554526934 | 1.22682195287219 | 15.7618268928578 | Switzerland |
| 1998 | 17.48822940513 | 0.880035152091359 | 1.26102096590621 | 16.2272084392238 | Switzerland |
| 1999 | 17.99308236373 | 0.882045586320071 | 1.27282181883156 | 16.7202605448984 | Switzerland |
| 2000 | 18.85145024233 | 0.885243530890309 | 1.29063571493692 | 17.5608145273931 | Switzerland |
| 2001 | 19.32825051281 | 0.888445951179249 | 1.24927285129444 | 18.0789776615156 | Switzerland |
| 2002 | 19.62733130372 | 0.890902811034425 | 1.23343799200778 | 18.3938933117122 | Switzerland |
| 2003 | 20.36042294059 | 0.892780807435311 | 1.25201963512067 | 19.1084033054693 | Switzerland |
| 2004 | 21.17646733761 | 0.894909812216857 | 1.28283244341661 | 19.8936348941934 | Switzerland |
| 2005 | 22.04216364455 | 0.897272629528173 | 1.24905884494414 | 20.7931047996059 | Switzerland |
| 2006 | 23.27392634248 | 0.899962304608863 | 1.26272323902308 | 22.0112031034569 | Switzerland |
| 2007 | 23.5251010981 | 0.902913412619355 | 1.2605449007624 | 22.2645561973376 | Switzerland |
| 2008 | 23.668534726 | 0.90577845443162 | 1.24972666155015 | 22.4188080644499 | Switzerland |
| 2009 | 23.7480164306 | 0.908049997864177 | 1.29044162073387 | 22.4575748098661 | Switzerland |
| 2010 | 23.03132830969 | 0.910530522112102 | 1.25316511570551 | 21.7781631939845 | Switzerland |
| 2011 | 22.10114775513 | 0.91327657336798 | 1.25932698955017 | 20.8418207655798 | Switzerland |
| 2012 | 21.73019168985 | 0.91571489343801 | 1.25227008586957 | 20.4779216039804 | Switzerland |
| 2013 | 21.71945393831 | 0.917955005608177 | 1.25416829839828 | 20.4652856399117 | Switzerland |
| 2014 | 21.44959023308 | 0.919994182026162 | 1.2564047512966 | 20.1931854817834 | Switzerland |
| 2015 | 21.30575725353 | 0.921832990505373 | 1.25557570597333 | 20.0501815475567 | Switzerland |
| 2016 | 20.18982702612 | 0.923930598455048 | 1.237075329515 | 18.952751696605 | Switzerland |
| 2017 | 19.44981514013 | 0.926202291104072 | 1.26251330826621 | 18.1873018318638 | Switzerland |
| 2018 | 17.9545403499 | 0.928550590010717 | 1.24790660308276 | 16.7066337468172 | Switzerland |
| 2019 | 16.84379896877 | 0.930682101951976 | 1.27281682758322 | 15.5709821411868 | Switzerland |
| 2020 | 16.38665433293 | 0.932027644512362 | 1.25368619256238 | 15.1329681403676 | Switzerland |
| 2021 | 16.00343853978 | 0.933059110986638 | 1.26860342369418 | 14.7348351160858 | Switzerland |
| 1990 | 5.63194418411 | 0.744334126300131 | 2.06261771820556 | 3.56932646590444 | United Kingdom |
| 1991 | 5.65697555514 | 0.749148231365388 | 2.0674063687475 | 3.5895691863925 | United Kingdom |
| 1992 | 5.80306457161 | 0.754556689127024 | 2.00585644874289 | 3.79720812286711 | United Kingdom |
| 1993 | 6.1809256144 | 0.760154350107152 | 1.82945931687136 | 4.35146629752864 | United Kingdom |
| 1994 | 6.41968084547 | 0.765164600469893 | 1.71458721540525 | 4.70509363006475 | United Kingdom |
| 1995 | 6.73035270438 | 0.768656863963456 | 1.58551114012463 | 5.14484156425537 | United Kingdom |
| 1996 | 7.22744563375 | 0.771576484075751 | 1.5642145793815 | 5.6632310543685 | United Kingdom |
| 1997 | 7.63523083299 | 0.775050121968743 | 1.49029572297867 | 6.14493511001133 | United Kingdom |
| 1998 | 8.12017351368 | 0.779057165114363 | 1.48429574715884 | 6.63587776652116 | United Kingdom |
| 1999 | 8.65107558078 | 0.784036475142408 | 1.46643030577128 | 7.18464527500872 | United Kingdom |
| 2000 | 9.16977882798 | 0.789218959863246 | 1.46693148558022 | 7.70284734239978 | United Kingdom |
| 2001 | 9.70110259156 | 0.793513165354113 | 1.42801139059042 | 8.27309120096958 | United Kingdom |
| 2002 | 10.24151443171 | 0.796655559574601 | 1.46889163387073 | 8.77262279783927 | United Kingdom |
| 2003 | 11.39560751551 | 0.799186111437993 | 1.45369239880455 | 9.94191511670545 | United Kingdom |
| 2004 | 12.67323696076 | 0.801950870000537 | 1.44844298524674 | 11.2247939755133 | United Kingdom |
| 2005 | 15.41469056527 | 0.804489193801038 | 1.47034648650116 | 13.9443440787688 | United Kingdom |
| 2006 | 19.33841452912 | 0.806817045960102 | 1.47216287242364 | 17.8662516566964 | United Kingdom |
| 2007 | 19.98954181163 | 0.80935277163371 | 1.44920125527946 | 18.5403405563505 | United Kingdom |
| 2008 | 16.85122953785 | 0.812039287236426 | 1.44869983012274 | 15.4025297077273 | United Kingdom |
| 2009 | 13.72711851342 | 0.814793642346203 | 1.47109768098849 | 12.2560208324315 | United Kingdom |
| 2010 | 11.48644200107 | 0.818033993642746 | 1.45933274771659 | 10.0271092533534 | United Kingdom |
| 2011 | 10.29563751583 | 0.821718995421102 | 1.44039548668022 | 8.85524202914978 | United Kingdom |
| 2012 | 9.17342897664 | 0.826453754133025 | 1.43709753221212 | 7.73633144442788 | United Kingdom |
| 2013 | 8.25103431256 | 0.831508668228002 | 1.41394844228902 | 6.83708587027098 | United Kingdom |
| 2014 | 7.50295934424 | 0.835516642361539 | 1.43883323431 | 6.06412610993 | United Kingdom |
| 2015 | 7.06433711251 | 0.838951918781599 | 1.42472742209531 | 5.63960969041469 | United Kingdom |
| 2016 | 6.38282357635 | 0.842460232628565 | 1.38286933588261 | 4.99995424046739 | United Kingdom |
| 2017 | 5.48003034073 | 0.846263617759747 | 1.34844527516156 | 4.13158506556844 | United Kingdom |
| 2018 | 4.78161728122 | 0.849969787501756 | 1.35490908165514 | 3.42670819956486 | United Kingdom |
| 2019 | 4.52425419485 | 0.854130572600886 | 1.32702740320647 | 3.19722679164353 | United Kingdom |
| 2020 | 3.93382465992 | 0.856920290432659 | 1.3092028993415 | 2.6246217605785 | United Kingdom |
| 2021 | 4.01598201184 | 0.859000181772086 | 1.32726511242194 | 2.68871689941806 | United Kingdom |
| 1990 | 183.97108668275 | 0.587397284272094 | 13.1693029783754 | 170.801783704375 | Argentina |
| 1991 | 163.515550456 | 0.591707540464012 | 13.3852102621599 | 150.13034019384 | Argentina |
| 1992 | 137.41461944387 | 0.597996324377555 | 13.2711007606019 | 124.143518683268 | Argentina |
| 1993 | 116.95614517573 | 0.603739312309972 | 13.2849817195634 | 103.671163456167 | Argentina |
| 1994 | 109.80579015709 | 0.609787330316145 | 13.1795792835519 | 96.6262108735381 | Argentina |
| 1995 | 113.3790695654 | 0.614473705638113 | 13.0147316123953 | 100.364337953005 | Argentina |
| 1996 | 110.48245284701 | 0.618942623582172 | 12.9904606751196 | 97.4919921718904 | Argentina |
| 1997 | 117.18476184687 | 0.623934408870184 | 12.8858138989373 | 104.298947947933 | Argentina |
| 1998 | 110.84700383754 | 0.6287234356344 | 12.6317761672818 | 98.2152276702582 | Argentina |
| 1999 | 94.94555946509 | 0.633175129405293 | 10.8194764414241 | 84.1260830236659 | Argentina |
| 2000 | 83.69271222678 | 0.638194471801002 | 7.94177982890494 | 75.7509323978751 | Argentina |
| 2001 | 80.89702881835 | 0.642175704746832 | 7.7759264961985 | 73.1211023221515 | Argentina |
| 2002 | 84.5451621801 | 0.644472506550469 | 5.91851811405329 | 78.6266440660467 | Argentina |
| 2003 | 74.04062035939 | 0.645906577030896 | 5.40373164813141 | 68.6368887112586 | Argentina |
| 2004 | 61.68316271983 | 0.648296828210031 | 5.98187641310045 | 55.7012863067295 | Argentina |
| 2005 | 57.85434904709 | 0.653368829991767 | 5.42648730639382 | 52.4278617406962 | Argentina |
| 2006 | 56.87459112138 | 0.657445899915566 | 4.65365873671809 | 52.2209323846619 | Argentina |
| 2007 | 54.08582302922 | 0.660187575972401 | 4.6309626883987 | 49.4548603408213 | Argentina |
| 2008 | 55.26824476537 | 0.6630792353937 | 4.42815539153843 | 50.8400893738316 | Argentina |
| 2009 | 55.08174762622 | 0.665911944148118 | 4.24806881076813 | 50.8336788154519 | Argentina |
| 2010 | 55.21348198297 | 0.669925880151809 | 3.49169962495337 | 51.7217823580166 | Argentina |
| 2011 | 51.46764231952 | 0.675242697368294 | 3.01338114790463 | 48.4542611716154 | Argentina |
| 2012 | 45.36544279285 | 0.679218239775544 | 2.8053548015775 | 42.5600879912725 | Argentina |
| 2013 | 45.5788117476 | 0.681633214735039 | 2.88070939933042 | 42.6981023482696 | Argentina |
| 2014 | 45.40202591767 | 0.684974509115572 | 2.70437139011733 | 42.6976545275527 | Argentina |
| 2015 | 40.44798206643 | 0.691710125973835 | 2.62364372613203 | 37.824338340298 | Argentina |
| 2016 | 36.16030930784 | 0.698618031454613 | 2.42487766884625 | 33.7354316389938 | Argentina |
| 2017 | 31.72480602111 | 0.705135528765331 | 2.29639038958162 | 29.4284156315284 | Argentina |
| 2018 | 26.13275518778 | 0.712888693454577 | 2.24054049774508 | 23.8922146900349 | Argentina |
| 2019 | 24.746612084 | 0.719002842751183 | 2.20847579733588 | 22.5381362866641 | Argentina |
| 2020 | 22.16846308225 | 0.721294302628992 | 2.22635997438134 | 19.9421031078687 | Argentina |
| 2021 | 21.05880308583 | 0.723122972582249 | 2.14915560716808 | 18.9096474786619 | Argentina |
| 1990 | 85.8893926983 | 0.586495099638694 | 13.3212145031003 | 72.5681781951997 | Chile |
| 1991 | 74.39924266076 | 0.593006580479257 | 13.3471404021121 | 61.0521022586479 | Chile |
| 1992 | 63.64041410696 | 0.599106083381125 | 13.3561951974067 | 50.2842189095533 | Chile |
| 1993 | 52.90960975808 | 0.604828509357091 | 13.4451698489675 | 39.4644399091125 | Chile |
| 1994 | 49.34815237809 | 0.61067393285511 | 13.3696387877964 | 35.9785135902936 | Chile |
| 1995 | 50.73141604595 | 0.617234676665645 | 13.1657078472613 | 37.5657081986887 | Chile |
| 1996 | 51.06322416832 | 0.623694459543547 | 12.9632497566904 | 38.0999744116296 | Chile |
| 1997 | 49.44470371654 | 0.630540084786116 | 12.1411869482776 | 37.3035167682624 | Chile |
| 1998 | 47.1456453095 | 0.638082758454141 | 8.50994742041829 | 38.6356978890817 | Chile |
| 1999 | 43.7106084294 | 0.645928084390867 | 6.09455746176446 | 37.6160509676355 | Chile |
| 2000 | 40.11554905347 | 0.652989027528663 | 5.1595587868195 | 34.9559902666505 | Chile |
| 2001 | 38.25003778103 | 0.660265510120952 | 4.57399080053048 | 33.6760469804995 | Chile |
| 2002 | 38.13238812898 | 0.667888408106028 | 3.67893045119019 | 34.4534576777898 | Chile |
| 2003 | 37.23794074414 | 0.673445927084645 | 3.42496862865161 | 33.8129721154884 | Chile |
| 2004 | 34.80877323596 | 0.678258212006936 | 2.83523731445 | 31.97353592151 | Chile |
| 2005 | 31.4427769619 | 0.682589191171846 | 2.69867140198662 | 28.7441055599134 | Chile |
| 2006 | 29.14983552166 | 0.685892748248682 | 2.82500546392956 | 26.3248300577304 | Chile |
| 2007 | 27.11380874358 | 0.688930323304188 | 2.51942528865465 | 24.5943834549253 | Chile |
| 2008 | 27.08953472898 | 0.692359787453618 | 2.53849202723281 | 24.5510427017472 | Chile |
| 2009 | 26.67944445944 | 0.696602140685353 | 2.41790409899984 | 24.2615403604402 | Chile |
| 2010 | 24.55062895813 | 0.702304703544999 | 2.37334847731044 | 22.1772804808196 | Chile |
| 2011 | 23.3634947465 | 0.708597935629908 | 2.27440796808914 | 21.0890867784109 | Chile |
| 2012 | 22.08038423552 | 0.714743477340726 | 2.24060121106018 | 19.8397830244598 | Chile |
| 2013 | 21.44058386224 | 0.720448781307689 | 2.19104592974391 | 19.2495379324961 | Chile |
| 2014 | 23.32854466973 | 0.727275926904869 | 2.19521391444529 | 21.1333307552847 | Chile |
| 2015 | 21.7730231099 | 0.736098082475337 | 2.17086812624323 | 19.6021549836568 | Chile |
| 2016 | 19.95704611551 | 0.744972224319701 | 2.05468408180863 | 17.9023620337014 | Chile |
| 2017 | 20.2075555908 | 0.752058077213427 | 2.00178706061382 | 18.2057685301862 | Chile |
| 2018 | 19.69124393797 | 0.758625713610628 | 1.90315319539197 | 17.788090742578 | Chile |
| 2019 | 19.03110654915 | 0.765120322816907 | 1.77282254884939 | 17.2582840003006 | Chile |
| 2020 | 16.14903023176 | 0.769213676196926 | 1.69941897244885 | 14.4496112593111 | Chile |
| 2021 | 15.28436309136 | 0.771514715903929 | 1.55447118116018 | 13.7298919101998 | Chile |
| 1990 | 189.94774005887 | 0.58192185512198 | 13.2730516521955 | 176.674688406675 | Uruguay |
| 1991 | 171.49638569004 | 0.586156118239456 | 13.4733740476284 | 158.023011642412 | Uruguay |
| 1992 | 138.75490678405 | 0.589822355398741 | 13.5279443562451 | 125.226962427805 | Uruguay |
| 1993 | 134.09964444218 | 0.592295025274764 | 13.4848116537943 | 120.614832788386 | Uruguay |
| 1994 | 134.73840326014 | 0.595188424402801 | 13.4625260274222 | 121.275877232718 | Uruguay |
| 1995 | 144.92409553681 | 0.596825409130546 | 13.1269769976644 | 131.797118539146 | Uruguay |
| 1996 | 145.75883385434 | 0.5997751037831 | 13.4119063993061 | 132.346927455034 | Uruguay |
| 1997 | 153.3963326416 | 0.605665051157748 | 13.4426585178991 | 139.953674123701 | Uruguay |
| 1998 | 138.27850345847 | 0.612700815160604 | 13.1826962910999 | 125.09580716737 | Uruguay |
| 1999 | 120.323847943 | 0.619009271243139 | 13.0386138234984 | 107.285234119502 | Uruguay |
| 2000 | 105.48337948883 | 0.623208290785701 | 12.9914780085877 | 92.4919014802423 | Uruguay |
| 2001 | 97.32740450392 | 0.62579606753003 | 13.0649980974009 | 84.2624064065191 | Uruguay |
| 2002 | 100.01236798235 | 0.628285799994443 | 12.671347118831 | 87.341020863519 | Uruguay |
| 2003 | 102.52988537308 | 0.631437773906681 | 11.9261548391054 | 90.6037305339746 | Uruguay |
| 2004 | 91.15370757628 | 0.634216906381842 | 11.2064699730539 | 79.9472376032261 | Uruguay |
| 2005 | 80.87780930371 | 0.63697576957158 | 8.75785470874174 | 72.1199545949682 | Uruguay |
| 2006 | 67.78694469892 | 0.640590696178162 | 7.76943578674101 | 60.017508912179 | Uruguay |
| 2007 | 69.38472574592 | 0.64633171377937 | 5.52121035386904 | 63.863515392051 | Uruguay |
| 2008 | 67.3416251358 | 0.653525522497867 | 4.98067725040127 | 62.3609478853987 | Uruguay |
| 2009 | 65.8158716374 | 0.660321134636261 | 4.58774583717114 | 61.2281258002289 | Uruguay |
| 2010 | 69.35881632238 | 0.666099135301691 | 3.67824637456753 | 65.6805699478125 | Uruguay |
| 2011 | 65.64113865897 | 0.667079733158413 | 3.88307812741134 | 61.7580605315587 | Uruguay |
| 2012 | 60.69458257561 | 0.665802191838499 | 4.31792628589818 | 56.3766562897118 | Uruguay |
| 2013 | 59.28797122205 | 0.668228360836938 | 3.76581883648118 | 55.5221523855688 | Uruguay |
| 2014 | 51.82128506933 | 0.673334260363154 | 3.46993002959365 | 48.3513550397364 | Uruguay |
| 2015 | 46.98313391989 | 0.680595506073599 | 2.91443154245889 | 44.0687023774311 | Uruguay |
| 2016 | 44.30103259394 | 0.689816733014055 | 2.54086704184754 | 41.7601655520925 | Uruguay |
| 2017 | 41.19585383381 | 0.699189455881847 | 2.40737294848591 | 38.7884808853241 | Uruguay |
| 2018 | 40.34841152424 | 0.706904930126484 | 2.30228984950712 | 38.0461216747329 | Uruguay |
| 2019 | 39.740131387 | 0.712536588078123 | 2.22945879211766 | 37.5106725948823 | Uruguay |
| 2020 | 38.40973681804 | 0.716169766093523 | 2.21629856175962 | 36.1934382562804 | Uruguay |
| 2021 | 35.69439726963 | 0.719283444839432 | 2.20024742390782 | 33.4941498457222 | Uruguay |
| 1990 | 1.79631553519 | 0.781977864427607 | 1.45736757650556 | 0.338947958684444 | Canada |
| 1991 | 1.7946341061 | 0.784159360080438 | 1.48058734559217 | 0.314046760507826 | Canada |
| 1992 | 1.769274116 | 0.787030536551619 | 1.42036929250409 | 0.348904823495909 | Canada |
| 1993 | 1.9049622706 | 0.789716593022703 | 1.46072174102258 | 0.444240529577419 | Canada |
| 1994 | 1.89877019871 | 0.79259851803789 | 1.50651009509591 | 0.392260103614091 | Canada |
| 1995 | 1.91361532574 | 0.796521165940602 | 1.492188905834 | 0.421426419906 | Canada |
| 1996 | 1.9032073188 | 0.801064970257054 | 1.4724985321716 | 0.4307087866284 | Canada |
| 1997 | 1.94415241732 | 0.804833356387694 | 1.48266311449481 | 0.461489302825185 | Canada |
| 1998 | 2.02146559869 | 0.808135440512777 | 1.38514599216462 | 0.636319606525384 | Canada |
| 1999 | 2.23507359802 | 0.812265940092679 | 1.44738997237 | 0.78768362565 | Canada |
| 2000 | 2.54218388311 | 0.816685059408778 | 1.413964510862 | 1.128219372248 | Canada |
| 2001 | 2.79650673719 | 0.820696250274983 | 1.42402620657606 | 1.37248053061394 | Canada |
| 2002 | 3.18475907771 | 0.824402271645546 | 1.43455837682625 | 1.75020070088375 | Canada |
| 2003 | 3.501779976 | 0.827801096249711 | 1.4564051846681 | 2.0453747913319 | Canada |
| 2004 | 3.91775885159 | 0.830928109937158 | 1.42175278600125 | 2.49600606558875 | Canada |
| 2005 | 4.34884695517 | 0.833288293645254 | 1.39048634017562 | 2.95836061499438 | Canada |
| 2006 | 4.53672595791 | 0.834915660275685 | 1.41400159543485 | 3.12272436247515 | Canada |
| 2007 | 4.68427839595 | 0.836625096194775 | 1.39371249853204 | 3.29056589741796 | Canada |
| 2008 | 4.68288917013 | 0.838913684557505 | 1.42300083749548 | 3.25988833263452 | Canada |
| 2009 | 4.5244746806 | 0.841583670650067 | 1.35917164058387 | 3.16530304001613 | Canada |
| 2010 | 4.44652514211 | 0.844853488930283 | 1.35900452330229 | 3.08752061880771 | Canada |
| 2011 | 4.22014634275 | 0.848017833149098 | 1.3644684428656 | 2.8556778998844 | Canada |
| 2012 | 4.07590222453 | 0.850978048108266 | 1.34337848975964 | 2.73252373477036 | Canada |
| 2013 | 3.96938928475 | 0.853844945122214 | 1.32109960090071 | 2.64828968384929 | Canada |
| 2014 | 3.6272282603 | 0.856759933938005 | 1.30924292142366 | 2.31798533887634 | Canada |
| 2015 | 3.42711239122 | 0.859532903766548 | 1.29722442885028 | 2.12988796236972 | Canada |
| 2016 | 3.10839667842 | 0.862174225749794 | 1.31010154993424 | 1.79829512848576 | Canada |
| 2017 | 2.79473254513 | 0.865053829759503 | 1.27623887884122 | 1.51849366628878 | Canada |
| 2018 | 2.63518681359 | 0.867905541356997 | 1.27248273366189 | 1.36270407992811 | Canada |
| 2019 | 2.37050071114 | 0.870554479228955 | 1.24999333622788 | 1.12050737491212 | Canada |
| 2020 | 2.25920487595 | 0.871992631299453 | 1.25422718780061 | 1.00497768814939 | Canada |
| 2021 | 1.96934464264 | 0.873170679735405 | 1.25627573827739 | 0.713068904362609 | Canada |
| 1990 | 3.01275543485 | 0.763647690398232 | 1.78447877830231 | 1.22827665654769 | United States of America |
| 1991 | 2.93691281749 | 0.766044294817203 | 1.60501371799698 | 1.33189909949302 | United States of America |
| 1992 | 2.66590124965 | 0.769595491571717 | 1.65527483100562 | 1.01062641864438 | United States of America |
| 1993 | 2.48416601538 | 0.773103516745741 | 1.64998144536486 | 0.834184570015143 | United States of America |
| 1994 | 2.6246657157 | 0.776368587517635 | 1.5522511406412 | 1.0724145750588 | United States of America |
| 1995 | 2.54958088301 | 0.779410253958491 | 1.48476240754714 | 1.06481847546286 | United States of America |
| 1996 | 2.68063381318 | 0.781934769815664 | 1.48455775479943 | 1.19607605838057 | United States of America |
| 1997 | 2.85196916267 | 0.783979277617936 | 1.48228149027556 | 1.36968767239444 | United States of America |
| 1998 | 2.95822193219 | 0.786076331639174 | 1.42346612046057 | 1.53475581172943 | United States of America |
| 1999 | 2.73052397156 | 0.788544535320099 | 1.44563483946731 | 1.28488913209269 | United States of America |
| 2000 | 2.80377668875 | 0.792316650292886 | 1.50953323274606 | 1.29424345600394 | United States of America |
| 2001 | 2.84934268022 | 0.796676792932995 | 1.46524849603114 | 1.38409418418886 | United States of America |
| 2002 | 2.94963409714 | 0.800403318331674 | 1.43197247461667 | 1.51766162252333 | United States of America |
| 2003 | 3.09776072991 | 0.803228024762672 | 1.44539095417577 | 1.65236977573423 | United States of America |
| 2004 | 3.02948876917 | 0.805304692151156 | 1.49613022844333 | 1.53335854072667 | United States of America |
| 2005 | 2.54030408265 | 0.80603307970225 | 1.43032697936829 | 1.10997710328171 | United States of America |
| 2006 | 2.2585654581 | 0.80678358318377 | 1.45412029941206 | 0.804445158687941 | United States of America |
| 2007 | 2.19905648326 | 0.809688049965476 | 1.411555779615 | 0.787500703645 | United States of America |
| 2008 | 2.0827819108 | 0.814577102408992 | 1.42471675803778 | 0.658065152762222 | United States of America |
| 2009 | 1.9395489342 | 0.820294574449102 | 1.42410819302053 | 0.515440741179474 | United States of America |
| 2010 | 1.81901038339 | 0.825884658277638 | 1.410479006421 | 0.408531376969 | United States of America |
| 2011 | 1.84167979225 | 0.830266387766787 | 1.4474736593384 | 0.3942061329116 | United States of America |
| 2012 | 1.81777112201 | 0.833958192158326 | 1.41213023323 | 0.40564088878 | United States of America |
| 2013 | 1.81288719718 | 0.837412988286127 | 1.3855189042125 | 0.4273682929675 | United States of America |
| 2014 | 1.71104090743 | 0.84074652832772 | 1.42758974948632 | 0.283451157943684 | United States of America |
| 2015 | 1.7460898838 | 0.844370336219248 | 1.335195214282 | 0.410894669518 | United States of America |
| 2016 | 1.70469663838 | 0.848015521315013 | 1.38261156000733 | 0.322085078372667 | United States of America |
| 2017 | 1.56355250577 | 0.851630299087012 | 1.33067095924429 | 0.232881546525714 | United States of America |
| 2018 | 1.45875300898 | 0.855049193484192 | 1.33603368220364 | 0.122719326776364 | United States of America |
| 2019 | 1.394703187 | 0.858578064552935 | 1.301614813471 | 0.093088373529 | United States of America |
| 2020 | 1.3248798926 | 0.860792773409847 | 1.27247640153905 | 0.0524034910609523 | United States of America |
| 2021 | 1.28267520801 | 0.862448354015145 | 1.25704607125762 | 0.0256291367523809 | United States of America |
| 1990 | 49.50324678869 | 0.612104590576197 | 13.2170317295042 | 36.2862150591858 | Antigua and Barbuda |
| 1991 | 44.53768366303 | 0.618817128671612 | 12.9587064606831 | 31.5789772023469 | Antigua and Barbuda |
| 1992 | 43.99084463681 | 0.624695818117472 | 12.8874936872209 | 31.1033509495891 | Antigua and Barbuda |
| 1993 | 46.89591439739 | 0.630075853661038 | 12.0665452164845 | 34.8293691809055 | Antigua and Barbuda |
| 1994 | 45.58382287369 | 0.634768955380056 | 11.1197415856047 | 34.4640812880853 | Antigua and Barbuda |
| 1995 | 45.73356995592 | 0.638008628575253 | 8.10557752085135 | 37.6279924350686 | Antigua and Barbuda |
| 1996 | 44.84285519456 | 0.641647192231223 | 7.61203243450651 | 37.2308227600535 | Antigua and Barbuda |
| 1997 | 41.75636784498 | 0.645895590676441 | 5.96545788680553 | 35.7909099581745 | Antigua and Barbuda |
| 1998 | 38.03511829637 | 0.650614287301836 | 5.33146072937474 | 32.7036575669953 | Antigua and Barbuda |
| 1999 | 33.55822711303 | 0.655534994042462 | 4.94782642883676 | 28.6104006841932 | Antigua and Barbuda |
| 2000 | 29.09177617534 | 0.660819064213085 | 4.82969969512578 | 24.2620764802142 | Antigua and Barbuda |
| 2001 | 24.36227330514 | 0.665620631875941 | 4.3249710569907 | 20.0373022481493 | Antigua and Barbuda |
| 2002 | 22.34514785515 | 0.670360801218314 | 3.75155089416431 | 18.5935969609857 | Antigua and Barbuda |
| 2003 | 22.11644537271 | 0.67531148966572 | 3.16538351166582 | 18.9510618610442 | Antigua and Barbuda |
| 2004 | 22.19325877819 | 0.680412429142464 | 2.8280490471951 | 19.3652097309949 | Antigua and Barbuda |
| 2005 | 23.59181329716 | 0.685486437018442 | 2.80579233328734 | 20.7860209638727 | Antigua and Barbuda |
| 2006 | 23.58420296154 | 0.690826021180964 | 2.48389652805567 | 21.1003064334843 | Antigua and Barbuda |
| 2007 | 23.47679903792 | 0.69636003908324 | 2.42020086895945 | 21.0565981689605 | Antigua and Barbuda |
| 2008 | 22.32931172523 | 0.702175362144575 | 2.38085162554138 | 19.9484600996886 | Antigua and Barbuda |
| 2009 | 20.97967131684 | 0.707128563802673 | 2.30456467085617 | 18.6751066459838 | Antigua and Barbuda |
| 2010 | 20.23715022315 | 0.711432245950457 | 2.23168856711306 | 18.0054616560369 | Antigua and Barbuda |
| 2011 | 19.35145522603 | 0.715489989160124 | 2.21548353966022 | 17.1359716863698 | Antigua and Barbuda |
| 2012 | 18.68226347606 | 0.719509323328322 | 2.22066792806845 | 16.4615955479916 | Antigua and Barbuda |
| 2013 | 18.9665519683 | 0.722864977889834 | 2.18252652117131 | 16.7840254471287 | Antigua and Barbuda |
| 2014 | 19.97160861483 | 0.725585978309816 | 2.17529438371463 | 17.7963142311154 | Antigua and Barbuda |
| 2015 | 19.20103214667 | 0.728372762315346 | 2.17947953383839 | 17.0215526128316 | Antigua and Barbuda |
| 2016 | 18.67033533192 | 0.731598943677227 | 2.193184311714 | 16.477151020206 | Antigua and Barbuda |
| 2017 | 18.48296788903 | 0.734963713327539 | 2.14438163390137 | 16.3385862551286 | Antigua and Barbuda |
| 2018 | 18.01676258276 | 0.738879105607608 | 2.181773132865 | 15.834989449895 | Antigua and Barbuda |
| 2019 | 17.61494280526 | 0.743052960248658 | 2.11073800897558 | 15.5042047962844 | Antigua and Barbuda |
| 2020 | 16.47290883554 | 0.746345330021488 | 2.08585945805291 | 14.3870493774871 | Antigua and Barbuda |
| 2021 | 15.52698791134 | 0.749886887369379 | 2.06306399675639 | 13.4639239145836 | Antigua and Barbuda |
| 1990 | 61.95795412487 | 0.693509268349695 | 2.4134112006618 | 59.5445429242082 | Bahamas |
| 1991 | 65.7188095694 | 0.689126783528471 | 2.48149029924103 | 63.237319270159 | Bahamas |
| 1992 | 65.59042401835 | 0.68734383814569 | 2.74516280780183 | 62.8452612105482 | Bahamas |
| 1993 | 60.6074797229 | 0.693816501452916 | 2.45989041200917 | 58.1475893108908 | Bahamas |
| 1994 | 50.05484251203 | 0.706819891740348 | 2.31972774120403 | 47.735114770826 | Bahamas |
| 1995 | 42.91141003164 | 0.71938160672878 | 2.21628965280085 | 40.6951203788392 | Bahamas |
| 1996 | 37.15213488793 | 0.728478844477514 | 2.18976902884288 | 34.9623658590871 | Bahamas |
| 1997 | 34.13845762269 | 0.734781214819761 | 2.17741613203068 | 31.9610414906593 | Bahamas |
| 1998 | 31.27704978716 | 0.739281293356304 | 2.15621632008056 | 29.1208334670794 | Bahamas |
| 1999 | 28.54269054699 | 0.741622847933781 | 2.07744164391721 | 26.4652489030728 | Bahamas |
| 2000 | 25.30358324636 | 0.742517946132475 | 2.08785316986231 | 23.2157300764977 | Bahamas |
| 2001 | 21.02560504344 | 0.743592881699638 | 2.10226599577538 | 18.9233390476646 | Bahamas |
| 2002 | 19.50628583431 | 0.74625032205653 | 2.09403409548589 | 17.4122517388241 | Bahamas |
| 2003 | 18.52156630026 | 0.74991671397758 | 2.04504100185196 | 16.476525298408 | Bahamas |
| 2004 | 18.06423523746 | 0.753759643287481 | 2.02021542767352 | 16.0440198097865 | Bahamas |
| 2005 | 18.46037177252 | 0.756782661013009 | 1.95408382947465 | 16.5062879430454 | Bahamas |
| 2006 | 18.09475838797 | 0.759185938528465 | 1.91617780045969 | 16.1785805875103 | Bahamas |
| 2007 | 17.85138972842 | 0.761990996911577 | 1.72810517447849 | 16.1232845539415 | Bahamas |
| 2008 | 16.85813475089 | 0.765590503207612 | 1.77384586819677 | 15.0842888826932 | Bahamas |
| 2009 | 15.88166905382 | 0.769561673980251 | 1.59008304507268 | 14.2915860087473 | Bahamas |
| 2010 | 14.44020529454 | 0.774119809925167 | 1.59295765559483 | 12.8472476389452 | Bahamas |
| 2011 | 14.12178532063 | 0.778281890749903 | 1.50745538187348 | 12.6143299387565 | Bahamas |
| 2012 | 13.33846563601 | 0.781838639259066 | 1.472435216585 | 11.866030419425 | Bahamas |
| 2013 | 13.61961536148 | 0.784623910745264 | 1.44584572279909 | 12.1737696386809 | Bahamas |
| 2014 | 14.75912181211 | 0.787253492401512 | 1.44896073849523 | 13.3101610736148 | Bahamas |
| 2015 | 13.42879843168 | 0.789956145087734 | 1.46159249281611 | 11.9672059388639 | Bahamas |
| 2016 | 13.07725184178 | 0.792738695555901 | 1.44922529337772 | 11.6280265484023 | Bahamas |
| 2017 | 12.67390722959 | 0.795347180655707 | 1.41115418714019 | 11.2627530424498 | Bahamas |
| 2018 | 12.32562729044 | 0.798179034346548 | 1.46067291249448 | 10.8649543779455 | Bahamas |
| 2019 | 12.04036301252 | 0.801033070483021 | 1.42200543206846 | 10.6183575804515 | Bahamas |
| 2020 | 11.34900276117 | 0.802948018163753 | 1.47395735775261 | 9.87504540341739 | Bahamas |
| 2021 | 11.11780143396 | 0.805020667882124 | 1.47584750692085 | 9.64195392703915 | Bahamas |
| 1990 | 51.74942776415 | 0.653582516816854 | 4.95285167927176 | 46.7965760848782 | Barbados |
| 1991 | 50.37005621046 | 0.656654073238155 | 5.03765141653246 | 45.3324047939275 | Barbados |
| 1992 | 45.80205112111 | 0.662446635199289 | 4.53690942846133 | 41.2651416926487 | Barbados |
| 1993 | 42.81622307591 | 0.668815011829235 | 3.71483378037774 | 39.1013892955323 | Barbados |
| 1994 | 43.44802165541 | 0.673409365188399 | 3.43419383679792 | 40.0138278186121 | Barbados |
| 1995 | 44.08986433399 | 0.676012246725867 | 3.05142032555186 | 41.0384440084381 | Barbados |
| 1996 | 43.33192648662 | 0.677193053529371 | 2.9215248376973 | 40.4104016489227 | Barbados |
| 1997 | 37.76449059861 | 0.678644826009168 | 2.89725311377147 | 34.8672374848385 | Barbados |
| 1998 | 28.21790154546 | 0.679827167153174 | 2.86352114351846 | 25.3543804019415 | Barbados |
| 1999 | 24.51310309627 | 0.680398459240343 | 2.71786018158765 | 21.7952429146824 | Barbados |
| 2000 | 22.91412169954 | 0.681304198777811 | 2.90839739029209 | 20.0057243092479 | Barbados |
| 2001 | 17.38526922892 | 0.683329389407697 | 2.8614310799 | 14.52383814902 | Barbados |
| 2002 | 17.76660826028 | 0.68780757593531 | 2.59670282814049 | 15.1699054321395 | Barbados |
| 2003 | 20.36154604087 | 0.693235961982327 | 2.38623529404697 | 17.975310746823 | Barbados |
| 2004 | 16.37369854985 | 0.698029702584033 | 2.39631604017444 | 13.9773825096756 | Barbados |
| 2005 | 17.66164712432 | 0.701510049493398 | 2.33074644592067 | 15.3309006783993 | Barbados |
| 2006 | 17.7882641902 | 0.703856185878429 | 2.29043683260333 | 15.4978273575967 | Barbados |
| 2007 | 17.09078189461 | 0.705871649427123 | 2.31752815802951 | 14.7732537365805 | Barbados |
| 2008 | 16.64906654844 | 0.70851449513133 | 2.28142234667265 | 14.3676442017673 | Barbados |
| 2009 | 15.73411759858 | 0.712143361929882 | 2.22218354569741 | 13.5119340528826 | Barbados |
| 2010 | 15.33243150253 | 0.716208546340619 | 2.19233730676725 | 13.1400941957627 | Barbados |
| 2011 | 16.9215743446 | 0.720060687326812 | 2.20580400729095 | 14.715770337309 | Barbados |
| 2012 | 13.59086400643 | 0.724162457760429 | 2.19982253273821 | 11.3910414736918 | Barbados |
| 2013 | 13.91930262705 | 0.727826170485545 | 2.17230370029783 | 11.7469989267522 | Barbados |
| 2014 | 13.88010572871 | 0.730383286769139 | 2.16373251940053 | 11.7163732093095 | Barbados |
| 2015 | 13.7356508304 | 0.73260299258046 | 2.14830486762679 | 11.5873459627732 | Barbados |
| 2016 | 13.41248539561 | 0.73491253498334 | 2.15437721755754 | 11.2581081780525 | Barbados |
| 2017 | 13.24134993104 | 0.737157255293201 | 2.16029376771231 | 11.0810561633277 | Barbados |
| 2018 | 12.72418973371 | 0.739531436514553 | 2.15892255566127 | 10.5652671780487 | Barbados |
| 2019 | 12.49500453693 | 0.742239308607991 | 2.07989615864207 | 10.4151083782879 | Barbados |
| 2020 | 12.15254384169 | 0.74436664552373 | 2.05563421715633 | 10.0969096245337 | Barbados |
| 2021 | 12.06141670234 | 0.746748764251474 | 2.05383844941037 | 10.0075782529296 | Barbados |
| 1990 | 378.96370668617 | 0.423726992172569 | 232.721946113827 | 146.241760572343 | Belize |
| 1991 | 317.59092479134 | 0.433834392305066 | 201.620492419863 | 115.970432371477 | Belize |
| 1992 | 297.99251007435 | 0.444198507291524 | 115.817300131317 | 182.175209943033 | Belize |
| 1993 | 278.6426659435 | 0.455258576782351 | 70.9919119133569 | 207.650754030143 | Belize |
| 1994 | 240.03977066364 | 0.465729308156419 | 62.9030991222751 | 177.136671541365 | Belize |
| 1995 | 225.24506368442 | 0.475278511253692 | 56.1771609926793 | 169.067902691741 | Belize |
| 1996 | 213.79613931357 | 0.482702164726655 | 53.912178114848 | 159.883961198722 | Belize |
| 1997 | 204.4283381205 | 0.488020129434782 | 50.036995877124 | 154.391342243376 | Belize |
| 1998 | 190.52906617219 | 0.491819335269081 | 49.6015105169946 | 140.927555655195 | Belize |
| 1999 | 161.86717591778 | 0.495070195578772 | 49.2437571122304 | 112.62341880555 | Belize |
| 2000 | 134.97766145401 | 0.498643730395849 | 24.4822665117234 | 110.495394942287 | Belize |
| 2001 | 108.71188074082 | 0.502399433843715 | 23.4477573473959 | 85.2641233934241 | Belize |
| 2002 | 93.16452144514 | 0.506871655304811 | 22.7888931563724 | 70.3756282887676 | Belize |
| 2003 | 94.42998843439 | 0.512679459131973 | 22.4712730790489 | 71.9587153553411 | Belize |
| 2004 | 90.30849794868 | 0.519697333321487 | 22.2437643606956 | 68.0647335879844 | Belize |
| 2005 | 94.59589251792 | 0.527231061781742 | 22.1767266294113 | 72.4191658885087 | Belize |
| 2006 | 86.36981141145 | 0.534889621081104 | 21.5907562576636 | 64.7790551537864 | Belize |
| 2007 | 79.39701711792 | 0.541900694400229 | 21.1637745727733 | 58.2332425451467 | Belize |
| 2008 | 74.98097628395 | 0.548093223354439 | 20.5903619128194 | 54.3906143711306 | Belize |
| 2009 | 70.68730051081 | 0.553465869255233 | 19.6000688214334 | 51.0872316893766 | Belize |
| 2010 | 60.54794302603 | 0.558546893327125 | 17.5912593787812 | 42.9566836472488 | Belize |
| 2011 | 57.71493332615 | 0.563440362640477 | 16.0427463628329 | 41.6721869633171 | Belize |
| 2012 | 51.5389651941 | 0.568456235800016 | 14.2571940625058 | 37.2817711315942 | Belize |
| 2013 | 50.12931478121 | 0.573236457430755 | 13.2305117803296 | 36.8988030008804 | Belize |
| 2014 | 52.38803212018 | 0.578091325788653 | 13.2729794668256 | 39.1150526533544 | Belize |
| 2015 | 51.53497854609 | 0.583279094493031 | 13.3657967408895 | 38.1691818052005 | Belize |
| 2016 | 50.33779701449 | 0.588275912195168 | 13.069687120488 | 37.268109894002 | Belize |
| 2017 | 48.01561764944 | 0.593337290122785 | 13.2679321503575 | 34.7476854990826 | Belize |
| 2018 | 46.64160739288 | 0.598512014950432 | 13.5069609639298 | 33.1346464289502 | Belize |
| 2019 | 44.19871194638 | 0.603351601174544 | 13.4585626630141 | 30.7401492833659 | Belize |
| 2020 | 42.32867383303 | 0.607060046133776 | 12.9237763735261 | 29.4048974595039 | Belize |
| 2021 | 40.30428039392 | 0.610229002222948 | 13.2201512921303 | 27.0841291017897 | Belize |
| 1990 | 66.78821900723 | 0.558019070765374 | 18.0776893828979 | 48.7105296243321 | Cuba |
| 1991 | 58.6528052159 | 0.563413638292236 | 16.0916765618411 | 42.5611286540589 | Cuba |
| 1992 | 49.58661474882 | 0.566169005573441 | 15.1045862514873 | 34.4820284973327 | Cuba |
| 1993 | 50.15321949772 | 0.565441046515952 | 15.0802325108609 | 35.0729869868591 | Cuba |
| 1994 | 51.95724358846 | 0.563186312783606 | 16.8721875084932 | 35.0850560799668 | Cuba |
| 1995 | 49.96412216993 | 0.560765902424453 | 16.3750423971717 | 33.5890797727583 | Cuba |
| 1996 | 40.38276149437 | 0.559346328872574 | 18.2057977871727 | 22.1769637071973 | Cuba |
| 1997 | 36.47834320724 | 0.558924568054562 | 17.5774939159692 | 18.9008492912708 | Cuba |
| 1998 | 33.00763569856 | 0.559363220275698 | 17.5180477232617 | 15.4895879752983 | Cuba |
| 1999 | 27.9695408514 | 0.561820952733584 | 16.943195549601 | 11.026345301799 | Cuba |
| 2000 | 24.04179601054 | 0.566676901631981 | 15.3515943153811 | 8.69020169515892 | Cuba |
| 2001 | 18.90837286288 | 0.572753860839461 | 13.3937574502777 | 5.51461541260226 | Cuba |
| 2002 | 17.09288728413 | 0.579968290233664 | 13.4380918689213 | 3.65479541520873 | Cuba |
| 2003 | 17.62430682903 | 0.587647761532625 | 13.4878462721019 | 4.13646055692814 | Cuba |
| 2004 | 16.76772173419 | 0.594348715355523 | 13.2107657937904 | 3.55695594039958 | Cuba |
| 2005 | 18.81949606985 | 0.599863798514665 | 13.3263667673777 | 5.49312930247231 | Cuba |
| 2006 | 17.22217586282 | 0.604970567275803 | 13.2958827032489 | 3.92629315957109 | Cuba |
| 2007 | 16.55788858155 | 0.607618098577978 | 13.3326307748254 | 3.2252578067246 | Cuba |
| 2008 | 17.20684070829 | 0.60940092330195 | 13.3064401740984 | 3.90040053419163 | Cuba |
| 2009 | 16.65041565119 | 0.611775641002492 | 13.0588214537381 | 3.59159419745186 | Cuba |
| 2010 | 14.86020921953 | 0.615261262451076 | 13.0782699488231 | 1.78193927070685 | Cuba |
| 2011 | 14.76121195832 | 0.620386689831088 | 13.0409113136227 | 1.7203006446973 | Cuba |
| 2012 | 14.72316826516 | 0.626156024890616 | 13.0086304325222 | 1.71453783263778 | Cuba |
| 2013 | 13.78680102642 | 0.631839801039436 | 11.9222649022722 | 1.86453612414775 | Cuba |
| 2014 | 14.52429279949 | 0.637304129079553 | 8.73331655231857 | 5.79097624717143 | Cuba |
| 2015 | 13.08110378389 | 0.642818963057111 | 7.76994055011224 | 5.31116323377776 | Cuba |
| 2016 | 12.16691547324 | 0.647907866261711 | 6.36121582206949 | 5.80569965117051 | Cuba |
| 2017 | 12.63998212503 | 0.652309286413179 | 4.95041594786377 | 7.68956617716623 | Cuba |
| 2018 | 12.72411750796 | 0.65685865401747 | 5.02978323606783 | 7.69433427189217 | Cuba |
| 2019 | 14.09557013899 | 0.661674716522849 | 4.48604871587804 | 9.60952142311196 | Cuba |
| 2020 | 11.91427435198 | 0.665210747223401 | 4.38219485946101 | 7.53207949251899 | Cuba |
| 2021 | 10.39224407812 | 0.668729864363244 | 3.39866565938944 | 6.99357841873056 | Cuba |
| 1990 | 70.90592092621 | 0.563602590366912 | 16.100243057132 | 54.805677869078 | Dominica |
| 1991 | 69.7595096513 | 0.565237843041271 | 15.3080453348885 | 54.4514643164115 | Dominica |
| 1992 | 67.72806076641 | 0.569570683969256 | 14.1165191233867 | 53.6115416430233 | Dominica |
| 1993 | 64.81584321364 | 0.578811810513113 | 13.3073893627942 | 51.5084538508458 | Dominica |
| 1994 | 62.40457025774 | 0.590119147153383 | 13.5130848843922 | 48.8914853733477 | Dominica |
| 1995 | 58.71805610574 | 0.601986931512718 | 13.1231988657263 | 45.5948572400137 | Dominica |
| 1996 | 55.51058883009 | 0.614382049775589 | 13.2797092595466 | 42.2308795705434 | Dominica |
| 1997 | 52.67979456047 | 0.625057352694054 | 12.8400879224261 | 39.8397066380439 | Dominica |
| 1998 | 50.08263613396 | 0.63301799374648 | 10.8768501329098 | 39.2057860010502 | Dominica |
| 1999 | 49.1579639237 | 0.63945391525062 | 7.49960836627716 | 41.6583555574228 | Dominica |
| 2000 | 46.00142194426 | 0.645830936825434 | 6.99432732925087 | 39.0070946150091 | Dominica |
| 2001 | 45.03682073311 | 0.652368567094588 | 5.11501922745881 | 39.9218015056512 | Dominica |
| 2002 | 42.69143538197 | 0.658423554407679 | 5.02154462014588 | 37.6698907618241 | Dominica |
| 2003 | 42.20180976365 | 0.663678867784012 | 4.49433284739629 | 37.7074769162537 | Dominica |
| 2004 | 40.74649624159 | 0.668952131656395 | 3.66352715799324 | 37.0829690835968 | Dominica |
| 2005 | 40.25122358048 | 0.67454907679023 | 3.16984979666838 | 37.0813737838116 | Dominica |
| 2006 | 39.08403021222 | 0.680342481749478 | 2.81655149111938 | 36.2674787211006 | Dominica |
| 2007 | 40.03889327648 | 0.685415917499458 | 2.70247194880471 | 37.3364213276753 | Dominica |
| 2008 | 39.30417757338 | 0.69019751901742 | 2.50972033543653 | 36.7944572379435 | Dominica |
| 2009 | 42.40116849172 | 0.694461869014732 | 2.44853199023524 | 39.9526365014848 | Dominica |
| 2010 | 44.36315497162 | 0.698489193418408 | 2.33734830514618 | 42.0258066664738 | Dominica |
| 2011 | 47.71054924086 | 0.702463260148908 | 2.37211089848452 | 45.3384383423755 | Dominica |
| 2012 | 49.19595758866 | 0.706870607879807 | 2.27284373285775 | 46.9231138558023 | Dominica |
| 2013 | 51.393850738 | 0.71221047502743 | 2.23252774258532 | 49.1613229954147 | Dominica |
| 2014 | 51.5098023829 | 0.718455556016605 | 2.20889138568709 | 49.3009109972129 | Dominica |
| 2015 | 50.86339265355 | 0.724785536415403 | 2.17729715855311 | 48.6860954949969 | Dominica |
| 2016 | 49.58484834562 | 0.73176080052363 | 2.17583830834246 | 47.4090100372775 | Dominica |
| 2017 | 49.5440315358 | 0.736062472571312 | 2.16263344799 | 47.38139808781 | Dominica |
| 2018 | 50.07435121361 | 0.738935953651369 | 2.15041613937242 | 47.9239350742376 | Dominica |
| 2019 | 50.93087279339 | 0.74213206575999 | 2.08789185058681 | 48.8429809428032 | Dominica |
| 2020 | 51.51271801032 | 0.744419943891979 | 2.06454204666855 | 49.4481759636515 | Dominica |
| 2021 | 51.81548833421 | 0.746967184749286 | 2.0702248512848 | 49.7452634829252 | Dominica |
| 1990 | 906.22662689297 | 0.442654076123481 | 107.790018829473 | 798.436608063497 | Dominican Republic |
| 1991 | 843.81868245609 | 0.446144577831215 | 77.8857921560378 | 765.932890300052 | Dominican Republic |
| 1992 | 775.63927760373 | 0.449953080528465 | 71.370269479873 | 704.269008123857 | Dominican Republic |
| 1993 | 714.58333288974 | 0.454229297152068 | 69.4401011677749 | 645.143231721965 | Dominican Republic |
| 1994 | 645.86179279287 | 0.458620613654854 | 63.6267649809672 | 582.235027811903 | Dominican Republic |
| 1995 | 584.85364171005 | 0.463313869288046 | 60.8402026187142 | 524.013439091336 | Dominican Republic |
| 1996 | 519.99921462969 | 0.468516938419476 | 57.8583261700636 | 462.140888459626 | Dominican Republic |
| 1997 | 462.38462482235 | 0.474504388639617 | 58.3496697282749 | 404.034955094075 | Dominican Republic |
| 1998 | 405.73967261113 | 0.481170939346086 | 54.8760724838387 | 350.863600127291 | Dominican Republic |
| 1999 | 358.65421838128 | 0.488178541467036 | 50.1605254285434 | 308.493692952737 | Dominican Republic |
| 2000 | 326.73706416142 | 0.495468586151319 | 50.3626007335194 | 276.374463427901 | Dominican Republic |
| 2001 | 307.77377270802 | 0.50277541519763 | 23.0452121647407 | 284.728560543279 | Dominican Republic |
| 2002 | 282.86672265193 | 0.510298018698881 | 22.5110016742918 | 260.355720977638 | Dominican Republic |
| 2003 | 265.79551420309 | 0.517395574464313 | 22.3433024340859 | 243.452211769004 | Dominican Republic |
| 2004 | 259.98550021727 | 0.524543274827677 | 22.1575096040431 | 237.827990613227 | Dominican Republic |
| 2005 | 253.4827424987 | 0.532228649477556 | 22.1396334311013 | 231.343109067599 | Dominican Republic |
| 2006 | 234.04153683723 | 0.540222689966408 | 21.1983654637033 | 212.843171373527 | Dominican Republic |
| 2007 | 215.60240499246 | 0.548187021157434 | 20.6517157739058 | 194.950689218554 | Dominican Republic |
| 2008 | 196.41884030892 | 0.555371313172153 | 18.6706418573856 | 177.748198451534 | Dominican Republic |
| 2009 | 183.12306793782 | 0.561783754117237 | 16.4330923032532 | 166.689975634567 | Dominican Republic |
| 2010 | 179.24399412766 | 0.568130581856948 | 14.5561757745214 | 164.687818353139 | Dominican Republic |
| 2011 | 208.29229564594 | 0.573834812622029 | 13.4243204802298 | 194.86797516571 | Dominican Republic |
| 2012 | 211.93383098218 | 0.578966380988324 | 13.5171731449176 | 198.416657837262 | Dominican Republic |
| 2013 | 167.41979546913 | 0.583625825103645 | 13.3490884161417 | 154.070707052988 | Dominican Republic |
| 2014 | 151.97348227958 | 0.588236742676906 | 13.5124245629585 | 138.461057716621 | Dominican Republic |
| 2015 | 136.86500458627 | 0.592924164325808 | 13.2878223379256 | 123.577182248344 | Dominican Republic |
| 2016 | 132.09006446475 | 0.597619609207884 | 13.3560123625423 | 118.734052102208 | Dominican Republic |
| 2017 | 122.43009615772 | 0.602140291257967 | 13.2180520261371 | 109.212044131583 | Dominican Republic |
| 2018 | 116.83252521756 | 0.606960838531609 | 13.3656515534835 | 103.466873664077 | Dominican Republic |
| 2019 | 111.82356805059 | 0.611913021025697 | 13.1279115805874 | 98.6956564700026 | Dominican Republic |
| 2020 | 105.59809795549 | 0.615635625027175 | 13.0138071763811 | 92.5842907791089 | Dominican Republic |
| 2021 | 104.65015399174 | 0.619388201281128 | 12.9115634128425 | 91.7385905788975 | Dominican Republic |
| 1990 | 66.22689409147 | 0.436734419397497 | 66.22689409147 | 0 | Grenada |
| 1991 | 55.38730678904 | 0.445887952925777 | 55.38730678904 | 0 | Grenada |
| 1992 | 54.41878651112 | 0.455910058376885 | 54.41878651112 | 0 | Grenada |
| 1993 | 54.78232817164 | 0.465994889867929 | 54.604885218291 | 0.17744295334905 | Grenada |
| 1994 | 50.07788474261 | 0.476410340600106 | 50.07788474261 | 0 | Grenada |
| 1995 | 47.00248232046 | 0.486558998595925 | 47.00248232046 | 0 | Grenada |
| 1996 | 44.7873411419 | 0.496589807805192 | 27.0288074567277 | 17.7585336851723 | Grenada |
| 1997 | 41.5080995223 | 0.50621760158124 | 22.8630093521874 | 18.6450901701126 | Grenada |
| 1998 | 35.38471715206 | 0.516590019085488 | 22.3088345509122 | 13.0758826011478 | Grenada |
| 1999 | 25.39461065028 | 0.527333411440571 | 22.1381874950015 | 3.25642315527848 | Grenada |
| 2000 | 21.5789682553 | 0.538388116408646 | 21.2706266318813 | 0.308341623418727 | Grenada |
| 2001 | 20.32268702349 | 0.549058969647808 | 20.131420085427 | 0.191266938063002 | Grenada |
| 2002 | 18.60498945708 | 0.559473127591996 | 17.2761305117569 | 1.32885894532312 | Grenada |
| 2003 | 18.45221763196 | 0.569718037363691 | 14.1579176773058 | 4.29429995465415 | Grenada |
| 2004 | 18.2040783406 | 0.578487456332464 | 13.3365860577408 | 4.8674922828592 | Grenada |
| 2005 | 19.18327549076 | 0.587158876770892 | 13.5302033761438 | 5.65307211461617 | Grenada |
| 2006 | 19.27701855622 | 0.59405451978646 | 13.4565355332606 | 5.82048302295941 | Grenada |
| 2007 | 18.50968228143 | 0.600766335502731 | 13.4181423174628 | 5.09153996396724 | Grenada |
| 2008 | 17.99978934517 | 0.606879281941233 | 13.4265186304845 | 4.57327071468545 | Grenada |
| 2009 | 17.04057077759 | 0.61180525701265 | 13.3153712208263 | 3.72519955676366 | Grenada |
| 2010 | 15.94477726301 | 0.616486997376644 | 13.2281133704169 | 2.71666389259311 | Grenada |
| 2011 | 16.06693341067 | 0.620978521316098 | 12.8775703791477 | 3.18936303152226 | Grenada |
| 2012 | 15.58883776453 | 0.624977512249081 | 12.8069773363835 | 2.78186042814649 | Grenada |
| 2013 | 15.58079261455 | 0.629048335732456 | 12.5810767303209 | 2.99971588422909 | Grenada |
| 2014 | 16.89583898432 | 0.633734695952301 | 11.0464982537736 | 5.8493407305464 | Grenada |
| 2015 | 15.69298853295 | 0.638936083726672 | 7.52953646409015 | 8.16345206885985 | Grenada |
| 2016 | 14.57328405419 | 0.644335233932856 | 6.04630236910055 | 8.52698168508945 | Grenada |
| 2017 | 13.66723954798 | 0.649831521980215 | 6.02481142830281 | 7.64242811967719 | Grenada |
| 2018 | 13.34029543668 | 0.655372772077438 | 5.13545457480793 | 8.20484086187207 | Grenada |
| 2019 | 12.67135998815 | 0.660808511595714 | 4.63400328972037 | 8.03735669842963 | Grenada |
| 2020 | 12.19223792371 | 0.665086346623039 | 4.48328227128907 | 7.70895565242093 | Grenada |
| 2021 | 11.49143962364 | 0.668993028326993 | 3.74605797863762 | 7.74538164500238 | Grenada |
| 1990 | 644.6237902576 | 0.460430128793009 | 60.5050800479365 | 584.118710209664 | Guyana |
| 1991 | 576.42758974745 | 0.462677068801868 | 63.2822991589598 | 513.14529058849 | Guyana |
| 1992 | 545.02444224566 | 0.466753989262645 | 56.165683254984 | 488.858758990676 | Guyana |
| 1993 | 534.50358627229 | 0.472174228924914 | 57.1829907968912 | 477.320595475399 | Guyana |
| 1994 | 494.59369750918 | 0.478992924260878 | 52.4349595914258 | 442.158737917754 | Guyana |
| 1995 | 458.59544636221 | 0.486573457084383 | 50.3113564725079 | 408.284089889702 | Guyana |
| 1996 | 416.71930921099 | 0.495216036467396 | 51.2336396679562 | 365.485669543034 | Guyana |
| 1997 | 400.99337979462 | 0.504362778751228 | 22.982887053293 | 378.010492741327 | Guyana |
| 1998 | 381.54768700704 | 0.513068898204645 | 22.5093267319365 | 359.038360275104 | Guyana |
| 1999 | 409.51886655516 | 0.521595978096732 | 22.159322034812 | 387.359544520348 | Guyana |
| 2000 | 487.24961812105 | 0.528693991503479 | 22.1557897810587 | 465.093828339991 | Guyana |
| 2001 | 358.86298302773 | 0.534869805890103 | 21.5235648515336 | 337.339418176196 | Guyana |
| 2002 | 298.15412684832 | 0.54051427331456 | 21.2167586421446 | 276.937368206175 | Guyana |
| 2003 | 270.32762891571 | 0.545516473248843 | 21.0303532889583 | 249.297275626752 | Guyana |
| 2004 | 246.18885727986 | 0.550326370085983 | 19.6757965850622 | 226.513060694798 | Guyana |
| 2005 | 271.77414123002 | 0.554844797400739 | 18.5495788447345 | 253.224562385286 | Guyana |
| 2006 | 255.83840590697 | 0.55946561076404 | 17.9757772985789 | 237.862628608391 | Guyana |
| 2007 | 218.51659424626 | 0.564541135103012 | 15.97779346089 | 202.53880078537 | Guyana |
| 2008 | 200.67205702004 | 0.569595205505363 | 14.2471385107045 | 186.424918509335 | Guyana |
| 2009 | 204.59284848728 | 0.574769931497353 | 13.4919152635933 | 191.100933223687 | Guyana |
| 2010 | 180.17190864361 | 0.580083911433493 | 13.5249310270659 | 166.646977616544 | Guyana |
| 2011 | 157.50199548415 | 0.585688269016646 | 13.2275709942538 | 144.274424489896 | Guyana |
| 2012 | 144.74170714411 | 0.591336582127807 | 13.4136256122034 | 131.328081531907 | Guyana |
| 2013 | 136.15817498105 | 0.596826294655764 | 13.1352432581148 | 123.022931722935 | Guyana |
| 2014 | 133.32734091286 | 0.602136510795571 | 13.4267737598408 | 119.900567153019 | Guyana |
| 2015 | 123.12930476303 | 0.60760548677096 | 13.1918299339868 | 109.937474829043 | Guyana |
| 2016 | 116.23451988107 | 0.613914359668043 | 13.3615238922629 | 102.872995988807 | Guyana |
| 2017 | 110.95713691797 | 0.620602607413008 | 13.1227140207115 | 97.8344228972585 | Guyana |
| 2018 | 110.0737434652 | 0.627167040808132 | 13.0565683730601 | 97.0171750921399 | Guyana |
| 2019 | 96.11595101528 | 0.633640346503077 | 11.011700200126 | 85.104250815154 | Guyana |
| 2020 | 86.79256782685 | 0.642284644781673 | 7.86144270309323 | 78.9311251237568 | Guyana |
| 2021 | 79.96858783446 | 0.650812335285137 | 5.739776566523 | 74.228811267937 | Guyana |
| 1990 | 2973.43129389051 | 0.310334630409749 | 961.478617071245 | 2011.95267681926 | Haiti |
| 1991 | 2816.75489892787 | 0.315455441278148 | 838.581590379301 | 1978.17330854857 | Haiti |
| 1992 | 2724.57801355937 | 0.320389978747448 | 851.371212766622 | 1873.20680079275 | Haiti |
| 1993 | 2619.37116955576 | 0.324895850993773 | 765.615229393729 | 1853.75594016203 | Haiti |
| 1994 | 2520.5925803353 | 0.328399455968527 | 745.107593438138 | 1775.48498689716 | Haiti |
| 1995 | 2419.58968749435 | 0.332999151562276 | 685.139915979397 | 1734.44977151495 | Haiti |
| 1996 | 2280.13297135224 | 0.33822939619677 | 597.107421919553 | 1683.02554943269 | Haiti |
| 1997 | 2159.87555777065 | 0.343880049177945 | 599.760506756676 | 1560.11505101397 | Haiti |
| 1998 | 1995.6641871751 | 0.349698073003298 | 596.897688987571 | 1398.76649818753 | Haiti |
| 1999 | 1886.64574899205 | 0.355980742770846 | 591.187014385416 | 1295.45873460663 | Haiti |
| 2000 | 1753.28634635281 | 0.36257220895481 | 575.00417936411 | 1178.2821669887 | Haiti |
| 2001 | 1671.68270857436 | 0.368643507933658 | 574.821210746317 | 1096.86149782804 | Haiti |
| 2002 | 1598.28258142431 | 0.374473324901094 | 545.497981020284 | 1052.78460040403 | Haiti |
| 2003 | 1540.37978540642 | 0.380067254956705 | 545.07367941776 | 995.30610598866 | Haiti |
| 2004 | 1488.80790967323 | 0.385061511883196 | 512.0636723898 | 976.74423728343 | Haiti |
| 2005 | 1435.9582885757 | 0.389845419506401 | 509.320125914561 | 926.638162661139 | Haiti |
| 2006 | 1355.68451171532 | 0.394322001247404 | 456.060848934812 | 899.623662780508 | Haiti |
| 2007 | 1282.62259472925 | 0.39897344059179 | 459.601399115128 | 823.021195614122 | Haiti |
| 2008 | 1203.53429536673 | 0.40328587122688 | 392.308878723895 | 811.225416642835 | Haiti |
| 2009 | 1130.68548114339 | 0.407944423962943 | 312.256335443609 | 818.429145699781 | Haiti |
| 2010 | 1366.25280491718 | 0.411840105174603 | 299.805422612547 | 1066.44738230463 | Haiti |
| 2011 | 2175.45602618186 | 0.416177190225703 | 274.332389979566 | 1901.12363620229 | Haiti |
| 2012 | 1043.60455134069 | 0.420021527912646 | 243.296873502056 | 800.307677838634 | Haiti |
| 2013 | 1120.26495360921 | 0.423985549390571 | 233.695889047346 | 886.569064561864 | Haiti |
| 2014 | 982.60870188616 | 0.427940902171937 | 211.972407992871 | 770.636293893289 | Haiti |
| 2015 | 918.13661820338 | 0.431756760061604 | 208.246883164349 | 709.889735039031 | Haiti |
| 2016 | 950.1992477888 | 0.435288722267081 | 184.2953679012 | 765.9038798876 | Haiti |
| 2017 | 848.38275079532 | 0.438541003090222 | 115.769851452576 | 732.612899342744 | Haiti |
| 2018 | 825.67183321736 | 0.44167892537118 | 111.003941462422 | 714.667891754938 | Haiti |
| 2019 | 804.99842818291 | 0.444328853815805 | 107.637752706009 | 697.360675476901 | Haiti |
| 2020 | 775.89281566431 | 0.446390613582292 | 71.1902717727784 | 704.702543891531 | Haiti |
| 2021 | 757.67101699263 | 0.448278284992742 | 76.0732965879245 | 681.597720404705 | Haiti |
| 1990 | 238.1335126469 | 0.534781233684701 | 21.5547982280506 | 216.578714418849 | Jamaica |
| 1991 | 205.09954550917 | 0.539594479788248 | 21.2168105248478 | 183.882734984322 | Jamaica |
| 1992 | 186.04315150478 | 0.545088752562521 | 21.1717235101045 | 164.871427994675 | Jamaica |
| 1993 | 165.28360840643 | 0.551218491600381 | 19.6681661931863 | 145.615442213244 | Jamaica |
| 1994 | 145.39420016023 | 0.557643773203113 | 17.6753702442725 | 127.718829915957 | Jamaica |
| 1995 | 115.3650231636 | 0.564315037773069 | 16.0823988141804 | 99.2826243494196 | Jamaica |
| 1996 | 71.71870063071 | 0.570940131286319 | 13.2172819664388 | 58.5014186642712 | Jamaica |
| 1997 | 55.17415711972 | 0.577212091097575 | 13.4169592765359 | 41.7571978431841 | Jamaica |
| 1998 | 49.0251991665 | 0.58304803468123 | 13.3973893817733 | 35.6278097847267 | Jamaica |
| 1999 | 44.36608675812 | 0.588846467179198 | 13.522696223132 | 30.843390534988 | Jamaica |
| 2000 | 40.22126011565 | 0.594592916825086 | 13.3680430613474 | 26.8532170543026 | Jamaica |
| 2001 | 31.90668875809 | 0.600192022232271 | 13.4021267096958 | 18.5045620483942 | Jamaica |
| 2002 | 29.40741105576 | 0.605591748141932 | 13.4583493843085 | 15.9490616714515 | Jamaica |
| 2003 | 30.74165455915 | 0.610877423593022 | 13.3250121104175 | 17.4166424487325 | Jamaica |
| 2004 | 29.63517675221 | 0.615836278207827 | 12.981509740675 | 16.653667011535 | Jamaica |
| 2005 | 30.09814975513 | 0.620400056664326 | 12.9479488654516 | 17.1502008896784 | Jamaica |
| 2006 | 35.08807920728 | 0.625018859668551 | 12.8679266401208 | 22.2201525671592 | Jamaica |
| 2007 | 33.68387166627 | 0.629549469674028 | 11.8923389046358 | 21.7915327616342 | Jamaica |
| 2008 | 32.02967245793 | 0.633967851311631 | 10.948776861247 | 21.080895596683 | Jamaica |
| 2009 | 31.27849624252 | 0.638110835001433 | 8.73773952735254 | 22.5407567151675 | Jamaica |
| 2010 | 29.76381376707 | 0.642129387979891 | 8.14873692928643 | 21.6150768377836 | Jamaica |
| 2011 | 29.80532542261 | 0.646395830061733 | 5.470532627815 | 24.334792794795 | Jamaica |
| 2012 | 29.65863057012 | 0.650583760450656 | 5.50761039737172 | 24.1510201727483 | Jamaica |
| 2013 | 27.15355596307 | 0.654573624159258 | 4.93258685214513 | 22.2209691109249 | Jamaica |
| 2014 | 32.40278120419 | 0.658505341253061 | 4.81596812417656 | 27.5868130800134 | Jamaica |
| 2015 | 30.60734825356 | 0.662465809295028 | 4.55167084138091 | 26.0556774121791 | Jamaica |
| 2016 | 29.42109525001 | 0.666469278740004 | 3.60596217562929 | 25.8151330743807 | Jamaica |
| 2017 | 28.2974923025 | 0.670291045200821 | 3.69501374349485 | 24.6024785590052 | Jamaica |
| 2018 | 27.18790298767 | 0.674075312952044 | 3.11562340405 | 24.07227958362 | Jamaica |
| 2019 | 26.26436051257 | 0.677778539230129 | 2.92421876718508 | 23.3401417453849 | Jamaica |
| 2020 | 25.20239164137 | 0.680567176976565 | 2.87285176691699 | 22.329539874453 | Jamaica |
| 2021 | 23.44862558695 | 0.683263063531091 | 2.74188518482181 | 20.7067404021282 | Jamaica |
| 1990 | 125.95531593579 | 0.496296570103573 | 36.06215341606 | 89.89316251973 | Saint Lucia |
| 1991 | 109.33892830392 | 0.505975554986447 | 23.0956868753554 | 86.2432414285646 | Saint Lucia |
| 1992 | 101.33747847759 | 0.515368260657711 | 22.1962587574081 | 79.1412197201819 | Saint Lucia |
| 1993 | 104.19447387927 | 0.524234949005948 | 22.1537215802004 | 82.0407522990696 | Saint Lucia |
| 1994 | 92.93436613732 | 0.532731082607556 | 22.1263720189262 | 70.8079941183938 | Saint Lucia |
| 1995 | 86.4720374554 | 0.541479793625538 | 21.2135997344688 | 65.2584377209312 | Saint Lucia |
| 1996 | 80.11470698087 | 0.550242411723114 | 19.7245182377704 | 60.3901887430996 | Saint Lucia |
| 1997 | 73.23430192734 | 0.558293460901167 | 17.8931809902262 | 55.3411209371138 | Saint Lucia |
| 1998 | 61.4323836913 | 0.566138702625704 | 15.2172459088652 | 46.2151377824348 | Saint Lucia |
| 1999 | 51.14572295964 | 0.573428555367193 | 13.4246529680135 | 37.7210699916265 | Saint Lucia |
| 2000 | 42.44497281786 | 0.579788896229167 | 13.6397896141635 | 28.8051832036965 | Saint Lucia |
| 2001 | 35.28859444701 | 0.585087557754993 | 13.1376851876097 | 22.1509092594003 | Saint Lucia |
| 2002 | 31.73181317309 | 0.590167578497987 | 13.3779389715426 | 18.3538742015474 | Saint Lucia |
| 2003 | 31.22211522797 | 0.595675145724796 | 13.4481835134022 | 17.7739317145678 | Saint Lucia |
| 2004 | 30.94166577291 | 0.601625481597697 | 13.5820429102109 | 17.3596228626991 | Saint Lucia |
| 2005 | 33.59796236677 | 0.60723986604169 | 13.3951576678415 | 20.2028046989285 | Saint Lucia |
| 2006 | 32.38592468163 | 0.612755299779844 | 13.1919063827741 | 19.1940182988559 | Saint Lucia |
| 2007 | 31.86450584485 | 0.617994714022193 | 12.8971251843818 | 18.9673806604682 | Saint Lucia |
| 2008 | 31.29659957607 | 0.62327300064738 | 13.07967261128 | 18.21692696479 | Saint Lucia |
| 2009 | 28.99344777933 | 0.628169848746241 | 12.532100247351 | 16.461347531979 | Saint Lucia |
| 2010 | 26.53158700858 | 0.632782904571138 | 11.3363586641807 | 15.1952283443993 | Saint Lucia |
| 2011 | 27.05385867776 | 0.637439441748952 | 8.04695277484835 | 19.0069059029116 | Saint Lucia |
| 2012 | 26.04733467138 | 0.641567127255833 | 7.10317607255186 | 18.9441585988281 | Saint Lucia |
| 2013 | 26.06418610208 | 0.645153079091964 | 6.41010178076762 | 19.6540843213124 | Saint Lucia |
| 2014 | 28.25414538944 | 0.648743506935936 | 5.48627888178033 | 22.7678665076597 | Saint Lucia |
| 2015 | 27.01269811428 | 0.652157480490287 | 5.20450030301831 | 21.8081978112617 | Saint Lucia |
| 2016 | 25.3198847656 | 0.655653340205716 | 4.83515418708233 | 20.4847305785177 | Saint Lucia |
| 2017 | 24.1599535126 | 0.659341345742569 | 4.56055658049492 | 19.5993969321051 | Saint Lucia |
| 2018 | 23.08478865346 | 0.663086681831917 | 4.69218223946815 | 18.3926064139918 | Saint Lucia |
| 2019 | 22.20472519874 | 0.666840358993444 | 3.55452253979824 | 18.6502026589418 | Saint Lucia |
| 2020 | 20.61220892578 | 0.669720489592338 | 3.4480259214234 | 17.1641830043566 | Saint Lucia |
| 2021 | 20.10221217185 | 0.672509735365292 | 3.50337319870943 | 16.5988389731406 | Saint Lucia |
| 1990 | 243.48851198475 | 0.475930186469041 | 56.4571950756383 | 187.031316909112 | Saint Vincent and the Grenadines |
| 1991 | 225.86477322473 | 0.481075658352439 | 53.1719054543075 | 172.692867770422 | Saint Vincent and the Grenadines |
| 1992 | 204.57713272389 | 0.485977871294048 | 53.5075018983029 | 151.069630825587 | Saint Vincent and the Grenadines |
| 1993 | 191.60806128716 | 0.490998790902684 | 49.8972196384862 | 141.710841648674 | Saint Vincent and the Grenadines |
| 1994 | 175.43022877324 | 0.496379132267915 | 36.0914097355935 | 139.338819037647 | Saint Vincent and the Grenadines |
| 1995 | 158.26438955989 | 0.502986089658165 | 23.1622419364931 | 135.102147623397 | Saint Vincent and the Grenadines |
| 1996 | 144.09107794702 | 0.509527157829315 | 22.7483840213987 | 121.342693925621 | Saint Vincent and the Grenadines |
| 1997 | 129.91535688815 | 0.51548261862783 | 22.2586922238969 | 107.656664664253 | Saint Vincent and the Grenadines |
| 1998 | 113.08190168751 | 0.52120967882065 | 22.144836204019 | 90.937065483491 | Saint Vincent and the Grenadines |
| 1999 | 96.26904911856 | 0.527099551882504 | 22.1656575204375 | 74.1033915981225 | Saint Vincent and the Grenadines |
| 2000 | 80.61134797316 | 0.533173289927656 | 22.134618162142 | 58.476729811018 | Saint Vincent and the Grenadines |
| 2001 | 69.14722251522 | 0.539455711639871 | 21.168332306359 | 47.978890208861 | Saint Vincent and the Grenadines |
| 2002 | 58.94197197867 | 0.546063615669324 | 20.5288821492975 | 38.4130898293725 | Saint Vincent and the Grenadines |
| 2003 | 61.88782758364 | 0.552693724058086 | 19.7653605814112 | 42.1224670022288 | Saint Vincent and the Grenadines |
| 2004 | 57.54049136136 | 0.558663542945828 | 17.8996213589376 | 39.6408700024223 | Saint Vincent and the Grenadines |
| 2005 | 59.70792067746 | 0.563588116289597 | 16.125950009734 | 43.581970667726 | Saint Vincent and the Grenadines |
| 2006 | 57.71092513897 | 0.568221747811185 | 14.1795117647226 | 43.5314133742474 | Saint Vincent and the Grenadines |
| 2007 | 56.65937894405 | 0.572819052303515 | 13.5109941572072 | 43.1483847868428 | Saint Vincent and the Grenadines |
| 2008 | 52.63824131697 | 0.577611144975623 | 13.6241398879946 | 39.0141014289754 | Saint Vincent and the Grenadines |
| 2009 | 48.56780146773 | 0.582549107234974 | 13.4058656451556 | 35.1619358225744 | Saint Vincent and the Grenadines |
| 2010 | 44.34726952922 | 0.587190383760787 | 13.2759122769871 | 31.0713572522329 | Saint Vincent and the Grenadines |
| 2011 | 43.59300711017 | 0.590259095650209 | 13.4774463893612 | 30.1155607208088 | Saint Vincent and the Grenadines |
| 2012 | 41.5963353633 | 0.592153907530225 | 13.5038514330765 | 28.0924839302235 | Saint Vincent and the Grenadines |
| 2013 | 43.67568130796 | 0.594614074720551 | 13.3544821920402 | 30.3211991159198 | Saint Vincent and the Grenadines |
| 2014 | 48.7460962385 | 0.598378671838632 | 13.2535851595695 | 35.4925110789305 | Saint Vincent and the Grenadines |
| 2015 | 44.15841062726 | 0.603754134616218 | 13.2122288504411 | 30.9461817768188 | Saint Vincent and the Grenadines |
| 2016 | 37.38650119523 | 0.610059210417339 | 13.3338763942055 | 24.0526248010245 | Saint Vincent and the Grenadines |
| 2017 | 34.46783434962 | 0.616438472725892 | 13.0585847907869 | 21.4092495588331 | Saint Vincent and the Grenadines |
| 2018 | 32.34968903682 | 0.622634944331176 | 13.0210894055116 | 19.3285996313084 | Saint Vincent and the Grenadines |
| 2019 | 30.32353750788 | 0.628325985755241 | 12.7711296453855 | 17.5524078624945 | Saint Vincent and the Grenadines |
| 2020 | 27.83151180658 | 0.632860904072373 | 11.1850051907725 | 16.6465066158075 | Saint Vincent and the Grenadines |
| 2021 | 27.68195434869 | 0.637195962512539 | 8.1615356377716 | 19.5204187109184 | Saint Vincent and the Grenadines |
| 1990 | 549.33118984429 | 0.50205430482691 | 23.3962853662234 | 525.934904478067 | Suriname |
| 1991 | 531.06193403925 | 0.506655794671834 | 22.7639351594052 | 508.297998879845 | Suriname |
| 1992 | 511.45312236419 | 0.510959772316403 | 22.5134730348027 | 488.939649329387 | Suriname |
| 1993 | 482.41195748913 | 0.514419436992343 | 22.5099022300519 | 459.902055259078 | Suriname |
| 1994 | 461.54031387745 | 0.517650245235505 | 22.3313568300687 | 439.208957047381 | Suriname |
| 1995 | 437.77669271628 | 0.521002622374316 | 22.1487720692057 | 415.627920647074 | Suriname |
| 1996 | 413.91249829126 | 0.525059318240488 | 22.1484176506784 | 391.764080640582 | Suriname |
| 1997 | 392.10696497714 | 0.529750811513369 | 22.1290070919746 | 369.977957885165 | Suriname |
| 1998 | 365.3449771302 | 0.534599696353116 | 21.5110521780025 | 343.833924952197 | Suriname |
| 1999 | 339.29135435258 | 0.538932791302378 | 21.1958038771733 | 318.095550475407 | Suriname |
| 2000 | 300.9212370399 | 0.543660023350067 | 21.1424602223952 | 279.778776817505 | Suriname |
| 2001 | 273.78042157135 | 0.548683523928075 | 20.5539728564297 | 253.22644871492 | Suriname |
| 2002 | 265.23327226973 | 0.553879432238792 | 18.6086783988042 | 246.624593870926 | Suriname |
| 2003 | 251.22371766673 | 0.559418373479841 | 18.016149222973 | 233.207568443757 | Suriname |
| 2004 | 237.02568449838 | 0.565109708384423 | 16.0846541830242 | 220.941030315356 | Suriname |
| 2005 | 224.54572820811 | 0.570462186195307 | 13.5289988160036 | 211.016729392106 | Suriname |
| 2006 | 212.19334664113 | 0.575647048246499 | 13.3518046946559 | 198.841541946474 | Suriname |
| 2007 | 205.79909548694 | 0.580654574084671 | 13.1928845209761 | 192.606210965964 | Suriname |
| 2008 | 195.03703133962 | 0.585644825421143 | 13.2164748358235 | 181.820556503796 | Suriname |
| 2009 | 185.2375775697 | 0.590512804838156 | 13.2259724520722 | 172.011605117628 | Suriname |
| 2010 | 174.26958745565 | 0.595469745192738 | 13.4045478921063 | 160.865039563544 | Suriname |
| 2011 | 163.13747033663 | 0.600356336126983 | 13.354325392126 | 149.783144944504 | Suriname |
| 2012 | 154.81355456233 | 0.604741870150833 | 13.3198431238229 | 141.493711438507 | Suriname |
| 2013 | 149.694292864 | 0.608910612397383 | 13.2384199751916 | 136.455872888808 | Suriname |
| 2014 | 143.46603765417 | 0.61270442287871 | 13.3595825066532 | 130.106455147517 | Suriname |
| 2015 | 138.02914234432 | 0.616097315384298 | 12.9687537753914 | 125.060388568929 | Suriname |
| 2016 | 134.91962879147 | 0.618868087951188 | 12.9477031435901 | 121.97192564788 | Suriname |
| 2017 | 135.06899509981 | 0.621684179841572 | 12.8604820771447 | 122.208513022665 | Suriname |
| 2018 | 131.99055240555 | 0.624708011000566 | 12.8243963444524 | 119.166156061098 | Suriname |
| 2019 | 129.51374351608 | 0.62789678100938 | 12.7015450523972 | 116.812198463683 | Suriname |
| 2020 | 122.32881539818 | 0.630683705083472 | 12.0049840835062 | 110.323831314674 | Suriname |
| 2021 | 119.41086644169 | 0.63366573910901 | 10.8570572397394 | 108.553809201951 | Suriname |
| 1990 | 95.23551767305 | 0.623970150133519 | 12.9132975546537 | 82.3222201183963 | Trinidad and Tobago |
| 1991 | 88.27670011075 | 0.62875891817345 | 12.5904232217136 | 75.6862768890364 | Trinidad and Tobago |
| 1992 | 85.90374953109 | 0.634039551508711 | 10.6443505748361 | 75.2593989562539 | Trinidad and Tobago |
| 1993 | 94.55166522492 | 0.639191913980039 | 7.91446895630265 | 86.6371962686174 | Trinidad and Tobago |
| 1994 | 90.12731466281 | 0.644242910113573 | 5.99890216525956 | 84.1284124975504 | Trinidad and Tobago |
| 1995 | 85.28319966896 | 0.649220055474201 | 5.65576711015141 | 79.6274325588086 | Trinidad and Tobago |
| 1996 | 79.94412398415 | 0.654169455231313 | 5.13149057233945 | 74.8126334118105 | Trinidad and Tobago |
| 1997 | 77.77211581959 | 0.659293633341366 | 4.60317751343041 | 73.1689383061596 | Trinidad and Tobago |
| 1998 | 73.65653275881 | 0.664507714329849 | 4.49401824766595 | 69.162514511144 | Trinidad and Tobago |
| 1999 | 71.6356638799 | 0.670244573860158 | 3.86727834860767 | 67.7683855312923 | Trinidad and Tobago |
| 2000 | 63.92944297743 | 0.676683835395074 | 3.0758026196416 | 60.8536403577884 | Trinidad and Tobago |
| 2001 | 50.69393320776 | 0.682763494477584 | 2.80459187731184 | 47.8893413304482 | Trinidad and Tobago |
| 2002 | 45.93880611947 | 0.688872270964441 | 2.47692377285732 | 43.4618823466127 | Trinidad and Tobago |
| 2003 | 45.63888416418 | 0.695828341273334 | 2.40023834079879 | 43.2386458233812 | Trinidad and Tobago |
| 2004 | 38.01124593086 | 0.702610808512884 | 2.35909333594353 | 35.6521525949165 | Trinidad and Tobago |
| 2005 | 40.47603933705 | 0.708520984658684 | 2.31935867779158 | 38.1566806592584 | Trinidad and Tobago |
| 2006 | 37.86018020252 | 0.714600209518824 | 2.26731237715 | 35.59286782537 | Trinidad and Tobago |
| 2007 | 33.44241219202 | 0.72019641678818 | 2.19689566070941 | 31.2455165313106 | Trinidad and Tobago |
| 2008 | 34.14200550664 | 0.725720701823497 | 2.17372018834672 | 31.9682853182933 | Trinidad and Tobago |
| 2009 | 31.15685278584 | 0.729538891843969 | 2.17091010551808 | 28.9859426803219 | Trinidad and Tobago |
| 2010 | 27.98950903815 | 0.733237076528245 | 2.17695268024091 | 25.8125563579091 | Trinidad and Tobago |
| 2011 | 28.58642880805 | 0.736802404534694 | 2.15122408112544 | 26.4352047269246 | Trinidad and Tobago |
| 2012 | 27.31143560711 | 0.739999073209787 | 2.10915256682 | 25.20228304029 | Trinidad and Tobago |
| 2013 | 26.07776641866 | 0.743593955236757 | 2.10106202710081 | 23.9767043915592 | Trinidad and Tobago |
| 2014 | 24.86823074171 | 0.747338962511986 | 2.06675570805206 | 22.8014750336579 | Trinidad and Tobago |
| 2015 | 23.39586138863 | 0.751510682495009 | 2.02300893380209 | 21.3728524548279 | Trinidad and Tobago |
| 2016 | 23.45339646474 | 0.754953347556518 | 2.00510848242365 | 21.4482879823163 | Trinidad and Tobago |
| 2017 | 21.86890142791 | 0.758226987870251 | 1.91878652770194 | 19.9501149002081 | Trinidad and Tobago |
| 2018 | 20.53549792735 | 0.761241990776049 | 1.7543358183886 | 18.7811621089614 | Trinidad and Tobago |
| 2019 | 20.27434576564 | 0.764169599010201 | 1.67953385095763 | 18.5948119146824 | Trinidad and Tobago |
| 2020 | 19.18498194395 | 0.766422080818681 | 1.70549674715942 | 17.4794851967906 | Trinidad and Tobago |
| 2021 | 18.96451837094 | 0.768763253980677 | 1.62883575952615 | 17.3356826114138 | Trinidad and Tobago |
| 1990 | 2619.76158407521 | 0.423917961315613 | 233.649809844133 | 2386.11177423108 | Bolivia (Plurinational State of) |
| 1991 | 2755.89603199575 | 0.429397795077356 | 204.228739823495 | 2551.66729217226 | Bolivia (Plurinational State of) |
| 1992 | 2649.41778791561 | 0.434835541945787 | 187.633991837491 | 2461.78379607812 | Bolivia (Plurinational State of) |
| 1993 | 2370.79107020278 | 0.440601645503372 | 124.506213857147 | 2246.28485634563 | Bolivia (Plurinational State of) |
| 1994 | 2034.33012209035 | 0.446822500209606 | 75.7475804467533 | 1958.5825416436 | Bolivia (Plurinational State of) |
| 1995 | 1921.15469191443 | 0.45347924306274 | 82.6177695474242 | 1838.53692236701 | Bolivia (Plurinational State of) |
| 1996 | 1816.39679823173 | 0.460220668843174 | 66.0703711166158 | 1750.32642711511 | Bolivia (Plurinational State of) |
| 1997 | 1631.61659818452 | 0.466982896503528 | 58.7025307032638 | 1572.91406748126 | Bolivia (Plurinational State of) |
| 1998 | 1512.77302755968 | 0.473796200765922 | 57.0453760472277 | 1455.72765151245 | Bolivia (Plurinational State of) |
| 1999 | 1403.77825483647 | 0.480194310456872 | 54.1983421965648 | 1349.57991263991 | Bolivia (Plurinational State of) |
| 2000 | 1304.66896040735 | 0.486327903067974 | 53.569413586567 | 1251.09954682078 | Bolivia (Plurinational State of) |
| 2001 | 1221.03506962491 | 0.492030763865071 | 50.9740937039908 | 1170.06097592092 | Bolivia (Plurinational State of) |
| 2002 | 1137.49858865045 | 0.497348836505264 | 28.7476238464356 | 1108.75096480401 | Bolivia (Plurinational State of) |
| 2003 | 1056.06350904631 | 0.502365802781048 | 23.3741188840219 | 1032.68939016229 | Bolivia (Plurinational State of) |
| 2004 | 982.24084161902 | 0.507273035649156 | 22.6230918688759 | 959.617749750144 | Bolivia (Plurinational State of) |
| 2005 | 921.25276953925 | 0.512166491375209 | 22.6445276585199 | 898.60824188073 | Bolivia (Plurinational State of) |
| 2006 | 859.19649588847 | 0.517146193689981 | 22.3400592615079 | 836.856436626962 | Bolivia (Plurinational State of) |
| 2007 | 802.84576161715 | 0.521946130357677 | 22.140376302523 | 780.705385314627 | Bolivia (Plurinational State of) |
| 2008 | 706.29784499601 | 0.526943750292372 | 22.1293962680653 | 684.168448727945 | Bolivia (Plurinational State of) |
| 2009 | 622.23205227403 | 0.531792440464007 | 22.1312268215866 | 600.100825452443 | Bolivia (Plurinational State of) |
| 2010 | 577.21299029036 | 0.5370090684329 | 21.2580585291593 | 555.954931761201 | Bolivia (Plurinational State of) |
| 2011 | 535.82688568158 | 0.542680744993221 | 21.174836685921 | 514.652048995659 | Bolivia (Plurinational State of) |
| 2012 | 498.78509008547 | 0.548615481889587 | 20.4687324256188 | 478.316357659851 | Bolivia (Plurinational State of) |
| 2013 | 469.49980911631 | 0.554788215843767 | 18.7088680246585 | 450.790941091651 | Bolivia (Plurinational State of) |
| 2014 | 438.95825019349 | 0.560934581048908 | 16.7769132670349 | 422.181336926455 | Bolivia (Plurinational State of) |
| 2015 | 410.03671141664 | 0.567000897613403 | 14.5525994570315 | 395.484111959609 | Bolivia (Plurinational State of) |
| 2016 | 381.45710302878 | 0.573080316306652 | 13.2354581695026 | 368.221644859277 | Bolivia (Plurinational State of) |
| 2017 | 356.14540273037 | 0.579241284614228 | 13.4139707642244 | 342.731431966146 | Bolivia (Plurinational State of) |
| 2018 | 331.75340505687 | 0.585184106450667 | 13.1596051877259 | 318.593799869144 | Bolivia (Plurinational State of) |
| 2019 | 312.81979933171 | 0.590692043089904 | 13.1032186397069 | 299.716580692003 | Bolivia (Plurinational State of) |
| 2020 | 296.07476208898 | 0.594854443460131 | 13.1575659601671 | 282.917196128813 | Bolivia (Plurinational State of) |
| 2021 | 281.09319540173 | 0.5990107988623 | 13.2257943014437 | 267.867401100286 | Bolivia (Plurinational State of) |
| 1990 | 2359.0957225143 | 0.518430614137285 | 22.2818283349523 | 2336.81389417935 | Ecuador |
| 1991 | 2777.96415441492 | 0.517679421435991 | 22.3016915965236 | 2755.6624628184 | Ecuador |
| 1992 | 2307.87094224778 | 0.518379070003192 | 22.3012453007999 | 2285.56969694698 | Ecuador |
| 1993 | 1696.70111384882 | 0.520707290609383 | 22.1676633578686 | 1674.53345049095 | Ecuador |
| 1994 | 1291.74382517811 | 0.524908290908027 | 22.1621914757163 | 1269.58163370239 | Ecuador |
| 1995 | 1254.00970187875 | 0.529380751671009 | 22.0788713903248 | 1231.93083048843 | Ecuador |
| 1996 | 1232.75294624503 | 0.532691399133141 | 22.1443337792198 | 1210.60861246581 | Ecuador |
| 1997 | 1127.42694839056 | 0.535349652252826 | 21.513909985187 | 1105.91303840537 | Ecuador |
| 1998 | 1073.62395188362 | 0.537196329914338 | 21.3166300895544 | 1052.30732179407 | Ecuador |
| 1999 | 959.74918616026 | 0.53903230782875 | 21.3078713385551 | 938.441314821705 | Ecuador |
| 2000 | 916.97043482867 | 0.5430447789024 | 21.171451422226 | 895.798983406444 | Ecuador |
| 2001 | 852.72000644128 | 0.548552149690973 | 20.4103022611088 | 832.309704180171 | Ecuador |
| 2002 | 767.28961484025 | 0.554897325204538 | 18.8190456774167 | 748.470569162833 | Ecuador |
| 2003 | 712.57193208084 | 0.560955484518967 | 16.7012361919873 | 695.870695888853 | Ecuador |
| 2004 | 634.04561867134 | 0.566290292625305 | 15.0596018351906 | 618.98601683615 | Ecuador |
| 2005 | 563.65565412123 | 0.569927344748438 | 13.2581029349732 | 550.397551186257 | Ecuador |
| 2006 | 502.30583623423 | 0.572516273171735 | 13.346691957128 | 488.959144277102 | Ecuador |
| 2007 | 453.17788158919 | 0.574704702310921 | 13.1852492687297 | 439.99263232046 | Ecuador |
| 2008 | 376.08475499934 | 0.578125040269336 | 13.4913819528054 | 362.593373046535 | Ecuador |
| 2009 | 334.09804835537 | 0.58248425757379 | 13.408685824019 | 320.689362531351 | Ecuador |
| 2010 | 263.82835767897 | 0.588763055747133 | 13.3296466253163 | 250.498711053654 | Ecuador |
| 2011 | 216.94047844305 | 0.596729076106647 | 13.2693558880765 | 203.671122554973 | Ecuador |
| 2012 | 174.73155513446 | 0.605454319333254 | 13.3598686490072 | 161.371686485453 | Ecuador |
| 2013 | 135.46568114149 | 0.614080274696922 | 13.1177097309781 | 122.347971410512 | Ecuador |
| 2014 | 119.30647504121 | 0.622027432994838 | 12.8948506938268 | 106.411624347383 | Ecuador |
| 2015 | 119.00142157938 | 0.628546709567119 | 12.7511126106124 | 106.250308968768 | Ecuador |
| 2016 | 103.4990982484 | 0.634281142866422 | 11.1327980084689 | 92.3663002399311 | Ecuador |
| 2017 | 82.49990258233 | 0.640011218750157 | 7.96344240061985 | 74.5364601817101 | Ecuador |
| 2018 | 76.25535833354 | 0.645888520186892 | 5.7269647151938 | 70.5283936183462 | Ecuador |
| 2019 | 79.06643569167 | 0.651787987044895 | 5.1157618420372 | 73.9506738496328 | Ecuador |
| 2020 | 66.17847351757 | 0.65671445724893 | 4.61832845307949 | 61.5601450644905 | Ecuador |
| 2021 | 61.30833322782 | 0.66101705262103 | 4.57074791251494 | 56.737585315305 | Ecuador |
| 1990 | 1181.36574872231 | 0.510419852074136 | 22.4351835789349 | 1158.93056514338 | Peru |
| 1991 | 1491.561844666 | 0.512161524663903 | 22.4362395115527 | 1469.12560515445 | Peru |
| 1992 | 1155.6581157655 | 0.513821551605533 | 22.4724205490735 | 1133.18569521643 | Peru |
| 1993 | 1027.96982673298 | 0.515981506410929 | 22.3541219236666 | 1005.61570480931 | Peru |
| 1994 | 837.84304807861 | 0.519306080333588 | 22.1992313154336 | 815.643816763176 | Peru |
| 1995 | 747.28866482825 | 0.523640526677899 | 22.0682251506924 | 725.220439677558 | Peru |
| 1996 | 648.19771929652 | 0.528312042097199 | 22.1772513419037 | 626.020467954616 | Peru |
| 1997 | 583.98171659569 | 0.533576491199309 | 22.1211763163368 | 561.860540279353 | Peru |
| 1998 | 536.47962046262 | 0.538721717199262 | 21.2541341259386 | 515.225486336681 | Peru |
| 1999 | 481.14454305787 | 0.543853376891845 | 21.2213955265522 | 459.923147531318 | Peru |
| 2000 | 430.81627902273 | 0.548952201243799 | 20.6195712130846 | 410.196707809645 | Peru |
| 2001 | 374.98767729652 | 0.553775453477556 | 18.8706447109119 | 356.117032585608 | Peru |
| 2002 | 334.77493346539 | 0.558853262904608 | 17.7715855880568 | 317.003347877333 | Peru |
| 2003 | 305.32946688333 | 0.563871071930545 | 15.9085571187256 | 289.420909764604 | Peru |
| 2004 | 278.37840970828 | 0.56906302441994 | 14.1228781768878 | 264.255531531392 | Peru |
| 2005 | 255.95208892996 | 0.574517001323253 | 13.4326392545077 | 242.519449675452 | Peru |
| 2006 | 230.44207265415 | 0.580503364933972 | 13.4076512009479 | 217.034421453202 | Peru |
| 2007 | 202.6863573427 | 0.586958489258033 | 13.4436472777922 | 189.242710064908 | Peru |
| 2008 | 187.11800736309 | 0.593750810824365 | 13.3634282516472 | 173.754579111443 | Peru |
| 2009 | 162.70030598129 | 0.599954992710361 | 13.2672370780629 | 149.433068903227 | Peru |
| 2010 | 143.46009490097 | 0.606520931663104 | 13.3939106870865 | 130.066184213884 | Peru |
| 2011 | 130.43118556935 | 0.613051220911462 | 13.2967763465178 | 117.134409222832 | Peru |
| 2012 | 121.69469035182 | 0.619355264313384 | 12.9990630507241 | 108.695627301096 | Peru |
| 2013 | 116.8921851725 | 0.62551902425403 | 12.938799514805 | 103.953385657695 | Peru |
| 2014 | 113.15007856507 | 0.631114354280049 | 11.8903255817463 | 101.259752983324 | Peru |
| 2015 | 108.0288500888 | 0.636278270548633 | 10.7433053256821 | 97.2855447631179 | Peru |
| 2016 | 104.65682165536 | 0.641285279069023 | 8.40338501421067 | 96.2534366411493 | Peru |
| 2017 | 101.85565035915 | 0.64618085494837 | 6.20700224536287 | 95.6486481137871 | Peru |
| 2018 | 100.95452763013 | 0.650977317324287 | 5.04852830517648 | 95.9059993249535 | Peru |
| 2019 | 90.75974265895 | 0.655433734747709 | 5.29869936676158 | 85.4610432921884 | Peru |
| 2020 | 77.64113535391 | 0.658672243500517 | 4.60506553509028 | 73.0360698188197 | Peru |
| 2021 | 69.08466848087 | 0.662054036755862 | 4.68378982608705 | 64.4008786547829 | Peru |
| 1990 | 1938.61257670277 | 0.480720053627064 | 53.2150895815615 | 1885.39748712121 | Colombia |
| 1991 | 1769.64762004857 | 0.482339893974629 | 54.0205231422993 | 1715.62709690627 | Colombia |
| 1992 | 1699.1170748558 | 0.484572358989267 | 53.8111139678566 | 1645.30596088794 | Colombia |
| 1993 | 1474.05018651385 | 0.488229373054794 | 49.2562755237733 | 1424.79391099008 | Colombia |
| 1994 | 1312.44567090728 | 0.493278181852073 | 50.2353200654982 | 1262.21035084178 | Colombia |
| 1995 | 1174.16353912707 | 0.49910275736379 | 23.9940011511802 | 1150.16953797589 | Colombia |
| 1996 | 1057.85385912658 | 0.504986969396651 | 22.9121984007714 | 1034.94166072581 | Colombia |
| 1997 | 956.26741695995 | 0.511079502295084 | 22.4948126387461 | 933.772604321204 | Colombia |
| 1998 | 876.69031290728 | 0.517022504946273 | 22.2758429763611 | 854.414469930919 | Colombia |
| 1999 | 721.93912169756 | 0.522435940409787 | 22.18900630065 | 699.75011539691 | Colombia |
| 2000 | 637.28365901906 | 0.527839094091296 | 22.1706989845584 | 615.112960034502 | Colombia |
| 2001 | 551.17676585368 | 0.533076500590306 | 22.0964707716914 | 529.080295081989 | Colombia |
| 2002 | 604.03851685895 | 0.538189342486614 | 21.3129343143161 | 582.725582544634 | Colombia |
| 2003 | 598.19253686157 | 0.54326513104442 | 21.170699210044 | 577.021837651526 | Colombia |
| 2004 | 521.00566312396 | 0.548601831852621 | 20.5139201839854 | 500.491742939975 | Colombia |
| 2005 | 451.97872812284 | 0.554089190974456 | 18.7132925794409 | 433.265435543399 | Colombia |
| 2006 | 406.0740171811 | 0.56018273477503 | 17.4914352283168 | 388.582581952783 | Colombia |
| 2007 | 379.21223271725 | 0.566822644330018 | 14.3886534371939 | 364.823579280056 | Colombia |
| 2008 | 328.77487670912 | 0.573494651915472 | 13.5008120140114 | 315.274064695109 | Colombia |
| 2009 | 297.36891304281 | 0.579876468776209 | 13.2426033228303 | 284.12630971998 | Colombia |
| 2010 | 233.78715683107 | 0.586408108388396 | 13.6339730706709 | 220.153183760399 | Colombia |
| 2011 | 189.21608266078 | 0.593340477060868 | 13.4265860827306 | 175.789496578049 | Colombia |
| 2012 | 172.19156395739 | 0.600254237718889 | 13.6027040702476 | 158.588859887142 | Colombia |
| 2013 | 165.61778680125 | 0.607250636585575 | 13.2602352852672 | 152.357551515983 | Colombia |
| 2014 | 162.03819519862 | 0.614151922522411 | 13.0570785450305 | 148.98111665359 | Colombia |
| 2015 | 165.84558615311 | 0.620889228107476 | 12.9918643852253 | 152.853721767885 | Colombia |
| 2016 | 153.65491554989 | 0.627554497248436 | 12.8564755767504 | 140.79843997314 | Colombia |
| 2017 | 150.81591858235 | 0.634130359426935 | 11.0930691350895 | 139.722849447261 | Colombia |
| 2018 | 167.48670730742 | 0.640255071652482 | 8.39393302670534 | 159.092774280715 | Colombia |
| 2019 | 208.11602312848 | 0.646038852086803 | 6.20282408580085 | 201.913199042679 | Colombia |
| 2020 | 173.38266955798 | 0.650855354021142 | 5.75440181804108 | 167.628267739939 | Colombia |
| 2021 | 157.68806005855 | 0.655442912568827 | 4.97557924696827 | 152.712480811582 | Colombia |
| 1990 | 344.36071130132 | 0.534125181350537 | 21.507466979531 | 322.853244321789 | Costa Rica |
| 1991 | 286.36772388041 | 0.539342163753933 | 21.1652670152985 | 265.202456865112 | Costa Rica |
| 1992 | 245.13693183042 | 0.5446242375755 | 21.1905066079988 | 223.946425222421 | Costa Rica |
| 1993 | 230.87368081728 | 0.549946625586597 | 19.7907353883565 | 211.082945428924 | Costa Rica |
| 1994 | 226.98964629692 | 0.555171250068523 | 18.8307165079327 | 208.158929788987 | Costa Rica |
| 1995 | 214.43442904205 | 0.560547086077833 | 17.6723861212102 | 196.76204292084 | Costa Rica |
| 1996 | 202.69988566162 | 0.565180861960769 | 15.1793050065775 | 187.520580655043 | Costa Rica |
| 1997 | 202.47718718922 | 0.570128417464587 | 13.4026500324297 | 189.07453715679 | Costa Rica |
| 1998 | 184.55803241723 | 0.576067050570833 | 13.2529586711432 | 171.305073746087 | Costa Rica |
| 1999 | 158.42985588815 | 0.582229491567193 | 13.3433564302763 | 145.086499457874 | Costa Rica |
| 2000 | 133.86855081738 | 0.588243191082194 | 13.5562141758567 | 120.312336641523 | Costa Rica |
| 2001 | 124.91213272237 | 0.594889114531861 | 13.1515703226171 | 111.760562399753 | Costa Rica |
| 2002 | 113.22737551381 | 0.600894432967727 | 13.1212737032018 | 100.106101810608 | Costa Rica |
| 2003 | 106.35130257174 | 0.605765686833733 | 13.4257808599027 | 92.9255217118373 | Costa Rica |
| 2004 | 91.882725141 | 0.610315699466228 | 13.5159456838886 | 78.3667794571114 | Costa Rica |
| 2005 | 83.62137422324 | 0.614412618734781 | 13.4150126105188 | 70.2063616127212 | Costa Rica |
| 2006 | 73.20365651509 | 0.617850162499434 | 12.9857639175392 | 60.2178925975508 | Costa Rica |
| 2007 | 76.08358266254 | 0.621200571426243 | 12.9297844570523 | 63.1537982054877 | Costa Rica |
| 2008 | 67.87846800545 | 0.625703910462395 | 13.0173695183966 | 54.8610984870534 | Costa Rica |
| 2009 | 64.45842875591 | 0.631291379026346 | 12.0910099580387 | 52.3674187978713 | Costa Rica |
| 2010 | 62.84691930321 | 0.63659542601002 | 10.6188476256329 | 52.2280716775771 | Costa Rica |
| 2011 | 56.34736415542 | 0.641201295145018 | 7.50713097628972 | 48.8402331791303 | Costa Rica |
| 2012 | 55.03976344563 | 0.646574092912526 | 6.07177208930205 | 48.9679913563279 | Costa Rica |
| 2013 | 48.57083262497 | 0.651945812639188 | 4.47572911290466 | 44.0951035120653 | Costa Rica |
| 2014 | 52.92266876743 | 0.656890456365803 | 5.03158402307561 | 47.8910847443544 | Costa Rica |
| 2015 | 48.64731541048 | 0.662030135918026 | 4.66614469537192 | 43.9811707151081 | Costa Rica |
| 2016 | 50.89179728994 | 0.667430091500307 | 3.75178640434804 | 47.140010885592 | Costa Rica |
| 2017 | 48.80655761351 | 0.673621391630886 | 3.12188963400214 | 45.6846679795079 | Costa Rica |
| 2018 | 48.44150084034 | 0.681296147310122 | 2.83207677664848 | 45.6094240636915 | Costa Rica |
| 2019 | 57.56286163296 | 0.690049088214507 | 2.57918011771478 | 54.9836815152452 | Costa Rica |
| 2020 | 50.04954239327 | 0.696194605172093 | 2.45412632771632 | 47.5954160655537 | Costa Rica |
| 2021 | 48.04149512417 | 0.700340476999325 | 2.31413160296567 | 45.7273635212043 | Costa Rica |
| 1990 | 3475.13204579385 | 0.373057974861269 | 543.644960489176 | 2931.48708530467 | El Salvador |
| 1991 | 3422.95561245116 | 0.374855325024228 | 541.740285378172 | 2881.21532707299 | El Salvador |
| 1992 | 2970.03915163391 | 0.377076642695912 | 549.829556956635 | 2420.20959467727 | El Salvador |
| 1993 | 2612.64104037163 | 0.380307326030095 | 508.813026116593 | 2103.82801425504 | El Salvador |
| 1994 | 2399.46935341236 | 0.38514909404887 | 521.689483814371 | 1877.77986959799 | El Salvador |
| 1995 | 2091.70055928362 | 0.391780266086918 | 475.738710608796 | 1615.96184867482 | El Salvador |
| 1996 | 1855.29395976948 | 0.400178467721391 | 463.476099088193 | 1391.81786068129 | El Salvador |
| 1997 | 1615.24175123629 | 0.410455825709854 | 302.89803282033 | 1312.34371841596 | El Salvador |
| 1998 | 1391.50006162555 | 0.421421189598308 | 249.970165136806 | 1141.52989648874 | El Salvador |
| 1999 | 1178.89982657364 | 0.432733147562664 | 207.250196723446 | 971.649629850194 | El Salvador |
| 2000 | 1014.83199817374 | 0.443962665887944 | 107.783694091153 | 907.048304082587 | El Salvador |
| 2001 | 875.07073919142 | 0.454177658570425 | 75.1800737662482 | 799.890665425172 | El Salvador |
| 2002 | 766.71470621304 | 0.462968859826757 | 61.3913906833028 | 705.323315529737 | El Salvador |
| 2003 | 672.75926076209 | 0.470397412659385 | 56.3705672937671 | 616.388693468323 | El Salvador |
| 2004 | 607.03998060975 | 0.476536664263465 | 52.7322296097463 | 554.307751000004 | El Salvador |
| 2005 | 548.87254986418 | 0.48204171929172 | 53.3132056139762 | 495.559344250204 | El Salvador |
| 2006 | 457.39086804531 | 0.486826000431208 | 49.330392889569 | 408.060475155741 | El Salvador |
| 2007 | 409.75948102102 | 0.490809751570084 | 51.4351606559491 | 358.324320365071 | El Salvador |
| 2008 | 377.52864977956 | 0.495077738859345 | 50.4669407022078 | 327.061709077352 | El Salvador |
| 2009 | 372.51889116529 | 0.499489714249212 | 23.9469239425521 | 348.571967222738 | El Salvador |
| 2010 | 339.24378006429 | 0.504171335202455 | 22.9323260932988 | 316.311453970991 | El Salvador |
| 2011 | 314.21700525338 | 0.508969239001561 | 22.7743396327584 | 291.442665620622 | El Salvador |
| 2012 | 290.59271029222 | 0.513396874335326 | 22.4796434405257 | 268.113066851694 | El Salvador |
| 2013 | 270.48093151935 | 0.518121907716899 | 22.3917747247948 | 248.089156794555 | El Salvador |
| 2014 | 248.58497209424 | 0.523468900855703 | 22.1599961686008 | 226.424975925639 | El Salvador |
| 2015 | 225.24745125182 | 0.529386950266238 | 22.1765930462275 | 203.070858205593 | El Salvador |
| 2016 | 210.19953103435 | 0.535650269148693 | 21.5647605790144 | 188.634770455336 | El Salvador |
| 2017 | 197.76658188552 | 0.541998957448254 | 21.1678306466482 | 176.598751238872 | El Salvador |
| 2018 | 186.9261074982 | 0.548143173580896 | 20.5848223344889 | 166.341285163711 | El Salvador |
| 2019 | 176.94444800655 | 0.55403164778242 | 18.8538916092728 | 158.090556397277 | El Salvador |
| 2020 | 164.97970536496 | 0.558919894116032 | 18.0879172002373 | 146.891788164723 | El Salvador |
| 2021 | 157.02178249315 | 0.563775187564695 | 15.9123292309421 | 141.109453262208 | El Salvador |
| 1990 | 6437.15915531583 | 0.311792455277134 | 965.819802533725 | 5471.3393527821 | Guatemala |
| 1991 | 6015.93000375771 | 0.314329483676405 | 963.801065145681 | 5052.12893861203 | Guatemala |
| 1992 | 5026.00299203552 | 0.319165481239087 | 846.721869267738 | 4179.28112276778 | Guatemala |
| 1993 | 4519.91740569839 | 0.326840818669512 | 760.203542597482 | 3759.71386310091 | Guatemala |
| 1994 | 4307.3694065199 | 0.33346910005957 | 681.781422345335 | 3625.58798417456 | Guatemala |
| 1995 | 4010.77990045656 | 0.339642100810868 | 602.253831156065 | 3408.5260693005 | Guatemala |
| 1996 | 3794.09458587679 | 0.346961212155122 | 597.398244499806 | 3196.69634137698 | Guatemala |
| 1997 | 3550.2673839493 | 0.355995963296002 | 584.842526284627 | 2965.42485766467 | Guatemala |
| 1998 | 3478.66073971984 | 0.366461192871903 | 578.985495174438 | 2899.6752445454 | Guatemala |
| 1999 | 3147.95864620546 | 0.374455316256445 | 546.332092803907 | 2601.62655340155 | Guatemala |
| 2000 | 3195.24865116412 | 0.379877296535484 | 548.193767943076 | 2647.05488322104 | Guatemala |
| 2001 | 3098.88007680705 | 0.386927685682523 | 514.580967494897 | 2584.29910931215 | Guatemala |
| 2002 | 2845.95308869802 | 0.396899639101221 | 464.404755147903 | 2381.54833355012 | Guatemala |
| 2003 | 2506.19645486412 | 0.408234164151934 | 311.467491869904 | 2194.72896299422 | Guatemala |
| 2004 | 2213.52590523489 | 0.419777064914249 | 263.630718406414 | 1949.89518682848 | Guatemala |
| 2005 | 2061.90623111918 | 0.430327529380029 | 204.979004075045 | 1856.92722704413 | Guatemala |
| 2006 | 1871.49592477631 | 0.438856375714513 | 120.538989748602 | 1750.95693502771 | Guatemala |
| 2007 | 1819.07447725668 | 0.447245964554675 | 72.1517594149328 | 1746.92271784175 | Guatemala |
| 2008 | 1738.9376167711 | 0.456541865045794 | 66.8913075839449 | 1672.04630918716 | Guatemala |
| 2009 | 1583.70427038169 | 0.464003076644959 | 63.6102063016655 | 1520.09406408002 | Guatemala |
| 2010 | 1536.88827741472 | 0.470477363646759 | 58.6564647839887 | 1478.23181263073 | Guatemala |
| 2011 | 1500.15787338799 | 0.476909684291273 | 53.2417565638507 | 1446.91611682414 | Guatemala |
| 2012 | 1471.53676527979 | 0.481862853245964 | 53.3052864190162 | 1418.23147886077 | Guatemala |
| 2013 | 1398.5929917422 | 0.487286692231106 | 50.2322283092127 | 1348.36076343299 | Guatemala |
| 2014 | 1390.93406832087 | 0.494027188122075 | 48.9394240860362 | 1341.99464423483 | Guatemala |
| 2015 | 1335.97423561611 | 0.500655606612947 | 23.2931701201927 | 1312.68106549592 | Guatemala |
| 2016 | 1385.41650179615 | 0.507149791967016 | 22.771479524004 | 1362.64502227215 | Guatemala |
| 2017 | 1308.50523199422 | 0.51373568188905 | 22.474334822222 | 1286.030897172 | Guatemala |
| 2018 | 1279.01502010756 | 0.52099546880295 | 22.1306329162847 | 1256.88438719128 | Guatemala |
| 2019 | 1348.72695165915 | 0.528630469662807 | 22.1440400826333 | 1326.58291157652 | Guatemala |
| 2020 | 1147.54594567835 | 0.534571414248156 | 21.6406823953595 | 1125.90526328299 | Guatemala |
| 2021 | 1072.93991494317 | 0.539972423611408 | 21.2183905594349 | 1051.72152438373 | Guatemala |
| 1990 | 6773.51012988003 | 0.332042889051331 | 684.56349140534 | 6088.94663847469 | Honduras |
| 1991 | 6441.78933557163 | 0.337034649170168 | 677.847007925264 | 5763.94232764637 | Honduras |
| 1992 | 6031.88308575357 | 0.342305656800435 | 592.215048306839 | 5439.66803744673 | Honduras |
| 1993 | 5837.25010051943 | 0.347838445661094 | 590.838939587862 | 5246.41116093157 | Honduras |
| 1994 | 5419.47767519361 | 0.353164851646287 | 592.624864718094 | 4826.85281047552 | Honduras |
| 1995 | 4931.53025630607 | 0.358787268251117 | 591.595577580845 | 4339.93467872523 | Honduras |
| 1996 | 4331.25123181745 | 0.364328424323186 | 579.66326000326 | 3751.58797181419 | Honduras |
| 1997 | 3902.50270271935 | 0.369983893774573 | 575.033517710893 | 3327.46918500846 | Honduras |
| 1998 | 3574.11382564649 | 0.375950111095537 | 547.287199676219 | 3026.82662597027 | Honduras |
| 1999 | 3232.9456678708 | 0.38175231641304 | 503.976528800393 | 2728.96913907041 | Honduras |
| 2000 | 2935.9393118618 | 0.387988052509884 | 511.842039612254 | 2424.09727224955 | Honduras |
| 2001 | 2701.10041727233 | 0.394174011784655 | 463.356858675509 | 2237.74355859682 | Honduras |
| 2002 | 2495.77701609537 | 0.400371798466223 | 461.267189953816 | 2034.50982614155 | Honduras |
| 2003 | 2318.73262200554 | 0.406668380994452 | 368.19858144146 | 1950.53404056408 | Honduras |
| 2004 | 2169.93649515174 | 0.413061815539536 | 304.282030932878 | 1865.65446421886 | Honduras |
| 2005 | 2028.74706155986 | 0.419612331829645 | 264.084769136003 | 1764.66229242386 | Honduras |
| 2006 | 1913.80727083544 | 0.426352996817521 | 232.822162026368 | 1680.98510880907 | Honduras |
| 2007 | 1791.08356154965 | 0.433240314987847 | 206.009265866105 | 1585.07429568354 | Honduras |
| 2008 | 1712.77822624094 | 0.439998163793669 | 113.493097305836 | 1599.2851289351 | Honduras |
| 2009 | 1489.44816338772 | 0.446151054184542 | 81.556153948402 | 1407.89200943932 | Honduras |
| 2010 | 1408.2896783889 | 0.452382226389866 | 74.8583875655672 | 1333.43129082333 | Honduras |
| 2011 | 1321.16945174575 | 0.458700328960259 | 63.5177294363072 | 1257.65172230944 | Honduras |
| 2012 | 1228.72146543066 | 0.46492515492495 | 62.723029603112 | 1165.99843582755 | Honduras |
| 2013 | 1164.58745406562 | 0.470766833403007 | 56.6757173810143 | 1107.91173668461 | Honduras |
| 2014 | 1102.66942663721 | 0.476587799009017 | 55.0037988582542 | 1047.66562777896 | Honduras |
| 2015 | 1038.21156241451 | 0.482294176352814 | 54.9968663589237 | 983.214696055586 | Honduras |
| 2016 | 983.80878879515 | 0.487705931888242 | 49.2512358818353 | 934.557552913315 | Honduras |
| 2017 | 939.06645456796 | 0.493130694434939 | 49.7201087850914 | 889.346345782869 | Honduras |
| 2018 | 875.10680884172 | 0.498657226690384 | 23.9009933653886 | 851.205815476331 | Honduras |
| 2019 | 815.65759751094 | 0.504156505325121 | 23.0851096542275 | 792.572487856713 | Honduras |
| 2020 | 759.21519072212 | 0.508669245938408 | 22.8716643572849 | 736.343526364835 | Honduras |
| 2021 | 720.45331799973 | 0.513037248307344 | 22.5684609018247 | 697.884857097905 | Honduras |
| 1990 | 1628.90335747901 | 0.504996083384899 | 23.0193445609849 | 1605.88401291803 | Mexico |
| 1991 | 1457.41331237409 | 0.509510670028127 | 22.650993367258 | 1434.76231900683 | Mexico |
| 1992 | 1213.5718511987 | 0.514638056848767 | 22.5012223921176 | 1191.07062880658 | Mexico |
| 1993 | 1044.02943781035 | 0.52049316730006 | 22.1149989016448 | 1021.9144389087 | Mexico |
| 1994 | 863.79547588929 | 0.526749240631648 | 22.1595003399044 | 841.635975549386 | Mexico |
| 1995 | 750.30346782297 | 0.531554141210122 | 22.1006579388635 | 728.202809884107 | Mexico |
| 1996 | 648.32990761954 | 0.536530826504039 | 21.2976382192995 | 627.03226940024 | Mexico |
| 1997 | 574.18574325075 | 0.541988171199547 | 21.1506252358875 | 553.035118014863 | Mexico |
| 1998 | 500.41373461374 | 0.547842121275417 | 20.4706259085467 | 479.943108705193 | Mexico |
| 1999 | 437.56060116165 | 0.553813369752955 | 18.9293851854411 | 418.631215976209 | Mexico |
| 2000 | 395.98332407013 | 0.559532546937005 | 17.5997439963275 | 378.383580073802 | Mexico |
| 2001 | 365.70427001469 | 0.564774176443707 | 16.2097499770508 | 349.494520037639 | Mexico |
| 2002 | 337.23459191781 | 0.570113531020311 | 13.3247914137115 | 323.909800504099 | Mexico |
| 2003 | 310.72743495375 | 0.575698214652787 | 13.1522917538119 | 297.575143199938 | Mexico |
| 2004 | 279.43389613145 | 0.580984982494851 | 13.5008858889649 | 265.933010242485 | Mexico |
| 2005 | 264.61749942758 | 0.585224080225789 | 13.2247718806147 | 251.392727546965 | Mexico |
| 2006 | 236.07206266253 | 0.588543226375938 | 13.3546510423786 | 222.717411620151 | Mexico |
| 2007 | 221.64714796683 | 0.591447407661459 | 13.1777528261216 | 208.469395140708 | Mexico |
| 2008 | 194.84457180732 | 0.594454558028247 | 13.3052682289743 | 181.539303578346 | Mexico |
| 2009 | 162.95168488687 | 0.596684357960512 | 13.5660234781528 | 149.385661408717 | Mexico |
| 2010 | 141.68185309139 | 0.599527028083825 | 13.203840142057 | 128.478012949333 | Mexico |
| 2011 | 133.65552172625 | 0.60364609507818 | 13.4532572451456 | 120.202264481104 | Mexico |
| 2012 | 133.90230077959 | 0.608914289338676 | 13.3962291397383 | 120.506071639852 | Mexico |
| 2013 | 136.30534973042 | 0.615089992820613 | 12.890531086397 | 123.414818644023 | Mexico |
| 2014 | 123.96228241211 | 0.621857404659516 | 12.9628803841914 | 110.999402027919 | Mexico |
| 2015 | 116.13842587421 | 0.628896723914502 | 12.5496732720716 | 103.588752602138 | Mexico |
| 2016 | 109.06160272995 | 0.635925348800174 | 10.4806817440703 | 98.5809209858796 | Mexico |
| 2017 | 90.27145755822 | 0.642667198400828 | 7.90254683250955 | 82.3689107257104 | Mexico |
| 2018 | 80.52904621596 | 0.649053317386265 | 6.21528834926208 | 74.3137578666979 | Mexico |
| 2019 | 82.68494073505 | 0.655095338007006 | 4.9844492269737 | 77.7004915080763 | Mexico |
| 2020 | 73.22745163628 | 0.660119061584711 | 4.42783417752351 | 68.7996174587565 | Mexico |
| 2021 | 71.94053271951 | 0.664575304259183 | 4.61172313406765 | 67.3288095854424 | Mexico |
| 1990 | 3641.93574989866 | 0.346035234504054 | 607.859836044861 | 3034.0759138538 | Nicaragua |
| 1991 | 3285.70004700108 | 0.35174062237875 | 587.02377691092 | 2698.67627009016 | Nicaragua |
| 1992 | 3004.81943537501 | 0.357712704444006 | 584.307283778104 | 2420.51215159691 | Nicaragua |
| 1993 | 2967.24550725274 | 0.36356206850317 | 572.893069699111 | 2394.35243755363 | Nicaragua |
| 1994 | 2565.68279587346 | 0.369727967895652 | 574.4515719111 | 1991.23122396236 | Nicaragua |
| 1995 | 2351.30711755783 | 0.37662534522155 | 547.295874377952 | 1804.01124317988 | Nicaragua |
| 1996 | 2020.9009884101 | 0.384087266113663 | 517.049349301447 | 1503.85163910865 | Nicaragua |
| 1997 | 1688.4711073004 | 0.391775893494114 | 470.377036949687 | 1218.09407035071 | Nicaragua |
| 1998 | 1481.9254257803 | 0.399807534850283 | 461.954500377125 | 1019.97092540318 | Nicaragua |
| 1999 | 1266.67773103726 | 0.408085964812584 | 318.484334173092 | 948.193396864168 | Nicaragua |
| 2000 | 1106.19252618285 | 0.416108362910212 | 270.532653615728 | 835.659872567122 | Nicaragua |
| 2001 | 984.95680366047 | 0.423790295277938 | 232.792488530174 | 752.164315130296 | Nicaragua |
| 2002 | 875.99571179963 | 0.43081375452391 | 205.465177055539 | 670.530534744091 | Nicaragua |
| 2003 | 780.61473726373 | 0.437248152494534 | 121.612791353342 | 659.001945910388 | Nicaragua |
| 2004 | 706.21680816366 | 0.443274510891091 | 110.67189726295 | 595.54491090071 | Nicaragua |
| 2005 | 648.68800889726 | 0.448844739044126 | 69.6696009680399 | 579.01840792922 | Nicaragua |
| 2006 | 528.33829470774 | 0.453950499137161 | 71.1497597352531 | 457.188534972487 | Nicaragua |
| 2007 | 481.82491445386 | 0.459042797856105 | 61.321306692779 | 420.503607761081 | Nicaragua |
| 2008 | 440.37301021065 | 0.463837699218163 | 63.4021095399391 | 376.970900670711 | Nicaragua |
| 2009 | 409.33101684769 | 0.467961838989771 | 57.3782803425822 | 351.952736505108 | Nicaragua |
| 2010 | 378.45991741849 | 0.472171999845355 | 56.3447292431205 | 322.11518817537 | Nicaragua |
| 2011 | 346.43924160439 | 0.4765934659805 | 53.0786172559083 | 293.360624348482 | Nicaragua |
| 2012 | 319.35658185598 | 0.481296900452524 | 54.659817919592 | 264.696763936388 | Nicaragua |
| 2013 | 298.9105657825 | 0.486105470178736 | 53.0018158138656 | 245.908749968634 | Nicaragua |
| 2014 | 278.66515182517 | 0.491207346240634 | 50.3621020000128 | 228.303049825157 | Nicaragua |
| 2015 | 262.07547056229 | 0.496391662501118 | 32.8887824807884 | 229.186688081502 | Nicaragua |
| 2016 | 241.54537392692 | 0.501853344913168 | 23.2309898338843 | 218.314384093036 | Nicaragua |
| 2017 | 230.14530858578 | 0.507482021944309 | 22.630767474499 | 207.514541111281 | Nicaragua |
| 2018 | 219.9479307974 | 0.512338227599941 | 22.6135788604249 | 197.334351936975 | Nicaragua |
| 2019 | 210.94996073851 | 0.516597045511253 | 22.2063779348515 | 188.743582803658 | Nicaragua |
| 2020 | 195.92530926457 | 0.520292670400273 | 22.1198958548837 | 173.805413409686 | Nicaragua |
| 2021 | 184.58523683778 | 0.523958472235925 | 22.2375661497462 | 162.347670688034 | Nicaragua |
| 1990 | 911.67640143789 | 0.546048123421677 | 20.6284106301397 | 891.04799080775 | Panama |
| 1991 | 861.07332763827 | 0.550484218189226 | 19.752723795705 | 841.320603842565 | Panama |
| 1992 | 762.87693720109 | 0.55497275492945 | 18.8326566009005 | 744.044280600189 | Panama |
| 1993 | 595.40774913852 | 0.55916866562951 | 17.7885882837865 | 577.619160854734 | Panama |
| 1994 | 537.48523889197 | 0.561714534049174 | 16.6550159069202 | 520.83022298505 | Panama |
| 1995 | 527.69890101802 | 0.56335302312626 | 16.4046411454726 | 511.294259872547 | Panama |
| 1996 | 519.86793203025 | 0.565310070207258 | 15.2231157454028 | 504.644816284847 | Panama |
| 1997 | 512.86736117668 | 0.569395938819109 | 13.887235011945 | 498.980126164735 | Panama |
| 1998 | 541.61600359455 | 0.574697317744379 | 13.3163877829634 | 528.299615811586 | Panama |
| 1999 | 477.57923641804 | 0.580432842358969 | 13.7477010926917 | 463.831535325348 | Panama |
| 2000 | 468.03910868507 | 0.587378392850747 | 13.5981582074217 | 454.440950477648 | Panama |
| 2001 | 466.22990576496 | 0.594535700601609 | 13.5005992953247 | 452.729306469635 | Panama |
| 2002 | 438.44057529778 | 0.600138677301078 | 13.4848245039 | 424.95575079388 | Panama |
| 2003 | 463.19812579418 | 0.604043364755692 | 13.3063908592286 | 449.891734934951 | Panama |
| 2004 | 475.26873617566 | 0.606877030015399 | 13.5474935339105 | 461.72124264175 | Panama |
| 2005 | 487.99756356302 | 0.609494124501125 | 13.3646894701725 | 474.632874092847 | Panama |
| 2006 | 420.9083458249 | 0.612249712793622 | 13.2079053895696 | 407.70044043533 | Panama |
| 2007 | 447.49310178324 | 0.615285417478616 | 13.1255768854714 | 434.367524897769 | Panama |
| 2008 | 395.01420857855 | 0.61895890692398 | 12.9897519477159 | 382.024456630834 | Panama |
| 2009 | 447.72393921755 | 0.622402805678368 | 12.9755524372321 | 434.748386780318 | Panama |
| 2010 | 393.64312948703 | 0.625116323303972 | 13.0679166672463 | 380.575212819784 | Panama |
| 2011 | 352.5463655275 | 0.628633942402595 | 12.5722633741464 | 339.974102153354 | Panama |
| 2012 | 340.73030824003 | 0.634263435389173 | 10.7915919664563 | 329.938716273574 | Panama |
| 2013 | 338.86238370603 | 0.641324352588811 | 7.57430642703329 | 331.288077278997 | Panama |
| 2014 | 319.77971885635 | 0.649108173564656 | 5.51675237671155 | 314.262966479638 | Panama |
| 2015 | 279.16052342635 | 0.657962046843146 | 4.87127240658512 | 274.289251019765 | Panama |
| 2016 | 320.17490711882 | 0.667276758021587 | 3.50568349855187 | 316.669223620268 | Panama |
| 2017 | 340.42724817339 | 0.676783503431544 | 2.83637515704198 | 337.590873016348 | Panama |
| 2018 | 321.39442258097 | 0.686719924606419 | 2.58303735526296 | 318.811385225707 | Panama |
| 2019 | 306.82917502283 | 0.696670680246603 | 2.39016068864633 | 304.439014334184 | Panama |
| 2020 | 274.97924838206 | 0.703788489528527 | 2.26987959093537 | 272.709368791125 | Panama |
| 2021 | 260.54632762105 | 0.708864827518009 | 2.26633770752681 | 258.279989913523 | Panama |
| 1990 | 1560.21398649314 | 0.516891218383329 | 22.2263700141129 | 1537.98761647903 | Venezuela (Bolivarian Republic of) |
| 1991 | 1188.51273914692 | 0.522272295981046 | 22.2230385106246 | 1166.2897006363 | Venezuela (Bolivarian Republic of) |
| 1992 | 1139.10307564518 | 0.529517990878198 | 22.1568072450841 | 1116.9462684001 | Venezuela (Bolivarian Republic of) |
| 1993 | 1150.79742018445 | 0.536279978196324 | 21.2927116900271 | 1129.50470849442 | Venezuela (Bolivarian Republic of) |
| 1994 | 1360.48542072769 | 0.541866057904128 | 21.2079155406534 | 1339.27750518704 | Venezuela (Bolivarian Republic of) |
| 1995 | 1299.43398198907 | 0.546348233569071 | 20.5402482447358 | 1278.89373374433 | Venezuela (Bolivarian Republic of) |
| 1996 | 1178.63675900565 | 0.548649226233627 | 20.5043305411687 | 1158.13242846448 | Venezuela (Bolivarian Republic of) |
| 1997 | 1001.31281904163 | 0.552364437231236 | 19.8469452725462 | 981.465873769084 | Venezuela (Bolivarian Republic of) |
| 1998 | 919.98210487007 | 0.557243117253564 | 18.6809600953251 | 901.301144774745 | Venezuela (Bolivarian Republic of) |
| 1999 | 797.07386884841 | 0.560277999100226 | 17.8421766695597 | 779.23169217885 | Venezuela (Bolivarian Republic of) |
| 2000 | 704.88721676191 | 0.56350626012771 | 16.1631869968102 | 688.7240297651 | Venezuela (Bolivarian Republic of) |
| 2001 | 657.93897576572 | 0.565458381141816 | 15.3641645853542 | 642.574811180366 | Venezuela (Bolivarian Republic of) |
| 2002 | 674.8322165081 | 0.562604461024786 | 16.604553170019 | 658.227663338081 | Venezuela (Bolivarian Republic of) |
| 2003 | 836.88365653155 | 0.555212224179424 | 18.8556917013652 | 818.027964830185 | Venezuela (Bolivarian Republic of) |
| 2004 | 658.5007125198 | 0.55097225564581 | 19.8946866175489 | 638.606025902251 | Venezuela (Bolivarian Republic of) |
| 2005 | 546.80828230158 | 0.553941679272419 | 18.9363549525403 | 527.87192734904 | Venezuela (Bolivarian Republic of) |
| 2006 | 437.88235147595 | 0.563334189710813 | 16.6398274347389 | 421.242524041211 | Venezuela (Bolivarian Republic of) |
| 2007 | 361.61676271497 | 0.575367174436532 | 13.5084912808876 | 348.108271434082 | Venezuela (Bolivarian Republic of) |
| 2008 | 309.67268882325 | 0.586184886513804 | 13.5119082342215 | 296.160780589028 | Venezuela (Bolivarian Republic of) |
| 2009 | 297.52265782045 | 0.593288421972038 | 13.4232832420223 | 284.099374578428 | Venezuela (Bolivarian Republic of) |
| 2010 | 310.22582250586 | 0.599127922953431 | 13.4020216751044 | 296.823800830756 | Venezuela (Bolivarian Republic of) |
| 2011 | 288.24029141019 | 0.604823483689087 | 13.0553891253861 | 275.184902284804 | Venezuela (Bolivarian Republic of) |
| 2012 | 266.32527719538 | 0.610187465323048 | 13.3049603457595 | 253.020316849621 | Venezuela (Bolivarian Republic of) |
| 2013 | 253.67965656895 | 0.614441024133659 | 13.354579347211 | 240.325077221739 | Venezuela (Bolivarian Republic of) |
| 2014 | 274.68628361935 | 0.614686806192499 | 13.3656936984516 | 261.320589920898 | Venezuela (Bolivarian Republic of) |
| 2015 | 292.03426746523 | 0.610675356525105 | 13.3663052859697 | 278.66796217926 | Venezuela (Bolivarian Republic of) |
| 2016 | 343.06304419749 | 0.606993212893547 | 13.333713152872 | 329.729331044618 | Venezuela (Bolivarian Republic of) |
| 2017 | 370.72417844999 | 0.605160130674623 | 13.4983548063937 | 357.225823643596 | Venezuela (Bolivarian Republic of) |
| 2018 | 351.87890630746 | 0.604068247287088 | 13.1293130633864 | 338.749593244074 | Venezuela (Bolivarian Republic of) |
| 2019 | 345.92731263788 | 0.602313886713059 | 13.3319331521681 | 332.595379485712 | Venezuela (Bolivarian Republic of) |
| 2020 | 324.85809778828 | 0.60003478118243 | 13.4510599500086 | 311.407037838271 | Venezuela (Bolivarian Republic of) |
| 2021 | 298.15981059755 | 0.596513058857335 | 13.1253154096961 | 285.034495187854 | Venezuela (Bolivarian Republic of) |
| 1990 | 2309.71781875249 | 0.500070509167008 | 23.4330211406695 | 2286.28479761182 | Brazil |
| 1991 | 2136.92162755235 | 0.50478479847852 | 22.9622341522772 | 2113.95939340007 | Brazil |
| 1992 | 1972.51886776547 | 0.508539943299266 | 22.7899761673693 | 1949.7288915981 | Brazil |
| 1993 | 1862.86390421526 | 0.512081793142278 | 22.5551909219735 | 1840.30871329329 | Brazil |
| 1994 | 1688.95846772206 | 0.515760842475151 | 22.2664226782061 | 1666.69204504385 | Brazil |
| 1995 | 1492.76417178155 | 0.519685962646796 | 22.3209453408196 | 1470.44322644073 | Brazil |
| 1996 | 1317.95724804215 | 0.523863329360558 | 22.1779512313805 | 1295.77929681077 | Brazil |
| 1997 | 1198.8813730917 | 0.528177888704233 | 22.0953195427643 | 1176.78605354894 | Brazil |
| 1998 | 1171.77124955961 | 0.532481169179124 | 22.1813058856351 | 1149.58994367397 | Brazil |
| 1999 | 1061.6979929692 | 0.536972034740505 | 21.2884683965514 | 1040.40952457265 | Brazil |
| 2000 | 920.55800102393 | 0.542051497254562 | 21.1723005299733 | 899.385700493957 | Brazil |
| 2001 | 812.68255139516 | 0.547304429543334 | 20.6352226681696 | 792.04732872699 | Brazil |
| 2002 | 741.94100218122 | 0.552776034620744 | 19.768443917073 | 722.172558264147 | Brazil |
| 2003 | 679.76248268399 | 0.55814571028409 | 17.8625839815572 | 661.899898702433 | Brazil |
| 2004 | 613.07521674025 | 0.563772906923854 | 15.950760656619 | 597.124456083631 | Brazil |
| 2005 | 566.72523757799 | 0.569458117692368 | 14.0615885008687 | 552.663649077121 | Brazil |
| 2006 | 512.94461583963 | 0.575338774786184 | 13.1968902864642 | 499.747725553166 | Brazil |
| 2007 | 439.86481039745 | 0.581588755483488 | 13.3534609002836 | 426.511349497166 | Brazil |
| 2008 | 382.0546513798 | 0.588077817972719 | 13.2541960592937 | 368.800455320506 | Brazil |
| 2009 | 326.77759207297 | 0.594073296681944 | 13.2785450349755 | 313.499047037995 | Brazil |
| 2010 | 275.1824708454 | 0.600445846154568 | 13.2972226412038 | 261.885248204196 | Brazil |
| 2011 | 224.84056635889 | 0.606777290886098 | 13.6647199445861 | 211.175846414304 | Brazil |
| 2012 | 204.59513202666 | 0.612619336859312 | 13.3298399152884 | 191.265292111372 | Brazil |
| 2013 | 185.14421426672 | 0.61821823662824 | 13.0944370578594 | 172.049777208861 | Brazil |
| 2014 | 154.8083974101 | 0.623433813964528 | 12.9433843319242 | 141.865013078176 | Brazil |
| 2015 | 136.1290334987 | 0.628149803496659 | 12.6284998347786 | 123.500533663921 | Brazil |
| 2016 | 127.10366994734 | 0.632235114321365 | 12.0593761189473 | 115.044293828393 | Brazil |
| 2017 | 111.81837111778 | 0.636422187733083 | 10.7453650347528 | 101.073006083027 | Brazil |
| 2018 | 96.68135147473 | 0.640802974287966 | 8.03899838501282 | 88.6423530897172 | Brazil |
| 2019 | 82.52288727299 | 0.645298005000543 | 5.655635498363 | 76.867251774627 | Brazil |
| 2020 | 68.22623878535 | 0.649201568351328 | 5.92021157093536 | 62.3060272144146 | Brazil |
| 2021 | 63.08091145062 | 0.65304388724724 | 4.99970248190694 | 58.0812089687131 | Brazil |
| 1990 | 1218.4059806048 | 0.469527785365773 | 56.4505402742256 | 1161.95544033057 | Paraguay |
| 1991 | 1168.73512042532 | 0.474352286489022 | 56.0043709654133 | 1112.73074945991 | Paraguay |
| 1992 | 1217.22729109617 | 0.479314168681291 | 55.5622103867851 | 1161.66508070938 | Paraguay |
| 1993 | 1127.80263204599 | 0.484614200390594 | 55.3007835341701 | 1072.50184851182 | Paraguay |
| 1994 | 1044.70349237763 | 0.490226934517601 | 49.7290764363171 | 994.974415941313 | Paraguay |
| 1995 | 991.6602118985 | 0.496336876135338 | 39.619778836379 | 952.040433062121 | Paraguay |
| 1996 | 903.65530294775 | 0.502322804124491 | 23.4124446378657 | 880.242858309884 | Paraguay |
| 1997 | 798.22921804166 | 0.508286947996083 | 22.5980292059125 | 775.631188835747 | Paraguay |
| 1998 | 712.44560497854 | 0.513819895911737 | 22.5594169856746 | 689.886187992865 | Paraguay |
| 1999 | 645.25805062998 | 0.518720583214312 | 22.2166105813911 | 623.041440048589 | Paraguay |
| 2000 | 583.56944720423 | 0.52300779310211 | 22.0981867578466 | 561.471260446383 | Paraguay |
| 2001 | 551.17448012029 | 0.526916763005844 | 22.1751568533733 | 528.999323266917 | Paraguay |
| 2002 | 529.44254272364 | 0.531177357815443 | 22.1167579452044 | 507.325784778436 | Paraguay |
| 2003 | 490.49776386511 | 0.53536012554352 | 21.6175558024682 | 468.880208062642 | Paraguay |
| 2004 | 448.97709468698 | 0.539556655420765 | 21.1822961975166 | 427.794798489463 | Paraguay |
| 2005 | 413.11614492818 | 0.543622697051923 | 21.1744529476482 | 391.941691980532 | Paraguay |
| 2006 | 373.27736038305 | 0.547943304899878 | 20.6981091876749 | 352.579251195375 | Paraguay |
| 2007 | 323.33730482613 | 0.552758676429378 | 19.7513419838708 | 303.585962842259 | Paraguay |
| 2008 | 285.81757464968 | 0.55801690264969 | 17.8145375438537 | 268.003037105826 | Paraguay |
| 2009 | 260.89996497243 | 0.562900058514746 | 16.538037714288 | 244.361927258142 | Paraguay |
| 2010 | 215.34866941356 | 0.568846562482396 | 14.2037925281306 | 201.144876885429 | Paraguay |
| 2011 | 200.25558418057 | 0.574954463115124 | 13.2139745716592 | 187.041609608911 | Paraguay |
| 2012 | 188.07241903575 | 0.580650185867766 | 13.2090168917669 | 174.863402143983 | Paraguay |
| 2013 | 176.63122439249 | 0.587247042264483 | 13.2548773284301 | 163.37634706406 | Paraguay |
| 2014 | 162.59022425778 | 0.593953153052431 | 13.445816893193 | 149.144407364587 | Paraguay |
| 2015 | 141.39659260232 | 0.600484125243426 | 13.9139330967643 | 127.482659505556 | Paraguay |
| 2016 | 134.58500811101 | 0.607018144917351 | 13.420873806055 | 121.164134304955 | Paraguay |
| 2017 | 135.68318580209 | 0.613581590966399 | 13.235915975156 | 122.447269826934 | Paraguay |
| 2018 | 132.23264747469 | 0.620032361543249 | 12.9222698875748 | 119.310377587115 | Paraguay |
| 2019 | 125.43028305036 | 0.626070139299015 | 13.048629759286 | 112.381653291074 | Paraguay |
| 2020 | 118.8706693396 | 0.631057688560962 | 12.1629826989879 | 106.707686640612 | Paraguay |
| 2021 | 117.31169511791 | 0.635718098852038 | 10.56080019328 | 106.75089492463 | Paraguay |
| 1990 | 235.06914543646 | 0.460486905849358 | 62.9398357023356 | 172.129309734124 | Algeria |
| 1991 | 212.35584851464 | 0.468319196672477 | 58.7847128738349 | 153.571135640805 | Algeria |
| 1992 | 194.63697863027 | 0.475950937138063 | 57.7108436121 | 136.92613501817 | Algeria |
| 1993 | 180.07239512912 | 0.483255591216591 | 52.0253425179887 | 128.047052611131 | Algeria |
| 1994 | 167.46111409269 | 0.490450660309052 | 48.9787978337906 | 118.482316258899 | Algeria |
| 1995 | 154.26141997308 | 0.497957543180561 | 24.6482392825791 | 129.613180690501 | Algeria |
| 1996 | 142.7828644658 | 0.505990967161627 | 22.9470347445448 | 119.835829721255 | Algeria |
| 1997 | 136.50097963285 | 0.514056565839869 | 22.3958035488087 | 114.105176084041 | Algeria |
| 1998 | 128.96409195898 | 0.522382418796352 | 22.1148129183017 | 106.849279040678 | Algeria |
| 1999 | 122.58172214477 | 0.530785582833015 | 22.1816232846667 | 100.400098860103 | Algeria |
| 2000 | 116.7229453183 | 0.539481507570335 | 21.1571524921643 | 95.5657928261357 | Algeria |
| 2001 | 111.67918319414 | 0.54752927722376 | 20.4429407580309 | 91.2362424361091 | Algeria |
| 2002 | 106.64547138734 | 0.555172221658197 | 18.725666547292 | 87.919804840048 | Algeria |
| 2003 | 102.35921921194 | 0.562536956171446 | 16.623804078368 | 85.735415133572 | Algeria |
| 2004 | 99.44319118245 | 0.569352851934701 | 14.2702212136092 | 85.1729699688408 | Algeria |
| 2005 | 94.95267248992 | 0.576001964539977 | 13.4271515010982 | 81.5255209888218 | Algeria |
| 2006 | 89.93664152143 | 0.582126361864573 | 13.404081835912 | 76.532559685518 | Algeria |
| 2007 | 84.30725979895 | 0.587790538325364 | 13.1387818414455 | 71.1684779575045 | Algeria |
| 2008 | 79.08458493204 | 0.593190973247903 | 13.4582216986366 | 65.6263632334034 | Algeria |
| 2009 | 73.48678283138 | 0.597870383895018 | 13.324056027774 | 60.162726803606 | Algeria |
| 2010 | 68.86605636242 | 0.602823534656761 | 13.3530501321959 | 55.5130062302241 | Algeria |
| 2011 | 64.51318789145 | 0.607825529405856 | 13.196651522221 | 51.316536369229 | Algeria |
| 2012 | 60.54638833301 | 0.612703533153522 | 13.3118620565643 | 47.2345262764457 | Algeria |
| 2013 | 57.85628782602 | 0.617422204461461 | 13.0479414859293 | 44.8083463400907 | Algeria |
| 2014 | 55.32116657095 | 0.622087291667118 | 12.946012349553 | 42.375154221397 | Algeria |
| 2015 | 52.37028806052 | 0.626745568394753 | 13.039285195584 | 39.331002864936 | Algeria |
| 2016 | 48.8515460089 | 0.631710735505334 | 12.0508574021512 | 36.8006886067488 | Algeria |
| 2017 | 44.4729574686 | 0.636973175875889 | 7.93562530531897 | 36.537332163281 | Algeria |
| 2018 | 41.42864806876 | 0.642500039273145 | 7.59202166054016 | 33.8366264082198 | Algeria |
| 2019 | 38.43084707166 | 0.648210784879009 | 5.82301616472922 | 32.6078309069308 | Algeria |
| 2020 | 35.24674531324 | 0.65365146606094 | 5.36750574409 | 29.87923956915 | Algeria |
| 2021 | 33.48807244532 | 0.659500924324217 | 4.50120523860347 | 28.9868672067165 | Algeria |
| 1990 | 111.07244795872 | 0.584578851787694 | 13.7620275532911 | 97.3104204054289 | Bahrain |
| 1991 | 104.31648966605 | 0.590596896738332 | 13.4319943871862 | 90.8844952788638 | Bahrain |
| 1992 | 97.24628071859 | 0.596040229262693 | 13.4319289746467 | 83.8143517439433 | Bahrain |
| 1993 | 89.6288999575 | 0.602165621752441 | 13.3557638512631 | 76.2731361062369 | Bahrain |
| 1994 | 81.63269718385 | 0.60813372491926 | 13.3956686674658 | 68.2370285163842 | Bahrain |
| 1995 | 72.84630918668 | 0.613850721750031 | 13.3792139472951 | 59.4670952393849 | Bahrain |
| 1996 | 63.69471665963 | 0.619485376148992 | 13.0640371072116 | 50.6306795524184 | Bahrain |
| 1997 | 57.87698206924 | 0.624596061581476 | 12.96751012129 | 44.90947194795 | Bahrain |
| 1998 | 54.84420932122 | 0.630738301217212 | 11.9756145889287 | 42.8685947322913 | Bahrain |
| 1999 | 52.21117377552 | 0.638083186958308 | 8.51722280382091 | 43.6939509716991 | Bahrain |
| 2000 | 50.8292625193 | 0.646750697322184 | 6.0568844202507 | 44.7723780990493 | Bahrain |
| 2001 | 50.45482729021 | 0.657196063695041 | 4.71018648790914 | 45.7446408023009 | Bahrain |
| 2002 | 50.34589637384 | 0.664901201084495 | 4.40555978839089 | 45.9403365854491 | Bahrain |
| 2003 | 50.67082599328 | 0.669564110612245 | 3.81458186368211 | 46.8562441295979 | Bahrain |
| 2004 | 50.816003347 | 0.674147775423852 | 3.31370767124337 | 47.5022956757566 | Bahrain |
| 2005 | 50.20470879834 | 0.679790598294553 | 2.88720630806579 | 47.3175024902742 | Bahrain |
| 2006 | 48.80480515238 | 0.686144289377392 | 2.69292528412789 | 46.1118798682521 | Bahrain |
| 2007 | 47.11467567439 | 0.693045146522931 | 2.48846786147295 | 44.626207812917 | Bahrain |
| 2008 | 39.8573203982 | 0.699806387259847 | 2.37840363661877 | 37.4789167615812 | Bahrain |
| 2009 | 37.91035131019 | 0.704885840093356 | 2.32183256887774 | 35.5885187413123 | Bahrain |
| 2010 | 38.65021343525 | 0.707902620468186 | 2.29206464487095 | 36.358148790379 | Bahrain |
| 2011 | 34.07623456713 | 0.708958738960692 | 2.24283953111707 | 31.8333950360129 | Bahrain |
| 2012 | 32.57245471992 | 0.710362417505028 | 2.25259912316597 | 30.319855596754 | Bahrain |
| 2013 | 31.04303183669 | 0.713343155899609 | 2.23573531161784 | 28.8072965250722 | Bahrain |
| 2014 | 29.77876565091 | 0.716768059555516 | 2.14938719695 | 27.62937845396 | Bahrain |
| 2015 | 28.29123273048 | 0.719926604884411 | 2.18586489914134 | 26.1053678313387 | Bahrain |
| 2016 | 26.40022873062 | 0.723611296528675 | 2.19433773146515 | 24.2058909991549 | Bahrain |
| 2017 | 24.82165988665 | 0.729323102530322 | 2.16763775440774 | 22.6540221322423 | Bahrain |
| 2018 | 23.15283145227 | 0.736192583172625 | 2.15644213731772 | 20.9963893149523 | Bahrain |
| 2019 | 21.73340507184 | 0.742847804247627 | 2.10812639390875 | 19.6252786779312 | Bahrain |
| 2020 | 19.3416268357 | 0.748103083095047 | 2.04913035054729 | 17.2924964851527 | Bahrain |
| 2021 | 18.4865589913 | 0.753043204117567 | 2.01278026117381 | 16.4737787301262 | Bahrain |
| 1990 | 1372.9226783598 | 0.417182741758852 | 261.09156390994 | 1111.83111444986 | Egypt |
| 1991 | 1200.53787552902 | 0.426909452028558 | 234.428416934812 | 966.109458594208 | Egypt |
| 1992 | 1050.40544446801 | 0.437744555478775 | 122.655781531078 | 927.749662936932 | Egypt |
| 1993 | 905.02715191007 | 0.448490053084385 | 66.2962151754701 | 838.7309367346 | Egypt |
| 1994 | 784.07372504478 | 0.458727004203869 | 67.4804308884354 | 716.593294156345 | Egypt |
| 1995 | 695.11608850227 | 0.468035368434364 | 58.7468270194376 | 636.369261482832 | Egypt |
| 1996 | 594.99154616874 | 0.476116627128094 | 56.2511297594342 | 538.740416409306 | Egypt |
| 1997 | 539.68923775529 | 0.482970886044098 | 53.2282226920723 | 486.461015063218 | Egypt |
| 1998 | 498.20474802831 | 0.489184070606589 | 50.1941108971483 | 448.010637131162 | Egypt |
| 1999 | 467.40170822637 | 0.495502619174384 | 49.3811158007196 | 418.02059242565 | Egypt |
| 2000 | 430.57318254967 | 0.502169886288414 | 23.2815526102764 | 407.291629939394 | Egypt |
| 2001 | 385.55958735627 | 0.508716824226239 | 22.8351088206854 | 362.724478535585 | Egypt |
| 2002 | 340.50427544882 | 0.515038129463872 | 22.5329030635829 | 317.971372385237 | Egypt |
| 2003 | 305.01330117963 | 0.520623395469455 | 22.1320237688382 | 282.881277410792 | Egypt |
| 2004 | 274.61166116602 | 0.525181677996097 | 22.1518559320532 | 252.459805233967 | Egypt |
| 2005 | 247.87647973765 | 0.528665483094012 | 22.1449650733861 | 225.731514664264 | Egypt |
| 2006 | 231.93212388293 | 0.530395807271736 | 22.0617228286519 | 209.870401054278 | Egypt |
| 2007 | 225.46187520257 | 0.529457593890747 | 22.2204479787161 | 203.241427223854 | Egypt |
| 2008 | 218.99147243193 | 0.5247438325858 | 22.0962496164238 | 196.895222815506 | Egypt |
| 2009 | 216.76589509507 | 0.51625583066946 | 22.22400927304 | 194.54188582203 | Egypt |
| 2010 | 207.43365958898 | 0.507715477095215 | 22.8309653769228 | 184.602694212057 | Egypt |
| 2011 | 192.29591074561 | 0.504594044306814 | 23.0012174598307 | 169.294693285779 | Egypt |
| 2012 | 177.66636004606 | 0.509157068362998 | 22.7588277723052 | 154.907532273755 | Egypt |
| 2013 | 169.44637328054 | 0.51933446386018 | 22.4043533290015 | 147.042019951539 | Egypt |
| 2014 | 151.2247631245 | 0.531749809455032 | 22.1978354583611 | 129.026927666139 | Egypt |
| 2015 | 141.18650656589 | 0.544370148675277 | 21.1388621231492 | 120.047644442741 | Egypt |
| 2016 | 132.01266202369 | 0.556012469227609 | 18.7418667812047 | 113.270795242485 | Egypt |
| 2017 | 123.9109984055 | 0.566914631824628 | 14.6308430331184 | 109.280155372382 | Egypt |
| 2018 | 116.97325300407 | 0.577442786164725 | 13.1671410340768 | 103.806111969993 | Egypt |
| 2019 | 111.51396965259 | 0.587736876524439 | 13.4096785176675 | 98.1042911349225 | Egypt |
| 2020 | 96.95553226383 | 0.597363341129599 | 13.6146289439528 | 83.3409033198772 | Egypt |
| 2021 | 92.29719910706 | 0.6067870937016 | 13.2825422160069 | 79.0146568910531 | Egypt |
| 1990 | 729.71817485764 | 0.453799943957447 | 72.4755336066151 | 657.242641251025 | Iran (Islamic Republic of) |
| 1991 | 676.02384945603 | 0.468982857673188 | 56.7381048221615 | 619.285744633868 | Iran (Islamic Republic of) |
| 1992 | 628.582993374 | 0.480626696650433 | 54.300075487291 | 574.282917886709 | Iran (Islamic Republic of) |
| 1993 | 596.00499813903 | 0.492277025057908 | 50.470720116782 | 545.534278022248 | Iran (Islamic Republic of) |
| 1994 | 569.09837310855 | 0.505072857378303 | 23.0023117122745 | 546.096061396276 | Iran (Islamic Republic of) |
| 1995 | 549.86806734067 | 0.517531493818527 | 22.2600454549556 | 527.608021885714 | Iran (Islamic Republic of) |
| 1996 | 495.425966834 | 0.528600719906526 | 22.1458905885493 | 473.280076245451 | Iran (Islamic Republic of) |
| 1997 | 471.41757142987 | 0.537727779314693 | 21.3675790862597 | 450.04999234361 | Iran (Islamic Republic of) |
| 1998 | 442.73454850608 | 0.546366017294819 | 20.5942131757079 | 422.140335330372 | Iran (Islamic Republic of) |
| 1999 | 415.83489825676 | 0.55562433168066 | 18.7400724654087 | 397.094825791351 | Iran (Islamic Republic of) |
| 2000 | 391.84486756159 | 0.565474561419707 | 15.104636975468 | 376.740230586122 | Iran (Islamic Republic of) |
| 2001 | 333.73233928854 | 0.574618208021481 | 13.3784591243656 | 320.353880164174 | Iran (Islamic Republic of) |
| 2002 | 305.6097712124 | 0.584097268490781 | 13.4568649932659 | 292.152906219134 | Iran (Islamic Republic of) |
| 2003 | 280.59866530595 | 0.593963012213832 | 13.4346646946391 | 267.164000611311 | Iran (Islamic Republic of) |
| 2004 | 260.39647205317 | 0.603717449978768 | 13.1083455438281 | 247.288126509342 | Iran (Islamic Republic of) |
| 2005 | 243.79229651268 | 0.613291055585393 | 13.20242217851 | 230.58987433417 | Iran (Islamic Republic of) |
| 2006 | 225.7848277021 | 0.621268095172314 | 13.002255226374 | 212.782572475726 | Iran (Islamic Republic of) |
| 2007 | 208.4015109229 | 0.627749441937933 | 12.7494220943618 | 195.652088828538 | Iran (Islamic Republic of) |
| 2008 | 190.35476556511 | 0.632862230135051 | 11.0193973228203 | 179.33536824229 | Iran (Islamic Republic of) |
| 2009 | 171.13673113186 | 0.637398379541013 | 7.92725519971367 | 163.209475932146 | Iran (Islamic Republic of) |
| 2010 | 153.85609581403 | 0.642559170275463 | 7.14587228912107 | 146.710223524909 | Iran (Islamic Republic of) |
| 2011 | 142.42210260894 | 0.647899089013155 | 5.9269661908896 | 136.49513641805 | Iran (Islamic Republic of) |
| 2012 | 129.58722728231 | 0.651500586996546 | 5.74249970410616 | 123.844727578204 | Iran (Islamic Republic of) |
| 2013 | 122.1474521285 | 0.654475289198172 | 4.81512287400188 | 117.332329254498 | Iran (Islamic Republic of) |
| 2014 | 110.12560483574 | 0.657961510912541 | 4.9404184757347 | 105.185186360005 | Iran (Islamic Republic of) |
| 2015 | 97.28717199071 | 0.662057986508516 | 4.41418287637521 | 92.8729891143348 | Iran (Islamic Republic of) |
| 2016 | 80.43212079332 | 0.667652445135305 | 3.67322019665024 | 76.7589005966698 | Iran (Islamic Republic of) |
| 2017 | 69.5774251153 | 0.674343462089889 | 2.8842085567513 | 66.6932165585487 | Iran (Islamic Republic of) |
| 2018 | 62.11341018452 | 0.680909620594576 | 2.93021353996957 | 59.1831966445504 | Iran (Islamic Republic of) |
| 2019 | 57.51849248558 | 0.686741661948379 | 2.86151315062348 | 54.6569793349565 | Iran (Islamic Republic of) |
| 2020 | 40.68504750175 | 0.691918763371909 | 2.56129062872833 | 38.1237568730217 | Iran (Islamic Republic of) |
| 2021 | 28.58819953186 | 0.697207397661899 | 2.43366697363909 | 26.1545325582209 | Iran (Islamic Republic of) |
| 1990 | 574.32125409988 | 0.412044172813982 | 289.301788382424 | 285.019465717456 | Iraq |
| 1991 | 592.59942374802 | 0.415814115814778 | 278.781343526281 | 313.818080221739 | Iraq |
| 1992 | 586.20962214215 | 0.42035359441045 | 244.325302374125 | 341.884319768025 | Iraq |
| 1993 | 562.5953623997 | 0.42483493253075 | 231.335843872264 | 331.259518527436 | Iraq |
| 1994 | 535.76910584567 | 0.428751730675342 | 214.467954189748 | 321.301151655922 | Iraq |
| 1995 | 519.69668174993 | 0.432075545408255 | 204.56788414266 | 315.12879760727 | Iraq |
| 1996 | 505.75586669968 | 0.436818478749736 | 126.69530006007 | 379.06056663961 | Iraq |
| 1997 | 480.9545554025 | 0.44310402693832 | 113.08880504773 | 367.86575035477 | Iraq |
| 1998 | 450.32699396437 | 0.452384092352145 | 73.6343859986357 | 376.692607965734 | Iraq |
| 1999 | 422.77421381752 | 0.462736441391784 | 59.5114746808203 | 363.2627391367 | Iraq |
| 2000 | 388.45106915672 | 0.472376399238703 | 54.9651949283947 | 333.485874228325 | Iraq |
| 2001 | 358.53021277211 | 0.481458319619666 | 52.8899645958684 | 305.640248176242 | Iraq |
| 2002 | 337.71139565941 | 0.488664736761323 | 50.254048321357 | 287.457347338053 | Iraq |
| 2003 | 330.87935983167 | 0.492174444727086 | 50.2750802667237 | 280.604279564946 | Iraq |
| 2004 | 308.81366255722 | 0.498933881288036 | 24.1205649692119 | 284.693097588008 | Iraq |
| 2005 | 289.34663121265 | 0.504719430828624 | 23.0287296860747 | 266.317901526575 | Iraq |
| 2006 | 269.87584188799 | 0.510918049053858 | 22.5752033598218 | 247.300638528168 | Iraq |
| 2007 | 258.97811181463 | 0.517698972378274 | 22.4698392230402 | 236.50827259159 | Iraq |
| 2008 | 239.07914075709 | 0.525242907568095 | 22.1673860149238 | 216.911754742166 | Iraq |
| 2009 | 215.66158785219 | 0.533200913619797 | 22.1350864214277 | 193.526501430762 | Iraq |
| 2010 | 197.85721707695 | 0.542343278401218 | 21.212944474086 | 176.644272602864 | Iraq |
| 2011 | 179.93506906729 | 0.553048555118566 | 19.8680854068422 | 160.066983660448 | Iraq |
| 2012 | 158.0459317168 | 0.564745467448645 | 16.2142758621571 | 141.831655854643 | Iraq |
| 2013 | 143.57671859958 | 0.576968997890727 | 13.2674469531578 | 130.309271646422 | Iraq |
| 2014 | 140.49810909902 | 0.588317651574763 | 13.1027361926627 | 127.395372906357 | Iraq |
| 2015 | 128.28181992705 | 0.599494007105483 | 13.5018212557208 | 114.779998671329 | Iraq |
| 2016 | 121.15046954024 | 0.611508645751767 | 13.3567733821189 | 107.793696158121 | Iraq |
| 2017 | 115.21190563459 | 0.622674531557115 | 12.8873780310841 | 102.324527603506 | Iraq |
| 2018 | 108.17419986938 | 0.633232108100178 | 11.0796455529696 | 97.0945543164104 | Iraq |
| 2019 | 116.70104154975 | 0.64363531790137 | 7.8473035155864 | 108.853738034164 | Iraq |
| 2020 | 97.6961379723 | 0.653300379638783 | 4.88857965131729 | 92.8075583209827 | Iraq |
| 2021 | 93.93130775661 | 0.662626230762701 | 4.52685561524418 | 89.4044521413658 | Iraq |
| 1990 | 100.63938093027 | 0.53914746775111 | 21.210338435342 | 79.429042494928 | Jordan |
| 1991 | 96.42603221581 | 0.54281941081865 | 21.1837052642204 | 75.2423269515896 | Jordan |
| 1992 | 91.59939722363 | 0.547458846457137 | 20.5912772804466 | 71.0081199431834 | Jordan |
| 1993 | 87.03252760554 | 0.552182350404081 | 19.7409710427078 | 67.2915565628322 | Jordan |
| 1994 | 83.1003543275 | 0.557254334819626 | 18.5005473189063 | 64.5998070085937 | Jordan |
| 1995 | 79.58110317527 | 0.562571536302321 | 16.8541427244162 | 62.7269604508538 | Jordan |
| 1996 | 76.48591361693 | 0.567529655438914 | 14.5064434075971 | 61.979470209333 | Jordan |
| 1997 | 73.10330411627 | 0.572581508488338 | 13.1447510220379 | 59.9585530942321 | Jordan |
| 1998 | 71.31299734465 | 0.577643570372855 | 13.2805877149148 | 58.0324096297352 | Jordan |
| 1999 | 68.78419575762 | 0.582759198560708 | 13.5425493267103 | 55.2416464309097 | Jordan |
| 2000 | 66.13559782215 | 0.587909393807207 | 13.5921910541812 | 52.5434067679688 | Jordan |
| 2001 | 63.34545954007 | 0.593182802809495 | 13.5464366696714 | 49.7990228703986 | Jordan |
| 2002 | 60.3572225057 | 0.59900038468688 | 13.2646060073994 | 47.0926164983006 | Jordan |
| 2003 | 56.7571980223 | 0.60494595024538 | 13.3090777765492 | 43.4481202457508 | Jordan |
| 2004 | 54.02217372184 | 0.611934403689721 | 13.4438757096471 | 40.5782980121929 | Jordan |
| 2005 | 51.44990106832 | 0.619909227769316 | 13.0785525052846 | 38.3713485630354 | Jordan |
| 2006 | 47.99793291724 | 0.627803812947762 | 12.4938752121185 | 35.5040577051215 | Jordan |
| 2007 | 44.37742137704 | 0.635670276218909 | 10.864862187925 | 33.512559189115 | Jordan |
| 2008 | 41.93867558597 | 0.643892693421901 | 7.65011156739419 | 34.2885640185758 | Jordan |
| 2009 | 40.0109232382 | 0.652196319480578 | 4.6176313706462 | 35.3932918675538 | Jordan |
| 2010 | 37.93717977669 | 0.659949494519026 | 4.52530273019351 | 33.4118770464965 | Jordan |
| 2011 | 36.48089475035 | 0.667396679645637 | 3.7953311065103 | 32.6855636438397 | Jordan |
| 2012 | 35.62452100643 | 0.674568924665591 | 3.14995805335027 | 32.4745629530797 | Jordan |
| 2013 | 34.7966745455 | 0.681119962167508 | 2.85094606175606 | 31.9457284837439 | Jordan |
| 2014 | 33.76620059021 | 0.687134039943201 | 2.58941595527585 | 31.1767846349341 | Jordan |
| 2015 | 29.11299023206 | 0.692952031873164 | 2.42969333617433 | 26.6832968958857 | Jordan |
| 2016 | 28.1531757427 | 0.698473302300541 | 2.4281365790108 | 25.7250391636892 | Jordan |
| 2017 | 26.61223279014 | 0.703923826241846 | 2.28223051073373 | 24.3300022794063 | Jordan |
| 2018 | 24.43385366499 | 0.709412879293601 | 2.19376508042309 | 22.2400885845669 | Jordan |
| 2019 | 23.6565899565 | 0.714998945753142 | 2.246586569224 | 21.410003387276 | Jordan |
| 2020 | 22.21116176885 | 0.720068239915947 | 2.20846500482986 | 20.0026967640201 | Jordan |
| 2021 | 22.2279610469 | 0.725307226832443 | 2.19414120847754 | 20.0338198384225 | Jordan |
| 1990 | 54.59250177748 | 0.66451790400099 | 4.51576523639729 | 50.0767365410827 | Kuwait |
| 1991 | 33.8401840189 | 0.66880962078386 | 3.711511013695 | 30.128673005205 | Kuwait |
| 1992 | 35.3937346175 | 0.670159110400931 | 3.65039294004736 | 31.7433416774526 | Kuwait |
| 1993 | 29.98375222201 | 0.670036252235978 | 3.56536044289868 | 26.4183917791113 | Kuwait |
| 1994 | 36.13902333974 | 0.671393212861768 | 3.57632416073034 | 32.5626991790097 | Kuwait |
| 1995 | 22.23110956429 | 0.677215523332779 | 2.90191425120423 | 19.3291953130858 | Kuwait |
| 1996 | 16.40030203204 | 0.686093303524213 | 2.92070670962403 | 13.479595322416 | Kuwait |
| 1997 | 16.55241951985 | 0.695789388591065 | 2.39825926420698 | 14.154160255643 | Kuwait |
| 1998 | 19.92005294063 | 0.704383735664098 | 2.30690646152516 | 17.6131464791048 | Kuwait |
| 1999 | 24.57876810351 | 0.711841328373508 | 2.23525970761176 | 22.3435083958982 | Kuwait |
| 2000 | 27.40377809395 | 0.718357562401374 | 2.22816783344343 | 25.1756102605066 | Kuwait |
| 2001 | 22.14369653902 | 0.722840631295124 | 2.17144268941705 | 19.972253849603 | Kuwait |
| 2002 | 24.36701308021 | 0.72681697562953 | 2.17241230027348 | 22.1946007799365 | Kuwait |
| 2003 | 25.57229546515 | 0.731778684982861 | 2.16124379346348 | 23.4110516716865 | Kuwait |
| 2004 | 24.16074623292 | 0.738153706974425 | 2.13941440347418 | 22.0213318294458 | Kuwait |
| 2005 | 24.17412272195 | 0.746384913346372 | 2.06096961085833 | 22.1131531110917 | Kuwait |
| 2006 | 22.4011672259 | 0.754928492574677 | 1.98450794955303 | 20.416659276347 | Kuwait |
| 2007 | 23.57549293242 | 0.762411483964357 | 1.70747800092197 | 21.868014931498 | Kuwait |
| 2008 | 24.30920031095 | 0.770002614917808 | 1.58940309778436 | 22.7197972131656 | Kuwait |
| 2009 | 21.87134055455 | 0.777546551267578 | 1.5400800644794 | 20.3312604900706 | Kuwait |
| 2010 | 18.49316476891 | 0.784873929223183 | 1.520248330065 | 16.972916438845 | Kuwait |
| 2011 | 18.53892881552 | 0.791812065474587 | 1.45422320569915 | 17.0847056098208 | Kuwait |
| 2012 | 18.81766468682 | 0.798356987724222 | 1.436496993695 | 17.381167693125 | Kuwait |
| 2013 | 17.99285366899 | 0.80458383431311 | 1.45976504836386 | 16.5330886206261 | Kuwait |
| 2014 | 17.71120453841 | 0.810743232598608 | 1.45757595115259 | 16.2536285872574 | Kuwait |
| 2015 | 16.66733504381 | 0.816998412782804 | 1.4362225475986 | 15.2311124962114 | Kuwait |
| 2016 | 16.19701975081 | 0.823026527054531 | 1.43482848774167 | 14.7621912630683 | Kuwait |
| 2017 | 14.19888184056 | 0.828886986643824 | 1.41329959334562 | 12.7855822472144 | Kuwait |
| 2018 | 13.56309330588 | 0.834371461321704 | 1.43404840491544 | 12.1290449009646 | Kuwait |
| 2019 | 13.27073936937 | 0.839688306553232 | 1.41926532816263 | 11.8514740412074 | Kuwait |
| 2020 | 11.83777782618 | 0.843459812023503 | 1.3733085435574 | 10.4644692826226 | Kuwait |
| 2021 | 11.61074850548 | 0.846651054742869 | 1.355528055073 | 10.255220450407 | Kuwait |
| 1990 | 207.98041123199 | 0.536718968751988 | 21.2626781807172 | 186.717733051273 | Lebanon |
| 1991 | 198.74014113622 | 0.539197491838137 | 21.3060811318501 | 177.43406000437 | Lebanon |
| 1992 | 188.68604237674 | 0.541793515767809 | 21.1692737532081 | 167.516768623532 | Lebanon |
| 1993 | 215.62519390449 | 0.544581306172966 | 21.1740151900153 | 194.451178714475 | Lebanon |
| 1994 | 169.33608266189 | 0.547684033143162 | 20.4625471245123 | 148.873535537378 | Lebanon |
| 1995 | 160.85858690055 | 0.5514717204066 | 19.8445019522501 | 141.0140849483 | Lebanon |
| 1996 | 151.85899922784 | 0.556436158565937 | 18.7840546289632 | 133.074944598877 | Lebanon |
| 1997 | 142.1151049563 | 0.562120817980966 | 16.701056870202 | 125.414048086098 | Lebanon |
| 1998 | 134.61041093069 | 0.569059070131885 | 13.9932463596114 | 120.617164571079 | Lebanon |
| 1999 | 129.46689754165 | 0.576078527772757 | 13.4232430741509 | 116.043654467499 | Lebanon |
| 2000 | 123.161608508 | 0.582698277151947 | 13.1023707599134 | 110.059237748087 | Lebanon |
| 2001 | 120.04885650667 | 0.589185502386114 | 13.3214014002182 | 106.727455106452 | Lebanon |
| 2002 | 114.20571156785 | 0.595978787643548 | 13.4915770568524 | 100.714134510998 | Lebanon |
| 2003 | 108.94801222781 | 0.602967240409336 | 13.408489619597 | 95.539522608213 | Lebanon |
| 2004 | 103.65683816832 | 0.610845760385912 | 13.5598943510805 | 90.0969438172395 | Lebanon |
| 2005 | 99.8697723406 | 0.619391355094372 | 12.9772126409089 | 86.8925596996911 | Lebanon |
| 2006 | 93.21410291031 | 0.627791217131142 | 12.6765200544704 | 80.5375828558396 | Lebanon |
| 2007 | 88.69650009539 | 0.636938234123424 | 8.63511262011949 | 80.0613874752705 | Lebanon |
| 2008 | 84.01938096256 | 0.646667663001479 | 5.79098965653616 | 78.2283913060238 | Lebanon |
| 2009 | 82.29352994074 | 0.657433186212217 | 4.87198160891077 | 77.4215483318292 | Lebanon |
| 2010 | 79.29983982336 | 0.668804524925046 | 3.58578235057941 | 75.7140574727806 | Lebanon |
| 2011 | 77.77548204026 | 0.680920616624062 | 2.86988414331681 | 74.9055978969432 | Lebanon |
| 2012 | 75.92011690247 | 0.693910983172664 | 2.44880431906373 | 73.4713125834063 | Lebanon |
| 2013 | 70.96376399773 | 0.704811001480686 | 2.28623455286667 | 68.6775294448633 | Lebanon |
| 2014 | 65.42693595052 | 0.71242387252594 | 2.22505619363217 | 63.2018797568878 | Lebanon |
| 2015 | 63.90327242893 | 0.7186972923939 | 2.20202453729824 | 61.7012478916318 | Lebanon |
| 2016 | 64.17360195381 | 0.724583346730978 | 2.15821913589901 | 62.015382817911 | Lebanon |
| 2017 | 63.68833137489 | 0.729963293744747 | 2.16810002148173 | 61.5202313534083 | Lebanon |
| 2018 | 61.94934780224 | 0.734746243401704 | 2.18919970415513 | 59.7601480980849 | Lebanon |
| 2019 | 60.46557386132 | 0.738644009352405 | 2.15920412003159 | 58.3063697412884 | Lebanon |
| 2020 | 55.76515210912 | 0.742009720142934 | 2.0691408125971 | 53.6960112965229 | Lebanon |
| 2021 | 55.06143595577 | 0.74474635096338 | 2.07588168367488 | 52.9855542720951 | Lebanon |
| 1990 | 295.29650814283 | 0.527998168231366 | 22.1822313228422 | 273.114276819988 | Libya |
| 1991 | 249.3145956388 | 0.541432398663791 | 21.1687995589409 | 228.145796079859 | Libya |
| 1992 | 206.56098436594 | 0.55446822725249 | 18.737365208606 | 187.823619157334 | Libya |
| 1993 | 178.20713525531 | 0.566798415093121 | 14.7055178627277 | 163.501617392582 | Libya |
| 1994 | 157.24144612969 | 0.578651415542216 | 13.2894696012516 | 143.951976528438 | Libya |
| 1995 | 137.10485266637 | 0.589578634415899 | 13.2476683603978 | 123.857184305972 | Libya |
| 1996 | 110.88510199982 | 0.60035004409905 | 13.5764718134512 | 97.3086301863687 | Libya |
| 1997 | 100.50141867948 | 0.610620315262943 | 13.4077913776444 | 87.0936273018356 | Libya |
| 1998 | 90.34198697427 | 0.619973983675942 | 12.9253240153788 | 77.4166629588912 | Libya |
| 1999 | 82.04566382718 | 0.628600604307387 | 12.5972492691495 | 69.4484145580305 | Libya |
| 2000 | 73.86791463749 | 0.63704793460596 | 8.30448813104203 | 65.563426506448 | Libya |
| 2001 | 68.80643937156 | 0.644685131438978 | 6.3111567097423 | 62.4952826618177 | Libya |
| 2002 | 64.91692920759 | 0.651669147460857 | 5.15346849029662 | 59.7634607172934 | Libya |
| 2003 | 61.37564709831 | 0.659345124852632 | 4.65147320563083 | 56.7241738926792 | Libya |
| 2004 | 56.71067647862 | 0.666913926262631 | 3.78557276798676 | 52.9251037106332 | Libya |
| 2005 | 53.24230492414 | 0.675322267622666 | 3.22435401482209 | 50.0179509093179 | Libya |
| 2006 | 50.85803205846 | 0.683701999618589 | 2.80618580041592 | 48.0518462580441 | Libya |
| 2007 | 48.6549702902 | 0.691855134182363 | 2.43065284261642 | 46.2243174475836 | Libya |
| 2008 | 45.41752508463 | 0.698873253326827 | 2.32793244405153 | 43.0895926405785 | Libya |
| 2009 | 41.61787467775 | 0.704998470605237 | 2.29376463696942 | 39.3241100407806 | Libya |
| 2010 | 40.43048288244 | 0.711362496328616 | 2.26872654843062 | 38.1617563340094 | Libya |
| 2011 | 37.16199278598 | 0.711466657224803 | 2.27313428265462 | 34.8888585033254 | Libya |
| 2012 | 35.3921202803 | 0.716700693100353 | 2.20156384389466 | 33.1905564364053 | Libya |
| 2013 | 30.77890336692 | 0.717665249612534 | 2.19024424493765 | 28.5886591219824 | Libya |
| 2014 | 31.19863187329 | 0.716042207086096 | 2.22706817064767 | 28.9715637026423 | Libya |
| 2015 | 31.95643972827 | 0.713651367022809 | 2.21438184021716 | 29.7420578880528 | Libya |
| 2016 | 35.12605352302 | 0.710760140384142 | 2.21755966638316 | 32.9084938566368 | Libya |
| 2017 | 35.67689959993 | 0.71066626361534 | 2.20761258115418 | 33.4692870187758 | Libya |
| 2018 | 32.271960429 | 0.712720437649946 | 2.25457531759043 | 30.0173851114096 | Libya |
| 2019 | 31.08127075035 | 0.716157238515615 | 2.19112891206367 | 28.8901418382863 | Libya |
| 2020 | 30.0475247108 | 0.720270392863655 | 2.20314258849898 | 27.844382122301 | Libya |
| 2021 | 29.76793901 | 0.725771398888229 | 2.17791230014306 | 27.5900267098569 | Libya |
| 1990 | 2417.33772615983 | 0.358072869533996 | 589.73775881669 | 1827.59996734314 | Morocco |
| 1991 | 2186.12452033269 | 0.3649846287647 | 574.682318973768 | 1611.44220135892 | Morocco |
| 1992 | 2016.71769804851 | 0.371187194023026 | 545.909695663472 | 1470.80800238504 | Morocco |
| 1993 | 1859.95598167534 | 0.37677897731043 | 543.00548152333 | 1316.95050015201 | Morocco |
| 1994 | 1718.81377304713 | 0.382849450670084 | 510.027874879551 | 1208.78589816758 | Morocco |
| 1995 | 1594.382484471 | 0.388164109911114 | 515.25556397324 | 1079.12692049776 | Morocco |
| 1996 | 1475.07555638583 | 0.394150096117346 | 468.468955513307 | 1006.60660087252 | Morocco |
| 1997 | 1375.61126641032 | 0.399504009010139 | 460.374999245986 | 915.236267164334 | Morocco |
| 1998 | 1281.21637721259 | 0.405038791986878 | 397.203559276109 | 884.012817936481 | Morocco |
| 1999 | 1195.08002861168 | 0.410303190621115 | 314.551370138439 | 880.528658473241 | Morocco |
| 2000 | 1117.92628320458 | 0.415344026415974 | 274.743794717267 | 843.182488487313 | Morocco |
| 2001 | 1048.7497479808 | 0.420595133422507 | 248.897849556632 | 799.851898424168 | Morocco |
| 2002 | 986.47346342773 | 0.425775750183888 | 234.185181667306 | 752.288281760424 | Morocco |
| 2003 | 908.13233387103 | 0.431217810181359 | 205.2272126785 | 702.90512119253 | Morocco |
| 2004 | 848.71148149761 | 0.436822351876544 | 120.096983227923 | 728.614498269687 | Morocco |
| 2005 | 792.41026813143 | 0.442531339370103 | 111.180195429824 | 681.230072701606 | Morocco |
| 2006 | 733.2164774566 | 0.448676160448964 | 71.2940438817781 | 661.922433574822 | Morocco |
| 2007 | 670.68994817298 | 0.455036324601373 | 74.099511827354 | 596.590436345626 | Morocco |
| 2008 | 604.97689072604 | 0.461803552255995 | 62.045232324926 | 542.931658401114 | Morocco |
| 2009 | 555.8901161757 | 0.468929893059803 | 56.5777321604326 | 499.312384015267 | Morocco |
| 2010 | 481.44124090177 | 0.476269799414457 | 58.1826968084299 | 423.25854409334 | Morocco |
| 2011 | 412.01887386677 | 0.483922151996701 | 53.882830256783 | 358.136043609987 | Morocco |
| 2012 | 357.91066876948 | 0.491603391761499 | 49.8803007458818 | 308.030368023598 | Morocco |
| 2013 | 315.81127582852 | 0.499528606645883 | 24.09166055187 | 291.71961527665 | Morocco |
| 2014 | 305.25357701544 | 0.507443265160336 | 22.6428511259554 | 282.610725889485 | Morocco |
| 2015 | 277.12801819665 | 0.515582892643316 | 22.3339236109684 | 254.794094585682 | Morocco |
| 2016 | 251.48781567433 | 0.523518592215067 | 22.1230836372992 | 229.364732037031 | Morocco |
| 2017 | 228.26945204119 | 0.531610861944961 | 22.1636062931139 | 206.105845748076 | Morocco |
| 2018 | 208.65635926056 | 0.539729670716936 | 21.187614947661 | 187.468744312899 | Morocco |
| 2019 | 186.78024706491 | 0.547837444469332 | 20.5956969730118 | 166.184550091898 | Morocco |
| 2020 | 169.21735276561 | 0.555246068582659 | 18.5622841979279 | 150.655068567682 | Morocco |
| 2021 | 157.09624296627 | 0.562698300927195 | 16.6991615663777 | 140.397081399892 | Morocco |
| 1990 | 378.04340150672 | 0.401792210145272 | 378.04340150672 | 0 | Palestine |
| 1991 | 337.59871700898 | 0.405727150469488 | 337.59871700898 | 0 | Palestine |
| 1992 | 297.4206323159 | 0.411146868747336 | 281.501095778981 | 15.9195365369193 | Palestine |
| 1993 | 259.05795623044 | 0.416779648694491 | 252.94678001226 | 6.11117621817954 | Palestine |
| 1994 | 223.29793602904 | 0.423136672782837 | 223.29793602904 | 0 | Palestine |
| 1995 | 193.93283368589 | 0.42930702715431 | 193.93283368589 | 0 | Palestine |
| 1996 | 169.85267891841 | 0.434731371082746 | 169.85267891841 | 0 | Palestine |
| 1997 | 149.7620237503 | 0.440901776082897 | 106.554198064698 | 43.2078256856017 | Palestine |
| 1998 | 132.92766929457 | 0.447782415404353 | 76.4632282742446 | 56.4644410203254 | Palestine |
| 1999 | 120.06009108854 | 0.454909692070064 | 69.0542291895041 | 51.0058618990359 | Palestine |
| 2000 | 111.59581175155 | 0.460681496412606 | 64.232937365264 | 47.362874386286 | Palestine |
| 2001 | 105.87219731676 | 0.465299102197142 | 63.1549693831534 | 42.7172279336066 | Palestine |
| 2002 | 102.36967578436 | 0.468600256734998 | 57.168703764236 | 45.200972020124 | Palestine |
| 2003 | 98.63363973815 | 0.472881732260502 | 58.3242406589829 | 40.3093990791671 | Palestine |
| 2004 | 94.98309735192 | 0.478527322159265 | 52.4439070571207 | 42.5391902947993 | Palestine |
| 2005 | 93.31263288781 | 0.485320585803801 | 54.5031185798285 | 38.8095143079815 | Palestine |
| 2006 | 89.23497093293 | 0.491890406552455 | 50.0116339800677 | 39.2233369528623 | Palestine |
| 2007 | 86.17035028201 | 0.498978425791333 | 24.0730112557336 | 62.0973390262764 | Palestine |
| 2008 | 79.52320923454 | 0.506849478804306 | 22.7764475555445 | 56.7467616789955 | Palestine |
| 2009 | 71.78180681156 | 0.515815835660256 | 22.2248562405555 | 49.5569505710045 | Palestine |
| 2010 | 64.75327250501 | 0.525711905369362 | 22.1419664249385 | 42.6113060800715 | Palestine |
| 2011 | 60.25764504955 | 0.536777629566224 | 21.2559781371948 | 39.0016669123552 | Palestine |
| 2012 | 55.76934279044 | 0.548295425023352 | 20.5440296242496 | 35.2253131661903 | Palestine |
| 2013 | 51.89840438008 | 0.55916348091297 | 18.154377460183 | 33.744026919897 | Palestine |
| 2014 | 48.50555239804 | 0.569239881995993 | 14.1621064612736 | 34.3434459367664 | Palestine |
| 2015 | 46.17027938454 | 0.579204260933414 | 13.0316719496056 | 33.1386074349344 | Palestine |
| 2016 | 38.05713477769 | 0.589492769984704 | 13.6824906586172 | 24.3746441190728 | Palestine |
| 2017 | 35.80444950998 | 0.599157186635533 | 13.2931230969609 | 22.5113264130191 | Palestine |
| 2018 | 33.24449529626 | 0.608124860877158 | 13.1694399849434 | 20.0750553113166 | Palestine |
| 2019 | 30.94950180251 | 0.616466838514416 | 12.7575383817817 | 18.1919634207283 | Palestine |
| 2020 | 27.97232387645 | 0.623810015023286 | 12.811853540542 | 15.160470335908 | Palestine |
| 2021 | 27.00842134263 | 0.631011665054344 | 11.9437102615875 | 15.0647110810425 | Palestine |
| 1990 | 292.41531370773 | 0.429270948783208 | 213.466072025341 | 78.949241682389 | Oman |
| 1991 | 258.71184384925 | 0.442268122012418 | 114.174451443769 | 144.537392405481 | Oman |
| 1992 | 221.5768709697 | 0.457737972074553 | 66.9707882650246 | 154.606082704675 | Oman |
| 1993 | 194.36269978374 | 0.474999032775331 | 58.2483894734555 | 136.114310310284 | Oman |
| 1994 | 174.10174393836 | 0.493513107048941 | 49.4571530739066 | 124.644590864453 | Oman |
| 1995 | 152.86361113777 | 0.513194359370639 | 22.5259428917611 | 130.337668246009 | Oman |
| 1996 | 134.77193328793 | 0.533246393920158 | 22.1591641384832 | 112.612769149447 | Oman |
| 1997 | 119.96935855776 | 0.554315537130654 | 18.9470851056253 | 101.022273452135 | Oman |
| 1998 | 108.58028785109 | 0.574474902223599 | 13.4227536746692 | 95.1575341764208 | Oman |
| 1999 | 98.98760103482 | 0.592398470428405 | 13.0384875439216 | 85.9491134908984 | Oman |
| 2000 | 89.83858292972 | 0.608421540646462 | 13.3995509422395 | 76.4390319874805 | Oman |
| 2001 | 81.92455607074 | 0.622160627189522 | 12.9611068686698 | 68.9634492020702 | Oman |
| 2002 | 75.44001486664 | 0.63516212584598 | 11.0858829710626 | 64.3541318955774 | Oman |
| 2003 | 69.88803273447 | 0.647249092690596 | 5.9849975115139 | 63.9030352229561 | Oman |
| 2004 | 65.20325839311 | 0.65762611814533 | 4.71132945132371 | 60.4919289417863 | Oman |
| 2005 | 60.96496811183 | 0.666401230917978 | 3.6084635137616 | 57.3565045980684 | Oman |
| 2006 | 60.28361869384 | 0.675353885063336 | 2.97479059714099 | 57.308828096699 | Oman |
| 2007 | 61.20892229024 | 0.685055582772953 | 2.76095104808778 | 58.4479712421522 | Oman |
| 2008 | 63.73023531912 | 0.694952262906783 | 2.38310821492293 | 61.3471271041971 | Oman |
| 2009 | 66.26065648944 | 0.703098570806704 | 2.3260628110177 | 63.9345936784223 | Oman |
| 2010 | 66.02446490589 | 0.710688694921138 | 2.24674252643038 | 63.7777223794596 | Oman |
| 2011 | 66.71692498273 | 0.718264528020208 | 2.20725596329219 | 64.5096690194378 | Oman |
| 2012 | 66.94397655222 | 0.726450074292121 | 2.19790268707351 | 64.7460738651465 | Oman |
| 2013 | 66.45762400597 | 0.733432490235765 | 2.14951739759714 | 64.3081066083729 | Oman |
| 2014 | 65.53409068962 | 0.738743771923443 | 2.14543060635481 | 63.3886600832652 | Oman |
| 2015 | 66.06913607997 | 0.743597182373955 | 2.10429421645212 | 63.9648418635179 | Oman |
| 2016 | 67.367257981 | 0.74879719338257 | 2.05379698626969 | 65.3134609947303 | Oman |
| 2017 | 66.81844131515 | 0.753786057672964 | 2.0241071255293 | 64.7943341896207 | Oman |
| 2018 | 64.46169681819 | 0.759179513636833 | 1.92257792503453 | 62.5391188931555 | Oman |
| 2019 | 61.91454763155 | 0.764527863267196 | 1.72483896209123 | 60.1897086694588 | Oman |
| 2020 | 56.96536633587 | 0.768854215658189 | 1.64760559648557 | 55.3177607393844 | Oman |
| 2021 | 53.12139272092 | 0.773391601871737 | 1.62265961515918 | 51.4987331057608 | Oman |
| 1990 | 52.04714659787 | 0.651208375960545 | 5.89663983318814 | 46.1505067646819 | Qatar |
| 1991 | 49.2523533712 | 0.655338766647052 | 5.11035650260027 | 44.1419968685997 | Qatar |
| 1992 | 46.49357261547 | 0.660673763366912 | 4.437315570109 | 42.056257045361 | Qatar |
| 1993 | 44.04460251267 | 0.666194398536698 | 3.63227542770953 | 40.4123270849605 | Qatar |
| 1994 | 42.08785101238 | 0.672025383885115 | 3.15983548460485 | 38.9280155277751 | Qatar |
| 1995 | 40.19138474193 | 0.678320339337967 | 3.01497302948585 | 37.1764117124441 | Qatar |
| 1996 | 38.79363001285 | 0.68483591897584 | 2.882397373007 | 35.911232639843 | Qatar |
| 1997 | 37.42790690775 | 0.693109653614432 | 2.45644927917167 | 34.9714576285783 | Qatar |
| 1998 | 36.3190930422 | 0.701409063669436 | 2.33364069390449 | 33.9854523482955 | Qatar |
| 1999 | 35.36245467838 | 0.708880981468347 | 2.25400132389118 | 33.1084533544888 | Qatar |
| 2000 | 34.32419205229 | 0.714831609105106 | 2.22583997046786 | 32.0983520818221 | Qatar |
| 2001 | 33.86228521201 | 0.720831064006284 | 2.18296583209438 | 31.6793193799156 | Qatar |
| 2002 | 32.89178757662 | 0.727112174151443 | 2.1903333758853 | 30.7014542007347 | Qatar |
| 2003 | 31.90731258342 | 0.733536599318407 | 2.1624460017473 | 29.7448665816727 | Qatar |
| 2004 | 31.06440719561 | 0.740223208352271 | 2.09215750317616 | 28.9722496924338 | Qatar |
| 2005 | 30.57191983384 | 0.747189358624771 | 2.05982012312552 | 28.5120997107145 | Qatar |
| 2006 | 29.7721655901 | 0.754123028435068 | 2.01506666361071 | 27.7570989264893 | Qatar |
| 2007 | 29.19571322026 | 0.760960134224895 | 1.80155022793286 | 27.3941629923271 | Qatar |
| 2008 | 28.71673800607 | 0.767659233393271 | 1.61880755470875 | 27.0979304513613 | Qatar |
| 2009 | 25.06910619716 | 0.77427684914439 | 1.57718845575914 | 23.4919177414009 | Qatar |
| 2010 | 23.92914434326 | 0.780807073920169 | 1.52876981683971 | 22.4003745264203 | Qatar |
| 2011 | 22.77324674234 | 0.7872124503355 | 1.40727469458138 | 21.3659720477586 | Qatar |
| 2012 | 21.78830957819 | 0.793494900808854 | 1.43930734106362 | 20.3490022371264 | Qatar |
| 2013 | 20.4859031982 | 0.799611554876573 | 1.49185604606967 | 18.9940471521303 | Qatar |
| 2014 | 19.45119514029 | 0.805725710213966 | 1.45657819852839 | 17.9946169417616 | Qatar |
| 2015 | 18.48462989894 | 0.811872851894902 | 1.43959130284698 | 17.045038596093 | Qatar |
| 2016 | 17.72024156326 | 0.817979840558073 | 1.46417004264333 | 16.2560715206167 | Qatar |
| 2017 | 17.17067798923 | 0.824016868434921 | 1.44356906002723 | 15.7271089292028 | Qatar |
| 2018 | 16.59395123321 | 0.829962126581261 | 1.40569713654983 | 15.1882540966602 | Qatar |
| 2019 | 16.06844196305 | 0.835805284348454 | 1.38157738697473 | 14.6868645760753 | Qatar |
| 2020 | 15.35820891405 | 0.841467395353032 | 1.39082062895937 | 13.9673882850906 | Qatar |
| 2021 | 13.74522439352 | 0.846860584038945 | 1.33912693791656 | 12.4060974556034 | Qatar |
| 1990 | 478.37831247074 | 0.538954515496368 | 21.2626328144354 | 457.115679656305 | Saudi Arabia |
| 1991 | 430.36926401602 | 0.549907716626613 | 19.7152824460822 | 410.653981569938 | Saudi Arabia |
| 1992 | 387.10577402428 | 0.561144266304623 | 16.7012707073853 | 370.404503316895 | Saudi Arabia |
| 1993 | 347.56117160773 | 0.572186913451422 | 13.2397220023318 | 334.321449605398 | Saudi Arabia |
| 1994 | 313.47079598949 | 0.582878871179675 | 13.4522829066191 | 300.018513082871 | Saudi Arabia |
| 1995 | 282.66130031543 | 0.593251423065509 | 13.4036029306881 | 269.257697384742 | Saudi Arabia |
| 1996 | 251.58674386014 | 0.603648418303976 | 13.4333698883401 | 238.1533739718 | Saudi Arabia |
| 1997 | 226.77886934014 | 0.613749116453829 | 13.3621629673782 | 213.416706372762 | Saudi Arabia |
| 1998 | 205.83930503277 | 0.623518214245881 | 12.8878049342988 | 192.951500098471 | Saudi Arabia |
| 1999 | 188.54315286264 | 0.632796447356413 | 11.0327448725939 | 177.510407990046 | Saudi Arabia |
| 2000 | 172.69869381743 | 0.642311774343419 | 7.13226018349722 | 165.566433633933 | Saudi Arabia |
| 2001 | 158.88537593001 | 0.651502091755966 | 5.54024058619141 | 153.345135343819 | Saudi Arabia |
| 2002 | 145.13887118285 | 0.660356855701209 | 4.63248537795618 | 140.506385804894 | Saudi Arabia |
| 2003 | 131.8698269404 | 0.669839576958755 | 3.85612420600041 | 128.0137027344 | Saudi Arabia |
| 2004 | 119.04334113516 | 0.679917856369452 | 2.94624576561488 | 116.097095369545 | Saudi Arabia |
| 2005 | 108.10085244185 | 0.690520851691753 | 2.54604818494437 | 105.554804256906 | Saudi Arabia |
| 2006 | 98.22197052774 | 0.70115261257815 | 2.37120189301228 | 95.8507686347277 | Saudi Arabia |
| 2007 | 89.77194216839 | 0.711442141049593 | 2.24284723059062 | 87.5290949377994 | Saudi Arabia |
| 2008 | 81.88702398164 | 0.721960204973632 | 2.18348300063775 | 79.7035409810022 | Saudi Arabia |
| 2009 | 75.13289309463 | 0.731158488162749 | 2.17055228215975 | 72.9623408124703 | Saudi Arabia |
| 2010 | 69.07247986847 | 0.740300669205333 | 2.1239318856326 | 66.9485479828374 | Saudi Arabia |
| 2011 | 63.59132707542 | 0.749959451001828 | 2.05456645142591 | 61.5367606239941 | Saudi Arabia |
| 2012 | 58.32934162625 | 0.759054935383679 | 1.90273887711034 | 56.4266027491397 | Saudi Arabia |
| 2013 | 47.90385579316 | 0.767387864281482 | 1.66367279900845 | 46.2401829941515 | Saudi Arabia |
| 2014 | 44.68191264357 | 0.775178132630626 | 1.53114099348943 | 43.1507716500806 | Saudi Arabia |
| 2015 | 42.04465627741 | 0.782300801582157 | 1.46068074414397 | 40.583975533266 | Saudi Arabia |
| 2016 | 39.98561066833 | 0.788867882259991 | 1.47952778865986 | 38.5060828796701 | Saudi Arabia |
| 2017 | 38.06560665732 | 0.794886287697922 | 1.45650360452258 | 36.6091030527974 | Saudi Arabia |
| 2018 | 36.373725025 | 0.800656984976864 | 1.48995663461371 | 34.8837683903863 | Saudi Arabia |
| 2019 | 35.00834905843 | 0.806087840988648 | 1.46033425803033 | 33.5480148003997 | Saudi Arabia |
| 2020 | 30.66395456506 | 0.810541860714478 | 1.44077799384808 | 29.2231765712119 | Saudi Arabia |
| 2021 | 30.24081844079 | 0.815143493214829 | 1.43713283039221 | 28.8036856103978 | Saudi Arabia |
| 1990 | 285.72176317602 | 0.430492642865419 | 203.592530701542 | 82.1292324744782 | Syrian Arab Republic |
| 1991 | 261.52059069133 | 0.437907493360423 | 111.745912240773 | 149.774678450557 | Syrian Arab Republic |
| 1992 | 236.64165647228 | 0.445904235849208 | 75.299238673053 | 161.342417799227 | Syrian Arab Republic |
| 1993 | 211.2173539995 | 0.45414785985736 | 77.864599141207 | 133.352754858293 | Syrian Arab Republic |
| 1994 | 190.03438398652 | 0.462437297890694 | 65.2395782701535 | 124.794805716366 | Syrian Arab Republic |
| 1995 | 169.53645326158 | 0.470692042726613 | 57.5689383812531 | 111.967514880327 | Syrian Arab Republic |
| 1996 | 145.61436085677 | 0.478743085009033 | 54.8731977690575 | 90.7411630877125 | Syrian Arab Republic |
| 1997 | 124.84825474331 | 0.48623235022627 | 53.1823680795159 | 71.665886663794 | Syrian Arab Republic |
| 1998 | 105.79556634733 | 0.493707991654398 | 49.371869711566 | 56.423696635764 | Syrian Arab Republic |
| 1999 | 96.38973010044 | 0.500474869218632 | 23.2617763588513 | 73.1279537415887 | Syrian Arab Republic |
| 2000 | 82.68105043161 | 0.507143685736932 | 22.6711806067067 | 60.0098698249033 | Syrian Arab Republic |
| 2001 | 73.43263871949 | 0.513850111268667 | 22.4221563453276 | 51.0104823741624 | Syrian Arab Republic |
| 2002 | 67.05153526357 | 0.521309744436802 | 22.1177237451757 | 44.9338115183944 | Syrian Arab Republic |
| 2003 | 58.69525864232 | 0.528935756199872 | 22.1039038418189 | 36.5913548005011 | Syrian Arab Republic |
| 2004 | 54.88658633552 | 0.538084469307822 | 21.3005143270828 | 33.5860720084372 | Syrian Arab Republic |
| 2005 | 55.05769956478 | 0.549750448700287 | 19.8217634450318 | 35.2359361197482 | Syrian Arab Republic |
| 2006 | 55.14033332223 | 0.560484761476108 | 17.6854790212875 | 37.4548543009425 | Syrian Arab Republic |
| 2007 | 50.9928445332 | 0.570028120377629 | 13.3612954211328 | 37.6315491120672 | Syrian Arab Republic |
| 2008 | 51.05282326905 | 0.578990492745018 | 13.3323709802316 | 37.7204522888184 | Syrian Arab Republic |
| 2009 | 48.18639660872 | 0.587789117723187 | 13.2384426360946 | 34.9479539726254 | Syrian Arab Republic |
| 2010 | 45.65276029533 | 0.59596776440437 | 13.3569391334486 | 32.2958211618814 | Syrian Arab Republic |
| 2011 | 44.13589463141 | 0.60152445568341 | 13.6657361390354 | 30.4701584923746 | Syrian Arab Republic |
| 2012 | 40.90579458175 | 0.60287260117891 | 13.4123135068003 | 27.4934810749497 | Syrian Arab Republic |
| 2013 | 39.12808123896 | 0.6024101339716 | 13.3106868632845 | 25.8173943756755 | Syrian Arab Republic |
| 2014 | 39.73621902252 | 0.602235759940116 | 13.5428208008456 | 26.1933982216744 | Syrian Arab Republic |
| 2015 | 39.33070466922 | 0.602282913873841 | 13.5219652891532 | 25.8087393800668 | Syrian Arab Republic |
| 2016 | 37.56220818272 | 0.602911181092802 | 13.3097286907371 | 24.2524794919829 | Syrian Arab Republic |
| 2017 | 34.71127729416 | 0.604896366029073 | 13.4512869709285 | 21.2599903232315 | Syrian Arab Republic |
| 2018 | 31.88847529602 | 0.608507163893069 | 13.2763130049153 | 18.6121622911047 | Syrian Arab Republic |
| 2019 | 29.06514450946 | 0.613336835963498 | 13.3495282033003 | 15.7156163061597 | Syrian Arab Republic |
| 2020 | 25.57636354007 | 0.61772759307296 | 12.7704461415724 | 12.8059173984976 | Syrian Arab Republic |
| 2021 | 23.67602292292 | 0.62300407463119 | 12.9991846007161 | 10.6768383222039 | Syrian Arab Republic |
| 1990 | 127.70504254351 | 0.471138521303638 | 59.9330993758263 | 67.7719431676837 | Tunisia |
| 1991 | 114.56121764603 | 0.479543056615384 | 53.0185543282503 | 61.5426633177797 | Tunisia |
| 1992 | 102.77886640414 | 0.488285281381984 | 48.6625495526611 | 54.1163168514789 | Tunisia |
| 1993 | 92.1297916502 | 0.496852038879262 | 29.8534018465072 | 62.2763898036928 | Tunisia |
| 1994 | 82.22843563103 | 0.505564882262378 | 22.8309497920991 | 59.3974858389309 | Tunisia |
| 1995 | 73.65380046742 | 0.514196940056936 | 22.5422813290553 | 51.1115191383647 | Tunisia |
| 1996 | 65.60583533249 | 0.523294273698708 | 22.0936349257404 | 43.5122004067496 | Tunisia |
| 1997 | 58.94911195151 | 0.532064044257322 | 22.1029684057978 | 36.8461435457122 | Tunisia |
| 1998 | 53.33816530678 | 0.540498160807659 | 21.2406335031251 | 32.0975318036549 | Tunisia |
| 1999 | 49.8156245193 | 0.548724563004994 | 20.5686429514104 | 29.2469815678896 | Tunisia |
| 2000 | 46.93394105162 | 0.556672649979971 | 18.7110379000565 | 28.2229031515635 | Tunisia |
| 2001 | 44.42135827264 | 0.564421010157184 | 16.3675955144068 | 28.0537627582332 | Tunisia |
| 2002 | 42.5921850991 | 0.571719533904953 | 13.3269974856154 | 29.2651876134846 | Tunisia |
| 2003 | 40.65603776022 | 0.578882668513321 | 13.2858421143383 | 27.3701956458817 | Tunisia |
| 2004 | 38.16410350327 | 0.586032594266679 | 13.7001085301561 | 24.4639949731139 | Tunisia |
| 2005 | 36.36969778686 | 0.592937759304517 | 13.3437120974324 | 23.0259856894276 | Tunisia |
| 2006 | 34.85763599294 | 0.59975851290592 | 13.3894390568052 | 21.4681969361348 | Tunisia |
| 2007 | 33.42719234324 | 0.606696784272835 | 13.5540652734747 | 19.8731270697653 | Tunisia |
| 2008 | 32.0030263433 | 0.613529322048559 | 13.2984102813924 | 18.7046160619076 | Tunisia |
| 2009 | 30.76630079294 | 0.620106833480493 | 13.2537193246699 | 17.5125814682701 | Tunisia |
| 2010 | 29.64205085028 | 0.626432773734803 | 12.9904688439877 | 16.6515820062923 | Tunisia |
| 2011 | 27.93862783379 | 0.632019840139461 | 12.006968824953 | 15.931659008837 | Tunisia |
| 2012 | 26.26226512634 | 0.637578344563551 | 7.91699872956188 | 18.3452663967781 | Tunisia |
| 2013 | 24.83553501917 | 0.643008511949972 | 7.51647912881191 | 17.3190558903581 | Tunisia |
| 2014 | 23.48183927805 | 0.648297395047989 | 5.62717683853775 | 17.8546624395123 | Tunisia |
| 2015 | 22.24996169422 | 0.653413537772423 | 5.0598970070272 | 17.1900646871928 | Tunisia |
| 2016 | 21.19407816341 | 0.658353516621193 | 4.61610790243508 | 16.5779702609749 | Tunisia |
| 2017 | 19.44275760402 | 0.663222845186053 | 4.44405882763333 | 14.9986987763867 | Tunisia |
| 2018 | 17.92132713031 | 0.668218997464981 | 3.7333371375577 | 14.1879899927523 | Tunisia |
| 2019 | 16.83801779292 | 0.673170551594905 | 3.28180208011491 | 13.5562157128051 | Tunisia |
| 2020 | 15.55222794649 | 0.677615147910612 | 2.88659750891462 | 12.6656304375754 | Tunisia |
| 2021 | 14.89147037742 | 0.682432215876962 | 2.59476498087578 | 12.2967053965442 | Tunisia |
| 1990 | 601.51728393626 | 0.461606983777208 | 60.5053932015555 | 541.011890734704 | Turkey |
| 1991 | 546.52580841401 | 0.469244066030772 | 56.5452023044227 | 489.980606109587 | Turkey |
| 1992 | 498.4814071774 | 0.476814569059173 | 53.9016168356123 | 444.579790341788 | Turkey |
| 1993 | 456.2394192786 | 0.484656970229106 | 52.559582734527 | 403.679836544073 | Turkey |
| 1994 | 425.20722976343 | 0.491619950496974 | 50.494436449568 | 374.712793313862 | Turkey |
| 1995 | 392.96475832258 | 0.498824840241401 | 24.6766065783166 | 368.288151744263 | Turkey |
| 1996 | 359.66651374646 | 0.506238546050969 | 22.7543478373815 | 336.912165909079 | Turkey |
| 1997 | 331.46713438926 | 0.513933355794628 | 22.4621091661726 | 309.005025223087 | Turkey |
| 1998 | 302.60032394035 | 0.521509897761014 | 22.141568351449 | 280.458755588901 | Turkey |
| 1999 | 271.15254129013 | 0.528512415528019 | 22.1347531683726 | 249.017788121757 | Turkey |
| 2000 | 244.74692320266 | 0.535866331151872 | 21.465234758252 | 223.281688444408 | Turkey |
| 2001 | 226.53051780336 | 0.542359130151394 | 21.1786726186218 | 205.351845184738 | Turkey |
| 2002 | 208.18541139536 | 0.549023644141264 | 20.5143762204518 | 187.671035174908 | Turkey |
| 2003 | 184.2141860638 | 0.556102526706668 | 18.6351071065764 | 165.579078957224 | Turkey |
| 2004 | 165.84166493776 | 0.564023999784751 | 16.0318414650189 | 149.809823472741 | Turkey |
| 2005 | 147.26187008497 | 0.572495886408665 | 13.2311693903976 | 134.030700694572 | Turkey |
| 2006 | 131.93509677984 | 0.581383178782649 | 13.352137010795 | 118.582959769045 | Turkey |
| 2007 | 118.96334335359 | 0.590442387296341 | 13.3004230245438 | 105.662920329046 | Turkey |
| 2008 | 108.35925926117 | 0.599212061486959 | 13.210799992821 | 95.148459268349 | Turkey |
| 2009 | 97.15594816578 | 0.607104617902237 | 13.1759617608266 | 83.9799864049534 | Turkey |
| 2010 | 88.25583252386 | 0.615597227656952 | 12.9544299626655 | 75.3014025611945 | Turkey |
| 2011 | 76.84631209996 | 0.624787210625257 | 12.9109483345979 | 63.9353637653621 | Turkey |
| 2012 | 70.39300721383 | 0.634097091952487 | 10.8929331159017 | 59.5000740979283 | Turkey |
| 2013 | 65.49070830379 | 0.643723822649817 | 8.08340431744132 | 57.4073039863487 | Turkey |
| 2014 | 61.20388568825 | 0.653388834851172 | 5.14847431825525 | 56.0554113699947 | Turkey |
| 2015 | 56.2925725682 | 0.662971738649364 | 4.39757994761789 | 51.8949926205821 | Turkey |
| 2016 | 51.91710461066 | 0.671948147539094 | 3.44898892580416 | 48.4681156848558 | Turkey |
| 2017 | 47.71291392311 | 0.681096272126073 | 2.74960859915194 | 44.9633053239581 | Turkey |
| 2018 | 44.58406971778 | 0.689895866860746 | 2.44294816259207 | 42.1411215551879 | Turkey |
| 2019 | 42.29799087587 | 0.69826377357682 | 2.3473039489093 | 39.9506869269607 | Turkey |
| 2020 | 38.70639128737 | 0.70579974360086 | 2.27908486107145 | 36.4273064262985 | Turkey |
| 2021 | 37.56765241467 | 0.712692673098478 | 2.24903923979274 | 35.3186131748773 | Turkey |
| 1990 | 87.89478664747 | 0.644412271353864 | 6.4499855025738 | 81.4448011448962 | United Arab Emirates |
| 1991 | 81.62307527119 | 0.660739899044445 | 4.7630412626372 | 76.8600340085528 | United Arab Emirates |
| 1992 | 77.82613825405 | 0.675926211137966 | 3.23186038867406 | 74.5942778653759 | United Arab Emirates |
| 1993 | 67.98413899893 | 0.689463800557258 | 2.43069604953695 | 65.5534429493931 | United Arab Emirates |
| 1994 | 61.62963632342 | 0.701656792310882 | 2.30310533667951 | 59.3265309867405 | United Arab Emirates |
| 1995 | 54.76873763483 | 0.713138287743939 | 2.23105101214705 | 52.537686622683 | United Arab Emirates |
| 1996 | 48.82908562897 | 0.724181976276083 | 2.20683520982822 | 46.6222504191418 | United Arab Emirates |
| 1997 | 47.451376887 | 0.734723175496013 | 2.17330413002821 | 45.2780727569718 | United Arab Emirates |
| 1998 | 48.65140591753 | 0.744476623052101 | 2.05029884589545 | 46.6011070716345 | United Arab Emirates |
| 1999 | 52.04186291845 | 0.753253916450152 | 2.00619828400353 | 50.0356646344465 | United Arab Emirates |
| 2000 | 52.81828334123 | 0.760969094123031 | 1.86209879644859 | 50.9561845447814 | United Arab Emirates |
| 2001 | 53.80742716895 | 0.767858792936623 | 1.61448675847694 | 52.1929404104731 | United Arab Emirates |
| 2002 | 54.92978412219 | 0.774346424129537 | 1.5945617655965 | 53.3352223565935 | United Arab Emirates |
| 2003 | 54.56056059826 | 0.781143991614649 | 1.48923030881343 | 53.0713302894466 | United Arab Emirates |
| 2004 | 51.20282486885 | 0.788525951082133 | 1.45334113285125 | 49.7494837359988 | United Arab Emirates |
| 2005 | 49.48435106652 | 0.796428085714925 | 1.460478767475 | 48.023872299045 | United Arab Emirates |
| 2006 | 46.94272463559 | 0.805537022535992 | 1.4281737867623 | 45.5145508488277 | United Arab Emirates |
| 2007 | 49.76441482313 | 0.814870878080389 | 1.45369876169536 | 48.3107160614346 | United Arab Emirates |
| 2008 | 53.04196716893 | 0.822793435350208 | 1.44913584302731 | 51.5928313259027 | United Arab Emirates |
| 2009 | 57.45601994102 | 0.82830416539166 | 1.45389141742435 | 56.0021285235957 | United Arab Emirates |
| 2010 | 59.59238174177 | 0.831284254488885 | 1.42200522820611 | 58.1703765135639 | United Arab Emirates |
| 2011 | 58.48911816733 | 0.832912803937778 | 1.43166757129439 | 57.0574505960356 | United Arab Emirates |
| 2012 | 56.86614770086 | 0.833586109601576 | 1.41575397635675 | 55.4503937245032 | United Arab Emirates |
| 2013 | 55.15402461937 | 0.833803818475535 | 1.42954560515672 | 53.7244790142133 | United Arab Emirates |
| 2014 | 46.50077059751 | 0.833858726882329 | 1.41264569243964 | 45.0881249050704 | United Arab Emirates |
| 2015 | 43.59631056507 | 0.834175498027292 | 1.416297690082 | 42.180012874988 | United Arab Emirates |
| 2016 | 41.06218805201 | 0.835220709910024 | 1.40701197193111 | 39.6551760800789 | United Arab Emirates |
| 2017 | 38.94038087951 | 0.837213552633191 | 1.41018208809973 | 37.5301987914103 | United Arab Emirates |
| 2018 | 34.28769526543 | 0.839900140178067 | 1.39769743783194 | 32.8899978275981 | United Arab Emirates |
| 2019 | 30.24227644646 | 0.842985326255825 | 1.35814257930444 | 28.8841338671556 | United Arab Emirates |
| 2020 | 27.23826607104 | 0.846151975991622 | 1.35511849033333 | 25.8831475807067 | United Arab Emirates |
| 2021 | 23.82931140298 | 0.849317734035032 | 1.33162214863333 | 22.4976892543467 | United Arab Emirates |
| 1990 | 3960.57129766427 | 0.215664585570251 | 2305.06609636282 | 1655.50520130145 | Yemen |
| 1991 | 3723.75221499983 | 0.222885408676441 | 2140.32633868754 | 1583.42587631229 | Yemen |
| 1992 | 3531.63796311369 | 0.230415045958638 | 1935.85481932434 | 1595.78314378935 | Yemen |
| 1993 | 3322.60972042659 | 0.238025246263208 | 1715.51400327133 | 1607.09571715526 | Yemen |
| 1994 | 3108.93328483892 | 0.245879426395306 | 1690.32031212652 | 1418.6129727124 | Yemen |
| 1995 | 2866.02255128754 | 0.253962571622302 | 1529.49563496673 | 1336.52691632081 | Yemen |
| 1996 | 2640.18299625952 | 0.262278612845771 | 1401.0829150425 | 1239.10008121702 | Yemen |
| 1997 | 2433.34107602589 | 0.270782535711374 | 1229.45518322017 | 1203.88589280572 | Yemen |
| 1998 | 2217.49909746431 | 0.279500507186773 | 1202.64590871223 | 1014.85318875209 | Yemen |
| 1999 | 2014.68575129729 | 0.288261301367107 | 1133.27899488969 | 881.406756407604 | Yemen |
| 2000 | 1825.20895631157 | 0.297145157465494 | 1070.06420315833 | 755.144753153235 | Yemen |
| 2001 | 1638.98804360055 | 0.306112423990989 | 1014.19909273781 | 624.788950862738 | Yemen |
| 2002 | 1530.8723715165 | 0.31506502273303 | 817.944058024322 | 712.928313492178 | Yemen |
| 2003 | 1382.26645551637 | 0.324081688959523 | 755.069893914824 | 627.196561601546 | Yemen |
| 2004 | 1223.51703217304 | 0.333371285035603 | 684.9547049659 | 538.56232720714 | Yemen |
| 2005 | 1066.37423071212 | 0.343462561815387 | 592.875583323737 | 473.498647388383 | Yemen |
| 2006 | 929.2174409854 | 0.353472947292631 | 594.446232698263 | 334.771208287137 | Yemen |
| 2007 | 882.90606931657 | 0.363301217705729 | 573.884274846164 | 309.021794470406 | Yemen |
| 2008 | 796.65711818698 | 0.373140288261273 | 544.311686043772 | 252.345432143208 | Yemen |
| 2009 | 708.14275777205 | 0.382762252118378 | 515.399104019823 | 192.743653752227 | Yemen |
| 2010 | 622.25592659972 | 0.392936641182874 | 468.518746449018 | 153.737180150702 | Yemen |
| 2011 | 573.66560488622 | 0.40147180053512 | 460.747276864955 | 112.918328021265 | Yemen |
| 2012 | 466.01711640251 | 0.409561348808444 | 315.513175397184 | 150.503941005326 | Yemen |
| 2013 | 426.45396154126 | 0.417642404804605 | 262.944992404348 | 163.508969136912 | Yemen |
| 2014 | 378.16302324325 | 0.425189667293499 | 232.43296507318 | 145.73005817007 | Yemen |
| 2015 | 351.56286994704 | 0.430197789315438 | 207.022855716211 | 144.540014230829 | Yemen |
| 2016 | 381.29045593584 | 0.434232982818394 | 203.806223844094 | 177.484232091746 | Yemen |
| 2017 | 521.53523011435 | 0.437549155246091 | 116.88788875069 | 404.64734136366 | Yemen |
| 2018 | 404.47730502858 | 0.440727991364019 | 120.753060381133 | 283.724244647447 | Yemen |
| 2019 | 366.23474656909 | 0.444001399873635 | 104.872257992464 | 261.362488576626 | Yemen |
| 2020 | 241.42980057595 | 0.44689306923482 | 84.230619362549 | 157.199181213401 | Yemen |
| 2021 | 219.95048276671 | 0.450376375244811 | 74.3819431107795 | 145.56853965593 | Yemen |
| 1990 | 3346.7747029581 | 0.173832165473389 | 2342.55794588555 | 1004.21675707255 | Afghanistan |
| 1991 | 3471.76361944538 | 0.176472549509622 | 2336.09430049905 | 1135.66931894633 | Afghanistan |
| 1992 | 3568.27844035992 | 0.179633724437178 | 2342.13874351797 | 1226.13969684195 | Afghanistan |
| 1993 | 3734.20709989321 | 0.18018371261059 | 2343.38127080258 | 1390.82582909063 | Afghanistan |
| 1994 | 3365.29658770678 | 0.178518660381455 | 2351.65188914963 | 1013.64469855715 | Afghanistan |
| 1995 | 3483.72523035126 | 0.17827912756413 | 2334.02543615708 | 1149.69979419418 | Afghanistan |
| 1996 | 3381.22876664764 | 0.178085403812013 | 2326.07420247379 | 1055.15456417385 | Afghanistan |
| 1997 | 3443.35836962164 | 0.177884124107706 | 2332.17333141421 | 1111.18503820743 | Afghanistan |
| 1998 | 3406.16080425536 | 0.177513403151835 | 2354.71956273345 | 1051.44124152191 | Afghanistan |
| 1999 | 3284.24950462658 | 0.177075239430658 | 2340.46340602864 | 943.786098597937 | Afghanistan |
| 2000 | 3166.17379411943 | 0.177025771968387 | 2325.72299250107 | 840.450801618356 | Afghanistan |
| 2001 | 3121.79140364672 | 0.177773144391168 | 2344.24206508174 | 777.549338564978 | Afghanistan |
| 2002 | 2942.80330629914 | 0.183808429860528 | 2355.52375049562 | 587.279555803521 | Afghanistan |
| 2003 | 2966.39201679406 | 0.19051899147664 | 2339.09756928743 | 627.294447506627 | Afghanistan |
| 2004 | 2832.12691468581 | 0.196903541380771 | 2345.72952324745 | 486.397391438365 | Afghanistan |
| 2005 | 2611.95670311171 | 0.203928133334003 | 2336.86971992789 | 275.086983183821 | Afghanistan |
| 2006 | 2320.59430855296 | 0.211074194179476 | 2271.58117606722 | 49.0131324857362 | Afghanistan |
| 2007 | 2076.4201183316 | 0.219627610708056 | 2076.4201183316 | 0 | Afghanistan |
| 2008 | 1816.86419941811 | 0.22808917063877 | 1816.86419941811 | 0 | Afghanistan |
| 2009 | 1584.67836549325 | 0.237900824236794 | 1584.67836549325 | 0 | Afghanistan |
| 2010 | 1436.73958172013 | 0.247759948877388 | 1436.73958172013 | 0 | Afghanistan |
| 2011 | 1307.28985429793 | 0.257041675822107 | 1307.28985429793 | 0 | Afghanistan |
| 2012 | 1189.90709722147 | 0.266484406571128 | 1189.90709722147 | 0 | Afghanistan |
| 2013 | 1170.15161812874 | 0.275636558072642 | 1170.15161812874 | 0 | Afghanistan |
| 2014 | 1096.77980195222 | 0.284030351035349 | 1096.77980195222 | 0 | Afghanistan |
| 2015 | 1030.48801112989 | 0.291849504924728 | 1030.48801112989 | 0 | Afghanistan |
| 2016 | 983.17365943905 | 0.299630695812728 | 983.17365943905 | 0 | Afghanistan |
| 2017 | 916.58523111516 | 0.307424618143983 | 916.58523111516 | 0 | Afghanistan |
| 2018 | 767.75645100258 | 0.314866093354221 | 767.75645100258 | 0 | Afghanistan |
| 2019 | 713.26620893179 | 0.322454046974177 | 713.26620893179 | 0 | Afghanistan |
| 2020 | 636.90261963324 | 0.32983006796412 | 636.90261963324 | 0 | Afghanistan |
| 2021 | 561.21919581233 | 0.337199997837472 | 561.21919581233 | 0 | Afghanistan |
| 1990 | 3263.6422568293 | 0.228548934038183 | 1914.06428869175 | 1349.57796813755 | Bangladesh |
| 1991 | 3499.0008608778 | 0.237207412585347 | 1918.61808962518 | 1580.38277125262 | Bangladesh |
| 1992 | 3311.35271394 | 0.245247955858354 | 1714.589998578 | 1596.762715362 | Bangladesh |
| 1993 | 3165.24231035171 | 0.252090719955727 | 1544.02527365774 | 1621.21703669397 | Bangladesh |
| 1994 | 2967.80493253927 | 0.258997294481156 | 1394.85053769024 | 1572.95439484903 | Bangladesh |
| 1995 | 2710.10489750011 | 0.265293918356151 | 1377.29207279787 | 1332.81282470224 | Bangladesh |
| 1996 | 2383.99893151337 | 0.271177780650834 | 1247.73809781324 | 1136.26083370013 | Bangladesh |
| 1997 | 2065.66612219716 | 0.277773280387452 | 1195.02936331342 | 870.636758883735 | Bangladesh |
| 1998 | 1753.5395484487 | 0.284515940436142 | 1131.56727564227 | 621.972272806434 | Bangladesh |
| 1999 | 1497.91097816346 | 0.290951431147001 | 1131.39999858947 | 366.510979573985 | Bangladesh |
| 2000 | 1312.60464051086 | 0.297251975652563 | 1086.44697578642 | 226.157664724443 | Bangladesh |
| 2001 | 1172.47586978986 | 0.303188546281531 | 1039.94256401079 | 132.533305779071 | Bangladesh |
| 2002 | 1053.566877062 | 0.308640093479048 | 958.523443646311 | 95.0434334156887 | Bangladesh |
| 2003 | 948.82421058133 | 0.3143600400365 | 931.630088199373 | 17.1941223819573 | Bangladesh |
| 2004 | 872.86220687988 | 0.320922851356659 | 812.312151863609 | 60.5500550162707 | Bangladesh |
| 2005 | 795.8551520354 | 0.328090510389871 | 743.427897570145 | 52.4272544652549 | Bangladesh |
| 2006 | 732.45336104564 | 0.3356905979604 | 674.302877609506 | 58.1504834361342 | Bangladesh |
| 2007 | 697.91355221098 | 0.343932545818298 | 594.200152339943 | 103.713399871037 | Bangladesh |
| 2008 | 629.64448966271 | 0.352520032963442 | 582.990880219269 | 46.6536094434409 | Bangladesh |
| 2009 | 569.17199386665 | 0.361481512825909 | 564.455799671646 | 4.71619419500371 | Bangladesh |
| 2010 | 517.75975155069 | 0.370598956236852 | 517.75975155069 | 0 | Bangladesh |
| 2011 | 481.41055011164 | 0.380124885726479 | 481.41055011164 | 0 | Bangladesh |
| 2012 | 431.74401934198 | 0.39062355195618 | 431.74401934198 | 0 | Bangladesh |
| 2013 | 401.36736557116 | 0.401706477929242 | 401.36736557116 | 0 | Bangladesh |
| 2014 | 375.22650350229 | 0.413263168891725 | 271.695456793178 | 103.531046709112 | Bangladesh |
| 2015 | 373.28145845959 | 0.42548212139419 | 234.303805428414 | 138.977653031176 | Bangladesh |
| 2016 | 367.9500412236 | 0.437709906182211 | 119.012432765247 | 248.937608458353 | Bangladesh |
| 2017 | 357.71818233683 | 0.449752413112756 | 68.5539814742154 | 289.164200862615 | Bangladesh |
| 2018 | 334.79128626157 | 0.461717748857041 | 66.1249717714524 | 268.666314490118 | Bangladesh |
| 2019 | 315.4443242019 | 0.473264997623618 | 57.7426204322832 | 257.701703769617 | Bangladesh |
| 2020 | 255.51975008625 | 0.483079169271683 | 52.3194610447616 | 203.200289041488 | Bangladesh |
| 2021 | 241.32502589743 | 0.492420884955353 | 49.4997767547165 | 191.825249142713 | Bangladesh |
| 1990 | 9721.02955120026 | 0.215039850206523 | 2296.18713733266 | 7424.8424138676 | Bhutan |
| 1991 | 9218.24218135706 | 0.221312439958873 | 2164.3756204836 | 7053.86656087346 | Bhutan |
| 1992 | 8729.70431881859 | 0.228148271920468 | 1919.01248962124 | 6810.69182919735 | Bhutan |
| 1993 | 8651.16521072542 | 0.236004687298356 | 1931.49874027743 | 6719.66647044799 | Bhutan |
| 1994 | 8008.73574455066 | 0.244331246046266 | 1731.8347650425 | 6276.90097950816 | Bhutan |
| 1995 | 7499.40510182915 | 0.253291956861352 | 1506.36473602795 | 5993.0403658012 | Bhutan |
| 1996 | 6775.09369773208 | 0.262362745638857 | 1392.6243436163 | 5382.46935411578 | Bhutan |
| 1997 | 6449.54942994152 | 0.271487422119903 | 1252.20343912987 | 5197.34599081165 | Bhutan |
| 1998 | 5470.21144737825 | 0.280440384324268 | 1197.93918830501 | 4272.27225907324 | Bhutan |
| 1999 | 4183.46531997239 | 0.28951048779873 | 1135.15462754075 | 3048.31069243164 | Bhutan |
| 2000 | 4497.01284667667 | 0.298804874805939 | 1088.72158361502 | 3408.29126306165 | Bhutan |
| 2001 | 3989.61366779498 | 0.308200652419302 | 971.90030523545 | 3017.71336255953 | Bhutan |
| 2002 | 3620.24725944728 | 0.3179435358725 | 845.000217776118 | 2775.24704167116 | Bhutan |
| 2003 | 3397.8498335001 | 0.327670422082998 | 764.957527749703 | 2632.8923057504 | Bhutan |
| 2004 | 3114.9721206162 | 0.337286494753829 | 597.881378162753 | 2517.09074245345 | Bhutan |
| 2005 | 2814.85029012708 | 0.346998577564248 | 597.013347738758 | 2217.83694238832 | Bhutan |
| 2006 | 2504.44620367904 | 0.356824209168712 | 591.6440496757 | 1912.80215400334 | Bhutan |
| 2007 | 2231.10299997563 | 0.367560497563767 | 573.777121017406 | 1657.32587895822 | Bhutan |
| 2008 | 1967.83591173722 | 0.378233542838379 | 546.162680296588 | 1421.67323144063 | Bhutan |
| 2009 | 1694.85219697685 | 0.388977161920706 | 502.384487613431 | 1192.46770936342 | Bhutan |
| 2010 | 1497.74344315504 | 0.399751936485664 | 466.03728820284 | 1031.7061549522 | Bhutan |
| 2011 | 1362.7975311006 | 0.410149248617919 | 303.001353363458 | 1059.79617773714 | Bhutan |
| 2012 | 1244.33056404687 | 0.419443216044258 | 261.75328635455 | 982.57727769232 | Bhutan |
| 2013 | 1139.2720210013 | 0.427790674624113 | 215.887908080438 | 923.384112920862 | Bhutan |
| 2014 | 1054.74574024855 | 0.434969269672115 | 193.93371769375 | 860.8120225548 | Bhutan |
| 2015 | 982.09231855017 | 0.44181074828187 | 116.741247130904 | 865.351071419266 | Bhutan |
| 2016 | 908.86046468103 | 0.448401854533875 | 72.4630490589831 | 836.397415622047 | Bhutan |
| 2017 | 836.39186317705 | 0.454280496545609 | 77.3638505968227 | 759.028012580227 | Bhutan |
| 2018 | 773.694685608 | 0.459624710991057 | 64.665232338739 | 709.029453269261 | Bhutan |
| 2019 | 718.655093787 | 0.464633353490442 | 62.7061778273345 | 655.948915959666 | Bhutan |
| 2020 | 672.6340866467 | 0.468714478980695 | 57.1216831839103 | 615.51240346279 | Bhutan |
| 2021 | 625.94314395756 | 0.473062378179926 | 57.4718931570861 | 568.471250800474 | Bhutan |
| 1990 | 5649.0471497475 | 0.332593603012361 | 672.012282486151 | 4977.03486726135 | India |
| 1991 | 5532.34904535017 | 0.338591779142716 | 599.132114005248 | 4933.21693134492 | India |
| 1992 | 5463.46417371189 | 0.344591727089114 | 589.119485433368 | 4874.34468827852 | India |
| 1993 | 5259.5894143454 | 0.350685396969938 | 590.020868478865 | 4669.56854586653 | India |
| 1994 | 5033.1919381828 | 0.356996030489868 | 587.303714886983 | 4445.88822329582 | India |
| 1995 | 4859.36127074662 | 0.363418500556262 | 576.625187881057 | 4282.73608286556 | India |
| 1996 | 4627.8334264504 | 0.370018643898342 | 580.123833260315 | 4047.70959319009 | India |
| 1997 | 4385.43947895435 | 0.37635876286452 | 543.204775945577 | 3842.23470300877 | India |
| 1998 | 4084.45775210149 | 0.382817848453708 | 511.308303144281 | 3573.14944895721 | India |
| 1999 | 3732.11434249097 | 0.389453889040872 | 506.907383958527 | 3225.20695853244 | India |
| 2000 | 3451.29154309746 | 0.395752331114503 | 467.774686227956 | 2983.5168568695 | India |
| 2001 | 3223.86684113132 | 0.401837472284192 | 393.67328614275 | 2830.19355498857 | India |
| 2002 | 3087.25283769567 | 0.407411413581757 | 371.629524972781 | 2715.62331272289 | India |
| 2003 | 2937.29702013825 | 0.413115782576536 | 294.075358509928 | 2643.22166162832 | India |
| 2004 | 2775.52699942926 | 0.419184960649408 | 263.699157112811 | 2511.82784231645 | India |
| 2005 | 2633.28682103176 | 0.425913061870066 | 232.187598875284 | 2401.09922215648 | India |
| 2006 | 2478.43071240106 | 0.433321558442149 | 201.537910382225 | 2276.89280201884 | India |
| 2007 | 2321.45952523967 | 0.441351460389445 | 111.948612274824 | 2209.51091296485 | India |
| 2008 | 2168.76362882499 | 0.44923585077668 | 79.669782688794 | 2089.0938461362 | India |
| 2009 | 2009.50016192611 | 0.457672331967412 | 61.3743152139694 | 1948.12584671214 | India |
| 2010 | 1856.3147931271 | 0.467018995596075 | 60.0305478851796 | 1796.28424524192 | India |
| 2011 | 1709.73676710156 | 0.476832909015635 | 52.9067715306756 | 1656.82999557088 | India |
| 2012 | 1576.07276442499 | 0.487210875591002 | 50.223182849816 | 1525.84958157517 | India |
| 2013 | 1470.7925057698 | 0.498317981286522 | 23.9804258923251 | 1446.81207987747 | India |
| 2014 | 1319.83595423664 | 0.509722099778239 | 22.761126849273 | 1297.07482738737 | India |
| 2015 | 1185.47437708728 | 0.521242718548368 | 22.111588708662 | 1163.36278837862 | India |
| 2016 | 1042.75451528918 | 0.532220023184224 | 22.1242545707531 | 1020.63026071843 | India |
| 2017 | 926.38792556402 | 0.54238850718096 | 21.1610206818927 | 905.226904882127 | India |
| 2018 | 853.21717565624 | 0.551930326455295 | 19.8241667902633 | 833.393008865977 | India |
| 2019 | 735.02590720337 | 0.560809926886154 | 16.5270893691891 | 718.498817834181 | India |
| 2020 | 610.40648500322 | 0.568138149330912 | 14.6563038810835 | 595.750181122136 | India |
| 2021 | 550.05345709765 | 0.575401649396992 | 13.3156071471759 | 536.737849950474 | India |
| 1990 | 8162.91244765031 | 0.199560654182432 | 2349.03831289112 | 5813.87413475919 | Nepal |
| 1991 | 8016.75929207891 | 0.205687442933642 | 2350.7054109867 | 5666.05388109221 | Nepal |
| 1992 | 7137.84665610501 | 0.212113089793247 | 2290.18309414761 | 4847.6635619574 | Nepal |
| 1993 | 6689.38510571795 | 0.218843431287675 | 2289.49681498358 | 4399.88829073437 | Nepal |
| 1994 | 6257.34989824379 | 0.226073059669684 | 2150.62400553677 | 4106.72589270702 | Nepal |
| 1995 | 5838.88711901536 | 0.233253154418138 | 1945.70482972999 | 3893.18228928537 | Nepal |
| 1996 | 5430.04611959209 | 0.240700615739874 | 1723.25408765036 | 3706.79203194173 | Nepal |
| 1997 | 4988.6959958089 | 0.248382796560591 | 1525.28970776626 | 3463.40628804264 | Nepal |
| 1998 | 4586.870366333 | 0.256227178679174 | 1505.09935326168 | 3081.77101307132 | Nepal |
| 1999 | 4162.76901718722 | 0.264359180313156 | 1380.48289701067 | 2782.28612017655 | Nepal |
| 2000 | 3779.90231076887 | 0.272719114894473 | 1247.77315240899 | 2532.12915835988 | Nepal |
| 2001 | 3357.07888701777 | 0.281287467400935 | 1190.75361360925 | 2166.32527340852 | Nepal |
| 2002 | 2838.96994647721 | 0.289513607929153 | 1141.10536227071 | 1697.8645842065 | Nepal |
| 2003 | 2381.51569717303 | 0.297772595071107 | 1078.51201855687 | 1303.00367861616 | Nepal |
| 2004 | 2019.96778613809 | 0.306196530322722 | 1026.17667139448 | 993.791114743615 | Nepal |
| 2005 | 1916.36372505901 | 0.314661796325301 | 940.412910257682 | 975.950814801328 | Nepal |
| 2006 | 1772.50821135689 | 0.323056345509418 | 779.287007316874 | 993.221204040016 | Nepal |
| 2007 | 1592.51546453658 | 0.331445610061963 | 676.987833192069 | 915.527631344511 | Nepal |
| 2008 | 1424.60062088441 | 0.340150705786803 | 596.787478817052 | 827.813142067358 | Nepal |
| 2009 | 1397.33136418279 | 0.348701893463869 | 602.232327842773 | 795.099036340017 | Nepal |
| 2010 | 1148.45805010192 | 0.356868100367112 | 584.594662558757 | 563.863387543163 | Nepal |
| 2011 | 965.14031225942 | 0.364642604726269 | 580.48783243581 | 384.65247982361 | Nepal |
| 2012 | 840.41032347169 | 0.372437587888826 | 548.834298365598 | 291.576025106092 | Nepal |
| 2013 | 783.14227118959 | 0.380022326031393 | 545.763258901313 | 237.379012288277 | Nepal |
| 2014 | 686.21592465664 | 0.387689088395059 | 518.649645995621 | 167.566278661019 | Nepal |
| 2015 | 627.10108380434 | 0.395021154474843 | 464.906616323711 | 162.194467480629 | Nepal |
| 2016 | 565.13878614586 | 0.401628776477432 | 467.96964632846 | 97.1691398173996 | Nepal |
| 2017 | 521.63160490448 | 0.408463874114472 | 305.372862235955 | 216.258742668525 | Nepal |
| 2018 | 465.53634371114 | 0.41523668446613 | 269.575806787754 | 195.960536923386 | Nepal |
| 2019 | 436.26333172656 | 0.42200725174098 | 247.538644465661 | 188.724687260899 | Nepal |
| 2020 | 419.82575260181 | 0.427417089099115 | 213.688243905914 | 206.137508695896 | Nepal |
| 2021 | 380.39643375336 | 0.43317463481343 | 206.432412898428 | 173.964020854932 | Nepal |
| 1990 | 7472.10782835859 | 0.310467621430628 | 967.945340332439 | 6504.16248802615 | Pakistan |
| 1991 | 7090.49047931992 | 0.316265666853964 | 848.335778972761 | 6242.15470034716 | Pakistan |
| 1992 | 6790.89370291142 | 0.322248050692924 | 831.390841761337 | 5959.50286115008 | Pakistan |
| 1993 | 6511.15448674076 | 0.328219252463403 | 738.396494310263 | 5772.7579924305 | Pakistan |
| 1994 | 6184.81019173906 | 0.334433128288919 | 670.485341372935 | 5514.32485036613 | Pakistan |
| 1995 | 5863.6307091935 | 0.340926296299194 | 595.656315284755 | 5267.97439390874 | Pakistan |
| 1996 | 5458.16432471697 | 0.347656202917864 | 591.22031289682 | 4866.94401182015 | Pakistan |
| 1997 | 5126.02965031221 | 0.354218627290782 | 594.687623352979 | 4531.34202695923 | Pakistan |
| 1998 | 4796.33225097479 | 0.360875335601641 | 587.537732193412 | 4208.79451878138 | Pakistan |
| 1999 | 4557.93526961302 | 0.367641396836045 | 572.566818661789 | 3985.36845095123 | Pakistan |
| 2000 | 4327.91105060002 | 0.374319132467609 | 543.731534380301 | 3784.17951621972 | Pakistan |
| 2001 | 4103.65552571259 | 0.38059392725045 | 511.103352368385 | 3592.55217334421 | Pakistan |
| 2002 | 3824.19709510331 | 0.386629992356461 | 515.049177922123 | 3309.14791718119 | Pakistan |
| 2003 | 3552.97095857848 | 0.392719262841918 | 469.237946595919 | 3083.73301198256 | Pakistan |
| 2004 | 3335.20994819591 | 0.399084167395961 | 456.637666523423 | 2878.57228167249 | Pakistan |
| 2005 | 3171.56119176938 | 0.40566743657674 | 394.295963306006 | 2777.26522846337 | Pakistan |
| 2006 | 3018.32695235132 | 0.412234830478788 | 298.31614729581 | 2720.01080505551 | Pakistan |
| 2007 | 2877.09499292633 | 0.418519755739044 | 257.772817090351 | 2619.32217583598 | Pakistan |
| 2008 | 2881.39436654804 | 0.424348199605747 | 236.341652801183 | 2645.05271374686 | Pakistan |
| 2009 | 2771.16339668277 | 0.430019832570808 | 202.668234810684 | 2568.49516187209 | Pakistan |
| 2010 | 2577.16643932536 | 0.4356406573129 | 184.526896232466 | 2392.63954309289 | Pakistan |
| 2011 | 2389.02774775289 | 0.441455585075092 | 105.661193074167 | 2283.36655467872 | Pakistan |
| 2012 | 2192.37568397363 | 0.447096599297877 | 79.0374022734934 | 2113.33828170014 | Pakistan |
| 2013 | 2091.90396899748 | 0.452823095279952 | 77.0772659005648 | 2014.82670309692 | Pakistan |
| 2014 | 1963.70689451022 | 0.458781424149631 | 59.9282138025602 | 1903.77868070766 | Pakistan |
| 2015 | 1858.0235818063 | 0.464792667642097 | 61.6157996215756 | 1796.40778218472 | Pakistan |
| 2016 | 1605.27546422591 | 0.471022167867849 | 56.8815992114051 | 1548.3938650145 | Pakistan |
| 2017 | 1367.20770878794 | 0.4775441366317 | 53.591137996305 | 1313.61657079164 | Pakistan |
| 2018 | 1131.0711480645 | 0.484326128033838 | 54.810802106718 | 1076.26034595778 | Pakistan |
| 2019 | 985.34322382237 | 0.491022981031026 | 49.1499609307102 | 936.19326289166 | Pakistan |
| 2020 | 1006.18056565376 | 0.497364405611904 | 27.3389136218798 | 978.84165203188 | Pakistan |
| 2021 | 905.62911845804 | 0.504028688613448 | 22.9838490162692 | 882.645269441771 | Pakistan |
| 1990 | 10083.1236063036 | 0.270736935221319 | 1233.81872420177 | 8849.30488210183 | Angola |
| 1991 | 9643.39814070878 | 0.275160624620397 | 1244.96822791192 | 8398.42991279686 | Angola |
| 1992 | 9087.58086107764 | 0.279231499654396 | 1205.69444329697 | 7881.88641778067 | Angola |
| 1993 | 8740.22355294436 | 0.281867702816568 | 1199.58126127552 | 7540.64229166884 | Angola |
| 1994 | 8482.73212356246 | 0.284487594233541 | 1135.96971752315 | 7346.76240603931 | Angola |
| 1995 | 8182.08859113643 | 0.287447813395598 | 1134.90478139667 | 7047.18380973976 | Angola |
| 1996 | 7716.15470139538 | 0.29142127918547 | 1129.80729099094 | 6586.34741040444 | Angola |
| 1997 | 7401.30721001444 | 0.295433993200957 | 1075.24899493679 | 6326.05821507765 | Angola |
| 1998 | 7081.77719436853 | 0.299553353195731 | 1087.96622290427 | 5993.81097146426 | Angola |
| 1999 | 6738.86236278176 | 0.303720041599183 | 1020.84275761662 | 5718.01960516514 | Angola |
| 2000 | 6427.65534989088 | 0.307603311847111 | 961.065075160437 | 5466.59027473044 | Angola |
| 2001 | 6050.03677852529 | 0.311697534329848 | 965.164529953127 | 5084.87224857216 | Angola |
| 2002 | 5620.89750258223 | 0.316706570519731 | 842.612261104413 | 4778.28524147782 | Angola |
| 2003 | 5164.67096782574 | 0.321777361754333 | 802.729471944563 | 4361.94149588118 | Angola |
| 2004 | 4917.67605592693 | 0.32739526565521 | 752.308412361618 | 4165.36764356531 | Angola |
| 2005 | 4432.10993017421 | 0.333785188103559 | 673.500984566758 | 3758.60894560745 | Angola |
| 2006 | 4474.14019770573 | 0.34101262184868 | 601.802112041339 | 3872.33808566439 | Angola |
| 2007 | 3776.91109309049 | 0.34884684076221 | 595.225465639302 | 3181.68562745119 | Angola |
| 2008 | 3309.61801515368 | 0.357082005452131 | 587.959494328256 | 2721.65852082542 | Angola |
| 2009 | 2915.4013929321 | 0.364407743836554 | 571.162823991288 | 2344.23856894081 | Angola |
| 2010 | 2621.01259681893 | 0.372033537037443 | 551.650485164543 | 2069.36211165439 | Angola |
| 2011 | 2326.92233020216 | 0.380006613064486 | 544.994557346417 | 1781.92777285574 | Angola |
| 2012 | 2005.84674886846 | 0.387891857376226 | 512.423405027521 | 1493.42334384094 | Angola |
| 2013 | 1704.13458478842 | 0.395796328674416 | 468.494245815976 | 1235.64033897244 | Angola |
| 2014 | 1374.36762943925 | 0.403720085675527 | 392.035321413362 | 982.332308025888 | Angola |
| 2015 | 1202.34944756681 | 0.411372026050491 | 288.245908419722 | 914.103539147089 | Angola |
| 2016 | 1046.13119071593 | 0.418822062446251 | 261.17828282912 | 784.95290788681 | Angola |
| 2017 | 917.04167002113 | 0.426177082467176 | 235.591939220082 | 681.449730801048 | Angola |
| 2018 | 795.20028881758 | 0.433355005074473 | 206.495815850115 | 588.704472967465 | Angola |
| 2019 | 717.20892446828 | 0.440454598010708 | 117.489894171848 | 599.719030296432 | Angola |
| 2020 | 617.42264100505 | 0.447283991858922 | 73.554223229601 | 543.868417775449 | Angola |
| 2021 | 550.77373582351 | 0.453721949492174 | 68.9244788375167 | 481.849256985993 | Angola |
| 1990 | 25295.312539876 | 0.216825190875377 | 2297.51229165325 | 22997.8002482227 | Central African Republic |
| 1991 | 24819.1595036834 | 0.220191656485842 | 2138.83725356482 | 22680.3222501186 | Central African Republic |
| 1992 | 24401.2041231735 | 0.223007541295157 | 2138.29465580685 | 22262.9094673666 | Central African Republic |
| 1993 | 23844.1581974081 | 0.225451125286014 | 2128.65862624421 | 21715.4995711639 | Central African Republic |
| 1994 | 23073.0588384301 | 0.228487724402372 | 1937.53151603813 | 21135.527322392 | Central African Republic |
| 1995 | 22550.978923588 | 0.231696792476692 | 1947.98244963911 | 20602.9964739489 | Central African Republic |
| 1996 | 22215.4427022354 | 0.233989591180505 | 1909.26742399284 | 20306.1752782426 | Central African Republic |
| 1997 | 22129.6606713221 | 0.236671999328608 | 1928.68469178447 | 20200.9759795376 | Central African Republic |
| 1998 | 21229.1177974324 | 0.239743528872866 | 1703.27054156313 | 19525.8472558693 | Central African Republic |
| 1999 | 20716.3797879072 | 0.242988530190618 | 1711.88559717445 | 19004.4941907328 | Central African Republic |
| 2000 | 20233.7213680618 | 0.246021131876903 | 1702.59902790375 | 18531.1223401581 | Central African Republic |
| 2001 | 20056.1644711559 | 0.249246740494197 | 1499.11778457956 | 18557.0466865763 | Central African Republic |
| 2002 | 19573.857125771 | 0.252524369440269 | 1510.15400373192 | 18063.7031220391 | Central African Republic |
| 2003 | 19097.7677467546 | 0.25510151863237 | 1519.43456978381 | 17578.3331769708 | Central African Republic |
| 2004 | 18852.8151190198 | 0.257778778286371 | 1369.55834666916 | 17483.2567723506 | Central African Republic |
| 2005 | 18274.4542115545 | 0.26045191761313 | 1392.87520250119 | 16881.5790090533 | Central African Republic |
| 2006 | 18569.7394877374 | 0.263712446226446 | 1373.42896408536 | 17196.310523652 | Central African Republic |
| 2007 | 18649.3270211875 | 0.2673321946143 | 1258.72424866456 | 17390.6027725229 | Central African Republic |
| 2008 | 18495.0154594967 | 0.27115811844024 | 1243.38995430529 | 17251.6255051914 | Central African Republic |
| 2009 | 18197.1946285578 | 0.275338515436591 | 1247.03022704015 | 16950.1644015177 | Central African Republic |
| 2010 | 17627.6283911378 | 0.279981381762208 | 1198.86441467985 | 16428.7639764579 | Central African Republic |
| 2011 | 16721.3830868876 | 0.285097245088929 | 1124.5967141861 | 15596.7863727015 | Central African Republic |
| 2012 | 15563.4828309893 | 0.290460023001198 | 1136.68314836942 | 14426.7996826199 | Central African Republic |
| 2013 | 14976.0439519826 | 0.290104929568684 | 1129.79286094956 | 13846.251091033 | Central African Republic |
| 2014 | 14692.7041456971 | 0.289999498191724 | 1129.32309266056 | 13563.3810530365 | Central African Republic |
| 2015 | 14457.164630933 | 0.290827871178726 | 1134.65643309018 | 13322.5081978428 | Central African Republic |
| 2016 | 14015.0639358272 | 0.29243039953372 | 1079.13198303852 | 12935.9319527887 | Central African Republic |
| 2017 | 13552.4980865422 | 0.294752243072343 | 1076.10693228457 | 12476.3911542576 | Central African Republic |
| 2018 | 12915.5289303435 | 0.298011223092936 | 1074.32496210266 | 11841.2039682408 | Central African Republic |
| 2019 | 12196.0504094409 | 0.301953064146936 | 1018.3947911234 | 11177.6556183175 | Central African Republic |
| 2020 | 10965.9863487999 | 0.305426298180724 | 1023.75019017192 | 9942.23615862797 | Central African Republic |
| 2021 | 10380.6896139573 | 0.309167689992842 | 955.728835089087 | 9424.96077886821 | Central African Republic |
| 1990 | 7203.70259455177 | 0.420654655109748 | 243.752536195074 | 6959.9500583567 | Congo |
| 1991 | 6990.02524653798 | 0.426734373972141 | 235.012648909142 | 6755.01259762884 | Congo |
| 1992 | 6792.55739015303 | 0.431987661930376 | 203.63262080563 | 6588.9247693474 | Congo |
| 1993 | 6637.19916527965 | 0.436606593595936 | 180.463262442304 | 6456.73590283735 | Congo |
| 1994 | 6469.22015428873 | 0.440168346550654 | 121.253611845754 | 6347.96654244298 | Congo |
| 1995 | 6326.66831485464 | 0.443744125258797 | 114.596270188465 | 6212.07204466617 | Congo |
| 1996 | 6229.36287123586 | 0.447566642471598 | 77.6039989097514 | 6151.75887232611 | Congo |
| 1997 | 6178.09956148131 | 0.451026961311742 | 67.1386862395593 | 6110.96087524175 | Congo |
| 1998 | 5956.89610121097 | 0.454445045209992 | 71.6272864522069 | 5885.26881475876 | Congo |
| 1999 | 5674.36284923428 | 0.457176539853177 | 69.1973469016742 | 5605.16550233261 | Congo |
| 2000 | 5457.51687908033 | 0.460764813822723 | 69.1195929090938 | 5388.39728617124 | Congo |
| 2001 | 5150.21977872825 | 0.463751682463704 | 61.0218661574994 | 5089.19791257075 | Congo |
| 2002 | 4955.46118557131 | 0.467037267744411 | 55.9645412595848 | 4899.49664431173 | Congo |
| 2003 | 4685.89063147764 | 0.470134373890519 | 55.8479979943888 | 4630.04263348325 | Congo |
| 2004 | 4401.96824385828 | 0.473612435565969 | 58.0604318464337 | 4343.90781201185 | Congo |
| 2005 | 4356.96051078165 | 0.478137626987132 | 53.9923032290714 | 4302.96820755258 | Congo |
| 2006 | 4127.42829416577 | 0.483355821647145 | 54.3417013283815 | 4073.08659283739 | Congo |
| 2007 | 4012.63669487387 | 0.48764073952289 | 50.4358276634356 | 3962.20086721043 | Congo |
| 2008 | 3759.87111504993 | 0.492822854998204 | 50.8915616924334 | 3708.9795533575 | Congo |
| 2009 | 3481.33346610715 | 0.498299969081535 | 24.7606808358321 | 3456.57278527132 | Congo |
| 2010 | 3121.92393632976 | 0.505542849123574 | 22.991935479246 | 3098.93200085051 | Congo |
| 2011 | 2892.88120868963 | 0.513815777196028 | 22.5130893041562 | 2870.36811938547 | Congo |
| 2012 | 2591.57310679762 | 0.522928282789849 | 22.1442085922653 | 2569.42889820535 | Congo |
| 2013 | 2266.56140542605 | 0.531551678858768 | 22.1387560597598 | 2244.42264936629 | Congo |
| 2014 | 1871.96779182385 | 0.540594486203119 | 21.2243492950882 | 1850.74344252876 | Congo |
| 2015 | 1702.82721350566 | 0.548414029703538 | 20.57960086553 | 1682.24761264013 | Congo |
| 2016 | 1540.57438636283 | 0.555108607713661 | 18.8605103214656 | 1521.71387604136 | Congo |
| 2017 | 1376.90525525265 | 0.561834100154124 | 16.7035865105791 | 1360.20166874207 | Congo |
| 2018 | 1225.60073756905 | 0.567793969630843 | 14.6405459229068 | 1210.96019164614 | Congo |
| 2019 | 1698.40895341045 | 0.573373665303225 | 13.4275984230673 | 1684.98135498738 | Congo |
| 2020 | 1045.94913331313 | 0.578451546357964 | 13.4862540189027 | 1032.46287929423 | Congo |
| 2021 | 969.65341472933 | 0.583075236262154 | 13.2936942159725 | 956.359720513358 | Congo |
| 1990 | 7893.11211975766 | 0.289843210276958 | 1134.52423676501 | 6758.58788299265 | Democratic Republic of the Congo |
| 1991 | 7801.19410966524 | 0.290431567230931 | 1138.14685661557 | 6663.04725304967 | Democratic Republic of the Congo |
| 1992 | 7600.86689254402 | 0.290959242467126 | 1137.34710934167 | 6463.51978320235 | Democratic Republic of the Congo |
| 1993 | 7583.106360458 | 0.289628789613637 | 1130.58006348856 | 6452.52629696944 | Democratic Republic of the Congo |
| 1994 | 8401.41673881095 | 0.286859145655148 | 1138.925105728 | 7262.49163308295 | Democratic Republic of the Congo |
| 1995 | 7645.09418040593 | 0.283967532380546 | 1185.87424361969 | 6459.21993678624 | Democratic Republic of the Congo |
| 1996 | 7782.40351722832 | 0.280413159880167 | 1209.26473318255 | 6573.13878404577 | Democratic Republic of the Congo |
| 1997 | 7921.2288152753 | 0.276336502842209 | 1210.94594905685 | 6710.28286621845 | Democratic Republic of the Congo |
| 1998 | 8212.89946991757 | 0.271813823470493 | 1234.5875156752 | 6978.31195424237 | Democratic Republic of the Congo |
| 1999 | 8243.04323046919 | 0.267367662319482 | 1271.81377936491 | 6971.22945110428 | Democratic Republic of the Congo |
| 2000 | 8451.84224060172 | 0.262456746954109 | 1410.13938602833 | 7041.70285457339 | Democratic Republic of the Congo |
| 2001 | 8410.89675004103 | 0.257857504999669 | 1392.28048109974 | 7018.61626894129 | Democratic Republic of the Congo |
| 2002 | 8268.86918335472 | 0.254136360333323 | 1501.27870226971 | 6767.59048108501 | Democratic Republic of the Congo |
| 2003 | 8143.67400961783 | 0.252333884691643 | 1512.40320159885 | 6631.27080801898 | Democratic Republic of the Congo |
| 2004 | 7666.07264500339 | 0.252470918843583 | 1520.52034966653 | 6145.55229533686 | Democratic Republic of the Congo |
| 2005 | 7311.40370539607 | 0.254161023299852 | 1508.04103716599 | 5803.36266823008 | Democratic Republic of the Congo |
| 2006 | 6952.99268610677 | 0.257355122656431 | 1382.17557941503 | 5570.81710669174 | Democratic Republic of the Congo |
| 2007 | 6580.01338210413 | 0.262272267392268 | 1375.68780396322 | 5204.32557814091 | Democratic Republic of the Congo |
| 2008 | 6068.82600291554 | 0.268103872304045 | 1266.16481977892 | 4802.66118313662 | Democratic Republic of the Congo |
| 2009 | 5623.59281005462 | 0.274361143997572 | 1245.8491139504 | 4377.74369610422 | Democratic Republic of the Congo |
| 2010 | 5122.15576224228 | 0.282223754466439 | 1188.5295423944 | 3933.62621984788 | Democratic Republic of the Congo |
| 2011 | 4561.10293996579 | 0.290872953141928 | 1133.43607308715 | 3427.66686687864 | Democratic Republic of the Congo |
| 2012 | 3976.88757571495 | 0.300454783216411 | 1041.80744974807 | 2935.08012596688 | Democratic Republic of the Congo |
| 2013 | 3407.13410132142 | 0.310831256324369 | 964.37623459716 | 2442.75786672426 | Democratic Republic of the Congo |
| 2014 | 2903.08635630555 | 0.321622927081767 | 824.876185240908 | 2078.21017106464 | Democratic Republic of the Congo |
| 2015 | 2411.88437796552 | 0.33242130666296 | 669.066417043746 | 1742.81796092177 | Democratic Republic of the Congo |
| 2016 | 2029.48988662478 | 0.341988787240158 | 596.057405621336 | 1433.43248100344 | Democratic Republic of the Congo |
| 2017 | 1669.37746191548 | 0.351102035190521 | 591.361792716621 | 1078.01566919886 | Democratic Republic of the Congo |
| 2018 | 1473.81131274633 | 0.36028767655131 | 594.327466731796 | 879.483846014534 | Democratic Republic of the Congo |
| 2019 | 1218.04960433394 | 0.36934068409702 | 573.757380611837 | 644.292223722103 | Democratic Republic of the Congo |
| 2020 | 1035.38626595014 | 0.376712271159843 | 551.790569149965 | 483.595696800175 | Democratic Republic of the Congo |
| 2021 | 907.28309599424 | 0.38317984920136 | 517.915727151923 | 389.367368842317 | Democratic Republic of the Congo |
| 1990 | 15413.7121754646 | 0.268783629292318 | 1245.60135566968 | 14168.1108197949 | Equatorial Guinea |
| 1991 | 14992.898384388 | 0.275296991793454 | 1235.2525841886 | 13757.6458001994 | Equatorial Guinea |
| 1992 | 14435.0357016372 | 0.283487763833002 | 1189.28913004001 | 13245.7465715972 | Equatorial Guinea |
| 1993 | 13855.7257420096 | 0.292236031708562 | 1072.23088003962 | 12783.49486197 | Equatorial Guinea |
| 1994 | 13264.6940157646 | 0.30068634012364 | 1033.41351584899 | 12231.2804999156 | Equatorial Guinea |
| 1995 | 12728.1761690852 | 0.310443551427585 | 972.695125588044 | 11755.4810434972 | Equatorial Guinea |
| 1996 | 11763.5587945428 | 0.324601220926654 | 761.003738345224 | 11002.5550561976 | Equatorial Guinea |
| 1997 | 9895.63498981644 | 0.350550963012752 | 591.011039693451 | 9304.62395012299 | Equatorial Guinea |
| 1998 | 8471.34510214622 | 0.371237163466133 | 541.102254864465 | 7930.24284728176 | Equatorial Guinea |
| 1999 | 7198.22826003428 | 0.391128022748702 | 457.926697860774 | 6740.3015621735 | Equatorial Guinea |
| 2000 | 6096.28436117286 | 0.411383449802448 | 301.26254200898 | 5795.02181916388 | Equatorial Guinea |
| 2001 | 4824.17607624382 | 0.433590056530441 | 201.751269564192 | 4622.42480667963 | Equatorial Guinea |
| 2002 | 3938.72911442684 | 0.451938999588547 | 68.0622965630563 | 3870.66681786378 | Equatorial Guinea |
| 2003 | 3235.17107705726 | 0.467846832610047 | 57.3987056029078 | 3177.77237145435 | Equatorial Guinea |
| 2004 | 2712.39756457831 | 0.483879611699357 | 55.0051078770462 | 2657.39245670126 | Equatorial Guinea |
| 2005 | 2647.97341727483 | 0.498683715051723 | 24.0225317913491 | 2623.95088548348 | Equatorial Guinea |
| 2006 | 1979.77353402742 | 0.511579828259094 | 22.4775622131749 | 1957.29597181425 | Equatorial Guinea |
| 2007 | 1662.93341818761 | 0.524076461332969 | 22.1362929065875 | 1640.79712528102 | Equatorial Guinea |
| 2008 | 1416.20984245092 | 0.537018989870245 | 21.3522509500866 | 1394.85759150083 | Equatorial Guinea |
| 2009 | 1262.18536018102 | 0.548514682896356 | 20.5223513846986 | 1241.66300879632 | Equatorial Guinea |
| 2010 | 1098.27883737133 | 0.55910937700551 | 17.7973095834321 | 1080.4815277879 | Equatorial Guinea |
| 2011 | 942.17597730405 | 0.570277424014124 | 13.5564872833117 | 928.619490020738 | Equatorial Guinea |
| 2012 | 759.61546934011 | 0.581833899261353 | 13.4269550700676 | 746.188514270042 | Equatorial Guinea |
| 2013 | 613.45897474319 | 0.592800613077549 | 13.5701193973189 | 599.888855345871 | Equatorial Guinea |
| 2014 | 535.01084032372 | 0.60347316716397 | 13.502098005436 | 521.508742318284 | Equatorial Guinea |
| 2015 | 466.26832270197 | 0.61313462386841 | 13.3809273256931 | 452.887395376277 | Equatorial Guinea |
| 2016 | 440.99978530626 | 0.621858156591175 | 13.0491644230488 | 427.950620883211 | Equatorial Guinea |
| 2017 | 435.24142939815 | 0.630234538276623 | 11.8720253718577 | 423.369404026292 | Equatorial Guinea |
| 2018 | 422.38383716569 | 0.638022822887303 | 8.73674436194823 | 413.647092803742 | Equatorial Guinea |
| 2019 | 408.81381554886 | 0.645303092753852 | 6.21104603074691 | 402.602769518113 | Equatorial Guinea |
| 2020 | 379.95693736528 | 0.652124902941809 | 5.02753390575351 | 374.929403459526 | Equatorial Guinea |
| 2021 | 361.21361129922 | 0.657857455522296 | 5.15982070312909 | 356.053790596091 | Equatorial Guinea |
| 1990 | 4350.83532218519 | 0.455421186671872 | 64.8777384887054 | 4285.95758369649 | Gabon |
| 1991 | 4080.08812578772 | 0.46156309767229 | 65.1534483323914 | 4014.93467745533 | Gabon |
| 1992 | 3854.63654844861 | 0.467024048058252 | 57.5451154187961 | 3797.09143302981 | Gabon |
| 1993 | 3639.62061794986 | 0.47239446294 | 58.1401144825764 | 3581.48050346728 | Gabon |
| 1994 | 3414.01096715964 | 0.47806194683023 | 54.5024701155443 | 3359.5084970441 | Gabon |
| 1995 | 3187.6708729853 | 0.483826218373876 | 54.1712905976097 | 3133.49958238769 | Gabon |
| 1996 | 2972.26586431452 | 0.48946145974564 | 49.6429929042694 | 2922.62287141025 | Gabon |
| 1997 | 2772.21439927375 | 0.495074161867882 | 49.2091727605936 | 2723.00522651316 | Gabon |
| 1998 | 2616.1817218937 | 0.500372549878702 | 23.2712307348621 | 2592.91049115884 | Gabon |
| 1999 | 2490.86074751777 | 0.504806350324196 | 22.8814458233552 | 2467.97930169441 | Gabon |
| 2000 | 2396.83080264412 | 0.509133087403875 | 22.7724546087549 | 2374.05834803537 | Gabon |
| 2001 | 2253.19526532746 | 0.513045709061385 | 22.4047192302264 | 2230.79054609723 | Gabon |
| 2002 | 2141.53090142739 | 0.516917620976304 | 22.2142045569445 | 2119.31669687045 | Gabon |
| 2003 | 2042.40065505615 | 0.52105141238927 | 22.1067098108314 | 2020.29394524532 | Gabon |
| 2004 | 1976.4990083649 | 0.525479071446482 | 22.1159728929668 | 1954.38303547193 | Gabon |
| 2005 | 1982.6260928438 | 0.530488574568447 | 22.2087744640179 | 1960.41731837978 | Gabon |
| 2006 | 1855.96560145745 | 0.534797715554935 | 21.5895121272382 | 1834.37608933021 | Gabon |
| 2007 | 1731.31663969661 | 0.539441424484129 | 21.1811198143907 | 1710.13551988222 | Gabon |
| 2008 | 1580.97428443915 | 0.544110892340982 | 21.2011803087204 | 1559.77310413043 | Gabon |
| 2009 | 1432.81301751937 | 0.548591318536338 | 20.5960626122642 | 1412.21695490711 | Gabon |
| 2010 | 1270.0041671465 | 0.554022705553671 | 18.8304615932246 | 1251.17370555328 | Gabon |
| 2011 | 1126.1473493293 | 0.560232082065429 | 17.5853520785301 | 1108.56199725077 | Gabon |
| 2012 | 992.24992024483 | 0.566842963853275 | 14.3614650108318 | 977.888455233998 | Gabon |
| 2013 | 934.9462884971 | 0.573808130133551 | 13.2450566118062 | 921.701231885294 | Gabon |
| 2014 | 883.13344223819 | 0.580915656182647 | 13.5404817155857 | 869.592960522604 | Gabon |
| 2015 | 825.67091331685 | 0.58836499960774 | 13.3034081596989 | 812.367505157151 | Gabon |
| 2016 | 751.77582657135 | 0.596062304964046 | 13.2975646123722 | 738.478261958978 | Gabon |
| 2017 | 695.42938107826 | 0.603816251524064 | 13.3473944448251 | 682.081986633435 | Gabon |
| 2018 | 633.80952675493 | 0.611724379630504 | 13.2437354371345 | 620.565791317795 | Gabon |
| 2019 | 568.71023012188 | 0.619904726884927 | 13.1212140081692 | 555.589016113711 | Gabon |
| 2020 | 497.71921898496 | 0.627609647280361 | 12.5917015194653 | 485.127517465495 | Gabon |
| 2021 | 447.00882151765 | 0.634691392755259 | 10.9239228892635 | 436.084898628386 | Gabon |
| 1990 | 8875.15381598471 | 0.205867359800096 | 2322.30789317533 | 6552.84592280938 | Burundi |
| 1991 | 8301.05511549207 | 0.208495720433731 | 2333.57856329115 | 5967.47655220092 | Burundi |
| 1992 | 8221.23083473105 | 0.210864619064193 | 2349.13505191069 | 5872.09578282036 | Burundi |
| 1993 | 7965.2125588153 | 0.213249386007653 | 2301.84938152426 | 5663.36317729104 | Burundi |
| 1994 | 7773.80272721607 | 0.215222973163092 | 2305.46772697782 | 5468.33500023825 | Burundi |
| 1995 | 7054.7878870031 | 0.215631675834044 | 2296.34889116152 | 4758.43899584158 | Burundi |
| 1996 | 6507.82483489781 | 0.215526325356923 | 2307.86912821652 | 4199.9557066813 | Burundi |
| 1997 | 6514.85459546346 | 0.215761819839635 | 2289.52326247958 | 4225.33133298388 | Burundi |
| 1998 | 6245.85102229932 | 0.216402603342113 | 2287.17109092557 | 3958.67993137375 | Burundi |
| 1999 | 5882.49043895042 | 0.216855766644471 | 2293.26442790602 | 3589.2260110444 | Burundi |
| 2000 | 5655.82619576145 | 0.21686985832205 | 2284.11838778006 | 3371.70780798139 | Burundi |
| 2001 | 5560.9068210874 | 0.216988725649785 | 2295.43911822847 | 3265.46770285893 | Burundi |
| 2002 | 5734.32355287684 | 0.217782476516408 | 2293.26261482892 | 3441.06093804792 | Burundi |
| 2003 | 5739.48911818535 | 0.21896153733539 | 2289.47893532921 | 3450.01018285614 | Burundi |
| 2004 | 5902.04638396541 | 0.220918708918727 | 2131.26423745157 | 3770.78214651384 | Burundi |
| 2005 | 5902.11893654854 | 0.223084371742497 | 2158.64682437417 | 3743.47211217437 | Burundi |
| 2006 | 5767.72659340881 | 0.225821889923957 | 2134.84197950372 | 3632.88461390509 | Burundi |
| 2007 | 5762.04426766574 | 0.228996818715961 | 1913.26666706563 | 3848.77760060011 | Burundi |
| 2008 | 5584.55782351441 | 0.232853551450426 | 1885.09284593276 | 3699.46497758165 | Burundi |
| 2009 | 5328.90109852811 | 0.236943039904587 | 1906.9971655592 | 3421.90393296891 | Burundi |
| 2010 | 5009.06906034395 | 0.241447785964046 | 1695.73437059726 | 3313.33468974669 | Burundi |
| 2011 | 4836.96635091383 | 0.246277277175607 | 1697.98169552901 | 3138.98465538482 | Burundi |
| 2012 | 4562.49688165823 | 0.251572302738587 | 1507.29905494275 | 3055.19782671548 | Burundi |
| 2013 | 3812.52940496046 | 0.257191355215958 | 1405.84883271582 | 2406.68057224464 | Burundi |
| 2014 | 3574.55823022607 | 0.262794721997486 | 1376.11232316037 | 2198.4459070657 | Burundi |
| 2015 | 3264.21597798127 | 0.267327938241439 | 1260.18568751431 | 2004.03029046696 | Burundi |
| 2016 | 2946.00057810329 | 0.271587851896567 | 1250.50410312527 | 1695.49647497802 | Burundi |
| 2017 | 2694.24111781301 | 0.275840504541502 | 1193.85698253903 | 1500.38413527398 | Burundi |
| 2018 | 2433.54264978443 | 0.279733289674752 | 1206.42918222767 | 1227.11346755676 | Burundi |
| 2019 | 2244.31688875113 | 0.283442514974853 | 1199.11553422498 | 1045.20135452615 | Burundi |
| 2020 | 2085.42218255807 | 0.286496000475545 | 1132.24005124509 | 953.182131312976 | Burundi |
| 2021 | 1964.55999840785 | 0.289374364823265 | 1136.33403651914 | 828.225961888706 | Burundi |
| 1990 | 6034.88678449026 | 0.270048115983623 | 1234.5515910306 | 4800.33519345966 | Comoros |
| 1991 | 5724.08920967384 | 0.279145649193181 | 1201.04539754845 | 4523.04381212539 | Comoros |
| 1992 | 5353.6380000632 | 0.288506271423925 | 1130.4538634436 | 4223.1841366196 | Comoros |
| 1993 | 4995.25911560993 | 0.297607416571855 | 1071.24324970397 | 3924.01586590596 | Comoros |
| 1994 | 4677.35122048628 | 0.305706587428224 | 1027.97175593207 | 3649.37946455421 | Comoros |
| 1995 | 4299.97393529801 | 0.314220340550933 | 969.667948449246 | 3330.30598684876 | Comoros |
| 1996 | 4008.43797160951 | 0.322249069327522 | 813.958402875667 | 3194.47956873384 | Comoros |
| 1997 | 3849.32601423624 | 0.330148124863673 | 679.769173520709 | 3169.55684071553 | Comoros |
| 1998 | 4561.14063151966 | 0.33751791405714 | 603.814396327183 | 3957.32623519248 | Comoros |
| 1999 | 3842.07024044917 | 0.34477469444905 | 597.2736729428 | 3244.79656750637 | Comoros |
| 2000 | 5318.26825853317 | 0.351875505604714 | 589.25887561167 | 4729.0093829215 | Comoros |
| 2001 | 3370.84343552166 | 0.359211999278107 | 584.192551559205 | 2786.65088396245 | Comoros |
| 2002 | 4129.82429953957 | 0.366486126823935 | 573.307577234477 | 3556.51672230509 | Comoros |
| 2003 | 3051.72643857927 | 0.373410566246042 | 544.195417301019 | 2507.53102127825 | Comoros |
| 2004 | 2916.81954947859 | 0.380035976109197 | 545.354179690623 | 2371.46536978797 | Comoros |
| 2005 | 2831.73416100387 | 0.386891844663173 | 512.92703742744 | 2318.80712357643 | Comoros |
| 2006 | 2776.1238698581 | 0.393521434414153 | 456.260379410269 | 2319.86349044783 | Comoros |
| 2007 | 3446.94472571008 | 0.399051094324319 | 467.722496329412 | 2979.22222938067 | Comoros |
| 2008 | 2768.94341543756 | 0.404649755421042 | 395.739624154624 | 2373.20379128294 | Comoros |
| 2009 | 2525.95973767754 | 0.410253575216193 | 313.640173588769 | 2212.31956408877 | Comoros |
| 2010 | 2219.077144232 | 0.415543376261308 | 273.369005107817 | 1945.70813912418 | Comoros |
| 2011 | 2110.83721401397 | 0.421092971866532 | 246.877547225815 | 1863.95966678815 | Comoros |
| 2012 | 1883.66039525772 | 0.42672995742339 | 234.345889239866 | 1649.31450601785 | Comoros |
| 2013 | 1685.84189788534 | 0.432789931825945 | 206.50915689831 | 1479.33274098703 | Comoros |
| 2014 | 1734.43176038267 | 0.438844505062188 | 111.098063507084 | 1623.33369687559 | Comoros |
| 2015 | 1606.75976862103 | 0.444712393572096 | 116.631874104155 | 1490.12789451688 | Comoros |
| 2016 | 1465.61292159159 | 0.450506978607507 | 69.0976047928748 | 1396.51531679872 | Comoros |
| 2017 | 1396.44427324724 | 0.456280841429385 | 65.7415248642228 | 1330.70274838302 | Comoros |
| 2018 | 1283.49510290731 | 0.461951343654461 | 61.7027508946434 | 1221.79235201267 | Comoros |
| 2019 | 1191.78218345042 | 0.46738801351741 | 56.4884033680522 | 1135.29378008237 | Comoros |
| 2020 | 1175.5527344467 | 0.472003132433584 | 56.332293378353 | 1119.22044106835 | Comoros |
| 2021 | 1118.35248025171 | 0.475978688247514 | 58.4695614541278 | 1059.88291879758 | Comoros |
| 1990 | 9902.21480623266 | 0.337781788993477 | 602.457344328114 | 9299.75746190454 | Djibouti |
| 1991 | 8795.24765172891 | 0.338770010818962 | 592.685471687701 | 8202.56218004121 | Djibouti |
| 1992 | 8718.7503506407 | 0.340986061300938 | 597.410392287068 | 8121.33995835363 | Djibouti |
| 1993 | 11534.69653148 | 0.344904393563471 | 600.201084971483 | 10934.4954465085 | Djibouti |
| 1994 | 9239.32997568033 | 0.34798140392569 | 597.40850178203 | 8641.9214738983 | Djibouti |
| 1995 | 8677.17287218136 | 0.351245633337663 | 592.828284364272 | 8084.34458781709 | Djibouti |
| 1996 | 8159.99650411818 | 0.354385634187117 | 590.40714671434 | 7569.58935740384 | Djibouti |
| 1997 | 8217.5732944018 | 0.35809640096391 | 596.968716875441 | 7620.60457752636 | Djibouti |
| 1998 | 7046.98725896301 | 0.361596763593584 | 573.026672631581 | 6473.96058633143 | Djibouti |
| 1999 | 6370.65023276868 | 0.365305211425089 | 573.493581624286 | 5797.15665114439 | Djibouti |
| 2000 | 6012.56277757529 | 0.369015094624057 | 579.881533401965 | 5432.68124417333 | Djibouti |
| 2001 | 5543.38240351999 | 0.372853155297047 | 542.203336277135 | 5001.17906724285 | Djibouti |
| 2002 | 5088.70684851433 | 0.37665000401959 | 546.664649919668 | 4542.04219859466 | Djibouti |
| 2003 | 4845.46878270268 | 0.380686615222992 | 519.366852498468 | 4326.10193020421 | Djibouti |
| 2004 | 4694.31746132974 | 0.384987705849076 | 503.285049930311 | 4191.03241139943 | Djibouti |
| 2005 | 4289.36526741767 | 0.389568705689455 | 516.67429130878 | 3772.69097610889 | Djibouti |
| 2006 | 4171.76206493962 | 0.395037470761535 | 459.864740363207 | 3711.89732457641 | Djibouti |
| 2007 | 3969.78005468451 | 0.40061854555971 | 460.108485052626 | 3509.67156963188 | Djibouti |
| 2008 | 3649.2850711682 | 0.406658303036044 | 367.125378898668 | 3282.15969226953 | Djibouti |
| 2009 | 3455.85969196436 | 0.411969334510517 | 297.652913823133 | 3158.20677814123 | Djibouti |
| 2010 | 3549.82295181185 | 0.416999349607211 | 261.198377031404 | 3288.62457478045 | Djibouti |
| 2011 | 2964.86933132982 | 0.422316140090975 | 245.543531332033 | 2719.32579999779 | Djibouti |
| 2012 | 2718.02294983048 | 0.427711440066409 | 214.34709812631 | 2503.67585170417 | Djibouti |
| 2013 | 2543.81323052708 | 0.43336043651961 | 208.214246971706 | 2335.59898355537 | Djibouti |
| 2014 | 2112.79637305545 | 0.439455721647865 | 107.845669564553 | 2004.9507034909 | Djibouti |
| 2015 | 1942.43606789984 | 0.445855284807829 | 111.632381665141 | 1830.8036862347 | Djibouti |
| 2016 | 1834.42037477632 | 0.453442853845541 | 75.3414394273306 | 1759.07893534899 | Djibouti |
| 2017 | 1642.41242399255 | 0.460440151860865 | 67.2245593907596 | 1575.18786460179 | Djibouti |
| 2018 | 1433.90933498688 | 0.46770567196373 | 57.1093394182487 | 1376.79999556863 | Djibouti |
| 2019 | 1282.02276001871 | 0.475263242846912 | 57.3810106735414 | 1224.64174934517 | Djibouti |
| 2020 | 1142.91265681285 | 0.481879945321652 | 53.9317166672767 | 1088.98094014557 | Djibouti |
| 2021 | 992.89474802549 | 0.4879583707169 | 50.2371797966162 | 942.657568228874 | Djibouti |
| 1990 | 15329.2783178318 | 0.216028238900354 | 2314.43025153432 | 13014.8480662975 | Eritrea |
| 1991 | 14902.1127271847 | 0.222574737210924 | 2138.69659111831 | 12763.4161360664 | Eritrea |
| 1992 | 15126.8405220372 | 0.230296065775865 | 1913.58830613683 | 13213.2522159004 | Eritrea |
| 1993 | 14291.4828018852 | 0.239385041260314 | 1727.25558416596 | 12564.2272177192 | Eritrea |
| 1994 | 12634.4786559386 | 0.250752541052101 | 1518.62246500755 | 11115.856190931 | Eritrea |
| 1995 | 11340.3117333185 | 0.261498484754305 | 1402.62560051677 | 9937.68613280173 | Eritrea |
| 1996 | 10155.2471356916 | 0.272527054525239 | 1243.01358351009 | 8912.23355218151 | Eritrea |
| 1997 | 9310.91455915344 | 0.283551720614883 | 1200.58711729572 | 8110.32744185772 | Eritrea |
| 1998 | 8712.49166432389 | 0.2937648278699 | 1079.32392487761 | 7633.16773944628 | Eritrea |
| 1999 | 8273.65211442632 | 0.303142339840286 | 1030.17350514605 | 7243.47860928027 | Eritrea |
| 2000 | 7879.14579212825 | 0.310248161765321 | 958.752350325136 | 6920.39344180311 | Eritrea |
| 2001 | 7765.28401558873 | 0.317247987543295 | 844.166156187231 | 6921.1178594015 | Eritrea |
| 2002 | 7477.82170434137 | 0.323566584840077 | 767.602449566069 | 6710.2192547753 | Eritrea |
| 2003 | 6925.17654427823 | 0.328593580738491 | 746.992703155226 | 6178.183841123 | Eritrea |
| 2004 | 6699.48838795802 | 0.332879380249727 | 669.696679492189 | 6029.79170846583 | Eritrea |
| 2005 | 6322.4998103545 | 0.336682510811316 | 678.313027274124 | 5644.18678308038 | Eritrea |
| 2006 | 5984.00941916917 | 0.339835823599651 | 595.393554192753 | 5388.61586497642 | Eritrea |
| 2007 | 5704.07748840686 | 0.342843174407113 | 599.150542548032 | 5104.92694585883 | Eritrea |
| 2008 | 5437.17284390558 | 0.343427800730465 | 589.575197345517 | 4847.59764656006 | Eritrea |
| 2009 | 4941.84101245998 | 0.345147742630626 | 598.541611938673 | 4343.29940052131 | Eritrea |
| 2010 | 4635.99715739188 | 0.348146176900867 | 595.174567118901 | 4040.82259027298 | Eritrea |
| 2011 | 4241.12087180821 | 0.35416314653227 | 592.198298751279 | 3648.92257305693 | Eritrea |
| 2012 | 3894.98640859205 | 0.360112751410199 | 587.206316360469 | 3307.78009223158 | Eritrea |
| 2013 | 3738.36699789246 | 0.364343653311207 | 569.212851416339 | 3169.15414647612 | Eritrea |
| 2014 | 3071.5633641232 | 0.372527587512835 | 546.515553455258 | 2525.04781066794 | Eritrea |
| 2015 | 2896.25378992787 | 0.376935162282393 | 546.260831521955 | 2349.99295840592 | Eritrea |
| 2016 | 2793.16685816472 | 0.382171665821118 | 501.524815489173 | 2291.64204267555 | Eritrea |
| 2017 | 2627.3750723549 | 0.385936029208135 | 508.900367280463 | 2118.47470507444 | Eritrea |
| 2018 | 2456.15027371154 | 0.39115488426675 | 465.475572451094 | 1990.67470126045 | Eritrea |
| 2019 | 2336.53104926482 | 0.396330558249013 | 463.114755313685 | 1873.41629395113 | Eritrea |
| 2020 | 2191.29528279876 | 0.400459411382762 | 462.8922291095 | 1728.40305368926 | Eritrea |
| 2021 | 2057.01818766248 | 0.403863942828898 | 390.740092767621 | 1666.27809489486 | Eritrea |
| 1990 | 18787.8468774259 | 0.148033884957629 | 3520.12634877425 | 15267.7205286517 | Ethiopia |
| 1991 | 18265.1311980498 | 0.149599710414418 | 3202.0516870643 | 15063.0795109855 | Ethiopia |
| 1992 | 17873.0542897459 | 0.15017438640674 | 3232.83722678078 | 14640.2170629651 | Ethiopia |
| 1993 | 17439.7193144167 | 0.151582364387045 | 3243.9088738962 | 14195.8104405205 | Ethiopia |
| 1994 | 16981.671818533 | 0.152982636940145 | 3094.24169862814 | 13887.4301199049 | Ethiopia |
| 1995 | 16197.4185103659 | 0.155085604929282 | 3112.5318830212 | 13084.8866273447 | Ethiopia |
| 1996 | 15273.0089466803 | 0.158127229482336 | 2873.41147024827 | 12399.597476432 | Ethiopia |
| 1997 | 14392.5167765252 | 0.161874742949531 | 2628.95204705216 | 11763.564729473 | Ethiopia |
| 1998 | 13524.2182421493 | 0.16507771008878 | 2519.34986198724 | 11004.8683801621 | Ethiopia |
| 1999 | 12621.2146634553 | 0.168903545355995 | 2347.25758197071 | 10273.9570814846 | Ethiopia |
| 2000 | 11796.4635695682 | 0.173418529946986 | 2354.44234962535 | 9442.02121994285 | Ethiopia |
| 2001 | 10978.446344478 | 0.178972968614763 | 2350.02701995641 | 8628.41932452159 | Ethiopia |
| 2002 | 10217.07709086 | 0.184138012848519 | 2321.15882982503 | 7895.91826103497 | Ethiopia |
| 2003 | 9345.593381044 | 0.188694814030626 | 2353.12888073186 | 6992.46450031214 | Ethiopia |
| 2004 | 8533.81652592516 | 0.195030701395242 | 2334.21958419635 | 6199.59694172881 | Ethiopia |
| 2005 | 8083.76635813279 | 0.202868242862353 | 2343.18628297377 | 5740.58007515902 | Ethiopia |
| 2006 | 7629.30147514449 | 0.211524228741913 | 2313.32284183862 | 5315.97863330587 | Ethiopia |
| 2007 | 7183.67374229751 | 0.221819930759394 | 2164.4028746851 | 5019.27086761241 | Ethiopia |
| 2008 | 6697.4124798054 | 0.232872185647818 | 1935.78242383341 | 4761.63005597199 | Ethiopia |
| 2009 | 6251.35950084858 | 0.244265818456334 | 1724.95848002973 | 4526.40102081885 | Ethiopia |
| 2010 | 5591.75075829929 | 0.256081459859031 | 1512.64584861711 | 4079.10490968218 | Ethiopia |
| 2011 | 5306.70514194222 | 0.267880862080398 | 1258.87106491583 | 4047.83407702639 | Ethiopia |
| 2012 | 4901.35455250279 | 0.279006957893266 | 1192.72751752584 | 3708.62703497695 | Ethiopia |
| 2013 | 4479.49314718459 | 0.289885266889051 | 1138.39549876847 | 3341.09764841612 | Ethiopia |
| 2014 | 4237.3523910147 | 0.300484547961246 | 1015.83222180133 | 3221.52016921337 | Ethiopia |
| 2015 | 3939.47642234682 | 0.310736418709888 | 964.332874823876 | 2975.14354752294 | Ethiopia |
| 2016 | 3672.17635483568 | 0.320431867242816 | 830.444101959729 | 2841.73225287595 | Ethiopia |
| 2017 | 3582.93782962718 | 0.329469243719685 | 745.841198653749 | 2837.09663097343 | Ethiopia |
| 2018 | 3428.39282045111 | 0.338050074820716 | 598.429991706156 | 2829.96282874495 | Ethiopia |
| 2019 | 3211.84422922397 | 0.346422663706934 | 597.571021569569 | 2614.2732076544 | Ethiopia |
| 2020 | 2905.90888633416 | 0.353216349837522 | 594.447098625009 | 2311.46178770915 | Ethiopia |
| 2021 | 2694.06544941008 | 0.358823295416803 | 593.685511324296 | 2100.37993808578 | Ethiopia |
| 1990 | 6711.72564999708 | 0.333850293102656 | 680.851314178207 | 6030.87433581887 | Kenya |
| 1991 | 6408.31044646678 | 0.340476220279675 | 591.633155807446 | 5816.67729065933 | Kenya |
| 1992 | 6211.21167617041 | 0.346298636491363 | 592.971201667231 | 5618.24047450318 | Kenya |
| 1993 | 6000.73735873135 | 0.351611769443661 | 603.082100807956 | 5397.65525792339 | Kenya |
| 1994 | 5831.10495325917 | 0.356779193882903 | 597.98923708831 | 5233.11571617086 | Kenya |
| 1995 | 5660.16050928017 | 0.361535806569139 | 569.211270616385 | 5090.94923866378 | Kenya |
| 1996 | 5501.92316567726 | 0.366203076018008 | 573.393034068735 | 4928.53013160853 | Kenya |
| 1997 | 5598.30614733675 | 0.370436464683744 | 577.079905527128 | 5021.22624180962 | Kenya |
| 1998 | 5650.34307942184 | 0.374581400693507 | 546.08321394885 | 5104.25986547299 | Kenya |
| 1999 | 5550.2334850181 | 0.378630009408301 | 544.79329252581 | 5005.44019249229 | Kenya |
| 2000 | 5204.22279084961 | 0.382395616264527 | 504.442311850982 | 4699.78047899863 | Kenya |
| 2001 | 4957.15984344515 | 0.386188414766973 | 509.010125817259 | 4448.14971762789 | Kenya |
| 2002 | 4821.37404233579 | 0.389886256007927 | 514.972388130523 | 4306.40165420527 | Kenya |
| 2003 | 4796.16613579255 | 0.393680479758456 | 461.496534783008 | 4334.66960100954 | Kenya |
| 2004 | 4649.77534908504 | 0.397743651275382 | 466.694657416365 | 4183.08069166868 | Kenya |
| 2005 | 4579.52986278234 | 0.402444011242538 | 392.650457552461 | 4186.87940522988 | Kenya |
| 2006 | 4500.54734955742 | 0.407745764308496 | 314.378430540159 | 4186.16891901726 | Kenya |
| 2007 | 4523.41584283615 | 0.413736227073594 | 273.032926817585 | 4250.38291601857 | Kenya |
| 2008 | 4375.10011905529 | 0.419738146655789 | 260.545403945763 | 4114.55471510953 | Kenya |
| 2009 | 4331.91756504831 | 0.426123888497405 | 235.663650382268 | 4096.25391466604 | Kenya |
| 2010 | 4264.65218960387 | 0.433305839416248 | 205.296653646044 | 4059.35553595783 | Kenya |
| 2011 | 4065.48901578756 | 0.440893830428021 | 107.600642676256 | 3957.8883731113 | Kenya |
| 2012 | 3772.04359855349 | 0.448666395692026 | 70.2938842160328 | 3701.74971433746 | Kenya |
| 2013 | 3480.37815826261 | 0.456666941870395 | 65.3490807502967 | 3415.02907751231 | Kenya |
| 2014 | 2987.70344248782 | 0.464724302152848 | 60.5689844894694 | 2927.13445799835 | Kenya |
| 2015 | 2715.25208779979 | 0.473084051301145 | 57.6219226934075 | 2657.63016510638 | Kenya |
| 2016 | 2448.20050118439 | 0.481709072334755 | 54.4847341341077 | 2393.71576705028 | Kenya |
| 2017 | 2307.12104149655 | 0.490296619363422 | 49.7384157028587 | 2257.38262579369 | Kenya |
| 2018 | 2048.7972645716 | 0.499101274473837 | 24.1513601291327 | 2024.64590444247 | Kenya |
| 2019 | 1888.99115106999 | 0.508004210679317 | 22.6979257528328 | 1866.29322531716 | Kenya |
| 2020 | 1720.64300062848 | 0.516136571818187 | 22.2997880125827 | 1698.3432126159 | Kenya |
| 2021 | 1577.23487607048 | 0.523768077100117 | 22.1591922050322 | 1555.07568386545 | Kenya |
| 1990 | 14938.7965296484 | 0.279889464787504 | 1194.22499073443 | 13744.571538914 | Madagascar |
| 1991 | 14437.3238783948 | 0.280429588849502 | 1197.64631156211 | 13239.6775668327 | Madagascar |
| 1992 | 13988.0758367865 | 0.280501282838033 | 1208.33030916543 | 12779.7455276211 | Madagascar |
| 1993 | 13579.7937437524 | 0.280204299153331 | 1196.93000970893 | 12382.8637340435 | Madagascar |
| 1994 | 13189.8383655247 | 0.279615149051304 | 1195.93420088806 | 11993.9041646366 | Madagascar |
| 1995 | 12782.2047510021 | 0.279164003276889 | 1190.7552442735 | 11591.4495067286 | Madagascar |
| 1996 | 12387.9492403843 | 0.279066492612967 | 1197.64145191681 | 11190.3077884675 | Madagascar |
| 1997 | 11998.7625576648 | 0.279426189949827 | 1197.60748342369 | 10801.1550742411 | Madagascar |
| 1998 | 11446.0502828337 | 0.280317670990881 | 1199.72619444171 | 10246.324088392 | Madagascar |
| 1999 | 11450.8230598237 | 0.281571072098772 | 1207.95041457265 | 10242.872645251 | Madagascar |
| 2000 | 10657.0311463617 | 0.283190720794041 | 1194.65784194939 | 9462.37330441231 | Madagascar |
| 2001 | 10325.9938400809 | 0.285668180077333 | 1126.61778572999 | 9199.37605435091 | Madagascar |
| 2002 | 9790.72248731326 | 0.287153148772741 | 1135.55303041911 | 8655.16945689415 | Madagascar |
| 2003 | 9458.95132164428 | 0.289675457328935 | 1120.65916017021 | 8338.29216147407 | Madagascar |
| 2004 | 9165.35885593937 | 0.292629884521451 | 1089.15416467585 | 8076.20469126353 | Madagascar |
| 2005 | 9672.41629365008 | 0.295919326698766 | 1093.06533685601 | 8579.35095679407 | Madagascar |
| 2006 | 9489.95624674313 | 0.299723392356893 | 1037.1886810779 | 8452.76756566523 | Madagascar |
| 2007 | 9407.66483173216 | 0.304026244970223 | 1027.97603360915 | 8379.688798123 | Madagascar |
| 2008 | 9401.37460174621 | 0.308990264960967 | 965.430923807282 | 8435.94367793893 | Madagascar |
| 2009 | 9232.48559344716 | 0.313450891076854 | 954.650330901799 | 8277.83526254536 | Madagascar |
| 2010 | 8692.15898082796 | 0.318353184360276 | 824.42084562317 | 7867.73813520479 | Madagascar |
| 2011 | 8615.89685303673 | 0.323801042883395 | 754.785130293517 | 7861.11172274321 | Madagascar |
| 2012 | 8290.53911588887 | 0.329947540970422 | 678.586288544577 | 7611.95282734429 | Madagascar |
| 2013 | 7715.74865166314 | 0.336519231511991 | 669.314971797602 | 7046.43367986554 | Madagascar |
| 2014 | 7039.27776364797 | 0.343352610546557 | 603.518255896298 | 6435.75950775167 | Madagascar |
| 2015 | 6607.28742981407 | 0.350443595243704 | 592.055696549488 | 6015.23173326458 | Madagascar |
| 2016 | 6231.00592617557 | 0.35802634126176 | 592.413312588855 | 5638.59261358671 | Madagascar |
| 2017 | 5844.5267545084 | 0.36623558721941 | 579.463472886164 | 5265.06328162224 | Madagascar |
| 2018 | 5420.33019574085 | 0.3749696444566 | 545.016425652486 | 4875.31377008836 | Madagascar |
| 2019 | 4895.91755181636 | 0.384209195656002 | 518.162027518983 | 4377.75552429738 | Madagascar |
| 2020 | 4752.70766514914 | 0.392778708989164 | 464.127007284479 | 4288.58065786466 | Madagascar |
| 2021 | 4377.38420901519 | 0.40024694263784 | 461.50453822996 | 3915.87967078523 | Madagascar |
| 1990 | 21768.8506315044 | 0.204010244634822 | 2325.69946827615 | 19443.1511632282 | Malawi |
| 1991 | 21149.188301872 | 0.206043785604482 | 2330.56289275198 | 18818.62540912 | Malawi |
| 1992 | 20089.5501771489 | 0.206732358761786 | 2323.39524359797 | 17766.1549335509 | Malawi |
| 1993 | 19429.4306269617 | 0.20809120460636 | 2327.98804846613 | 17101.4425784956 | Malawi |
| 1994 | 17974.375768517 | 0.209144565934722 | 2330.47927598114 | 15643.8964925359 | Malawi |
| 1995 | 17271.7613907521 | 0.211756231927772 | 2300.76757133519 | 14970.9938194169 | Malawi |
| 1996 | 15856.101873486 | 0.215264474919532 | 2314.46154262176 | 13541.6403308642 | Malawi |
| 1997 | 14211.0073703148 | 0.219416251726227 | 2299.85522543725 | 11911.1521448775 | Malawi |
| 1998 | 12886.6839238682 | 0.223122329682106 | 2132.95531493723 | 10753.728608931 | Malawi |
| 1999 | 13145.7660892795 | 0.22706836834043 | 2139.28351657815 | 11006.4825727014 | Malawi |
| 2000 | 11943.7122786876 | 0.230900810051145 | 1933.53823526822 | 10010.1740434194 | Malawi |
| 2001 | 11735.3259578489 | 0.234020506815413 | 1919.7192988738 | 9815.6066589751 | Malawi |
| 2002 | 10554.52677075 | 0.237602155431587 | 1918.95284993242 | 8635.57392081759 | Malawi |
| 2003 | 9426.03758996248 | 0.241559227766521 | 1746.71951753433 | 7679.31807242815 | Malawi |
| 2004 | 8494.80957237712 | 0.246434539760473 | 1694.53548683711 | 6800.27408554001 | Malawi |
| 2005 | 7747.1053602284 | 0.251672317552778 | 1500.48933538104 | 6246.61602484736 | Malawi |
| 2006 | 7353.15710455956 | 0.257595649191555 | 1386.27482224466 | 5966.8822823149 | Malawi |
| 2007 | 6705.94848805862 | 0.265226927963354 | 1395.77974752698 | 5310.16874053164 | Malawi |
| 2008 | 5997.78358705073 | 0.273689856434299 | 1229.39347584542 | 4768.39011120531 | Malawi |
| 2009 | 5769.87277620314 | 0.282296508700983 | 1198.53577592401 | 4571.33700027913 | Malawi |
| 2010 | 5496.27805669076 | 0.291179619969706 | 1139.91165772637 | 4356.36639896439 | Malawi |
| 2011 | 5317.61316415669 | 0.300448509120473 | 1032.61451455511 | 4284.99864960158 | Malawi |
| 2012 | 4584.39497652765 | 0.30919762412021 | 972.601987533819 | 3611.79298899383 | Malawi |
| 2013 | 4067.35094260582 | 0.317936213061346 | 846.853809663229 | 3220.49713294259 | Malawi |
| 2014 | 3721.89567463336 | 0.327042425505685 | 772.763384726443 | 2949.13228990692 | Malawi |
| 2015 | 3436.89912389317 | 0.33614013833142 | 664.747914219066 | 2772.1512096741 | Malawi |
| 2016 | 3178.82771399196 | 0.344731541741046 | 595.815223298597 | 2583.01249069336 | Malawi |
| 2017 | 2915.76093064716 | 0.353121067414073 | 584.920553360805 | 2330.84037728635 | Malawi |
| 2018 | 2738.82930573811 | 0.361470410425433 | 594.591365739846 | 2144.23793999826 | Malawi |
| 2019 | 2569.85876736757 | 0.37011460502829 | 573.133162778595 | 1996.72560458898 | Malawi |
| 2020 | 2320.51921694643 | 0.377879020413869 | 545.572198472235 | 1774.94701847419 | Malawi |
| 2021 | 2130.94889121952 | 0.384553633730301 | 508.491425584449 | 1622.45746563507 | Malawi |
| 1990 | 151.31974788545 | 0.544586533153919 | 21.1161257344234 | 130.203622151027 | Mauritius |
| 1991 | 106.60623400345 | 0.548209120513745 | 20.5828581598043 | 86.0233758436457 | Mauritius |
| 1992 | 100.65710567795 | 0.55345463047762 | 19.7124659242704 | 80.9446397536796 | Mauritius |
| 1993 | 87.70299321952 | 0.560688639662726 | 17.5866698378832 | 70.1163233816368 | Mauritius |
| 1994 | 88.41543852473 | 0.569092171296203 | 14.135184124769 | 74.280254399961 | Mauritius |
| 1995 | 83.07498218543 | 0.577319390278784 | 13.4291671974986 | 69.6458149879314 | Mauritius |
| 1996 | 81.99492948271 | 0.584797060785555 | 13.3010748356654 | 68.6938546470446 | Mauritius |
| 1997 | 66.75492403023 | 0.591497734589023 | 13.5794188238692 | 53.1755052063608 | Mauritius |
| 1998 | 65.67093175188 | 0.597363039395481 | 13.2953889395938 | 52.3755428122862 | Mauritius |
| 1999 | 69.78499643866 | 0.602034233757503 | 13.2635759991811 | 56.5214204394789 | Mauritius |
| 2000 | 66.50475468487 | 0.6070879010983 | 13.4178191840392 | 53.0869355008308 | Mauritius |
| 2001 | 64.93641600695 | 0.612240817263126 | 13.3345272487517 | 51.6018887581982 | Mauritius |
| 2002 | 64.46470531784 | 0.617190006807373 | 12.9551723025017 | 51.5095330153383 | Mauritius |
| 2003 | 59.5427478896 | 0.622764532968127 | 13.009670026327 | 46.533077863273 | Mauritius |
| 2004 | 63.72408509118 | 0.629030192646747 | 12.7140541398451 | 51.0100309513349 | Mauritius |
| 2005 | 61.22746313442 | 0.634766579912803 | 10.6781558530511 | 50.5493072813689 | Mauritius |
| 2006 | 68.16565263596 | 0.639545182683383 | 8.33532223905704 | 59.830330396903 | Mauritius |
| 2007 | 73.03246916728 | 0.64435865560727 | 6.15780086026403 | 66.874668307016 | Mauritius |
| 2008 | 73.49285901499 | 0.649630852006361 | 5.38646201286344 | 68.1063970021266 | Mauritius |
| 2009 | 70.61882325779 | 0.655000295441282 | 5.14545224017819 | 65.4733710176118 | Mauritius |
| 2010 | 62.2311038168 | 0.660469800171028 | 4.75890221756134 | 57.4722015992387 | Mauritius |
| 2011 | 61.29294951666 | 0.66649762677449 | 3.58522367356707 | 57.7077258430929 | Mauritius |
| 2012 | 60.41067999206 | 0.673127650407144 | 3.37628915127944 | 57.0343908407806 | Mauritius |
| 2013 | 57.39959573806 | 0.680114023433391 | 2.90550815664564 | 54.4940875814144 | Mauritius |
| 2014 | 52.33060887569 | 0.686616503167309 | 2.73250000651491 | 49.5981088691751 | Mauritius |
| 2015 | 51.17291872451 | 0.692239729639954 | 2.57714159952145 | 48.5957771249886 | Mauritius |
| 2016 | 50.37163836649 | 0.697020562490311 | 2.41884436354136 | 47.9527940029486 | Mauritius |
| 2017 | 49.82343537581 | 0.70161813647985 | 2.3967892523374 | 47.4266461234726 | Mauritius |
| 2018 | 52.22268364545 | 0.706266650972885 | 2.33222109730355 | 49.8904625481465 | Mauritius |
| 2019 | 47.61967705398 | 0.710935044634248 | 2.25134671187435 | 45.3683303421056 | Mauritius |
| 2020 | 42.68677296 | 0.714629803881546 | 2.25893444925031 | 40.4278385107497 | Mauritius |
| 2021 | 39.21728581765 | 0.718260445552653 | 2.22085894117118 | 36.9964268764788 | Mauritius |
| 1990 | 10185.742487734 | 0.173064715394479 | 2320.8247427168 | 7864.9177450172 | Mozambique |
| 1991 | 9157.13756500344 | 0.175550435322203 | 2346.02777019807 | 6811.10979480537 | Mozambique |
| 1992 | 8725.98484440375 | 0.17728518828037 | 2356.17713793577 | 6369.80770646798 | Mozambique |
| 1993 | 8247.87800642051 | 0.179851807348523 | 2326.78612880327 | 5921.09187761723 | Mozambique |
| 1994 | 7541.50506459685 | 0.182236860582392 | 2338.09099404232 | 5203.41407055453 | Mozambique |
| 1995 | 7106.3094911932 | 0.183427981878199 | 2358.12782892629 | 4748.18166226691 | Mozambique |
| 1996 | 6808.66518087588 | 0.185705869370502 | 2344.56521370711 | 4464.09996716877 | Mozambique |
| 1997 | 6681.88965220522 | 0.188650435754761 | 2307.04285152268 | 4374.84680068254 | Mozambique |
| 1998 | 6512.69609223993 | 0.191998629373624 | 2330.30753330541 | 4182.38855893452 | Mozambique |
| 1999 | 6446.41764016076 | 0.195435287798378 | 2344.38268103807 | 4102.03495912269 | Mozambique |
| 2000 | 5785.57404227497 | 0.198534396867784 | 2334.24348001599 | 3451.33056225898 | Mozambique |
| 2001 | 5306.99072460752 | 0.20224192395185 | 2351.98713079704 | 2955.00359381048 | Mozambique |
| 2002 | 4992.1596609368 | 0.206390059635981 | 2340.03687455678 | 2652.12278638002 | Mozambique |
| 2003 | 4740.26634764842 | 0.210534489558039 | 2330.11392809331 | 2410.15241955511 | Mozambique |
| 2004 | 4499.28371896382 | 0.214972457474336 | 2304.88170707409 | 2194.40201188973 | Mozambique |
| 2005 | 4220.59491961464 | 0.219692372119565 | 2139.71130844351 | 2080.88361117113 | Mozambique |
| 2006 | 4389.50045696728 | 0.22475808709223 | 2132.26482613663 | 2257.23563083065 | Mozambique |
| 2007 | 4192.35951481029 | 0.230158226784202 | 1926.28620046488 | 2266.07331434541 | Mozambique |
| 2008 | 4021.18010065693 | 0.235790170219554 | 1932.97983697297 | 2088.20026368396 | Mozambique |
| 2009 | 3800.25581971113 | 0.241360656275235 | 1688.4410532932 | 2111.81476641793 | Mozambique |
| 2010 | 3436.8029713055 | 0.246751499854601 | 1681.57976104711 | 1755.22321025839 | Mozambique |
| 2011 | 3031.32666944846 | 0.252232471060626 | 1539.61021197342 | 1491.71645747504 | Mozambique |
| 2012 | 2878.47872549166 | 0.258315562328104 | 1372.99149609328 | 1505.48722939838 | Mozambique |
| 2013 | 2681.06984547845 | 0.265280462139272 | 1405.66401500432 | 1275.40583047413 | Mozambique |
| 2014 | 2542.22302534046 | 0.272892813606321 | 1232.15289504843 | 1310.07013029203 | Mozambique |
| 2015 | 2144.63688367117 | 0.2810326852532 | 1201.0211397372 | 943.615743933974 | Mozambique |
| 2016 | 1918.52721431411 | 0.289152424860155 | 1131.32873338475 | 787.198480929362 | Mozambique |
| 2017 | 1669.72990869875 | 0.297164772494457 | 1079.9113967529 | 589.818511945849 | Mozambique |
| 2018 | 1497.88813812437 | 0.305226771938642 | 1025.38103989787 | 472.507098226502 | Mozambique |
| 2019 | 1476.53379868989 | 0.313174508096083 | 961.267410524402 | 515.266388165488 | Mozambique |
| 2020 | 1345.77542682375 | 0.320291181995807 | 839.700133347594 | 506.075293476156 | Mozambique |
| 2021 | 1224.06429219393 | 0.326462613636377 | 757.750294034145 | 466.313998159785 | Mozambique |
| 1990 | 10154.5509659198 | 0.275097189589071 | 1230.8680663829 | 8923.6828995369 | Rwanda |
| 1991 | 10330.3939319829 | 0.276194698465909 | 1205.40780839911 | 9124.98612358379 | Rwanda |
| 1992 | 9899.14504931077 | 0.277178171463842 | 1199.90707451087 | 8699.2379747999 | Rwanda |
| 1993 | 8784.60083423788 | 0.276980133306619 | 1200.03214475687 | 7584.568689481 | Rwanda |
| 1994 | 24208.7342055673 | 0.272331257765128 | 1250.30696296203 | 22958.4272426053 | Rwanda |
| 1995 | 11818.3046022152 | 0.271102226876129 | 1238.45272028257 | 10579.8518819326 | Rwanda |
| 1996 | 11363.1967517575 | 0.271097922261967 | 1246.85434846157 | 10116.3424032959 | Rwanda |
| 1997 | 9746.94468639538 | 0.272455860850458 | 1232.49460809635 | 8514.45007829903 | Rwanda |
| 1998 | 9047.85471383625 | 0.274582550589084 | 1245.62287169462 | 7802.23184214163 | Rwanda |
| 1999 | 8742.03235275169 | 0.277014387103527 | 1202.27576233324 | 7539.75659041845 | Rwanda |
| 2000 | 8462.30703369512 | 0.280983454342867 | 1197.10122929429 | 7265.20580440083 | Rwanda |
| 2001 | 8058.61572520493 | 0.286082054881284 | 1128.46100973787 | 6930.15471546706 | Rwanda |
| 2002 | 7645.11112368526 | 0.292955430049408 | 1070.28936735599 | 6574.82175632927 | Rwanda |
| 2003 | 7192.83566729921 | 0.299676918389748 | 1033.14631776435 | 6159.68934953486 | Rwanda |
| 2004 | 7267.0477518583 | 0.306943916198519 | 1024.42242486795 | 6242.62532699035 | Rwanda |
| 2005 | 6757.26169789237 | 0.314219577820047 | 961.724680432569 | 5795.5370174598 | Rwanda |
| 2006 | 6242.80685336199 | 0.321998519469469 | 833.375245246692 | 5409.4316081153 | Rwanda |
| 2007 | 5622.36498617512 | 0.329616590545695 | 741.356822475896 | 4881.00816369922 | Rwanda |
| 2008 | 5036.68824692862 | 0.337039226343398 | 675.547103729294 | 4361.14114319933 | Rwanda |
| 2009 | 4364.76385878688 | 0.344494632549039 | 598.661994402616 | 3766.10186438426 | Rwanda |
| 2010 | 4004.80452222462 | 0.351659415720748 | 597.944409259792 | 3406.86011296483 | Rwanda |
| 2011 | 3676.38178075263 | 0.358940339871084 | 599.623856967786 | 3076.75792378484 | Rwanda |
| 2012 | 3013.93279647716 | 0.366411820814214 | 574.333060124709 | 2439.59973635245 | Rwanda |
| 2013 | 2662.74652834499 | 0.373769682430802 | 551.255891472481 | 2111.49063687251 | Rwanda |
| 2014 | 2655.7099357919 | 0.381533179436292 | 503.354652325172 | 2152.35528346673 | Rwanda |
| 2015 | 2535.14816980777 | 0.389895414951459 | 508.070890210168 | 2027.0772795976 | Rwanda |
| 2016 | 2339.61947511884 | 0.39828795853881 | 459.534340659884 | 1880.08513445896 | Rwanda |
| 2017 | 2029.87004632309 | 0.406411474976626 | 361.93726742106 | 1667.93277890203 | Rwanda |
| 2018 | 1836.95562496337 | 0.414657911701104 | 269.966892581885 | 1566.98873238148 | Rwanda |
| 2019 | 1724.03077712391 | 0.42308672715334 | 245.244483376303 | 1478.78629374761 | Rwanda |
| 2020 | 1646.32882367792 | 0.429896595971404 | 204.462234442432 | 1441.86658923549 | Rwanda |
| 2021 | 1498.83615050432 | 0.435588705789772 | 182.613197419618 | 1316.2229530847 | Rwanda |
| 1990 | 128.95693206122 | 0.575526495579186 | 13.3780624182446 | 115.578869642975 | Seychelles |
| 1991 | 123.08824068457 | 0.582698331394814 | 13.3376229891805 | 109.750617695389 | Seychelles |
| 1992 | 117.48303081883 | 0.590052918893135 | 13.1847476442596 | 104.29828317457 | Seychelles |
| 1993 | 113.56309212913 | 0.598044193906834 | 13.3398182441209 | 100.223273885009 | Seychelles |
| 1994 | 106.61525021599 | 0.606041267633362 | 13.3165412949412 | 93.2987089210488 | Seychelles |
| 1995 | 102.07917033294 | 0.613853280633323 | 13.2163426169489 | 88.8628277159911 | Seychelles |
| 1996 | 96.79670527175 | 0.622135170108466 | 12.9069912926177 | 83.8897139791323 | Seychelles |
| 1997 | 91.57271939648 | 0.630697731713198 | 12.0991458900335 | 79.4735735064465 | Seychelles |
| 1998 | 88.98705022162 | 0.63843888230513 | 8.69621338148211 | 80.2908368401379 | Seychelles |
| 1999 | 88.74826338509 | 0.645339665687648 | 5.85525652360849 | 82.8930068614815 | Seychelles |
| 2000 | 88.51008592669 | 0.651551988453137 | 5.38222811216494 | 83.1278578145251 | Seychelles |
| 2001 | 87.63718694847 | 0.656490668328199 | 4.74801038808641 | 82.8891765603836 | Seychelles |
| 2002 | 87.06840870551 | 0.660907789985091 | 4.75217376031066 | 82.3162349451993 | Seychelles |
| 2003 | 86.39342336958 | 0.664162026621557 | 4.3901102502328 | 82.0033131193472 | Seychelles |
| 2004 | 86.5916560601 | 0.666461353259832 | 3.76629785510808 | 82.8253582049919 | Seychelles |
| 2005 | 86.07602110186 | 0.669264742237803 | 3.85039891172388 | 82.2256221901361 | Seychelles |
| 2006 | 84.79058596372 | 0.672149914351772 | 3.63735923104263 | 81.1532267326774 | Seychelles |
| 2007 | 158.24215194094 | 0.675077437552466 | 3.22545950624833 | 155.016692434692 | Seychelles |
| 2008 | 82.0276192101 | 0.676590946064689 | 3.04297287976289 | 78.9846463303371 | Seychelles |
| 2009 | 80.73629892909 | 0.67784702810111 | 2.86807924866899 | 77.868219680421 | Seychelles |
| 2010 | 80.07030901628 | 0.680163639820996 | 2.78152701744657 | 77.2887819988334 | Seychelles |
| 2011 | 78.83498279832 | 0.683681491395532 | 2.80024615006655 | 76.0347366482535 | Seychelles |
| 2012 | 77.67370231353 | 0.687629394362418 | 2.66372415195103 | 75.009978161579 | Seychelles |
| 2013 | 75.97626629721 | 0.692525733840497 | 2.42998928287606 | 73.5462770143339 | Seychelles |
| 2014 | 73.88686474656 | 0.697114588650508 | 2.44952274657045 | 71.4373419999895 | Seychelles |
| 2015 | 71.62556226003 | 0.701722819856481 | 2.34861377515471 | 69.2769484848753 | Seychelles |
| 2016 | 69.79299347133 | 0.706183622956323 | 2.27699991870539 | 67.5159935526246 | Seychelles |
| 2017 | 58.79358615158 | 0.711027281077533 | 2.24904330729877 | 56.5445428442812 | Seychelles |
| 2018 | 57.15126451823 | 0.716407819415594 | 2.18937472125798 | 54.961889796972 | Seychelles |
| 2019 | 55.36206107904 | 0.722073684746157 | 2.18079969830657 | 53.1812613807334 | Seychelles |
| 2020 | 53.20360218448 | 0.726663769455011 | 2.18137395182302 | 51.022228232657 | Seychelles |
| 2021 | 52.35933173283 | 0.730150774629382 | 2.16614698669906 | 50.1931847461309 | Seychelles |
| 1990 | 17391.280901794 | 0.0488485644444749 | 17391.280901794 | 0 | Somalia |
| 1991 | 17361.8382185886 | 0.0494223815231112 | 17361.8382185886 | 0 | Somalia |
| 1992 | 17788.3146394006 | 0.050115531337966 | 17459.3509214617 | 328.963717938947 | Somalia |
| 1993 | 17688.1174165594 | 0.0507979992557536 | 17393.9949796932 | 294.122436866226 | Somalia |
| 1994 | 18315.1458995033 | 0.0514716956452511 | 17397.9436232997 | 917.202276203614 | Somalia |
| 1995 | 16077.6567018275 | 0.052148606291336 | 16077.6567018275 | 0 | Somalia |
| 1996 | 15327.2012396845 | 0.052840548398447 | 15327.2012396845 | 0 | Somalia |
| 1997 | 14947.7602980604 | 0.0535709673179049 | 14947.7602980604 | 0 | Somalia |
| 1998 | 14532.3256054627 | 0.054321526804773 | 14532.3256054627 | 0 | Somalia |
| 1999 | 13854.5236136165 | 0.0550718380312836 | 13854.5236136165 | 0 | Somalia |
| 2000 | 13841.7426591114 | 0.0558420632221002 | 13841.7426591114 | 0 | Somalia |
| 2001 | 12644.591104587 | 0.0566431646698027 | 12644.591104587 | 0 | Somalia |
| 2002 | 11826.876955102 | 0.0574576117178968 | 11826.876955102 | 0 | Somalia |
| 2003 | 11052.946865471 | 0.0582721495971463 | 11052.946865471 | 0 | Somalia |
| 2004 | 10318.3916689618 | 0.059076945378295 | 10318.3916689618 | 0 | Somalia |
| 2005 | 9590.68272946183 | 0.0598985393277897 | 9590.68272946183 | 0 | Somalia |
| 2006 | 9094.89579666944 | 0.0607414841530955 | 9094.89579666944 | 0 | Somalia |
| 2007 | 9882.39407318744 | 0.0615981512915218 | 9348.0400235077 | 534.354049679736 | Somalia |
| 2008 | 9209.20927523463 | 0.062475386934885 | 9131.77111233563 | 77.4381628989995 | Somalia |
| 2009 | 8717.47547928431 | 0.0633822252221486 | 8717.47547928431 | 0 | Somalia |
| 2010 | 8251.14561071956 | 0.0643221275495221 | 8251.14561071956 | 0 | Somalia |
| 2011 | 8377.97637411669 | 0.065306811339105 | 8296.97521430004 | 81.001159816653 | Somalia |
| 2012 | 7963.07622506346 | 0.0663548789687034 | 7963.07622506346 | 0 | Somalia |
| 2013 | 7786.59261809511 | 0.0674594862548888 | 7786.59261809511 | 0 | Somalia |
| 2014 | 7437.09527377978 | 0.0686092985197366 | 7437.09527377978 | 0 | Somalia |
| 2015 | 7001.06122189414 | 0.0698115214335177 | 7001.06122189414 | 0 | Somalia |
| 2016 | 6901.92311075261 | 0.0710640470412588 | 6901.92311075261 | 0 | Somalia |
| 2017 | 6276.80035549278 | 0.0723347759051063 | 6276.80035549278 | 0 | Somalia |
| 2018 | 5909.5692171337 | 0.0736422483519594 | 5909.5692171337 | 0 | Somalia |
| 2019 | 5827.27817663392 | 0.0749798109966195 | 5827.27817663392 | 0 | Somalia |
| 2020 | 5476.39225315873 | 0.0763633695596333 | 5476.39225315873 | 0 | Somalia |
| 2021 | 5016.96021200913 | 0.0776881089723748 | 5016.96021200913 | 0 | Somalia |
| 1990 | 13541.5353921901 | 0.259306073677984 | 1415.86162687369 | 12125.6737653164 | United Republic of Tanzania |
| 1991 | 13194.6015607815 | 0.262416238461239 | 1375.7217443331 | 11818.8798164484 | United Republic of Tanzania |
| 1992 | 13932.7555345962 | 0.264989447606401 | 1367.82283812001 | 12564.9326964762 | United Republic of Tanzania |
| 1993 | 12527.0457439376 | 0.26725491530928 | 1269.7353167032 | 11257.3104272344 | United Republic of Tanzania |
| 1994 | 12319.0486991265 | 0.269417204337003 | 1231.06079953713 | 11087.9878995894 | United Republic of Tanzania |
| 1995 | 11863.6147352808 | 0.27207820236791 | 1234.9688206576 | 10628.6459146232 | United Republic of Tanzania |
| 1996 | 11283.953453428 | 0.2755079091009 | 1230.09253745865 | 10053.8609159693 | United Republic of Tanzania |
| 1997 | 11751.3349669956 | 0.279259183750542 | 1198.33646390255 | 10552.9985030931 | United Republic of Tanzania |
| 1998 | 10850.5383387023 | 0.283152610359192 | 1187.55836253276 | 9662.97997616954 | United Republic of Tanzania |
| 1999 | 9507.77490004948 | 0.287381679042618 | 1133.2719523917 | 8374.50294765779 | United Republic of Tanzania |
| 2000 | 8542.9218796402 | 0.291827264752142 | 1148.78091152938 | 7394.14096811082 | United Republic of Tanzania |
| 2001 | 7899.03354047021 | 0.296715645317522 | 1079.43221785683 | 6819.60132261338 | United Republic of Tanzania |
| 2002 | 7328.27251512503 | 0.301983995351723 | 1029.62932225715 | 6298.64319286788 | United Republic of Tanzania |
| 2003 | 6648.57654795617 | 0.307615488417032 | 966.222750907055 | 5682.35379704911 | United Republic of Tanzania |
| 2004 | 6118.81280850479 | 0.313772983484529 | 961.866947217914 | 5156.94586128688 | United Republic of Tanzania |
| 2005 | 5563.2344989486 | 0.320437339078038 | 844.750852942633 | 4718.48364600597 | United Republic of Tanzania |
| 2006 | 5452.41656826499 | 0.327234001890678 | 748.464732622458 | 4703.95183564253 | United Republic of Tanzania |
| 2007 | 5445.33641403797 | 0.334313236676657 | 671.032547743245 | 4774.30386629472 | United Republic of Tanzania |
| 2008 | 5438.91617461837 | 0.341352630337404 | 591.122273968305 | 4847.79390065007 | United Republic of Tanzania |
| 2009 | 5478.39287932289 | 0.348378034950913 | 592.63774906698 | 4885.75513025591 | United Republic of Tanzania |
| 2010 | 5270.69361780813 | 0.355567563218872 | 593.600355139647 | 4677.09326266848 | United Republic of Tanzania |
| 2011 | 5012.82289316409 | 0.363036319968136 | 574.655232398359 | 4438.16766076573 | United Republic of Tanzania |
| 2012 | 4817.753387825 | 0.370412488284105 | 576.342162637067 | 4241.41122518793 | United Republic of Tanzania |
| 2013 | 3923.04285100962 | 0.377963171371427 | 545.679503757254 | 3377.36334725237 | United Republic of Tanzania |
| 2014 | 3524.70989816014 | 0.385974579014964 | 518.116452929592 | 3006.59344523055 | United Republic of Tanzania |
| 2015 | 3493.00224966325 | 0.394277756847982 | 464.264572884707 | 3028.73767677854 | United Republic of Tanzania |
| 2016 | 3309.05623760166 | 0.402879307812708 | 393.214727408621 | 2915.84151019304 | United Republic of Tanzania |
| 2017 | 3045.59801782994 | 0.411824974100396 | 294.283087579627 | 2751.31493025031 | United Republic of Tanzania |
| 2018 | 2677.24960356118 | 0.420985364340548 | 244.46378477711 | 2432.78581878407 | United Republic of Tanzania |
| 2019 | 2422.98187419947 | 0.430225378925537 | 204.454635966466 | 2218.527238233 | United Republic of Tanzania |
| 2020 | 2350.41324035641 | 0.438747237740344 | 113.530526890852 | 2236.88271346556 | United Republic of Tanzania |
| 2021 | 2137.22278563274 | 0.446568273334685 | 75.3962984414053 | 2061.82648719133 | United Republic of Tanzania |
| 1990 | 5199.48162638861 | 0.18700109571602 | 2327.60588879526 | 2871.87573759335 | Uganda |
| 1991 | 5122.63155594378 | 0.188924156324117 | 2327.74804868895 | 2794.88350725482 | Uganda |
| 1992 | 4975.0324117263 | 0.191003433034892 | 2317.28223935513 | 2657.75017237117 | Uganda |
| 1993 | 4734.24690909627 | 0.193557455978809 | 2333.10483154878 | 2401.14207754749 | Uganda |
| 1994 | 4468.05510423168 | 0.197293192708182 | 2361.90073434778 | 2106.1543698839 | Uganda |
| 1995 | 4188.64333416794 | 0.201835423450881 | 2317.85794523838 | 1870.78538892956 | Uganda |
| 1996 | 3962.1103628367 | 0.207080730880773 | 2322.95330917982 | 1639.15705365688 | Uganda |
| 1997 | 3773.5300237136 | 0.213091696195112 | 2301.12356047991 | 1472.40646323369 | Uganda |
| 1998 | 3488.40971015702 | 0.219712441898334 | 2132.27379666056 | 1356.13591349646 | Uganda |
| 1999 | 3230.01864494905 | 0.226916944982192 | 2144.8341680835 | 1085.18447686555 | Uganda |
| 2000 | 2966.01848363718 | 0.234323225413513 | 1900.46699071004 | 1065.55149292714 | Uganda |
| 2001 | 2819.49502325938 | 0.242186388084657 | 1714.02077193423 | 1105.47425132515 | Uganda |
| 2002 | 2668.65402989539 | 0.250552388304413 | 1514.71553446508 | 1153.93849543031 | Uganda |
| 2003 | 2432.1652442592 | 0.259164882748285 | 1384.78094499965 | 1047.38429925955 | Uganda |
| 2004 | 2386.45144064885 | 0.268004611155769 | 1278.19378700391 | 1108.25765364494 | Uganda |
| 2005 | 2191.82699169256 | 0.277369192598006 | 1203.85974452103 | 987.967247171532 | Uganda |
| 2006 | 1978.23109683738 | 0.287030582587568 | 1130.66344746295 | 847.567649374432 | Uganda |
| 2007 | 1809.33461058729 | 0.296740420486269 | 1078.53683581596 | 730.797774771328 | Uganda |
| 2008 | 1660.37988743149 | 0.306748843313354 | 1014.64493122726 | 645.734956204231 | Uganda |
| 2009 | 1538.32577820997 | 0.316731152894585 | 834.671323129088 | 703.654455080882 | Uganda |
| 2010 | 1501.7552887719 | 0.326543661078545 | 759.554891883167 | 742.200396888733 | Uganda |
| 2011 | 1390.22406760153 | 0.336217715783435 | 673.960973310486 | 716.263094291044 | Uganda |
| 2012 | 1306.76788240549 | 0.345364515038614 | 602.89682914228 | 703.87105326321 | Uganda |
| 2013 | 1295.35266778701 | 0.354381615123395 | 589.163072581061 | 706.189595205949 | Uganda |
| 2014 | 1252.92880153272 | 0.363430903180067 | 574.651105625609 | 678.277695907111 | Uganda |
| 2015 | 1165.33559076257 | 0.372537985916872 | 544.061836411218 | 621.273754351352 | Uganda |
| 2016 | 1143.09100981779 | 0.381312226396987 | 513.613677639482 | 629.477332178308 | Uganda |
| 2017 | 982.78330941229 | 0.390082258680085 | 502.931356498878 | 479.851952913412 | Uganda |
| 2018 | 775.62409578684 | 0.399117776823962 | 454.109011987814 | 321.515083799026 | Uganda |
| 2019 | 678.48980249622 | 0.408431557149204 | 311.609380346681 | 366.880422149539 | Uganda |
| 2020 | 673.90254807595 | 0.416846079190348 | 258.557684530114 | 415.344863545836 | Uganda |
| 2021 | 613.52510417655 | 0.423261181495743 | 235.359918814765 | 378.165185361785 | Uganda |
| 1990 | 16903.178802863 | 0.304008549420206 | 1029.50424520799 | 15873.674557655 | Zambia |
| 1991 | 17599.1072811601 | 0.305842359512691 | 1013.55591023047 | 16585.5513709296 | Zambia |
| 1992 | 16983.0488501856 | 0.307316277250908 | 1028.28575573216 | 15954.7630944534 | Zambia |
| 1993 | 15935.1174748652 | 0.308780208280919 | 960.243040986796 | 14974.8744338784 | Zambia |
| 1994 | 15046.3423559408 | 0.309223449091581 | 968.285992588958 | 14078.0563633518 | Zambia |
| 1995 | 14572.3086824957 | 0.309995813177242 | 971.748100447869 | 13600.5605820478 | Zambia |
| 1996 | 14080.8710487444 | 0.311326690947126 | 957.877940554518 | 13122.9931081899 | Zambia |
| 1997 | 13597.0543100897 | 0.313087329617191 | 957.660575811268 | 12639.3937342784 | Zambia |
| 1998 | 13229.6385435273 | 0.314926434901042 | 846.060239155653 | 12383.5783043716 | Zambia |
| 1999 | 13371.9698903742 | 0.317396437417001 | 843.638001762629 | 12528.3318886116 | Zambia |
| 2000 | 12707.606969052 | 0.320625321501856 | 837.670152367658 | 11869.9368166843 | Zambia |
| 2001 | 15481.6126239742 | 0.324401368208402 | 755.615796040747 | 14725.9968279335 | Zambia |
| 2002 | 11247.127312232 | 0.328680121871397 | 740.762272463598 | 10506.3650397684 | Zambia |
| 2003 | 10901.8578965837 | 0.33372139842644 | 671.559673057515 | 10230.2982235262 | Zambia |
| 2004 | 11189.1947672816 | 0.339488916807796 | 584.675524399658 | 10604.5192428819 | Zambia |
| 2005 | 10607.298393481 | 0.346048863943135 | 588.777707522049 | 10018.520685959 | Zambia |
| 2006 | 10042.5728767736 | 0.353540709176488 | 591.037223279623 | 9451.53565349398 | Zambia |
| 2007 | 9219.15294603953 | 0.362450967280465 | 575.843622813454 | 8643.30932322608 | Zambia |
| 2008 | 8663.31087359656 | 0.371688495720519 | 535.848584808176 | 8127.46228878838 | Zambia |
| 2009 | 7780.39597313128 | 0.381667118197368 | 510.950390586146 | 7269.44558254513 | Zambia |
| 2010 | 6957.66292157149 | 0.392470988784596 | 467.004997488673 | 6490.65792408282 | Zambia |
| 2011 | 6253.84451714958 | 0.403475629468659 | 394.595341443134 | 5859.24917570645 | Zambia |
| 2012 | 4967.51347100002 | 0.414574501227966 | 273.930798411577 | 4693.58267258844 | Zambia |
| 2013 | 4473.79050107426 | 0.425492301821784 | 234.465122424033 | 4239.32537865023 | Zambia |
| 2014 | 3946.97033318696 | 0.436244005506199 | 183.124049166679 | 3763.84628402028 | Zambia |
| 2015 | 3543.01746068674 | 0.446744718544927 | 72.1973968159751 | 3470.82006387076 | Zambia |
| 2016 | 3171.38298558769 | 0.457122596045687 | 65.4137005151117 | 3105.96928507258 | Zambia |
| 2017 | 3046.81512816514 | 0.467532631330298 | 58.1494275347673 | 2988.66570063037 | Zambia |
| 2018 | 2671.39368061763 | 0.477887711662038 | 53.1495393523026 | 2618.24414126533 | Zambia |
| 2019 | 2423.81455496271 | 0.487911522977487 | 49.6897497171163 | 2374.12480524559 | Zambia |
| 2020 | 2179.001904384 | 0.49748556148019 | 26.392580026061 | 2152.60932435794 | Zambia |
| 2021 | 1893.72680768943 | 0.505948954434909 | 23.0730036666673 | 1870.65380402276 | Zambia |
| 1990 | 4060.38849830457 | 0.418077748220986 | 264.645892039456 | 3795.74260626511 | Botswana |
| 1991 | 3886.40935768568 | 0.42975228585408 | 205.519029521238 | 3680.89032816444 | Botswana |
| 1992 | 3726.34437914473 | 0.440490843191281 | 108.812355613541 | 3617.53202353119 | Botswana |
| 1993 | 3561.52280333068 | 0.450523613884069 | 75.1789508916812 | 3486.343852439 | Botswana |
| 1994 | 3467.58592221601 | 0.459954966910237 | 65.5650759101037 | 3402.02084630591 | Botswana |
| 1995 | 3385.84643822052 | 0.469266513316808 | 57.3877469995108 | 3328.45869122101 | Botswana |
| 1996 | 3312.66943200395 | 0.478588352164802 | 52.2585718231723 | 3260.41086018078 | Botswana |
| 1997 | 3262.21478809959 | 0.487767857070753 | 50.0268964245673 | 3212.18789167502 | Botswana |
| 1998 | 3388.07289580305 | 0.496193621706379 | 36.6772795186838 | 3351.39561628437 | Botswana |
| 1999 | 3384.1885047068 | 0.504978826176952 | 22.9164389033217 | 3361.27206580348 | Botswana |
| 2000 | 3344.2438940638 | 0.513498223414294 | 22.4726325940644 | 3321.77126146974 | Botswana |
| 2001 | 3300.96765620031 | 0.521370181612928 | 22.1463016443924 | 3278.82135455592 | Botswana |
| 2002 | 3234.28519616753 | 0.529252963791219 | 22.105648606371 | 3212.17954756116 | Botswana |
| 2003 | 3115.04860255032 | 0.537000179431652 | 21.3259968937198 | 3093.7226056566 | Botswana |
| 2004 | 2951.38789883671 | 0.544349222513256 | 21.1640172821278 | 2930.22388155458 | Botswana |
| 2005 | 2870.49515613642 | 0.551706969608463 | 19.6385363475207 | 2850.8566197889 | Botswana |
| 2006 | 5469.19099148403 | 0.559221620417772 | 17.9724942964002 | 5451.21849718763 | Botswana |
| 2007 | 2865.54082084717 | 0.566702496208929 | 15.2827781805547 | 2850.25804266661 | Botswana |
| 2008 | 2783.39127328558 | 0.57388380509654 | 13.4198097149444 | 2769.97146357064 | Botswana |
| 2009 | 2719.68480519801 | 0.579696411585388 | 13.4268829781534 | 2706.25792221986 | Botswana |
| 2010 | 2695.69257265216 | 0.585824150481272 | 13.3249381238734 | 2682.36763452829 | Botswana |
| 2011 | 2600.87285737989 | 0.591985456875054 | 13.4212174782627 | 2587.45163990163 | Botswana |
| 2012 | 2358.6323416531 | 0.597739552771288 | 13.2373347418412 | 2345.39500691126 | Botswana |
| 2013 | 2274.2224972722 | 0.604064730659858 | 13.2676903982842 | 2260.95480687392 | Botswana |
| 2014 | 2223.38167253365 | 0.610086048809866 | 13.4649396635295 | 2209.91673287012 | Botswana |
| 2015 | 2158.58430122785 | 0.615411123648458 | 12.9820219476201 | 2145.60227928023 | Botswana |
| 2016 | 2087.60052868968 | 0.620797574794607 | 12.9745248478737 | 2074.62600384181 | Botswana |
| 2017 | 2046.6452923435 | 0.625704953034907 | 12.9467146820276 | 2033.69857766147 | Botswana |
| 2018 | 1985.94531710197 | 0.630606840950645 | 11.9675922012637 | 1973.97772490071 | Botswana |
| 2019 | 1939.63210479048 | 0.635324093279727 | 10.9899692938314 | 1928.64213549665 | Botswana |
| 2020 | 1892.51151094671 | 0.639275298342726 | 7.7047376824446 | 1884.80677326427 | Botswana |
| 2021 | 1858.39721067235 | 0.642721628921122 | 7.55503499380383 | 1850.84217567855 | Botswana |
| 1990 | 7901.56429088604 | 0.339155125342909 | 597.385241875523 | 7304.17904901052 | Lesotho |
| 1991 | 7803.82522850847 | 0.34549657132085 | 594.211422355634 | 7209.61380615284 | Lesotho |
| 1992 | 7563.11115718847 | 0.352169629526247 | 591.863088121683 | 6971.24806906679 | Lesotho |
| 1993 | 7263.84342957787 | 0.358780457204597 | 590.030779366936 | 6673.81265021093 | Lesotho |
| 1994 | 6927.35070007611 | 0.365310801307453 | 573.47077902399 | 6353.87992105212 | Lesotho |
| 1995 | 6520.69926823985 | 0.3712653154353 | 541.943688881724 | 5978.75557935813 | Lesotho |
| 1996 | 6238.01075517631 | 0.377221201711086 | 542.454868506865 | 5695.55588666945 | Lesotho |
| 1997 | 6128.60513689703 | 0.383096647768047 | 512.437756432753 | 5616.16738046428 | Lesotho |
| 1998 | 6069.97712967997 | 0.388406478488836 | 510.341563348401 | 5559.63556633157 | Lesotho |
| 1999 | 6212.64363868869 | 0.393495000758155 | 467.868304995699 | 5744.77533369299 | Lesotho |
| 2000 | 6341.71256064549 | 0.398635802456027 | 461.47556106535 | 5880.23699958014 | Lesotho |
| 2001 | 6501.73813929634 | 0.403990126208872 | 396.836120750116 | 6104.90201854622 | Lesotho |
| 2002 | 6569.04419830432 | 0.409021656522558 | 314.082061034997 | 6254.96213726932 | Lesotho |
| 2003 | 6539.99441477404 | 0.413982570645541 | 276.009074923767 | 6263.98533985027 | Lesotho |
| 2004 | 6593.2651220049 | 0.418670918560125 | 266.911706826887 | 6326.35341517801 | Lesotho |
| 2005 | 6515.76968019011 | 0.423211227878272 | 232.362750910842 | 6283.40692927927 | Lesotho |
| 2006 | 6396.25480044771 | 0.427806731919536 | 215.031011171646 | 6181.22378927606 | Lesotho |
| 2007 | 6483.08915603207 | 0.432745038329554 | 207.968347193788 | 6275.12080883828 | Lesotho |
| 2008 | 6552.53051624469 | 0.438340567457201 | 114.143944561377 | 6438.38657168331 | Lesotho |
| 2009 | 6039.82127400612 | 0.443580126941483 | 112.203222130595 | 5927.61805187553 | Lesotho |
| 2010 | 5668.74268504168 | 0.44896647618497 | 76.0921338923397 | 5592.65055114934 | Lesotho |
| 2011 | 5701.19756468607 | 0.454530098376785 | 70.6866015284394 | 5630.51096315763 | Lesotho |
| 2012 | 5447.70595645335 | 0.460419689024454 | 62.642777914228 | 5385.06317853912 | Lesotho |
| 2013 | 4790.58792031436 | 0.466692255685783 | 57.1626524996899 | 4733.42526781467 | Lesotho |
| 2014 | 4521.87823386155 | 0.473188157042242 | 58.3466336466382 | 4463.53160021491 | Lesotho |
| 2015 | 4553.49343093768 | 0.479754456396731 | 54.224881740176 | 4499.2685491975 | Lesotho |
| 2016 | 4505.86345967755 | 0.486144826981745 | 53.5114667940625 | 4452.35199288349 | Lesotho |
| 2017 | 4356.66532712681 | 0.491978154790894 | 50.3595479640205 | 4306.30577916279 | Lesotho |
| 2018 | 4332.00225197991 | 0.497381790902339 | 26.9457334624497 | 4305.05651851746 | Lesotho |
| 2019 | 4314.11179169645 | 0.502457316539537 | 23.3418562987032 | 4290.76993539775 | Lesotho |
| 2020 | 4314.59711673817 | 0.50674665490739 | 22.8248569793093 | 4291.77225975886 | Lesotho |
| 2021 | 4294.2918653449 | 0.510393065681381 | 22.6025275302403 | 4271.68933781466 | Lesotho |
| 1990 | 5159.7572118768 | 0.45004023335046 | 76.9634135331164 | 5082.79379834368 | Namibia |
| 1991 | 4946.70526555829 | 0.454534440388721 | 74.9827829473035 | 4871.72248261099 | Namibia |
| 1992 | 4710.67524958316 | 0.460021313919932 | 62.1847099501543 | 4648.490539633 | Namibia |
| 1993 | 4459.73234425964 | 0.465734134805029 | 62.9613861103764 | 4396.77095814926 | Namibia |
| 1994 | 4184.41274511372 | 0.47187321693073 | 57.4513270072889 | 4126.96141810643 | Namibia |
| 1995 | 3910.49510116942 | 0.477794328919676 | 53.6831968152549 | 3856.81190435417 | Namibia |
| 1996 | 3702.33712509833 | 0.483116378270553 | 53.9745494152203 | 3648.36257568311 | Namibia |
| 1997 | 3578.97073592493 | 0.488012938242902 | 50.2717264923426 | 3528.69900943259 | Namibia |
| 1998 | 3671.17640970681 | 0.492812548000801 | 50.3333056563849 | 3620.84310405043 | Namibia |
| 1999 | 3665.71719782862 | 0.49761386200554 | 26.4319937552283 | 3639.28520407339 | Namibia |
| 2000 | 3659.1633718152 | 0.502406957891221 | 23.4947974798456 | 3635.66857433535 | Namibia |
| 2001 | 3573.37898085585 | 0.506719032722361 | 22.7086473992178 | 3550.67033345663 | Namibia |
| 2002 | 3593.69315599645 | 0.510818274709975 | 22.4692319190393 | 3571.22392407741 | Namibia |
| 2003 | 3548.01184959038 | 0.514574645155077 | 22.5197219660266 | 3525.49212762435 | Namibia |
| 2004 | 3459.02355304341 | 0.51910360486724 | 22.2143281100688 | 3436.80922493334 | Namibia |
| 2005 | 3479.5928955982 | 0.523881735538067 | 22.1696068050832 | 3457.42328879312 | Namibia |
| 2006 | 3451.67768725179 | 0.529350117774113 | 22.1587323569481 | 3429.51895489484 | Namibia |
| 2007 | 3529.75151340918 | 0.535062125820852 | 21.5476378610984 | 3508.20387554808 | Namibia |
| 2008 | 3509.68343191152 | 0.54089699471582 | 21.1733112595878 | 3488.51012065193 | Namibia |
| 2009 | 3375.59646567295 | 0.546516477315561 | 20.5307778154299 | 3355.06568785752 | Namibia |
| 2010 | 3203.75811702659 | 0.552741565170742 | 19.8393920373974 | 3183.91872498919 | Namibia |
| 2011 | 3041.7489528805 | 0.559618899481539 | 17.4457420219494 | 3024.30321085855 | Namibia |
| 2012 | 2796.53738852933 | 0.566978697200188 | 14.4899177754885 | 2782.04747075384 | Namibia |
| 2013 | 2747.78778560283 | 0.574590344212504 | 13.2840976787265 | 2734.5036879241 | Namibia |
| 2014 | 2443.7930132595 | 0.582321424930035 | 13.4188059947934 | 2430.37420726471 | Namibia |
| 2015 | 2238.37579706688 | 0.589821040312457 | 13.3324529285331 | 2225.04334413835 | Namibia |
| 2016 | 2099.88920201817 | 0.596283059335379 | 13.2964320861001 | 2086.59276993207 | Namibia |
| 2017 | 1986.77125517161 | 0.601754644588282 | 13.5314954718781 | 1973.23975969973 | Namibia |
| 2018 | 1926.21934254132 | 0.606618403183865 | 13.4087305267131 | 1912.81061201461 | Namibia |
| 2019 | 1872.27301975889 | 0.610921091420642 | 13.3582868819405 | 1858.91473287695 | Namibia |
| 2020 | 1821.69934064982 | 0.614435889089058 | 13.3433175627066 | 1808.35602308711 | Namibia |
| 2021 | 1760.24893834372 | 0.617564872149664 | 13.074046473675 | 1747.17489187005 | Namibia |
| 1990 | 7206.75853235356 | 0.541571435204427 | 21.1596812460546 | 7185.59885110751 | South Africa |
| 1991 | 6863.97336511269 | 0.546921135754198 | 20.6292995415488 | 6843.34406557114 | South Africa |
| 1992 | 6572.61636235937 | 0.552066898744688 | 19.8031601093874 | 6552.81320224998 | South Africa |
| 1993 | 6300.40179303237 | 0.557198439031159 | 18.6082983304163 | 6281.79349470195 | South Africa |
| 1994 | 6086.05913811725 | 0.562448636018678 | 16.7877434235689 | 6069.27139469368 | South Africa |
| 1995 | 5866.26889651478 | 0.567905303801023 | 14.6801158236676 | 5851.58878069111 | South Africa |
| 1996 | 5646.8755094643 | 0.573453987768516 | 13.4686359222964 | 5633.406873542 | South Africa |
| 1997 | 5557.24384029114 | 0.57909927788854 | 13.2998213634206 | 5543.94401892772 | South Africa |
| 1998 | 5528.53331943992 | 0.58443731296776 | 13.3343773792437 | 5515.19894206068 | South Africa |
| 1999 | 5469.40983411658 | 0.589407348753061 | 13.2930476680447 | 5456.11678644854 | South Africa |
| 2000 | 5528.44863301129 | 0.594093682936262 | 13.3622594827618 | 5515.08637352853 | South Africa |
| 2001 | 5527.87788830805 | 0.597884168238822 | 13.3076468819911 | 5514.57024142606 | South Africa |
| 2002 | 5510.21041739155 | 0.600921675691185 | 13.1734372299204 | 5497.03698016163 | South Africa |
| 2003 | 5365.60436279347 | 0.603515602003029 | 13.3446664059382 | 5352.25969638753 | South Africa |
| 2004 | 5253.20439175085 | 0.606262513759854 | 13.1869693391565 | 5240.01742241169 | South Africa |
| 2005 | 5153.03901333072 | 0.609989979082156 | 13.5201849903616 | 5139.51882834036 | South Africa |
| 2006 | 5063.51547040044 | 0.615014689925222 | 12.989877877165 | 5050.52559252328 | South Africa |
| 2007 | 5094.51806336886 | 0.620608149811677 | 12.9626569082821 | 5081.55540646058 | South Africa |
| 2008 | 5152.80274046291 | 0.625948031125006 | 12.9451859685423 | 5139.85755449437 | South Africa |
| 2009 | 4624.84801129507 | 0.630645626147581 | 11.9432472482703 | 4612.9047640468 | South Africa |
| 2010 | 4408.64038436487 | 0.635268538092523 | 11.0739263151261 | 4397.56645804974 | South Africa |
| 2011 | 4178.94942637423 | 0.639991722232976 | 7.82074375951595 | 4171.12868261471 | South Africa |
| 2012 | 3893.54462792704 | 0.644715660880732 | 6.31780164203903 | 3887.226826285 | South Africa |
| 2013 | 3547.19725104554 | 0.64943055353059 | 5.62919185183304 | 3541.56805919371 | South Africa |
| 2014 | 3343.40130160343 | 0.653964494596397 | 4.87154184928534 | 3338.52975975414 | South Africa |
| 2015 | 3132.30436045763 | 0.658417761080757 | 5.07338234620651 | 3127.23097811142 | South Africa |
| 2016 | 2838.45807484927 | 0.662642486535973 | 4.63394050695261 | 2833.82413434232 | South Africa |
| 2017 | 2554.27162254619 | 0.666622608224758 | 3.81550663462706 | 2550.45611591156 | South Africa |
| 2018 | 2332.26055208568 | 0.670335365040043 | 3.73887047517253 | 2328.52168161051 | South Africa |
| 2019 | 2195.33736511847 | 0.674041036954557 | 3.34267349535576 | 2191.99469162311 | South Africa |
| 2020 | 1857.65119171175 | 0.677166096009468 | 2.79948824139416 | 1854.85170347036 | South Africa |
| 2021 | 1808.27845733696 | 0.679626598156588 | 2.92188964432622 | 1805.35656769263 | South Africa |
| 1990 | 7226.94932882448 | 0.399420954712507 | 458.951423283668 | 6767.99790554081 | Eswatini |
| 1991 | 7013.36044079552 | 0.408269968877275 | 308.38214561535 | 6704.97829518017 | Eswatini |
| 1992 | 7115.02884476837 | 0.416683379093578 | 269.848724481695 | 6845.18012028667 | Eswatini |
| 1993 | 6708.36184644735 | 0.424802994934769 | 235.020335552067 | 6473.34151089528 | Eswatini |
| 1994 | 6578.94275288819 | 0.432503472188562 | 205.983117137258 | 6372.95963575093 | Eswatini |
| 1995 | 6436.2895635303 | 0.440127046710174 | 115.881105359054 | 6320.40845817125 | Eswatini |
| 1996 | 6298.97980330671 | 0.447399238142412 | 74.2575325632591 | 6224.72227074345 | Eswatini |
| 1997 | 6181.87365140052 | 0.454089854339089 | 72.7016331739147 | 6109.1720182266 | Eswatini |
| 1998 | 6104.37808636287 | 0.46013698389181 | 68.0385371354246 | 6036.33954922744 | Eswatini |
| 1999 | 6175.36004941775 | 0.465990845350804 | 64.5276782750228 | 6110.83237114273 | Eswatini |
| 2000 | 6463.95347328335 | 0.471852843205366 | 56.9287639078609 | 6407.02470937549 | Eswatini |
| 2001 | 7130.70012781535 | 0.477233374481226 | 52.9585676063626 | 7077.74156020899 | Eswatini |
| 2002 | 6317.18103110879 | 0.482474423324763 | 55.3039052907395 | 6261.87712581805 | Eswatini |
| 2003 | 6415.1701078771 | 0.487608829048368 | 49.5916174805722 | 6365.57849039653 | Eswatini |
| 2004 | 6388.23573555439 | 0.492669158763782 | 49.7144913430087 | 6338.52124421138 | Eswatini |
| 2005 | 6012.15201963226 | 0.498043848851584 | 24.8589197164946 | 5987.29309991577 | Eswatini |
| 2006 | 5751.62089672169 | 0.503658293748865 | 23.0960564456716 | 5728.52484027602 | Eswatini |
| 2007 | 5612.20889246698 | 0.509260218872045 | 22.7594778141888 | 5589.44941465279 | Eswatini |
| 2008 | 5387.40342190957 | 0.514730895784455 | 22.4884647187637 | 5364.91495719081 | Eswatini |
| 2009 | 5091.16714931014 | 0.520427398115377 | 22.1924728998493 | 5068.97467641029 | Eswatini |
| 2010 | 4618.6555088526 | 0.526245801589325 | 22.1308994555028 | 4596.5246093971 | Eswatini |
| 2011 | 4228.67984565741 | 0.531913910153764 | 22.1822802454891 | 4206.49756541192 | Eswatini |
| 2012 | 3960.6999267456 | 0.537798022788039 | 21.3175413308915 | 3939.38238541471 | Eswatini |
| 2013 | 3672.97556907185 | 0.543836416273961 | 21.1577744214789 | 3651.81779465037 | Eswatini |
| 2014 | 3376.78567258418 | 0.549613848251705 | 19.7902233041709 | 3356.99544928001 | Eswatini |
| 2015 | 2842.77654672123 | 0.555373738956414 | 18.9168751722666 | 2823.85967154896 | Eswatini |
| 2016 | 2713.44222667079 | 0.561061185783082 | 16.8944399120629 | 2696.54778675873 | Eswatini |
| 2017 | 2573.23638895782 | 0.566698492780748 | 15.1910647192968 | 2558.04532423852 | Eswatini |
| 2018 | 2365.59349720855 | 0.572154807441339 | 13.6048553843757 | 2351.98864182417 | Eswatini |
| 2019 | 2264.73300793567 | 0.577206305034792 | 13.2265132823321 | 2251.50649465334 | Eswatini |
| 2020 | 2157.99468484481 | 0.581653071099534 | 13.2103900179153 | 2144.78429482689 | Eswatini |
| 2021 | 2037.97188170298 | 0.58545971329224 | 13.2795706401348 | 2024.69231106285 | Eswatini |
| 1990 | 1585.61163048049 | 0.398559340611047 | 458.283891743237 | 1127.32773873725 | Zimbabwe |
| 1991 | 1509.85317841957 | 0.406052876694226 | 367.492824374016 | 1142.36035404555 | Zimbabwe |
| 1992 | 1628.38564198915 | 0.412083825921141 | 302.344388026241 | 1326.04125396291 | Zimbabwe |
| 1993 | 1591.22587614598 | 0.417974200219438 | 260.20472232685 | 1331.02115381913 | Zimbabwe |
| 1994 | 1436.96558469011 | 0.424051564461987 | 231.632036357397 | 1205.33354833271 | Zimbabwe |
| 1995 | 1375.6010729328 | 0.428929040100791 | 214.537662647445 | 1161.06341028535 | Zimbabwe |
| 1996 | 1301.62164025839 | 0.43418719166794 | 204.941797264808 | 1096.67984299358 | Zimbabwe |
| 1997 | 1302.90143708769 | 0.438141987585594 | 113.99709512104 | 1188.90434196665 | Zimbabwe |
| 1998 | 1333.88760322517 | 0.441093134678486 | 108.578686637515 | 1225.30891658765 | Zimbabwe |
| 1999 | 1454.19908779202 | 0.443246603614544 | 110.219340467332 | 1343.97974732469 | Zimbabwe |
| 2000 | 1550.69162800718 | 0.444841401738061 | 100.337914490862 | 1450.35371351632 | Zimbabwe |
| 2001 | 1570.48577405433 | 0.446354680553508 | 77.2619577038795 | 1493.22381635045 | Zimbabwe |
| 2002 | 1718.29977459307 | 0.446970895206592 | 81.2019505345873 | 1637.09782405848 | Zimbabwe |
| 2003 | 1701.2682889402 | 0.446002181962281 | 75.782107656719 | 1625.48618128348 | Zimbabwe |
| 2004 | 1702.19097722758 | 0.443696404187693 | 108.018998633836 | 1594.17197859374 | Zimbabwe |
| 2005 | 1812.93205738056 | 0.439651656979407 | 120.36314116948 | 1692.56891621108 | Zimbabwe |
| 2006 | 1895.91541579959 | 0.434405758403472 | 204.88334146001 | 1691.03207433958 | Zimbabwe |
| 2007 | 1801.05969039138 | 0.429165139621798 | 217.96972705731 | 1583.08996333407 | Zimbabwe |
| 2008 | 2948.42097830308 | 0.422448974219341 | 248.077852694657 | 2700.34312560842 | Zimbabwe |
| 2009 | 3293.49993698788 | 0.418330613224402 | 257.207007144885 | 3036.292929843 | Zimbabwe |
| 2010 | 1973.54677448587 | 0.417305356749818 | 258.671583101588 | 1714.87519138428 | Zimbabwe |
| 2011 | 2035.47584900181 | 0.419729694705381 | 259.219636524933 | 1776.25621247688 | Zimbabwe |
| 2012 | 1898.42808913731 | 0.425748519814194 | 234.750824842028 | 1663.67726429528 | Zimbabwe |
| 2013 | 1689.11227715278 | 0.432881888218656 | 203.392789685004 | 1485.71948746778 | Zimbabwe |
| 2014 | 1382.97744417303 | 0.439997226175148 | 117.973578933409 | 1265.00386523962 | Zimbabwe |
| 2015 | 1290.5119410419 | 0.446552872040041 | 73.2146575082463 | 1217.29728353365 | Zimbabwe |
| 2016 | 1266.0886755112 | 0.452473036911982 | 73.8338521106954 | 1192.2548234005 | Zimbabwe |
| 2017 | 1130.94946426453 | 0.458424984120402 | 69.6384340497843 | 1061.31103021475 | Zimbabwe |
| 2018 | 1147.86673346827 | 0.464379262217529 | 65.0650143546769 | 1082.80171911359 | Zimbabwe |
| 2019 | 1093.18827592337 | 0.46838443787862 | 57.9732764034447 | 1035.21499951993 | Zimbabwe |
| 2020 | 1041.30901723783 | 0.471573170735407 | 57.6340979329865 | 983.674919304843 | Zimbabwe |
| 2021 | 1095.58625929929 | 0.473819486202707 | 56.7914391381862 | 1038.7948201611 | Zimbabwe |
| 1990 | 10186.2486009439 | 0.218907153827645 | 2305.45085433168 | 7880.79774661222 | Benin |
| 1991 | 10399.5080910351 | 0.222539019363851 | 2139.06356574757 | 8260.44452528753 | Benin |
| 1992 | 9678.48686211013 | 0.226323465232837 | 2131.85182619478 | 7546.63503591535 | Benin |
| 1993 | 9328.89663733734 | 0.230312593690675 | 1943.93921281259 | 7384.95742452475 | Benin |
| 1994 | 9021.0949878255 | 0.234032035653662 | 1903.52343936151 | 7117.57154846399 | Benin |
| 1995 | 8615.86885044254 | 0.238009696338161 | 1674.0216518515 | 6941.84719859104 | Benin |
| 1996 | 8576.82585868182 | 0.241895322542988 | 1657.9711852401 | 6918.85467344172 | Benin |
| 1997 | 7855.29966227795 | 0.245731534038315 | 1691.43025357046 | 6163.86940870749 | Benin |
| 1998 | 7423.22243937897 | 0.249376450881329 | 1523.94862951591 | 5899.27380986306 | Benin |
| 1999 | 7104.02370431508 | 0.253215340715821 | 1517.79992205012 | 5586.22378226496 | Benin |
| 2000 | 6789.59091094102 | 0.257380681388323 | 1383.08148962666 | 5406.50942131436 | Benin |
| 2001 | 6385.95778864646 | 0.261388010509781 | 1378.6302633419 | 5007.32752530456 | Benin |
| 2002 | 6209.29331449338 | 0.265417457761242 | 1395.99074045478 | 4813.3025740386 | Benin |
| 2003 | 5783.49695483736 | 0.269544281064264 | 1238.94108570969 | 4544.55586912767 | Benin |
| 2004 | 5633.18450767389 | 0.273941144522725 | 1232.72023739812 | 4400.46427027577 | Benin |
| 2005 | 5265.96647383064 | 0.278406750274251 | 1193.13642943758 | 4072.83004439306 | Benin |
| 2006 | 5048.48368119065 | 0.282933935672546 | 1192.42139464277 | 3856.06228654788 | Benin |
| 2007 | 4697.64044377741 | 0.287673054621831 | 1135.6422115436 | 3561.99823223381 | Benin |
| 2008 | 4426.05825094579 | 0.292627446283673 | 1072.69881558798 | 3353.35943535781 | Benin |
| 2009 | 4236.26294533197 | 0.297702227030519 | 1084.15619653349 | 3152.10674879848 | Benin |
| 2010 | 4089.34457293939 | 0.302809008308589 | 1021.28483370718 | 3068.05973923221 | Benin |
| 2011 | 3838.84775449666 | 0.30792553783669 | 964.503833825392 | 2874.34392067127 | Benin |
| 2012 | 3747.26318883598 | 0.313373588474229 | 968.212648018279 | 2779.0505408177 | Benin |
| 2013 | 3527.5748338457 | 0.319318688191374 | 847.540222958508 | 2680.03461088719 | Benin |
| 2014 | 3212.95719958604 | 0.325727271772744 | 759.383320483136 | 2453.5738791029 | Benin |
| 2015 | 3132.56308310551 | 0.332216350180226 | 684.081684150949 | 2448.48139895456 | Benin |
| 2016 | 2918.53087709462 | 0.338763921416219 | 593.800844711546 | 2324.73003238307 | Benin |
| 2017 | 2682.38584915325 | 0.345606758793044 | 594.5133966676 | 2087.87245248565 | Benin |
| 2018 | 2335.82941722182 | 0.352652695728689 | 594.373449038158 | 1741.45596818366 | Benin |
| 2019 | 1879.73955085596 | 0.360031163133997 | 595.493111739049 | 1284.24643911691 | Benin |
| 2020 | 1854.87981877023 | 0.36696448721315 | 572.304886325869 | 1282.57493244436 | Benin |
| 2021 | 1670.45124786349 | 0.373486574239249 | 544.424493451 | 1126.02675441249 | Benin |
| 1990 | 10085.7674598152 | 0.129695615380271 | 5064.64473305319 | 5021.12272676201 | Burkina Faso |
| 1991 | 9710.9667327779 | 0.133299127658948 | 4877.1492242188 | 4833.8175085591 | Burkina Faso |
| 1992 | 9366.54870418616 | 0.136594798422558 | 4566.53791769714 | 4800.01078648902 | Burkina Faso |
| 1993 | 9084.62460155956 | 0.139825464930799 | 4345.64358171678 | 4738.98101984278 | Burkina Faso |
| 1994 | 8835.93570560804 | 0.142848972509425 | 3877.16867889218 | 4958.76702671586 | Burkina Faso |
| 1995 | 8642.53585753462 | 0.146115672699206 | 3468.38443913463 | 5174.15141839999 | Burkina Faso |
| 1996 | 8339.82841796078 | 0.150041686384544 | 3221.06967950663 | 5118.75873845415 | Burkina Faso |
| 1997 | 8026.48619625464 | 0.154228401481324 | 3064.5577803978 | 4961.92841585684 | Burkina Faso |
| 1998 | 7732.50818217627 | 0.158707048449452 | 2827.8540715698 | 4904.65411060647 | Burkina Faso |
| 1999 | 7588.4340906208 | 0.163468066858732 | 2671.40033460845 | 4917.03375601235 | Burkina Faso |
| 2000 | 7776.96613287953 | 0.168084340000467 | 2334.65698153036 | 5442.30915134917 | Burkina Faso |
| 2001 | 7296.26244568335 | 0.172996395327051 | 2349.78326564605 | 4946.4791800373 | Burkina Faso |
| 2002 | 6956.92302846618 | 0.17798198994173 | 2343.44901988236 | 4613.47400858382 | Burkina Faso |
| 2003 | 6266.99403511411 | 0.183253539277265 | 2333.91123765223 | 3933.08279746188 | Burkina Faso |
| 2004 | 5740.14479661911 | 0.188564995004729 | 2339.09137537705 | 3401.05342124206 | Burkina Faso |
| 2005 | 5408.39977307539 | 0.194086128180827 | 2331.74917930936 | 3076.65059376603 | Burkina Faso |
| 2006 | 5151.42954896335 | 0.199571952068224 | 2339.75069051141 | 2811.67885845194 | Burkina Faso |
| 2007 | 4673.0451232199 | 0.204891092363641 | 2341.69026242121 | 2331.35486079869 | Burkina Faso |
| 2008 | 4435.32899512099 | 0.210223294804678 | 2340.34789924366 | 2094.98109587733 | Burkina Faso |
| 2009 | 4145.28870050125 | 0.215423373997381 | 2285.82606852891 | 1859.46263197234 | Burkina Faso |
| 2010 | 3969.57238634739 | 0.221001030335074 | 2153.27148790573 | 1816.30089844166 | Burkina Faso |
| 2011 | 3845.29062174416 | 0.226696631667163 | 2137.34542757262 | 1707.94519417154 | Burkina Faso |
| 2012 | 3808.09258815636 | 0.23258913471079 | 1904.88163571578 | 1903.21095244058 | Burkina Faso |
| 2013 | 3322.01146371376 | 0.238497491410915 | 1731.04616164796 | 1590.9653020658 | Burkina Faso |
| 2014 | 3183.8604273391 | 0.244378901166049 | 1698.38442201821 | 1485.47600532089 | Burkina Faso |
| 2015 | 3207.13510688147 | 0.250208260823773 | 1514.2391750243 | 1692.89593185717 | Burkina Faso |
| 2016 | 3199.13782611132 | 0.256081773936025 | 1516.91834614161 | 1682.21947996971 | Burkina Faso |
| 2017 | 3062.1247728571 | 0.26203300853891 | 1405.80068270668 | 1656.32409015042 | Burkina Faso |
| 2018 | 2824.94940571899 | 0.268102072864973 | 1267.6441156915 | 1557.30529002749 | Burkina Faso |
| 2019 | 2648.44634106863 | 0.274287092352859 | 1242.51095547357 | 1405.93538559506 | Burkina Faso |
| 2020 | 2446.84501938052 | 0.279871317215145 | 1210.44774765178 | 1236.39727172874 | Burkina Faso |
| 2021 | 2300.98854973736 | 0.285118401552141 | 1136.14042655025 | 1164.84812318711 | Burkina Faso |
| 1990 | 7443.97311422222 | 0.303055334404352 | 1023.60588344732 | 6420.3672307749 | Cameroon |
| 1991 | 7544.89873113091 | 0.30962345806122 | 958.897573207962 | 6586.00115792295 | Cameroon |
| 1992 | 7289.64884686456 | 0.315601268856928 | 834.711242264364 | 6454.9376046002 | Cameroon |
| 1993 | 7210.73143461357 | 0.320831654317412 | 851.132086989203 | 6359.59934762437 | Cameroon |
| 1994 | 7159.18299734059 | 0.325410122747214 | 759.026355741471 | 6400.15664159912 | Cameroon |
| 1995 | 6999.90397833565 | 0.329539317887926 | 741.696172106803 | 6258.20780622885 | Cameroon |
| 1996 | 7168.41028418204 | 0.333425981814059 | 689.214203732849 | 6479.19608044919 | Cameroon |
| 1997 | 6909.86715368176 | 0.337346000940944 | 598.077658753917 | 6311.78949492784 | Cameroon |
| 1998 | 6736.02510257938 | 0.341197089780886 | 590.855063514453 | 6145.17003906493 | Cameroon |
| 1999 | 6582.47352900655 | 0.344955364212408 | 590.480762577645 | 5991.9927664289 | Cameroon |
| 2000 | 6627.77287719777 | 0.348814788893637 | 593.377263784251 | 6034.39561341352 | Cameroon |
| 2001 | 6459.2291882702 | 0.352631872778337 | 590.366515217991 | 5868.86267305221 | Cameroon |
| 2002 | 6920.33244186999 | 0.356511816668113 | 585.963918066177 | 6334.36852380381 | Cameroon |
| 2003 | 6797.85262117216 | 0.360668581210006 | 586.61869322264 | 6211.23392794952 | Cameroon |
| 2004 | 6570.49401099784 | 0.365100029903911 | 576.762641252125 | 5993.73136974571 | Cameroon |
| 2005 | 6388.76479055756 | 0.369545756449213 | 576.515758060412 | 5812.24903249715 | Cameroon |
| 2006 | 6235.43872328478 | 0.374355877714557 | 550.612241884069 | 5684.82648140071 | Cameroon |
| 2007 | 5841.09204028735 | 0.379490349180985 | 542.2212470577 | 5298.87079322965 | Cameroon |
| 2008 | 5584.7911151682 | 0.38483830406879 | 513.501912284524 | 5071.28920288368 | Cameroon |
| 2009 | 5455.62778595747 | 0.390288642984762 | 509.592285403831 | 4946.03550055364 | Cameroon |
| 2010 | 5487.10319613857 | 0.396067145154693 | 463.859470727729 | 5023.24372541084 | Cameroon |
| 2011 | 5157.13250316931 | 0.402092798748116 | 394.315101371802 | 4762.81740179751 | Cameroon |
| 2012 | 4475.86834001177 | 0.408580122601571 | 314.47673391143 | 4161.39160610034 | Cameroon |
| 2013 | 4160.25497951761 | 0.415607964760584 | 277.378070067475 | 3882.87690945014 | Cameroon |
| 2014 | 3546.68880102268 | 0.423033560011266 | 245.765745998179 | 3300.9230550245 | Cameroon |
| 2015 | 3111.87923290496 | 0.430830276458225 | 210.610254770436 | 2901.26897813452 | Cameroon |
| 2016 | 2981.77102165736 | 0.438878945356817 | 124.830655484648 | 2856.94036617271 | Cameroon |
| 2017 | 2700.00832277543 | 0.447185100165971 | 73.3692711191138 | 2626.63905165632 | Cameroon |
| 2018 | 2496.77505119444 | 0.455687846771621 | 72.5255942627854 | 2424.24945693165 | Cameroon |
| 2019 | 2215.32059242546 | 0.464246472572462 | 64.9226368726395 | 2150.39795555282 | Cameroon |
| 2020 | 2013.08106140279 | 0.472285846440991 | 55.2986114597299 | 1957.78244994306 | Cameroon |
| 2021 | 1773.67121235877 | 0.479691223419746 | 55.1626359789939 | 1718.50857637978 | Cameroon |
| 1990 | 5279.81008604809 | 0.27672336711542 | 1201.51958322356 | 4078.29050282453 | Cabo Verde |
| 1991 | 4891.79515954677 | 0.282931721669337 | 1215.68519615044 | 3676.10996339633 | Cabo Verde |
| 1992 | 4634.29313783828 | 0.288460371177891 | 1135.51624629409 | 3498.77689154419 | Cabo Verde |
| 1993 | 4411.67500595439 | 0.294517285990211 | 1081.70815218614 | 3329.96685376825 | Cabo Verde |
| 1994 | 5070.59496280173 | 0.302216794344931 | 1037.03507482373 | 4033.559887978 | Cabo Verde |
| 1995 | 9112.8736145418 | 0.310675633896478 | 963.272603801038 | 8149.60101074076 | Cabo Verde |
| 1996 | 3454.08304316902 | 0.319736229276549 | 825.19014401299 | 2628.89289915603 | Cabo Verde |
| 1997 | 2954.6948360008 | 0.329049585287328 | 746.168451027529 | 2208.52638497327 | Cabo Verde |
| 1998 | 2582.81820451568 | 0.338786069354652 | 595.571418745156 | 1987.24678577052 | Cabo Verde |
| 1999 | 2319.54902407392 | 0.349296918199719 | 590.695715622247 | 1728.85330845167 | Cabo Verde |
| 2000 | 2036.37443638883 | 0.35989901703054 | 589.788648782928 | 1446.5857876059 | Cabo Verde |
| 2001 | 1865.29824531055 | 0.370070275603364 | 577.44013477029 | 1287.85811054026 | Cabo Verde |
| 2002 | 1725.80555375096 | 0.379846674271366 | 546.376246820581 | 1179.42930693038 | Cabo Verde |
| 2003 | 1559.00824085804 | 0.389419785822276 | 514.631278612246 | 1044.37696224579 | Cabo Verde |
| 2004 | 1415.46587028896 | 0.398838732156207 | 465.130396616288 | 950.335473672672 | Cabo Verde |
| 2005 | 1277.39686595541 | 0.408009221053434 | 310.1314692892 | 967.26539666621 | Cabo Verde |
| 2006 | 1253.64774878306 | 0.417433785875035 | 260.262263304221 | 993.385485478839 | Cabo Verde |
| 2007 | 1139.45358091736 | 0.427379786660776 | 219.659777675977 | 919.793803241383 | Cabo Verde |
| 2008 | 1027.6329261952 | 0.437372129942837 | 117.288979225567 | 910.343946969633 | Cabo Verde |
| 2009 | 920.83463631825 | 0.446691505793351 | 78.0801960451863 | 842.754440273064 | Cabo Verde |
| 2010 | 904.95432004658 | 0.455647568382572 | 71.2588144272483 | 833.695505619332 | Cabo Verde |
| 2011 | 816.66411790703 | 0.464473968892355 | 60.6764900251356 | 755.987627881894 | Cabo Verde |
| 2012 | 777.01672952899 | 0.472857771380441 | 56.4157780288472 | 720.600951500143 | Cabo Verde |
| 2013 | 718.9652704831 | 0.480796480653885 | 54.2135406110905 | 664.75172987201 | Cabo Verde |
| 2014 | 713.60920735306 | 0.488259896216045 | 49.2948830432915 | 664.314324309769 | Cabo Verde |
| 2015 | 659.45311998772 | 0.495229489103676 | 50.7217232690634 | 608.731396718657 | Cabo Verde |
| 2016 | 613.95679814227 | 0.501855423977514 | 23.3608991040918 | 590.595899038178 | Cabo Verde |
| 2017 | 559.56054401052 | 0.508435792446345 | 22.7476317901381 | 536.812912220382 | Cabo Verde |
| 2018 | 524.25828798925 | 0.515285886261897 | 22.2246983576351 | 502.033589631615 | Cabo Verde |
| 2019 | 478.54489994254 | 0.522488008219635 | 22.1628677077012 | 456.382032234839 | Cabo Verde |
| 2020 | 434.30702237187 | 0.528461594777472 | 22.1314398290103 | 412.17558254286 | Cabo Verde |
| 2021 | 408.66555336838 | 0.53353453903661 | 22.0968406158041 | 386.568712752576 | Cabo Verde |
| 1990 | 24494.4777782341 | 0.114638829376832 | 5234.32519285838 | 19260.1525853757 | Chad |
| 1991 | 25725.2039758077 | 0.117954633635022 | 5257.89341239604 | 20467.3105634117 | Chad |
| 1992 | 23514.9843903272 | 0.121132831941026 | 5215.89684753071 | 18299.0875427965 | Chad |
| 1993 | 23177.4137723706 | 0.123600072412422 | 5281.17820805326 | 17896.2355643173 | Chad |
| 1994 | 22806.009212522 | 0.126080571521756 | 5209.46741804959 | 17596.5417944724 | Chad |
| 1995 | 22368.1733486211 | 0.128289491688625 | 5135.57877485192 | 17232.5945737692 | Chad |
| 1996 | 22129.9048175306 | 0.130169483922475 | 5086.03704171752 | 17043.8677758131 | Chad |
| 1997 | 22120.9986330574 | 0.132136021297442 | 4876.04169448367 | 17244.9569385737 | Chad |
| 1998 | 21583.085454307 | 0.134090304250411 | 4951.30999769959 | 16631.7754566074 | Chad |
| 1999 | 21255.772007378 | 0.135825260914887 | 4624.02013632169 | 16631.7518710563 | Chad |
| 2000 | 21205.1800229795 | 0.137412374447162 | 4646.53018736729 | 16558.6498356122 | Chad |
| 2001 | 21085.7496443456 | 0.13966369176859 | 4372.785983332 | 16712.9636610136 | Chad |
| 2002 | 20981.5416611198 | 0.142041700148116 | 3883.64172078434 | 17097.8999403355 | Chad |
| 2003 | 20515.9859842084 | 0.145348052064453 | 3908.27913324484 | 16607.7068509636 | Chad |
| 2004 | 19966.840797948 | 0.150910867941762 | 3224.16871583145 | 16742.6720821166 | Chad |
| 2005 | 19063.383028464 | 0.157033036048947 | 2833.50756682981 | 16229.8754616342 | Chad |
| 2006 | 19012.5360678838 | 0.162576402156357 | 2638.91966684966 | 16373.6164010341 | Chad |
| 2007 | 18989.5551833999 | 0.168161061828519 | 2339.2438781221 | 16650.3113052778 | Chad |
| 2008 | 18434.344907919 | 0.173396783580077 | 2330.77517636569 | 16103.5697315533 | Chad |
| 2009 | 18026.132853846 | 0.178335360569271 | 2345.58781993665 | 15680.5450339093 | Chad |
| 2010 | 17460.0799574736 | 0.184110621219396 | 2319.2164955411 | 15140.8634619325 | Chad |
| 2011 | 16819.5511657705 | 0.189591609430813 | 2333.921797754 | 14485.6293680165 | Chad |
| 2012 | 15682.7161421105 | 0.195262748994652 | 2317.93632755377 | 13364.7798145567 | Chad |
| 2013 | 15435.4488621803 | 0.200952883793321 | 2342.49012442397 | 13092.9587377563 | Chad |
| 2014 | 14679.8315448487 | 0.206658835963237 | 2317.93763433854 | 12361.8939105102 | Chad |
| 2015 | 14385.537929091 | 0.212428696994434 | 2299.89119959871 | 12085.6467294923 | Chad |
| 2016 | 13932.9355205288 | 0.217532179615503 | 2294.46592630426 | 11638.4695942245 | Chad |
| 2017 | 13597.6491684841 | 0.222206950439072 | 2153.64711548679 | 11444.0020529973 | Chad |
| 2018 | 12967.9870021798 | 0.226913880545936 | 2134.53691665898 | 10833.4500855208 | Chad |
| 2019 | 12523.4403070645 | 0.231704754518571 | 1908.98533731519 | 10614.4549697493 | Chad |
| 2020 | 11816.7705365876 | 0.236103082769538 | 1926.32036414796 | 9890.45017243964 | Chad |
| 2021 | 11317.4748932035 | 0.240436018662427 | 1732.32332881529 | 9585.15156438821 | Chad |
| 1990 | 5080.31966319017 | 0.279320352514124 | 1192.01592829883 | 3888.30373489134 | Côte d'Ivoire |
| 1991 | 4989.94193138588 | 0.286492681425857 | 1133.86486668367 | 3856.07706470221 | Côte d'Ivoire |
| 1992 | 4900.50822823183 | 0.293451293054487 | 1066.02300115289 | 3834.48522707894 | Côte d'Ivoire |
| 1993 | 4841.46814444213 | 0.299743215098528 | 1027.04837006884 | 3814.41977437329 | Côte d'Ivoire |
| 1994 | 4785.08960877848 | 0.305210652688978 | 1037.44598240314 | 3747.64362637534 | Côte d'Ivoire |
| 1995 | 4754.77380178313 | 0.310105507687377 | 962.189751778029 | 3792.5840500051 | Côte d'Ivoire |
| 1996 | 4546.89359583266 | 0.314965385351403 | 821.79475837111 | 3725.09883746155 | Côte d'Ivoire |
| 1997 | 4375.75663167286 | 0.319843979495224 | 847.562733418115 | 3528.19389825474 | Côte d'Ivoire |
| 1998 | 4228.52914647246 | 0.324712872541106 | 771.647080031928 | 3456.88206644053 | Côte d'Ivoire |
| 1999 | 4036.02793161897 | 0.329201009286605 | 741.217659407101 | 3294.81027221187 | Côte d'Ivoire |
| 2000 | 3845.48469651119 | 0.333015393275065 | 673.19563933849 | 3172.2890571727 | Côte d'Ivoire |
| 2001 | 3712.1781896718 | 0.336545511228203 | 672.911483360594 | 3039.26670631121 | Côte d'Ivoire |
| 2002 | 3450.2753101678 | 0.339977638379859 | 598.368901759417 | 2851.90640840838 | Côte d'Ivoire |
| 2003 | 3275.1666068937 | 0.343348931560052 | 606.362033922158 | 2668.80457297154 | Côte d'Ivoire |
| 2004 | 3241.12840641452 | 0.346375411313471 | 595.163611602991 | 2645.96479481153 | Côte d'Ivoire |
| 2005 | 3245.79238741917 | 0.34872791321654 | 600.148371156312 | 2645.64401626286 | Côte d'Ivoire |
| 2006 | 3342.22341424938 | 0.35071594385815 | 592.320596644531 | 2749.90281760485 | Côte d'Ivoire |
| 2007 | 3222.24007972334 | 0.35272747838259 | 596.683377949207 | 2625.55670177413 | Côte d'Ivoire |
| 2008 | 3032.07919659233 | 0.355087642720634 | 594.462165970663 | 2437.61703062167 | Côte d'Ivoire |
| 2009 | 2871.66938032566 | 0.357938754729774 | 594.175347909277 | 2277.49403241638 | Côte d'Ivoire |
| 2010 | 2696.95836675545 | 0.361205186672734 | 587.471445047532 | 2109.48692170792 | Côte d'Ivoire |
| 2011 | 2476.87244502782 | 0.364083650087326 | 569.358456565535 | 1907.51398846228 | Côte d'Ivoire |
| 2012 | 2525.18216959116 | 0.367831685487642 | 574.32966360955 | 1950.85250598161 | Côte d'Ivoire |
| 2013 | 2564.14814816593 | 0.372353790323331 | 546.599961072732 | 2017.5481870932 | Côte d'Ivoire |
| 2014 | 2465.86658191193 | 0.377575443834257 | 548.919600933611 | 1916.94698097832 | Côte d'Ivoire |
| 2015 | 2403.14754181104 | 0.383491379717949 | 503.131962107979 | 1900.01557970306 | Côte d'Ivoire |
| 2016 | 2208.40209732701 | 0.389825191524618 | 507.104900649107 | 1701.2971966779 | Côte d'Ivoire |
| 2017 | 1829.21884188069 | 0.396693974091431 | 460.603505167483 | 1368.61533671321 | Côte d'Ivoire |
| 2018 | 1584.75552861111 | 0.404091932623989 | 391.878989483215 | 1192.8765391279 | Côte d'Ivoire |
| 2019 | 1398.94043071891 | 0.411725580444537 | 302.766381917016 | 1096.17404880189 | Côte d'Ivoire |
| 2020 | 1238.24404194249 | 0.419040103341885 | 260.45578953884 | 977.78825240365 | Côte d'Ivoire |
| 2021 | 1125.33129483406 | 0.425941883067361 | 232.013932883851 | 893.317361950209 | Côte d'Ivoire |
| 1990 | 10112.6443968198 | 0.238714846451473 | 1694.6455792174 | 8417.9988176024 | Gambia |
| 1991 | 9859.07076679365 | 0.2450101639184 | 1676.52513316563 | 8182.54563362802 | Gambia |
| 1992 | 9670.19073081732 | 0.251087863304524 | 1514.62248445007 | 8155.56824636725 | Gambia |
| 1993 | 9143.66247252615 | 0.25699614074155 | 1525.35678209672 | 7618.30569042943 | Gambia |
| 1994 | 8614.32408490073 | 0.262424792137193 | 1399.91442453319 | 7214.40966036754 | Gambia |
| 1995 | 8689.19228001887 | 0.267696506742935 | 1277.95842336593 | 7411.23385665294 | Gambia |
| 1996 | 7944.35733909551 | 0.272950190161523 | 1236.78965727385 | 6707.56768182166 | Gambia |
| 1997 | 7424.4935565418 | 0.278274122042772 | 1197.65037405254 | 6226.84318248926 | Gambia |
| 1998 | 7222.49641757596 | 0.283946056199827 | 1205.70977157752 | 6016.78664599844 | Gambia |
| 1999 | 6992.97387122733 | 0.290070621097832 | 1141.48227317474 | 5851.49159805259 | Gambia |
| 2000 | 6164.67486140133 | 0.296354282057957 | 1070.73384026666 | 5093.94102113467 | Gambia |
| 2001 | 5645.90370885844 | 0.302543256845694 | 1028.76180648674 | 4617.1419023717 | Gambia |
| 2002 | 5291.04302540261 | 0.307712926799456 | 967.511210712868 | 4323.53181468974 | Gambia |
| 2003 | 5073.66993724505 | 0.312930916018188 | 966.695266569057 | 4106.97467067599 | Gambia |
| 2004 | 5673.844647523 | 0.31861502922509 | 838.820833514232 | 4835.02381400877 | Gambia |
| 2005 | 5456.85306704705 | 0.323568350048743 | 750.029031783492 | 4706.82403526356 | Gambia |
| 2006 | 4971.05344668598 | 0.328151431165807 | 755.054990543553 | 4215.99845614243 | Gambia |
| 2007 | 4635.21773663258 | 0.332640147075474 | 674.150305223148 | 3961.06743140943 | Gambia |
| 2008 | 4021.56659587091 | 0.337315245461033 | 595.576090412456 | 3425.99050545845 | Gambia |
| 2009 | 3531.01284669585 | 0.342389614346251 | 590.342276745019 | 2940.67056995083 | Gambia |
| 2010 | 3174.80763751849 | 0.347997829857342 | 592.327330956191 | 2582.4803065623 | Gambia |
| 2011 | 2638.19683387342 | 0.35286923573207 | 590.529003554334 | 2047.66783031909 | Gambia |
| 2012 | 2534.32066805533 | 0.358373649782742 | 593.740847594974 | 1940.57982046036 | Gambia |
| 2013 | 2097.02241250491 | 0.36425587830746 | 571.430666286584 | 1525.59174621833 | Gambia |
| 2014 | 2123.52902272232 | 0.369901300206273 | 574.68828293911 | 1548.84073978321 | Gambia |
| 2015 | 1903.13717289706 | 0.375840484637477 | 547.867728745628 | 1355.26944415143 | Gambia |
| 2016 | 1912.38760356896 | 0.381569217140042 | 514.897721256942 | 1397.48988231202 | Gambia |
| 2017 | 2058.25497774612 | 0.387273644458255 | 512.676040988512 | 1545.57893675761 | Gambia |
| 2018 | 1954.34801024382 | 0.393155679437306 | 461.03917642156 | 1493.30883382226 | Gambia |
| 2019 | 1893.53767184442 | 0.39918978005783 | 469.230516496423 | 1424.307155348 | Gambia |
| 2020 | 1685.2755082604 | 0.40452419422916 | 394.125014301447 | 1291.15049395895 | Gambia |
| 2021 | 1528.20068915604 | 0.40971415951073 | 305.93821899051 | 1222.26247016553 | Gambia |
| 1990 | 7232.15984335372 | 0.373112004793066 | 548.639531983493 | 6683.52031137023 | Ghana |
| 1991 | 6703.63785354735 | 0.37923925587052 | 544.988441689077 | 6158.64941185827 | Ghana |
| 1992 | 6307.33506830558 | 0.384856767297978 | 510.801837872713 | 5796.53323043287 | Ghana |
| 1993 | 5985.32011425178 | 0.389968494182018 | 513.911946881004 | 5471.40816737078 | Ghana |
| 1994 | 5692.96534874581 | 0.394953662708371 | 460.396535385967 | 5232.56881335984 | Ghana |
| 1995 | 5413.99131684884 | 0.39993020380395 | 461.531097924965 | 4952.46021892388 | Ghana |
| 1996 | 5107.13260861849 | 0.405003046925554 | 399.156658523509 | 4707.97595009498 | Ghana |
| 1997 | 4734.84825759727 | 0.410038073297741 | 309.795647534932 | 4425.05261006234 | Ghana |
| 1998 | 4464.62146439668 | 0.414888532103537 | 267.855910584527 | 4196.76555381215 | Ghana |
| 1999 | 4162.04703618973 | 0.419755496390431 | 258.013919956416 | 3904.03311623331 | Ghana |
| 2000 | 3961.00057067408 | 0.424725211650337 | 234.905730668159 | 3726.09484000592 | Ghana |
| 2001 | 3747.27479096338 | 0.42941184646569 | 208.468100856415 | 3538.80669010696 | Ghana |
| 2002 | 3598.64400583848 | 0.434104128009022 | 204.850254398203 | 3393.79375144028 | Ghana |
| 2003 | 3677.81164783949 | 0.439118212630728 | 118.526942173241 | 3559.28470566625 | Ghana |
| 2004 | 3553.54686104612 | 0.444236977254313 | 104.049081153292 | 3449.49777989283 | Ghana |
| 2005 | 3447.76557314837 | 0.449360019930072 | 78.7530353909197 | 3369.01253775745 | Ghana |
| 2006 | 3288.9771580824 | 0.454692737065749 | 70.9742661550191 | 3218.00289192738 | Ghana |
| 2007 | 3059.03650592479 | 0.460606245250364 | 64.4219547244872 | 2994.6145512003 | Ghana |
| 2008 | 2559.19918454286 | 0.467259066649708 | 56.2924646650101 | 2502.90671987785 | Ghana |
| 2009 | 2294.3593210253 | 0.473848047331171 | 58.5542424540513 | 2235.80507857125 | Ghana |
| 2010 | 2043.38841216329 | 0.480777891769648 | 52.4781983819093 | 1990.91021378138 | Ghana |
| 2011 | 1933.81264361065 | 0.489020973808153 | 50.0923389837209 | 1883.72030462693 | Ghana |
| 2012 | 1512.65656743734 | 0.497513931634325 | 25.6680433212724 | 1486.98852411607 | Ghana |
| 2013 | 1362.22556866126 | 0.506004646555731 | 22.9625819113985 | 1339.26298674986 | Ghana |
| 2014 | 1342.970556421 | 0.514119061082341 | 22.5571213295029 | 1320.4134350915 | Ghana |
| 2015 | 1282.16173934479 | 0.521539202423625 | 22.1729051719454 | 1259.98883417284 | Ghana |
| 2016 | 1178.15876288922 | 0.528629585500254 | 22.1410340749816 | 1156.01772881424 | Ghana |
| 2017 | 1127.78278198156 | 0.536169940006201 | 21.3449509740562 | 1106.4378310075 | Ghana |
| 2018 | 1003.14915215562 | 0.543840539336404 | 21.1901297106266 | 981.959022444993 | Ghana |
| 2019 | 894.88370698779 | 0.551632026781383 | 19.8584626747667 | 875.025244313023 | Ghana |
| 2020 | 827.9091626946 | 0.558462173287852 | 17.5880921614354 | 810.321070533165 | Ghana |
| 2021 | 735.9911461261 | 0.564930389658009 | 15.7563513664861 | 720.234794759614 | Ghana |
| 1990 | 13889.8732412085 | 0.17829542065613 | 2350.51059479316 | 11539.3626464153 | Guinea |
| 1991 | 13123.9332018235 | 0.181459390831103 | 2336.85950085239 | 10787.0737009711 | Guinea |
| 1992 | 12322.9915291643 | 0.184485016316931 | 2321.30410502596 | 10001.6874241383 | Guinea |
| 1993 | 11787.2132945934 | 0.187686241926773 | 2360.8992390908 | 9426.3140555026 | Guinea |
| 1994 | 11787.6194480363 | 0.191128836868019 | 2325.43579008557 | 9462.18365795073 | Guinea |
| 1995 | 11549.6952934153 | 0.194573244617025 | 2328.40204448361 | 9221.29324893169 | Guinea |
| 1996 | 10327.8537374433 | 0.198633755485464 | 2324.30162104254 | 8003.55211640076 | Guinea |
| 1997 | 9713.98508952275 | 0.203280448621307 | 2330.17089782257 | 7383.81419170018 | Guinea |
| 1998 | 9100.2902620406 | 0.208003382623507 | 2332.58153045917 | 6767.70873158143 | Guinea |
| 1999 | 8258.72682538326 | 0.212412705488692 | 2288.80124682803 | 5969.92557855523 | Guinea |
| 2000 | 7539.60409046742 | 0.216649791599883 | 2331.08283306847 | 5208.52125739895 | Guinea |
| 2001 | 6913.71811699727 | 0.221090940982099 | 2143.74311185108 | 4769.97500514619 | Guinea |
| 2002 | 6455.80002707383 | 0.225898779303173 | 2155.32644881671 | 4300.47357825712 | Guinea |
| 2003 | 6187.59514084693 | 0.230522593906606 | 1949.45001240668 | 4238.14512844025 | Guinea |
| 2004 | 6276.28952433994 | 0.235134970165926 | 1918.98244552741 | 4357.30707881253 | Guinea |
| 2005 | 5928.89565975507 | 0.239691845090143 | 1713.23766115855 | 4215.65799859652 | Guinea |
| 2006 | 5675.74157850047 | 0.24401012651719 | 1704.47413545264 | 3971.26744304782 | Guinea |
| 2007 | 5282.77616568238 | 0.248471690169451 | 1515.65014766451 | 3767.12601801787 | Guinea |
| 2008 | 4787.8281820308 | 0.253167688592502 | 1522.62363895773 | 3265.20454307307 | Guinea |
| 2009 | 4207.79676856648 | 0.257552785014134 | 1365.74026972793 | 2842.05649883854 | Guinea |
| 2010 | 3993.23652967014 | 0.262405948891134 | 1385.53897277721 | 2607.69755689293 | Guinea |
| 2011 | 3625.26510209369 | 0.267624599664508 | 1270.05402156845 | 2355.21108052524 | Guinea |
| 2012 | 3301.61522866485 | 0.273428318991395 | 1238.47079117173 | 2063.14443749312 | Guinea |
| 2013 | 3053.0655542044 | 0.279600444049448 | 1207.04698705646 | 1846.01856714794 | Guinea |
| 2014 | 2786.36199009888 | 0.2859612643771 | 1140.06068573659 | 1646.30130436229 | Guinea |
| 2015 | 2583.12473355928 | 0.292363614463187 | 1090.74907150513 | 1492.37566205415 | Guinea |
| 2016 | 2400.40085376973 | 0.299112412084657 | 1076.04241947105 | 1324.35843429868 | Guinea |
| 2017 | 2139.7021582423 | 0.306881461581969 | 1014.06995381619 | 1125.63220442611 | Guinea |
| 2018 | 1880.19042257597 | 0.314839587576224 | 949.182916857532 | 931.007505718438 | Guinea |
| 2019 | 1677.18105432186 | 0.322839848435435 | 772.813892510175 | 904.367161811685 | Guinea |
| 2020 | 1497.59837723124 | 0.329877997039031 | 676.467039210456 | 821.131338020784 | Guinea |
| 2021 | 1228.69265283692 | 0.33640129335069 | 673.544523044125 | 555.148129792795 | Guinea |
| 1990 | 12548.5025545616 | 0.207614838569111 | 2318.89876133882 | 10229.6037932228 | Guinea-Bissau |
| 1991 | 12004.1556413454 | 0.212505984149041 | 2301.18381606536 | 9702.97182528004 | Guinea-Bissau |
| 1992 | 11545.5114137136 | 0.217026088469754 | 2290.32625041112 | 9255.18516330249 | Guinea-Bissau |
| 1993 | 11084.716143302 | 0.221678978443034 | 2129.80996862423 | 8954.90617467777 | Guinea-Bissau |
| 1994 | 12303.8528100567 | 0.226434932988846 | 2141.32479640211 | 10162.5280136546 | Guinea-Bissau |
| 1995 | 10100.5859896379 | 0.231223526184368 | 1921.62780761907 | 8178.95818201883 | Guinea-Bissau |
| 1996 | 17683.7872024549 | 0.236106373654915 | 1936.15774728787 | 15747.629455167 | Guinea-Bissau |
| 1997 | 15539.4623362763 | 0.241360583940643 | 1703.19082034488 | 13836.2715159314 | Guinea-Bissau |
| 1998 | 8615.35212104815 | 0.244371797123174 | 1692.52688325609 | 6922.82523779206 | Guinea-Bissau |
| 1999 | 8135.47656161764 | 0.24821731725385 | 1514.47271259488 | 6621.00384902276 | Guinea-Bissau |
| 2000 | 8299.7941291794 | 0.252026519139868 | 1518.98031167181 | 6780.81381750759 | Guinea-Bissau |
| 2001 | 8271.8152910895 | 0.255786767601624 | 1512.97588635763 | 6758.83940473187 | Guinea-Bissau |
| 2002 | 8094.32452577562 | 0.259321815568398 | 1374.42119977796 | 6719.90332599766 | Guinea-Bissau |
| 2003 | 7698.47019295076 | 0.262747723500015 | 1379.46028676281 | 6319.00990618795 | Guinea-Bissau |
| 2004 | 7336.83955960195 | 0.26627173587343 | 1376.14805612022 | 5960.69150348173 | Guinea-Bissau |
| 2005 | 9425.48686891744 | 0.270056131535634 | 1229.59544911604 | 8195.8914198014 | Guinea-Bissau |
| 2006 | 6248.31787122624 | 0.273803986556405 | 1251.371790196 | 4996.94608103024 | Guinea-Bissau |
| 2007 | 6906.35795698774 | 0.277805016731205 | 1196.08252879266 | 5710.27542819508 | Guinea-Bissau |
| 2008 | 7740.85385059631 | 0.282151501527761 | 1211.22334635963 | 6529.63050423668 | Guinea-Bissau |
| 2009 | 5604.7478225221 | 0.286423345485565 | 1140.7989902653 | 4463.9488322568 | Guinea-Bissau |
| 2010 | 5053.59411248363 | 0.291141639242703 | 1131.20970897893 | 3922.3844035047 | Guinea-Bissau |
| 2011 | 4379.02035190199 | 0.29668296654448 | 1086.04778668468 | 3292.97256521731 | Guinea-Bissau |
| 2012 | 3291.43599146216 | 0.301562341875562 | 1022.90000775201 | 2268.53598371015 | Guinea-Bissau |
| 2013 | 3168.03975443379 | 0.30660200942877 | 1026.17952937344 | 2141.86022506035 | Guinea-Bissau |
| 2014 | 2753.10026016235 | 0.311602905000269 | 963.382158425465 | 1789.71810173689 | Guinea-Bissau |
| 2015 | 2396.98317035391 | 0.31709001451773 | 837.395905805525 | 1559.58726454838 | Guinea-Bissau |
| 2016 | 2344.64446893957 | 0.32297967010132 | 750.110157615791 | 1594.53431132378 | Guinea-Bissau |
| 2017 | 2122.57682500802 | 0.329326492160232 | 748.971738070215 | 1373.6050869378 | Guinea-Bissau |
| 2018 | 1811.19475993629 | 0.335667832836675 | 674.882980443327 | 1136.31177949296 | Guinea-Bissau |
| 2019 | 1584.37705810124 | 0.342156114875433 | 599.562482868962 | 984.814575232278 | Guinea-Bissau |
| 2020 | 1446.48613681534 | 0.347798997943486 | 591.541026052227 | 854.945110763113 | Guinea-Bissau |
| 2021 | 1292.61890313275 | 0.353109621364536 | 594.60302289953 | 698.01588023322 | Guinea-Bissau |
| 1990 | 19254.3815285284 | 0.235296846955071 | 1946.83802584258 | 17307.5435026858 | Liberia |
| 1991 | 23819.5296020906 | 0.238212217752062 | 1719.94937901991 | 22099.5802230707 | Liberia |
| 1992 | 21576.108488138 | 0.23828331265798 | 1683.07300903443 | 19893.0354791036 | Liberia |
| 1993 | 18053.4259095548 | 0.235984406116034 | 1933.63971187035 | 16119.7861976845 | Liberia |
| 1994 | 17999.3671169022 | 0.232656773774217 | 1920.37412568752 | 16078.9929912147 | Liberia |
| 1995 | 19154.3234814149 | 0.228424478219389 | 1923.4151795745 | 17230.9083018404 | Liberia |
| 1996 | 18806.1906242984 | 0.222682591493435 | 2159.83584056687 | 16646.3547837315 | Liberia |
| 1997 | 17233.2761424683 | 0.219672764660528 | 2149.50832669555 | 15083.7678157727 | Liberia |
| 1998 | 16327.4305352983 | 0.219053854841174 | 2289.8102300822 | 14037.6203052161 | Liberia |
| 1999 | 15309.3959688343 | 0.222212867280137 | 2143.97290597686 | 13165.4230628574 | Liberia |
| 2000 | 14333.4031923078 | 0.231173361787615 | 1912.02608145141 | 12421.3771108564 | Liberia |
| 2001 | 12953.5164241724 | 0.239594386141111 | 1712.23716689114 | 11241.2792572813 | Liberia |
| 2002 | 11336.0873101698 | 0.248311971373795 | 1516.49332007364 | 9819.59399009616 | Liberia |
| 2003 | 10448.8001617932 | 0.251595457910319 | 1507.81880607561 | 8940.98135571759 | Liberia |
| 2004 | 9474.24565201361 | 0.255179610402999 | 1514.5532988092 | 7959.69235320441 | Liberia |
| 2005 | 9253.08347720553 | 0.258967990380421 | 1385.64406995973 | 7867.4394072458 | Liberia |
| 2006 | 8144.83489509634 | 0.262897258459796 | 1375.44515674688 | 6769.38973834946 | Liberia |
| 2007 | 7047.96119698009 | 0.267239568498419 | 1274.07619735129 | 5773.8849996288 | Liberia |
| 2008 | 6554.3990228406 | 0.271853595673486 | 1237.32655857468 | 5317.07246426592 | Liberia |
| 2009 | 6789.62565724519 | 0.276804110528483 | 1195.03690522605 | 5594.58875201914 | Liberia |
| 2010 | 6787.71781874953 | 0.28223575229287 | 1194.90113364495 | 5592.81668510458 | Liberia |
| 2011 | 6603.36702190231 | 0.288015144991527 | 1136.73667248297 | 5466.63034941934 | Liberia |
| 2012 | 6464.48426633269 | 0.294854315315019 | 1067.8628233388 | 5396.62144299389 | Liberia |
| 2013 | 6033.16360228627 | 0.302985656345751 | 1025.02727620721 | 5008.13632607906 | Liberia |
| 2014 | 5274.14919936795 | 0.310955439531843 | 959.235828746685 | 4314.91337062127 | Liberia |
| 2015 | 4714.75204077068 | 0.318485165511089 | 844.819505890054 | 3869.93253488063 | Liberia |
| 2016 | 4058.48869337907 | 0.325085173839682 | 760.400254676699 | 3298.08843870237 | Liberia |
| 2017 | 3532.84961445736 | 0.331527785784429 | 685.085310669953 | 2847.76430378741 | Liberia |
| 2018 | 3196.37770681535 | 0.337784577514724 | 598.93537132343 | 2597.44233549192 | Liberia |
| 2019 | 3010.43338977157 | 0.343403262226321 | 595.599059902276 | 2414.83432986929 | Liberia |
| 2020 | 2731.01709207056 | 0.348133889761756 | 596.942954014402 | 2134.07413805616 | Liberia |
| 2021 | 2484.14840867142 | 0.352442451643965 | 597.007869104498 | 1887.14053956692 | Liberia |
| 1990 | 9848.90668260063 | 0.126526427537221 | 5242.89733911182 | 4606.00934348881 | Mali |
| 1991 | 9492.56099704819 | 0.129128150017575 | 5086.8531208235 | 4405.70787622469 | Mali |
| 1992 | 9208.06907567557 | 0.131537448786118 | 4914.03246565961 | 4294.03661001596 | Mali |
| 1993 | 8872.45039105917 | 0.134177054462465 | 4911.27773409857 | 3961.1726569606 | Mali |
| 1994 | 8519.58503279835 | 0.136937829291313 | 4663.73562596799 | 3855.84940683036 | Mali |
| 1995 | 8276.48410543722 | 0.139866225457785 | 4342.45681182074 | 3934.02729361647 | Mali |
| 1996 | 8147.08904835222 | 0.143078915935706 | 3854.94660741494 | 4292.14244093728 | Mali |
| 1997 | 7097.71999761608 | 0.146414326236527 | 3494.04424751074 | 3603.67575010534 | Mali |
| 1998 | 6570.30842528772 | 0.149986729501612 | 3214.59645927713 | 3355.71196601059 | Mali |
| 1999 | 6247.09738828798 | 0.153737335688568 | 3071.9348004516 | 3175.16258783638 | Mali |
| 2000 | 6053.21533369122 | 0.157190027521405 | 2831.06158302765 | 3222.15375066357 | Mali |
| 2001 | 5731.04200595473 | 0.161344447555084 | 2651.71740958193 | 3079.3245963728 | Mali |
| 2002 | 6214.48599504416 | 0.165420695773092 | 2467.19227777376 | 3747.2937172704 | Mali |
| 2003 | 6080.89579456918 | 0.169733502039948 | 2352.76070267572 | 3728.13509189346 | Mali |
| 2004 | 5827.76048161078 | 0.173779552322233 | 2332.04913334781 | 3495.71134826297 | Mali |
| 2005 | 5560.29293132159 | 0.178029844366254 | 2337.57945733877 | 3222.71347398282 | Mali |
| 2006 | 5201.04976838744 | 0.182452468201007 | 2345.3021964521 | 2855.74757193534 | Mali |
| 2007 | 4611.52092416107 | 0.186912994942121 | 2322.73962571821 | 2288.78129844286 | Mali |
| 2008 | 4347.12393034854 | 0.191536745613522 | 2330.27277771379 | 2016.85115263475 | Mali |
| 2009 | 3964.20512010782 | 0.196537650861103 | 2334.85840991565 | 1629.34671019217 | Mali |
| 2010 | 3533.48528233236 | 0.201803535998378 | 2335.70785877042 | 1197.77742356194 | Mali |
| 2011 | 3177.31058979517 | 0.207097221274123 | 2315.51170465287 | 861.7988851423 | Mali |
| 2012 | 3035.37412758372 | 0.212465060096693 | 2326.04253182962 | 709.331595754101 | Mali |
| 2013 | 2773.99841430603 | 0.217993867180652 | 2297.82411976155 | 476.174294544477 | Mali |
| 2014 | 2407.88936112424 | 0.223729979734488 | 2133.68970831521 | 274.19965280903 | Mali |
| 2015 | 2148.12031355516 | 0.229826262811848 | 1904.6460828553 | 243.47423069986 | Mali |
| 2016 | 2153.80515301198 | 0.236186231331436 | 1923.45627880199 | 230.34887420999 | Mali |
| 2017 | 2027.36148468859 | 0.242726134546644 | 1714.12714848898 | 313.23433619961 | Mali |
| 2018 | 1855.9157503691 | 0.249419730729374 | 1530.57924269171 | 325.336507677388 | Mali |
| 2019 | 1685.76288924144 | 0.256250745993336 | 1511.87580950541 | 173.887079736032 | Mali |
| 2020 | 1522.98319045077 | 0.262629218759395 | 1363.64146292908 | 159.341727521686 | Mali |
| 2021 | 1323.25147152765 | 0.268579940925684 | 1248.6413870814 | 74.6100844462478 | Mali |
| 1990 | 7199.91727186595 | 0.33578094163222 | 665.745678415563 | 6534.17159345039 | Mauritania |
| 1991 | 6884.86973689415 | 0.341135174811723 | 592.298126834785 | 6292.57161005937 | Mauritania |
| 1992 | 6613.12635801795 | 0.346531288170585 | 599.73552671248 | 6013.39083130547 | Mauritania |
| 1993 | 6370.68007073997 | 0.35217657188477 | 592.046580226194 | 5778.63349051378 | Mauritania |
| 1994 | 5843.58686697375 | 0.357585911567431 | 589.108889497227 | 5254.47797747652 | Mauritania |
| 1995 | 5519.55033607698 | 0.363037695183683 | 576.53282280941 | 4943.01751326757 | Mauritania |
| 1996 | 5867.75959099522 | 0.368508328412644 | 572.832557176529 | 5294.92703381869 | Mauritania |
| 1997 | 5566.11961883702 | 0.373021338681094 | 546.662294821845 | 5019.45732401518 | Mauritania |
| 1998 | 5561.48603645207 | 0.377080249599675 | 548.030255597132 | 5013.45578085494 | Mauritania |
| 1999 | 5297.33452390097 | 0.381112297471017 | 508.105093968474 | 4789.2294299325 | Mauritania |
| 2000 | 5243.89458526708 | 0.384205379717954 | 519.432973136373 | 4724.46161213071 | Mauritania |
| 2001 | 5047.96876083862 | 0.386487415894351 | 514.564238704326 | 4533.40452213429 | Mauritania |
| 2002 | 4907.83187237983 | 0.388564477604617 | 510.866637695569 | 4396.96523468426 | Mauritania |
| 2003 | 4551.24932146684 | 0.39110487913777 | 459.605856071967 | 4091.64346539487 | Mauritania |
| 2004 | 4348.86868944849 | 0.394012933734168 | 462.330846130021 | 3886.53784331847 | Mauritania |
| 2005 | 4569.52710314153 | 0.39749910452189 | 460.455164836749 | 4109.07193830478 | Mauritania |
| 2006 | 4251.93822270227 | 0.403159466975078 | 392.808451444806 | 3859.12977125746 | Mauritania |
| 2007 | 3791.35364764647 | 0.408377122155099 | 317.867904730207 | 3473.48574291626 | Mauritania |
| 2008 | 3348.88300850893 | 0.413233621211221 | 273.880591500064 | 3075.00241700887 | Mauritania |
| 2009 | 3248.30657444532 | 0.4179106088283 | 255.770378960748 | 2992.53619548457 | Mauritania |
| 2010 | 2838.12195413331 | 0.423202436351832 | 233.404464317228 | 2604.71748981608 | Mauritania |
| 2011 | 2568.35763853827 | 0.428867289887467 | 215.674238861974 | 2352.6833996763 | Mauritania |
| 2012 | 2502.2012172433 | 0.434724375629838 | 209.690610226054 | 2292.51060701725 | Mauritania |
| 2013 | 2259.54634286009 | 0.441068910286714 | 110.696189498769 | 2148.85015336132 | Mauritania |
| 2014 | 1825.32241735788 | 0.447653881356557 | 73.6887300673582 | 1751.63368729052 | Mauritania |
| 2015 | 1623.9388297259 | 0.454523487871252 | 72.0686814234449 | 1551.87014830245 | Mauritania |
| 2016 | 1419.62346094124 | 0.461269544556856 | 65.2990613596389 | 1354.3243995816 | Mauritania |
| 2017 | 1315.78726117538 | 0.468452365279582 | 58.9012996731324 | 1256.88596150225 | Mauritania |
| 2018 | 1177.0901133757 | 0.475839507248469 | 56.8491105903579 | 1120.24100278534 | Mauritania |
| 2019 | 1166.18241511866 | 0.483767737935156 | 53.6552832107104 | 1112.52713190795 | Mauritania |
| 2020 | 1041.32655009107 | 0.491365602592056 | 49.4506537414753 | 991.875896349595 | Mauritania |
| 2021 | 997.32823794071 | 0.498945100442996 | 24.5896533731221 | 972.738584567588 | Mauritania |
| 1990 | 15327.8957598441 | 0.0808684798233195 | 5262.3266608844 | 10065.5690989597 | Niger |
| 1991 | 15305.1444386138 | 0.0828482077018824 | 5233.81226174853 | 10071.3321768653 | Niger |
| 1992 | 15070.5967067534 | 0.0847715776568405 | 5220.21395217555 | 9850.38275457785 | Niger |
| 1993 | 14716.9402123284 | 0.0866964903938328 | 5287.61740701863 | 9429.32280530977 | Niger |
| 1994 | 14251.6358370839 | 0.0886087332313997 | 5225.83079466785 | 9025.80504241605 | Niger |
| 1995 | 13820.2073228938 | 0.090473708405212 | 5243.56761104688 | 8576.63971184692 | Niger |
| 1996 | 13195.1567988852 | 0.09234635941853 | 5230.30782520639 | 7964.84897367881 | Niger |
| 1997 | 12639.2436211852 | 0.0942191148661978 | 5229.36027203385 | 7409.88334915135 | Niger |
| 1998 | 12089.993852115 | 0.0965451528067399 | 5276.02181614635 | 6813.97203596865 | Niger |
| 1999 | 11593.3427331782 | 0.0988136856452598 | 5280.32505546125 | 6313.01767771695 | Niger |
| 2000 | 11260.4641612265 | 0.100886948404838 | 5224.95091362745 | 6035.51324759905 | Niger |
| 2001 | 10817.908130107 | 0.103271776631217 | 5195.40406578175 | 5622.50406432525 | Niger |
| 2002 | 10114.8262920602 | 0.105741108731078 | 5262.97251016174 | 4851.85378189846 | Niger |
| 2003 | 9672.08596529766 | 0.108322891429361 | 5262.89139449385 | 4409.19457080381 | Niger |
| 2004 | 8649.64321273273 | 0.110764495003726 | 5243.70005856783 | 3405.9431541649 | Niger |
| 2005 | 8866.69055484977 | 0.11345202277039 | 5271.29759441657 | 3595.3929604332 | Niger |
| 2006 | 7955.16635708509 | 0.116247074448783 | 5262.37188833974 | 2692.79446874535 | Niger |
| 2007 | 7252.02658630483 | 0.11897162605295 | 5213.6290535524 | 2038.39753275243 | Niger |
| 2008 | 5994.43487255668 | 0.121957786240242 | 5178.41085564067 | 816.024016916006 | Niger |
| 2009 | 5587.3643583259 | 0.124829381330847 | 5158.01534530715 | 429.349013018747 | Niger |
| 2010 | 5068.6968233437 | 0.127952995735601 | 5036.36144125959 | 32.3353820841057 | Niger |
| 2011 | 4804.33946012706 | 0.130958067074432 | 4804.33946012706 | 0 | Niger |
| 2012 | 4426.45845663704 | 0.134469718123206 | 4426.45845663704 | 0 | Niger |
| 2013 | 4251.96685813307 | 0.137978170438014 | 4251.96685813307 | 0 | Niger |
| 2014 | 3649.08073605813 | 0.141731738162587 | 3649.08073605813 | 0 | Niger |
| 2015 | 3253.36204402269 | 0.14544050326966 | 3253.36204402269 | 0 | Niger |
| 2016 | 3128.5709422289 | 0.149200109150915 | 3128.5709422289 | 0 | Niger |
| 2017 | 2981.77077670685 | 0.152959169593186 | 2981.77077670685 | 0 | Niger |
| 2018 | 2699.95159553256 | 0.15687519554195 | 2699.95159553256 | 0 | Niger |
| 2019 | 2498.03342641896 | 0.160889483579043 | 2498.03342641896 | 0 | Niger |
| 2020 | 2417.97073830702 | 0.164539264581005 | 2417.97073830702 | 0 | Niger |
| 2021 | 2236.82640939552 | 0.168072773803995 | 2236.82640939552 | 0 | Niger |
| 1990 | 31289.465708292 | 0.305868046855462 | 1023.72387710613 | 30265.7418311859 | Nigeria |
| 1991 | 33166.1805783305 | 0.308489225313095 | 972.720621402469 | 32193.459956928 | Nigeria |
| 1992 | 30447.2993859111 | 0.311234052054088 | 957.81963526434 | 29489.4797506468 | Nigeria |
| 1993 | 29851.6131153993 | 0.313983059877659 | 955.625608806188 | 28895.9875065931 | Nigeria |
| 1994 | 29482.5230420348 | 0.316747446775232 | 813.884680213726 | 28668.6383618211 | Nigeria |
| 1995 | 29088.42724391 | 0.319637438693347 | 830.237908253043 | 28258.189335657 | Nigeria |
| 1996 | 29008.8551409789 | 0.323137684748282 | 766.219195703491 | 28242.6359452754 | Nigeria |
| 1997 | 28474.6990507636 | 0.326690552886558 | 756.010338671651 | 27718.688712092 | Nigeria |
| 1998 | 28270.5434212962 | 0.330263851422919 | 676.767587129899 | 27593.7758341663 | Nigeria |
| 1999 | 28231.4779952349 | 0.333936499661916 | 676.867902426963 | 27554.6100928079 | Nigeria |
| 2000 | 27964.7437565635 | 0.338201742797398 | 603.807514718643 | 27360.9362418449 | Nigeria |
| 2001 | 27689.9343840165 | 0.343029750745818 | 591.586750124476 | 27098.347633892 | Nigeria |
| 2002 | 27258.6865945701 | 0.349092528396007 | 599.658199993924 | 26659.0283945762 | Nigeria |
| 2003 | 26469.1687992929 | 0.355911392103181 | 588.219932872446 | 25880.9488664205 | Nigeria |
| 2004 | 25841.2640056556 | 0.36378081411686 | 580.575996841315 | 25260.6880088143 | Nigeria |
| 2005 | 24825.2197010064 | 0.372583553203769 | 547.930354130995 | 24277.2893468754 | Nigeria |
| 2006 | 24688.9935640299 | 0.381026040235948 | 518.336859234792 | 24170.6567047951 | Nigeria |
| 2007 | 23954.9236585315 | 0.38977964917576 | 506.143767448889 | 23448.7798910826 | Nigeria |
| 2008 | 22502.3842351223 | 0.398037706162251 | 463.209520039278 | 22039.174715083 | Nigeria |
| 2009 | 21435.4856899744 | 0.40635330747071 | 363.057248885042 | 21072.4284410894 | Nigeria |
| 2010 | 20617.3051910435 | 0.414997747961223 | 271.008399405619 | 20346.2967916379 | Nigeria |
| 2011 | 18747.5506321661 | 0.42335191474477 | 234.076653232579 | 18513.4739789335 | Nigeria |
| 2012 | 17223.1997970678 | 0.431780375863827 | 203.512078157171 | 17019.6877189106 | Nigeria |
| 2013 | 15867.0394180872 | 0.440124663848588 | 122.972337312974 | 15744.0670807742 | Nigeria |
| 2014 | 14660.8409527398 | 0.44876682284048 | 68.8916296018944 | 14591.9493231379 | Nigeria |
| 2015 | 13499.8886787897 | 0.457240991469025 | 64.8620658157103 | 13435.026612974 | Nigeria |
| 2016 | 12778.8268661202 | 0.465183780994652 | 61.2919808454618 | 12717.5348852747 | Nigeria |
| 2017 | 11766.2100382999 | 0.472972868984332 | 56.3386611249515 | 11709.8713771749 | Nigeria |
| 2018 | 10904.438933861 | 0.480844413426724 | 53.445738408573 | 10850.9931954524 | Nigeria |
| 2019 | 10024.6531244256 | 0.488787361344695 | 50.6960377639193 | 9973.95708666168 | Nigeria |
| 2020 | 8661.63651812702 | 0.496204741160216 | 35.6866920169185 | 8625.9498261101 | Nigeria |
| 2021 | 8171.55639895896 | 0.503390832763244 | 22.9997022544664 | 8148.55669670449 | Nigeria |
| 1990 | 8521.37351503884 | 0.3095428523248 | 956.686439850447 | 7564.68707518839 | Sao Tome and Principe |
| 1991 | 7612.69478728749 | 0.310601028486155 | 965.955175683668 | 6646.73961160382 | Sao Tome and Principe |
| 1992 | 7363.12072578774 | 0.3115154473454 | 966.752592586919 | 6396.36813320082 | Sao Tome and Principe |
| 1993 | 7140.12217152932 | 0.31274355801833 | 961.641368688919 | 6178.4808028404 | Sao Tome and Principe |
| 1994 | 6937.52221487407 | 0.314261087139278 | 962.669330362977 | 5974.85288451109 | Sao Tome and Principe |
| 1995 | 6360.49863862962 | 0.315843919852344 | 847.03963997172 | 5513.4589986579 | Sao Tome and Principe |
| 1996 | 5899.18637846651 | 0.317729288379101 | 840.34367406122 | 5058.84270440529 | Sao Tome and Principe |
| 1997 | 5299.53318821202 | 0.319795669969502 | 850.067587538277 | 4449.46560067374 | Sao Tome and Principe |
| 1998 | 4757.35555119849 | 0.322519218688325 | 751.053182746502 | 4006.30236845199 | Sao Tome and Principe |
| 1999 | 4452.89631733592 | 0.325651915603451 | 758.568866583222 | 3694.3274507527 | Sao Tome and Principe |
| 2000 | 3055.70597325069 | 0.329460109182343 | 740.880707469825 | 2314.82526578087 | Sao Tome and Principe |
| 2001 | 2774.58571520296 | 0.334484786134682 | 682.837751388964 | 2091.747963814 | Sao Tome and Principe |
| 2002 | 2378.16400569411 | 0.340302836742805 | 592.694670132068 | 1785.46933556204 | Sao Tome and Principe |
| 2003 | 2095.62270565544 | 0.346851981499259 | 591.389455068642 | 1504.2332505868 | Sao Tome and Principe |
| 2004 | 1834.90464644416 | 0.353722383532282 | 588.646613889926 | 1246.25803255423 | Sao Tome and Principe |
| 2005 | 3416.51044358101 | 0.361105881091861 | 583.983140814873 | 2832.52730276614 | Sao Tome and Principe |
| 2006 | 2632.99384475087 | 0.369413007194338 | 578.183464027409 | 2054.81038072346 | Sao Tome and Principe |
| 2007 | 1947.56352914916 | 0.377813517075578 | 547.683057727572 | 1399.88047142159 | Sao Tome and Principe |
| 2008 | 2024.21605897531 | 0.386688943651063 | 508.188245848488 | 1516.02781312682 | Sao Tome and Principe |
| 2009 | 1568.07181613875 | 0.39582123767217 | 464.789746548959 | 1103.28206958979 | Sao Tome and Principe |
| 2010 | 1504.97431030682 | 0.405530666154327 | 396.953085207521 | 1108.0212250993 | Sao Tome and Principe |
| 2011 | 1098.33303544467 | 0.41578372764052 | 270.932610410542 | 827.400425034128 | Sao Tome and Principe |
| 2012 | 828.76157866184 | 0.425721314870657 | 231.911689703666 | 596.849888958174 | Sao Tome and Principe |
| 2013 | 759.07174387203 | 0.435592459999619 | 186.097611522818 | 572.974132349212 | Sao Tome and Principe |
| 2014 | 795.79361714777 | 0.44514623672643 | 106.464842749629 | 689.328774398141 | Sao Tome and Principe |
| 2015 | 703.02483821629 | 0.454051473195403 | 73.2926856066946 | 629.732152609595 | Sao Tome and Principe |
| 2016 | 531.46666150301 | 0.463230081728275 | 66.3366198071095 | 465.1300416959 | Sao Tome and Principe |
| 2017 | 478.93769505059 | 0.47217092996608 | 55.6204889719703 | 423.31720607862 | Sao Tome and Principe |
| 2018 | 446.47069433136 | 0.481098204433028 | 52.8264135265946 | 393.644280804765 | Sao Tome and Principe |
| 2019 | 410.17518154934 | 0.489838229899105 | 49.4321115302297 | 360.74307001911 | Sao Tome and Principe |
| 2020 | 374.93806849522 | 0.497861408762174 | 26.0871547592405 | 348.85091373598 | Sao Tome and Principe |
| 2021 | 329.10902107267 | 0.505413746708186 | 23.0087048081021 | 306.100316264568 | Sao Tome and Principe |
| 1990 | 26498.37190883 | 0.238047613296703 | 1683.30454224505 | 24815.067366585 | Senegal |
| 1991 | 25157.265475335 | 0.244482178069769 | 1669.0864042455 | 23488.1790710895 | Senegal |
| 1992 | 23891.448986717 | 0.250416531795468 | 1504.8353711278 | 22386.6136155892 | Senegal |
| 1993 | 22876.6350284964 | 0.255767963935604 | 1520.96869848752 | 21355.6663300089 | Senegal |
| 1994 | 21905.2847440059 | 0.260379997494734 | 1395.61066874817 | 20509.6740752577 | Senegal |
| 1995 | 20631.8836265311 | 0.264438773208067 | 1371.31562498491 | 19260.5680015462 | Senegal |
| 1996 | 19699.3069605607 | 0.268318964653468 | 1282.63772740507 | 18416.6692331556 | Senegal |
| 1997 | 18050.0317709512 | 0.272072875469797 | 1231.84333353023 | 16818.188437421 | Senegal |
| 1998 | 17111.8348155783 | 0.275995656114005 | 1196.45460550278 | 15915.3802100755 | Senegal |
| 1999 | 16274.1560752336 | 0.280111918035525 | 1198.42037718024 | 15075.7356980534 | Senegal |
| 2000 | 14464.8237284926 | 0.283784902973132 | 1195.98369947958 | 13268.840029013 | Senegal |
| 2001 | 16267.456743 | 0.287451899345797 | 1126.44614642616 | 15141.0105965738 | Senegal |
| 2002 | 14490.5740647397 | 0.291046287851898 | 1152.66006970793 | 13337.9139950318 | Senegal |
| 2003 | 12343.452831932 | 0.294887172086492 | 1086.60753079634 | 11256.8453011357 | Senegal |
| 2004 | 12479.7652009447 | 0.298984520918874 | 1074.29793103001 | 11405.4672699147 | Senegal |
| 2005 | 11773.0374201656 | 0.30325720904165 | 1027.45642404576 | 10745.5809961198 | Senegal |
| 2006 | 9976.19955025977 | 0.307633910231713 | 949.152319380194 | 9027.04723087958 | Senegal |
| 2007 | 9052.40494068796 | 0.312570297567491 | 969.603693288003 | 8082.80124739996 | Senegal |
| 2008 | 8120.18708969154 | 0.317894088643487 | 835.857609548457 | 7284.32948014308 | Senegal |
| 2009 | 7198.61557121762 | 0.323657725834671 | 770.518156069145 | 6428.09741514848 | Senegal |
| 2010 | 6031.94561360097 | 0.329804072240346 | 747.119638897525 | 5284.82597470345 | Senegal |
| 2011 | 5360.82230782844 | 0.336059897035461 | 673.311589026679 | 4687.51071880176 | Senegal |
| 2012 | 4414.09596410001 | 0.34297711398143 | 594.89695223655 | 3819.19901186346 | Senegal |
| 2013 | 3927.15852772708 | 0.350134004435728 | 598.083546636299 | 3329.07498109078 | Senegal |
| 2014 | 3608.01934944328 | 0.357181573156172 | 591.146284140907 | 3016.87306530237 | Senegal |
| 2015 | 3116.10743584537 | 0.364144496085842 | 583.278962181083 | 2532.82847366429 | Senegal |
| 2016 | 3055.4568219864 | 0.371114525115408 | 539.778821803113 | 2515.67800018329 | Senegal |
| 2017 | 2818.90215994699 | 0.378351476689464 | 555.670200645802 | 2263.23195930119 | Senegal |
| 2018 | 2579.44776956762 | 0.386079665694996 | 510.807040694856 | 2068.64072887276 | Senegal |
| 2019 | 2353.59613792807 | 0.394012316088147 | 474.263790172167 | 1879.3323477559 | Senegal |
| 2020 | 2083.30294131685 | 0.401197515620742 | 462.542617756606 | 1620.76032356024 | Senegal |
| 2021 | 1816.58073195319 | 0.408054192634391 | 307.81935366058 | 1508.76137829261 | Senegal |
| 1990 | 14081.1403899372 | 0.211569335210502 | 2301.8164437128 | 11779.3239462244 | Sierra Leone |
| 1991 | 13440.6802156087 | 0.213013566916176 | 2297.01413398974 | 11143.666081619 | Sierra Leone |
| 1992 | 13839.7059603624 | 0.214223040293117 | 2316.38647224826 | 11523.3194881141 | Sierra Leone |
| 1993 | 14087.9820770646 | 0.216089200983916 | 2311.88485182552 | 11776.0972252391 | Sierra Leone |
| 1994 | 14876.995984838 | 0.218277410522878 | 2296.51346408621 | 12580.4825207518 | Sierra Leone |
| 1995 | 13927.5417039635 | 0.219822603796338 | 2154.23568016034 | 11773.3060238032 | Sierra Leone |
| 1996 | 12582.6708141924 | 0.219969269802127 | 2126.200502266 | 10456.4703119264 | Sierra Leone |
| 1997 | 11974.5699649152 | 0.219089790932757 | 2300.77134033079 | 9673.79862458441 | Sierra Leone |
| 1998 | 11964.4417641033 | 0.218584312916824 | 2305.03454145483 | 9659.40722264847 | Sierra Leone |
| 1999 | 12140.0722859774 | 0.217943041857131 | 2303.7540695394 | 9836.318216438 | Sierra Leone |
| 2000 | 12368.0529880961 | 0.218492836742255 | 2305.68166105817 | 10062.3713270379 | Sierra Leone |
| 2001 | 11567.2294414403 | 0.22039123267412 | 2132.60778680105 | 9434.62165463925 | Sierra Leone |
| 2002 | 10588.8438781262 | 0.22458582518118 | 2162.07463177627 | 8426.76924634993 | Sierra Leone |
| 2003 | 9835.1692950733 | 0.229235900070887 | 1939.20186816621 | 7895.96742690709 | Sierra Leone |
| 2004 | 10351.4111632229 | 0.234131673808901 | 1924.89777905272 | 8426.51338417018 | Sierra Leone |
| 2005 | 9687.79059861622 | 0.23954993975681 | 1695.82973132938 | 7991.96086728684 | Sierra Leone |
| 2006 | 9149.28930391211 | 0.245647886413805 | 1704.62269389971 | 7444.6666100124 | Sierra Leone |
| 2007 | 8311.64225693114 | 0.252523076983211 | 1527.29765456266 | 6784.34460236848 | Sierra Leone |
| 2008 | 7510.69783704574 | 0.259781562101124 | 1383.58769781156 | 6127.11013923418 | Sierra Leone |
| 2009 | 6295.65897410027 | 0.267058380231042 | 1254.93208189486 | 5040.72689220541 | Sierra Leone |
| 2010 | 5966.84722504656 | 0.274270274980285 | 1251.48539947483 | 4715.36182557173 | Sierra Leone |
| 2011 | 5454.95845619983 | 0.28167357489027 | 1195.81235742582 | 4259.14609877401 | Sierra Leone |
| 2012 | 5312.53644829476 | 0.290338511565667 | 1139.39279155149 | 4173.14365674327 | Sierra Leone |
| 2013 | 4559.83608572596 | 0.300736693229616 | 1030.74127457363 | 3529.09481115233 | Sierra Leone |
| 2014 | 3675.00913085685 | 0.310348867321001 | 958.704519121275 | 2716.30461173557 | Sierra Leone |
| 2015 | 3329.42450039824 | 0.316998649992998 | 825.595247874796 | 2503.82925252344 | Sierra Leone |
| 2016 | 3125.28736268165 | 0.324091564628372 | 765.297970187649 | 2359.989392494 | Sierra Leone |
| 2017 | 2967.013793791 | 0.331213431016392 | 672.526396773541 | 2294.48739701746 | Sierra Leone |
| 2018 | 2707.4903762539 | 0.338341563502228 | 596.569261421639 | 2110.92111483226 | Sierra Leone |
| 2019 | 2497.00634876923 | 0.345770018534214 | 595.457678679091 | 1901.54867009014 | Sierra Leone |
| 2020 | 2289.85871518296 | 0.352412021842938 | 592.476647683551 | 1697.38206749941 | Sierra Leone |
| 2021 | 2101.96077633325 | 0.3586658807038 | 587.21236814471 | 1514.74840818854 | Sierra Leone |
| 1990 | 12033.37104555 | 0.269692273286049 | 1232.87535065809 | 10800.4956948919 | Togo |
| 1991 | 11916.4705770555 | 0.274530567688902 | 1244.50002544534 | 10671.9705516102 | Togo |
| 1992 | 11493.575167916 | 0.278789310483021 | 1197.76584041001 | 10295.809327506 | Togo |
| 1993 | 11184.218936037 | 0.281224630552395 | 1206.38129764372 | 9977.83763839328 | Togo |
| 1994 | 10879.2678706526 | 0.284653387233478 | 1130.78089986423 | 9748.48697078837 | Togo |
| 1995 | 10459.9489956075 | 0.288536691543625 | 1135.85287766349 | 9324.096117944 | Togo |
| 1996 | 10028.3845545986 | 0.292606677265023 | 1093.09823238429 | 8935.28632221431 | Togo |
| 1997 | 9545.6350369743 | 0.296947835200193 | 1089.91422006871 | 8455.72081690559 | Togo |
| 1998 | 9749.12448803089 | 0.300022332903466 | 1028.67407530935 | 8720.45041272154 | Togo |
| 1999 | 9004.87153901184 | 0.303020232352969 | 1020.59163076684 | 7984.279908245 | Togo |
| 2000 | 8784.36235759376 | 0.305472963037407 | 1024.03765649639 | 7760.32470109737 | Togo |
| 2001 | 8588.30905683558 | 0.307828257876202 | 972.573240614638 | 7615.73581622094 | Togo |
| 2002 | 9061.572568393 | 0.310469909626286 | 968.029905833483 | 8093.54266255952 | Togo |
| 2003 | 8701.53749055031 | 0.313792063910754 | 971.478478995694 | 7730.05901155462 | Togo |
| 2004 | 8345.4382836901 | 0.316687944336242 | 846.331830031766 | 7499.10645365833 | Togo |
| 2005 | 8024.87541324235 | 0.319025579582374 | 852.253846026753 | 7172.6215672156 | Togo |
| 2006 | 7829.60626679473 | 0.321659389933158 | 823.535205549576 | 7006.07106124515 | Togo |
| 2007 | 7267.35171531494 | 0.324382497112276 | 765.102889980024 | 6502.24882533492 | Togo |
| 2008 | 7012.13761176786 | 0.327743136228821 | 752.844776891126 | 6259.29283487673 | Togo |
| 2009 | 6953.51535282924 | 0.331558542074185 | 666.740180982183 | 6286.77517184706 | Togo |
| 2010 | 6558.12826178149 | 0.335899583453674 | 666.722245972034 | 5891.40601580946 | Togo |
| 2011 | 6257.1538256857 | 0.340777259848227 | 601.761490126588 | 5655.39233555911 | Togo |
| 2012 | 5727.04185939485 | 0.346320495474737 | 590.085268253158 | 5136.95659114169 | Togo |
| 2013 | 5170.63857371619 | 0.352278604950937 | 586.009049568222 | 4584.62952414797 | Togo |
| 2014 | 4412.9165580255 | 0.358661442459501 | 595.163391836233 | 3817.75316618927 | Togo |
| 2015 | 4071.00997478021 | 0.365600168512118 | 576.007292498253 | 3495.00268228196 | Togo |
| 2016 | 3869.52964306703 | 0.372910027688811 | 546.228928009633 | 3323.3007150574 | Togo |
| 2017 | 3629.39688229587 | 0.380336231070946 | 509.929781195536 | 3119.46710110033 | Togo |
| 2018 | 3241.02299354389 | 0.387921228739501 | 509.067622416763 | 2731.95537112713 | Togo |
| 2019 | 3043.82380700973 | 0.395667268909215 | 464.08581194852 | 2579.73799506121 | Togo |
| 2020 | 2738.37730382054 | 0.402356922397572 | 396.755810418571 | 2341.62149340197 | Togo |
| 2021 | 2467.72426701859 | 0.408533695477986 | 309.523692303615 | 2158.20057471498 | Togo |
| 1990 | 238.86964057827 | 0.613633923933335 | 13.3925905146744 | 225.477050063596 | American Samoa |
| 1991 | 233.78231445577 | 0.615847611881171 | 12.9441014268051 | 220.838213028965 | American Samoa |
| 1992 | 228.20212025941 | 0.618583654206334 | 12.8579165831079 | 215.344203676302 | American Samoa |
| 1993 | 222.96229514856 | 0.622078597664072 | 13.0259412765633 | 209.936353871997 | American Samoa |
| 1994 | 216.61719291057 | 0.626053240674018 | 12.9761474686151 | 203.641045441955 | American Samoa |
| 1995 | 210.74696788224 | 0.629814053909773 | 12.0316498154989 | 198.715318066741 | American Samoa |
| 1996 | 206.19305005407 | 0.632706982787519 | 11.1899621083524 | 195.003087945718 | American Samoa |
| 1997 | 198.63738194106 | 0.634926685456248 | 11.1482521753501 | 187.48912976571 | American Samoa |
| 1998 | 196.10367585714 | 0.636597975713911 | 10.356398708725 | 185.747277148415 | American Samoa |
| 1999 | 191.40606293497 | 0.638322193134778 | 8.48397019328308 | 182.922092741687 | American Samoa |
| 2000 | 187.03486442002 | 0.640609550029113 | 8.23648255313518 | 178.798381866885 | American Samoa |
| 2001 | 183.47823174106 | 0.643214421156583 | 8.00840987947016 | 175.46982186159 | American Samoa |
| 2002 | 182.95225341083 | 0.646002062148026 | 5.5679088682975 | 177.384344542532 | American Samoa |
| 2003 | 180.58811606642 | 0.648951964151548 | 6.0979373272964 | 174.490178739124 | American Samoa |
| 2004 | 177.89313852935 | 0.651921140709413 | 4.88297452271412 | 173.010164006636 | American Samoa |
| 2005 | 174.09718470305 | 0.655061005497018 | 5.46048068940684 | 168.636704013643 | American Samoa |
| 2006 | 171.46394678809 | 0.657725722991147 | 4.62217211564 | 166.84177467245 | American Samoa |
| 2007 | 167.4937164923 | 0.660781175742006 | 4.51979796857821 | 162.973918523722 | American Samoa |
| 2008 | 163.83007373625 | 0.664025761610397 | 4.52942007427273 | 159.300653661977 | American Samoa |
| 2009 | 164.70324952108 | 0.667300532067021 | 3.65708806244165 | 161.046161458638 | American Samoa |
| 2010 | 164.51222844729 | 0.671248915725392 | 3.28519238659824 | 161.227036060692 | American Samoa |
| 2011 | 164.32197139261 | 0.675979288249651 | 2.92999072688718 | 161.391980665723 | American Samoa |
| 2012 | 164.28770626499 | 0.680861538744503 | 2.8034419874068 | 161.484264277583 | American Samoa |
| 2013 | 165.02477858427 | 0.685833061049061 | 2.62089968065062 | 162.403878903619 | American Samoa |
| 2014 | 166.60682207534 | 0.691343136814841 | 2.4584627217311 | 164.148359353609 | American Samoa |
| 2015 | 170.40722153559 | 0.697162912183353 | 2.40423467842906 | 168.002986857161 | American Samoa |
| 2016 | 174.93136127542 | 0.702726397771802 | 2.34581724489726 | 172.585544030523 | American Samoa |
| 2017 | 175.76903899179 | 0.707647909217363 | 2.27699663717451 | 173.492042354616 | American Samoa |
| 2018 | 175.34692835709 | 0.712800591003034 | 2.20733499251731 | 173.139593364573 | American Samoa |
| 2019 | 173.68774407511 | 0.717308133053242 | 2.19067415038113 | 171.497069924729 | American Samoa |
| 2020 | 188.46437131995 | 0.720838500394591 | 2.19020007297 | 186.27417124698 | American Samoa |
| 2021 | 169.92032279797 | 0.723727533059592 | 2.17860803546426 | 167.741714762506 | American Samoa |
| 1990 | 21.62105856724 | 0.696451195907995 | 2.41625357862 | 19.20480498862 | Bermuda |
| 1991 | 19.30829892561 | 0.700276851342371 | 2.34978040626368 | 16.9585185193463 | Bermuda |
| 1992 | 18.58122131714 | 0.703984094837886 | 2.2725460856139 | 16.3086752315261 | Bermuda |
| 1993 | 18.69335492047 | 0.707678222374744 | 2.30444506687396 | 16.388909853596 | Bermuda |
| 1994 | 16.91760159097 | 0.711068997576994 | 2.20897040594588 | 14.7086311850241 | Bermuda |
| 1995 | 15.27188317839 | 0.714468828293519 | 2.25003446364403 | 13.021848714746 | Bermuda |
| 1996 | 13.60479984819 | 0.71783167141956 | 2.2020201697363 | 11.4027796784537 | Bermuda |
| 1997 | 12.13949595915 | 0.721642812571346 | 2.17606381096114 | 9.96343214818886 | Bermuda |
| 1998 | 10.51121875918 | 0.725853528350146 | 2.17043522972761 | 8.34078352945239 | Bermuda |
| 1999 | 8.88134587551 | 0.730539822974827 | 2.15726777117698 | 6.72407810433302 | Bermuda |
| 2000 | 7.52436958747 | 0.73599650207483 | 2.17285948013018 | 5.35151010733982 | Bermuda |
| 2001 | 7.01603039576 | 0.741918907664508 | 2.12088852627792 | 4.89514186948208 | Bermuda |
| 2002 | 6.8772286175 | 0.747544503099499 | 2.04646259139333 | 4.83076602610667 | Bermuda |
| 2003 | 6.92540844207 | 0.753305163354703 | 2.02597831902442 | 4.89943012304558 | Bermuda |
| 2004 | 7.02255731584 | 0.759008884830255 | 1.93769982444686 | 5.08485749139314 | Bermuda |
| 2005 | 7.31975539371 | 0.764690902756676 | 1.76303299553833 | 5.55672239817167 | Bermuda |
| 2006 | 7.36963476824 | 0.770596769707219 | 1.64029478364 | 5.7293399846 | Bermuda |
| 2007 | 7.14465244319 | 0.776114264028476 | 1.52105767317439 | 5.62359477001561 | Bermuda |
| 2008 | 6.88721043788 | 0.780358325453696 | 1.48859808316047 | 5.39861235471954 | Bermuda |
| 2009 | 6.51436093247 | 0.7847591074268 | 1.45867630503918 | 5.05568462743082 | Bermuda |
| 2010 | 6.26481084108 | 0.789181466953459 | 1.47396782275278 | 4.79084301832722 | Bermuda |
| 2011 | 6.14408111646 | 0.793380523639009 | 1.45251173520139 | 4.69156938125861 | Bermuda |
| 2012 | 5.9404108067 | 0.797484869823732 | 1.41246427248283 | 4.52794653421717 | Bermuda |
| 2013 | 5.82601977241 | 0.801388200821251 | 1.43515858783805 | 4.39086118457195 | Bermuda |
| 2014 | 5.72446893248 | 0.804923406558971 | 1.46479686851448 | 4.25967206396552 | Bermuda |
| 2015 | 5.51159998261 | 0.807998794744826 | 1.50150662524324 | 4.01009335736676 | Bermuda |
| 2016 | 5.37545762369 | 0.810550842307499 | 1.46126092007935 | 3.91419670361065 | Bermuda |
| 2017 | 5.15283053354 | 0.812807228761017 | 1.44681894097432 | 3.70601159256568 | Bermuda |
| 2018 | 4.93192124867 | 0.814892801994945 | 1.41356292910591 | 3.51835831956409 | Bermuda |
| 2019 | 4.82162492251 | 0.817000338369953 | 1.41272792632222 | 3.40889699618778 | Bermuda |
| 2020 | 4.71155480233 | 0.819203419907163 | 1.40441308224 | 3.30714172009 | Bermuda |
| 2021 | 4.9093232808 | 0.821365422389484 | 1.44422772064786 | 3.46509556015214 | Bermuda |
| 1990 | 60.69322350978 | 0.564514853922858 | 16.2700073404643 | 44.4232161693157 | Cook Islands |
| 1991 | 58.596737069 | 0.570622775576656 | 12.778741501276 | 45.817995567724 | Cook Islands |
| 1992 | 56.73662268053 | 0.57712346797279 | 13.4125131475944 | 43.3241095329356 | Cook Islands |
| 1993 | 55.32916291148 | 0.584059175401459 | 13.1756874651064 | 42.1534754463736 | Cook Islands |
| 1994 | 54.52315236009 | 0.591540867591394 | 13.2245125290834 | 41.2986398310066 | Cook Islands |
| 1995 | 54.07712359461 | 0.598789967711714 | 13.2861549796706 | 40.7909686149394 | Cook Islands |
| 1996 | 53.27861604365 | 0.606414151024421 | 13.4503685397962 | 39.8282475038538 | Cook Islands |
| 1997 | 51.75489841089 | 0.613991564375745 | 13.2417900721876 | 38.5131083387024 | Cook Islands |
| 1998 | 50.71369329549 | 0.621202227562338 | 13.0160909925594 | 37.6976023029306 | Cook Islands |
| 1999 | 51.56203128576 | 0.628029029838686 | 12.5064984902729 | 39.0555327954871 | Cook Islands |
| 2000 | 50.4511831554 | 0.635365607290649 | 10.9143597738306 | 39.5368233815694 | Cook Islands |
| 2001 | 49.0560278608 | 0.644421442097654 | 5.97629017268407 | 43.0797376881159 | Cook Islands |
| 2002 | 47.75893026712 | 0.652790488145509 | 4.95804533258347 | 42.8008849345365 | Cook Islands |
| 2003 | 46.73600738274 | 0.661465037633189 | 4.57396257807389 | 42.1620448046661 | Cook Islands |
| 2004 | 45.44197503302 | 0.668769675110248 | 3.59542699575025 | 41.8465480372698 | Cook Islands |
| 2005 | 45.20752639475 | 0.67400410252196 | 3.36638074679857 | 41.8411456479514 | Cook Islands |
| 2006 | 44.78775290905 | 0.680644001453893 | 2.76505102610041 | 42.0227018829496 | Cook Islands |
| 2007 | 44.70795248151 | 0.687505190649195 | 2.42099572261149 | 42.2869567588985 | Cook Islands |
| 2008 | 43.18580307372 | 0.695592068858646 | 2.48090025859164 | 40.7049028151284 | Cook Islands |
| 2009 | 41.56535075447 | 0.70377697980234 | 2.33168551640018 | 39.2336652380698 | Cook Islands |
| 2010 | 40.632054145 | 0.711861611963853 | 2.21589889018277 | 38.4161552548172 | Cook Islands |
| 2011 | 39.09261732901 | 0.719444665855079 | 2.22549875538217 | 36.8671185736278 | Cook Islands |
| 2012 | 37.19579073811 | 0.726963385157533 | 2.16650252668486 | 35.0292882114251 | Cook Islands |
| 2013 | 35.00586903561 | 0.73331701541165 | 2.16285952348147 | 32.8430095121285 | Cook Islands |
| 2014 | 34.22223340647 | 0.73959570406711 | 2.17450934201487 | 32.0477240644551 | Cook Islands |
| 2015 | 34.12645459946 | 0.746550296501143 | 2.07914275156879 | 32.0473118478912 | Cook Islands |
| 2016 | 34.18869790009 | 0.752353086814977 | 2.01169751121447 | 32.1770003888755 | Cook Islands |
| 2017 | 33.43242509245 | 0.758233571650419 | 1.94117482746343 | 31.4912502649866 | Cook Islands |
| 2018 | 32.79241984819 | 0.764041181894148 | 1.67318258675706 | 31.1192372614329 | Cook Islands |
| 2019 | 32.18535540849 | 0.769795912507863 | 1.62548677438455 | 30.5598686341055 | Cook Islands |
| 2020 | 49.77627206381 | 0.774700577781853 | 1.52799824450279 | 48.2482738193072 | Cook Islands |
| 2021 | 51.52556226211 | 0.779109954659683 | 1.5084592340771 | 50.0171030280329 | Cook Islands |
| 1990 | 6.69679929678 | 0.732258251665998 | 2.13747767874406 | 4.55932161803594 | Greenland |
| 1991 | 6.41159558499 | 0.730626532070962 | 2.1575002055887 | 4.2540953794013 | Greenland |
| 1992 | 5.94857262094 | 0.729781103358168 | 2.19138103518031 | 3.75719158575969 | Greenland |
| 1993 | 5.64106129871 | 0.729894841568107 | 2.18106296585357 | 3.45999833285643 | Greenland |
| 1994 | 5.29942860049 | 0.730875896131236 | 2.15426195016914 | 3.14516665032086 | Greenland |
| 1995 | 5.05369390455 | 0.73083163848634 | 2.15588833680771 | 2.89780556774229 | Greenland |
| 1996 | 4.82615265841 | 0.730216225093773 | 2.20668833146 | 2.61946432695 | Greenland |
| 1997 | 4.60122529036 | 0.730551086919016 | 2.18061235448711 | 2.42061293587289 | Greenland |
| 1998 | 4.40937469814 | 0.733242587548811 | 2.18742408238714 | 2.22195061575286 | Greenland |
| 1999 | 4.25392622689 | 0.736557130056076 | 2.17171657778326 | 2.08220964910674 | Greenland |
| 2000 | 4.14973420415 | 0.740857009793347 | 2.11165945785673 | 2.03807474629326 | Greenland |
| 2001 | 4.12672097451 | 0.746289605484857 | 2.04450149772023 | 2.08221947678977 | Greenland |
| 2002 | 4.15949950413 | 0.752878370778087 | 2.05076499589111 | 2.10873450823889 | Greenland |
| 2003 | 4.17284704748 | 0.759444151937822 | 1.914182443091 | 2.258664604389 | Greenland |
| 2004 | 4.12032225383 | 0.765401567469496 | 1.6928955343724 | 2.4274267194576 | Greenland |
| 2005 | 4.10520305012 | 0.772099579848736 | 1.56866910114943 | 2.53653394897057 | Greenland |
| 2006 | 4.06788804045 | 0.779141406404636 | 1.47512262100107 | 2.59276541944893 | Greenland |
| 2007 | 4.06832537043 | 0.785321921636087 | 1.44099874562595 | 2.62732662480405 | Greenland |
| 2008 | 4.06190630702 | 0.79144125736451 | 1.40806736305421 | 2.65383894396579 | Greenland |
| 2009 | 3.96930785139 | 0.797025750029497 | 1.46871273820794 | 2.50059511318206 | Greenland |
| 2010 | 3.90770412443 | 0.803003131492839 | 1.43304169603595 | 2.47466242839405 | Greenland |
| 2011 | 3.83941159368 | 0.808105185095196 | 1.39442636638 | 2.4449852273 | Greenland |
| 2012 | 3.69810775991 | 0.811684561566459 | 1.4852437710878 | 2.2128639888222 | Greenland |
| 2013 | 3.64944319188 | 0.814368307545592 | 1.436806692291 | 2.212636499589 | Greenland |
| 2014 | 3.51588980249 | 0.816527059982666 | 1.441449994235 | 2.074439808255 | Greenland |
| 2015 | 3.54349023544 | 0.8177191894267 | 1.41881852557595 | 2.12467170986405 | Greenland |
| 2016 | 3.39535046913 | 0.818961901690003 | 1.44822903975846 | 1.94712142937154 | Greenland |
| 2017 | 3.20244503478 | 0.819781100917251 | 1.45617043894394 | 1.74627459583606 | Greenland |
| 2018 | 2.98962978918 | 0.820815513545804 | 1.4572615274188 | 1.5323682617612 | Greenland |
| 2019 | 2.81845890721 | 0.822339996424292 | 1.41669780620167 | 1.40176110100833 | Greenland |
| 2020 | 2.95681326216 | 0.824359028380898 | 1.46944742777417 | 1.48736583438583 | Greenland |
| 2021 | 2.54311090489 | 0.82621033634361 | 1.43360554604128 | 1.10950535884872 | Greenland |
| 1990 | 99.86434138124 | 0.67622030520232 | 2.98152644126544 | 96.8828149399746 | Guam |
| 1991 | 100.93045193933 | 0.669974291504161 | 3.66719616017949 | 97.2632557791505 | Guam |
| 1992 | 102.1014058609 | 0.666356340381872 | 3.60397850170988 | 98.4974273591901 | Guam |
| 1993 | 101.67731963522 | 0.667318181427308 | 3.54895907349 | 98.12836056173 | Guam |
| 1994 | 98.98089244505 | 0.672111711813636 | 3.28388699834746 | 95.6970054467025 | Guam |
| 1995 | 96.0725650347 | 0.678120215047177 | 2.90078925326103 | 93.171775781439 | Guam |
| 1996 | 93.49189808683 | 0.68417373470043 | 2.69499774397622 | 90.7969003428538 | Guam |
| 1997 | 90.40756206616 | 0.691684870710781 | 2.56232524193284 | 87.8452368242272 | Guam |
| 1998 | 88.62060724375 | 0.701819289007173 | 2.3717030104319 | 86.2489042333181 | Guam |
| 1999 | 86.96696351175 | 0.713641593327183 | 2.22778980556192 | 84.7391737061881 | Guam |
| 2000 | 85.42641555201 | 0.724802357160022 | 2.16853956423724 | 83.2578759877728 | Guam |
| 2001 | 85.03825266458 | 0.734267375715648 | 2.16360015688418 | 82.8746525076958 | Guam |
| 2002 | 86.70523629052 | 0.741284245225143 | 2.09326277764919 | 84.6119735128708 | Guam |
| 2003 | 90.27714135179 | 0.745911530185679 | 2.08747459723878 | 88.1896667545512 | Guam |
| 2004 | 94.20836403301 | 0.749016519617639 | 2.04916273628862 | 92.1592012967214 | Guam |
| 2005 | 98.21832579119 | 0.750865874718511 | 2.05044365357914 | 96.1678821376109 | Guam |
| 2006 | 102.27905319583 | 0.75117624504969 | 2.02750730193202 | 100.251545893898 | Guam |
| 2007 | 105.52686489461 | 0.752625470789067 | 2.01808809922577 | 103.508776795384 | Guam |
| 2008 | 107.55932017448 | 0.755152408307309 | 2.01846969226 | 105.54085048222 | Guam |
| 2009 | 110.58583651045 | 0.75825229605245 | 1.87385779836027 | 108.71197871209 | Guam |
| 2010 | 113.58101725225 | 0.761484317334794 | 1.67613178437883 | 111.904885467871 | Guam |
| 2011 | 116.1983948844 | 0.763987394174646 | 1.77528680519964 | 114.4231080792 | Guam |
| 2012 | 118.44535034136 | 0.766845984308226 | 1.68043926744259 | 116.764911073917 | Guam |
| 2013 | 120.24473506015 | 0.770224956660541 | 1.54347175353902 | 118.701263306611 | Guam |
| 2014 | 122.47365035462 | 0.773441684088001 | 1.63251442973485 | 120.841135924885 | Guam |
| 2015 | 124.85679278617 | 0.776723614176227 | 1.58454814261738 | 123.272244643553 | Guam |
| 2016 | 125.29120327861 | 0.780511644317696 | 1.45477190351447 | 123.836431375096 | Guam |
| 2017 | 122.55622424565 | 0.78555492004746 | 1.46401075274145 | 121.092213492909 | Guam |
| 2018 | 121.46563944227 | 0.791294772555245 | 1.46890440892236 | 119.996735033348 | Guam |
| 2019 | 119.25137516883 | 0.796775451912585 | 1.47110548114147 | 117.780269687689 | Guam |
| 2020 | 110.32382744199 | 0.800969472273547 | 1.47392200084104 | 108.849905441149 | Guam |
| 2021 | 107.42305603368 | 0.803982202863145 | 1.44287628870216 | 105.980179744978 | Guam |
| 1990 | 6.96124548168 | 0.845495153448926 | 1.36941559346655 | 5.59182988821345 | Monaco |
| 1991 | 7.08567036649 | 0.848303689705517 | 1.358760507774 | 5.726909858716 | Monaco |
| 1992 | 7.14489002408 | 0.850863939877657 | 1.36859877042132 | 5.77629125365868 | Monaco |
| 1993 | 7.32747993529 | 0.853264991769943 | 1.30344545991951 | 6.02403447537049 | Monaco |
| 1994 | 7.46060751152 | 0.855626926997873 | 1.29894706876733 | 6.16166044275267 | Monaco |
| 1995 | 7.59620774625 | 0.857986147163975 | 1.31213989344771 | 6.28406785280229 | Monaco |
| 1996 | 7.75224923324 | 0.860245431724109 | 1.29058396368095 | 6.46166526955905 | Monaco |
| 1997 | 7.90678367779 | 0.862446719992273 | 1.2725893476483 | 6.6341943301417 | Monaco |
| 1998 | 8.05459796263 | 0.8647165130785 | 1.251751437453 | 6.802846525177 | Monaco |
| 1999 | 8.16197804699 | 0.866842394455442 | 1.29287789708 | 6.86910014991 | Monaco |
| 2000 | 8.27008460915 | 0.868973583095515 | 1.25268946010026 | 7.01739514904974 | Monaco |
| 2001 | 8.34259997712 | 0.871094754955376 | 1.27958715408235 | 7.06301282303765 | Monaco |
| 2002 | 8.43652245385 | 0.873182768875724 | 1.27358194980463 | 7.16294050404537 | Monaco |
| 2003 | 8.51459232742 | 0.875197652216285 | 1.28053652233979 | 7.23405580508021 | Monaco |
| 2004 | 8.59636585938 | 0.87723154881107 | 1.27706741459938 | 7.31929844478063 | Monaco |
| 2005 | 8.66288453199 | 0.879288088865188 | 1.2834227336515 | 7.3794617983385 | Monaco |
| 2006 | 8.75359750539 | 0.881274148230096 | 1.26493743821471 | 7.48866006717529 | Monaco |
| 2007 | 8.8764187831 | 0.883262291250478 | 1.23087922389295 | 7.64553955920705 | Monaco |
| 2008 | 8.961788966 | 0.885331393874596 | 1.26644670225827 | 7.69534226374173 | Monaco |
| 2009 | 9.05326443378 | 0.887328278515505 | 1.28403284544528 | 7.76923158833472 | Monaco |
| 2010 | 9.09300340793 | 0.889262841317756 | 1.27083871282079 | 7.82216469510921 | Monaco |
| 2011 | 9.08081250891 | 0.891237592262334 | 1.2674439747561 | 7.8133685341539 | Monaco |
| 2012 | 9.01663331941 | 0.893073821857862 | 1.26895809172581 | 7.74767522768419 | Monaco |
| 2013 | 8.89192946178 | 0.89486447196548 | 1.2736108583313 | 7.6183186034487 | Monaco |
| 2014 | 8.74613180513 | 0.896547873900622 | 1.31208689114417 | 7.43404491398583 | Monaco |
| 2015 | 8.57364624152 | 0.89835633653211 | 1.27454872924088 | 7.29909751227912 | Monaco |
| 2016 | 8.1239832213 | 0.900051302707716 | 1.26164701881796 | 6.86233620248204 | Monaco |
| 2017 | 7.38650165476 | 0.901752711895654 | 1.26979868562632 | 6.11670296913368 | Monaco |
| 2018 | 6.64184348421 | 0.903388686597922 | 1.26893203856791 | 5.37291144564209 | Monaco |
| 2019 | 6.24836903525 | 0.905030990844668 | 1.26462554185911 | 4.98374349339089 | Monaco |
| 2020 | 6.95593556638 | 0.90668543677348 | 1.24927721016091 | 5.70665835621909 | Monaco |
| 2021 | 6.7983069496 | 0.908262830989574 | 1.24612803367167 | 5.55217891592833 | Monaco |
| 1990 | 620.98674356456 | 0.539145980867897 | 21.2778665387666 | 599.708877025793 | Nauru |
| 1991 | 616.57006505994 | 0.538433557023675 | 21.2562746569665 | 595.313790402973 | Nauru |
| 1992 | 612.31665344199 | 0.537038892847501 | 21.2767965425602 | 591.03985689943 | Nauru |
| 1993 | 623.28330215276 | 0.535059741994823 | 21.5024040336874 | 601.780898119073 | Nauru |
| 1994 | 635.91747123319 | 0.533001096624389 | 22.1989193730176 | 613.718551860172 | Nauru |
| 1995 | 649.05103970513 | 0.530890925037636 | 22.1231334696701 | 626.92790623546 | Nauru |
| 1996 | 663.71067056838 | 0.528544473806068 | 22.0827688053421 | 641.627901763038 | Nauru |
| 1997 | 664.34065878271 | 0.526241589925116 | 22.1129843879284 | 642.227674394782 | Nauru |
| 1998 | 696.68533873015 | 0.523885302498396 | 22.1452439665895 | 674.54009476356 | Nauru |
| 1999 | 710.05040512177 | 0.521894324468662 | 22.1668055302933 | 687.883599591477 | Nauru |
| 2000 | 721.13119459687 | 0.52025488766476 | 22.1878099173447 | 698.943384679525 | Nauru |
| 2001 | 722.34977065475 | 0.519235424968716 | 22.4115191658743 | 699.938251488876 | Nauru |
| 2002 | 709.9940776223 | 0.518438705224942 | 22.2176253074462 | 687.776452314854 | Nauru |
| 2003 | 684.7844575682 | 0.518459986012372 | 22.2074436085882 | 662.577013959612 | Nauru |
| 2004 | 675.74059542818 | 0.518809540150096 | 22.2443655982436 | 653.496229829936 | Nauru |
| 2005 | 659.66227197416 | 0.51989286311802 | 22.1632984833084 | 637.498973490852 | Nauru |
| 2006 | 635.20714736929 | 0.522673178762107 | 22.1656013187508 | 613.041546050539 | Nauru |
| 2007 | 619.89879892473 | 0.523201350232829 | 22.0963789869964 | 597.802419937734 | Nauru |
| 2008 | 583.02962928457 | 0.526084096271176 | 22.0906081368256 | 560.939021147744 | Nauru |
| 2009 | 579.12291991265 | 0.530221091913464 | 22.1236391574874 | 556.999280755163 | Nauru |
| 2010 | 562.54894842071 | 0.535689994782251 | 21.5749498318879 | 540.973998588822 | Nauru |
| 2011 | 535.97831196762 | 0.542342742706768 | 21.1644556772976 | 514.813856290322 | Nauru |
| 2012 | 507.71857575324 | 0.549890620999273 | 19.6019867102663 | 488.116589042974 | Nauru |
| 2013 | 468.37172782994 | 0.560112906115882 | 17.4203480970708 | 450.951379732869 | Nauru |
| 2014 | 421.24308915433 | 0.571424363669984 | 13.348009378403 | 407.895079775927 | Nauru |
| 2015 | 395.183014384 | 0.581283234356014 | 13.5782084348206 | 381.604805949179 | Nauru |
| 2016 | 376.58885656117 | 0.590102988337037 | 13.5502240782667 | 363.038632482903 | Nauru |
| 2017 | 352.05305121237 | 0.597619075639454 | 13.8814162174967 | 338.171634994873 | Nauru |
| 2018 | 330.63952409624 | 0.605311878971834 | 13.3058320165314 | 317.333692079709 | Nauru |
| 2019 | 317.85307235454 | 0.612697252950554 | 13.3678801420948 | 304.485192212445 | Nauru |
| 2020 | 301.75821827212 | 0.619330076183652 | 13.0254121834569 | 288.732806088663 | Nauru |
| 2021 | 287.39404865304 | 0.625177833565553 | 12.9243289089179 | 274.469719744122 | Nauru |
| 1990 | 370.96247407029 | 0.587532983713503 | 13.3298840728264 | 357.632589997464 | Niue |
| 1991 | 372.53457474823 | 0.593882056791176 | 13.2958021432283 | 359.238772605002 | Niue |
| 1992 | 372.46853924625 | 0.599881243015555 | 13.4748750349523 | 358.993664211298 | Niue |
| 1993 | 371.58989622475 | 0.605514778865402 | 13.2969116221312 | 358.292984602619 | Niue |
| 1994 | 372.93362128607 | 0.610650175126661 | 13.266552219009 | 359.667069067061 | Niue |
| 1995 | 373.6603590177 | 0.615374609096138 | 13.08350955824 | 360.57684945946 | Niue |
| 1996 | 377.41910604777 | 0.619734554803463 | 13.0309843997136 | 364.388121648056 | Niue |
| 1997 | 370.05469324499 | 0.623913847776035 | 12.9796948903844 | 357.074998354606 | Niue |
| 1998 | 385.20338058408 | 0.627578934289331 | 12.4581848148789 | 372.745195769201 | Niue |
| 1999 | 382.6190755601 | 0.63085717205161 | 12.1006036079596 | 370.51847195214 | Niue |
| 2000 | 375.62154438064 | 0.634068950171114 | 11.0593301996676 | 364.562214180972 | Niue |
| 2001 | 365.98665143562 | 0.637366627073445 | 8.13206776113704 | 357.854583674483 | Niue |
| 2002 | 347.61250775339 | 0.641122021248484 | 7.45697892047527 | 340.155528832915 | Niue |
| 2003 | 328.28779784875 | 0.645779539849075 | 5.72904293209884 | 322.558754916651 | Niue |
| 2004 | 313.11243121912 | 0.650614832382256 | 5.35371734634317 | 307.758713872777 | Niue |
| 2005 | 293.1004077532 | 0.657281264925745 | 4.54319138280812 | 288.557216370392 | Niue |
| 2006 | 276.09231564824 | 0.664162983137176 | 4.48157047499629 | 271.610745173244 | Niue |
| 2007 | 263.34892313697 | 0.670199299468215 | 3.52275315826579 | 259.826169978704 | Niue |
| 2008 | 252.78044849669 | 0.67639685901135 | 3.03479312728463 | 249.745655369405 | Niue |
| 2009 | 248.93841182481 | 0.682579172713086 | 2.87045269920475 | 246.067959125605 | Niue |
| 2010 | 250.58091683361 | 0.686280630120473 | 2.69344453579513 | 247.887472297815 | Niue |
| 2011 | 252.90059757107 | 0.690315577811745 | 2.6569504460958 | 250.243647124974 | Niue |
| 2012 | 252.52368792258 | 0.694150948675056 | 2.40088071652458 | 250.122807206055 | Niue |
| 2013 | 252.63491581531 | 0.697221077939175 | 2.48755217955485 | 250.147363635755 | Niue |
| 2014 | 251.24094882262 | 0.700545923914193 | 2.38434093681256 | 248.856607885807 | Niue |
| 2015 | 251.11941943906 | 0.704232979022297 | 2.3217417025148 | 248.797677736545 | Niue |
| 2016 | 221.58304915687 | 0.707476099423504 | 2.30462546657901 | 219.278423690291 | Niue |
| 2017 | 217.14783472199 | 0.711220995949878 | 2.23696200662245 | 214.910872715368 | Niue |
| 2018 | 212.75173161708 | 0.7151082296411 | 2.2189470995318 | 210.532784517548 | Niue |
| 2019 | 207.40836122041 | 0.719078885206029 | 2.22876971688533 | 205.179591503525 | Niue |
| 2020 | 550.78971367631 | 0.722790249813625 | 2.1646399816621 | 548.625073694648 | Niue |
| 2021 | 555.59507071752 | 0.726222049709539 | 2.19428313615225 | 553.400787581368 | Niue |
| 1990 | 93.25540659788 | 0.708593838127328 | 2.27456243630978 | 90.9808441615702 | Northern Mariana Islands |
| 1991 | 92.07620026354 | 0.712223642490096 | 2.25376059353134 | 89.8224396700087 | Northern Mariana Islands |
| 1992 | 88.43290846919 | 0.715787607029664 | 2.19470677324463 | 86.2382016959454 | Northern Mariana Islands |
| 1993 | 83.42856551803 | 0.719179395545622 | 2.20254341610778 | 81.2260221019222 | Northern Mariana Islands |
| 1994 | 78.49332216494 | 0.72251049388152 | 2.16687113037569 | 76.3264510345643 | Northern Mariana Islands |
| 1995 | 75.43785281279 | 0.726219053033259 | 2.18404076872688 | 73.2538120440631 | Northern Mariana Islands |
| 1996 | 75.94367376654 | 0.730074085649478 | 2.15308295257975 | 73.7905908139603 | Northern Mariana Islands |
| 1997 | 79.22348236794 | 0.733795365689826 | 2.17415183419648 | 77.0493305337435 | Northern Mariana Islands |
| 1998 | 83.96142682273 | 0.737423126925004 | 2.16321698848024 | 81.7982098342498 | Northern Mariana Islands |
| 1999 | 87.00388084648 | 0.740934815031098 | 2.07633908110324 | 84.9275417653768 | Northern Mariana Islands |
| 2000 | 87.08139536746 | 0.744492865066434 | 2.06994384578139 | 85.0114515216786 | Northern Mariana Islands |
| 2001 | 84.8626832158 | 0.747170605600551 | 2.06471133331753 | 82.7979718824825 | Northern Mariana Islands |
| 2002 | 80.73627067365 | 0.748755188793797 | 2.05234191385278 | 78.6839287597972 | Northern Mariana Islands |
| 2003 | 75.20314470548 | 0.750171533040464 | 2.07454614101722 | 73.1285985644628 | Northern Mariana Islands |
| 2004 | 71.34778699083 | 0.751528840504646 | 2.03204235692352 | 69.3157446339065 | Northern Mariana Islands |
| 2005 | 70.89956960564 | 0.751826692283402 | 2.03352312989739 | 68.8660464757426 | Northern Mariana Islands |
| 2006 | 74.03090059032 | 0.751797809045081 | 2.04295685155521 | 71.9879437387648 | Northern Mariana Islands |
| 2007 | 77.30872061528 | 0.751608416153259 | 2.03714192458667 | 75.2715786906933 | Northern Mariana Islands |
| 2008 | 78.88616896472 | 0.750807191804257 | 2.05660007104423 | 76.8295688936758 | Northern Mariana Islands |
| 2009 | 79.36900000686 | 0.748597173133935 | 2.05517627404557 | 77.3138237328144 | Northern Mariana Islands |
| 2010 | 77.68208034081 | 0.746617261581083 | 2.06136075634522 | 75.6207195844648 | Northern Mariana Islands |
| 2011 | 75.97002076489 | 0.744411908430807 | 2.09354929935705 | 73.876471465533 | Northern Mariana Islands |
| 2012 | 75.07465786867 | 0.742937350752288 | 2.08590684796085 | 72.9887510207092 | Northern Mariana Islands |
| 2013 | 75.32268736984 | 0.742284360325222 | 2.09969247824289 | 73.2229948915971 | Northern Mariana Islands |
| 2014 | 76.73272122857 | 0.742605449321406 | 2.08827061047817 | 74.6444506180918 | Northern Mariana Islands |
| 2015 | 79.53436949123 | 0.743797398071258 | 2.07444919383226 | 77.4599202973977 | Northern Mariana Islands |
| 2016 | 80.17118741627 | 0.748455465810463 | 2.05722429732627 | 78.1139631189437 | Northern Mariana Islands |
| 2017 | 77.71174730244 | 0.756495478910201 | 2.00929902513157 | 75.7024482773084 | Northern Mariana Islands |
| 2018 | 76.44143907684 | 0.761255400013642 | 1.77425837866775 | 74.6671806981722 | Northern Mariana Islands |
| 2019 | 74.7252300757 | 0.765770647172973 | 1.63520277698985 | 73.0900272987102 | Northern Mariana Islands |
| 2020 | 72.36398853492 | 0.76942765953698 | 1.56983317323403 | 70.794155361686 | Northern Mariana Islands |
| 2021 | 69.13228479859 | 0.771535213383281 | 1.52322531290063 | 67.6090594856894 | Northern Mariana Islands |
| 1990 | 730.65766720984 | 0.662909509792389 | 4.63255981265519 | 726.025107397185 | Palau |
| 1991 | 689.65921831671 | 0.668230731109012 | 3.70101683477915 | 685.958201481931 | Palau |
| 1992 | 650.43171916082 | 0.673302462400621 | 3.36220407070143 | 647.069515090119 | Palau |
| 1993 | 623.73579669626 | 0.676648715394758 | 3.21489597193326 | 620.520900724327 | Palau |
| 1994 | 597.92032248814 | 0.680195774851961 | 2.84331363694181 | 595.077008851198 | Palau |
| 1995 | 569.59799380636 | 0.684832139585057 | 2.75165757427843 | 566.846336232082 | Palau |
| 1996 | 543.08056313569 | 0.69009567251107 | 2.54180864566773 | 540.538754490022 | Palau |
| 1997 | 509.10379093342 | 0.695122122982618 | 2.41586654771726 | 506.687924385703 | Palau |
| 1998 | 490.24468305801 | 0.69965290951971 | 2.3558462518177 | 487.888836806192 | Palau |
| 1999 | 479.35669843071 | 0.703115321964044 | 2.309899118324 | 477.046799312386 | Palau |
| 2000 | 465.20488332518 | 0.70519694495869 | 2.2832874466924 | 462.921595878488 | Palau |
| 2001 | 452.52009824114 | 0.707402386499998 | 2.2738088191241 | 450.246289422016 | Palau |
| 2002 | 443.5719893642 | 0.709587643886923 | 2.23817454265744 | 441.333814821543 | Palau |
| 2003 | 437.08198019466 | 0.711000220048301 | 2.25402110889688 | 434.827959085763 | Palau |
| 2004 | 435.56847629638 | 0.712543811157683 | 2.2395513637393 | 433.328924932641 | Palau |
| 2005 | 428.94131518271 | 0.71433983112227 | 2.22873460461355 | 426.712580578096 | Palau |
| 2006 | 423.531412916 | 0.716353431401992 | 2.23277347115438 | 421.298639444846 | Palau |
| 2007 | 414.69591174447 | 0.718809541917246 | 2.21106929959362 | 412.484842444876 | Palau |
| 2008 | 359.07482230282 | 0.720806622774243 | 2.18315502221152 | 356.891667280608 | Palau |
| 2009 | 354.31204436724 | 0.722288266788472 | 2.16041628301661 | 352.151628084223 | Palau |
| 2010 | 342.84191440613 | 0.724065956349117 | 2.16696593949612 | 340.674948466634 | Palau |
| 2011 | 329.34237329398 | 0.726733445911764 | 2.16999563174013 | 327.17237766224 | Palau |
| 2012 | 319.09256501876 | 0.729612899417556 | 2.1699631514011 | 316.922601867359 | Palau |
| 2013 | 312.71359569514 | 0.731953474727171 | 2.16620929908605 | 310.547386396054 | Palau |
| 2014 | 304.83690035339 | 0.734768296510748 | 2.16031489660473 | 302.676585456785 | Palau |
| 2015 | 300.03030445664 | 0.73816642523263 | 2.17505682193735 | 297.855247634703 | Palau |
| 2016 | 294.74168473303 | 0.741361910823289 | 2.10376885113837 | 292.637915881892 | Palau |
| 2017 | 287.35869727004 | 0.744224767037247 | 2.09083297890372 | 285.267864291136 | Palau |
| 2018 | 275.67009483772 | 0.747761249656357 | 2.05069288444973 | 273.61940195327 | Palau |
| 2019 | 269.65022889481 | 0.750715306594841 | 2.04331446053974 | 267.60691443427 | Palau |
| 2020 | 266.97828968517 | 0.75278092905906 | 2.01265957807667 | 264.965630107093 | Palau |
| 2021 | 262.66910480995 | 0.75404693141209 | 2.03081314800915 | 260.638291661941 | Palau |
| 1990 | 20.76295376604 | 0.658758145866254 | 4.62269200440435 | 16.1402617616356 | Puerto Rico |
| 1991 | 19.33239556832 | 0.66326172041123 | 4.45767625404552 | 14.8747193142745 | Puerto Rico |
| 1992 | 20.45546888464 | 0.667272191591073 | 3.90964040951423 | 16.5458284751258 | Puerto Rico |
| 1993 | 22.87497567047 | 0.671024105168892 | 3.63527532157262 | 19.2397003488974 | Puerto Rico |
| 1994 | 21.93760944515 | 0.675450810240548 | 2.98125591139485 | 18.9563535337551 | Puerto Rico |
| 1995 | 22.17946716343 | 0.680673949739819 | 2.89820350279215 | 19.2812636606378 | Puerto Rico |
| 1996 | 19.27264911294 | 0.685865893043239 | 2.86022736691196 | 16.412421746028 | Puerto Rico |
| 1997 | 18.61828959636 | 0.691274381965422 | 2.55622707401016 | 16.0620625223498 | Puerto Rico |
| 1998 | 16.26140869929 | 0.697040445469226 | 2.36158689012917 | 13.8998218091608 | Puerto Rico |
| 1999 | 14.42094700529 | 0.702850670729254 | 2.33119780094484 | 12.0897492043452 | Puerto Rico |
| 2000 | 12.72692317832 | 0.710035652009262 | 2.21734986992406 | 10.5095733083959 | Puerto Rico |
| 2001 | 10.41144460622 | 0.718955861108189 | 2.17765499907089 | 8.23378960714911 | Puerto Rico |
| 2002 | 10.0700756757 | 0.725956855617272 | 2.18688680319321 | 7.88318887250679 | Puerto Rico |
| 2003 | 9.58494892141 | 0.730475137768497 | 2.16018204314375 | 7.42476687826625 | Puerto Rico |
| 2004 | 9.28513853037 | 0.734219545590062 | 2.16718311032462 | 7.11795542004539 | Puerto Rico |
| 2005 | 9.48409445729 | 0.737742266137191 | 2.13606404789514 | 7.34803040939487 | Puerto Rico |
| 2006 | 9.12408914711 | 0.741629431374531 | 2.10840215500796 | 7.01568699210204 | Puerto Rico |
| 2007 | 8.87127806121 | 0.74588380735408 | 2.08207026389944 | 6.78920779731056 | Puerto Rico |
| 2008 | 9.02350611129 | 0.750922167274437 | 2.05916562315763 | 6.96434048813237 | Puerto Rico |
| 2009 | 9.19381917669 | 0.756361694934789 | 1.98586433491512 | 7.20795484177488 | Puerto Rico |
| 2010 | 8.72732821608 | 0.761763253126039 | 1.64382765257361 | 7.08350056350639 | Puerto Rico |
| 2011 | 9.20857232868 | 0.766784881779412 | 1.55155696384879 | 7.65701536483121 | Puerto Rico |
| 2012 | 9.79896036554 | 0.772311804167716 | 1.577002505084 | 8.221957860456 | Puerto Rico |
| 2013 | 8.7237435903 | 0.778461161794537 | 1.54252664144524 | 7.18121694885476 | Puerto Rico |
| 2014 | 9.3697173301 | 0.785120344673962 | 1.46158177812 | 7.90813555198 | Puerto Rico |
| 2015 | 8.6989063092 | 0.791800166848976 | 1.43513930405452 | 7.26376700514548 | Puerto Rico |
| 2016 | 7.61694232432 | 0.799084082615032 | 1.44622777117789 | 6.1707145531421 | Puerto Rico |
| 2017 | 7.35011103584 | 0.806648960516056 | 1.42571918887781 | 5.92439184696219 | Puerto Rico |
| 2018 | 10.94073233473 | 0.812906066317222 | 1.44435876170692 | 9.49637357302308 | Puerto Rico |
| 2019 | 11.86961643903 | 0.818843190899062 | 1.44972957578365 | 10.4198868632463 | Puerto Rico |
| 2020 | 11.61772917582 | 0.822918436314863 | 1.42508546100188 | 10.1926437148181 | Puerto Rico |
| 2021 | 10.74899717941 | 0.825525846914029 | 1.42568219878778 | 9.32331498062222 | Puerto Rico |
| 1990 | 265.86259114198 | 0.580685876593206 | 13.3564024442051 | 252.506188697775 | Saint Kitts and Nevis |
| 1991 | 224.30279711965 | 0.590254950402484 | 13.3436473394721 | 210.959149780178 | Saint Kitts and Nevis |
| 1992 | 213.89734239233 | 0.599187189594131 | 13.5297849654683 | 200.367557426862 | Saint Kitts and Nevis |
| 1993 | 218.82218734612 | 0.607235891628758 | 13.3569370855926 | 205.465250260527 | Saint Kitts and Nevis |
| 1994 | 209.00202724454 | 0.614514657962512 | 13.4293110792147 | 195.572716165325 | Saint Kitts and Nevis |
| 1995 | 192.95471826918 | 0.620716497645849 | 13.0323410231553 | 179.922377246025 | Saint Kitts and Nevis |
| 1996 | 179.09456635987 | 0.626207410919295 | 13.0379208449715 | 166.056645514898 | Saint Kitts and Nevis |
| 1997 | 156.67529253828 | 0.631274740057709 | 11.9389924440756 | 144.736300094204 | Saint Kitts and Nevis |
| 1998 | 145.43732800557 | 0.635539987956464 | 10.3098431567942 | 135.127484848776 | Saint Kitts and Nevis |
| 1999 | 119.10836172245 | 0.63971373866775 | 7.6303799973054 | 111.477981725145 | Saint Kitts and Nevis |
| 2000 | 102.54547305534 | 0.644353376846687 | 6.20565329376571 | 96.3398197615743 | Saint Kitts and Nevis |
| 2001 | 84.23302879766 | 0.64922665301934 | 6.02895502208364 | 78.2040737755764 | Saint Kitts and Nevis |
| 2002 | 75.95374243077 | 0.654593408970176 | 4.81440977075298 | 71.139332660017 | Saint Kitts and Nevis |
| 2003 | 72.24900622905 | 0.659925311695556 | 4.591106960545 | 67.657899268505 | Saint Kitts and Nevis |
| 2004 | 70.05154984604 | 0.667568129909661 | 3.830289109997 | 66.221260736043 | Saint Kitts and Nevis |
| 2005 | 73.00758696683 | 0.679145457347323 | 2.78025926440354 | 70.2273277024265 | Saint Kitts and Nevis |
| 2006 | 74.14853230052 | 0.690078666648128 | 2.49323591053868 | 71.6552963899813 | Saint Kitts and Nevis |
| 2007 | 72.33203858564 | 0.697727627549003 | 2.40808083671561 | 69.9239577489244 | Saint Kitts and Nevis |
| 2008 | 69.19216391927 | 0.704141493783467 | 2.31532027865418 | 66.8768436406158 | Saint Kitts and Nevis |
| 2009 | 68.14951236395 | 0.709499498252707 | 2.25171197520165 | 65.8978003887484 | Saint Kitts and Nevis |
| 2010 | 65.63443517604 | 0.714460415458321 | 2.19671330625973 | 63.4377218697803 | Saint Kitts and Nevis |
| 2011 | 70.02992634838 | 0.719324380918298 | 2.20311702191899 | 67.826809326461 | Saint Kitts and Nevis |
| 2012 | 67.15415141698 | 0.723436152222763 | 2.19826602744566 | 64.9558853895343 | Saint Kitts and Nevis |
| 2013 | 68.59120417805 | 0.726706592636212 | 2.19182701696959 | 66.3993771610804 | Saint Kitts and Nevis |
| 2014 | 73.21004284904 | 0.729530218928803 | 2.1858711183658 | 71.0241717306742 | Saint Kitts and Nevis |
| 2015 | 68.6486943959 | 0.732220671984012 | 2.13586454955048 | 66.5128298463495 | Saint Kitts and Nevis |
| 2016 | 66.26935238645 | 0.735566138078794 | 2.141827942145 | 64.127524444305 | Saint Kitts and Nevis |
| 2017 | 62.39483402702 | 0.739470681017895 | 2.15995044367829 | 60.2348835833417 | Saint Kitts and Nevis |
| 2018 | 59.03333419461 | 0.743736461382785 | 2.09745728738106 | 56.9358769072289 | Saint Kitts and Nevis |
| 2019 | 56.09765598131 | 0.748152066317114 | 2.07316557630894 | 54.0244904050011 | Saint Kitts and Nevis |
| 2020 | 51.78015073882 | 0.75156840922289 | 2.04452767135329 | 49.7356230674667 | Saint Kitts and Nevis |
| 2021 | 51.02314743467 | 0.754987054945241 | 2.02502996537855 | 48.9981174692915 | Saint Kitts and Nevis |
| 1990 | 6.91697356215 | 0.813244887891478 | 1.44693608189357 | 5.47003748025643 | San Marino |
| 1991 | 7.00710480126 | 0.818282512759725 | 1.47332972920758 | 5.53377507205242 | San Marino |
| 1992 | 7.06983002682 | 0.822782757963244 | 1.42669978450872 | 5.64313024231128 | San Marino |
| 1993 | 7.1584436639 | 0.827382324106265 | 1.38738109769795 | 5.77106256620205 | San Marino |
| 1994 | 7.23928118293 | 0.8319709830207 | 1.39768643396641 | 5.84159474896359 | San Marino |
| 1995 | 7.33582311523 | 0.836762725678003 | 1.43110353935758 | 5.90471957587242 | San Marino |
| 1996 | 7.41405840844 | 0.841389494577046 | 1.39437608697143 | 6.01968232146857 | San Marino |
| 1997 | 7.47772047879 | 0.845864864873086 | 1.35663985645625 | 6.12108062233375 | San Marino |
| 1998 | 7.54498382608 | 0.850474771150175 | 1.31910430684278 | 6.22587951923722 | San Marino |
| 1999 | 7.60352136448 | 0.855338613208904 | 1.3294003015635 | 6.2741210629165 | San Marino |
| 2000 | 7.68798258029 | 0.859695363255401 | 1.31787545711783 | 6.37010712317217 | San Marino |
| 2001 | 7.74861481682 | 0.863747756857469 | 1.29287323368429 | 6.45574158313571 | San Marino |
| 2002 | 7.84762127537 | 0.865680591501536 | 1.26821065421463 | 6.57941062115537 | San Marino |
| 2003 | 7.91949052049 | 0.867522008779968 | 1.27975454695829 | 6.63973597353171 | San Marino |
| 2004 | 8.00742385344 | 0.869287134173222 | 1.26531808355132 | 6.74210576988868 | San Marino |
| 2005 | 8.08613916529 | 0.87097959576402 | 1.28467235382222 | 6.80146681146778 | San Marino |
| 2006 | 8.16089774016 | 0.872652149736731 | 1.26649496917681 | 6.89440277098319 | San Marino |
| 2007 | 8.27027965192 | 0.874307253083233 | 1.24915777854316 | 7.02112187337684 | San Marino |
| 2008 | 8.38934019001 | 0.875991881471404 | 1.27836775124295 | 7.11097243876705 | San Marino |
| 2009 | 8.46539706872 | 0.877705228262122 | 1.264042766487 | 7.201354302233 | San Marino |
| 2010 | 8.49513628504 | 0.879440813617125 | 1.251444127475 | 7.243692157565 | San Marino |
| 2011 | 8.47476314594 | 0.881197517807579 | 1.25805726070426 | 7.21670588523575 | San Marino |
| 2012 | 8.42209133297 | 0.88292453140369 | 1.26812662811826 | 7.15396470485174 | San Marino |
| 2013 | 8.3526774295 | 0.884592626900131 | 1.26706134408813 | 7.08561608541187 | San Marino |
| 2014 | 8.25489184518 | 0.885353693736433 | 1.28741912312756 | 6.96747272205244 | San Marino |
| 2015 | 8.1456414743 | 0.884693365070092 | 1.24825240100087 | 6.89738907329913 | San Marino |
| 2016 | 7.78079732338 | 0.884477664772925 | 1.24994844598127 | 6.53084887739873 | San Marino |
| 2017 | 7.08951609001 | 0.884557606743879 | 1.26728282130396 | 5.82223326870604 | San Marino |
| 2018 | 6.39634916018 | 0.885133515535323 | 1.26713281967806 | 5.12921634050194 | San Marino |
| 2019 | 6.02952886423 | 0.886200660194545 | 1.24349721051 | 4.78603165372 | San Marino |
| 2020 | 5.86203647842 | 0.886867937194416 | 1.24791349667409 | 4.61412298174591 | San Marino |
| 2021 | 5.60766807507 | 0.888005473958106 | 1.22013833154971 | 4.38752974352029 | San Marino |
| 1990 | 1004.59521242089 | 0.521942386048128 | 22.2102764355672 | 982.384935985323 | Tokelau |
| 1991 | 905.08733685942 | 0.526628473491586 | 22.1320214295584 | 882.955315429862 | Tokelau |
| 1992 | 824.01503606609 | 0.531307850030329 | 22.1387866737396 | 801.87624939235 | Tokelau |
| 1993 | 766.84494202156 | 0.535935005438943 | 21.4774911089053 | 745.367450912655 | Tokelau |
| 1994 | 725.24482756014 | 0.540523664725072 | 21.2115450360583 | 704.033282524082 | Tokelau |
| 1995 | 696.17654230037 | 0.545295634483764 | 21.1827535310887 | 674.993788769281 | Tokelau |
| 1996 | 673.94853329212 | 0.55028265171702 | 19.8704296076949 | 654.078103684425 | Tokelau |
| 1997 | 627.23588271098 | 0.555631325529965 | 18.5661381553497 | 608.66974455563 | Tokelau |
| 1998 | 594.90380108365 | 0.561047610799914 | 16.9442781980071 | 577.959522885643 | Tokelau |
| 1999 | 603.29364803254 | 0.566415405899331 | 15.2291854453255 | 588.064462587215 | Tokelau |
| 2000 | 567.10239310017 | 0.571814369871981 | 13.1615645981811 | 553.940828501989 | Tokelau |
| 2001 | 521.15725007117 | 0.576915597204096 | 13.4252853723307 | 507.731964698839 | Tokelau |
| 2002 | 479.53966062421 | 0.581858317064991 | 13.3996920772584 | 466.139968546952 | Tokelau |
| 2003 | 434.98870459547 | 0.586854346188713 | 13.2688767221093 | 421.719827873361 | Tokelau |
| 2004 | 408.01196967007 | 0.592342442430319 | 13.570900413028 | 394.441069257042 | Tokelau |
| 2005 | 370.23105866172 | 0.59817611685198 | 13.1698257548859 | 357.061232906834 | Tokelau |
| 2006 | 338.97434523572 | 0.604429389636372 | 13.3129400543852 | 325.661405181335 | Tokelau |
| 2007 | 312.02235179182 | 0.61116323669051 | 13.181742819257 | 298.840608972563 | Tokelau |
| 2008 | 286.92544582164 | 0.617940246542751 | 12.886213696689 | 274.039232124951 | Tokelau |
| 2009 | 262.59733597314 | 0.624070078096847 | 12.9028421122811 | 249.694493860859 | Tokelau |
| 2010 | 259.18158897485 | 0.630175540158128 | 12.0486837111167 | 247.132905263733 | Tokelau |
| 2011 | 253.73172097676 | 0.636016518005944 | 10.2951247252118 | 243.436596251548 | Tokelau |
| 2012 | 247.22364310083 | 0.641611991197784 | 7.4832088455 | 239.74043425533 | Tokelau |
| 2013 | 237.20663500504 | 0.647966148124354 | 6.28711764906552 | 230.919517355974 | Tokelau |
| 2014 | 229.34796147278 | 0.653231999411508 | 5.21503205907882 | 224.132929413701 | Tokelau |
| 2015 | 224.11380816324 | 0.658798994926187 | 4.47418139619197 | 219.639626767048 | Tokelau |
| 2016 | 220.63578145095 | 0.663974319058952 | 4.4915578898243 | 216.144223561126 | Tokelau |
| 2017 | 212.47438351 | 0.669054133759442 | 3.750939684696 | 208.723443825304 | Tokelau |
| 2018 | 206.95262769675 | 0.674179514031409 | 3.21481421803597 | 203.737813478714 | Tokelau |
| 2019 | 202.05912801201 | 0.679247839201533 | 2.83008664963631 | 199.229041362374 | Tokelau |
| 2020 | 1639.74895336958 | 0.682937378311047 | 2.73944450985204 | 1637.00950885973 | Tokelau |
| 2021 | 1663.6247500805 | 0.68642562083005 | 2.84250295791427 | 1660.78224712259 | Tokelau |
| 1990 | 1493.26385564869 | 0.406247565957081 | 357.281050659835 | 1135.98280498886 | Tuvalu |
| 1991 | 1314.331688981 | 0.414819936897447 | 274.312641417359 | 1040.01904756364 | Tuvalu |
| 1992 | 1166.20801946416 | 0.422901979744667 | 244.401080952058 | 921.806938512102 | Tuvalu |
| 1993 | 1046.05632768702 | 0.430518260028408 | 204.545449780604 | 841.510877906416 | Tuvalu |
| 1994 | 931.73460398412 | 0.438563050767836 | 115.805626361352 | 815.928977622768 | Tuvalu |
| 1995 | 859.02016294029 | 0.444962241977075 | 105.53094625737 | 753.48921668292 | Tuvalu |
| 1996 | 805.54018239043 | 0.449804979978272 | 77.4247413749849 | 728.115441015445 | Tuvalu |
| 1997 | 719.28869533077 | 0.455368427922084 | 72.844747503507 | 646.443947827263 | Tuvalu |
| 1998 | 646.23854712818 | 0.462454678093366 | 63.3056670286946 | 582.932880099485 | Tuvalu |
| 1999 | 625.10829381114 | 0.468845801641229 | 58.0262246588977 | 567.082069152242 | Tuvalu |
| 2000 | 560.4145699963 | 0.474956823462581 | 56.3071692496883 | 504.107400746612 | Tuvalu |
| 2001 | 502.24536539201 | 0.480850886398825 | 55.2944004490477 | 446.950964942962 | Tuvalu |
| 2002 | 452.77262109553 | 0.487252252447393 | 51.071993565334 | 401.700627530196 | Tuvalu |
| 2003 | 412.37571959247 | 0.492754711063713 | 49.8938955504454 | 362.481824042025 | Tuvalu |
| 2004 | 361.0597222717 | 0.497692152366724 | 25.0641125945269 | 335.995609677173 | Tuvalu |
| 2005 | 343.05860150338 | 0.501851309525404 | 23.4185117584853 | 319.640089744895 | Tuvalu |
| 2006 | 361.24223098992 | 0.505975410569688 | 22.8528573543956 | 338.389373635524 | Tuvalu |
| 2007 | 343.81188882879 | 0.51050430052828 | 22.6604417320439 | 321.151447096746 | Tuvalu |
| 2008 | 320.88450473877 | 0.515611600817149 | 22.2084735549493 | 298.676031183821 | Tuvalu |
| 2009 | 315.21396586254 | 0.519870565592834 | 22.2202426735827 | 292.993723188957 | Tuvalu |
| 2010 | 311.51145668368 | 0.52345550974778 | 22.1464887954548 | 289.364967888225 | Tuvalu |
| 2011 | 301.16648261382 | 0.527744244866808 | 22.155045533873 | 279.011437079947 | Tuvalu |
| 2012 | 301.12590533609 | 0.531203234840239 | 22.2336396654267 | 278.892265670663 | Tuvalu |
| 2013 | 297.91820318715 | 0.53516623462033 | 21.5258356925038 | 276.392367494646 | Tuvalu |
| 2014 | 291.02228017303 | 0.539122244261915 | 21.3374876842824 | 269.684792488748 | Tuvalu |
| 2015 | 288.29467701337 | 0.544047880204377 | 21.1890264050289 | 267.105650608341 | Tuvalu |
| 2016 | 283.74165850815 | 0.549173928060589 | 20.3010396041996 | 263.44061890395 | Tuvalu |
| 2017 | 265.62297585532 | 0.554466480141161 | 19.0330628364567 | 246.589913018863 | Tuvalu |
| 2018 | 251.05481473992 | 0.559859395089525 | 17.4022761887992 | 233.652538551121 | Tuvalu |
| 2019 | 234.46643114504 | 0.566045488622333 | 15.3271813568743 | 219.139249788166 | Tuvalu |
| 2020 | 220.78392056961 | 0.571666102458017 | 13.228648569531 | 207.555272000079 | Tuvalu |
| 2021 | 208.907303224 | 0.576620529344684 | 13.3553775953763 | 195.551925628624 | Tuvalu |
| 1990 | 31.7532582 | 0.655160855636097 | 5.0249989621761 | 26.7282592378239 | United States Virgin Islands |
| 1991 | 29.72999540134 | 0.664018659064364 | 4.37304812754596 | 25.356947273794 | United States Virgin Islands |
| 1992 | 28.34280114402 | 0.67108187072399 | 3.66308888262491 | 24.6797122613951 | United States Virgin Islands |
| 1993 | 27.14589743604 | 0.676872744796339 | 3.01048831457936 | 24.1354091214606 | United States Virgin Islands |
| 1994 | 26.00204445999 | 0.681982223083549 | 2.83801604234094 | 23.1640284176491 | United States Virgin Islands |
| 1995 | 24.66032494708 | 0.686730210603479 | 2.76871995769688 | 21.8916049893831 | United States Virgin Islands |
| 1996 | 23.51582966462 | 0.691247698919766 | 2.48326172903379 | 21.0325679355862 | United States Virgin Islands |
| 1997 | 22.35424625338 | 0.69559820385004 | 2.42581888007583 | 19.9284273733042 | United States Virgin Islands |
| 1998 | 21.21417886912 | 0.699768685957964 | 2.46265532790633 | 18.7515235412137 | United States Virgin Islands |
| 1999 | 20.19969376032 | 0.703949341926433 | 2.26712287247469 | 17.9325708878453 | United States Virgin Islands |
| 2000 | 19.14770738818 | 0.708436564859144 | 2.32171866633731 | 16.8259887218427 | United States Virgin Islands |
| 2001 | 18.26130506024 | 0.713751114883336 | 2.25266226744184 | 16.0086427927982 | United States Virgin Islands |
| 2002 | 17.14969862089 | 0.724398272335199 | 2.16601399022213 | 14.9836846306679 | United States Virgin Islands |
| 2003 | 16.44681797737 | 0.734609849926843 | 2.1735642003938 | 14.2732537769762 | United States Virgin Islands |
| 2004 | 15.8735659598 | 0.744613598166223 | 2.07683854100088 | 13.7967274187991 | United States Virgin Islands |
| 2005 | 15.43904926152 | 0.754267557852673 | 2.03630348387322 | 13.4027457776468 | United States Virgin Islands |
| 2006 | 14.99040339765 | 0.763447495711391 | 1.73545069588173 | 13.2549527017683 | United States Virgin Islands |
| 2007 | 14.68889205259 | 0.772180593452306 | 1.54420049182532 | 13.1446915607647 | United States Virgin Islands |
| 2008 | 14.30296658768 | 0.780342622959945 | 1.49993110255818 | 12.8030354851218 | United States Virgin Islands |
| 2009 | 14.12490351273 | 0.787055145666672 | 1.42342887973386 | 12.7014746329961 | United States Virgin Islands |
| 2010 | 13.95519463094 | 0.793219696295781 | 1.43777295748173 | 12.5174216734583 | United States Virgin Islands |
| 2011 | 13.8066254015 | 0.798071698185583 | 1.51599299512407 | 12.2906324063759 | United States Virgin Islands |
| 2012 | 13.53960297761 | 0.80109285050392 | 1.42391432945717 | 12.1156886481528 | United States Virgin Islands |
| 2013 | 13.09573267152 | 0.803405789381199 | 1.45777662661558 | 11.6379560449044 | United States Virgin Islands |
| 2014 | 12.68145541884 | 0.805641378374046 | 1.44168062834145 | 11.2397747904985 | United States Virgin Islands |
| 2015 | 12.31063966234 | 0.807889245154254 | 1.43080850718773 | 10.8798311551523 | United States Virgin Islands |
| 2016 | 12.14543404551 | 0.810199044380962 | 1.42759792815432 | 10.7178361173557 | United States Virgin Islands |
| 2017 | 12.06322403666 | 0.812405171667542 | 1.43667681980187 | 10.6265472168581 | United States Virgin Islands |
| 2018 | 11.66734249525 | 0.814825439654601 | 1.44098535337875 | 10.2263571418713 | United States Virgin Islands |
| 2019 | 11.53011909964 | 0.817429008110061 | 1.40234214796667 | 10.1277769516733 | United States Virgin Islands |
| 2020 | 11.38692350659 | 0.819601712348942 | 1.41093663096326 | 9.97598687562674 | United States Virgin Islands |
| 2021 | 11.05958819083 | 0.821830853383701 | 1.45010862193833 | 9.60947956889167 | United States Virgin Islands |
| 1990 | 20185.2990403812 | 0.20665649952096 | 2322.32109114688 | 17862.9779492343 | South Sudan |
| 1991 | 19565.0673298638 | 0.208932715815866 | 2342.73416098062 | 17222.3331688832 | South Sudan |
| 1992 | 19157.9465337791 | 0.21119940064631 | 2304.17003317602 | 16853.7765006031 | South Sudan |
| 1993 | 18664.351925031 | 0.213416470699326 | 2315.90969822539 | 16348.4422268056 | South Sudan |
| 1994 | 18050.4752440427 | 0.21561208907535 | 2315.6783777865 | 15734.7968662562 | South Sudan |
| 1995 | 17692.8197363804 | 0.217920462775347 | 2306.26669810158 | 15386.5530382788 | South Sudan |
| 1996 | 16969.6901661159 | 0.220399437179258 | 2147.39853586565 | 14822.2916302503 | South Sudan |
| 1997 | 16304.8694847653 | 0.223037171256267 | 2149.01426871513 | 14155.8552160502 | South Sudan |
| 1998 | 15698.3800235776 | 0.225788868984577 | 2129.34001158363 | 13569.040011994 | South Sudan |
| 1999 | 14975.892533996 | 0.228648585296784 | 1943.24434760425 | 13032.6481863918 | South Sudan |
| 2000 | 14485.2380132197 | 0.231730343895352 | 1913.88871385366 | 12571.349299366 | South Sudan |
| 2001 | 13937.2432620601 | 0.235014127905546 | 1919.50651268114 | 12017.736749379 | South Sudan |
| 2002 | 13323.0208407441 | 0.238341185794991 | 1712.58749523469 | 11610.4333455094 | South Sudan |
| 2003 | 12782.2766534632 | 0.241797191221707 | 1712.95982838651 | 11069.3168250767 | South Sudan |
| 2004 | 12595.41864425 | 0.245462498724322 | 1712.46820246381 | 10882.9504417862 | South Sudan |
| 2005 | 13019.3354203288 | 0.249280903501851 | 1515.28872721696 | 11504.0466931118 | South Sudan |
| 2006 | 14490.4706372718 | 0.253300507898377 | 1524.49761952768 | 12965.9730177441 | South Sudan |
| 2007 | 13761.0310025926 | 0.257491763500287 | 1396.86020982446 | 12364.1707927681 | South Sudan |
| 2008 | 13246.9739664296 | 0.261740458461839 | 1400.36000779412 | 11846.6139586355 | South Sudan |
| 2009 | 12630.840209341 | 0.266179234510504 | 1391.04219216676 | 11239.7980171742 | South Sudan |
| 2010 | 11771.1858765208 | 0.270572622006731 | 1245.83311465549 | 10525.3527618653 | South Sudan |
| 2011 | 11080.7266857654 | 0.274719142558607 | 1251.2039273302 | 9829.5227584352 | South Sudan |
| 2012 | 11080.587122168 | 0.275066142602006 | 1237.50446716052 | 9843.08265500748 | South Sudan |
| 2013 | 11210.0760318882 | 0.275813942458139 | 1199.14122868365 | 10010.9348032046 | South Sudan |
| 2014 | 11314.4138902769 | 0.276311461476096 | 1188.99012357464 | 10125.4237667023 | South Sudan |
| 2015 | 11332.5617221105 | 0.275833634320407 | 1192.85586706857 | 10139.7058550419 | South Sudan |
| 2016 | 11576.0382023767 | 0.274455491179089 | 1251.22458172883 | 10324.8136206479 | South Sudan |
| 2017 | 11923.6819614949 | 0.274387859268159 | 1249.89120337491 | 10673.79075812 | South Sudan |
| 2018 | 12386.9329143873 | 0.27498784845828 | 1244.17427235394 | 11142.7586420334 | South Sudan |
| 2019 | 11530.122865022 | 0.275706200830302 | 1197.94070344715 | 10332.1821615749 | South Sudan |
| 2020 | 10507.2263869567 | 0.276787923789994 | 1205.32521969789 | 9301.90116725881 | South Sudan |
| 2021 | 9653.36746992752 | 0.278371125484101 | 1200.91346424897 | 8452.45400567855 | South Sudan |
| 1990 | 4299.65857831421 | 0.292178643209656 | 1071.5423492249 | 3228.11622908931 | Sudan |
| 1991 | 4086.79343968883 | 0.296473445829652 | 1086.8168267122 | 2999.97661297663 | Sudan |
| 1992 | 3894.54022411309 | 0.30086367654421 | 1028.52464695308 | 2866.01557716001 | Sudan |
| 1993 | 3715.03587739031 | 0.305409325988089 | 1012.98814849335 | 2702.04772889696 | Sudan |
| 1994 | 3550.90379480983 | 0.310096430272723 | 976.32381370237 | 2574.57998110746 | Sudan |
| 1995 | 3368.43800375891 | 0.315208259227984 | 836.508240915621 | 2531.92976284329 | Sudan |
| 1996 | 3321.67002419288 | 0.320680229352556 | 831.254884358345 | 2490.41513983454 | Sudan |
| 1997 | 3002.87500514169 | 0.326761462290204 | 776.008080438332 | 2226.86692470336 | Sudan |
| 1998 | 3070.96805776673 | 0.333087760646334 | 680.321670003437 | 2390.64638776329 | Sudan |
| 1999 | 2652.76938052411 | 0.339754883653516 | 608.81130726027 | 2043.95807326384 | Sudan |
| 2000 | 2421.06867761971 | 0.346875808615915 | 599.515263644871 | 1821.55341397484 | Sudan |
| 2001 | 2234.55326473362 | 0.354215514631628 | 588.653335975653 | 1645.89992875797 | Sudan |
| 2002 | 2070.05640789273 | 0.36172823705999 | 572.994276423785 | 1497.06213146894 | Sudan |
| 2003 | 1907.96782184203 | 0.369693652760892 | 581.861316034243 | 1326.10650580779 | Sudan |
| 2004 | 1781.549293188 | 0.377920656442902 | 552.668478843232 | 1228.88081434477 | Sudan |
| 2005 | 1695.70514156579 | 0.386685712077585 | 517.568281006094 | 1178.1368605597 | Sudan |
| 2006 | 1684.75305358253 | 0.39615137434576 | 465.205558837594 | 1219.54749474494 | Sudan |
| 2007 | 1471.49431899201 | 0.406079992219235 | 363.638174405902 | 1107.85614458611 | Sudan |
| 2008 | 1373.28258786055 | 0.416087599939819 | 273.990150864211 | 1099.29243699634 | Sudan |
| 2009 | 1268.91041875577 | 0.425975917452396 | 233.372066656979 | 1035.53835209879 | Sudan |
| 2010 | 1139.17207054959 | 0.436171097454113 | 186.724563162561 | 952.447507387029 | Sudan |
| 2011 | 933.91379395403 | 0.447239441530175 | 72.828372915277 | 861.085421038753 | Sudan |
| 2012 | 827.27159669577 | 0.456790264757693 | 65.287029063889 | 761.984567631881 | Sudan |
| 2013 | 739.25033279143 | 0.466435995212048 | 58.7553850068041 | 680.494947784626 | Sudan |
| 2014 | 669.28115367075 | 0.476362134920594 | 56.5490616641313 | 612.732092006619 | Sudan |
| 2015 | 586.19326132637 | 0.486549437288546 | 53.9903698683378 | 532.202891458032 | Sudan |
| 2016 | 514.76703249575 | 0.496848218716792 | 30.6423575355484 | 484.124674960202 | Sudan |
| 2017 | 541.84081400595 | 0.50688353389451 | 22.6891781221845 | 519.151635883766 | Sudan |
| 2018 | 387.57927977862 | 0.516214086476663 | 22.2239516643799 | 365.35532811424 | Sudan |
| 2019 | 345.80250259529 | 0.525009122461798 | 22.0798049714067 | 323.722697623883 | Sudan |
| 2020 | 310.98000361499 | 0.533455421993626 | 22.1264333280068 | 288.853570286983 | Sudan |
| 2021 | 284.70451200239 | 0.54194973514933 | 21.1554831877646 | 263.549028814625 | Sudan |

Table S13 Frontier Analysis Based on SDI and DALYs for Adenovirus Associated Diarrheal in Neonates and Children Under Five Years of Age in 204 Countries and Territories

| **Year** | **DALYs rate** | **SDI** | **Frontier** | **Effective difference** | **Location** |
| --- | --- | --- | --- | --- | --- |
| 1990 | 315.78461893388 | 0.458668935267616 | 92.5665456940902 | 223.21807323979 | China |
| 1991 | 293.35031099398 | 0.467301969740226 | 85.4262731461701 | 207.92403784781 | China |
| 1992 | 260.68565651289 | 0.475784030669638 | 77.7974988435087 | 182.888157669381 | China |
| 1993 | 235.16427303949 | 0.484353830709851 | 77.5364942815711 | 157.627778757919 | China |
| 1994 | 214.81101302359 | 0.49257864459854 | 73.0115318207771 | 141.799481202813 | China |
| 1995 | 195.39120504688 | 0.501500907580649 | 16.6511184539839 | 178.740086592896 | China |
| 1996 | 173.38821641079 | 0.512033995790715 | 15.5883292885042 | 157.799887122286 | China |
| 1997 | 149.56716418119 | 0.522326866102753 | 15.1565293263697 | 134.41063485482 | China |
| 1998 | 127.75302428249 | 0.531379484348035 | 15.0732944025945 | 112.679729879896 | China |
| 1999 | 109.98164665608 | 0.540333688117168 | 15.0642578924392 | 94.9173887636408 | China |
| 2000 | 97.0950903708 | 0.547989192872889 | 14.4763262487604 | 82.6187641220396 | China |
| 2001 | 85.16406884271 | 0.553680373692626 | 13.0545925781986 | 72.1094762645114 | China |
| 2002 | 75.51902040988 | 0.560542936414416 | 11.8581955695433 | 63.6608248403367 | China |
| 2003 | 69.28390051825 | 0.569018827276063 | 9.50480845925089 | 59.7790920589991 | China |
| 2004 | 62.4930572173 | 0.578178090807068 | 8.65806386388953 | 53.8349933534105 | China |
| 2005 | 53.43356360538 | 0.58805219357166 | 8.69418646689855 | 44.7393771384815 | China |
| 2006 | 43.82922790911 | 0.598758170327953 | 9.11271255694696 | 34.716515352163 | China |
| 2007 | 34.55607321886 | 0.609520246163436 | 8.90469681061089 | 25.6513764082491 | China |
| 2008 | 26.88310580808 | 0.619454832781724 | 8.95067011412453 | 17.9324356939555 | China |
| 2009 | 20.989510547 | 0.62930772749348 | 8.21187433545333 | 12.7776362115467 | China |
| 2010 | 16.4025906436 | 0.64152144342547 | 5.87812513727262 | 10.5244655063274 | China |
| 2011 | 13.49157490237 | 0.651447977618785 | 4.86248400800875 | 8.62909089436125 | China |
| 2012 | 12.03743275978 | 0.657144292359298 | 4.70962730949759 | 7.32780545028241 | China |
| 2013 | 11.00540779298 | 0.66310300729639 | 4.33593063713073 | 6.66947715584927 | China |
| 2014 | 10.0230007436 | 0.668640847002655 | 3.87383614626523 | 6.14916459733477 | China |
| 2015 | 9.4223952738 | 0.671940110944872 | 3.79121157594302 | 5.63118369785698 | China |
| 2016 | 8.6153035557 | 0.676218437253933 | 3.65717072084815 | 4.95813283485185 | China |
| 2017 | 6.99709321507 | 0.6846196607825 | 3.33857554997075 | 3.65851766509925 | China |
| 2018 | 5.65205789876 | 0.693717996192013 | 3.14788248644833 | 2.50417541231167 | China |
| 2019 | 4.66224054351 | 0.703686823937232 | 3.01743005320059 | 1.64481049030941 | China |
| 2020 | 6.80367726547 | 0.713364584593745 | 2.73330046586095 | 4.07037679960905 | China |
| 2021 | 6.11336706668 | 0.721629759719777 | 2.44853367037514 | 3.66483339630486 | China |
| 1990 | 24.53094084961 | 0.497780128289885 | 17.8424230569655 | 6.68851779264448 | Democratic People's Republic of Korea |
| 1991 | 23.54883470533 | 0.49912051226047 | 17.3568170403125 | 6.19201766501753 | Democratic People's Republic of Korea |
| 1992 | 22.61558124331 | 0.499629841478392 | 17.2669139215741 | 5.34866732173587 | Democratic People's Republic of Korea |
| 1993 | 21.57354568963 | 0.49970064634474 | 16.6143433888921 | 4.95920230073794 | Democratic People's Republic of Korea |
| 1994 | 20.67427913574 | 0.499531339948334 | 17.3102576317503 | 3.36402150398965 | Democratic People's Republic of Korea |
| 1995 | 19.74422869907 | 0.499080529317303 | 17.1137918609524 | 2.63043683811761 | Democratic People's Republic of Korea |
| 1996 | 18.93533503458 | 0.498429013609349 | 17.3188313569037 | 1.61650367767633 | Democratic People's Republic of Korea |
| 1997 | 18.34045603512 | 0.497136868597342 | 17.6572768351532 | 0.683179199966791 | Democratic People's Republic of Korea |
| 1998 | 17.7591001525 | 0.49615920966594 | 17.7591001525 | 0 | Democratic People's Republic of Korea |
| 1999 | 17.17445013811 | 0.496484753521051 | 17.17445013811 | 0 | Democratic People's Republic of Korea |
| 2000 | 16.61876956256 | 0.497461994823193 | 16.61876956256 | 0 | Democratic People's Republic of Korea |
| 2001 | 16.12349190128 | 0.499674042505593 | 16.12349190128 | 0 | Democratic People's Republic of Korea |
| 2002 | 15.81022555743 | 0.502633568731371 | 15.81022555743 | 0 | Democratic People's Republic of Korea |
| 2003 | 15.64037829785 | 0.506165243055573 | 15.64037829785 | 0 | Democratic People's Republic of Korea |
| 2004 | 15.35734197441 | 0.510229134162392 | 15.35734197441 | 0 | Democratic People's Republic of Korea |
| 2005 | 14.9929196173 | 0.515097762842417 | 14.9929196173 | 0 | Democratic People's Republic of Korea |
| 2006 | 14.9446923624 | 0.519741439913669 | 14.9446923624 | 0 | Democratic People's Republic of Korea |
| 2007 | 15.1216411021 | 0.524066965087203 | 15.0041536717636 | 0.117487430336364 | Democratic People's Republic of Korea |
| 2008 | 15.54425219204 | 0.52881349761598 | 15.0615806988553 | 0.482671493184746 | Democratic People's Republic of Korea |
| 2009 | 15.69706002048 | 0.533114411172631 | 15.1067010667263 | 0.590358953753727 | Democratic People's Republic of Korea |
| 2010 | 15.65252809038 | 0.53719291837066 | 15.0403652474638 | 0.612162842916183 | Democratic People's Republic of Korea |
| 2011 | 15.04807834902 | 0.541212269847829 | 14.9872180277044 | 0.0608603213156105 | Democratic People's Republic of Korea |
| 2012 | 14.06608336897 | 0.545331713402902 | 14.06608336897 | 0 | Democratic People's Republic of Korea |
| 2013 | 12.87916159922 | 0.549483297235091 | 12.87916159922 | 0 | Democratic People's Republic of Korea |
| 2014 | 11.79858639652 | 0.553603559944788 | 11.79858639652 | 0 | Democratic People's Republic of Korea |
| 2015 | 11.04480985833 | 0.557321271453133 | 11.04480985833 | 0 | Democratic People's Republic of Korea |
| 2016 | 10.5508352115 | 0.560743562553338 | 10.5508352115 | 0 | Democratic People's Republic of Korea |
| 2017 | 10.10005003773 | 0.563388288880609 | 10.10005003773 | 0 | Democratic People's Republic of Korea |
| 2018 | 9.63786503969 | 0.565161484391211 | 9.63786503969 | 0 | Democratic People's Republic of Korea |
| 2019 | 9.25904115383 | 0.566765705893725 | 9.25904115383 | 0 | Democratic People's Republic of Korea |
| 2020 | 8.97500661716 | 0.56818855103523 | 8.97500661716 | 0 | Democratic People's Republic of Korea |
| 2021 | 7.9333979888 | 0.569854634067777 | 7.9333979888 | 0 | Democratic People's Republic of Korea |
| 1990 | 16.47657774084 | 0.667633853818721 | 3.90025340338926 | 12.5763243374507 | Taiwan (Province of China) |
| 1991 | 16.46150873513 | 0.676537236907717 | 3.61524680012582 | 12.8462619350042 | Taiwan (Province of China) |
| 1992 | 13.67466282172 | 0.684803635601178 | 3.28725602548644 | 10.3874067962336 | Taiwan (Province of China) |
| 1993 | 11.45899086146 | 0.692924130193594 | 3.04433732298721 | 8.4146535384728 | Taiwan (Province of China) |
| 1994 | 11.06650208864 | 0.701078749506874 | 2.97604658674356 | 8.09045550189644 | Taiwan (Province of China) |
| 1995 | 10.35419743878 | 0.709626820870811 | 2.70852160350875 | 7.64567583527125 | Taiwan (Province of China) |
| 1996 | 9.35990292788 | 0.719128415346056 | 2.556977596172 | 6.802925331708 | Taiwan (Province of China) |
| 1997 | 8.09024911385 | 0.72914549717473 | 2.17549118864745 | 5.91475792520255 | Taiwan (Province of China) |
| 1998 | 6.98021937259 | 0.738125787434293 | 2.04275518659875 | 4.93746418599125 | Taiwan (Province of China) |
| 1999 | 6.12274783519 | 0.745338399386342 | 1.7762114915982 | 4.3465363435918 | Taiwan (Province of China) |
| 2000 | 5.96059794236 | 0.752926841487622 | 1.76378705100422 | 4.19681089135578 | Taiwan (Province of China) |
| 2001 | 5.73755910169 | 0.760829809097496 | 1.66697178105 | 4.07058732064 | Taiwan (Province of China) |
| 2002 | 5.24936780979 | 0.769008983647841 | 1.71086312929725 | 3.53850468049275 | Taiwan (Province of China) |
| 2003 | 4.67210127978 | 0.776955803379445 | 1.70391819364083 | 2.96818308613917 | Taiwan (Province of China) |
| 2004 | 6.56478887786 | 0.784677977861477 | 1.6757006584659 | 4.8890882193941 | Taiwan (Province of China) |
| 2005 | 6.95828447494 | 0.792445129331779 | 1.60224583678791 | 5.35603863815209 | Taiwan (Province of China) |
| 2006 | 6.47743075538 | 0.800174674875457 | 1.56790209656381 | 4.90952865881619 | Taiwan (Province of China) |
| 2007 | 6.351894949 | 0.808137693890723 | 1.52020201911886 | 4.83169292988114 | Taiwan (Province of China) |
| 2008 | 6.71887926238 | 0.81540069133486 | 1.524073693436 | 5.194805568944 | Taiwan (Province of China) |
| 2009 | 6.7332523335 | 0.821432279265941 | 1.48631612070813 | 5.24693621279188 | Taiwan (Province of China) |
| 2010 | 6.87837448965 | 0.827226752015366 | 1.49155978174727 | 5.38681470790273 | Taiwan (Province of China) |
| 2011 | 7.0355273778 | 0.832064073807912 | 1.48813756887919 | 5.54738980892081 | Taiwan (Province of China) |
| 2012 | 7.53960890701 | 0.836677664574998 | 1.4737570840746 | 6.0658518229354 | Taiwan (Province of China) |
| 2013 | 7.08853347484 | 0.841280462756768 | 1.4574285540544 | 5.6311049207856 | Taiwan (Province of China) |
| 2014 | 7.12623964612 | 0.845695560406305 | 1.46543365137822 | 5.66080599474178 | Taiwan (Province of China) |
| 2015 | 7.19617757615 | 0.850055268699097 | 1.44703384156073 | 5.74914373458927 | Taiwan (Province of China) |
| 2016 | 6.78285866952 | 0.854466037759635 | 1.4292046846422 | 5.35365398487781 | Taiwan (Province of China) |
| 2017 | 6.59316163574 | 0.858888018761485 | 1.44866819897791 | 5.14449343676209 | Taiwan (Province of China) |
| 2018 | 6.52837425316 | 0.863158360983117 | 1.43027473590667 | 5.09809951725333 | Taiwan (Province of China) |
| 2019 | 6.39499053068 | 0.86741184993072 | 1.44494876353889 | 4.95004176714111 | Taiwan (Province of China) |
| 2020 | 6.38140321768 | 0.871247947250766 | 1.41605133147491 | 4.96535188620509 | Taiwan (Province of China) |
| 2021 | 6.29643688792 | 0.874747053187929 | 1.40641050425457 | 4.89002638366543 | Taiwan (Province of China) |
| 1990 | 2495.99510718331 | 0.289075059080125 | 1102.55559891303 | 1393.43950827028 | Cambodia |
| 1991 | 2433.93510669133 | 0.29247920029669 | 1034.35093569002 | 1399.58417100131 | Cambodia |
| 1992 | 2310.90245244675 | 0.296900954885372 | 1032.46515300031 | 1278.43729944644 | Cambodia |
| 1993 | 2226.84044382344 | 0.301627201679918 | 962.753170740191 | 1264.08727308325 | Cambodia |
| 1994 | 2177.87277467909 | 0.305722592004088 | 963.385210512037 | 1214.48756416705 | Cambodia |
| 1995 | 2051.34855484762 | 0.310678881281017 | 872.401524504794 | 1178.94703034283 | Cambodia |
| 1996 | 1908.59427492941 | 0.31563096971546 | 775.35989041694 | 1133.23438451247 | Cambodia |
| 1997 | 1793.76874210499 | 0.320503749678941 | 780.978318133015 | 1012.79042397198 | Cambodia |
| 1998 | 1697.66459064049 | 0.324974298583536 | 716.158815733067 | 981.505774907423 | Cambodia |
| 1999 | 1588.63759474482 | 0.330224246107348 | 669.204317042304 | 919.433277702516 | Cambodia |
| 2000 | 1430.80001133393 | 0.336075029953322 | 647.767102770415 | 783.032908563515 | Cambodia |
| 2001 | 1296.73172545168 | 0.342507910583009 | 570.901493141562 | 725.830232310118 | Cambodia |
| 2002 | 1153.47065008452 | 0.349465712270985 | 558.915656523323 | 594.554993561197 | Cambodia |
| 2003 | 1015.53371216693 | 0.356929660458733 | 505.766947489554 | 509.766764677376 | Cambodia |
| 2004 | 901.35856216486 | 0.364854644690823 | 431.389017071679 | 469.969545093181 | Cambodia |
| 2005 | 790.47350060354 | 0.373331814716366 | 358.775628390433 | 431.697872213107 | Cambodia |
| 2006 | 684.53917739217 | 0.381698166189418 | 322.410846655372 | 362.128330736798 | Cambodia |
| 2007 | 600.79093405435 | 0.389876229632594 | 285.768611999671 | 315.022322054679 | Cambodia |
| 2008 | 525.22499894029 | 0.397679519850279 | 263.155745800657 | 262.069253139633 | Cambodia |
| 2009 | 457.19167184024 | 0.404220933365413 | 238.061190310556 | 219.130481529684 | Cambodia |
| 2010 | 404.45113347836 | 0.410211121336958 | 215.743825986314 | 188.707307492046 | Cambodia |
| 2011 | 359.00641478003 | 0.415964408667437 | 197.795683255197 | 161.210731524833 | Cambodia |
| 2012 | 320.81474806277 | 0.4217697537185 | 192.185532878549 | 128.629215184221 | Cambodia |
| 2013 | 281.07027610688 | 0.427715060183387 | 167.754050206711 | 113.316225900169 | Cambodia |
| 2014 | 253.91624824415 | 0.433597748994237 | 131.019603513015 | 122.896644731135 | Cambodia |
| 2015 | 231.88019372017 | 0.43948735809131 | 129.093442082304 | 102.786751637866 | Cambodia |
| 2016 | 211.67270760631 | 0.445358172105636 | 109.992896984024 | 101.679810622286 | Cambodia |
| 2017 | 193.0795783273 | 0.451221744385535 | 107.920932560274 | 85.1586457670257 | Cambodia |
| 2018 | 178.05390199913 | 0.457341489845745 | 98.7808048196936 | 79.2730971794364 | Cambodia |
| 2019 | 163.74731725968 | 0.463643430446969 | 92.1267414350454 | 71.6205758246346 | Cambodia |
| 2020 | 152.22138421178 | 0.468876053670987 | 83.0769349795227 | 69.1444492322573 | Cambodia |
| 2021 | 142.50237919185 | 0.473621490708013 | 80.7733559737131 | 61.7290232181369 | Cambodia |
| 1990 | 6876.19496380873 | 0.457134954421362 | 100.046694403594 | 6776.14826940514 | Indonesia |
| 1991 | 6214.80280893739 | 0.466505583269134 | 85.3048715452606 | 6129.49793739213 | Indonesia |
| 1992 | 5591.6355872199 | 0.475753157454434 | 78.7838291946632 | 5512.85175802524 | Indonesia |
| 1993 | 5079.44636034077 | 0.484775240859362 | 77.1543235738512 | 5002.29203676692 | Indonesia |
| 1994 | 4614.16395665528 | 0.493607099523711 | 70.5363315042721 | 4543.62762515101 | Indonesia |
| 1995 | 4167.8946566433 | 0.502008381392835 | 16.5491684488624 | 4151.34548819444 | Indonesia |
| 1996 | 3731.84033461292 | 0.510108146446556 | 15.9389145854269 | 3715.90142002749 | Indonesia |
| 1997 | 3379.95434759133 | 0.51810218881129 | 15.3216921218413 | 3364.63265546949 | Indonesia |
| 1998 | 3138.09105179837 | 0.524045603868488 | 15.1202861582721 | 3122.9707656401 | Indonesia |
| 1999 | 2898.70531901063 | 0.529176329629629 | 15.0665563992594 | 2883.63876261137 | Indonesia |
| 2000 | 2651.61372623732 | 0.533789587270659 | 15.0662977501551 | 2636.54742848716 | Indonesia |
| 2001 | 2461.75932421077 | 0.537977981115366 | 15.0784568690791 | 2446.68086734169 | Indonesia |
| 2002 | 2283.20983899637 | 0.542245676060877 | 15.0242496430253 | 2268.18558935334 | Indonesia |
| 2003 | 2113.90671890343 | 0.546550264351478 | 14.5565201480338 | 2099.3501987554 | Indonesia |
| 2004 | 1957.53589735791 | 0.550902314227472 | 13.7706153675032 | 1943.76528199041 | Indonesia |
| 2005 | 1821.75156362223 | 0.555424913216581 | 12.9703049775205 | 1808.78125864471 | Indonesia |
| 2006 | 1681.67179574177 | 0.560265924242442 | 12.0896046271285 | 1669.58219111464 | Indonesia |
| 2007 | 1563.85172882005 | 0.565811896183758 | 10.4574596329242 | 1553.39426918713 | Indonesia |
| 2008 | 1450.06102522357 | 0.572225266933015 | 8.75573390341778 | 1441.30529132015 | Indonesia |
| 2009 | 1325.53617119798 | 0.578866902230273 | 8.77693968314512 | 1316.75923151483 | Indonesia |
| 2010 | 1203.21383732644 | 0.585982419406785 | 8.80246680487667 | 1194.41137052156 | Indonesia |
| 2011 | 1094.30596986737 | 0.593652984587084 | 8.80093109548556 | 1085.50503877188 | Indonesia |
| 2012 | 1009.5685300685 | 0.601185701344525 | 8.8371411613591 | 1000.73138890714 | Indonesia |
| 2013 | 945.46466163113 | 0.608509904334102 | 8.71901672814049 | 936.74564490299 | Indonesia |
| 2014 | 888.02231918627 | 0.615499887091834 | 9.08428545904633 | 878.938033727224 | Indonesia |
| 2015 | 835.92565323808 | 0.622222469472476 | 8.96231708591859 | 826.963336152161 | Indonesia |
| 2016 | 767.40108077825 | 0.62866662466714 | 8.39377883292052 | 759.00730194533 | Indonesia |
| 2017 | 721.797779139 | 0.634895744678987 | 6.92990009797168 | 714.867879041028 | Indonesia |
| 2018 | 687.63665613572 | 0.640948750163115 | 5.8588996824839 | 681.777756453236 | Indonesia |
| 2019 | 649.69720171001 | 0.64686545689526 | 6.36674376628662 | 643.330457943723 | Indonesia |
| 2020 | 604.58712694521 | 0.651926647922518 | 5.15782517774457 | 599.429301767465 | Indonesia |
| 2021 | 580.20319132671 | 0.656868336261681 | 4.94705973520351 | 575.256131591506 | Indonesia |
| 1990 | 13169.5969598585 | 0.264283163752662 | 1330.66489014702 | 11838.9320697115 | Lao People's Democratic Republic |
| 1991 | 12418.0969262058 | 0.268787617854408 | 1237.160509938 | 11180.9364162678 | Lao People's Democratic Republic |
| 1992 | 11683.2906219781 | 0.273435312470827 | 1213.96388470287 | 10469.3267372752 | Lao People's Democratic Republic |
| 1993 | 11556.3740403702 | 0.278140417914198 | 1165.22304123662 | 10391.1509991336 | Lao People's Democratic Republic |
| 1994 | 11390.8235420508 | 0.283270229855729 | 1159.94809897975 | 10230.875443071 | Lao People's Democratic Republic |
| 1995 | 9851.08322845209 | 0.288795341159004 | 1098.36844513508 | 8752.71478331701 | Lao People's Democratic Republic |
| 1996 | 9341.90475601931 | 0.294838372041673 | 1042.11761716668 | 8299.78713885263 | Lao People's Democratic Republic |
| 1997 | 8761.05348164403 | 0.301477967015192 | 961.336011452814 | 7799.71747019122 | Lao People's Democratic Republic |
| 1998 | 8230.36111546637 | 0.308402544731754 | 873.293371408867 | 7357.0677440575 | Lao People's Democratic Republic |
| 1999 | 7687.76444989936 | 0.315972615438411 | 781.208381573374 | 6906.55606832599 | Lao People's Democratic Republic |
| 2000 | 7200.82339182079 | 0.323947359495962 | 715.614163504922 | 6485.20922831587 | Lao People's Democratic Republic |
| 2001 | 6472.77464639295 | 0.332580408807204 | 644.703352555412 | 5828.07129383754 | Lao People's Democratic Republic |
| 2002 | 5967.88285573662 | 0.341667271144462 | 580.265937575843 | 5387.61691816078 | Lao People's Democratic Republic |
| 2003 | 5397.51082205541 | 0.350658178383381 | 557.058714840507 | 4840.4521072149 | Lao People's Democratic Republic |
| 2004 | 4907.59056131159 | 0.359681759216616 | 494.71062301613 | 4412.87993829546 | Lao People's Democratic Republic |
| 2005 | 4457.93554772349 | 0.368728677775792 | 409.874022794623 | 4048.06152492887 | Lao People's Democratic Republic |
| 2006 | 4021.10438149752 | 0.378122280697802 | 347.409749256809 | 3673.69463224071 | Lao People's Democratic Republic |
| 2007 | 3582.41743598782 | 0.387723649113709 | 286.36683779394 | 3296.05059819388 | Lao People's Democratic Republic |
| 2008 | 3372.98905642702 | 0.397011298824877 | 264.536841430617 | 3108.4522149964 | Lao People's Democratic Republic |
| 2009 | 3016.85867194039 | 0.406139021683818 | 239.662390414973 | 2777.19628152542 | Lao People's Democratic Republic |
| 2010 | 2704.84231203947 | 0.414865566497825 | 215.013804768952 | 2489.82850727052 | Lao People's Democratic Republic |
| 2011 | 2376.61110864583 | 0.42335452259834 | 177.39171317272 | 2199.21939547311 | Lao People's Democratic Republic |
| 2012 | 2136.4914559837 | 0.431149360954477 | 135.620273722479 | 2000.87118226122 | Lao People's Democratic Republic |
| 2013 | 1882.82902545502 | 0.438691038635463 | 126.730451772373 | 1756.09857368265 | Lao People's Democratic Republic |
| 2014 | 1645.10361792098 | 0.4458694242056 | 111.967933186564 | 1533.13568473442 | Lao People's Democratic Republic |
| 2015 | 1475.64086833873 | 0.452749603276049 | 105.363709650227 | 1370.2771586885 | Lao People's Democratic Republic |
| 2016 | 1320.90902321787 | 0.459583995539449 | 92.2899644946908 | 1228.61905872318 | Lao People's Democratic Republic |
| 2017 | 1168.43728948976 | 0.466168891657656 | 85.766994312129 | 1082.67029517763 | Lao People's Democratic Republic |
| 2018 | 1069.58165698214 | 0.472618102538068 | 82.8241619121979 | 986.757495069942 | Lao People's Democratic Republic |
| 2019 | 979.45996108347 | 0.478811372841658 | 77.4267542508127 | 902.033206832657 | Lao People's Democratic Republic |
| 2020 | 833.26380031499 | 0.484192959345392 | 77.1063785846417 | 756.157421730348 | Lao People's Democratic Republic |
| 2021 | 778.41690317738 | 0.489136091456599 | 75.1311533110453 | 703.285749866335 | Lao People's Democratic Republic |
| 1990 | 233.85894554467 | 0.545799405460294 | 14.584186243107 | 219.274759301563 | Malaysia |
| 1991 | 199.51287259366 | 0.551683548009526 | 13.7357415954646 | 185.777130998195 | Malaysia |
| 1992 | 193.9737399074 | 0.558186752245966 | 12.3187320337978 | 181.655007873602 | Malaysia |
| 1993 | 178.82724481935 | 0.565845656303133 | 10.3938068752785 | 168.433437944071 | Malaysia |
| 1994 | 153.54798744542 | 0.574380445989544 | 8.77294602339148 | 144.775041422029 | Malaysia |
| 1995 | 150.84883639901 | 0.583441102596569 | 8.80122462652813 | 142.047611772482 | Malaysia |
| 1996 | 120.30491733427 | 0.593152135005711 | 8.83847719645571 | 111.466440137814 | Malaysia |
| 1997 | 115.11044722664 | 0.603418105152686 | 8.91545618272677 | 106.194991043913 | Malaysia |
| 1998 | 106.36112898279 | 0.612645412003281 | 8.918984201065 | 97.442144781725 | Malaysia |
| 1999 | 98.68287646664 | 0.621724901078498 | 8.88418874638344 | 89.7986877202566 | Malaysia |
| 2000 | 80.29583243638 | 0.630523543762275 | 8.10144116579984 | 72.1943912705802 | Malaysia |
| 2001 | 75.83890669789 | 0.638257476929833 | 6.73426569262443 | 69.1046410052656 | Malaysia |
| 2002 | 71.7772805436 | 0.645044248017871 | 6.01435027116625 | 65.7629302724337 | Malaysia |
| 2003 | 67.18647807258 | 0.65088567830407 | 5.24334586882042 | 61.9431322037596 | Malaysia |
| 2004 | 67.57036378883 | 0.656243325827196 | 4.85164408406918 | 62.7187197047608 | Malaysia |
| 2005 | 70.8897867427 | 0.661242241061834 | 4.40893305117299 | 66.480853691527 | Malaysia |
| 2006 | 68.41232797551 | 0.665956287100151 | 4.15912047392829 | 64.2532075015817 | Malaysia |
| 2007 | 68.40169473109 | 0.670843725887123 | 3.84596517623067 | 64.5557295548593 | Malaysia |
| 2008 | 69.21118358701 | 0.676399910213952 | 3.596156521074 | 65.615027065936 | Malaysia |
| 2009 | 69.47269061535 | 0.682070653985994 | 3.581584097186 | 65.891106518164 | Malaysia |
| 2010 | 63.28376425612 | 0.688258621606068 | 3.38396726489941 | 59.8997969912206 | Malaysia |
| 2011 | 65.26367611243 | 0.69408340345083 | 3.17220312727136 | 62.0914729851586 | Malaysia |
| 2012 | 63.44785084658 | 0.699664734135674 | 2.87518285428835 | 60.5726679922916 | Malaysia |
| 2013 | 59.43441841874 | 0.70493071683803 | 3.00726401215 | 56.42715440659 | Malaysia |
| 2014 | 62.26381206612 | 0.709953756222256 | 2.70397023145625 | 59.5598418346638 | Malaysia |
| 2015 | 65.5003115078 | 0.714659871236347 | 2.69434320631688 | 62.8059683014831 | Malaysia |
| 2016 | 62.58492846445 | 0.719223424980679 | 2.62519862359965 | 59.9597298408504 | Malaysia |
| 2017 | 64.59572638198 | 0.724235411990887 | 2.38779953521466 | 62.2079268467653 | Malaysia |
| 2018 | 62.2190148113 | 0.72938807812528 | 2.25610752193031 | 59.9629072893697 | Malaysia |
| 2019 | 62.39358828023 | 0.734587751127645 | 2.11076363229866 | 60.2828246479313 | Malaysia |
| 2020 | 57.58781675799 | 0.738743992650626 | 1.82488293847072 | 55.7629338195193 | Malaysia |
| 2021 | 56.44869251416 | 0.742523828414751 | 1.80256551973128 | 54.6461269944287 | Malaysia |
| 1990 | 4386.79062675735 | 0.331601544442323 | 656.137573824479 | 3730.65305293287 | Maldives |
| 1991 | 3859.00233491643 | 0.344398606997735 | 573.352026396066 | 3285.65030852036 | Maldives |
| 1992 | 3337.88623302837 | 0.358252339504646 | 490.578118715709 | 2847.30811431266 | Maldives |
| 1993 | 2881.13669619631 | 0.372806998520911 | 356.214681759384 | 2524.92201443693 | Maldives |
| 1994 | 2424.55160404246 | 0.388081604129663 | 286.271708498727 | 2138.27989554373 | Maldives |
| 1995 | 1945.4969624927 | 0.403945582505039 | 240.874428226455 | 1704.62253426624 | Maldives |
| 1996 | 1763.77911544457 | 0.420519127829701 | 197.523383885338 | 1566.25573155923 | Maldives |
| 1997 | 1431.54374057921 | 0.437436025558501 | 127.293218579163 | 1304.25052200005 | Maldives |
| 1998 | 1262.4696491701 | 0.453973361371121 | 106.202397767295 | 1156.2672514028 | Maldives |
| 1999 | 1041.75000697123 | 0.469338065717424 | 82.9119680062689 | 958.838038964961 | Maldives |
| 2000 | 930.71085710802 | 0.483495670503175 | 77.319902798431 | 853.390954309589 | Maldives |
| 2001 | 802.75923839808 | 0.495923695035155 | 70.905929976864 | 731.853308421216 | Maldives |
| 2002 | 677.63542300813 | 0.507404859766873 | 16.047819084454 | 661.587603923676 | Maldives |
| 2003 | 614.5429519002 | 0.518926864396739 | 15.286638190295 | 599.256313709905 | Maldives |
| 2004 | 552.68648360213 | 0.529910190937908 | 15.0789430681144 | 537.607540534016 | Maldives |
| 2005 | 484.25861984599 | 0.53825333247429 | 15.0870776655811 | 469.171542180409 | Maldives |
| 2006 | 463.70652396939 | 0.547950411527519 | 14.5014032027953 | 449.205120766595 | Maldives |
| 2007 | 422.48957438776 | 0.557529246632533 | 12.0583299523907 | 410.431244435369 | Maldives |
| 2008 | 386.64570296903 | 0.566839634379964 | 9.85593749424273 | 376.789765474787 | Maldives |
| 2009 | 357.98256245178 | 0.574442059853853 | 8.9142058572409 | 349.068356594539 | Maldives |
| 2010 | 333.55904918838 | 0.581980986393534 | 8.8091807451175 | 324.749868443263 | Maldives |
| 2011 | 309.5560609553 | 0.589684459954112 | 9.04967862388212 | 300.506382331418 | Maldives |
| 2012 | 287.84522505313 | 0.596948519185372 | 8.8615392105777 | 278.983685842552 | Maldives |
| 2013 | 267.77645954608 | 0.604238519255647 | 8.75902705676475 | 259.017432489315 | Maldives |
| 2014 | 249.09205948135 | 0.611578071403856 | 8.72618114835256 | 240.365878332997 | Maldives |
| 2015 | 232.74438898314 | 0.618454389892933 | 8.76407851145812 | 223.980310471682 | Maldives |
| 2016 | 219.1620800663 | 0.624875949954596 | 8.78573713769827 | 210.376342928602 | Maldives |
| 2017 | 206.53864239244 | 0.631083288156097 | 8.32985790714203 | 198.208784485298 | Maldives |
| 2018 | 192.4667719107 | 0.637205659403498 | 6.60524254365614 | 185.861529367044 | Maldives |
| 2019 | 182.99268125455 | 0.643082793641918 | 6.40741556313494 | 176.585265691415 | Maldives |
| 2020 | 170.04681891326 | 0.647608589281031 | 6.07497610327782 | 163.971842809982 | Maldives |
| 2021 | 169.68481252958 | 0.650886627343107 | 5.0566143577622 | 164.628198171818 | Maldives |
| 1990 | 15853.7469957584 | 0.319219723709677 | 779.458755826012 | 15074.2882399324 | Myanmar |
| 1991 | 15388.5151246098 | 0.322034718697423 | 782.035110662856 | 14606.4800139469 | Myanmar |
| 1992 | 14655.708850833 | 0.325628883748026 | 725.92646214862 | 13929.7823886844 | Myanmar |
| 1993 | 13827.5082376559 | 0.329707434417887 | 719.132715651781 | 13108.3755220041 | Myanmar |
| 1994 | 12842.7939832669 | 0.334290120297598 | 650.523060150759 | 12192.2709231161 | Myanmar |
| 1995 | 11854.2072013808 | 0.339590975967104 | 616.030348012938 | 11238.1768533679 | Myanmar |
| 1996 | 10942.8246050897 | 0.345191641257382 | 576.214558005291 | 10366.6100470844 | Myanmar |
| 1997 | 10064.6057160385 | 0.350892883218861 | 563.121020066112 | 9501.48469597239 | Myanmar |
| 1998 | 9197.04580532523 | 0.356696902058465 | 553.041772828775 | 8644.00403249645 | Myanmar |
| 1999 | 8319.94463289815 | 0.36303773228042 | 468.722804103959 | 7851.22182879419 | Myanmar |
| 2000 | 7483.6812796252 | 0.370360017053284 | 402.692048201591 | 7080.98923142361 | Myanmar |
| 2001 | 6502.61560393179 | 0.378171980483692 | 349.744397863498 | 6152.87120606829 | Myanmar |
| 2002 | 5663.85150709796 | 0.386343628220189 | 317.089585875532 | 5346.76192122243 | Myanmar |
| 2003 | 4949.43296738074 | 0.395218766380276 | 264.569041005304 | 4684.86392637544 | Myanmar |
| 2004 | 4637.65352998171 | 0.40469235815513 | 239.072968292236 | 4398.58056168947 | Myanmar |
| 2005 | 4272.87443102605 | 0.414662082498037 | 217.448274313143 | 4055.42615671291 | Myanmar |
| 2006 | 3856.22223463432 | 0.424705070565889 | 176.653007685651 | 3679.56922694867 | Myanmar |
| 2007 | 3460.22208234952 | 0.434536927066905 | 132.422854256922 | 3327.7992280926 | Myanmar |
| 2008 | 3175.03449071719 | 0.443595531234043 | 111.267926119444 | 3063.76656459775 | Myanmar |
| 2009 | 2889.53283213566 | 0.451954883959276 | 106.601500726414 | 2782.93133140925 | Myanmar |
| 2010 | 2672.61631223408 | 0.460180429354898 | 92.3923528932042 | 2580.22395934088 | Myanmar |
| 2011 | 2518.3911674557 | 0.467850871609568 | 85.70928054323 | 2432.68188691247 | Myanmar |
| 2012 | 2412.30305236222 | 0.475336422547245 | 77.7592853492819 | 2334.54376701294 | Myanmar |
| 2013 | 2350.75773046743 | 0.482821855058075 | 76.7756667690866 | 2273.98206369834 | Myanmar |
| 2014 | 2250.78681171312 | 0.490255145456161 | 75.7400651625375 | 2175.04674655058 | Myanmar |
| 2015 | 2118.14659327255 | 0.497374156127321 | 26.1175320391572 | 2092.02906123339 | Myanmar |
| 2016 | 2000.08278153071 | 0.504115978117272 | 16.1819664505149 | 1983.9008150802 | Myanmar |
| 2017 | 1848.01637131968 | 0.510823431317416 | 15.702545295871 | 1832.31382602381 | Myanmar |
| 2018 | 1693.68478752504 | 0.517557469010629 | 15.3525928534412 | 1678.3321946716 | Myanmar |
| 2019 | 1564.5984606419 | 0.524108970350153 | 15.0664241656638 | 1549.53203647624 | Myanmar |
| 2020 | 1347.80160294646 | 0.529434702877063 | 15.0410020121761 | 1332.76060093428 | Myanmar |
| 2021 | 1241.03315508961 | 0.533900839611906 | 15.1171970113793 | 1225.91595807823 | Myanmar |
| 1990 | 2692.37857928898 | 0.510011796397722 | 15.9050566793837 | 2676.4735226096 | Philippines |
| 1991 | 2452.27325516089 | 0.513707183203398 | 15.7533789660622 | 2436.51987619483 | Philippines |
| 1992 | 2274.8764073293 | 0.517109502298333 | 15.3744290196401 | 2259.50197830966 | Philippines |
| 1993 | 2117.15537144323 | 0.520600378172241 | 15.1499513033575 | 2102.00542013987 | Philippines |
| 1994 | 1898.01461620729 | 0.524613606961947 | 15.0567252259356 | 1882.95789098135 | Philippines |
| 1995 | 1735.1463308307 | 0.528827907097189 | 15.0624551062527 | 1720.08387572445 | Philippines |
| 1996 | 1571.15293981227 | 0.532938848718821 | 15.1417239635536 | 1556.01121584872 | Philippines |
| 1997 | 1431.97763441991 | 0.536589867582292 | 15.0573223735311 | 1416.92031204638 | Philippines |
| 1998 | 1317.44523654632 | 0.539496470377039 | 15.0424192573026 | 1302.40281728902 | Philippines |
| 1999 | 1314.70874402507 | 0.542257968063782 | 15.0292702230385 | 1299.67947380203 | Philippines |
| 2000 | 1286.0314088856 | 0.544997722532077 | 15.0122054036868 | 1271.01920348191 | Philippines |
| 2001 | 1212.48654513313 | 0.547409431560733 | 14.4706952456418 | 1198.01584988749 | Philippines |
| 2002 | 1124.20658585756 | 0.549521153378409 | 13.8655202648821 | 1110.34106559268 | Philippines |
| 2003 | 1031.45334147156 | 0.551567967518738 | 13.8732612757758 | 1017.58008019578 | Philippines |
| 2004 | 923.95035255887 | 0.553704287358934 | 12.8312066252487 | 911.119145933621 | Philippines |
| 2005 | 900.82077995683 | 0.555664102826939 | 13.1407734052535 | 887.680006551577 | Philippines |
| 2006 | 857.31643684792 | 0.557976265254754 | 12.267539938162 | 845.048896909758 | Philippines |
| 2007 | 823.22691441182 | 0.560822023745201 | 11.2861853634917 | 811.940729048328 | Philippines |
| 2008 | 786.54382351586 | 0.563974721376474 | 10.9230654689361 | 775.620758046924 | Philippines |
| 2009 | 754.25588415106 | 0.567355819963632 | 9.94441147208044 | 744.31147267898 | Philippines |
| 2010 | 712.80059909123 | 0.571765888250344 | 8.61053318168083 | 704.190065909549 | Philippines |
| 2011 | 677.36058811924 | 0.577240283466517 | 8.69503104938062 | 668.665557069859 | Philippines |
| 2012 | 616.53051891805 | 0.58376762501942 | 8.84958832978608 | 607.680930588264 | Philippines |
| 2013 | 554.93361129724 | 0.590837549266941 | 8.66672658848132 | 546.266884708759 | Philippines |
| 2014 | 517.38293565211 | 0.598384520042983 | 8.78017191930612 | 508.602763732804 | Philippines |
| 2015 | 482.89658659998 | 0.606215624393378 | 8.95888988432768 | 473.937696715652 | Philippines |
| 2016 | 470.98725729373 | 0.614224960974851 | 8.79224777473076 | 462.195009518999 | Philippines |
| 2017 | 443.01030810054 | 0.622315028960912 | 8.66419234161789 | 434.346115758922 | Philippines |
| 2018 | 418.87629816166 | 0.630464275930184 | 8.03102289497512 | 410.845275266685 | Philippines |
| 2019 | 403.22401593189 | 0.638547384381803 | 6.58096900893765 | 396.643046922952 | Philippines |
| 2020 | 371.86520508111 | 0.644867233233664 | 6.165990661155 | 365.699214419955 | Philippines |
| 2021 | 356.53869504574 | 0.651219328702707 | 5.13173881301053 | 351.406956232729 | Philippines |
| 1990 | 699.24159596203 | 0.522622552851521 | 15.2115046189242 | 684.030091343106 | Sri Lanka |
| 1991 | 577.13458508279 | 0.528239202759485 | 15.1142039221801 | 562.02038116061 | Sri Lanka |
| 1992 | 525.96107380003 | 0.533345535471094 | 15.0615002642035 | 510.899573535827 | Sri Lanka |
| 1993 | 498.58880815193 | 0.538891102297622 | 15.1363465077772 | 483.452461644153 | Sri Lanka |
| 1994 | 452.56272130127 | 0.545124551180714 | 15.0431499510378 | 437.519571350232 | Sri Lanka |
| 1995 | 371.29434933399 | 0.551744058346111 | 13.9448914764071 | 357.349457857583 | Sri Lanka |
| 1996 | 350.88349172026 | 0.558938887614985 | 12.3303980447573 | 338.553093675503 | Sri Lanka |
| 1997 | 312.85435302078 | 0.566128422363729 | 10.3830490896364 | 302.471303931144 | Sri Lanka |
| 1998 | 264.71096705177 | 0.572326186668561 | 8.85007960152937 | 255.860887450241 | Sri Lanka |
| 1999 | 219.66324515884 | 0.57763004764168 | 9.0135723590568 | 210.649672799783 | Sri Lanka |
| 2000 | 200.26470128272 | 0.582942637570578 | 8.67852252654901 | 191.586178756171 | Sri Lanka |
| 2001 | 186.58593225956 | 0.587556599070554 | 8.72953682867667 | 177.856395430883 | Sri Lanka |
| 2002 | 165.79454555382 | 0.59235912694583 | 8.81249647524403 | 156.982049078576 | Sri Lanka |
| 2003 | 154.00510769651 | 0.597410284061105 | 8.87634567469714 | 145.128762021813 | Sri Lanka |
| 2004 | 146.40807800225 | 0.60225006214936 | 8.89990969952485 | 137.508168302725 | Sri Lanka |
| 2005 | 151.06849089806 | 0.606906655593188 | 8.98404974397099 | 142.084441154089 | Sri Lanka |
| 2006 | 137.28528709581 | 0.611807083317854 | 8.91767809006329 | 128.367609005747 | Sri Lanka |
| 2007 | 125.53011027705 | 0.616979265050006 | 8.73636080489062 | 116.793749472159 | Sri Lanka |
| 2008 | 123.0444512383 | 0.622414317259024 | 8.80382140654968 | 114.24062983175 | Sri Lanka |
| 2009 | 120.6820066517 | 0.628149630493025 | 8.04536750003957 | 112.63663915166 | Sri Lanka |
| 2010 | 113.6377545244 | 0.634263123413356 | 6.87485714197465 | 106.762897382425 | Sri Lanka |
| 2011 | 104.36358285821 | 0.640799571244289 | 6.29532871584216 | 98.0682541423678 | Sri Lanka |
| 2012 | 94.76785980743 | 0.648049815659369 | 6.05317264011781 | 88.7146871673122 | Sri Lanka |
| 2013 | 90.24132808737 | 0.655435837718287 | 4.90859450386907 | 85.3327335835009 | Sri Lanka |
| 2014 | 81.9280044431 | 0.662782434226903 | 4.47052409017344 | 77.4574803529266 | Sri Lanka |
| 2015 | 74.37556291354 | 0.669760291875565 | 3.86344388480958 | 70.5121190287304 | Sri Lanka |
| 2016 | 69.3253734225 | 0.67627744148358 | 3.58177014692141 | 65.7436032755786 | Sri Lanka |
| 2017 | 65.20315557119 | 0.682405274024772 | 3.5784478971046 | 61.6247076740854 | Sri Lanka |
| 2018 | 61.99329433565 | 0.688125858195178 | 3.32607872908281 | 58.6672156065672 | Sri Lanka |
| 2019 | 59.08588303457 | 0.69351606326589 | 3.13837352733148 | 55.9475095072385 | Sri Lanka |
| 2020 | 56.73566696786 | 0.697648535082413 | 3.09759756146256 | 53.6380694063974 | Sri Lanka |
| 2021 | 51.15583683067 | 0.70153493519423 | 2.95852913006397 | 48.197307700606 | Sri Lanka |
| 1990 | 428.72767900642 | 0.506644860754466 | 15.9810775631569 | 412.746601443263 | Thailand |
| 1991 | 381.81962421653 | 0.515911465809998 | 15.3561210729445 | 366.463503143585 | Thailand |
| 1992 | 338.44070024946 | 0.525161868897941 | 15.0450220588806 | 323.395678190579 | Thailand |
| 1993 | 300.31056971628 | 0.534303709087494 | 15.0679256526993 | 285.242644063581 | Thailand |
| 1994 | 267.44131583011 | 0.543325546554827 | 15.0140051080337 | 252.427310722076 | Thailand |
| 1995 | 240.05445300552 | 0.552452208814004 | 13.8401877942329 | 226.214265211287 | Thailand |
| 1996 | 219.6329645618 | 0.561302486534141 | 11.3723669264501 | 208.26059763535 | Thailand |
| 1997 | 211.76878498345 | 0.568449492093676 | 9.42581942102387 | 202.342965562426 | Thailand |
| 1998 | 216.38745906304 | 0.573817902004847 | 8.91404066778384 | 207.473418395256 | Thailand |
| 1999 | 216.446964128 | 0.578854167794919 | 8.90655849246578 | 207.540405635534 | Thailand |
| 2000 | 213.80243952849 | 0.58350091705013 | 8.93881964312577 | 204.863619885364 | Thailand |
| 2001 | 196.52071185558 | 0.587661560775708 | 9.00705284368508 | 187.513659011895 | Thailand |
| 2002 | 184.69801019875 | 0.59175909789129 | 8.8851505104077 | 175.812859688342 | Thailand |
| 2003 | 172.31996589193 | 0.596048920136592 | 9.01368089475726 | 163.306284997173 | Thailand |
| 2004 | 161.6993364416 | 0.600520665140167 | 8.92675080342429 | 152.772585638176 | Thailand |
| 2005 | 149.57695099549 | 0.605040775160747 | 8.76910184994393 | 140.807849145546 | Thailand |
| 2006 | 137.33930901335 | 0.610356678024317 | 8.81249829620068 | 128.526810717149 | Thailand |
| 2007 | 119.62758529739 | 0.616368591131489 | 9.14475276961814 | 110.482832527772 | Thailand |
| 2008 | 96.73275538297 | 0.621067542847306 | 8.85798979970056 | 87.8747655832694 | Thailand |
| 2009 | 77.07483693202 | 0.624329070216978 | 8.72248382167256 | 68.3523531103475 | Thailand |
| 2010 | 68.87278622382 | 0.62858943983977 | 8.18991874887444 | 60.6828674749456 | Thailand |
| 2011 | 63.26177075653 | 0.633226727672561 | 6.74806502878644 | 56.5137057277436 | Thailand |
| 2012 | 59.77761293646 | 0.638410972213962 | 6.60956967212111 | 53.1680432643389 | Thailand |
| 2013 | 55.03843525925 | 0.643672915801455 | 6.14591373024273 | 48.8925215290073 | Thailand |
| 2014 | 52.74102802426 | 0.648772057471972 | 5.99298272059541 | 46.7480453036646 | Thailand |
| 2015 | 51.34317518573 | 0.654015622617677 | 4.86205253120528 | 46.4811226545247 | Thailand |
| 2016 | 53.28178849434 | 0.659369302952365 | 4.36413483661083 | 48.9176536577292 | Thailand |
| 2017 | 54.14407780534 | 0.664804133690469 | 4.24863465811154 | 49.8954431472285 | Thailand |
| 2018 | 57.61150587774 | 0.670225586853729 | 3.86155640270247 | 53.7499494750375 | Thailand |
| 2019 | 57.90030639248 | 0.675273900833315 | 3.58608890269082 | 54.3142174897892 | Thailand |
| 2020 | 57.87048933353 | 0.679119989171207 | 3.60452723117473 | 54.2659621023553 | Thailand |
| 2021 | 57.76213160306 | 0.682547932974593 | 3.53078343361286 | 54.2313481694471 | Thailand |
| 1990 | 8946.87272569783 | 0.262468083434918 | 1359.98673897182 | 7586.88598672601 | Timor-Leste |
| 1991 | 8386.14065946312 | 0.270943348789144 | 1234.62492379994 | 7151.51573566318 | Timor-Leste |
| 1992 | 7791.25156365998 | 0.279916760012752 | 1154.4521673894 | 6636.79939627058 | Timor-Leste |
| 1993 | 7213.8889556959 | 0.289266628937942 | 1098.73830664559 | 6115.15064905031 | Timor-Leste |
| 1994 | 6605.97878408019 | 0.298763188841394 | 984.540905918636 | 5621.43787816155 | Timor-Leste |
| 1995 | 6000.5652691355 | 0.308018683645478 | 859.197057950151 | 5141.36821118535 | Timor-Leste |
| 1996 | 5402.86262003101 | 0.317588750054214 | 778.262679403487 | 4624.59994062752 | Timor-Leste |
| 1997 | 4859.19422219933 | 0.326705373863583 | 721.410084968917 | 4137.78413723041 | Timor-Leste |
| 1998 | 4373.14873447224 | 0.334894497001343 | 650.736162257516 | 3722.41257221472 | Timor-Leste |
| 1999 | 4065.78686536923 | 0.339252140436899 | 597.962779842092 | 3467.82408552714 | Timor-Leste |
| 2000 | 3696.20127235705 | 0.343793649201481 | 576.038033231261 | 3120.16323912579 | Timor-Leste |
| 2001 | 3265.1702146728 | 0.349490320159863 | 560.491424847041 | 2704.67878982576 | Timor-Leste |
| 2002 | 2915.40274393146 | 0.354162019516716 | 552.410337350207 | 2362.99240658125 | Timor-Leste |
| 2003 | 2672.58332426427 | 0.35844002540281 | 498.402300260772 | 2174.1810240035 | Timor-Leste |
| 2004 | 2278.55837098264 | 0.362687998477417 | 466.129650372049 | 1812.42872061059 | Timor-Leste |
| 2005 | 2043.336034433 | 0.366913584279333 | 413.961562776419 | 1629.37447165658 | Timor-Leste |
| 2006 | 1759.0978021341 | 0.370654092006941 | 402.880664194355 | 1356.21713793974 | Timor-Leste |
| 2007 | 1561.90758665187 | 0.375390767982559 | 351.183502127531 | 1210.72408452434 | Timor-Leste |
| 2008 | 1342.2063083853 | 0.381227527778766 | 323.750200188305 | 1018.456108197 | Timor-Leste |
| 2009 | 1226.92110358953 | 0.387944823498752 | 283.853140675061 | 943.06796291447 | Timor-Leste |
| 2010 | 1117.61467399578 | 0.394887019369648 | 281.268322241402 | 836.346351754378 | Timor-Leste |
| 2011 | 1056.75133067162 | 0.401872356301348 | 233.21969926453 | 823.53163140709 | Timor-Leste |
| 2012 | 985.18957513393 | 0.408910300117845 | 222.622970929343 | 762.566604204587 | Timor-Leste |
| 2013 | 904.99338986485 | 0.41521787782997 | 216.264131259655 | 688.729258605195 | Timor-Leste |
| 2014 | 851.6000533857 | 0.421155537971188 | 194.139048217555 | 657.461005168145 | Timor-Leste |
| 2015 | 813.78707355051 | 0.426383620035982 | 176.690324993003 | 637.096748557507 | Timor-Leste |
| 2016 | 777.3407796915 | 0.431207272013621 | 136.902098028484 | 640.438681663016 | Timor-Leste |
| 2017 | 742.47429895544 | 0.434519733594658 | 131.390983156872 | 611.083315798568 | Timor-Leste |
| 2018 | 716.7179686674 | 0.437018662969572 | 127.764937629965 | 588.953031037435 | Timor-Leste |
| 2019 | 640.58800003547 | 0.44019584549571 | 126.110306567499 | 514.477693467971 | Timor-Leste |
| 2020 | 620.33475750956 | 0.442349336035765 | 112.970709690137 | 507.364047819423 | Timor-Leste |
| 2021 | 602.54520770706 | 0.444667618881833 | 111.593411620566 | 490.951796086493 | Timor-Leste |
| 1990 | 302.432447615 | 0.407630047693296 | 240.777909856003 | 61.654537758997 | Viet Nam |
| 1991 | 276.76014546705 | 0.413217249557429 | 213.808706610446 | 62.9514388566038 | Viet Nam |
| 1992 | 258.81401708257 | 0.419781657140794 | 196.922890327544 | 61.8911267550257 | Viet Nam |
| 1993 | 224.42901121967 | 0.427072184949202 | 177.290308501066 | 47.1387027186038 | Viet Nam |
| 1994 | 201.49719148906 | 0.435106272492167 | 129.608875825998 | 71.8883156630618 | Viet Nam |
| 1995 | 177.49363887608 | 0.443858785203356 | 115.805417618491 | 61.6882212575894 | Viet Nam |
| 1996 | 153.66662590384 | 0.453102410398964 | 107.888504806404 | 45.778121097436 | Viet Nam |
| 1997 | 135.07774234853 | 0.462853518365539 | 92.2840048384509 | 42.7937375100791 | Viet Nam |
| 1998 | 120.69059215795 | 0.47247889159957 | 82.661253770838 | 38.029338387112 | Viet Nam |
| 1999 | 108.33869347671 | 0.48144577410536 | 76.9748608913741 | 31.3638325853359 | Viet Nam |
| 2000 | 97.30141100766 | 0.489802045414549 | 75.3812622715588 | 21.9201487361012 | Viet Nam |
| 2001 | 86.35787168839 | 0.497630349065707 | 21.3046585784266 | 65.0532131099634 | Viet Nam |
| 2002 | 76.78401603283 | 0.505453447065355 | 16.212900422822 | 60.571115610008 | Viet Nam |
| 2003 | 70.14590631181 | 0.513367511118634 | 15.7279396617882 | 54.4179666500218 | Viet Nam |
| 2004 | 64.47260465168 | 0.521229389687461 | 15.1104777207873 | 49.3621269308927 | Viet Nam |
| 2005 | 58.05669077673 | 0.528956332075378 | 15.0652333474494 | 42.9914574292806 | Viet Nam |
| 2006 | 51.58396311246 | 0.53630065301368 | 15.0280117011912 | 36.5559514112688 | Viet Nam |
| 2007 | 46.15035944576 | 0.543342652110377 | 15.0209783380665 | 31.1293811076935 | Viet Nam |
| 2008 | 41.95951282935 | 0.550127119086299 | 13.9530665746441 | 28.0064462547059 | Viet Nam |
| 2009 | 38.14028991965 | 0.55645594448371 | 13.0110199512925 | 25.1292699683575 | Viet Nam |
| 2010 | 34.84876197348 | 0.562768571224979 | 11.3497942943024 | 23.4989676791776 | Viet Nam |
| 2011 | 32.37627681812 | 0.568945458254757 | 9.63654759114963 | 22.7397292269704 | Viet Nam |
| 2012 | 29.25561639672 | 0.575078671720175 | 8.93020302652224 | 20.3254133701978 | Viet Nam |
| 2013 | 26.16596763218 | 0.581029214733769 | 8.82505163875984 | 17.3409159934202 | Viet Nam |
| 2014 | 23.7511291163 | 0.586913814919078 | 8.75851587747 | 14.99261323883 | Viet Nam |
| 2015 | 22.47721442809 | 0.592849573281283 | 8.81102595775781 | 13.6661884703322 | Viet Nam |
| 2016 | 21.22281849998 | 0.598849485299412 | 8.88745019172609 | 12.3353683082539 | Viet Nam |
| 2017 | 20.10767393581 | 0.605019420569412 | 8.83845366366218 | 11.2692202721478 | Viet Nam |
| 2018 | 19.13406161605 | 0.611351406113218 | 8.76813651324824 | 10.3659251028018 | Viet Nam |
| 2019 | 18.21898405682 | 0.6177338152062 | 8.80211836738775 | 9.41686568943225 | Viet Nam |
| 2020 | 17.4484904008 | 0.623090320139283 | 8.99439094939196 | 8.45409945140804 | Viet Nam |
| 2021 | 15.13919141605 | 0.627933720680671 | 8.25259484940268 | 6.88659656664732 | Viet Nam |
| 1990 | 418.18439852083 | 0.53464890774309 | 15.0872258509384 | 403.097172669892 | Fiji |
| 1991 | 409.03233391247 | 0.538778259951718 | 15.0642112343955 | 393.968122678075 | Fiji |
| 1992 | 388.06236275979 | 0.543188551973659 | 15.0142114045298 | 373.04815135526 | Fiji |
| 1993 | 386.76489504422 | 0.547776473718793 | 14.5559568712609 | 372.208938172959 | Fiji |
| 1994 | 375.71728065087 | 0.552849019704515 | 13.7103887554945 | 362.006891895375 | Fiji |
| 1995 | 361.6281989266 | 0.558530972290103 | 11.6973634298905 | 349.93083549671 | Fiji |
| 1996 | 350.4604085552 | 0.565041878958725 | 10.9638260249938 | 339.496582530206 | Fiji |
| 1997 | 337.99704677242 | 0.571187752703497 | 8.85909980451778 | 329.137946967902 | Fiji |
| 1998 | 347.60370106504 | 0.577172353765379 | 8.95861556054256 | 338.645085504497 | Fiji |
| 1999 | 340.71869806213 | 0.583573942431191 | 8.99579097271174 | 331.722907089418 | Fiji |
| 2000 | 333.45170306617 | 0.589196631128333 | 8.70356323489791 | 324.748139831272 | Fiji |
| 2001 | 329.04825837032 | 0.59449741944042 | 8.79760073035817 | 320.250657639962 | Fiji |
| 2002 | 327.44185476547 | 0.59932203091066 | 8.65859732388613 | 318.783257441584 | Fiji |
| 2003 | 325.55569794087 | 0.603489622121197 | 8.7363624339732 | 316.819335506897 | Fiji |
| 2004 | 334.91519229621 | 0.607544889853603 | 8.83376488895888 | 326.081427407251 | Fiji |
| 2005 | 335.19735472361 | 0.610812943594047 | 8.76467645974122 | 326.432678263869 | Fiji |
| 2006 | 341.44567692163 | 0.613932767413255 | 8.84808843163452 | 332.597588489995 | Fiji |
| 2007 | 353.8357483979 | 0.616582915169589 | 8.79740864540037 | 345.0383397525 | Fiji |
| 2008 | 351.94626220023 | 0.618958809102014 | 8.78998987760096 | 343.156272322629 | Fiji |
| 2009 | 351.23789673652 | 0.620883465082065 | 8.94161872025911 | 342.296278016261 | Fiji |
| 2010 | 351.93895566869 | 0.62330878605608 | 8.70478950590102 | 343.234166162789 | Fiji |
| 2011 | 343.52480343885 | 0.626438251319165 | 8.69716985901833 | 334.827633579832 | Fiji |
| 2012 | 332.6428728565 | 0.629881334893454 | 8.36708715744519 | 324.275785699055 | Fiji |
| 2013 | 281.153263327 | 0.634278114312534 | 7.14965455463458 | 274.003608772365 | Fiji |
| 2014 | 264.06568688013 | 0.6393900664467 | 6.6005755655931 | 257.465111314537 | Fiji |
| 2015 | 254.69754751066 | 0.645026352546626 | 6.10649753191975 | 248.59104997874 | Fiji |
| 2016 | 249.31242408479 | 0.650742680102873 | 5.04117271586878 | 244.271251368921 | Fiji |
| 2017 | 238.6822505754 | 0.656464010258719 | 5.06555334834708 | 233.616697227053 | Fiji |
| 2018 | 229.06851445592 | 0.661991453647996 | 4.29110366525012 | 224.77741079067 | Fiji |
| 2019 | 217.14200132644 | 0.667060979290814 | 3.87351887916407 | 213.268482447276 | Fiji |
| 2020 | 208.33269102674 | 0.671431490601499 | 3.76571533053091 | 204.566975696209 | Fiji |
| 2021 | 199.39605351873 | 0.675051630810876 | 3.60634178092476 | 195.789711737805 | Fiji |
| 1990 | 3244.44103977967 | 0.410389821247376 | 218.397613174423 | 3026.04342660525 | Kiribati |
| 1991 | 3078.54928612009 | 0.412884199130178 | 216.913538706038 | 2861.63574741405 | Kiribati |
| 1992 | 2894.21252549699 | 0.415577609430112 | 197.919843744472 | 2696.29268175252 | Kiribati |
| 1993 | 2719.05379301543 | 0.418481660624087 | 196.033706569611 | 2523.02008644582 | Kiribati |
| 1994 | 2530.65342713646 | 0.421716217125176 | 194.236065430224 | 2336.41736170624 | Kiribati |
| 1995 | 2368.13581881776 | 0.425024354020195 | 175.867616444894 | 2192.26820237287 | Kiribati |
| 1996 | 2235.66162474184 | 0.428300682254543 | 164.262168927008 | 2071.39945581483 | Kiribati |
| 1997 | 2079.7293636267 | 0.431641940565898 | 138.5495872222 | 1941.1797764045 | Kiribati |
| 1998 | 1940.75759442562 | 0.435741904126434 | 126.571562700775 | 1814.18603172485 | Kiribati |
| 1999 | 1904.24347547571 | 0.439692923227333 | 127.732251614581 | 1776.51122386113 | Kiribati |
| 2000 | 1784.36577032369 | 0.444436339062538 | 110.962780234176 | 1673.40299008951 | Kiribati |
| 2001 | 1669.94453120908 | 0.448782300735609 | 106.496531119238 | 1563.44800008984 | Kiribati |
| 2002 | 1555.39446915669 | 0.453462297779448 | 107.128475613696 | 1448.26599354299 | Kiribati |
| 2003 | 1448.02848399528 | 0.458208373737044 | 92.6757159592546 | 1355.35276803603 | Kiribati |
| 2004 | 1351.93751982228 | 0.462336213231137 | 89.4106534294001 | 1262.52686639288 | Kiribati |
| 2005 | 1287.65984407834 | 0.466722276344394 | 84.7596850775242 | 1202.90015900082 | Kiribati |
| 2006 | 1241.42929542906 | 0.470557196660738 | 82.6339641580106 | 1158.79533127105 | Kiribati |
| 2007 | 1255.4917278918 | 0.474227450118041 | 80.4878411933737 | 1175.00388669843 | Kiribati |
| 2008 | 1233.32606223783 | 0.47720193306214 | 77.8825648641696 | 1155.44349737366 | Kiribati |
| 2009 | 1215.55416092087 | 0.480035601443872 | 76.6899593442942 | 1138.86420157658 | Kiribati |
| 2010 | 1201.10086829391 | 0.482447689410665 | 76.7180596035817 | 1124.38280869033 | Kiribati |
| 2011 | 1179.4059058421 | 0.485028211089828 | 77.2148147403495 | 1102.19109110175 | Kiribati |
| 2012 | 1118.22495867939 | 0.488175863692029 | 75.4561253858 | 1042.76883329359 | Kiribati |
| 2013 | 1077.56810959944 | 0.491880767182311 | 76.0345072708765 | 1001.53360232856 | Kiribati |
| 2014 | 1035.15138408884 | 0.495317241227584 | 70.7888828145369 | 964.362501274303 | Kiribati |
| 2015 | 920.89274135731 | 0.500059699036215 | 16.7449652830528 | 904.147776074257 | Kiribati |
| 2016 | 870.85883921975 | 0.505207837257253 | 16.1761653554535 | 854.682673864297 | Kiribati |
| 2017 | 832.36171255893 | 0.510019240979789 | 15.9750952715901 | 816.38661728734 | Kiribati |
| 2018 | 782.33009040666 | 0.514810383548853 | 15.7803576587789 | 766.549732747881 | Kiribati |
| 2019 | 735.24095087091 | 0.519441321098362 | 15.328046399129 | 719.912904471781 | Kiribati |
| 2020 | 720.15140937074 | 0.523454113096381 | 15.1111553130786 | 705.040254057661 | Kiribati |
| 2021 | 691.23712091828 | 0.527186582842681 | 15.0412329118809 | 676.195888006399 | Kiribati |
| 1990 | 480.41391884665 | 0.430839288199048 | 142.551522691244 | 337.862396155406 | Marshall Islands |
| 1991 | 471.51968752682 | 0.435571411977162 | 128.105846754658 | 343.413840772162 | Marshall Islands |
| 1992 | 445.85008629673 | 0.440491018138956 | 125.252226797697 | 320.597859499033 | Marshall Islands |
| 1993 | 416.74594861735 | 0.445479084259875 | 111.163251252301 | 305.582697365049 | Marshall Islands |
| 1994 | 391.96040996182 | 0.450848221273844 | 104.970201364748 | 286.990208597072 | Marshall Islands |
| 1995 | 369.59732663722 | 0.45685967435209 | 98.4851624135385 | 271.112164223682 | Marshall Islands |
| 1996 | 356.72076651354 | 0.461209430189201 | 91.287358120815 | 265.433408392725 | Marshall Islands |
| 1997 | 337.73986230442 | 0.464752728246042 | 92.2540642970995 | 245.48579800732 | Marshall Islands |
| 1998 | 332.01695463919 | 0.468133032354917 | 85.2502869561526 | 246.766667683037 | Marshall Islands |
| 1999 | 330.310611066 | 0.471292726535272 | 82.8776565984332 | 247.432954467567 | Marshall Islands |
| 2000 | 1161.66687630934 | 0.474562536385418 | 80.8211840097431 | 1080.8456922996 | Marshall Islands |
| 2001 | 317.85604962144 | 0.478588316369524 | 76.9044944128854 | 240.951555208555 | Marshall Islands |
| 2002 | 312.10157360218 | 0.482776259493159 | 77.2543165950156 | 234.847257007164 | Marshall Islands |
| 2003 | 311.39841461614 | 0.486248620277286 | 75.0570355762038 | 236.341379039936 | Marshall Islands |
| 2004 | 307.06829795338 | 0.489573077431328 | 75.3835817647803 | 231.6847161886 | Marshall Islands |
| 2005 | 303.70358186414 | 0.49327139054966 | 73.089390117634 | 230.614191746506 | Marshall Islands |
| 2006 | 292.10741155507 | 0.497456174718598 | 24.0413114983151 | 268.066100056755 | Marshall Islands |
| 2007 | 280.46976432792 | 0.502250042471655 | 16.6549443913146 | 263.814819936605 | Marshall Islands |
| 2008 | 262.8436281678 | 0.506359587700651 | 16.1022886828654 | 246.741339484935 | Marshall Islands |
| 2009 | 243.20520094272 | 0.510794535917681 | 15.6444738294391 | 227.560727113281 | Marshall Islands |
| 2010 | 242.46452557042 | 0.51608513761736 | 15.3511684653966 | 227.113357105023 | Marshall Islands |
| 2011 | 235.29436811285 | 0.521177086955837 | 15.1093218699705 | 220.18504624288 | Marshall Islands |
| 2012 | 228.76501320605 | 0.526042019095978 | 15.0817825803899 | 213.68323062566 | Marshall Islands |
| 2013 | 221.66751896652 | 0.531446300319586 | 15.0923435032738 | 206.575175463246 | Marshall Islands |
| 2014 | 216.80806803665 | 0.536640130947104 | 15.0989752795762 | 201.709092757074 | Marshall Islands |
| 2015 | 211.178501877 | 0.541793166838195 | 15.0419198517422 | 196.136582025258 | Marshall Islands |
| 2016 | 217.21859913236 | 0.546782936000428 | 14.438371686223 | 202.780227446137 | Marshall Islands |
| 2017 | 198.87615376396 | 0.55206515210282 | 13.8845530819613 | 184.991600681999 | Marshall Islands |
| 2018 | 189.70127370921 | 0.557599278468739 | 11.8704284794383 | 177.830845229772 | Marshall Islands |
| 2019 | 177.14824252847 | 0.563428749789962 | 10.9266417242022 | 166.221600804268 | Marshall Islands |
| 2020 | 179.96630865452 | 0.568837509186186 | 9.44095082710165 | 170.525357827418 | Marshall Islands |
| 2021 | 169.85774677689 | 0.574091128177309 | 8.81748480105906 | 161.040261975831 | Marshall Islands |
| 1990 | 550.8930338915 | 0.462511616715497 | 89.4476199295963 | 461.445413961904 | Micronesia (Federated States of) |
| 1991 | 465.45645864087 | 0.468144831239529 | 84.8974556322799 | 380.55900300859 | Micronesia (Federated States of) |
| 1992 | 439.71190110331 | 0.473792936437038 | 79.672435652917 | 360.039465450393 | Micronesia (Federated States of) |
| 1993 | 413.13276937049 | 0.479939595446532 | 77.57413101503 | 335.55863835546 | Micronesia (Federated States of) |
| 1994 | 389.55250571762 | 0.485300005800881 | 78.5178957176027 | 311.034610000017 | Micronesia (Federated States of) |
| 1995 | 361.36808217259 | 0.491015185412641 | 75.1474976162377 | 286.220584556352 | Micronesia (Federated States of) |
| 1996 | 342.05715704863 | 0.495570308271861 | 70.9957383895847 | 271.061418659045 | Micronesia (Federated States of) |
| 1997 | 312.51990025547 | 0.499110105936474 | 17.1476145473196 | 295.37228570815 | Micronesia (Federated States of) |
| 1998 | 293.35140344013 | 0.5028615644834 | 16.2421230978122 | 277.109280342318 | Micronesia (Federated States of) |
| 1999 | 274.74831971096 | 0.506663430751627 | 15.8998897908579 | 258.848429920102 | Micronesia (Federated States of) |
| 2000 | 1607.59308117207 | 0.511004227582949 | 15.6703454102682 | 1591.9227357618 | Micronesia (Federated States of) |
| 2001 | 236.81985103343 | 0.515316814346477 | 15.2711232404512 | 221.548727792979 | Micronesia (Federated States of) |
| 2002 | 220.86632628361 | 0.519545671945836 | 15.3157994673413 | 205.550526816269 | Micronesia (Federated States of) |
| 2003 | 206.68383638008 | 0.523829598854531 | 15.2060922729252 | 191.477744107155 | Micronesia (Federated States of) |
| 2004 | 200.36849101446 | 0.527610964634803 | 15.0991135697128 | 185.269377444747 | Micronesia (Federated States of) |
| 2005 | 198.45839385906 | 0.531703321036516 | 15.0446760521527 | 183.413717806907 | Micronesia (Federated States of) |
| 2006 | 188.26785675166 | 0.535718681873052 | 15.0582170716233 | 173.209639680037 | Micronesia (Federated States of) |
| 2007 | 178.29312316756 | 0.53943146430145 | 15.1585906638804 | 163.13453250368 | Micronesia (Federated States of) |
| 2008 | 158.43507980322 | 0.542766353836032 | 15.0334604718768 | 143.401619331343 | Micronesia (Federated States of) |
| 2009 | 150.80962465895 | 0.546275496220168 | 14.5070417802821 | 136.302582878668 | Micronesia (Federated States of) |
| 2010 | 143.61179921863 | 0.550122546321787 | 13.8608577332994 | 129.750941485331 | Micronesia (Federated States of) |
| 2011 | 150.14796337575 | 0.554240410692063 | 13.2149512911436 | 136.933012084606 | Micronesia (Federated States of) |
| 2012 | 149.18879734806 | 0.557965852891456 | 11.9286534470292 | 137.260143901031 | Micronesia (Federated States of) |
| 2013 | 148.47437435416 | 0.561082820885874 | 11.298544554355 | 137.175829799805 | Micronesia (Federated States of) |
| 2014 | 147.5977878352 | 0.563840029745074 | 10.8146933628482 | 136.783094472352 | Micronesia (Federated States of) |
| 2015 | 146.90297487925 | 0.567151341569495 | 9.88986337196308 | 137.013111507287 | Micronesia (Federated States of) |
| 2016 | 145.91539634535 | 0.570458101091096 | 8.88114792650921 | 137.034248418841 | Micronesia (Federated States of) |
| 2017 | 139.91986459917 | 0.574039832793086 | 8.6528857424346 | 131.266978856735 | Micronesia (Federated States of) |
| 2018 | 132.83824523447 | 0.577555080818811 | 8.92776520339468 | 123.910480031075 | Micronesia (Federated States of) |
| 2019 | 124.98832524287 | 0.581195254501754 | 8.94782993698589 | 116.040495305884 | Micronesia (Federated States of) |
| 2020 | 113.2245787771 | 0.584527228769055 | 8.9034940029508 | 104.321084774149 | Micronesia (Federated States of) |
| 2021 | 107.72703444498 | 0.587534967103163 | 8.78107958381945 | 98.9459548611606 | Micronesia (Federated States of) |
| 1990 | 2836.04060081697 | 0.310668628637835 | 863.991283481025 | 1972.04931733594 | Papua New Guinea |
| 1991 | 2748.16362643034 | 0.314967305387282 | 775.070242918271 | 1973.09338351207 | Papua New Guinea |
| 1992 | 2640.5036627548 | 0.319530259271955 | 776.540851648822 | 1863.96281110598 | Papua New Guinea |
| 1993 | 2502.08006675397 | 0.32500598378048 | 724.824937468801 | 1777.25512928517 | Papua New Guinea |
| 1994 | 2346.02255757527 | 0.330664467960637 | 670.903319898032 | 1675.11923767724 | Papua New Guinea |
| 1995 | 2206.18627871329 | 0.335421285048649 | 650.894672220224 | 1555.29160649307 | Papua New Guinea |
| 1996 | 2111.72646990755 | 0.340348698133771 | 581.076655159915 | 1530.64981474763 | Papua New Guinea |
| 1997 | 2015.6500517708 | 0.344258324561358 | 569.186340447444 | 1446.46371132336 | Papua New Guinea |
| 1998 | 2035.04019930111 | 0.347801407739934 | 569.801108695418 | 1465.23909060569 | Papua New Guinea |
| 1999 | 2002.67188119778 | 0.351250620970096 | 553.459358930766 | 1449.21252226701 | Papua New Guinea |
| 2000 | 1973.13163792 | 0.354135790847746 | 554.637157569203 | 1418.4944803508 | Papua New Guinea |
| 2001 | 1905.77341123214 | 0.356588986333728 | 555.288420580194 | 1350.48499065195 | Papua New Guinea |
| 2002 | 1850.26902120028 | 0.358572086402703 | 497.836471700327 | 1352.43254949995 | Papua New Guinea |
| 2003 | 1754.29252816372 | 0.360736816415114 | 484.980040294038 | 1269.31248786968 | Papua New Guinea |
| 2004 | 1726.99682566737 | 0.36282754151245 | 465.8218098885 | 1261.17501577887 | Papua New Guinea |
| 2005 | 1770.54770583377 | 0.365294964904992 | 420.29280990615 | 1350.25489592762 | Papua New Guinea |
| 2006 | 1786.24743248451 | 0.367826759122837 | 418.745852814053 | 1367.50157967046 | Papua New Guinea |
| 2007 | 1790.32950670671 | 0.370794146921454 | 401.918892778942 | 1388.41061392777 | Papua New Guinea |
| 2008 | 1771.7745933063 | 0.37342620922524 | 353.000852252405 | 1418.7737410539 | Papua New Guinea |
| 2009 | 1850.8418045633 | 0.376528284761462 | 351.238491312223 | 1499.60331325108 | Papua New Guinea |
| 2010 | 1828.06614798249 | 0.380286777637627 | 329.90516481152 | 1498.16098317097 | Papua New Guinea |
| 2011 | 1707.700496285 | 0.383587539302927 | 313.202174927552 | 1394.49832135745 | Papua New Guinea |
| 2012 | 1671.75408950933 | 0.386781027656579 | 317.876123233902 | 1353.87796627543 | Papua New Guinea |
| 2013 | 1623.98274085156 | 0.389926437008588 | 290.724517490979 | 1333.25822336058 | Papua New Guinea |
| 2014 | 1565.93755816047 | 0.394004571266826 | 281.387010663223 | 1284.55054749725 | Papua New Guinea |
| 2015 | 1488.14602253656 | 0.398285863731033 | 259.535803031713 | 1228.61021950485 | Papua New Guinea |
| 2016 | 1456.2095921346 | 0.402407266844816 | 239.662339945711 | 1216.54725218889 | Papua New Guinea |
| 2017 | 1412.78185824132 | 0.406153435448224 | 244.356433176486 | 1168.42542506483 | Papua New Guinea |
| 2018 | 1348.62825204549 | 0.409165082697567 | 221.688636108609 | 1126.93961593688 | Papua New Guinea |
| 2019 | 1294.68126058128 | 0.412311146526574 | 213.689149254245 | 1080.99211132704 | Papua New Guinea |
| 2020 | 1265.22778728729 | 0.415186878578667 | 215.352998793869 | 1049.87478849342 | Papua New Guinea |
| 2021 | 1206.04635112242 | 0.417797443084353 | 197.543948212145 | 1008.50240291027 | Papua New Guinea |
| 1990 | 103.74730973335 | 0.48749142771166 | 75.2482298177951 | 28.4990799155548 | Samoa |
| 1991 | 98.61408900693 | 0.490987145586308 | 76.2735477221591 | 22.3405412847709 | Samoa |
| 1992 | 93.34566500805 | 0.494027447933208 | 70.6992919774001 | 22.6463730306499 | Samoa |
| 1993 | 88.49131163052 | 0.497079263536296 | 30.8529273713663 | 57.6383842591537 | Samoa |
| 1994 | 84.04736358667 | 0.499484836329879 | 17.2822895997035 | 66.7650739869666 | Samoa |
| 1995 | 79.76078405836 | 0.502369734936465 | 16.6016949339566 | 63.1590891244034 | Samoa |
| 1996 | 75.99294528422 | 0.505778026475631 | 16.0852057781489 | 59.9077395060711 | Samoa |
| 1997 | 72.81847107528 | 0.509096382265271 | 15.9677177254006 | 56.8507533498794 | Samoa |
| 1998 | 68.57493403541 | 0.512367565788016 | 15.6849028164477 | 52.8900312189622 | Samoa |
| 1999 | 69.91243168225 | 0.515409049865666 | 15.3353119824422 | 54.5771196998079 | Samoa |
| 2000 | 66.38641453856 | 0.518902326533937 | 15.3027870462028 | 51.0836274923572 | Samoa |
| 2001 | 63.16948812163 | 0.522971625527944 | 15.1162861201506 | 48.0532020014794 | Samoa |
| 2002 | 59.27025666493 | 0.527242669970423 | 15.1561206672008 | 44.1141359977292 | Samoa |
| 2003 | 56.34098772413 | 0.531603736377418 | 15.071458386995 | 41.269529337135 | Samoa |
| 2004 | 54.34464888403 | 0.53600327592574 | 15.0869366812356 | 39.2577122027944 | Samoa |
| 2005 | 53.20894898374 | 0.540557907991258 | 15.0793745191711 | 38.1295744645689 | Samoa |
| 2006 | 51.40163055949 | 0.544812068358444 | 15.0266045649921 | 36.3750259944979 | Samoa |
| 2007 | 49.86116067227 | 0.54911075247208 | 14.4983891071976 | 35.3627715650724 | Samoa |
| 2008 | 48.69188612362 | 0.553164595646872 | 13.7377140860577 | 34.9541720375623 | Samoa |
| 2009 | 48.55117249532 | 0.556474015274728 | 12.9122466638179 | 35.6389258315021 | Samoa |
| 2010 | 49.42390684704 | 0.559622905103398 | 12.1969629539792 | 37.2269438930608 | Samoa |
| 2011 | 49.3411200852 | 0.562891574259145 | 11.3979595489371 | 37.9431605362629 | Samoa |
| 2012 | 48.76074753695 | 0.565320388101849 | 10.337585579423 | 38.423161957527 | Samoa |
| 2013 | 47.39318598137 | 0.56744853592103 | 9.92358479131454 | 37.4696011900555 | Samoa |
| 2014 | 46.04895805023 | 0.569493660728475 | 9.547012900371 | 36.501945149859 | Samoa |
| 2015 | 44.658854274 | 0.571998982964235 | 8.93846566200016 | 35.7203886119998 | Samoa |
| 2016 | 43.78381230983 | 0.575402051396993 | 8.90105813485391 | 34.8827541749761 | Samoa |
| 2017 | 43.55877758159 | 0.578925435094317 | 8.74066424418016 | 34.8181133374098 | Samoa |
| 2018 | 44.05379347561 | 0.582320660518804 | 8.82405976274025 | 35.2297337128698 | Samoa |
| 2019 | 43.08373719536 | 0.586256695142635 | 8.92520282323662 | 34.1585343721234 | Samoa |
| 2020 | 43.54271019178 | 0.590075295612806 | 9.07286599306357 | 34.4698441987164 | Samoa |
| 2021 | 40.31553796256 | 0.593392769469481 | 8.73632072481519 | 31.5792172377448 | Samoa |
| 1990 | 1000.3565776548 | 0.301217167448465 | 959.716178172139 | 40.6403994826612 | Solomon Islands |
| 1991 | 958.98980234155 | 0.305830667512655 | 952.316014717617 | 6.67378762393253 | Solomon Islands |
| 1992 | 902.50792224267 | 0.311431696691098 | 858.464444959724 | 44.0434772829459 | Solomon Islands |
| 1993 | 847.92348931172 | 0.31715109185957 | 776.898711476928 | 71.0247778347924 | Solomon Islands |
| 1994 | 793.29003747773 | 0.323300246722871 | 718.9251623191 | 74.36487515863 | Solomon Islands |
| 1995 | 732.88381535 | 0.329968564261603 | 672.414582679276 | 60.4692326707238 | Solomon Islands |
| 1996 | 685.68759884024 | 0.336208053088528 | 645.031970364749 | 40.655628475491 | Solomon Islands |
| 1997 | 640.33592303077 | 0.341835626898821 | 569.096145892219 | 71.2397771385506 | Solomon Islands |
| 1998 | 677.6039919924 | 0.347135060018002 | 569.36436411404 | 108.23962787836 | Solomon Islands |
| 1999 | 666.86973711162 | 0.351867191410364 | 554.004162860785 | 112.865574250835 | Solomon Islands |
| 2000 | 642.67295329241 | 0.354799143417026 | 555.356521178465 | 87.3164321139454 | Solomon Islands |
| 2001 | 613.52637130969 | 0.356626745168074 | 553.479950028802 | 60.0464212808876 | Solomon Islands |
| 2002 | 583.60438242962 | 0.357910115982585 | 502.767925719312 | 80.8364567103079 | Solomon Islands |
| 2003 | 530.96574383044 | 0.359424358912125 | 482.300755887254 | 48.6649879431858 | Solomon Islands |
| 2004 | 473.74516079472 | 0.361290558162268 | 462.910166763827 | 10.8349940308926 | Solomon Islands |
| 2005 | 455.52912700918 | 0.363580018964866 | 453.008412243446 | 2.52071476573411 | Solomon Islands |
| 2006 | 435.46219870159 | 0.366443697706899 | 410.609915204331 | 24.8522834972591 | Solomon Islands |
| 2007 | 442.44530889766 | 0.369827951415349 | 401.026555232589 | 41.4187536650711 | Solomon Islands |
| 2008 | 420.38219338838 | 0.3737648835675 | 354.286528244433 | 66.0956651439473 | Solomon Islands |
| 2009 | 399.71803621495 | 0.377821027515966 | 341.525695820933 | 58.1923403940167 | Solomon Islands |
| 2010 | 388.78078507243 | 0.382717589747291 | 324.147607850243 | 64.6331772221868 | Solomon Islands |
| 2011 | 363.64836102345 | 0.388055336574909 | 289.674588704382 | 73.973772319068 | Solomon Islands |
| 2012 | 333.41218960956 | 0.393004639707306 | 282.491449881276 | 50.9207397282845 | Solomon Islands |
| 2013 | 325.57691852327 | 0.397906249572343 | 261.356937507177 | 64.2199810160926 | Solomon Islands |
| 2014 | 308.74640125216 | 0.402244744701609 | 233.45920503583 | 75.28719621633 | Solomon Islands |
| 2015 | 319.50361118469 | 0.406233896996201 | 243.960855306789 | 75.5427558779007 | Solomon Islands |
| 2016 | 305.14813298003 | 0.410406962023453 | 217.310578184974 | 87.8375547950557 | Solomon Islands |
| 2017 | 290.61515761641 | 0.414676394150034 | 215.875935678361 | 74.7392219380488 | Solomon Islands |
| 2018 | 274.20668945216 | 0.418889220879761 | 195.41058332148 | 78.7961061306802 | Solomon Islands |
| 2019 | 257.4779512116 | 0.422736386173248 | 182.789172861668 | 74.6887783499318 | Solomon Islands |
| 2020 | 211.3915425576 | 0.426112428669488 | 177.589156889571 | 33.8023856680287 | Solomon Islands |
| 2021 | 202.805235842 | 0.429360316406646 | 140.327960848483 | 62.4772749935168 | Solomon Islands |
| 1990 | 136.57852433731 | 0.491806839500397 | 74.6965415320248 | 61.8819828052852 | Tonga |
| 1991 | 129.70434208258 | 0.498166205111113 | 19.1523830926207 | 110.551958989959 | Tonga |
| 1992 | 122.67632378583 | 0.503659848303175 | 16.1736408915628 | 106.502682894267 | Tonga |
| 1993 | 117.66449613324 | 0.508904449646887 | 16.1019188552212 | 101.562577278019 | Tonga |
| 1994 | 112.15512844193 | 0.514207834722848 | 15.6360917247675 | 96.5190367171625 | Tonga |
| 1995 | 106.76659462532 | 0.520215020279115 | 15.1375086836439 | 91.6290859416761 | Tonga |
| 1996 | 102.37307038345 | 0.525808135436488 | 15.0803253267417 | 87.2927450567083 | Tonga |
| 1997 | 95.84553791279 | 0.531009091678332 | 15.0708793415968 | 80.7746585711932 | Tonga |
| 1998 | 92.10318698184 | 0.536172313703023 | 15.1157028929807 | 76.9874840888593 | Tonga |
| 1999 | 96.16205933259 | 0.541317019874755 | 15.0269299059403 | 81.1351294266497 | Tonga |
| 2000 | 92.43177561457 | 0.546303195085387 | 14.4721714034479 | 77.9596042111221 | Tonga |
| 2001 | 88.10206120964 | 0.551054251063729 | 13.8310414560003 | 74.2710197536397 | Tonga |
| 2002 | 83.31244826663 | 0.556008345246101 | 13.4002055948879 | 69.9122426717421 | Tonga |
| 2003 | 78.95356407499 | 0.560736066200254 | 11.9538357808416 | 66.9997282941484 | Tonga |
| 2004 | 73.60621309796 | 0.564806378895597 | 10.8121976809464 | 62.7940154170136 | Tonga |
| 2005 | 72.96915393057 | 0.568449169182724 | 9.49284777851424 | 63.4763061520558 | Tonga |
| 2006 | 73.23816317062 | 0.57161986831651 | 8.7716533525453 | 64.4665098180747 | Tonga |
| 2007 | 72.00730187706 | 0.574351510103056 | 8.74071243747554 | 63.2665894395845 | Tonga |
| 2008 | 69.58785900051 | 0.57730541521781 | 8.74338664712966 | 60.8444723533803 | Tonga |
| 2009 | 69.33961154654 | 0.579170749598751 | 8.84579606152136 | 60.4938154850186 | Tonga |
| 2010 | 69.18755284153 | 0.580657298614066 | 9.0281310598452 | 60.1594217816848 | Tonga |
| 2011 | 67.95702425277 | 0.58265672287008 | 8.84470772643232 | 59.1123165263377 | Tonga |
| 2012 | 66.32880382662 | 0.584770880221314 | 8.89843292770174 | 57.4303708989183 | Tonga |
| 2013 | 65.0578676073 | 0.587351141040311 | 8.78835290421109 | 56.2695147030889 | Tonga |
| 2014 | 63.43101182331 | 0.590826852746506 | 8.84725551665443 | 54.5837563066556 | Tonga |
| 2015 | 62.32980360893 | 0.594965747674029 | 8.91984473063963 | 53.4099588782904 | Tonga |
| 2016 | 61.12315465325 | 0.60034366853075 | 8.81212298494039 | 52.3110316683096 | Tonga |
| 2017 | 58.15239600305 | 0.606243591849879 | 8.90634988042649 | 49.2460461226235 | Tonga |
| 2018 | 55.89852805755 | 0.611953941537156 | 8.91101008388213 | 46.9875179736679 | Tonga |
| 2019 | 53.43569630952 | 0.617324063407956 | 9.08949813707642 | 44.3461981724436 | Tonga |
| 2020 | 53.1433457615 | 0.622104455604093 | 8.91100605805913 | 44.2323397034409 | Tonga |
| 2021 | 45.26625376427 | 0.626349935807987 | 8.72559404073154 | 36.5406597235385 | Tonga |
| 1990 | 831.1758788153 | 0.353100252005321 | 552.735220614671 | 278.44065820063 | Vanuatu |
| 1991 | 794.09073810788 | 0.357005378847917 | 511.736757237558 | 282.353980870322 | Vanuatu |
| 1992 | 751.94366523923 | 0.360893295561675 | 479.40898062209 | 272.53468461714 | Vanuatu |
| 1993 | 722.49728276695 | 0.364444155251801 | 462.796843094371 | 259.700439672579 | Vanuatu |
| 1994 | 688.2065380319 | 0.368501002014456 | 414.851877155575 | 273.354660876325 | Vanuatu |
| 1995 | 657.38310694191 | 0.372479125756324 | 355.37700913142 | 302.00609781049 | Vanuatu |
| 1996 | 634.02037614899 | 0.376460884544884 | 354.079664509896 | 279.940711639094 | Vanuatu |
| 1997 | 604.33348550971 | 0.380667171234513 | 327.776959331283 | 276.556526178427 | Vanuatu |
| 1998 | 575.9418947113 | 0.384690196444633 | 321.626877754413 | 254.315016956887 | Vanuatu |
| 1999 | 626.35535806607 | 0.388300526237242 | 286.504520986845 | 339.850837079225 | Vanuatu |
| 2000 | 610.80253376977 | 0.392158348897928 | 285.413495192239 | 325.389038577531 | Vanuatu |
| 2001 | 588.85746682016 | 0.395325055353778 | 265.048811595784 | 323.808655224376 | Vanuatu |
| 2002 | 557.87928690016 | 0.397723782197823 | 264.834649335418 | 293.044637564742 | Vanuatu |
| 2003 | 522.13929530616 | 0.400396455507663 | 260.373908941499 | 261.765386364661 | Vanuatu |
| 2004 | 464.33470168087 | 0.403226096857709 | 234.536968888849 | 229.797732792021 | Vanuatu |
| 2005 | 444.1134318088 | 0.406488572045146 | 239.936757379881 | 204.176674428919 | Vanuatu |
| 2006 | 416.9366371395 | 0.410501728664985 | 216.750733776146 | 200.185903363354 | Vanuatu |
| 2007 | 432.90603466937 | 0.41491557794022 | 216.585219680351 | 216.320814989019 | Vanuatu |
| 2008 | 416.64600584184 | 0.41985323747475 | 193.303565646686 | 223.342440195154 | Vanuatu |
| 2009 | 395.64931005172 | 0.424854650160333 | 178.059075050649 | 217.590235001071 | Vanuatu |
| 2010 | 376.0221466373 | 0.429658048936263 | 138.31925181047 | 237.70289482683 | Vanuatu |
| 2011 | 352.56200557755 | 0.434283044387506 | 132.706221045985 | 219.855784531565 | Vanuatu |
| 2012 | 315.3005782642 | 0.438661997905717 | 128.080401086304 | 187.220177177896 | Vanuatu |
| 2013 | 294.06871970563 | 0.442720176072726 | 114.484543744562 | 179.584175961068 | Vanuatu |
| 2014 | 290.32043466977 | 0.446687298111776 | 113.782981899556 | 176.537452770214 | Vanuatu |
| 2015 | 281.04317829127 | 0.450329861394857 | 107.64999545377 | 173.3931828375 | Vanuatu |
| 2016 | 303.12108002321 | 0.454072763242304 | 105.871495681719 | 197.249584341491 | Vanuatu |
| 2017 | 296.93505747082 | 0.45801683397471 | 95.0815379792593 | 201.853519491561 | Vanuatu |
| 2018 | 290.14236868671 | 0.462080220969235 | 91.4722559053567 | 198.670112781353 | Vanuatu |
| 2019 | 276.26885068073 | 0.466276161662358 | 84.7251682092901 | 191.54368247144 | Vanuatu |
| 2020 | 263.28409164602 | 0.470038390856612 | 82.8801349515896 | 180.40395669443 | Vanuatu |
| 2021 | 232.74632856789 | 0.473100705820327 | 79.9779514194118 | 152.768377148478 | Vanuatu |
| 1990 | 665.57665053874 | 0.54441453483767 | 15.0159940919691 | 650.560656446771 | Armenia |
| 1991 | 653.1982717688 | 0.547561862706728 | 14.5178974995337 | 638.680374269266 | Armenia |
| 1992 | 680.68142086171 | 0.548974723873954 | 14.4586581739682 | 666.222762687742 | Armenia |
| 1993 | 641.11359350175 | 0.551214673543035 | 13.9039210077938 | 627.209672493956 | Armenia |
| 1994 | 618.4461988202 | 0.553783844340157 | 12.9070343960716 | 605.539164424128 | Armenia |
| 1995 | 560.55624478799 | 0.556815513215437 | 12.9270084199391 | 547.629236368051 | Armenia |
| 1996 | 485.084335987 | 0.560537777557715 | 12.0488639365318 | 473.035472050468 | Armenia |
| 1997 | 445.02087816199 | 0.564627461798536 | 10.5768141092092 | 434.444064052781 | Armenia |
| 1998 | 378.65990689211 | 0.569556662578666 | 9.51335509075679 | 369.146551801353 | Armenia |
| 1999 | 322.54683301245 | 0.574323064436665 | 8.85503913150594 | 313.691793880944 | Armenia |
| 2000 | 277.38689988625 | 0.579437569012042 | 8.88352295717185 | 268.503376929078 | Armenia |
| 2001 | 246.97723692575 | 0.585419295040696 | 8.73812991744937 | 238.239107008301 | Armenia |
| 2002 | 202.75187683764 | 0.592710614375924 | 8.73694212959468 | 194.014934708045 | Armenia |
| 2003 | 176.18678171888 | 0.600809170405726 | 8.94256538230944 | 167.244216336571 | Armenia |
| 2004 | 162.89429307109 | 0.60903176604033 | 8.9290276381176 | 153.965265432972 | Armenia |
| 2005 | 147.63199401779 | 0.617759634102041 | 8.95404922709129 | 138.677944790699 | Armenia |
| 2006 | 132.83047217503 | 0.626688335239636 | 8.84464542683372 | 123.985826748196 | Armenia |
| 2007 | 96.0606635265 | 0.635870981516541 | 6.95429577221343 | 89.1063677542866 | Armenia |
| 2008 | 79.28417583963 | 0.64443159113194 | 6.15025356314213 | 73.1339222764879 | Armenia |
| 2009 | 60.8681680181 | 0.649714844735141 | 5.35121356095156 | 55.5169544571484 | Armenia |
| 2010 | 44.43514820347 | 0.654811533750253 | 5.1200747388059 | 39.3150734646641 | Armenia |
| 2011 | 38.93041179967 | 0.659818092317619 | 4.38417136560467 | 34.5462404340653 | Armenia |
| 2012 | 25.42029290222 | 0.664833142570704 | 4.19229050553902 | 21.228002396681 | Armenia |
| 2013 | 21.01379354369 | 0.669534495666631 | 3.82461296530644 | 17.1891805783836 | Armenia |
| 2014 | 19.13489261715 | 0.673932189530685 | 3.8071758461544 | 15.3277167709956 | Armenia |
| 2015 | 17.41912397237 | 0.678054634729575 | 3.5977671664528 | 13.8213568059172 | Armenia |
| 2016 | 20.05077850487 | 0.681746395416543 | 3.57007796972871 | 16.4807005351413 | Armenia |
| 2017 | 16.45776722982 | 0.685980265385199 | 3.37666860699185 | 13.0810986228282 | Armenia |
| 2018 | 17.00274205654 | 0.690276871944721 | 3.3789851306851 | 13.6237569258549 | Armenia |
| 2019 | 16.90360566483 | 0.694765961567774 | 3.15720715323194 | 13.7463985115981 | Armenia |
| 2020 | 15.77953064157 | 0.69826889937921 | 3.20474231141382 | 12.5747883301562 | Armenia |
| 2021 | 14.7768259782 | 0.701833194147139 | 2.96256160680388 | 11.8142643713961 | Armenia |
| 1990 | 1398.33363603641 | 0.595986032704332 | 9.06737557669529 | 1389.26626045971 | Azerbaijan |
| 1991 | 1325.25688236532 | 0.595807711845649 | 8.8788520254617 | 1316.37803033986 | Azerbaijan |
| 1992 | 1262.11493654767 | 0.594651335166956 | 8.78787814481 | 1253.32705840286 | Azerbaijan |
| 1993 | 1209.10412328017 | 0.59354267649819 | 8.78845132936916 | 1200.3156719508 | Azerbaijan |
| 1994 | 1154.43387457468 | 0.5920887850585 | 8.96753491369773 | 1145.46633966098 | Azerbaijan |
| 1995 | 1097.04618985682 | 0.589441456728463 | 8.91411001914 | 1088.13207983768 | Azerbaijan |
| 1996 | 1042.74781178313 | 0.586315946466504 | 8.70576450004559 | 1034.04204728308 | Azerbaijan |
| 1997 | 986.75345591729 | 0.583221812602983 | 8.82120906138522 | 977.932246855905 | Azerbaijan |
| 1998 | 938.01285916517 | 0.580392893004131 | 8.87057305122437 | 929.142286113946 | Azerbaijan |
| 1999 | 887.03927303537 | 0.579192506195412 | 8.8742323202242 | 878.165040715146 | Azerbaijan |
| 2000 | 819.688601247 | 0.580020573148448 | 8.91426479664291 | 810.774336450357 | Azerbaijan |
| 2001 | 727.0732692453 | 0.582193708342128 | 8.91880303506233 | 718.154466210238 | Azerbaijan |
| 2002 | 657.77917052058 | 0.585716143276774 | 9.04763231371111 | 648.731538206869 | Azerbaijan |
| 2003 | 606.97789182959 | 0.590038705393348 | 8.7454223373976 | 598.232469492192 | Azerbaijan |
| 2004 | 563.1836784711 | 0.594646084750935 | 8.74409149521275 | 554.439586975887 | Azerbaijan |
| 2005 | 514.70613775795 | 0.602485193033144 | 8.943465840726 | 505.762671917224 | Azerbaijan |
| 2006 | 466.41577887684 | 0.61377109654975 | 8.88778412904419 | 457.527994747796 | Azerbaijan |
| 2007 | 419.90107125905 | 0.626110925531736 | 8.98054984418573 | 410.920521414864 | Azerbaijan |
| 2008 | 385.7521619606 | 0.636901109120982 | 6.8269975899235 | 378.925164370676 | Azerbaijan |
| 2009 | 356.99595551152 | 0.645163340636595 | 6.17072444325274 | 350.825231068267 | Azerbaijan |
| 2010 | 335.40581116106 | 0.652056507002412 | 5.18171443039722 | 330.224096730663 | Azerbaijan |
| 2011 | 315.29647740958 | 0.657971629199692 | 4.57869643708895 | 310.717780972491 | Azerbaijan |
| 2012 | 297.13638393442 | 0.662897001762113 | 4.29890279680238 | 292.837481137618 | Azerbaijan |
| 2013 | 280.43854277629 | 0.667658779253801 | 3.88124735180637 | 276.557295424484 | Azerbaijan |
| 2014 | 263.74010659498 | 0.672162741185153 | 3.8953694338509 | 259.844737161129 | Azerbaijan |
| 2015 | 251.37333046062 | 0.676244911134277 | 3.61600595787566 | 247.757324502744 | Azerbaijan |
| 2016 | 242.56475010399 | 0.679807642651878 | 3.61291073608054 | 238.951839367909 | Azerbaijan |
| 2017 | 235.75813541511 | 0.683263203610851 | 3.32102388372076 | 232.437111531389 | Azerbaijan |
| 2018 | 229.32338237597 | 0.68656109318329 | 3.38937222318681 | 225.934010152783 | Azerbaijan |
| 2019 | 223.34536138824 | 0.689834714583805 | 3.39844038890234 | 219.946920999338 | Azerbaijan |
| 2020 | 202.51696804991 | 0.692605192199032 | 3.18245575998303 | 199.334512289927 | Azerbaijan |
| 2021 | 198.55727855007 | 0.694851274415291 | 3.14098406042355 | 195.416294489646 | Azerbaijan |
| 1990 | 360.25338061836 | 0.656136044291497 | 4.8441676530049 | 355.409212965355 | Georgia |
| 1991 | 303.53486297612 | 0.662113185865682 | 4.44594789638075 | 299.088915079739 | Georgia |
| 1992 | 302.44275188337 | 0.665021427814483 | 4.27006761261034 | 298.17268427076 | Georgia |
| 1993 | 351.81674939337 | 0.663466862184598 | 4.19568297701612 | 347.621066416354 | Georgia |
| 1994 | 405.32706744229 | 0.658134139130798 | 4.36032959534461 | 400.966737846945 | Georgia |
| 1995 | 370.73362292638 | 0.651363604039082 | 5.12234778239189 | 365.611275143988 | Georgia |
| 1996 | 321.92016158652 | 0.644558090070583 | 5.99604738485731 | 315.924114201663 | Georgia |
| 1997 | 314.07139985244 | 0.63852563128688 | 6.72603734297955 | 307.34536250946 | Georgia |
| 1998 | 256.12537431348 | 0.633347931127712 | 6.9399606911258 | 249.185413622354 | Georgia |
| 1999 | 203.43740736941 | 0.63027794625322 | 8.37688789500231 | 195.060519474408 | Georgia |
| 2000 | 146.19490939062 | 0.630265990578475 | 8.3282801541796 | 137.86662923644 | Georgia |
| 2001 | 131.41227998779 | 0.633388300444106 | 6.95253030744132 | 124.459749680349 | Georgia |
| 2002 | 115.51647087668 | 0.638370108205142 | 6.75576018681627 | 108.760710689864 | Georgia |
| 2003 | 96.23361072952 | 0.644065578985868 | 6.07863444652059 | 90.1549762829994 | Georgia |
| 2004 | 84.04605978391 | 0.648920978029192 | 6.33959157127808 | 77.7064682126319 | Georgia |
| 2005 | 61.26048878668 | 0.65361600499668 | 4.95357803418333 | 56.3069107524967 | Georgia |
| 2006 | 46.093061406 | 0.658403371769116 | 4.80124986911781 | 41.2918115368822 | Georgia |
| 2007 | 43.82299370767 | 0.663697966220568 | 4.19250964373817 | 39.6304840639318 | Georgia |
| 2008 | 41.57749431092 | 0.668442495947816 | 3.86198039068094 | 37.7155139202391 | Georgia |
| 2009 | 37.78909978629 | 0.672449380923053 | 3.79766621038643 | 33.9914335759036 | Georgia |
| 2010 | 34.07550879606 | 0.677034892187658 | 3.63047429477964 | 30.4450345012804 | Georgia |
| 2011 | 29.19567410187 | 0.681567044251293 | 3.61483509738092 | 25.5808390044891 | Georgia |
| 2012 | 26.7246170926 | 0.685519223189507 | 3.38596893192584 | 23.3386481606742 | Georgia |
| 2013 | 24.98441561928 | 0.688603268140652 | 3.35376391371048 | 21.6306517055695 | Georgia |
| 2014 | 20.3732592228 | 0.692155206871812 | 3.22105095112 | 17.15220827168 | Georgia |
| 2015 | 18.31207306468 | 0.697316132597582 | 3.08105629627795 | 15.2310167684021 | Georgia |
| 2016 | 18.4610254888 | 0.703731490142933 | 2.83815718032808 | 15.6228683084719 | Georgia |
| 2017 | 13.63533129045 | 0.710874718456081 | 2.52298790264081 | 11.1123433878092 | Georgia |
| 2018 | 12.61082286593 | 0.717993653891294 | 2.58428791032226 | 10.0265349556077 | Georgia |
| 2019 | 10.25878626727 | 0.724536461948472 | 2.3045411288398 | 7.9542451384302 | Georgia |
| 2020 | 9.52247618626 | 0.729125888954526 | 2.22353779801286 | 7.29893838824714 | Georgia |
| 2021 | 9.15444268585 | 0.732473604476306 | 2.38043547934211 | 6.7740072065079 | Georgia |
| 1990 | 735.10277262742 | 0.589435803839118 | 8.7138606536787 | 726.388911973741 | Kazakhstan |
| 1991 | 713.55575125898 | 0.590967831817803 | 8.90845408861049 | 704.64729717037 | Kazakhstan |
| 1992 | 796.25190648438 | 0.596127664006257 | 8.76214209966148 | 787.489764384719 | Kazakhstan |
| 1993 | 793.73155931474 | 0.603138328452166 | 8.94580068160831 | 784.785758633132 | Kazakhstan |
| 1994 | 802.11385554046 | 0.610591100120018 | 8.80580455797736 | 793.308050982483 | Kazakhstan |
| 1995 | 772.87536880227 | 0.618573582182698 | 8.96868624865638 | 763.906682553614 | Kazakhstan |
| 1996 | 696.318943094 | 0.626013137564744 | 8.81293828473 | 687.50600480927 | Kazakhstan |
| 1997 | 705.15505217254 | 0.631166626279517 | 8.14199112766484 | 697.013061044875 | Kazakhstan |
| 1998 | 536.06819857859 | 0.635388623908262 | 7.09068999735244 | 528.977508581238 | Kazakhstan |
| 1999 | 446.91422482021 | 0.64018048502742 | 6.63182499483347 | 440.282399825377 | Kazakhstan |
| 2000 | 368.76545800174 | 0.644689623243828 | 5.93505632575813 | 362.830401675982 | Kazakhstan |
| 2001 | 294.55920090406 | 0.648397258264821 | 5.94458944851354 | 288.614611455546 | Kazakhstan |
| 2002 | 226.61426983735 | 0.651529335252827 | 4.95988917923508 | 221.654380658115 | Kazakhstan |
| 2003 | 180.16980859918 | 0.654835925835483 | 4.93020647587143 | 175.239602123309 | Kazakhstan |
| 2004 | 162.1850334958 | 0.658733071329599 | 4.35169842640379 | 157.833335069396 | Kazakhstan |
| 2005 | 135.01996340998 | 0.662943941893493 | 4.58651807523957 | 130.43344533474 | Kazakhstan |
| 2006 | 134.3013625967 | 0.667048858291012 | 3.89828225722753 | 130.403080339472 | Kazakhstan |
| 2007 | 125.36125175579 | 0.671367207568052 | 3.84576596195783 | 121.515485793832 | Kazakhstan |
| 2008 | 126.29275090871 | 0.676352868998898 | 3.60726498486887 | 122.685485923841 | Kazakhstan |
| 2009 | 95.92384243921 | 0.681606838672477 | 3.58901043837182 | 92.3348320008382 | Kazakhstan |
| 2010 | 73.93832842661 | 0.686996643005111 | 3.32932190155194 | 70.6090065250581 | Kazakhstan |
| 2011 | 57.44377101615 | 0.691758951738966 | 3.08807976511966 | 54.3556912510304 | Kazakhstan |
| 2012 | 40.06224342917 | 0.695312766608011 | 3.09674688418951 | 36.9654965449805 | Kazakhstan |
| 2013 | 30.53179000897 | 0.699053593413088 | 2.84122098445973 | 27.6905690245103 | Kazakhstan |
| 2014 | 23.52868713443 | 0.703511920757132 | 2.79383523577937 | 20.7348518986506 | Kazakhstan |
| 2015 | 24.12923331363 | 0.70822973614897 | 2.75933076702703 | 21.369902546603 | Kazakhstan |
| 2016 | 20.49684501014 | 0.712569040397695 | 2.710399534688 | 17.786445475452 | Kazakhstan |
| 2017 | 16.79522744156 | 0.716026687894304 | 2.47120761206327 | 14.3240198294967 | Kazakhstan |
| 2018 | 15.82633828226 | 0.718531998551374 | 2.52526151564961 | 13.3010767666104 | Kazakhstan |
| 2019 | 15.71064590512 | 0.720797601617411 | 2.56841855858629 | 13.1422273465337 | Kazakhstan |
| 2020 | 14.31023630052 | 0.722788768207953 | 2.42377850334625 | 11.8864577971737 | Kazakhstan |
| 2021 | 13.52855368402 | 0.725144495100349 | 2.33506169166176 | 11.1934919923582 | Kazakhstan |
| 1990 | 991.91036800883 | 0.519407652158871 | 15.4165142217296 | 976.4938537871 | Kyrgyzstan |
| 1991 | 958.67602749375 | 0.523746714213702 | 15.138365305302 | 943.537662188448 | Kyrgyzstan |
| 1992 | 983.05801823693 | 0.52790155215442 | 15.0943514189207 | 967.963666818009 | Kyrgyzstan |
| 1993 | 898.26998418438 | 0.532431008334573 | 15.0523964427161 | 883.217587741664 | Kyrgyzstan |
| 1994 | 892.66239425539 | 0.536232980141406 | 15.0751244491316 | 877.587269806258 | Kyrgyzstan |
| 1995 | 863.07646470855 | 0.539052820319823 | 15.1012401261228 | 847.975224582427 | Kyrgyzstan |
| 1996 | 797.8608943086 | 0.541628927165702 | 15.0368476918802 | 782.82404661672 | Kyrgyzstan |
| 1997 | 907.65405331823 | 0.543341150499015 | 15.0300856567981 | 892.623967661432 | Kyrgyzstan |
| 1998 | 906.56321469323 | 0.543258545983634 | 15.0542776433673 | 891.508937049863 | Kyrgyzstan |
| 1999 | 865.88077771695 | 0.54286728228234 | 15.0201229302106 | 850.86065478674 | Kyrgyzstan |
| 2000 | 727.32058132951 | 0.543036486604175 | 15.0195607880236 | 712.301020541486 | Kyrgyzstan |
| 2001 | 638.54730975784 | 0.543805550685403 | 15.0386913862388 | 623.508618371601 | Kyrgyzstan |
| 2002 | 535.09879737308 | 0.544374670344746 | 15.0472537065739 | 520.051543666506 | Kyrgyzstan |
| 2003 | 424.95959690469 | 0.545649261714426 | 14.5474687506636 | 410.412128154026 | Kyrgyzstan |
| 2004 | 382.1210939128 | 0.547262342515335 | 14.5216147141951 | 367.599479198605 | Kyrgyzstan |
| 2005 | 373.04212172265 | 0.548260448903511 | 14.4657312662699 | 358.57639045638 | Kyrgyzstan |
| 2006 | 377.04941616196 | 0.549383593833376 | 14.5346411445772 | 362.514775017383 | Kyrgyzstan |
| 2007 | 359.66328469997 | 0.551146191596044 | 13.9376730140022 | 345.725611685968 | Kyrgyzstan |
| 2008 | 323.03617497248 | 0.553206382142723 | 13.9775055873298 | 309.05866938515 | Kyrgyzstan |
| 2009 | 262.49426742636 | 0.55512386607295 | 12.867727182121 | 249.626540244239 | Kyrgyzstan |
| 2010 | 217.09766909299 | 0.556416544522946 | 13.1758924324979 | 203.921776660492 | Kyrgyzstan |
| 2011 | 204.92025311707 | 0.55892333472436 | 12.1148865003974 | 192.805366616673 | Kyrgyzstan |
| 2012 | 174.62431661365 | 0.561541573671693 | 11.4410786285315 | 163.183237985119 | Kyrgyzstan |
| 2013 | 140.47989504964 | 0.565776769347093 | 10.4424255410982 | 130.037469508542 | Kyrgyzstan |
| 2014 | 133.76246106188 | 0.570569357153487 | 9.19328173490937 | 124.569179326971 | Kyrgyzstan |
| 2015 | 127.03240700766 | 0.575463230818727 | 8.78325125694014 | 118.24915575072 | Kyrgyzstan |
| 2016 | 97.92038070363 | 0.580388550869674 | 8.82463402002728 | 89.0957466836027 | Kyrgyzstan |
| 2017 | 75.46786171015 | 0.585766095993466 | 8.83160866569209 | 66.6362530444579 | Kyrgyzstan |
| 2018 | 67.87668483842 | 0.591237134219733 | 8.94470632301937 | 58.9319785154006 | Kyrgyzstan |
| 2019 | 66.00629796893 | 0.596501638087194 | 8.85298903405753 | 57.1533089348725 | Kyrgyzstan |
| 2020 | 60.76729022305 | 0.600499553804348 | 8.82645021057242 | 51.9408400124776 | Kyrgyzstan |
| 2021 | 59.76083079609 | 0.603979328412055 | 8.67525468120333 | 51.0855761148867 | Kyrgyzstan |
| 1990 | 511.36685537674 | 0.466550117857047 | 85.544456552644 | 425.822398824096 | Mongolia |
| 1991 | 472.10974588024 | 0.472199013666192 | 84.0195589983414 | 388.090186881899 | Mongolia |
| 1992 | 438.99098032677 | 0.477586553799195 | 78.231590750522 | 360.759389576248 | Mongolia |
| 1993 | 412.46601465996 | 0.482726727049412 | 76.6800671674376 | 335.785947492522 | Mongolia |
| 1994 | 387.62425701745 | 0.488276683033118 | 74.9663806161492 | 312.657876401301 | Mongolia |
| 1995 | 361.51195997292 | 0.495006781085721 | 70.8068997840896 | 290.70506018883 | Mongolia |
| 1996 | 359.64984017913 | 0.501703717883367 | 16.604857904694 | 343.044982274436 | Mongolia |
| 1997 | 322.43629208172 | 0.50855826800798 | 15.9588067748713 | 306.477485306849 | Mongolia |
| 1998 | 302.91753277926 | 0.514793382702856 | 15.6612822281117 | 287.256250551148 | Mongolia |
| 1999 | 285.86611217236 | 0.520781189245005 | 15.2012294751762 | 270.664882697184 | Mongolia |
| 2000 | 270.75900221365 | 0.526650344907534 | 15.1418993821762 | 255.617102831474 | Mongolia |
| 2001 | 241.06317745683 | 0.532620200843235 | 15.0828891331046 | 225.980288323725 | Mongolia |
| 2002 | 220.46220092669 | 0.538424834911619 | 15.0772794591651 | 205.384921467525 | Mongolia |
| 2003 | 206.10476888901 | 0.543971650708528 | 15.0208543551586 | 191.083914533851 | Mongolia |
| 2004 | 192.99409867332 | 0.549551499752672 | 13.9562265662631 | 179.037872107057 | Mongolia |
| 2005 | 181.55795281462 | 0.55468028439734 | 12.9007495628542 | 168.657203251766 | Mongolia |
| 2006 | 174.14790773812 | 0.55956113814646 | 11.9745182080256 | 162.173389530094 | Mongolia |
| 2007 | 168.11270028863 | 0.564236795141046 | 10.8292183390527 | 157.283481949577 | Mongolia |
| 2008 | 161.35882030788 | 0.568500598715617 | 9.56967054703396 | 151.789149760846 | Mongolia |
| 2009 | 146.41011017933 | 0.571341685964544 | 8.97619798955986 | 137.43391218977 | Mongolia |
| 2010 | 130.96479807671 | 0.574406669398145 | 8.94991174736173 | 122.014886329348 | Mongolia |
| 2011 | 119.01655822295 | 0.57860066312097 | 8.9885279698826 | 110.028030253067 | Mongolia |
| 2012 | 111.70589040536 | 0.583255885768315 | 8.94788852372324 | 102.758001881637 | Mongolia |
| 2013 | 99.98179535768 | 0.588080810956893 | 8.84101486160188 | 91.1407804960781 | Mongolia |
| 2014 | 88.76109625446 | 0.592867352927119 | 8.88529394221256 | 79.8758023122474 | Mongolia |
| 2015 | 72.70938774799 | 0.59685964756958 | 8.72222510896803 | 63.987162639022 | Mongolia |
| 2016 | 65.48870905638 | 0.600260766781331 | 8.95551202693913 | 56.5331970294409 | Mongolia |
| 2017 | 63.19464565472 | 0.60372196450642 | 8.71584490436944 | 54.4788007503506 | Mongolia |
| 2018 | 58.92641914803 | 0.607501833007913 | 8.95351222448123 | 49.9729069235488 | Mongolia |
| 2019 | 56.08962131795 | 0.611250680890372 | 8.77512591479649 | 47.3144954031535 | Mongolia |
| 2020 | 47.46047337255 | 0.61462963730677 | 8.82108007267644 | 38.6393932998736 | Mongolia |
| 2021 | 49.24309406789 | 0.617621564638127 | 8.83849935014797 | 40.404594717742 | Mongolia |
| 1990 | 2802.94191579807 | 0.466155413419203 | 85.8443989857576 | 2717.09751681231 | Tajikistan |
| 1991 | 2784.57820902189 | 0.472342173046228 | 82.1889671807351 | 2702.38924184115 | Tajikistan |
| 1992 | 2811.23100069365 | 0.47574519497737 | 78.6657726226752 | 2732.56522807097 | Tajikistan |
| 1993 | 2841.28806950835 | 0.478238812995371 | 78.1897417243774 | 2763.09832778397 | Tajikistan |
| 1994 | 2823.66732324749 | 0.478726011215724 | 76.7941947905723 | 2746.87312845692 | Tajikistan |
| 1995 | 2812.84844920604 | 0.477586868115015 | 78.2288285601346 | 2734.61962064591 | Tajikistan |
| 1996 | 2788.83603133733 | 0.474346027462684 | 80.2495344507231 | 2708.58649688661 | Tajikistan |
| 1997 | 2735.76447759007 | 0.470274795927363 | 83.0582839387835 | 2652.70619365129 | Tajikistan |
| 1998 | 2679.04989648153 | 0.465341742120163 | 84.8103390522384 | 2594.23955742929 | Tajikistan |
| 1999 | 2602.55804414825 | 0.46050561615396 | 94.0452167307296 | 2508.51282741752 | Tajikistan |
| 2000 | 2510.69641167059 | 0.457061333395443 | 99.8887746131762 | 2410.80763705741 | Tajikistan |
| 2001 | 2372.38679730188 | 0.45625651276009 | 100.30899727337 | 2272.07780002851 | Tajikistan |
| 2002 | 2206.94169558638 | 0.458807780304158 | 93.1583431623211 | 2113.78335242406 | Tajikistan |
| 2003 | 2055.62590425254 | 0.463329706383786 | 91.5620418926598 | 1964.06386235988 | Tajikistan |
| 2004 | 1910.37335773424 | 0.469154214043517 | 82.3799153954831 | 1827.99344233876 | Tajikistan |
| 2005 | 1773.94473704329 | 0.474637785688857 | 79.7050721589107 | 1694.23966488438 | Tajikistan |
| 2006 | 1695.18203180862 | 0.479651831177988 | 77.2243298356047 | 1617.95770197302 | Tajikistan |
| 2007 | 1608.56160225847 | 0.484211156232784 | 77.392160538539 | 1531.16944171993 | Tajikistan |
| 2008 | 1522.85901204135 | 0.488441135968382 | 75.6541631240183 | 1447.20484891733 | Tajikistan |
| 2009 | 1448.82433158702 | 0.49206404051479 | 72.7332072507077 | 1376.09112433631 | Tajikistan |
| 2010 | 1361.41815868624 | 0.495893963626257 | 70.6250232500272 | 1290.79313543621 | Tajikistan |
| 2011 | 1288.43367136782 | 0.499869804401369 | 16.8622678369646 | 1271.57140353086 | Tajikistan |
| 2012 | 1224.19333484937 | 0.503953884549027 | 16.0112216502008 | 1208.18211319917 | Tajikistan |
| 2013 | 1170.9759803192 | 0.507970675899606 | 16.0334445501416 | 1154.94253576906 | Tajikistan |
| 2014 | 1119.13443115143 | 0.511989517777097 | 15.6343494011823 | 1103.50008175025 | Tajikistan |
| 2015 | 959.42600259432 | 0.515783244686634 | 15.3313427568878 | 944.094659837432 | Tajikistan |
| 2016 | 912.54945019926 | 0.519897002395847 | 15.1363102130715 | 897.413139986189 | Tajikistan |
| 2017 | 867.15303557028 | 0.524492606909067 | 15.071412606817 | 852.081622963463 | Tajikistan |
| 2018 | 834.85155911805 | 0.529153916295379 | 15.0873997638826 | 819.764159354167 | Tajikistan |
| 2019 | 829.31141295248 | 0.533769951484441 | 15.0783568043617 | 814.233056148118 | Tajikistan |
| 2020 | 773.47524453579 | 0.537690530515565 | 15.0481102265519 | 758.427134309238 | Tajikistan |
| 2021 | 760.44133569843 | 0.541511187051998 | 15.0370116669558 | 745.404324031474 | Tajikistan |
| 1990 | 2219.36570708388 | 0.563126887105135 | 11.329104221413 | 2208.03660286247 | Turkmenistan |
| 1991 | 2206.20155856717 | 0.564939385894165 | 10.7102728327118 | 2195.49128573446 | Turkmenistan |
| 1992 | 2246.06410116236 | 0.565587505223265 | 10.4584049652217 | 2235.60569619714 | Turkmenistan |
| 1993 | 2282.66555552119 | 0.567174449333284 | 9.94777992912222 | 2272.71777559207 | Turkmenistan |
| 1994 | 2227.08963469935 | 0.567288975507041 | 9.83078420004616 | 2217.2588504993 | Turkmenistan |
| 1995 | 2193.61661915132 | 0.567198634711689 | 9.89952505687882 | 2183.71709409444 | Turkmenistan |
| 1996 | 2031.42726776547 | 0.567061261285221 | 10.0037328455411 | 2021.42353491993 | Turkmenistan |
| 1997 | 1815.26865164731 | 0.565623083668948 | 10.4181406657664 | 1804.85051098154 | Turkmenistan |
| 1998 | 1582.00693701097 | 0.564405264391893 | 10.6121482435917 | 1571.39478876738 | Turkmenistan |
| 1999 | 1383.55444011034 | 0.564716005636632 | 10.7181184749547 | 1372.83632163539 | Turkmenistan |
| 2000 | 1187.29925938034 | 0.567421033351252 | 9.95881105275242 | 1177.34044832759 | Turkmenistan |
| 2001 | 995.96479437088 | 0.57104381892434 | 9.03223823464796 | 986.932556136232 | Turkmenistan |
| 2002 | 805.82147043786 | 0.575596165867541 | 8.78823224604434 | 797.033238191816 | Turkmenistan |
| 2003 | 634.88930163081 | 0.580820125927832 | 8.85970021961253 | 626.029601411198 | Turkmenistan |
| 2004 | 511.9368093352 | 0.586712349142411 | 8.8774753792725 | 503.059333955928 | Turkmenistan |
| 2005 | 488.74582668124 | 0.593388894864117 | 8.91121206854218 | 479.834614612698 | Turkmenistan |
| 2006 | 428.87674527025 | 0.599896069365258 | 8.7403323873055 | 420.136412882945 | Turkmenistan |
| 2007 | 382.87874077081 | 0.606042198134731 | 8.71540055833629 | 374.163340212474 | Turkmenistan |
| 2008 | 368.10456349451 | 0.611923308807263 | 8.63435220949211 | 359.470211285018 | Turkmenistan |
| 2009 | 332.87127014523 | 0.617652905277457 | 8.74408049625394 | 324.127189648976 | Turkmenistan |
| 2010 | 278.9974197663 | 0.623234588644236 | 8.80118703029658 | 270.196232736003 | Turkmenistan |
| 2011 | 225.89028377333 | 0.629233721702303 | 8.19924309110169 | 217.691040682228 | Turkmenistan |
| 2012 | 146.30916686918 | 0.635263183091614 | 6.90140761437818 | 139.407759254802 | Turkmenistan |
| 2013 | 128.05678348821 | 0.641361893351002 | 6.38483227196765 | 121.671951216242 | Turkmenistan |
| 2014 | 91.54367380335 | 0.647542416932676 | 5.9585827652422 | 85.5850910381078 | Turkmenistan |
| 2015 | 93.54478996127 | 0.65335278951701 | 5.05380960926417 | 88.4909803520058 | Turkmenistan |
| 2016 | 90.48004396264 | 0.658945613392641 | 4.55728747661087 | 85.9227564860291 | Turkmenistan |
| 2017 | 89.15261380573 | 0.664284691942071 | 4.22475075548258 | 84.9278630502474 | Turkmenistan |
| 2018 | 80.17048848315 | 0.669433598508271 | 3.89835577655027 | 76.2721327065997 | Turkmenistan |
| 2019 | 74.9897174173 | 0.674303535870544 | 3.64741566201016 | 71.3423017552898 | Turkmenistan |
| 2020 | 70.0848986686 | 0.678298179976148 | 3.58389356323418 | 66.5010051053658 | Turkmenistan |
| 2021 | 67.22033631248 | 0.682160775869611 | 3.57257011641447 | 63.6477661960655 | Turkmenistan |
| 1990 | 1418.01063492525 | 0.500241735138187 | 16.454557409052 | 1401.5560775162 | Uzbekistan |
| 1991 | 1417.35301939415 | 0.501544609458551 | 16.6980541977455 | 1400.6549651964 | Uzbekistan |
| 1992 | 1470.93794406445 | 0.502905194308151 | 16.3722670059632 | 1454.56567705849 | Uzbekistan |
| 1993 | 1425.29554887194 | 0.506556615160302 | 15.9549250200578 | 1409.34062385188 | Uzbekistan |
| 1994 | 1398.26374746801 | 0.5110185249116 | 15.720859055244 | 1382.54288841277 | Uzbekistan |
| 1995 | 1228.36911670472 | 0.51698626355212 | 15.3789214392984 | 1212.99019526542 | Uzbekistan |
| 1996 | 1000.73463758018 | 0.524392909803147 | 15.1220231920466 | 985.612614388133 | Uzbekistan |
| 1997 | 791.47277167601 | 0.532686328470226 | 15.0588500939315 | 776.413921582079 | Uzbekistan |
| 1998 | 560.30358049275 | 0.541279069137397 | 15.0417843234037 | 545.261796169346 | Uzbekistan |
| 1999 | 448.198301793 | 0.549739390933737 | 13.7577936062512 | 434.440508186749 | Uzbekistan |
| 2000 | 339.57421854706 | 0.557522310762499 | 12.128620273409 | 327.445598273651 | Uzbekistan |
| 2001 | 312.88119010528 | 0.564803382897778 | 10.9774378566324 | 301.903752248648 | Uzbekistan |
| 2002 | 290.63101302114 | 0.571690142599581 | 8.73507662977346 | 281.895936391367 | Uzbekistan |
| 2003 | 271.53892418012 | 0.57800653838209 | 8.81493852410467 | 262.723985656015 | Uzbekistan |
| 2004 | 249.55256529067 | 0.583942986424626 | 8.71468820632026 | 240.83787708435 | Uzbekistan |
| 2005 | 192.77826386779 | 0.589530776570668 | 8.7825998265621 | 183.995664041228 | Uzbekistan |
| 2006 | 113.03743044502 | 0.594498056671551 | 8.91698880059944 | 104.120441644421 | Uzbekistan |
| 2007 | 95.10295735671 | 0.599628054727829 | 8.777443598214 | 86.325513758496 | Uzbekistan |
| 2008 | 90.84313567154 | 0.604889158157396 | 8.94500916580622 | 81.8981265057338 | Uzbekistan |
| 2009 | 87.12435233073 | 0.610351745244056 | 8.59949287935515 | 78.5248594513749 | Uzbekistan |
| 2010 | 83.29259814 | 0.616097907401681 | 8.75636312906237 | 74.5362350109376 | Uzbekistan |
| 2011 | 77.18921762395 | 0.621344618930939 | 8.99500686762654 | 68.1942107563235 | Uzbekistan |
| 2012 | 62.89269408018 | 0.62620212543294 | 8.92536905595841 | 53.9673250242216 | Uzbekistan |
| 2013 | 45.84304566089 | 0.630796403406203 | 8.33385680767847 | 37.5091888532115 | Uzbekistan |
| 2014 | 43.32008758561 | 0.635257049718365 | 6.90120410001793 | 36.4188834855921 | Uzbekistan |
| 2015 | 46.39810565844 | 0.639805638905225 | 6.69100182801089 | 39.7071038304291 | Uzbekistan |
| 2016 | 42.93273172415 | 0.644230597300764 | 6.14231996348762 | 36.7904117606624 | Uzbekistan |
| 2017 | 25.63817537156 | 0.648119346591586 | 5.80283358478079 | 19.8353417867792 | Uzbekistan |
| 2018 | 28.93845086521 | 0.651918144182724 | 5.11039875124183 | 23.8280521139682 | Uzbekistan |
| 2019 | 26.58441541321 | 0.655836959268026 | 4.81426333428693 | 21.7701520789231 | Uzbekistan |
| 2020 | 24.6194265953 | 0.659124204371032 | 4.6823906791968 | 19.9370359161032 | Uzbekistan |
| 2021 | 23.67635090593 | 0.662621694181816 | 4.19988570756091 | 19.4764651983691 | Uzbekistan |
| 1990 | 139.13624955036 | 0.557773300317074 | 12.1183363264519 | 127.017913223908 | Albania |
| 1991 | 143.8171333912 | 0.555629411790174 | 13.0190928809511 | 130.798040510249 | Albania |
| 1992 | 140.59450206836 | 0.553107897492912 | 13.896045471901 | 126.698456596459 | Albania |
| 1993 | 134.96446817617 | 0.552453327893755 | 13.9334644389519 | 121.031003737218 | Albania |
| 1994 | 154.92424918237 | 0.553763669315605 | 12.8525326661424 | 142.071716516228 | Albania |
| 1995 | 115.43712893866 | 0.557040889185472 | 12.9093832113893 | 102.527745727271 | Albania |
| 1996 | 105.55637192716 | 0.562248886206679 | 11.5110295256957 | 94.0453424014643 | Albania |
| 1997 | 100.7543112042 | 0.566802804232969 | 9.71383740477 | 91.04047379943 | Albania |
| 1998 | 94.40067038225 | 0.572534101516668 | 8.97222163699562 | 85.4284487452544 | Albania |
| 1999 | 81.55068095048 | 0.578295461805887 | 8.8559296317171 | 72.6947513187629 | Albania |
| 2000 | 71.44026312396 | 0.58466418835901 | 8.94984122742147 | 62.4904218965385 | Albania |
| 2001 | 63.90531688881 | 0.592412946510661 | 8.86599342932469 | 55.0393234594853 | Albania |
| 2002 | 57.41424390937 | 0.599865977568749 | 8.87071809956098 | 48.543525809809 | Albania |
| 2003 | 51.69254396939 | 0.606971165987994 | 8.72748535402143 | 42.9650586153686 | Albania |
| 2004 | 46.55319756159 | 0.613864246664272 | 8.88919528167155 | 37.6640022799184 | Albania |
| 2005 | 42.81548026638 | 0.620679614590226 | 8.72852859784227 | 34.0869516685377 | Albania |
| 2006 | 40.94829076031 | 0.627485617497143 | 8.06456572916829 | 32.8837250311417 | Albania |
| 2007 | 39.87031038181 | 0.634167968985356 | 7.10279066700526 | 32.7675197148047 | Albania |
| 2008 | 39.47875238332 | 0.640766134349698 | 6.11810911610727 | 33.3606432672127 | Albania |
| 2009 | 39.16813575172 | 0.646816130123751 | 5.84279417473855 | 33.3253415769815 | Albania |
| 2010 | 38.96813696524 | 0.652654202642964 | 5.0376336029409 | 33.9305033622991 | Albania |
| 2011 | 39.63841297061 | 0.658196772707548 | 4.48530669094655 | 35.1531062796634 | Albania |
| 2012 | 39.5314904567 | 0.66355677961127 | 4.23568820756606 | 35.2958022491339 | Albania |
| 2013 | 38.88027745932 | 0.668739558985928 | 3.94205873756826 | 34.9382187217517 | Albania |
| 2014 | 37.81904288448 | 0.67411298539372 | 3.79951495791159 | 34.0195279265684 | Albania |
| 2015 | 37.00009328567 | 0.679668600655757 | 3.6273417373874 | 33.3727515482826 | Albania |
| 2016 | 36.32121967817 | 0.68503664759447 | 3.36302750650362 | 32.9581921716664 | Albania |
| 2017 | 35.12702193945 | 0.690379199197076 | 3.31972019938119 | 31.8073017400688 | Albania |
| 2018 | 34.00507858388 | 0.695566877664306 | 3.1833108441127 | 30.8217677397673 | Albania |
| 2019 | 31.36495172764 | 0.700287863295906 | 2.8860667756362 | 28.4788849520038 | Albania |
| 2020 | 29.661140997 | 0.703790245372837 | 2.90397283293667 | 26.7571681640633 | Albania |
| 2021 | 25.87328782342 | 0.706849790878611 | 2.74799800557814 | 23.1252898178419 | Albania |
| 1990 | 20.19850384054 | 0.541132539726015 | 15.0625038275996 | 5.13600001294036 | Bosnia and Herzegovina |
| 1991 | 19.24847399518 | 0.541346250550883 | 15.0805486911933 | 4.16792530398667 | Bosnia and Herzegovina |
| 1992 | 19.15531487876 | 0.539303227421668 | 15.0564105474567 | 4.09890433130328 | Bosnia and Herzegovina |
| 1993 | 18.92734906842 | 0.53603188160081 | 15.0471671320291 | 3.88018193639088 | Bosnia and Herzegovina |
| 1994 | 19.05599762497 | 0.533853389744284 | 15.0791656520149 | 3.97683197295509 | Bosnia and Herzegovina |
| 1995 | 19.17537875087 | 0.534025536110865 | 15.0807971900327 | 4.09458156083733 | Bosnia and Herzegovina |
| 1996 | 17.75999329314 | 0.545549215040903 | 14.5205563301169 | 3.23943696302313 | Bosnia and Herzegovina |
| 1997 | 16.80690920711 | 0.563565414584231 | 10.7986568897271 | 6.00825231738291 | Bosnia and Herzegovina |
| 1998 | 16.01972363591 | 0.58182951538312 | 8.82869076267803 | 7.19103287323197 | Bosnia and Herzegovina |
| 1999 | 14.17280806201 | 0.597997468748356 | 8.76666660276536 | 5.40614145924464 | Bosnia and Herzegovina |
| 2000 | 13.33685163681 | 0.610950684608214 | 8.74920879042393 | 4.58764284638607 | Bosnia and Herzegovina |
| 2001 | 12.41983878094 | 0.621989463929258 | 8.91560213734216 | 3.50423664359784 | Bosnia and Herzegovina |
| 2002 | 13.92835042726 | 0.632075517723657 | 8.23895078594917 | 5.68939964131083 | Bosnia and Herzegovina |
| 2003 | 14.52946040101 | 0.640635102031521 | 6.658150815048 | 7.871309585962 | Bosnia and Herzegovina |
| 2004 | 14.62871559083 | 0.64779373168743 | 6.11754165004667 | 8.51117394078333 | Bosnia and Herzegovina |
| 2005 | 14.55519277471 | 0.653859136710253 | 5.01971171409089 | 9.5354810606191 | Bosnia and Herzegovina |
| 2006 | 14.97806071646 | 0.659813662252595 | 4.19465907126692 | 10.7834016451931 | Bosnia and Herzegovina |
| 2007 | 15.09158913847 | 0.665578735044097 | 4.21243343366396 | 10.879155704806 | Bosnia and Herzegovina |
| 2008 | 14.91234253734 | 0.671100813673566 | 3.95172897968261 | 10.9606135576574 | Bosnia and Herzegovina |
| 2009 | 14.58301169759 | 0.676032900022704 | 3.5568553052369 | 11.0261563923531 | Bosnia and Herzegovina |
| 2010 | 14.41267373035 | 0.680824195636093 | 3.60444515795441 | 10.8082285723956 | Bosnia and Herzegovina |
| 2011 | 13.52486069135 | 0.685399264182467 | 3.38262558823441 | 10.1422351031156 | Bosnia and Herzegovina |
| 2012 | 13.43600163649 | 0.689506566189932 | 3.37058223852459 | 10.0654193979654 | Bosnia and Herzegovina |
| 2013 | 13.72971361274 | 0.693508711211908 | 3.11754227452821 | 10.6121713382118 | Bosnia and Herzegovina |
| 2014 | 12.89337042985 | 0.697031893012906 | 3.10706427288531 | 9.78630615696469 | Bosnia and Herzegovina |
| 2015 | 13.45888548009 | 0.700446047366461 | 3.03271211430648 | 10.4261733657835 | Bosnia and Herzegovina |
| 2016 | 13.40247724601 | 0.704079343718599 | 2.83402045304194 | 10.5684567929681 | Bosnia and Herzegovina |
| 2017 | 13.09849007668 | 0.70805343112809 | 2.79918565487091 | 10.2993044218091 | Bosnia and Herzegovina |
| 2018 | 12.55417515023 | 0.712347302038319 | 2.7323749383084 | 9.8218002119216 | Bosnia and Herzegovina |
| 2019 | 12.21421215397 | 0.716857124768862 | 2.58713360715935 | 9.62707854681064 | Bosnia and Herzegovina |
| 2020 | 11.04587426293 | 0.720202056378697 | 2.53077506977339 | 8.51509919315661 | Bosnia and Herzegovina |
| 2021 | 10.30058782249 | 0.72307789321024 | 2.326649040318 | 7.973938782172 | Bosnia and Herzegovina |
| 1990 | 18.42362343185 | 0.633446498017686 | 6.9553806147304 | 11.4682428171196 | Bulgaria |
| 1991 | 21.4547713953 | 0.643069487769455 | 6.0861517595819 | 15.3686196357181 | Bulgaria |
| 1992 | 21.88467934005 | 0.651186876165789 | 5.38459811470203 | 16.500081225348 | Bulgaria |
| 1993 | 19.48688045022 | 0.659161569264411 | 4.43816612219613 | 15.0487143280239 | Bulgaria |
| 1994 | 23.03302039601 | 0.667430460276086 | 3.83835873584397 | 19.194661660166 | Bulgaria |
| 1995 | 18.56710813068 | 0.673999976701174 | 3.84438909563724 | 14.7227190350428 | Bulgaria |
| 1996 | 17.74699612693 | 0.678862583541026 | 3.6208067454361 | 14.1261893814939 | Bulgaria |
| 1997 | 20.16477019795 | 0.680221466342146 | 3.58718497011483 | 16.5775852278352 | Bulgaria |
| 1998 | 16.89703263826 | 0.678496876732148 | 3.60357367824012 | 13.2934589600199 | Bulgaria |
| 1999 | 14.21955125341 | 0.677446543282232 | 3.6289351688534 | 10.5906160845566 | Bulgaria |
| 2000 | 15.48859218367 | 0.681004523812614 | 3.57513584196 | 11.91345634171 | Bulgaria |
| 2001 | 13.42002903429 | 0.686487366008627 | 3.38149203598061 | 10.0385369983094 | Bulgaria |
| 2002 | 13.60003247367 | 0.69151167375307 | 3.09791690868129 | 10.5021155649887 | Bulgaria |
| 2003 | 12.10310160285 | 0.69556596473307 | 3.21005381483186 | 8.89304778801814 | Bulgaria |
| 2004 | 11.28418137823 | 0.699881950859544 | 2.87783464024246 | 8.40634673798754 | Bulgaria |
| 2005 | 10.10550457339 | 0.704471206966935 | 2.91548655186217 | 7.19001802152783 | Bulgaria |
| 2006 | 9.64521679408 | 0.708868293577424 | 2.7840440000914 | 6.8611727939886 | Bulgaria |
| 2007 | 9.36203134062 | 0.713192402424104 | 2.67781699255667 | 6.68421434806333 | Bulgaria |
| 2008 | 10.47887061725 | 0.717672450499802 | 2.5418195176275 | 7.9370510996225 | Bulgaria |
| 2009 | 10.56072988926 | 0.723676626795129 | 2.48596270621407 | 8.07476718304592 | Bulgaria |
| 2010 | 12.26575882566 | 0.730701737095494 | 2.15603754100929 | 10.1097212846507 | Bulgaria |
| 2011 | 14.82763380458 | 0.735749281014389 | 2.10958115111647 | 12.7180526534635 | Bulgaria |
| 2012 | 17.75108991364 | 0.739177517343213 | 2.00402682996203 | 15.747063083678 | Bulgaria |
| 2013 | 19.88716547249 | 0.742135948467762 | 1.93318582856829 | 17.9539796439217 | Bulgaria |
| 2014 | 22.22968768676 | 0.745372642577071 | 1.83363976141787 | 20.3960479253421 | Bulgaria |
| 2015 | 20.49324409042 | 0.748979058373083 | 1.7365430383437 | 18.7567010520763 | Bulgaria |
| 2016 | 19.30980997432 | 0.752259198563744 | 1.83521012108603 | 17.474599853234 | Bulgaria |
| 2017 | 20.23738134411 | 0.75566586464625 | 1.82876907810324 | 18.4086122660068 | Bulgaria |
| 2018 | 19.47110464152 | 0.759148460251881 | 1.83834189692658 | 17.6327627445934 | Bulgaria |
| 2019 | 18.28147693653 | 0.762359790844767 | 1.71988561257779 | 16.5615913239522 | Bulgaria |
| 2020 | 16.52254060529 | 0.765089710642011 | 1.73158773360119 | 14.7909528716888 | Bulgaria |
| 2021 | 16.77765627148 | 0.768150939342518 | 1.72871060915013 | 15.0489456623299 | Bulgaria |
| 1990 | 8.88189282552 | 0.668906358291928 | 3.8983995431344 | 4.9834932823856 | Croatia |
| 1991 | 8.64473565092 | 0.674724599866063 | 3.6102398831434 | 5.0344957677766 | Croatia |
| 1992 | 9.05777226414 | 0.678093133084916 | 3.58173856569023 | 5.47603369844977 | Croatia |
| 1993 | 8.48825512095 | 0.678221047043161 | 3.58861735635644 | 4.89963776459356 | Croatia |
| 1994 | 8.45651606211 | 0.677548132655056 | 3.62500767607125 | 4.83150838603875 | Croatia |
| 1995 | 7.01649991669 | 0.677029465132868 | 3.58878823900447 | 3.42771167768553 | Croatia |
| 1996 | 6.54877730042 | 0.679507318367616 | 3.609698280455 | 2.939079019965 | Croatia |
| 1997 | 6.44778412149 | 0.685365010443468 | 3.43635757498771 | 3.01142654650229 | Croatia |
| 1998 | 5.59923635337 | 0.691365518598319 | 3.01164575024241 | 2.58759060312759 | Croatia |
| 1999 | 4.42695954176 | 0.696636411104102 | 3.18675663449614 | 1.24020290726386 | Croatia |
| 2000 | 4.40370623823 | 0.703039898563322 | 2.84812242660618 | 1.55558381162382 | Croatia |
| 2001 | 4.23021405298 | 0.709536840872917 | 2.66030716818267 | 1.56990688479733 | Croatia |
| 2002 | 4.00277345838 | 0.715383421635164 | 2.55661703730486 | 1.44615642107514 | Croatia |
| 2003 | 3.56288624614 | 0.721130377216859 | 2.58622885220385 | 0.976657393936154 | Croatia |
| 2004 | 3.41878828777 | 0.726327651623968 | 2.39444882169667 | 1.02433946607333 | Croatia |
| 2005 | 3.35873469916 | 0.732154139815163 | 2.12922569232464 | 1.22950900683536 | Croatia |
| 2006 | 3.22329548902 | 0.738066878515765 | 1.94205999997414 | 1.28123548904586 | Croatia |
| 2007 | 3.45607177827 | 0.743148776515466 | 1.73180219224781 | 1.72426958602219 | Croatia |
| 2008 | 3.77416130973 | 0.748469602229701 | 1.70554152070237 | 2.06861978902763 | Croatia |
| 2009 | 4.53613371012 | 0.754065605198048 | 1.89234188915381 | 2.64379182096619 | Croatia |
| 2010 | 5.47696135145 | 0.759472251706366 | 1.77432259738054 | 3.70263875406946 | Croatia |
| 2011 | 7.34748989351 | 0.763686381293279 | 1.790714103845 | 5.556775789665 | Croatia |
| 2012 | 8.66953839346 | 0.767405335599999 | 1.78478222665036 | 6.88475616680964 | Croatia |
| 2013 | 10.46694443202 | 0.771151880279813 | 1.70654751147481 | 8.76039692054519 | Croatia |
| 2014 | 11.62202065894 | 0.774479240521915 | 1.73168301202787 | 9.89033764691213 | Croatia |
| 2015 | 12.98631442376 | 0.777865888163329 | 1.70299297529315 | 11.2833214484669 | Croatia |
| 2016 | 13.63842901868 | 0.781443605436965 | 1.67681498459082 | 11.9616140340892 | Croatia |
| 2017 | 14.40233137024 | 0.785074536823775 | 1.65031391249577 | 12.7520174577442 | Croatia |
| 2018 | 14.15102289599 | 0.788671410129235 | 1.62948090964942 | 12.5215419863406 | Croatia |
| 2019 | 12.47380304895 | 0.792475415454183 | 1.56311712436244 | 10.9106859245876 | Croatia |
| 2020 | 11.60322699687 | 0.795462271729482 | 1.54647770057926 | 10.0567492962907 | Croatia |
| 2021 | 11.07549162046 | 0.798341026936276 | 1.52815416308187 | 9.54733745737812 | Croatia |
| 1990 | 21.56006574263 | 0.681848021213857 | 3.56973818444839 | 17.9903275581816 | Czechia |
| 1991 | 22.04688846418 | 0.688002859136679 | 3.34006589206901 | 18.706822572111 | Czechia |
| 1992 | 21.91502068554 | 0.697577809122723 | 3.0715751358665 | 18.8434455496735 | Czechia |
| 1993 | 19.12938245365 | 0.710853415862456 | 2.70704314362328 | 16.4223393100267 | Czechia |
| 1994 | 17.89623832007 | 0.725969686167497 | 2.40587146337754 | 15.4903668566925 | Czechia |
| 1995 | 13.97524339714 | 0.737981721993762 | 2.06478405594938 | 11.9104593411906 | Czechia |
| 1996 | 11.81888823071 | 0.746041379938535 | 1.87786402975825 | 9.94102420095175 | Czechia |
| 1997 | 12.25178738516 | 0.751680343695868 | 1.75097011236077 | 10.5008172727992 | Czechia |
| 1998 | 11.11656629166 | 0.757033755457614 | 1.77290578829984 | 9.34366050336016 | Czechia |
| 1999 | 9.10495975064 | 0.762428880357609 | 1.72554516725509 | 7.37941458338491 | Czechia |
| 2000 | 9.08799091299 | 0.767766159168669 | 1.78215110034134 | 7.30583981264866 | Czechia |
| 2001 | 8.57473128106 | 0.772167239650309 | 1.79654577570688 | 6.77818550535313 | Czechia |
| 2002 | 8.8232781521 | 0.776927934062507 | 1.751845398556 | 7.071432753544 | Czechia |
| 2003 | 8.07062423637 | 0.782448081111072 | 1.65195288102057 | 6.41867135534943 | Czechia |
| 2004 | 7.59442321945 | 0.787126404974676 | 1.62612109404283 | 5.96830212540717 | Czechia |
| 2005 | 6.92327058391 | 0.79133394820415 | 1.57473990070289 | 5.34853068320711 | Czechia |
| 2006 | 6.78486966697 | 0.794922783687463 | 1.59003159275975 | 5.19483807421025 | Czechia |
| 2007 | 7.02414703648 | 0.798254117717088 | 1.53160498922 | 5.49254204726 | Czechia |
| 2008 | 7.3073625071 | 0.8017648456328 | 1.52758072776867 | 5.77978177933133 | Czechia |
| 2009 | 8.72357616063 | 0.804962224676367 | 1.53115059334837 | 7.19242556728163 | Czechia |
| 2010 | 11.00034979641 | 0.808747327653098 | 1.54166865277352 | 9.45868114363648 | Czechia |
| 2011 | 13.75776648713 | 0.812054784320429 | 1.52396822487521 | 12.2337982622548 | Czechia |
| 2012 | 16.35419863106 | 0.814230401372392 | 1.50051321260765 | 14.8536854184524 | Czechia |
| 2013 | 18.17814952754 | 0.815615361108922 | 1.52931235728671 | 16.6488371702533 | Czechia |
| 2014 | 20.57689018561 | 0.816227257169909 | 1.51864060336032 | 19.0582495822497 | Czechia |
| 2015 | 23.5917064903 | 0.816564684423948 | 1.51350108579018 | 22.0782054045098 | Czechia |
| 2016 | 26.05570010705 | 0.817153025533518 | 1.50425202591516 | 24.5514480811348 | Czechia |
| 2017 | 26.24500817566 | 0.819067680353287 | 1.49171026277063 | 24.7532979128894 | Czechia |
| 2018 | 26.91437279865 | 0.822034486079137 | 1.49905836659278 | 25.4153144320572 | Czechia |
| 2019 | 28.66972246568 | 0.824784472798171 | 1.49319941076892 | 27.1765230549111 | Czechia |
| 2020 | 23.63763442841 | 0.82662663116323 | 1.48801850220259 | 22.1496159262074 | Czechia |
| 2021 | 22.34536969254 | 0.828450433122785 | 1.48850401094717 | 20.8568656815928 | Czechia |
| 1990 | 21.2831020491 | 0.649419990664883 | 4.72845334818407 | 16.5546487009159 | Hungary |
| 1991 | 22.18051379175 | 0.653707154681922 | 5.0449915900593 | 17.1355222016907 | Hungary |
| 1992 | 21.10501883773 | 0.660382905455002 | 4.61418997229952 | 16.4908288654305 | Hungary |
| 1993 | 19.13464416367 | 0.667543556201878 | 3.79955511069638 | 15.3350890529736 | Hungary |
| 1994 | 17.99860694116 | 0.675051406249796 | 3.63275371467288 | 14.3658532264871 | Hungary |
| 1995 | 14.83018851978 | 0.683186411756315 | 3.38695314123797 | 11.443235378542 | Hungary |
| 1996 | 13.73289955971 | 0.690883333624564 | 3.16544969915906 | 10.5674498605509 | Hungary |
| 1997 | 13.30239733744 | 0.697935513627811 | 3.09768213545567 | 10.2047152019843 | Hungary |
| 1998 | 12.88736826215 | 0.704202719984894 | 2.89216251619545 | 9.99520574595455 | Hungary |
| 1999 | 10.75147416604 | 0.709980626709343 | 2.87774713855852 | 7.87372702748148 | Hungary |
| 2000 | 11.75212565348 | 0.71580528057596 | 2.53967067732604 | 9.21245497615396 | Hungary |
| 2001 | 10.35909164095 | 0.722099055327699 | 2.38144121232279 | 7.97765042862721 | Hungary |
| 2002 | 9.59692766673 | 0.7283845957697 | 2.18611153027667 | 7.41081613645333 | Hungary |
| 2003 | 8.7790330213 | 0.734450657925147 | 2.12704292370611 | 6.65199009759389 | Hungary |
| 2004 | 8.09230757268 | 0.740099974680479 | 1.97199808859939 | 6.12030948408061 | Hungary |
| 2005 | 7.87530656495 | 0.745356806381464 | 1.85001761373308 | 6.02528895121692 | Hungary |
| 2006 | 7.56248006507 | 0.750515380971436 | 1.79217630935659 | 5.77030375571341 | Hungary |
| 2007 | 7.60069081169 | 0.754942942046377 | 1.79997686443711 | 5.8007139472529 | Hungary |
| 2008 | 8.19834528605 | 0.75931746766686 | 1.75811946779478 | 6.44022581825522 | Hungary |
| 2009 | 9.31180849413 | 0.763563686880312 | 1.70536609411564 | 7.60644240001436 | Hungary |
| 2010 | 12.23420071656 | 0.767328649261652 | 1.70504744070206 | 10.5291532758579 | Hungary |
| 2011 | 16.79213261554 | 0.769701942198238 | 1.82494942211093 | 14.9671831934291 | Hungary |
| 2012 | 22.13909120165 | 0.770784245285382 | 1.6576220796335 | 20.4814691220165 | Hungary |
| 2013 | 26.75201808718 | 0.77156169789519 | 1.70661716834631 | 25.0454009188337 | Hungary |
| 2014 | 29.26157597388 | 0.772575045716759 | 1.73523047509947 | 27.5263454987805 | Hungary |
| 2015 | 30.9811444367 | 0.774047382518708 | 1.7458764926702 | 29.2352679440298 | Hungary |
| 2016 | 29.21939434677 | 0.776150305326983 | 1.68381778024167 | 27.5355765665283 | Hungary |
| 2017 | 27.84401952303 | 0.779116417184348 | 1.72824159282973 | 26.1157779302003 | Hungary |
| 2018 | 27.83782815019 | 0.782083661193681 | 1.65650900061164 | 26.1813191495784 | Hungary |
| 2019 | 32.65880787886 | 0.784910308752534 | 1.64118726753615 | 31.0176206113238 | Hungary |
| 2020 | 29.525094963 | 0.787620389699141 | 1.62582101397273 | 27.8992739490273 | Hungary |
| 2021 | 27.56641686177 | 0.790754768173338 | 1.5599315977079 | 26.0064852640621 | Hungary |
| 1990 | 311.28124318383 | 0.60902609384517 | 8.92884177440124 | 302.352401409429 | North Macedonia |
| 1991 | 290.91583091148 | 0.611337452940336 | 8.87514695178753 | 282.040683959692 | North Macedonia |
| 1992 | 279.70036149976 | 0.612708856229581 | 8.87565822659036 | 270.82470327317 | North Macedonia |
| 1993 | 236.00612679836 | 0.613195098529578 | 8.79533930887506 | 227.210787489485 | North Macedonia |
| 1994 | 219.76168121851 | 0.614362905009181 | 8.86729133250908 | 210.894389886001 | North Macedonia |
| 1995 | 192.12013820494 | 0.616994877821318 | 8.8774072571816 | 183.242730947758 | North Macedonia |
| 1996 | 156.53490314085 | 0.620944017369103 | 8.93105402523818 | 147.603849115612 | North Macedonia |
| 1997 | 140.9523980577 | 0.626223811356564 | 8.69911417146614 | 132.253283886234 | North Macedonia |
| 1998 | 133.5997690692 | 0.632059410181692 | 8.11414161533694 | 125.485627453863 | North Macedonia |
| 1999 | 96.35951338136 | 0.63668725909008 | 7.02043605980522 | 89.3390773215548 | North Macedonia |
| 2000 | 77.80588395119 | 0.641651242944231 | 6.24416069408492 | 71.5617232571051 | North Macedonia |
| 2001 | 72.22177493705 | 0.648049675806335 | 5.91918468660587 | 66.3025902504441 | North Macedonia |
| 2002 | 66.43027269706 | 0.655398790077733 | 4.92297448016292 | 61.5072982168971 | North Macedonia |
| 2003 | 66.57019183404 | 0.663408891257403 | 4.22767444754707 | 62.3425173864929 | North Macedonia |
| 2004 | 58.96882282629 | 0.671237520250547 | 3.90357266162537 | 55.0652501646646 | North Macedonia |
| 2005 | 51.61895006607 | 0.678047272834883 | 3.60334145123043 | 48.0156086148396 | North Macedonia |
| 2006 | 53.21491139448 | 0.684044336465219 | 3.40774042811967 | 49.8071709663603 | North Macedonia |
| 2007 | 51.43178652966 | 0.689729480929241 | 3.44722989102453 | 47.9845566386355 | North Macedonia |
| 2008 | 52.50066934395 | 0.695376955204976 | 3.08618469957948 | 49.4144846443705 | North Macedonia |
| 2009 | 55.56605688658 | 0.700453052486829 | 2.91957046872769 | 52.6464864178523 | North Macedonia |
| 2010 | 49.17973011872 | 0.705589323990971 | 2.84427833525439 | 46.3354517834656 | North Macedonia |
| 2011 | 49.66073173917 | 0.710592520608989 | 2.6903712407927 | 46.9703604983773 | North Macedonia |
| 2012 | 55.73505876723 | 0.715173147623766 | 2.65299656483308 | 53.0820622023969 | North Macedonia |
| 2013 | 56.31421873533 | 0.719671149159541 | 2.562604035609 | 53.751614699721 | North Macedonia |
| 2014 | 55.03825720956 | 0.724089588503488 | 2.4369200242939 | 52.6013371852661 | North Macedonia |
| 2015 | 54.19662763363 | 0.728603773495479 | 2.2118476553787 | 51.9847799782513 | North Macedonia |
| 2016 | 60.30639429792 | 0.733173624358204 | 2.17591645997576 | 58.1304778379442 | North Macedonia |
| 2017 | 52.3664797918 | 0.737339955393252 | 1.98099492773038 | 50.3854848640696 | North Macedonia |
| 2018 | 39.65035435244 | 0.741334369868002 | 1.81760545698423 | 37.8327488954558 | North Macedonia |
| 2019 | 38.26370155152 | 0.74519356873628 | 1.77105513163169 | 36.4926464198883 | North Macedonia |
| 2020 | 32.82369417556 | 0.747849899969173 | 1.81512602639294 | 31.0085681491671 | North Macedonia |
| 2021 | 27.66490389116 | 0.750629703270047 | 1.81649134684571 | 25.8484125443143 | North Macedonia |
| 1990 | 5.09108745434 | 0.674225719793669 | 3.68416180555731 | 1.40692564878269 | Montenegro |
| 1991 | 4.76839995904 | 0.674305722529628 | 3.78943289011075 | 0.97896706892925 | Montenegro |
| 1992 | 4.623887102 | 0.672664771832277 | 3.69791147273129 | 0.92597562926871 | Montenegro |
| 1993 | 4.61742518596 | 0.668665719204089 | 3.83360538648234 | 0.78381979947766 | Montenegro |
| 1994 | 4.64759407152 | 0.665011931002947 | 4.08111377465 | 0.56648029687 | Montenegro |
| 1995 | 4.60513250078 | 0.662765437115331 | 4.02598026687905 | 0.579152233900952 | Montenegro |
| 1996 | 4.6013900599 | 0.663414768746409 | 4.11381129098 | 0.48757876892 | Montenegro |
| 1997 | 4.5140460365 | 0.665542031695028 | 4.0787077765175 | 0.4353382599825 | Montenegro |
| 1998 | 4.413783495 | 0.669024770846263 | 3.86731302013302 | 0.546470474866977 | Montenegro |
| 1999 | 4.35102178768 | 0.672096102691543 | 3.75154255892615 | 0.599479228753846 | Montenegro |
| 2000 | 4.3177999735 | 0.677306850284635 | 3.62385275717476 | 0.693947216325238 | Montenegro |
| 2001 | 4.31226403359 | 0.683433454098582 | 3.36973124320311 | 0.942532790386889 | Montenegro |
| 2002 | 4.12500400372 | 0.68993948313756 | 3.41044853635949 | 0.714555467360513 | Montenegro |
| 2003 | 3.95956296679 | 0.696517506230494 | 3.12965082980744 | 0.829912136982558 | Montenegro |
| 2004 | 3.73260776257 | 0.702998276030296 | 2.87747966777976 | 0.855128094790244 | Montenegro |
| 2005 | 3.59800045292 | 0.709065464700775 | 2.58810067138576 | 1.00989978153424 | Montenegro |
| 2006 | 3.50066961335 | 0.715459074033141 | 2.55403359134308 | 0.946636022006923 | Montenegro |
| 2007 | 3.39266455594 | 0.722503939575403 | 2.27617628864719 | 1.11648826729281 | Montenegro |
| 2008 | 3.5373269506 | 0.729981755712811 | 2.37092549005244 | 1.16640146054756 | Montenegro |
| 2009 | 3.59010267426 | 0.736538925645409 | 2.11001419609429 | 1.48008847816571 | Montenegro |
| 2010 | 3.5991919164 | 0.743347873367499 | 1.69063154015074 | 1.90856037624926 | Montenegro |
| 2011 | 3.34099187239 | 0.750080328162275 | 1.86611675557686 | 1.47487511681314 | Montenegro |
| 2012 | 3.16236320541 | 0.755578379953158 | 1.81801715894464 | 1.34434604646536 | Montenegro |
| 2013 | 2.98947296093 | 0.760886907477753 | 1.73311603099394 | 1.25635692993606 | Montenegro |
| 2014 | 2.82076296783 | 0.765776526527122 | 1.74552870594552 | 1.07523426188448 | Montenegro |
| 2015 | 2.54787612552 | 0.770561277708901 | 1.841446462505 | 0.706429663015 | Montenegro |
| 2016 | 2.37049499129 | 0.775200770239438 | 1.75035051641138 | 0.620144474878621 | Montenegro |
| 2017 | 2.02860103874 | 0.779764631848314 | 1.61684807717161 | 0.411752961568387 | Montenegro |
| 2018 | 1.77850868844 | 0.784408971391706 | 1.6168802426845 | 0.1616284457555 | Montenegro |
| 2019 | 1.68612949251 | 0.789026903095641 | 1.60235680182364 | 0.0837726906863636 | Montenegro |
| 2020 | 1.57039286661 | 0.792554392852858 | 1.53380633015125 | 0.0365865364587501 | Montenegro |
| 2021 | 1.52696084955 | 0.795800584021403 | 1.50741438838174 | 0.019546461168261 | Montenegro |
| 1990 | 6.84820192009 | 0.62722788770829 | 6.84820192009 | 0 | Poland |
| 1991 | 5.37918661852 | 0.63267147050056 | 5.37918661852 | 0 | Poland |
| 1992 | 4.77763279032 | 0.64066179337278 | 4.77763279032 | 0 | Poland |
| 1993 | 3.83704563528 | 0.648950499724012 | 3.83704563528 | 0 | Poland |
| 1994 | 3.8688438693 | 0.658010514323026 | 3.85595269334595 | 0.0128911759540542 | Poland |
| 1995 | 3.50507266407 | 0.666678866524195 | 3.50507266407 | 0 | Poland |
| 1996 | 3.47328811054 | 0.674378829090982 | 3.47328811054 | 0 | Poland |
| 1997 | 2.90503992141 | 0.682587612300048 | 2.90503992141 | 0 | Poland |
| 1998 | 2.5837876251 | 0.690805670678968 | 2.5837876251 | 0 | Poland |
| 1999 | 2.40503208705 | 0.6985360555797 | 2.40503208705 | 0 | Poland |
| 2000 | 2.17842178081 | 0.706662051990276 | 2.17842178081 | 0 | Poland |
| 2001 | 2.18740912767 | 0.714831304495085 | 2.18676717432286 | 0.000641953347142898 | Poland |
| 2002 | 2.02339289416 | 0.722009458087754 | 2.02339289416 | 0 | Poland |
| 2003 | 1.69919472227 | 0.728155253652566 | 1.69919472227 | 0 | Poland |
| 2004 | 1.52302306034 | 0.733673191846001 | 1.52302306034 | 0 | Poland |
| 2005 | 1.57779865004 | 0.738428766593663 | 1.56801729473643 | 0.00978135530357149 | Poland |
| 2006 | 1.45026171633 | 0.742363708580365 | 1.45026171633 | 0 | Poland |
| 2007 | 1.83825432869 | 0.746008817400637 | 1.62309814402077 | 0.215156184669231 | Poland |
| 2008 | 1.92867994818 | 0.750521426475047 | 1.73446410341632 | 0.194215844763684 | Poland |
| 2009 | 2.18457253851 | 0.756427378476364 | 1.72819388465462 | 0.456378653855384 | Poland |
| 2010 | 2.51918559562 | 0.763184973687688 | 1.73957755478731 | 0.779608040832692 | Poland |
| 2011 | 2.66824126061 | 0.769407164213607 | 1.82785452607577 | 0.840386734534231 | Poland |
| 2012 | 3.14964163273 | 0.774680767244991 | 1.76709241280184 | 1.38254921992816 | Poland |
| 2013 | 4.17745673951 | 0.779434257316854 | 1.78451913434658 | 2.39293760516342 | Poland |
| 2014 | 4.9315654792 | 0.783682368684721 | 1.7046749013686 | 3.2268905778314 | Poland |
| 2015 | 5.56046159066 | 0.78744534315882 | 1.66363777677745 | 3.89682381388255 | Poland |
| 2016 | 6.2854126139 | 0.790651014611623 | 1.61727623146564 | 4.66813638243436 | Poland |
| 2017 | 7.60657656989 | 0.79453034463794 | 1.5610528341885 | 6.0455237357015 | Poland |
| 2018 | 7.49638335371 | 0.79947898187813 | 1.5384385695513 | 5.9579447841587 | Poland |
| 2019 | 7.1090439978 | 0.804791331675008 | 1.53265583821022 | 5.57638815958978 | Poland |
| 2020 | 6.50136313111 | 0.808795317506383 | 1.52527248309297 | 4.97609064801703 | Poland |
| 2021 | 6.21680559241 | 0.812042808667712 | 1.52871075373563 | 4.68809483867437 | Poland |
| 1990 | 75.46538291497 | 0.619298862250078 | 8.940499889599 | 66.524883025371 | Romania |
| 1991 | 56.09442283714 | 0.626795627433335 | 8.77815456388 | 47.31626827326 | Romania |
| 1992 | 55.79448400886 | 0.630475081501162 | 7.85285647592643 | 47.9416275329336 | Romania |
| 1993 | 58.12927718897 | 0.633801422118109 | 6.88008788402672 | 51.2491893049433 | Romania |
| 1994 | 65.59135703949 | 0.638371931788576 | 6.59088679368353 | 59.0004702458065 | Romania |
| 1995 | 53.70042150491 | 0.643718879089372 | 6.11093596569477 | 47.5894855392152 | Romania |
| 1996 | 57.44386628372 | 0.648104195492848 | 6.12763768133573 | 51.3162286023843 | Romania |
| 1997 | 54.64149625893 | 0.651225576178548 | 5.22448883141329 | 49.4170074275167 | Romania |
| 1998 | 53.50095012284 | 0.654927768840234 | 4.89202594787439 | 48.6089241749656 | Romania |
| 1999 | 46.94398518704 | 0.65954484605753 | 4.28820512399268 | 42.6557800630473 | Romania |
| 2000 | 47.97851964065 | 0.66529434615185 | 4.22966157661468 | 43.7488580640353 | Romania |
| 2001 | 41.09837874208 | 0.672020166603488 | 3.82899360440459 | 37.2693851376754 | Romania |
| 2002 | 36.2212077151 | 0.678297416747085 | 3.56270361757508 | 32.6585040975249 | Romania |
| 2003 | 32.99312591369 | 0.68407492928742 | 3.34284416956088 | 29.6502817441291 | Romania |
| 2004 | 30.87483398041 | 0.690417054639929 | 3.3882053392403 | 27.4866286411697 | Romania |
| 2005 | 26.40695604457 | 0.694998949594957 | 3.12006254166108 | 23.2868935029089 | Romania |
| 2006 | 22.12166869388 | 0.699490765699603 | 2.90178921626525 | 19.2198794776148 | Romania |
| 2007 | 15.96028522673 | 0.704523726685579 | 2.9258150562354 | 13.0344701704946 | Romania |
| 2008 | 15.75092490642 | 0.710757127721139 | 2.72689375754803 | 13.024031148872 | Romania |
| 2009 | 15.26841327126 | 0.717445122377497 | 2.61066123211431 | 12.6577520391457 | Romania |
| 2010 | 16.26527453993 | 0.723891348732225 | 2.37237831806667 | 13.8928962218633 | Romania |
| 2011 | 17.61361305315 | 0.729018272021873 | 2.24899893760382 | 15.3646141155462 | Romania |
| 2012 | 18.48912711921 | 0.73389248238628 | 1.88597140703719 | 16.6031557121728 | Romania |
| 2013 | 19.8847918785 | 0.737880472910291 | 2.08343185531877 | 17.8013600231812 | Romania |
| 2014 | 19.80685608049 | 0.740395466703106 | 1.79160060675815 | 18.0152554737318 | Romania |
| 2015 | 20.58047770824 | 0.743473529634312 | 1.91765613722642 | 18.6628215710136 | Romania |
| 2016 | 20.50235496992 | 0.747058709528536 | 1.79426402968317 | 18.7080909402368 | Romania |
| 2017 | 21.59009668825 | 0.751117366992228 | 1.78146616875143 | 19.8086305194986 | Romania |
| 2018 | 20.61503793162 | 0.755533333480467 | 1.70330906771873 | 18.9117288639013 | Romania |
| 2019 | 18.83502003302 | 0.760283970607593 | 1.74587905665487 | 17.0891409763651 | Romania |
| 2020 | 17.40123505202 | 0.764276444363666 | 1.834140925985 | 15.567094126035 | Romania |
| 2021 | 17.28354759247 | 0.768453864365965 | 1.76494709806014 | 15.5186004944099 | Romania |
| 1990 | 22.50192283099 | 0.630511019536119 | 8.3395816149454 | 14.1623412160446 | Serbia |
| 1991 | 21.22337099391 | 0.638016934037528 | 6.65305817306443 | 14.5703128208456 | Serbia |
| 1992 | 18.16502291596 | 0.641971993828041 | 6.19376907743881 | 11.9712538385212 | Serbia |
| 1993 | 17.95945104767 | 0.642096034424249 | 5.82597335243317 | 12.1334776952368 | Serbia |
| 1994 | 18.93258342329 | 0.642223220931048 | 6.3312432120406 | 12.6013402112494 | Serbia |
| 1995 | 17.94581214454 | 0.642739671389532 | 6.14371256359148 | 11.8020995809485 | Serbia |
| 1996 | 16.31800745478 | 0.644846417030162 | 5.96217018795484 | 10.3558372668252 | Serbia |
| 1997 | 14.9099893796 | 0.64716209445227 | 6.07920453032548 | 8.83078484927452 | Serbia |
| 1998 | 13.77055708245 | 0.65024598899818 | 5.10458258008388 | 8.66597450236612 | Serbia |
| 1999 | 11.72694937736 | 0.655534174135398 | 4.87672274595722 | 6.85022663140278 | Serbia |
| 2000 | 11.24947161076 | 0.660752800457895 | 4.7830102441139 | 6.4664613666461 | Serbia |
| 2001 | 11.45453847877 | 0.665407334806451 | 4.3204398691583 | 7.1340986096117 | Serbia |
| 2002 | 12.03148029194 | 0.671511071572143 | 3.81289956535776 | 8.21858072658224 | Serbia |
| 2003 | 13.19941971639 | 0.678334928718905 | 3.66806511227827 | 9.53135460411173 | Serbia |
| 2004 | 11.6233089842 | 0.686819841842618 | 3.35586159899339 | 8.26744738520661 | Serbia |
| 2005 | 10.30536001251 | 0.69609756790601 | 3.12645784880731 | 7.17890216370269 | Serbia |
| 2006 | 9.54045163669 | 0.704945018093173 | 2.89522903654117 | 6.64522260014883 | Serbia |
| 2007 | 8.83503530634 | 0.713242304525204 | 2.6749172576144 | 6.1601180487256 | Serbia |
| 2008 | 6.88790266081 | 0.721505305994409 | 2.70670853381216 | 4.18119412699784 | Serbia |
| 2009 | 8.1408018762 | 0.729965595799672 | 2.15082697841828 | 5.98997489778172 | Serbia |
| 2010 | 7.86845283548 | 0.740091311255812 | 1.93410426547705 | 5.93434857000296 | Serbia |
| 2011 | 7.74609756414 | 0.750072761758797 | 1.87895961661089 | 5.86713794752911 | Serbia |
| 2012 | 7.22122633421 | 0.75710508155 | 1.71504827157239 | 5.50617806263761 | Serbia |
| 2013 | 7.82858523772 | 0.762170908106883 | 1.71138449250326 | 6.11720074521674 | Serbia |
| 2014 | 7.97757778803 | 0.766043091132074 | 1.73630469942574 | 6.24127308860426 | Serbia |
| 2015 | 8.22915968584 | 0.769842222617687 | 1.7254037023718 | 6.5037559834682 | Serbia |
| 2016 | 8.33866837874 | 0.773654077278106 | 1.72575535795214 | 6.61291302078786 | Serbia |
| 2017 | 8.37785559699 | 0.777460384270565 | 1.74730047376167 | 6.63055512322833 | Serbia |
| 2018 | 7.95771821398 | 0.781494973790012 | 1.73133085509177 | 6.22638735888823 | Serbia |
| 2019 | 7.70298629891 | 0.785768300182747 | 1.5991337863066 | 6.1038525126034 | Serbia |
| 2020 | 6.89417669164 | 0.789183332686432 | 1.59594008631265 | 5.29823660532735 | Serbia |
| 2021 | 6.54665361902 | 0.792416293685483 | 1.58602116416514 | 4.96063245485486 | Serbia |
| 1990 | 9.28349737408 | 0.653853505137647 | 4.88621481216385 | 4.39728256191615 | Slovakia |
| 1991 | 9.51634074904 | 0.658731402179317 | 4.2321804585035 | 5.2841602905365 | Slovakia |
| 1992 | 8.97997149266 | 0.665830110587888 | 4.27247995973064 | 4.70749153292936 | Slovakia |
| 1993 | 8.14852620377 | 0.67596793706605 | 3.60689427224281 | 4.54163193152719 | Slovakia |
| 1994 | 7.65227113566 | 0.688136476595487 | 3.35415501658102 | 4.29811611907898 | Slovakia |
| 1995 | 7.18975838368 | 0.697476536273994 | 3.17396865673214 | 4.01578972694786 | Slovakia |
| 1996 | 6.95740891158 | 0.704405003157211 | 2.82102930169 | 4.13637960989 | Slovakia |
| 1997 | 6.77284100576 | 0.711513209633452 | 2.71590953535818 | 4.05693147040182 | Slovakia |
| 1998 | 6.78649600087 | 0.719058766219034 | 2.57734297691683 | 4.20915302395317 | Slovakia |
| 1999 | 6.56702675778 | 0.726144172630556 | 2.36641511574385 | 4.20061164203615 | Slovakia |
| 2000 | 6.38929542741 | 0.734046240313494 | 2.15813422472944 | 4.23116120268056 | Slovakia |
| 2001 | 5.72499363667 | 0.741392553570632 | 2.00147319482854 | 3.72352044184146 | Slovakia |
| 2002 | 5.96164696159 | 0.746791141582656 | 1.83835766071744 | 4.12328930087256 | Slovakia |
| 2003 | 6.47437068208 | 0.75116726952438 | 1.78671793627762 | 4.68765274580238 | Slovakia |
| 2004 | 6.70732897387 | 0.755836693705671 | 1.87549549231681 | 4.83183348155319 | Slovakia |
| 2005 | 6.8314777556 | 0.761724185107018 | 1.730528429098 | 5.100949326502 | Slovakia |
| 2006 | 6.70541681301 | 0.767673036224795 | 1.754447375638 | 4.950969437372 | Slovakia |
| 2007 | 6.77675772215 | 0.772914152845047 | 1.7850271829575 | 4.9917305391925 | Slovakia |
| 2008 | 7.08695082463 | 0.777197399879439 | 1.68149449798059 | 5.40545632664941 | Slovakia |
| 2009 | 7.55917954995 | 0.781052260265285 | 1.71717765883956 | 5.84200189111044 | Slovakia |
| 2010 | 7.66129008879 | 0.786254422327679 | 1.64618766147841 | 6.01510242731159 | Slovakia |
| 2011 | 7.53717745841 | 0.791465382423228 | 1.56066172013071 | 5.97651573827929 | Slovakia |
| 2012 | 7.7054621309 | 0.795456215611276 | 1.55349057304861 | 6.15197155785139 | Slovakia |
| 2013 | 7.76386793591 | 0.797903722940591 | 1.53746420338268 | 6.22640373252732 | Slovakia |
| 2014 | 8.18682936018 | 0.799618796308309 | 1.56655738132063 | 6.62027197885938 | Slovakia |
| 2015 | 8.85152010214 | 0.800394179928449 | 1.57117125556143 | 7.28034884657857 | Slovakia |
| 2016 | 9.36159990089 | 0.800878210489993 | 1.55762970833918 | 7.80397019255082 | Slovakia |
| 2017 | 9.62662649091 | 0.802277316569847 | 1.55652432880323 | 8.07010216210677 | Slovakia |
| 2018 | 9.67571488058 | 0.804357302109054 | 1.54252973408778 | 8.13318514649222 | Slovakia |
| 2019 | 9.18456028321 | 0.80644845685688 | 1.54651035002264 | 7.63804993318736 | Slovakia |
| 2020 | 8.61297246732 | 0.80830596588062 | 1.52365061176129 | 7.08932185555871 | Slovakia |
| 2021 | 8.19736583736 | 0.810610529921693 | 1.52020276622453 | 6.67716307113547 | Slovakia |
| 1990 | 4.31355067941 | 0.727463928368452 | 2.39706371714412 | 1.91648696226588 | Slovenia |
| 1991 | 4.15551955488 | 0.732666985968602 | 2.30448054658914 | 1.85103900829086 | Slovenia |
| 1992 | 4.23930865265 | 0.737391551733707 | 1.98864711939 | 2.25066153326 | Slovenia |
| 1993 | 3.83274782733 | 0.741774171511168 | 2.14231960457897 | 1.69042822275103 | Slovenia |
| 1994 | 3.70228882925 | 0.746234861332899 | 1.87848892990577 | 1.82379989934423 | Slovenia |
| 1995 | 3.09906497791 | 0.750994974989173 | 1.78928523651561 | 1.30977974139439 | Slovenia |
| 1996 | 2.83480723922 | 0.756348987126594 | 1.74677656389032 | 1.08803067532968 | Slovenia |
| 1997 | 2.83861033355 | 0.762275291907702 | 1.74319598716977 | 1.09541434638023 | Slovenia |
| 1998 | 2.71954997206 | 0.767728525502705 | 1.71406608730815 | 1.00548388475185 | Slovenia |
| 1999 | 2.37935942787 | 0.773236192288015 | 1.75484191293559 | 0.624517514934412 | Slovenia |
| 2000 | 2.41597136031 | 0.779578009745196 | 1.63823160759692 | 0.777739752713077 | Slovenia |
| 2001 | 2.30712553542 | 0.786240775635761 | 1.6159921215075 | 0.6911334139125 | Slovenia |
| 2002 | 2.34344527294 | 0.791805569443613 | 1.55949323702773 | 0.783952035912273 | Slovenia |
| 2003 | 2.2901467073 | 0.796371530737719 | 1.55547654963346 | 0.734670157666538 | Slovenia |
| 2004 | 2.24840358814 | 0.800962240122577 | 1.58616152846448 | 0.662242059675517 | Slovenia |
| 2005 | 2.25427440406 | 0.805601248185604 | 1.53644673960462 | 0.717827664455384 | Slovenia |
| 2006 | 2.18917649459 | 0.809708595222897 | 1.51395947869893 | 0.675217015891071 | Slovenia |
| 2007 | 2.20953929625 | 0.813006319197598 | 1.505971809601 | 0.703567486649 | Slovenia |
| 2008 | 2.31049664895 | 0.816347250277984 | 1.49894631754889 | 0.811550331401111 | Slovenia |
| 2009 | 2.5595268656 | 0.818878560380979 | 1.49314130685 | 1.06638555875 | Slovenia |
| 2010 | 2.93943536028 | 0.82117848598052 | 1.49984438072447 | 1.43959097955553 | Slovenia |
| 2011 | 3.46270084397 | 0.823023560855118 | 1.48912231455179 | 1.97357852941821 | Slovenia |
| 2012 | 3.69719600001 | 0.824336562677773 | 1.48509205222 | 2.21210394779 | Slovenia |
| 2013 | 4.07029872303 | 0.825826275074906 | 1.46260349823804 | 2.60769522479196 | Slovenia |
| 2014 | 4.03150354509 | 0.827799245827941 | 1.47786672147755 | 2.55363682361245 | Slovenia |
| 2015 | 4.20652947885 | 0.829837070735453 | 1.48659088250897 | 2.71993859634103 | Slovenia |
| 2016 | 4.27562923523 | 0.831367915132541 | 1.49623592246881 | 2.77939331276119 | Slovenia |
| 2017 | 4.24929517081 | 0.833038139449118 | 1.47417092922026 | 2.77512424158974 | Slovenia |
| 2018 | 4.25351165872 | 0.835472480610787 | 1.48620920245667 | 2.76730245626333 | Slovenia |
| 2019 | 4.58422516837 | 0.838266782297842 | 1.45734381421875 | 3.12688135415125 | Slovenia |
| 2020 | 4.20523854793 | 0.840373845574037 | 1.46278569254351 | 2.74245285538649 | Slovenia |
| 2021 | 4.04153490845 | 0.842430731281465 | 1.48723344574447 | 2.55430146270553 | Slovenia |
| 1990 | 59.93964749306 | 0.622446576329452 | 9.03087466160621 | 50.9087728314538 | Belarus |
| 1991 | 61.51860999278 | 0.627038266774523 | 8.72367302525328 | 52.7949369675267 | Belarus |
| 1992 | 62.66072289398 | 0.632891737136784 | 6.97504034686877 | 55.6856825471112 | Belarus |
| 1993 | 68.47611093838 | 0.639394169351269 | 6.79344834325582 | 61.6826625951242 | Belarus |
| 1994 | 75.71568212347 | 0.64465278058669 | 6.02986911288774 | 69.6858130105823 | Belarus |
| 1995 | 72.30015487989 | 0.648959503797064 | 4.91306868084423 | 67.3870861990458 | Belarus |
| 1996 | 61.77460088776 | 0.652855220750827 | 4.87197677258662 | 56.9026241151734 | Belarus |
| 1997 | 58.11485651334 | 0.655627296459504 | 5.15951414729239 | 52.9553423660476 | Belarus |
| 1998 | 63.28694266625 | 0.657246466657203 | 4.65633672652318 | 58.6306059397268 | Belarus |
| 1999 | 64.16069067384 | 0.660171009515746 | 4.34017569929194 | 59.820514974548 | Belarus |
| 2000 | 56.64512160414 | 0.664705822252137 | 4.06928811585333 | 52.5758334882867 | Belarus |
| 2001 | 50.98313688143 | 0.671333268854148 | 3.8214268627284 | 47.1617100187016 | Belarus |
| 2002 | 37.4728303042 | 0.678121782135605 | 3.605480975816 | 33.867349328384 | Belarus |
| 2003 | 32.50416999992 | 0.68486855613275 | 3.37773960651452 | 29.1264303934055 | Belarus |
| 2004 | 29.17389698117 | 0.692502135029653 | 3.15860253293015 | 26.0152944482398 | Belarus |
| 2005 | 25.53181428238 | 0.698985482904355 | 2.95237520514036 | 22.5794390772396 | Belarus |
| 2006 | 21.72321959005 | 0.705119102674127 | 2.86616151881924 | 18.8570580712308 | Belarus |
| 2007 | 19.81799259193 | 0.711496561875696 | 2.79049697986985 | 17.0274956120602 | Belarus |
| 2008 | 17.42103838721 | 0.719120871487284 | 2.4878683474525 | 14.9331700397575 | Belarus |
| 2009 | 16.38302928067 | 0.727292783613496 | 2.41361815366458 | 13.9694111270054 | Belarus |
| 2010 | 15.21792021954 | 0.734954626211817 | 2.01746784421338 | 13.2004523753266 | Belarus |
| 2011 | 14.92674101406 | 0.739504325946655 | 1.81941828706836 | 13.1073227269916 | Belarus |
| 2012 | 14.34295028177 | 0.743468837307909 | 1.82556052585136 | 12.5173897559186 | Belarus |
| 2013 | 13.87478224042 | 0.748529322885796 | 1.80129688020674 | 12.0734853602133 | Belarus |
| 2014 | 13.29653804916 | 0.753511479851413 | 1.79871802730109 | 11.4978200218589 | Belarus |
| 2015 | 12.73642242165 | 0.758205742923593 | 1.75635827771703 | 10.980064143933 | Belarus |
| 2016 | 12.92443577077 | 0.764328221802501 | 1.7333129241483 | 11.1911228466217 | Belarus |
| 2017 | 12.81129476537 | 0.771131465670885 | 1.735059122902 | 11.076235642468 | Belarus |
| 2018 | 11.87569498818 | 0.776568956673705 | 1.73906119095714 | 10.1366337972229 | Belarus |
| 2019 | 11.19251427015 | 0.780508018535969 | 1.6544878179608 | 9.5380264521892 | Belarus |
| 2020 | 10.36146265997 | 0.782381564892966 | 1.67790515155608 | 8.68355750841392 | Belarus |
| 2021 | 10.17334794228 | 0.784484710906639 | 1.66500793914843 | 8.50834000313157 | Belarus |
| 1990 | 51.69010073323 | 0.674967632130919 | 3.65187591647706 | 48.0382248167529 | Estonia |
| 1991 | 47.00924271877 | 0.685223089296634 | 3.36304717320984 | 43.6461955455602 | Estonia |
| 1992 | 47.8676029374 | 0.695586673779285 | 3.09362754181671 | 44.7739753955833 | Estonia |
| 1993 | 49.22940190497 | 0.703693149995024 | 2.90226199597121 | 46.3271399089988 | Estonia |
| 1994 | 49.72118932121 | 0.708456180899464 | 2.81046795646929 | 46.9107213647407 | Estonia |
| 1995 | 47.6500458758 | 0.713172725189506 | 2.66591832032481 | 44.9841275554752 | Estonia |
| 1996 | 38.99795449395 | 0.718813024217038 | 2.57619568890181 | 36.4217588050482 | Estonia |
| 1997 | 36.20802622048 | 0.724652439639785 | 2.41574019628291 | 33.7922860241971 | Estonia |
| 1998 | 36.07220776853 | 0.729027035230187 | 2.20368101324365 | 33.8685267552863 | Estonia |
| 1999 | 37.94175155116 | 0.733250616186121 | 2.17245291314646 | 35.7692986380135 | Estonia |
| 2000 | 33.23701780856 | 0.739713154351944 | 1.92107314439544 | 31.3159446641646 | Estonia |
| 2001 | 29.44803881128 | 0.746602207163017 | 1.82072999973104 | 27.627308811549 | Estonia |
| 2002 | 24.9456632729 | 0.752629898252246 | 1.81841820829039 | 23.1272450646096 | Estonia |
| 2003 | 23.69139407521 | 0.75781567757793 | 1.71984787976955 | 21.9715461954404 | Estonia |
| 2004 | 21.35719801967 | 0.763223727128029 | 1.73648636249105 | 19.6207116571789 | Estonia |
| 2005 | 19.97783660146 | 0.769112181926314 | 1.72677866020859 | 18.2510579412514 | Estonia |
| 2006 | 18.33024989809 | 0.774451841771662 | 1.74226443015043 | 16.5879854679396 | Estonia |
| 2007 | 17.75426872449 | 0.781074442846485 | 1.67033241105292 | 16.0839363134371 | Estonia |
| 2008 | 17.13799141644 | 0.788598873704278 | 1.60144741976475 | 15.5365439966752 | Estonia |
| 2009 | 16.14144461273 | 0.794728212699059 | 1.57679635634423 | 14.5646482563858 | Estonia |
| 2010 | 15.75287485584 | 0.800829717976428 | 1.53555168215072 | 14.2173231736893 | Estonia |
| 2011 | 14.58251214147 | 0.806633077522166 | 1.54290020739019 | 13.0396119340798 | Estonia |
| 2012 | 14.0305585721 | 0.811109357704526 | 1.53234023429085 | 12.4982183378092 | Estonia |
| 2013 | 13.45875280639 | 0.81488717677447 | 1.52643523241437 | 11.9323175739756 | Estonia |
| 2014 | 12.96739051432 | 0.818770732051979 | 1.49810422639161 | 11.4692862879284 | Estonia |
| 2015 | 12.79941876831 | 0.823221278492715 | 1.47945545247543 | 11.3199633158346 | Estonia |
| 2016 | 12.22766830612 | 0.827364360092018 | 1.49002541484125 | 10.7376428912787 | Estonia |
| 2017 | 11.92516036479 | 0.830975311283378 | 1.46869796303277 | 10.4564624017572 | Estonia |
| 2018 | 11.28412742099 | 0.834566296193434 | 1.47338098339304 | 9.81074643759696 | Estonia |
| 2019 | 10.83626010646 | 0.838573508963879 | 1.46718686936132 | 9.36907323709868 | Estonia |
| 2020 | 10.47827504944 | 0.841817139147138 | 1.47181469063621 | 9.00646035880379 | Estonia |
| 2021 | 10.27550606727 | 0.844917787360624 | 1.47220121639255 | 8.80330485087745 | Estonia |
| 1990 | 44.65446343854 | 0.680193638285329 | 3.62142869337067 | 41.0330347451693 | Latvia |
| 1991 | 48.59564513112 | 0.688117297290074 | 3.42361627398677 | 45.1720288571332 | Latvia |
| 1992 | 51.09239869666 | 0.696157035504877 | 3.06818723809108 | 48.0242114585689 | Latvia |
| 1993 | 54.18233525911 | 0.705249855159515 | 2.93753664501047 | 51.2447986140995 | Latvia |
| 1994 | 55.24686355218 | 0.712531352821191 | 2.68671691462312 | 52.5601466375569 | Latvia |
| 1995 | 52.3517038722 | 0.717639459211637 | 2.47302594264694 | 49.8786779295531 | Latvia |
| 1996 | 41.87483667981 | 0.721139537892551 | 2.51039475551551 | 39.3644419242945 | Latvia |
| 1997 | 36.98166916458 | 0.723746580428208 | 2.40657079486029 | 34.5750983697197 | Latvia |
| 1998 | 35.96085403874 | 0.725427335933284 | 2.41480360820431 | 33.5460504305357 | Latvia |
| 1999 | 35.28104755114 | 0.727219684959525 | 2.37826964698448 | 32.9027779041555 | Latvia |
| 2000 | 31.00349169453 | 0.730951448728138 | 2.20306070619947 | 28.8004309883305 | Latvia |
| 2001 | 28.87484087226 | 0.73629419637135 | 2.10784493491976 | 26.7669959373402 | Latvia |
| 2002 | 25.6933505542 | 0.741870440285041 | 1.83237091159311 | 23.8609796426069 | Latvia |
| 2003 | 22.72596964521 | 0.748168161846997 | 1.85784046608473 | 20.8681291791253 | Latvia |
| 2004 | 19.70482042696 | 0.755263211009698 | 1.81988366383646 | 17.8849367631235 | Latvia |
| 2005 | 18.03680536114 | 0.761929432669523 | 1.76685035360267 | 16.2699550075373 | Latvia |
| 2006 | 16.36930037825 | 0.768459928850117 | 1.7973171223459 | 14.5719832559041 | Latvia |
| 2007 | 15.70101559483 | 0.776481958669804 | 1.75693448217582 | 13.9440811126542 | Latvia |
| 2008 | 14.3024599835 | 0.786324573303851 | 1.6229219402366 | 12.6795380432634 | Latvia |
| 2009 | 13.30744829697 | 0.794504426648315 | 1.57063228234439 | 11.7368160146256 | Latvia |
| 2010 | 12.74770819495 | 0.798171594063957 | 1.5358045546336 | 11.2119036403164 | Latvia |
| 2011 | 12.57014899884 | 0.799080776427927 | 1.55684135783489 | 11.0133076410051 | Latvia |
| 2012 | 12.19406305042 | 0.801026473960894 | 1.5364352560386 | 10.6576277943814 | Latvia |
| 2013 | 11.91002440213 | 0.802660490277978 | 1.54101031669263 | 10.3690140854374 | Latvia |
| 2014 | 11.76569896339 | 0.803721486035489 | 1.53541299952548 | 10.2302859638645 | Latvia |
| 2015 | 11.59396919525 | 0.806985994927815 | 1.5231331344868 | 10.0708360607632 | Latvia |
| 2016 | 10.86536212395 | 0.811463590660605 | 1.50179642250759 | 9.36356570144241 | Latvia |
| 2017 | 10.13512967197 | 0.816923118079976 | 1.49619476814 | 8.63893490383 | Latvia |
| 2018 | 9.01153957913 | 0.821419809272627 | 1.49819907288811 | 7.51334050624189 | Latvia |
| 2019 | 8.38434674029 | 0.824781776742666 | 1.48985924150469 | 6.89448749878531 | Latvia |
| 2020 | 7.85887637194 | 0.827900448409988 | 1.478708955025 | 6.380167416915 | Latvia |
| 2021 | 7.59215141291 | 0.830663516299797 | 1.477235118672 | 6.114916294238 | Latvia |
| 1990 | 62.38430537856 | 0.668503937884641 | 3.83349986539941 | 58.5508055131606 | Lithuania |
| 1991 | 76.65902341283 | 0.673234120792409 | 3.72274492356453 | 72.9362784892655 | Lithuania |
| 1992 | 84.03497101593 | 0.680992879643758 | 3.55955367080386 | 80.4754173451261 | Lithuania |
| 1993 | 78.21749565198 | 0.687480621018131 | 3.35940558541937 | 74.8580900665606 | Lithuania |
| 1994 | 74.41938153905 | 0.69115165298429 | 3.17221763129099 | 71.247163907759 | Lithuania |
| 1995 | 65.4934699097 | 0.694734380082166 | 3.12895244175554 | 62.3645174679445 | Lithuania |
| 1996 | 52.93353363791 | 0.698591300871233 | 2.90277966104062 | 50.0307539768694 | Lithuania |
| 1997 | 47.62856122172 | 0.702840949666947 | 2.8309592062175 | 44.7976020155025 | Lithuania |
| 1998 | 45.04352402817 | 0.707468182196716 | 2.84644313996982 | 42.1970808882002 | Lithuania |
| 1999 | 46.86023269027 | 0.712834170940662 | 2.69541267551714 | 44.1648200147529 | Lithuania |
| 2000 | 41.1572742057 | 0.719896959838609 | 2.63515238002592 | 38.5221218256741 | Lithuania |
| 2001 | 35.46167170013 | 0.727133584877313 | 2.45887839593895 | 33.002793304191 | Lithuania |
| 2002 | 31.48733356064 | 0.733793397915696 | 2.04145735106623 | 29.4458762095738 | Lithuania |
| 2003 | 27.72267021749 | 0.741229600875632 | 1.87590099667582 | 25.8467692208142 | Lithuania |
| 2004 | 26.08079249031 | 0.748949762305515 | 1.83265166924667 | 24.2481408210633 | Lithuania |
| 2005 | 23.46867979909 | 0.756352359983789 | 1.81501529798687 | 21.6536645011031 | Lithuania |
| 2006 | 21.7376111414 | 0.763059124350272 | 1.74358951376262 | 19.9940216276374 | Lithuania |
| 2007 | 20.29435605511 | 0.770229638520692 | 1.77627062536424 | 18.5180854297458 | Lithuania |
| 2008 | 19.43773846192 | 0.778273711505288 | 1.68927757103775 | 17.7484608908822 | Lithuania |
| 2009 | 18.14135093239 | 0.785689739125479 | 1.63104218974072 | 16.5103087426493 | Lithuania |
| 2010 | 17.32996107117 | 0.793364681757828 | 1.5733744691525 | 15.7565866020175 | Lithuania |
| 2011 | 16.28142181631 | 0.800924994333688 | 1.53612445713113 | 14.7452973591789 | Lithuania |
| 2012 | 15.17922794253 | 0.8078270261595 | 1.52248180757381 | 13.6567461349562 | Lithuania |
| 2013 | 15.36914081287 | 0.813219722329104 | 1.54088408950143 | 13.8282567233686 | Lithuania |
| 2014 | 15.86007056888 | 0.817886376021861 | 1.51064219988778 | 14.3494283689922 | Lithuania |
| 2015 | 16.65485332853 | 0.823112629019743 | 1.49451876279708 | 15.1603345657329 | Lithuania |
| 2016 | 16.86351942695 | 0.829303695106582 | 1.4846368358303 | 15.3788825911197 | Lithuania |
| 2017 | 15.56711857404 | 0.8358717398516 | 1.47121267009164 | 14.0959059039484 | Lithuania |
| 2018 | 15.0339076534 | 0.841918910402675 | 1.47437442882574 | 13.5595332245743 | Lithuania |
| 2019 | 14.17365681557 | 0.847906338850069 | 1.45565376646643 | 12.7180030491036 | Lithuania |
| 2020 | 12.78526304793 | 0.85275238761541 | 1.4469253719178 | 11.3383376760122 | Lithuania |
| 2021 | 12.31922546293 | 0.856484049109405 | 1.43603056610297 | 10.883194896827 | Lithuania |
| 1990 | 274.11837635541 | 0.604251762377742 | 8.55722216605263 | 265.561154189357 | Republic of Moldova |
| 1991 | 228.00957634084 | 0.607270773865529 | 8.94841291476797 | 219.061163426072 | Republic of Moldova |
| 1992 | 225.75647940714 | 0.609664558440288 | 8.75986875873947 | 216.996610648401 | Republic of Moldova |
| 1993 | 238.34830550909 | 0.614152920432239 | 8.79227296504622 | 229.556032544044 | Republic of Moldova |
| 1994 | 256.38386160357 | 0.617603766824611 | 8.94555924024123 | 247.438302363329 | Republic of Moldova |
| 1995 | 224.70849478861 | 0.620951321121127 | 8.71984368899047 | 215.98865109962 | Republic of Moldova |
| 1996 | 199.82174427421 | 0.623113983844076 | 8.59110374830862 | 191.230640525901 | Republic of Moldova |
| 1997 | 176.35672161354 | 0.624370104753213 | 8.92211115958911 | 167.434610453951 | Republic of Moldova |
| 1998 | 159.08580559308 | 0.62411676120849 | 8.69565011022014 | 150.39015548286 | Republic of Moldova |
| 1999 | 150.01085043068 | 0.622834528688654 | 9.15689142100342 | 140.853959009677 | Republic of Moldova |
| 2000 | 138.04392294495 | 0.622004933502906 | 8.95320842889205 | 129.090714516058 | Republic of Moldova |
| 2001 | 129.61976384948 | 0.622936409943787 | 8.91087998302896 | 120.708883866451 | Republic of Moldova |
| 2002 | 115.02794192273 | 0.625874844526255 | 9.20057137706656 | 105.827370545663 | Republic of Moldova |
| 2003 | 105.58210899814 | 0.629985044767633 | 8.43800756597414 | 97.1441014321659 | Republic of Moldova |
| 2004 | 92.9846802243 | 0.635394821992876 | 7.22622611307717 | 85.7584541112228 | Republic of Moldova |
| 2005 | 79.19969090267 | 0.641530965095913 | 6.05633074001116 | 73.1433601626588 | Republic of Moldova |
| 2006 | 64.48160527297 | 0.647996263561811 | 6.15502391602923 | 58.3265813569408 | Republic of Moldova |
| 2007 | 58.30690387611 | 0.65421585059876 | 4.83833024828091 | 53.4685736278291 | Republic of Moldova |
| 2008 | 55.53043090075 | 0.661046597063232 | 4.30631932723712 | 51.2241115735129 | Republic of Moldova |
| 2009 | 54.3706900488 | 0.666284393801024 | 4.05593670930508 | 50.3147533394949 | Republic of Moldova |
| 2010 | 49.174027143 | 0.671989615961323 | 3.8699799083214 | 45.3040472346786 | Republic of Moldova |
| 2011 | 51.61085194995 | 0.678004136643775 | 3.62448882994814 | 47.9863631200019 | Republic of Moldova |
| 2012 | 44.51022205701 | 0.682979795888934 | 3.45459397190478 | 41.0556280851052 | Republic of Moldova |
| 2013 | 41.01686304636 | 0.688519174704066 | 3.33265200059648 | 37.6842110457635 | Republic of Moldova |
| 2014 | 38.4444433746 | 0.694104557276994 | 3.21139999500785 | 35.2330433795922 | Republic of Moldova |
| 2015 | 40.76708603388 | 0.699355166217478 | 2.94979022134563 | 37.8172958125344 | Republic of Moldova |
| 2016 | 38.80711469685 | 0.70514454200476 | 2.92186104378299 | 35.885253653067 | Republic of Moldova |
| 2017 | 40.8530815771 | 0.711206637139458 | 2.73767116828485 | 38.1154104088152 | Republic of Moldova |
| 2018 | 40.80138827956 | 0.717244426652362 | 2.57451235766952 | 38.2268759218905 | Republic of Moldova |
| 2019 | 37.72263276659 | 0.723011647628002 | 2.44496139405618 | 35.2776713725338 | Republic of Moldova |
| 2020 | 35.31477157827 | 0.727710540349217 | 2.43522669088456 | 32.8795448873854 | Republic of Moldova |
| 2021 | 33.38554052243 | 0.732214875427507 | 2.23810125027467 | 31.1474392721553 | Republic of Moldova |
| 1990 | 132.88351512363 | 0.671600578021767 | 3.76623428391221 | 129.117280839718 | Russian Federation |
| 1991 | 127.85162803291 | 0.680099211484661 | 3.59211948232292 | 124.259508550587 | Russian Federation |
| 1992 | 123.92126060042 | 0.688068021982768 | 3.39193875144062 | 120.529321848979 | Russian Federation |
| 1993 | 138.84368175371 | 0.692036194318927 | 3.145240021584 | 135.698441732126 | Russian Federation |
| 1994 | 140.07888240694 | 0.694144966076361 | 3.19923789736557 | 136.879644509574 | Russian Federation |
| 1995 | 140.09441235895 | 0.697854213747881 | 3.10882756417343 | 136.985584794777 | Russian Federation |
| 1996 | 124.62937628616 | 0.701468230734779 | 2.91935751183372 | 121.710018774326 | Russian Federation |
| 1997 | 117.68360522731 | 0.703752867289891 | 2.89314705191286 | 114.790458175397 | Russian Federation |
| 1998 | 118.20475029296 | 0.705198518088381 | 2.85921369203873 | 115.345536600921 | Russian Federation |
| 1999 | 129.9712431385 | 0.706863537909859 | 2.76718507515671 | 127.204058063343 | Russian Federation |
| 2000 | 119.16068450868 | 0.708643647769121 | 2.82061950060451 | 116.340065008075 | Russian Federation |
| 2001 | 110.80971621903 | 0.710570359001004 | 2.77024250100914 | 108.039473718021 | Russian Federation |
| 2002 | 95.29842436737 | 0.713460956547605 | 2.75327710498956 | 92.5451472623804 | Russian Federation |
| 2003 | 85.9897301469 | 0.718875116049301 | 2.52692348782225 | 83.4628066590777 | Russian Federation |
| 2004 | 72.35265356369 | 0.726305319497695 | 2.40091849727311 | 69.9517350664169 | Russian Federation |
| 2005 | 63.42656979074 | 0.733592776524904 | 2.26313944425738 | 61.1634303464826 | Russian Federation |
| 2006 | 54.43973513242 | 0.740064663588135 | 2.00694632579886 | 52.4327888066211 | Russian Federation |
| 2007 | 49.71341750833 | 0.746191512265979 | 1.87567350728403 | 47.837744001046 | Russian Federation |
| 2008 | 43.19005698168 | 0.752623243282606 | 1.72815407154877 | 41.4619029101312 | Russian Federation |
| 2009 | 38.48881950852 | 0.757960404689196 | 1.78549482411593 | 36.7033246844041 | Russian Federation |
| 2010 | 36.22809317871 | 0.763320116187813 | 1.76901406955724 | 34.4590791091528 | Russian Federation |
| 2011 | 34.64164890686 | 0.767633587927234 | 1.78639920084471 | 32.8552497060153 | Russian Federation |
| 2012 | 37.01122004861 | 0.77192038427212 | 1.75328927594936 | 35.2579307726606 | Russian Federation |
| 2013 | 33.8283088536 | 0.776526978961411 | 1.78435191562263 | 32.0439569379774 | Russian Federation |
| 2014 | 30.72126495582 | 0.780617524284297 | 1.67426855023907 | 29.0469964055809 | Russian Federation |
| 2015 | 26.22700562049 | 0.784913773964283 | 1.66670855679806 | 24.5602970636919 | Russian Federation |
| 2016 | 23.72112059417 | 0.790330149439335 | 1.57498482296188 | 22.1461357712081 | Russian Federation |
| 2017 | 21.01236906873 | 0.79566948446832 | 1.56555085568708 | 19.4468182130429 | Russian Federation |
| 2018 | 17.99763687866 | 0.800108144129611 | 1.55115499666722 | 16.4464818819928 | Russian Federation |
| 2019 | 16.21088761083 | 0.803658201264112 | 1.52130474785238 | 14.6895828629776 | Russian Federation |
| 2020 | 15.65017096017 | 0.806011474298182 | 1.54516428822193 | 14.1050066719481 | Russian Federation |
| 2021 | 15.41912718777 | 0.808536004906566 | 1.51774772394625 | 13.9013794638237 | Russian Federation |
| 1990 | 64.30132120127 | 0.647461465528387 | 6.30527749360911 | 57.9960437076609 | Ukraine |
| 1991 | 64.02667085502 | 0.651950021194653 | 5.03837247247186 | 58.9882983825481 | Ukraine |
| 1992 | 69.07704839729 | 0.657600333165733 | 5.09980135775526 | 63.9772470395347 | Ukraine |
| 1993 | 78.13282870131 | 0.66286740459174 | 4.32122966765615 | 73.8115990336538 | Ukraine |
| 1994 | 103.28457798914 | 0.665506374866164 | 4.29210226187951 | 98.9924757272605 | Ukraine |
| 1995 | 90.26225745906 | 0.666950166648749 | 3.85974787769133 | 86.4025095813687 | Ukraine |
| 1996 | 80.69531801674 | 0.668104744367524 | 3.87287421477485 | 76.8224438019652 | Ukraine |
| 1997 | 76.35813071616 | 0.669207845755283 | 3.82956467919708 | 72.5285660369629 | Ukraine |
| 1998 | 63.9517964204 | 0.670322300548544 | 3.83713338419208 | 60.1146630362079 | Ukraine |
| 1999 | 57.64430822342 | 0.670642905451183 | 3.86267254482952 | 53.7816356785905 | Ukraine |
| 2000 | 46.82916235081 | 0.671095000943321 | 3.84882269763352 | 42.9803396531765 | Ukraine |
| 2001 | 40.05514625353 | 0.672467819796801 | 3.83608136934792 | 36.2190648841821 | Ukraine |
| 2002 | 37.21742989684 | 0.674543220896083 | 3.58808802523034 | 33.6293418716097 | Ukraine |
| 2003 | 31.44300924892 | 0.678614636476308 | 3.60876334680538 | 27.8342459021146 | Ukraine |
| 2004 | 26.86205870562 | 0.685380936146257 | 3.40270824679238 | 23.4593504588276 | Ukraine |
| 2005 | 24.59702601398 | 0.692023300673985 | 3.13058684082415 | 21.4664391731558 | Ukraine |
| 2006 | 22.47839561611 | 0.698093382536473 | 3.18245654094113 | 19.2959390751689 | Ukraine |
| 2007 | 20.20840616928 | 0.704202307705725 | 2.97232504070806 | 17.2360811285719 | Ukraine |
| 2008 | 20.65177831993 | 0.710980183909559 | 2.68792703662712 | 17.9638512833029 | Ukraine |
| 2009 | 17.40896537467 | 0.716185465482395 | 2.59719121954365 | 14.8117741551263 | Ukraine |
| 2010 | 16.22059437007 | 0.72101987022281 | 2.45129672937638 | 13.7692976406936 | Ukraine |
| 2011 | 15.4383315538 | 0.724891957373533 | 2.40896798304585 | 13.0293635707542 | Ukraine |
| 2012 | 14.63010798613 | 0.728817199844111 | 2.19435853522255 | 12.4357494509075 | Ukraine |
| 2013 | 14.0404393036 | 0.734033572821072 | 2.11042555326444 | 11.9300137503356 | Ukraine |
| 2014 | 10.62658451845 | 0.739883556959807 | 1.82299175816684 | 8.80359276028316 | Ukraine |
| 2015 | 11.57774424294 | 0.743790863159826 | 1.87702948532415 | 9.70071475761585 | Ukraine |
| 2016 | 11.86098199364 | 0.746857988843031 | 1.7843454456542 | 10.0766365479858 | Ukraine |
| 2017 | 11.37238947874 | 0.750593324392773 | 1.93582074362378 | 9.43656873511622 | Ukraine |
| 2018 | 10.40828425302 | 0.754451727274318 | 1.75799471892917 | 8.65028953409083 | Ukraine |
| 2019 | 9.96468529061 | 0.757980081219807 | 1.81901414237458 | 8.14567114823542 | Ukraine |
| 2020 | 10.24686783408 | 0.759929872218543 | 1.72066593775 | 8.52620189633 | Ukraine |
| 2021 | 9.87701089655 | 0.760773912846782 | 1.806261237755 | 8.070749658795 | Ukraine |
| 1990 | 3.84959248162 | 0.66608191698766 | 3.84485256189156 | 0.00473991972844434 | Brunei Darussalam |
| 1991 | 3.85199524685 | 0.671233831388631 | 3.75353741553879 | 0.0984578313112121 | Brunei Darussalam |
| 1992 | 3.80085237932 | 0.676702526223706 | 3.58063870937214 | 0.220213669947857 | Brunei Darussalam |
| 1993 | 3.74749744603 | 0.682175439911044 | 3.52449126517611 | 0.223006180853889 | Brunei Darussalam |
| 1994 | 3.67463474014 | 0.68739409348098 | 3.30901636444176 | 0.365618375698236 | Brunei Darussalam |
| 1995 | 3.57696400354 | 0.692471156008533 | 3.10324112729533 | 0.473722876244667 | Brunei Darussalam |
| 1996 | 3.4838981379 | 0.697602233240377 | 3.12213893296488 | 0.361759204935122 | Brunei Darussalam |
| 1997 | 3.41041671333 | 0.702944386203433 | 2.81252853678062 | 0.597888176549375 | Brunei Darussalam |
| 1998 | 3.3437051985 | 0.708630119469791 | 2.57211995834464 | 0.771585240155357 | Brunei Darussalam |
| 1999 | 3.30099482411 | 0.714851237118356 | 2.59531314240686 | 0.705681681703143 | Brunei Darussalam |
| 2000 | 3.35130845249 | 0.72155249267874 | 2.66098358932036 | 0.690324863169643 | Brunei Darussalam |
| 2001 | 3.36388116604 | 0.728535069338112 | 2.27709629317711 | 1.08678487286289 | Brunei Darussalam |
| 2002 | 3.47437741225 | 0.735343916434566 | 2.068918681273 | 1.405458730977 | Brunei Darussalam |
| 2003 | 3.57804823944 | 0.741578091315293 | 1.90840126494971 | 1.66964697449029 | Brunei Darussalam |
| 2004 | 3.61475077953 | 0.747311714682537 | 1.70459929754125 | 1.91015148198875 | Brunei Darussalam |
| 2005 | 3.66806492673 | 0.752481792632817 | 1.80892556933222 | 1.85913935739778 | Brunei Darussalam |
| 2006 | 3.6929991445 | 0.75731865511743 | 1.78905667139585 | 1.90394247310415 | Brunei Darussalam |
| 2007 | 3.75341551232 | 0.761643128654341 | 1.74218578835871 | 2.01122972396129 | Brunei Darussalam |
| 2008 | 3.82542552531 | 0.765714260433754 | 1.84579579731677 | 1.97962972799323 | Brunei Darussalam |
| 2009 | 3.88309029978 | 0.769601681624648 | 1.74534141415667 | 2.13774888562333 | Brunei Darussalam |
| 2010 | 3.94323270525 | 0.773219221249055 | 1.6894785851225 | 2.2537541201275 | Brunei Darussalam |
| 2011 | 4.06178743379 | 0.776631893191597 | 1.7369429572181 | 2.32484447657191 | Brunei Darussalam |
| 2012 | 4.10577360816 | 0.780216551684601 | 1.70095925251432 | 2.40481435564568 | Brunei Darussalam |
| 2013 | 4.08469810871 | 0.784017040989581 | 1.69935157964972 | 2.38534652906028 | Brunei Darussalam |
| 2014 | 4.03383878752 | 0.787733289361499 | 1.58579264458122 | 2.44804614293878 | Brunei Darussalam |
| 2015 | 3.97719155199 | 0.791367566956237 | 1.56950666603 | 2.40768488596 | Brunei Darussalam |
| 2016 | 3.89144304548 | 0.794980772397229 | 1.58002058255727 | 2.31142246292273 | Brunei Darussalam |
| 2017 | 3.84505729759 | 0.798309936375838 | 1.55492894290951 | 2.29012835468049 | Brunei Darussalam |
| 2018 | 3.76825201477 | 0.801436553226482 | 1.52642796508533 | 2.24182404968467 | Brunei Darussalam |
| 2019 | 3.69351003252 | 0.804499388246421 | 1.538210772015 | 2.155299260505 | Brunei Darussalam |
| 2020 | 3.56447952715 | 0.80742409075008 | 1.51428605924118 | 2.05019346790882 | Brunei Darussalam |
| 2021 | 3.40686804205 | 0.810234366947283 | 1.54911719329571 | 1.85775084875429 | Brunei Darussalam |
| 1990 | 13.83498449317 | 0.790253515528297 | 1.57418515237297 | 12.260799340797 | Japan |
| 1991 | 14.03445606208 | 0.794616189497547 | 1.59937333340111 | 12.4350827286789 | Japan |
| 1992 | 14.1432438701 | 0.799156224748283 | 1.54101619375712 | 12.6022276763429 | Japan |
| 1993 | 14.19535109592 | 0.802972994199584 | 1.55018985508952 | 12.6451612408305 | Japan |
| 1994 | 14.69792698114 | 0.806689483098495 | 1.54676291487329 | 13.1511640662667 | Japan |
| 1995 | 14.5213160406 | 0.810510771484257 | 1.52240674363119 | 12.9989092969688 | Japan |
| 1996 | 14.33211151379 | 0.813870745535327 | 1.48936423314344 | 12.8427472806466 | Japan |
| 1997 | 14.92858656147 | 0.816675782376649 | 1.50175550403298 | 13.426831057437 | Japan |
| 1998 | 15.26509147938 | 0.819016201504856 | 1.49812883298026 | 13.7669626463997 | Japan |
| 1999 | 14.74795497218 | 0.82106663705133 | 1.49659329118281 | 13.2513616809972 | Japan |
| 2000 | 14.75739180521 | 0.822938998623378 | 1.48243879779804 | 13.274953007412 | Japan |
| 2001 | 14.75203307111 | 0.824723241254759 | 1.47816649652868 | 13.2738665745813 | Japan |
| 2002 | 15.0341687197 | 0.82693677356819 | 1.50031246469263 | 13.5338562550074 | Japan |
| 2003 | 15.44549891288 | 0.829392223273124 | 1.48725437960453 | 13.9582445332755 | Japan |
| 2004 | 15.65206473698 | 0.832037381275171 | 1.49084592566846 | 14.1612188113115 | Japan |
| 2005 | 16.65623231445 | 0.834288685362289 | 1.47636791971786 | 15.1798643947321 | Japan |
| 2006 | 17.49539291703 | 0.836222264006662 | 1.47476290888722 | 16.0206300081428 | Japan |
| 2007 | 17.44689319054 | 0.83829415979033 | 1.47814858992123 | 15.9687446006188 | Japan |
| 2008 | 16.9059984196 | 0.840301217168455 | 1.47863410026841 | 15.4273643193316 | Japan |
| 2009 | 16.03391971912 | 0.842133664144895 | 1.46313086757386 | 14.5707888515461 | Japan |
| 2010 | 16.20987851404 | 0.844361810771781 | 1.46898478124458 | 14.7408937327954 | Japan |
| 2011 | 16.24494137706 | 0.846750266822805 | 1.45925409858329 | 14.7856872784767 | Japan |
| 2012 | 16.5290827468 | 0.849061489406189 | 1.45654622207361 | 15.0725365247264 | Japan |
| 2013 | 15.67315022334 | 0.851356993626047 | 1.43691708797408 | 14.2362331353659 | Japan |
| 2014 | 14.85388424178 | 0.85369274935716 | 1.45138304300226 | 13.4025011987777 | Japan |
| 2015 | 14.46135520193 | 0.856097498738686 | 1.41920634546774 | 13.0421488564623 | Japan |
| 2016 | 14.224023986 | 0.858698173210648 | 1.42324814094984 | 12.8007758450502 | Japan |
| 2017 | 13.7744668451 | 0.861393308858287 | 1.41154612754615 | 12.3629207175538 | Japan |
| 2018 | 13.53392117398 | 0.864237136659234 | 1.44129892080448 | 12.0926222531755 | Japan |
| 2019 | 12.67001437454 | 0.867148183486851 | 1.40963305212019 | 11.2603813224198 | Japan |
| 2020 | 12.03977391237 | 0.869106879336459 | 1.40349069489803 | 10.636283217472 | Japan |
| 2021 | 12.43991457842 | 0.871241812572128 | 1.4100959243758 | 11.0298186540442 | Japan |
| 1990 | 3.61228425453 | 0.692329306595283 | 3.08372856375237 | 0.528555690777632 | Republic of Korea |
| 1991 | 3.33637426397 | 0.702631128494537 | 2.84581614120857 | 0.490558122761429 | Republic of Korea |
| 1992 | 3.10875453914 | 0.712440841630159 | 2.69085830558475 | 0.41789623355525 | Republic of Korea |
| 1993 | 2.85415477719 | 0.722286739631754 | 2.47951712213036 | 0.374637655059643 | Republic of Korea |
| 1994 | 2.63310501246 | 0.732197906155207 | 2.12656944507634 | 0.506535567383659 | Republic of Korea |
| 1995 | 2.42487637418 | 0.741940029590316 | 1.922787355874 | 0.502089018306 | Republic of Korea |
| 1996 | 2.23883370258 | 0.751553240476733 | 1.76042962549875 | 0.47840407708125 | Republic of Korea |
| 1997 | 2.04886630987 | 0.760591683388116 | 1.64668766989667 | 0.402178639973334 | Republic of Korea |
| 1998 | 1.87921640621 | 0.76828188532262 | 1.65058628367263 | 0.228630122537368 | Republic of Korea |
| 1999 | 1.73892794219 | 0.776773436337718 | 1.62420241252577 | 0.114725529664231 | Republic of Korea |
| 2000 | 1.73093843369 | 0.785428821409438 | 1.65629851361609 | 0.074639920073913 | Republic of Korea |
| 2001 | 1.72657041508 | 0.793496280690955 | 1.55553229823484 | 0.171038116845161 | Republic of Korea |
| 2002 | 1.69112592145 | 0.801024295470106 | 1.54217072956423 | 0.148955191885769 | Republic of Korea |
| 2003 | 1.66899229864 | 0.807687785876648 | 1.54639161426192 | 0.122600684378077 | Republic of Korea |
| 2004 | 1.63380692013 | 0.813720926701095 | 1.51092352209417 | 0.122883398035833 | Republic of Korea |
| 2005 | 1.58172311423 | 0.819093399889013 | 1.494398029519 | 0.0873250847109999 | Republic of Korea |
| 2006 | 1.57312192139 | 0.824179820431794 | 1.48261586158412 | 0.0905060598058824 | Republic of Korea |
| 2007 | 1.55385858505 | 0.82912795326169 | 1.46329525970571 | 0.0905633253442857 | Republic of Korea |
| 2008 | 1.49081077168 | 0.833894626872214 | 1.46173500668231 | 0.0290757649976923 | Republic of Korea |
| 2009 | 1.41562061857 | 0.838108330520764 | 1.41562061857 | 0 | Republic of Korea |
| 2010 | 1.37087015213 | 0.84251142929369 | 1.37087015213 | 0 | Republic of Korea |
| 2011 | 1.31931875139 | 0.846913228744834 | 1.31931875139 | 0 | Republic of Korea |
| 2012 | 1.28082904243 | 0.851140262773848 | 1.28082904243 | 0 | Republic of Korea |
| 2013 | 1.23662806315 | 0.855441000170575 | 1.209688198545 | 0.0269398646050001 | Republic of Korea |
| 2014 | 1.19696785334 | 0.859665567500037 | 1.19696785334 | 0 | Republic of Korea |
| 2015 | 1.1585971537 | 0.863907408481354 | 1.1585971537 | 0 | Republic of Korea |
| 2016 | 1.13834052455 | 0.868317824083966 | 1.13834052455 | 0 | Republic of Korea |
| 2017 | 1.12476296301 | 0.87261776393019 | 1.12476296301 | 0 | Republic of Korea |
| 2018 | 1.13585142839 | 0.876823316849569 | 1.13585142839 | 0 | Republic of Korea |
| 2019 | 1.1719026818 | 0.88073150966869 | 1.1719026818 | 0 | Republic of Korea |
| 2020 | 1.10289929242 | 0.883717569055359 | 1.10289929242 | 0 | Republic of Korea |
| 1990 | 5.84082725062 | 0.686404444349548 | 3.41585870152395 | 2.42496854909605 | Singapore |
| 1991 | 4.53135368431 | 0.695055557646282 | 3.15926003504786 | 1.37209364926214 | Singapore |
| 1992 | 3.70603934281 | 0.703427834229016 | 3.03459143860359 | 0.67144790420641 | Singapore |
| 1993 | 3.19379345431 | 0.712315203759158 | 2.69107350459065 | 0.502719949719355 | Singapore |
| 1994 | 3.08675325569 | 0.721256796938456 | 2.63460354716788 | 0.452149708522121 | Singapore |
| 1995 | 2.65058790305 | 0.729982231248165 | 2.19577339775571 | 0.454814505294286 | Singapore |
| 1996 | 2.40667911981 | 0.739028234741754 | 1.81945220456622 | 0.587226915243784 | Singapore |
| 1997 | 2.42810362158 | 0.747571897605582 | 1.75194762646609 | 0.676155995113913 | Singapore |
| 1998 | 3.11495501562 | 0.754873203577067 | 1.71165167782257 | 1.40330333779743 | Singapore |
| 1999 | 2.33816979149 | 0.760831136110222 | 1.71267053692815 | 0.625499254561852 | Singapore |
| 2000 | 2.3800871589 | 0.767717370394546 | 1.74700409710636 | 0.633083061793636 | Singapore |
| 2001 | 1.96752824797 | 0.774294656621655 | 1.67178159467316 | 0.295746653296842 | Singapore |
| 2002 | 1.89236285099 | 0.77973576984914 | 1.72722794233 | 0.16513490866 | Singapore |
| 2003 | 1.76117384802 | 0.784165555316375 | 1.59043721205476 | 0.170736635965238 | Singapore |
| 2004 | 1.68767223366 | 0.789852773877056 | 1.5945751356856 | 0.0930970979744001 | Singapore |
| 2005 | 1.81486707383 | 0.796455750753104 | 1.52107675700786 | 0.293790316822143 | Singapore |
| 2006 | 2.08926776876 | 0.803342694397764 | 1.55098910001913 | 0.53827866874087 | Singapore |
| 2007 | 2.1584240853 | 0.810625304101189 | 1.55900432269192 | 0.599419762608077 | Singapore |
| 2008 | 2.26994082845 | 0.818124162030734 | 1.52133379614722 | 0.748607032302778 | Singapore |
| 2009 | 2.43603235418 | 0.824251515940882 | 1.49060957647579 | 0.945422777704211 | Singapore |
| 2010 | 2.49588354509 | 0.830222159553543 | 1.47428617263094 | 1.02159737245906 | Singapore |
| 2011 | 2.40646463436 | 0.833829607920547 | 1.47799340499926 | 0.92847122936074 | Singapore |
| 2012 | 2.28026172301 | 0.837391806757054 | 1.47294344184586 | 0.807318281164138 | Singapore |
| 2013 | 1.99650164659 | 0.840420177683154 | 1.47626339459233 | 0.520238251997667 | Singapore |
| 2014 | 1.62139564554 | 0.842894537933952 | 1.45441392646 | 0.16698171908 | Singapore |
| 2015 | 1.54783205614 | 0.84540185703118 | 1.46159751501893 | 0.0862345411210714 | Singapore |
| 2016 | 1.58321440617 | 0.847596998735509 | 1.45879520784231 | 0.124419198327692 | Singapore |
| 2017 | 1.41114144224 | 0.849051517606326 | 1.41114144224 | 0 | Singapore |
| 2018 | 1.43441585308 | 0.850762728725121 | 1.418730498985 | 0.0156853540949999 | Singapore |
| 2019 | 1.35450201317 | 0.852497337757222 | 1.35450201317 | 0 | Singapore |
| 2020 | 1.18274833394 | 0.854296009592945 | 1.18274833394 | 0 | Singapore |
| 2021 | 1.24406730551 | 0.856097766112729 | 1.24406730551 | 0 | Singapore |
| 1990 | 8.8882567599 | 0.725982519157417 | 2.35516441071107 | 6.53309234918893 | Australia |
| 1991 | 7.63045349241 | 0.729759858781312 | 2.2074296111195 | 5.4230238812905 | Australia |
| 1992 | 6.62894352716 | 0.73390140434283 | 2.09673769327281 | 4.53220583388719 | Australia |
| 1993 | 5.28084621753 | 0.738374743085692 | 2.02412090354902 | 3.25672531398098 | Australia |
| 1994 | 4.77057390537 | 0.742846571880912 | 1.84247099848545 | 2.92810290688455 | Australia |
| 1995 | 4.38283980983 | 0.747426824081883 | 1.89454046738677 | 2.48829934244323 | Australia |
| 1996 | 4.07528872377 | 0.751991736090285 | 1.80102661789385 | 2.27426210587615 | Australia |
| 1997 | 3.31475440407 | 0.756633901347637 | 1.69022354996 | 1.62453085411 | Australia |
| 1998 | 2.72212027887 | 0.761133265356106 | 1.80577063501475 | 0.91634964385525 | Australia |
| 1999 | 3.06328849125 | 0.765697505930953 | 1.74221376342469 | 1.32107472782531 | Australia |
| 2000 | 2.89283236711 | 0.770223843075912 | 1.731939790722 | 1.160892576388 | Australia |
| 2001 | 3.08737398494 | 0.774662787384155 | 1.7092012191059 | 1.3781727658341 | Australia |
| 2002 | 3.42966492796 | 0.779360940006122 | 1.73837065151828 | 1.69129427644172 | Australia |
| 2003 | 3.90115398974 | 0.783600180802991 | 1.6443599843575 | 2.2567940053825 | Australia |
| 2004 | 4.44703362362 | 0.787016543130049 | 1.66438241039077 | 2.78265121322923 | Australia |
| 2005 | 4.56958107595 | 0.789615521287164 | 1.62999965652447 | 2.93958141942553 | Australia |
| 2006 | 4.91372708262 | 0.791155687742942 | 1.60599989362684 | 3.30772718899316 | Australia |
| 2007 | 4.92964181581 | 0.792532682754436 | 1.59073720719717 | 3.33890460861283 | Australia |
| 2008 | 4.50981175489 | 0.795292900277512 | 1.58652223736844 | 2.92328951752156 | Australia |
| 2009 | 4.91232425988 | 0.799174373217167 | 1.54003273959303 | 3.37229152028697 | Australia |
| 2010 | 4.44365191862 | 0.803561543809643 | 1.51004374265763 | 2.93360817596237 | Australia |
| 2011 | 4.78211712302 | 0.807520219913819 | 1.54290418521857 | 3.23921293780143 | Australia |
| 2012 | 4.7270948642 | 0.811644461488936 | 1.52623824454383 | 3.20085661965617 | Australia |
| 2013 | 4.26682067729 | 0.816453661448137 | 1.52472357661028 | 2.74209710067972 | Australia |
| 2014 | 4.33446944299 | 0.820840850321621 | 1.50176901556364 | 2.83270042742636 | Australia |
| 2015 | 4.09111013751 | 0.824860715173108 | 1.48904921749533 | 2.60206092001467 | Australia |
| 2016 | 3.68954183898 | 0.828559943182664 | 1.47248870745257 | 2.21705313152743 | Australia |
| 2017 | 3.5468465413 | 0.831798265550707 | 1.48505707827568 | 2.06178946302432 | Australia |
| 2018 | 3.32181151427 | 0.835354624190932 | 1.470408781262 | 1.851402733008 | Australia |
| 2019 | 3.30230240359 | 0.839317426059217 | 1.4662234342975 | 1.8360789692925 | Australia |
| 2020 | 3.01975298533 | 0.842051314482691 | 1.46579617808081 | 1.55395680724919 | Australia |
| 2021 | 2.47921834529 | 0.844252813727575 | 1.47031309983353 | 1.00890524545647 | Australia |
| 1990 | 14.98181478764 | 0.752321654884833 | 1.75740766852381 | 13.2244071191162 | New Zealand |
| 1991 | 12.27718545891 | 0.755616090554557 | 1.70706121906655 | 10.5701242398435 | New Zealand |
| 1992 | 9.74905586118 | 0.759461372351944 | 1.75514415383521 | 7.99391170734479 | New Zealand |
| 1993 | 9.0845515317 | 0.763634604405382 | 1.76156703907646 | 7.32298449262354 | New Zealand |
| 1994 | 9.14982766122 | 0.767118657876439 | 1.75301148057389 | 7.39681618064611 | New Zealand |
| 1995 | 8.74998511815 | 0.769998036424639 | 1.79636172272571 | 6.95362339542429 | New Zealand |
| 1996 | 8.88396418697 | 0.773575283251135 | 1.72555927118805 | 7.15840491578195 | New Zealand |
| 1997 | 9.87242385878 | 0.778205776484177 | 1.67053921115038 | 8.20188464762962 | New Zealand |
| 1998 | 7.86146801387 | 0.78173075719617 | 1.6980944002122 | 6.1633736136578 | New Zealand |
| 1999 | 8.55492078797 | 0.784428637621383 | 1.71935488936717 | 6.83556589860283 | New Zealand |
| 2000 | 9.63705451178 | 0.787659557491679 | 1.65263678527292 | 7.98441772650708 | New Zealand |
| 2001 | 8.46219946368 | 0.791426079291696 | 1.5828429171819 | 6.8793565464981 | New Zealand |
| 2002 | 8.20110234242 | 0.794519590986489 | 1.56138032431104 | 6.63972201810896 | New Zealand |
| 2003 | 7.66328738381 | 0.796286883438684 | 1.53256372809386 | 6.13072365571614 | New Zealand |
| 2004 | 8.25279312893 | 0.798177476508371 | 1.55716062759887 | 6.69563250133113 | New Zealand |
| 2005 | 8.75673287739 | 0.799107294338124 | 1.55529392336136 | 7.20143895402864 | New Zealand |
| 2006 | 10.60412733061 | 0.798456865675295 | 1.55787634270625 | 9.04625098790375 | New Zealand |
| 2007 | 15.20480655784 | 0.798447615942533 | 1.55416814422913 | 13.6506384136109 | New Zealand |
| 2008 | 18.02913664487 | 0.799669513648105 | 1.54299690726092 | 16.4861397376091 | New Zealand |
| 2009 | 20.85309836554 | 0.801727534577573 | 1.53889171480155 | 19.3142066507384 | New Zealand |
| 2010 | 19.58744526801 | 0.805133184751295 | 1.51450829926757 | 18.0729369687424 | New Zealand |
| 2011 | 19.39136223455 | 0.809760351959867 | 1.53017581837807 | 17.8611864161719 | New Zealand |
| 2012 | 18.99386679967 | 0.814957563823326 | 1.50639912649797 | 17.487467673172 | New Zealand |
| 2013 | 16.44249787143 | 0.820145989650422 | 1.48403620322208 | 14.9584616682079 | New Zealand |
| 2014 | 17.8336220783 | 0.823908943687562 | 1.48530892881041 | 16.3483131494896 | New Zealand |
| 2015 | 14.19491817328 | 0.82746249334817 | 1.4790615742727 | 12.7158565990073 | New Zealand |
| 2016 | 12.12489650429 | 0.831843966678668 | 1.48260511515 | 10.64229138914 | New Zealand |
| 2017 | 12.81019123516 | 0.836301794613403 | 1.47268972256182 | 11.3375015125982 | New Zealand |
| 2018 | 11.21500003361 | 0.840597863184845 | 1.45718049896469 | 9.7578195346453 | New Zealand |
| 2019 | 11.61739940721 | 0.844533208807291 | 1.46475855444407 | 10.1526408527659 | New Zealand |
| 2020 | 9.69477883863 | 0.847398736836909 | 1.46440608531083 | 8.23037275331917 | New Zealand |
| 2021 | 10.05161517538 | 0.849442498970719 | 1.45798822942309 | 8.59362694595691 | New Zealand |
| 1990 | 5.30850305231 | 0.761463880346533 | 1.73276594879808 | 3.57573710351192 | Andorra |
| 1991 | 5.44061917592 | 0.764709071138693 | 1.79463619646395 | 3.64598297945605 | Andorra |
| 1992 | 5.64307416434 | 0.767188282487657 | 1.72894367968978 | 3.91413048465022 | Andorra |
| 1993 | 5.69938570978 | 0.768953827567811 | 1.76186262251535 | 3.93752308726465 | Andorra |
| 1994 | 5.75892479801 | 0.77002376454004 | 1.6522176416982 | 4.1067071563118 | Andorra |
| 1995 | 5.88971927103 | 0.77098868338163 | 1.6758064669 | 4.21391280413 | Andorra |
| 1996 | 6.03114771229 | 0.772403470644367 | 1.67604828713867 | 4.35509942515133 | Andorra |
| 1997 | 6.24762850746 | 0.774580970737089 | 1.67497685895946 | 4.57265164850054 | Andorra |
| 1998 | 6.46027371478 | 0.776972577800595 | 1.670438647636 | 4.789835067144 | Andorra |
| 1999 | 6.61287044466 | 0.779629634395052 | 1.64760140280286 | 4.96526904185714 | Andorra |
| 2000 | 6.63690842034 | 0.781976505693047 | 1.69860246918706 | 4.93830595115294 | Andorra |
| 2001 | 6.62352270205 | 0.785417034980389 | 1.61076580974875 | 5.01275689230125 | Andorra |
| 2002 | 6.6965612843 | 0.79015875913382 | 1.60875894308479 | 5.08780234121521 | Andorra |
| 2003 | 6.86578543859 | 0.797278900724701 | 1.55757102909352 | 5.30821440949648 | Andorra |
| 2004 | 7.01816167837 | 0.80473197995372 | 1.53325830880512 | 5.48490336956488 | Andorra |
| 2005 | 7.07229910197 | 0.812213979716634 | 1.53173621852476 | 5.54056288344524 | Andorra |
| 2006 | 7.11868150097 | 0.819787259850839 | 1.49066712868875 | 5.62801437228125 | Andorra |
| 2007 | 7.16970804229 | 0.826617254254178 | 1.47304717857024 | 5.69666086371976 | Andorra |
| 2008 | 7.25921217026 | 0.83182171450476 | 1.48690618825491 | 5.77230598200509 | Andorra |
| 2009 | 7.31717861313 | 0.835929886554712 | 1.478222986092 | 5.838955627038 | Andorra |
| 2010 | 7.28341139654 | 0.839489966459192 | 1.45984304349095 | 5.82356835304905 | Andorra |
| 2011 | 7.25212037115 | 0.842794802354863 | 1.46405854712085 | 5.78806182402915 | Andorra |
| 2012 | 7.14953537119 | 0.845810830539451 | 1.46182958811042 | 5.68770578307958 | Andorra |
| 2013 | 7.05721461241 | 0.848329909768827 | 1.45828638338167 | 5.59892822902833 | Andorra |
| 2014 | 6.96624455769 | 0.851010491664786 | 1.4500362014319 | 5.5162083562581 | Andorra |
| 2015 | 6.90128265116 | 0.853591217830289 | 1.44649592477043 | 5.45478672638956 | Andorra |
| 2016 | 6.55478074425 | 0.856257052608717 | 1.42523723051537 | 5.12954351373463 | Andorra |
| 2017 | 5.90477251583 | 0.85947703010266 | 1.4331509794197 | 4.4716215364103 | Andorra |
| 2018 | 5.28208180857 | 0.862588950920789 | 1.4138151587486 | 3.8682666498214 | Andorra |
| 2019 | 4.96625459465 | 0.865621023596139 | 1.43221819952971 | 3.53403639512029 | Andorra |
| 2020 | 4.90417558602 | 0.867449169102346 | 1.43959600676972 | 3.46457957925028 | Andorra |
| 2021 | 4.75921941808 | 0.869444112735696 | 1.40717695643273 | 3.35204246164727 | Andorra |
| 1990 | 10.44270492388 | 0.749853692572025 | 1.69024431691839 | 8.75246060696161 | Austria |
| 1991 | 10.55816517863 | 0.751652734176599 | 1.81068526938804 | 8.74747990924196 | Austria |
| 1992 | 10.69129230641 | 0.754375285612199 | 1.74804551998719 | 8.94324678642281 | Austria |
| 1993 | 10.78205196456 | 0.758804553724473 | 1.756692065601 | 9.025359898959 | Austria |
| 1994 | 11.01884003021 | 0.764704364300466 | 1.78079749335418 | 9.23804253685582 | Austria |
| 1995 | 10.91809792627 | 0.769827471008921 | 1.75580792162529 | 9.16229000464471 | Austria |
| 1996 | 10.90739501375 | 0.774446565189292 | 1.76943197024481 | 9.13796304350518 | Austria |
| 1997 | 10.92038708666 | 0.77923285637593 | 1.69993449485324 | 9.22045259180676 | Austria |
| 1998 | 11.06978529139 | 0.783521015501574 | 1.66712001068231 | 9.40266528070769 | Austria |
| 1999 | 11.22616007977 | 0.787451584183882 | 1.61988940016066 | 9.60627067960935 | Austria |
| 2000 | 11.73513559973 | 0.791847679870807 | 1.58317286959239 | 10.1519627301376 | Austria |
| 2001 | 12.08901729824 | 0.795770117733764 | 1.56099787012352 | 10.5280194281165 | Austria |
| 2002 | 12.52958663485 | 0.799154119574311 | 1.56014245594489 | 10.9694441789051 | Austria |
| 2003 | 13.27375875422 | 0.802043253134005 | 1.55612228858 | 11.71763646564 | Austria |
| 2004 | 13.72057993267 | 0.804889143740108 | 1.51902365335167 | 12.2015562793183 | Austria |
| 2005 | 14.09840412906 | 0.808419194340084 | 1.53931768287411 | 12.5590864461859 | Austria |
| 2006 | 13.99954051895 | 0.812064609467116 | 1.5401466343031 | 12.4593938846469 | Austria |
| 2007 | 13.49734362217 | 0.815480521576858 | 1.51148974224726 | 11.9858538799227 | Austria |
| 2008 | 12.52556498278 | 0.818793658319811 | 1.50471965846883 | 11.0208453243112 | Austria |
| 2009 | 11.33802506241 | 0.821541167154033 | 1.49772055266593 | 9.84030450974407 | Austria |
| 2010 | 10.44210933591 | 0.824500109333185 | 1.49098881651515 | 8.95112051939485 | Austria |
| 2011 | 9.36217980312 | 0.82800199897916 | 1.47885842077985 | 7.88332138234015 | Austria |
| 2012 | 8.49823023918 | 0.83111330933878 | 1.48645513988585 | 7.01177509929415 | Austria |
| 2013 | 7.67283576367 | 0.833679780262374 | 1.47261745277735 | 6.20021831089265 | Austria |
| 2014 | 7.05178960366 | 0.835838577321076 | 1.46802777356289 | 5.58376183009711 | Austria |
| 2015 | 6.61092775122 | 0.837579895310124 | 1.47169982517111 | 5.13922792604889 | Austria |
| 2016 | 6.19690640875 | 0.839883789894186 | 1.47917215862673 | 4.71773425012327 | Austria |
| 2017 | 5.87009788967 | 0.843173330746292 | 1.47182870594 | 4.39826918373 | Austria |
| 2018 | 5.34261020144 | 0.846614470307794 | 1.4683565230203 | 3.8742536784197 | Austria |
| 2019 | 5.03326945158 | 0.849862514846872 | 1.46023631022612 | 3.57303314135388 | Austria |
| 2020 | 4.78954216452 | 0.852020385174284 | 1.46274141580206 | 3.32680074871794 | Austria |
| 2021 | 4.59366369353 | 0.853837004256539 | 1.45961296562757 | 3.13405072790243 | Austria |
| 1990 | 10.77999124689 | 0.737390656400179 | 2.12557555513447 | 8.65441569175553 | Belgium |
| 1991 | 11.54488110275 | 0.74217307419685 | 1.89565352312755 | 9.64922757962245 | Belgium |
| 1992 | 12.11072431015 | 0.748012299186744 | 1.7801135679356 | 10.3306107422144 | Belgium |
| 1993 | 11.59500303901 | 0.753685087006788 | 1.78641698889935 | 9.80858605011065 | Belgium |
| 1994 | 11.01653006575 | 0.758903637592037 | 1.76677918309509 | 9.24975088265491 | Belgium |
| 1995 | 10.0993258199 | 0.763170400784407 | 1.76889795041981 | 8.33042786948019 | Belgium |
| 1996 | 9.46698670122 | 0.766826075213302 | 1.7766509823383 | 7.6903357188817 | Belgium |
| 1997 | 9.61763965362 | 0.770813953023256 | 1.7989244837303 | 7.8187151698897 | Belgium |
| 1998 | 10.21954132963 | 0.774228267511882 | 1.77957502136736 | 8.43996630826264 | Belgium |
| 1999 | 10.69990337931 | 0.777041040124219 | 1.72573413829469 | 8.9741692410153 | Belgium |
| 2000 | 11.83670033737 | 0.780396331183289 | 1.7253642415442 | 10.1113360958258 | Belgium |
| 2001 | 12.37263250256 | 0.784713043367342 | 1.62590819326438 | 10.7467243092956 | Belgium |
| 2002 | 12.88335008146 | 0.788836054052762 | 1.60641662600231 | 11.2769334554577 | Belgium |
| 2003 | 13.75291602014 | 0.792014383577294 | 1.57935030913369 | 12.1735657110063 | Belgium |
| 2004 | 14.28480493158 | 0.795224935706338 | 1.58805346290764 | 12.6967514686724 | Belgium |
| 2005 | 16.080552303 | 0.798497235786438 | 1.54792038247175 | 14.5326319205283 | Belgium |
| 2006 | 18.03083810343 | 0.801572762913827 | 1.53360292641156 | 16.4972351770184 | Belgium |
| 2007 | 19.44519224635 | 0.804612664215848 | 1.54381632550323 | 17.9013759208468 | Belgium |
| 2008 | 18.79061244053 | 0.807757944740662 | 1.51557208010717 | 17.2750403604228 | Belgium |
| 2009 | 18.09744177857 | 0.811055825478368 | 1.540995449426 | 16.556446329144 | Belgium |
| 2010 | 17.37524959608 | 0.815030206751301 | 1.50383553665346 | 15.8714140594265 | Belgium |
| 2011 | 15.31041282113 | 0.819470362048624 | 1.47208892772109 | 13.8383238934089 | Belgium |
| 2012 | 15.1336965613 | 0.824226016040103 | 1.49114389420381 | 13.6425526670962 | Belgium |
| 2013 | 14.47679864376 | 0.828619831627193 | 1.48227468746729 | 12.9945239562927 | Belgium |
| 2014 | 13.56494738102 | 0.832425051502658 | 1.47703651107793 | 12.0879108699421 | Belgium |
| 2015 | 13.08683840062 | 0.835878400447772 | 1.47327107912 | 11.6135673215 | Belgium |
| 2016 | 12.65196177465 | 0.839462940488534 | 1.46338674636403 | 11.188575028286 | Belgium |
| 2017 | 13.49812959542 | 0.843212044031047 | 1.46500551518727 | 12.0331240802327 | Belgium |
| 2018 | 13.56360313049 | 0.846617625234845 | 1.46652508603792 | 12.0970780444521 | Belgium |
| 2019 | 12.92497926879 | 0.849498394807879 | 1.4703288130049 | 11.4546504557851 | Belgium |
| 2020 | 11.22481742456 | 0.851346648590241 | 1.4418942596945 | 9.7829231648655 | Belgium |
| 2021 | 11.10611866321 | 0.853654015777112 | 1.46375752130776 | 9.64236114190224 | Belgium |
| 1990 | 29.52710461039 | 0.648230871689669 | 6.09316283682355 | 23.4339417735665 | Cyprus |
| 1991 | 28.40406739181 | 0.655726169264618 | 5.06134749026522 | 23.3427199015448 | Cyprus |
| 1992 | 26.88859771805 | 0.666011849072818 | 4.15382508245212 | 22.7347726355979 | Cyprus |
| 1993 | 25.11120518842 | 0.677547476543954 | 3.58930962710974 | 21.5218955613103 | Cyprus |
| 1994 | 23.49566172915 | 0.688829360602013 | 3.32548805863277 | 20.1701736705172 | Cyprus |
| 1995 | 22.11085485712 | 0.699923898437876 | 2.9191739057619 | 19.1916809513581 | Cyprus |
| 1996 | 21.06584897225 | 0.709839946474019 | 2.71835930364895 | 18.347489668601 | Cyprus |
| 1997 | 20.21726731988 | 0.718864243512848 | 2.56707474926741 | 17.6501925706126 | Cyprus |
| 1998 | 19.36326017937 | 0.727964381748969 | 2.45884459645031 | 16.9044155829197 | Cyprus |
| 1999 | 18.61942462323 | 0.736846134920381 | 2.08052039549212 | 16.5389042277379 | Cyprus |
| 2000 | 17.99752497793 | 0.745199300565367 | 1.78464303507824 | 16.2128819428518 | Cyprus |
| 2001 | 17.48772502739 | 0.753594717257543 | 1.74812826137217 | 15.7395967660178 | Cyprus |
| 2002 | 17.22024677468 | 0.76150246522841 | 1.88329781471029 | 15.3369489599697 | Cyprus |
| 2003 | 17.03054659332 | 0.767896215824759 | 1.67863606805642 | 15.3519105252636 | Cyprus |
| 2004 | 16.97647617682 | 0.77376477492064 | 1.76184671170648 | 15.2146294651135 | Cyprus |
| 2005 | 16.97005847198 | 0.779031361179798 | 1.71780380095824 | 15.2522546710218 | Cyprus |
| 2006 | 16.71184320486 | 0.784560964983202 | 1.65700349897636 | 15.0548397058836 | Cyprus |
| 2007 | 16.41284316649 | 0.792068382747098 | 1.58746211013984 | 14.8253810563502 | Cyprus |
| 2008 | 16.12643252423 | 0.799454089093792 | 1.56484507830632 | 14.5615874459237 | Cyprus |
| 2009 | 15.80960785105 | 0.805112085484562 | 1.50932339402655 | 14.3002844570235 | Cyprus |
| 2010 | 15.25375568089 | 0.809840187817991 | 1.53412190083394 | 13.7196337800561 | Cyprus |
| 2011 | 14.66120127973 | 0.813726132185002 | 1.52072293397302 | 13.140478345757 | Cyprus |
| 2012 | 14.12527699465 | 0.816978525424299 | 1.49865183198259 | 12.6266251626674 | Cyprus |
| 2013 | 13.4515804557 | 0.819380601631525 | 1.50859522491826 | 11.9429852307817 | Cyprus |
| 2014 | 13.151641335 | 0.821184302169431 | 1.4990110419707 | 11.6526302930293 | Cyprus |
| 2015 | 12.86332273397 | 0.822732850898765 | 1.48889472928362 | 11.3744280046864 | Cyprus |
| 2016 | 12.27815729059 | 0.824283624143557 | 1.48146310947475 | 10.7966941811153 | Cyprus |
| 2017 | 11.32491270124 | 0.826139877968847 | 1.48868540726912 | 9.83622729397088 | Cyprus |
| 2018 | 10.52398669946 | 0.828343243063834 | 1.48551476893736 | 9.03847193052264 | Cyprus |
| 2019 | 9.96797667448 | 0.830980727806796 | 1.4890566200688 | 8.4789200544112 | Cyprus |
| 2020 | 9.56037453916 | 0.833059522677264 | 1.4745669897377 | 8.0858075494223 | Cyprus |
| 2021 | 9.42294047525 | 0.835630545002355 | 1.46931997249703 | 7.95362050275297 | Cyprus |
| 1990 | 14.5725367081 | 0.801154655014013 | 1.52707861360467 | 13.0454580944953 | Denmark |
| 1991 | 14.37393580625 | 0.804540349639536 | 1.52484853536475 | 12.8490872708853 | Denmark |
| 1992 | 13.72615942937 | 0.808164433760271 | 1.5432317588356 | 12.1829276705344 | Denmark |
| 1993 | 11.97058401625 | 0.811585712633093 | 1.53104051951661 | 10.4395434967334 | Denmark |
| 1994 | 11.83974332588 | 0.815138614432107 | 1.50503487690309 | 10.3347084489769 | Denmark |
| 1995 | 11.38644818433 | 0.819104611612713 | 1.49367083601203 | 9.89277734831797 | Denmark |
| 1996 | 11.37095239108 | 0.82337524309543 | 1.48736730353529 | 9.88358508754471 | Denmark |
| 1997 | 11.20477806276 | 0.827679979394938 | 1.50550146818106 | 9.69927659457894 | Denmark |
| 1998 | 11.22946126446 | 0.831683237018017 | 1.47704717541333 | 9.75241408904667 | Denmark |
| 1999 | 11.45237182904 | 0.835381542910635 | 1.46964450873242 | 9.98272732030758 | Denmark |
| 2000 | 12.89900869398 | 0.8391842975615 | 1.46809636076078 | 11.4309123332192 | Denmark |
| 2001 | 13.8314833102 | 0.843177750467757 | 1.46698328600656 | 12.3645000241934 | Denmark |
| 2002 | 14.52905156156 | 0.847086356838798 | 1.47601014118726 | 13.0530414203727 | Denmark |
| 2003 | 15.7518856674 | 0.850441325949586 | 1.461042691128 | 14.290842976272 | Denmark |
| 2004 | 16.8136903644 | 0.853304103185433 | 1.44743189863 | 15.36625846577 | Denmark |
| 2005 | 18.32798272555 | 0.855690732240651 | 1.44958111340852 | 16.8784016121415 | Denmark |
| 2006 | 19.36728484461 | 0.858033209615474 | 1.41667405210852 | 17.9506107925015 | Denmark |
| 2007 | 22.18773788234 | 0.859781733794886 | 1.43390027320109 | 20.7538376091389 | Denmark |
| 2008 | 22.90294364489 | 0.861782061067554 | 1.42260869672052 | 21.4803349481695 | Denmark |
| 2009 | 21.7795571223 | 0.86394804976016 | 1.41949895238067 | 20.3600581699193 | Denmark |
| 2010 | 21.3108428133 | 0.866767079164022 | 1.42563422541556 | 19.8852085878844 | Denmark |
| 2011 | 19.45169787272 | 0.869778682281296 | 1.41465114982587 | 18.0370467228941 | Denmark |
| 2012 | 18.79972397994 | 0.872517510381731 | 1.41343793316983 | 17.3862860467702 | Denmark |
| 2013 | 18.95587819934 | 0.874932973936641 | 1.40762804006641 | 17.5482501592736 | Denmark |
| 2014 | 19.17952198723 | 0.876814931654032 | 1.40938833548569 | 17.7701336517443 | Denmark |
| 2015 | 18.79370515702 | 0.878543944867921 | 1.39965352792405 | 17.3940516290959 | Denmark |
| 2016 | 18.31429800829 | 0.8805897834924 | 1.38788760885582 | 16.9264103994342 | Denmark |
| 2017 | 18.31412264843 | 0.883688486994588 | 1.40542807392821 | 16.9086945745018 | Denmark |
| 2018 | 16.95845473696 | 0.887529846224258 | 1.38584800346018 | 15.5726067334998 | Denmark |
| 2019 | 14.93592502489 | 0.891624571480342 | 1.38481008082538 | 13.5511149440646 | Denmark |
| 2020 | 14.37243301817 | 0.894368296843327 | 1.39450593946481 | 12.9779270787052 | Denmark |
| 2021 | 14.17094579724 | 0.896424204101212 | 1.37586058271742 | 12.7950852145226 | Denmark |
| 1990 | 6.00951344737 | 0.756221508893542 | 1.81918800175872 | 4.19032544561128 | Finland |
| 1991 | 6.16965891233 | 0.758410686787082 | 1.75535411306162 | 4.41430479926838 | Finland |
| 1992 | 6.24960395763 | 0.760804099620088 | 1.79448460261392 | 4.45511935501608 | Finland |
| 1993 | 6.10359265066 | 0.763526603650282 | 1.72813064813186 | 4.37546200252814 | Finland |
| 1994 | 6.15659471981 | 0.767101900863416 | 1.75872265788264 | 4.39787206192736 | Finland |
| 1995 | 6.19455310101 | 0.771163742219649 | 1.71808557991644 | 4.47646752109356 | Finland |
| 1996 | 6.27797594875 | 0.774809058593956 | 1.72303270421382 | 4.55494324453618 | Finland |
| 1997 | 6.42731212925 | 0.778814214501226 | 1.70690948698073 | 4.72040264226927 | Finland |
| 1998 | 6.63572711605 | 0.782305059190857 | 1.68678385905021 | 4.94894325699979 | Finland |
| 1999 | 6.83648126345 | 0.78577398760406 | 1.644257151025 | 5.192224112425 | Finland |
| 2000 | 7.18509423267 | 0.78998522981789 | 1.61908210845723 | 5.56601212421277 | Finland |
| 2001 | 7.37898970453 | 0.79445383485974 | 1.57285971653344 | 5.80612998799656 | Finland |
| 2002 | 7.76633190377 | 0.798770274163901 | 1.54551388568857 | 6.22081801808143 | Finland |
| 2003 | 8.33973214814 | 0.802325430617225 | 1.54279621166429 | 6.79693593647571 | Finland |
| 2004 | 8.82387232686 | 0.805694227411326 | 1.54396956640837 | 7.27990276045163 | Finland |
| 2005 | 9.17778386328 | 0.808936787277645 | 1.51961076444245 | 7.65817309883755 | Finland |
| 2006 | 9.34241762146 | 0.812081481875967 | 1.52458136659787 | 7.81783625486213 | Finland |
| 2007 | 9.56820977126 | 0.815597613277792 | 1.49118056893286 | 8.07702920232714 | Finland |
| 2008 | 9.55899860014 | 0.818875543929948 | 1.48781073366297 | 8.07118786647703 | Finland |
| 2009 | 9.31380595731 | 0.821457587971858 | 1.48610044108577 | 7.82770551622423 | Finland |
| 2010 | 9.05430373273 | 0.824844982269378 | 1.4843221326754 | 7.5699816000546 | Finland |
| 2011 | 8.53315022299 | 0.82827057673312 | 1.49070256690653 | 7.04244765608347 | Finland |
| 2012 | 8.0564889914 | 0.831434483518693 | 1.46365433964204 | 6.59283465175796 | Finland |
| 2013 | 7.24300413097 | 0.834425400661261 | 1.4786865878639 | 5.7643175431061 | Finland |
| 2014 | 6.8537406656 | 0.837431871657021 | 1.47292720813388 | 5.38081345746612 | Finland |
| 2015 | 6.15230693293 | 0.840878476913666 | 1.45778108912103 | 4.69452584380897 | Finland |
| 2016 | 5.76109471606 | 0.844622338388364 | 1.45686835881 | 4.30422635725 | Finland |
| 2017 | 5.52525061403 | 0.848586127623286 | 1.46999136600433 | 4.05525924802567 | Finland |
| 2018 | 5.213209177 | 0.852202943239694 | 1.44086411001036 | 3.77234506698964 | Finland |
| 2019 | 4.99785145341 | 0.855365178519151 | 1.41992238300568 | 3.57792907040432 | Finland |
| 2020 | 4.78497274731 | 0.857655552665083 | 1.43422434036837 | 3.35074840694163 | Finland |
| 2021 | 4.77349925971 | 0.859831367662218 | 1.40270007675784 | 3.37079918295216 | Finland |
| 1990 | 9.46369338799 | 0.730747465817975 | 2.28422991865115 | 7.17946346933885 | France |
| 1991 | 10.0928581212 | 0.736452382335769 | 1.98923540876939 | 8.10362271243061 | France |
| 1992 | 10.54750279877 | 0.742871973277562 | 1.91692771296918 | 8.63057508580082 | France |
| 1993 | 10.70784998363 | 0.748582326771669 | 1.851173326661 | 8.856676656969 | France |
| 1994 | 10.02507859075 | 0.753176131807871 | 1.84410452915959 | 8.18097406159041 | France |
| 1995 | 9.40327406622 | 0.75712578885604 | 1.73151940995352 | 7.67175465626648 | France |
| 1996 | 9.38012179276 | 0.760899578763994 | 1.73205606295641 | 7.64806572980359 | France |
| 1997 | 9.11506929584 | 0.764650672066045 | 1.7938806283558 | 7.3211886674842 | France |
| 1998 | 9.19237006403 | 0.767852560919953 | 1.75249478988944 | 7.43987527414055 | France |
| 1999 | 9.21053393313 | 0.770304362540569 | 1.76239513264545 | 7.44813880048455 | France |
| 2000 | 9.48447016076 | 0.772792033272814 | 1.75276238982107 | 7.73170777093893 | France |
| 2001 | 9.65113831903 | 0.776225157250438 | 1.75515632451511 | 7.89598199451489 | France |
| 2002 | 9.14959364649 | 0.780088871976379 | 1.71536492129649 | 7.43422872519351 | France |
| 2003 | 9.6352555471 | 0.783178757362352 | 1.6539831762002 | 7.9812723708998 | France |
| 2004 | 9.7662556864 | 0.786084654751087 | 1.64383922091814 | 8.12241646548186 | France |
| 2005 | 9.88729653904 | 0.789042086328118 | 1.5957265444856 | 8.2915699945544 | France |
| 2006 | 10.63276332438 | 0.792321919625469 | 1.56789426276965 | 9.06486906161035 | France |
| 2007 | 11.25488723153 | 0.795557651447132 | 1.55888842811625 | 9.69599880341375 | France |
| 2008 | 11.88568456968 | 0.798177429586454 | 1.55771837639739 | 10.3279661932826 | France |
| 2009 | 11.98502154385 | 0.800508669748406 | 1.56595119005816 | 10.4190703537918 | France |
| 2010 | 11.60400640434 | 0.803240246888394 | 1.5160913176972 | 10.0879150866428 | France |
| 2011 | 10.38817526386 | 0.806378738306578 | 1.55349127295207 | 8.83468399090793 | France |
| 2012 | 10.33339873253 | 0.809615388940868 | 1.52993847893241 | 8.80346025359759 | France |
| 2013 | 10.38640810414 | 0.812984015183301 | 1.52013702262225 | 8.86627108151775 | France |
| 2014 | 10.21609632666 | 0.816551447578728 | 1.51993104258469 | 8.69616528407531 | France |
| 2015 | 10.38185133048 | 0.820238385480897 | 1.48878762661627 | 8.89306370386373 | France |
| 2016 | 9.60884796965 | 0.82373092859985 | 1.46591803658432 | 8.14292993306568 | France |
| 2017 | 9.43489317257 | 0.827098440641564 | 1.47758422362511 | 7.95730894894489 | France |
| 2018 | 9.32386431722 | 0.830448590187259 | 1.4833556749319 | 7.8405086422881 | France |
| 2019 | 9.00654788653 | 0.833772804699999 | 1.47539531742532 | 7.53115256910468 | France |
| 2020 | 8.45535776647 | 0.836049443246129 | 1.47147090217225 | 6.98388686429775 | France |
| 2021 | 8.24949974991 | 0.838364875196954 | 1.46570195790525 | 6.78379779200475 | France |
| 1990 | 11.17847023763 | 0.817077665790386 | 1.49688832235132 | 9.68158191527868 | Germany |
| 1991 | 11.82240998719 | 0.823339898340462 | 1.49112944295821 | 10.3312805442318 | Germany |
| 1992 | 12.19100567805 | 0.828323503378164 | 1.47810787713784 | 10.7128978009122 | Germany |
| 1993 | 12.47738766682 | 0.832286809367201 | 1.49419339853118 | 10.9831942682888 | Germany |
| 1994 | 12.74072802286 | 0.835561963052536 | 1.48298368233121 | 11.2577443405288 | Germany |
| 1995 | 13.06472744818 | 0.837643151522198 | 1.46829381751745 | 11.5964336306626 | Germany |
| 1996 | 13.34779053944 | 0.839188129123403 | 1.45989354794481 | 11.8878969914952 | Germany |
| 1997 | 13.97651117879 | 0.841385902988361 | 1.47083497568891 | 12.5056762031011 | Germany |
| 1998 | 14.50043186439 | 0.843854978100034 | 1.45485421352806 | 13.0455776508619 | Germany |
| 1999 | 14.95649077679 | 0.846232398245621 | 1.4591161733442 | 13.4973746034458 | Germany |
| 2000 | 15.72679671387 | 0.848925756438545 | 1.45525636255297 | 14.271540351317 | Germany |
| 2001 | 16.25871680629 | 0.852070412843074 | 1.44532523901094 | 14.8133915672791 | Germany |
| 2002 | 16.94759198932 | 0.855066792837203 | 1.41911965371719 | 15.5284723356028 | Germany |
| 2003 | 18.13465308531 | 0.857675790269356 | 1.44423045516524 | 16.6904226301448 | Germany |
| 2004 | 19.00512263209 | 0.860240332885484 | 1.40757155771855 | 17.5975510743715 | Germany |
| 2005 | 20.05729365928 | 0.862894178369357 | 1.41024806261975 | 18.6470455966603 | Germany |
| 2006 | 21.41565673456 | 0.865873706890654 | 1.4205347629731 | 19.9951219715869 | Germany |
| 2007 | 22.68737323339 | 0.869140440024023 | 1.422854442841 | 21.264518790549 | Germany |
| 2008 | 22.07222620696 | 0.872552849301022 | 1.40775503169727 | 20.6644711752627 | Germany |
| 2009 | 21.59087877053 | 0.875087911282131 | 1.40058601483571 | 20.1902927556943 | Germany |
| 2010 | 20.87886476381 | 0.877946710427414 | 1.41251706980848 | 19.4663476940015 | Germany |
| 2011 | 19.48092127425 | 0.881027507503078 | 1.41410085276719 | 18.0668204214828 | Germany |
| 2012 | 18.05755443214 | 0.88365640827103 | 1.39884165381395 | 16.6587127783261 | Germany |
| 2013 | 17.38818071776 | 0.885926953064586 | 1.39747708527476 | 15.9907036324852 | Germany |
| 2014 | 16.17548819375 | 0.887651614379013 | 1.36822080612306 | 14.8072673876269 | Germany |
| 2015 | 15.7289794527 | 0.888867853966898 | 1.4037912175687 | 14.3251882351313 | Germany |
| 2016 | 14.97675249744 | 0.89089636945676 | 1.39534478435386 | 13.5814077130861 | Germany |
| 2017 | 13.43409002375 | 0.894050472424158 | 1.3893021502977 | 12.0447878734523 | Germany |
| 2018 | 12.01686013762 | 0.896975545420426 | 1.38561150745895 | 10.6312486301611 | Germany |
| 2019 | 9.9470150212 | 0.899703158084962 | 1.38948887702879 | 8.55752614417121 | Germany |
| 2020 | 8.81128115516 | 0.901438614019463 | 1.39204566924289 | 7.41923548591711 | Germany |
| 2021 | 8.39790644131 | 0.902957091057572 | 1.40983257758186 | 6.98807386372814 | Germany |
| 1990 | 3.5350534556 | 0.67418646507665 | 3.52818285754104 | 0.00687059805895851 | Greece |
| 1991 | 3.52473936446 | 0.680316480101809 | 3.50196689085488 | 0.022772473605122 | Greece |
| 1992 | 3.53488180617 | 0.687055389369745 | 3.33865662279875 | 0.19622518337125 | Greece |
| 1993 | 3.54818444775 | 0.69332892644804 | 3.10170451702424 | 0.446479930725758 | Greece |
| 1994 | 3.57610312424 | 0.699153022352574 | 2.8790694093671 | 0.697033714872895 | Greece |
| 1995 | 3.62026792921 | 0.704862873096307 | 2.96644784845296 | 0.653820080757038 | Greece |
| 1996 | 3.68931853356 | 0.710139335918529 | 2.72085017665474 | 0.968468356905263 | Greece |
| 1997 | 3.77353791493 | 0.715687267664404 | 2.56216789688231 | 1.21137001804769 | Greece |
| 1998 | 3.89097889606 | 0.721593982464726 | 2.51338868712743 | 1.37759020893257 | Greece |
| 1999 | 4.0015510546 | 0.727529454753368 | 2.4250564414527 | 1.5764946131473 | Greece |
| 2000 | 4.12184283265 | 0.732710539506218 | 2.254617638164 | 1.867225194486 | Greece |
| 2001 | 4.22655690647 | 0.737154008101854 | 2.12916793257822 | 2.09738897389178 | Greece |
| 2002 | 4.35700130796 | 0.741945221017726 | 1.87322111237647 | 2.48378019558353 | Greece |
| 2003 | 4.44564980647 | 0.747185955887149 | 1.82589144673829 | 2.61975835973171 | Greece |
| 2004 | 4.58566886486 | 0.752418606565094 | 1.78285902182703 | 2.80280984303297 | Greece |
| 2005 | 4.71910540036 | 0.75638439564551 | 1.78524163613239 | 2.93386376422761 | Greece |
| 2006 | 4.89657460219 | 0.760209695750845 | 1.81689003692839 | 3.07968456526161 | Greece |
| 2007 | 5.05424282457 | 0.763927173080314 | 1.73882033302556 | 3.31542249154444 | Greece |
| 2008 | 5.09957122606 | 0.767527657538734 | 1.75082088749333 | 3.34875033856667 | Greece |
| 2009 | 5.1913348524 | 0.771247355848723 | 1.7335910425704 | 3.4577438098296 | Greece |
| 2010 | 5.20956951077 | 0.775155814653294 | 1.727757225325 | 3.481812285445 | Greece |
| 2011 | 5.04756443333 | 0.778525866909133 | 1.66894137607 | 3.37862305726 | Greece |
| 2012 | 4.94746104722 | 0.780789482881484 | 1.7047305009905 | 3.2427305462295 | Greece |
| 2013 | 4.94257446724 | 0.781949051742451 | 1.74451762867484 | 3.19805683856516 | Greece |
| 2014 | 4.91637777199 | 0.782442012668796 | 1.72397095266877 | 3.19240681932123 | Greece |
| 2015 | 4.85382024264 | 0.782489523445532 | 1.61327697733844 | 3.24054326530156 | Greece |
| 2016 | 4.62598022443 | 0.782798442836071 | 1.68220195226833 | 2.94377827216167 | Greece |
| 2017 | 4.11417904272 | 0.784108593896727 | 1.72498510966929 | 2.38919393305071 | Greece |
| 2018 | 3.65238982677 | 0.785999752836636 | 1.64150394851171 | 2.01088587825829 | Greece |
| 2019 | 3.38552147733 | 0.788138138237775 | 1.66729835701744 | 1.71822312031256 | Greece |
| 2020 | 3.15935091823 | 0.789632219998033 | 1.62530977170973 | 1.53404114652027 | Greece |
| 2021 | 3.00211782674 | 0.791854407912829 | 1.55513174428906 | 1.44698608245094 | Greece |
| 1990 | 5.55124463016 | 0.764212516960703 | 1.76984706377625 | 3.78139756638375 | Iceland |
| 1991 | 5.67466754792 | 0.769027393143331 | 1.75333655365804 | 3.92133099426196 | Iceland |
| 1992 | 5.82392803123 | 0.772706414740194 | 1.8194826218046 | 4.0044454094254 | Iceland |
| 1993 | 5.85098256552 | 0.776389422872018 | 1.7068904072702 | 4.1440921582498 | Iceland |
| 1994 | 5.91415997986 | 0.77996796073086 | 1.69098219858244 | 4.22317778127756 | Iceland |
| 1995 | 6.00013995339 | 0.782740057259954 | 1.68714840675039 | 4.31299154663961 | Iceland |
| 1996 | 6.16280315106 | 0.785356419811944 | 1.67108838493581 | 4.49171476612419 | Iceland |
| 1997 | 6.07585369427 | 0.78852833520489 | 1.61764216964829 | 4.45821152462171 | Iceland |
| 1998 | 6.10507451374 | 0.792626368445487 | 1.58443730105535 | 4.52063721268465 | Iceland |
| 1999 | 6.2114029273 | 0.797368807995672 | 1.55935996051163 | 4.65204296678837 | Iceland |
| 2000 | 6.43005240199 | 0.803030330314896 | 1.54902759241717 | 4.88102480957283 | Iceland |
| 2001 | 6.67456020861 | 0.808965785947657 | 1.51697401587415 | 5.15758619273585 | Iceland |
| 2002 | 6.90281885977 | 0.813400392129504 | 1.54117792364894 | 5.36164093612106 | Iceland |
| 2003 | 7.19910993309 | 0.816597066979147 | 1.51432029673864 | 5.68478963635136 | Iceland |
| 2004 | 7.55394555436 | 0.819528086895788 | 1.49412648453447 | 6.05981906982553 | Iceland |
| 2005 | 7.92004680953 | 0.822766536023531 | 1.4954380039596 | 6.4246088055704 | Iceland |
| 2006 | 8.42701264587 | 0.82624474349372 | 1.48031490862541 | 6.94669773724459 | Iceland |
| 2007 | 8.86324015579 | 0.830289889861998 | 1.4701261405837 | 7.3931140152063 | Iceland |
| 2008 | 9.01949375993 | 0.834622678087519 | 1.47204530511455 | 7.54744845481545 | Iceland |
| 2009 | 8.99839347465 | 0.838121013089424 | 1.47026642791315 | 7.52812704673685 | Iceland |
| 2010 | 8.87158443667 | 0.841384642074063 | 1.4609710101334 | 7.4106134265366 | Iceland |
| 2011 | 8.46628374201 | 0.844619929908845 | 1.46209141323459 | 7.00419232877541 | Iceland |
| 2012 | 8.25076881146 | 0.84729944372556 | 1.44830499133662 | 6.80246382012338 | Iceland |
| 2013 | 8.16513928746 | 0.850173947991231 | 1.46518214437509 | 6.69995714308491 | Iceland |
| 2014 | 8.11033310753 | 0.853558696790214 | 1.44574433920212 | 6.66458876832788 | Iceland |
| 2015 | 8.04333960866 | 0.857724976230652 | 1.44715926950238 | 6.59618033915762 | Iceland |
| 2016 | 7.69323212791 | 0.861844466505036 | 1.4198694208712 | 6.2733627070388 | Iceland |
| 2017 | 7.18632833402 | 0.865702890708071 | 1.41679514340667 | 5.76953319061333 | Iceland |
| 2018 | 6.55247149513 | 0.869219821760483 | 1.38919164358875 | 5.16327985154125 | Iceland |
| 2019 | 5.99089944718 | 0.872425580161805 | 1.41216817912366 | 4.57873126805634 | Iceland |
| 2020 | 5.83521116738 | 0.874323153425611 | 1.42645029281163 | 4.40876087456837 | Iceland |
| 2021 | 5.74639376925 | 0.876361679802766 | 1.38773982195231 | 4.35865394729769 | Iceland |
| 1990 | 5.00573461436 | 0.719891818965305 | 2.54258571633978 | 2.46314889802022 | Ireland |
| 1991 | 5.00929084793 | 0.725195018704741 | 2.31109774924321 | 2.69819309868679 | Ireland |
| 1992 | 5.03223436591 | 0.731918691315293 | 2.17157542957475 | 2.86065893633525 | Ireland |
| 1993 | 5.0744240503 | 0.738384073791795 | 2.09078617340721 | 2.98363787689279 | Ireland |
| 1994 | 5.03687545928 | 0.744011727859478 | 1.87149069555214 | 3.16538476372786 | Ireland |
| 1995 | 5.2115029334 | 0.748921575045051 | 1.79731988070024 | 3.41418305269976 | Ireland |
| 1996 | 5.36778542758 | 0.754010123608321 | 1.78387153878212 | 3.58391388879788 | Ireland |
| 1997 | 5.61849743595 | 0.759970383842882 | 1.77031376870793 | 3.84818366724207 | Ireland |
| 1998 | 5.84066060627 | 0.766488720882709 | 1.66911422239389 | 4.17154638387611 | Ireland |
| 1999 | 6.17011608175 | 0.773538834439684 | 1.71156401349756 | 4.45855206825244 | Ireland |
| 2000 | 6.51259351369 | 0.780190655170267 | 1.67993681851 | 4.83265669518 | Ireland |
| 2001 | 6.88235343629 | 0.786353740708812 | 1.64818356687561 | 5.23416986941439 | Ireland |
| 2002 | 7.18818901376 | 0.793010605962827 | 1.6008748641522 | 5.5873141496078 | Ireland |
| 2003 | 7.67972629689 | 0.800005865377216 | 1.54780195212436 | 6.13192434476564 | Ireland |
| 2004 | 7.913388739 | 0.806853140411776 | 1.53250257766279 | 6.38088616133721 | Ireland |
| 2005 | 8.21917682219 | 0.811713818388319 | 1.51986688419105 | 6.69930993799895 | Ireland |
| 2006 | 8.89170759832 | 0.814505479095719 | 1.50244075173085 | 7.38926684658915 | Ireland |
| 2007 | 9.66073523598 | 0.816931026031536 | 1.50806081710823 | 8.15267441887177 | Ireland |
| 2008 | 10.34898908038 | 0.819932128191974 | 1.47848594929855 | 8.87050313108146 | Ireland |
| 2009 | 10.54029738334 | 0.823929666301939 | 1.48179440172127 | 9.05850298161873 | Ireland |
| 2010 | 10.64680537943 | 0.828103848001623 | 1.485101684985 | 9.161703694445 | Ireland |
| 2011 | 10.35615595252 | 0.83180479150489 | 1.48048576548083 | 8.87567018703917 | Ireland |
| 2012 | 10.51989207189 | 0.835325358632175 | 1.48225718554033 | 9.03763488634967 | Ireland |
| 2013 | 10.87196863088 | 0.838644432713483 | 1.46595206517063 | 9.40601656570938 | Ireland |
| 2014 | 11.00514465116 | 0.842082114682265 | 1.46365509793527 | 9.54148955322473 | Ireland |
| 2015 | 11.07845129424 | 0.847437886654412 | 1.46828619459595 | 9.61016509964405 | Ireland |
| 2016 | 10.3324838824 | 0.85264250194342 | 1.4446374303262 | 8.8878464520738 | Ireland |
| 2017 | 8.84080315896 | 0.857816163802237 | 1.41364509222196 | 7.42715806673804 | Ireland |
| 2018 | 7.30689530118 | 0.863925284412195 | 1.41449478253103 | 5.89240051864897 | Ireland |
| 2019 | 6.24357674267 | 0.869412503348037 | 1.45029015313651 | 4.79328658953349 | Ireland |
| 2020 | 5.51113019289 | 0.871958822388357 | 1.43308074647061 | 4.07804944641939 | Ireland |
| 2021 | 5.43703675397 | 0.873753849787408 | 1.38760183017065 | 4.04943492379935 | Ireland |
| 1990 | 12.41147008636 | 0.709178347365152 | 2.77031229350927 | 9.64115779285073 | Israel |
| 1991 | 12.52329581621 | 0.713371633399082 | 2.70525081827448 | 9.81804499793552 | Israel |
| 1992 | 13.6756454551 | 0.717897923179142 | 2.50752407066068 | 11.1681213844393 | Israel |
| 1993 | 12.73496137879 | 0.72225017554813 | 2.40784748480446 | 10.3271138939855 | Israel |
| 1994 | 11.76002025437 | 0.726654735721658 | 2.5533251704806 | 9.2066950838894 | Israel |
| 1995 | 10.96731425338 | 0.731127859056384 | 2.18252556886437 | 8.78478868451563 | Israel |
| 1996 | 10.83840426471 | 0.734714025875415 | 2.09966323282476 | 8.73874103188524 | Israel |
| 1997 | 11.10421339666 | 0.738015276501763 | 2.16143275162283 | 8.94278064503717 | Israel |
| 1998 | 11.67212533145 | 0.741927189995428 | 1.85715200833163 | 9.81497332311837 | Israel |
| 1999 | 12.34053689521 | 0.745443338031474 | 1.81415109952641 | 10.5263857956836 | Israel |
| 2000 | 13.27919874259 | 0.749184084123824 | 1.82722728961123 | 11.4519714529788 | Israel |
| 2001 | 13.50280362613 | 0.752834296773304 | 1.78628270410119 | 11.7165209220288 | Israel |
| 2002 | 14.57278029017 | 0.755988075842232 | 1.78186093868206 | 12.7909193514879 | Israel |
| 2003 | 15.67951628939 | 0.758875408886722 | 1.76382370901292 | 13.9156925803771 | Israel |
| 2004 | 16.29458082216 | 0.762213291396151 | 1.81326729397475 | 14.4813135281853 | Israel |
| 2005 | 17.3781710294 | 0.765543461435524 | 1.74022183737718 | 15.6379491920228 | Israel |
| 2006 | 19.53635764134 | 0.768733897655938 | 1.74286997216937 | 17.7934876691706 | Israel |
| 2007 | 20.55790848403 | 0.770756143247979 | 1.76590585012779 | 18.7920026339022 | Israel |
| 2008 | 20.45717348329 | 0.772011614541036 | 1.71217119231389 | 18.7450022909761 | Israel |
| 2009 | 19.45660349312 | 0.773303913379524 | 1.73791555484103 | 17.718687938279 | Israel |
| 2010 | 18.59971704589 | 0.775259530065334 | 1.77145643544242 | 16.8282606104476 | Israel |
| 2011 | 16.90710228725 | 0.77764966916418 | 1.67204892179974 | 15.2350533654503 | Israel |
| 2012 | 16.06114702048 | 0.780403414638078 | 1.68944015328222 | 14.3717068671978 | Israel |
| 2013 | 15.15331254759 | 0.783267166608914 | 1.7187979395518 | 13.4345146080382 | Israel |
| 2014 | 14.30398794923 | 0.78615108379553 | 1.61566525807738 | 12.6883226911526 | Israel |
| 2015 | 13.93858307827 | 0.788874456945394 | 1.65721759666286 | 12.2813654816071 | Israel |
| 2016 | 13.68282593816 | 0.791772445367912 | 1.59609410165396 | 12.086731836506 | Israel |
| 2017 | 13.85492436601 | 0.795173437476151 | 1.555616578389 | 12.299307787621 | Israel |
| 2018 | 13.13438920956 | 0.799124993659694 | 1.52930941847552 | 11.6050797910845 | Israel |
| 2019 | 12.47806987442 | 0.803232990528098 | 1.52704501458167 | 10.9510248598383 | Israel |
| 2020 | 10.0055156617 | 0.806351589333701 | 1.52847937862115 | 8.47703628307885 | Israel |
| 2021 | 8.40225578047 | 0.809011651557631 | 1.53813790814659 | 6.86411787232341 | Israel |
| 1990 | 6.32220755668 | 0.706255223740403 | 3.02653839004143 | 3.29566916663857 | Italy |
| 1991 | 6.34921593564 | 0.711457348523815 | 2.84445305764043 | 3.50476287799957 | Italy |
| 1992 | 6.62508397001 | 0.716649746121184 | 2.5753743046375 | 4.0497096653725 | Italy |
| 1993 | 6.44926064223 | 0.721720992266354 | 2.4568810985016 | 3.9923795437284 | Italy |
| 1994 | 6.13121342565 | 0.726870913262732 | 2.40216755883759 | 3.72904586681241 | Italy |
| 1995 | 5.76172133002 | 0.731644081628044 | 2.28409150571775 | 3.47762982430225 | Italy |
| 1996 | 6.04630700373 | 0.736050870453315 | 2.10938592014048 | 3.93692108358952 | Italy |
| 1997 | 6.40642161724 | 0.739972336484864 | 1.8970773582705 | 4.5093442589695 | Italy |
| 1998 | 6.69226160186 | 0.743101595998998 | 1.82001843634648 | 4.87224316551352 | Italy |
| 1999 | 7.12502835884 | 0.746074084421097 | 1.72836969016619 | 5.39665866867381 | Italy |
| 2000 | 7.58356966588 | 0.749675986238207 | 1.79788465254962 | 5.78568501333038 | Italy |
| 2001 | 7.9879982353 | 0.753554839457876 | 1.75483835937172 | 6.23315987592828 | Italy |
| 2002 | 7.85323844583 | 0.757191479404768 | 1.75374491538795 | 6.09949353044204 | Italy |
| 2003 | 8.29793501519 | 0.76009374379023 | 1.74728666581846 | 6.55064834937154 | Italy |
| 2004 | 8.5541558215 | 0.762787749063442 | 1.74439878626431 | 6.80975703523569 | Italy |
| 2005 | 8.70054553352 | 0.765689914012792 | 1.73599802575937 | 6.96454750776063 | Italy |
| 2006 | 8.57523643659 | 0.768402407690909 | 1.80099612847051 | 6.77424030811949 | Italy |
| 2007 | 8.64504275042 | 0.770698380337301 | 1.78070231301453 | 6.86434043740547 | Italy |
| 2008 | 9.0430314481 | 0.773068469998338 | 1.71685438886292 | 7.32617705923708 | Italy |
| 2009 | 9.44288893467 | 0.775499262002478 | 1.7420281313932 | 7.7008608032768 | Italy |
| 2010 | 10.04680100644 | 0.778109205917719 | 1.74694413795119 | 8.29985686848881 | Italy |
| 2011 | 10.68001073825 | 0.780703467869893 | 1.69035425673783 | 8.98965648151217 | Italy |
| 2012 | 11.35765975643 | 0.783090981066118 | 1.66509869949014 | 9.69256105693986 | Italy |
| 2013 | 11.64704512243 | 0.785356877137869 | 1.62791492212755 | 10.0191302003025 | Italy |
| 2014 | 12.2507480058 | 0.787533956365705 | 1.61787196177117 | 10.6328760440288 | Italy |
| 2015 | 13.07901770413 | 0.789902118846972 | 1.62194021631286 | 11.4570774878171 | Italy |
| 2016 | 14.20472741362 | 0.792477244295977 | 1.5772866412375 | 12.6274407723825 | Italy |
| 2017 | 14.19229164863 | 0.795311186445406 | 1.57200393798604 | 12.620287710644 | Italy |
| 2018 | 13.83742183563 | 0.798362690372083 | 1.52936688092678 | 12.3080549547032 | Italy |
| 2019 | 11.47581719147 | 0.801530020572533 | 1.53470437719188 | 9.94111281427812 | Italy |
| 2020 | 10.73759403188 | 0.803635679966778 | 1.5232696888555 | 9.2143243430245 | Italy |
| 2021 | 10.33850012417 | 0.805773533613512 | 1.54702910482343 | 8.79147101934657 | Italy |
| 1990 | 8.64129406866 | 0.781051609326011 | 1.69991698939042 | 6.94137707926958 | Luxembourg |
| 1991 | 9.10908774372 | 0.785961442083159 | 1.62824765153333 | 7.48084009218667 | Luxembourg |
| 1992 | 9.04054990777 | 0.789053363703187 | 1.62130225593755 | 7.41924765183245 | Luxembourg |
| 1993 | 8.49489636979 | 0.792843836314663 | 1.56267602889558 | 6.93222034089442 | Luxembourg |
| 1994 | 8.22057965023 | 0.797824008094164 | 1.55253742060414 | 6.66804222962586 | Luxembourg |
| 1995 | 7.6309698848 | 0.802682469757881 | 1.52485821176884 | 6.10611167303116 | Luxembourg |
| 1996 | 7.92648496102 | 0.80651613780194 | 1.52029617978438 | 6.40618878123562 | Luxembourg |
| 1997 | 7.72932319915 | 0.810235288443831 | 1.54097659554232 | 6.18834660360768 | Luxembourg |
| 1998 | 8.3992557339 | 0.813889026223085 | 1.50633808483103 | 6.89291764906897 | Luxembourg |
| 1999 | 8.3616129474 | 0.817570995147661 | 1.49977944147966 | 6.86183350592034 | Luxembourg |
| 2000 | 8.42280642458 | 0.822006568181537 | 1.52711457812294 | 6.89569184645706 | Luxembourg |
| 2001 | 9.81590688706 | 0.825312816180712 | 1.48796607901589 | 8.32794080804411 | Luxembourg |
| 2002 | 10.1602394859 | 0.828223491084071 | 1.4775386358618 | 8.6827008500382 | Luxembourg |
| 2003 | 10.61629038699 | 0.830983945726346 | 1.47626439626475 | 9.14002599072525 | Luxembourg |
| 2004 | 10.55097836456 | 0.833672567288171 | 1.4920678981734 | 9.0589104663866 | Luxembourg |
| 2005 | 10.54597987068 | 0.836675263504864 | 1.47926583103638 | 9.06671403964362 | Luxembourg |
| 2006 | 10.96296244095 | 0.840574453017841 | 1.4677373708881 | 9.4952250700619 | Luxembourg |
| 2007 | 11.86378053347 | 0.84470700603675 | 1.46274996173646 | 10.4010305717335 | Luxembourg |
| 2008 | 10.96366030569 | 0.847978883152449 | 1.46914580943784 | 9.49451449625216 | Luxembourg |
| 2009 | 10.87218857896 | 0.850688725320833 | 1.453245314438 | 9.418943264522 | Luxembourg |
| 2010 | 11.71291911502 | 0.85325827666028 | 1.44879653575611 | 10.2641225792639 | Luxembourg |
| 2011 | 10.45225684045 | 0.856216833587911 | 1.42893353583045 | 9.02332330461955 | Luxembourg |
| 2012 | 8.78016239891 | 0.859789031199815 | 1.3983096204775 | 7.3818527784325 | Luxembourg |
| 2013 | 9.68327241667 | 0.863368792293471 | 1.42399527337796 | 8.25927714329204 | Luxembourg |
| 2014 | 9.21911585148 | 0.86669355608594 | 1.42255516856782 | 7.79656068291218 | Luxembourg |
| 2015 | 8.83484880403 | 0.87011293699584 | 1.39969528109615 | 7.43515352293385 | Luxembourg |
| 2016 | 9.57499056707 | 0.873420879802852 | 1.43577010513419 | 8.13922046193581 | Luxembourg |
| 2017 | 9.05608784095 | 0.876347684357551 | 1.3815349599702 | 7.6745528809798 | Luxembourg |
| 2018 | 9.79269584919 | 0.878637895036722 | 1.42706913329804 | 8.36562671589196 | Luxembourg |
| 2019 | 8.19001379187 | 0.880593895218451 | 1.38556095930148 | 6.80445283256852 | Luxembourg |
| 2020 | 7.82178390774 | 0.882495316094676 | 1.40351597859412 | 6.41826792914588 | Luxembourg |
| 2021 | 7.5222564999 | 0.884428955125693 | 1.38999162106359 | 6.13226487883641 | Luxembourg |
| 1990 | 4.75715162168 | 0.656504584147482 | 4.27618712878909 | 0.48096449289091 | Malta |
| 1991 | 5.00761374864 | 0.66111269147347 | 4.26242494165579 | 0.745188806984211 | Malta |
| 1992 | 5.08151019832 | 0.666324647620912 | 4.01165994485154 | 1.06985025346846 | Malta |
| 1993 | 5.13720052237 | 0.672120257474545 | 3.83898888089906 | 1.29821164147094 | Malta |
| 1994 | 5.2107413603 | 0.67784731358543 | 3.5841564385455 | 1.6265849217545 | Malta |
| 1995 | 5.37473518164 | 0.682617703672922 | 3.42992607028829 | 1.94480911135171 | Malta |
| 1996 | 5.40979991153 | 0.68662326099066 | 3.4431265366846 | 1.9666733748454 | Malta |
| 1997 | 5.48117611811 | 0.691756406092708 | 3.18590786141976 | 2.29526825669024 | Malta |
| 1998 | 5.61404269258 | 0.698095287002014 | 2.87817161979409 | 2.73587107278591 | Malta |
| 1999 | 5.76281195246 | 0.704734545351825 | 2.92071069910429 | 2.84210125335571 | Malta |
| 2000 | 5.94830024074 | 0.711805254226466 | 2.89199864955657 | 3.05630159118343 | Malta |
| 2001 | 6.12428129137 | 0.717803037638719 | 2.46454014515857 | 3.65974114621143 | Malta |
| 2002 | 6.36156734229 | 0.722790274731056 | 2.51025248768167 | 3.85131485460833 | Malta |
| 2003 | 6.65780533528 | 0.727511133046943 | 2.46547277918851 | 4.19233255609149 | Malta |
| 2004 | 6.97417912225 | 0.731933852781332 | 2.19216526996045 | 4.78201385228954 | Malta |
| 2005 | 7.34128848491 | 0.736188746914511 | 2.14952887019519 | 5.19175961471481 | Malta |
| 2006 | 7.77011922928 | 0.740140181028362 | 1.96796900057405 | 5.80215022870595 | Malta |
| 2007 | 8.20726742457 | 0.744237811910071 | 1.79902618895696 | 6.40824123561304 | Malta |
| 2008 | 8.44919079377 | 0.748427095431231 | 1.76555952193818 | 6.68363127183182 | Malta |
| 2009 | 8.47228501255 | 0.752171058957125 | 1.75034853737964 | 6.72193647517036 | Malta |
| 2010 | 8.43160420261 | 0.756250500159098 | 1.75922763805295 | 6.67237656455705 | Malta |
| 2011 | 8.09797786984 | 0.760310546511914 | 1.84632550642939 | 6.25165236341061 | Malta |
| 2012 | 7.96743186317 | 0.764537880070192 | 1.75110670307316 | 6.21632516009684 | Malta |
| 2013 | 7.8956326947 | 0.768616139641066 | 1.7315000972196 | 6.1641325974804 | Malta |
| 2014 | 7.76874862838 | 0.772728741377431 | 1.72623469037059 | 6.04251393800941 | Malta |
| 2015 | 7.74104350852 | 0.777276905450885 | 1.74553839787143 | 5.99550511064857 | Malta |
| 2016 | 7.46509992228 | 0.781673409574621 | 1.68119024629958 | 5.78390967598042 | Malta |
| 2017 | 7.00942438003 | 0.786410183585369 | 1.59064443483756 | 5.41877994519244 | Malta |
| 2018 | 6.41076506095 | 0.79096561824237 | 1.60110138944744 | 4.80966367150256 | Malta |
| 2019 | 6.05028521333 | 0.795197845728697 | 1.58084351111553 | 4.46944170221447 | Malta |
| 2020 | 5.60942658615 | 0.798375166241004 | 1.55477164218 | 4.05465494397 | Malta |
| 2021 | 5.49776964716 | 0.801585033998971 | 1.53893684698933 | 3.95883280017067 | Malta |
| 1990 | 7.30898403475 | 0.794612123302941 | 1.55184624080152 | 5.75713779394848 | Netherlands |
| 1991 | 7.44979768249 | 0.799285437176668 | 1.56219897350368 | 5.88759870898632 | Netherlands |
| 1992 | 7.69631128984 | 0.803965978232379 | 1.52165350208394 | 6.17465778775606 | Netherlands |
| 1993 | 7.84230844293 | 0.808278967039783 | 1.53448813688171 | 6.30782030604829 | Netherlands |
| 1994 | 7.87202294478 | 0.812530215874006 | 1.49627093214562 | 6.37575201263438 | Netherlands |
| 1995 | 8.00579537203 | 0.816662769214157 | 1.53147655312457 | 6.47431881890544 | Netherlands |
| 1996 | 8.26021680656 | 0.819984219587417 | 1.49096399535691 | 6.76925281120309 | Netherlands |
| 1997 | 8.48554741972 | 0.82288177029344 | 1.49026323633808 | 6.99528418338192 | Netherlands |
| 1998 | 8.83025172134 | 0.825882233724006 | 1.49660789317709 | 7.33364382816291 | Netherlands |
| 1999 | 9.29559871509 | 0.828693827126542 | 1.47407222963566 | 7.82152648545434 | Netherlands |
| 2000 | 9.95345560303 | 0.831913983967645 | 1.47751613622902 | 8.47593946680098 | Netherlands |
| 2001 | 10.62627971149 | 0.835518148042208 | 1.46213073399064 | 9.16414897749936 | Netherlands |
| 2002 | 11.28892676756 | 0.838676076741848 | 1.4723472456841 | 9.8165795218759 | Netherlands |
| 2003 | 11.90411723527 | 0.841639477052547 | 1.47547340284515 | 10.4286438324248 | Netherlands |
| 2004 | 12.49031211618 | 0.844807347619539 | 1.44496257060353 | 11.0453495455765 | Netherlands |
| 2005 | 13.556254093 | 0.847934189839563 | 1.45850049964019 | 12.0977535933598 | Netherlands |
| 2006 | 14.22007986485 | 0.850798807603514 | 1.45326190059432 | 12.7668179642557 | Netherlands |
| 2007 | 14.6788423795 | 0.853595391042959 | 1.44985415693644 | 13.2289882225636 | Netherlands |
| 2008 | 15.05470198631 | 0.8563684666871 | 1.41626785307133 | 13.6384341332387 | Netherlands |
| 2009 | 15.38094909677 | 0.858600459092211 | 1.40336060190545 | 13.9775884948645 | Netherlands |
| 2010 | 15.33241597611 | 0.861255297781524 | 1.41681632051721 | 13.9155996555928 | Netherlands |
| 2011 | 14.5783332691 | 0.864246678180707 | 1.42621191887086 | 13.1521213502291 | Netherlands |
| 2012 | 14.4816453989 | 0.866950626243189 | 1.40301253899378 | 13.0786328599062 | Netherlands |
| 2013 | 14.41019279898 | 0.8694029789072 | 1.40808560298611 | 13.0021071959939 | Netherlands |
| 2014 | 14.25301327556 | 0.871848914319002 | 1.41816304091418 | 12.8348502346458 | Netherlands |
| 2015 | 14.10288595362 | 0.874402841072396 | 1.41486285351244 | 12.6880231001076 | Netherlands |
| 2016 | 13.82465270888 | 0.876784051787216 | 1.4080828748361 | 12.4165698340439 | Netherlands |
| 2017 | 13.17187364951 | 0.87930015206581 | 1.40004183695383 | 11.7718318125562 | Netherlands |
| 2018 | 12.28939166676 | 0.881900218359353 | 1.38400695901667 | 10.9053847077433 | Netherlands |
| 2019 | 11.91441300087 | 0.884643839807538 | 1.3889385130034 | 10.5254744878666 | Netherlands |
| 2020 | 11.49342555258 | 0.886558566295774 | 1.3859551340966 | 10.1074704184834 | Netherlands |
| 2021 | 11.21271964058 | 0.888464256397732 | 1.3980549395573 | 9.8146647010227 | Netherlands |
| 1990 | 3.54830819755 | 0.795887277333644 | 1.54982062251216 | 1.99848757503784 | Norway |
| 1991 | 3.36225056418 | 0.800468885755463 | 1.552631345585 | 1.809619218595 | Norway |
| 1992 | 3.43149753858 | 0.805437473231399 | 1.52594916836677 | 1.90554837021323 | Norway |
| 1993 | 3.45858777572 | 0.810565689028095 | 1.52998374704083 | 1.92860402867917 | Norway |
| 1994 | 3.57463022271 | 0.815414668396239 | 1.49441025749514 | 2.08021996521486 | Norway |
| 1995 | 3.69863285146 | 0.820223522303065 | 1.47993045022484 | 2.21870240123516 | Norway |
| 1996 | 3.84966369835 | 0.825701668363619 | 1.48516228107517 | 2.36450141727483 | Norway |
| 1997 | 3.76516156302 | 0.831468770271884 | 1.50131759871576 | 2.26384396430424 | Norway |
| 1998 | 3.82129109183 | 0.836562809269057 | 1.47644859732162 | 2.34484249450838 | Norway |
| 1999 | 3.96156916205 | 0.841130236756497 | 1.46866331928658 | 2.49290584276342 | Norway |
| 2000 | 4.03031686161 | 0.846475451318379 | 1.46513798885864 | 2.56517887275136 | Norway |
| 2001 | 4.00251510856 | 0.852111935736606 | 1.43788623135632 | 2.56462887720368 | Norway |
| 2002 | 4.09032552841 | 0.856757888176133 | 1.4294132095675 | 2.6609123188425 | Norway |
| 2003 | 4.04140050978 | 0.860302576313355 | 1.41879740249537 | 2.62260310728463 | Norway |
| 2004 | 3.85904545294 | 0.863468908330188 | 1.41437156357579 | 2.44467388936421 | Norway |
| 2005 | 4.20799330356 | 0.866217487824484 | 1.42167893204444 | 2.78631437151556 | Norway |
| 2006 | 4.34214745506 | 0.868588434600934 | 1.45247457350475 | 2.88967288155525 | Norway |
| 2007 | 4.40815713997 | 0.870929669588489 | 1.41496273440806 | 2.99319440556194 | Norway |
| 2008 | 4.69023687163 | 0.873745121433024 | 1.39727727378909 | 3.29295959784091 | Norway |
| 2009 | 4.9909382469 | 0.876582817210667 | 1.45983299209233 | 3.53110525480767 | Norway |
| 2010 | 4.76224960139 | 0.880378381331352 | 1.42993989852742 | 3.33230970286258 | Norway |
| 2011 | 4.41868103577 | 0.884351666714408 | 1.40986508124538 | 3.00881595452462 | Norway |
| 2012 | 4.25311340136 | 0.888160475097858 | 1.41023379203919 | 2.84287960932081 | Norway |
| 2013 | 4.03902026087 | 0.891989617651275 | 1.35945075813289 | 2.6795695027371 | Norway |
| 2014 | 3.86871633569 | 0.895499545979859 | 1.3898986173445 | 2.4788177183455 | Norway |
| 2015 | 3.75446515748 | 0.89891264398603 | 1.382916398675 | 2.371548758805 | Norway |
| 2016 | 3.505831107 | 0.902584186845854 | 1.370894246625 | 2.134936860375 | Norway |
| 2017 | 3.63606995422 | 0.906044048809343 | 1.3936837285159 | 2.2423862257041 | Norway |
| 2018 | 3.86068372218 | 0.909261782035669 | 1.40676040862978 | 2.45392331355022 | Norway |
| 2019 | 3.96879880423 | 0.912278760654598 | 1.39178620817378 | 2.57701259605622 | Norway |
| 2020 | 3.64189919357 | 0.914529920283541 | 1.40772129291357 | 2.23417790065643 | Norway |
| 2021 | 3.62277457401 | 0.916132810119013 | 1.40184185719067 | 2.22093271681933 | Norway |
| 1990 | 15.64894580087 | 0.599777756742926 | 8.72995740987423 | 6.91898839099577 | Portugal |
| 1991 | 13.61293163583 | 0.607269448643355 | 9.02443542612034 | 4.58849620970966 | Portugal |
| 1992 | 12.80247432016 | 0.614808079780387 | 8.81925115477357 | 3.98322316538643 | Portugal |
| 1993 | 13.39259151238 | 0.622101176817746 | 8.99593271080391 | 4.39665880157609 | Portugal |
| 1994 | 10.36009739485 | 0.629306386686867 | 8.19544040600468 | 2.16465698884532 | Portugal |
| 1995 | 9.23694765863 | 0.635498761462007 | 6.2603560073086 | 2.9765916513214 | Portugal |
| 1996 | 10.43294198369 | 0.640864525833465 | 6.26012432414917 | 4.17281765954083 | Portugal |
| 1997 | 10.63099909361 | 0.64619290440225 | 6.33966854492255 | 4.29133054868745 | Portugal |
| 1998 | 10.36241869984 | 0.651335993931501 | 5.44442640322617 | 4.91799229661383 | Portugal |
| 1999 | 9.07910743361 | 0.656120666715453 | 4.891232640585 | 4.187874793025 | Portugal |
| 2000 | 8.26450704848 | 0.66161085244449 | 4.52077493052328 | 3.74373211795672 | Portugal |
| 2001 | 8.13967933896 | 0.667247150668184 | 3.78591994400378 | 4.35375939495622 | Portugal |
| 2002 | 7.18011520197 | 0.67258393952723 | 3.85583052006458 | 3.32428468190542 | Portugal |
| 2003 | 6.59824153085 | 0.677663411506582 | 3.56175465114023 | 3.03648687970977 | Portugal |
| 2004 | 6.37199977484 | 0.682352926478464 | 3.52522286628568 | 2.84677690855432 | Portugal |
| 2005 | 6.48573173144 | 0.686887104684271 | 3.29852094861109 | 3.18721078282891 | Portugal |
| 2006 | 6.60799831251 | 0.691158795071141 | 3.10518984199674 | 3.50280847051326 | Portugal |
| 2007 | 7.11109129817 | 0.694974852517783 | 3.14616636648588 | 3.96492493168412 | Portugal |
| 2008 | 7.24278415901 | 0.69860459852573 | 2.80754181659852 | 4.43524234241148 | Portugal |
| 2009 | 7.40452018021 | 0.702223398701003 | 2.9793833172597 | 4.4251368629503 | Portugal |
| 2010 | 6.62153117808 | 0.706563554661067 | 2.95083680176967 | 3.67069437631033 | Portugal |
| 2011 | 6.28716494195 | 0.71126289291359 | 2.68424155266432 | 3.60292338928568 | Portugal |
| 2012 | 6.31627349349 | 0.715804084405661 | 2.54610530550923 | 3.77016818798077 | Portugal |
| 2013 | 5.95032333599 | 0.719836991474169 | 2.70258289807286 | 3.24774043791714 | Portugal |
| 2014 | 5.82804274264 | 0.723021281895034 | 2.42072773706936 | 3.40731500557064 | Portugal |
| 2015 | 6.09661533954 | 0.725756220353222 | 2.43822285765163 | 3.65839248188837 | Portugal |
| 2016 | 6.3366292077 | 0.728300849268768 | 2.28062137780889 | 4.05600782989111 | Portugal |
| 2017 | 6.40769069512 | 0.73122553459241 | 2.10505939241406 | 4.30263130270594 | Portugal |
| 2018 | 6.25191249212 | 0.73440294716366 | 1.98644282271289 | 4.26546966940711 | Portugal |
| 2019 | 5.76037463448 | 0.738090480746574 | 2.13780900084925 | 3.62256563363075 | Portugal |
| 2020 | 5.03595177483 | 0.741037384212082 | 1.78873272922095 | 3.24721904560905 | Portugal |
| 2021 | 4.79498960838 | 0.744151850665712 | 1.91795730377483 | 2.87703230460517 | Portugal |
| 1990 | 6.78588776768 | 0.63667316626401 | 6.20485903215739 | 0.581028735522609 | Spain |
| 1991 | 6.59108712429 | 0.644130744620668 | 5.48489336702195 | 1.10619375726805 | Spain |
| 1992 | 6.7045142922 | 0.65159759683081 | 5.1082856764825 | 1.5962286157175 | Spain |
| 1993 | 6.51687764213 | 0.658637568907277 | 4.32604911400558 | 2.19082852812442 | Spain |
| 1994 | 6.19494973561 | 0.665321549207614 | 4.20651609273479 | 1.98843364287521 | Spain |
| 1995 | 6.14833976967 | 0.671576034104742 | 3.78967317956595 | 2.35866659010405 | Spain |
| 1996 | 6.15873450056 | 0.677088943314314 | 3.56579422364115 | 2.59294027691885 | Spain |
| 1997 | 6.08264295501 | 0.68242949710256 | 3.55302448413433 | 2.52961847087567 | Spain |
| 1998 | 6.14143276868 | 0.687490716991391 | 3.31566264215 | 2.82577012653 | Spain |
| 1999 | 6.0898386222 | 0.692245196919437 | 3.12869334775261 | 2.96114527444739 | Spain |
| 2000 | 6.37812716791 | 0.697056031108428 | 3.05124586214709 | 3.32688130576291 | Spain |
| 2001 | 6.43776261704 | 0.701726069724186 | 2.8710900046382 | 3.56667261240179 | Spain |
| 2002 | 6.6355282366 | 0.706309654864074 | 2.99053783313386 | 3.64499040346614 | Spain |
| 2003 | 6.90107496069 | 0.710678584323191 | 2.79293035866857 | 4.10814460202143 | Spain |
| 2004 | 7.17431025328 | 0.714886229907558 | 2.62513432707372 | 4.54917592620628 | Spain |
| 2005 | 7.45060627788 | 0.718717288882423 | 2.55952145950302 | 4.89108481837698 | Spain |
| 2006 | 8.05039898131 | 0.721970128302216 | 2.52754164462447 | 5.52285733668553 | Spain |
| 2007 | 8.6533965589 | 0.725211442824273 | 2.43731211316667 | 6.21608444573333 | Spain |
| 2008 | 8.53953981859 | 0.729411041261585 | 2.29228596686951 | 6.24725385172049 | Spain |
| 2009 | 8.39884171321 | 0.733956054531859 | 2.07419550784182 | 6.32464620536818 | Spain |
| 2010 | 7.98504285037 | 0.738352055066564 | 2.0598737366934 | 5.9251691136766 | Spain |
| 2011 | 7.48743429977 | 0.742096450004589 | 1.92828158334711 | 5.5591527164229 | Spain |
| 2012 | 7.69710867885 | 0.745181867042751 | 1.75219109017074 | 5.94491758867926 | Spain |
| 2013 | 7.84871287396 | 0.747651982075541 | 1.91144966876786 | 5.93726320519214 | Spain |
| 2014 | 8.44787873588 | 0.74980092875112 | 1.84040411428055 | 6.60747462159945 | Spain |
| 2015 | 8.67974216632 | 0.752269822995419 | 1.7294050548813 | 6.9503371114387 | Spain |
| 2016 | 8.4588051797 | 0.75475793948614 | 1.75534831410161 | 6.70345686559839 | Spain |
| 2017 | 7.92914752445 | 0.757663276271395 | 1.76114074714838 | 6.16800677730162 | Spain |
| 2018 | 7.14769481683 | 0.760659574783565 | 1.743700500925 | 5.403994315905 | Spain |
| 2019 | 6.73848474435 | 0.763942918439527 | 1.79151023545308 | 4.94697450889692 | Spain |
| 2020 | 6.26485790111 | 0.766506025058573 | 1.77947297704897 | 4.48538492406103 | Spain |
| 2021 | 6.18031183009 | 0.769283697979998 | 1.77847990866838 | 4.40183192142162 | Spain |
| 1990 | 1.53852069145 | 0.78553579188163 | 1.52613120014056 | 0.0123894913094444 | Sweden |
| 1991 | 1.50371142825 | 0.790096440686927 | 1.49183371449 | 0.0118777137599999 | Sweden |
| 1992 | 1.49244183942 | 0.795431457884408 | 1.485411818905 | 0.00703002051500001 | Sweden |
| 1993 | 1.48760065945 | 0.801009521825474 | 1.47948349790217 | 0.00811716154782616 | Sweden |
| 1994 | 1.4271250269 | 0.806700264491967 | 1.4271250269 | 0 | Sweden |
| 1995 | 1.41818613588 | 0.81224465438799 | 1.41818613588 | 0 | Sweden |
| 1996 | 1.42401207394 | 0.817227025218912 | 1.42401207394 | 0 | Sweden |
| 1997 | 1.43748265107 | 0.82169405939303 | 1.43338676453 | 0.00409588654000004 | Sweden |
| 1998 | 1.50299812975 | 0.825731994976955 | 1.46025691859667 | 0.0427412111533332 | Sweden |
| 1999 | 1.55968014137 | 0.82948709884742 | 1.49171384439269 | 0.0679662969773078 | Sweden |
| 2000 | 1.65028912389 | 0.833028111989963 | 1.467729354875 | 0.182559769015 | Sweden |
| 2001 | 1.8709214476 | 0.835956398134875 | 1.48210624145579 | 0.38881520614421 | Sweden |
| 2002 | 2.00849077851 | 0.838741526580818 | 1.46032500449435 | 0.548165774015652 | Sweden |
| 2003 | 2.15354178928 | 0.841663138093711 | 1.462176023715 | 0.691365765565 | Sweden |
| 2004 | 2.07575652821 | 0.844506881898062 | 1.49438504191478 | 0.581371486295217 | Sweden |
| 2005 | 2.15762980116 | 0.846876343860947 | 1.4788890775737 | 0.678740723586296 | Sweden |
| 2006 | 2.24746319155 | 0.849115871424855 | 1.43785115364692 | 0.809612037903077 | Sweden |
| 2007 | 2.35167644782 | 0.851420847176242 | 1.41454391341409 | 0.937132534405909 | Sweden |
| 2008 | 2.40250308243 | 0.853705850789255 | 1.43514645389036 | 0.967356628539643 | Sweden |
| 2009 | 2.78508565752 | 0.85570274765727 | 1.42061058311115 | 1.36447507440885 | Sweden |
| 2010 | 2.91863115079 | 0.858727940541151 | 1.42610055149606 | 1.49253059929394 | Sweden |
| 2011 | 2.81713133077 | 0.861892379268929 | 1.42904368595 | 1.38808764482 | Sweden |
| 2012 | 2.89563427268 | 0.864706671533252 | 1.38486387305313 | 1.51077039962688 | Sweden |
| 2013 | 2.8987141501 | 0.867383767613666 | 1.44577750959795 | 1.45293664050205 | Sweden |
| 2014 | 2.72026338461 | 0.869923325091982 | 1.41585535063814 | 1.30440803397186 | Sweden |
| 2015 | 2.76941867491 | 0.87238851487302 | 1.41736426529718 | 1.35205440961282 | Sweden |
| 2016 | 2.83025072671 | 0.874661654943114 | 1.40278516332192 | 1.42746556338808 | Sweden |
| 2017 | 2.7707692637 | 0.877120611340732 | 1.41788107553171 | 1.35288818816829 | Sweden |
| 2018 | 2.68259166124 | 0.879888911893351 | 1.44387765788833 | 1.23871400335167 | Sweden |
| 2019 | 2.60113379686 | 0.882984115679233 | 1.37732267818769 | 1.22381111867231 | Sweden |
| 2020 | 2.52558197811 | 0.88506201036603 | 1.37383872291417 | 1.15174325519583 | Sweden |
| 2021 | 2.37753159705 | 0.886880298870342 | 1.41154337500379 | 0.965988222046207 | Sweden |
| 1990 | 11.4213670659 | 0.862766843874175 | 1.41447747073759 | 10.0068895951624 | Switzerland |
| 1991 | 11.72950068291 | 0.864796867138941 | 1.40293278307094 | 10.3265678998391 | Switzerland |
| 1992 | 11.70596664461 | 0.86773607877979 | 1.42084445363615 | 10.2851221909738 | Switzerland |
| 1993 | 11.42593449828 | 0.870863557610096 | 1.4128727954813 | 10.0130617027987 | Switzerland |
| 1994 | 10.99167619841 | 0.873140871296934 | 1.38162703098242 | 9.61004916742758 | Switzerland |
| 1995 | 10.63795117502 | 0.874835330674703 | 1.43291268353442 | 9.20503849148558 | Switzerland |
| 1996 | 10.59209954606 | 0.876640556809649 | 1.39298489823439 | 9.19911464782561 | Switzerland |
| 1997 | 10.78384388989 | 0.87848554526934 | 1.40381179012 | 9.38003209977 | Switzerland |
| 1998 | 11.07193256606 | 0.880035152091359 | 1.38466542255462 | 9.68726714350538 | Switzerland |
| 1999 | 11.38188388928 | 0.882045586320071 | 1.37894035304822 | 10.0029435362318 | Switzerland |
| 2000 | 11.9490791126 | 0.885243530890309 | 1.40155821038422 | 10.5475209022158 | Switzerland |
| 2001 | 12.24128346078 | 0.888445951179249 | 1.39551396093056 | 10.8457694998494 | Switzerland |
| 2002 | 12.4242041532 | 0.890902811034425 | 1.38141690726127 | 11.0427872459387 | Switzerland |
| 2003 | 12.95276208588 | 0.892780807435311 | 1.38932817755968 | 11.5634339083203 | Switzerland |
| 2004 | 13.54279160708 | 0.894909812216857 | 1.40479106502277 | 12.1380005420572 | Switzerland |
| 2005 | 14.1686638218 | 0.897272629528173 | 1.38699181024118 | 12.7816720115588 | Switzerland |
| 2006 | 15.02940546782 | 0.899962304608863 | 1.40319905647746 | 13.6262064113425 | Switzerland |
| 2007 | 15.13277473259 | 0.902913412619355 | 1.38519386507291 | 13.7475808675171 | Switzerland |
| 2008 | 15.12685857055 | 0.90577845443162 | 1.40262903232365 | 13.7242295382264 | Switzerland |
| 2009 | 15.10810509953 | 0.908049997864177 | 1.38750636828262 | 13.7205987312474 | Switzerland |
| 2010 | 14.55598564273 | 0.910530522112102 | 1.37442983259321 | 13.1815558101368 | Switzerland |
| 2011 | 13.8746389132 | 0.91327657336798 | 1.3880594745313 | 12.4865794386687 | Switzerland |
| 2012 | 13.61171471603 | 0.91571489343801 | 1.40678920862194 | 12.2049255074081 | Switzerland |
| 2013 | 13.63932541085 | 0.917955005608177 | 1.40891641688947 | 12.2304089939605 | Switzerland |
| 2014 | 13.47891858126 | 0.919994182026162 | 1.39786131067917 | 12.0810572705808 | Switzerland |
| 2015 | 13.4206456863 | 0.921832990505373 | 1.37662761687889 | 12.0440180694211 | Switzerland |
| 2016 | 12.69807963291 | 0.923930598455048 | 1.38565911668317 | 11.3124205162268 | Switzerland |
| 2017 | 12.27892521386 | 0.926202291104072 | 1.36299563692473 | 10.9159295769353 | Switzerland |
| 2018 | 11.33605106281 | 0.928550590010717 | 1.37983487551286 | 9.95621618729714 | Switzerland |
| 2019 | 10.61793297647 | 0.930682101951976 | 1.39681664540118 | 9.22111633106882 | Switzerland |
| 2020 | 10.31213466055 | 0.932027644512362 | 1.39829508090574 | 8.91383957964426 | Switzerland |
| 2021 | 10.07656672952 | 0.933059110986638 | 1.38857028769081 | 8.68799644182919 | Switzerland |
| 1990 | 6.6390581165 | 0.744334126300131 | 1.73523791095526 | 4.90382020554474 | United Kingdom |
| 1991 | 6.63779314617 | 0.749148231365388 | 1.86550789714163 | 4.77228524902837 | United Kingdom |
| 1992 | 6.81130842427 | 0.754556689127024 | 1.77735037444295 | 5.03395804982705 | United Kingdom |
| 1993 | 7.34889057577 | 0.760154350107152 | 1.79078232279263 | 5.55810825297737 | United Kingdom |
| 1994 | 7.66455459794 | 0.765164600469893 | 1.81688720487018 | 5.84766739306982 | United Kingdom |
| 1995 | 8.10064109909 | 0.768656863963456 | 1.71644404254395 | 6.38419705654605 | United Kingdom |
| 1996 | 8.8232836225 | 0.771576484075751 | 1.80678174202775 | 7.01650188047225 | United Kingdom |
| 1997 | 9.42523669613 | 0.775050121968743 | 1.76586437543054 | 7.65937232069946 | United Kingdom |
| 1998 | 10.15954916176 | 0.779057165114363 | 1.73675554390275 | 8.42279361785725 | United Kingdom |
| 1999 | 10.94686488105 | 0.784036475142408 | 1.663784590885 | 9.283080290165 | United Kingdom |
| 2000 | 11.69321766696 | 0.789218959863246 | 1.618385267145 | 10.074832399815 | United Kingdom |
| 2001 | 12.42552445618 | 0.793513165354113 | 1.57223866340071 | 10.8532857927793 | United Kingdom |
| 2002 | 13.2314319756 | 0.796655559574601 | 1.55619641232433 | 11.6752355632757 | United Kingdom |
| 2003 | 14.99613574708 | 0.799186111437993 | 1.56688159384866 | 13.4292541532313 | United Kingdom |
| 2004 | 16.91290054017 | 0.801950870000537 | 1.52597343425733 | 15.3869271059127 | United Kingdom |
| 2005 | 21.09328976283 | 0.804489193801038 | 1.5325134094631 | 19.5607763533669 | United Kingdom |
| 2006 | 27.11865763899 | 0.806817045960102 | 1.55053510801421 | 25.5681225309758 | United Kingdom |
| 2007 | 28.13020690204 | 0.80935277163371 | 1.526753025566 | 26.603453876474 | United Kingdom |
| 2008 | 23.42723773339 | 0.812039287236426 | 1.5113808976286 | 21.9158568357614 | United Kingdom |
| 2009 | 18.67620094855 | 0.814793642346203 | 1.5136541378773 | 17.1625468106727 | United Kingdom |
| 2010 | 15.2463444324 | 0.818033993642746 | 1.50121947099651 | 13.7451249614035 | United Kingdom |
| 2011 | 13.45373522063 | 0.821718995421102 | 1.48195992975453 | 11.9717752908755 | United Kingdom |
| 2012 | 11.83423014767 | 0.826453754133025 | 1.47574449513062 | 10.3584856525394 | United Kingdom |
| 2013 | 10.56166290261 | 0.831508668228002 | 1.48124186422288 | 9.08042103838712 | United Kingdom |
| 2014 | 9.59773835567 | 0.835516642361539 | 1.47690217246566 | 8.12083618320434 | United Kingdom |
| 2015 | 9.10795900448 | 0.838951918781599 | 1.45886859862077 | 7.64909040585923 | United Kingdom |
| 2016 | 8.26159415742 | 0.842460232628565 | 1.46520214013412 | 6.79639201728588 | United Kingdom |
| 2017 | 7.11389005704 | 0.846263617759747 | 1.45751844620262 | 5.65637161083738 | United Kingdom |
| 2018 | 6.23786042845 | 0.849969787501756 | 1.44943210716647 | 4.78842832128353 | United Kingdom |
| 2019 | 5.95457340125 | 0.854130572600886 | 1.45441898067545 | 4.50015442057455 | United Kingdom |
| 2020 | 5.07628865402 | 0.856920290432659 | 1.44514902739778 | 3.63113962662222 | United Kingdom |
| 2021 | 5.10977512913 | 0.859000181772086 | 1.41880208032972 | 3.69097304880028 | United Kingdom |
| 1990 | 168.90440840187 | 0.587397284272094 | 8.96253593330147 | 159.941872468569 | Argentina |
| 1991 | 144.25829081368 | 0.591707540464012 | 9.07225409514845 | 135.186036718532 | Argentina |
| 1992 | 116.73082084016 | 0.597996324377555 | 8.94865007355962 | 107.7821707666 | Argentina |
| 1993 | 96.40755566122 | 0.603739312309972 | 8.67686333064069 | 87.7306923305793 | Argentina |
| 1994 | 88.56496772052 | 0.609787330316145 | 8.63237969764062 | 79.9325880228794 | Argentina |
| 1995 | 91.23716563106 | 0.614473705638113 | 8.68735758692492 | 82.5498080441351 | Argentina |
| 1996 | 89.50286974154 | 0.618942623582172 | 9.04669194856104 | 80.456177792979 | Argentina |
| 1997 | 96.40191994515 | 0.623934408870184 | 8.86891913216424 | 87.5330008129858 | Argentina |
| 1998 | 91.9254773387 | 0.6287234356344 | 8.027736894674 | 83.897740444026 | Argentina |
| 1999 | 79.71954483018 | 0.633175129405293 | 6.79899023484324 | 72.9205545953368 | Argentina |
| 2000 | 70.03152442709 | 0.638194471801002 | 6.61069552802176 | 63.4208288990682 | Argentina |
| 2001 | 68.27970086315 | 0.642175704746832 | 6.35658069739804 | 61.923120165752 | Argentina |
| 2002 | 73.45036522856 | 0.644472506550469 | 6.1736807657905 | 67.2766844627695 | Argentina |
| 2003 | 66.35406406162 | 0.645906577030896 | 5.83792894135043 | 60.5161351202696 | Argentina |
| 2004 | 55.91116026397 | 0.648296828210031 | 5.89910816002239 | 50.0120521039476 | Argentina |
| 2005 | 52.46848546295 | 0.653368829991767 | 5.05020207605879 | 47.4182833868912 | Argentina |
| 2006 | 51.57291801515 | 0.657445899915566 | 4.78999495069661 | 46.7829230644534 | Argentina |
| 2007 | 49.29432566071 | 0.660187575972401 | 4.33707630105646 | 44.9572493596535 | Argentina |
| 2008 | 50.31185295042 | 0.6630792353937 | 4.49649713622426 | 45.8153558141957 | Argentina |
| 2009 | 50.24783397796 | 0.665911944148118 | 4.14453956707246 | 46.1032944108875 | Argentina |
| 2010 | 50.03788190067 | 0.669925880151809 | 3.87403971321475 | 46.1638421874553 | Argentina |
| 2011 | 46.39412956444 | 0.675242697368294 | 3.63209163625853 | 42.7620379281815 | Argentina |
| 2012 | 40.82483500613 | 0.679218239775544 | 3.60322612769916 | 37.2216088784308 | Argentina |
| 2013 | 40.7946479469 | 0.681633214735039 | 3.58235406188298 | 37.212293885017 | Argentina |
| 2014 | 40.45544617019 | 0.684974509115572 | 3.30042856681711 | 37.1550176033729 | Argentina |
| 2015 | 36.17029109739 | 0.691710125973835 | 3.18890125196884 | 32.9813898454212 | Argentina |
| 2016 | 32.51540507448 | 0.698618031454613 | 2.89400364883868 | 29.6214014256413 | Argentina |
| 2017 | 28.61819376617 | 0.705135528765331 | 2.85293720542828 | 25.7652565607417 | Argentina |
| 2018 | 24.07577976176 | 0.712888693454577 | 2.64609796293271 | 21.4296817988273 | Argentina |
| 2019 | 22.89940546166 | 0.719002842751183 | 2.53883945566963 | 20.3605660059904 | Argentina |
| 2020 | 20.39496594606 | 0.721294302628992 | 2.54204888904857 | 17.8529170570114 | Argentina |
| 2021 | 19.54592360631 | 0.723122972582249 | 2.41128384959295 | 17.1346397567171 | Argentina |
| 1990 | 99.9195197758 | 0.586495099638694 | 8.84178213539718 | 91.0777376404028 | Chile |
| 1991 | 84.3497107563 | 0.593006580479257 | 8.89863153948731 | 75.4510792168127 | Chile |
| 1992 | 70.38631883028 | 0.599106083381125 | 8.98253490492746 | 61.4037839253525 | Chile |
| 1993 | 57.12401684914 | 0.604828509357091 | 8.57966188194946 | 48.5443549671905 | Chile |
| 1994 | 52.46417100292 | 0.61067393285511 | 8.95346959936175 | 43.5107014035583 | Chile |
| 1995 | 53.55948130625 | 0.617234676665645 | 8.71286389064083 | 44.8466174156092 | Chile |
| 1996 | 53.72698441971 | 0.623694459543547 | 8.85369469691541 | 44.8732897227946 | Chile |
| 1997 | 51.54384306615 | 0.630540084786116 | 8.279839157344 | 43.264003908806 | Chile |
| 1998 | 48.95479333194 | 0.638082758454141 | 6.48747611359351 | 42.4673172183465 | Chile |
| 1999 | 44.90499536075 | 0.645928084390867 | 6.27769269952083 | 38.6273026612292 | Chile |
| 2000 | 40.68058579547 | 0.652989027528663 | 4.94051305134474 | 35.7400727441253 | Chile |
| 2001 | 38.36828281975 | 0.660265510120952 | 4.37227478848013 | 33.9960080312699 | Chile |
| 2002 | 38.06832466346 | 0.667888408106028 | 3.82359231338568 | 34.2447323500743 | Chile |
| 2003 | 36.96718708181 | 0.673445927084645 | 3.83406459291537 | 33.1331224888946 | Chile |
| 2004 | 34.2686587107 | 0.678258212006936 | 3.61097861657216 | 30.6576800941278 | Chile |
| 2005 | 30.58849204894 | 0.682589191171846 | 3.38059184339274 | 27.2079002055473 | Chile |
| 2006 | 28.2344235321 | 0.685892748248682 | 3.37703695318306 | 24.8573865789169 | Chile |
| 2007 | 26.29493589358 | 0.688930323304188 | 3.38286409849139 | 22.9120717950886 | Chile |
| 2008 | 26.68735077274 | 0.692359787453618 | 3.19966648685705 | 23.487684285883 | Chile |
| 2009 | 26.51521740937 | 0.696602140685353 | 3.15391001135426 | 23.3613073980157 | Chile |
| 2010 | 24.39632168241 | 0.702304703544999 | 2.97996083110735 | 21.4163608513026 | Chile |
| 2011 | 23.1952306893 | 0.708597935629908 | 2.81409193592279 | 20.3811387533772 | Chile |
| 2012 | 21.93292635504 | 0.714743477340726 | 2.76203252536896 | 19.170893829671 | Chile |
| 2013 | 21.39027748486 | 0.720448781307689 | 2.56121429600641 | 18.8290631888536 | Chile |
| 2014 | 23.58640263068 | 0.727275926904869 | 2.43428131114324 | 21.1521213195368 | Chile |
| 2015 | 21.91137358065 | 0.736098082475337 | 2.02310882231161 | 19.8882647583384 | Chile |
| 2016 | 19.99693286247 | 0.744972224319701 | 1.842849775695 | 18.154083086775 | Chile |
| 2017 | 20.54624772771 | 0.752058077213427 | 1.87328750968082 | 18.6729602180292 | Chile |
| 2018 | 20.26032915151 | 0.758625713610628 | 1.77431068395738 | 18.4860184675526 | Chile |
| 2019 | 19.56173746139 | 0.765120322816907 | 1.81582336341871 | 17.7459140979713 | Chile |
| 2020 | 16.38011072029 | 0.769213676196926 | 1.71434254487634 | 14.6657681754137 | Chile |
| 2021 | 15.53829226118 | 0.771514715903929 | 1.78337830091797 | 13.754913960262 | Chile |
| 1990 | 174.34898384948 | 0.58192185512198 | 8.89687776893328 | 165.452106080547 | Uruguay |
| 1991 | 156.93445505295 | 0.586156118239456 | 8.94011682840513 | 147.994338224545 | Uruguay |
| 1992 | 126.35685301043 | 0.589822355398741 | 8.94640395243177 | 117.410449057998 | Uruguay |
| 1993 | 121.90089681747 | 0.592295025274764 | 9.20367753626328 | 112.697219281207 | Uruguay |
| 1994 | 122.45589970285 | 0.595188424402801 | 8.66808784628419 | 113.787811856566 | Uruguay |
| 1995 | 131.70494571951 | 0.596825409130546 | 8.91254609989657 | 122.792399619613 | Uruguay |
| 1996 | 132.50854403009 | 0.5997751037831 | 8.98157074906054 | 123.526973281029 | Uruguay |
| 1997 | 138.85250374466 | 0.605665051157748 | 8.92035717770438 | 129.932146566956 | Uruguay |
| 1998 | 124.1690816085 | 0.612700815160604 | 9.02261496348455 | 115.146466645015 | Uruguay |
| 1999 | 106.61447984834 | 0.619009271243139 | 8.89480440686648 | 97.7196754414735 | Uruguay |
| 2000 | 92.28250008039 | 0.623208290785701 | 9.0198086638072 | 83.2626914165828 | Uruguay |
| 2001 | 84.74612109648 | 0.62579606753003 | 8.80153588872273 | 75.9445852077573 | Uruguay |
| 2002 | 86.98655474678 | 0.628285799994443 | 8.05508985432319 | 78.9314648924568 | Uruguay |
| 2003 | 89.05617825501 | 0.631437773906681 | 8.18940670613853 | 80.8667715488715 | Uruguay |
| 2004 | 79.11840677515 | 0.634216906381842 | 6.76302584710955 | 72.3553809280405 | Uruguay |
| 2005 | 69.78381516512 | 0.63697576957158 | 6.84635673104387 | 62.9374584340761 | Uruguay |
| 2006 | 57.71282867368 | 0.640590696178162 | 6.76346427822757 | 50.9493643954524 | Uruguay |
| 2007 | 59.32300438551 | 0.64633171377937 | 6.10111224580255 | 53.2218921397074 | Uruguay |
| 2008 | 57.25914099573 | 0.653525522497867 | 5.10949626981232 | 52.1496447259177 | Uruguay |
| 2009 | 56.18240757973 | 0.660321134636261 | 4.49640170024026 | 51.6860058794897 | Uruguay |
| 2010 | 59.50146654249 | 0.666099135301691 | 4.02443240245478 | 55.4770341400352 | Uruguay |
| 2011 | 56.17852707114 | 0.667079733158413 | 3.94698465858545 | 52.2315424125545 | Uruguay |
| 2012 | 51.93832860361 | 0.665802191838499 | 4.20042790867926 | 47.7379006949307 | Uruguay |
| 2013 | 50.71751025876 | 0.668228360836938 | 3.84592993124306 | 46.8715803275169 | Uruguay |
| 2014 | 43.88397711841 | 0.673334260363154 | 3.8516677639425 | 40.0323093544675 | Uruguay |
| 2015 | 39.51750650367 | 0.680595506073599 | 3.52473915904492 | 35.9927673446251 | Uruguay |
| 2016 | 37.04848460112 | 0.689816733014055 | 3.36720599176087 | 33.6812786093591 | Uruguay |
| 2017 | 34.02748515892 | 0.699189455881847 | 2.86964962633987 | 31.1578355325801 | Uruguay |
| 2018 | 33.17837716562 | 0.706904930126484 | 2.75824854543678 | 30.4201286201832 | Uruguay |
| 2019 | 32.66685766534 | 0.712536588078123 | 2.54660671615034 | 30.1202509491897 | Uruguay |
| 2020 | 31.64932338013 | 0.716169766093523 | 2.58980939608392 | 29.0595139840461 | Uruguay |
| 2021 | 29.62774292194 | 0.719283444839432 | 2.60376080195788 | 27.0239821199821 | Uruguay |
| 1990 | 5.36552711782 | 0.781977864427607 | 1.65902892463083 | 3.70649819318917 | Canada |
| 1991 | 5.36105188486 | 0.784159360080438 | 1.66221243829769 | 3.69883944656231 | Canada |
| 1992 | 5.26305517309 | 0.787030536551619 | 1.64985992575071 | 3.61319524733929 | Canada |
| 1993 | 5.69293772602 | 0.789716593022703 | 1.65732619117754 | 4.03561153484246 | Canada |
| 1994 | 5.65248122628 | 0.79259851803789 | 1.57453572328897 | 4.07794550299103 | Canada |
| 1995 | 5.69024683073 | 0.796521165940602 | 1.52833057259442 | 4.16191625813558 | Canada |
| 1996 | 5.64370864521 | 0.801064970257054 | 1.54092978683346 | 4.10277885837654 | Canada |
| 1997 | 5.76090666689 | 0.804833356387694 | 1.53365143339343 | 4.22725523349657 | Canada |
| 1998 | 6.01653225759 | 0.808135440512777 | 1.52729223412209 | 4.48924002346791 | Canada |
| 1999 | 6.71529681941 | 0.812265940092679 | 1.496871143805 | 5.218425675605 | Canada |
| 2000 | 7.72381778712 | 0.816685059408778 | 1.51606865082077 | 6.20774913629923 | Canada |
| 2001 | 8.57089779769 | 0.820696250274983 | 1.51472528282618 | 7.05617251486382 | Canada |
| 2002 | 9.87271829668 | 0.824402271645546 | 1.49431739609533 | 8.37840090058467 | Canada |
| 2003 | 10.93495995279 | 0.827801096249711 | 1.47495077136696 | 9.46000918142304 | Canada |
| 2004 | 12.3239577911 | 0.830928109937158 | 1.46629934943638 | 10.8576584416636 | Canada |
| 2005 | 13.76654175813 | 0.833288293645254 | 1.48094230884702 | 12.285599449283 | Canada |
| 2006 | 14.42315438182 | 0.834915660275685 | 1.47238992790556 | 12.9507644539144 | Canada |
| 2007 | 14.97043514448 | 0.836625096194775 | 1.48172464293633 | 13.4887105015437 | Canada |
| 2008 | 15.04017807675 | 0.838913684557505 | 1.47212181900707 | 13.5680562577429 | Canada |
| 2009 | 14.57074402909 | 0.841583670650067 | 1.4650563256519 | 13.1056877034381 | Canada |
| 2010 | 14.36364743981 | 0.844853488930283 | 1.47190290980156 | 12.8917445300084 | Canada |
| 2011 | 13.64165097296 | 0.848017833149098 | 1.45976932471016 | 12.1818816482498 | Canada |
| 2012 | 13.17967281124 | 0.850978048108266 | 1.45824151721557 | 11.7214312940244 | Canada |
| 2013 | 12.82144718825 | 0.853844945122214 | 1.4463569854425 | 11.3750902028075 | Canada |
| 2014 | 11.69926273534 | 0.856759933938005 | 1.44722241774767 | 10.2520403175923 | Canada |
| 2015 | 11.05940944803 | 0.859532903766548 | 1.4206904825778 | 9.6387189654522 | Canada |
| 2016 | 10.03328785867 | 0.862174225749794 | 1.42440418264914 | 8.60888367602086 | Canada |
| 2017 | 9.01407012882 | 0.865053829759503 | 1.39986363385604 | 7.61420649496396 | Canada |
| 2018 | 8.50974264101 | 0.867905541356997 | 1.40457157034828 | 7.10517107066172 | Canada |
| 2019 | 7.67001488848 | 0.870554479228955 | 1.39527576928707 | 6.27473911919293 | Canada |
| 2020 | 7.33260696182 | 0.871992631299453 | 1.41543052921825 | 5.91717643260175 | Canada |
| 2021 | 6.40216456142 | 0.873170679735405 | 1.408930952682 | 4.993233608738 | Canada |
| 1990 | 12.57644312027 | 0.763647690398232 | 1.83061911866712 | 10.7458240016029 | United States of America |
| 1991 | 12.61989710756 | 0.766044294817203 | 1.78010951588822 | 10.8397875916718 | United States of America |
| 1992 | 11.65465822294 | 0.769595491571717 | 1.73076795151283 | 9.92389027142717 | United States of America |
| 1993 | 10.78577062434 | 0.773103516745741 | 1.7398547424582 | 9.0459158818818 | United States of America |
| 1994 | 11.38012579056 | 0.776368587517635 | 1.74121285602162 | 9.63891293453838 | United States of America |
| 1995 | 11.01649271158 | 0.779410253958491 | 1.66732791107695 | 9.34916480050305 | United States of America |
| 1996 | 11.45822175087 | 0.781934769815664 | 1.67955067066939 | 9.77867108020061 | United States of America |
| 1997 | 11.68309229252 | 0.783979277617936 | 1.64352966225283 | 10.0395626302672 | United States of America |
| 1998 | 11.44956630461 | 0.786076331639174 | 1.64290472489865 | 9.80666157971135 | United States of America |
| 1999 | 10.35291719353 | 0.788544535320099 | 1.61648388154224 | 8.73643331198776 | United States of America |
| 2000 | 10.39327890511 | 0.792316650292886 | 1.58485965903864 | 8.80841924607136 | United States of America |
| 2001 | 10.50464242938 | 0.796676792932995 | 1.55225486192468 | 8.95238756745532 | United States of America |
| 2002 | 11.19013787793 | 0.800403318331674 | 1.52015417327686 | 9.66998370465314 | United States of America |
| 2003 | 12.40910403402 | 0.803228024762672 | 1.54625088470439 | 10.8628531493156 | United States of America |
| 2004 | 12.38546265915 | 0.805304692151156 | 1.55645305606746 | 10.8290096030825 | United States of America |
| 2005 | 10.34906149017 | 0.80603307970225 | 1.54453937539585 | 8.80452211477415 | United States of America |
| 2006 | 8.89404370137 | 0.80678358318377 | 1.53834499149673 | 7.35569870987327 | United States of America |
| 2007 | 8.11815038836 | 0.809688049965476 | 1.52223897405382 | 6.59591141430618 | United States of America |
| 2008 | 7.10594432887 | 0.814577102408992 | 1.51705050747886 | 5.58889382139114 | United States of America |
| 2009 | 6.19656610442 | 0.820294574449102 | 1.48809770526444 | 4.70846839915556 | United States of America |
| 2010 | 5.62670705885 | 0.825884658277638 | 1.47433501633457 | 4.15237204251543 | United States of America |
| 2011 | 5.68834543033 | 0.830266387766787 | 1.46319986284116 | 4.22514556748884 | United States of America |
| 2012 | 5.61323622201 | 0.833958192158326 | 1.48456065968061 | 4.12867556232939 | United States of America |
| 2013 | 5.60939974361 | 0.837412988286127 | 1.47514621820417 | 4.13425352540583 | United States of America |
| 2014 | 5.27496448934 | 0.84074652832772 | 1.47725540601467 | 3.79770908332533 | United States of America |
| 2015 | 5.35832301033 | 0.844370336219248 | 1.459837070646 | 3.898485939684 | United States of America |
| 2016 | 5.18368236115 | 0.848015521315013 | 1.46488980234105 | 3.71879255880895 | United States of America |
| 2017 | 4.69877654404 | 0.851630299087012 | 1.47150859104412 | 3.22726795299588 | United States of America |
| 2018 | 4.27003495164 | 0.855049193484192 | 1.43771675705851 | 2.83231819458149 | United States of America |
| 2019 | 4.05118437003 | 0.858578064552935 | 1.44680313508025 | 2.60438123494975 | United States of America |
| 2020 | 3.8067670496 | 0.860792773409847 | 1.41299083991152 | 2.39377620968848 | United States of America |
| 2021 | 3.68658265979 | 0.862448354015145 | 1.42440393726122 | 2.26217872252878 | United States of America |
| 1990 | 175.55794028246 | 0.612104590576197 | 8.83426360667637 | 166.723676675784 | Antigua and Barbuda |
| 1991 | 157.78040147515 | 0.618817128671612 | 8.697665388675 | 149.082736086475 | Antigua and Barbuda |
| 1992 | 155.79729411114 | 0.624695818117472 | 8.85359894990973 | 146.94369516123 | Antigua and Barbuda |
| 1993 | 166.42954093666 | 0.630075853661038 | 8.12863137343649 | 158.300909563224 | Antigua and Barbuda |
| 1994 | 161.62026257072 | 0.634768955380056 | 7.11390404897859 | 154.506358521741 | Antigua and Barbuda |
| 1995 | 161.89085681736 | 0.638008628575253 | 6.59050426071563 | 155.300352556644 | Antigua and Barbuda |
| 1996 | 158.93990553175 | 0.641647192231223 | 6.31301519871857 | 152.626890333031 | Antigua and Barbuda |
| 1997 | 147.73321706179 | 0.645895590676441 | 6.09720754912859 | 141.636009512661 | Antigua and Barbuda |
| 1998 | 134.23836726718 | 0.650614287301836 | 4.82583967503556 | 129.412527592144 | Antigua and Barbuda |
| 1999 | 118.02141173545 | 0.655534994042462 | 4.89750820021578 | 113.123903535234 | Antigua and Barbuda |
| 2000 | 102.14935136131 | 0.660819064213085 | 4.44748334411727 | 97.7018680171927 | Antigua and Barbuda |
| 2001 | 85.43472161066 | 0.665620631875941 | 4.09484428336727 | 81.3398773272927 | Antigua and Barbuda |
| 2002 | 78.19201410987 | 0.670360801218314 | 3.88507392949465 | 74.3069401803753 | Antigua and Barbuda |
| 2003 | 77.37518895513 | 0.67531148966572 | 3.6125625242477 | 73.7626264308823 | Antigua and Barbuda |
| 2004 | 77.56883714317 | 0.680412429142464 | 3.56611847324494 | 74.0027186699251 | Antigua and Barbuda |
| 2005 | 82.40514877859 | 0.685486437018442 | 3.40482908019338 | 79.0003196983966 | Antigua and Barbuda |
| 2006 | 82.33989041868 | 0.690826021180964 | 3.21358812319586 | 79.1263022954841 | Antigua and Barbuda |
| 2007 | 82.02222790822 | 0.69636003908324 | 3.18641356330733 | 78.8358143449127 | Antigua and Barbuda |
| 2008 | 77.91826511177 | 0.702175362144575 | 3.06990513593389 | 74.8483599758361 | Antigua and Barbuda |
| 2009 | 73.13057357769 | 0.707128563802673 | 2.81352796781053 | 70.3170456098795 | Antigua and Barbuda |
| 2010 | 70.42428016314 | 0.711432245950457 | 2.77249135301804 | 67.651788810122 | Antigua and Barbuda |
| 2011 | 67.19153754615 | 0.715489989160124 | 2.66367509275462 | 64.5278624533954 | Antigua and Barbuda |
| 2012 | 64.81328390704 | 0.719509323328322 | 2.55040983613304 | 62.262874070907 | Antigua and Barbuda |
| 2013 | 65.73431652347 | 0.722864977889834 | 2.35103912659761 | 63.3832773968724 | Antigua and Barbuda |
| 2014 | 69.24054423508 | 0.725585978309816 | 2.43330332200682 | 66.8072409130732 | Antigua and Barbuda |
| 2015 | 66.60630439463 | 0.728372762315346 | 2.30775545487632 | 64.2985489397537 | Antigua and Barbuda |
| 2016 | 64.80933649783 | 0.731598943677227 | 2.3217043307131 | 62.4876321671169 | Antigua and Barbuda |
| 2017 | 64.07636886558 | 0.734963713327539 | 1.91784400702483 | 62.1585248585552 | Antigua and Barbuda |
| 2018 | 62.42685550785 | 0.738879105607608 | 1.96528723669333 | 60.4615682711567 | Antigua and Barbuda |
| 2019 | 60.98327529632 | 0.743052960248658 | 1.92328118589246 | 59.0599941104275 | Antigua and Barbuda |
| 2020 | 56.94746594341 | 0.746345330021488 | 1.8332621919174 | 55.1142037514926 | Antigua and Barbuda |
| 2021 | 53.57685038143 | 0.749886887369379 | 1.79331906373955 | 51.7835313176905 | Antigua and Barbuda |
| 1990 | 229.85711820309 | 0.693509268349695 | 3.20122603782244 | 226.655892165268 | Bahamas |
| 1991 | 244.12449301865 | 0.689126783528471 | 3.31738202988605 | 240.807110988764 | Bahamas |
| 1992 | 243.46338403072 | 0.68734383814569 | 3.3567335872868 | 240.106650443433 | Bahamas |
| 1993 | 224.26376822504 | 0.693816501452916 | 3.2219601924207 | 221.041808032619 | Bahamas |
| 1994 | 183.90514359485 | 0.706819891740348 | 2.72681322554189 | 181.178330369308 | Bahamas |
| 1995 | 156.79498729048 | 0.71938160672878 | 2.41972580070437 | 154.375261489776 | Bahamas |
| 1996 | 135.22706367969 | 0.728478844477514 | 2.23171843926373 | 132.995345240426 | Bahamas |
| 1997 | 124.36247874547 | 0.734781214819761 | 2.0484397107975 | 122.314039034673 | Bahamas |
| 1998 | 114.06146620383 | 0.739281293356304 | 1.86210288080537 | 112.199363323025 | Bahamas |
| 1999 | 104.26580455698 | 0.741622847933781 | 1.89609941588512 | 102.369705141095 | Bahamas |
| 2000 | 92.4481680939 | 0.742517946132475 | 1.80646047195544 | 90.6417076219445 | Bahamas |
| 2001 | 76.71314407006 | 0.743592881699638 | 1.86937832755589 | 74.8437657425041 | Bahamas |
| 2002 | 71.0586296702 | 0.74625032205653 | 1.89419883873984 | 69.1644308314602 | Bahamas |
| 2003 | 67.25667027948 | 0.74991671397758 | 1.88625190518117 | 65.3704183742988 | Bahamas |
| 2004 | 65.52874980318 | 0.753759643287481 | 1.7432865932256 | 63.7854632099544 | Bahamas |
| 2005 | 66.89412302682 | 0.756782661013009 | 1.822491652775 | 65.071631374045 | Bahamas |
| 2006 | 65.52921486284 | 0.759185938528465 | 1.77899236664492 | 63.7502224961951 | Bahamas |
| 2007 | 64.57220989492 | 0.761990996911577 | 1.82015866116586 | 62.7520512337541 | Bahamas |
| 2008 | 60.87307965478 | 0.765590503207612 | 1.80609585094762 | 59.0669838038324 | Bahamas |
| 2009 | 57.33676515192 | 0.769561673980251 | 1.750498282485 | 55.586266869435 | Bahamas |
| 2010 | 51.99412458861 | 0.774119809925167 | 1.67336653086547 | 50.3207580577445 | Bahamas |
| 2011 | 50.83592681362 | 0.778281890749903 | 1.6643513683575 | 49.1715754452625 | Bahamas |
| 2012 | 47.87354541036 | 0.781838639259066 | 1.66507676802857 | 46.2084686423314 | Bahamas |
| 2013 | 49.09892920467 | 0.784623910745264 | 1.63540106869492 | 47.4635281359751 | Bahamas |
| 2014 | 53.43235116316 | 0.787253492401512 | 1.63852923030794 | 51.7938219328521 | Bahamas |
| 2015 | 48.50480817219 | 0.789956145087734 | 1.58392348913385 | 46.9208846830562 | Bahamas |
| 2016 | 47.26331166851 | 0.792738695555901 | 1.56230164711853 | 45.7010100213915 | Bahamas |
| 2017 | 45.81946047451 | 0.795347180655707 | 1.57604775038711 | 44.2434127241229 | Bahamas |
| 2018 | 44.57435703043 | 0.798179034346548 | 1.55201819307181 | 43.0223388373582 | Bahamas |
| 2019 | 43.52562374875 | 0.801033070483021 | 1.53876933681944 | 41.9868544119306 | Bahamas |
| 2020 | 41.10863326 | 0.802948018163753 | 1.55662423980836 | 39.5520090201916 | Bahamas |
| 2021 | 40.33102727849 | 0.805020667882124 | 1.53347979410697 | 38.797547484383 | Bahamas |
| 1990 | 149.27849636234 | 0.653582516816854 | 5.21590633235614 | 144.062590029984 | Barbados |
| 1991 | 140.37195125756 | 0.656654073238155 | 4.93067072331476 | 135.441280534245 | Barbados |
| 1992 | 124.364933113 | 0.662446635199289 | 4.30499354789143 | 120.059939565109 | Barbados |
| 1993 | 114.30537156773 | 0.668815011829235 | 3.8446551109507 | 110.460716456779 | Barbados |
| 1994 | 115.1265967171 | 0.673409365188399 | 3.83264859204392 | 111.293948125056 | Barbados |
| 1995 | 117.06752254708 | 0.676012246725867 | 3.60543559031571 | 113.462086956764 | Barbados |
| 1996 | 116.95577147101 | 0.677193053529371 | 3.60841623891968 | 113.34735523209 | Barbados |
| 1997 | 105.37452929616 | 0.678644826009168 | 3.61602793395257 | 101.758501362207 | Barbados |
| 1998 | 81.87278549326 | 0.679827167153174 | 3.60980788255413 | 78.2629776107059 | Barbados |
| 1999 | 73.71666603796 | 0.680398459240343 | 3.60534879103583 | 70.1113172469242 | Barbados |
| 2000 | 69.90619219921 | 0.681304198777811 | 3.55398132522171 | 66.3522108739883 | Barbados |
| 2001 | 52.90376748655 | 0.683329389407697 | 3.38078136988923 | 49.5229861166608 | Barbados |
| 2002 | 54.01732865139 | 0.68780757593531 | 3.39452898510691 | 50.6227996662831 | Barbados |
| 2003 | 61.83876724469 | 0.693235961982327 | 3.14982095781661 | 58.6889462868734 | Barbados |
| 2004 | 49.55664377374 | 0.698029702584033 | 3.21630820598301 | 46.340335567757 | Barbados |
| 2005 | 53.52772643838 | 0.701510049493398 | 2.85689080794532 | 50.6708356304347 | Barbados |
| 2006 | 53.47898146148 | 0.703856185878429 | 2.87105849523444 | 50.6079229662456 | Barbados |
| 2007 | 50.43992039198 | 0.705871649427123 | 2.9205705851808 | 47.5193498067992 | Barbados |
| 2008 | 48.11010864647 | 0.70851449513133 | 2.85516230870763 | 45.2549463377624 | Barbados |
| 2009 | 44.5561469405 | 0.712143361929882 | 2.73070238156559 | 41.8254445589344 | Barbados |
| 2010 | 43.04836490697 | 0.716208546340619 | 2.51375058531721 | 40.5346143216528 | Barbados |
| 2011 | 47.75365556966 | 0.720060687326812 | 2.56695461780588 | 45.1867009518541 | Barbados |
| 2012 | 38.04411190191 | 0.724162457760429 | 2.39625420175362 | 35.6478577001564 | Barbados |
| 2013 | 39.0174099761 | 0.727826170485545 | 2.45406466812017 | 36.5633453079798 | Barbados |
| 2014 | 39.02065458161 | 0.730383286769139 | 2.16904496674587 | 36.8516096148641 | Barbados |
| 2015 | 38.69639922652 | 0.73260299258046 | 2.25400365119984 | 36.4423955753202 | Barbados |
| 2016 | 37.87400397332 | 0.73491253498334 | 2.04096314765557 | 35.8330408256644 | Barbados |
| 2017 | 37.4878781304 | 0.737157255293201 | 1.89405703931528 | 35.5938210910847 | Barbados |
| 2018 | 36.05852286512 | 0.739531436514553 | 1.86394735286733 | 34.1945755122527 | Barbados |
| 2019 | 35.42582736329 | 0.742239308607991 | 1.89183913653254 | 33.5339882267575 | Barbados |
| 2020 | 34.48235728651 | 0.74436664552373 | 1.81497549192587 | 32.6673817945841 | Barbados |
| 2021 | 34.20518378767 | 0.746748764251474 | 1.77199223363759 | 32.4331915540324 | Barbados |
| 1990 | 1394.75823179435 | 0.423726992172569 | 178.89049532879 | 1215.86773646556 | Belize |
| 1991 | 1165.41793591596 | 0.433834392305066 | 130.097329579745 | 1035.32060633621 | Belize |
| 1992 | 1093.88582991016 | 0.444198507291524 | 111.344608732489 | 982.541221177671 | Belize |
| 1993 | 1023.61906994508 | 0.455258576782351 | 98.4277232676938 | 925.191346677386 | Belize |
| 1994 | 879.54690635069 | 0.465729308156419 | 85.3313241913465 | 794.215582159344 | Belize |
| 1995 | 822.56034557432 | 0.475278511253692 | 77.8517644823088 | 744.708581092011 | Belize |
| 1996 | 780.84769328826 | 0.482702164726655 | 76.1721537100138 | 704.675539578246 | Belize |
| 1997 | 746.59307367327 | 0.488020129434782 | 75.5422761252824 | 671.050797547988 | Belize |
| 1998 | 693.45592988579 | 0.491819335269081 | 76.2787221492778 | 617.177207736512 | Belize |
| 1999 | 590.68472485828 | 0.495070195578772 | 70.3502926765487 | 520.334432181731 | Belize |
| 2000 | 492.4173005052 | 0.498643730395849 | 17.8562373422802 | 474.56106316292 | Belize |
| 2001 | 396.28392150604 | 0.502399433843715 | 16.5670234425253 | 379.716898063515 | Belize |
| 2002 | 339.1445632229 | 0.506871655304811 | 15.9350722427085 | 323.209490980192 | Belize |
| 2003 | 342.84064330438 | 0.512679459131973 | 15.7026764096208 | 327.137966894759 | Belize |
| 2004 | 327.1500028581 | 0.519697333321487 | 15.3330385633305 | 311.816964294769 | Belize |
| 2005 | 343.61077835802 | 0.527231061781742 | 15.0990181575162 | 328.511760200504 | Belize |
| 2006 | 313.30184996281 | 0.534889621081104 | 15.0742058256732 | 298.227644137137 | Belize |
| 2007 | 286.80642139052 | 0.541900694400229 | 15.0334067240073 | 271.773014666513 | Belize |
| 2008 | 269.19295647915 | 0.548093223354439 | 14.571549182109 | 254.621407297041 | Belize |
| 2009 | 254.63179442967 | 0.553465869255233 | 13.7729639709247 | 240.858830458745 | Belize |
| 2010 | 217.26766996564 | 0.558546893327125 | 12.002651935073 | 205.265018030567 | Belize |
| 2011 | 207.3743321366 | 0.563440362640477 | 11.0269409345495 | 196.34739120205 | Belize |
| 2012 | 186.14398875551 | 0.568456235800016 | 9.58430623207286 | 176.559682523437 | Belize |
| 2013 | 181.64739672061 | 0.573236457430755 | 8.89727753701609 | 172.750119183594 | Belize |
| 2014 | 190.16120818563 | 0.578091325788653 | 8.7403396067533 | 181.420868578877 | Belize |
| 2015 | 187.5271033996 | 0.583279094493031 | 8.86714891911575 | 178.659954480484 | Belize |
| 2016 | 181.91144070985 | 0.588275912195168 | 8.69653063764585 | 173.214910072204 | Belize |
| 2017 | 173.50669591406 | 0.593337290122785 | 8.7513911384475 | 164.755304775613 | Belize |
| 2018 | 168.59544136386 | 0.598512014950432 | 9.10304705149671 | 159.492394312363 | Belize |
| 2019 | 159.65932149595 | 0.603351601174544 | 8.89375348192551 | 150.765568014024 | Belize |
| 2020 | 152.84956122283 | 0.607060046133776 | 8.79933108422451 | 144.050230138605 | Belize |
| 2021 | 145.65402564599 | 0.610229002222948 | 8.63698397172649 | 137.017041674264 | Belize |
| 1990 | 243.18322708395 | 0.558019070765374 | 12.0683408215263 | 231.114886262424 | Cuba |
| 1991 | 212.36345318479 | 0.563413638292236 | 10.8721204834148 | 201.491332701375 | Cuba |
| 1992 | 178.16275601499 | 0.566169005573441 | 10.1704240656938 | 167.992331949296 | Cuba |
| 1993 | 179.80825015616 | 0.565441046515952 | 10.4916079423034 | 169.316642213857 | Cuba |
| 1994 | 186.39784746567 | 0.563186312783606 | 11.5311537465501 | 174.86669371912 | Cuba |
| 1995 | 178.20991765354 | 0.560765902424453 | 11.3703727221272 | 166.839544931413 | Cuba |
| 1996 | 142.89988181954 | 0.559346328872574 | 12.1342012145158 | 130.765680605024 | Cuba |
| 1997 | 129.14352181289 | 0.558924568054562 | 12.0407893911497 | 117.10273242174 | Cuba |
| 1998 | 116.83371444463 | 0.559363220275698 | 12.0706224371949 | 104.763092007435 | Cuba |
| 1999 | 99.13412791421 | 0.561820952733584 | 11.1920098590207 | 87.9421180551893 | Cuba |
| 2000 | 85.08487174386 | 0.566676901631981 | 10.3481649091894 | 74.7367068346706 | Cuba |
| 2001 | 66.84238374642 | 0.572753860839461 | 8.68318276550528 | 58.1592009809147 | Cuba |
| 2002 | 60.05148747296 | 0.579968290233664 | 8.74546238823081 | 51.3060250847292 | Cuba |
| 2003 | 61.83903696166 | 0.587647761532625 | 8.93664826965618 | 52.9023886920038 | Cuba |
| 2004 | 58.52662401794 | 0.594348715355523 | 9.05589160278677 | 49.4707324151532 | Cuba |
| 2005 | 66.15822145464 | 0.599863798514665 | 8.94865706554098 | 57.209564389099 | Cuba |
| 2006 | 60.58736662944 | 0.604970567275803 | 8.73710588292326 | 51.8502607465167 | Cuba |
| 2007 | 58.21180319826 | 0.607618098577978 | 8.88811002310859 | 49.3236931751514 | Cuba |
| 2008 | 60.79056442924 | 0.60940092330195 | 8.85025561621057 | 51.9403088130294 | Cuba |
| 2009 | 58.91288077561 | 0.611775641002492 | 8.78926559104225 | 50.1236151845677 | Cuba |
| 2010 | 52.26968259387 | 0.615261262451076 | 8.77276889687113 | 43.4969136969989 | Cuba |
| 2011 | 51.59044574676 | 0.620386689831088 | 8.99448278202824 | 42.5959629647318 | Cuba |
| 2012 | 51.22848367942 | 0.626156024890616 | 8.68938861120925 | 42.5390950682107 | Cuba |
| 2013 | 47.81607483384 | 0.631839801039436 | 8.05566632178836 | 39.7604085120516 | Cuba |
| 2014 | 50.47109667167 | 0.637304129079553 | 6.71456628927 | 43.7565303824 | Cuba |
| 2015 | 45.18243223931 | 0.642818963057111 | 6.11504108052911 | 39.0673911587809 | Cuba |
| 2016 | 42.18736394267 | 0.647907866261711 | 6.09671903356293 | 36.0906449091071 | Cuba |
| 2017 | 44.041022433 | 0.652309286413179 | 5.06023420329147 | 38.9807882297085 | Cuba |
| 2018 | 44.45470451658 | 0.65685865401747 | 4.83541966486683 | 39.6192848517132 | Cuba |
| 2019 | 49.3860167577 | 0.661674716522849 | 4.26648828159942 | 45.1195284761006 | Cuba |
| 2020 | 41.6609980847 | 0.665210747223401 | 4.238919158203 | 37.422078926497 | Cuba |
| 2021 | 36.39370046191 | 0.668729864363244 | 3.87241841765257 | 32.5212820442574 | Cuba |
| 1990 | 258.88342311502 | 0.563602590366912 | 10.9722262052543 | 247.911196909766 | Dominica |
| 1991 | 254.89059785283 | 0.565237843041271 | 10.6557119553809 | 244.234885897449 | Dominica |
| 1992 | 247.16111484153 | 0.569570683969256 | 9.51449234987562 | 237.646622491654 | Dominica |
| 1993 | 236.45583050368 | 0.578811810513113 | 8.80890174650868 | 227.646928757171 | Dominica |
| 1994 | 227.17952540621 | 0.590119147153383 | 8.93543399983849 | 218.244091406371 | Dominica |
| 1995 | 213.11063010032 | 0.601986931512718 | 8.82676797108064 | 204.283862129239 | Dominica |
| 1996 | 201.06803083517 | 0.614382049775589 | 8.72678143333296 | 192.341249401837 | Dominica |
| 1997 | 190.30078258348 | 0.625057352694054 | 8.83630685556163 | 181.464475727918 | Dominica |
| 1998 | 180.87163088478 | 0.63301799374648 | 7.07213707196131 | 173.799493812819 | Dominica |
| 1999 | 177.74190015258 | 0.63945391525062 | 6.46610438210392 | 171.275795770476 | Dominica |
| 2000 | 166.36194815519 | 0.645830936825434 | 6.34187076240825 | 160.020077392782 | Dominica |
| 2001 | 163.11876574879 | 0.652368567094588 | 5.10910489871488 | 158.009660850075 | Dominica |
| 2002 | 154.65394598998 | 0.658423554407679 | 4.81584140482525 | 149.838104585155 | Dominica |
| 2003 | 153.08651708098 | 0.663678867784012 | 4.22000674169254 | 148.866510339287 | Dominica |
| 2004 | 147.77272023243 | 0.668952131656395 | 3.87035377912925 | 143.902366453301 | Dominica |
| 2005 | 146.04686672424 | 0.67454907679023 | 3.60049775637365 | 142.446368967866 | Dominica |
| 2006 | 141.79789210725 | 0.680342481749478 | 3.58635786355506 | 138.211534243695 | Dominica |
| 2007 | 145.45624368971 | 0.685415917499458 | 3.39428067328888 | 142.061963016421 | Dominica |
| 2008 | 143.05197202927 | 0.69019751901742 | 3.40190759698 | 139.65006443229 | Dominica |
| 2009 | 154.69787041503 | 0.694461869014732 | 2.99985589753614 | 151.698014517494 | Dominica |
| 2010 | 162.22189458702 | 0.698489193418408 | 3.00848532747043 | 159.21340925955 | Dominica |
| 2011 | 174.97048225175 | 0.702463260148908 | 2.95483908378771 | 172.015643167962 | Dominica |
| 2012 | 180.60966425419 | 0.706870607879807 | 2.69736076233973 | 177.91230349185 | Dominica |
| 2013 | 188.8034390326 | 0.71221047502743 | 2.77361888983384 | 186.029820142766 | Dominica |
| 2014 | 189.12911129319 | 0.718455556016605 | 2.50637142099062 | 186.622739872199 | Dominica |
| 2015 | 186.49527848788 | 0.724785536415403 | 2.36736865561922 | 184.127909832261 | Dominica |
| 2016 | 181.70629283679 | 0.73176080052363 | 2.29075426289473 | 179.415538573895 | Dominica |
| 2017 | 181.44805777816 | 0.736062472571312 | 2.03101080516512 | 179.417046972995 | Dominica |
| 2018 | 183.40077895808 | 0.738935953651369 | 1.88408974368111 | 181.516689214399 | Dominica |
| 2019 | 186.52037734002 | 0.74213206575999 | 1.89852885317381 | 184.621848486846 | Dominica |
| 2020 | 188.92546284807 | 0.744419943891979 | 1.77044130844703 | 187.155021539623 | Dominica |
| 2021 | 190.35474140728 | 0.746967184749286 | 1.82279780477806 | 188.531943602502 | Dominica |
| 1990 | 3308.44239729953 | 0.442654076123481 | 111.786520974889 | 3196.65587632464 | Dominican Republic |
| 1991 | 3093.79375611008 | 0.446144577831215 | 109.6641569808 | 2984.12959912928 | Dominican Republic |
| 1992 | 2850.69115649441 | 0.449953080528465 | 108.157533868597 | 2742.53362262581 | Dominican Republic |
| 1993 | 2630.14698577115 | 0.454229297152068 | 107.183832765265 | 2522.96315300589 | Dominican Republic |
| 1994 | 2373.1957634882 | 0.458620613654854 | 93.74379162508 | 2279.45197186312 | Dominican Republic |
| 1995 | 2145.34283935665 | 0.463313869288046 | 92.4416954980665 | 2052.90114385858 | Dominican Republic |
| 1996 | 1906.92873312342 | 0.468516938419476 | 85.0093730699532 | 1821.91936005347 | Dominican Republic |
| 1997 | 1695.24805613808 | 0.474504388639617 | 80.3327988206822 | 1614.9152573174 | Dominican Republic |
| 1998 | 1488.01449513311 | 0.481170939346086 | 77.5499052427691 | 1410.46458989034 | Dominican Republic |
| 1999 | 1315.93143228843 | 0.488178541467036 | 75.089149490085 | 1240.84228279834 | Dominican Republic |
| 2000 | 1196.25055297925 | 0.495468586151319 | 71.4450437866088 | 1124.80550919264 | Dominican Republic |
| 2001 | 1126.10226882676 | 0.50277541519763 | 16.1902236033942 | 1109.91204522337 | Dominican Republic |
| 2002 | 1037.91790524408 | 0.510298018698881 | 15.7496585042569 | 1022.16824673982 | Dominican Republic |
| 2003 | 974.02665497552 | 0.517395574464313 | 15.3450358216817 | 958.681619153838 | Dominican Republic |
| 2004 | 951.52241624245 | 0.524543274827677 | 15.0737854948106 | 936.448630747639 | Dominican Republic |
| 2005 | 928.8195018335 | 0.532228649477556 | 15.0721672070263 | 913.747334626474 | Dominican Republic |
| 2006 | 854.46131544564 | 0.540222689966408 | 15.0600192782284 | 839.401296167412 | Dominican Republic |
| 2007 | 783.13516546247 | 0.548187021157434 | 14.5945485558901 | 768.54061690658 | Dominican Republic |
| 2008 | 712.81767006998 | 0.555371313172153 | 13.0118035187464 | 699.805866551234 | Dominican Republic |
| 2009 | 665.75081248969 | 0.561783754117237 | 11.3480081197257 | 654.402804369964 | Dominican Republic |
| 2010 | 651.27949291293 | 0.568130581856948 | 9.88460773237877 | 641.394885180551 | Dominican Republic |
| 2011 | 758.42874531672 | 0.573834812622029 | 9.03226959734147 | 749.396475719378 | Dominican Republic |
| 2012 | 776.94985711457 | 0.578966380988324 | 8.98420103631514 | 767.965656078255 | Dominican Republic |
| 2013 | 616.44070720668 | 0.583625825103645 | 8.95923960879965 | 607.48146759788 | Dominican Republic |
| 2014 | 561.00867969841 | 0.588236742676906 | 8.79418214999189 | 552.214497548418 | Dominican Republic |
| 2015 | 503.24758717843 | 0.592924164325808 | 8.76371877843163 | 494.483868399998 | Dominican Republic |
| 2016 | 484.41281120554 | 0.597619609207884 | 8.74520630501407 | 475.667604900526 | Dominican Republic |
| 2017 | 448.90486836706 | 0.602140291257967 | 9.02221289780881 | 439.882655469251 | Dominican Republic |
| 2018 | 429.04798615336 | 0.606960838531609 | 8.97478496266606 | 420.073201190694 | Dominican Republic |
| 2019 | 409.43234801798 | 0.611913021025697 | 8.89822858569341 | 400.534119432287 | Dominican Republic |
| 2020 | 387.84417221301 | 0.615635625027175 | 8.99301373995472 | 378.851158473055 | Dominican Republic |
| 2021 | 384.95955951385 | 0.619388201281128 | 8.77079982345988 | 376.18875969039 | Dominican Republic |
| 1990 | 238.8766926676 | 0.436734419397497 | 128.888939473036 | 109.987753194564 | Grenada |
| 1991 | 199.83256773733 | 0.445887952925777 | 110.171883957895 | 89.6606837794348 | Grenada |
| 1992 | 196.42774053894 | 0.455910058376885 | 98.7706059654494 | 97.6571345734905 | Grenada |
| 1993 | 198.06052543184 | 0.465994889867929 | 85.5793080757055 | 112.481217356134 | Grenada |
| 1994 | 180.90077529011 | 0.476410340600106 | 77.7294159689721 | 103.171359321138 | Grenada |
| 1995 | 169.85793693112 | 0.486558998595925 | 74.9485121960314 | 94.9094247350886 | Grenada |
| 1996 | 161.65179788681 | 0.496589807805192 | 33.6052316818696 | 128.04656620494 | Grenada |
| 1997 | 149.85352110329 | 0.50621760158124 | 16.0021018896589 | 133.851419213631 | Grenada |
| 1998 | 127.65250486379 | 0.516590019085488 | 15.3122751332879 | 112.340229730502 | Grenada |
| 1999 | 91.43701347159 | 0.527333411440571 | 15.0587240168489 | 76.3782894547412 | Grenada |
| 2000 | 77.58482554319 | 0.538388116408646 | 15.0367603192979 | 62.5480652238921 | Grenada |
| 2001 | 72.93859223014 | 0.549058969647808 | 14.4797259944537 | 58.4588662356863 | Grenada |
| 2002 | 66.37125083982 | 0.559473127591996 | 11.8208073974205 | 54.5504434423995 | Grenada |
| 2003 | 65.73999565405 | 0.569718037363691 | 9.44068181582509 | 56.2993138382249 | Grenada |
| 2004 | 64.80307214613 | 0.578487456332464 | 8.83082616230114 | 55.9722459838289 | Grenada |
| 2005 | 68.37583527272 | 0.587158876770892 | 8.83312296640522 | 59.5427123063148 | Grenada |
| 2006 | 68.65454020312 | 0.59405451978646 | 8.73808920503631 | 59.9164509980837 | Grenada |
| 2007 | 65.81835232077 | 0.600766335502731 | 8.67738577497278 | 57.1409665457972 | Grenada |
| 2008 | 64.00202700582 | 0.606879281941233 | 8.82506959132924 | 55.1769574144908 | Grenada |
| 2009 | 60.58093153607 | 0.61180525701265 | 8.91339218980583 | 51.6675393462642 | Grenada |
| 2010 | 56.65069528913 | 0.616486997376644 | 8.56714034422154 | 48.0835549449085 | Grenada |
| 2011 | 57.20052704904 | 0.620978521316098 | 8.6487066125593 | 48.5518204364807 | Grenada |
| 2012 | 55.54943240073 | 0.624977512249081 | 8.66217584487141 | 46.8872565558586 | Grenada |
| 2013 | 55.57480041449 | 0.629048335732456 | 8.05160469369919 | 47.5231957207908 | Grenada |
| 2014 | 60.33887311169 | 0.633734695952301 | 6.91257382480139 | 53.4262992868886 | Grenada |
| 2015 | 56.02787459793 | 0.638936083726672 | 6.81439874256614 | 49.2134758553639 | Grenada |
| 2016 | 51.94349326908 | 0.644335233932856 | 6.17092908748803 | 45.772564181592 | Grenada |
| 2017 | 48.4916600359 | 0.649831521980215 | 5.36024881085269 | 43.1314112250473 | Grenada |
| 2018 | 47.32326543876 | 0.655372772077438 | 4.82809250651 | 42.49517293225 | Grenada |
| 2019 | 44.92645973329 | 0.660808511595714 | 4.38312497908766 | 40.5433347542023 | Grenada |
| 2020 | 43.23465797392 | 0.665086346623039 | 4.12610888622148 | 39.1085490876985 | Grenada |
| 2021 | 40.83275644293 | 0.668993028326993 | 3.99030807976782 | 36.8424483631622 | Grenada |
| 1990 | 2440.92915155314 | 0.460430128793009 | 94.1393040881237 | 2346.78984746502 | Guyana |
| 1991 | 2192.90204733042 | 0.462677068801868 | 91.5774738760406 | 2101.32457345438 | Guyana |
| 1992 | 2073.64327957243 | 0.466753989262645 | 85.7062028805763 | 1987.93707669185 | Guyana |
| 1993 | 2027.89031029006 | 0.472174228924914 | 82.4564544089979 | 1945.43385588106 | Guyana |
| 1994 | 1882.64974799959 | 0.478992924260878 | 76.9843088783748 | 1805.66543912122 | Guyana |
| 1995 | 1744.58518518324 | 0.486573457084383 | 75.1703526318675 | 1669.41483255137 | Guyana |
| 1996 | 1586.93289644375 | 0.495216036467396 | 71.2589289749491 | 1515.6739674688 | Guyana |
| 1997 | 1525.4231459302 | 0.504362778751228 | 16.1715003602986 | 1509.2516455699 | Guyana |
| 1998 | 1446.56377276593 | 0.513068898204645 | 15.7435341498379 | 1430.82023861609 | Guyana |
| 1999 | 1554.13833799664 | 0.521595978096732 | 15.2469593595245 | 1538.89137863712 | Guyana |
| 2000 | 1856.49866612424 | 0.528693991503479 | 15.1098770392761 | 1841.38878908496 | Guyana |
| 2001 | 1358.0515786886 | 0.534869805890103 | 15.0687627534086 | 1342.98281593519 | Guyana |
| 2002 | 1115.29748475678 | 0.54051427331456 | 15.1025233907701 | 1100.19496136601 | Guyana |
| 2003 | 1012.44082388842 | 0.545516473248843 | 14.5562904979032 | 997.884533390517 | Guyana |
| 2004 | 925.70980957573 | 0.550326370085983 | 13.8435903737647 | 911.866219201965 | Guyana |
| 2005 | 1020.64990285024 | 0.554844797400739 | 13.1156318622058 | 1007.53427098803 | Guyana |
| 2006 | 961.31413142356 | 0.55946561076404 | 12.2798541295161 | 949.034277294044 | Guyana |
| 2007 | 822.35140217474 | 0.564541135103012 | 10.8898520053936 | 811.461550169346 | Guyana |
| 2008 | 751.33664149366 | 0.569595205505363 | 9.49175176582013 | 741.84488972784 | Guyana |
| 2009 | 765.933347304 | 0.574769931497353 | 8.8026821306476 | 757.130665173352 | Guyana |
| 2010 | 671.27144704862 | 0.580083911433493 | 8.77119922394987 | 662.50024782467 | Guyana |
| 2011 | 580.7860615468 | 0.585688269016646 | 8.75558337336879 | 572.030478173431 | Guyana |
| 2012 | 534.8940994756 | 0.591336582127807 | 8.82264756825163 | 526.071451907348 | Guyana |
| 2013 | 503.18153727308 | 0.596826294655764 | 8.73146679583239 | 494.450070477248 | Guyana |
| 2014 | 489.95899156356 | 0.602136510795571 | 8.76839763679341 | 481.190593926767 | Guyana |
| 2015 | 452.11241109977 | 0.60760548677096 | 8.78594636863962 | 443.32646473113 | Guyana |
| 2016 | 426.97110711437 | 0.613914359668043 | 8.83852040890674 | 418.132586705463 | Guyana |
| 2017 | 408.08419788175 | 0.620602607413008 | 8.76495666794905 | 399.319241213801 | Guyana |
| 2018 | 405.15704866696 | 0.627167040808132 | 8.68587278524226 | 396.471175881718 | Guyana |
| 2019 | 353.38309543131 | 0.633640346503077 | 6.93191111641901 | 346.451184314891 | Guyana |
| 2020 | 319.02481459135 | 0.642284644781673 | 5.91684767314506 | 313.107966918205 | Guyana |
| 2021 | 293.92415353882 | 0.650812335285137 | 5.11204737961689 | 288.812106159203 | Guyana |
| 1990 | 13268.0709410342 | 0.310334630409749 | 879.247228277478 | 12388.8237127567 | Haiti |
| 1991 | 12570.9913277159 | 0.315455441278148 | 778.627152875953 | 11792.3641748399 | Haiti |
| 1992 | 12165.6979336976 | 0.320389978747448 | 781.793947421824 | 11383.9039862758 | Haiti |
| 1993 | 11684.0169765543 | 0.324895850993773 | 720.902158912876 | 10963.1148176414 | Haiti |
| 1994 | 11261.842753651 | 0.328399455968527 | 719.107351978775 | 10542.7354016722 | Haiti |
| 1995 | 10820.0093436187 | 0.332999151562276 | 652.016704968583 | 10167.9926386501 | Haiti |
| 1996 | 10198.0210868709 | 0.33822939619677 | 597.49327406208 | 9600.52781280882 | Haiti |
| 1997 | 9657.96540501352 | 0.343880049177945 | 573.066673642686 | 9084.89873137083 | Haiti |
| 1998 | 8945.94999235773 | 0.349698073003298 | 560.023897066352 | 8385.92609529138 | Haiti |
| 1999 | 8461.56218013515 | 0.355980742770846 | 555.160100927642 | 7906.40207920751 | Haiti |
| 2000 | 7851.97514610076 | 0.36257220895481 | 466.961887888916 | 7385.01325821184 | Haiti |
| 2001 | 7495.3976989603 | 0.368643507933658 | 415.207064400562 | 7080.19063455974 | Haiti |
| 2002 | 7172.55280089362 | 0.374473324901094 | 353.916375082895 | 6818.63642581073 | Haiti |
| 2003 | 6905.6428181688 | 0.380067254956705 | 327.142063783271 | 6578.50075438553 | Haiti |
| 2004 | 6674.05158338716 | 0.385061511883196 | 316.077688574728 | 6357.97389481243 | Haiti |
| 2005 | 6427.58778914781 | 0.389845419506401 | 290.64895254867 | 6136.93883659914 | Haiti |
| 2006 | 6103.76141453206 | 0.394322001247404 | 288.509471342243 | 5815.25194318982 | Haiti |
| 2007 | 5845.87108955242 | 0.39897344059179 | 259.612183964486 | 5586.25890558793 | Haiti |
| 2008 | 5577.94930659607 | 0.40328587122688 | 237.968095276444 | 5339.98121131963 | Haiti |
| 2009 | 5318.45879500304 | 0.407944423962943 | 234.99859424257 | 5083.46020076047 | Haiti |
| 2010 | 6500.62177627295 | 0.411840105174603 | 215.64285741674 | 6284.97891885621 | Haiti |
| 2011 | 10405.5430970551 | 0.416177190225703 | 203.338906456806 | 10202.2041905983 | Haiti |
| 2012 | 4946.85239990224 | 0.420021527912646 | 194.993997760344 | 4751.8584021419 | Haiti |
| 2013 | 5317.71834296803 | 0.423985549390571 | 177.50618307668 | 5140.21215989135 | Haiti |
| 2014 | 4672.19501525905 | 0.427940902171937 | 164.39627763563 | 4507.79873762342 | Haiti |
| 2015 | 4376.88104253358 | 0.431756760061604 | 139.33971728418 | 4237.5413252494 | Haiti |
| 2016 | 4490.95269729161 | 0.435288722267081 | 127.623580595833 | 4363.32911669578 | Haiti |
| 2017 | 3930.8220328239 | 0.438541003090222 | 125.703176225308 | 3805.11885659859 | Haiti |
| 2018 | 3747.73560441561 | 0.44167892537118 | 120.847989789716 | 3626.88761462589 | Haiti |
| 2019 | 3623.75547056669 | 0.444328853815805 | 112.174274942056 | 3511.58119562463 | Haiti |
| 2020 | 3493.61455035666 | 0.446390613582292 | 112.030753150436 | 3381.58379720622 | Haiti |
| 2021 | 3414.9791524473 | 0.448278284992742 | 106.350779207703 | 3308.6283732396 | Haiti |
| 1990 | 844.82604382413 | 0.534781233684701 | 15.0610653223889 | 829.764978501741 | Jamaica |
| 1991 | 725.82954786434 | 0.539594479788248 | 15.0692857987485 | 710.760262065592 | Jamaica |
| 1992 | 658.28290506338 | 0.545088752562521 | 15.03703339211 | 643.24587167127 | Jamaica |
| 1993 | 585.31700266906 | 0.551218491600381 | 13.966209280979 | 571.350793388081 | Jamaica |
| 1994 | 515.10512322523 | 0.557643773203113 | 11.8611683715257 | 503.243954853704 | Jamaica |
| 1995 | 408.25661926765 | 0.564315037773069 | 10.8959786977702 | 397.36064056988 | Jamaica |
| 1996 | 253.10359114072 | 0.570940131286319 | 8.80901574412208 | 244.294575396598 | Jamaica |
| 1997 | 194.691151852 | 0.577212091097575 | 8.86872656496659 | 185.822425287033 | Jamaica |
| 1998 | 173.20799829253 | 0.58304803468123 | 9.05633463198783 | 164.151663660542 | Jamaica |
| 1999 | 156.95632310719 | 0.588846467179198 | 8.59480493190341 | 148.361518175287 | Jamaica |
| 2000 | 142.50943045539 | 0.594592916825086 | 8.92611146987775 | 133.583318985512 | Jamaica |
| 2001 | 112.96603861692 | 0.600192022232271 | 8.69056669979037 | 104.27547191713 | Jamaica |
| 2002 | 103.64462159592 | 0.605591748141932 | 8.80559668931936 | 94.8390249066006 | Jamaica |
| 2003 | 108.21960732827 | 0.610877423593022 | 8.98970898520161 | 99.2298983430684 | Jamaica |
| 2004 | 104.33755374416 | 0.615836278207827 | 8.8804617748252 | 95.4570919693348 | Jamaica |
| 2005 | 107.41554134151 | 0.620400056664326 | 8.97735029612443 | 98.4381910453856 | Jamaica |
| 2006 | 126.12343585435 | 0.625018859668551 | 9.16957155053637 | 116.953864303814 | Jamaica |
| 2007 | 121.23424323176 | 0.629549469674028 | 8.27487429581975 | 112.95936893594 | Jamaica |
| 2008 | 114.98356637201 | 0.633967851311631 | 7.14756017713471 | 107.836006194875 | Jamaica |
| 2009 | 112.102654393 | 0.638110835001433 | 6.73569378327905 | 105.366960609721 | Jamaica |
| 2010 | 106.17715739287 | 0.642129387979891 | 6.22875567241147 | 99.9484017204585 | Jamaica |
| 2011 | 105.43192067062 | 0.646395830061733 | 6.30806439369373 | 99.1238562769263 | Jamaica |
| 2012 | 105.75530872793 | 0.650583760450656 | 5.38586141192831 | 100.369447316002 | Jamaica |
| 2013 | 98.1355427587 | 0.654573624159258 | 4.87539056546101 | 93.260152193239 | Jamaica |
| 2014 | 116.83184141105 | 0.658505341253061 | 4.55964440049414 | 112.272197010556 | Jamaica |
| 2015 | 110.3963482704 | 0.662465809295028 | 4.41495658176073 | 105.981391688639 | Jamaica |
| 2016 | 106.15860665611 | 0.666469278740004 | 4.01848043113986 | 102.14012622497 | Jamaica |
| 2017 | 102.1619200087 | 0.670291045200821 | 3.81596854738084 | 98.3459514613191 | Jamaica |
| 2018 | 98.25903696992 | 0.674075312952044 | 3.83330363391964 | 94.4257333360004 | Jamaica |
| 2019 | 94.98884868873 | 0.677778539230129 | 3.59519506422346 | 91.3936536245065 | Jamaica |
| 2020 | 91.29939488475 | 0.680567176976565 | 3.58301214953 | 87.71638273522 | Jamaica |
| 2021 | 85.17167174609 | 0.683263063531091 | 3.3923924322911 | 81.7792793137989 | Jamaica |
| 1990 | 456.77826006114 | 0.496296570103573 | 38.8843022317119 | 417.893957829428 | Saint Lucia |
| 1991 | 396.58006052715 | 0.505975554986447 | 16.2476102666024 | 380.332450260548 | Saint Lucia |
| 1992 | 366.89114851046 | 0.515368260657711 | 15.322857734328 | 351.568290776132 | Saint Lucia |
| 1993 | 376.6464409698 | 0.524234949005948 | 15.0504982783451 | 361.595942691455 | Saint Lucia |
| 1994 | 334.86939911742 | 0.532731082607556 | 15.0543522022764 | 319.815046915144 | Saint Lucia |
| 1995 | 311.25340926918 | 0.541479793625538 | 15.032898530129 | 296.220510739051 | Saint Lucia |
| 1996 | 287.90937132683 | 0.550242411723114 | 13.6711437940981 | 274.238227532732 | Saint Lucia |
| 1997 | 262.31311298979 | 0.558293460901167 | 12.0451772251228 | 250.267935764667 | Saint Lucia |
| 1998 | 219.90462128038 | 0.566138702625704 | 10.1817797014397 | 209.72284157894 | Saint Lucia |
| 1999 | 182.44911599186 | 0.573428555367193 | 8.89483872040906 | 173.554277271451 | Saint Lucia |
| 2000 | 151.37819368414 | 0.579788896229167 | 8.94555410918221 | 142.432639574958 | Saint Lucia |
| 2001 | 125.84001685975 | 0.585087557754993 | 9.050225047725 | 116.789791812025 | Saint Lucia |
| 2002 | 113.06768013179 | 0.590167578497987 | 8.91813638391836 | 104.149543747872 | Saint Lucia |
| 2003 | 111.40985612405 | 0.595675145724796 | 8.83696765238291 | 102.572888471667 | Saint Lucia |
| 2004 | 110.49795270812 | 0.601625481597697 | 8.86917594409036 | 101.62877676403 | Saint Lucia |
| 2005 | 120.20063780546 | 0.60723986604169 | 8.90482791405165 | 111.295809891408 | Saint Lucia |
| 2006 | 115.77471109082 | 0.612755299779844 | 8.88717794080041 | 106.88753315002 | Saint Lucia |
| 2007 | 114.01621312935 | 0.617994714022193 | 8.86633683743905 | 105.149876291911 | Saint Lucia |
| 2008 | 112.09295863821 | 0.62327300064738 | 8.88873841361667 | 103.204220224593 | Saint Lucia |
| 2009 | 103.84122223445 | 0.628169848746241 | 8.22252771462209 | 95.6186945198279 | Saint Lucia |
| 2010 | 94.96163042814 | 0.632782904571138 | 6.87861091008931 | 88.0830195180507 | Saint Lucia |
| 2011 | 96.97207737583 | 0.637439441748952 | 6.56916881635609 | 90.4029085594739 | Saint Lucia |
| 2012 | 93.30994331942 | 0.641567127255833 | 5.97487509342563 | 87.3350682259944 | Saint Lucia |
| 2013 | 93.49112409811 | 0.645153079091964 | 6.00519279993455 | 87.4859312981755 | Saint Lucia |
| 2014 | 101.51830275404 | 0.648743506935936 | 5.91320354867593 | 95.6050992053641 | Saint Lucia |
| 2015 | 97.19724741794 | 0.652157480490287 | 4.94389788989803 | 92.253349528042 | Saint Lucia |
| 2016 | 91.13800536107 | 0.655653340205716 | 4.92223326122397 | 86.215772099846 | Saint Lucia |
| 2017 | 86.9986836031 | 0.659341345742569 | 4.32887861173657 | 82.6698049913634 | Saint Lucia |
| 2018 | 83.17081326605 | 0.663086681831917 | 4.27799657717758 | 78.8928166888724 | Saint Lucia |
| 2019 | 80.00216029724 | 0.666840358993444 | 3.8821925747454 | 76.1199677224946 | Saint Lucia |
| 2020 | 74.2676984048 | 0.669720489592338 | 3.89232295879 | 70.37537544601 | Saint Lucia |
| 2021 | 72.56974385662 | 0.672509735365292 | 3.75948089750145 | 68.8102629591185 | Saint Lucia |
| 1990 | 893.47031047609 | 0.475930186469041 | 78.4158442684452 | 815.054466207645 | Saint Vincent and the Grenadines |
| 1991 | 828.66369690407 | 0.481075658352439 | 77.0451020612989 | 751.618594842771 | Saint Vincent and the Grenadines |
| 1992 | 750.25349140469 | 0.485977871294048 | 75.4243578754264 | 674.829133529264 | Saint Vincent and the Grenadines |
| 1993 | 702.40263860241 | 0.490998790902684 | 75.2479865939536 | 627.154652008456 | Saint Vincent and the Grenadines |
| 1994 | 642.45759694489 | 0.496379132267915 | 41.4575964204576 | 601.000000524432 | Saint Vincent and the Grenadines |
| 1995 | 579.17518011803 | 0.502986089658165 | 16.1152994462005 | 563.059880671829 | Saint Vincent and the Grenadines |
| 1996 | 527.05142899342 | 0.509527157829315 | 15.9985409928389 | 511.052888000581 | Saint Vincent and the Grenadines |
| 1997 | 474.70403837045 | 0.51548261862783 | 15.3492135211037 | 459.354824849346 | Saint Vincent and the Grenadines |
| 1998 | 412.40520721516 | 0.52120967882065 | 15.1510312792953 | 397.254175935865 | Saint Vincent and the Grenadines |
| 1999 | 350.38674901154 | 0.527099551882504 | 15.1219277802587 | 335.264821231281 | Saint Vincent and the Grenadines |
| 2000 | 293.170243646 | 0.533173289927656 | 15.0544549211389 | 278.115788724861 | Saint Vincent and the Grenadines |
| 2001 | 251.33089591773 | 0.539455711639871 | 15.0687044734026 | 236.262191444327 | Saint Vincent and the Grenadines |
| 2002 | 213.18622673663 | 0.546063615669324 | 14.5549804330267 | 198.631246303603 | Saint Vincent and the Grenadines |
| 2003 | 222.22528578102 | 0.552693724058086 | 13.6074078456918 | 208.617877935328 | Saint Vincent and the Grenadines |
| 2004 | 207.25422033019 | 0.558663542945828 | 12.113938865203 | 195.140281464987 | Saint Vincent and the Grenadines |
| 2005 | 214.60212647269 | 0.563588116289597 | 10.8970628771721 | 203.705063595518 | Saint Vincent and the Grenadines |
| 2006 | 209.20928561014 | 0.568221747811185 | 9.52262898819151 | 199.686656621948 | Saint Vincent and the Grenadines |
| 2007 | 205.70925775837 | 0.572819052303515 | 8.83337059174323 | 196.875887166627 | Saint Vincent and the Grenadines |
| 2008 | 190.94882574756 | 0.577611144975623 | 8.81615721368235 | 182.132668533878 | Saint Vincent and the Grenadines |
| 2009 | 175.99584976596 | 0.582549107234974 | 8.86616199209631 | 167.129687773864 | Saint Vincent and the Grenadines |
| 2010 | 160.4692678303 | 0.587190383760787 | 8.98071723440551 | 151.488550595894 | Saint Vincent and the Grenadines |
| 2011 | 157.58733215835 | 0.590259095650209 | 8.70050336122676 | 148.886828797123 | Saint Vincent and the Grenadines |
| 2012 | 150.23825118381 | 0.592153907530225 | 8.81532750032579 | 141.422923683484 | Saint Vincent and the Grenadines |
| 2013 | 157.24493515344 | 0.594614074720551 | 8.97789155466078 | 148.267043598779 | Saint Vincent and the Grenadines |
| 2014 | 174.67555558278 | 0.598378671838632 | 8.7604755909682 | 165.915079991812 | Saint Vincent and the Grenadines |
| 2015 | 158.36569452751 | 0.603754134616218 | 8.79329502624443 | 149.572399501266 | Saint Vincent and the Grenadines |
| 2016 | 134.81824520963 | 0.610059210417339 | 8.80447801131392 | 126.013767198316 | Saint Vincent and the Grenadines |
| 2017 | 124.16334114287 | 0.616438472725892 | 8.99867089819957 | 115.16467024467 | Saint Vincent and the Grenadines |
| 2018 | 116.44755629489 | 0.622634944331176 | 8.95035399809478 | 107.497202296795 | Saint Vincent and the Grenadines |
| 2019 | 109.06702333838 | 0.628325985755241 | 7.87717387076192 | 101.189849467618 | Saint Vincent and the Grenadines |
| 2020 | 100.20891818179 | 0.632860904072373 | 6.88683717509228 | 93.3220810066977 | Saint Vincent and the Grenadines |
| 2021 | 99.79585770609 | 0.637195962512539 | 6.60984250447632 | 93.1860152016137 | Saint Vincent and the Grenadines |
| 1990 | 2066.38481530122 | 0.50205430482691 | 16.6207687847131 | 2049.76404651651 | Suriname |
| 1991 | 1993.31064809316 | 0.506655794671834 | 15.9183695865185 | 1977.39227850664 | Suriname |
| 1992 | 1918.16313910672 | 0.510959772316403 | 15.6670389459174 | 1902.4961001608 | Suriname |
| 1993 | 1807.24257267159 | 0.514419436992343 | 15.59569192322 | 1791.64688074837 | Suriname |
| 1994 | 1728.0272950243 | 0.517650245235505 | 15.3461108270864 | 1712.68118419721 | Suriname |
| 1995 | 1640.05190241463 | 0.521002622374316 | 15.1587462797318 | 1624.8931561349 | Suriname |
| 1996 | 1552.23945830089 | 0.525059318240488 | 15.0725692848932 | 1537.166889016 | Suriname |
| 1997 | 1469.33879792066 | 0.529750811513369 | 15.070481583583 | 1454.26831633708 | Suriname |
| 1998 | 1368.80569022248 | 0.534599696353116 | 15.0483286803994 | 1353.75736154208 | Suriname |
| 1999 | 1271.29012983432 | 0.538932791302378 | 15.0532820621588 | 1256.23684777216 | Suriname |
| 2000 | 1129.27081856986 | 0.543660023350067 | 15.0438383620496 | 1114.22698020781 | Suriname |
| 2001 | 1028.32422750855 | 0.548683523928075 | 14.5129773838965 | 1013.81125012465 | Suriname |
| 2002 | 992.3277367618 | 0.553879432238792 | 13.0468557845389 | 979.280880977261 | Suriname |
| 2003 | 938.73627340175 | 0.559418373479841 | 12.0353043116484 | 926.700969090102 | Suriname |
| 2004 | 885.22930896171 | 0.565109708384423 | 10.8407023111952 | 874.388606650515 | Suriname |
| 2005 | 837.32830529348 | 0.570462186195307 | 8.9633822545947 | 828.364923038885 | Suriname |
| 2006 | 790.28775735256 | 0.575647048246499 | 9.17263365329378 | 781.115123699266 | Suriname |
| 2007 | 766.75562143751 | 0.580654574084671 | 8.7920458680136 | 757.963575569496 | Suriname |
| 2008 | 726.98759292638 | 0.585644825421143 | 9.0122410148601 | 717.97535191152 | Suriname |
| 2009 | 690.83087502298 | 0.590512804838156 | 8.9946457777438 | 681.836229245236 | Suriname |
| 2010 | 650.45469999423 | 0.595469745192738 | 8.8749153405531 | 641.579784653677 | Suriname |
| 2011 | 608.39749524464 | 0.600356336126983 | 8.84186037754143 | 599.555634867099 | Suriname |
| 2012 | 578.26486534216 | 0.604741870150833 | 8.72763202502493 | 569.537233317135 | Suriname |
| 2013 | 558.26436500124 | 0.608910612397383 | 8.77195567757896 | 549.492409323661 | Suriname |
| 2014 | 535.7487179456 | 0.61270442287871 | 8.81261171750458 | 526.936106228095 | Suriname |
| 2015 | 513.43753774997 | 0.616097315384298 | 8.84217341382329 | 504.595364336147 | Suriname |
| 2016 | 502.05318086681 | 0.618868087951188 | 8.73423834020701 | 493.318942526603 | Suriname |
| 2017 | 502.72683998195 | 0.621684179841572 | 8.79432539213635 | 493.932514589814 | Suriname |
| 2018 | 489.8711661287 | 0.624708011000566 | 8.95561909975659 | 480.915547028943 | Suriname |
| 2019 | 480.36455964934 | 0.62789678100938 | 8.44645059825976 | 471.91810905108 | Suriname |
| 2020 | 453.4468383743 | 0.630683705083472 | 8.33197809844373 | 445.114860275856 | Suriname |
| 2021 | 441.59365246999 | 0.63366573910901 | 7.08326335637287 | 434.510389113617 | Suriname |
| 1990 | 348.55080461615 | 0.623970150133519 | 8.85355623398354 | 339.697248382166 | Trinidad and Tobago |
| 1991 | 322.27574488469 | 0.62875891817345 | 8.32994737429806 | 313.945797510392 | Trinidad and Tobago |
| 1992 | 313.37252831494 | 0.634039551508711 | 6.91321555403065 | 306.459312760909 | Trinidad and Tobago |
| 1993 | 346.09528280083 | 0.639191913980039 | 6.78879384454756 | 339.306488956282 | Trinidad and Tobago |
| 1994 | 328.87339676969 | 0.644242910113573 | 6.1317211442182 | 322.741675625472 | Trinidad and Tobago |
| 1995 | 310.87392370992 | 0.649220055474201 | 4.92921983833441 | 305.944703871586 | Trinidad and Tobago |
| 1996 | 291.2619343236 | 0.654169455231313 | 5.00310278939393 | 286.258831534206 | Trinidad and Tobago |
| 1997 | 283.13245696579 | 0.659293633341366 | 4.43777920332902 | 278.694677762461 | Trinidad and Tobago |
| 1998 | 268.46975385086 | 0.664507714329849 | 4.2095961251316 | 264.260157725728 | Trinidad and Tobago |
| 1999 | 261.01121191603 | 0.670244573860158 | 3.88541409689887 | 257.125797819131 | Trinidad and Tobago |
| 2000 | 232.9855596034 | 0.676683835395074 | 3.58057419417068 | 229.404985409229 | Trinidad and Tobago |
| 2001 | 183.42471148666 | 0.682763494477584 | 3.29805869165057 | 180.126652795009 | Trinidad and Tobago |
| 2002 | 166.79539783058 | 0.688872270964441 | 3.42374311604152 | 163.371654714538 | Trinidad and Tobago |
| 2003 | 164.36399287069 | 0.695828341273334 | 3.19408655242726 | 161.169906318263 | Trinidad and Tobago |
| 2004 | 136.89147961114 | 0.702610808512884 | 2.87892270468134 | 134.012556906459 | Trinidad and Tobago |
| 2005 | 145.85035065427 | 0.708520984658684 | 2.8097134839636 | 143.040637170306 | Trinidad and Tobago |
| 2006 | 136.11726429284 | 0.714600209518824 | 2.71380187178829 | 133.403462421052 | Trinidad and Tobago |
| 2007 | 120.29247022327 | 0.72019641678818 | 2.48606952645638 | 117.806400696814 | Trinidad and Tobago |
| 2008 | 123.57702846698 | 0.725720701823497 | 2.42399821702974 | 121.15303024995 | Trinidad and Tobago |
| 2009 | 112.70528803959 | 0.729538891843969 | 2.28728456982829 | 110.418003469762 | Trinidad and Tobago |
| 2010 | 101.17771227543 | 0.733237076528245 | 2.25895577521093 | 98.9187565002191 | Trinidad and Tobago |
| 2011 | 103.46924202737 | 0.736802404534694 | 2.08646814499582 | 101.382773882374 | Trinidad and Tobago |
| 2012 | 99.30176957696 | 0.739999073209787 | 1.86769899991631 | 97.4340705770437 | Trinidad and Tobago |
| 2013 | 94.74317595424 | 0.743593955236757 | 1.84472354913609 | 92.8984524051039 | Trinidad and Tobago |
| 2014 | 90.34884599919 | 0.747338962511986 | 1.85131858631971 | 88.4975274128703 | Trinidad and Tobago |
| 2015 | 84.97172969697 | 0.751510682495009 | 1.7866343135319 | 83.1850953834381 | Trinidad and Tobago |
| 2016 | 85.18300170658 | 0.754953347556518 | 1.66124142951488 | 83.5217602770651 | Trinidad and Tobago |
| 2017 | 79.30465280859 | 0.758226987870251 | 1.74815709868222 | 77.5564957099078 | Trinidad and Tobago |
| 2018 | 74.42106131167 | 0.761241990776049 | 1.74602444768747 | 72.6750368639825 | Trinidad and Tobago |
| 2019 | 73.54000812306 | 0.764169599010201 | 1.72028399426409 | 71.8197241287959 | Trinidad and Tobago |
| 2020 | 69.70974854336 | 0.766422080818681 | 1.751358126465 | 67.958390416895 | Trinidad and Tobago |
| 2021 | 69.02856853713 | 0.768763253980677 | 1.76405960786184 | 67.2645089292682 | Trinidad and Tobago |
| 1990 | 3307.24377189249 | 0.423917961315613 | 177.212404495761 | 3130.03136739673 | Bolivia (Plurinational State of) |
| 1991 | 3519.46498234138 | 0.429397795077356 | 135.590374872616 | 3383.87460746876 | Bolivia (Plurinational State of) |
| 1992 | 3391.59311455504 | 0.434835541945787 | 128.47234851003 | 3263.12076604501 | Bolivia (Plurinational State of) |
| 1993 | 3024.73632978697 | 0.440601645503372 | 127.961177196203 | 2896.77515259077 | Bolivia (Plurinational State of) |
| 1994 | 2576.17452905485 | 0.446822500209606 | 113.593500871442 | 2462.58102818341 | Bolivia (Plurinational State of) |
| 1995 | 2432.23998143763 | 0.45347924306274 | 105.588725339051 | 2326.65125609858 | Bolivia (Plurinational State of) |
| 1996 | 2299.55037452932 | 0.460220668843174 | 93.5896196777389 | 2205.96075485158 | Bolivia (Plurinational State of) |
| 1997 | 2056.64861868654 | 0.466982896503528 | 85.0530611669726 | 1971.59555751957 | Bolivia (Plurinational State of) |
| 1998 | 1906.2792576891 | 0.473796200765922 | 80.3343180998138 | 1825.94493958929 | Bolivia (Plurinational State of) |
| 1999 | 1769.36369395678 | 0.480194310456872 | 76.7388844904167 | 1692.62480946636 | Bolivia (Plurinational State of) |
| 2000 | 1646.33724596167 | 0.486327903067974 | 74.988730768253 | 1571.34851519342 | Bolivia (Plurinational State of) |
| 2001 | 1541.90295894846 | 0.492030763865071 | 72.2700369661949 | 1469.63292198227 | Bolivia (Plurinational State of) |
| 2002 | 1438.85894572804 | 0.497348836505264 | 23.4323225784588 | 1415.42662314958 | Bolivia (Plurinational State of) |
| 2003 | 1338.51122282081 | 0.502365802781048 | 16.6059122020942 | 1321.90531061872 | Bolivia (Plurinational State of) |
| 2004 | 1245.71036389684 | 0.507273035649156 | 15.929007404068 | 1229.78135649277 | Bolivia (Plurinational State of) |
| 2005 | 1170.98165950731 | 0.512166491375209 | 15.6464309472551 | 1155.33522856005 | Bolivia (Plurinational State of) |
| 2006 | 1091.13099677338 | 0.517146193689981 | 15.2679997645785 | 1075.8629970088 | Bolivia (Plurinational State of) |
| 2007 | 1020.80534235123 | 0.521946130357677 | 15.1903814340354 | 1005.61496091719 | Bolivia (Plurinational State of) |
| 2008 | 900.1031161368 | 0.526943750292372 | 15.1198546221054 | 884.983261514695 | Bolivia (Plurinational State of) |
| 2009 | 793.855857496 | 0.531792440464007 | 15.0959307042761 | 778.759926791724 | Bolivia (Plurinational State of) |
| 2010 | 737.40565862984 | 0.5370090684329 | 15.0731742823921 | 722.332484347448 | Bolivia (Plurinational State of) |
| 2011 | 683.92917876369 | 0.542680744993221 | 15.0153384194821 | 668.913840344208 | Bolivia (Plurinational State of) |
| 2012 | 635.60912787319 | 0.548615481889587 | 14.4712011872414 | 621.137926685949 | Bolivia (Plurinational State of) |
| 2013 | 595.63420849982 | 0.554788215843767 | 13.1387534033049 | 582.495455096515 | Bolivia (Plurinational State of) |
| 2014 | 554.70383138891 | 0.560934581048908 | 11.4287269384301 | 543.27510445048 | Bolivia (Plurinational State of) |
| 2015 | 516.34927866306 | 0.567000897613403 | 9.72329522723027 | 506.62598343583 | Bolivia (Plurinational State of) |
| 2016 | 479.7272390328 | 0.573080316306652 | 9.02326254381176 | 470.703976488988 | Bolivia (Plurinational State of) |
| 2017 | 447.5125852729 | 0.579241284614228 | 8.79137582274953 | 438.72120945015 | Bolivia (Plurinational State of) |
| 2018 | 416.37648703367 | 0.585184106450667 | 9.12539263641261 | 407.251094397257 | Bolivia (Plurinational State of) |
| 2019 | 392.27010205399 | 0.590692043089904 | 8.81766179861593 | 383.452440255374 | Bolivia (Plurinational State of) |
| 2020 | 371.13350525021 | 0.594854443460131 | 8.93490835239506 | 362.198596897815 | Bolivia (Plurinational State of) |
| 2021 | 351.59257698 | 0.5990107988623 | 8.85955183220701 | 342.733025147793 | Bolivia (Plurinational State of) |
| 1990 | 1745.51544924735 | 0.518430614137285 | 15.2460411702641 | 1730.26940807709 | Ecuador |
| 1991 | 2099.1331646042 | 0.517679421435991 | 15.364584493208 | 2083.76858011099 | Ecuador |
| 1992 | 1730.63944558341 | 0.518379070003192 | 15.2449781885998 | 1715.39446739481 | Ecuador |
| 1993 | 1246.26197764332 | 0.520707290609383 | 15.1603395853147 | 1231.10163805801 | Ecuador |
| 1994 | 935.48749961115 | 0.524908290908027 | 15.0701308622127 | 920.417368748937 | Ecuador |
| 1995 | 903.45560602046 | 0.529380751671009 | 15.1230664464894 | 888.332539573971 | Ecuador |
| 1996 | 893.90807216024 | 0.532691399133141 | 15.1368609524098 | 878.77121120783 | Ecuador |
| 1997 | 823.78845402806 | 0.535349652252826 | 15.0992966476594 | 808.689157380401 | Ecuador |
| 1998 | 796.86001102327 | 0.537196329914338 | 15.067962111804 | 781.792048911466 | Ecuador |
| 1999 | 714.80223948129 | 0.53903230782875 | 15.1025132864862 | 699.699726194804 | Ecuador |
| 2000 | 689.20825205087 | 0.5430447789024 | 15.0177246891681 | 674.190527361702 | Ecuador |
| 2001 | 651.41134109705 | 0.548552149690973 | 14.5014534811821 | 636.909887615868 | Ecuador |
| 2002 | 584.04138118407 | 0.554897325204538 | 12.8803193239999 | 571.16106186007 | Ecuador |
| 2003 | 535.79452809566 | 0.560955484518967 | 11.398343651615 | 524.396184444045 | Ecuador |
| 2004 | 477.23872534255 | 0.566290292625305 | 10.2965164235536 | 466.942208918996 | Ecuador |
| 2005 | 425.63815738679 | 0.569927344748438 | 8.85931618299476 | 416.778841203795 | Ecuador |
| 2006 | 376.75682816059 | 0.572516273171735 | 8.61660553607667 | 368.140222624513 | Ecuador |
| 2007 | 339.63918070223 | 0.574704702310921 | 8.86993843389077 | 330.769242268339 | Ecuador |
| 2008 | 282.82768212846 | 0.578125040269336 | 9.01878730164968 | 273.80889482681 | Ecuador |
| 2009 | 250.94915516478 | 0.58248425757379 | 8.82801238293435 | 242.121142781846 | Ecuador |
| 2010 | 196.85596646723 | 0.588763055747133 | 8.91441155032783 | 187.941554916902 | Ecuador |
| 2011 | 160.42373453138 | 0.596729076106647 | 8.70766034426083 | 151.716074187119 | Ecuador |
| 2012 | 131.92923339112 | 0.605454319333254 | 8.85959134445122 | 123.069642046669 | Ecuador |
| 2013 | 103.98409653315 | 0.614080274696922 | 8.78321690893434 | 95.2008796242156 | Ecuador |
| 2014 | 95.00827394697 | 0.622027432994838 | 8.98251076359675 | 86.0257631833732 | Ecuador |
| 2015 | 93.95438677838 | 0.628546709567119 | 8.37317657925178 | 85.5812101991282 | Ecuador |
| 2016 | 81.02082523915 | 0.634281142866422 | 7.13096555012847 | 73.8898596890215 | Ecuador |
| 2017 | 65.55197445256 | 0.640011218750157 | 6.56442102467969 | 58.9875534278803 | Ecuador |
| 2018 | 61.87815955971 | 0.645888520186892 | 5.73663838952901 | 56.141521170181 | Ecuador |
| 2019 | 65.33247208903 | 0.651787987044895 | 5.03217422898557 | 60.3002978600444 | Ecuador |
| 2020 | 55.06558179103 | 0.65671445724893 | 4.8219152974718 | 50.2436664935582 | Ecuador |
| 2021 | 51.07963794254 | 0.66101705262103 | 4.3698434818227 | 46.7097944607173 | Ecuador |
| 1990 | 1674.40192211374 | 0.510419852074136 | 15.6892445986568 | 1658.71267751508 | Peru |
| 1991 | 2186.52542487265 | 0.512161524663903 | 15.709311222582 | 2170.81611365007 | Peru |
| 1992 | 1666.84439580938 | 0.513821551605533 | 15.7549013554441 | 1651.08949445394 | Peru |
| 1993 | 1480.15603811329 | 0.515981506410929 | 15.2973923552395 | 1464.85864575805 | Peru |
| 1994 | 1196.58481843413 | 0.519306080333588 | 15.3609230492493 | 1181.22389538488 | Peru |
| 1995 | 1068.14026417696 | 0.523640526677899 | 15.2191886461651 | 1052.9210755308 | Peru |
| 1996 | 918.86338441294 | 0.528312042097199 | 15.0983867858814 | 903.764997627059 | Peru |
| 1997 | 824.30722853395 | 0.533576491199309 | 15.0590639141524 | 809.248164619798 | Peru |
| 1998 | 758.0331150351 | 0.538721717199262 | 15.0492357679498 | 742.98387926715 | Peru |
| 1999 | 679.3273909822 | 0.543853376891845 | 15.026694695691 | 664.300696286509 | Peru |
| 2000 | 607.91621640273 | 0.548952201243799 | 14.5044454780903 | 593.41177092464 | Peru |
| 2001 | 534.31290055881 | 0.553775453477556 | 13.2296444875931 | 521.083256071217 | Peru |
| 2002 | 490.26806969305 | 0.558853262904608 | 12.0010387151957 | 478.267030977854 | Peru |
| 2003 | 460.08584068328 | 0.563871071930545 | 10.7302054806331 | 449.355635202647 | Peru |
| 2004 | 429.01642526208 | 0.56906302441994 | 9.42489107244774 | 419.591534189632 | Peru |
| 2005 | 398.28709003477 | 0.574517001323253 | 8.91476962560592 | 389.372320409164 | Peru |
| 2006 | 359.74701199811 | 0.580503364933972 | 8.84602673191521 | 350.900985266195 | Peru |
| 2007 | 316.00389032237 | 0.586958489258033 | 9.06518458307861 | 306.938705739291 | Peru |
| 2008 | 292.45859378204 | 0.593750810824365 | 8.83563682496027 | 283.62295695708 | Peru |
| 2009 | 255.53133352376 | 0.599954992710361 | 8.78296387802534 | 246.748369645735 | Peru |
| 2010 | 225.07729769483 | 0.606520931663104 | 8.91625000806535 | 216.161047686765 | Peru |
| 2011 | 201.83428773981 | 0.613051220911462 | 8.71498242274324 | 193.119305317067 | Peru |
| 2012 | 183.71059281598 | 0.619355264313384 | 8.79045934601986 | 174.92013346996 | Peru |
| 2013 | 171.46134298532 | 0.62551902425403 | 8.7608127168257 | 162.700530268494 | Peru |
| 2014 | 161.94318291773 | 0.631114354280049 | 8.38846386139158 | 153.554719056338 | Peru |
| 2015 | 152.71271735904 | 0.636278270548633 | 7.11753457985609 | 145.595182779184 | Peru |
| 2016 | 147.87882173302 | 0.641285279069023 | 6.1958878167132 | 141.682933916307 | Peru |
| 2017 | 143.83606598337 | 0.64618085494837 | 5.89443301763143 | 137.941632965739 | Peru |
| 2018 | 142.79528579261 | 0.650977317324287 | 5.09770378984742 | 137.697582002763 | Peru |
| 2019 | 128.10868633983 | 0.655433734747709 | 5.02501612269843 | 123.083670217132 | Peru |
| 2020 | 109.66662307882 | 0.658672243500517 | 4.50049193976694 | 105.166131139053 | Peru |
| 2021 | 97.86902258669 | 0.662054036755862 | 4.61905460469537 | 93.2499679819946 | Peru |
| 1990 | 845.52092627702 | 0.480720053627064 | 77.3736345692621 | 768.147291707758 | Colombia |
| 1991 | 790.45834241445 | 0.482339893974629 | 77.9278695732475 | 712.530472841202 | Colombia |
| 1992 | 775.7363940997 | 0.484572358989267 | 76.7005947665724 | 699.035799333128 | Colombia |
| 1993 | 684.08247418038 | 0.488229373054794 | 75.50802373322 | 608.57445044716 | Colombia |
| 1994 | 615.54007392215 | 0.493278181852073 | 73.5698974336533 | 541.970176488497 | Colombia |
| 1995 | 552.6493405718 | 0.49910275736379 | 17.6025050776416 | 535.046835494158 | Colombia |
| 1996 | 495.62258153666 | 0.504986969396651 | 16.2101277129294 | 479.412453823731 | Colombia |
| 1997 | 446.60537109883 | 0.511079502295084 | 15.7150371049857 | 430.890333993844 | Colombia |
| 1998 | 408.67467902089 | 0.517022504946273 | 15.3037779422918 | 393.370901078598 | Colombia |
| 1999 | 335.69476994966 | 0.522435940409787 | 15.1343239858219 | 320.560445963838 | Colombia |
| 2000 | 296.09554735005 | 0.527839094091296 | 15.0699998265349 | 281.025547523515 | Colombia |
| 2001 | 256.56795141233 | 0.533076500590306 | 15.109992742803 | 241.457958669527 | Colombia |
| 2002 | 282.59598625578 | 0.538189342486614 | 15.0592971375991 | 267.536689118181 | Colombia |
| 2003 | 278.28160240412 | 0.54326513104442 | 15.0256925019874 | 263.255909902133 | Colombia |
| 2004 | 239.04299669589 | 0.548601831852621 | 14.4928642082786 | 224.550132487611 | Colombia |
| 2005 | 203.57851677753 | 0.554089190974456 | 13.3059511771515 | 190.272565600379 | Colombia |
| 2006 | 178.98604639519 | 0.56018273477503 | 11.961774455786 | 167.024271939404 | Colombia |
| 2007 | 161.9058889635 | 0.566822644330018 | 9.79541974319224 | 152.110469220308 | Colombia |
| 2008 | 136.74996718206 | 0.573494651915472 | 8.78649277061412 | 127.963474411446 | Colombia |
| 2009 | 122.0812567538 | 0.579876468776209 | 9.15030793685206 | 112.930948816948 | Colombia |
| 2010 | 94.57786810701 | 0.586408108388396 | 8.84305784128892 | 85.7348102657211 | Colombia |
| 2011 | 76.12276936316 | 0.593340477060868 | 9.03552944137973 | 67.0872399217803 | Colombia |
| 2012 | 70.1142439071 | 0.600254237718889 | 8.80594510577484 | 61.3082988013252 | Colombia |
| 2013 | 68.10631172946 | 0.607250636585575 | 8.87231194821316 | 59.2339997812468 | Colombia |
| 2014 | 66.77232617919 | 0.614151922522411 | 8.82311233192057 | 57.9492138472694 | Colombia |
| 2015 | 67.32321524264 | 0.620889228107476 | 9.04630374447708 | 58.2769114981629 | Colombia |
| 2016 | 62.60562323105 | 0.627554497248436 | 8.49435681232817 | 54.1112664187218 | Colombia |
| 2017 | 60.94175905437 | 0.634130359426935 | 7.0080813339223 | 53.9336777204477 | Colombia |
| 2018 | 67.34312801751 | 0.640255071652482 | 6.52430871186609 | 60.8188193056439 | Colombia |
| 2019 | 84.64665357084 | 0.646038852086803 | 6.12084549395731 | 78.5258080768827 | Colombia |
| 2020 | 72.0055300064 | 0.650855354021142 | 4.824705555185 | 67.180824451215 | Colombia |
| 2021 | 66.05953423521 | 0.655442912568827 | 4.74313019113759 | 61.3164040440724 | Colombia |
| 1990 | 230.64995124762 | 0.534125181350537 | 15.1234154367906 | 215.526535810829 | Costa Rica |
| 1991 | 189.8043128358 | 0.539342163753933 | 15.0959582632763 | 174.708354572524 | Costa Rica |
| 1992 | 161.21626473428 | 0.5446242375755 | 15.0457839044097 | 146.17048082987 | Costa Rica |
| 1993 | 151.40028898349 | 0.549946625586597 | 13.7605931359708 | 137.639695847519 | Costa Rica |
| 1994 | 148.772354306 | 0.555171250068523 | 12.7535337335236 | 136.018820572476 | Costa Rica |
| 1995 | 140.95063220157 | 0.560547086077833 | 11.9096854200722 | 129.040946781498 | Costa Rica |
| 1996 | 132.86356829902 | 0.565180861960769 | 10.178300870555 | 122.685267428465 | Costa Rica |
| 1997 | 133.06404468009 | 0.570128417464587 | 8.70150580456653 | 124.362538875523 | Costa Rica |
| 1998 | 121.68692210073 | 0.576067050570833 | 8.98921314549634 | 112.697708955234 | Costa Rica |
| 1999 | 104.31981860274 | 0.582229491567193 | 8.7115389077347 | 95.6082796950053 | Costa Rica |
| 2000 | 86.78190470714 | 0.588243191082194 | 8.81745695323609 | 77.9644477539039 | Costa Rica |
| 2001 | 81.05355825052 | 0.594889114531861 | 9.07307648340545 | 71.9804817671145 | Costa Rica |
| 2002 | 73.08111985268 | 0.600894432967727 | 8.84813064246928 | 64.2329892102107 | Costa Rica |
| 2003 | 67.7368835974 | 0.605765686833733 | 9.13628821320395 | 58.600595384196 | Costa Rica |
| 2004 | 57.93125858358 | 0.610315699466228 | 8.79516582861735 | 49.1360927549626 | Costa Rica |
| 2005 | 52.77513716336 | 0.614412618734781 | 9.00995900451814 | 43.7651781588419 | Costa Rica |
| 2006 | 45.61044223938 | 0.617850162499434 | 8.76928078986676 | 36.8411614495132 | Costa Rica |
| 2007 | 47.30779611098 | 0.621200571426243 | 9.10092580999051 | 38.2068703009895 | Costa Rica |
| 2008 | 42.12081625169 | 0.625703910462395 | 8.89750118685172 | 33.2233150648383 | Costa Rica |
| 2009 | 40.1111967719 | 0.631291379026346 | 8.09880875439621 | 32.0123880175038 | Costa Rica |
| 2010 | 38.95185241053 | 0.63659542601002 | 6.94845690480129 | 32.0033955057287 | Costa Rica |
| 2011 | 34.75911365508 | 0.641201295145018 | 6.25992797082397 | 28.499185684256 | Costa Rica |
| 2012 | 34.18280997049 | 0.646574092912526 | 6.48300379484033 | 27.6998061756497 | Costa Rica |
| 2013 | 29.98052168174 | 0.651945812639188 | 4.95300205322071 | 25.0275196285193 | Costa Rica |
| 2014 | 32.64624049615 | 0.656890456365803 | 4.8840124468896 | 27.7622280492604 | Costa Rica |
| 2015 | 29.97644061101 | 0.662030135918026 | 4.14687086238026 | 25.8295697486297 | Costa Rica |
| 2016 | 31.40948872134 | 0.667430091500307 | 3.87234266590775 | 27.5371460554323 | Costa Rica |
| 2017 | 29.99606448069 | 0.673621391630886 | 3.80643113937814 | 26.1896333413119 | Costa Rica |
| 2018 | 29.95040970677 | 0.681296147310122 | 3.57720210678072 | 26.3732075999893 | Costa Rica |
| 2019 | 35.79672136808 | 0.690049088214507 | 3.33449502836176 | 32.4622263397182 | Costa Rica |
| 2020 | 30.95393920025 | 0.696194605172093 | 3.12232096548406 | 27.8316182347659 | Costa Rica |
| 2021 | 29.67816963458 | 0.700340476999325 | 2.84772474233231 | 26.8304448922477 | Costa Rica |
| 1990 | 2352.25427686084 | 0.373057974861269 | 354.236896262012 | 1998.01738059883 | El Salvador |
| 1991 | 2320.43591684123 | 0.374855325024228 | 352.552298345203 | 1967.88361849603 | El Salvador |
| 1992 | 2015.62775493848 | 0.377076642695912 | 346.615892943242 | 1669.01186199524 | El Salvador |
| 1993 | 1774.01442629897 | 0.380307326030095 | 324.391843202702 | 1449.62258309627 | El Salvador |
| 1994 | 1629.30929865378 | 0.38514909404887 | 314.369632488656 | 1314.93966616512 | El Salvador |
| 1995 | 1419.16352694132 | 0.391780266086918 | 285.29326355187 | 1133.87026338945 | El Salvador |
| 1996 | 1255.46280559671 | 0.400178467721391 | 260.764612037592 | 994.698193559118 | El Salvador |
| 1997 | 1092.34409557256 | 0.410455825709854 | 221.198518563071 | 871.145577009489 | El Salvador |
| 1998 | 939.59216180817 | 0.421421189598308 | 196.358110495291 | 743.234051312879 | El Salvador |
| 1999 | 794.14459300873 | 0.432733147562664 | 139.70629508046 | 654.43829792827 | El Salvador |
| 2000 | 682.77216414055 | 0.443962665887944 | 111.124372333887 | 571.647791806663 | El Salvador |
| 2001 | 587.92997275338 | 0.454177658570425 | 106.493228894485 | 481.436743858895 | El Salvador |
| 2002 | 514.75161789702 | 0.462968859826757 | 92.8477490036477 | 421.903868893372 | El Salvador |
| 2003 | 450.55797454168 | 0.470397412659385 | 82.8065627769406 | 367.751411764739 | El Salvador |
| 2004 | 405.75996342263 | 0.476536664263465 | 78.1173054947636 | 327.642657927866 | El Salvador |
| 2005 | 365.52843118971 | 0.48204171929172 | 77.2449495265339 | 288.283481663176 | El Salvador |
| 2006 | 304.77803435763 | 0.486826000431208 | 75.7120259189006 | 229.066008438729 | El Salvador |
| 2007 | 273.28325656836 | 0.490809751570084 | 75.369542413555 | 197.913714154805 | El Salvador |
| 2008 | 252.38044439598 | 0.495077738859345 | 70.698353234309 | 181.682091161671 | El Salvador |
| 2009 | 249.61395667196 | 0.499489714249212 | 17.3585504490936 | 232.255406222866 | El Salvador |
| 2010 | 228.22827585063 | 0.504171335202455 | 16.1531752931959 | 212.075100557434 | El Salvador |
| 2011 | 211.82941010457 | 0.508969239001561 | 15.9819407359377 | 195.847469368632 | El Salvador |
| 2012 | 196.14667477924 | 0.513396874335326 | 15.7989419652249 | 180.347732814015 | El Salvador |
| 2013 | 182.797032704 | 0.518121907716899 | 15.4182842858475 | 167.378748418152 | El Salvador |
| 2014 | 167.9775468487 | 0.523468900855703 | 15.1917490425322 | 152.785797806168 | El Salvador |
| 2015 | 151.86459673772 | 0.529386950266238 | 15.0617156671445 | 136.802881070576 | El Salvador |
| 2016 | 141.58622008994 | 0.535650269148693 | 15.0899571924362 | 126.496262897504 | El Salvador |
| 2017 | 132.74767251523 | 0.541998957448254 | 15.0536293908745 | 117.694043124356 | El Salvador |
| 2018 | 124.98782847573 | 0.548143173580896 | 14.6308901708703 | 110.35693830486 | El Salvador |
| 2019 | 118.14957608494 | 0.55403164778242 | 13.1060062144938 | 105.043569870446 | El Salvador |
| 2020 | 110.50023070175 | 0.558919894116032 | 12.0605391015312 | 98.4396916002188 | El Salvador |
| 2021 | 105.05930000295 | 0.563775187564695 | 10.8288111264261 | 94.2304888765239 | El Salvador |
| 1990 | 3764.09811181403 | 0.311792455277134 | 870.527631312095 | 2893.57048050193 | Guatemala |
| 1991 | 3612.07400412526 | 0.314329483676405 | 865.128125551317 | 2746.94587857394 | Guatemala |
| 1992 | 3105.83371520083 | 0.319165481239087 | 772.74136533094 | 2333.09234986989 | Guatemala |
| 1993 | 2859.74567487176 | 0.326840818669512 | 722.06170474109 | 2137.68397013067 | Guatemala |
| 1994 | 2771.49815291596 | 0.33346910005957 | 649.808317066244 | 2121.68983584972 | Guatemala |
| 1995 | 2600.51028846607 | 0.339642100810868 | 601.185387073355 | 1999.32490139271 | Guatemala |
| 1996 | 2460.15925401316 | 0.346961212155122 | 563.016934059972 | 1897.14231995319 | Guatemala |
| 1997 | 2300.84908532006 | 0.355995963296002 | 553.428538816292 | 1747.42054650377 | Guatemala |
| 1998 | 2259.80940868878 | 0.366461192871903 | 415.93691744104 | 1843.87249124774 | Guatemala |
| 1999 | 2046.78995402638 | 0.374455316256445 | 353.761473238159 | 1693.02848078822 | Guatemala |
| 2000 | 2078.79271129641 | 0.379877296535484 | 350.13588611716 | 1728.65682517925 | Guatemala |
| 2001 | 2041.89280983054 | 0.386927685682523 | 321.543914486504 | 1720.34889534404 | Guatemala |
| 2002 | 1931.63434184499 | 0.396899639101221 | 260.233034480162 | 1671.40130736483 | Guatemala |
| 2003 | 1761.9352249839 | 0.408234164151934 | 238.630403895198 | 1523.3048210887 | Guatemala |
| 2004 | 1604.86713619377 | 0.419777064914249 | 193.009530273227 | 1411.85760592054 | Guatemala |
| 2005 | 1516.07338418395 | 0.430327529380029 | 135.820945586873 | 1380.25243859708 | Guatemala |
| 2006 | 1377.11551428846 | 0.438856375714513 | 129.756352522684 | 1247.35916176578 | Guatemala |
| 2007 | 1340.55614718463 | 0.447245964554675 | 110.704589329867 | 1229.85155785476 | Guatemala |
| 2008 | 1283.75431323228 | 0.456541865045794 | 99.4783525197977 | 1184.27596071248 | Guatemala |
| 2009 | 1167.56252236369 | 0.464003076644959 | 91.2969230438448 | 1076.26559931985 | Guatemala |
| 2010 | 1131.14719706566 | 0.470477363646759 | 82.8962622704502 | 1048.25093479521 | Guatemala |
| 2011 | 1104.17234541715 | 0.476909684291273 | 78.8772994196948 | 1025.29504599746 | Guatemala |
| 2012 | 1084.5660280738 | 0.481862853245964 | 76.6324939872795 | 1007.93353408652 | Guatemala |
| 2013 | 1033.81252438804 | 0.487286692231106 | 75.1927886170092 | 958.619735771031 | Guatemala |
| 2014 | 1030.55994636711 | 0.494027188122075 | 71.2652439472892 | 959.294702419821 | Guatemala |
| 2015 | 991.57715395219 | 0.500655606612947 | 16.554699362289 | 975.022454589901 | Guatemala |
| 2016 | 1008.8871610769 | 0.507149791967016 | 15.9533019847702 | 992.93385909213 | Guatemala |
| 2017 | 908.85952351838 | 0.51373568188905 | 15.6833477706074 | 893.176175747773 | Guatemala |
| 2018 | 850.24366230708 | 0.52099546880295 | 15.1604735149062 | 835.083188792174 | Guatemala |
| 2019 | 879.94846212175 | 0.528630469662807 | 15.0849008292729 | 864.863561292477 | Guatemala |
| 2020 | 750.595983024 | 0.534571414248156 | 15.0716131989721 | 735.524369825028 | Guatemala |
| 2021 | 703.92997877345 | 0.539972423611408 | 15.0288268998057 | 688.901151873644 | Guatemala |
| 1990 | 5508.67836483002 | 0.332042889051331 | 647.866037240134 | 4860.81232758989 | Honduras |
| 1991 | 5229.6369643497 | 0.337034649170168 | 642.901259468946 | 4586.73570488075 | Honduras |
| 1992 | 4888.5420920228 | 0.342305656800435 | 571.322095239254 | 4317.21999678355 | Honduras |
| 1993 | 4732.84017927207 | 0.347838445661094 | 575.03059880433 | 4157.80958046774 | Honduras |
| 1994 | 4391.26494442431 | 0.353164851646287 | 553.615491476662 | 3837.64945294765 | Honduras |
| 1995 | 3992.2686295891 | 0.358787268251117 | 497.960673409457 | 3494.30795617964 | Honduras |
| 1996 | 3498.31656985749 | 0.364328424323186 | 457.419951355534 | 3040.89661850196 | Honduras |
| 1997 | 3150.21539402598 | 0.369983893774573 | 400.077288239679 | 2750.1381057863 | Honduras |
| 1998 | 2881.84301854966 | 0.375950111095537 | 358.444217369626 | 2523.39880118003 | Honduras |
| 1999 | 2603.02851135177 | 0.38175231641304 | 324.567691690192 | 2278.46081966158 | Honduras |
| 2000 | 2364.01324292557 | 0.387988052509884 | 284.167259155927 | 2079.84598376964 | Honduras |
| 2001 | 2175.25762635584 | 0.394174011784655 | 287.615400643345 | 1887.6422257125 | Honduras |
| 2002 | 2011.08212063366 | 0.400371798466223 | 261.077376183115 | 1750.00474445054 | Honduras |
| 2003 | 1868.34897112291 | 0.406668380994452 | 237.605874504465 | 1630.74309661845 | Honduras |
| 2004 | 1748.22757225412 | 0.413061815539536 | 214.665843030195 | 1533.56172922393 | Honduras |
| 2005 | 1633.11662290829 | 0.419612331829645 | 193.902851762829 | 1439.21377114546 | Honduras |
| 2006 | 1539.4220392119 | 0.426352996817521 | 177.443783517153 | 1361.97825569475 | Honduras |
| 2007 | 1440.85841113704 | 0.433240314987847 | 132.392654815966 | 1308.46575632107 | Honduras |
| 2008 | 1378.99299665844 | 0.439998163793669 | 129.063761540993 | 1249.92923511745 | Honduras |
| 2009 | 1201.86459573608 | 0.446151054184542 | 109.835785980744 | 1092.02880975534 | Honduras |
| 2010 | 1137.08790224172 | 0.452382226389866 | 106.0023039446 | 1031.08559829712 | Honduras |
| 2011 | 1080.05094411361 | 0.458700328960259 | 93.2716291240397 | 986.77931498957 | Honduras |
| 2012 | 1035.99386962777 | 0.46492515492495 | 91.5368550757722 | 944.457014551998 | Honduras |
| 2013 | 1017.39640211008 | 0.470766833403007 | 82.634407704492 | 934.761994405588 | Honduras |
| 2014 | 990.94189188363 | 0.476587799009017 | 77.9082018524656 | 913.033690031164 | Honduras |
| 2015 | 942.46091517158 | 0.482294176352814 | 76.6806194927191 | 865.780295678861 | Honduras |
| 2016 | 892.72679012812 | 0.487705931888242 | 75.6225375550749 | 817.104252573045 | Honduras |
| 2017 | 850.96194150964 | 0.493130694434939 | 73.2020120192516 | 777.759929490388 | Honduras |
| 2018 | 791.32079431223 | 0.498657226690384 | 18.3538201592524 | 772.966974152978 | Honduras |
| 2019 | 737.53163327779 | 0.504156505325121 | 16.1957330041549 | 721.335900273635 | Honduras |
| 2020 | 686.7758069359 | 0.508669245938408 | 15.9182490427257 | 670.857557893174 | Honduras |
| 2021 | 651.41749170227 | 0.513037248307344 | 15.6212846377786 | 635.796207064491 | Honduras |
| 1990 | 2156.91085672004 | 0.504996083384899 | 16.2672680919141 | 2140.64358862813 | Mexico |
| 1991 | 1896.95928446064 | 0.509510670028127 | 15.9334485757888 | 1881.02583588485 | Mexico |
| 1992 | 1557.69753152991 | 0.514638056848767 | 15.6885608387977 | 1542.00897069111 | Mexico |
| 1993 | 1325.81041435308 | 0.52049316730006 | 15.1483308990357 | 1310.66208345404 | Mexico |
| 1994 | 1089.51322870753 | 0.526749240631648 | 15.1003803141354 | 1074.41284839339 | Mexico |
| 1995 | 942.23874390718 | 0.531554141210122 | 15.0382261993867 | 927.200517707793 | Mexico |
| 1996 | 814.86705759781 | 0.536530826504039 | 15.0715225541302 | 799.79553504368 | Mexico |
| 1997 | 723.87654666523 | 0.541988171199547 | 15.0481721271571 | 708.828374538073 | Mexico |
| 1998 | 630.1579360715 | 0.547842121275417 | 14.5753026838543 | 615.582633387646 | Mexico |
| 1999 | 551.95009865219 | 0.553813369752955 | 12.9463309436083 | 539.003767708582 | Mexico |
| 2000 | 500.27266057595 | 0.559532546937005 | 11.9479713035449 | 488.324689272405 | Mexico |
| 2001 | 461.65192154423 | 0.564774176443707 | 11.0373867560807 | 450.614534788149 | Mexico |
| 2002 | 427.32170901623 | 0.570113531020311 | 8.65723117705384 | 418.664477839176 | Mexico |
| 2003 | 397.79854064363 | 0.575698214652787 | 8.79628107274134 | 389.002259570889 | Mexico |
| 2004 | 360.50639660611 | 0.580984982494851 | 8.82487975303921 | 351.681516853071 | Mexico |
| 2005 | 341.42099043839 | 0.585224080225789 | 8.7949703398115 | 332.626020098578 | Mexico |
| 2006 | 304.09620194847 | 0.588543226375938 | 8.85816141658661 | 295.238040531883 | Mexico |
| 2007 | 282.60157590692 | 0.591447407661459 | 8.85479354568663 | 273.746782361233 | Mexico |
| 2008 | 244.43717155333 | 0.594454558028247 | 8.87421050996341 | 235.562961043367 | Mexico |
| 2009 | 203.70979543774 | 0.596684357960512 | 8.88517064909868 | 194.824624788641 | Mexico |
| 2010 | 175.78175074465 | 0.599527028083825 | 9.03388379409779 | 166.747866950552 | Mexico |
| 2011 | 163.36943278015 | 0.60364609507818 | 8.8282807646008 | 154.541152015549 | Mexico |
| 2012 | 158.6700893146 | 0.608914289338676 | 8.68500052528753 | 149.985088789312 | Mexico |
| 2013 | 155.67400886289 | 0.615089992820613 | 8.8593416255418 | 146.814667237348 | Mexico |
| 2014 | 136.78867965266 | 0.621857404659516 | 8.67918897313544 | 128.109490679525 | Mexico |
| 2015 | 125.39591470201 | 0.628896723914502 | 8.26133587597541 | 117.134578826035 | Mexico |
| 2016 | 117.76455358626 | 0.635925348800174 | 6.8257685408913 | 110.938785045369 | Mexico |
| 2017 | 99.50030397329 | 0.642667198400828 | 6.14410366299138 | 93.3562003102986 | Mexico |
| 2018 | 91.63231785271 | 0.649053317386265 | 5.03971988983557 | 86.5925979628744 | Mexico |
| 2019 | 94.92321793676 | 0.655095338007006 | 4.80327358086901 | 90.119944355891 | Mexico |
| 2020 | 90.54671621155 | 0.660119061584711 | 4.48722503569382 | 86.0594911758562 | Mexico |
| 2021 | 88.67619397747 | 0.664575304259183 | 4.41871115061058 | 84.2574828268594 | Mexico |
| 1990 | 2200.95302787019 | 0.346035234504054 | 570.314073940521 | 1630.63895392967 | Nicaragua |
| 1991 | 1977.00473403418 | 0.35174062237875 | 552.317617366722 | 1424.68711666746 | Nicaragua |
| 1992 | 1803.24478391075 | 0.357712704444006 | 493.517230775363 | 1309.72755313539 | Nicaragua |
| 1993 | 1780.6833418106 | 0.36356206850317 | 467.906157247971 | 1312.77718456263 | Nicaragua |
| 1994 | 1536.1666922173 | 0.369727967895652 | 403.310055476807 | 1132.85663674049 | Nicaragua |
| 1995 | 1407.13065861001 | 0.37662534522155 | 350.253120893827 | 1056.87753771618 | Nicaragua |
| 1996 | 1208.41131683362 | 0.384087266113663 | 317.556730368258 | 890.854586465362 | Nicaragua |
| 1997 | 1007.5695707048 | 0.391775893494114 | 288.511294696833 | 719.058276007967 | Nicaragua |
| 1998 | 883.92851878553 | 0.399807534850283 | 264.065345240704 | 619.863173544826 | Nicaragua |
| 1999 | 753.69900618342 | 0.408085964812584 | 240.35992360881 | 513.33908257461 | Nicaragua |
| 2000 | 658.46913014733 | 0.416108362910212 | 201.227173736486 | 457.241956410843 | Nicaragua |
| 2001 | 584.29947926988 | 0.423790295277938 | 176.685051235269 | 407.614428034611 | Nicaragua |
| 2002 | 513.3224415568 | 0.43081375452391 | 142.990568987652 | 370.331872569148 | Nicaragua |
| 2003 | 449.94070213997 | 0.437248152494534 | 128.866367351986 | 321.074334787984 | Nicaragua |
| 2004 | 401.32276023514 | 0.443274510891091 | 113.799032606439 | 287.523727628701 | Nicaragua |
| 2005 | 366.85082387007 | 0.448844739044126 | 105.438951123857 | 261.411872746213 | Nicaragua |
| 2006 | 303.05488307333 | 0.453950499137161 | 105.401898419557 | 197.652984653773 | Nicaragua |
| 2007 | 276.86530371645 | 0.459042797856105 | 92.8474207270371 | 184.017882989413 | Nicaragua |
| 2008 | 254.20322666235 | 0.463837699218163 | 93.7382330824917 | 160.464993579858 | Nicaragua |
| 2009 | 237.5496078029 | 0.467961838989771 | 85.6044181433949 | 151.945189659505 | Nicaragua |
| 2010 | 220.19355208133 | 0.472171999845355 | 82.6791750751056 | 137.514377006224 | Nicaragua |
| 2011 | 203.80641148266 | 0.4765934659805 | 79.165243019728 | 124.641168462932 | Nicaragua |
| 2012 | 192.90764605002 | 0.481296900452524 | 77.6404240332058 | 115.267222016814 | Nicaragua |
| 2013 | 186.54166591332 | 0.486105470178736 | 74.8966346143135 | 111.645031299006 | Nicaragua |
| 2014 | 179.09957235342 | 0.491207346240634 | 75.4247976486784 | 103.674774704742 | Nicaragua |
| 2015 | 172.33949064966 | 0.496391662501118 | 46.6357451352669 | 125.703745514393 | Nicaragua |
| 2016 | 161.8229833512 | 0.501853344913168 | 16.6139998377977 | 145.208983513402 | Nicaragua |
| 2017 | 156.62908736137 | 0.507482021944309 | 15.918432501013 | 140.710654860357 | Nicaragua |
| 2018 | 151.229254736 | 0.512338227599941 | 15.6365330541729 | 135.592721681827 | Nicaragua |
| 2019 | 145.45103047874 | 0.516597045511253 | 15.288773115117 | 130.162257363623 | Nicaragua |
| 2020 | 135.23973442958 | 0.520292670400273 | 15.1207149285698 | 120.11901950101 | Nicaragua |
| 2021 | 127.49221948832 | 0.523958472235925 | 15.1631310218268 | 112.329088466493 | Nicaragua |
| 1990 | 591.84808077149 | 0.546048123421677 | 14.4277636671267 | 577.420317104363 | Panama |
| 1991 | 558.98403921642 | 0.550484218189226 | 13.9968980756734 | 544.987141140747 | Panama |
| 1992 | 494.78583388756 | 0.55497275492945 | 13.070078466173 | 481.715755421387 | Panama |
| 1993 | 386.42932284155 | 0.55916866562951 | 12.2278706765844 | 374.201452164966 | Panama |
| 1994 | 348.99338235752 | 0.561714534049174 | 11.2262365663748 | 337.767145791145 | Panama |
| 1995 | 342.43199637328 | 0.56335302312626 | 11.4170218907465 | 331.014974482534 | Panama |
| 1996 | 337.12217305771 | 0.565310070207258 | 10.3433815101214 | 326.778791547589 | Panama |
| 1997 | 331.96959500418 | 0.569395938819109 | 9.45500292165165 | 322.514592082528 | Panama |
| 1998 | 348.94523316768 | 0.574697317744379 | 8.86080161528928 | 340.084431552391 | Panama |
| 1999 | 306.44065072907 | 0.580432842358969 | 8.79075247239 | 297.64989825668 | Panama |
| 2000 | 297.88045280686 | 0.587378392850747 | 8.9279650016242 | 288.952487805236 | Panama |
| 2001 | 296.67835607863 | 0.594535700601609 | 8.76773747729725 | 287.910618601333 | Panama |
| 2002 | 278.68510002966 | 0.600138677301078 | 8.77093191988736 | 269.914168109773 | Panama |
| 2003 | 292.90052880243 | 0.604043364755692 | 8.86109746261482 | 284.039431339815 | Panama |
| 2004 | 298.97905754834 | 0.606877030015399 | 8.61199902256959 | 290.36705852577 | Panama |
| 2005 | 307.08086146712 | 0.609494124501125 | 8.88336280212422 | 298.197498664996 | Panama |
| 2006 | 265.16791076555 | 0.612249712793622 | 8.86374476188963 | 256.30416600366 | Panama |
| 2007 | 281.47301081909 | 0.615285417478616 | 8.78229792966207 | 272.690712889428 | Panama |
| 2008 | 248.10006857501 | 0.61895890692398 | 8.88729008208452 | 239.212778492925 | Panama |
| 2009 | 280.42046261374 | 0.622402805678368 | 9.04561377260987 | 271.37484884113 | Panama |
| 2010 | 247.35400985279 | 0.625116323303972 | 8.81217670225941 | 238.541833150531 | Panama |
| 2011 | 221.49977172682 | 0.628633942402595 | 8.47247711454508 | 213.027294612275 | Panama |
| 2012 | 214.23648119772 | 0.634263435389173 | 7.20341534529551 | 207.033065852424 | Panama |
| 2013 | 211.6348672992 | 0.641324352588811 | 6.24403252049879 | 205.390834778701 | Panama |
| 2014 | 198.66859944256 | 0.649108173564656 | 5.04759159688843 | 193.621007845672 | Panama |
| 2015 | 172.20810270705 | 0.657962046843146 | 4.82302984636426 | 167.385072860686 | Panama |
| 2016 | 198.22181965298 | 0.667276758021587 | 3.85633382250085 | 194.365485830479 | Panama |
| 2017 | 211.68402750593 | 0.676783503431544 | 3.59237276788897 | 208.091654738041 | Panama |
| 2018 | 198.00840923679 | 0.686719924606419 | 3.35734579699139 | 194.651063439799 | Panama |
| 2019 | 188.89727245855 | 0.696670680246603 | 3.23954667290817 | 185.657725785642 | Panama |
| 2020 | 168.66069731982 | 0.703788489528527 | 2.92013787608901 | 165.740559443731 | Panama |
| 2021 | 160.13001874076 | 0.708864827518009 | 2.79377192195368 | 157.336246818806 | Panama |
| 1990 | 707.0934258042 | 0.516891218383329 | 15.3536086961428 | 691.739817108057 | Venezuela (Bolivarian Republic of) |
| 1991 | 537.90609534317 | 0.522272295981046 | 15.1510664616215 | 522.755028881548 | Venezuela (Bolivarian Republic of) |
| 1992 | 515.59040388225 | 0.529517990878198 | 15.1136346792624 | 500.476769202988 | Venezuela (Bolivarian Republic of) |
| 1993 | 519.68368879124 | 0.536279978196324 | 15.0906534376281 | 504.593035353612 | Venezuela (Bolivarian Republic of) |
| 1994 | 614.15735637018 | 0.541866057904128 | 15.0621050259972 | 599.095251344183 | Venezuela (Bolivarian Republic of) |
| 1995 | 585.18219322138 | 0.546348233569071 | 14.488602352594 | 570.693590868786 | Venezuela (Bolivarian Republic of) |
| 1996 | 527.92091747139 | 0.548649226233627 | 14.4479150043342 | 513.473002467056 | Venezuela (Bolivarian Republic of) |
| 1997 | 443.67751502707 | 0.552364437231236 | 13.8414713541498 | 429.83604367292 | Venezuela (Bolivarian Republic of) |
| 1998 | 401.85725460015 | 0.557243117253564 | 12.8867662296378 | 388.970488370512 | Venezuela (Bolivarian Republic of) |
| 1999 | 343.69521173985 | 0.560277999100226 | 11.8349454379413 | 331.860266301909 | Venezuela (Bolivarian Republic of) |
| 2000 | 302.56701695463 | 0.56350626012771 | 11.0680067594386 | 291.499010195191 | Venezuela (Bolivarian Republic of) |
| 2001 | 279.30176805802 | 0.565458381141816 | 10.3494258063294 | 268.952342251691 | Venezuela (Bolivarian Republic of) |
| 2002 | 281.72622910534 | 0.562604461024786 | 11.3164324922957 | 270.409796613044 | Venezuela (Bolivarian Republic of) |
| 2003 | 341.7753426363 | 0.555212224179424 | 13.1175932047631 | 328.657749431537 | Venezuela (Bolivarian Republic of) |
| 2004 | 262.15617975573 | 0.55097225564581 | 13.7546021936719 | 248.401577562058 | Venezuela (Bolivarian Republic of) |
| 2005 | 213.47328354936 | 0.553941679272419 | 12.9678487948831 | 200.505434754477 | Venezuela (Bolivarian Republic of) |
| 2006 | 170.88773620871 | 0.563334189710813 | 11.4252697998279 | 159.462466408882 | Venezuela (Bolivarian Republic of) |
| 2007 | 142.12075251914 | 0.575367174436532 | 8.88660055426381 | 133.234151964876 | Venezuela (Bolivarian Republic of) |
| 2008 | 123.54169358353 | 0.586184886513804 | 8.78703230757649 | 114.754661275954 | Venezuela (Bolivarian Republic of) |
| 2009 | 120.39985156674 | 0.593288421972038 | 9.04331522026444 | 111.356536346476 | Venezuela (Bolivarian Republic of) |
| 2010 | 126.14486062458 | 0.599127922953431 | 8.76585565970952 | 117.37900496487 | Venezuela (Bolivarian Republic of) |
| 2011 | 116.53428377619 | 0.604823483689087 | 8.99403862261515 | 107.540245153575 | Venezuela (Bolivarian Republic of) |
| 2012 | 107.08393645788 | 0.610187465323048 | 8.83560391235011 | 98.2483325455299 | Venezuela (Bolivarian Republic of) |
| 2013 | 101.65174993694 | 0.614441024133659 | 8.75415097489988 | 92.8975989620401 | Venezuela (Bolivarian Republic of) |
| 2014 | 110.18072749374 | 0.614686806192499 | 8.88859273585338 | 101.292134757887 | Venezuela (Bolivarian Republic of) |
| 2015 | 117.59278703639 | 0.610675356525105 | 9.02893889264438 | 108.563848143746 | Venezuela (Bolivarian Republic of) |
| 2016 | 138.85930368142 | 0.606993212893547 | 8.6643944427571 | 130.194909238663 | Venezuela (Bolivarian Republic of) |
| 2017 | 150.49580566461 | 0.605160130674623 | 8.86561085578394 | 141.630194808826 | Venezuela (Bolivarian Republic of) |
| 2018 | 142.36199650232 | 0.604068247287088 | 8.92622558828273 | 133.435770914037 | Venezuela (Bolivarian Republic of) |
| 2019 | 139.03088551906 | 0.602313886713059 | 9.01940489202634 | 130.011480627034 | Venezuela (Bolivarian Republic of) |
| 2020 | 130.38694067814 | 0.60003478118243 | 8.86182690661734 | 121.525113771523 | Venezuela (Bolivarian Republic of) |
| 2021 | 120.12149877634 | 0.596513058857335 | 8.96621661773343 | 111.155282158607 | Venezuela (Bolivarian Republic of) |
| 1990 | 2127.14127808183 | 0.500070509167008 | 16.7331392339162 | 2110.40813884791 | Brazil |
| 1991 | 1955.68989083082 | 0.50478479847852 | 16.2241786858068 | 1939.46571214501 | Brazil |
| 1992 | 1775.79585684143 | 0.508539943299266 | 15.9235808105115 | 1759.87227603092 | Brazil |
| 1993 | 1644.87611306009 | 0.512081793142278 | 15.7057977018207 | 1629.17031535827 | Brazil |
| 1994 | 1469.70040616317 | 0.515760842475151 | 15.268586771858 | 1454.43181939131 | Brazil |
| 1995 | 1298.76076699253 | 0.519685962646796 | 15.2863308632061 | 1283.47443612932 | Brazil |
| 1996 | 1133.90212453149 | 0.523863329360558 | 15.1817796243669 | 1118.72034490712 | Brazil |
| 1997 | 992.86055094043 | 0.528177888704233 | 15.0525296737528 | 977.808021266677 | Brazil |
| 1998 | 920.84952513818 | 0.532481169179124 | 15.128131460094 | 905.721393678086 | Brazil |
| 1999 | 801.7841154843 | 0.536972034740505 | 15.0420705490263 | 786.742044935274 | Brazil |
| 2000 | 683.78552127257 | 0.542051497254562 | 15.0476732666574 | 668.737848005913 | Brazil |
| 2001 | 598.57264844104 | 0.547304429543334 | 14.4806088590008 | 584.092039582039 | Brazil |
| 2002 | 532.97687665666 | 0.552776034620744 | 13.9259074105082 | 519.050969246152 | Brazil |
| 2003 | 465.12535119603 | 0.55814571028409 | 11.7863744877213 | 453.338976708309 | Brazil |
| 2004 | 409.57024480499 | 0.563772906923854 | 10.9328279050258 | 398.637416899964 | Brazil |
| 2005 | 380.44464725964 | 0.569458117692368 | 9.54761128705277 | 370.897035972587 | Brazil |
| 2006 | 360.87151356594 | 0.575338774786184 | 8.815395063025 | 352.056118502915 | Brazil |
| 2007 | 336.96412863985 | 0.581588755483488 | 8.88783541511219 | 328.076293224738 | Brazil |
| 2008 | 319.96036160961 | 0.588077817972719 | 8.85136693463356 | 311.108994674976 | Brazil |
| 2009 | 293.6345844067 | 0.594073296681944 | 8.65395738596132 | 284.980627020739 | Brazil |
| 2010 | 254.01272843422 | 0.600445846154568 | 8.80911970180384 | 245.203608732416 | Brazil |
| 2011 | 206.52325770203 | 0.606777290886098 | 9.01059169774187 | 197.512666004288 | Brazil |
| 2012 | 188.62815191982 | 0.612619336859312 | 8.71967292979695 | 179.908478990023 | Brazil |
| 2013 | 173.50089316384 | 0.61821823662824 | 8.65279129279086 | 164.848101871049 | Brazil |
| 2014 | 146.59067956515 | 0.623433813964528 | 8.97043804872325 | 137.620241516427 | Brazil |
| 2015 | 129.35447308692 | 0.628149803496659 | 8.142169002716 | 121.212304084204 | Brazil |
| 2016 | 126.85329772071 | 0.632235114321365 | 8.20209309316247 | 118.651204627548 | Brazil |
| 2017 | 120.87124545408 | 0.636422187733083 | 6.70611097784571 | 114.165134476234 | Brazil |
| 2018 | 113.57635565547 | 0.640802974287966 | 6.06504899901518 | 107.511306656455 | Brazil |
| 2019 | 101.52050235572 | 0.645298005000543 | 5.69199982668773 | 95.8285025290323 | Brazil |
| 2020 | 85.89514653144 | 0.649201568351328 | 4.83689692530072 | 81.0582496061393 | Brazil |
| 2021 | 60.82820921445 | 0.65304388724724 | 5.20424993638736 | 55.6239592780626 | Brazil |
| 1990 | 3139.82809980571 | 0.469527785365773 | 82.3345592092739 | 3057.49354059644 | Paraguay |
| 1991 | 3024.37555385903 | 0.474352286489022 | 79.7140065270827 | 2944.66154733195 | Paraguay |
| 1992 | 3157.15835774975 | 0.479314168681291 | 77.5240420868979 | 3079.63431566285 | Paraguay |
| 1993 | 2937.12302646583 | 0.484614200390594 | 77.0721013837698 | 2860.05092508206 | Paraguay |
| 1994 | 2727.81523073934 | 0.490226934517601 | 75.4520920477666 | 2652.36313869157 | Paraguay |
| 1995 | 2597.79992927324 | 0.496336876135338 | 47.0153633851473 | 2550.78456588809 | Paraguay |
| 1996 | 2369.08306393021 | 0.502322804124491 | 16.7657601269299 | 2352.31730380328 | Paraguay |
| 1997 | 2099.10157614033 | 0.508286947996083 | 15.9385493648551 | 2083.16302677547 | Paraguay |
| 1998 | 1881.4559072957 | 0.513819895911737 | 15.686864333271 | 1865.76904296243 | Paraguay |
| 1999 | 1707.42615549952 | 0.518720583214312 | 15.3508506482054 | 1692.07530485131 | Paraguay |
| 2000 | 1549.14993902922 | 0.52300779310211 | 15.1120045456631 | 1534.03793448356 | Paraguay |
| 2001 | 1465.40014580607 | 0.526916763005844 | 15.1000962625027 | 1450.30004954357 | Paraguay |
| 2002 | 1405.725420048 | 0.531177357815443 | 15.0574293094687 | 1390.66799073853 | Paraguay |
| 2003 | 1297.70388989028 | 0.53536012554352 | 15.0729080934847 | 1282.6309817968 | Paraguay |
| 2004 | 1185.20171727431 | 0.539556655420765 | 15.0779369489263 | 1170.12378032538 | Paraguay |
| 2005 | 1087.13842593263 | 0.543622697051923 | 15.0333882037009 | 1072.10503772893 | Paraguay |
| 2006 | 982.59979625495 | 0.547943304899878 | 14.5682999678906 | 968.031496287059 | Paraguay |
| 2007 | 853.70946424972 | 0.552758676429378 | 13.8211761437443 | 839.888288105976 | Paraguay |
| 2008 | 755.775470756 | 0.55801690264969 | 11.9826186280375 | 743.792852127963 | Paraguay |
| 2009 | 689.30551637308 | 0.562900058514746 | 11.7581706057222 | 677.547345767358 | Paraguay |
| 2010 | 568.2042171708 | 0.568846562482396 | 9.50475658852844 | 558.699460582272 | Paraguay |
| 2011 | 521.6870051569 | 0.574954463115124 | 8.92644673295531 | 512.760558423945 | Paraguay |
| 2012 | 477.04728498579 | 0.580650185867766 | 8.83740310755337 | 468.209881878237 | Paraguay |
| 2013 | 434.44462029343 | 0.587247042264483 | 8.82092828122847 | 425.623692012202 | Paraguay |
| 2014 | 389.14098879101 | 0.593953153052431 | 8.85599172780854 | 380.284997063201 | Paraguay |
| 2015 | 335.07825824293 | 0.600484125243426 | 8.98620475732608 | 326.092053485604 | Paraguay |
| 2016 | 318.82223383429 | 0.607018144917351 | 9.00208928563 | 309.82014454866 | Paraguay |
| 2017 | 321.41666092397 | 0.613581590966399 | 8.96778899931827 | 312.448871924652 | Paraguay |
| 2018 | 312.9568040468 | 0.620032361543249 | 9.0198402588626 | 303.936963787937 | Paraguay |
| 2019 | 297.18479830897 | 0.626070139299015 | 8.85441038258385 | 288.330387926386 | Paraguay |
| 2020 | 282.35563619226 | 0.631057688560962 | 8.19801690186178 | 274.157619290398 | Paraguay |
| 2021 | 280.24861853896 | 0.635718098852038 | 7.08779487264946 | 273.160823666311 | Paraguay |
| 1990 | 232.92786438566 | 0.460486905849358 | 91.6946992834796 | 141.23316510218 | Algeria |
| 1991 | 210.08136893513 | 0.468319196672477 | 85.0264061307589 | 125.054962804371 | Algeria |
| 1992 | 192.23474267639 | 0.475950937138063 | 77.5330854921942 | 114.701657184196 | Algeria |
| 1993 | 177.59330087814 | 0.483255591216591 | 77.5587108907386 | 100.034589987401 | Algeria |
| 1994 | 164.76921286419 | 0.490450660309052 | 75.3533521594709 | 89.4158607047191 | Algeria |
| 1995 | 151.40090828767 | 0.497957543180561 | 17.9637727136965 | 133.437135573973 | Algeria |
| 1996 | 139.83948196383 | 0.505990967161627 | 16.2175768611983 | 123.621905102632 | Algeria |
| 1997 | 132.92383327682 | 0.514056565839869 | 15.6085124524949 | 117.315320824325 | Algeria |
| 1998 | 125.31390162238 | 0.522382418796352 | 15.1387952434273 | 110.175106378953 | Algeria |
| 1999 | 119.01431476252 | 0.530785582833015 | 15.0403419526812 | 103.973972809839 | Algeria |
| 2000 | 113.2001796579 | 0.539481507570335 | 15.0627727967575 | 98.1374068611425 | Algeria |
| 2001 | 108.16694405921 | 0.54752927722376 | 14.4718657553575 | 93.6950783038525 | Algeria |
| 2002 | 103.26637100117 | 0.555172221658197 | 12.8793476281124 | 90.3870233730576 | Algeria |
| 2003 | 99.15887841006 | 0.562536956171446 | 11.3597324262911 | 87.7991459837689 | Algeria |
| 2004 | 96.24325769264 | 0.569352851934701 | 9.62228360771747 | 86.6209740849225 | Algeria |
| 2005 | 91.86320595894 | 0.576001964539977 | 8.85011876852906 | 83.0130871904109 | Algeria |
| 2006 | 86.93143761185 | 0.582126361864573 | 8.81730152792725 | 78.1141360839227 | Algeria |
| 2007 | 81.47757379105 | 0.587790538325364 | 9.05180149328743 | 72.4257722977626 | Algeria |
| 2008 | 76.40663388418 | 0.593190973247903 | 8.98265511075347 | 67.4239787734265 | Algeria |
| 2009 | 70.98507559254 | 0.597870383895018 | 8.70177301728451 | 62.2833025752555 | Algeria |
| 2010 | 66.45792416667 | 0.602823534656761 | 8.67542234540875 | 57.7825018212612 | Algeria |
| 2011 | 62.19950263501 | 0.607825529405856 | 8.91155233526345 | 53.2879502997466 | Algeria |
| 2012 | 58.31307221479 | 0.612703533153522 | 8.89490758447983 | 49.4181646303102 | Algeria |
| 2013 | 55.71058637321 | 0.617422204461461 | 8.98153121771634 | 46.7290551554937 | Algeria |
| 2014 | 53.21420880424 | 0.622087291667118 | 8.92722693017 | 44.28698187407 | Algeria |
| 2015 | 50.34841650021 | 0.626745568394753 | 8.69965739253619 | 41.6487591076738 | Algeria |
| 2016 | 46.94260713545 | 0.631710735505334 | 8.48789374532413 | 38.4547133901259 | Algeria |
| 2017 | 42.74756286362 | 0.636973175875889 | 6.89048794017407 | 35.8570749234459 | Algeria |
| 2018 | 39.81417223949 | 0.642500039273145 | 6.1494191067154 | 33.6647531327746 | Algeria |
| 2019 | 36.87830549392 | 0.648210784879009 | 6.42041340624412 | 30.4578920876759 | Algeria |
| 2020 | 33.76476722349 | 0.65365146606094 | 4.81870203601427 | 28.9460651874757 | Algeria |
| 2021 | 31.97732663976 | 0.659500924324217 | 4.41322133472969 | 27.5641053050303 | Algeria |
| 1990 | 107.90098307203 | 0.584578851787694 | 8.71268246031736 | 99.1883006117126 | Bahrain |
| 1991 | 100.93783984531 | 0.590596896738332 | 8.88742092442522 | 92.0504189208848 | Bahrain |
| 1992 | 93.72390780415 | 0.596040229262693 | 8.85038691787957 | 84.8735208862704 | Bahrain |
| 1993 | 85.98161809209 | 0.602165621752441 | 8.79714413699115 | 77.1844739550989 | Bahrain |
| 1994 | 77.98075228402 | 0.60813372491926 | 8.79224175319763 | 69.1885105308224 | Bahrain |
| 1995 | 69.16145482807 | 0.613850721750031 | 8.90212477128912 | 60.2593300567809 | Bahrain |
| 1996 | 60.14628614115 | 0.619485376148992 | 8.89422216636826 | 51.2520639747817 | Bahrain |
| 1997 | 54.40760114969 | 0.624596061581476 | 8.84206383312763 | 45.5655373165624 | Bahrain |
| 1998 | 51.23433401483 | 0.630738301217212 | 8.16061080935245 | 43.0737232054775 | Bahrain |
| 1999 | 48.62174941747 | 0.638083186958308 | 6.71163280046542 | 41.9101166170046 | Bahrain |
| 2000 | 47.28140228237 | 0.646750697322184 | 6.081040641495 | 41.200361640875 | Bahrain |
| 2001 | 46.94649893269 | 0.657196063695041 | 4.92543110245036 | 42.0210678302396 | Bahrain |
| 2002 | 46.84261542983 | 0.664901201084495 | 4.24984838202453 | 42.5927670478055 | Bahrain |
| 2003 | 47.26698751253 | 0.669564110612245 | 3.92379740900274 | 43.3431901035273 | Bahrain |
| 2004 | 47.44940830406 | 0.674147775423852 | 3.79098288146763 | 43.6584254225924 | Bahrain |
| 2005 | 46.86701271649 | 0.679790598294553 | 3.57153456581806 | 43.2954781506719 | Bahrain |
| 2006 | 45.53236568478 | 0.686144289377392 | 3.32068987625423 | 42.2116758085258 | Bahrain |
| 2007 | 43.98480531804 | 0.693045146522931 | 3.1040937438797 | 40.8807115741603 | Bahrain |
| 2008 | 37.22573993149 | 0.699806387259847 | 3.02342646288839 | 34.2023134686016 | Bahrain |
| 2009 | 35.46061325794 | 0.704885840093356 | 2.91842000808937 | 32.5421932498506 | Bahrain |
| 2010 | 36.15857821976 | 0.707902620468186 | 2.88377053181833 | 33.2748076879417 | Bahrain |
| 2011 | 31.7676476843 | 0.708958738960692 | 2.87048002209311 | 28.8971676622069 | Bahrain |
| 2012 | 30.28637094054 | 0.710362417505028 | 2.80549204762373 | 27.4808788929163 | Bahrain |
| 2013 | 28.83526642754 | 0.713343155899609 | 2.69013164309795 | 26.1451347844421 | Bahrain |
| 2014 | 27.62196789386 | 0.716768059555516 | 2.60632554318725 | 25.0156423506727 | Bahrain |
| 2015 | 26.1879748017 | 0.719926604884411 | 2.63223587125048 | 23.5557389304495 | Bahrain |
| 2016 | 24.32144228687 | 0.723611296528675 | 2.45459956794684 | 21.8668427189232 | Bahrain |
| 2017 | 22.80250863266 | 0.729323102530322 | 2.17747673699625 | 20.6250318956637 | Bahrain |
| 2018 | 21.22865993242 | 0.736192583172625 | 2.01805192960717 | 19.2106080028128 | Bahrain |
| 2019 | 19.89154040114 | 0.742847804247627 | 1.7474170998281 | 18.1441233013119 | Bahrain |
| 2020 | 17.73211701759 | 0.748103083095047 | 1.85006404347165 | 15.8820529741184 | Bahrain |
| 2021 | 16.95910755652 | 0.753043204117567 | 1.79999695320437 | 15.1591106033156 | Bahrain |
| 1990 | 1790.96982547624 | 0.417182741758852 | 197.871107379046 | 1593.09871809719 | Egypt |
| 1991 | 1627.04554950937 | 0.426909452028558 | 178.357093012011 | 1448.68845649736 | Egypt |
| 1992 | 1470.18106481371 | 0.437744555478775 | 127.947180075145 | 1342.23388473857 | Egypt |
| 1993 | 1301.12427648017 | 0.448490053084385 | 106.333436992331 | 1194.79083948784 | Egypt |
| 1994 | 1150.80185307488 | 0.458727004203869 | 94.5995712200496 | 1056.20228185483 | Egypt |
| 1995 | 1033.59686325516 | 0.468035368434364 | 85.461937411911 | 948.134925843249 | Egypt |
| 1996 | 880.83117189426 | 0.476116627128094 | 77.862266419097 | 802.968905475163 | Egypt |
| 1997 | 782.3365101974 | 0.482970886044098 | 77.126741154916 | 705.209769042484 | Egypt |
| 1998 | 703.34461902284 | 0.489184070606589 | 74.7701128674359 | 628.574506155404 | Egypt |
| 1999 | 645.73760981389 | 0.495502619174384 | 70.3742068264153 | 575.363402987475 | Egypt |
| 2000 | 590.04459421344 | 0.502169886288414 | 16.9524410326426 | 573.092153180797 | Egypt |
| 2001 | 527.92433904318 | 0.508716824226239 | 15.9795553030895 | 511.94478374009 | Egypt |
| 2002 | 466.27555549048 | 0.515038129463872 | 15.6830317184402 | 450.59252377204 | Egypt |
| 2003 | 416.9190656482 | 0.520623395469455 | 15.1800397077308 | 401.739025940469 | Egypt |
| 2004 | 375.63232089406 | 0.525181677996097 | 15.1367000537025 | 360.495620840358 | Egypt |
| 2005 | 339.49142255122 | 0.528665483094012 | 15.0646291710314 | 324.426793380189 | Egypt |
| 2006 | 316.49673215662 | 0.530395807271736 | 15.0904464683551 | 301.406285688265 | Egypt |
| 2007 | 303.33281560362 | 0.529457593890747 | 15.0866270411368 | 288.246188562483 | Egypt |
| 2008 | 289.07558129767 | 0.5247438325858 | 15.1071411282588 | 273.968440169411 | Egypt |
| 2009 | 281.05257466059 | 0.51625583066946 | 15.3637442143656 | 265.688830446224 | Egypt |
| 2010 | 265.82398151855 | 0.507715477095215 | 15.8555974691554 | 249.968384049395 | Egypt |
| 2011 | 246.47336736913 | 0.504594044306814 | 16.1750553198185 | 230.298312049311 | Egypt |
| 2012 | 229.84469461641 | 0.509157068362998 | 16.0780571580149 | 213.766637458395 | Egypt |
| 2013 | 222.38658052272 | 0.51933446386018 | 15.3107759557455 | 207.075804566975 | Egypt |
| 2014 | 200.69690543101 | 0.531749809455032 | 15.0975783970046 | 185.599327034005 | Egypt |
| 2015 | 188.3215745887 | 0.544370148675277 | 15.0181826466306 | 173.303391942069 | Egypt |
| 2016 | 175.51467818453 | 0.556012469227609 | 13.1145830658458 | 162.400095118684 | Egypt |
| 2017 | 164.51368316439 | 0.566914631824628 | 9.8280454678025 | 154.685637696587 | Egypt |
| 2018 | 154.92575463287 | 0.577442786164725 | 8.94813386470232 | 145.977620768168 | Egypt |
| 2019 | 147.62723811876 | 0.587736876524439 | 8.81614016040076 | 138.811097958359 | Egypt |
| 2020 | 128.62144260527 | 0.597363341129599 | 8.93252571037241 | 119.688916894898 | Egypt |
| 2021 | 122.23865721509 | 0.6067870937016 | 8.79825328100618 | 113.440403934084 | Egypt |
| 1990 | 314.25609613045 | 0.453799943957447 | 105.824530886878 | 208.431565243572 | Iran (Islamic Republic of) |
| 1991 | 276.51509655747 | 0.468982857673188 | 82.5732440749753 | 193.941852482495 | Iran (Islamic Republic of) |
| 1992 | 246.82443302013 | 0.480626696650433 | 76.936947578754 | 169.887485441376 | Iran (Islamic Republic of) |
| 1993 | 227.02175486488 | 0.492277025057908 | 73.3260028071073 | 153.695752057773 | Iran (Islamic Republic of) |
| 1994 | 212.33574794047 | 0.505072857378303 | 16.0760933991274 | 196.259654541343 | Iran (Islamic Republic of) |
| 1995 | 203.3812597502 | 0.517531493818527 | 15.3193670663789 | 188.061892683821 | Iran (Islamic Republic of) |
| 1996 | 183.83621629793 | 0.528600719906526 | 15.1163495808582 | 168.719866717072 | Iran (Islamic Republic of) |
| 1997 | 176.12165050273 | 0.537727779314693 | 15.0549474632194 | 161.066703039511 | Iran (Islamic Republic of) |
| 1998 | 166.7345042105 | 0.546366017294819 | 14.481184511958 | 152.253319698542 | Iran (Islamic Republic of) |
| 1999 | 157.55192255434 | 0.55562433168066 | 12.8986063670816 | 144.653316187258 | Iran (Islamic Republic of) |
| 2000 | 149.06669138433 | 0.565474561419707 | 10.2734857284886 | 138.793205655841 | Iran (Islamic Republic of) |
| 2001 | 126.54271097978 | 0.574618208021481 | 8.89234935703623 | 117.650361622744 | Iran (Islamic Republic of) |
| 2002 | 115.38861038815 | 0.584097268490781 | 8.76883734549169 | 106.619773042658 | Iran (Islamic Republic of) |
| 2003 | 105.35386281479 | 0.593963012213832 | 8.98563374479052 | 96.3682290699995 | Iran (Islamic Republic of) |
| 2004 | 97.26324151745 | 0.603717449978768 | 8.66613844207851 | 88.5971030753715 | Iran (Islamic Republic of) |
| 2005 | 90.7264348232 | 0.613291055585393 | 8.90812663537882 | 81.8183081878212 | Iran (Islamic Republic of) |
| 2006 | 84.22873145488 | 0.621268095172314 | 9.00338821079231 | 75.2253432440877 | Iran (Islamic Republic of) |
| 2007 | 78.25875519519 | 0.627749441937933 | 8.3685221747097 | 69.8902330204803 | Iran (Islamic Republic of) |
| 2008 | 72.06181867036 | 0.632862230135051 | 7.01763631260843 | 65.0441823577516 | Iran (Islamic Republic of) |
| 2009 | 65.13597922814 | 0.637398379541013 | 6.7595625427221 | 58.3764166854179 | Iran (Islamic Republic of) |
| 2010 | 58.62898526148 | 0.642559170275463 | 5.76877240740828 | 52.8602128540717 | Iran (Islamic Republic of) |
| 2011 | 54.04016491663 | 0.647899089013155 | 5.93171751240914 | 48.1084474042209 | Iran (Islamic Republic of) |
| 2012 | 48.818507921 | 0.651500586996546 | 5.3910093478545 | 43.4274985731455 | Iran (Islamic Republic of) |
| 2013 | 45.68836417684 | 0.654475289198172 | 4.76221328683424 | 40.9261508900058 | Iran (Islamic Republic of) |
| 2014 | 40.91179390239 | 0.657961510912541 | 4.7050363177587 | 36.2067575846313 | Iran (Islamic Republic of) |
| 2015 | 35.96808785977 | 0.662057986508516 | 4.3631256465931 | 31.6049622131769 | Iran (Islamic Republic of) |
| 2016 | 29.71329158008 | 0.667652445135305 | 3.91933226216284 | 25.7939593179172 | Iran (Islamic Republic of) |
| 2017 | 25.80089604773 | 0.674343462089889 | 3.68582829167786 | 22.1150677560521 | Iran (Islamic Republic of) |
| 2018 | 23.07802977642 | 0.680909620594576 | 3.56451793389365 | 19.5135118425263 | Iran (Islamic Republic of) |
| 2019 | 21.36118245075 | 0.686741661948379 | 3.34302056451864 | 18.0181618862314 | Iran (Islamic Republic of) |
| 2020 | 14.40903591347 | 0.691918763371909 | 3.08994647509465 | 11.3190894383753 | Iran (Islamic Republic of) |
| 2021 | 9.95947131386 | 0.697207397661899 | 3.02745192593687 | 6.93201938792313 | Iran (Islamic Republic of) |
| 1990 | 555.32228538572 | 0.412044172813982 | 217.348679389085 | 337.973605996635 | Iraq |
| 1991 | 572.54868110128 | 0.415814115814778 | 199.880025970082 | 372.668655131198 | Iraq |
| 1992 | 565.73222308031 | 0.42035359441045 | 194.876216652235 | 370.856006428075 | Iraq |
| 1993 | 542.75891352488 | 0.42483493253075 | 176.220908004386 | 366.538005520494 | Iraq |
| 1994 | 518.09372150118 | 0.428751730675342 | 166.003296947532 | 352.090424553648 | Iraq |
| 1995 | 503.11391597589 | 0.432075545408255 | 134.379090818194 | 368.734825157696 | Iraq |
| 1996 | 488.2936463826 | 0.436818478749736 | 126.317687377663 | 361.975959004937 | Iraq |
| 1997 | 464.05132956587 | 0.44310402693832 | 110.88948060985 | 353.16184895602 | Iraq |
| 1998 | 433.7911034561 | 0.452384092352145 | 109.085784636603 | 324.705318819497 | Iraq |
| 1999 | 406.39892385783 | 0.462736441391784 | 89.4630630143757 | 316.935860843454 | Iraq |
| 2000 | 372.8707713357 | 0.472376399238703 | 82.3198262964766 | 290.550945039223 | Iraq |
| 2001 | 344.41483878133 | 0.481458319619666 | 76.4919538116175 | 267.922884969713 | Iraq |
| 2002 | 324.58026634327 | 0.488664736761323 | 75.4943161761022 | 249.085950167168 | Iraq |
| 2003 | 317.05948088767 | 0.492174444727086 | 72.8670162421292 | 244.192464645541 | Iraq |
| 2004 | 295.56535623768 | 0.498933881288036 | 18.1381517088377 | 277.427204528842 | Iraq |
| 2005 | 276.41931801238 | 0.504719430828624 | 16.1630300293659 | 260.256287983014 | Iraq |
| 2006 | 257.27464810858 | 0.510918049053858 | 15.6970603784708 | 241.577587730109 | Iraq |
| 2007 | 246.11994637993 | 0.517698972378274 | 15.3286170446992 | 230.791329335231 | Iraq |
| 2008 | 226.84938081966 | 0.525242907568095 | 15.0577373502669 | 211.791643469393 | Iraq |
| 2009 | 204.70134528263 | 0.533200913619797 | 15.0807353106334 | 189.620609971997 | Iraq |
| 2010 | 187.95480922012 | 0.542343278401218 | 15.0406496257354 | 172.914159594385 | Iraq |
| 2011 | 170.55707247636 | 0.553048555118566 | 13.9629016203827 | 156.594170855977 | Iraq |
| 2012 | 149.90949473996 | 0.564745467448645 | 11.0481442093451 | 138.861350530615 | Iraq |
| 2013 | 135.8905270592 | 0.576968997890727 | 8.99464350584661 | 126.895883553353 | Iraq |
| 2014 | 132.86677995326 | 0.588317651574763 | 8.88180952459747 | 123.984970428663 | Iraq |
| 2015 | 121.59476524011 | 0.599494007105483 | 8.82803602532137 | 112.766729214789 | Iraq |
| 2016 | 114.63391944315 | 0.611508645751767 | 8.85688146160479 | 105.777037981545 | Iraq |
| 2017 | 109.00694002047 | 0.622674531557115 | 8.91864399886552 | 100.088296021604 | Iraq |
| 2018 | 102.40702397277 | 0.633232108100178 | 6.983523153569 | 95.423500819201 | Iraq |
| 2019 | 110.14073593738 | 0.64363531790137 | 6.03086800469137 | 104.109867932689 | Iraq |
| 2020 | 92.5916899841 | 0.653300379638783 | 5.25434778141587 | 87.3373422026841 | Iraq |
| 2021 | 88.87947544098 | 0.662626230762701 | 4.28639922415857 | 84.5930762168214 | Iraq |
| 1990 | 130.03478755368 | 0.53914746775111 | 15.1441342724087 | 114.890653281271 | Jordan |
| 1991 | 125.00083318318 | 0.54281941081865 | 15.0149651374566 | 109.985868045723 | Jordan |
| 1992 | 119.06648226219 | 0.547458846457137 | 14.4026519413875 | 104.663830320802 | Jordan |
| 1993 | 113.22272845301 | 0.552182350404081 | 14.0120539940601 | 99.2106744589499 | Jordan |
| 1994 | 108.00160787051 | 0.557254334819626 | 12.6481525037059 | 95.3534553668041 | Jordan |
| 1995 | 103.07587026296 | 0.562571536302321 | 11.2919087794401 | 91.7839614835199 | Jordan |
| 1996 | 98.79719897439 | 0.567529655438914 | 9.74100251804636 | 89.0561964563436 | Jordan |
| 1997 | 94.2630737693 | 0.572581508488338 | 8.82941044905158 | 85.4336633202484 | Jordan |
| 1998 | 91.78340143816 | 0.577643570372855 | 8.87014502249699 | 82.913256415663 | Jordan |
| 1999 | 88.38334941642 | 0.582759198560708 | 8.81750657622653 | 79.5658428401935 | Jordan |
| 2000 | 84.88568721281 | 0.587909393807207 | 8.97442370482232 | 75.9112635079877 | Jordan |
| 2001 | 81.31340987854 | 0.593182802809495 | 8.73463942757174 | 72.5787704509683 | Jordan |
| 2002 | 77.40766584275 | 0.59900038468688 | 9.0249314359595 | 68.3827344067905 | Jordan |
| 2003 | 72.7558148604 | 0.60494595024538 | 8.82247325100881 | 63.9333416093912 | Jordan |
| 2004 | 69.25056600693 | 0.611934403689721 | 8.96951582874219 | 60.2810501781878 | Jordan |
| 2005 | 65.86144869666 | 0.619909227769316 | 8.76249191555 | 57.09895678111 | Jordan |
| 2006 | 61.25856245103 | 0.627803812947762 | 8.1055821176778 | 53.1529803333522 | Jordan |
| 2007 | 56.32916993244 | 0.635670276218909 | 7.17635436112312 | 49.1528155713169 | Jordan |
| 2008 | 52.82015142106 | 0.643892693421901 | 6.43621856023213 | 46.3839328608279 | Jordan |
| 2009 | 49.89278535076 | 0.652196319480578 | 4.94932395340046 | 44.9434613973595 | Jordan |
| 2010 | 46.97032826673 | 0.659949494519026 | 4.38556688442743 | 42.5847613823026 | Jordan |
| 2011 | 44.97978650647 | 0.667396679645637 | 3.85503254461 | 41.12475396186 | Jordan |
| 2012 | 43.86524245445 | 0.674568924665591 | 3.59793043706346 | 40.2673120173865 | Jordan |
| 2013 | 42.8151273878 | 0.681119962167508 | 3.59257417102294 | 39.2225532167771 | Jordan |
| 2014 | 41.57206030074 | 0.687134039943201 | 3.39607800254545 | 38.1759822981945 | Jordan |
| 2015 | 35.81270280963 | 0.692952031873164 | 3.17740990028966 | 32.6352929093403 | Jordan |
| 2016 | 34.74701473421 | 0.698473302300541 | 3.07054414713848 | 31.6764705870715 | Jordan |
| 2017 | 33.06772015026 | 0.703923826241846 | 2.95071211400948 | 30.1170080362505 | Jordan |
| 2018 | 30.56951097058 | 0.709412879293601 | 2.69483754126677 | 27.8746734293132 | Jordan |
| 2019 | 29.74587603747 | 0.714998945753142 | 2.62126069188541 | 27.1246153455846 | Jordan |
| 2020 | 28.00009546807 | 0.720068239915947 | 2.63266086831762 | 25.3674345997524 | Jordan |
| 2021 | 28.01832463949 | 0.725307226832443 | 2.37688638069868 | 25.6414382587913 | Jordan |
| 1990 | 48.60398491222 | 0.66451790400099 | 4.22385274377344 | 44.3801321684466 | Kuwait |
| 1991 | 29.08444266487 | 0.66880962078386 | 3.82256718536154 | 25.2618754795085 | Kuwait |
| 1992 | 31.04844406825 | 0.670159110400931 | 3.88263867962629 | 27.1658053886237 | Kuwait |
| 1993 | 26.54014566726 | 0.670036252235978 | 3.8460676576613 | 22.6940780095987 | Kuwait |
| 1994 | 32.90300924414 | 0.671393212861768 | 3.78374656286914 | 29.1192626812709 | Kuwait |
| 1995 | 19.58961661159 | 0.677215523332779 | 3.60512015683936 | 15.9844964547506 | Kuwait |
| 1996 | 13.69967869229 | 0.686093303524213 | 3.35063269357491 | 10.3490459987151 | Kuwait |
| 1997 | 13.6950397891 | 0.695789388591065 | 3.11150184472023 | 10.5835379443798 | Kuwait |
| 1998 | 16.85480765833 | 0.704383735664098 | 2.95092143426508 | 13.9038862240649 | Kuwait |
| 1999 | 21.28683187335 | 0.711841328373508 | 2.7350707597295 | 18.5517611136205 | Kuwait |
| 2000 | 24.0303185807 | 0.718357562401374 | 2.54057920761159 | 21.4897393730884 | Kuwait |
| 2001 | 18.96647337874 | 0.722840631295124 | 2.47157693052145 | 16.4948964482186 | Kuwait |
| 2002 | 21.19575604473 | 0.72681697562953 | 2.40280720565688 | 18.7929488390731 | Kuwait |
| 2003 | 22.38164987051 | 0.731778684982861 | 2.27663407708846 | 20.1050157934215 | Kuwait |
| 2004 | 20.94600401104 | 0.738153706974425 | 2.13335981663106 | 18.8126441944089 | Kuwait |
| 2005 | 21.1248473746 | 0.746384913346372 | 1.84573344874 | 19.27911392586 | Kuwait |
| 2006 | 19.42975997353 | 0.754928492574677 | 1.76751498197794 | 17.6622449915521 | Kuwait |
| 2007 | 20.48013357742 | 0.762411483964357 | 1.76224017160418 | 18.7178934058158 | Kuwait |
| 2008 | 21.22250006398 | 0.770002614917808 | 1.74093439512155 | 19.4815656688585 | Kuwait |
| 2009 | 19.02921248618 | 0.777546551267578 | 1.75314585638794 | 17.2760666297921 | Kuwait |
| 2010 | 15.8345643909 | 0.784873929223183 | 1.65110210382265 | 14.1834622870774 | Kuwait |
| 2011 | 15.96580363672 | 0.791812065474587 | 1.56998602390671 | 14.3958176128133 | Kuwait |
| 2012 | 16.21422377062 | 0.798356987724222 | 1.54011945043556 | 14.6741043201844 | Kuwait |
| 2013 | 15.35184642478 | 0.80458383431311 | 1.53509434973068 | 13.8167520750493 | Kuwait |
| 2014 | 15.10167344767 | 0.810743232598608 | 1.51923766755513 | 13.5824357801149 | Kuwait |
| 2015 | 14.09706116882 | 0.816998412782804 | 1.5261018102696 | 12.5709593585504 | Kuwait |
| 2016 | 13.67505253731 | 0.823026527054531 | 1.47978360555125 | 12.1952689317588 | Kuwait |
| 2017 | 11.90815902692 | 0.828886986643824 | 1.50023302930114 | 10.4079259976189 | Kuwait |
| 2018 | 11.39023507501 | 0.834371461321704 | 1.48522171741945 | 9.90501335759055 | Kuwait |
| 2019 | 11.21661552891 | 0.839688306553232 | 1.46363530945652 | 9.75298021945348 | Kuwait |
| 2020 | 10.00165933775 | 0.843459812023503 | 1.47334180993957 | 8.52831752781043 | Kuwait |
| 2021 | 9.85300129232 | 0.846651054742869 | 1.46114633082774 | 8.39185496149226 | Kuwait |
| 1990 | 207.31382054686 | 0.536718968751988 | 15.0694755834128 | 192.244344963447 | Lebanon |
| 1991 | 197.12926257241 | 0.539197491838137 | 15.0583198758407 | 182.070942696569 | Lebanon |
| 1992 | 186.57594943219 | 0.541793515767809 | 15.0251578825346 | 171.550791549655 | Lebanon |
| 1993 | 212.53320089181 | 0.544581306172966 | 15.0401129201311 | 197.493087971679 | Lebanon |
| 1994 | 166.66910224061 | 0.547684033143162 | 14.5170398018586 | 152.152062438751 | Lebanon |
| 1995 | 157.76007806815 | 0.5514717204066 | 13.8652979887332 | 143.894780079417 | Lebanon |
| 1996 | 148.46714018607 | 0.556436158565937 | 13.107851019139 | 135.359289166931 | Lebanon |
| 1997 | 138.44856998353 | 0.562120817980966 | 11.1404518789925 | 127.308118104537 | Lebanon |
| 1998 | 130.67865778041 | 0.569059070131885 | 9.54035295077462 | 121.138304829635 | Lebanon |
| 1999 | 125.29012533146 | 0.576078527772757 | 8.79964550594737 | 116.490479825513 | Lebanon |
| 2000 | 119.02049224794 | 0.582698277151947 | 9.08508312714468 | 109.935409120795 | Lebanon |
| 2001 | 115.50178858948 | 0.589185502386114 | 8.6252516370741 | 106.876536952406 | Lebanon |
| 2002 | 109.668662702 | 0.595978787643548 | 8.7796052093811 | 100.889057492619 | Lebanon |
| 2003 | 104.49558280569 | 0.602967240409336 | 8.82605428866297 | 95.669528517027 | Lebanon |
| 2004 | 99.35521662111 | 0.610845760385912 | 9.03023275551627 | 90.3249838655937 | Lebanon |
| 2005 | 95.6479582041 | 0.619391355094372 | 8.74477553504286 | 86.9031826690571 | Lebanon |
| 2006 | 89.60302537842 | 0.627791217131142 | 8.23160395638844 | 81.3714214220316 | Lebanon |
| 2007 | 85.26175393329 | 0.636938234123424 | 6.61174854789429 | 78.6500053853957 | Lebanon |
| 2008 | 80.79214671344 | 0.646667663001479 | 6.02635906714046 | 74.7657876462995 | Lebanon |
| 2009 | 78.76010840764 | 0.657433186212217 | 4.83276178915293 | 73.9273466184871 | Lebanon |
| 2010 | 75.77468714109 | 0.668804524925046 | 3.85819681334527 | 71.9164903277447 | Lebanon |
| 2011 | 74.30838176434 | 0.680920616624062 | 3.58257032670444 | 70.7258114376356 | Lebanon |
| 2012 | 72.55952646139 | 0.693910983172664 | 3.15385263221103 | 69.405673829179 | Lebanon |
| 2013 | 67.9791999605 | 0.704811001480686 | 2.85414106927781 | 65.1250588912222 | Lebanon |
| 2014 | 62.91533963561 | 0.71242387252594 | 2.58111501395212 | 60.3342246216579 | Lebanon |
| 2015 | 61.45023842311 | 0.7186972923939 | 2.51193628664355 | 58.9383021364665 | Lebanon |
| 2016 | 61.65727669908 | 0.724583346730978 | 2.42931083001097 | 59.227965869069 | Lebanon |
| 2017 | 61.09467128524 | 0.729963293744747 | 2.29023890441716 | 58.8044323808228 | Lebanon |
| 2018 | 59.56133716179 | 0.734746243401704 | 2.07477643916932 | 57.4865607226207 | Lebanon |
| 2019 | 58.32641124293 | 0.738644009352405 | 1.915896248699 | 56.410514994231 | Lebanon |
| 2020 | 53.96280035315 | 0.742009720142934 | 1.909017632433 | 52.053782720717 | Lebanon |
| 2021 | 53.37923316726 | 0.74474635096338 | 1.82756497957659 | 51.5516681876834 | Lebanon |
| 1990 | 394.10263621087 | 0.527998168231366 | 15.1041249804312 | 378.998511230439 | Libya |
| 1991 | 330.50483302569 | 0.541432398663791 | 15.0263308538399 | 315.47850217185 | Libya |
| 1992 | 271.81058257592 | 0.55446822725249 | 13.1410866148808 | 258.669495961039 | Libya |
| 1993 | 233.07154607675 | 0.566798415093121 | 10.0990777908392 | 222.972468285911 | Libya |
| 1994 | 204.38821760501 | 0.578651415542216 | 8.85632255635203 | 195.531895048658 | Libya |
| 1995 | 176.80945331051 | 0.589578634415899 | 8.88043289285618 | 167.929020417654 | Libya |
| 1996 | 141.94403351046 | 0.60035004409905 | 8.72497636063494 | 133.219057149825 | Libya |
| 1997 | 128.20584126214 | 0.610620315262943 | 9.00163529672792 | 119.204205965412 | Libya |
| 1998 | 114.58554365376 | 0.619973983675942 | 8.7865170430764 | 105.799026610684 | Libya |
| 1999 | 103.43821941029 | 0.628600604307387 | 8.23273858467952 | 95.2054808256105 | Libya |
| 2000 | 92.56320344074 | 0.63704793460596 | 6.61975878317597 | 85.943444657564 | Libya |
| 2001 | 85.765061556 | 0.644685131438978 | 5.7601310264815 | 80.0049305295185 | Libya |
| 2002 | 80.54484463007 | 0.651669147460857 | 4.96728473215514 | 75.5775598979149 | Libya |
| 2003 | 75.83771133461 | 0.659345124852632 | 4.46791388925514 | 71.3697974453549 | Libya |
| 2004 | 69.71694886387 | 0.666913926262631 | 3.93153387317786 | 65.7854149906921 | Libya |
| 2005 | 65.19520069428 | 0.675322267622666 | 3.59234512327312 | 61.6028555710069 | Libya |
| 2006 | 61.91409170512 | 0.683701999618589 | 3.38283616668533 | 58.5312555384347 | Libya |
| 2007 | 59.2505206257 | 0.691855134182363 | 3.18009344396226 | 56.0704271817377 | Libya |
| 2008 | 55.39562374864 | 0.698873253326827 | 2.8366149234728 | 52.5590088251672 | Libya |
| 2009 | 50.81734409719 | 0.704998470605237 | 2.84487670373667 | 47.9724673934533 | Libya |
| 2010 | 49.48492119904 | 0.711362496328616 | 2.86547248170736 | 46.6194487173326 | Libya |
| 2011 | 45.44657437405 | 0.711466657224803 | 2.72214057067178 | 42.7244338033782 | Libya |
| 2012 | 43.37733015645 | 0.716700693100353 | 2.6063833014875 | 40.7709468549625 | Libya |
| 2013 | 38.06643060227 | 0.717665249612534 | 2.54153354881463 | 35.5248970534554 | Libya |
| 2014 | 38.84403902745 | 0.716042207086096 | 2.53574347319685 | 36.3082955542531 | Libya |
| 2015 | 40.18053077977 | 0.713651367022809 | 2.76473206200824 | 37.4157987177618 | Libya |
| 2016 | 44.534231573 | 0.710760140384142 | 2.74000568251439 | 41.7942258904856 | Libya |
| 2017 | 45.34900576549 | 0.71066626361534 | 2.64700277865 | 42.70200298684 | Libya |
| 2018 | 40.87932679255 | 0.712720437649946 | 2.69576986639716 | 38.1835569261528 | Libya |
| 2019 | 39.30265087127 | 0.716157238515615 | 2.60738463095817 | 36.6952662403118 | Libya |
| 2020 | 37.921661489 | 0.720270392863655 | 2.604194741254 | 35.317466747746 | Libya |
| 2021 | 37.51962891442 | 0.725771398888229 | 2.40995912096984 | 35.1096697934502 | Libya |
| 1990 | 2353.45312273457 | 0.358072869533996 | 500.160619291247 | 1853.29250344332 | Morocco |
| 1991 | 2129.40895777567 | 0.3649846287647 | 433.663241138365 | 1695.7457166373 | Morocco |
| 1992 | 1965.07257053015 | 0.371187194023026 | 406.20303401389 | 1558.86953651626 | Morocco |
| 1993 | 1814.07042703447 | 0.37677897731043 | 349.765045183484 | 1464.30538185099 | Morocco |
| 1994 | 1676.88604134786 | 0.382849450670084 | 323.288486560925 | 1353.59755478694 | Morocco |
| 1995 | 1555.89395767366 | 0.388164109911114 | 286.592287400219 | 1269.30167027344 | Morocco |
| 1996 | 1439.77013344489 | 0.394150096117346 | 289.479945075865 | 1150.29018836902 | Morocco |
| 1997 | 1343.85119301885 | 0.399504009010139 | 259.015735893136 | 1084.83545712571 | Morocco |
| 1998 | 1251.68739911892 | 0.405038791986878 | 243.345084159657 | 1008.34231495926 | Morocco |
| 1999 | 1167.61232484378 | 0.410303190621115 | 217.662160658312 | 949.950164185468 | Morocco |
| 2000 | 1092.53842243422 | 0.415344026415974 | 197.520448428613 | 895.017974005607 | Morocco |
| 2001 | 1025.60111133844 | 0.420595133422507 | 198.577242907485 | 827.023868430955 | Morocco |
| 2002 | 965.5013438409 | 0.425775750183888 | 178.737053200386 | 786.764290640514 | Morocco |
| 2003 | 889.72855006657 | 0.431217810181359 | 141.079775662991 | 748.648774403579 | Morocco |
| 2004 | 830.48163147644 | 0.436822351876544 | 128.545173765897 | 701.936457710543 | Morocco |
| 2005 | 774.13238109889 | 0.442531339370103 | 112.608300643541 | 661.524080455349 | Morocco |
| 2006 | 714.42941717743 | 0.448676160448964 | 105.912644689754 | 608.516772487676 | Morocco |
| 2007 | 651.10905240017 | 0.455036324601373 | 99.1747598911773 | 551.934292508993 | Morocco |
| 2008 | 586.66795512538 | 0.461803552255995 | 91.5954479338913 | 495.072507191489 | Morocco |
| 2009 | 536.27299846551 | 0.468929893059803 | 83.0689996575188 | 453.203998807991 | Morocco |
| 2010 | 464.38395831738 | 0.476269799414457 | 79.1247540046681 | 385.259204312712 | Morocco |
| 2011 | 397.72943463602 | 0.483922151996701 | 76.946536809861 | 320.782897826159 | Morocco |
| 2012 | 346.57378700067 | 0.491603391761499 | 75.3316801501165 | 271.242106850553 | Morocco |
| 2013 | 305.89288686416 | 0.499528606645883 | 17.3640887184677 | 288.528798145692 | Morocco |
| 2014 | 292.80563634982 | 0.507443265160336 | 15.9916039602492 | 276.814032389571 | Morocco |
| 2015 | 264.94550098163 | 0.515582892643316 | 15.3538899082984 | 249.591611073332 | Morocco |
| 2016 | 240.10819508841 | 0.523518592215067 | 15.1727135505311 | 224.935481537879 | Morocco |
| 2017 | 218.08735423916 | 0.531610861944961 | 15.113999924484 | 202.973354314676 | Morocco |
| 2018 | 199.14452165945 | 0.539729670716936 | 15.0866897376994 | 184.057831921751 | Morocco |
| 2019 | 178.86884814821 | 0.547837444469332 | 14.540725709008 | 164.328122439202 | Morocco |
| 2020 | 161.55169383513 | 0.555246068582659 | 12.713970087533 | 148.837723747597 | Morocco |
| 2021 | 149.52742932065 | 0.562698300927195 | 11.2513998252782 | 138.276029495372 | Morocco |
| 1990 | 294.64266494253 | 0.401792210145272 | 236.227808624361 | 58.4148563181689 | Palestine |
| 1991 | 263.75492221086 | 0.405727150469488 | 228.74981329195 | 35.00510891891 | Palestine |
| 1992 | 232.89863027882 | 0.411146868747336 | 214.018644262793 | 18.8799860160269 | Palestine |
| 1993 | 203.34075115806 | 0.416779648694491 | 192.781408391901 | 10.5593427661589 | Palestine |
| 1994 | 175.37723643851 | 0.423136672782837 | 173.536012993373 | 1.84122344513719 | Palestine |
| 1995 | 152.35378650541 | 0.42930702715431 | 136.704701113238 | 15.6490853921719 | Palestine |
| 1996 | 133.40566518727 | 0.434731371082746 | 124.906628011354 | 8.49903717591621 | Palestine |
| 1997 | 117.55140081349 | 0.440901776082897 | 116.301030511095 | 1.2503703023946 | Palestine |
| 1998 | 104.0853778658 | 0.447782415404353 | 102.51681165884 | 1.56856620696 | Palestine |
| 1999 | 93.84007659157 | 0.454909692070064 | 93.84007659157 | 0 | Palestine |
| 2000 | 87.16177959171 | 0.460681496412606 | 86.9369740864689 | 0.224805505241093 | Palestine |
| 2001 | 82.64975090818 | 0.465299102197142 | 82.64975090818 | 0 | Palestine |
| 2002 | 80.1977071643 | 0.468600256734998 | 80.1977071643 | 0 | Palestine |
| 2003 | 77.63520614304 | 0.472881732260502 | 77.63520614304 | 0 | Palestine |
| 2004 | 74.93788703391 | 0.478527322159265 | 74.93788703391 | 0 | Palestine |
| 2005 | 73.31303986735 | 0.485320585803801 | 73.31303986735 | 0 | Palestine |
| 2006 | 69.87409036259 | 0.491890406552455 | 69.87409036259 | 0 | Palestine |
| 2007 | 67.15967613377 | 0.498978425791333 | 17.9077651986592 | 49.2519109351108 | Palestine |
| 2008 | 61.71716697246 | 0.506849478804306 | 15.9662778324897 | 45.7508891399703 | Palestine |
| 2009 | 55.52310151437 | 0.515815835660256 | 15.2952044521815 | 40.2278970621885 | Palestine |
| 2010 | 49.8964765147 | 0.525711905369362 | 15.0277953010232 | 34.8686812136768 | Palestine |
| 2011 | 46.14141570627 | 0.536777629566224 | 15.0642520649363 | 31.0771636413337 | Palestine |
| 2012 | 42.30734761954 | 0.548295425023352 | 14.5494013704982 | 27.7579462490418 | Palestine |
| 2013 | 38.96153431583 | 0.55916348091297 | 12.1008623677458 | 26.8606719480842 | Palestine |
| 2014 | 36.10611706314 | 0.569239881995993 | 9.47388231605018 | 26.6322347470898 | Palestine |
| 2015 | 34.27290218394 | 0.579204260933414 | 8.87572721730747 | 25.3971749666325 | Palestine |
| 2016 | 28.27989667469 | 0.589492769984704 | 9.00684394844298 | 19.273052726247 | Palestine |
| 2017 | 26.70598853667 | 0.599157186635533 | 8.65984131144808 | 18.0461472252219 | Palestine |
| 2018 | 24.92859084889 | 0.608124860877158 | 8.53493185273894 | 16.3936589961511 | Palestine |
| 2019 | 23.34473748997 | 0.616466838514416 | 9.04703616825424 | 14.2977013217158 | Palestine |
| 2020 | 21.23283029395 | 0.623810015023286 | 8.869181580721 | 12.363648713229 | Palestine |
| 2021 | 20.50005602271 | 0.631011665054344 | 8.08748839717964 | 12.4125676255304 | Palestine |
| 1990 | 114.90776074557 | 0.429270948783208 | 114.90776074557 | 0 | Oman |
| 1991 | 101.40574392891 | 0.442268122012418 | 101.40574392891 | 0 | Oman |
| 1992 | 86.70592398386 | 0.457737972074553 | 86.70592398386 | 0 | Oman |
| 1993 | 76.01819962695 | 0.474999032775331 | 76.01819962695 | 0 | Oman |
| 1994 | 67.92006593039 | 0.493513107048941 | 67.92006593039 | 0 | Oman |
| 1995 | 59.4295825947 | 0.513194359370639 | 15.6376799040934 | 43.7919026906066 | Oman |
| 1996 | 52.20249983671 | 0.533246393920158 | 15.0802482183299 | 37.1222516183801 | Oman |
| 1997 | 46.26687356263 | 0.554315537130654 | 12.9223830490617 | 33.3444905135683 | Oman |
| 1998 | 41.74108933593 | 0.574474902223599 | 8.90882265984048 | 32.8322666760895 | Oman |
| 1999 | 37.93817847533 | 0.592398470428405 | 9.12019005544531 | 28.8179884198847 | Oman |
| 2000 | 34.35629083217 | 0.608421540646462 | 8.90861139895264 | 25.4476794332174 | Oman |
| 2001 | 31.3264007915 | 0.622160627189522 | 8.77141052483148 | 22.5549902666685 | Oman |
| 2002 | 28.89744531182 | 0.63516212584598 | 7.00252751414893 | 21.8949177976711 | Oman |
| 2003 | 26.81829795587 | 0.647249092690596 | 5.96867140433826 | 20.8496265515317 | Oman |
| 2004 | 25.03882844623 | 0.65762611814533 | 4.82554515541648 | 20.2132832908135 | Oman |
| 2005 | 23.41135618968 | 0.666401230917978 | 4.09984212047862 | 19.3115140692014 | Oman |
| 2006 | 23.14143226939 | 0.675353885063336 | 3.65837703037467 | 19.4830552390153 | Oman |
| 2007 | 23.49489561351 | 0.685055582772953 | 3.37529596643159 | 20.1195996470784 | Oman |
| 2008 | 24.47673437352 | 0.694952262906783 | 3.07934431515034 | 21.3973900583697 | Oman |
| 2009 | 25.46027050502 | 0.703098570806704 | 2.92980664227271 | 22.5304638627473 | Oman |
| 2010 | 25.39870530779 | 0.710688694921138 | 2.75937445783912 | 22.6393308499509 | Oman |
| 2011 | 25.68083750327 | 0.718264528020208 | 2.60192981077823 | 23.0789076924918 | Oman |
| 2012 | 25.76734632005 | 0.726450074292121 | 2.35277137662421 | 23.4145749434258 | Oman |
| 2013 | 25.55994351592 | 0.733432490235765 | 2.18263606594611 | 23.3773074499739 | Oman |
| 2014 | 25.21271073354 | 0.738743771923443 | 1.88335964388571 | 23.3293510896543 | Oman |
| 2015 | 25.43623568952 | 0.743597182373955 | 1.86171231672729 | 23.5745233727927 | Oman |
| 2016 | 25.94532252274 | 0.74879719338257 | 1.88066676583813 | 24.0646557569019 | Oman |
| 2017 | 25.73956616321 | 0.753786057672964 | 1.83168894781036 | 23.9078772153996 | Oman |
| 2018 | 24.80259816406 | 0.759179513636833 | 1.67401971122391 | 23.1285784528361 | Oman |
| 2019 | 23.80466660926 | 0.764527863267196 | 1.7047309352019 | 22.0999356740581 | Oman |
| 2020 | 22.03174620616 | 0.768854215658189 | 1.73460627012225 | 20.2971399360377 | Oman |
| 2021 | 20.55369244849 | 0.773391601871737 | 1.71606720216338 | 18.8376252463266 | Oman |
| 1990 | 49.74039532188 | 0.651208375960545 | 4.95937489219922 | 44.7810204296808 | Qatar |
| 1991 | 46.97770550877 | 0.655338766647052 | 5.37605807149286 | 41.6016474372771 | Qatar |
| 1992 | 44.28617321241 | 0.660673763366912 | 4.33719179741264 | 39.9489814149974 | Qatar |
| 1993 | 41.86952976384 | 0.666194398536698 | 4.03273761008647 | 37.8367921537535 | Qatar |
| 1994 | 39.84999535322 | 0.672025383885115 | 3.78522440156976 | 36.0647709516502 | Qatar |
| 1995 | 37.97653679477 | 0.678320339337967 | 3.58884065110648 | 34.3876961436635 | Qatar |
| 1996 | 36.53399638049 | 0.68483591897584 | 3.33616284672972 | 33.1978335337603 | Qatar |
| 1997 | 35.16904373298 | 0.693109653614432 | 3.06233510879487 | 32.1067086241851 | Qatar |
| 1998 | 34.03742116498 | 0.701409063669436 | 2.84082262051386 | 31.1965985444661 | Qatar |
| 1999 | 33.04571434433 | 0.708880981468347 | 2.68527963139671 | 30.3604347129333 | Qatar |
| 2000 | 31.98989962769 | 0.714831609105106 | 2.63422344351034 | 29.3556761841797 | Qatar |
| 2001 | 31.49101701092 | 0.720831064006284 | 2.43942228736034 | 29.0515947235597 | Qatar |
| 2002 | 30.54821149715 | 0.727112174151443 | 2.33012186850136 | 28.2180896286486 | Qatar |
| 2003 | 29.58245211812 | 0.733536599318407 | 2.19222524753462 | 27.3902268705854 | Qatar |
| 2004 | 28.81842637979 | 0.740223208352271 | 1.74528715296724 | 27.0731392268228 | Qatar |
| 2005 | 28.36054060467 | 0.747189358624771 | 1.83250500248024 | 26.5280356021898 | Qatar |
| 2006 | 27.63192016446 | 0.754123028435068 | 1.799273836271 | 25.832646328189 | Qatar |
| 2007 | 27.08486361454 | 0.760960134224895 | 1.74275424878 | 25.34210936576 | Qatar |
| 2008 | 26.60045493371 | 0.767659233393271 | 1.75678706083962 | 24.8436678728704 | Qatar |
| 2009 | 23.15366960656 | 0.77427684914439 | 1.74257162329351 | 21.4110979832665 | Qatar |
| 2010 | 22.02284433651 | 0.780807073920169 | 1.64979519031116 | 20.3730491461988 | Qatar |
| 2011 | 20.90012247775 | 0.7872124503355 | 1.62780706822565 | 19.2723154095243 | Qatar |
| 2012 | 19.93338889582 | 0.793494900808854 | 1.57174777478587 | 18.3616411210341 | Qatar |
| 2013 | 18.67345434858 | 0.799611554876573 | 1.54088942418127 | 17.1325649243987 | Qatar |
| 2014 | 17.69918443401 | 0.805725710213966 | 1.52274486355848 | 16.1764395704515 | Qatar |
| 2015 | 16.77815940153 | 0.811872851894902 | 1.52433782690797 | 15.253821574622 | Qatar |
| 2016 | 16.05083799533 | 0.817979840558073 | 1.52427441499887 | 14.5265635803311 | Qatar |
| 2017 | 15.55244239408 | 0.824016868434921 | 1.48989490023697 | 14.062547493843 | Qatar |
| 2018 | 15.02934025248 | 0.829962126581261 | 1.48423401333574 | 13.5451062391443 | Qatar |
| 2019 | 14.53593303986 | 0.835805284348454 | 1.48509436972021 | 13.0508386701398 | Qatar |
| 2020 | 13.89135525753 | 0.841467395353032 | 1.46673660886574 | 12.4246186486643 | Qatar |
| 2021 | 12.3914746967 | 0.846860584038945 | 1.46343689074556 | 10.9280378059544 | Qatar |
| 1990 | 349.5252383516 | 0.538954515496368 | 15.0804532175574 | 334.444785134043 | Saudi Arabia |
| 1991 | 314.51332231688 | 0.549907716626613 | 13.8203113815468 | 300.693010935333 | Saudi Arabia |
| 1992 | 282.96806027019 | 0.561144266304623 | 11.4491119987089 | 271.518948271481 | Saudi Arabia |
| 1993 | 253.78520596413 | 0.572186913451422 | 8.85337195821827 | 244.931834005912 | Saudi Arabia |
| 1994 | 228.47572447205 | 0.582878871179675 | 8.99904027984842 | 219.476684192202 | Saudi Arabia |
| 1995 | 205.18354249961 | 0.593251423065509 | 8.77211729003709 | 196.411425209573 | Saudi Arabia |
| 1996 | 182.26117116716 | 0.603648418303976 | 8.68660867003625 | 173.574562497124 | Saudi Arabia |
| 1997 | 163.78419800695 | 0.613749116453829 | 8.89338250873486 | 154.890815498215 | Saudi Arabia |
| 1998 | 148.35757895136 | 0.623518214245881 | 8.6789020959816 | 139.678676855378 | Saudi Arabia |
| 1999 | 135.50197354064 | 0.632796447356413 | 7.06574642791829 | 128.436227112722 | Saudi Arabia |
| 2000 | 123.71715263645 | 0.642311774343419 | 6.12651964374316 | 117.590632992707 | Saudi Arabia |
| 2001 | 113.45574137335 | 0.651502091755966 | 5.05150788692976 | 108.40423348642 | Saudi Arabia |
| 2002 | 103.54790899824 | 0.660356855701209 | 4.68879218230088 | 98.8591168159391 | Saudi Arabia |
| 2003 | 94.04457719275 | 0.669839576958755 | 3.88583749345559 | 90.1587396992944 | Saudi Arabia |
| 2004 | 84.82461885534 | 0.679917856369452 | 3.6026874645042 | 81.2219313908358 | Saudi Arabia |
| 2005 | 76.92472870985 | 0.690520851691753 | 3.38139766578207 | 73.5433310440679 | Saudi Arabia |
| 2006 | 69.76617268929 | 0.70115261257815 | 2.94138414355928 | 66.8247885457307 | Saudi Arabia |
| 2007 | 63.60683086283 | 0.711442141049593 | 2.73720606824877 | 60.8696247945812 | Saudi Arabia |
| 2008 | 57.89501047257 | 0.721960204973632 | 2.59034116117635 | 55.3046693113937 | Saudi Arabia |
| 2009 | 53.01072609437 | 0.731158488162749 | 2.20038590745052 | 50.8103401869195 | Saudi Arabia |
| 2010 | 48.69124629042 | 0.740300669205333 | 1.877368101228 | 46.813878189192 | Saudi Arabia |
| 2011 | 44.71267020618 | 0.749959451001828 | 1.84561097507444 | 42.8670592311056 | Saudi Arabia |
| 2012 | 40.98753520584 | 0.759054935383679 | 1.7536673974787 | 39.2338678083613 | Saudi Arabia |
| 2013 | 33.6809484814 | 0.767387864281482 | 1.80325617766342 | 31.8776923037366 | Saudi Arabia |
| 2014 | 31.37029377602 | 0.775178132630626 | 1.75282573803973 | 29.6174680379803 | Saudi Arabia |
| 2015 | 29.43338058977 | 0.782300801582157 | 1.69471482159865 | 27.7386657681714 | Saudi Arabia |
| 2016 | 27.91196954629 | 0.788867882259991 | 1.61479235006956 | 26.2971771962204 | Saudi Arabia |
| 2017 | 26.44343057591 | 0.794886287697922 | 1.56900401115284 | 24.8744265647572 | Saudi Arabia |
| 2018 | 25.16686476312 | 0.800656984976864 | 1.53770206595284 | 23.6291626971672 | Saudi Arabia |
| 2019 | 24.18266010213 | 0.806087840988648 | 1.5321489773707 | 22.6505111247593 | Saudi Arabia |
| 2020 | 21.14833650559 | 0.810541860714478 | 1.51551708072973 | 19.6328194248603 | Saudi Arabia |
| 2021 | 20.78635361882 | 0.815143493214829 | 1.51280028169758 | 19.2735533371224 | Saudi Arabia |
| 1990 | 269.15446989599 | 0.430492642865419 | 141.594908547192 | 127.559561348798 | Syrian Arab Republic |
| 1991 | 245.82545780875 | 0.437907493360423 | 124.751120431081 | 121.074337377669 | Syrian Arab Republic |
| 1992 | 221.39453951461 | 0.445904235849208 | 109.935742828981 | 111.458796685629 | Syrian Arab Republic |
| 1993 | 196.59943230779 | 0.45414785985736 | 106.119231203119 | 90.480201104671 | Syrian Arab Republic |
| 1994 | 176.00354301579 | 0.462437297890694 | 90.3794491616993 | 85.6240938540907 | Syrian Arab Republic |
| 1995 | 155.9644063335 | 0.470692042726613 | 83.1388837961774 | 72.8255225373226 | Syrian Arab Republic |
| 1996 | 132.40435445077 | 0.478743085009033 | 77.0764722283628 | 55.3278822224072 | Syrian Arab Republic |
| 1997 | 112.14808952939 | 0.48623235022627 | 76.1923931000353 | 35.9556964293547 | Syrian Arab Republic |
| 1998 | 93.9111720669 | 0.493707991654398 | 70.4111634803373 | 23.5000085865627 | Syrian Arab Republic |
| 1999 | 83.64768571123 | 0.500474869218632 | 16.6275411998508 | 67.0201445113792 | Syrian Arab Republic |
| 2000 | 70.61036157588 | 0.507143685736932 | 15.9250328030325 | 54.6853287728475 | Syrian Arab Republic |
| 2001 | 61.98359372865 | 0.513850111268667 | 15.6251376978135 | 46.3584560308365 | Syrian Arab Republic |
| 2002 | 56.04641502569 | 0.521309744436802 | 15.1144357578659 | 40.9319792678241 | Syrian Arab Republic |
| 2003 | 48.63388989843 | 0.528935756199872 | 15.1304998136103 | 33.5033900848197 | Syrian Arab Republic |
| 2004 | 45.42340383301 | 0.538084469307822 | 15.0541098033241 | 30.3692940296859 | Syrian Arab Republic |
| 2005 | 45.50433221826 | 0.549750448700287 | 13.701177829039 | 31.803154389221 | Syrian Arab Republic |
| 2006 | 45.54063744926 | 0.560484761476108 | 11.9836317724975 | 33.5570056767625 | Syrian Arab Republic |
| 2007 | 42.06596998009 | 0.570028120377629 | 8.98151076931162 | 33.0844592107784 | Syrian Arab Republic |
| 2008 | 42.17286181841 | 0.578990492745018 | 8.79939395901884 | 33.3734678593912 | Syrian Arab Republic |
| 2009 | 39.6730361606 | 0.587789117723187 | 8.78630529255645 | 30.8867308680435 | Syrian Arab Republic |
| 2010 | 37.61055546238 | 0.59596776440437 | 8.72988946660208 | 28.8806659957779 | Syrian Arab Republic |
| 2011 | 36.44985402609 | 0.60152445568341 | 8.93326659570552 | 27.5165874303845 | Syrian Arab Republic |
| 2012 | 33.96614991229 | 0.60287260117891 | 8.94278117635333 | 25.0233687359367 | Syrian Arab Republic |
| 2013 | 32.61752659425 | 0.6024101339716 | 8.91186867570095 | 23.7056579185491 | Syrian Arab Republic |
| 2014 | 33.08307954908 | 0.602235759940116 | 8.85939225005197 | 24.223687299028 | Syrian Arab Republic |
| 2015 | 32.66241422112 | 0.602282913873841 | 8.66074251332106 | 24.0016717077989 | Syrian Arab Republic |
| 2016 | 31.07431856569 | 0.602911181092802 | 8.93104251080556 | 22.1432760548844 | Syrian Arab Republic |
| 2017 | 28.78679028094 | 0.604896366029073 | 8.95246541476032 | 19.8343248661797 | Syrian Arab Republic |
| 2018 | 26.55943581752 | 0.608507163893069 | 8.9375485044871 | 17.6218873130329 | Syrian Arab Republic |
| 2019 | 24.29007492005 | 0.613336835963498 | 8.78339676243935 | 15.5066781576106 | Syrian Arab Republic |
| 2020 | 21.42811645379 | 0.61772759307296 | 8.81577418211594 | 12.6123422716741 | Syrian Arab Republic |
| 2021 | 19.93856149954 | 0.62300407463119 | 8.92278151932403 | 11.015779980216 | Syrian Arab Republic |
| 1990 | 527.61819219987 | 0.471138521303638 | 82.7254359837882 | 444.892756216082 | Tunisia |
| 1991 | 472.81487407799 | 0.479543056615384 | 77.1455396786853 | 395.669334399305 | Tunisia |
| 1992 | 424.54621469056 | 0.488285281381984 | 74.5425480879064 | 350.003666602654 | Tunisia |
| 1993 | 381.3096402362 | 0.496852038879262 | 27.8580573212832 | 353.451582914917 | Tunisia |
| 1994 | 339.98865808861 | 0.505564882262378 | 16.1877121136751 | 323.800945974935 | Tunisia |
| 1995 | 303.24667961303 | 0.514196940056936 | 15.5953445596673 | 287.651335053363 | Tunisia |
| 1996 | 266.10857606803 | 0.523294273698708 | 15.2038959069085 | 250.904680161122 | Tunisia |
| 1997 | 232.37716255759 | 0.532064044257322 | 15.0728424089643 | 217.304320148626 | Tunisia |
| 1998 | 203.13406994675 | 0.540498160807659 | 15.0858060091205 | 188.048263937629 | Tunisia |
| 1999 | 183.64931359298 | 0.548724563004994 | 14.4348927440667 | 169.214420848913 | Tunisia |
| 2000 | 170.7718777476 | 0.556672649979971 | 13.0443166736643 | 157.727561073936 | Tunisia |
| 2001 | 161.59193757314 | 0.564421010157184 | 10.8869356176293 | 150.705001955511 | Tunisia |
| 2002 | 154.74626989015 | 0.571719533904953 | 8.7972052692676 | 145.949064620882 | Tunisia |
| 2003 | 147.40991393469 | 0.578882668513321 | 8.98745357054169 | 138.422460364148 | Tunisia |
| 2004 | 138.21419435583 | 0.586032594266679 | 9.06929039702325 | 129.144903958807 | Tunisia |
| 2005 | 131.649157896 | 0.592937759304517 | 8.85759326886875 | 122.791564627131 | Tunisia |
| 2006 | 126.90299508443 | 0.59975851290592 | 8.65351415472697 | 118.249480929703 | Tunisia |
| 2007 | 123.65401514785 | 0.606696784272835 | 8.91000401402 | 114.74401113383 | Tunisia |
| 2008 | 120.76836428791 | 0.613529322048559 | 8.88428546608944 | 111.884078821821 | Tunisia |
| 2009 | 118.35402137396 | 0.620106833480493 | 8.99325627923 | 109.36076509473 | Tunisia |
| 2010 | 115.3975030182 | 0.626432773734803 | 8.8141402757788 | 106.583362742421 | Tunisia |
| 2011 | 109.33270613521 | 0.632019840139461 | 8.21605770730675 | 101.116648427903 | Tunisia |
| 2012 | 103.19563124293 | 0.637578344563551 | 6.52145901238465 | 96.6741722305454 | Tunisia |
| 2013 | 97.85871332106 | 0.643008511949972 | 6.36075872416176 | 91.4979545968982 | Tunisia |
| 2014 | 92.50763799206 | 0.648297395047989 | 6.0544275267203 | 86.4532104653397 | Tunisia |
| 2015 | 87.47998829965 | 0.653413537772423 | 5.24381126471786 | 82.2361770349321 | Tunisia |
| 2016 | 83.16556383579 | 0.658353516621193 | 4.50377241829373 | 78.6617914174963 | Tunisia |
| 2017 | 76.12563023782 | 0.663222845186053 | 4.1733101161412 | 71.9523201216788 | Tunisia |
| 2018 | 70.01487962101 | 0.668218997464981 | 3.87104700172761 | 66.1438326192824 | Tunisia |
| 2019 | 65.68490763122 | 0.673170551594905 | 3.81288041081594 | 61.8720272204041 | Tunisia |
| 2020 | 60.41297715686 | 0.677615147910612 | 3.61455581388573 | 56.7984213429743 | Tunisia |
| 2021 | 57.81440303962 | 0.682432215876962 | 3.62123707948672 | 54.1931659601333 | Tunisia |
| 1990 | 1050.81354464713 | 0.461606983777208 | 91.4323688665237 | 959.381175780606 | Turkey |
| 1991 | 951.11994677502 | 0.469244066030772 | 82.937881182375 | 868.182065592645 | Turkey |
| 1992 | 865.08205454458 | 0.476814569059173 | 78.4267695844133 | 786.655284960167 | Turkey |
| 1993 | 790.02598755343 | 0.484656970229106 | 77.0780747700935 | 712.947912783337 | Turkey |
| 1994 | 734.48330514875 | 0.491619950496974 | 75.3002612412759 | 659.183043907474 | Turkey |
| 1995 | 678.92976926657 | 0.498824840241401 | 17.9282535757412 | 661.001515690829 | Turkey |
| 1996 | 620.82744886682 | 0.506238546050969 | 16.06115751136 | 604.76629135546 | Turkey |
| 1997 | 570.41462306233 | 0.513933355794628 | 15.7477558686627 | 554.666867193667 | Turkey |
| 1998 | 519.03372436091 | 0.521509897761014 | 15.1947710378967 | 503.838953323013 | Turkey |
| 1999 | 463.177335972 | 0.528512415528019 | 15.0997273323843 | 448.077608639616 | Turkey |
| 2000 | 416.52039588967 | 0.535866331151872 | 15.1125337502388 | 401.407862139431 | Turkey |
| 2001 | 383.28110583693 | 0.542359130151394 | 15.0347451701235 | 368.246360666807 | Turkey |
| 2002 | 350.86349712358 | 0.549023644141264 | 14.4923251634278 | 336.371171960152 | Turkey |
| 2003 | 309.62606424405 | 0.556102526706668 | 12.886055179403 | 296.740009064647 | Turkey |
| 2004 | 276.94825852575 | 0.564023999784751 | 10.6556521441322 | 266.292606381618 | Turkey |
| 2005 | 244.05299641572 | 0.572495886408665 | 8.96107686490864 | 235.091919550811 | Turkey |
| 2006 | 217.99398780828 | 0.581383178782649 | 9.016186894925 | 208.977800913355 | Turkey |
| 2007 | 196.63521604437 | 0.590442387296341 | 8.72691604535784 | 187.908299999012 | Turkey |
| 2008 | 179.39074776853 | 0.599212061486959 | 8.99321123866158 | 170.397536529868 | Turkey |
| 2009 | 161.17592890229 | 0.607104617902237 | 8.95406609994448 | 152.221862802346 | Turkey |
| 2010 | 146.75712059487 | 0.615597227656952 | 8.90365976457839 | 137.853460830292 | Turkey |
| 2011 | 127.89717134778 | 0.624787210625257 | 8.86340446664872 | 119.033766881131 | Turkey |
| 2012 | 117.3350829978 | 0.634097091952487 | 7.18183439078608 | 110.153248607014 | Turkey |
| 2013 | 109.32519298289 | 0.643723822649817 | 6.03710769707807 | 103.288085285812 | Turkey |
| 2014 | 102.25456919307 | 0.653388834851172 | 4.98163930508446 | 97.2729298879855 | Turkey |
| 2015 | 94.11330410691 | 0.662971738649364 | 4.36587830581716 | 89.7474258010928 | Turkey |
| 2016 | 86.83907208551 | 0.671948147539094 | 3.80189368477261 | 83.0371784007374 | Turkey |
| 2017 | 79.88976983317 | 0.681096272126073 | 3.57852882756491 | 76.3112410056051 | Turkey |
| 2018 | 74.66865144154 | 0.689895866860746 | 3.36701410092864 | 71.3016373406114 | Turkey |
| 2019 | 70.73430626681 | 0.69826377357682 | 3.18209985030706 | 67.5522064165029 | Turkey |
| 2020 | 64.52837632439 | 0.70579974360086 | 2.88436960459342 | 61.6440067197966 | Turkey |
| 2021 | 62.41585232201 | 0.712692673098478 | 2.72376213874415 | 59.6920901832658 | Turkey |
| 1990 | 81.8295950328 | 0.644412271353864 | 5.98033498708631 | 75.8492600457137 | United Arab Emirates |
| 1991 | 75.55645239973 | 0.660739899044445 | 4.48178232951735 | 71.0746700702126 | United Arab Emirates |
| 1992 | 71.64746930922 | 0.675926211137966 | 3.62029036127098 | 68.027178947949 | United Arab Emirates |
| 1993 | 62.03622552355 | 0.689463800557258 | 3.41196204652467 | 58.6242634770253 | United Arab Emirates |
| 1994 | 55.92174669344 | 0.701656792310882 | 2.94780534252212 | 52.9739413509179 | United Arab Emirates |
| 1995 | 49.20551158228 | 0.713138287743939 | 2.66982891114852 | 46.5356826711315 | United Arab Emirates |
| 1996 | 43.57262613921 | 0.724181976276083 | 2.38295808872531 | 41.1896680504847 | United Arab Emirates |
| 1997 | 42.26127948796 | 0.734723175496013 | 2.02085044922231 | 40.2404290387377 | United Arab Emirates |
| 1998 | 43.35241814824 | 0.744476623052101 | 1.86056196533362 | 41.4918561829064 | United Arab Emirates |
| 1999 | 46.55145640996 | 0.753253916450152 | 1.76631183906157 | 44.7851445708984 | United Arab Emirates |
| 2000 | 47.32569224402 | 0.760969094123031 | 1.74785901971432 | 45.5778332243057 | United Arab Emirates |
| 2001 | 48.35814889915 | 0.767858792936623 | 1.75092195651983 | 46.6072269426302 | United Arab Emirates |
| 2002 | 49.51913558327 | 0.774346424129537 | 1.74451753904649 | 47.7746180442235 | United Arab Emirates |
| 2003 | 49.26199847194 | 0.781143991614649 | 1.72068280247145 | 47.5413156694685 | United Arab Emirates |
| 2004 | 46.11651723991 | 0.788525951082133 | 1.66797916458613 | 44.4485380753239 | United Arab Emirates |
| 2005 | 44.30879199478 | 0.796428085714925 | 1.54271702399274 | 42.7660749707873 | United Arab Emirates |
| 2006 | 42.02552640413 | 0.805537022535992 | 1.5303231960589 | 40.4952032080711 | United Arab Emirates |
| 2007 | 45.31110860266 | 0.814870878080389 | 1.50611437432349 | 43.8049942283365 | United Arab Emirates |
| 2008 | 49.01503279332 | 0.822793435350208 | 1.49242177005776 | 47.5226110232622 | United Arab Emirates |
| 2009 | 53.3346998308 | 0.82830416539166 | 1.48486731783082 | 51.8498325129692 | United Arab Emirates |
| 2010 | 55.02021585974 | 0.831284254488885 | 1.4780975399129 | 53.5421183198271 | United Arab Emirates |
| 2011 | 53.59027493367 | 0.832912803937778 | 1.4921676559589 | 52.0981072777111 | United Arab Emirates |
| 2012 | 51.87051334427 | 0.833586109601576 | 1.48383303816091 | 50.3866803061091 | United Arab Emirates |
| 2013 | 50.20185788411 | 0.833803818475535 | 1.49045398553295 | 48.711403898577 | United Arab Emirates |
| 2014 | 42.33873369108 | 0.833858726882329 | 1.47823976844429 | 40.8604939226357 | United Arab Emirates |
| 2015 | 39.65189008058 | 0.834175498027292 | 1.47620896229835 | 38.1756811182816 | United Arab Emirates |
| 2016 | 37.28487026364 | 0.835220709910024 | 1.47815064827439 | 35.8067196153656 | United Arab Emirates |
| 2017 | 35.36289314089 | 0.837213552633191 | 1.47852083314343 | 33.8843723077466 | United Arab Emirates |
| 2018 | 31.01681782469 | 0.839900140178067 | 1.4760940863369 | 29.5407237383531 | United Arab Emirates |
| 2019 | 27.21794342959 | 0.842985326255825 | 1.46352943734987 | 25.7544139922401 | United Arab Emirates |
| 2020 | 24.50130782698 | 0.846151975991622 | 1.46190816456414 | 23.0393996624159 | United Arab Emirates |
| 2021 | 21.368960662 | 0.849317734035032 | 1.46855277009102 | 19.900407891909 | United Arab Emirates |
| 1990 | 3859.56453396498 | 0.215664585570251 | 2339.31928880941 | 1520.24524515557 | Yemen |
| 1991 | 3626.91971688969 | 0.222885408676441 | 2088.8667105714 | 1538.05300631829 | Yemen |
| 1992 | 3436.36662538512 | 0.230415045958638 | 1906.55607423122 | 1529.8105511539 | Yemen |
| 1993 | 3233.67282117782 | 0.238025246263208 | 1703.52994973261 | 1530.14287144521 | Yemen |
| 1994 | 3026.2710742722 | 0.245879426395306 | 1687.41345239169 | 1338.85762188052 | Yemen |
| 1995 | 2790.94688864503 | 0.253962571622302 | 1496.52362269592 | 1294.42326594911 | Yemen |
| 1996 | 2571.19489197343 | 0.262278612845771 | 1352.80073637091 | 1218.39415560252 | Yemen |
| 1997 | 2372.48998792616 | 0.270782535711374 | 1230.3693252507 | 1142.12066267546 | Yemen |
| 1998 | 2165.07160685243 | 0.279500507186773 | 1150.98076563945 | 1014.09084121298 | Yemen |
| 1999 | 1967.3686608402 | 0.288261301367107 | 1109.50538706903 | 857.863273771174 | Yemen |
| 2000 | 1783.19638441786 | 0.297145157465494 | 1035.75083333825 | 747.445551079611 | Yemen |
| 2001 | 1603.07677504731 | 0.306112423990989 | 961.133917884662 | 641.942857162648 | Yemen |
| 2002 | 1493.29258817249 | 0.31506502273303 | 775.818075872485 | 717.474512300005 | Yemen |
| 2003 | 1349.61621106972 | 0.324081688959523 | 719.287069054275 | 630.329142015445 | Yemen |
| 2004 | 1195.15608641783 | 0.333371285035603 | 647.771673672476 | 547.384412745354 | Yemen |
| 2005 | 1042.00284962787 | 0.343462561815387 | 574.204204003008 | 467.798645624862 | Yemen |
| 2006 | 907.59678739083 | 0.353472947292631 | 555.300183144753 | 352.296604246077 | Yemen |
| 2007 | 853.08020570568 | 0.363301217705729 | 469.141157038864 | 383.939048666815 | Yemen |
| 2008 | 766.08032224713 | 0.373140288261273 | 349.520756455128 | 416.559565792002 | Yemen |
| 2009 | 678.18572657393 | 0.382762252118378 | 323.304780005266 | 354.880946568664 | Yemen |
| 2010 | 593.97770332592 | 0.392936641182874 | 286.93308609923 | 307.04461722669 | Yemen |
| 2011 | 547.36387707778 | 0.40147180053512 | 265.128770883708 | 282.235106194072 | Yemen |
| 2012 | 445.39453200466 | 0.409561348808444 | 220.748928665924 | 224.645603338736 | Yemen |
| 2013 | 406.36185698904 | 0.417642404804605 | 191.814898617468 | 214.546958371572 | Yemen |
| 2014 | 360.09107218723 | 0.425189667293499 | 179.22406492406 | 180.86700726317 | Yemen |
| 2015 | 334.11847561842 | 0.430197789315438 | 136.418751518663 | 197.699724099757 | Yemen |
| 2016 | 363.88619181648 | 0.434232982818394 | 130.634810228931 | 233.251381587549 | Yemen |
| 2017 | 501.60791708946 | 0.437549155246091 | 128.183499033218 | 373.424418056242 | Yemen |
| 2018 | 388.85758808744 | 0.440727991364019 | 129.173308837471 | 259.684279249969 | Yemen |
| 2019 | 352.85034384789 | 0.444001399873635 | 110.177123784181 | 242.673220063709 | Yemen |
| 2020 | 232.87912092626 | 0.44689306923482 | 113.202257850114 | 119.676863076146 | Yemen |
| 2021 | 214.18950021629 | 0.450376375244811 | 107.350198172732 | 106.839302043558 | Yemen |
| 1990 | 3188.57177652516 | 0.173832165473389 | 3188.57177652516 | 0 | Afghanistan |
| 1991 | 3315.18385633 | 0.176472549509622 | 3237.88384971231 | 77.3000066176915 | Afghanistan |
| 1992 | 3424.47247799991 | 0.179633724437178 | 3023.03590597186 | 401.436572028052 | Afghanistan |
| 1993 | 3615.65012360302 | 0.18018371261059 | 3018.32670094296 | 597.323422660063 | Afghanistan |
| 1994 | 3284.02227107341 | 0.178518660381455 | 3016.49054717482 | 267.531723898594 | Afghanistan |
| 1995 | 3406.65380541854 | 0.17827912756413 | 3018.91498969039 | 387.738815728152 | Afghanistan |
| 1996 | 3287.05349616537 | 0.178085403812013 | 3012.14099001641 | 274.912506148961 | Afghanistan |
| 1997 | 3320.11781241473 | 0.177884124107706 | 3010.90955723304 | 309.208255181689 | Afghanistan |
| 1998 | 3269.54645699826 | 0.177513403151835 | 3094.17522492164 | 175.371232076623 | Afghanistan |
| 1999 | 3145.14316602079 | 0.177075239430658 | 3080.17019118207 | 64.9729748387235 | Afghanistan |
| 2000 | 3033.16846470299 | 0.177025771968387 | 3033.16846470299 | 0 | Afghanistan |
| 2001 | 2985.78311552069 | 0.177773144391168 | 2985.78311552069 | 0 | Afghanistan |
| 2002 | 2813.83531107123 | 0.183808429860528 | 2813.83531107123 | 0 | Afghanistan |
| 2003 | 2811.3656369254 | 0.19051899147664 | 2811.3656369254 | 0 | Afghanistan |
| 2004 | 2678.70498994104 | 0.196903541380771 | 2678.70498994104 | 0 | Afghanistan |
| 2005 | 2474.984390012 | 0.203928133334003 | 2474.984390012 | 0 | Afghanistan |
| 2006 | 2201.45830658302 | 0.211074194179476 | 2201.45830658302 | 0 | Afghanistan |
| 2007 | 1970.34416798091 | 0.219627610708056 | 1970.34416798091 | 0 | Afghanistan |
| 2008 | 1732.71198460323 | 0.22808917063877 | 1732.71198460323 | 0 | Afghanistan |
| 2009 | 1518.81282368153 | 0.237900824236794 | 1518.81282368153 | 0 | Afghanistan |
| 2010 | 1383.3992587934 | 0.247759948877388 | 1383.3992587934 | 0 | Afghanistan |
| 2011 | 1261.66422735297 | 0.257041675822107 | 1261.66422735297 | 0 | Afghanistan |
| 2012 | 1156.56722918717 | 0.266484406571128 | 1156.56722918717 | 0 | Afghanistan |
| 2013 | 1132.74766090436 | 0.275636558072642 | 1132.74766090436 | 0 | Afghanistan |
| 2014 | 1063.15417343642 | 0.284030351035349 | 1063.15417343642 | 0 | Afghanistan |
| 2015 | 1001.5779657176 | 0.291849504924728 | 1001.5779657176 | 0 | Afghanistan |
| 2016 | 954.04761540284 | 0.299630695812728 | 951.560886612676 | 2.48672879016362 | Afghanistan |
| 2017 | 889.99906856358 | 0.307424618143983 | 858.787810984063 | 31.2112575795171 | Afghanistan |
| 2018 | 748.20861909493 | 0.314866093354221 | 748.20861909493 | 0 | Afghanistan |
| 2019 | 693.17902118552 | 0.322454046974177 | 693.17902118552 | 0 | Afghanistan |
| 2020 | 619.53114092258 | 0.32983006796412 | 619.53114092258 | 0 | Afghanistan |
| 2021 | 548.56007780288 | 0.337199997837472 | 548.56007780288 | 0 | Afghanistan |
| 1990 | 5797.08142727282 | 0.228548934038183 | 1884.79602763453 | 3912.28539963829 | Bangladesh |
| 1991 | 5735.22670136175 | 0.237207412585347 | 1908.85790208622 | 3826.36879927553 | Bangladesh |
| 1992 | 5085.56987982626 | 0.245247955858354 | 1647.75458218652 | 3437.81529763974 | Bangladesh |
| 1993 | 4641.35482368781 | 0.252090719955727 | 1509.78047771419 | 3131.57434597362 | Bangladesh |
| 1994 | 4225.70539858891 | 0.258997294481156 | 1323.73592290673 | 2901.96947568218 | Bangladesh |
| 1995 | 3819.37991647516 | 0.265293918356151 | 1348.40188347299 | 2470.97803300217 | Bangladesh |
| 1996 | 3389.94611805658 | 0.271177780650834 | 1237.97372164617 | 2151.97239641041 | Bangladesh |
| 1997 | 3008.76019761938 | 0.277773280387452 | 1159.73460111264 | 1849.02559650674 | Bangladesh |
| 1998 | 2630.09390186122 | 0.284515940436142 | 1112.16862218645 | 1517.92527967477 | Bangladesh |
| 1999 | 2309.89000674023 | 0.290951431147001 | 1091.76270855886 | 1218.12729818137 | Bangladesh |
| 2000 | 2053.9978238985 | 0.297251975652563 | 1033.89125897844 | 1020.10656492006 | Bangladesh |
| 2001 | 1826.58611051688 | 0.303188546281531 | 967.014964966048 | 859.571145550831 | Bangladesh |
| 2002 | 1610.22010747498 | 0.308640093479048 | 878.568596350842 | 731.651511124138 | Bangladesh |
| 2003 | 1409.70821219283 | 0.3143600400365 | 859.471926845983 | 550.236285346847 | Bangladesh |
| 2004 | 1264.09716152682 | 0.320922851356659 | 771.904041565905 | 492.193119960915 | Bangladesh |
| 2005 | 1136.40124933007 | 0.328090510389871 | 724.066344064653 | 412.334905265417 | Bangladesh |
| 2006 | 1042.55814261472 | 0.3356905979604 | 657.436673814596 | 385.121468800124 | Bangladesh |
| 2007 | 989.81662855312 | 0.343932545818298 | 574.541775756375 | 415.274852796745 | Bangladesh |
| 2008 | 890.71528320108 | 0.352520032963442 | 555.122275689659 | 335.593007511421 | Bangladesh |
| 2009 | 804.52191763267 | 0.361481512825909 | 470.697465883847 | 333.824451748823 | Bangladesh |
| 2010 | 733.03284240992 | 0.370598956236852 | 402.145194708437 | 330.887647701483 | Bangladesh |
| 2011 | 685.80427182112 | 0.380124885726479 | 322.824575411507 | 362.979696409613 | Bangladesh |
| 2012 | 622.75269028726 | 0.39062355195618 | 283.080243519063 | 339.672446768197 | Bangladesh |
| 2013 | 586.95006945609 | 0.401706477929242 | 240.056088075867 | 346.893981380223 | Bangladesh |
| 2014 | 556.09169845793 | 0.413263168891725 | 216.330828561588 | 339.760869896342 | Bangladesh |
| 2015 | 557.22508130164 | 0.42548212139419 | 177.178955401572 | 380.046125900068 | Bangladesh |
| 2016 | 555.69178495405 | 0.437709906182211 | 127.706971794866 | 427.984813159184 | Bangladesh |
| 2017 | 549.1918185765 | 0.449752413112756 | 106.251600428101 | 442.9402181484 | Bangladesh |
| 2018 | 521.77371143711 | 0.461717748857041 | 89.6676466497461 | 432.106064787364 | Bangladesh |
| 2019 | 494.79520025808 | 0.473264997623618 | 79.5439155876314 | 415.251284670449 | Bangladesh |
| 2020 | 389.52279271529 | 0.483079169271683 | 76.7076870872273 | 312.815105628063 | Bangladesh |
| 2021 | 368.31740798038 | 0.492420884955353 | 72.8013704492113 | 295.516037531169 | Bangladesh |
| 1990 | 8146.79709513387 | 0.215039850206523 | 2333.30137134514 | 5813.49572378873 | Bhutan |
| 1991 | 7751.32662110967 | 0.221312439958873 | 2093.61355614513 | 5657.71306496454 | Bhutan |
| 1992 | 7327.47426248489 | 0.228148271920468 | 1857.44948302913 | 5470.02477945576 | Bhutan |
| 1993 | 7214.2414822097 | 0.236004687298356 | 1847.61752951436 | 5366.62395269534 | Bhutan |
| 1994 | 6664.24485734093 | 0.244331246046266 | 1662.15709914926 | 5002.08775819167 | Bhutan |
| 1995 | 6240.27084544141 | 0.253291956861352 | 1475.56564663024 | 4764.70519881117 | Bhutan |
| 1996 | 5658.42149864946 | 0.262362745638857 | 1364.03346241243 | 4294.38803623703 | Bhutan |
| 1997 | 5392.63110460788 | 0.271487422119903 | 1234.41345641107 | 4158.21764819681 | Bhutan |
| 1998 | 4601.66024067928 | 0.280440384324268 | 1150.52176703518 | 3451.1384736441 | Bhutan |
| 1999 | 3560.26210548448 | 0.28951048779873 | 1099.44275286146 | 2460.81935262302 | Bhutan |
| 2000 | 3795.19918318354 | 0.298804874805939 | 986.784736689933 | 2808.41444649361 | Bhutan |
| 2001 | 3373.25940192595 | 0.308200652419302 | 877.192588420374 | 2496.06681350558 | Bhutan |
| 2002 | 3059.98690338913 | 0.3179435358725 | 780.728919001706 | 2279.25798438742 | Bhutan |
| 2003 | 2867.22140523964 | 0.327670422082998 | 716.793791155297 | 2150.42761408434 | Bhutan |
| 2004 | 2628.25700154305 | 0.337286494753829 | 592.726342425771 | 2035.53065911728 | Bhutan |
| 2005 | 2377.38863252761 | 0.346998577564248 | 569.506906774046 | 1807.88172575356 | Bhutan |
| 2006 | 2117.60799645653 | 0.356824209168712 | 553.504849072242 | 1564.10314738429 | Bhutan |
| 2007 | 1885.92991466998 | 0.367560497563767 | 412.118633626687 | 1473.81128104329 | Bhutan |
| 2008 | 1662.45508900913 | 0.378233542838379 | 347.631512769222 | 1314.82357623991 | Bhutan |
| 2009 | 1432.71134330486 | 0.388977161920706 | 286.219142871779 | 1146.49220043308 | Bhutan |
| 2010 | 1267.0454704976 | 0.399751936485664 | 265.962061302809 | 1001.08340919479 | Bhutan |
| 2011 | 1155.54930271043 | 0.410149248617919 | 220.118259924691 | 935.431042785739 | Bhutan |
| 2012 | 1056.05011939446 | 0.419443216044258 | 195.45374285598 | 860.59637653848 | Bhutan |
| 2013 | 966.67146454032 | 0.427790674624113 | 168.342449380608 | 798.329015159712 | Bhutan |
| 2014 | 894.66232463965 | 0.434969269672115 | 128.581324504558 | 766.081000135092 | Bhutan |
| 2015 | 831.84536646448 | 0.44181074828187 | 122.988924094904 | 708.856442369576 | Bhutan |
| 2016 | 769.14710427648 | 0.448401854533875 | 106.838208004375 | 662.308896272105 | Bhutan |
| 2017 | 707.60365080839 | 0.454280496545609 | 107.504926591474 | 600.098724216916 | Bhutan |
| 2018 | 654.20611214762 | 0.459624710991057 | 93.4978474106555 | 560.708264736965 | Bhutan |
| 2019 | 607.46088283638 | 0.464633353490442 | 91.4784739816517 | 515.982408854728 | Bhutan |
| 2020 | 568.25399735312 | 0.468714478980695 | 82.9527607365627 | 485.301236616557 | Bhutan |
| 2021 | 528.71191409448 | 0.473062378179926 | 79.8369250318891 | 448.874989062591 | Bhutan |
| 1990 | 11815.283490752 | 0.332593603012361 | 648.510333717993 | 11166.773157034 | India |
| 1991 | 11527.6109180044 | 0.338591779142716 | 595.501845782755 | 10932.1090722216 | India |
| 1992 | 11332.7912631257 | 0.344591727089114 | 575.039580446531 | 10757.7516826792 | India |
| 1993 | 10851.2722800828 | 0.350685396969938 | 558.169594275953 | 10293.1026858068 | India |
| 1994 | 10333.3811406001 | 0.356996030489868 | 497.442846131776 | 9835.93829446833 | India |
| 1995 | 9932.62807697772 | 0.363418500556262 | 467.124609092896 | 9465.50346788482 | India |
| 1996 | 9439.60552231957 | 0.370018643898342 | 405.502117540293 | 9034.10340477928 | India |
| 1997 | 8939.1275087815 | 0.37635876286452 | 351.930313371085 | 8587.19719541041 | India |
| 1998 | 8337.85713738888 | 0.382817848453708 | 324.722579922753 | 8013.13455746613 | India |
| 1999 | 7608.06665155889 | 0.389453889040872 | 285.762921220881 | 7322.30373033801 | India |
| 2000 | 7024.56052648246 | 0.395752331114503 | 263.570474254995 | 6760.99005222746 | India |
| 2001 | 6377.52922989514 | 0.401837472284192 | 236.047030500911 | 6141.48219939423 | India |
| 2002 | 5746.12295130356 | 0.407411413581757 | 239.171214644656 | 5506.9517366589 | India |
| 2003 | 5071.38283985276 | 0.413115782576536 | 212.809931104969 | 4858.57290874779 | India |
| 2004 | 4486.51836498705 | 0.419184960649408 | 195.481284285026 | 4291.03708070202 | India |
| 2005 | 4125.68580828951 | 0.425913061870066 | 177.257844796995 | 3948.42796349251 | India |
| 2006 | 3872.27027959623 | 0.433321558442149 | 132.290833362204 | 3739.97944623403 | India |
| 2007 | 3628.92787150164 | 0.441351460389445 | 123.462249850355 | 3505.46562165128 | India |
| 2008 | 3394.63968743388 | 0.44923585077668 | 105.864207681626 | 3288.77547975225 | India |
| 2009 | 3160.10979074189 | 0.457672331967412 | 98.907806607628 | 3061.20198413426 | India |
| 2010 | 2925.64329141036 | 0.467018995596075 | 85.5973066861268 | 2840.04598472423 | India |
| 2011 | 2727.98243279372 | 0.476832909015635 | 77.9828405496835 | 2649.99959224404 | India |
| 2012 | 2586.01873769425 | 0.487210875591002 | 75.8440063664076 | 2510.17473132784 | India |
| 2013 | 2498.9578230101 | 0.498317981286522 | 17.4133379965843 | 2481.54448501352 | India |
| 2014 | 2318.17354768215 | 0.509722099778239 | 15.8779045571213 | 2302.29564312503 | India |
| 2015 | 2136.73051138274 | 0.521242718548368 | 15.1220913037726 | 2121.60842007897 | India |
| 2016 | 1904.8747839886 | 0.532220023184224 | 15.0976555787816 | 1889.77712840982 | India |
| 2017 | 1698.9339709738 | 0.54238850718096 | 15.0376549367108 | 1683.89631603709 | India |
| 2018 | 1579.08551960942 | 0.551930326455295 | 13.8566930654087 | 1565.22882654401 | India |
| 2019 | 1406.22206215927 | 0.560809926886154 | 11.3907296488706 | 1394.8313325104 | India |
| 2020 | 1697.55586787156 | 0.568138149330912 | 9.98369763516824 | 1687.57217023639 | India |
| 2021 | 1532.95035928564 | 0.575401649396992 | 8.85077637363849 | 1524.099582912 | India |
| 1990 | 2882.03009256578 | 0.199560654182432 | 2738.77625726835 | 143.253835297428 | Nepal |
| 1991 | 2837.2706239557 | 0.205687442933642 | 2578.13589761853 | 259.134726337165 | Nepal |
| 1992 | 2523.05722848236 | 0.212113089793247 | 2317.30543958725 | 205.751788895106 | Nepal |
| 1993 | 2368.23644179878 | 0.218843431287675 | 2284.8473741909 | 83.38906760788 | Nepal |
| 1994 | 2216.54380720509 | 0.226073059669684 | 2033.41553469659 | 183.128272508505 | Nepal |
| 1995 | 2073.3334917871 | 0.233253154418138 | 1867.51613024791 | 205.81736153919 | Nepal |
| 1996 | 1932.71318108717 | 0.240700615739874 | 1630.69788635373 | 302.015294733441 | Nepal |
| 1997 | 1779.16259083766 | 0.248382796560591 | 1491.50954845489 | 287.653042382772 | Nepal |
| 1998 | 1637.6329525532 | 0.256227178679174 | 1473.18406264259 | 164.448889910612 | Nepal |
| 1999 | 1488.81483143388 | 0.264359180313156 | 1339.31002830607 | 149.50480312781 | Nepal |
| 2000 | 1354.23569997038 | 0.272719114894473 | 1223.9478534421 | 130.287846528284 | Nepal |
| 2001 | 1224.5445131462 | 0.281287467400935 | 1150.61139968033 | 73.9331134658728 | Nepal |
| 2002 | 1077.73618706447 | 0.289513607929153 | 1069.20037420903 | 8.53581285544396 | Nepal |
| 2003 | 949.79148343506 | 0.297772595071107 | 949.79148343506 | 0 | Nepal |
| 2004 | 839.7436538169 | 0.306196530322722 | 839.7436538169 | 0 | Nepal |
| 2005 | 805.36400828947 | 0.314661796325301 | 805.36400828947 | 0 | Nepal |
| 2006 | 735.98933398272 | 0.323056345509418 | 710.929638686798 | 25.059695295922 | Nepal |
| 2007 | 649.37924102643 | 0.331445610061963 | 634.256203640479 | 15.1230373859506 | Nepal |
| 2008 | 570.60877922141 | 0.340150705786803 | 558.717344748495 | 11.8914344729151 | Nepal |
| 2009 | 551.95566886347 | 0.348701893463869 | 550.175054039014 | 1.78061482445571 | Nepal |
| 2010 | 450.87128450728 | 0.356868100367112 | 450.87128450728 | 0 | Nepal |
| 2011 | 379.21755710253 | 0.364642604726269 | 379.21755710253 | 0 | Nepal |
| 2012 | 329.63242032006 | 0.372437587888826 | 329.63242032006 | 0 | Nepal |
| 2013 | 305.42299707926 | 0.380022326031393 | 305.42299707926 | 0 | Nepal |
| 2014 | 266.77970446685 | 0.387689088395059 | 266.77970446685 | 0 | Nepal |
| 2015 | 242.58286045342 | 0.395021154474843 | 242.58286045342 | 0 | Nepal |
| 2016 | 218.8543601485 | 0.401628776477432 | 218.8543601485 | 0 | Nepal |
| 2017 | 203.57760074157 | 0.408463874114472 | 203.57760074157 | 0 | Nepal |
| 2018 | 183.30507514022 | 0.41523668446613 | 183.30507514022 | 0 | Nepal |
| 2019 | 172.42433091329 | 0.42200725174098 | 172.42433091329 | 0 | Nepal |
| 2020 | 153.57034185426 | 0.427417089099115 | 153.57034185426 | 0 | Nepal |
| 2021 | 139.20112030705 | 0.43317463481343 | 128.972337333795 | 10.2287829732547 | Nepal |
| 1990 | 3883.04535709999 | 0.310467621430628 | 868.570302494172 | 3014.47505460582 | Pakistan |
| 1991 | 3688.89730163662 | 0.316265666853964 | 784.14981894029 | 2904.74748269633 | Pakistan |
| 1992 | 3528.70580106995 | 0.322248050692924 | 774.622028966716 | 2754.08377210323 | Pakistan |
| 1993 | 3376.8026274216 | 0.328219252463403 | 722.004281828377 | 2654.79834559322 | Pakistan |
| 1994 | 3206.54353383448 | 0.334433128288919 | 656.729418958835 | 2549.81411487564 | Pakistan |
| 1995 | 3037.62372572448 | 0.340926296299194 | 575.502639090749 | 2462.12108663373 | Pakistan |
| 1996 | 2827.48438535468 | 0.347656202917864 | 574.807125942626 | 2252.67725941205 | Pakistan |
| 1997 | 2669.10094360281 | 0.354218627290782 | 554.755280988601 | 2114.34566261421 | Pakistan |
| 1998 | 2509.88968842972 | 0.360875335601641 | 484.290707016253 | 2025.59898141347 | Pakistan |
| 1999 | 2407.79130403136 | 0.367641396836045 | 410.805149741592 | 1996.98615428977 | Pakistan |
| 2000 | 2308.80446206252 | 0.374319132467609 | 359.374631538736 | 1949.42983052378 | Pakistan |
| 2001 | 2225.39693541998 | 0.38059392725045 | 322.514219863672 | 1902.88271555631 | Pakistan |
| 2002 | 2134.85328667297 | 0.386629992356461 | 317.271102078178 | 1817.58218459479 | Pakistan |
| 2003 | 2051.01057759489 | 0.392719262841918 | 284.396799704838 | 1766.61377789005 | Pakistan |
| 2004 | 1981.58245759007 | 0.399084167395961 | 258.183401035388 | 1723.39905655468 | Pakistan |
| 2005 | 1906.80618404186 | 0.40566743657674 | 238.239960669102 | 1668.56622337276 | Pakistan |
| 2006 | 1798.94745496638 | 0.412234830478788 | 216.042308296507 | 1582.90514666987 | Pakistan |
| 2007 | 1685.40683608838 | 0.418519755739044 | 195.798790446151 | 1489.60804564223 | Pakistan |
| 2008 | 1641.07533794247 | 0.424348199605747 | 176.256563172091 | 1464.81877477038 | Pakistan |
| 2009 | 1548.22095092374 | 0.430019832570808 | 138.13206056573 | 1410.08889035801 | Pakistan |
| 2010 | 1428.49769882937 | 0.4356406573129 | 129.105102087713 | 1299.39259674166 | Pakistan |
| 2011 | 1322.971807338 | 0.441455585075092 | 120.268899530923 | 1202.70290780708 | Pakistan |
| 2012 | 1213.87628845681 | 0.447096599297877 | 110.936320920722 | 1102.93996753609 | Pakistan |
| 2013 | 1155.5414771852 | 0.452823095279952 | 104.205828098026 | 1051.33564908717 | Pakistan |
| 2014 | 1084.11322656695 | 0.458781424149631 | 94.4654848324435 | 989.647741734507 | Pakistan |
| 2015 | 1023.73421153655 | 0.464792667642097 | 90.9982732261884 | 932.735938310362 | Pakistan |
| 2016 | 909.93607103742 | 0.471022167867849 | 83.0876811282417 | 826.848389909178 | Pakistan |
| 2017 | 825.60354773905 | 0.4775441366317 | 78.174334851969 | 747.429212887081 | Pakistan |
| 2018 | 731.40401242943 | 0.484326128033838 | 77.2854821055229 | 654.118530323907 | Pakistan |
| 2019 | 659.39062123258 | 0.491022981031026 | 75.1084176654598 | 584.28220356712 | Pakistan |
| 2020 | 512.39897474954 | 0.497364405611904 | 26.617317795252 | 485.781656954288 | Pakistan |
| 2021 | 462.39556638349 | 0.504028688613448 | 16.1378579561026 | 446.257708427387 | Pakistan |
| 1990 | 3965.284350764 | 0.270736935221319 | 1235.28318987928 | 2730.00116088472 | Angola |
| 1991 | 3791.9667731013 | 0.275160624620397 | 1206.03557918036 | 2585.93119392094 | Angola |
| 1992 | 3572.20451517733 | 0.279231499654396 | 1152.84399245113 | 2419.3605227262 | Angola |
| 1993 | 3432.96331989927 | 0.281867702816568 | 1149.49851099854 | 2283.46480890073 | Angola |
| 1994 | 3340.49862598911 | 0.284487594233541 | 1105.44365506542 | 2235.05497092369 | Angola |
| 1995 | 3227.83754164502 | 0.287447813395598 | 1101.76679835561 | 2126.07074328941 | Angola |
| 1996 | 3044.74694618631 | 0.29142127918547 | 1097.30991125212 | 1947.43703493419 | Angola |
| 1997 | 2926.10946988695 | 0.295433993200957 | 1041.2380451892 | 1884.87142469775 | Angola |
| 1998 | 2799.92327278216 | 0.299553353195731 | 987.605686422823 | 1812.31758635934 | Angola |
| 1999 | 2664.29206901144 | 0.303720041599183 | 964.563426744579 | 1699.72864226686 | Angola |
| 2000 | 2540.04554034312 | 0.307603311847111 | 878.995576817557 | 1661.04996352556 | Angola |
| 2001 | 2391.68826401522 | 0.311697534329848 | 869.189512432286 | 1522.49875158293 | Angola |
| 2002 | 2224.08638393462 | 0.316706570519731 | 787.727523483046 | 1436.35886045157 | Angola |
| 2003 | 2048.68161979666 | 0.321777361754333 | 778.70541459057 | 1269.97620520609 | Angola |
| 2004 | 1950.122803508 | 0.32739526565521 | 718.39217016485 | 1231.73063334315 | Angola |
| 2005 | 1761.11607853512 | 0.333785188103559 | 650.973365631699 | 1110.14271290342 | Angola |
| 2006 | 1788.07091348705 | 0.34101262184868 | 566.783303914696 | 1221.28760957235 | Angola |
| 2007 | 1475.98298808995 | 0.34884684076221 | 557.959007589124 | 918.023980500825 | Angola |
| 2008 | 1263.73222423143 | 0.357082005452131 | 504.579368825851 | 759.152855405579 | Angola |
| 2009 | 1094.81882805914 | 0.364407743836554 | 456.6253757834 | 638.19345227574 | Angola |
| 2010 | 975.9826458978 | 0.372033537037443 | 402.448976150342 | 573.533669747458 | Angola |
| 2011 | 867.43240123964 | 0.380006613064486 | 347.533752388218 | 519.898648851422 | Angola |
| 2012 | 751.15991300053 | 0.387891857376226 | 283.376713810159 | 467.783199190371 | Angola |
| 2013 | 640.21758923696 | 0.395796328674416 | 258.347299537677 | 381.870289699283 | Angola |
| 2014 | 518.35296729741 | 0.403720085675527 | 231.669673121336 | 286.683294176074 | Angola |
| 2015 | 453.27076014704 | 0.411372026050491 | 215.874901425057 | 237.395858721983 | Angola |
| 2016 | 400.18895129039 | 0.418822062446251 | 192.985680049799 | 207.203271240591 | Angola |
| 2017 | 362.07675318397 | 0.426177082467176 | 176.834039653596 | 185.242713530374 | Angola |
| 2018 | 322.84191122356 | 0.433355005074473 | 130.671130174391 | 192.170781049169 | Angola |
| 2019 | 294.67145620571 | 0.440454598010708 | 129.480017168221 | 165.191439037489 | Angola |
| 2020 | 254.56111810951 | 0.447283991858922 | 114.135476696598 | 140.425641412912 | Angola |
| 2021 | 227.76700373044 | 0.453721949492174 | 109.044234046783 | 118.722769683657 | Angola |
| 1990 | 2767.58040127652 | 0.216825190875377 | 2321.10735889248 | 446.473042384043 | Central African Republic |
| 1991 | 2714.80425457904 | 0.220191656485842 | 2063.13894740662 | 651.665307172418 | Central African Republic |
| 1992 | 2672.34012974568 | 0.223007541295157 | 2108.58644074509 | 563.753689000589 | Central African Republic |
| 1993 | 2613.67792712121 | 0.225451125286014 | 2076.0138960248 | 537.664031096411 | Central African Republic |
| 1994 | 2531.97738071441 | 0.228487724402372 | 1882.62682424477 | 649.350556469637 | Central African Republic |
| 1995 | 2474.98661473061 | 0.231696792476692 | 1863.94850998154 | 611.038104749069 | Central African Republic |
| 1996 | 2434.70050007207 | 0.233989591180505 | 1884.55783880749 | 550.142661264585 | Central African Republic |
| 1997 | 2426.79660407589 | 0.236671999328608 | 1876.77498925989 | 550.021614815999 | Central African Republic |
| 1998 | 2323.09023875832 | 0.239743528872866 | 1653.13950787901 | 669.950730879307 | Central African Republic |
| 1999 | 2263.18310409035 | 0.242988530190618 | 1629.41324895844 | 633.76985513191 | Central African Republic |
| 2000 | 2209.61389923371 | 0.246021131876903 | 1646.70624655327 | 562.907652680442 | Central African Republic |
| 2001 | 2186.00888271704 | 0.249246740494197 | 1484.86263095263 | 701.146251764407 | Central African Republic |
| 2002 | 2130.35628313864 | 0.252524369440269 | 1524.83186087565 | 605.524422262987 | Central African Republic |
| 2003 | 2074.76870332452 | 0.25510151863237 | 1499.94285888302 | 574.825844441502 | Central African Republic |
| 2004 | 2048.76549061868 | 0.257778778286371 | 1318.99132442221 | 729.774166196466 | Central African Republic |
| 2005 | 1984.624617962 | 0.26045191761313 | 1340.32807122747 | 644.296546734532 | Central African Republic |
| 2006 | 2006.24421732337 | 0.263712446226446 | 1348.5602109852 | 657.684006338171 | Central African Republic |
| 2007 | 2007.42673079118 | 0.2673321946143 | 1204.53350032669 | 802.893230464492 | Central African Republic |
| 2008 | 1982.94087149043 | 0.27115811844024 | 1217.7841868092 | 765.156684681228 | Central African Republic |
| 2009 | 1946.01816667168 | 0.275338515436591 | 1223.28563904426 | 722.732527627415 | Central African Republic |
| 2010 | 1880.11139349155 | 0.279981381762208 | 1169.72628693003 | 710.385106561519 | Central African Republic |
| 2011 | 1787.43131567588 | 0.285097245088929 | 1103.43846250989 | 683.99285316599 | Central African Republic |
| 2012 | 1666.11340381925 | 0.290460023001198 | 1085.76001297535 | 580.353390843899 | Central African Republic |
| 2013 | 1603.75621563049 | 0.290104929568684 | 1094.9666919843 | 508.789523646188 | Central African Republic |
| 2014 | 1570.57450806748 | 0.289999498191724 | 1094.03490562527 | 476.539602442207 | Central African Republic |
| 2015 | 1538.83998793768 | 0.290827871178726 | 1095.88631965021 | 442.953668287472 | Central African Republic |
| 2016 | 1489.92191863202 | 0.29243039953372 | 1031.74945716023 | 458.172461471786 | Central African Republic |
| 2017 | 1443.38774840329 | 0.294752243072343 | 1027.75766184017 | 415.630086563117 | Central African Republic |
| 2018 | 1384.16361856457 | 0.298011223092936 | 992.0951490393 | 392.06846952527 | Central African Republic |
| 2019 | 1311.89843733993 | 0.301953064146936 | 965.854007156262 | 346.044430183668 | Central African Republic |
| 2020 | 1182.20201864461 | 0.305426298180724 | 965.258533072656 | 216.943485571954 | Central African Republic |
| 2021 | 1122.6582466321 | 0.309167689992842 | 874.629536809249 | 248.028709822851 | Central African Republic |
| 1990 | 3251.99550370893 | 0.420654655109748 | 193.790579894401 | 3058.20492381453 | Congo |
| 1991 | 3163.87623369261 | 0.426734373972141 | 179.061472068074 | 2984.81476162454 | Congo |
| 1992 | 3081.45815177451 | 0.431987661930376 | 136.764156537803 | 2944.69399523671 | Congo |
| 1993 | 3014.76562161041 | 0.436606593595936 | 131.07607743318 | 2883.68954417723 | Congo |
| 1994 | 2938.98884085829 | 0.440168346550654 | 128.751234405774 | 2810.23760645252 | Congo |
| 1995 | 2872.50199377318 | 0.443744125258797 | 111.788014258256 | 2760.71397951492 | Congo |
| 1996 | 2824.34624257195 | 0.447566642471598 | 112.234296640741 | 2712.11194593121 | Congo |
| 1997 | 2801.26635677482 | 0.451026961311742 | 104.376318537199 | 2696.89003823762 | Congo |
| 1998 | 2704.61739809463 | 0.454445045209992 | 105.220848819764 | 2599.39654927487 | Congo |
| 1999 | 2578.93968243965 | 0.457176539853177 | 99.679411243118 | 2479.26027119653 | Congo |
| 2000 | 2473.39906798403 | 0.460764813822723 | 92.6115558579897 | 2380.78751212604 | Congo |
| 2001 | 2337.05324658339 | 0.463751682463704 | 90.8452873357156 | 2246.20795924767 | Congo |
| 2002 | 2241.43072597475 | 0.467037267744411 | 85.9203412623369 | 2155.51038471241 | Congo |
| 2003 | 2116.98924801705 | 0.470134373890519 | 82.9050780299173 | 2034.08416998713 | Congo |
| 2004 | 1991.36582266885 | 0.473612435565969 | 80.6298657358545 | 1910.735956933 | Congo |
| 2005 | 1964.31426344342 | 0.478137626987132 | 78.1631860173543 | 1886.15107742607 | Congo |
| 2006 | 1854.93895346709 | 0.483355821647145 | 77.2345468703809 | 1777.70440659671 | Congo |
| 2007 | 1793.08742899964 | 0.48764073952289 | 74.9273471733339 | 1718.16008182631 | Congo |
| 2008 | 1662.85777234085 | 0.492822854998204 | 72.8915662995366 | 1589.96620604131 | Congo |
| 2009 | 1523.61260152633 | 0.498299969081535 | 19.4528302589889 | 1504.15977126734 | Congo |
| 2010 | 1360.04289110868 | 0.505542849123574 | 16.1575422084271 | 1343.88534890025 | Congo |
| 2011 | 1259.74772554511 | 0.513815777196028 | 15.7736211297499 | 1243.97410441536 | Congo |
| 2012 | 1126.61277410992 | 0.522928282789849 | 15.281009708688 | 1111.33176440123 | Congo |
| 2013 | 982.61126643308 | 0.531551678858768 | 15.0642328909733 | 967.547033542107 | Congo |
| 2014 | 812.24619412419 | 0.540594486203119 | 15.0982327713465 | 797.147961352843 | Congo |
| 2015 | 739.30957696083 | 0.548414029703538 | 14.5342452121187 | 724.775331748711 | Congo |
| 2016 | 672.30265893524 | 0.555108607713661 | 12.9177888761224 | 659.384870059118 | Congo |
| 2017 | 608.14821367827 | 0.561834100154124 | 11.5640776393782 | 596.584136038892 | Congo |
| 2018 | 548.16454170779 | 0.567793969630843 | 9.96705274689988 | 538.19748896089 | Congo |
| 2019 | 775.34672533868 | 0.573373665303225 | 8.91625058394333 | 766.430474754737 | Congo |
| 2020 | 469.9556302848 | 0.578451546357964 | 9.1146588818983 | 460.840971402902 | Congo |
| 2021 | 436.12313472266 | 0.583075236262154 | 9.03211544911216 | 427.091019273548 | Congo |
| 1990 | 2692.49421377526 | 0.289843210276958 | 1087.49535560782 | 1604.99885816744 | Democratic Republic of the Congo |
| 1991 | 2664.90226015702 | 0.290431567230931 | 1089.51116850206 | 1575.39109165496 | Democratic Republic of the Congo |
| 1992 | 2594.86035773017 | 0.290959242467126 | 1096.13050226013 | 1498.72985547004 | Democratic Republic of the Congo |
| 1993 | 2590.60129642743 | 0.289628789613637 | 1091.97421112793 | 1498.6270852995 | Democratic Republic of the Congo |
| 1994 | 2878.5944953665 | 0.286859145655148 | 1113.11491196513 | 1765.47958340137 | Democratic Republic of the Congo |
| 1995 | 2611.07276761712 | 0.283967532380546 | 1150.61214637781 | 1460.4606212393 | Democratic Republic of the Congo |
| 1996 | 2658.45217348248 | 0.280413159880167 | 1159.4777623682 | 1498.97441111428 | Democratic Republic of the Congo |
| 1997 | 2706.44713200214 | 0.276336502842209 | 1162.89820886231 | 1543.54892313983 | Democratic Republic of the Congo |
| 1998 | 2806.83827176012 | 0.271813823470493 | 1226.271255817 | 1580.56701594312 | Democratic Republic of the Congo |
| 1999 | 2818.21839140017 | 0.267367662319482 | 1246.50893453804 | 1571.70945686213 | Democratic Republic of the Congo |
| 2000 | 2885.65758096553 | 0.262456746954109 | 1338.17465361389 | 1547.48292735164 | Democratic Republic of the Congo |
| 2001 | 2866.07376368986 | 0.257857504999669 | 1359.86600108522 | 1506.20776260464 | Democratic Republic of the Congo |
| 2002 | 2817.81952930074 | 0.254136360333323 | 1486.49945312695 | 1331.32007617379 | Democratic Republic of the Congo |
| 2003 | 2768.90753557619 | 0.252333884691643 | 1490.42905743414 | 1278.47847814205 | Democratic Republic of the Congo |
| 2004 | 2601.23902174121 | 0.252470918843583 | 1478.0199418128 | 1123.21907992841 | Democratic Republic of the Congo |
| 2005 | 2473.54524106536 | 0.254161023299852 | 1517.7467186598 | 955.798522405561 | Democratic Republic of the Congo |
| 2006 | 2347.77219902224 | 0.257355122656431 | 1369.9779509162 | 977.794248106035 | Democratic Republic of the Congo |
| 2007 | 2221.0508112609 | 0.262272267392268 | 1346.92637398477 | 874.124437276125 | Democratic Republic of the Congo |
| 2008 | 2049.82786557655 | 0.268103872304045 | 1247.24479820117 | 802.583067375381 | Democratic Republic of the Congo |
| 2009 | 1898.11539742812 | 0.274361143997572 | 1218.65462443598 | 679.460772992145 | Democratic Republic of the Congo |
| 2010 | 1728.28767118185 | 0.282223754466439 | 1154.02094313309 | 574.266728048758 | Democratic Republic of the Congo |
| 2011 | 1541.9556275676 | 0.290872953141928 | 1093.22245631533 | 448.733171252274 | Democratic Republic of the Congo |
| 2012 | 1345.40536745263 | 0.300454783216411 | 976.732950056665 | 368.672417395965 | Democratic Republic of the Congo |
| 2013 | 1152.32615117391 | 0.310831256324369 | 868.685805626901 | 283.640345547009 | Democratic Republic of the Congo |
| 2014 | 981.69975439041 | 0.321622927081767 | 776.550994366255 | 205.148760024155 | Democratic Republic of the Congo |
| 2015 | 815.21386609801 | 0.33242130666296 | 646.657289885861 | 168.556576212149 | Democratic Republic of the Congo |
| 2016 | 688.4143763688 | 0.341988787240158 | 568.735408860745 | 119.678967508055 | Democratic Republic of the Congo |
| 2017 | 565.89102367756 | 0.351102035190521 | 552.785862584302 | 13.1051610932579 | Democratic Republic of the Congo |
| 2018 | 500.16760796954 | 0.36028767655131 | 478.419229971484 | 21.7483779980558 | Democratic Republic of the Congo |
| 2019 | 416.44812013119 | 0.36934068409702 | 399.073857384482 | 17.374262746708 | Democratic Republic of the Congo |
| 2020 | 353.96429679282 | 0.376712271159843 | 336.408385920069 | 17.5559108727509 | Democratic Republic of the Congo |
| 2021 | 310.89434299594 | 0.38317984920136 | 307.898129755853 | 2.99621324008666 | Democratic Republic of the Congo |
| 1990 | 5133.7040162689 | 0.268783629292318 | 1214.94800385659 | 3918.75601241231 | Equatorial Guinea |
| 1991 | 4996.44240883332 | 0.275296991793454 | 1210.41017083577 | 3786.03223799755 | Equatorial Guinea |
| 1992 | 4811.31895007212 | 0.283487763833002 | 1151.1929405337 | 3660.12600953843 | Equatorial Guinea |
| 1993 | 4621.50982849433 | 0.292236031708562 | 1036.51104704987 | 3584.99878144446 | Equatorial Guinea |
| 1994 | 4428.37050276286 | 0.30068634012364 | 967.434937642169 | 3460.93556512069 | Equatorial Guinea |
| 1995 | 4253.81877984748 | 0.310443551427585 | 877.490385472403 | 3376.32839437508 | Equatorial Guinea |
| 1996 | 3946.41978419728 | 0.324601220926654 | 726.022092558089 | 3220.39769163919 | Equatorial Guinea |
| 1997 | 3339.04312461301 | 0.350550963012752 | 555.248764829585 | 2783.79435978342 | Equatorial Guinea |
| 1998 | 2873.31155126554 | 0.371237163466133 | 402.496215916822 | 2470.81533534872 | Equatorial Guinea |
| 1999 | 2452.74455210471 | 0.391128022748702 | 284.216602003168 | 2168.52795010154 | Equatorial Guinea |
| 2000 | 2084.8500187149 | 0.411383449802448 | 217.373956580992 | 1867.47606213391 | Equatorial Guinea |
| 2001 | 1658.52334711111 | 0.433590056530441 | 133.887585434025 | 1524.63576167709 | Equatorial Guinea |
| 2002 | 1358.47458132891 | 0.451938999588547 | 107.035611549628 | 1251.43896977928 | Equatorial Guinea |
| 2003 | 1120.10719738249 | 0.467846832610047 | 85.8463605053485 | 1034.26083687714 | Equatorial Guinea |
| 2004 | 942.2944881923 | 0.483879611699357 | 76.9076298244551 | 865.386858367845 | Equatorial Guinea |
| 2005 | 925.95045265528 | 0.498683715051723 | 17.2766116684062 | 908.673840986874 | Equatorial Guinea |
| 2006 | 690.52690146265 | 0.511579828259094 | 15.7353137955633 | 674.791587667087 | Equatorial Guinea |
| 2007 | 583.16421078406 | 0.524076461332969 | 15.0766400599478 | 568.087570724112 | Equatorial Guinea |
| 2008 | 498.32781379612 | 0.537018989870245 | 15.0622352542933 | 483.265578541827 | Equatorial Guinea |
| 2009 | 445.55709614032 | 0.548514682896356 | 14.4716168539621 | 431.085479286358 | Equatorial Guinea |
| 2010 | 389.25377624812 | 0.55910937700551 | 12.0541301212686 | 377.199646126851 | Equatorial Guinea |
| 2011 | 335.10624332064 | 0.570277424014124 | 8.73950639855369 | 326.366736922086 | Equatorial Guinea |
| 2012 | 270.51884007678 | 0.581833899261353 | 8.8775688691524 | 261.641271207628 | Equatorial Guinea |
| 2013 | 218.45710653555 | 0.592800613077549 | 8.86082598654105 | 209.596280549009 | Equatorial Guinea |
| 2014 | 189.86236702448 | 0.60347316716397 | 8.76365714352206 | 181.098709880958 | Equatorial Guinea |
| 2015 | 165.4519054805 | 0.61313462386841 | 8.95679207608667 | 156.495113404413 | Equatorial Guinea |
| 2016 | 156.22496848008 | 0.621858156591175 | 8.91834022970522 | 147.306628250375 | Equatorial Guinea |
| 2017 | 154.02984620314 | 0.630234538276623 | 8.06473268574127 | 145.965113517399 | Equatorial Guinea |
| 2018 | 150.25848794671 | 0.638022822887303 | 6.68799435585013 | 143.57049359086 | Equatorial Guinea |
| 2019 | 145.86540331989 | 0.645303092753852 | 5.75507441657538 | 140.110328903315 | Equatorial Guinea |
| 2020 | 135.78786920577 | 0.652124902941809 | 5.09550543953493 | 130.692363766235 | Equatorial Guinea |
| 2021 | 128.64218151751 | 0.657857455522296 | 4.86404412960462 | 123.778137387905 | Equatorial Guinea |
| 1990 | 2969.03726255664 | 0.455421186671872 | 100.221901831496 | 2868.81536072514 | Gabon |
| 1991 | 2787.22687857536 | 0.46156309767229 | 91.408484901179 | 2695.81839367418 | Gabon |
| 1992 | 2633.31673507093 | 0.467024048058252 | 85.1795516878709 | 2548.13718338306 | Gabon |
| 1993 | 2491.7433216625 | 0.47239446294 | 83.4740895645428 | 2408.26923209796 | Gabon |
| 1994 | 2342.09957450844 | 0.47806194683023 | 78.4447608210648 | 2263.65481368738 | Gabon |
| 1995 | 2193.05105363861 | 0.483826218373876 | 77.3517006374166 | 2115.69935300119 | Gabon |
| 1996 | 2051.49762913922 | 0.48946145974564 | 75.4495445052679 | 1976.04808463395 | Gabon |
| 1997 | 1916.14596847365 | 0.495074161867882 | 71.4349987009077 | 1844.71096977274 | Gabon |
| 1998 | 1811.67430073711 | 0.500372549878702 | 16.5722726116538 | 1795.10202812546 | Gabon |
| 1999 | 1724.82871271227 | 0.504806350324196 | 16.1237699137094 | 1708.70494279856 | Gabon |
| 2000 | 1660.99732225432 | 0.509133087403875 | 15.8844545991965 | 1645.11286765512 | Gabon |
| 2001 | 1555.16887521367 | 0.513045709061385 | 15.7608354708535 | 1539.40803974282 | Gabon |
| 2002 | 1467.45624969178 | 0.516917620976304 | 15.3236105755429 | 1452.13263911624 | Gabon |
| 2003 | 1387.26874065952 | 0.52105141238927 | 15.1771085799562 | 1372.09163207956 | Gabon |
| 2004 | 1331.9427938934 | 0.525479071446482 | 15.1276717757159 | 1316.81512211768 | Gabon |
| 2005 | 1324.14679001008 | 0.530488574568447 | 15.0864325302056 | 1309.06035747987 | Gabon |
| 2006 | 1240.52190317136 | 0.534797715554935 | 15.0812604124702 | 1225.44064275889 | Gabon |
| 2007 | 1156.4895777964 | 0.539441424484129 | 15.0860847166091 | 1141.40349307979 | Gabon |
| 2008 | 1052.00189866263 | 0.544110892340982 | 15.0351579447169 | 1036.96674071791 | Gabon |
| 2009 | 954.33467302606 | 0.548591318536338 | 14.4668944275374 | 939.867778598523 | Gabon |
| 2010 | 847.31265637964 | 0.554022705553671 | 13.1176479827128 | 834.195008396927 | Gabon |
| 2011 | 752.3167179377 | 0.560232082065429 | 12.1857775605418 | 740.130940377158 | Gabon |
| 2012 | 661.60966067041 | 0.566842963853275 | 9.79419574305127 | 651.815464927359 | Gabon |
| 2013 | 618.80050943476 | 0.573808130133551 | 8.84546032676432 | 609.955049107996 | Gabon |
| 2014 | 580.72484038501 | 0.580915656182647 | 8.88354452289744 | 571.841295862113 | Gabon |
| 2015 | 538.37445213451 | 0.58836499960774 | 8.77928786428946 | 529.59516427022 | Gabon |
| 2016 | 490.23631740596 | 0.596062304964046 | 8.9700601868875 | 481.266257219072 | Gabon |
| 2017 | 458.52051327991 | 0.603816251524064 | 8.80827744141056 | 449.712235838499 | Gabon |
| 2018 | 422.57632983355 | 0.611724379630504 | 8.7537769980039 | 413.822552835546 | Gabon |
| 2019 | 380.96194425414 | 0.619904726884927 | 8.79063268759579 | 372.171311566544 | Gabon |
| 2020 | 332.11170361401 | 0.627609647280361 | 8.36227473317053 | 323.74942888084 | Gabon |
| 2021 | 297.63915761988 | 0.634691392755259 | 7.14764416824039 | 290.49151345164 | Gabon |
| 1990 | 6646.63532359477 | 0.205867359800096 | 2585.71777706014 | 4060.91754653463 | Burundi |
| 1991 | 6221.34595614755 | 0.208495720433731 | 2579.795953733 | 3641.55000241455 | Burundi |
| 1992 | 6214.11793125119 | 0.210864619064193 | 2584.1968086084 | 3629.92112264279 | Burundi |
| 1993 | 5985.29637684755 | 0.213249386007653 | 2362.14212798679 | 3623.15424886076 | Burundi |
| 1994 | 5819.1495309514 | 0.215222973163092 | 2343.02290423844 | 3476.12662671296 | Burundi |
| 1995 | 5289.97411993809 | 0.215631675834044 | 2338.65580045872 | 2951.31831947937 | Burundi |
| 1996 | 4907.50575566802 | 0.215526325356923 | 2313.30288052786 | 2594.20287514016 | Burundi |
| 1997 | 4919.42621290551 | 0.215761819839635 | 2312.53375774634 | 2606.89245515917 | Burundi |
| 1998 | 4717.72631952799 | 0.216402603342113 | 2323.23240787274 | 2394.49391165525 | Burundi |
| 1999 | 4448.91200265865 | 0.216855766644471 | 2343.47442598365 | 2105.437576675 | Burundi |
| 2000 | 4286.80438035408 | 0.21686985832205 | 2367.62127743258 | 1919.1831029215 | Burundi |
| 2001 | 4224.30623152552 | 0.216988725649785 | 2320.35455673845 | 1903.95167478707 | Burundi |
| 2002 | 4344.30992849824 | 0.217782476516408 | 2314.73457438504 | 2029.5753541132 | Burundi |
| 2003 | 4346.5902235703 | 0.21896153733539 | 2278.91445626486 | 2067.67576730544 | Burundi |
| 2004 | 4468.54451873855 | 0.220918708918727 | 2072.46731102017 | 2396.07720771838 | Burundi |
| 2005 | 4472.47470882386 | 0.223084371742497 | 2057.20745690224 | 2415.26725192162 | Burundi |
| 2006 | 4368.54946756716 | 0.225821889923957 | 2090.71712381612 | 2277.83234375104 | Burundi |
| 2007 | 4361.48055505036 | 0.228996818715961 | 1891.20181042365 | 2470.2787446267 | Burundi |
| 2008 | 4235.29120152066 | 0.232853551450426 | 1917.23645687121 | 2318.05474464945 | Burundi |
| 2009 | 4041.55990934084 | 0.236943039904587 | 1876.15887480014 | 2165.4010345407 | Burundi |
| 2010 | 3800.54009218388 | 0.241447785964046 | 1668.40514078328 | 2132.1349514006 | Burundi |
| 2011 | 3670.87506838672 | 0.246277277175607 | 1657.89867077245 | 2012.97639761427 | Burundi |
| 2012 | 3461.25413341977 | 0.251572302738587 | 1483.11956821802 | 1978.13456520175 | Burundi |
| 2013 | 2893.93729968416 | 0.257191355215958 | 1363.47873581577 | 1530.45856386839 | Burundi |
| 2014 | 2710.15834230405 | 0.262794721997486 | 1341.91521618286 | 1368.24312612119 | Burundi |
| 2015 | 2475.16632044238 | 0.267327938241439 | 1234.50739925223 | 1240.65892119015 | Burundi |
| 2016 | 2237.55299616022 | 0.271587851896567 | 1247.04322882249 | 990.509767337727 | Burundi |
| 2017 | 2049.99166215859 | 0.275840504541502 | 1148.27696526282 | 901.714696895772 | Burundi |
| 2018 | 1850.67775146862 | 0.279733289674752 | 1172.962659426 | 677.715092042622 | Burundi |
| 2019 | 1712.67933681602 | 0.283442514974853 | 1144.94957115019 | 567.729765665827 | Burundi |
| 2020 | 1596.87886289467 | 0.286496000475545 | 1104.75298922869 | 492.125873665977 | Burundi |
| 2021 | 1508.20009378273 | 0.289374364823265 | 1108.21664146475 | 399.983452317978 | Burundi |
| 1990 | 4748.95849256843 | 0.270048115983623 | 1240.63434035543 | 3508.324152213 | Comoros |
| 1991 | 4503.52040908146 | 0.279145649193181 | 1157.89739200568 | 3345.62301707578 | Comoros |
| 1992 | 4216.21987656152 | 0.288506271423925 | 1101.12431867637 | 3115.09555788515 | Comoros |
| 1993 | 3934.43664888252 | 0.297607416571855 | 1026.44036101593 | 2907.99628786659 | Comoros |
| 1994 | 3680.91158564996 | 0.305706587428224 | 964.13727790081 | 2716.77430774915 | Comoros |
| 1995 | 3389.67667059554 | 0.314220340550933 | 862.623780985697 | 2527.05288960984 | Comoros |
| 1996 | 3159.00082839569 | 0.322249069327522 | 777.247331835229 | 2381.75349656046 | Comoros |
| 1997 | 3040.07352693987 | 0.330148124863673 | 669.516089584124 | 2370.55743735575 | Comoros |
| 1998 | 3631.14570466133 | 0.33751791405714 | 599.633598228184 | 3031.51210643315 | Comoros |
| 1999 | 3053.76041814603 | 0.34477469444905 | 569.77935744496 | 2483.98106070107 | Comoros |
| 2000 | 4277.81594207574 | 0.351875505604714 | 553.444580920578 | 3724.37136115516 | Comoros |
| 2001 | 2683.69122941292 | 0.359211999278107 | 497.918987944556 | 2185.77224146836 | Comoros |
| 2002 | 3308.93739638958 | 0.366486126823935 | 417.620317164839 | 2891.31707922474 | Comoros |
| 2003 | 2422.55184538369 | 0.373410566246042 | 357.146777647717 | 2065.40506773597 | Comoros |
| 2004 | 2307.70372961166 | 0.380035976109197 | 325.050543247457 | 1982.6531863642 | Comoros |
| 2005 | 2232.32334942456 | 0.386891844663173 | 319.260975742214 | 1913.06237368235 | Comoros |
| 2006 | 2186.22569933392 | 0.393521434414153 | 292.239162142622 | 1893.9865371913 | Comoros |
| 2007 | 2722.18567547381 | 0.399051094324319 | 265.498891522146 | 2456.68678395166 | Comoros |
| 2008 | 2164.82827602562 | 0.404649755421042 | 238.271232117733 | 1926.55704390789 | Comoros |
| 2009 | 1977.55368344631 | 0.410253575216193 | 220.498192487968 | 1757.05549095834 | Comoros |
| 2010 | 1749.61748104437 | 0.415543376261308 | 204.711146423438 | 1544.90633462093 | Comoros |
| 2011 | 1658.8463978711 | 0.421092971866532 | 193.807820638186 | 1465.03857723291 | Comoros |
| 2012 | 1484.49847063469 | 0.42672995742339 | 178.649181439736 | 1305.84928919495 | Comoros |
| 2013 | 1329.15814406544 | 0.432789931825945 | 137.319309834412 | 1191.83883423103 | Comoros |
| 2014 | 1354.11341475396 | 0.438844505062188 | 127.123659863575 | 1226.98975489039 | Comoros |
| 2015 | 1250.93474017342 | 0.444712393572096 | 109.813896106253 | 1141.12084406717 | Comoros |
| 2016 | 1143.47868677636 | 0.450506978607507 | 106.91868847122 | 1036.55999830514 | Comoros |
| 2017 | 1083.27552291236 | 0.456280841429385 | 98.7980573251226 | 984.477465587237 | Comoros |
| 2018 | 997.58338124402 | 0.461951343654461 | 91.1303100897424 | 906.453071154277 | Comoros |
| 2019 | 928.26122458109 | 0.46738801351741 | 85.4325812884875 | 842.828643292602 | Comoros |
| 2020 | 912.03781928405 | 0.472003132433584 | 83.0328443409051 | 829.004974943145 | Comoros |
| 2021 | 869.15221075301 | 0.475978688247514 | 77.9119754779921 | 791.240235275018 | Comoros |
| 1990 | 7709.54907905316 | 0.337781788993477 | 600.877159628579 | 7108.67191942458 | Djibouti |
| 1991 | 6901.90779025988 | 0.338770010818962 | 599.428166777282 | 6302.4796234826 | Djibouti |
| 1992 | 6851.31811839385 | 0.340986061300938 | 583.026756426955 | 6268.2913619669 | Djibouti |
| 1993 | 9075.12420272793 | 0.344904393563471 | 573.056765645645 | 8502.06743708229 | Djibouti |
| 1994 | 7141.40052788517 | 0.34798140392569 | 574.294675601801 | 6567.10585228337 | Djibouti |
| 1995 | 6663.59014896874 | 0.351245633337663 | 552.186806033347 | 6111.40334293539 | Djibouti |
| 1996 | 6287.31962427064 | 0.354385634187117 | 554.436922309567 | 5732.88270196107 | Djibouti |
| 1997 | 6403.66047082405 | 0.35809640096391 | 489.010885244762 | 5914.64958557929 | Djibouti |
| 1998 | 5482.02178588563 | 0.361596763593584 | 466.528777704963 | 5015.49300818067 | Djibouti |
| 1999 | 4978.3311207656 | 0.365305211425089 | 423.974410363678 | 4554.35671040192 | Djibouti |
| 2000 | 4720.76092210505 | 0.369015094624057 | 416.632361458693 | 4304.12856064636 | Djibouti |
| 2001 | 4362.63774315141 | 0.372853155297047 | 352.675774517407 | 4009.961968634 | Djibouti |
| 2002 | 4008.96530275709 | 0.37665000401959 | 356.450720883181 | 3652.51458187391 | Djibouti |
| 2003 | 3805.86985820673 | 0.380686615222992 | 328.433056352246 | 3477.43680185448 | Djibouti |
| 2004 | 3660.16549951318 | 0.384987705849076 | 322.198532935108 | 3337.96696657807 | Djibouti |
| 2005 | 3341.80871044934 | 0.389568705689455 | 282.562130148309 | 3059.24658030103 | Djibouti |
| 2006 | 3254.7315455564 | 0.395037470761535 | 263.539887046018 | 2991.19165851038 | Djibouti |
| 2007 | 3093.04582236465 | 0.40061854555971 | 260.122626024724 | 2832.92319633993 | Djibouti |
| 2008 | 2843.51002467896 | 0.406658303036044 | 239.038342843924 | 2604.47168183504 | Djibouti |
| 2009 | 2692.4590640463 | 0.411969334510517 | 217.915367361481 | 2474.54369668482 | Djibouti |
| 2010 | 2783.15963549495 | 0.416999349607211 | 190.554238082673 | 2592.60539741228 | Djibouti |
| 2011 | 2316.31335189382 | 0.422316140090975 | 183.934939381019 | 2132.3784125128 | Djibouti |
| 2012 | 2119.81621119736 | 0.427711440066409 | 164.78253544669 | 1955.03367575067 | Djibouti |
| 2013 | 1982.6328514404 | 0.43336043651961 | 130.510669447547 | 1852.12218199285 | Djibouti |
| 2014 | 1649.5464006223 | 0.439455721647865 | 128.298887807908 | 1521.24751281439 | Djibouti |
| 2015 | 1516.44713796292 | 0.445855284807829 | 112.488536343332 | 1403.95860161959 | Djibouti |
| 2016 | 1425.88505342933 | 0.453442853845541 | 108.871561349556 | 1317.01349207977 | Djibouti |
| 2017 | 1277.07302298222 | 0.460440151860865 | 93.7370063107388 | 1183.33601667148 | Djibouti |
| 2018 | 1115.90569701011 | 0.46770567196373 | 85.6706073673885 | 1030.23508964272 | Djibouti |
| 2019 | 996.5225950257 | 0.475263242846912 | 78.3442357516412 | 918.178359274059 | Djibouti |
| 2020 | 888.03124728555 | 0.481879945321652 | 76.7089216272202 | 811.32232565833 | Djibouti |
| 2021 | 775.37226803327 | 0.4879583707169 | 75.023171305978 | 700.349096727292 | Djibouti |
| 1990 | 11416.5779537804 | 0.216028238900354 | 2333.35852299531 | 9083.21943078508 | Eritrea |
| 1991 | 11088.4117743576 | 0.222574737210924 | 2119.41759788558 | 8968.99417647202 | Eritrea |
| 1992 | 11216.4499247547 | 0.230296065775865 | 1892.06093615613 | 9324.38898859857 | Eritrea |
| 1993 | 10561.3591742232 | 0.239385041260314 | 1677.57425443597 | 8883.78491978723 | Eritrea |
| 1994 | 9409.57113052073 | 0.250752541052101 | 1510.70753637104 | 7898.86359414969 | Eritrea |
| 1995 | 8501.0286202275 | 0.261498484754305 | 1357.17239185332 | 7143.85622837418 | Eritrea |
| 1996 | 7647.70959190293 | 0.272527054525239 | 1220.12541684122 | 6427.58417506171 | Eritrea |
| 1997 | 7041.16821771749 | 0.283551720614883 | 1156.81873215392 | 5884.34948556357 | Eritrea |
| 1998 | 6587.89504829338 | 0.2937648278699 | 1035.13529009142 | 5552.75975820196 | Eritrea |
| 1999 | 6251.72611360317 | 0.303142339840286 | 963.63950369721 | 5288.08660990596 | Eritrea |
| 2000 | 5959.22447771547 | 0.310248161765321 | 873.612345863836 | 5085.61213185163 | Eritrea |
| 2001 | 5864.12908023118 | 0.317247987543295 | 775.912839912008 | 5088.21624031917 | Eritrea |
| 2002 | 5646.73106272645 | 0.323566584840077 | 721.644928882579 | 4925.08613384387 | Eritrea |
| 2003 | 5231.87272393457 | 0.328593580738491 | 722.329969341866 | 4509.5427545927 | Eritrea |
| 2004 | 5044.12158704048 | 0.332879380249727 | 647.690284116356 | 4396.43130292412 | Eritrea |
| 2005 | 4757.32733919162 | 0.336682510811316 | 643.997807096343 | 4113.32953209528 | Eritrea |
| 2006 | 4500.93476498272 | 0.339835823599651 | 599.188264941777 | 3901.74650004094 | Eritrea |
| 2007 | 4287.10222490246 | 0.342843174407113 | 573.945650718015 | 3713.15657418445 | Eritrea |
| 2008 | 4086.84290896619 | 0.343427800730465 | 578.843012868257 | 3507.99989609793 | Eritrea |
| 2009 | 3728.23566095918 | 0.345147742630626 | 572.28797379 | 3155.94768716918 | Eritrea |
| 2010 | 3496.05924260004 | 0.348146176900867 | 576.820868830204 | 2919.23837376984 | Eritrea |
| 2011 | 3197.85743368551 | 0.35416314653227 | 553.956175165049 | 2643.90125852046 | Eritrea |
| 2012 | 2939.62582594413 | 0.360112751410199 | 496.21769718432 | 2443.40812875981 | Eritrea |
| 2013 | 2812.2916827142 | 0.364343653311207 | 457.678536060364 | 2354.61314665384 | Eritrea |
| 2014 | 2311.13130902549 | 0.372527587512835 | 359.069000698469 | 1952.06230832702 | Eritrea |
| 2015 | 2177.39670849392 | 0.376935162282393 | 345.28325239857 | 1832.11345609535 | Eritrea |
| 2016 | 2093.49055253052 | 0.382171665821118 | 323.248114799842 | 1770.24243773068 | Eritrea |
| 2017 | 1966.36672311901 | 0.385936029208135 | 317.661642322918 | 1648.70508079609 | Eritrea |
| 2018 | 1836.50566336278 | 0.39115488426675 | 281.811651128705 | 1554.69401223407 | Eritrea |
| 2019 | 1744.50062381443 | 0.396330558249013 | 267.043539266197 | 1477.45708454823 | Eritrea |
| 2020 | 1637.4788566639 | 0.400459411382762 | 260.299593425259 | 1377.17926323864 | Eritrea |
| 2021 | 1534.77922152578 | 0.403863942828898 | 239.667536603391 | 1295.11168492239 | Eritrea |
| 1990 | 6507.06178095175 | 0.148033884957629 | 4204.63231906929 | 2302.42946188246 | Ethiopia |
| 1991 | 6317.41936550659 | 0.149599710414418 | 4174.05177503725 | 2143.36759046934 | Ethiopia |
| 1992 | 6183.97909145563 | 0.15017438640674 | 4163.2398089634 | 2020.73928249223 | Ethiopia |
| 1993 | 6032.37387330946 | 0.151582364387045 | 4171.91428358982 | 1860.45958971964 | Ethiopia |
| 1994 | 5874.81127551854 | 0.152982636940145 | 4153.79436152055 | 1721.01691399799 | Ethiopia |
| 1995 | 5609.75775650804 | 0.155085604929282 | 4171.70390020202 | 1438.05385630602 | Ethiopia |
| 1996 | 5294.71654536722 | 0.158127229482336 | 4141.2251860609 | 1153.49135930632 | Ethiopia |
| 1997 | 4997.51767039286 | 0.161874742949531 | 4133.78961835433 | 863.728052038527 | Ethiopia |
| 1998 | 4709.36279557027 | 0.16507771008878 | 4132.14468714029 | 577.21810842998 | Ethiopia |
| 1999 | 4404.22366486114 | 0.168903545355995 | 4143.2279144567 | 260.995750404439 | Ethiopia |
| 2000 | 4124.0256880413 | 0.173418529946986 | 4064.86281247121 | 59.1628755700876 | Ethiopia |
| 2001 | 3841.81888780399 | 0.178972968614763 | 3042.57126700234 | 799.247620801646 | Ethiopia |
| 2002 | 3579.64700022254 | 0.184138012848519 | 2887.93180626575 | 691.715193956792 | Ethiopia |
| 2003 | 3283.46808881416 | 0.188694814030626 | 2876.82064560331 | 406.647443210854 | Ethiopia |
| 2004 | 3002.91050773907 | 0.195030701395242 | 2851.96475325217 | 150.9457544869 | Ethiopia |
| 2005 | 2847.7371856957 | 0.202868242862353 | 2746.0914758795 | 101.645709816202 | Ethiopia |
| 2006 | 2699.81352305825 | 0.211524228741913 | 2321.52538546133 | 378.288137596918 | Ethiopia |
| 2007 | 2537.34169908794 | 0.221819930759394 | 2087.44920138926 | 449.892497698683 | Ethiopia |
| 2008 | 2370.21405981486 | 0.232872185647818 | 1896.13218593586 | 474.081873879004 | Ethiopia |
| 2009 | 2218.88054878146 | 0.244265818456334 | 1668.76025752243 | 550.120291259033 | Ethiopia |
| 2010 | 1989.34378141214 | 0.256081459859031 | 1513.68194289688 | 475.661838515257 | Ethiopia |
| 2011 | 1883.76637547949 | 0.267880862080398 | 1239.4419664085 | 644.324409070987 | Ethiopia |
| 2012 | 1738.48323064931 | 0.279006957893266 | 1158.85466010143 | 579.628570547883 | Ethiopia |
| 2013 | 1582.47744663547 | 0.289885266889051 | 1090.86891177334 | 491.608534862133 | Ethiopia |
| 2014 | 1489.24946051117 | 0.300484547961246 | 969.528426353575 | 519.721034157595 | Ethiopia |
| 2015 | 1385.43683586288 | 0.310736418709888 | 865.840685158695 | 519.596150704184 | Ethiopia |
| 2016 | 1297.22585503221 | 0.320431867242816 | 779.298617058987 | 517.927237973222 | Ethiopia |
| 2017 | 1260.39549591785 | 0.329469243719685 | 719.649445774313 | 540.746050143537 | Ethiopia |
| 2018 | 1204.92450888962 | 0.338050074820716 | 608.227641344231 | 596.696867545389 | Ethiopia |
| 2019 | 1132.25486564058 | 0.346422663706934 | 572.705533127278 | 559.549332513302 | Ethiopia |
| 2020 | 1020.51305059622 | 0.353216349837522 | 553.207097443798 | 467.305953152422 | Ethiopia |
| 2021 | 948.31119234637 | 0.358823295416803 | 491.517597082156 | 456.793595264214 | Ethiopia |
| 1990 | 4329.65266156197 | 0.333850293102656 | 651.119916143525 | 3678.53274541844 | Kenya |
| 1991 | 4173.6069806824 | 0.340476220279675 | 570.227865716338 | 3603.37911496606 | Kenya |
| 1992 | 4075.30676156181 | 0.346298636491363 | 573.13365873353 | 3502.17310282828 | Kenya |
| 1993 | 3960.1875773059 | 0.351611769443661 | 553.943613463001 | 3406.2439638429 | Kenya |
| 1994 | 3859.51676138679 | 0.356779193882903 | 553.654289627359 | 3305.86247175943 | Kenya |
| 1995 | 3748.33569609011 | 0.361535806569139 | 462.749114656564 | 3285.58658143355 | Kenya |
| 1996 | 3617.92766828964 | 0.366203076018008 | 427.253154079358 | 3190.67451421028 | Kenya |
| 1997 | 3615.88529631121 | 0.370436464683744 | 404.144144285251 | 3211.74115202596 | Kenya |
| 1998 | 3572.20398957725 | 0.374581400693507 | 355.502032654647 | 3216.7019569226 | Kenya |
| 1999 | 3443.88176944142 | 0.378630009408301 | 347.881272844348 | 3096.00049659707 | Kenya |
| 2000 | 3195.1403379366 | 0.382395616264527 | 324.894051790133 | 2870.24628614647 | Kenya |
| 2001 | 3022.0101028762 | 0.386188414766973 | 321.962455936884 | 2700.04764693932 | Kenya |
| 2002 | 2910.60492273813 | 0.389886256007927 | 286.651621201058 | 2623.95330153707 | Kenya |
| 2003 | 2858.03096104719 | 0.393680479758456 | 282.269937814273 | 2575.76102323292 | Kenya |
| 2004 | 2742.71506913976 | 0.397743651275382 | 263.17131912116 | 2479.5437500186 | Kenya |
| 2005 | 2678.31286339951 | 0.402444011242538 | 236.141586797243 | 2442.17127660227 | Kenya |
| 2006 | 2604.79684606123 | 0.407745764308496 | 237.543063864592 | 2367.25378219664 | Kenya |
| 2007 | 2568.62364507725 | 0.413736227073594 | 217.936340118509 | 2350.68730495874 | Kenya |
| 2008 | 2440.58040514296 | 0.419738146655789 | 194.23800975218 | 2246.34239539078 | Kenya |
| 2009 | 2383.3378118554 | 0.426123888497405 | 177.352107025495 | 2205.9857048299 | Kenya |
| 2010 | 2312.95972699145 | 0.433305839416248 | 131.842865749865 | 2181.11686124158 | Kenya |
| 2011 | 2191.39612649068 | 0.440893830428021 | 126.697467324277 | 2064.6986591664 | Kenya |
| 2012 | 2030.65555071865 | 0.448666395692026 | 106.606146806366 | 1924.04940391228 | Kenya |
| 2013 | 1870.41377239807 | 0.456666941870395 | 100.417270946054 | 1769.99650145202 | Kenya |
| 2014 | 1614.42514994496 | 0.464724302152848 | 89.6564510941503 | 1524.76869885081 | Kenya |
| 2015 | 1471.10149868587 | 0.473084051301145 | 79.911829701465 | 1391.1896689844 | Kenya |
| 2016 | 1338.01681066663 | 0.481709072334755 | 76.3174507941345 | 1261.6993598725 | Kenya |
| 2017 | 1277.05485369189 | 0.490296619363422 | 75.136922701951 | 1201.91793098994 | Kenya |
| 2018 | 1154.7535003765 | 0.499101274473837 | 17.7614482871316 | 1136.99205208937 | Kenya |
| 2019 | 1073.2578664867 | 0.508004210679317 | 15.9740131506937 | 1057.28385333601 | Kenya |
| 2020 | 977.3109433232 | 0.516136571818187 | 15.4885132087729 | 961.822430114427 | Kenya |
| 2021 | 893.04032197954 | 0.523768077100117 | 15.1147119321828 | 877.925610047357 | Kenya |
| 1990 | 8079.56446530509 | 0.279889464787504 | 1164.22684036944 | 6915.33762493565 | Madagascar |
| 1991 | 7809.47304493982 | 0.280429588849502 | 1156.55916618397 | 6652.91387875585 | Madagascar |
| 1992 | 7580.41588411117 | 0.280501282838033 | 1151.05136741585 | 6429.36451669532 | Madagascar |
| 1993 | 7374.40071586646 | 0.280204299153331 | 1155.84881065485 | 6218.55190521161 | Madagascar |
| 1994 | 7176.91990611513 | 0.279615149051304 | 1154.57987769264 | 6022.34002842249 | Madagascar |
| 1995 | 6973.10900231792 | 0.279164003276889 | 1173.4225958508 | 5799.68640646712 | Madagascar |
| 1996 | 6769.61591522497 | 0.279066492612967 | 1156.01619885334 | 5613.59971637163 | Madagascar |
| 1997 | 6561.63084383202 | 0.279426189949827 | 1156.4020032996 | 5405.22884053242 | Madagascar |
| 1998 | 6272.06313529326 | 0.280317670990881 | 1155.66099548646 | 5116.4021398068 | Madagascar |
| 1999 | 6306.00474309606 | 0.281571072098772 | 1150.2012747133 | 5155.80346838276 | Madagascar |
| 2000 | 5862.0924235338 | 0.283190720794041 | 1158.75416718797 | 4703.33825634583 | Madagascar |
| 2001 | 5690.61257486403 | 0.285668180077333 | 1112.99400939036 | 4577.61856547367 | Madagascar |
| 2002 | 5390.86134738247 | 0.287153148772741 | 1110.66293714768 | 4280.19841023479 | Madagascar |
| 2003 | 5208.80328131112 | 0.289675457328935 | 1098.64011763061 | 4110.16316368051 | Madagascar |
| 2004 | 5050.09265117184 | 0.292629884521451 | 1026.8451070712 | 4023.24754410064 | Madagascar |
| 2005 | 5291.2678693899 | 0.295919326698766 | 1036.47937806532 | 4254.78849132458 | Madagascar |
| 2006 | 5158.01059784519 | 0.299723392356893 | 970.766379892317 | 4187.24421795287 | Madagascar |
| 2007 | 5046.7909678014 | 0.304026244970223 | 964.436462916575 | 4082.35450488483 | Madagascar |
| 2008 | 4960.19626941841 | 0.308990264960967 | 872.341487699646 | 4087.85478171876 | Madagascar |
| 2009 | 4805.05847004292 | 0.313450891076854 | 863.666183745017 | 3941.3922862979 | Madagascar |
| 2010 | 4521.13687539213 | 0.318353184360276 | 769.069394560344 | 3752.06748083179 | Madagascar |
| 2011 | 4523.53370050879 | 0.323801042883395 | 714.697247974046 | 3808.83645253474 | Madagascar |
| 2012 | 4447.85743355496 | 0.329947540970422 | 668.282648421347 | 3779.57478513361 | Madagascar |
| 2013 | 4249.67671131496 | 0.336519231511991 | 648.423494012454 | 3601.25321730251 | Madagascar |
| 2014 | 3953.11264289154 | 0.343352610546557 | 570.667584751138 | 3382.4450581404 | Madagascar |
| 2015 | 3743.16762799946 | 0.350443595243704 | 561.974404043856 | 3181.1932239556 | Madagascar |
| 2016 | 3532.33528751552 | 0.35802634126176 | 492.404551632692 | 3039.93073588283 | Madagascar |
| 2017 | 3307.82027537982 | 0.36623558721941 | 418.951844508689 | 2888.86843087113 | Madagascar |
| 2018 | 3067.81364072706 | 0.3749696444566 | 353.741327412206 | 2714.07231331485 | Madagascar |
| 2019 | 2775.83670534991 | 0.384209195656002 | 320.791728200598 | 2455.04497714931 | Madagascar |
| 2020 | 2992.38778446 | 0.392778708989164 | 283.965929338935 | 2708.42185512107 | Madagascar |
| 2021 | 2756.64766330579 | 0.40024694263784 | 263.01358087452 | 2493.63408243127 | Madagascar |
| 1990 | 15726.3509449458 | 0.204010244634822 | 2593.01966470785 | 13133.331280238 | Malawi |
| 1991 | 15274.4905716122 | 0.206043785604482 | 2570.50622986909 | 12703.9843417431 | Malawi |
| 1992 | 14468.1005465872 | 0.206732358761786 | 2577.20134830117 | 11890.899198286 | Malawi |
| 1993 | 13982.1003408694 | 0.20809120460636 | 2612.4559129714 | 11369.644427898 | Malawi |
| 1994 | 12849.0198031258 | 0.209144565934722 | 2597.89096366999 | 10251.1288394558 | Malawi |
| 1995 | 12300.2412544851 | 0.211756231927772 | 2382.91799647462 | 9917.32325801048 | Malawi |
| 1996 | 11292.2364927361 | 0.215264474919532 | 2330.08736136581 | 8962.14913137029 | Malawi |
| 1997 | 10158.7250819763 | 0.219416251726227 | 2296.47382277805 | 7862.25125919825 | Malawi |
| 1998 | 9262.93368186601 | 0.223122329682106 | 2090.64905264348 | 7172.28462922253 | Malawi |
| 1999 | 9480.83302123509 | 0.22706836834043 | 2060.87338051639 | 7419.9596407187 | Malawi |
| 2000 | 8589.05349313523 | 0.230900810051145 | 1908.07850086762 | 6680.97499226761 | Malawi |
| 2001 | 8510.7928505428 | 0.234020506815413 | 1859.57667068893 | 6651.21617985387 | Malawi |
| 2002 | 7657.39197446777 | 0.237602155431587 | 1867.13875779983 | 5790.25321666794 | Malawi |
| 2003 | 6826.93771681697 | 0.241559227766521 | 1651.12781307063 | 5175.80990374634 | Malawi |
| 2004 | 6166.48903365816 | 0.246434539760473 | 1655.55592681835 | 4510.93310683981 | Malawi |
| 2005 | 5632.772522598 | 0.251672317552778 | 1506.57076397961 | 4126.20175861839 | Malawi |
| 2006 | 5355.00563301732 | 0.257595649191555 | 1354.75985578798 | 4000.24577722934 | Malawi |
| 2007 | 4935.64665128944 | 0.265226927963354 | 1347.31786920494 | 3588.3287820845 | Malawi |
| 2008 | 4479.55463305988 | 0.273689856434299 | 1224.02469345156 | 3255.52993960832 | Malawi |
| 2009 | 4349.40217717134 | 0.282296508700983 | 1157.8148135146 | 3191.58736365674 | Malawi |
| 2010 | 4145.15002586836 | 0.291179619969706 | 1093.39623838123 | 3051.75378748713 | Malawi |
| 2011 | 3991.04695469508 | 0.300448509120473 | 968.790843917205 | 3022.25611077787 | Malawi |
| 2012 | 3434.79122546224 | 0.30919762412021 | 869.946643432205 | 2564.84458203003 | Malawi |
| 2013 | 3049.98677876827 | 0.317936213061346 | 777.840436759104 | 2272.14634200917 | Malawi |
| 2014 | 2792.63137450463 | 0.327042425505685 | 720.744954856234 | 2071.8864196484 | Malawi |
| 2015 | 2579.77422550206 | 0.33614013833142 | 649.96666671911 | 1929.80755878295 | Malawi |
| 2016 | 2392.12133190275 | 0.344731541741046 | 579.293844024948 | 1812.8274878778 | Malawi |
| 2017 | 2200.33115606002 | 0.353121067414073 | 553.293163219372 | 1647.03799284065 | Malawi |
| 2018 | 2060.2689715469 | 0.361470410425433 | 465.391366541079 | 1594.87760500582 | Malawi |
| 2019 | 1931.87250720402 | 0.37011460502829 | 407.250042242733 | 1524.62246496129 | Malawi |
| 2020 | 1748.74549367348 | 0.377879020413869 | 345.92071722899 | 1402.82477644449 | Malawi |
| 2021 | 1535.69364773509 | 0.384553633730301 | 318.006254334163 | 1217.68739340093 | Malawi |
| 1990 | 737.63666311878 | 0.544586533153919 | 15.0529114028223 | 722.583751715958 | Mauritius |
| 1991 | 514.53660750727 | 0.548209120513745 | 14.4676876570167 | 500.068919850253 | Mauritius |
| 1992 | 487.19195411288 | 0.55345463047762 | 13.8406462832771 | 473.351307829603 | Mauritius |
| 1993 | 423.46426244026 | 0.560688639662726 | 12.2676925651828 | 411.196569875077 | Mauritius |
| 1994 | 430.36433457687 | 0.569092171296203 | 9.57039690771682 | 420.793937669153 | Mauritius |
| 1995 | 406.51169610923 | 0.577319390278784 | 9.06991280836139 | 397.441783300869 | Mauritius |
| 1996 | 400.92660229496 | 0.584797060785555 | 8.89777217636977 | 392.02883011859 | Mauritius |
| 1997 | 325.89592207766 | 0.591497734589023 | 8.87251715104949 | 317.02340492661 | Mauritius |
| 1998 | 321.12083299017 | 0.597363039395481 | 8.76217813269671 | 312.358654857473 | Mauritius |
| 1999 | 340.68972706 | 0.602034233757503 | 8.79554806652418 | 331.894178993476 | Mauritius |
| 2000 | 322.93823494534 | 0.6070879010983 | 8.94417628203299 | 313.994058663307 | Mauritius |
| 2001 | 313.90182003843 | 0.612240817263126 | 8.85150019567 | 305.05031984276 | Mauritius |
| 2002 | 308.95451822144 | 0.617190006807373 | 8.88809699386093 | 300.066421227579 | Mauritius |
| 2003 | 284.25914590254 | 0.622764532968127 | 9.01081872157216 | 275.248327180968 | Mauritius |
| 2004 | 301.30477087209 | 0.629030192646747 | 8.2409467485303 | 293.06382412356 | Mauritius |
| 2005 | 289.11199005553 | 0.634766579912803 | 6.94566657686944 | 282.166323478661 | Mauritius |
| 2006 | 323.72092842764 | 0.639545182683383 | 6.59660909442472 | 317.124319333215 | Mauritius |
| 2007 | 351.0998615703 | 0.64435865560727 | 6.02390048345153 | 345.075961086848 | Mauritius |
| 2008 | 351.40765562587 | 0.649630852006361 | 5.08991562083483 | 346.317740005035 | Mauritius |
| 2009 | 336.38372979001 | 0.655000295441282 | 5.03530738309481 | 331.348422406915 | Mauritius |
| 2010 | 295.92220972304 | 0.660469800171028 | 4.55514113435433 | 291.367068588686 | Mauritius |
| 2011 | 293.7595998892 | 0.66649762677449 | 3.95001875852293 | 289.809581130677 | Mauritius |
| 2012 | 298.60978737737 | 0.673127650407144 | 3.7688627834059 | 294.840924593964 | Mauritius |
| 2013 | 294.49344793349 | 0.680114023433391 | 3.60991170870397 | 290.883536224786 | Mauritius |
| 2014 | 275.40925653977 | 0.686616503167309 | 3.36596716722651 | 272.043289372543 | Mauritius |
| 2015 | 272.89263231617 | 0.692239729639954 | 3.2262988263692 | 269.666333489801 | Mauritius |
| 2016 | 269.19165973155 | 0.697020562490311 | 3.13122161153725 | 266.060438120013 | Mauritius |
| 2017 | 267.68734256136 | 0.70161813647985 | 3.04735136690542 | 264.639991194455 | Mauritius |
| 2018 | 282.66532432856 | 0.706266650972885 | 2.84448928524976 | 279.82083504331 | Mauritius |
| 2019 | 259.91786091663 | 0.710935044634248 | 2.7666835467722 | 257.151177369858 | Mauritius |
| 2020 | 233.61483645948 | 0.714629803881546 | 2.69363104032195 | 230.921205419158 | Mauritius |
| 2021 | 214.03853864321 | 0.718260445552653 | 2.53943520564143 | 211.499103437569 | Mauritius |
| 1990 | 11012.2447665357 | 0.173064715394479 | 4176.19621234183 | 6836.04855419387 | Mozambique |
| 1991 | 10227.4219947758 | 0.175550435322203 | 3488.9370371534 | 6738.48495762239 | Mozambique |
| 1992 | 10113.00174149 | 0.17728518828037 | 3137.90043581579 | 6975.10130567421 | Mozambique |
| 1993 | 9863.47362062377 | 0.179851807348523 | 3028.21806112705 | 6835.25555949672 | Mozambique |
| 1994 | 9229.64542418835 | 0.182236860582392 | 3017.56774136193 | 6212.07768282642 | Mozambique |
| 1995 | 8817.75428097432 | 0.183427981878199 | 3017.08382392819 | 5800.67045704613 | Mozambique |
| 1996 | 8492.82525194627 | 0.185705869370502 | 2902.01153936668 | 5590.81371257959 | Mozambique |
| 1997 | 8356.60873802224 | 0.188650435754761 | 2893.44688458997 | 5463.16185343227 | Mozambique |
| 1998 | 8151.78459793776 | 0.191998629373624 | 2856.38614109771 | 5295.39845684005 | Mozambique |
| 1999 | 8091.32272943819 | 0.195435287798378 | 2834.40770468916 | 5256.91502474903 | Mozambique |
| 2000 | 7253.17880856886 | 0.198534396867784 | 2758.56994655572 | 4494.60886201314 | Mozambique |
| 2001 | 6609.88001209356 | 0.20224192395185 | 2744.80928994491 | 3865.07072214865 | Mozambique |
| 2002 | 6102.8840198036 | 0.206390059635981 | 2570.3283015095 | 3532.5557182941 | Mozambique |
| 2003 | 5665.53813282981 | 0.210534489558039 | 2581.95098371876 | 3083.58714911105 | Mozambique |
| 2004 | 5257.53061003175 | 0.214972457474336 | 2322.55690332568 | 2934.97370670608 | Mozambique |
| 2005 | 4850.29205400417 | 0.219692372119565 | 2064.34393821458 | 2785.94811578959 | Mozambique |
| 2006 | 4838.66278420255 | 0.22475808709223 | 2094.33589243669 | 2744.32689176586 | Mozambique |
| 2007 | 4400.66563257398 | 0.230158226784202 | 1884.0638076184 | 2516.60182495558 | Mozambique |
| 2008 | 4018.35349361641 | 0.235790170219554 | 1881.40929535207 | 2136.94419826434 | Mozambique |
| 2009 | 3652.47049806948 | 0.241360656275235 | 1654.4268119217 | 1998.04368614778 | Mozambique |
| 2010 | 3248.18712130229 | 0.246751499854601 | 1649.20847695653 | 1598.97864434576 | Mozambique |
| 2011 | 2886.82462730954 | 0.252232471060626 | 1493.74433763747 | 1393.08028967207 | Mozambique |
| 2012 | 2732.54693734426 | 0.258315562328104 | 1337.58552014892 | 1394.96141719534 | Mozambique |
| 2013 | 2538.6567468553 | 0.265280462139272 | 1324.66613070485 | 1213.99061615045 | Mozambique |
| 2014 | 2395.32992007376 | 0.272892813606321 | 1228.53531498452 | 1166.79460508924 | Mozambique |
| 2015 | 2014.12073252673 | 0.2810326852532 | 1160.01604001793 | 854.104692508796 | Mozambique |
| 2016 | 1853.57577275146 | 0.289152424860155 | 1105.95925604204 | 747.61651670942 | Mozambique |
| 2017 | 1719.82235175081 | 0.297164772494457 | 1037.39412803928 | 682.42822371153 | Mozambique |
| 2018 | 1637.05172344873 | 0.305226771938642 | 962.754099565275 | 674.297623883455 | Mozambique |
| 2019 | 1645.74017905223 | 0.313174508096083 | 862.642890693383 | 783.097288358847 | Mozambique |
| 2020 | 1498.9146281763 | 0.320291181995807 | 769.464727514164 | 729.449900662136 | Mozambique |
| 2021 | 1363.43771614489 | 0.326462613636377 | 717.643594010865 | 645.794122134025 | Mozambique |
| 1990 | 7658.98779003167 | 0.275097189589071 | 1234.745136437 | 6424.24265359467 | Rwanda |
| 1991 | 7784.01781661251 | 0.276194698465909 | 1150.19087085905 | 6633.82694575346 | Rwanda |
| 1992 | 7404.33101629534 | 0.277178171463842 | 1144.32675553364 | 6260.0042607617 | Rwanda |
| 1993 | 6557.93776151744 | 0.276980133306619 | 1158.37082350956 | 5399.56693800789 | Rwanda |
| 1994 | 19278.1096328296 | 0.272331257765128 | 1225.72399723134 | 18052.3856355983 | Rwanda |
| 1995 | 8715.02239242165 | 0.271102226876129 | 1242.72999469483 | 7472.29239772682 | Rwanda |
| 1996 | 8359.48859258244 | 0.271097922261967 | 1222.95139811665 | 7136.53719446579 | Rwanda |
| 1997 | 7288.75946199876 | 0.272455860850458 | 1241.06067124304 | 6047.69879075572 | Rwanda |
| 1998 | 6874.48470848657 | 0.274582550589084 | 1220.52688290414 | 5653.95782558243 | Rwanda |
| 1999 | 6684.88553570436 | 0.277014387103527 | 1159.89914024602 | 5524.98639545834 | Rwanda |
| 2000 | 6454.81252130614 | 0.280983454342867 | 1153.3665380302 | 5301.44598327594 | Rwanda |
| 2001 | 6138.07181355619 | 0.286082054881284 | 1101.23659022616 | 5036.83522333003 | Rwanda |
| 2002 | 5824.06879485471 | 0.292955430049408 | 1034.45043331557 | 4789.61836153914 | Rwanda |
| 2003 | 5472.20557836705 | 0.299676918389748 | 977.132920112594 | 4495.07265825446 | Rwanda |
| 2004 | 5504.19625528029 | 0.306943916198519 | 893.700947538489 | 4610.4953077418 | Rwanda |
| 2005 | 5132.32104206364 | 0.314219577820047 | 861.894617244438 | 4270.4264248192 | Rwanda |
| 2006 | 4742.64402622908 | 0.321998519469469 | 777.814650579551 | 3964.82937564953 | Rwanda |
| 2007 | 4272.87305217206 | 0.329616590545695 | 718.549889807897 | 3554.32316236416 | Rwanda |
| 2008 | 3836.53081158939 | 0.337039226343398 | 645.861300278967 | 3190.66951131042 | Rwanda |
| 2009 | 3344.14407892431 | 0.344494632549039 | 572.011239114786 | 2772.13283980952 | Rwanda |
| 2010 | 3056.06086030365 | 0.351659415720748 | 555.126212314632 | 2500.93464798902 | Rwanda |
| 2011 | 2806.82182556283 | 0.358940339871084 | 507.21276390791 | 2299.60906165492 | Rwanda |
| 2012 | 2307.17442313618 | 0.366411820814214 | 424.444330267413 | 1882.73009286877 | Rwanda |
| 2013 | 2041.58892165042 | 0.373769682430802 | 348.016868953279 | 1693.57205269714 | Rwanda |
| 2014 | 2027.73888807336 | 0.381533179436292 | 325.134628890151 | 1702.60425918321 | Rwanda |
| 2015 | 1935.5896263319 | 0.389895414951459 | 285.9625206087 | 1649.6271057232 | Rwanda |
| 2016 | 1785.05687052043 | 0.39828795853881 | 264.452108637489 | 1520.60476188294 | Rwanda |
| 2017 | 1550.16000420904 | 0.406411474976626 | 241.27329662872 | 1308.88670758032 | Rwanda |
| 2018 | 1403.73047403077 | 0.414657911701104 | 214.564880283204 | 1189.16559374757 | Rwanda |
| 2019 | 1318.85392869352 | 0.42308672715334 | 182.601713796251 | 1136.25221489727 | Rwanda |
| 2020 | 1256.06106976417 | 0.429896595971404 | 135.950365500759 | 1120.11070426341 | Rwanda |
| 2021 | 1146.23835770861 | 0.435588705789772 | 128.749698477217 | 1017.48865923139 | Rwanda |
| 1990 | 230.20693556605 | 0.575526495579186 | 8.98688251321483 | 221.220053052835 | Seychelles |
| 1991 | 219.48510024122 | 0.582698331394814 | 8.75364782993893 | 210.731452411281 | Seychelles |
| 1992 | 209.0990560451 | 0.590052918893135 | 8.88514385395989 | 200.21391219114 | Seychelles |
| 1993 | 201.60583899212 | 0.598044193906834 | 8.80112173779962 | 192.80471725432 | Seychelles |
| 1994 | 189.25041807973 | 0.606041267633362 | 8.88536955066 | 180.36504852907 | Seychelles |
| 1995 | 181.26629771141 | 0.613853280633323 | 8.75932319860123 | 172.506974512809 | Seychelles |
| 1996 | 171.85660320187 | 0.622135170108466 | 8.8089093303832 | 163.047693871487 | Seychelles |
| 1997 | 162.61516424889 | 0.630697731713198 | 8.29032771825404 | 154.324836530636 | Seychelles |
| 1998 | 158.11481581229 | 0.63843888230513 | 6.78783133139158 | 151.326984480898 | Seychelles |
| 1999 | 157.65254805184 | 0.645339665687648 | 6.05681518586622 | 151.595732865974 | Seychelles |
| 2000 | 157.28344948666 | 0.651551988453137 | 4.93481678950096 | 152.348632697159 | Seychelles |
| 2001 | 155.62428325545 | 0.656490668328199 | 4.87568774126242 | 150.748595514188 | Seychelles |
| 2002 | 154.5959119247 | 0.660907789985091 | 4.25592330016699 | 150.339988624533 | Seychelles |
| 2003 | 153.51529307441 | 0.664162026621557 | 4.2840535645518 | 149.231239509858 | Seychelles |
| 2004 | 154.02359991876 | 0.666461353259832 | 4.00502444399582 | 150.018575474764 | Seychelles |
| 2005 | 153.29318099341 | 0.669264742237803 | 3.83239721697271 | 149.460783776437 | Seychelles |
| 2006 | 151.26767587266 | 0.672149914351772 | 3.77128239651122 | 147.496393476149 | Seychelles |
| 2007 | 285.53932131565 | 0.675077437552466 | 3.59400438060867 | 281.945316935041 | Seychelles |
| 2008 | 146.55982988453 | 0.676590946064689 | 3.59665569575612 | 142.963174188774 | Seychelles |
| 2009 | 144.28372515793 | 0.67784702810111 | 3.6032507317631 | 140.680474426167 | Seychelles |
| 2010 | 143.15236091167 | 0.680163639820996 | 3.59938996789797 | 139.552970943772 | Seychelles |
| 2011 | 140.94028874914 | 0.683681491395532 | 3.37440956960099 | 137.565879179539 | Seychelles |
| 2012 | 138.88771119505 | 0.687629394362418 | 3.36379707626078 | 135.523914118789 | Seychelles |
| 2013 | 135.83590696931 | 0.692525733840497 | 3.12118279760042 | 132.71472417171 | Seychelles |
| 2014 | 132.14244213786 | 0.697114588650508 | 3.16800391199829 | 128.974438225862 | Seychelles |
| 2015 | 128.23704385709 | 0.701722819856481 | 2.91086517290037 | 125.32617868419 | Seychelles |
| 2016 | 125.25204594845 | 0.706183622956323 | 3.00901232997405 | 122.243033618476 | Seychelles |
| 2017 | 105.54903128635 | 0.711027281077533 | 2.69064929911152 | 102.858381987238 | Seychelles |
| 2018 | 102.67046007101 | 0.716407819415594 | 2.49831489123241 | 100.172145179778 | Seychelles |
| 2019 | 99.45610464632 | 0.722073684746157 | 2.4315539183387 | 97.0245507279813 | Seychelles |
| 2020 | 95.68456254533 | 0.726663769455011 | 2.36475162281394 | 93.319810922516 | Seychelles |
| 2021 | 94.62534289619 | 0.730150774629382 | 2.26420433702 | 92.36113855917 | Seychelles |
| 1990 | 13357.8596519749 | 0.0488485644444749 | 13357.8596519749 | 0 | Somalia |
| 1991 | 13325.8867071325 | 0.0494223815231112 | 13325.8867071325 | 0 | Somalia |
| 1992 | 13620.9666500739 | 0.050115531337966 | 13381.4374706724 | 239.529179401454 | Somalia |
| 1993 | 13528.7379235053 | 0.0507979992557536 | 13371.4138404293 | 157.324083075975 | Somalia |
| 1994 | 14183.1534352972 | 0.0514716956452511 | 13415.7854163344 | 767.368018962825 | Somalia |
| 1995 | 12490.2154396497 | 0.052148606291336 | 12490.2154396497 | 0 | Somalia |
| 1996 | 11964.1416421937 | 0.052840548398447 | 11964.1416421937 | 0 | Somalia |
| 1997 | 11721.8342190716 | 0.0535709673179049 | 11721.8342190716 | 0 | Somalia |
| 1998 | 11429.5102575907 | 0.054321526804773 | 11429.5102575907 | 0 | Somalia |
| 1999 | 10931.5346801336 | 0.0550718380312836 | 10931.5346801336 | 0 | Somalia |
| 2000 | 10967.985626879 | 0.0558420632221002 | 10950.5195482302 | 17.4660786488384 | Somalia |
| 2001 | 10018.5129952058 | 0.0566431646698027 | 10018.5129952058 | 0 | Somalia |
| 2002 | 9377.89189753807 | 0.0574576117178968 | 9377.89189753807 | 0 | Somalia |
| 2003 | 8765.54138929218 | 0.0582721495971463 | 8765.54138929218 | 0 | Somalia |
| 2004 | 8177.90081518909 | 0.059076945378295 | 8177.90081518909 | 0 | Somalia |
| 2005 | 7602.82395161977 | 0.0598985393277897 | 7602.82395161977 | 0 | Somalia |
| 2006 | 7208.15338223626 | 0.0607414841530955 | 7208.15338223626 | 0 | Somalia |
| 2007 | 7825.42687906651 | 0.0615981512915218 | 7341.48340661006 | 483.94347245645 | Somalia |
| 2008 | 7216.61070478292 | 0.062475386934885 | 7210.66545824022 | 5.94524654270208 | Somalia |
| 2009 | 6817.97653608136 | 0.0633822252221486 | 6817.97653608136 | 0 | Somalia |
| 2010 | 6447.49094900497 | 0.0643221275495221 | 6447.49094900497 | 0 | Somalia |
| 2011 | 6573.10788158763 | 0.065306811339105 | 6496.16751038075 | 76.9403712068788 | Somalia |
| 2012 | 6204.03723013474 | 0.0663548789687034 | 6204.03723013474 | 0 | Somalia |
| 2013 | 6070.96345189094 | 0.0674594862548888 | 6070.96345189094 | 0 | Somalia |
| 2014 | 5815.44857697019 | 0.0686092985197366 | 5815.44857697019 | 0 | Somalia |
| 2015 | 5508.8860736313 | 0.0698115214335177 | 5508.8860736313 | 0 | Somalia |
| 2016 | 5471.52416079646 | 0.0710640470412588 | 5471.52416079646 | 0 | Somalia |
| 2017 | 5003.12831331984 | 0.0723347759051063 | 5003.12831331984 | 0 | Somalia |
| 2018 | 4712.06488557853 | 0.0736422483519594 | 4712.06488557853 | 0 | Somalia |
| 2019 | 4645.43872951498 | 0.0749798109966195 | 4645.43872951498 | 0 | Somalia |
| 2020 | 4379.20127557297 | 0.0763633695596333 | 4379.20127557297 | 0 | Somalia |
| 2021 | 4048.42868036841 | 0.0776881089723748 | 4048.42868036841 | 0 | Somalia |
| 1990 | 10000.4236156548 | 0.259306073677984 | 1353.16884936334 | 8647.25476629146 | United Republic of Tanzania |
| 1991 | 9743.5972759938 | 0.262416238461239 | 1350.74873777468 | 8392.84853821912 | United Republic of Tanzania |
| 1992 | 10326.7382291242 | 0.264989447606401 | 1324.42851493563 | 9002.30971418857 | United Republic of Tanzania |
| 1993 | 9260.35081262474 | 0.26725491530928 | 1225.43013039842 | 8034.92068222632 | United Republic of Tanzania |
| 1994 | 9114.66121182077 | 0.269417204337003 | 1256.65404884585 | 7858.00716297492 | United Republic of Tanzania |
| 1995 | 8783.12796807793 | 0.27207820236791 | 1230.65090135061 | 7552.47706672732 | United Republic of Tanzania |
| 1996 | 8363.35159135837 | 0.2755079091009 | 1240.28568410912 | 7123.06590724925 | United Republic of Tanzania |
| 1997 | 8750.74481037789 | 0.279259183750542 | 1147.78942389954 | 7602.95538647835 | United Republic of Tanzania |
| 1998 | 8078.59439876384 | 0.283152610359192 | 1157.17390844872 | 6921.42049031512 | United Republic of Tanzania |
| 1999 | 7067.94129831531 | 0.287381679042618 | 1109.40529946583 | 5958.53599884948 | United Republic of Tanzania |
| 2000 | 6341.29643555085 | 0.291827264752142 | 1096.80282375307 | 5244.49361179778 | United Republic of Tanzania |
| 2001 | 5838.27480729223 | 0.296715645317522 | 1031.5986645831 | 4806.67614270914 | United Republic of Tanzania |
| 2002 | 5364.87026776207 | 0.301983995351723 | 957.977790399083 | 4406.89247736299 | United Republic of Tanzania |
| 2003 | 4801.48880324918 | 0.307615488417032 | 874.984589421995 | 3926.50421382719 | United Republic of Tanzania |
| 2004 | 4370.8279540496 | 0.313772983484529 | 867.621850666354 | 3503.20610338325 | United Republic of Tanzania |
| 2005 | 3947.17921373566 | 0.320437339078038 | 774.958463148923 | 3172.22075058674 | United Republic of Tanzania |
| 2006 | 3834.30363946167 | 0.327234001890678 | 718.189872711333 | 3116.11376675034 | United Republic of Tanzania |
| 2007 | 3771.89676202336 | 0.334313236676657 | 646.286391152306 | 3125.61037087105 | United Republic of Tanzania |
| 2008 | 3712.15339188483 | 0.341352630337404 | 571.96264702967 | 3140.19074485516 | United Republic of Tanzania |
| 2009 | 3686.4519410272 | 0.348378034950913 | 572.354331895971 | 3114.09760913123 | United Republic of Tanzania |
| 2010 | 3522.08381283226 | 0.355567563218872 | 553.746347811708 | 2968.33746502055 | United Republic of Tanzania |
| 2011 | 3345.35179986777 | 0.363036319968136 | 464.856306697675 | 2880.4954931701 | United Republic of Tanzania |
| 2012 | 3209.77042795218 | 0.370412488284105 | 404.359117350442 | 2805.41131060174 | United Republic of Tanzania |
| 2013 | 2624.06188164317 | 0.377963171371427 | 342.916108788611 | 2281.14577285456 | United Republic of Tanzania |
| 2014 | 2360.13668754952 | 0.385974579014964 | 318.369483701188 | 2041.76720384833 | United Republic of Tanzania |
| 2015 | 2334.83152756838 | 0.394277756847982 | 289.395954585152 | 2045.43557298323 | United Republic of Tanzania |
| 2016 | 2204.65923321099 | 0.402879307812708 | 239.447264717273 | 1965.21196849372 | United Republic of Tanzania |
| 2017 | 2011.026801638 | 0.411824974100396 | 218.716002675238 | 1792.31079896276 | United Republic of Tanzania |
| 2018 | 1751.17815782926 | 0.420985364340548 | 193.138649317795 | 1558.03950851146 | United Republic of Tanzania |
| 2019 | 1582.44596869711 | 0.430225378925537 | 139.358207118576 | 1443.08776157853 | United Republic of Tanzania |
| 2020 | 1698.37557084219 | 0.438747237740344 | 128.842349753426 | 1569.53322108876 | United Republic of Tanzania |
| 2021 | 1541.02657167828 | 0.446568273334685 | 112.510551666966 | 1428.51602001131 | United Republic of Tanzania |
| 1990 | 10603.8983445903 | 0.18700109571602 | 2892.3493943992 | 7711.5489501911 | Uganda |
| 1991 | 10471.1513858911 | 0.188924156324117 | 2882.79775443643 | 7588.35363145467 | Uganda |
| 1992 | 10193.5618892307 | 0.191003433034892 | 2840.20927589132 | 7353.35261333938 | Uganda |
| 1993 | 9712.48130516165 | 0.193557455978809 | 2858.93969877693 | 6853.54160638472 | Uganda |
| 1994 | 9175.99569025115 | 0.197293192708182 | 2765.78404712566 | 6410.21164312549 | Uganda |
| 1995 | 8601.00463631161 | 0.201835423450881 | 2740.46564625433 | 5860.53899005728 | Uganda |
| 1996 | 8137.34558529143 | 0.207080730880773 | 2587.65317797518 | 5549.69240731625 | Uganda |
| 1997 | 7740.71514264708 | 0.213091696195112 | 2336.86384053635 | 5403.85130211073 | Uganda |
| 1998 | 7165.39362171054 | 0.219712441898334 | 2092.08541984888 | 5073.30820186166 | Uganda |
| 1999 | 6648.39219450327 | 0.226916944982192 | 2065.18562268835 | 4583.20657181492 | Uganda |
| 2000 | 6101.11170747152 | 0.234323225413513 | 1890.94118507841 | 4210.17052239311 | Uganda |
| 2001 | 5783.10054945154 | 0.242186388084657 | 1672.39839162245 | 4110.7021578291 | Uganda |
| 2002 | 5462.2858893916 | 0.250552388304413 | 1483.89976770074 | 3978.38612169086 | Uganda |
| 2003 | 4961.84654616244 | 0.259164882748285 | 1367.17238381792 | 3594.67416234452 | Uganda |
| 2004 | 4838.56372522444 | 0.268004611155769 | 1246.42748983241 | 3592.13623539203 | Uganda |
| 2005 | 4447.57628978296 | 0.277369192598006 | 1153.97710479845 | 3293.59918498451 | Uganda |
| 2006 | 4014.38201055368 | 0.287030582587568 | 1105.43378001277 | 2908.94823054091 | Uganda |
| 2007 | 3669.96812190895 | 0.296740420486269 | 1026.74720633846 | 2643.22091557049 | Uganda |
| 2008 | 3362.78245146906 | 0.306748843313354 | 886.748355265925 | 2476.03409620313 | Uganda |
| 2009 | 3114.58232606823 | 0.316731152894585 | 778.706446726159 | 2335.87587934207 | Uganda |
| 2010 | 3026.90206138922 | 0.326543661078545 | 716.471140565719 | 2310.4309208235 | Uganda |
| 2011 | 2771.87394784529 | 0.336217715783435 | 651.047408212496 | 2120.82653963279 | Uganda |
| 2012 | 2555.89455694095 | 0.345364515038614 | 571.470069026214 | 1984.42448791474 | Uganda |
| 2013 | 2454.02358610503 | 0.354381615123395 | 554.410222349272 | 1899.61336375576 | Uganda |
| 2014 | 2316.84117706286 | 0.363430903180067 | 466.067944695165 | 1850.7732323677 | Uganda |
| 2015 | 2135.39906086518 | 0.372537985916872 | 351.340487331572 | 1784.05857353361 | Uganda |
| 2016 | 2086.20248063458 | 0.381312226396987 | 326.411144264529 | 1759.79133637005 | Uganda |
| 2017 | 1797.28264239272 | 0.390082258680085 | 282.024478467015 | 1515.25816392571 | Uganda |
| 2018 | 1424.7017848457 | 0.399117776823962 | 262.087946859466 | 1162.61383798623 | Uganda |
| 2019 | 1249.71607530079 | 0.408431557149204 | 239.001343783745 | 1010.71473151705 | Uganda |
| 2020 | 1235.31928188946 | 0.416846079190348 | 193.825628119426 | 1041.49365377003 | Uganda |
| 2021 | 1129.60952621325 | 0.423261181495743 | 178.732332837374 | 950.877193375876 | Uganda |
| 1990 | 21616.8793189584 | 0.304008549420206 | 964.002386932564 | 20652.8769320258 | Zambia |
| 1991 | 22677.4985777354 | 0.305842359512691 | 960.542444589122 | 21716.9561331463 | Zambia |
| 1992 | 21885.0153247855 | 0.307316277250908 | 877.608207368048 | 21007.4071174175 | Zambia |
| 1993 | 20498.2468148733 | 0.308780208280919 | 858.162176028783 | 19640.0846388445 | Zambia |
| 1994 | 19335.0455456708 | 0.309223449091581 | 867.071194783049 | 18467.9743508878 | Zambia |
| 1995 | 18767.8948098989 | 0.309995813177242 | 874.137531012765 | 17893.7572788861 | Zambia |
| 1996 | 18125.3188897873 | 0.311326690947126 | 870.202146132105 | 17255.1167436552 | Zambia |
| 1997 | 17368.7475976879 | 0.313087329617191 | 862.294043134323 | 16506.4535545536 | Zambia |
| 1998 | 16743.9791056477 | 0.314926434901042 | 779.409395670345 | 15964.5697099774 | Zambia |
| 1999 | 16864.5720044284 | 0.317396437417001 | 777.230052584373 | 16087.341951844 | Zambia |
| 2000 | 15945.4854227566 | 0.320625321501856 | 777.534917717817 | 15167.9505050388 | Zambia |
| 2001 | 19821.2738786982 | 0.324401368208402 | 722.480952898537 | 19098.7929257997 | Zambia |
| 2002 | 14164.2122201819 | 0.328680121871397 | 720.939128228275 | 13443.2730919536 | Zambia |
| 2003 | 13735.4217818791 | 0.33372139842644 | 656.544876299414 | 13078.8769055797 | Zambia |
| 2004 | 14031.9311335424 | 0.339488916807796 | 589.380848509004 | 13442.5502850334 | Zambia |
| 2005 | 13275.3146160523 | 0.346048863943135 | 579.723498645907 | 12695.5911174064 | Zambia |
| 2006 | 12567.2941561672 | 0.353540709176488 | 553.816925591583 | 12013.4772305756 | Zambia |
| 2007 | 11537.4295760549 | 0.362450967280465 | 467.474488493398 | 11069.9550875615 | Zambia |
| 2008 | 10822.2538736028 | 0.371688495720519 | 402.235772115125 | 10420.0181014877 | Zambia |
| 2009 | 9737.49858206586 | 0.381667118197368 | 324.849622829472 | 9412.64895923639 | Zambia |
| 2010 | 8726.12995448819 | 0.392470988784596 | 287.450115878464 | 8438.67983860973 | Zambia |
| 2011 | 7936.36830872771 | 0.403475629468659 | 239.206142835923 | 7697.16216589179 | Zambia |
| 2012 | 6469.08166868289 | 0.414574501227966 | 215.800799298226 | 6253.28086938466 | Zambia |
| 2013 | 6007.05540650705 | 0.425492301821784 | 179.821820870361 | 5827.23358563669 | Zambia |
| 2014 | 5450.5407579121 | 0.436244005506199 | 129.776214709285 | 5320.76454320281 | Zambia |
| 2015 | 4957.04894786809 | 0.446744718544927 | 111.196201918455 | 4845.85274594964 | Zambia |
| 2016 | 4439.54398039902 | 0.457122596045687 | 100.340317100414 | 4339.20366329861 | Zambia |
| 2017 | 4260.86101472907 | 0.467532631330298 | 85.2519628713951 | 4175.60905185767 | Zambia |
| 2018 | 3732.59845625061 | 0.477887711662038 | 77.9610124194472 | 3654.63744383116 | Zambia |
| 2019 | 3380.56714446552 | 0.487911522977487 | 76.0808540019202 | 3304.4862904636 | Zambia |
| 2020 | 3038.52701949275 | 0.49748556148019 | 20.3571972221821 | 3018.16982227057 | Zambia |
| 2021 | 2652.0816717858 | 0.505948954434909 | 16.104096519727 | 2635.97757526607 | Zambia |
| 1990 | 1163.91200966088 | 0.418077748220986 | 195.701049145865 | 968.210960515014 | Botswana |
| 1991 | 1118.04232668135 | 0.42975228585408 | 138.123855311123 | 979.918471370227 | Botswana |
| 1992 | 1077.7119535899 | 0.440490843191281 | 130.2551431773 | 947.456810412601 | Botswana |
| 1993 | 1041.36986066313 | 0.450523613884069 | 107.071865182614 | 934.297995480516 | Botswana |
| 1994 | 1022.32310943388 | 0.459954966910237 | 92.9255761262831 | 929.397533307597 | Botswana |
| 1995 | 1005.78175856531 | 0.469266513316808 | 82.5498008397907 | 923.231957725519 | Botswana |
| 1996 | 977.47920254518 | 0.478588352164802 | 77.1976047271062 | 900.281597818074 | Botswana |
| 1997 | 935.06252965226 | 0.487767857070753 | 75.3733539417882 | 859.689175710472 | Botswana |
| 1998 | 929.22297073135 | 0.496193621706379 | 48.0345972675501 | 881.1883734638 | Botswana |
| 1999 | 894.74063074293 | 0.504978826176952 | 16.2127887735094 | 878.527841969421 | Botswana |
| 2000 | 870.03732937104 | 0.513498223414294 | 15.687762940005 | 854.349566431035 | Botswana |
| 2001 | 857.76452819142 | 0.521370181612928 | 15.2043464146465 | 842.560181776773 | Botswana |
| 2002 | 839.71544312808 | 0.529252963791219 | 15.1100969625946 | 824.605346165485 | Botswana |
| 2003 | 806.29866059611 | 0.537000179431652 | 15.045666576752 | 791.252994019358 | Botswana |
| 2004 | 759.46250925006 | 0.544349222513256 | 15.0671131617958 | 744.395396088264 | Botswana |
| 2005 | 729.63172275563 | 0.551706969608463 | 13.7979336952273 | 715.833789060403 | Botswana |
| 2006 | 1409.57398972524 | 0.559221620417772 | 12.1973816440928 | 1397.37660808115 | Botswana |
| 2007 | 754.11818124631 | 0.566702496208929 | 10.2176010780189 | 743.900580168291 | Botswana |
| 2008 | 762.87691671808 | 0.57388380509654 | 8.93393718555275 | 753.942979532527 | Botswana |
| 2009 | 769.33008684034 | 0.579696411585388 | 8.83916623992308 | 760.490920600417 | Botswana |
| 2010 | 771.36870918945 | 0.585824150481272 | 8.86758339737571 | 762.501125792074 | Botswana |
| 2011 | 744.05622609903 | 0.591985456875054 | 8.97706238335476 | 735.079163715675 | Botswana |
| 2012 | 675.883099555 | 0.597739552771288 | 9.0206055562659 | 666.862493998734 | Botswana |
| 2013 | 649.92338782913 | 0.604064730659858 | 8.97020687840333 | 640.953180950727 | Botswana |
| 2014 | 633.36934213043 | 0.610086048809866 | 8.73330316961559 | 624.636038960814 | Botswana |
| 2015 | 613.8549730335 | 0.615411123648458 | 8.83384724595118 | 605.021125787549 | Botswana |
| 2016 | 592.96392577591 | 0.620797574794607 | 8.83452117915979 | 584.12940459675 | Botswana |
| 2017 | 580.07967944776 | 0.625704953034907 | 8.99580422077078 | 571.083875226989 | Botswana |
| 2018 | 563.07876991939 | 0.630606840950645 | 8.06763729131415 | 555.011132628076 | Botswana |
| 2019 | 550.0509408534 | 0.635324093279727 | 6.90299124120366 | 543.147949612196 | Botswana |
| 2020 | 537.43605066397 | 0.639275298342726 | 6.86347844523443 | 530.572572218736 | Botswana |
| 2021 | 528.12984605658 | 0.642721628921122 | 6.26879533139608 | 521.861050725184 | Botswana |
| 1990 | 4413.11155142144 | 0.339155125342909 | 594.18190796658 | 3818.92964345486 | Lesotho |
| 1991 | 4371.20638332915 | 0.34549657132085 | 572.693265814714 | 3798.51311751444 | Lesotho |
| 1992 | 4244.71762584473 | 0.352169629526247 | 557.74457506216 | 3686.97305078257 | Lesotho |
| 1993 | 4085.53671379704 | 0.358780457204597 | 496.313767651832 | 3589.22294614521 | Lesotho |
| 1994 | 3913.52149014481 | 0.365310801307453 | 429.300406052196 | 3484.22108409261 | Lesotho |
| 1995 | 3710.70155503132 | 0.3712653154353 | 405.542126795207 | 3305.15942823611 | Lesotho |
| 1996 | 3570.48326998137 | 0.377221201711086 | 343.174856613732 | 3227.30841336764 | Lesotho |
| 1997 | 3530.85944930107 | 0.383096647768047 | 325.23999755645 | 3205.61945174462 | Lesotho |
| 1998 | 3514.36380115585 | 0.388406478488836 | 284.187163063293 | 3230.17663809256 | Lesotho |
| 1999 | 3608.55211694402 | 0.393495000758155 | 285.048842991503 | 3323.50327395252 | Lesotho |
| 2000 | 3687.63943706752 | 0.398635802456027 | 263.358848362971 | 3424.28058870455 | Lesotho |
| 2001 | 3781.19772537733 | 0.403990126208872 | 236.220999068198 | 3544.97672630913 | Lesotho |
| 2002 | 3824.42635918726 | 0.409021656522558 | 216.573471424676 | 3607.85288776258 | Lesotho |
| 2003 | 3809.87774684191 | 0.413982570645541 | 214.434464536473 | 3595.44328230544 | Lesotho |
| 2004 | 3843.84517284937 | 0.418670918560125 | 194.675034634111 | 3649.17013821526 | Lesotho |
| 2005 | 3799.77497623035 | 0.423211227878272 | 178.23698891042 | 3621.53798731993 | Lesotho |
| 2006 | 3732.52449705918 | 0.427806731919536 | 163.012349161496 | 3569.51214789768 | Lesotho |
| 2007 | 3766.01394787137 | 0.432745038329554 | 133.371914390316 | 3632.64203348105 | Lesotho |
| 2008 | 3776.34804244487 | 0.438340567457201 | 130.085482640244 | 3646.26255980463 | Lesotho |
| 2009 | 3452.23834041226 | 0.443580126941483 | 114.3092606742 | 3337.92907973806 | Lesotho |
| 2010 | 3227.71312084785 | 0.44896647618497 | 104.978049484988 | 3122.73507136286 | Lesotho |
| 2011 | 3220.70421620103 | 0.454530098376785 | 106.386103583622 | 3114.31811261741 | Lesotho |
| 2012 | 3067.69515498597 | 0.460419689024454 | 94.1748182263377 | 2973.52033675963 | Lesotho |
| 2013 | 2708.24949181117 | 0.466692255685783 | 85.7309631152616 | 2622.51852869591 | Lesotho |
| 2014 | 2557.89268424826 | 0.473188157042242 | 80.3103354465824 | 2477.58234880168 | Lesotho |
| 2015 | 2565.27121440358 | 0.479754456396731 | 77.4725226271623 | 2487.79869177642 | Lesotho |
| 2016 | 2537.18814998534 | 0.486144826981745 | 75.1584483685211 | 2462.02970161682 | Lesotho |
| 2017 | 2462.65817637369 | 0.491978154790894 | 72.7372790232597 | 2389.92089735043 | Lesotho |
| 2018 | 2463.04336250074 | 0.497381790902339 | 22.2383556880917 | 2440.80500681265 | Lesotho |
| 2019 | 2464.98497270166 | 0.502457316539537 | 16.4500313704023 | 2448.53494133126 | Lesotho |
| 2020 | 2467.4225800539 | 0.50674665490739 | 16.0423081231866 | 2451.38027193071 | Lesotho |
| 2021 | 2456.86131607323 | 0.510393065681381 | 15.5986192901038 | 2441.26269678313 | Lesotho |
| 1990 | 2801.79733353201 | 0.45004023335046 | 107.334286526537 | 2694.46304700547 | Namibia |
| 1991 | 2686.98834365343 | 0.454534440388721 | 107.232772348207 | 2579.75557130522 | Namibia |
| 1992 | 2569.7469816866 | 0.460021313919932 | 93.5157549086599 | 2476.23122677794 | Namibia |
| 1993 | 2442.66898007946 | 0.465734134805029 | 85.2286341923966 | 2357.44034588706 | Namibia |
| 1994 | 2304.87434773367 | 0.47187321693073 | 83.2422259927967 | 2221.63212174087 | Namibia |
| 1995 | 2171.19673452782 | 0.477794328919676 | 78.3566544581005 | 2092.84008006972 | Namibia |
| 1996 | 2072.83052018757 | 0.483116378270553 | 77.4319813829625 | 1995.39853880461 | Namibia |
| 1997 | 2016.85433518891 | 0.488012938242902 | 74.8248340770174 | 1942.02950111189 | Namibia |
| 1998 | 2074.63249109476 | 0.492812548000801 | 72.3411581508466 | 2002.29133294391 | Namibia |
| 1999 | 2079.09104232715 | 0.49761386200554 | 23.4442350554538 | 2055.6468072717 | Namibia |
| 2000 | 2082.30554615449 | 0.502406957891221 | 16.5804031603192 | 2065.72514299417 | Namibia |
| 2001 | 2043.71906278174 | 0.506719032722361 | 15.9728501252951 | 2027.74621265644 | Namibia |
| 2002 | 2055.05924027895 | 0.510818274709975 | 15.5928207954811 | 2039.46641948347 | Namibia |
| 2003 | 2030.68406504102 | 0.514574645155077 | 15.7193174437826 | 2014.96474759724 | Namibia |
| 2004 | 1982.38364921438 | 0.51910360486724 | 15.35835036626 | 1967.02529884812 | Namibia |
| 2005 | 1977.13986427727 | 0.523881735538067 | 15.1422382359208 | 1961.99762604135 | Namibia |
| 2006 | 1948.10379809081 | 0.529350117774113 | 15.0932064569359 | 1933.01059163387 | Namibia |
| 2007 | 1971.51289353688 | 0.535062125820852 | 15.094433799052 | 1956.41845973783 | Namibia |
| 2008 | 1948.05374567943 | 0.54089699471582 | 15.0467781214074 | 1933.00696755802 | Namibia |
| 2009 | 1874.46813561282 | 0.546516477315561 | 14.4635234761367 | 1860.00461213668 | Namibia |
| 2010 | 1776.10641118505 | 0.552741565170742 | 13.8581185334815 | 1762.24829265157 | Namibia |
| 2011 | 1681.85532541112 | 0.559618899481539 | 11.8254707749303 | 1670.02985463619 | Namibia |
| 2012 | 1544.12374340087 | 0.566978697200188 | 9.86646276272137 | 1534.25728063815 | Namibia |
| 2013 | 1508.23347174561 | 0.574590344212504 | 8.91211856743977 | 1499.32135317817 | Namibia |
| 2014 | 1341.193448789 | 0.582321424930035 | 8.84475041291951 | 1332.34869837608 | Namibia |
| 2015 | 1222.55841215791 | 0.589821040312457 | 8.82094102184297 | 1213.73747113607 | Namibia |
| 2016 | 1144.33669187913 | 0.596283059335379 | 8.85903455192033 | 1135.47765732721 | Namibia |
| 2017 | 1084.41163779886 | 0.601754644588282 | 8.84551498724889 | 1075.56612281161 | Namibia |
| 2018 | 1048.78740385718 | 0.606618403183865 | 8.93767996311308 | 1039.84972389407 | Namibia |
| 2019 | 1018.59828059549 | 0.610921091420642 | 8.85724516313448 | 1009.74103543236 | Namibia |
| 2020 | 987.84876833177 | 0.614435889089058 | 8.91176107487678 | 978.937007256893 | Namibia |
| 2021 | 955.63647832687 | 0.617564872149664 | 8.80521254782588 | 946.831265779044 | Namibia |
| 1990 | 4684.43462919291 | 0.541571435204427 | 15.0188769054893 | 4669.41575228742 | South Africa |
| 1991 | 4455.02471504354 | 0.546921135754198 | 14.3115873158671 | 4440.71312772767 | South Africa |
| 1992 | 4261.50318105204 | 0.552066898744688 | 13.8560718549444 | 4247.6471091971 | South Africa |
| 1993 | 4089.49768696835 | 0.557198439031159 | 12.899883743987 | 4076.59780322436 | South Africa |
| 1994 | 3960.3429882087 | 0.562448636018678 | 11.4701771335985 | 3948.8728110751 | South Africa |
| 1995 | 3838.89517346467 | 0.567905303801023 | 9.69756703685222 | 3829.19760642782 | South Africa |
| 1996 | 3733.03902059104 | 0.573453987768516 | 8.84182293092 | 3724.19719766012 | South Africa |
| 1997 | 3703.27785812228 | 0.57909927788854 | 8.99930308164545 | 3694.27855504063 | South Africa |
| 1998 | 3695.20504949666 | 0.58443731296776 | 8.9916130689168 | 3686.21343642774 | South Africa |
| 1999 | 3661.2620981817 | 0.589407348753061 | 8.87958684333032 | 3652.38251133837 | South Africa |
| 2000 | 3710.39394090144 | 0.594093682936262 | 8.93630353047391 | 3701.45763737097 | South Africa |
| 2001 | 3723.08980363763 | 0.597884168238822 | 8.71395645068889 | 3714.37584718694 | South Africa |
| 2002 | 3724.85428432208 | 0.600921675691185 | 9.1212697300002 | 3715.73301459208 | South Africa |
| 2003 | 3639.31468493737 | 0.603515602003029 | 8.72766780199248 | 3630.58701713538 | South Africa |
| 2004 | 3570.6100045348 | 0.606262513759854 | 8.80644186915977 | 3561.80356266564 | South Africa |
| 2005 | 3499.12803205531 | 0.609989979082156 | 8.93193447801513 | 3490.1960975773 | South Africa |
| 2006 | 3346.31648480971 | 0.615014689925222 | 8.55837918883471 | 3337.75810562088 | South Africa |
| 2007 | 3164.03296206128 | 0.620608149811677 | 8.84993332250989 | 3155.18302873877 | South Africa |
| 2008 | 2981.37945547126 | 0.625948031125006 | 8.9735175928906 | 2972.40593787837 | South Africa |
| 2009 | 2526.24743115513 | 0.630645626147581 | 8.28283533660689 | 2517.96459581852 | South Africa |
| 2010 | 2332.3036707574 | 0.635268538092523 | 6.83249136833339 | 2325.47117938907 | South Africa |
| 2011 | 2203.42670430371 | 0.639991722232976 | 7.06112565280629 | 2196.3655786509 | South Africa |
| 2012 | 2054.07737991276 | 0.644715660880732 | 6.15516587362194 | 2047.92221403914 | South Africa |
| 2013 | 1880.50378250493 | 0.64943055353059 | 5.34551150371056 | 1875.15827100122 | South Africa |
| 2014 | 1779.22346744994 | 0.653964494596397 | 4.93731130176515 | 1774.28615614817 | South Africa |
| 2015 | 1674.38147291309 | 0.658417761080757 | 4.53705438769077 | 1669.8444185254 | South Africa |
| 2016 | 1560.25289771066 | 0.662642486535973 | 4.32586845374218 | 1555.92702925692 | South Africa |
| 2017 | 1476.57223477756 | 0.666622608224758 | 4.05925025002037 | 1472.51298452754 | South Africa |
| 2018 | 1418.42165526224 | 0.670335365040043 | 3.83812075243676 | 1414.5835345098 | South Africa |
| 2019 | 1368.2375796175 | 0.674041036954557 | 3.76588201714622 | 1364.47169760035 | South Africa |
| 2020 | 1194.1912673208 | 0.677166096009468 | 3.61339648466145 | 1190.57787083614 | South Africa |
| 2021 | 1162.60590373912 | 0.679626598156588 | 3.5860416862581 | 1159.01986205286 | South Africa |
| 1990 | 4017.49820578455 | 0.399420954712507 | 260.928521164534 | 3756.56968462002 | Eswatini |
| 1991 | 3907.81096535978 | 0.408269968877275 | 235.752596870028 | 3672.05836848975 | Eswatini |
| 1992 | 3976.72650356474 | 0.416683379093578 | 196.860912770977 | 3779.86559079376 | Eswatini |
| 1993 | 3759.0965558081 | 0.424802994934769 | 180.025743387671 | 3579.07081242043 | Eswatini |
| 1994 | 3702.93760476668 | 0.432503472188562 | 135.470987192919 | 3567.46661757376 | Eswatini |
| 1995 | 3643.67190963196 | 0.440127046710174 | 128.481436861338 | 3515.19047277062 | Eswatini |
| 1996 | 3592.10315794255 | 0.447399238142412 | 112.136195107219 | 3479.96696283533 | Eswatini |
| 1997 | 3551.889160736 | 0.454089854339089 | 107.82073213606 | 3444.06842859994 | Eswatini |
| 1998 | 3532.89852518486 | 0.46013698389181 | 92.987276302513 | 3439.91124888235 | Eswatini |
| 1999 | 3586.08423888913 | 0.465990845350804 | 85.2147241486309 | 3500.8695147405 | Eswatini |
| 2000 | 3763.01473005235 | 0.471852843205366 | 82.73416951814 | 3680.28056053421 | Eswatini |
| 2001 | 4148.54435763362 | 0.477233374481226 | 77.3985600992638 | 4071.14579753436 | Eswatini |
| 2002 | 3692.56303884582 | 0.482474423324763 | 77.2648588437709 | 3615.29818000205 | Eswatini |
| 2003 | 3755.55601729546 | 0.487608829048368 | 75.4706319193849 | 3680.08538537608 | Eswatini |
| 2004 | 3748.34752503516 | 0.492669158763782 | 73.6189351748561 | 3674.7285898603 | Eswatini |
| 2005 | 3539.17941792586 | 0.498043848851584 | 18.3324177802131 | 3520.84700014565 | Eswatini |
| 2006 | 3385.54785175731 | 0.503658293748865 | 16.1120861441928 | 3369.43576561312 | Eswatini |
| 2007 | 3302.21891641551 | 0.509260218872045 | 15.8707730584438 | 3286.34814335707 | Eswatini |
| 2008 | 3170.93779689208 | 0.514730895784455 | 15.7422808624691 | 3155.19551602961 | Eswatini |
| 2009 | 2997.25990790544 | 0.520427398115377 | 15.2331060355788 | 2982.02680186986 | Eswatini |
| 2010 | 2705.23533761812 | 0.526245801589325 | 15.0947756836915 | 2690.14056193443 | Eswatini |
| 2011 | 2456.15850301446 | 0.531913910153764 | 15.0726629031006 | 2441.08584011136 | Eswatini |
| 2012 | 2295.25048954311 | 0.537798022788039 | 15.0491430109026 | 2280.20134653221 | Eswatini |
| 2013 | 2128.03018934994 | 0.543836416273961 | 15.0194157575928 | 2113.01077359235 | Eswatini |
| 2014 | 1952.76467376531 | 0.549613848251705 | 13.789839824032 | 1938.97483394128 | Eswatini |
| 2015 | 1648.29636076453 | 0.555373738956414 | 13.1450924899968 | 1635.15126827453 | Eswatini |
| 2016 | 1568.64753769451 | 0.561061185783082 | 11.5781161342425 | 1557.06942156027 | Eswatini |
| 2017 | 1482.77590880156 | 0.566698492780748 | 10.5409512634405 | 1472.23495753812 | Eswatini |
| 2018 | 1360.55923183299 | 0.572154807441339 | 8.7399831792007 | 1351.81924865379 | Eswatini |
| 2019 | 1298.51220744404 | 0.577206305034792 | 8.8442813676999 | 1289.66792607634 | Eswatini |
| 2020 | 1237.76944532327 | 0.581653071099534 | 8.69646779970442 | 1229.07297752357 | Eswatini |
| 2021 | 1171.04201641673 | 0.58545971329224 | 8.75622660000755 | 1162.28578981672 | Eswatini |
| 1990 | 1526.67416334437 | 0.398559340611047 | 256.797558820549 | 1269.87660452382 | Zimbabwe |
| 1991 | 1467.98161091998 | 0.406052876694226 | 239.797334908253 | 1228.18427601173 | Zimbabwe |
| 1992 | 1584.14838810171 | 0.412083825921141 | 214.415266669925 | 1369.73312143179 | Zimbabwe |
| 1993 | 1558.42214825141 | 0.417974200219438 | 197.835391164327 | 1360.58675708708 | Zimbabwe |
| 1994 | 1421.0610863005 | 0.424051564461987 | 179.034653546028 | 1242.02643275447 | Zimbabwe |
| 1995 | 1369.37197526948 | 0.428929040100791 | 164.246339980586 | 1205.12563528889 | Zimbabwe |
| 1996 | 1301.49051876511 | 0.43418719166794 | 130.376729071494 | 1171.11378969362 | Zimbabwe |
| 1997 | 1302.47848662305 | 0.438141987585594 | 128.851231414749 | 1173.6272552083 | Zimbabwe |
| 1998 | 1331.38903798515 | 0.441093134678486 | 119.954007698516 | 1211.43503028663 | Zimbabwe |
| 1999 | 1446.12254660321 | 0.443246603614544 | 111.741396368741 | 1334.38115023447 | Zimbabwe |
| 2000 | 1537.2929693281 | 0.444841401738061 | 111.312861668243 | 1425.98010765986 | Zimbabwe |
| 2001 | 1557.39984086821 | 0.446354680553508 | 111.484321443939 | 1445.91551942427 | Zimbabwe |
| 2002 | 1689.70072682137 | 0.446970895206592 | 112.475993048554 | 1577.22473377282 | Zimbabwe |
| 2003 | 1673.48967454665 | 0.446002181962281 | 108.562582227682 | 1564.92709231897 | Zimbabwe |
| 2004 | 1668.75067363284 | 0.443696404187693 | 111.461398314559 | 1557.28927531828 | Zimbabwe |
| 2005 | 1762.30404696785 | 0.439651656979407 | 127.445462217107 | 1634.85858475074 | Zimbabwe |
| 2006 | 1832.83594482449 | 0.434405758403472 | 131.162908788601 | 1701.67303603589 | Zimbabwe |
| 2007 | 1744.94907157459 | 0.429165139621798 | 167.624238932207 | 1577.32483264238 | Zimbabwe |
| 2008 | 2779.00129167648 | 0.422448974219341 | 182.856232472348 | 2596.14505920413 | Zimbabwe |
| 2009 | 3087.89620041194 | 0.418330613224402 | 195.569518286613 | 2892.32668212533 | Zimbabwe |
| 2010 | 1884.60998832755 | 0.417305356749818 | 194.734316423667 | 1689.87567190388 | Zimbabwe |
| 2011 | 1932.49138839225 | 0.419729694705381 | 192.483942678209 | 1740.00744571404 | Zimbabwe |
| 2012 | 1806.7578097904 | 0.425748519814194 | 179.943982014771 | 1626.81382777563 | Zimbabwe |
| 2013 | 1623.46940255207 | 0.432881888218656 | 143.022880360035 | 1480.44652219204 | Zimbabwe |
| 2014 | 1359.64258485244 | 0.439997226175148 | 128.420321596648 | 1231.22226325579 | Zimbabwe |
| 2015 | 1274.55670670419 | 0.446552872040041 | 111.847454307565 | 1162.70925239662 | Zimbabwe |
| 2016 | 1246.0838836102 | 0.452473036911982 | 106.39771962278 | 1139.68616398742 | Zimbabwe |
| 2017 | 1119.03140112781 | 0.458424984120402 | 95.4596422701698 | 1023.57175885764 | Zimbabwe |
| 2018 | 1130.01731268863 | 0.464379262217529 | 90.0127576961002 | 1040.00455499253 | Zimbabwe |
| 2019 | 1076.52595547699 | 0.46838443787862 | 85.1002810486606 | 991.42567442833 | Zimbabwe |
| 2020 | 1027.60678040063 | 0.471573170735407 | 82.1584830710975 | 945.448297329532 | Zimbabwe |
| 2021 | 1074.31515137144 | 0.473819486202707 | 80.4705278658656 | 993.844623505574 | Zimbabwe |
| 1990 | 18585.2543190009 | 0.218907153827645 | 2305.97869597381 | 16279.2756230271 | Benin |
| 1991 | 19033.551868089 | 0.222539019363851 | 2098.79281984727 | 16934.7590482417 | Benin |
| 1992 | 17644.9122554424 | 0.226323465232837 | 2064.85755312272 | 15580.0547023197 | Benin |
| 1993 | 17003.0624629612 | 0.230312593690675 | 1909.09050486088 | 15093.9719581003 | Benin |
| 1994 | 16448.0253648801 | 0.234032035653662 | 1886.51202849088 | 14561.5133363892 | Benin |
| 1995 | 15723.6274557089 | 0.238009696338161 | 1658.69005479563 | 14064.9374009133 | Benin |
| 1996 | 15715.9603916741 | 0.241895322542988 | 1677.33391729057 | 14038.6264743835 | Benin |
| 1997 | 14354.5199947026 | 0.245731534038315 | 1676.77477474808 | 12677.7452199545 | Benin |
| 1998 | 13574.3346973624 | 0.249376450881329 | 1462.73732502034 | 12111.5973723421 | Benin |
| 1999 | 13001.6979582695 | 0.253215340715821 | 1532.5856697911 | 11469.1122884784 | Benin |
| 2000 | 12424.3890915463 | 0.257380681388323 | 1342.68033292245 | 11081.7087586239 | Benin |
| 2001 | 11719.8859853634 | 0.261388010509781 | 1351.96212691565 | 10367.9238584477 | Benin |
| 2002 | 11378.6565132838 | 0.265417457761242 | 1333.22635336235 | 10045.4301599215 | Benin |
| 2003 | 10601.4193393541 | 0.269544281064264 | 1242.2165038777 | 9359.2028354764 | Benin |
| 2004 | 10312.3256545534 | 0.273941144522725 | 1223.09099855953 | 9089.23465599387 | Benin |
| 2005 | 9648.26710374357 | 0.278406750274251 | 1156.50932447242 | 8491.75777927115 | Benin |
| 2006 | 9255.1761159249 | 0.282933935672546 | 1151.39033621312 | 8103.78577971178 | Benin |
| 2007 | 8617.98251672177 | 0.287673054621831 | 1104.24860336842 | 7513.73391335335 | Benin |
| 2008 | 8127.73293253965 | 0.292627446283673 | 1032.1805120689 | 7095.55242047075 | Benin |
| 2009 | 7770.94608491979 | 0.297702227030519 | 1034.68840035884 | 6736.25768456095 | Benin |
| 2010 | 7485.6824572186 | 0.302809008308589 | 964.591231735298 | 6521.0912254833 | Benin |
| 2011 | 7018.7942297368 | 0.30792553783669 | 873.744339471588 | 6145.04989026521 | Benin |
| 2012 | 6808.10051623617 | 0.313373588474229 | 857.508537662556 | 5950.59197857361 | Benin |
| 2013 | 6382.36858943012 | 0.319318688191374 | 777.296951673355 | 5605.07163775677 | Benin |
| 2014 | 5802.39424567717 | 0.325727271772744 | 720.44140113671 | 5081.95284454046 | Benin |
| 2015 | 5627.70272365849 | 0.332216350180226 | 653.956750551807 | 4973.74597310668 | Benin |
| 2016 | 5239.92035701617 | 0.338763921416219 | 592.960048536548 | 4646.96030847962 | Benin |
| 2017 | 4814.74964830491 | 0.345606758793044 | 578.189442719013 | 4236.5602055859 | Benin |
| 2018 | 4205.83946268341 | 0.352652695728689 | 555.429350536222 | 3650.41011214719 | Benin |
| 2019 | 3398.74365073281 | 0.360031163133997 | 496.213096142842 | 2902.53055458997 | Benin |
| 2020 | 3339.92196564574 | 0.36696448721315 | 407.705814950025 | 2932.21615069571 | Benin |
| 2021 | 3014.47517076349 | 0.373486574239249 | 356.040092091637 | 2658.43507867185 | Benin |
| 1990 | 6829.52151007169 | 0.129695615380271 | 4169.44360724877 | 2660.07790282292 | Burkina Faso |
| 1991 | 6580.65267724127 | 0.133299127658948 | 4128.38619973798 | 2452.26647750329 | Burkina Faso |
| 1992 | 6361.34820167539 | 0.136594798422558 | 4177.27070417052 | 2184.07749750487 | Burkina Faso |
| 1993 | 6175.65703129895 | 0.139825464930799 | 4175.07981767785 | 2000.5772136211 | Burkina Faso |
| 1994 | 6014.62655976654 | 0.142848972509425 | 4132.03890025736 | 1882.58765950918 | Burkina Faso |
| 1995 | 5888.64585403496 | 0.146115672699206 | 4212.39993365964 | 1676.24592037532 | Burkina Faso |
| 1996 | 5687.84998498467 | 0.150041686384544 | 4182.08777544037 | 1505.7622095443 | Burkina Faso |
| 1997 | 5476.74244261466 | 0.154228401481324 | 4159.31441765568 | 1317.42802495898 | Burkina Faso |
| 1998 | 5284.57547451427 | 0.158707048449452 | 4181.47350397358 | 1103.10197054069 | Burkina Faso |
| 1999 | 5185.44536963975 | 0.163468066858732 | 4156.48758219895 | 1028.9577874408 | Burkina Faso |
| 2000 | 5301.36395911582 | 0.168084340000467 | 4160.7411510009 | 1140.62280811492 | Burkina Faso |
| 2001 | 4985.74116040143 | 0.172996395327051 | 4168.82994718955 | 816.911213211878 | Burkina Faso |
| 2002 | 4745.87919957084 | 0.17798198994173 | 3025.36134890284 | 1720.517850668 | Burkina Faso |
| 2003 | 4266.83549448325 | 0.183253539277265 | 3015.91702492854 | 1250.91846955471 | Burkina Faso |
| 2004 | 3904.07323970569 | 0.188564995004729 | 2891.33020478996 | 1012.74303491573 | Burkina Faso |
| 2005 | 3676.51776329442 | 0.194086128180827 | 2833.31479921739 | 843.202964077032 | Burkina Faso |
| 2006 | 3508.66832832428 | 0.199571952068224 | 2746.41770567228 | 762.250622651996 | Burkina Faso |
| 2007 | 3195.15494278428 | 0.204891092363641 | 2597.62637089714 | 597.528571887138 | Burkina Faso |
| 2008 | 3037.45255411922 | 0.210223294804678 | 2592.33729239586 | 445.115261723362 | Burkina Faso |
| 2009 | 2844.95598980381 | 0.215423373997381 | 2319.0423676836 | 525.913622120206 | Burkina Faso |
| 2010 | 2730.13774058197 | 0.221001030335074 | 2072.22797145031 | 657.909769131661 | Burkina Faso |
| 2011 | 2643.0920158795 | 0.226696631667163 | 2044.75108298384 | 598.340932895661 | Burkina Faso |
| 2012 | 2609.98533684848 | 0.23258913471079 | 1883.42911971885 | 726.556217129626 | Burkina Faso |
| 2013 | 2280.61588181626 | 0.238497491410915 | 1668.19837163867 | 612.417510177593 | Burkina Faso |
| 2014 | 2184.46700892063 | 0.244378901166049 | 1663.83067450837 | 520.636334412263 | Burkina Faso |
| 2015 | 2197.90351942608 | 0.250208260823773 | 1518.9482913446 | 678.955228081482 | Burkina Faso |
| 2016 | 2195.01840845667 | 0.256081773936025 | 1499.50243051354 | 695.515977943128 | Burkina Faso |
| 2017 | 2110.65413257207 | 0.26203300853891 | 1350.74004188094 | 759.914090691129 | Burkina Faso |
| 2018 | 1960.80248709444 | 0.268102072864973 | 1244.83521660632 | 715.967270488117 | Burkina Faso |
| 2019 | 1844.59530326572 | 0.274287092352859 | 1229.22504645754 | 615.370256808181 | Burkina Faso |
| 2020 | 1709.61869051194 | 0.279871317215145 | 1152.59323816145 | 557.025452350491 | Burkina Faso |
| 2021 | 1611.6935180918 | 0.285118401552141 | 1104.17965875904 | 507.513859332762 | Burkina Faso |
| 1990 | 7242.36464629034 | 0.303055334404352 | 961.391725324089 | 6280.97292096625 | Cameroon |
| 1991 | 7363.92133455366 | 0.30962345806122 | 868.884626705913 | 6495.03670784775 | Cameroon |
| 1992 | 7092.91652974109 | 0.315601268856928 | 775.497291821858 | 6317.41923791923 | Cameroon |
| 1993 | 7022.64562329758 | 0.320831654317412 | 780.845340448645 | 6241.80028284894 | Cameroon |
| 1994 | 6988.66989766727 | 0.325410122747214 | 719.308863093318 | 6269.36103457395 | Cameroon |
| 1995 | 6836.44542706227 | 0.329539317887926 | 726.863844890426 | 6109.58158217184 | Cameroon |
| 1996 | 7026.29440671399 | 0.333425981814059 | 659.967587980069 | 6366.32681873392 | Cameroon |
| 1997 | 6781.45532911749 | 0.337346000940944 | 607.670111218958 | 6173.78521789853 | Cameroon |
| 1998 | 6614.57780572938 | 0.341197089780886 | 582.585307066796 | 6031.99249866258 | Cameroon |
| 1999 | 6473.77050038946 | 0.344955364212408 | 565.846312777824 | 5907.92418761164 | Cameroon |
| 2000 | 6527.81813002347 | 0.348814788893637 | 557.088050080967 | 5970.7300799425 | Cameroon |
| 2001 | 6365.86683090354 | 0.352631872778337 | 555.244449038433 | 5810.62238186511 | Cameroon |
| 2002 | 6795.64221315794 | 0.356511816668113 | 552.209640979913 | 6243.43257217803 | Cameroon |
| 2003 | 6681.76980075063 | 0.360668581210006 | 484.12584359679 | 6197.64395715384 | Cameroon |
| 2004 | 6469.48768356708 | 0.365100029903911 | 418.221801537637 | 6051.26588202944 | Cameroon |
| 2005 | 6300.34064322213 | 0.369545756449213 | 403.28509149899 | 5897.05555172314 | Cameroon |
| 2006 | 6149.70990405731 | 0.374355877714557 | 352.34796771687 | 5797.36193634044 | Cameroon |
| 2007 | 5769.27669997003 | 0.379490349180985 | 349.623646212381 | 5419.65305375765 | Cameroon |
| 2008 | 5505.73847671042 | 0.38483830406879 | 315.858209223259 | 5189.88026748716 | Cameroon |
| 2009 | 5371.64497650979 | 0.390288642984762 | 289.423453732651 | 5082.22152277714 | Cameroon |
| 2010 | 5411.70046466748 | 0.396067145154693 | 261.401072563773 | 5150.29939210371 | Cameroon |
| 2011 | 5089.75089609004 | 0.402092798748116 | 239.565457162355 | 4850.18543892769 | Cameroon |
| 2012 | 4402.65794482027 | 0.408580122601571 | 219.835573414258 | 4182.82237140601 | Cameroon |
| 2013 | 4094.46558059504 | 0.415607964760584 | 199.44225006864 | 3895.0233305264 | Cameroon |
| 2014 | 3502.66559855625 | 0.423033560011266 | 183.299691447577 | 3319.36590710867 | Cameroon |
| 2015 | 3075.09321473728 | 0.430830276458225 | 139.569484790986 | 2935.52372994629 | Cameroon |
| 2016 | 2934.69063733428 | 0.438878945356817 | 125.794120136734 | 2808.89651719755 | Cameroon |
| 2017 | 2654.69415326452 | 0.447185100165971 | 112.985468073178 | 2541.70868519134 | Cameroon |
| 2018 | 2460.19983786988 | 0.455687846771621 | 99.2376014415842 | 2360.9622364283 | Cameroon |
| 2019 | 2186.5214515557 | 0.464246472572462 | 90.0154393361603 | 2096.50601221954 | Cameroon |
| 2020 | 1988.09496122366 | 0.472285846440991 | 83.6097135282537 | 1904.48524769541 | Cameroon |
| 2021 | 1756.55293507868 | 0.479691223419746 | 77.1963490223554 | 1679.35658605632 | Cameroon |
| 1990 | 5541.97179629393 | 0.27672336711542 | 1150.96641922168 | 4391.00537707225 | Cabo Verde |
| 1991 | 5126.4103140602 | 0.282931721669337 | 1164.27011747439 | 3962.14019658581 | Cabo Verde |
| 1992 | 4851.19725004818 | 0.288460371177891 | 1104.4719591686 | 3746.72529087958 | Cabo Verde |
| 1993 | 4619.38820348266 | 0.294517285990211 | 1028.02995040619 | 3591.35825307647 | Cabo Verde |
| 1994 | 5324.9763045624 | 0.302216794344931 | 963.687984080736 | 4361.28832048166 | Cabo Verde |
| 1995 | 9624.61491930763 | 0.310675633896478 | 877.088008277027 | 8747.5269110306 | Cabo Verde |
| 1996 | 3629.99647308765 | 0.319736229276549 | 779.812607841165 | 2850.18386524649 | Cabo Verde |
| 1997 | 3112.43813338422 | 0.329049585287328 | 717.869486139831 | 2394.56864724439 | Cabo Verde |
| 1998 | 2721.69955402447 | 0.338786069354652 | 600.21542799581 | 2121.48412602866 | Cabo Verde |
| 1999 | 2446.41343663077 | 0.349296918199719 | 554.612664307433 | 1891.80077232334 | Cabo Verde |
| 2000 | 2150.6975128765 | 0.35989901703054 | 482.759410733857 | 1667.93810214264 | Cabo Verde |
| 2001 | 1972.80437293987 | 0.370070275603364 | 402.564046101385 | 1570.24032683848 | Cabo Verde |
| 2002 | 1826.01869380849 | 0.379846674271366 | 345.799586898115 | 1480.21910691037 | Cabo Verde |
| 2003 | 1650.92521348272 | 0.389419785822276 | 291.182883791354 | 1359.74232969137 | Cabo Verde |
| 2004 | 1501.06707336465 | 0.398838732156207 | 266.196449551281 | 1234.87062381337 | Cabo Verde |
| 2005 | 1356.21235190843 | 0.408009221053434 | 233.44326083174 | 1122.76909107669 | Cabo Verde |
| 2006 | 1322.55197457111 | 0.417433785875035 | 194.569090710016 | 1127.98288386109 | Cabo Verde |
| 2007 | 1202.31911136564 | 0.427379786660776 | 177.672475708247 | 1024.64663565739 | Cabo Verde |
| 2008 | 1086.23172589992 | 0.437372129942837 | 125.196394663678 | 961.035331236242 | Cabo Verde |
| 2009 | 976.61192633128 | 0.446691505793351 | 112.137124107875 | 864.474802223405 | Cabo Verde |
| 2010 | 952.70298431272 | 0.455647568382572 | 99.3564585124398 | 853.34652580028 | Cabo Verde |
| 2011 | 857.47846508759 | 0.464473968892355 | 89.4314493622455 | 768.047015725345 | Cabo Verde |
| 2012 | 811.69409335978 | 0.472857771380441 | 82.5004477527917 | 729.193645606988 | Cabo Verde |
| 2013 | 747.71312755443 | 0.480796480653885 | 77.17333481073 | 670.5397927437 | Cabo Verde |
| 2014 | 735.03487713865 | 0.488259896216045 | 76.2491650142562 | 658.785712124394 | Cabo Verde |
| 2015 | 678.32619701032 | 0.495229489103676 | 70.4947525231628 | 607.831444487157 | Cabo Verde |
| 2016 | 630.92773892612 | 0.501855423977514 | 16.7002287636687 | 614.227510162451 | Cabo Verde |
| 2017 | 574.19992355688 | 0.508435792446345 | 15.7871685729892 | 558.412754983891 | Cabo Verde |
| 2018 | 537.09559892407 | 0.515285886261897 | 15.289837864511 | 521.805761059559 | Cabo Verde |
| 2019 | 491.01808399338 | 0.522488008219635 | 15.1668429936504 | 475.85124099973 | Cabo Verde |
| 2020 | 445.57476615593 | 0.528461594777472 | 15.1217539512333 | 430.453012204697 | Cabo Verde |
| 2021 | 418.73613570987 | 0.53353453903661 | 15.0448427837816 | 403.691292926088 | Cabo Verde |
| 1990 | 23540.2744444067 | 0.114638829376832 | 4169.5672871956 | 19370.7071572111 | Chad |
| 1991 | 24939.6907373256 | 0.117954633635022 | 4140.75857200573 | 20798.9321653199 | Chad |
| 1992 | 22665.658878129 | 0.121132831941026 | 4164.73281650787 | 18500.9260616211 | Chad |
| 1993 | 22336.7016981945 | 0.123600072412422 | 4136.50668174294 | 18200.1950164516 | Chad |
| 1994 | 21997.3959112674 | 0.126080571521756 | 4190.72833854385 | 17806.6675727235 | Chad |
| 1995 | 21588.2956062551 | 0.128289491688625 | 4165.76902863567 | 17422.5265776194 | Chad |
| 1996 | 21390.2295614067 | 0.130169483922475 | 4150.78198398162 | 17239.4475774251 | Chad |
| 1997 | 21416.5328478044 | 0.132136021297442 | 4171.55621763563 | 17244.9766301688 | Chad |
| 1998 | 20866.7931799582 | 0.134090304250411 | 4165.40948461219 | 16701.383695346 | Chad |
| 1999 | 20530.6649717226 | 0.135825260914887 | 4151.29192062549 | 16379.3730510971 | Chad |
| 2000 | 20455.1919749462 | 0.137412374447162 | 4190.73000056033 | 16264.4619743859 | Chad |
| 2001 | 20353.1057138838 | 0.13966369176859 | 4171.9458339781 | 16181.1598799057 | Chad |
| 2002 | 20222.5797304691 | 0.142041700148116 | 4149.50860841709 | 16073.071122052 | Chad |
| 2003 | 19783.9782956946 | 0.145348052064453 | 4191.85343705585 | 15592.1248586387 | Chad |
| 2004 | 19278.130913069 | 0.150910867941762 | 4182.82637216879 | 15095.3045409002 | Chad |
| 2005 | 18398.4265280301 | 0.157033036048947 | 4184.04216009774 | 14214.3843679324 | Chad |
| 2006 | 18314.334075987 | 0.162576402156357 | 4186.84438294207 | 14127.4896930449 | Chad |
| 2007 | 18254.9313813939 | 0.168161061828519 | 4190.07645892107 | 14064.8549224728 | Chad |
| 2008 | 17715.2352763512 | 0.173396783580077 | 4171.94102196777 | 13543.2942543834 | Chad |
| 2009 | 17343.65146417 | 0.178335360569271 | 3014.51118193411 | 14329.1402822359 | Chad |
| 2010 | 16808.9149159563 | 0.184110621219396 | 2879.12129368333 | 13929.793622273 | Chad |
| 2011 | 16246.2525703057 | 0.189591609430813 | 2879.52632018428 | 13366.7262501214 | Chad |
| 2012 | 15126.7662560538 | 0.195262748994652 | 2832.75086486703 | 12294.0153911868 | Chad |
| 2013 | 14894.5811622685 | 0.200952883793321 | 2752.06241457472 | 12142.5187476938 | Chad |
| 2014 | 14190.1400029971 | 0.206658835963237 | 2583.79451217924 | 11606.3454908179 | Chad |
| 2015 | 13893.3079412667 | 0.212428696994434 | 2355.52484122375 | 11537.7831000429 | Chad |
| 2016 | 13485.730128171 | 0.217532179615503 | 2332.96624153396 | 11152.763886637 | Chad |
| 2017 | 13163.0463229026 | 0.222206950439072 | 2096.53056184619 | 11066.5157610564 | Chad |
| 2018 | 12578.885360663 | 0.226913880545936 | 2081.81944263978 | 10497.0659180232 | Chad |
| 2019 | 12139.8800184246 | 0.231704754518571 | 1897.41030521855 | 10242.469713206 | Chad |
| 2020 | 11488.2244656054 | 0.236103082769538 | 1865.24050909696 | 9622.98395650844 | Chad |
| 2021 | 11022.3046604706 | 0.240436018662427 | 1668.79146880864 | 9353.51319166196 | Chad |
| 1990 | 7010.89646384618 | 0.279320352514124 | 1159.29799583352 | 5851.59846801266 | Côte d'Ivoire |
| 1991 | 6901.48994692027 | 0.286492681425857 | 1097.87675548965 | 5803.61319143062 | Côte d'Ivoire |
| 1992 | 6792.52541891527 | 0.293451293054487 | 1031.4993772448 | 5761.02604167047 | Côte d'Ivoire |
| 1993 | 6726.90143450718 | 0.299743215098528 | 979.760527364611 | 5747.14090714257 | Côte d'Ivoire |
| 1994 | 6655.92942395823 | 0.305210652688978 | 962.909000300705 | 5693.02042365752 | Côte d'Ivoire |
| 1995 | 6621.10708820063 | 0.310105507687377 | 868.732458636976 | 5752.37462956365 | Côte d'Ivoire |
| 1996 | 6345.09755971727 | 0.314965385351403 | 780.733825976276 | 5564.36373374099 | Côte d'Ivoire |
| 1997 | 6114.86035917396 | 0.319843979495224 | 780.199858612654 | 5334.66050056131 | Côte d'Ivoire |
| 1998 | 5922.4741354253 | 0.324712872541106 | 719.325975858464 | 5203.14815956684 | Côte d'Ivoire |
| 1999 | 5661.92355890842 | 0.329201009286605 | 724.07529677604 | 4937.84826213238 | Côte d'Ivoire |
| 2000 | 5398.62077476628 | 0.333015393275065 | 653.671923320011 | 4744.94885144627 | Côte d'Ivoire |
| 2001 | 5227.08240185459 | 0.336545511228203 | 652.221132612173 | 4574.86126924242 | Côte d'Ivoire |
| 2002 | 4854.78331045067 | 0.339977638379859 | 592.168543466722 | 4262.61476698395 | Côte d'Ivoire |
| 2003 | 4611.74572907489 | 0.343348931560052 | 571.775080823146 | 4039.97064825174 | Côte d'Ivoire |
| 2004 | 4566.25967668888 | 0.346375411313471 | 573.592718708133 | 3992.66695798075 | Côte d'Ivoire |
| 2005 | 4568.55349588938 | 0.34872791321654 | 557.630487415871 | 4010.92300847351 | Côte d'Ivoire |
| 2006 | 4685.90748889569 | 0.35071594385815 | 557.161398935154 | 4128.74608996054 | Côte d'Ivoire |
| 2007 | 4502.26934468706 | 0.35272747838259 | 552.481446934105 | 3949.78789775295 | Côte d'Ivoire |
| 2008 | 4236.36396659008 | 0.355087642720634 | 554.834514146449 | 3681.52945244363 | Côte d'Ivoire |
| 2009 | 4010.57826369983 | 0.357938754729774 | 489.375788034738 | 3521.20247566509 | Côte d'Ivoire |
| 2010 | 3768.6206610822 | 0.361205186672734 | 479.969797571934 | 3288.65086351027 | Côte d'Ivoire |
| 2011 | 3431.37844937727 | 0.364083650087326 | 458.773455933386 | 2972.60499344388 | Côte d'Ivoire |
| 2012 | 3398.13411805823 | 0.367831685487642 | 414.69402568526 | 2983.44009237297 | Côte d'Ivoire |
| 2013 | 3334.50320220693 | 0.372353790323331 | 401.333926811883 | 2933.16927539505 | Côte d'Ivoire |
| 2014 | 3127.18562797812 | 0.377575443834257 | 345.087472186469 | 2782.09815579165 | Côte d'Ivoire |
| 2015 | 3006.47156641802 | 0.383491379717949 | 321.079296990004 | 2685.39226942802 | Côte d'Ivoire |
| 2016 | 2757.37566644493 | 0.389825191524618 | 283.517900076602 | 2473.85776636833 | Côte d'Ivoire |
| 2017 | 2278.46847336039 | 0.396693974091431 | 262.545919840648 | 2015.92255351974 | Côte d'Ivoire |
| 2018 | 1977.66554561055 | 0.404091932623989 | 241.140520742957 | 1736.52502486759 | Côte d'Ivoire |
| 2019 | 1742.10134270413 | 0.411725580444537 | 215.09002152533 | 1527.0113211788 | Côte d'Ivoire |
| 2020 | 1545.10403490476 | 0.419040103341885 | 195.172449345128 | 1349.93158555963 | Côte d'Ivoire |
| 2021 | 1403.51753052871 | 0.425941883067361 | 178.257021120213 | 1225.2605094085 | Côte d'Ivoire |
| 1990 | 6525.8606480151 | 0.238714846451473 | 1646.91247934984 | 4878.94816866526 | Gambia |
| 1991 | 6361.01269333105 | 0.2450101639184 | 1682.95244830377 | 4678.06024502728 | Gambia |
| 1992 | 6228.80981339148 | 0.251087863304524 | 1502.99090829843 | 4725.81890509305 | Gambia |
| 1993 | 5893.55731241906 | 0.25699614074155 | 1482.18858966589 | 4411.36872275317 | Gambia |
| 1994 | 5566.67212020397 | 0.262424792137193 | 1338.35117391219 | 4228.32094629178 | Gambia |
| 1995 | 5585.19183392327 | 0.267696506742935 | 1239.02300316013 | 4346.16883076314 | Gambia |
| 1996 | 5131.10949558336 | 0.272950190161523 | 1225.17914214105 | 3905.93035344231 | Gambia |
| 1997 | 4801.18381528296 | 0.278274122042772 | 1166.14313732665 | 3635.04067795631 | Gambia |
| 1998 | 4658.18033662125 | 0.283946056199827 | 1157.41380606323 | 3500.76653055802 | Gambia |
| 1999 | 4492.83920495314 | 0.290070621097832 | 1099.7196669373 | 3393.11953801584 | Gambia |
| 2000 | 3986.65459672401 | 0.296354282057957 | 1024.36067229122 | 2962.29392443279 | Gambia |
| 2001 | 3673.00734529055 | 0.302543256845694 | 956.795160389331 | 2716.21218490122 | Gambia |
| 2002 | 3475.45015828332 | 0.307712926799456 | 868.936808651684 | 2606.51334963164 | Gambia |
| 2003 | 3361.51030129043 | 0.312930916018188 | 865.283525269913 | 2496.22677602052 | Gambia |
| 2004 | 3753.65159576984 | 0.31861502922509 | 780.196496892016 | 2973.45509887782 | Gambia |
| 2005 | 3637.55325345094 | 0.323568350048743 | 718.120901335418 | 2919.43235211552 | Gambia |
| 2006 | 3309.32783337557 | 0.328151431165807 | 718.670165345061 | 2590.65766803051 | Gambia |
| 2007 | 3079.52599554531 | 0.332640147075474 | 647.306313788214 | 2432.2196817571 | Gambia |
| 2008 | 2652.63218395645 | 0.337315245461033 | 595.746330966487 | 2056.88585298996 | Gambia |
| 2009 | 2313.97359272434 | 0.342389614346251 | 575.990715149154 | 1737.98287757519 | Gambia |
| 2010 | 2077.72329502632 | 0.347997829857342 | 568.741500663626 | 1508.98179436269 | Gambia |
| 2011 | 1742.578579115 | 0.35286923573207 | 556.234042331738 | 1186.34453678326 | Gambia |
| 2012 | 1672.48752015544 | 0.358373649782742 | 490.740432327064 | 1181.74708782838 | Gambia |
| 2013 | 1401.31961138754 | 0.36425587830746 | 462.062937261529 | 939.25667412601 | Gambia |
| 2014 | 1412.55322266984 | 0.369901300206273 | 404.426418476582 | 1008.12680419326 | Gambia |
| 2015 | 1269.95858102745 | 0.375840484637477 | 354.433330799657 | 915.525250227794 | Gambia |
| 2016 | 1267.82701473959 | 0.381569217140042 | 328.255490093386 | 939.571524646204 | Gambia |
| 2017 | 1347.7989109178 | 0.387273644458255 | 316.86659694852 | 1030.93231396928 | Gambia |
| 2018 | 1275.35403471797 | 0.393155679437306 | 285.167325645763 | 990.186709072207 | Gambia |
| 2019 | 1228.27462565509 | 0.39918978005783 | 266.47327735877 | 961.80134829632 | Gambia |
| 2020 | 1094.53122682569 | 0.40452419422916 | 239.056580089446 | 855.474646736244 | Gambia |
| 2021 | 991.67294130832 | 0.40971415951073 | 223.045390181369 | 768.627551126951 | Gambia |
| 1990 | 3277.80871283499 | 0.373112004793066 | 355.290564839633 | 2922.51814799536 | Ghana |
| 1991 | 3045.27972627881 | 0.37923925587052 | 346.367536941494 | 2698.91218933732 | Ghana |
| 1992 | 2870.38722145995 | 0.384856767297978 | 320.045598804434 | 2550.34162265552 | Ghana |
| 1993 | 2726.30460174938 | 0.389968494182018 | 289.198059449707 | 2437.10654229967 | Ghana |
| 1994 | 2594.13953403915 | 0.394953662708371 | 285.072828296158 | 2309.06670574299 | Ghana |
| 1995 | 2469.41273823579 | 0.39993020380395 | 261.901276891005 | 2207.51146134478 | Ghana |
| 1996 | 2337.01137754876 | 0.405003046925554 | 236.027790437338 | 2100.98358711142 | Ghana |
| 1997 | 2188.10103880854 | 0.410038073297741 | 216.765025206759 | 1971.33601360178 | Ghana |
| 1998 | 2092.01249919064 | 0.414888532103537 | 215.237017708176 | 1876.77548148246 | Ghana |
| 1999 | 1969.19875662032 | 0.419755496390431 | 194.555351585091 | 1774.64340503523 | Ghana |
| 2000 | 1885.17675379491 | 0.424725211650337 | 179.273891899874 | 1705.90286189504 | Ghana |
| 2001 | 1779.96798701793 | 0.42941184646569 | 137.640307265575 | 1642.32767975235 | Ghana |
| 2002 | 1705.3161412398 | 0.434104128009022 | 131.868509167282 | 1573.44763207252 | Ghana |
| 2003 | 1732.10109784238 | 0.439118212630728 | 127.404177140362 | 1604.69692070202 | Ghana |
| 2004 | 1671.90445715533 | 0.444236977254313 | 111.803521619672 | 1560.10093553566 | Ghana |
| 2005 | 1625.89706985673 | 0.449360019930072 | 107.267063326791 | 1518.63000652994 | Ghana |
| 2006 | 1552.97990212604 | 0.454692737065749 | 105.765538725585 | 1447.21436340046 | Ghana |
| 2007 | 1440.54392395826 | 0.460606245250364 | 93.0583032057332 | 1347.48562075253 | Ghana |
| 2008 | 1201.03170508189 | 0.467259066649708 | 86.0807864410198 | 1114.95091864087 | Ghana |
| 2009 | 1076.88427866745 | 0.473848047331171 | 80.6740821169502 | 996.2101965505 | Ghana |
| 2010 | 966.25410803238 | 0.480777891769648 | 77.2743165789488 | 888.979791453431 | Ghana |
| 2011 | 928.63172515678 | 0.489020973808153 | 75.6480267537922 | 852.983698402988 | Ghana |
| 2012 | 744.96701349708 | 0.497513931634325 | 21.0887567197597 | 723.87825677732 | Ghana |
| 2013 | 687.72137811469 | 0.506004646555731 | 16.2432734527659 | 671.478104661924 | Ghana |
| 2014 | 692.03720400761 | 0.514119061082341 | 15.7879667535239 | 676.249237254086 | Ghana |
| 2015 | 664.21284391933 | 0.521539202423625 | 15.1673381375833 | 649.045505781747 | Ghana |
| 2016 | 607.55423577312 | 0.528629585500254 | 15.1186780819328 | 592.435557691187 | Ghana |
| 2017 | 579.71622457821 | 0.536169940006201 | 15.0585934794605 | 564.65763109875 | Ghana |
| 2018 | 514.96243325099 | 0.543840539336404 | 15.0094513142166 | 499.952981936773 | Ghana |
| 2019 | 458.82109060428 | 0.551632026781383 | 14.0443737925254 | 444.776716811755 | Ghana |
| 2020 | 440.31777790412 | 0.558462173287852 | 12.4839523037069 | 427.833825600413 | Ghana |
| 2021 | 391.66025491599 | 0.564930389658009 | 10.5368886119801 | 381.12336630401 | Ghana |
| 1990 | 13479.5504700259 | 0.17829542065613 | 3022.87236376438 | 10456.6781062615 | Guinea |
| 1991 | 12746.586145698 | 0.181459390831103 | 3015.79308755238 | 9730.79305814562 | Guinea |
| 1992 | 12016.029249826 | 0.184485016316931 | 2904.01142684239 | 9112.01782298361 | Guinea |
| 1993 | 11512.1350014919 | 0.187686241926773 | 2893.2557727866 | 8618.8792287053 | Guinea |
| 1994 | 11567.3257687767 | 0.191128836868019 | 2842.83903534694 | 8724.48673342976 | Guinea |
| 1995 | 11367.803363054 | 0.194573244617025 | 2842.69562756137 | 8525.10773549263 | Guinea |
| 1996 | 10126.3655957488 | 0.198633755485464 | 2761.43160211888 | 7364.93399362992 | Guinea |
| 1997 | 9523.49364175769 | 0.203280448621307 | 2758.44439411218 | 6765.04924764551 | Guinea |
| 1998 | 8918.39340175689 | 0.208003382623507 | 2567.6209658709 | 6350.77243588599 | Guinea |
| 1999 | 8091.17225440886 | 0.212412705488692 | 2313.80355758446 | 5777.3686968244 | Guinea |
| 2000 | 7409.76378307293 | 0.216649791599883 | 2348.12013753178 | 5061.64364554115 | Guinea |
| 2001 | 6803.70040644604 | 0.221090940982099 | 2082.70812143949 | 4720.99228500655 | Guinea |
| 2002 | 6363.21326082098 | 0.225898779303173 | 2113.09470565971 | 4250.11855516127 | Guinea |
| 2003 | 6089.65515021945 | 0.230522593906606 | 1915.19647852137 | 4174.45867169808 | Guinea |
| 2004 | 6176.32045934568 | 0.235134970165926 | 1872.79905383398 | 4303.5214055117 | Guinea |
| 2005 | 5838.55002701354 | 0.239691845090143 | 1702.95137257048 | 4135.59865444306 | Guinea |
| 2006 | 5594.32136758978 | 0.24401012651719 | 1639.51452451982 | 3954.80684306996 | Guinea |
| 2007 | 5205.25789209701 | 0.248471690169451 | 1483.31504176896 | 3721.94285032805 | Guinea |
| 2008 | 4726.33422096473 | 0.253167688592502 | 1502.82628479424 | 3223.50793617049 | Guinea |
| 2009 | 4166.73857761718 | 0.257552785014134 | 1350.23648395759 | 2816.5020936596 | Guinea |
| 2010 | 3951.59499298605 | 0.262405948891134 | 1344.832923335 | 2606.76206965105 | Guinea |
| 2011 | 3592.00789070566 | 0.267624599664508 | 1232.19416591435 | 2359.81372479131 | Guinea |
| 2012 | 3288.4802894634 | 0.273428318991395 | 1217.65748541933 | 2070.82280404407 | Guinea |
| 2013 | 3032.93410292484 | 0.279600444049448 | 1158.07744250347 | 1874.85666042137 | Guinea |
| 2014 | 2762.68094621401 | 0.2859612643771 | 1104.29043064646 | 1658.39051556755 | Guinea |
| 2015 | 2560.43100036722 | 0.292363614463187 | 1033.00654935027 | 1527.42445101695 | Guinea |
| 2016 | 2375.85876855607 | 0.299112412084657 | 989.768025165197 | 1386.09074339087 | Guinea |
| 2017 | 2117.99296994032 | 0.306881461581969 | 883.895588731955 | 1234.09738120836 | Guinea |
| 2018 | 1863.54164161855 | 0.314839587576224 | 822.312102610161 | 1041.22953900839 | Guinea |
| 2019 | 1662.50492944261 | 0.322839848435435 | 742.006537154874 | 920.498392287736 | Guinea |
| 2020 | 1486.4550474473 | 0.329877997039031 | 668.76332538624 | 817.69172206106 | Guinea |
| 2021 | 1227.9372464857 | 0.33640129335069 | 650.184968291707 | 577.752278193993 | Guinea |
| 1990 | 11880.9666391765 | 0.207614838569111 | 2577.00682507522 | 9303.95981410128 | Guinea-Bissau |
| 1991 | 11393.8960363517 | 0.212505984149041 | 2319.14981041077 | 9074.74622594093 | Guinea-Bissau |
| 1992 | 10960.9578205133 | 0.217026088469754 | 2323.45704210406 | 8637.50077840924 | Guinea-Bissau |
| 1993 | 10527.7755003682 | 0.221678978443034 | 2096.35076765904 | 8431.42473270916 | Guinea-Bissau |
| 1994 | 11902.788515101 | 0.226434932988846 | 2065.22266637727 | 9837.56584872373 | Guinea-Bissau |
| 1995 | 9615.17640733755 | 0.231223526184368 | 1890.66479758639 | 7724.51160975116 | Guinea-Bissau |
| 1996 | 17787.4869443257 | 0.236106373654915 | 1897.40965148751 | 15890.0772928382 | Guinea-Bissau |
| 1997 | 15567.6753836435 | 0.241360583940643 | 1662.83585482422 | 13904.8395288193 | Guinea-Bissau |
| 1998 | 8232.2811033254 | 0.244371797123174 | 1666.83076780089 | 6565.45033552451 | Guinea-Bissau |
| 1999 | 7782.88611831087 | 0.24821731725385 | 1523.14280990239 | 6259.74330840848 | Guinea-Bissau |
| 2000 | 7922.57466835206 | 0.252026519139868 | 1487.80566661458 | 6434.76900173748 | Guinea-Bissau |
| 2001 | 7897.0560448816 | 0.255786767601624 | 1486.59903408588 | 6410.45701079572 | Guinea-Bissau |
| 2002 | 7712.1382875558 | 0.259321815568398 | 1348.82704056695 | 6363.31124698885 | Guinea-Bissau |
| 2003 | 7351.02559259361 | 0.262747723500015 | 1340.71856110756 | 6010.30703148605 | Guinea-Bissau |
| 2004 | 7003.88719660159 | 0.26627173587343 | 1321.14614819126 | 5682.74104841033 | Guinea-Bissau |
| 2005 | 9353.53612107276 | 0.270056131535634 | 1232.59907735516 | 8120.9370437176 | Guinea-Bissau |
| 2006 | 6000.12420384618 | 0.273803986556405 | 1213.86847946808 | 4786.2557243781 | Guinea-Bissau |
| 2007 | 6572.36018880369 | 0.277805016731205 | 1156.2046545173 | 5416.15553428639 | Guinea-Bissau |
| 2008 | 7536.14424886374 | 0.282151501527761 | 1157.52859427216 | 6378.61565459158 | Guinea-Bissau |
| 2009 | 5352.38032286372 | 0.286423345485565 | 1108.0002129676 | 4244.38010989612 | Guinea-Bissau |
| 2010 | 4830.65680960316 | 0.291141639242703 | 1089.17676768525 | 3741.48004191791 | Guinea-Bissau |
| 2011 | 4192.811735865 | 0.29668296654448 | 1046.705561208 | 3146.106174657 | Guinea-Bissau |
| 2012 | 3201.54804344699 | 0.301562341875562 | 967.942502009752 | 2233.60554143724 | Guinea-Bissau |
| 2013 | 3076.96759874482 | 0.30660200942877 | 887.75081522211 | 2189.21678352271 | Guinea-Bissau |
| 2014 | 2657.63777916049 | 0.311602905000269 | 866.327178869256 | 1791.31060029123 | Guinea-Bissau |
| 2015 | 2323.11133351546 | 0.31709001451773 | 780.073826970376 | 1543.03750654508 | Guinea-Bissau |
| 2016 | 2262.43026235761 | 0.32297967010132 | 741.245245436156 | 1521.18501692145 | Guinea-Bissau |
| 2017 | 2046.74306858927 | 0.329326492160232 | 721.589486051439 | 1325.15358253783 | Guinea-Bissau |
| 2018 | 1758.73442453798 | 0.335667832836675 | 650.229646851287 | 1108.50477768669 | Guinea-Bissau |
| 2019 | 1550.20520094045 | 0.342156114875433 | 574.13455451862 | 976.07064642183 | Guinea-Bissau |
| 2020 | 1415.97011866487 | 0.347798997943486 | 573.179897450075 | 842.790221214795 | Guinea-Bissau |
| 2021 | 1270.12001885392 | 0.353109621364536 | 557.748440029403 | 712.371578824517 | Guinea-Bissau |
| 1990 | 19231.4246673522 | 0.235296846955071 | 1864.43752679191 | 17366.9871405603 | Liberia |
| 1991 | 23308.2501424358 | 0.238212217752062 | 1648.45651619441 | 21659.7936262414 | Liberia |
| 1992 | 20822.4716713313 | 0.23828331265798 | 1681.56321241785 | 19140.9084589134 | Liberia |
| 1993 | 17707.9083353403 | 0.235984406116034 | 1887.40917313286 | 15820.4991622074 | Liberia |
| 1994 | 17944.2574741545 | 0.232656773774217 | 1917.08009389436 | 16027.1773802601 | Liberia |
| 1995 | 19174.1589739808 | 0.228424478219389 | 1894.62751572637 | 17279.5314582544 | Liberia |
| 1996 | 18781.8468301394 | 0.222682591493435 | 2096.40015422634 | 16685.4466759131 | Liberia |
| 1997 | 17251.285591742 | 0.219672764660528 | 2089.1903284534 | 15162.0952632886 | Liberia |
| 1998 | 16481.7423381678 | 0.219053854841174 | 2284.37094613385 | 14197.371392034 | Liberia |
| 1999 | 15554.239549781 | 0.222212867280137 | 2083.98344596059 | 13470.2561038204 | Liberia |
| 2000 | 14582.2947699024 | 0.231173361787615 | 1871.81273948041 | 12710.482030422 | Liberia |
| 2001 | 13157.3498709839 | 0.239594386141111 | 1689.88363129176 | 11467.4662396921 | Liberia |
| 2002 | 11498.2408975341 | 0.248311971373795 | 1500.62067587588 | 9997.62022165822 | Liberia |
| 2003 | 10606.4913557603 | 0.251595457910319 | 1494.6709388682 | 9111.8204168921 | Liberia |
| 2004 | 9598.59382603404 | 0.255179610402999 | 1524.59114031965 | 8074.0026857144 | Liberia |
| 2005 | 9306.27550428886 | 0.258967990380421 | 1353.15582956364 | 7953.11967472522 | Liberia |
| 2006 | 8196.26450689355 | 0.262897258459796 | 1349.40587895352 | 6846.85862794003 | Liberia |
| 2007 | 7107.89210981478 | 0.267239568498419 | 1245.59258429276 | 5862.29952552202 | Liberia |
| 2008 | 6606.18606079043 | 0.271853595673486 | 1221.98647755993 | 5384.1995832305 | Liberia |
| 2009 | 6816.88603219676 | 0.276804110528483 | 1163.87335847169 | 5653.01267372507 | Liberia |
| 2010 | 6799.14012271439 | 0.28223575229287 | 1154.20314829909 | 5644.9369744153 | Liberia |
| 2011 | 6585.9560640133 | 0.288015144991527 | 1116.1420300114 | 5469.8140340019 | Liberia |
| 2012 | 6436.90333181408 | 0.294854315315019 | 1034.658744559 | 5402.24458725508 | Liberia |
| 2013 | 6021.32503347739 | 0.302985656345751 | 959.166516667224 | 5062.15851681017 | Liberia |
| 2014 | 5281.8552067414 | 0.310955439531843 | 864.317694675533 | 4417.53751206587 | Liberia |
| 2015 | 4729.35457570714 | 0.318485165511089 | 781.581226953386 | 3947.77334875375 | Liberia |
| 2016 | 4075.03744690582 | 0.325085173839682 | 722.068774353985 | 3352.96867255183 | Liberia |
| 2017 | 3550.73009153211 | 0.331527785784429 | 648.921915360887 | 2901.80817617122 | Liberia |
| 2018 | 3212.25138725025 | 0.337784577514724 | 593.800930288455 | 2618.45045696179 | Liberia |
| 2019 | 3021.95864132658 | 0.343403262226321 | 567.939209361898 | 2454.01943196468 | Liberia |
| 2020 | 2745.11133536055 | 0.348133889761756 | 569.047261417738 | 2176.06407394281 | Liberia |
| 2021 | 2501.82940658111 | 0.352442451643965 | 553.928898689964 | 1947.90050789115 | Liberia |
| 1990 | 12518.8326179578 | 0.126526427537221 | 4177.92064227168 | 8340.91197568612 | Mali |
| 1991 | 12083.2377060176 | 0.129128150017575 | 4161.04303635441 | 7922.19466966319 | Mali |
| 1992 | 11724.3353984261 | 0.131537448786118 | 4178.07048674926 | 7546.26491167684 | Mali |
| 1993 | 11300.9053661293 | 0.134177054462465 | 4205.21206991609 | 7095.69329621321 | Mali |
| 1994 | 10867.843500008 | 0.136937829291313 | 4165.68784398442 | 6702.15565602358 | Mali |
| 1995 | 10607.6447490659 | 0.139866225457785 | 4196.29640903153 | 6411.34834003437 | Mali |
| 1996 | 10528.9475310226 | 0.143078915935706 | 4189.72203990227 | 6339.22549112033 | Mali |
| 1997 | 9153.88990417389 | 0.146414326236527 | 4183.42679646169 | 4970.4631077122 | Mali |
| 1998 | 8514.93747762526 | 0.149986729501612 | 4152.68291320451 | 4362.25456442075 | Mali |
| 1999 | 8126.39695113637 | 0.153737335688568 | 4141.61809244191 | 3984.77885869446 | Mali |
| 2000 | 7900.33917200629 | 0.157190027521405 | 4169.06470794266 | 3731.27446406363 | Mali |
| 2001 | 7490.55489490361 | 0.161344447555084 | 4178.55424921131 | 3312.0006456923 | Mali |
| 2002 | 8015.64131445476 | 0.165420695773092 | 4182.2603814513 | 3833.38093300346 | Mali |
| 2003 | 7792.55734273769 | 0.169733502039948 | 4168.06828030222 | 3624.48906243547 | Mali |
| 2004 | 7425.81258296719 | 0.173779552322233 | 4121.46041519481 | 3304.35216777238 | Mali |
| 2005 | 7063.01233343725 | 0.178029844366254 | 3019.62116514646 | 4043.39116829079 | Mali |
| 2006 | 6597.33993359083 | 0.182452468201007 | 3026.25024114576 | 3571.08969244507 | Mali |
| 2007 | 5848.71972871652 | 0.186912994942121 | 2890.6519372088 | 2958.06779150772 | Mali |
| 2008 | 5479.9221238957 | 0.191536745613522 | 2852.426483848 | 2627.4956400477 | Mali |
| 2009 | 4977.59732953519 | 0.196537650861103 | 2845.43656589647 | 2132.16076363872 | Mali |
| 2010 | 4441.63034306058 | 0.201803535998378 | 2737.86189282295 | 1703.76845023764 | Mali |
| 2011 | 4002.87605073924 | 0.207097221274123 | 2573.30063034422 | 1429.57542039502 | Mali |
| 2012 | 3811.4870782896 | 0.212465060096693 | 2329.51595225645 | 1481.97112603315 | Mali |
| 2013 | 3487.67621550899 | 0.217993867180652 | 2346.1441832906 | 1141.53203221839 | Mali |
| 2014 | 3031.26616298353 | 0.223729979734488 | 2106.92355859825 | 924.342604385281 | Mali |
| 2015 | 2707.21027971748 | 0.229826262811848 | 1866.75456090832 | 840.455718809157 | Mali |
| 2016 | 2720.05948818393 | 0.236186231331436 | 1890.4679360373 | 829.591552146625 | Mali |
| 2017 | 2604.27049717881 | 0.242726134546644 | 1658.82617710415 | 945.444320074665 | Mali |
| 2018 | 2426.52773937483 | 0.249419730729374 | 1492.47622821489 | 934.051511159936 | Mali |
| 2019 | 2229.4705572797 | 0.256250745993336 | 1479.72853423432 | 749.742023045382 | Mali |
| 2020 | 2021.53141037323 | 0.262629218759395 | 1341.7834020582 | 679.748008315026 | Mali |
| 2021 | 1770.18411638911 | 0.268579940925684 | 1216.99927468369 | 553.184841705425 | Mali |
| 1990 | 6971.22235722885 | 0.33578094163222 | 649.551548952757 | 6321.67080827609 | Mauritania |
| 1991 | 6676.68823336344 | 0.341135174811723 | 576.911897565277 | 6099.77633579816 | Mauritania |
| 1992 | 6415.70556903068 | 0.346531288170585 | 570.969903211142 | 5844.73566581954 | Mauritania |
| 1993 | 6171.89216245198 | 0.35217657188477 | 554.860985226015 | 5617.03117722597 | Mauritania |
| 1994 | 5681.81561855559 | 0.357585911567431 | 502.904888132279 | 5178.91073042331 | Mauritania |
| 1995 | 5374.04370084093 | 0.363037695183683 | 466.169488907407 | 4907.87421193352 | Mauritania |
| 1996 | 5710.86064104056 | 0.368508328412644 | 424.090616180407 | 5286.77002486015 | Mauritania |
| 1997 | 5404.58995378552 | 0.373021338681094 | 357.324727181877 | 5047.26522660364 | Mauritania |
| 1998 | 5397.73852033833 | 0.377080249599675 | 349.82363491393 | 5047.9148854244 | Mauritania |
| 1999 | 5157.73235404746 | 0.381112297471017 | 322.23494427284 | 4835.49740977462 | Mauritania |
| 2000 | 5109.35516136033 | 0.384205379717954 | 318.482176455329 | 4790.872984905 | Mauritania |
| 2001 | 4930.04839223058 | 0.386487415894351 | 315.881077743523 | 4614.16731448706 | Mauritania |
| 2002 | 4795.26090619313 | 0.388564477604617 | 288.795247888019 | 4506.46565830511 | Mauritania |
| 2003 | 4473.663838892 | 0.39110487913777 | 283.793503830687 | 4189.87033506131 | Mauritania |
| 2004 | 4273.46281531627 | 0.394012933734168 | 285.363574479846 | 3988.09924083642 | Mauritania |
| 2005 | 4498.31272409047 | 0.39749910452189 | 258.944738201581 | 4239.36798588889 | Mauritania |
| 2006 | 4164.65609761308 | 0.403159466975078 | 239.493397547828 | 3925.16270006525 | Mauritania |
| 2007 | 3717.21793906196 | 0.408377122155099 | 238.896150966898 | 3478.32178809506 | Mauritania |
| 2008 | 3296.13537889681 | 0.413233621211221 | 215.188066640862 | 3080.94731225595 | Mauritania |
| 2009 | 3192.96786187918 | 0.4179106088283 | 194.509882221981 | 2998.4579796572 | Mauritania |
| 2010 | 2791.95067412967 | 0.423202436351832 | 178.627566815991 | 2613.32310731368 | Mauritania |
| 2011 | 2529.41564586697 | 0.428867289887467 | 165.667033347402 | 2363.74861251957 | Mauritania |
| 2012 | 2451.95900857252 | 0.434724375629838 | 130.168475234909 | 2321.79053333761 | Mauritania |
| 2013 | 2218.16718995066 | 0.441068910286714 | 121.02849245157 | 2097.13869749909 | Mauritania |
| 2014 | 1793.36308122569 | 0.447653881356557 | 111.017650517494 | 1682.3454307082 | Mauritania |
| 2015 | 1592.51341332331 | 0.454523487871252 | 106.876757484673 | 1485.63665583864 | Mauritania |
| 2016 | 1391.94772463911 | 0.461269544556856 | 91.0223868442106 | 1300.9253377949 | Mauritania |
| 2017 | 1289.53522871981 | 0.468452365279582 | 85.7460407388768 | 1203.78918798093 | Mauritania |
| 2018 | 1153.36002173989 | 0.475839507248469 | 78.7153244711768 | 1074.64469726871 | Mauritania |
| 2019 | 1141.37514567831 | 0.483767737935156 | 76.3201449862711 | 1065.05500069204 | Mauritania |
| 2020 | 1018.78858447834 | 0.491365602592056 | 75.0590887159181 | 943.729495762422 | Mauritania |
| 2021 | 974.88008882777 | 0.498945100442996 | 17.8182694617233 | 957.061819366047 | Mauritania |
| 1990 | 44227.1567845364 | 0.0808684798233195 | 4149.31267975353 | 40077.8441047829 | Niger |
| 1991 | 44272.094720205 | 0.0828482077018824 | 4170.19805918128 | 40101.8966610237 | Niger |
| 1992 | 43491.6077247014 | 0.0847715776568405 | 4169.64662695378 | 39321.9610977476 | Niger |
| 1993 | 42566.5286804235 | 0.0866964903938328 | 4168.76532825498 | 38397.7633521685 | Niger |
| 1994 | 41282.8341419744 | 0.0886087332313997 | 4174.70800716493 | 37108.1261348095 | Niger |
| 1995 | 40136.82142379 | 0.090473708405212 | 4187.22864139856 | 35949.5927823914 | Niger |
| 1996 | 38390.3353631511 | 0.09234635941853 | 4210.21906232433 | 34180.1163008268 | Niger |
| 1997 | 36620.371651177 | 0.0942191148661978 | 4190.10252267719 | 32430.2691284998 | Niger |
| 1998 | 34908.3946843973 | 0.0965451528067399 | 4157.83087655324 | 30750.5638078441 | Niger |
| 1999 | 33404.428356773 | 0.0988136856452598 | 4168.81253219727 | 29235.6158245757 | Niger |
| 2000 | 32439.60007864 | 0.100886948404838 | 4189.82850651106 | 28249.7715721289 | Niger |
| 2001 | 31273.7478891709 | 0.103271776631217 | 4210.86316057358 | 27062.8847285973 | Niger |
| 2002 | 29411.2929115999 | 0.105741108731078 | 4173.73504148748 | 25237.5578701124 | Niger |
| 2003 | 28324.8833300171 | 0.108322891429361 | 4166.38461066803 | 24158.4987193491 | Niger |
| 2004 | 25546.4390056716 | 0.110764495003726 | 4163.49190216267 | 21382.9471035089 | Niger |
| 2005 | 26286.4676307507 | 0.11345202277039 | 4143.79980316679 | 22142.6678275839 | Niger |
| 2006 | 23504.7043722999 | 0.116247074448783 | 4161.59394848546 | 19343.1104238144 | Niger |
| 2007 | 21191.0809883565 | 0.11897162605295 | 4166.07650689174 | 17025.0044814648 | Niger |
| 2008 | 17316.2740480969 | 0.121957786240242 | 4190.19773673039 | 13126.0763113665 | Niger |
| 2009 | 15849.2511543996 | 0.124829381330847 | 4154.54513863257 | 11694.706015767 | Niger |
| 2010 | 14319.3525083421 | 0.127952995735601 | 4177.9826888796 | 10141.3698194625 | Niger |
| 2011 | 13621.0883162856 | 0.130958067074432 | 4160.91518326132 | 9460.17313302428 | Niger |
| 2012 | 12580.4169873197 | 0.134469718123206 | 4149.40878560954 | 8431.00820171016 | Niger |
| 2013 | 12072.1794624501 | 0.137978170438014 | 4127.48048243045 | 7944.69898001965 | Niger |
| 2014 | 10385.1997498357 | 0.141731738162587 | 4147.41411037148 | 6237.78563946422 | Niger |
| 2015 | 9282.6507838058 | 0.14544050326966 | 4145.81461919027 | 5136.83616461553 | Niger |
| 2016 | 8905.02458859479 | 0.149200109150915 | 4182.83571046595 | 4722.18887812884 | Niger |
| 2017 | 8433.17249279472 | 0.152959169593186 | 4174.69163822343 | 4258.48085457129 | Niger |
| 2018 | 7628.04523772154 | 0.15687519554195 | 4159.67293719683 | 3468.37230052471 | Niger |
| 2019 | 7047.18296384564 | 0.160889483579043 | 4196.05968265942 | 2851.12328118622 | Niger |
| 2020 | 6832.53723430078 | 0.164539264581005 | 4173.61146333193 | 2658.92577096885 | Niger |
| 2021 | 6349.54215446167 | 0.168072773803995 | 4163.41386490149 | 2186.12828956018 | Niger |
| 1990 | 26854.3678322755 | 0.305868046855462 | 961.471476343917 | 25892.8963559316 | Nigeria |
| 1991 | 27858.368734853 | 0.308489225313095 | 878.563130092037 | 26979.805604761 | Nigeria |
| 1992 | 24537.8859973951 | 0.311234052054088 | 872.811553577336 | 23665.0744438178 | Nigeria |
| 1993 | 23473.2050032588 | 0.313983059877659 | 863.938183820131 | 22609.2668194387 | Nigeria |
| 1994 | 22812.5494960651 | 0.316747446775232 | 781.736330417954 | 22030.8131656471 | Nigeria |
| 1995 | 22349.6021226207 | 0.319637438693347 | 772.570292718429 | 21577.0318299023 | Nigeria |
| 1996 | 22275.117223958 | 0.323137684748282 | 726.880967347857 | 21548.2362566101 | Nigeria |
| 1997 | 21786.8428892557 | 0.326690552886558 | 720.754770590886 | 21066.0881186648 | Nigeria |
| 1998 | 21503.8498084751 | 0.330263851422919 | 668.981984464235 | 20834.8678240109 | Nigeria |
| 1999 | 21362.0018427064 | 0.333936499661916 | 658.354322896271 | 20703.6475198101 | Nigeria |
| 2000 | 21012.2022260266 | 0.338201742797398 | 588.325597467783 | 20423.8766285588 | Nigeria |
| 2001 | 20610.8772208535 | 0.343029750745818 | 569.045221331816 | 20041.8319995217 | Nigeria |
| 2002 | 20053.1901215541 | 0.349092528396007 | 554.503215735133 | 19498.686905819 | Nigeria |
| 2003 | 19256.2963599308 | 0.355911392103181 | 555.408109643223 | 18700.8882502876 | Nigeria |
| 2004 | 18643.9070711692 | 0.36378081411686 | 457.669200395288 | 18186.2378707739 | Nigeria |
| 2005 | 17863.4251691622 | 0.372583553203769 | 354.937426102477 | 17508.4877430597 | Nigeria |
| 2006 | 17873.8561023599 | 0.381026040235948 | 320.749639652425 | 17553.1064627075 | Nigeria |
| 2007 | 17603.528626925 | 0.38977964917576 | 289.244659046483 | 17314.2839678785 | Nigeria |
| 2008 | 16900.5812510442 | 0.398037706162251 | 255.216349903272 | 16645.3649011409 | Nigeria |
| 2009 | 16367.3018548386 | 0.40635330747071 | 238.017087718278 | 16129.2847671203 | Nigeria |
| 2010 | 15863.8143611798 | 0.414997747961223 | 217.274871804508 | 15646.5394893753 | Nigeria |
| 2011 | 14477.1143518314 | 0.42335191474477 | 177.566860653759 | 14299.5474911776 | Nigeria |
| 2012 | 13377.6513560885 | 0.431780375863827 | 135.179216037744 | 13242.4721400508 | Nigeria |
| 2013 | 12433.63669403 | 0.440124663848588 | 127.52378471912 | 12306.1129093109 | Nigeria |
| 2014 | 11595.5618353654 | 0.44876682284048 | 108.177847332968 | 11487.3839880324 | Nigeria |
| 2015 | 10733.5963459119 | 0.457240991469025 | 99.1904894432794 | 10634.4058564686 | Nigeria |
| 2016 | 10172.1123206321 | 0.465183780994652 | 92.4142208018918 | 10079.6980998302 | Nigeria |
| 2017 | 9409.079467706 | 0.472972868984332 | 79.2478816761015 | 9329.8315860299 | Nigeria |
| 2018 | 8751.4849329922 | 0.480844413426724 | 77.4443105489665 | 8674.04062244323 | Nigeria |
| 2019 | 8077.87001554446 | 0.488787361344695 | 75.0603425720712 | 8002.80967297239 | Nigeria |
| 2020 | 6940.96330881563 | 0.496204741160216 | 46.0629957443269 | 6894.9003130713 | Nigeria |
| 2021 | 6577.40043074954 | 0.503390832763244 | 16.178428405001 | 6561.22200234454 | Nigeria |
| 1990 | 8534.27696386978 | 0.3095428523248 | 872.842544800699 | 7661.43441906908 | Sao Tome and Principe |
| 1991 | 7593.00955169363 | 0.310601028486155 | 875.736415106053 | 6717.27313658758 | Sao Tome and Principe |
| 1992 | 7345.85940225993 | 0.3115154473454 | 873.05014368801 | 6472.80925857192 | Sao Tome and Principe |
| 1993 | 7135.18641035764 | 0.31274355801833 | 867.358395320791 | 6267.82801503685 | Sao Tome and Principe |
| 1994 | 6944.58075917132 | 0.314261087139278 | 867.779346475785 | 6076.80141269553 | Sao Tome and Principe |
| 1995 | 6364.34376436241 | 0.315843919852344 | 780.031257475988 | 5584.31250688642 | Sao Tome and Principe |
| 1996 | 5907.6287286791 | 0.317729288379101 | 767.665231113761 | 5139.96349756534 | Sao Tome and Principe |
| 1997 | 5318.34182353983 | 0.319795669969502 | 778.389737643599 | 4539.95208589623 | Sao Tome and Principe |
| 1998 | 4801.14761113192 | 0.322519218688325 | 732.831422140125 | 4068.3161889918 | Sao Tome and Principe |
| 1999 | 4507.80581821724 | 0.325651915603451 | 721.453233635528 | 3786.35258458171 | Sao Tome and Principe |
| 2000 | 3163.42929621188 | 0.329460109182343 | 719.321160817896 | 2444.10813539398 | Sao Tome and Principe |
| 2001 | 2898.81494233518 | 0.334484786134682 | 644.653151514695 | 2254.16179082049 | Sao Tome and Principe |
| 2002 | 2497.58717716265 | 0.340302836742805 | 587.071382745212 | 1910.51579441744 | Sao Tome and Principe |
| 2003 | 2200.48281490854 | 0.346851981499259 | 573.765700460482 | 1626.71711444806 | Sao Tome and Principe |
| 2004 | 1936.21637358371 | 0.353722383532282 | 554.697385658619 | 1381.51898792509 | Sao Tome and Principe |
| 2005 | 3572.07742312455 | 0.361105881091861 | 489.731998249059 | 3082.34542487549 | Sao Tome and Principe |
| 2006 | 2715.11143431754 | 0.369413007194338 | 405.582268278279 | 2309.52916603926 | Sao Tome and Principe |
| 2007 | 1996.97048636473 | 0.377813517075578 | 350.42211499268 | 1646.54837137205 | Sao Tome and Principe |
| 2008 | 2067.80244881724 | 0.386688943651063 | 318.248046786557 | 1749.55440203068 | Sao Tome and Principe |
| 2009 | 1598.7757312209 | 0.39582123767217 | 262.082852826575 | 1336.69287839433 | Sao Tome and Principe |
| 2010 | 1540.65311351703 | 0.405530666154327 | 236.341501380939 | 1304.31161213609 | Sao Tome and Principe |
| 2011 | 1131.35294989872 | 0.41578372764052 | 199.878408442056 | 931.474541456664 | Sao Tome and Principe |
| 2012 | 857.83983829285 | 0.425721314870657 | 179.127343387967 | 678.712494904883 | Sao Tome and Principe |
| 2013 | 787.29976551888 | 0.435592459999619 | 124.721784153043 | 662.577981365837 | Sao Tome and Principe |
| 2014 | 825.36426955325 | 0.44514623672643 | 112.326391573404 | 713.037877979846 | Sao Tome and Principe |
| 2015 | 728.27048086516 | 0.454051473195403 | 107.279679451581 | 620.990801413579 | Sao Tome and Principe |
| 2016 | 552.31695987041 | 0.463230081728275 | 91.1305841430986 | 461.186375727311 | Sao Tome and Principe |
| 2017 | 493.51016342963 | 0.47217092996608 | 83.639034380177 | 409.871129049453 | Sao Tome and Principe |
| 2018 | 459.94807783335 | 0.481098204433028 | 77.0322659823867 | 382.915811850963 | Sao Tome and Principe |
| 2019 | 422.1992324969 | 0.489838229899105 | 74.5851351029727 | 347.614097393927 | Sao Tome and Principe |
| 2020 | 386.97093794236 | 0.497861408762174 | 18.278533121527 | 368.692404820833 | Sao Tome and Principe |
| 2021 | 339.63718059863 | 0.505413746708186 | 16.2572464067664 | 323.379934191864 | Sao Tome and Principe |
| 1990 | 12564.9434329665 | 0.238047613296703 | 1666.95875845492 | 10897.9846745116 | Senegal |
| 1991 | 11943.0393945392 | 0.244482178069769 | 1668.69487201216 | 10274.344522527 | Senegal |
| 1992 | 11347.1332784096 | 0.250416531795468 | 1495.03972615576 | 9852.09355225384 | Senegal |
| 1993 | 10881.5737566613 | 0.255767963935604 | 1481.24598109888 | 9400.32777556242 | Senegal |
| 1994 | 10440.551154416 | 0.260379997494734 | 1355.94837214418 | 9084.60278227182 | Senegal |
| 1995 | 9903.74588309568 | 0.264438773208067 | 1335.0466837881 | 8568.69919930758 | Senegal |
| 1996 | 9479.44441667982 | 0.268318964653468 | 1237.52251536377 | 8241.92190131605 | Senegal |
| 1997 | 8718.26378159532 | 0.272072875469797 | 1237.9135076197 | 7480.35027397562 | Senegal |
| 1998 | 8290.64980680498 | 0.275995656114005 | 1159.27750594542 | 7131.37230085956 | Senegal |
| 1999 | 7918.46091396525 | 0.280111918035525 | 1154.93911192639 | 6763.52180203886 | Senegal |
| 2000 | 7093.22569914547 | 0.283784902973132 | 1158.76407143072 | 5934.46162771475 | Senegal |
| 2001 | 8101.57357892873 | 0.287451899345797 | 1112.34727496556 | 6989.22630396317 | Senegal |
| 2002 | 7690.29936425967 | 0.291046287851898 | 1094.40122670059 | 6595.89813755908 | Senegal |
| 2003 | 7090.31185182428 | 0.294887172086492 | 1031.33530351801 | 6058.97654830627 | Senegal |
| 2004 | 7573.63393158605 | 0.298984520918874 | 993.027088204952 | 6580.6068433811 | Senegal |
| 2005 | 7394.89211611388 | 0.30325720904165 | 962.960210111932 | 6431.93190600195 | Senegal |
| 2006 | 6259.32786564529 | 0.307633910231713 | 874.985645537771 | 5384.34222010752 | Senegal |
| 2007 | 5684.06914638248 | 0.312570297567491 | 863.081447930958 | 4820.98769845152 | Senegal |
| 2008 | 5103.53547517606 | 0.317894088643487 | 779.559775533652 | 4323.97569964241 | Senegal |
| 2009 | 4531.24754646104 | 0.323657725834671 | 716.385909383749 | 3814.86163707729 | Senegal |
| 2010 | 3817.97371446196 | 0.329804072240346 | 717.935956015166 | 3100.03775844679 | Senegal |
| 2011 | 3400.03198957109 | 0.336059897035461 | 647.334015373581 | 2752.69797419751 | Senegal |
| 2012 | 2819.38887287026 | 0.34297711398143 | 574.136694160602 | 2245.25217870966 | Senegal |
| 2013 | 2509.91581767105 | 0.350134004435728 | 556.02117438602 | 1953.89464328503 | Senegal |
| 2014 | 2293.30361720993 | 0.357181573156172 | 497.158817666159 | 1796.14479954377 | Senegal |
| 2015 | 1987.51409979015 | 0.364144496085842 | 460.461069335659 | 1527.05303045449 | Senegal |
| 2016 | 1875.00623247368 | 0.371114525115408 | 406.662126732216 | 1468.34410574146 | Senegal |
| 2017 | 1596.48954536489 | 0.378351476689464 | 346.527853239088 | 1249.9616921258 | Senegal |
| 2018 | 1344.10253698119 | 0.386079665694996 | 321.330921918593 | 1022.7716150626 | Senegal |
| 2019 | 1181.06062889208 | 0.394012316088147 | 282.508174097447 | 898.552454794634 | Senegal |
| 2020 | 1045.70599675717 | 0.401197515620742 | 268.462650157053 | 777.243346600117 | Senegal |
| 2021 | 913.1239524454 | 0.408054192634391 | 238.95897360366 | 674.16497884174 | Senegal |
| 1990 | 13934.648636526 | 0.211569335210502 | 2347.50018928163 | 11587.1484472444 | Sierra Leone |
| 1991 | 13320.2040800401 | 0.213013566916176 | 2357.26675397189 | 10962.9373260682 | Sierra Leone |
| 1992 | 13695.2802513057 | 0.214223040293117 | 2338.00365016792 | 11357.2766011378 | Sierra Leone |
| 1993 | 13875.6699079267 | 0.216089200983916 | 2342.21586559601 | 11533.4540423307 | Sierra Leone |
| 1994 | 14765.4574055562 | 0.218277410522878 | 2315.32277160485 | 12450.1346339514 | Sierra Leone |
| 1995 | 13832.3302925096 | 0.219822603796338 | 2062.53563814843 | 11769.7946543612 | Sierra Leone |
| 1996 | 12454.3982629722 | 0.219969269802127 | 2094.47672692197 | 10359.9215360502 | Sierra Leone |
| 1997 | 11887.2961150689 | 0.219089790932757 | 2302.22358389974 | 9585.07253116916 | Sierra Leone |
| 1998 | 11927.4458210502 | 0.218584312916824 | 2338.12211444044 | 9589.32370660976 | Sierra Leone |
| 1999 | 12116.9546111406 | 0.217943041857131 | 2332.99781153804 | 9783.95679960256 | Sierra Leone |
| 2000 | 12339.7807834962 | 0.218492836742255 | 2344.77996195797 | 9995.00082153823 | Sierra Leone |
| 2001 | 11550.5670577262 | 0.22039123267412 | 2087.51131801237 | 9463.05573971383 | Sierra Leone |
| 2002 | 10593.6649065889 | 0.22458582518118 | 2103.22355963393 | 8490.44134695497 | Sierra Leone |
| 2003 | 9831.89920575133 | 0.229235900070887 | 1862.13225044845 | 7969.76695530288 | Sierra Leone |
| 2004 | 10254.8691728802 | 0.234131673808901 | 1877.25068383826 | 8377.61848904194 | Sierra Leone |
| 2005 | 9546.10042610244 | 0.23954993975681 | 1707.78865310091 | 7838.31177300153 | Sierra Leone |
| 2006 | 9008.50603524741 | 0.245647886413805 | 1663.49333858198 | 7345.01269666543 | Sierra Leone |
| 2007 | 8175.04678092737 | 0.252523076983211 | 1510.08620810598 | 6664.96057282139 | Sierra Leone |
| 2008 | 7406.9265705097 | 0.259781562101124 | 1352.74138874985 | 6054.18518175985 | Sierra Leone |
| 2009 | 6212.64742447087 | 0.267058380231042 | 1229.269761684 | 4983.37766278686 | Sierra Leone |
| 2010 | 5884.54842105645 | 0.274270274980285 | 1215.56081503186 | 4668.98760602459 | Sierra Leone |
| 2011 | 5386.11425518502 | 0.28167357489027 | 1152.16171960672 | 4233.9525355783 | Sierra Leone |
| 2012 | 5290.28552570322 | 0.290338511565667 | 1090.56048167988 | 4199.72504402334 | Sierra Leone |
| 2013 | 4514.49574515748 | 0.300736693229616 | 972.003035264554 | 3542.49270989293 | Sierra Leone |
| 2014 | 3657.09984654692 | 0.310348867321001 | 874.688674389943 | 2782.41117215698 | Sierra Leone |
| 2015 | 3319.26170323151 | 0.316998649992998 | 780.052419135365 | 2539.20928409614 | Sierra Leone |
| 2016 | 3117.53118585622 | 0.324091564628372 | 725.601606203711 | 2391.92957965251 | Sierra Leone |
| 2017 | 2957.52794379622 | 0.331213431016392 | 662.146301189077 | 2295.38164260714 | Sierra Leone |
| 2018 | 2704.79695058639 | 0.338341563502228 | 605.108923117725 | 2099.68802746866 | Sierra Leone |
| 2019 | 2496.45897252248 | 0.345770018534214 | 572.736396034893 | 1923.72257648759 | Sierra Leone |
| 2020 | 2291.8523194053 | 0.352412021842938 | 553.984400185217 | 1737.86791922008 | Sierra Leone |
| 2021 | 2105.23440459722 | 0.3586658807038 | 504.670264426929 | 1600.56414017029 | Sierra Leone |
| 1990 | 11570.5965780916 | 0.269692273286049 | 1213.97666942873 | 10356.6199086629 | Togo |
| 1991 | 11472.3496949473 | 0.274530567688902 | 1211.82685805549 | 10260.5228368918 | Togo |
| 1992 | 11050.3240456651 | 0.278789310483021 | 1155.39525577497 | 9894.92878989013 | Togo |
| 1993 | 10743.7850392244 | 0.281224630552395 | 1154.08031156008 | 9589.70472766432 | Togo |
| 1994 | 10447.8143566513 | 0.284653387233478 | 1111.40492706997 | 9336.40942958133 | Togo |
| 1995 | 10053.8699471334 | 0.288536691543625 | 1109.31099068028 | 8944.55895645312 | Togo |
| 1996 | 9660.1943886117 | 0.292606677265023 | 1039.07743673121 | 8621.11695188049 | Togo |
| 1997 | 9214.82459168598 | 0.296947835200193 | 1042.00644183544 | 8172.81814985054 | Togo |
| 1998 | 9478.19197268639 | 0.300022332903466 | 969.442019626002 | 8508.74995306039 | Togo |
| 1999 | 8726.30179435325 | 0.303020232352969 | 958.957848568652 | 7767.3439457846 | Togo |
| 2000 | 8513.92872151593 | 0.305472963037407 | 958.221591292844 | 7555.70713022309 | Togo |
| 2001 | 8352.31092067525 | 0.307828257876202 | 869.319245605809 | 7482.99167506944 | Togo |
| 2002 | 8763.39379532529 | 0.310469909626286 | 869.434480222001 | 7893.95931510329 | Togo |
| 2003 | 8434.82309232704 | 0.313792063910754 | 867.352098016588 | 7567.47099431045 | Togo |
| 2004 | 8117.29736531904 | 0.316687944336242 | 779.128944740767 | 7338.16842057827 | Togo |
| 2005 | 7818.01923725644 | 0.319025579582374 | 775.650720308998 | 7042.36851694744 | Togo |
| 2006 | 7641.44875123603 | 0.321659389933158 | 776.706054382558 | 6864.74269685347 | Togo |
| 2007 | 7109.41713182212 | 0.324382497112276 | 727.221961150447 | 6382.19517067167 | Togo |
| 2008 | 6865.78315149086 | 0.327743136228821 | 723.064317696898 | 6142.71883379396 | Togo |
| 2009 | 6790.15796255226 | 0.331558542074185 | 654.297598205629 | 6135.86036434663 | Togo |
| 2010 | 6408.9837967613 | 0.335899583453674 | 650.97554000537 | 5758.00825675593 | Togo |
| 2011 | 6105.82184933229 | 0.340777259848227 | 576.113477712909 | 5529.70837161938 | Togo |
| 2012 | 5591.83786986175 | 0.346320495474737 | 572.195479792699 | 5019.64239006905 | Togo |
| 2013 | 5057.66631321813 | 0.352278604950937 | 556.235040501286 | 4501.43127271684 | Togo |
| 2014 | 4331.19309867994 | 0.358661442459501 | 498.997629728837 | 3832.1954689511 | Togo |
| 2015 | 3992.7919291296 | 0.365600168512118 | 414.89743726435 | 3577.89449186525 | Togo |
| 2016 | 3794.67877356619 | 0.372910027688811 | 352.742510204807 | 3441.93626336138 | Togo |
| 2017 | 3557.41347381089 | 0.380336231070946 | 326.388184190093 | 3231.0252896208 | Togo |
| 2018 | 3178.13802350495 | 0.387921228739501 | 285.014019385498 | 2893.12400411945 | Togo |
| 2019 | 2976.72484600561 | 0.395667268909215 | 262.860169936069 | 2713.86467606954 | Togo |
| 2020 | 2676.09432800313 | 0.402356922397572 | 236.99360958077 | 2439.10071842236 | Togo |
| 2021 | 2415.67661876459 | 0.408533695477986 | 221.622445603126 | 2194.05417316146 | Togo |
| 1990 | 139.97163174176 | 0.613633923933335 | 9.06222283419822 | 130.909408907562 | American Samoa |
| 1991 | 136.58024328278 | 0.615847611881171 | 8.77440735975843 | 127.805835923022 | American Samoa |
| 1992 | 132.97656100013 | 0.618583654206334 | 8.94344192600071 | 124.033119074129 | American Samoa |
| 1993 | 129.77238579106 | 0.622078597664072 | 8.69262448040235 | 121.079761310658 | American Samoa |
| 1994 | 125.87788983517 | 0.626053240674018 | 8.91167410163771 | 116.966215733532 | American Samoa |
| 1995 | 122.21082459985 | 0.629814053909773 | 8.049081725988 | 114.161742873862 | American Samoa |
| 1996 | 119.41245102327 | 0.632706982787519 | 6.68874166669016 | 112.72370935658 | American Samoa |
| 1997 | 114.73418491572 | 0.634926685456248 | 7.36332703542418 | 107.370857880296 | American Samoa |
| 1998 | 112.81998621223 | 0.636597975713911 | 6.83044614205855 | 105.989540070171 | American Samoa |
| 1999 | 109.83251153418 | 0.638322193134778 | 6.50836857281372 | 103.324142961366 | American Samoa |
| 2000 | 107.06722334706 | 0.640609550029113 | 6.96744369778143 | 100.099779649279 | American Samoa |
| 2001 | 104.79652426267 | 0.643214421156583 | 6.03403138103983 | 98.7624928816302 | American Samoa |
| 2002 | 104.04732620937 | 0.646002062148026 | 5.80701590231128 | 98.2403103070587 | American Samoa |
| 2003 | 102.394733073 | 0.648951964151548 | 4.97646298735809 | 97.4182700856419 | American Samoa |
| 2004 | 100.65733945975 | 0.651921140709413 | 4.78149254318896 | 95.875846916561 | American Samoa |
| 2005 | 98.29746633619 | 0.655061005497018 | 4.82336867956304 | 93.474097656627 | American Samoa |
| 2006 | 96.67106806158 | 0.657725722991147 | 4.88557003761416 | 91.7854980239658 | American Samoa |
| 2007 | 94.37858225731 | 0.660781175742006 | 4.43234819791242 | 89.9462340593976 | American Samoa |
| 2008 | 92.21725811583 | 0.664025761610397 | 4.21184527131531 | 88.0054128445147 | American Samoa |
| 2009 | 92.79506851947 | 0.667300532067021 | 3.86713396816868 | 88.9279345513013 | American Samoa |
| 2010 | 92.63544657483 | 0.671248915725392 | 3.8165126549428 | 88.8189339198872 | American Samoa |
| 2011 | 92.3582996803 | 0.675979288249651 | 3.62613032239816 | 88.7321693579018 | American Samoa |
| 2012 | 92.09100867399 | 0.680861538744503 | 3.57141721739437 | 88.5195914565956 | American Samoa |
| 2013 | 92.42001941688 | 0.685833061049061 | 3.33485494185364 | 89.0851644750264 | American Samoa |
| 2014 | 93.26646638093 | 0.691343136814841 | 3.13360036034043 | 90.1328660205896 | American Samoa |
| 2015 | 95.22806734208 | 0.697162912183353 | 3.23720155438058 | 91.9908657876994 | American Samoa |
| 2016 | 97.79405268845 | 0.702726397771802 | 2.85333584020541 | 94.9407168482446 | American Samoa |
| 2017 | 98.43063535482 | 0.707647909217363 | 2.79084350488 | 95.63979184994 | American Samoa |
| 2018 | 98.41786407711 | 0.712800591003034 | 2.73305732397787 | 95.6848067531321 | American Samoa |
| 2019 | 97.8925710709 | 0.717308133053242 | 2.44352970785447 | 95.4490413630455 | American Samoa |
| 2020 | 106.16308244938 | 0.720838500394591 | 2.54203773022455 | 103.621044719155 | American Samoa |
| 2021 | 96.08763114411 | 0.723727533059592 | 2.40077378935704 | 93.686857354753 | American Samoa |
| 1990 | 75.91194449119 | 0.696451195907995 | 3.12986391415135 | 72.7820805770386 | Bermuda |
| 1991 | 67.4925467568 | 0.700276851342371 | 2.96593494032041 | 64.5266118164796 | Bermuda |
| 1992 | 64.67171648743 | 0.703984094837886 | 2.92664330538172 | 61.7450731820483 | Bermuda |
| 1993 | 65.11848501972 | 0.707678222374744 | 2.66020441360031 | 62.4582806061197 | Bermuda |
| 1994 | 58.39880468515 | 0.711068997576994 | 2.78974252269692 | 55.6090621624531 | Bermuda |
| 1995 | 52.35455587356 | 0.714468828293519 | 2.71850231067333 | 49.6360535628867 | Bermuda |
| 1996 | 46.37524263879 | 0.71783167141956 | 2.55677987738879 | 43.8184627614012 | Bermuda |
| 1997 | 41.19936968733 | 0.721642812571346 | 2.55121377780241 | 38.6481559095276 | Bermuda |
| 1998 | 35.48704808164 | 0.725853528350146 | 2.33927189355712 | 33.1477761880829 | Bermuda |
| 1999 | 29.75463907918 | 0.730539822974827 | 2.18865618231966 | 27.5659828968603 | Bermuda |
| 2000 | 24.990384373 | 0.73599650207483 | 2.04544903005192 | 22.9449353429481 | Bermuda |
| 2001 | 23.14343864427 | 0.741918907664508 | 1.85914794247365 | 21.2842907017963 | Bermuda |
| 2002 | 22.61313151931 | 0.747544503099499 | 1.71328414220985 | 20.8998473771002 | Bermuda |
| 2003 | 22.73389382502 | 0.753305163354703 | 1.77864762897176 | 20.9552461960482 | Bermuda |
| 2004 | 23.05665927097 | 0.759008884830255 | 1.6734296658337 | 21.3832296051363 | Bermuda |
| 2005 | 24.0593343673 | 0.764690902756676 | 1.8099816717175 | 22.2493526955825 | Bermuda |
| 2006 | 24.19752008238 | 0.770596769707219 | 1.75216860430609 | 22.4453514780739 | Bermuda |
| 2007 | 23.40018482855 | 0.776114264028476 | 1.7186029360313 | 21.6815818925187 | Bermuda |
| 2008 | 22.50525739043 | 0.780358325453696 | 1.68926162623709 | 20.8159957641929 | Bermuda |
| 2009 | 21.27352603987 | 0.7847591074268 | 1.66393762625435 | 19.6095884136156 | Bermuda |
| 2010 | 20.35291250496 | 0.789181466953459 | 1.61978415465514 | 18.7331283503049 | Bermuda |
| 2011 | 19.87511045889 | 0.793380523639009 | 1.58342488361986 | 18.2916855752701 | Bermuda |
| 2012 | 19.18203706485 | 0.797484869823732 | 1.56135609030354 | 17.6206809745465 | Bermuda |
| 2013 | 18.79722405512 | 0.801388200821251 | 1.54369767922051 | 17.2535263758995 | Bermuda |
| 2014 | 18.40608623648 | 0.804923406558971 | 1.54090447718233 | 16.8651817592977 | Bermuda |
| 2015 | 17.67165805595 | 0.807998794744826 | 1.53116291075702 | 16.140495145193 | Bermuda |
| 2016 | 17.25184659894 | 0.810550842307499 | 1.52325123624211 | 15.7285953626979 | Bermuda |
| 2017 | 16.51755119192 | 0.812807228761017 | 1.52469936608354 | 14.9928518258365 | Bermuda |
| 2018 | 15.78547395748 | 0.814892801994945 | 1.4989293620181 | 14.2865445954619 | Bermuda |
| 2019 | 15.4135361727 | 0.817000338369953 | 1.51741469905362 | 13.8961214736464 | Bermuda |
| 2020 | 15.06298270132 | 0.819203419907163 | 1.49579190567926 | 13.5671907956407 | Bermuda |
| 2021 | 15.88098215726 | 0.821365422389484 | 1.51021258951353 | 14.3707695677465 | Bermuda |
| 1990 | 32.15419655268 | 0.564514853922858 | 10.7134504179468 | 21.4407461347332 | Cook Islands |
| 1991 | 30.78645775179 | 0.570622775576656 | 8.98582164076915 | 21.8006361110208 | Cook Islands |
| 1992 | 29.60224241947 | 0.57712346797279 | 8.91419658633546 | 20.6880458331345 | Cook Islands |
| 1993 | 28.77024780824 | 0.584059175401459 | 8.69775659017 | 20.07249121807 | Cook Islands |
| 1994 | 28.25332948797 | 0.591540867591394 | 8.88425080464389 | 19.3690786833261 | Cook Islands |
| 1995 | 27.97803008399 | 0.598789967711714 | 8.81412968816667 | 19.1639003958233 | Cook Islands |
| 1996 | 27.48634380627 | 0.606414151024421 | 8.76645433586592 | 18.7198894704041 | Cook Islands |
| 1997 | 26.45422112167 | 0.613991564375745 | 8.81118041389194 | 17.6430407077781 | Cook Islands |
| 1998 | 25.72939839912 | 0.621202227562338 | 8.95855624133594 | 16.7708421577841 | Cook Islands |
| 1999 | 25.90865254709 | 0.628029029838686 | 8.44836901306731 | 17.4602835340227 | Cook Islands |
| 2000 | 25.1469621579 | 0.635365607290649 | 6.96895264777662 | 18.1780095101234 | Cook Islands |
| 2001 | 24.23716401921 | 0.644421442097654 | 5.95809952022697 | 18.279064498983 | Cook Islands |
| 2002 | 23.44058912483 | 0.652790488145509 | 5.07209728989412 | 18.3684918349359 | Cook Islands |
| 2003 | 22.84430845548 | 0.661465037633189 | 4.28699253765854 | 18.5573159178215 | Cook Islands |
| 2004 | 22.15845014475 | 0.668769675110248 | 3.93842289807448 | 18.2200272466755 | Cook Islands |
| 2005 | 21.99079020255 | 0.67400410252196 | 3.83058173660758 | 18.1602084659424 | Cook Islands |
| 2006 | 21.69852292405 | 0.680644001453893 | 3.592203138086 | 18.106319785964 | Cook Islands |
| 2007 | 21.51781877442 | 0.687505190649195 | 3.4126852511674 | 18.1051335232526 | Cook Islands |
| 2008 | 20.55372759708 | 0.695592068858646 | 3.15130116787529 | 17.4024264292047 | Cook Islands |
| 2009 | 19.58394202556 | 0.70377697980234 | 2.89678442116241 | 16.6871576043976 | Cook Islands |
| 2010 | 19.00827591174 | 0.711861611963853 | 2.86767091877097 | 16.140604992969 | Cook Islands |
| 2011 | 18.17689994613 | 0.719444665855079 | 2.51023459688418 | 15.6666653492458 | Cook Islands |
| 2012 | 17.10277733082 | 0.726963385157533 | 2.37586031701683 | 14.7269170138032 | Cook Islands |
| 2013 | 15.98091380164 | 0.73331701541165 | 2.20949867394743 | 13.7714151276926 | Cook Islands |
| 2014 | 15.57900152344 | 0.73959570406711 | 1.90404827479123 | 13.6749532486488 | Cook Islands |
| 2015 | 15.54686708904 | 0.746550296501143 | 1.93111631363946 | 13.6157507754005 | Cook Islands |
| 2016 | 15.58004344801 | 0.752353086814977 | 1.83224838166625 | 13.7477950663438 | Cook Islands |
| 2017 | 15.17268140243 | 0.758233571650419 | 1.78936265552915 | 13.3833187469008 | Cook Islands |
| 2018 | 14.83777141382 | 0.764041181894148 | 1.679934104526 | 13.157837309294 | Cook Islands |
| 2019 | 14.53205856487 | 0.769795912507863 | 1.76085822599787 | 12.7712003388721 | Cook Islands |
| 2020 | 25.25386576401 | 0.774700577781853 | 1.73348248379873 | 23.5203832802113 | Cook Islands |
| 2021 | 26.32934683059 | 0.779109954659683 | 1.76710258589619 | 24.5622442446938 | Cook Islands |
| 1990 | 22.24075337065 | 0.732258251665998 | 2.1613946795761 | 20.0793586910739 | Greenland |
| 1991 | 21.27904787537 | 0.730626532070962 | 2.23124089386261 | 19.0478069815074 | Greenland |
| 1992 | 19.70236360647 | 0.729781103358168 | 2.18530172778789 | 17.5170618786821 | Greenland |
| 1993 | 18.65037377677 | 0.729894841568107 | 2.17090283199491 | 16.4794709447751 | Greenland |
| 1994 | 17.48851165184 | 0.730875896131236 | 2.23922928986914 | 15.2492823619709 | Greenland |
| 1995 | 16.64921389548 | 0.73083163848634 | 2.16604351744543 | 14.4831703780346 | Greenland |
| 1996 | 15.8614845358 | 0.730216225093773 | 2.19754535516667 | 13.6639391806333 | Greenland |
| 1997 | 15.08472169151 | 0.730551086919016 | 2.2034461362648 | 12.8812755552452 | Greenland |
| 1998 | 14.41476292266 | 0.733242587548811 | 2.25773834515661 | 12.1570245775034 | Greenland |
| 1999 | 13.87686147888 | 0.736557130056076 | 2.15349605683476 | 11.7233654220452 | Greenland |
| 2000 | 13.51705314616 | 0.740857009793347 | 2.00995584707281 | 11.5070972990872 | Greenland |
| 2001 | 13.42163449398 | 0.746289605484857 | 1.81826307324526 | 11.6033714207347 | Greenland |
| 2002 | 13.5421005184 | 0.752878370778087 | 1.77421807444345 | 11.7678824439566 | Greenland |
| 2003 | 13.57598813733 | 0.759444151937822 | 1.82549483953115 | 11.7504932977989 | Greenland |
| 2004 | 13.39162951217 | 0.765401567469496 | 1.76099363983793 | 11.6306358723321 | Greenland |
| 2005 | 13.34239462458 | 0.772099579848736 | 1.82522273495391 | 11.5171718896261 | Greenland |
| 2006 | 13.24178465575 | 0.779141406404636 | 1.70379623052623 | 11.5379884252238 | Greenland |
| 2007 | 13.25318852932 | 0.785321921636087 | 1.67463289252036 | 11.5785556367996 | Greenland |
| 2008 | 13.24074078022 | 0.79144125736451 | 1.57161029902967 | 11.6691304811903 | Greenland |
| 2009 | 12.95136504571 | 0.797025750029497 | 1.54849342131806 | 11.4028716243919 | Greenland |
| 2010 | 12.74646420829 | 0.803003131492839 | 1.55404684900607 | 11.1924173592839 | Greenland |
| 2011 | 12.5215090775 | 0.808105185095196 | 1.54202791964468 | 10.9794811578553 | Greenland |
| 2012 | 12.05340466088 | 0.811684561566459 | 1.5388549486264 | 10.5145497122536 | Greenland |
| 2013 | 11.91642851175 | 0.814368307545592 | 1.51072239518274 | 10.4057061165673 | Greenland |
| 2014 | 11.46446590628 | 0.816527059982666 | 1.51501527194574 | 9.94945063433426 | Greenland |
| 2015 | 11.58112070554 | 0.8177191894267 | 1.491503537847 | 10.089617167693 | Greenland |
| 2016 | 11.07953126883 | 0.818961901690003 | 1.496361210695 | 9.583170058135 | Greenland |
| 2017 | 10.42435416916 | 0.819781100917251 | 1.50037007967588 | 8.92398408948412 | Greenland |
| 2018 | 9.71855352444 | 0.820815513545804 | 1.47845127180568 | 8.24010225263432 | Greenland |
| 2019 | 9.15599181112 | 0.822339996424292 | 1.50888864098516 | 7.64710317013484 | Greenland |
| 2020 | 9.60121177678 | 0.824359028380898 | 1.48621874060765 | 8.11499303617235 | Greenland |
| 2021 | 8.23566732156 | 0.82621033634361 | 1.4711746273469 | 6.7644926942131 | Greenland |
| 1990 | 58.270008693 | 0.67622030520232 | 3.62265189830914 | 54.6473567946909 | Guam |
| 1991 | 59.05719537089 | 0.669974291504161 | 3.79039294701301 | 55.266802423877 | Guam |
| 1992 | 59.93237788792 | 0.666356340381872 | 4.18334251795047 | 55.7490353699695 | Guam |
| 1993 | 59.67409716512 | 0.667318181427308 | 3.86299317120639 | 55.8111039939136 | Guam |
| 1994 | 57.85552530448 | 0.672111711813636 | 3.79423518938457 | 54.0612901150954 | Guam |
| 1995 | 55.69761931028 | 0.678120215047177 | 3.61889601541243 | 52.0787232948676 | Guam |
| 1996 | 53.70447826363 | 0.68417373470043 | 3.36336977632953 | 50.3411084873005 | Guam |
| 1997 | 51.27518692038 | 0.691684870710781 | 3.14364539476333 | 48.1315415256167 | Guam |
| 1998 | 49.55558003018 | 0.701819289007173 | 2.8630609789725 | 46.6925190512075 | Guam |
| 1999 | 47.99206707251 | 0.713641593327183 | 2.79381889693417 | 45.1982481755758 | Guam |
| 2000 | 46.70615503502 | 0.724802357160022 | 2.40895384036787 | 44.2972011946521 | Guam |
| 2001 | 46.21373308711 | 0.734267375715648 | 2.09214702862754 | 44.1215860584825 | Guam |
| 2002 | 47.11887172698 | 0.741284245225143 | 1.8938572287773 | 45.2250144982027 | Guam |
| 2003 | 49.34594199319 | 0.745911530185679 | 1.7513560767079 | 47.5945859164821 | Guam |
| 2004 | 51.86928334977 | 0.749016519617639 | 1.83287913810444 | 50.0364042116656 | Guam |
| 2005 | 54.33907975344 | 0.750865874718511 | 1.76480661196627 | 52.5742731414737 | Guam |
| 2006 | 56.7262302991 | 0.75117624504969 | 1.77656599353847 | 54.9496643055615 | Guam |
| 2007 | 58.68843141315 | 0.752625470789067 | 1.74225752277793 | 56.9461738903721 | Guam |
| 2008 | 59.8476701475 | 0.755152408307309 | 1.74562712147427 | 58.1020430260257 | Guam |
| 2009 | 61.56592418636 | 0.75825229605245 | 1.73189567128456 | 59.8340285150754 | Guam |
| 2010 | 63.28218671942 | 0.761484317334794 | 1.7624645700673 | 61.5197221493527 | Guam |
| 2011 | 64.84457057764 | 0.763987394174646 | 1.79410326407172 | 63.0504673135683 | Guam |
| 2012 | 66.14969376926 | 0.766845984308226 | 1.7774386775325 | 64.3722550917275 | Guam |
| 2013 | 67.18738433722 | 0.770224956660541 | 1.76268493855379 | 65.4246993986662 | Guam |
| 2014 | 68.49438546606 | 0.773441684088001 | 1.71827090795477 | 66.7761145581052 | Guam |
| 2015 | 69.79148256589 | 0.776723614176227 | 1.7306775129493 | 68.0608050529407 | Guam |
| 2016 | 69.9015376322 | 0.780511644317696 | 1.67957272634311 | 68.2219649058569 | Guam |
| 2017 | 68.24392257312 | 0.78555492004746 | 1.68410351012688 | 66.5598190629931 | Guam |
| 2018 | 67.56710020547 | 0.791294772555245 | 1.59162053500557 | 65.9754796704644 | Guam |
| 2019 | 66.34543640387 | 0.796775451912585 | 1.55333073074567 | 64.7921056731243 | Guam |
| 2020 | 61.88601740629 | 0.800969472273547 | 1.546289499222 | 60.339727907068 | Guam |
| 2021 | 60.63750758741 | 0.803982202863145 | 1.54856625696783 | 59.0889413304422 | Guam |
| 1990 | 5.76524076307 | 0.845495153448926 | 1.47332227678527 | 4.29191848628473 | Monaco |
| 1991 | 5.84293890612 | 0.848303689705517 | 1.46725597867054 | 4.37568292744946 | Monaco |
| 1992 | 5.87070884198 | 0.850863939877657 | 1.45884724161556 | 4.41186160036444 | Monaco |
| 1993 | 5.99706709063 | 0.853264991769943 | 1.46257566386102 | 4.53449142676898 | Monaco |
| 1994 | 6.08789139778 | 0.855626926997873 | 1.43925069716943 | 4.64864070061057 | Monaco |
| 1995 | 6.18326163974 | 0.857986147163975 | 1.4340733818045 | 4.7491882579355 | Monaco |
| 1996 | 6.29298814283 | 0.860245431724109 | 1.42007256787847 | 4.87291557495153 | Monaco |
| 1997 | 6.39795158672 | 0.862446719992273 | 1.44934593632514 | 4.94860565039486 | Monaco |
| 1998 | 6.5042297559 | 0.8647165130785 | 1.42234785188778 | 5.08188190401222 | Monaco |
| 1999 | 6.56411244358 | 0.866842394455442 | 1.42885327491833 | 5.13525916866167 | Monaco |
| 2000 | 6.63714901066 | 0.868973583095515 | 1.4092015490517 | 5.2279474616083 | Monaco |
| 2001 | 6.67590036006 | 0.871094754955376 | 1.36516093939742 | 5.31073942066258 | Monaco |
| 2002 | 6.73417527467 | 0.873182768875724 | 1.41417477318432 | 5.32000050148568 | Monaco |
| 2003 | 6.780625692 | 0.875197652216285 | 1.41713900368442 | 5.36348668831558 | Monaco |
| 2004 | 6.83261221472 | 0.87723154881107 | 1.42567121760125 | 5.40694099711875 | Monaco |
| 2005 | 6.87106326311 | 0.879288088865188 | 1.40133277821717 | 5.46973048489283 | Monaco |
| 2006 | 6.93246358086 | 0.881274148230096 | 1.38343735645026 | 5.54902622440974 | Monaco |
| 2007 | 7.01384468618 | 0.883262291250478 | 1.37961738693864 | 5.63422729924136 | Monaco |
| 2008 | 7.06959555585 | 0.885331393874596 | 1.38783074294298 | 5.68176481290702 | Monaco |
| 2009 | 7.13596135043 | 0.887328278515505 | 1.39238351920219 | 5.74357783122781 | Monaco |
| 2010 | 7.16972838561 | 0.889262841317756 | 1.423072974615 | 5.746655410995 | Monaco |
| 2011 | 7.15123360962 | 0.891237592262334 | 1.33237625099049 | 5.81885735862951 | Monaco |
| 2012 | 7.08190309407 | 0.893073821857862 | 1.39405549068175 | 5.68784760338825 | Monaco |
| 2013 | 6.96351233104 | 0.89486447196548 | 1.40093280939854 | 5.56257952164146 | Monaco |
| 2014 | 6.83023888006 | 0.896547873900622 | 1.39043773101841 | 5.43980114904159 | Monaco |
| 2015 | 6.67417352184 | 0.89835633653211 | 1.40745022232191 | 5.26672329951809 | Monaco |
| 2016 | 6.31662354835 | 0.900051302707716 | 1.37672538215 | 4.9398981662 | Monaco |
| 2017 | 5.728782479 | 0.901752711895654 | 1.37645259151342 | 4.35232988748658 | Monaco |
| 2018 | 5.15077448074 | 0.903388686597922 | 1.40547628363333 | 3.74529819710667 | Monaco |
| 2019 | 4.84431048414 | 0.905030990844668 | 1.40869187622064 | 3.43561860791936 | Monaco |
| 2020 | 5.553906098 | 0.90668543677348 | 1.41878506700708 | 4.13512103099292 | Monaco |
| 2021 | 5.43960012551 | 0.908262830989574 | 1.37587417709787 | 4.06372594841213 | Monaco |
| 1990 | 348.04568013874 | 0.539145980867897 | 15.0996691801758 | 332.946010958564 | Nauru |
| 1991 | 345.01923595976 | 0.538433557023675 | 15.0802420808282 | 329.938993878932 | Nauru |
| 1992 | 341.61298927544 | 0.537038892847501 | 15.0692104511713 | 326.543778824269 | Nauru |
| 1993 | 346.69164282142 | 0.535059741994823 | 15.0995381257594 | 331.592104695661 | Nauru |
| 1994 | 352.4774081942 | 0.533001096624389 | 15.0951615407789 | 337.382246653421 | Nauru |
| 1995 | 358.57232668774 | 0.530890925037636 | 15.1139746573387 | 343.458352030401 | Nauru |
| 1996 | 366.71450970753 | 0.528544473806068 | 15.0705822983104 | 351.64392740922 | Nauru |
| 1997 | 365.20843562681 | 0.526241589925116 | 15.0874838153972 | 350.120951811413 | Nauru |
| 1998 | 381.83547651972 | 0.523885302498396 | 15.1835596196634 | 366.651916900057 | Nauru |
| 1999 | 387.91439669696 | 0.521894324468662 | 15.2292373811416 | 372.685159315818 | Nauru |
| 2000 | 393.29374453867 | 0.52025488766476 | 15.1877921560929 | 378.105952382577 | Nauru |
| 2001 | 394.11194637642 | 0.519235424968716 | 15.377730597994 | 378.734215778426 | Nauru |
| 2002 | 386.99743440758 | 0.518438705224942 | 15.322060673763 | 371.675373733817 | Nauru |
| 2003 | 374.36195546788 | 0.518459986012372 | 15.2993028817216 | 359.062652586158 | Nauru |
| 2004 | 368.84944041337 | 0.518809540150096 | 15.3244352342837 | 353.525005179086 | Nauru |
| 2005 | 359.88547937459 | 0.51989286311802 | 15.1445712072666 | 344.740908167323 | Nauru |
| 2006 | 346.50684282394 | 0.522673178762107 | 15.1006786861514 | 331.406164137789 | Nauru |
| 2007 | 338.63618213116 | 0.523201350232829 | 15.1422449929722 | 323.493937138188 | Nauru |
| 2008 | 318.72776041881 | 0.526084096271176 | 15.1175479979233 | 303.610212420887 | Nauru |
| 2009 | 316.59443395613 | 0.530221091913464 | 15.0847322911908 | 301.509701664939 | Nauru |
| 2010 | 307.43767460054 | 0.535689994782251 | 15.0798986139003 | 292.35777598664 | Nauru |
| 2011 | 292.99706614359 | 0.542342742706768 | 15.0261974310342 | 277.970868712556 | Nauru |
| 2012 | 277.28504658444 | 0.549890620999273 | 14.0306654842924 | 263.254381100148 | Nauru |
| 2013 | 255.58674062375 | 0.560112906115882 | 11.7845354303354 | 243.802205193415 | Nauru |
| 2014 | 229.752668053 | 0.571424363669984 | 9.10831445946849 | 220.644353593532 | Nauru |
| 2015 | 215.16204864341 | 0.581283234356014 | 8.80496541655962 | 206.35708322685 | Nauru |
| 2016 | 204.8382112741 | 0.590102988337037 | 8.87957606730753 | 195.958635206792 | Nauru |
| 2017 | 191.31573059126 | 0.597619075639454 | 8.82145060083081 | 182.494279990429 | Nauru |
| 2018 | 179.64727390187 | 0.605311878971834 | 8.70382116846547 | 170.943452733405 | Nauru |
| 2019 | 172.23916806489 | 0.612697252950554 | 8.94474748206302 | 163.294420582827 | Nauru |
| 2020 | 163.35878099172 | 0.619330076183652 | 8.99426727945234 | 154.364513712268 | Nauru |
| 2021 | 155.57944121214 | 0.625177833565553 | 8.74585415178111 | 146.833587060359 | Nauru |
| 1990 | 200.98937614962 | 0.587532983713503 | 9.07399415150494 | 191.915381998115 | Niue |
| 1991 | 201.29156547976 | 0.593882056791176 | 8.8003043980748 | 192.491261081685 | Niue |
| 1992 | 200.64058295938 | 0.599881243015555 | 8.7425807591892 | 191.898002200191 | Niue |
| 1993 | 199.84692348979 | 0.605514778865402 | 8.76527925876132 | 191.081644231029 | Niue |
| 1994 | 200.29131302367 | 0.610650175126661 | 8.96672211567767 | 191.324590907992 | Niue |
| 1995 | 200.15006485693 | 0.615374609096138 | 9.06141734574262 | 191.088647511187 | Niue |
| 1996 | 201.26887404399 | 0.619734554803463 | 8.92923250178094 | 192.339641542209 | Niue |
| 1997 | 196.12740121747 | 0.623913847776035 | 8.91347489011646 | 187.213926327354 | Niue |
| 1998 | 202.14509680061 | 0.627578934289331 | 8.24943228374286 | 193.895664516867 | Niue |
| 1999 | 199.59937287794 | 0.63085717205161 | 8.29333918019 | 191.30603369775 | Niue |
| 2000 | 194.8852196951 | 0.634068950171114 | 7.21741553193816 | 187.667804163162 | Niue |
| 2001 | 188.90508890927 | 0.637366627073445 | 7.06104367541045 | 181.84404523386 | Niue |
| 2002 | 178.91527223315 | 0.641122021248484 | 6.14227667354817 | 172.772995559602 | Niue |
| 2003 | 168.71330423418 | 0.645779539849075 | 5.83757098382605 | 162.875733250354 | Niue |
| 2004 | 160.49899539061 | 0.650614832382256 | 5.01789207380317 | 155.481103316807 | Niue |
| 2005 | 149.85801341702 | 0.657281264925745 | 4.7694276346625 | 145.088585782358 | Niue |
| 2006 | 140.64011202178 | 0.664162983137176 | 4.25657311690797 | 136.383538904872 | Niue |
| 2007 | 133.92401432042 | 0.670199299468215 | 3.89692833411711 | 130.027085986303 | Niue |
| 2008 | 128.39688082213 | 0.67639685901135 | 3.56344099461227 | 124.833439827518 | Niue |
| 2009 | 126.61068285032 | 0.682579172713086 | 3.60221464885244 | 123.008468201468 | Niue |
| 2010 | 127.57515910112 | 0.686280630120473 | 3.4042596864255 | 124.170899414694 | Niue |
| 2011 | 128.96629328577 | 0.690315577811745 | 3.32748003107194 | 125.638813254698 | Niue |
| 2012 | 128.89895986059 | 0.694150948675056 | 3.09630089707049 | 125.80265896352 | Niue |
| 2013 | 129.17611532607 | 0.697221077939175 | 3.14013765017644 | 126.035977675894 | Niue |
| 2014 | 128.73020447804 | 0.700545923914193 | 2.92842979860552 | 125.801774679434 | Niue |
| 2015 | 128.66984216218 | 0.704232979022297 | 2.84988524218508 | 125.819956919995 | Niue |
| 2016 | 112.65865729433 | 0.707476099423504 | 2.76339252591343 | 109.895264768417 | Niue |
| 2017 | 110.44627843185 | 0.711220995949878 | 2.87688901439057 | 107.569389417459 | Niue |
| 2018 | 108.22994104929 | 0.7151082296411 | 2.52339306356299 | 105.706547985727 | Niue |
| 2019 | 105.60939561332 | 0.719078885206029 | 2.53479545509072 | 103.074600158229 | Niue |
| 2020 | 287.77601751421 | 0.722790249813625 | 2.46156377645793 | 285.314453737752 | Niue |
| 2021 | 290.48582190051 | 0.726222049709539 | 2.32486383957988 | 288.16095806093 | Niue |
| 1990 | 53.93275080285 | 0.708593838127328 | 2.68371767699607 | 51.2490331258539 | Northern Mariana Islands |
| 1991 | 52.84468741093 | 0.712223642490096 | 2.91405958438787 | 49.9306278265421 | Northern Mariana Islands |
| 1992 | 50.01414110628 | 0.715787607029664 | 2.60354670516657 | 47.4105944011134 | Northern Mariana Islands |
| 1993 | 46.28119836186 | 0.719179395545622 | 2.58872216772689 | 43.6924761941331 | Northern Mariana Islands |
| 1994 | 42.87047600469 | 0.72251049388152 | 2.38509723455912 | 40.4853787701309 | Northern Mariana Islands |
| 1995 | 41.02808599042 | 0.726219053033259 | 2.44485676974038 | 38.5832292206796 | Northern Mariana Islands |
| 1996 | 41.66905848654 | 0.730074085649478 | 2.255488661986 | 39.413569824554 | Northern Mariana Islands |
| 1997 | 44.20803958926 | 0.733795365689826 | 2.20838299226516 | 41.9996565969948 | Northern Mariana Islands |
| 1998 | 47.62015732209 | 0.737423126925004 | 2.04978950122207 | 45.5703678208679 | Northern Mariana Islands |
| 1999 | 49.74558399381 | 0.740934815031098 | 1.88649810310912 | 47.8590858907009 | Northern Mariana Islands |
| 2000 | 49.59862694377 | 0.744492865066434 | 1.83595685447795 | 47.7626700892921 | Northern Mariana Islands |
| 2001 | 47.30818867374 | 0.747170605600551 | 1.972339730391 | 45.335848943349 | Northern Mariana Islands |
| 2002 | 43.43635819378 | 0.748755188793797 | 1.75534173166183 | 41.6810164621182 | Northern Mariana Islands |
| 2003 | 39.06239899229 | 0.750171533040464 | 1.79055775269078 | 37.2718412395992 | Northern Mariana Islands |
| 2004 | 36.38916281694 | 0.751528840504646 | 1.81308411104403 | 34.576078705896 | Northern Mariana Islands |
| 2005 | 36.33081942901 | 0.751826692283402 | 1.79210114742537 | 34.5387182815846 | Northern Mariana Islands |
| 2006 | 38.67926464081 | 0.751797809045081 | 1.77977321992943 | 36.8994914208806 | Northern Mariana Islands |
| 2007 | 41.06548522663 | 0.751608416153259 | 1.78861174296784 | 39.2768734836622 | Northern Mariana Islands |
| 2008 | 42.22819993921 | 0.750807191804257 | 1.80696037479963 | 40.4212395644104 | Northern Mariana Islands |
| 2009 | 42.31555097515 | 0.748597173133935 | 1.85871021200354 | 40.4568407631465 | Northern Mariana Islands |
| 2010 | 41.02157519401 | 0.746617261581083 | 1.8452861918909 | 39.1762890021191 | Northern Mariana Islands |
| 2011 | 39.71257680078 | 0.744411908430807 | 1.83982646261313 | 37.8727503381669 | Northern Mariana Islands |
| 2012 | 39.20380937793 | 0.742937350752288 | 1.8549569808338 | 37.3488523970962 | Northern Mariana Islands |
| 2013 | 39.52031470656 | 0.742284360325222 | 1.95455870422531 | 37.5657560023347 | Northern Mariana Islands |
| 2014 | 40.53611685182 | 0.742605449321406 | 1.89240117090638 | 38.6437156809136 | Northern Mariana Islands |
| 2015 | 42.14971875646 | 0.743797398071258 | 1.85811758218138 | 40.2916011742786 | Northern Mariana Islands |
| 2016 | 42.48023250738 | 0.748455465810463 | 1.8386696004723 | 40.6415629069077 | Northern Mariana Islands |
| 2017 | 41.05727202937 | 0.756495478910201 | 1.75614072167171 | 39.3011313076983 | Northern Mariana Islands |
| 2018 | 40.31914464514 | 0.761255400013642 | 1.752665622843 | 38.566479022297 | Northern Mariana Islands |
| 2019 | 39.41226605436 | 0.765770647172973 | 1.76158009335155 | 37.6506859610085 | Northern Mariana Islands |
| 2020 | 38.10478680104 | 0.76942765953698 | 1.75419186621154 | 36.3505949348285 | Northern Mariana Islands |
| 2021 | 36.41149099331 | 0.771535213383281 | 1.79507786464081 | 34.6164131286692 | Northern Mariana Islands |
| 1990 | 451.36435234013 | 0.662909509792389 | 4.42390331110841 | 446.940449029022 | Palau |
| 1991 | 426.32496885983 | 0.668230731109012 | 3.91417158020022 | 422.41079727963 | Palau |
| 1992 | 402.94553221321 | 0.673302462400621 | 3.80271331074747 | 399.142818902463 | Palau |
| 1993 | 386.30488932194 | 0.676648715394758 | 3.62601960179899 | 382.678869720141 | Palau |
| 1994 | 370.41911997729 | 0.680195774851961 | 3.61163471427286 | 366.807485263017 | Palau |
| 1995 | 351.90806301805 | 0.684832139585057 | 3.33623432025595 | 348.571828697794 | Palau |
| 1996 | 334.48058374579 | 0.69009567251107 | 3.39081430828506 | 331.089769437505 | Palau |
| 1997 | 312.32200036842 | 0.695122122982618 | 3.11075855469464 | 309.211241813725 | Palau |
| 1998 | 299.46327258502 | 0.69965290951971 | 2.96924659533284 | 296.494025989687 | Palau |
| 1999 | 291.67567921295 | 0.703115321964044 | 2.92591452925466 | 288.749764683695 | Palau |
| 2000 | 282.667362542 | 0.70519694495869 | 3.0031403359092 | 279.664222206091 | Palau |
| 2001 | 274.8125078262 | 0.707402386499998 | 2.69285993261216 | 272.119647893588 | Palau |
| 2002 | 269.20805546701 | 0.709587643886923 | 2.7842805709957 | 266.423774896014 | Palau |
| 2003 | 264.98287038627 | 0.711000220048301 | 2.72675791503453 | 262.256112471235 | Palau |
| 2004 | 263.35832762753 | 0.712543811157683 | 2.73415380472853 | 260.624173822801 | Palau |
| 2005 | 259.08823038417 | 0.71433983112227 | 2.68908147147162 | 256.399148912698 | Palau |
| 2006 | 255.01635271033 | 0.716353431401992 | 2.53632108206654 | 252.480031628263 | Palau |
| 2007 | 249.27637695641 | 0.718809541917246 | 2.58811007966239 | 246.688266876748 | Palau |
| 2008 | 213.968442463 | 0.720806622774243 | 2.56882964773929 | 211.399612815261 | Palau |
| 2009 | 211.22454464198 | 0.722288266788472 | 2.42194903398029 | 208.802595608 | Palau |
| 2010 | 204.07720766093 | 0.724065956349117 | 2.45852703441868 | 201.618680626511 | Palau |
| 2011 | 195.94772855108 | 0.726733445911764 | 2.42225808466187 | 193.525470466418 | Palau |
| 2012 | 190.00922137125 | 0.729612899417556 | 2.29111122389843 | 187.718110147352 | Palau |
| 2013 | 185.5123152384 | 0.731953474727171 | 2.21295063502317 | 183.299364603377 | Palau |
| 2014 | 180.61009162508 | 0.734768296510748 | 2.05244164520556 | 178.557649979874 | Palau |
| 2015 | 177.51260508333 | 0.73816642523263 | 1.97252500380795 | 175.540080079522 | Palau |
| 2016 | 174.14546029774 | 0.741361910823289 | 1.97596874241389 | 172.169491555326 | Palau |
| 2017 | 169.63179246661 | 0.744224767037247 | 1.78473856972583 | 167.847053896884 | Palau |
| 2018 | 162.9330250883 | 0.747761249656357 | 1.90940031549122 | 161.023624772809 | Palau |
| 2019 | 159.40051217567 | 0.750715306594841 | 1.75152635123768 | 157.648985824432 | Palau |
| 2020 | 157.37201219792 | 0.75278092905906 | 1.78891707373879 | 155.583095124181 | Palau |
| 2021 | 154.57222926402 | 0.75404693141209 | 1.75407104338111 | 152.818158220639 | Palau |
| 1990 | 74.37657830169 | 0.658758145866254 | 4.60004427964766 | 69.7765340220423 | Puerto Rico |
| 1991 | 69.20056257185 | 0.66326172041123 | 4.36903196267521 | 64.8315306091748 | Puerto Rico |
| 1992 | 73.56519034672 | 0.667272191591073 | 3.92340410682377 | 69.6417862398962 | Puerto Rico |
| 1993 | 82.73235951729 | 0.671024105168892 | 3.93616610459136 | 78.7961934126986 | Puerto Rico |
| 1994 | 79.48601931004 | 0.675450810240548 | 3.65133406965941 | 75.8346852403806 | Puerto Rico |
| 1995 | 80.27497292803 | 0.680673949739819 | 3.59939080289349 | 76.6755821251365 | Puerto Rico |
| 1996 | 69.47261711351 | 0.685865893043239 | 3.38193450525242 | 66.0906826082576 | Puerto Rico |
| 1997 | 67.03023456142 | 0.691274381965422 | 3.14621879032 | 63.8840157711 | Puerto Rico |
| 1998 | 58.34517089652 | 0.697040445469226 | 3.17511962732554 | 55.1700512691945 | Puerto Rico |
| 1999 | 51.53079871108 | 0.702850670729254 | 2.89049722269741 | 48.6403014883826 | Puerto Rico |
| 2000 | 45.34593077752 | 0.710035652009262 | 2.73739415966676 | 42.6085366178532 | Puerto Rico |
| 2001 | 36.71048680127 | 0.718955861108189 | 2.53832751262092 | 34.1721592886491 | Puerto Rico |
| 2002 | 35.52034023703 | 0.725956855617272 | 2.41586181785967 | 33.1044784191703 | Puerto Rico |
| 2003 | 33.79193518497 | 0.730475137768497 | 2.28762187867222 | 31.5043133062978 | Puerto Rico |
| 2004 | 32.73346843089 | 0.734219545590062 | 1.98897194160904 | 30.744496489281 | Puerto Rico |
| 2005 | 33.38487857545 | 0.737742266137191 | 1.96914486069154 | 31.4157337147585 | Puerto Rico |
| 2006 | 32.02260145928 | 0.741629431374531 | 1.91356133263103 | 30.109040126649 | Puerto Rico |
| 2007 | 31.11719352466 | 0.74588380735408 | 1.94521429711269 | 29.1719792275473 | Puerto Rico |
| 2008 | 31.80418465232 | 0.750922167274437 | 1.78514250443695 | 30.019042147883 | Puerto Rico |
| 2009 | 32.67812722795 | 0.756361694934789 | 1.81122135303039 | 30.8669058749196 | Puerto Rico |
| 2010 | 30.92721226154 | 0.761763253126039 | 1.83221602644208 | 29.0949962350979 | Puerto Rico |
| 2011 | 32.75415212299 | 0.766784881779412 | 1.84884028814719 | 30.9053118348428 | Puerto Rico |
| 2012 | 35.13609153853 | 0.772311804167716 | 1.696000656144 | 33.440090882386 | Puerto Rico |
| 2013 | 31.12740565597 | 0.778461161794537 | 1.73411492293785 | 29.3932907330322 | Puerto Rico |
| 2014 | 33.83689090251 | 0.785120344673962 | 1.66410751738925 | 32.1727833851207 | Puerto Rico |
| 2015 | 31.42126144015 | 0.791800166848976 | 1.55809412920854 | 29.8631673109415 | Puerto Rico |
| 2016 | 27.12980798643 | 0.799084082615032 | 1.54416709056543 | 25.5856408958646 | Puerto Rico |
| 2017 | 26.03882249077 | 0.806648960516056 | 1.53449138463735 | 24.5043311061327 | Puerto Rico |
| 2018 | 39.36144823634 | 0.812906066317222 | 1.51725968362979 | 37.8441885527102 | Puerto Rico |
| 2019 | 42.8107666142 | 0.818843190899062 | 1.49183270939949 | 41.3189339048005 | Puerto Rico |
| 2020 | 41.99172846789 | 0.822918436314863 | 1.49182937436547 | 40.4998990935245 | Puerto Rico |
| 2021 | 39.03005092573 | 0.825525846914029 | 1.48383477961028 | 37.5462161461197 | Puerto Rico |
| 1990 | 961.82700589769 | 0.580685876593206 | 8.9299585994595 | 952.89704729823 | Saint Kitts and Nevis |
| 1991 | 810.74425397223 | 0.590254950402484 | 9.03071731484346 | 801.713536657387 | Saint Kitts and Nevis |
| 1992 | 770.32136564922 | 0.599187189594131 | 8.74288408728272 | 761.578481561937 | Saint Kitts and Nevis |
| 1993 | 788.00708013386 | 0.607235891628758 | 8.88962148055933 | 779.117458653301 | Saint Kitts and Nevis |
| 1994 | 752.78305449083 | 0.614514657962512 | 8.88203444896154 | 743.901020041868 | Saint Kitts and Nevis |
| 1995 | 693.41782418241 | 0.620716497645849 | 8.88377106115421 | 684.534053121256 | Saint Kitts and Nevis |
| 1996 | 642.34840227383 | 0.626207410919295 | 8.95737270068932 | 633.391029573141 | Saint Kitts and Nevis |
| 1997 | 558.17072904539 | 0.631274740057709 | 8.27460394248915 | 549.896125102901 | Saint Kitts and Nevis |
| 1998 | 516.02223411985 | 0.635539987956464 | 6.83497723699582 | 509.187256882854 | Saint Kitts and Nevis |
| 1999 | 421.2335743866 | 0.63971373866775 | 6.9204024262562 | 414.313171960344 | Saint Kitts and Nevis |
| 2000 | 362.17038213379 | 0.644353376846687 | 6.08112968082363 | 356.089252452966 | Saint Kitts and Nevis |
| 2001 | 297.26657106405 | 0.64922665301934 | 5.00337834614571 | 292.263192717904 | Saint Kitts and Nevis |
| 2002 | 268.52305751146 | 0.654593408970176 | 5.1997501569132 | 263.323307354547 | Saint Kitts and Nevis |
| 2003 | 255.48876800354 | 0.659925311695556 | 4.30173507295923 | 251.187032930581 | Saint Kitts and Nevis |
| 2004 | 247.19172829653 | 0.667568129909661 | 3.83633453308167 | 243.355393763448 | Saint Kitts and Nevis |
| 2005 | 256.7359115069 | 0.679145457347323 | 3.58589863661356 | 253.150012870286 | Saint Kitts and Nevis |
| 2006 | 259.98651026541 | 0.690078666648128 | 3.36770756712529 | 256.618802698285 | Saint Kitts and Nevis |
| 2007 | 253.76254926364 | 0.697727627549003 | 3.11958416337781 | 250.642965100262 | Saint Kitts and Nevis |
| 2008 | 243.24478925108 | 0.704141493783467 | 2.84000989706892 | 240.404779354011 | Saint Kitts and Nevis |
| 2009 | 240.36323051399 | 0.709499498252707 | 2.77402115919611 | 237.589209354794 | Saint Kitts and Nevis |
| 2010 | 232.00653295341 | 0.714460415458321 | 2.73026449733108 | 229.276268456079 | Saint Kitts and Nevis |
| 2011 | 248.77934098841 | 0.719324380918298 | 2.60703205526859 | 246.172308933141 | Saint Kitts and Nevis |
| 2012 | 236.37502164223 | 0.723436152222763 | 2.41492582649707 | 233.960095815733 | Saint Kitts and Nevis |
| 2013 | 242.74794488348 | 0.726706592636212 | 2.42069830482091 | 240.327246578659 | Saint Kitts and Nevis |
| 2014 | 260.62364731632 | 0.729530218928803 | 2.24414647269507 | 258.379500843625 | Saint Kitts and Nevis |
| 2015 | 244.44031597212 | 0.732220671984012 | 2.18061834692305 | 242.259697625197 | Saint Kitts and Nevis |
| 2016 | 235.69084108541 | 0.735566138078794 | 2.09459056411778 | 233.596250521292 | Saint Kitts and Nevis |
| 2017 | 221.73566768026 | 0.739470681017895 | 1.81590766231192 | 219.919760017948 | Saint Kitts and Nevis |
| 2018 | 209.56601974866 | 0.743736461382785 | 1.81956062927558 | 207.746459119384 | Saint Kitts and Nevis |
| 2019 | 199.01829494573 | 0.748152066317114 | 1.83573767452957 | 197.1825572712 | Saint Kitts and Nevis |
| 2020 | 183.48462061776 | 0.75156840922289 | 1.85650169901701 | 181.628118918743 | Saint Kitts and Nevis |
| 2021 | 180.77601775339 | 0.754987054945241 | 1.73752204966878 | 179.038495703721 | Saint Kitts and Nevis |
| 1990 | 5.65406017719 | 0.813244887891478 | 1.50688209521521 | 4.14717808197479 | San Marino |
| 1991 | 5.71377111403 | 0.818282512759725 | 1.49757673384895 | 4.21619438018105 | San Marino |
| 1992 | 5.74997579685 | 0.822782757963244 | 1.504809439032 | 4.245166357818 | San Marino |
| 1993 | 5.80905558169 | 0.827382324106265 | 1.47361906957029 | 4.33543651211971 | San Marino |
| 1994 | 5.86496654531 | 0.8319709830207 | 1.49853601562625 | 4.36643052968375 | San Marino |
| 1995 | 5.92424961741 | 0.836762725678003 | 1.48098101885565 | 4.44326859855435 | San Marino |
| 1996 | 5.97480268072 | 0.841389494577046 | 1.46564529521167 | 4.50915738550833 | San Marino |
| 1997 | 6.00802992029 | 0.845864864873086 | 1.46307069157154 | 4.54495922871846 | San Marino |
| 1998 | 6.0494022891 | 0.850474771150175 | 1.45416567075939 | 4.59523661834061 | San Marino |
| 1999 | 6.08081876713 | 0.855338613208904 | 1.43636867578409 | 4.64445009134591 | San Marino |
| 2000 | 6.12944103335 | 0.859695363255401 | 1.44890840978975 | 4.68053262356025 | San Marino |
| 2001 | 6.16138858908 | 0.863747756857469 | 1.42029691783486 | 4.74109167124514 | San Marino |
| 2002 | 6.22109845725 | 0.865680591501536 | 1.40066529495698 | 4.82043316229302 | San Marino |
| 2003 | 6.26159078294 | 0.867522008779968 | 1.41850964023349 | 4.84308114270651 | San Marino |
| 2004 | 6.31552327257 | 0.869287134173222 | 1.42387993233421 | 4.89164334023579 | San Marino |
| 2005 | 6.3646788759 | 0.87097959576402 | 1.42199265078071 | 4.94268622511929 | San Marino |
| 2006 | 6.4063271909 | 0.872652149736731 | 1.4105668630205 | 4.9957603278795 | San Marino |
| 2007 | 6.47820729326 | 0.874307253083233 | 1.40424729510233 | 5.07395999815767 | San Marino |
| 2008 | 6.55982681211 | 0.875991881471404 | 1.39608809054333 | 5.16373872156667 | San Marino |
| 2009 | 6.6105898534 | 0.877705228262122 | 1.41694829912132 | 5.19364155427868 | San Marino |
| 2010 | 6.62092487069 | 0.879440813617125 | 1.43175801927739 | 5.18916685141261 | San Marino |
| 2011 | 6.58717848064 | 0.881197517807579 | 1.39497013346467 | 5.19220834717533 | San Marino |
| 2012 | 6.53085135207 | 0.88292453140369 | 1.40123780077818 | 5.12961355129182 | San Marino |
| 2013 | 6.451667276 | 0.884592626900131 | 1.39466013734553 | 5.05700713865447 | San Marino |
| 2014 | 6.36072309388 | 0.885353693736433 | 1.3725740386874 | 4.9881490551926 | San Marino |
| 2015 | 6.26523989077 | 0.884693365070092 | 1.41692765302611 | 4.84831223774389 | San Marino |
| 2016 | 5.98473234694 | 0.884477664772925 | 1.40237646396065 | 4.58235588297935 | San Marino |
| 2017 | 5.45888635816 | 0.884557606743879 | 1.38260060983 | 4.07628574833 | San Marino |
| 2018 | 4.93526878437 | 0.885133515535323 | 1.44971554867583 | 3.48555323569417 | San Marino |
| 2019 | 4.65873192339 | 0.886200660194545 | 1.38636379031849 | 3.27236813307151 | San Marino |
| 2020 | 4.52249221927 | 0.886867937194416 | 1.40988437949026 | 3.11260783977974 | San Marino |
| 2021 | 4.3040989226 | 0.888005473958106 | 1.42677328315946 | 2.87732563944054 | San Marino |
| 1990 | 603.21022572332 | 0.521942386048128 | 15.1136207355193 | 588.096604987801 | Tokelau |
| 1991 | 544.10234017944 | 0.526628473491586 | 15.0842388612179 | 529.018101318222 | Tokelau |
| 1992 | 498.44501371851 | 0.531307850030329 | 15.1007189616438 | 483.344294756866 | Tokelau |
| 1993 | 466.20715762984 | 0.535935005438943 | 15.0333304052049 | 451.173827224635 | Tokelau |
| 1994 | 441.66743676836 | 0.540523664725072 | 15.051693690319 | 426.615743078041 | Tokelau |
| 1995 | 423.98557813685 | 0.545295634483764 | 15.0113137619758 | 408.974264374874 | Tokelau |
| 1996 | 409.36439471945 | 0.55028265171702 | 13.7724813514806 | 395.591913367969 | Tokelau |
| 1997 | 379.59401058573 | 0.555631325529965 | 12.8530722255147 | 366.740938360215 | Tokelau |
| 1998 | 359.96897125373 | 0.561047610799914 | 11.4403184949555 | 348.528652758775 | Tokelau |
| 1999 | 362.53312120331 | 0.566415405899331 | 10.4201352485758 | 352.112985954734 | Tokelau |
| 2000 | 341.35131648975 | 0.571814369871981 | 8.84399028441221 | 332.507326205338 | Tokelau |
| 2001 | 314.62592206439 | 0.576915597204096 | 8.86939063292541 | 305.756531431465 | Tokelau |
| 2002 | 289.2705441613 | 0.581858317064991 | 8.82462110060857 | 280.445923060691 | Tokelau |
| 2003 | 260.95506383416 | 0.586854346188713 | 8.7951515935268 | 252.159912240633 | Tokelau |
| 2004 | 242.08019493321 | 0.592342442430319 | 8.96954965172276 | 233.110645281487 | Tokelau |
| 2005 | 218.10874700545 | 0.59817611685198 | 8.77729151306608 | 209.331455492384 | Tokelau |
| 2006 | 199.10879033117 | 0.604429389636372 | 8.51244297777286 | 190.596347353397 | Tokelau |
| 2007 | 183.65886289471 | 0.61116323669051 | 8.92694207699572 | 174.731920817714 | Tokelau |
| 2008 | 169.57920133476 | 0.617940246542751 | 8.79521343194159 | 160.783987902818 | Tokelau |
| 2009 | 157.29906686965 | 0.624070078096847 | 8.98146586201549 | 148.317601007635 | Tokelau |
| 2010 | 155.88415740872 | 0.630175540158128 | 8.39121705871759 | 147.492940350002 | Tokelau |
| 2011 | 152.7297606338 | 0.636016518005944 | 7.08345370321708 | 145.646306930583 | Tokelau |
| 2012 | 148.70269863506 | 0.641611991197784 | 6.19740859587183 | 142.505290039188 | Tokelau |
| 2013 | 142.23708815338 | 0.647966148124354 | 5.8004631317229 | 136.436625021657 | Tokelau |
| 2014 | 136.93455069845 | 0.653231999411508 | 4.75303745218373 | 132.181513246266 | Tokelau |
| 2015 | 133.43820510928 | 0.658798994926187 | 4.42545193256292 | 129.012753176717 | Tokelau |
| 2016 | 131.1372094482 | 0.663974319058952 | 4.22211064599759 | 126.915098802202 | Tokelau |
| 2017 | 126.25480964455 | 0.669054133759442 | 3.85890122717556 | 122.395908417374 | Tokelau |
| 2018 | 123.1245957855 | 0.674179514031409 | 3.80382028379535 | 119.320775501705 | Tokelau |
| 2019 | 120.26998564689 | 0.679247839201533 | 3.60094740174254 | 116.669038245147 | Tokelau |
| 2020 | 1090.83363924554 | 0.682937378311047 | 3.39515698840923 | 1087.43848225713 | Tokelau |
| 2021 | 1105.58220507229 | 0.68642562083005 | 3.3886098620937 | 1102.1935952102 | Tokelau |
| 1990 | 870.94513221688 | 0.406247565957081 | 242.143170276112 | 628.801961940768 | Tuvalu |
| 1991 | 768.02258337886 | 0.414819936897447 | 215.073212070591 | 552.949371308269 | Tuvalu |
| 1992 | 681.49208230602 | 0.422901979744667 | 184.001153247441 | 497.490929058579 | Tuvalu |
| 1993 | 611.50625451585 | 0.430518260028408 | 136.496941121768 | 475.009313394082 | Tuvalu |
| 1994 | 544.26823248409 | 0.438563050767836 | 129.608018439721 | 414.660214044369 | Tuvalu |
| 1995 | 501.30816910668 | 0.444962241977075 | 112.449759394529 | 388.858409712151 | Tuvalu |
| 1996 | 469.69674668386 | 0.449804979978272 | 106.566842661679 | 363.129904022181 | Tuvalu |
| 1997 | 418.94200416166 | 0.455368427922084 | 98.2923743435983 | 320.649629818062 | Tuvalu |
| 1998 | 375.65192695058 | 0.462454678093366 | 89.789966718577 | 285.861960232003 | Tuvalu |
| 1999 | 358.72080087431 | 0.468845801641229 | 83.1690850498498 | 275.55171582446 | Tuvalu |
| 2000 | 321.50061647911 | 0.474956823462581 | 79.6694763618435 | 241.831140117267 | Tuvalu |
| 2001 | 288.10579318006 | 0.480850886398825 | 76.148044085426 | 211.957749094634 | Tuvalu |
| 2002 | 259.98710338546 | 0.487252252447393 | 74.7964527412809 | 185.190650644179 | Tuvalu |
| 2003 | 236.85251581071 | 0.492754711063713 | 72.9006049790357 | 163.951910831674 | Tuvalu |
| 2004 | 209.27736395925 | 0.497692152366724 | 22.0415847572991 | 187.235779201951 | Tuvalu |
| 2005 | 198.59059698055 | 0.501851309525404 | 16.7260763277342 | 181.864520652816 | Tuvalu |
| 2006 | 205.52328204878 | 0.505975410569688 | 16.2042806206774 | 189.319001428103 | Tuvalu |
| 2007 | 195.5923516822 | 0.51050430052828 | 15.7047415240748 | 179.887610158125 | Tuvalu |
| 2008 | 182.45724856847 | 0.515611600817149 | 15.3971967888297 | 167.06005177964 | Tuvalu |
| 2009 | 179.40368068352 | 0.519870565592834 | 15.1616583364536 | 164.242022347066 | Tuvalu |
| 2010 | 177.83091768447 | 0.52345550974778 | 15.1910657966683 | 162.639851887802 | Tuvalu |
| 2011 | 172.72764549982 | 0.527744244866808 | 15.1462976977988 | 157.581347802021 | Tuvalu |
| 2012 | 172.75145571306 | 0.531203234840239 | 15.0703328700379 | 157.681122843022 | Tuvalu |
| 2013 | 170.69163075956 | 0.53516623462033 | 15.0716974768794 | 155.619933282681 | Tuvalu |
| 2014 | 167.03187542118 | 0.539122244261915 | 15.1080111891968 | 151.923864231983 | Tuvalu |
| 2015 | 165.49973650185 | 0.544047880204377 | 15.0338904004225 | 150.465846101427 | Tuvalu |
| 2016 | 163.34444669211 | 0.549173928060589 | 14.3774725515936 | 148.966974140516 | Tuvalu |
| 2017 | 152.93348898849 | 0.554466480141161 | 12.9824677254766 | 139.951021263013 | Tuvalu |
| 2018 | 144.24469317299 | 0.559859395089525 | 12.1353554546715 | 132.109337718318 | Tuvalu |
| 2019 | 134.69685969968 | 0.566045488622333 | 10.3974875146511 | 124.299372185029 | Tuvalu |
| 2020 | 126.49734830966 | 0.571666102458017 | 8.9564781624798 | 117.54087014718 | Tuvalu |
| 2021 | 119.63323497627 | 0.576620529344684 | 8.78014567789 | 110.85308929838 | Tuvalu |
| 1990 | 114.93927318082 | 0.655160855636097 | 5.12153509693085 | 109.817738083889 | United States Virgin Islands |
| 1991 | 107.81462294186 | 0.664018659064364 | 4.14093931119909 | 103.673683630661 | United States Virgin Islands |
| 1992 | 102.1442847776 | 0.67108187072399 | 3.88839480212132 | 98.2558899754787 | United States Virgin Islands |
| 1993 | 97.58311054658 | 0.676872744796339 | 3.61460827968097 | 93.968502266899 | United States Virgin Islands |
| 1994 | 93.57117029883 | 0.681982223083549 | 3.54410785828781 | 90.0270624405422 | United States Virgin Islands |
| 1995 | 88.72659457811 | 0.686730210603479 | 3.34127774877486 | 85.3853168293351 | United States Virgin Islands |
| 1996 | 84.09068461773 | 0.691247698919766 | 3.12304986653739 | 80.9676347511926 | United States Virgin Islands |
| 1997 | 79.70279700685 | 0.69559820385004 | 3.15285920552086 | 76.5499378013292 | United States Virgin Islands |
| 1998 | 75.41913202351 | 0.699768685957964 | 2.88692963043597 | 72.532202393074 | United States Virgin Islands |
| 1999 | 71.83449336321 | 0.703949341926433 | 2.92785931377906 | 68.9066340494309 | United States Virgin Islands |
| 2000 | 68.03877220345 | 0.708436564859144 | 2.76578620054353 | 65.2729860029065 | United States Virgin Islands |
| 2001 | 64.91784753689 | 0.713751114883336 | 2.66421451880716 | 62.2536330180828 | United States Virgin Islands |
| 2002 | 60.69611267919 | 0.724398272335199 | 2.49469653174713 | 58.2014161474429 | United States Virgin Islands |
| 2003 | 58.00154500586 | 0.734609849926843 | 2.10056575437681 | 55.9009792514832 | United States Virgin Islands |
| 2004 | 55.90714320886 | 0.744613598166223 | 1.89993229536 | 54.0072109135 | United States Virgin Islands |
| 2005 | 54.30797668781 | 0.754267557852673 | 1.7304751652825 | 52.5775015225275 | United States Virgin Islands |
| 2006 | 52.65264895503 | 0.763447495711391 | 1.68174517216392 | 50.9709037828661 | United States Virgin Islands |
| 2007 | 51.44625445214 | 0.772180593452306 | 1.80321106053135 | 49.6430433916087 | United States Virgin Islands |
| 2008 | 50.3474703353 | 0.780342622959945 | 1.68395514000561 | 48.6635151952944 | United States Virgin Islands |
[truncated: 7,641 more chars]
